# Role of Forkhead Box P3 in IFNγ-Mediated PD-L1 Expression and Bladder Cancer Epithelial-to-Mesenchymal Transition

**DOI:** 10.1158/2767-9764.CRC-23-0493

**Published:** 2024-08-26

**Authors:** Hanwei Zhang, Ann Ly, Emily Chou, Liang Wang, Paul Zhang, Kris Prado, Yiqian Gu, Matteo Pellegrini, Arnold I. Chin

**Affiliations:** 1 Department of Urology, University of California, Los Angeles, California.; 2 Department of Microbiology, Immunology and Molecular Genetics, University of California, Los Angeles, California.; 3 Department of Molecular, Cell and Developmental Biology, University of California, Los Angeles, California.; 4 UCLA Broad Stem Cell Research Center, Los Angeles, California.

## Abstract

Antagonism of the PD-1/PD-L1 axis is a critical therapeutic strategy for patients with advanced bladder cancer. IFNγ functions as a key regulator of PD-L1 in both immune as well as cancer cells. Forkhead box P3 (FOXP3) is a transcription factor synonymous in T regulatory cell function but with increasingly described functions in cancer cells. Here, we investigated the relationship between FOXP3 and PD-L1 in bladder cancer. We showed that FOXP3 is critical in the ability for IFNγ to activate PD-L1 in bladder cancer cells. FOXP3 can bind to the PD-L1 promoter and induces a gene program that leads to regulation of multiple immune-related genes and genes involved in epithelial-to-mesenchymal transition (EMT). Using *in vitro* and *in vivo* human and murine models, we showed that FOXP3 can influence bladder cancer EMT as well as promote cancer metastases. Furthermore, FOXP3 may be a convergent factor for multiple activators of PD-L1, including the chemotherapeutic drug cisplatin.

**Significance:**

Historically a key transcription factor driving T regulatory cell function, FOXP3 has an increasingly recognized role in cancer cells. In bladder cancer, we defined a novel mechanism whereby FOXP3 mediates the activation of the immune checkpoint PD-L1 by the cytokine IFNγ. We also showed that FOXP3 induces other immune checkpoints as well as genes involved in EMT, promoting immune resistance and cancer metastases.

## Introduction

Bladder cancer affects more than 81,000 people in the United States and 570,000 individuals worldwide, leading to more than 200,000 deaths a year worldwide (1). Urothelial bladder cancer comprises the majority of bladder cancer cases, and up to 30% of patients initially present with muscle-invasive bladder cancer or metastatic disease at the time of diagnoses (2). Cisplatin-based chemotherapy has been the mainstay systemic therapy for patients with advanced urothelial bladder cancer for decades (3). However, recent studies have shown superiority of anti–PD-L1 checkpoint inhibitors in combination with chemotherapy over cisplatin-based chemotherapy alone in this patient population (4, 5). Urothelial bladder cancer is responsive to checkpoint inhibitors in part due to its high tumor mutational burden, infiltration of immune cells, and the expression of PD-L1 in up to 40% of tumors (3, 6, 7).

Immune checkpoints are diverse molecules important in regulating the delicate balance of immune homeostasis. Interaction of PD-1 (CD279), an Ig superfamily member of the CD28 family of T-cell coreceptors, with its ligand PD-L1 (CD274/B7-H1) downregulates CD8^+^ T-cell immunity in peripheral tissues (8). Cancers have developed mechanisms to evade the immune system by hijacking negative regulatory immune pathways, one of which is by increasing the expression of PD-L1 on tumor epithelial cells (8). Blocking this receptor–ligand interaction inhibits downregulation of cytotoxic CD8^+^ T lymphocytes (CTL) and sustains anti-tumor immune responses. The therapeutic targeting of inhibitory checkpoint molecules against CTL-associated protein 4 (CTLA-4), PD-1, and PD-L1 have led to profound clinical responses and have revolutionized the landscape of cancer immunotherapy across multiple cancer types (9).

PD-L1 is expressed on both hematopoietic cells, such as T, B, and myeloid cells, and nonhematopoietic tissues, including vascular endothelium and cancer cells (8, 9). The level of PD-L1 expression on both tumor and infiltrating immune cells is dynamic and correlates with response to anti–PD-1 or anti–PD-L1 blockade therapy (10). Indeed, a threshold level of PD-L1 expression is used as an inclusion criterion for the treatment of multiple cancer types (11). IFNγ, a cytokine produced largely by T cells and NK cells, has been attributed as a master regulator of PD-L1 expression through the JAK-STAT-IRF1 pathway (8, 9, 12). An increasing body of literature has described multiple transcriptional and translational regulatory mechanisms of PD-L1 in both immune cells as well as in a variety of cancer cells (13). Further understanding of PD-L1 regulation may lead to future therapies to augment antitumor immunity.

We have previously shown that bladder cancer expression of FOXP3 predominates as an alternatively spliced form, FOXP3Δ3, and negatively correlates with patient outcomes (14). Further analysis of our initial overexpression studies of FOXP3Δ3 in bladder cancer cell lines suggested correlation to PD-L1 expression. We hypothesized that FOXP3 may regulate a gene program that induces immune resistance in bladder cancer. Here, we tested this hypothesis and investigated the relationship between FOXP3Δ3 and PD-L1 in bladder cancer.

## Materials and Methods

### Cell culture

Human bladder cancer cell lines HT1376 (RRID: CVCL_1292), T24 (RRID: CVCL_0554), and SW780 (RRID: CVCL_1728) were all originally authenticated from ATCC (karyotyping and morphology). Murine MB49 cell lines were originally derived from carcinogen-induced urothelial cell carcinomas in C57Bl6 mice, obtained from Timothy Ratliff. *Mycoplasma* testing was conducted upon cell line receipt using MycoProbe Mycoplasma Detection Kit (R&D Systems, Cat# CUL001B) and used within 6 months of resuscitation from frozen aliquots (14, 15). PARCB1 and PARCB2 were generated from primary urothelial cells. No *Mycoplasma* testing was conducted, and cell lines were used within 6 months of resuscitation from frozen aliquots (16). Additional single-cell clones derived from these lines were expanded and frozen in multiple early-passage aliquots for experimentation and used within 6 months. Cell lines were maintained at 37°C with 5% CO_2_ in RPMI-1640 medium (Corning, Cat# 15-013-CV) or DMEM (Corning, Cat# 10-040-CV) supplemented with 10% FBS (Omega Scientific, Cat# FB-01) and 1% penicillin–streptomycin. For PD-L1 activation, cells were cultured in 6-well plates treated with human 100 ng/mL IFNγ (PeproTech, Cat# 300-02), human 100 ng/mL IL4 (PeproTech, Cat# 200-04), human 100 ng/mL IL10 (PeproTech, Cat# 200-10), mouse 100 ng/mL IFNγ (PeproTech, Cat# 315-05), 20 μmol/L cisplatin (Sigma-Aldrich, Cat# 232120), or 20 μmol/L ruxolitinib (Selleck Chemicals, Cat# S1378) for 48 hours as indicated. For IFNγ blocking, cells were cocultured with 2 µg/mL anti-human IFNγ Ab (BioLegend, Cat# 506502, RRID: AB_315435) or isotype control (BioLegend, Cat# 401402, RRID: AB_2801451). PARCB1 and PARCB2 lines were cultured in advanced DMEM/F12 (Gibco, Cat# 12634-010) containing 10% B27 (Thermo Fisher Scientific, Cat# 17504044), Glutamax (Thermo Fisher Scientific, Cat# 35050061), 10 ng/mL human FGF (PeproTech, Cat# 100-18B), and 10 ng/mL human EGF (PeproTech, Cat# AF-100-15).

### Generation of KO cells

HT1376 and SW780 cell lines deficient in FOXP3 were generated by CRISPR/Cas9 using two independent guide RNAs (gRNA), AAA​CCGCCTCGA​AGATCT​CGG​CCC (Gr3) and AAA​CGG​GGG​AAC​CTTCCAGGG​CCG (Gr4), targeting exon 2 (coding exon 1) and cloned into lenti-gRNA-puro (Addgene, Cat# 84752, RRID: Addgene_167911). MB49 cell lines deficient in FOXP3 were generated by CRISPR/Cas9 using two independent gRNAs, CCCCAG​GAG​TCT​TGC​CAA​GC (Gr5) and GTGGGG​GAC​CCTTCC​AAGGTC (Gr6), to target mouse *Foxp3* exon 1 and cloned into lenti-gRNA-puro. Lentivirus were produced by standard methods. Briefly, HEK293T cells at 80% confluence were cotransfected with lenti-CRISPR-gRNA, VSV-G envelope plasmid, and pCMV-dR8.2 packaging plasmid by X-treme transfection reagent (Roche, Cat# 6366244001) following the manufacturer’s instructions. After transfection for 48 hours, supernatants containing virus were collected and filtered at 0.45 μm. Transduction of cells were performed in the presence of 8 μg/mL polybrene (Sigma-Aldrich, Cat# TR-1003-G). gRNA-expressing single clones were established by a 2 to 3 weeks of selection with 1 μg/mL puromycin. Single-cell clones were generated and confirmed by Sanger sequencing. Gene knockout (KO) was confirmed by qPCR, Western blotting, and/or flow cytometry. CRISPR control lines were generated using gRNA targeting the luciferase gene.

### FOXP3 reconstitution

FOXP3 and FOXP3Δ3 cDNA was cloned into the lentiviral vector FUCW driven by a *Cytomegalovirus* promoter and GFP, red fluorescent protein, or blue fluorescent protein driven by a ubiquitin promoter, respectively. HT1376 CRISPR KO lines, PARCB1 and PARCB2 cells, in single-cell suspension were combined, with lentivirus expressing either FOXP3Δ3 or FOXP3 in the presence of 8 μg/mL polybrene. Single-cell clones were selected for reconstitution, and FOXP3 expression was confirmed by Western blotting and flow cytometry.

### qPCR

qPCR experiments were performed using SYBR Green on the ViiA 7 (Applied Biosciences) with primers for human *FOXP3Δ3* (5′-GCA​GCT​CTCAAC​GGT​GGA​T, 3′-CTG​ATC​ATG​GCT​GGG​CTC​TC), human *FOXP3* (5′- TGC​CTC​CTC​TTC​TTC​CTT​GA, 3′- TTG​AGA​GCT​GGT​GCA​TGA​AA), human *PD-L1* (5′-GGA​CAA​GCA​GTG​ACC​ATC​A, 3′-CCC​AGA​ATT​ACCAAG​TGA​GTC​CT), murine *Foxp3* (5′- CAC​CTA​TGC​CAC​CCT​TATCGG, 3′- CAT​GCG​AGT​AAA​CCA​ATG​GTA), and murine *PD-L1* (5′- AGTATG​GCA​GCA​ACG​TCA​CG, 3′- TCC​TTT​TCC​CAG​TAC​ACC​ACT​A). Total RNA from cultured cells were extracted using TRIzol reagent (Invitrogen, Cat#15596026). cDNAs were synthesized with a high-capacity cDNA reverse transcription kit (Thermo Fisher Scientific, Cat# 4368814).

### Immunoblotting

Human and mouse bladder cancer cell lines were lysed with 1% Triton X-100 lysis buffer containing proteinase inhibitors (Roche, Cat# 11 836 153 001), protein concentration measured by Pierce BCA Protein Assay Reagent A (Thermo Fisher Scientific, Cat# 23228), and separated on NuPAGE 4% to 12% Bis-Tris Gel (Invitrogen, Cat# NP0322Box). PARCB samples for PD-L1 immunoblotting were first deglycosylated with 1% PNGase F (New England Biolabs, Cat# P0704S). Blots were incubated with anti-human FOXP3 (1:5,000, Invitrogen, Cat# 14-4774-82, RRID: AB_467552), anti-human PD-L1 (0.25 μg/mL, R&D Systems, Cat# AF156, RRID: AB_2073445), anti-human phospho-STAT1 (1:1,000, Cell Signaling Technology, Cat# 9167, RRID: AB_561284), anti-mouse FOXP3 (1:5,000, Abcam, Cat# 54501, RRID: AB_880110), or anti-mouse PD-L1 (0.5 μg/mL, R&D Systems, Cat# AF1019, RRID: AB_354540), and secondary anti-goat or -rabbit horseradish peroxidase–conjugated Ab (1:2,000, SouthernBiotech, Cat# 6425-05, RRID: AB_2796346, Cat# 3030-05, RRID: AB_2716837, and Cat# 4040-05, RRID: AB_2795942), with membranes visualized on film enhanced by chemiluminescence (Cytiva, Cat# RPN2332). GAPDH (1:5,000, Thermo Fisher Scientific, Cat# MA5-15738-HRP, RRID: AB_2537659) was used as the loading control.

### Immunoprecipitation

For FOXP3 immunoprecipitation (IP), MB49 cell lysates were centrifuged at 4°C at 12,000 *g* for 10 minutes. Supernatants were mixed with 5 μL Dynabeads (Invitrogen, Cat# 10003D) after 1:5 dilution with IP diluting buffer for 2 hours at 4°C to preclear. Supernatants were collected and incubated with 10 μg/mL anti-mouse FOXP3 Ab (BioLegend, Cat# 126402, RRID: AB_1089120) for 4°C overnight. Twenty-five μL Dynabeads were added to the Ab–lysate mix and incubated for 4 hours at 4°C. Immune complexes were collected and washed 5 times with co-IP buffer, and bound proteins were eluted by 2× SDS-PAGE loading buffer for Western blotting.

### Flow cytometry

Human bladder cancer cell lines were collected with 0.05 or 0.25% trypsin-EDTA and washed with PBS containing 1% FBS. Human bladder lines were stained for anti-human PD-L1 (1:750, BV421 clone 29E.2A3, BioLegend, Cat# 329713, RRID: AB_10901164) and anti-human FOXP3 (1:20, Alexa Fluor 647 clone 259D/C7, BD Biosciences, Cat# 560045, RRID: AB_1645411) with intracellular staining performed using the manufacturer’s protocol (eBioscience, Cat# 00-5523). Murine bladder lines were stained with anti-mouse PD-L1 (1:1,000, BV421 clone 10F.9G2, BioLegend, Cat# 124315, RRID: AB_10897097) and anti-mouse FOXP3 (1:50, Alexa Fluor 647 clone MF23, BD Biosciences, Cat# 560402, RRID: AB_1645202). After staining, cells were analyzed on LSRFortessa Cell Analyzer (BD Bioscience). Data were collected using BD FACS Diva Software version 7 and analyzed with FlowJo v10.1.6 (RRID: SCR_008520).

### ChIP

Chromatin immunoprecipitation (ChIP) assays were performed with HT1376 cells in accordance with standard protocols. HT1376 cells were treated with IFNγ for 48 hours, cross-linked for 9 minutes with 1% formaldehyde at 37°C, and quenched with 140 nmol/L glycine for 5 minutes. After rinsing with PBS containing proteinase inhibitors, cells were detached by scraping in 1.5 mL lysis buffer. Chromatin was sheared by sonication (Qsonica Q800R, 40 cycles of 20 seconds on and 40 seconds off at 4°C) to obtain chromatin fragment lengths of 100 to 800 bp. After centrifugation at 14,000 RPM for 10 minutes at 4°C, the supernatant was collected and incubated with anti-FOXP3 (Abcam, Cat# 54501, RRID: AB_880110) or control Ab (rabbit IgG) overnight with rotation at 4°C and then incubated for 2 hours with 24 μL Dynabeads (Invitrogen, Cat# 10003D) for each 250 μL lysate. After washing, IP DNA was eluted and treated with Proteinase K, and the cross-links were reversed. DNA was purified with chloroform. DNA concentration was measured by Qubit, and 1 ng of ChIP DNA was used for PCR.

ChIP primers for binding sites 1 and 2 (5′-ACC​AGT​GAC​ATA​AAC​AGA​CCA​A, 3′-TCC​CAA​ATA​GTG​TTG​CTG​ATC​A), binding site 3 (5′-CGTCAG​TTT​GGA​TGT​TTG​GA, 3′-CCA​AAA​TTT​CAT​GAA​TCA​CTTCAC), binding site 4 (5′-GAA​ACC​TAC​CTG​TTC​TCC​CTC​T, 3′- AGAAAG​TAA​GTT​GCA​GAA​CAC​TAC​A), binding site 5 (5′-GGA​TTTCTG​CTA​CAT​GTA​GTG, 3′- AAT​ACC​ATT​AAG​GAC​TGA​ATT​CA), binding site 6 (5′-TCC​CAT​TAG​TGG​GTA​AGA​AAG, 3′-TTC​ACT​TAT​GAG​CTT​GAC​CA), and binding site 7 (5′-TTA​TTG​TGA​GAT​TGA​ACA​TCT​TTC​A, 3′-AAC​TAT​ACA​AAC​TAC​ACA​TAA​CCC).

### Luciferase reporter assay

The PD-L1 promoter sequence (−3,000–0 from the ATG initiation site) was cloned from HT1376 cells into the pGL4.10 luciferase vector at the Kpn1 and Xho1 restriction sites using the primers 5′- TAA​GCA​GCTAGCTGA​CCTCAC​AGG​CCT​GAA and 3′-TAA​GCA​CTC​GAG​CTTTCT​GGAATG​CCCTAA. Mutations to putative FOXP3-binding sites were generated using Q5 Site-Directed Mutagenesis Kit (New England Biolabs, Cat# E0554). Primers were used for BS1 [5′-CAG​TGA​CATTTACAG​ACC​AAA​AAA, 3′-GCT​AGG​CCC​TGA​GGA​TAG​ATT (ATAAACA to ATTTACA)], BS2 [5′- AAT​ATT​TTT​TAA​TTT​ATG​GGT​GA, 3′- ATTTTTTTG​CTA​ATT​TGAAAGATC (TAAACA to TAATTT)], BS3 [5′- GGT​CTG​CGG​GAC​ATTCTACG, 3′- AAG​TGA​TAT​AAT​GGA​AAG​AAC​CCC (TAAACA to GGGACA)], BS4 [5′-CCT​GTG​TCG​GGA​CAC​ACA​C, 3′-AAT​CAC​TGT​CAA​TAT​CTT​GGACAT​TTC (TAAACA to GGGTTT)], BS5 [5′- CTT​ACT​TTC​TAT​GAAAAGGGA​AAA​TCA​GTA​CA, 3′- TTG​CAG​AAC​ACT​ACA​TGT​AGCAGA​AA (TGTTTAC to AAAAGGG)], BS6 [5′- CAT​ATC​TTC​ACAGGG​ACT​AAA​TAT​T, 3′-ACA​AAT​AGG​CTTTCTTAC​C (ACAAACA to ACAGGGA)], and BS7 [5′-ATC​TTTCAT​AAA​AAG​GGA​TGT​CAC​CT, 3′-GTT​CAA​TCT​CAC​AAT​AAA​AAA​TATAAG​ATA​TGA​AA (TGTTTAC to AAAAGGG)]. Sequences were verified by Sanger sequencing.

Dual-luciferase reporter assay system (Promega, Cat# E1910) was used according to the manufacturer’s protocol. Briefly, 1.25 × 10^5^ 293T cells were plated in 24-well plates (Corning) for 24 hours prior to transfection. Transfection was performed with 1.2 μL Lipofectamine 3000 Transfection Reagent with 50 ng luciferase reporter (wild-type PD-L1 promoter or mutant PD-L1 promoter plasmid DNA), 500 ng FOXP3Δ3 or FOXP3, and 5 ng Renilla diluted in 50 μL of Opti-MEM (Gibco) used as the internal control. Forty-eight hours after transfection, cells were washed with 1 mL PBS and lysed with 100 μL lysis buffer for 20 minutes, and luciferase activity was measured using a BG-1 luminometer (GEM Biomedical).

### RNA-seq

Total RNA was isolated from HT1376 ^CT^ and HT1376 ^FOXP3 KO^ cells treated with IFNγ for 48 hours by Trizol (Thermo Fisher Scientific, Cat# 15596026). Triplicate samples were prepared for each condition. Libraries for RNA sequencing (RNA-seq) were prepared using the KAPA RNA HyperPrep Kit with RiboErase (Roche, Cat# KK8560) and sequenced on the NovaSeq S4 (Illumina) with 150 bp paired end reads at approximately 50 million reads per sample.

After RNA-seq, raw sequencing fastq files from HTSeq were aligned to the *Homo sapiens* GRCh38.103 reference genome using STAR. Contaminating bacterial *Mycoplasma* sequences were removed using Kraken. Aligned BAM files were converted into a non-normalized count matrix using htseq-count. Conversion from ENSEMBL ID to gene symbol using GRCh38.103 as the reference genome. After alignment, the input count matrix was normalized using the R package DeSeq2 median of ratios methods. Samples with read counts lower than 10 were omitted. Fold change calculations from the Deseq2-normalized count matrix were obtained by taking the log_2_fold of the ratio of normalized counts between two groups. Apeglm shrinkage provided more accurate estimates of the effect size. We reported events with *P* < 0.05 from DeSeq2. A *Z* score was calculated for each replicate and then averaged within groups. The R package pheatmap was used to plot heatmaps.

DeSeq2-normalized count matrix was used as input for gene set enrichment analysis. Because we had three replicates per experimental condition, we used the gene set permutation and performed 1,000 permutations to calculate the *P* value for gene lists. We compared our differentially expressed genes to the Molecular Signatures Database (MSigDB) collections H, C1, C2, C3, C4, C5, C6, C7, and C8.

### Animals

6 to 8 weeks old female C57BL/6 (RRID: MGI_2159769) and NOD-*scid* IL2Rgamma^null^ (NSG;RRID: IMSR JAX_005557) mice were acquired from the Jackson Laboratory. PARCB2 ^FOXP3Δ3^ and PARCB2 ^FOXP3^ cells (1 × 10^6^) were subcutaneously implanted in NSG animals. Comparing C57Bl6 and NSG backgrounds, HT1376 ^CT^ and HT1376 ^FOXP3 KO^ cells (2 × 10^5^) were resuspended in 50 μL PBS, mixed with 50 μL Matrigel (Corning, Cat# 356237), and injected into the flanks of mice. Tumor volume was measured every other day and calculated using the following formula: tumor length (mm) × tumor width (mm) × tumor height (mm) × 0.4. Mice were sacrificed when the first tumors reached 10 mm in diameter per protocol. Tumors were divided and fixed in formalin and flash-frozen in optimal cutting temperature compound. To examine metastases, MB49 ^CT^ and MB49 ^Foxp3 KO^ cells (1 × 10^6^) were intravenously introduced in the tail vein of C57Bl6 animals. At 2 weeks, animals were sacrificed, and lungs and liver were fixed in formalin and a portion flash frozen in optimal cutting temperature. Animals were maintained at the University of California, Los Angeles (UCLA) Division of Laboratory and Animal Medicine facilities under the approval and guidance of the Institutional Animal Care and Use Committee of the UCLA under the protocol #2010-023.

### IHC and immunofluorescence

Formalin-fixed sections were paraffin-embedded, sectioned at 5 μm, stained with hematoxylin and eosin, and examined by light microscopy. Human tumors were examined by immunofluorescence using anti-human FOXP3 (1:300, Invitrogen, Cat# 14-4774-82, RRID: AB_467552), anti-human PD-L1 (1:200, Cell Signaling, Cat# 13684T, RRID: AB_2687655), anti-human E-cadherin (1:200, Invitrogen, Cat# 4A2C7, RRID: AB_2925243), anti-human N-cadherin (1:100, SinoBiological, Cat# 11039-R020, RRID: AB_2860274), anti-human ICAM-1 (1:200, Cell Signaling, Cat# 67836S, RRID: AB_2799738), and secondary anti-mouse Alexa Fluor 488 (1:1,000, Vector Laboratories, Cat# DI-2488, RRID: AB_2307439) or secondary anti-rabbit Alexa Fluor 594 (1:1,000, Invitrogen, Cat# A11006, RRID: AB_141373). IHC was performed using anti-human chromogranin A (1:200, Sino Biological, Cat# 100256-T08, RRID: AB_2860064) or anti-human neuron-specific enolase (NSE) (1:200, Proteintech, Cat# 0149-1-AP, RRID: AB_2099180) and secondary anti-rabbit HRP (1:1,000, SouthernBiotech, Cat# 4050-05). Murine MB49 tumors were subject to immunofluorescence staining using anti-mouse PD-L1 (1:100 anti-mouse CD274, BioLegend, Cat# 124301, RRID: AB_961226), anti-mouse CD8 (1:100 R&D, Cat# MB116, RRID: AB_357497), anti-mouse CD4 (1:200, BD Pharmingen, Cat# 550280, RRID: AB_393575), anti-mouse IFNγ (1:100 R&D, Cat# MAB4851, RRID: AB_2123046), anti-mouse E-cadherin (1:200, Cell Signaling, Cat# 3195, RRID: AB_2123046), anti-mouse N-cadherin (1:200, BioLegend, Cat# 844701, RRID: AB_2750044), anti-mouse ICAM-1 (1:300, BioLegend, Cat# 116101, RRID: AB_313692), anti-mouse chromogranin A (1:300, Proteintech, Cat# 10529-1-AP, RRID: AB_2081122) or anti-mouse NSE (1:200, Proteintech, Cat# 10149-1-AP, RRID: AB_2099180), and secondary anti-rat Alexa Fluor 568 (1:1,000, Invitrogen, Cat# A11077, RRID: AB_141874) or anti-rat Alexa Fluor 488 (1:1,000, Invitrogen, Cat# 48262, RRID: AB_2896330) following standard protocols. Imaging was performed using Axio Imager 2 (Zeiss).

### Primary tumors

Primary urothelial bladder cancers [four high-grade muscle-invasive (HgT2) and two high-grade non–muscle invasive (HgT1)] were obtained following written informed consent under the approval of the UCLA Institutional Review Board (# 11-001363).

### Statistics

Two-tailed Student *t* test was performed when comparing two groups and one-way ANOVA when comparing multiple groups (significance *P* < 0.05). Number of replicates and *n* values are indicated. Error bars represent SD or SEM with *P* values as indicated.

### Data availability

Raw sequencing data are deposited to GEO GSE268243.

## Results

### FOXP3 mediates IFNγ-induced PD-L1 expression in bladder cancer cell lines dependent on JAK/STAT

**Figure 1:**
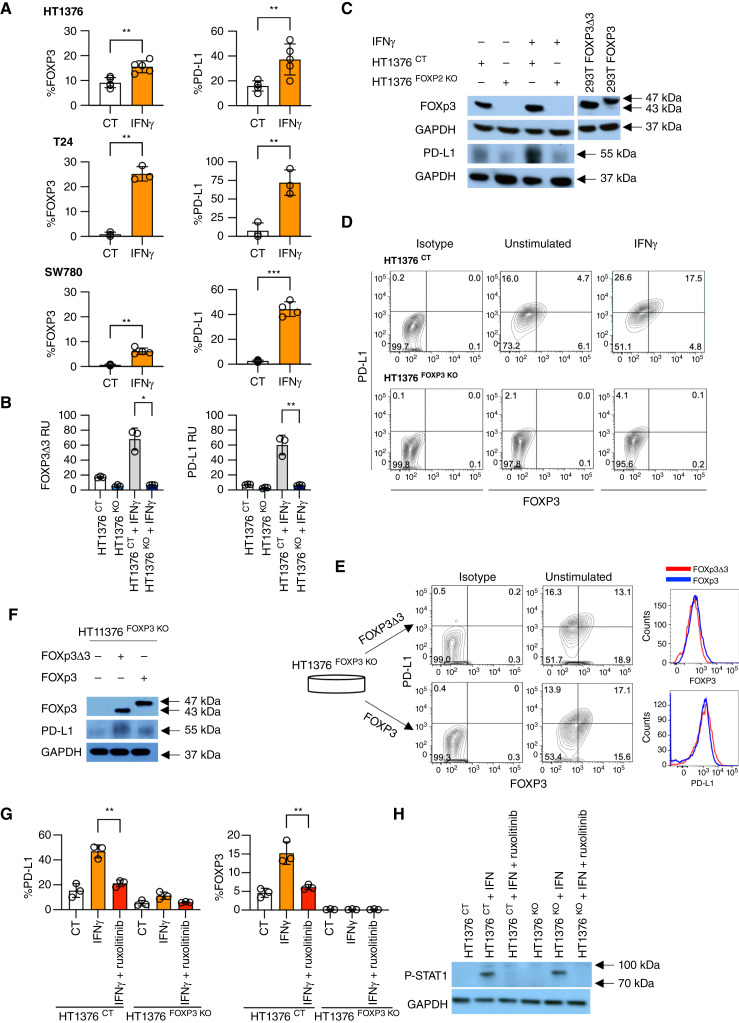
Dependency on FOXP3 in IFNγ-mediated PD-L1 expression. **A,** Expression of FOXP3 and PD-L1 in bladder cancer HT1376, T24, and SW780 cell lines without and with 100 ng/mL IFNγ stimulation by flow cytometry. The mean with SD of at least three independent experiments, *P* values indicated. *, *P* < 0.05; **, *P* < 0.01; ***, *P* < 0.001; ****, *P* < 0.0001. **B,** Expression of FOXP3 and PD-L1 in HT1376 ^CT^ and HT1376 ^FOXP3 KO^ bladder cancer cell lines without and with IFNγ stimulation by qPCR. The mean of triplicates and representative of three independent experiments, *P* values indicated. **C,** Expression of FOXP3 and PD-L1 in HT1376 ^CT^ and HT1376 ^FOXP3 KO^ lines without and with IFNγ stimulation by Western blotting. Sizes in kD as indicated. Sizes of FOXP3Δ3 and FOXP3 depicted by overexpression in 293T cells. Full Western blots shown in Supplementary Fig. S1. **D,** Flow cytometry of FOXP3 and PD-L1 expression in HT1376 ^CT^ and HT1376 ^FOXP3 KO^ lines without and with IFNγ stimulation. Representative of four independent experiments. **E,** Expression of FOXP3 and PD-L1 in rescue of a clonal HT1376 ^FOXP3 KO^ line by FOXP3Δ3 and FOXP3 overexpression. Representative of three independent experiments. **F,** Expression of FOXP3 and PD-L1 in HT1376 ^FOXP3 KO^ lines with reconstitution of FOXP3Δ3 and FOXP3 by Western blotting. Sizes in kD as indicated. Full Western blots shown in Supplementary Fig. S1. **G,** Flow cytometry of PD-L1 in HT1376 ^CT^ and HT1376 ^FOXP3 KO^ lines stimulated by IFNγ without and with the presence of JAK1/2 inhibitor ruxolitinib (20 μmol/L), *P* values indicated. **H,** Expression of phosphorylated STAT1 (P-STAT1) in HT1376 ^CT^ and HT1376 ^FOXP3 KO^ lines stimulated by IFNγ without and with the presence of JAK1/2 inhibitor ruxolitinib by Western blot. Full Western blots shown in Supplementary Fig. S1.

We first examined our prior RNAseq dataset in which FOXP3Δ3 was exogenously overexpressed in HT1376 bladder cancer cell lines and detected an increase of 5 to 8 folds in PD-L1 expression (14). As IFNγ is a key regulator for PD-L1 expression, we tested the ability for IFNγ to induce expression of endogenous FOXP3 and PD-L1 in HT1376, T24, and SW780 bladder cancer cell lines by flow cytometry. HT1376 cells have a high basal expression of FOXP3 and showed an approximately 50% induction of FOXP3 with a 2-fold induction of PD-L1 expression (Fig. 1A). Although T24 and SW780 lines have low basal expression of FOXP3, they exhibited a marked induction of both FOXP3 and PD-L1 upon IFNγ stimulation, with similar overall % expression as in HT1376 cells (Fig. 1A).

To determine if PD-L1 expression is dependent on FOXP3, we knocked out all isoforms of *FOXP3* by CRISPR/Cas9 targeting of exon 2 in HT1376 cells, which express high basal levels of FOXP3. Single-cell clones of representative HT1376 control KO (HT1376 ^CT^) and HT1376 FOXP3 CRISPR KO (HT1376 ^FOXP3 KO^) cell lines were stimulated with IFNγ and examined for expression of FOXP3 and PD-L1. In HT1376 ^FOXP3 KO^ cells, the absence of FOXP3 resulted in decreased basal PD-L1 expression and greatly impaired induction of PD-L1 by IFNγ, as measured by qPCR (Fig. 1B), Western blotting (Fig. 1C; Supplementary Fig. S1) and flow cytometry (Fig. 1D). To confirm our findings, we generated FOXP3 CRISPR KO in SW780 cells and similarly observed that FOXP3 is critical for the ability of IFNγ to induce PD-L1 expression (Supplementary Fig. S2A).

We previously have shown that bladder cancers predominantly express the FOXP3Δ3 isoform over the full-length FOXP3, including in HT1376 cells (14). To examine the ability of FOXP3 to rescue the KO phenotype, we reconstituted HT1376 ^FOXP3 KO^ cells with lentiviral vectors expressing FOXP3Δ3 or FOXP3. We found that both wild-type FOXP3 and FOXP3Δ3 have the similar capacity to restore induction of PD-L1 expression, as measured by flow cytometry (Fig. 1E) and Western blotting (Fig. 1F; Supplementary Fig. S1). Binding of IFNγ to the IFNγ receptor leads to activation of JAK1 and JAK2, phosphorylation and dimerization of STAT1, and subsequent translocation to the nucleus, enabling downstream gene expression (17). To determine if IFNγ activates FOXP3 through the JAK-STAT pathway, we stimulated HT1376 ^CT^ and HT1376 ^FOXP3 KO^ cells without and with the JAK1/2 inhibitor ruxolitinib. In HT1376 ^CT^ cells, ruxolitinib significantly curtained the ability for IFNγ to induce the expression of both PD-L1 and FOXP3 by flow cytometry, whereas minimal PD-L1 was induced by IFNγ irrespective of ruxolitinib in HT1376 ^FOXP3 KO^ cells, as expected (Fig. 1G). Loss of STAT1 phosphorylation by ruxolitinib in Western blotting confirmed adequate inhibition (Fig. 1H; Supplementary Fig. S1). Other cytokines that signal through the JAK/STAT pathway, including IL4 and IL10, were tested in HT1376 cells and did not show any significant induction of FOXP3 or PD-L1 (Supplementary Fig. S3).

### FOXP3 regulates IFNγ-dependent gene expression in immune activation and EMT

**Figure 2:**
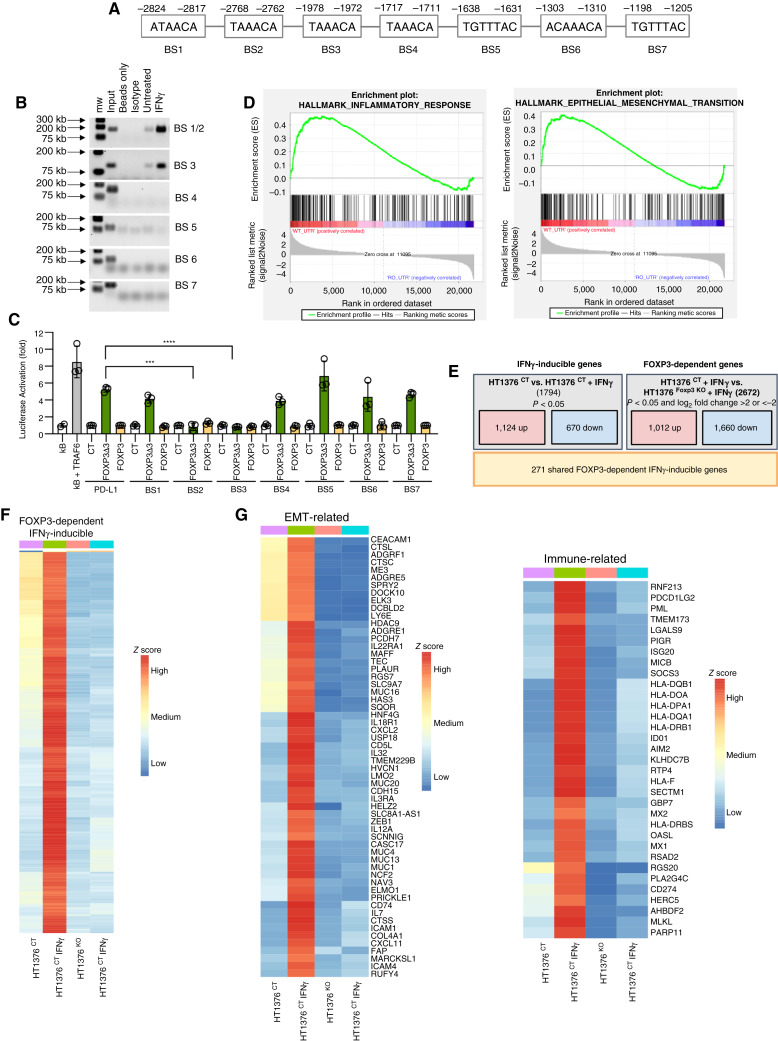
Binding of FOXP3 to the PD-L1 promoter and FOXP3-dependent IFNγ-mediated gene expression. **A,** Schematic of the PD-L1 promoter upstream of the transcription start site in HT1376 cells. **B,** ChIP PCR of the putative FOXP3-binding sites of the PD-L1 promoter. Input chromatin, beads only, isotype Ab and HT1376 cells untreated or treated with IFNγ as indicated. Sizes in kb as indicated. **C,** Activation of a PD-L1 reporter by overexpression of FOXP3Δ3 and FOXP3 by a luciferase reporter assay. All putative FOXP3-binding sites were individually mutated as shown. Activation of a NK-κB reporter by TRAF6 overexpression was used as a positive control. Control plasmid activation of each distinct promoter was set as baseline. Fold activation is shown and represents the mean of triplicates, *P* values indicated. *, *P* < 0.05; **, *P* < 0.01; ***, *P* < 0.001; ****, *P* < 0.0001. The results are representative of two independent experiments. **D,** GSEA of RNA-seq from clonal HT1376 ^CT^ and HT1376 ^FOXP3 KO^ lines performed in triplicates. FDRs and *P* values are shown in Supplementary Table S1. **E,** Derivation of FOXP3-dependent IFNγ-mediated genes in HT1376 ^CT^ and HT1376 ^FOXP3 KO^ lines without and with IFNγ stimulation. RNA-seq as the mean of triplicate samples. FDR and *P* values shown in Supplementary Tables S2 and S3. **F,** Heat map analysis of FOXP3-dependent IFNγ-mediated gene expression from HT1376 ^CT^ and HT1376 ^FOXP3 KO^ lines without and with IFNγ stimulation by *Z* score. *Z* scores are shown in Supplementary Fig. S4 and Supplementary Table S4. Normalized gene expression data are shown in Supplementary Table S5. **G,** Subset of heat maps highlighting genes important in immune regulation and EMT.

To understand the ability of FOXP3 to directly activate PD-L1, we screened the PD-L1 promoter sequence from the GRCh38 reference genome for the FOXP3-binding motifs A/G T/C AAACA and TGTTTAC and identified seven putative binding sites in the 3,000 base pairs upstream from the ATG transcriptional start site of the *PD-L1* promoter (Fig. 2A; refs. 18, 19). Using ChIP PCR, we tested these putative binding sites, showing binding at ATAAACA (−2,824 to −2,817) and TAAACA (−2,768 to −2,762), which were tested together because of their proximity. We also found binding at TAAACA (−1,978 to −1,972) but not at the other sequences (Fig. 2B). This is supported by a recent study showing binding of the PD-L1 promoter in pancreatic ductal adenocarcinoma cells by FOXP3 (20). To further validate these putative binding sites, we cloned this region of the PD-L1 promoter and tested individual binding sites through site-directed mutagenesis. Luciferase reporter assays confirmed the importance of BS2 and BS3, but not BS1, in the ability of FOXP3Δ3 to activate the PD-L1 promoter (Fig. 2C). We then examined the ability for FOXP3 to activate *PD-L1* and other genes through ChIP sequencing. However, we could not detect any specific binding of FOXP3 using all commercially available FOXP3 and FOXP3Δ3 antibodies in MT-2 T cell lines with high expression of endogenous FOXP3, wild-type HT1376, and IFNγ-stimulated HT1376 cells, suggesting limitations of the currently available antibodies (Supplementary Fig. S4).

Alternatively, to identify genes and pathways downstream of FOXP3 in context of IFNγ, we performed RNA-seq analysis of HT1376 ^CT^ and HT1376 ^FOXP3 KO^ lines without and with IFNγ stimulation. Initial gene set enrichment analysis of HT1376 ^CT^ and HT1376 ^FOXP3 KO^ cells identified FOXP3-dependent genes associated with inflammation and epithelial-to-mesenchymal transition (EMT) (Fig. 2D; Supplementary Table S1). We then identified IFNγ-regulated genes and FOXP3-dependent genes to capture overlapping genes that were both IFNγ-regulated and FOXP3-dependent (Fig. 2E; Supplementary Tables S2 and S3). We identified 271 genes that were both IFNγ-inducible and FOXP3-dependent depicted in a heatmap (Fig. 2F; Supplementary Fig. S5; Supplementary Tables S4 and S5). From this subset of FOXP3-dependent IFNγ-inducible genes, we highlighted genes related to inflammation, including *PD-L1* (CD274), other immune checkpoints, such as program cell death 1 ligand 2 (*PDCD1LG2*, also CD273/PD-L2/B7-DC) and indoleamine 2, 3-dioxygenase 1 (*IDO1*), and genes involved in EMT, including *CEACAM-1*, *ICAM-1*, *MUC1*, and *ZEB1* (Fig. 2G; refs. 21–26).

### Expression of FOXP3 in the genetically defined PARCB2 bladder cancer model acquires a more aggressive phenotype

**Figure 3:**
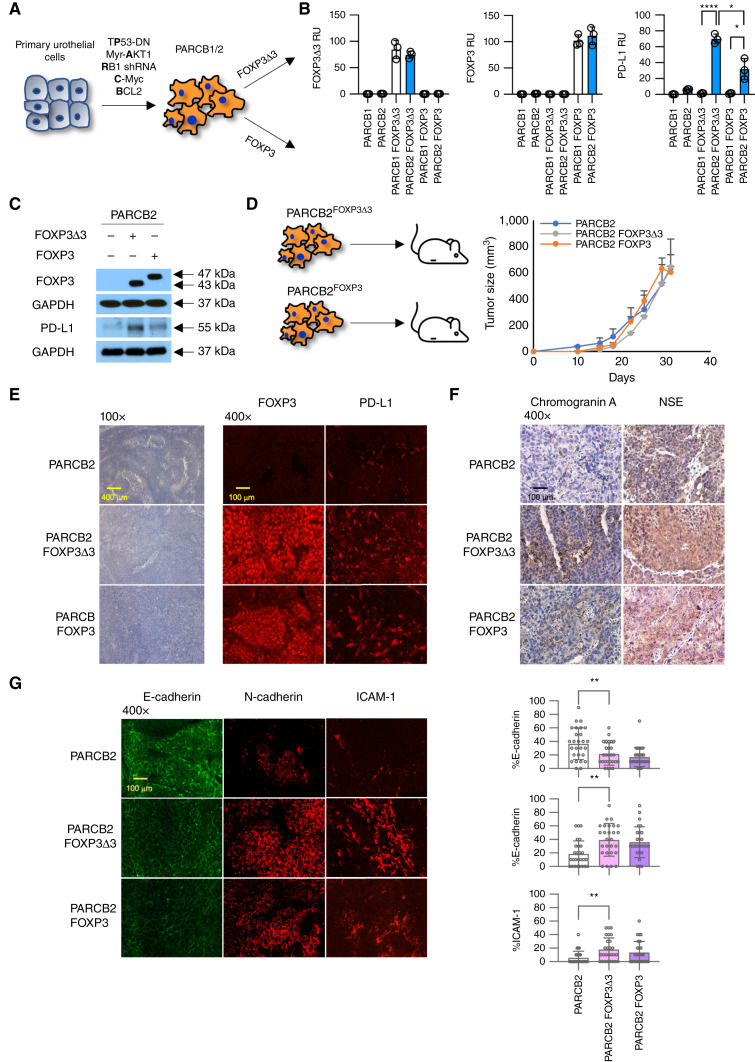
FOXP3 influences immune resistance and differentiation in the genetically defined PARCB2 bladder cancer model. **A,** Generation of clonal PARCB1/2 ^FOXP3Δ3^ and PARCB1/2 ^FOXP3^ lines through lentiviral infection of PARCB1 and PARCB2 cells. **B,** Expression of FOXP3Δ3, FOXP3, and PD-L1 in PARCB1/2, PARCB1/2 ^FOXP3Δ3^, and PARCB1/2 ^FOXP3^ representative clonal lines by qPCR. The mean and SD of two independent experiments, *P* values indicated. *, *P* < 0.05; **, *P* < 0.01; ***, *P* < 0.001; ****, *P* < 0.0001. **C,** Expression of FOXP3 and PD-L1 in PARCB2, PARCB2 ^FOXP3Δ3^, and PARCB2 ^FOXP3^ clonal lines by Western blotting. Sizes in kD as indicated. Full Western blots are shown in Supplementary Fig. S1. **D,** Growth of PARCB2, PARCB2 ^FOXP3Δ3^, and PARCB2 ^FOXP3^ cells in NSG mice. The mean and SEM of three animals per group. **E,** Histology and immunofluorescence of FOXP3 and PD-L1 in PARCB2, PARCB2 ^FOXP3Δ3^, and PARCB2 ^FOXP3^ tumors from NSG mice. Representative of three independent tumors. Exposure times listed in Supplementary Table S6. **F,** IHC of chromogranin A and NSE in PARCB2, PARCB2 ^FOXP3Δ3^, and PARCB2 ^FOXP3^ tumors from NSG mice. Representative of three independent tumors. **G,** Immunofluorescence of E-cadherin, N-cadherin, and ICAM-1 in PARCB2, PARCB2 ^FOXP3Δ3^, and PARCB2 ^FOXP3^ tumors from NSG mice. Representative of three independent tumors. Bar graphs represent the mean of 10 representative sections from three independent tumors. The mean and SD as shown, *P* values indicated. Exposure times listed in Supplementary Table S6.

We have previously developed a novel genetically defined bladder cancer model, PARCB, that transforms benign urothelial cells to aggressive bladder cancer with neuroendocrine features (16). This model is an ideal system to test the contribution of FOXP3Δ3 and FOXP3 expression with defined genetic drivers as opposed to the heterogeneity of traditional cell lines. We introduced lentiviral vectors expressing FOXP3Δ3 or FOXP3 into PARCB1 and PARCB2 lines to generate PARCB1/2 ^FOXP3Δ3^ and PARCB1/2 ^FOXP3^ lines, respectively (Fig. 3A). We have previously shown that PARCB1 lines are more similar to primary small-cell bladder cancers, whereas PARCB2 cells are more similar to urothelial bladder cancers (16). PARCB2 cells have both a low endogenous expression of FOXP3 and low basal expression of PD-L1. Compared with parental PARCB2 cells, overexpression of both FOXP3Δ3 and FOXP3 showed significant induction of PD-L1 gene expression by qPCR (Fig. 3B) and protein expression confirmed by Western blotting (Fig. 3C; Supplementary Fig. S1). In contrast, PARCB1 lines expressed undetectable levels of FOXP3 and PD-L1. Overexpression of FOXP3Δ3 or FOXP3 in PARCB1 lines led to minimal induction of PD-L1 expression, suggesting additional factors perhaps required in small-cell cancers that may be critical in expression of PD-L1 (Fig. 3B).

To examine the influence of FOXP3 in tumor architecture, we implanted PARCB2 ^FOXP3Δ3^ and PARCB2 ^FOXP3^ lines into immunocompromised NOD-*scid* IL2Rgamma^null^ (NSG) mice. We found no significant differences in tumor sizes (Fig. 3D). Interestingly, histology of the tumors showed more densely packed cells consistent with a neuroendocrine phenotype and decreased papillary features in tumors derived from PARCB2 ^FOXP3Δ3^ and PARCB2 ^FOXP3^ compared with parental PARCB2 cell lines (Fig. 3E). As expected, we observed increased immunostaining of both FOXP3 and PD-L1 in PARCB2 ^FOXP3Δ3^ and PARCB2 ^FOXP3^ tumors (Fig. 3E; Supplementary Table S6). The bias toward a neuroendocrine phenotype was confirmed by increased expression of chromogranin A and NSE in tumors derived from PARCB2 ^FOXP3Δ3^ and PARCB2 ^FOXP3^ cell lines (Fig. 3F; ref. 27). Characteristic of the EMT phenotype, we observed a loss of E-cadherin expression as well as increased N-cadherin and ICAM-1 expression by overexpression of either FOXP3Δ3 or FOXP3 (Fig. 3G; Supplementary Table S6; ref. 28).

### Loss of Foxp3 in a murine bladder cancer model leads to smaller tumors and fewer metastases

**Figure 4:**
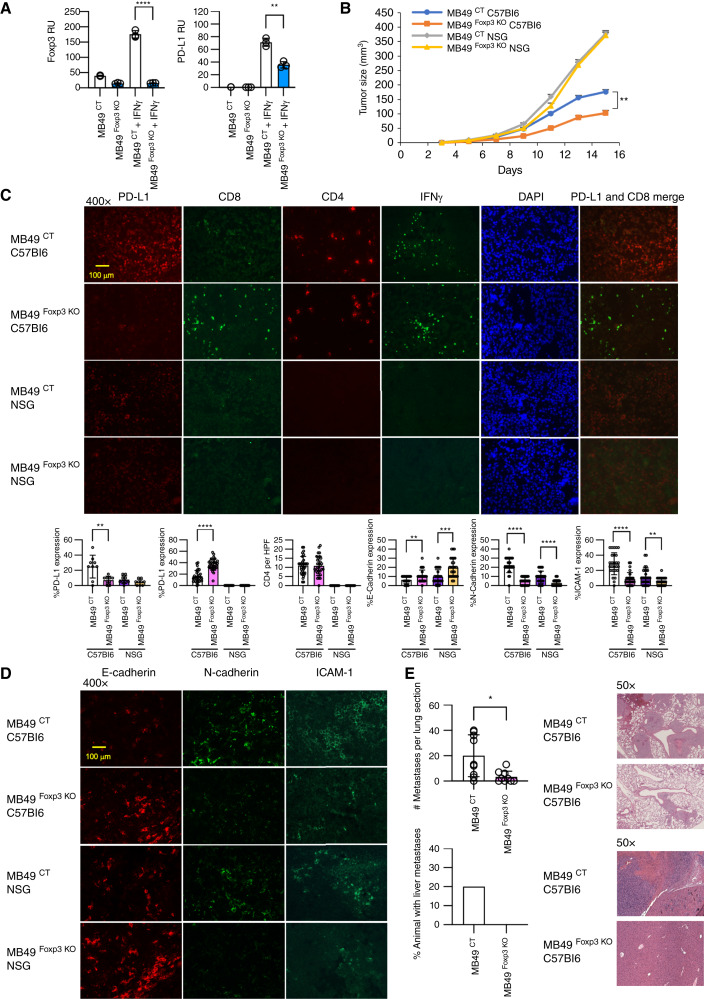
Impact of immune system on *Foxp3*-mediated tumor growth in a murine model of bladder cancer. **A,** Expression of *Foxp3* and *PD-L1* in wild-type control KO MB49 (MB49 ^CT^) and *Foxp3* CRISPR KO MB49 (MB49 ^Foxp3 KO^) bladder cancer cell lines without and with IFNγ stimulation by qPCR. The mean of triplicates and representative of three independent experiments, *P* values indicated. *, *P* < 0.05; **, *P* < 0.01; ***, *P* < 0.001; ****, *P* < 0.0001. **B,** Growth of MB49 ^CT^ and MB49 ^Foxp3 KO^ tumors in C57Bl6 and NSG animals. The means and SEMs of six animals per group, *P* values indicated. **C,** Immunofluorescence of PD-L1, CD8, CD4, and IFNγ in MB49 ^CT^ and MB49 ^Foxp3 KO^ tumors derived from C57Bl6 and NSG animals. DAPI staining and coexpression of PD-L1 and CD8 depicted. Images are representative of three independent tumors. Bar graphs represent expression of protein as indicated in MB49 ^CT^ and MB49 ^Foxp3 KO^ tumors derived from C57Bl6 and NSG animals for **C** and **D**. The mean and SD of 10 representative sections from three independent tumors, *P* values as indicated. Exposure times listed in Supplementary Table S6. **D,** Immunofluorescence of N-cadherin, E-cadherin, and ICAM-1 in MB49 ^CT KO^ and MB49 ^Foxp3 KO^ tumors derived from C57Bl6 and NSG animals. Images are representative of three independent tumors. Exposure times listed in Supplementary Table S6. **E,** Decreased metastases of MB49 bladder cancer in the absence of *Foxp3*. Metastatic lesions from a cross-section of a single lung from C57Bl6 mice intravenously injected with MB49 ^CT^ and MB49 ^Foxp3 KO^ cells. The means and SEMs are shown from 10 animals per group, *P* values indicated. Representative H&E sections of the lungs are shown. Percentage of animals that developed liver metastases from C57Bl6 mice intravenously injected with MB49 ^CT^ and MB49 ^Foxp3 KO^ cells are shown. Representative H&E sections of liver are shown.

A limitation of the PARCB model is the use of xenografts and immune-deficient NSG animals. To examine the contribution of an intact immune system, we utilized the murine bladder cancer cell line MB49. Compared with the human gene, murine *Foxp3* lacks the noncoding first exon and does not express isoforms (29, 30). We generated MB49 control CRISPR (MB49 ^CT^) and MB49 lines with FOXP3 deficiency by CRISPR/Cas9 (MB49 ^Foxp3 KO^). MB49 cells have a low basal expression of FOXP3 and PD-L1, whereas stimulation of MB49 cells by IFNγ leads to upregulation of both FOXP3 and PD-L1, consistent with our findings in human bladder cancer cell lines. KO of FOXP3 in MB49 cells decreased PD-L1 gene expression by qPCR (Fig. 4A) and markedly decreased protein expression by Western blotting and flow cytometry (Supplementary Fig. S1,S6A and S6B).

To examine the influence of the immune system *in vivo*, we compared the growth of MB49 ^CT^ and MB49 ^Foxp3 KO^ cells in a s.c. implantation model on syngenic C57Bl6 and immunodeficient NSG mice. In the immunocompetent C57Bl6 background, syngeneic tumors from MB49 ^CT^ cells grew larger than MB49 ^Foxp3 KO^ cells, exemplifying how the presence of FOXP3 confers a more aggressive phenotype (Fig. 4B). In the absence of an intact host immune system, tumors from MB49 ^CT^ and MB49 ^Foxp3 KO^ cells grew at a similar rate, a result akin to PARCB2 cells in NSG animals, demonstrating the importance of an intact immune system in FOXP3-mediated functions (Fig. 4B).

If a decrease in PD-L1 mediated by loss of FOXP3 contributed to the immune-dependent suppression of tumor growth, we would predict an increase in infiltration of CD8^+^ cells. Immunofluorescence staining of tumors confirmed this hypothesis with a decrease in PD-L1, an increase in CD8^+^ T cells, and similar levels of CD4^+^ T cells in MB49 ^Foxp3 KO^ compared with MB49 ^CT^ tumors in the C57Bl6 background. Similar expressions of IFNγ were observed consistent with an insensitivity to IFNγ as a mechanism for the differences seen in the absence of FOXP3 (Fig. 4C; Supplementary Table S6). As expected, no T cells or IFNγ were expressed in tumors from NSG animals.

The ability for FOXP3 to mediate EMT in MB49 cells, which have a high metastatic potential, was investigated (15). In a similar trend to PARCB cells, loss of FOXP3 led to an increase in E-cadherin expression and decrease in N-cadherin and ICAM-1 expressions (Fig. 4D; Supplementary Table S6). To functionally test the influence of FOXP3 in EMT, we examined the metastatic potential of MB49 cells with and without FOXP3. Syngeneic C57Bl6 mice were intravenously implanted with MB49 ^CT^ and MB49 ^Foxp3 KO^ cells, and the lungs and liver were examined for metastases. In wild-type MB49 ^CT^ mice, we noted an approximately 6-fold greater number of lung metastases compared with mice implanted with MB49 cells lacking FOXP3. In the liver, 20% of mice developed metastases implanted with MB49 ^CT^ cells, whereas no mice implanted with MB49 ^Foxp3 KO^ cells developed tumors (Fig. 4E). Unlike in PARCB cells, expression of the neuroendocrine marker chromogranin A was unchanged in MB49 ^CT^ and MB49 ^Foxp3 KO^ tumors, with minimal expression of NSE noted, suggesting that FOXP3 is not a driver of the neuroendocrine phenotype (Supplementary Fig. S6C).

### FOXP3 shared across other activators of PD-L1, including cisplatin chemotherapy

**Figure 5:**
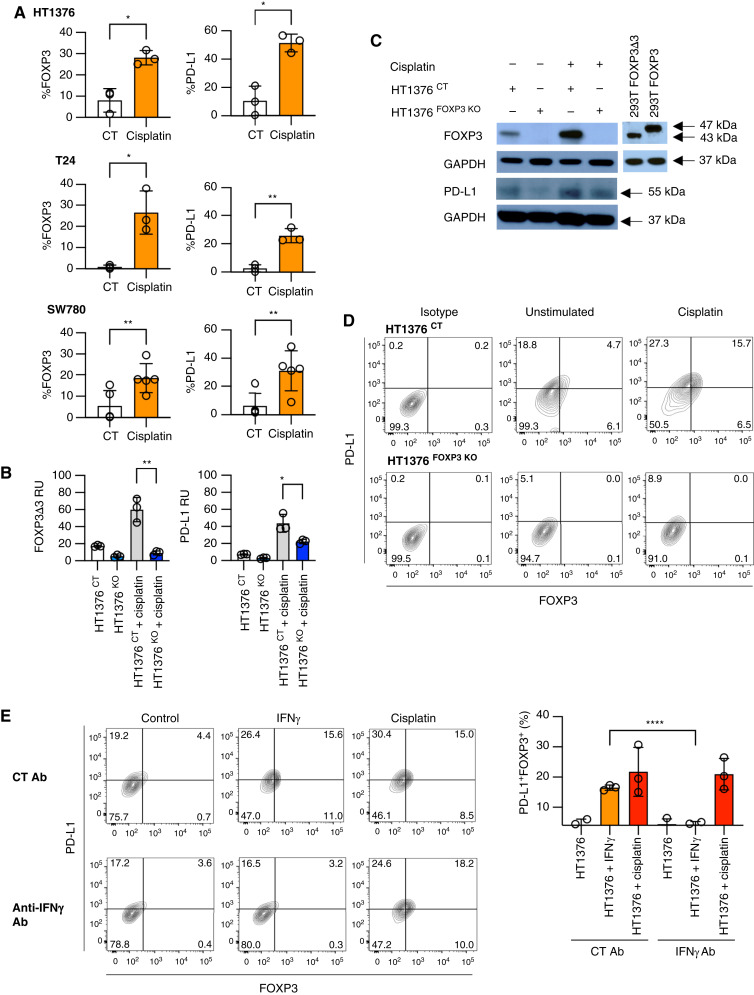
Cisplatin-mediated PD-L1 expression dependent on FOXP3 in bladder cancer cells. **A,** Expression of FOXP3 and PD-L1 in multiple bladder cancer cell lines without and with cisplatin stimulation by flow cytometry. The mean of at least three independent experiments, *P* values indicated. *, *P* < 0.05; **, *P* < 0.01; ***, *P* < 0.001; ****, *P* < 0.0001. **B,** Expression of *FOXP3* and *PD-L1* in HT1376 ^CT^ and HT1376 ^FOXP3 KO^ cell lines without and with cisplatin stimulation by qPCR. The mean and SD of triplicates and representative of three independent experiments, *P* values indicated. **C,** Expression of FOXP3 and PD-L1 in HT1376 ^CT^ and HT1376 ^FOXP3 KO^ lines without and with IFNγ stimulation by Western blotting. Sizes are in kD as indicated. Full Western blots are shown in Supplementary Fig. S1. **D,** Flow cytometry of FOXP3 and PD-L1 expression of HT1376 ^CT^ and HT1376 ^FOXP3 KO^ lines without and with IFNγ stimulation. Representative of three independent experiments. **E,** Expression of FOXP3 and PD-L1 in HT1376 ^CT^ cells stimulated by IFNγ or cisplatin with control or depleting antibodies to IFNγ by flow cytometry. Representative of three independent experiments. Bar graphs represent the mean and SD of three independent experiments, *P* values indicated.

We next asked if FOXP3 is common to multiple upstream activators of PD-L1 besides IFNγ. We tested the contribution for chemotherapy as an activator as induction of PD-L1 expression following chemotherapy has been observed clinically (31). We selected the platinum-based agent cisplatin which is ubiquitous to the treatment of patients with metastatic urothelial bladder cancer. We stimulated HT1376, T24, and SW780 cell lines with cisplatin and noted significant induction of both FOXP3 and PD-L1 in all lines tested (Fig. 5A). To determine if induction of PD-L1 by cisplatin is mediated by FOXP3, we treated HT1376 ^CT^ and HT1376 ^FOXP3 KO^ lines with cisplatin and noted decreased PD-L1 induction in the FOXP3 KO cells, as measured by qPCR (Fig. 5B), Western blotting (Fig. 5C; Supplementary Fig. S1), and flow cytometry (Fig. 5D). A similar trend was seen in SW780 ^FOXP3 KO^ lines (Supplementary Fig. S2B). To test if cisplatin induction of PD-L1 was mediated by IFNγ, we treated HT1376 cells with an IFNγ-depleting Ab. IFNγ depletion prevented PD-L1 induction by IFNγ but had no effect on PD-L1 induction by cisplatin (Fig. 5E). Thus, FOXP3-dependent cisplatin induced PD-L1 independent to IFNγ, suggesting that the transcriptional program of FOXP3 represents a shared pathway used by multiple activators of PD-L1 expression.

### Correlation of FOXP3 and PD-L1 in primary bladder cancers

**Figure 6:**
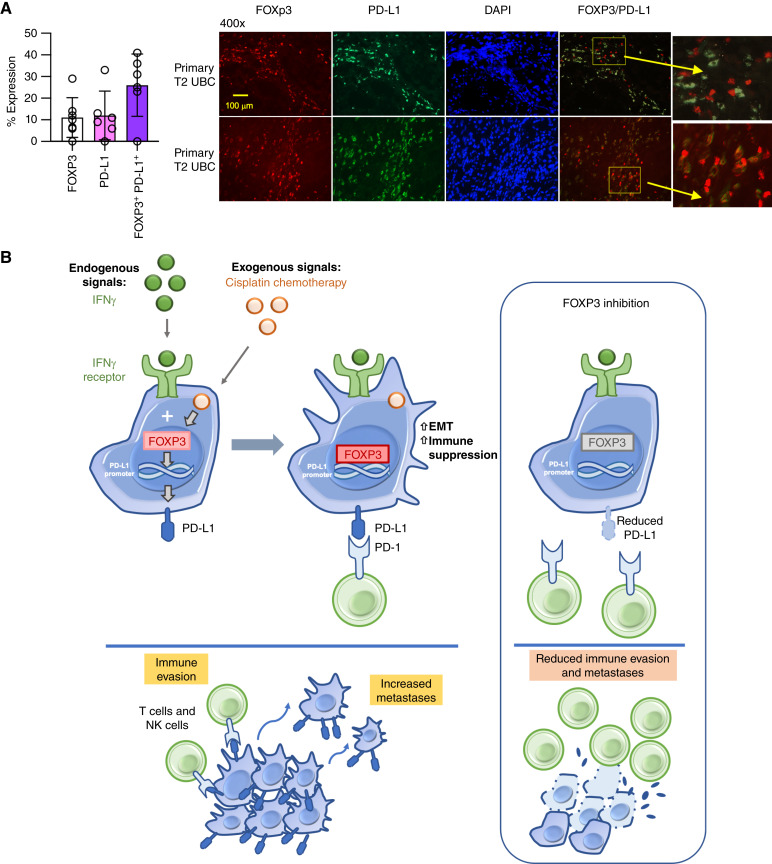
Correlation between FOXP3 and PD-L1 expression. **A,** Expression of pan-FOXP3 and PD-L1 and their co-localization was examined by immunofluorescence in primary high-grade urothelial carcinoma of the bladder. The mean of six primary tumors and representative immunofluorescence shown. Exposure times are listed in Supplementary Table S6. **B,** Schematic depicting the role of FOXP3 in cancer cells. FOXP3 mediates a transcriptional program through multiple activators that influences immune suppression and EMT. Activation of FOXP3 leads to immune evasion and increased metastases. On the right, potential inhibition of FOXP3 may provide therapeutic benefit.

These findings point to a critical role of FOXP3 in IFNγ-mediated PD-L1 expression with additional functional roles in promoting EMT in bladder cancer cells. To confirm our findings in primary tumors, we examined the correlation of FOXP3 and PD-L1 in six high-grade bladder cancer specimens and showed colocalization of FOXP3 and PD-L1 expression (Fig. 6A; Supplementary Table S6).

## Discussion

PD-L1 is an immune checkpoint that plays a central role in restraining T-cell activation as well as in the immune escape of cancers. As such, immune checkpoint inhibitors through blocking antibodies against PD-1 or PD-L1 augment antitumor T-cell responses and play a central role in immunotherapy across multiple cancer types (9). PD-L1 expression is dynamic and regulated through a multiplicity of mechanisms. This includes transcriptional regulation by inflammatory cytokines, including IFNγ, IL1β, IL4, IL6, and IL10, and oncogenic signaling pathways such as MYC, EGFR via STAT3, and β-catenin to highlight a few (13, 32–34). Additional mechanisms of PD-L1 regulation include post-transcriptional regulation by miRNAs and long ncRNAs, mTOR regulation of PD-L1 translation, and post-translational control through glycosylation or protection by CMTM6 from lysosomal degradation (35–39).

Previously, we have shown the clinical correlation of FOXP3 expression to worse overall survival in patients with urothelial bladder cancer as well as the induction of genes that mediate cancer cell differentiation and chemotherapy resistance (14). Here, using multiple human and murine cell lines, we have shown that FOXP3 is critical in regulating IFNγ-mediated PD-L1 expression. We showed direct binding of FOXP3 to the PD-L1 promoter at specific FOXP3-binding motifs, confirming results initially described in pancreatic cancer cells (20). In murine MB49 cells, loss of FOXP3 led to smaller tumors, decreased expression of PD-L1, and increased CD8^+^ T cells in tumor tissues *in vivo* but preserved tissue expression of IFNγ, supporting loss of IFNγ responsiveness. We examined other regulators of PD-L1 and showed that FOXP3 also mediated cisplatin-induced PD-L1 expression, suggesting a broader role as a common downstream mediator of PD-L1. Interestingly, we had previously shown that FOXP3 imparted cisplatin resistance *in vitro* (14).

Urothelial bladder cancer is composed of multiple molecular subtypes (40–42). These subtypes have imparted distinct susceptibilities and resistance to treatments, such as to chemotherapy or checkpoint blockage immunotherapy (43, 44). Previously, our PARCB model showed that from a defined molecular background, a single urothelial cell may be capable of developing into multiple histologic cancer subtypes, including neuroendocrine, urothelial, and squamous, differentiated in an immune compromised background *in vivo* (16). Here, we showed that expression of FOXP3 in our PARCB2 model skews toward a histologically more aggressive phenotype with a further increase in neuroendocrine markers and loss of papillary features.

In examining the role of FOXP3 in tumors cells, we described a gene expression program that promotes cancer cells through upregulation of PD-L1 expression and other inflammation-related genes and genes involved in EMT. FOXP3 has been shown to promote EMT in non–small cell lung cancer cells through a Wnt/β-catenin pathway (45). PD-L1 itself is associated with EMT, both directly inducing EMT as well as its own expression regulated by the induction of EMT (46). Of particular interest, additional checkpoints, including PD-L2 and IDO1, may also be regulated by FOXP3. FOXP3’s role in cancer cells may function in a broader role to promote immune resistance in cancer cells as well as instigating tumor metastases (Fig. 6B). Accordingly, loss of FOXP3 in our murine tumor model led to drastically fewer metastases to the lung and liver compared with the wild-type counterpart.

This study has several limitations that will provoke future studies. First, we could not achieve binding of endogenous or exogenous FOXP3 sufficient for ChIP sequencing analysis, which would complement our gene expression studies on the broader role of FOXP3 in cancer cells. This was attributed to limitations of current antibodies (47). Second, we showed no differences between FOXP3 versus FOXP3Δ3 in the ability to rescue IFNγ-mediated activation of PD-L1 in HT1376 cells, with a decreased ability of FOXP3 to activate PD-L1 in PARCB2 cells. Although the role of isoforms in Tregs has been unclear, a recent study explored the interdependence of FOXP3 and FOXP3ΔE2 (also referred to as Δ3 with noncoding exon 1), revealing a unique transcriptional program driven by FOXP3ΔE2 to foster Treg stability (29). The skewing of elevated FOXP3Δ3 to FOXP3 ratios in cancer cells likely presents a survival advantage to the cancer cell. Future investigation into the balance of isoform expression in cancer cells will complement the knowledge in Tregs. This may have functional impact when considering targeting FOXP3 in cancer therapy both with respect to cancer cells as well as in T regs.

In summary, we describe the role of FOXP3 in bladder cancer cells that promotes a more aggressive phenotype both histologically as well as functionally. Although we focused on its role in mediating PD-L1 expression, FOXP3 clearly plays a more expansive role in regulating gene expression across a panel of immune- and EMT-related genes in cancer cells. Whereas current checkpoint inhibitor therapies utilize blocking monoclonal antibodies against the ligand or its cognate receptor PD-1, knowledge of PD-L1 regulation may provide novel avenues for its targeting, including episomal sequestrating, ubiquitination, and targeting transcription regulation of PD-L1. Based on our findings, potential targeting of FOXP3 may impact PD-L1 expression and other immune checkpoints, as well as other cancer cell intrinsic functions in promoting EMT. The consequence of any therapy against FOXP3 will need to acknowledge its effect in cancer cells as well as well-established roles in T regulatory cell function, which in itself has been a focus of cancer therapy (48).

## Supplementary Material

Supplementary Figure 1 Complete Western blot membranes for respective figures

Supplementary Figure 2 Dependency on FOXP3 in IFNgamma- and cisplatin-mediated PD-L1 expression in SW780 cells

Supplementary Figure 3 Minimal activation of PD-L1 by IL-4 and IL-10 compared to IFNgamma in HT1376 cells

Supplementary Figure 4 FOXP3 antibodies not sufficient for ChIP Sequencing

Supplementary Figure 5 Composite Z-scores of FOXP3-dependent IFNgamma-inducible genes in HT1376 cells

Supplementary Figure 6 Generation of MB49 FOXP3 knockout cells by flow cytometry and Western blot. Immunofluorescence of chromogranin A and NSE.

Supplementary Table 1 GSEA FDR and p-values

Supplementary Table 2 FDRs and p-values of IFNgamma induced genes

Supplementary Table 3 FDRs and p-values of FOXP3-dependent genes

Supplementary Table 4 Genes and level of expression by z score of 271 IFNgamma induced and FOXP3-dependent genes

Supplementary Table 5 Normalized gene expression of RNA sequencing

Supplementary Table 6 Exposure times of antibodies used for immunofluorescence
