# Supplementary material for: Role of Forkhead Box P3 in IFNγ-Mediated PD-L1 Expression and Bladder Cancer Epithelial-to-Mesenchymal Transition
Source: Cancer Res Commun. 2024 Aug 26;4(8):2228–41. doi: 10.1158/2767-9764.CRC-23-0493 (PMC11345674; doi:10.1158/2767-9764.CRC-23-0493)

Supplementary Figure 1

Fig 1C

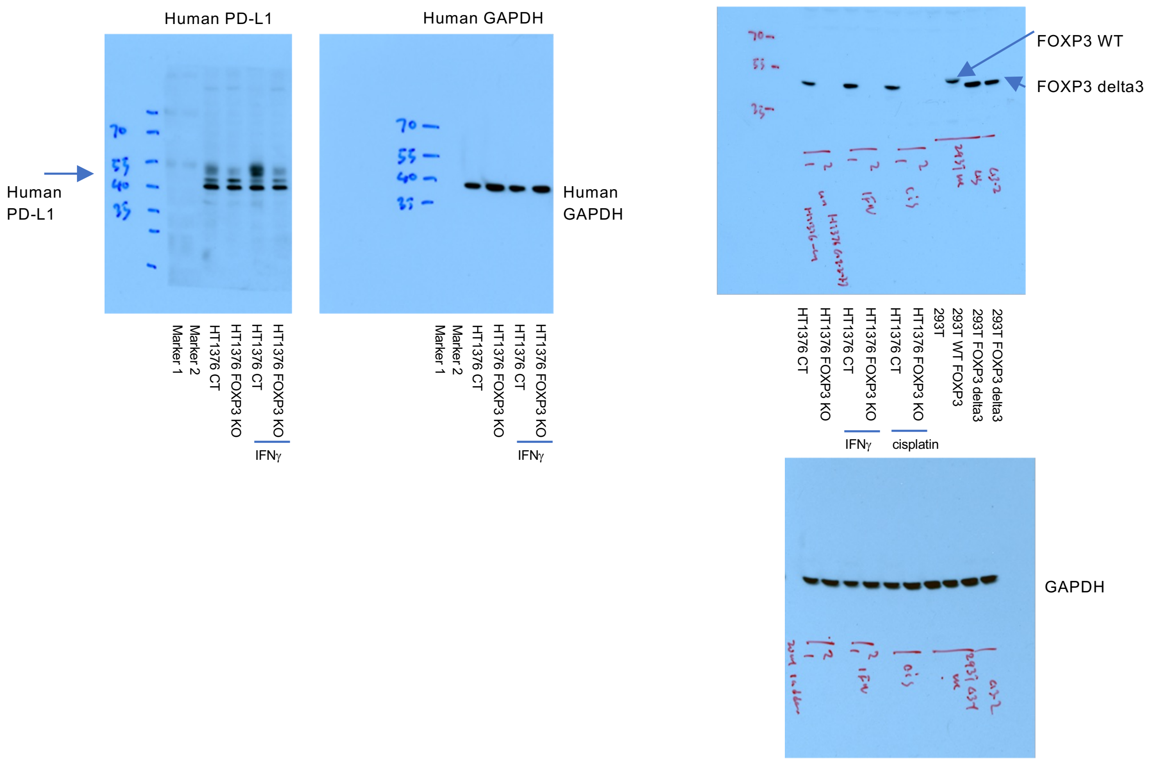

Fig 1F

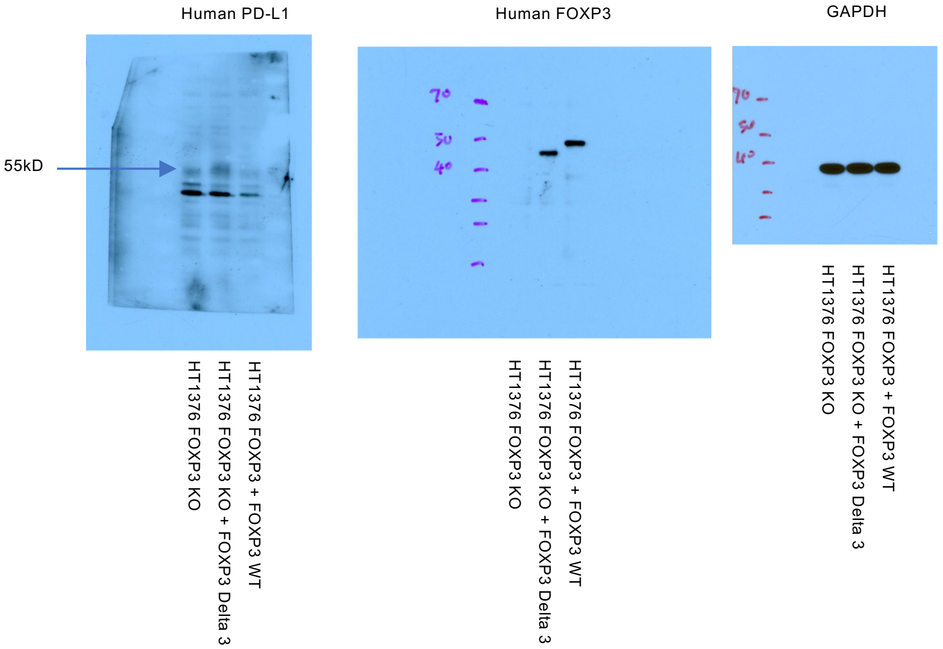

Supplementary Figure 1. Complete Western blot membranes for respective figures.

Fig 1H

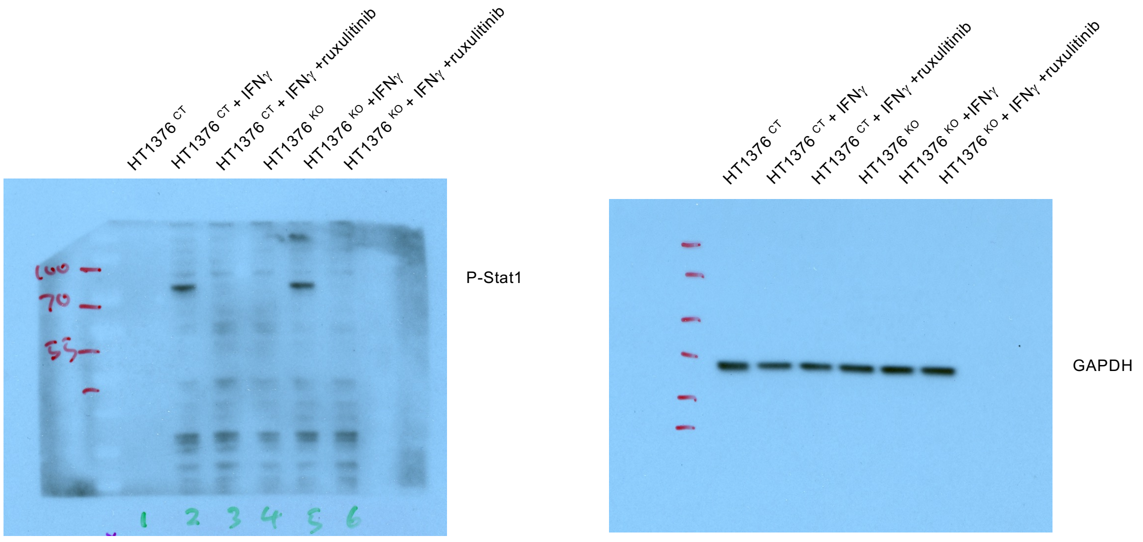
HT1376 CT
HT1376 CT + IFNγ
HT1376 CT + IFNγ +ruxulitinib
HT1376 KO
HT1376 KO +IFNγ
HT1376 KO + IFNγ +ruxulitinib
P-Stat1
GAPDH

Fig 3C

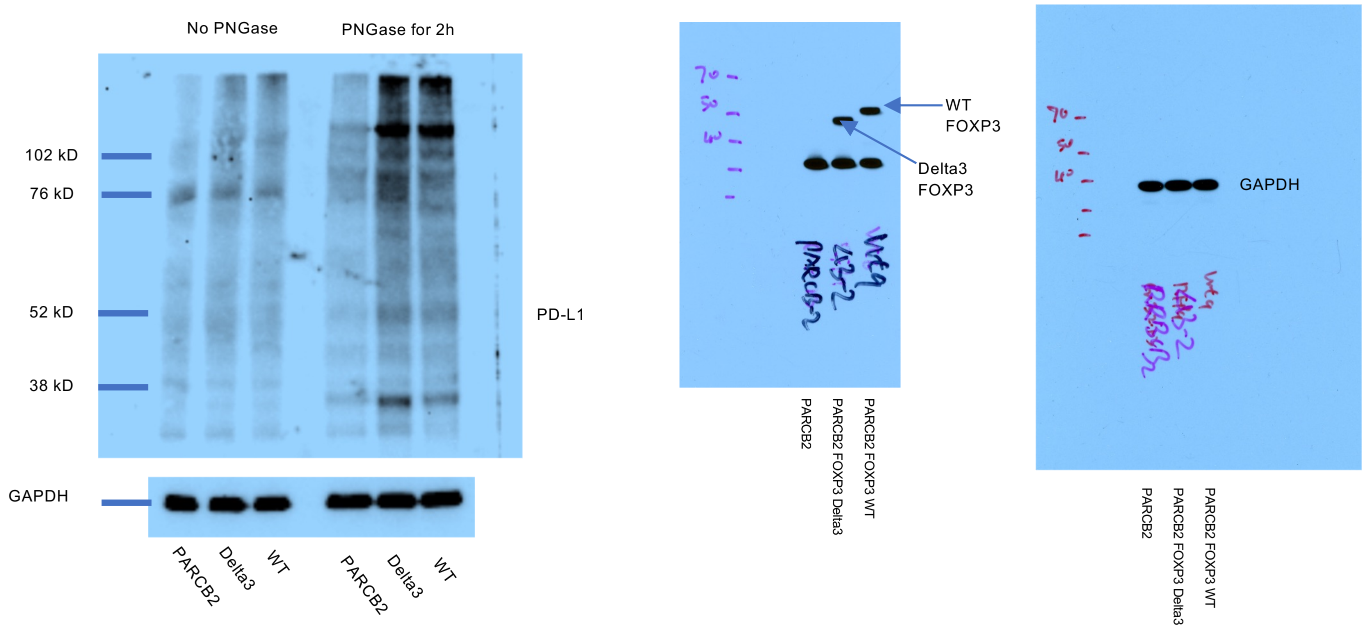
No PNGase
PNGase for 2h
102 kD
76 kD
52 kD
38 kD
PD-L1
GAPDH
PARCB2
Delta3
WT
PARCB2
Delta3
WT
WT
FOXP3
Delta3
FOXP3
PARCB2
PARCB2 FOXP3 Delta3
PARCB2 FOXP3 WT
GAPDH
PARCB2
PARCB2 FOXP3 Delta3
PARCB2 FOXP3 WT

Fig 5C

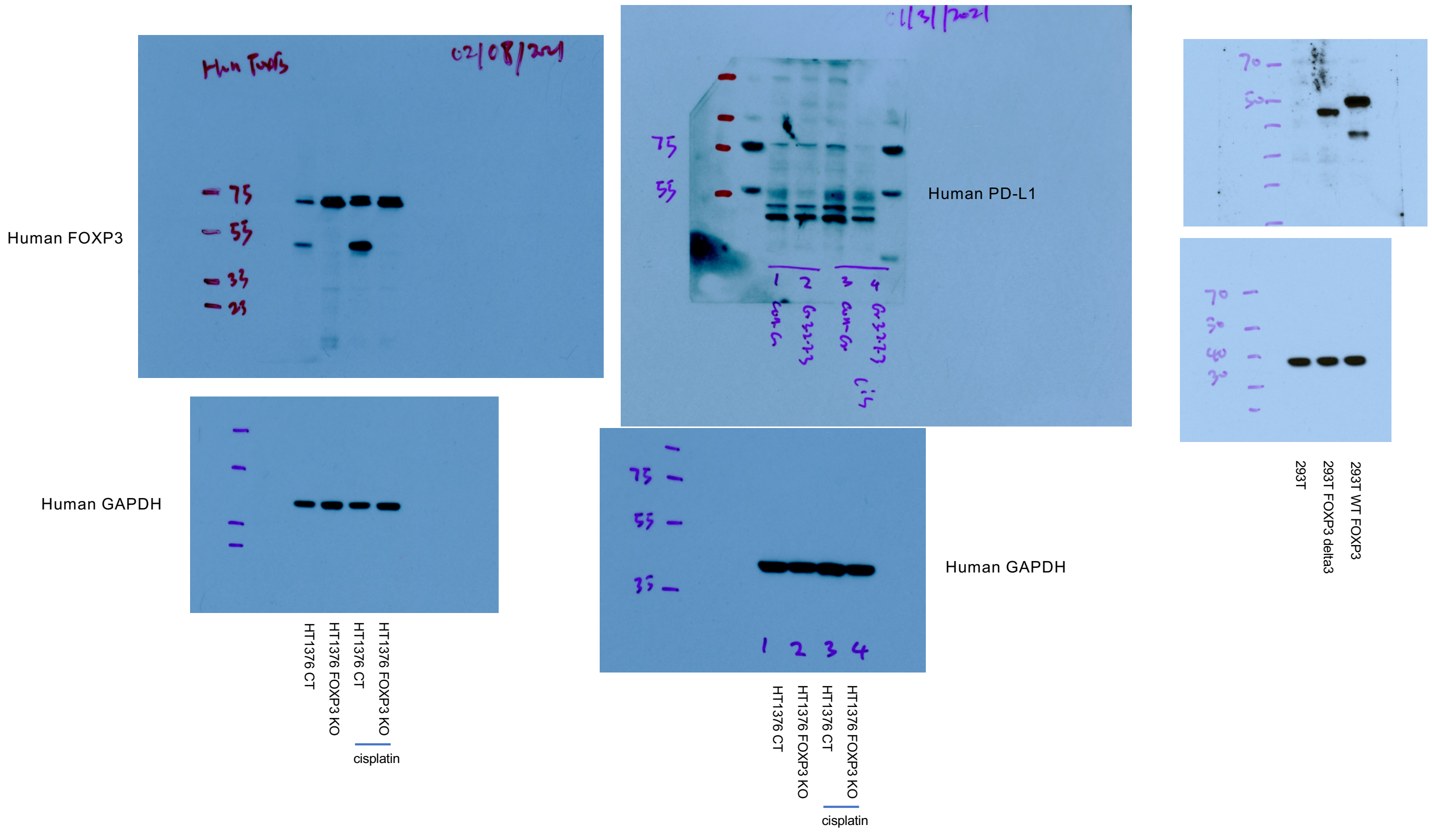

Supplementary Figure 6B

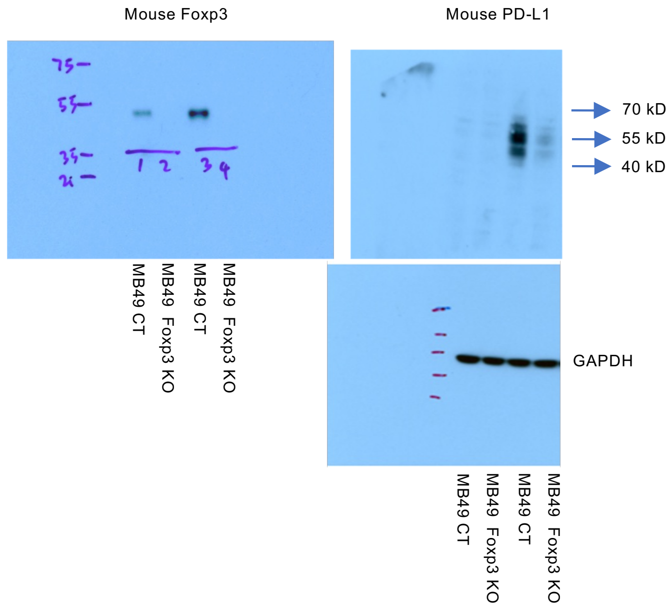

Supplement: Supplementary Figure 1 — Complete Western blot membranes for respective figures [file crc-23-0493_supplementary_figure_1_suppsf1.pdf]
