# Supplementary material for: Role of Forkhead Box P3 in IFNγ-Mediated PD-L1 Expression and Bladder Cancer Epithelial-to-Mesenchymal Transition
Source: Cancer Res Commun. 2024 Aug 26;4(8):2228–41. doi: 10.1158/2767-9764.CRC-23-0493 (PMC11345674; doi:10.1158/2767-9764.CRC-23-0493)
Supplement: Supplementary Figure 2 — Dependency on FOXP3 in IFNgamma- and cisplatin-mediated PD-L1 expression in SW780 cells [file crc-23-0493_supplementary_figure_2_suppsf2.pdf]

Supplementary Figure 2

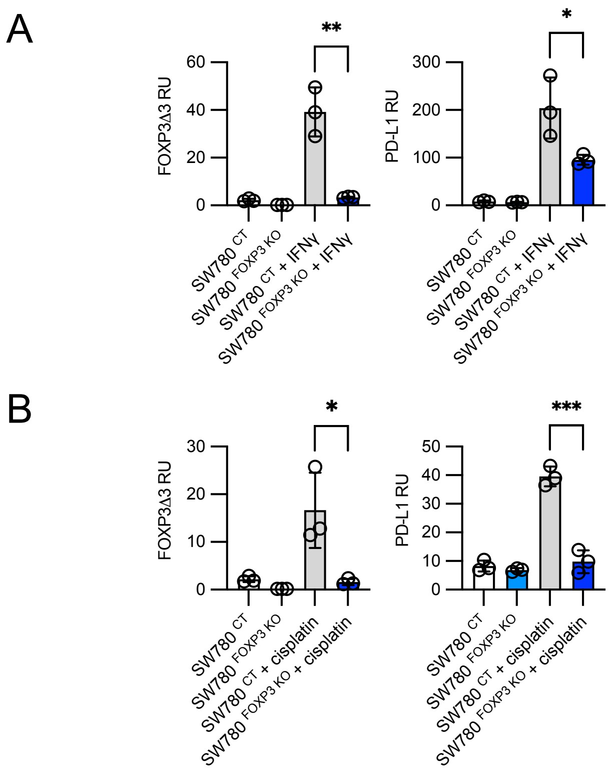

Supplementary Figure 2. Dependency on FOXP3 in IFNγ- and cisplatin-mediated PD-L1 expression in SW780 cells. (A) Expression of FOXP3 and PD-L1 in wild-type CRISPR control knockouts (SW780 $^{\mathrm{FOXP3\ CT}}$) and pan-FOXP3 CRISPR knockout (SW780$^{\mathrm{FOXP3\ KO}}$) bladder cancer cell lines without and with 100 ng/mL IFNγ stimulation by qPCR. Mean of triplicates and representative of 3 independent experiments, p values indicated. * $p < 0.05$, ** $p < 0.01$, *** $p < 0.001$, **** $p < 0.0001$. (B) Expression of FOXP3 and PD-L1 in SW780 $^{\mathrm{FOXP3\ CT}}$ and SW780$^{\mathrm{FOXP3\ KO}}$ bladder cancer cell lines without and with 20 µm cisplatin stimulation by qPCR. Mean of triplicates and representative of 2 independent experiments, p values indicated.
