# Supplementary material for: Role of Forkhead Box P3 in IFNγ-Mediated PD-L1 Expression and Bladder Cancer Epithelial-to-Mesenchymal Transition
Source: Cancer Res Commun. 2024 Aug 26;4(8):2228–41. doi: 10.1158/2767-9764.CRC-23-0493 (PMC11345674; doi:10.1158/2767-9764.CRC-23-0493)
Supplement: Supplementary Figure 3 — Minimal activation of PD-L1 by IL-4 and IL-10 compared to IFNgamma in HT1376 cells [file crc-23-0493_supplementary_figure_3_suppsf3.pdf]

Supplementary Figure 3

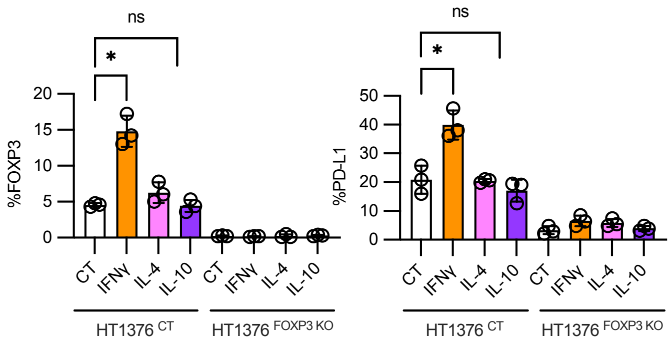

Supplementary Figure 3. Minimal activation of PD-L1 by IL-4 and IL-10 compared to IFNγ in HT1376 cells. Expression of FOXP3 and PD-L1 by flow cytometry comparing IFNγ, IL-4 and IL-10 stimulated HT1376 ^{CT} and HT1376 $^{FOXP3\ KO}$ cells. Mean of triplicates and representative of 2 independent experiments, p values indicated. ns not significant, * $p < 0.05$, ** $p < 0.01$, *** $p < 0.001$, **** $p < 0.0001$.
