# Supplementary material for: Role of Forkhead Box P3 in IFNγ-Mediated PD-L1 Expression and Bladder Cancer Epithelial-to-Mesenchymal Transition
Source: Cancer Res Commun. 2024 Aug 26;4(8):2228–41. doi: 10.1158/2767-9764.CRC-23-0493 (PMC11345674; doi:10.1158/2767-9764.CRC-23-0493)
Supplement: Supplementary Figure 4 — FOXP3 antibodies not sufficient for ChIP Sequencing [file crc-23-0493_supplementary_figure_4_suppsf4.pdf]

Supplementary Figure 4

A

Cell lines	Antibody
MT2	anti-human FOXP3 delta 2 (also known as delta 3 with non coded exon 1) mouse IgG2a BioLegend Clone A150080A (Cat# 694802)
	anti-human pan-FOXP3 rabbit polyclonal Abcam (Cat# ab54501)
	anti-human H3K4me3 rabbit polyclonal Invitrogen (Cat# 49-1005)
HT1376 without and with 100 ng IFNg	anti-human FOXP3 delta 2 mouse IgG2a BioLegend Clone A150080A (Cat# 694802)
	anti-human FOXP3 delta 2 mouse IgM Novus Clone 16J4G6 (Cat# NBP2-24953)
	anti-human FOXP3 WT mouse IgG1 BioLegend clone 206D (Cat# 320102)
	anti-human pan-FOXP3 rabbit polyclonal Abcam (Cat# ab54501)
	anti-human pan-FOXP3 rat IgG2a Invitrogen Clone PCH101 (Cat# 14-4776-82)
	anti-human H3K4me3 rabbit polyclonal Invitrogen (Cat# 49-1005)

B

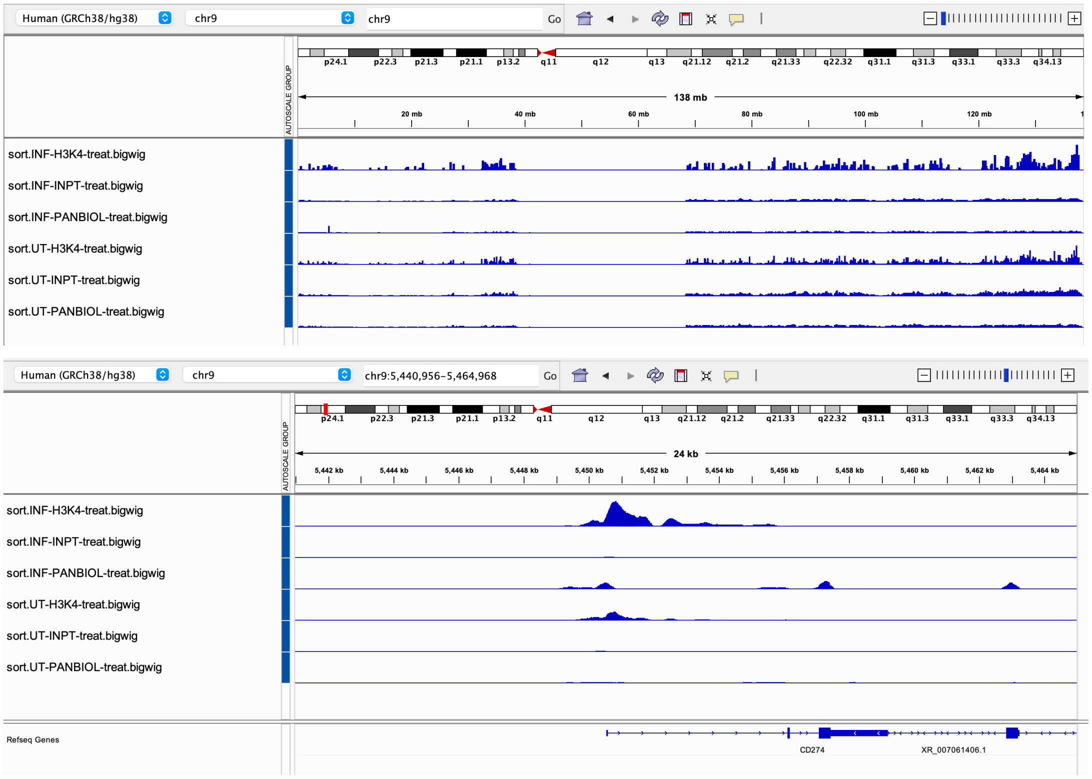

Supplementary Figure 4. FOXP3 antibodies not sufficient for ChIP Sequencing. (A) Summary of antibodies and cell lines used in ChIP-seq experiments. (B) Representative ChIP-seq shown in HT1376 cells untreated (UT) or treated with IFNγ (INF) as indicated. Chromatin precipitated using H3K4me3 control antibody (H3K4), input only (INPT) or pan-FOXP3 (PANBIOL) (Abcam #ab54501). BigWig files depicted at chromosome 9 (chr9) as well as *PD-L1* promotor on chr9 displayed using IGV. IGV tracks of the two H3K4me3 samples display high coverage across chr9, while the FOXP3 tracks show rare coverage. This suggests that this FOXP3 antibody is not sufficient for ChIP-seq. Fastq files were mapped to human genome (hg38) using bowtie2. Samtools v1.15 were used to sort bam files and create index. Sambamba v1.0.1 was used to remove duplicates. Bedtools v2.30.0 was used to filter out blacklisted regions. Macs2 was used to call peaks. bedGraphToBigWig v2.10 was used to create bigwig files. IGV v2.16.2 was used to display reads coverage.
