# Supplementary material for: Role of Forkhead Box P3 in IFNγ-Mediated PD-L1 Expression and Bladder Cancer Epithelial-to-Mesenchymal Transition
Source: Cancer Res Commun. 2024 Aug 26;4(8):2228–41. doi: 10.1158/2767-9764.CRC-23-0493 (PMC11345674; doi:10.1158/2767-9764.CRC-23-0493)
Supplement: Supplementary Figure 5 — Composite Z-scores of FOXP3-dependent IFNgamma-inducible genes in HT1376 cells [file crc-23-0493_supplementary_figure_5_suppsf5.pdf]

Supplementary Figure 5

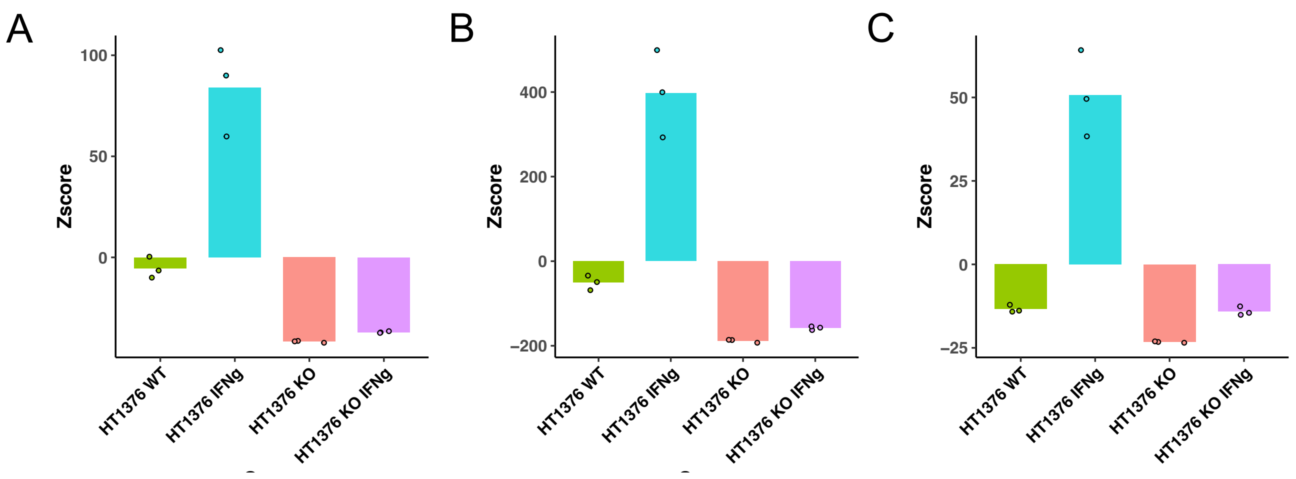

Supplementary Figure 5. Composite Z-scores of FOXP3-dependent IFNγ-inducible genes in HT1376 cells. The Z-scores of the genes of interest are summed for each sample. Bar plots display the average Z-score for all three samples in each group, while the dots represent the Z-scores of individual samples. (A) 271 gene panel, (B) EMT-related genes, and (C) immune-related genes.
