# Supplementary material for: Role of Forkhead Box P3 in IFNγ-Mediated PD-L1 Expression and Bladder Cancer Epithelial-to-Mesenchymal Transition
Source: Cancer Res Commun. 2024 Aug 26;4(8):2228–41. doi: 10.1158/2767-9764.CRC-23-0493 (PMC11345674; doi:10.1158/2767-9764.CRC-23-0493)
Supplement: Supplementary Figure 6 — Generation of MB49 FOXP3 knockout cells by flow cytometry and Western blot. Immunofluorescence of chromogranin A and NSE. [file crc-23-0493_supplementary_figure_6_suppsf6.pdf]

Supplementary Figure 6

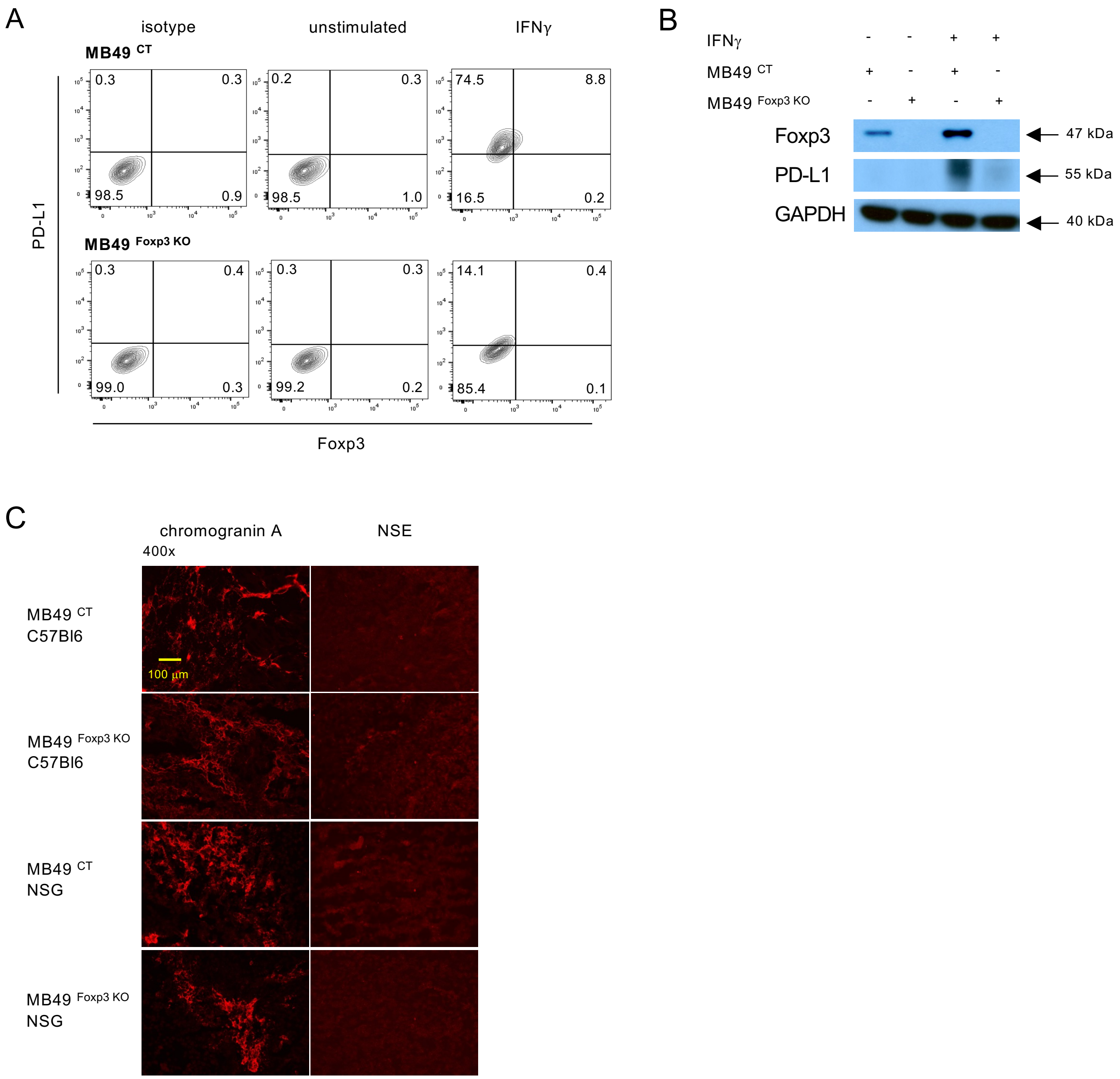

Supplementary Figure 6. (A) Flow cytometry of Foxp3 and PD-L1 in MB49 ^{CT} and MB49 $^{Foxp3\ KO}$ lines without and with IFNγ stimulation. Representative of 3 independent experiments. (B) Expression of FOXP3 and PD-L1 in MB49 ^{CT} and MB49 $^{Foxp3\ KO}$ lines without and with IFNγ stimulation by Western blotting. Foxp3 samples were immunoprecipitated. Size in kD as indicated. Full Western blots shown in Supplementary Fig. 1. (C) Immunofluorescence of chromogranin A and NSE in MB49 ^{CT} and MB49 $^{Foxp3\ KO}$ tumors derived from C57Bl6 and NSG animals. Images representative of 3 independent tumors. Exposure times listed in Supplementary Table 6.
