# Supplementary material for: Role of Forkhead Box P3 in IFNγ-Mediated PD-L1 Expression and Bladder Cancer Epithelial-to-Mesenchymal Transition
Source: Cancer Res Commun. 2024 Aug 26;4(8):2228–41. doi: 10.1158/2767-9764.CRC-23-0493 (PMC11345674; doi:10.1158/2767-9764.CRC-23-0493)
Supplement: Supplementary Table 1 — GSEA FDR and p-values [file crc-23-0493_supplementary_table_1_suppst1.pdf]

Table: Gene sets enriched in phenotype **WT_UTR (3 samples)** **[plain text format]**

	GS<br>follow link to MSigDB	GS DETAILS	SIZE	ES	NES	NOM p-val	FDR q-val	FWER p-val	RANK AT MAX	LEADING EDGE
1	HALLMARK_KRAS_SIGNALING_UP	Details ...	169	0.53	2.12	0.000	0.000	0.000	2733	tags=38%, list=13%, signal=44%
2	HALLMARK_ESTROGEN_RESPONSE_EARLY	Details ...	188	0.47	1.89	0.000	0.001	0.002	3271	tags=34%, list=15%, signal=39%
3	HALLMARK_INFLAMMATORY_RESPONSE	Details ...	164	0.47	1.85	0.000	0.001	0.003	3870	tags=37%, list=18%, signal=45%
4	HALLMARK_TNFA_SIGNALING_VIA_NFKB	Details ...	192	0.45	1.84	0.000	0.001	0.003	3684	tags=40%, list=17%, signal=48%
5	HALLMARK_COMPLEMENT	Details ...	177	0.46	1.81	0.000	0.001	0.006	2839	tags=28%, list=13%, signal=32%
6	HALLMARK_APICAL_SURFACE	Details ...	42	0.55	1.75	0.002	0.002	0.012	2667	tags=40%, list=12%, signal=46%
7	HALLMARK_APICAL_JUNCTION	Details ...	185	0.42	1.71	0.000	0.004	0.023	5026	tags=42%, list=23%, signal=54%
8	HALLMARK_UNFOLDED_PROTEIN_RESPONSE	Details ...	113	0.45	1.70	0.000	0.004	0.028	7272	tags=55%, list=33%, signal=82%
9	HALLMARK_IL2_STAT5_SIGNALING	Details ...	178	0.42	1.70	0.000	0.004	0.030	4710	tags=41%, list=22%, signal=52%
10	HALLMARK_APOPTOSIS	Details ...	148	0.42	1.66	0.000	0.005	0.043	4235	tags=41%, list=19%, signal=51%
11	HALLMARK_EPITHELIAL_MESENCHYMAL_TRANSITION	Details ...	176	0.41	1.64	0.000	0.007	0.060	2384	tags=30%, list=11%, signal=33%
12	HALLMARK_MTORC1_SIGNALING	Details ...	198	0.39	1.60	0.000	0.011	0.107	6893	tags=47%, list=32%, signal=68%
13	HALLMARK_ALLOGRAFT_REJECTION	Details ...	154	0.41	1.59	0.001	0.011	0.113	3746	tags=36%, list=17%, signal=44%
14	HALLMARK_GLYCOLYSIS	Details ...	185	0.39	1.55	0.000	0.016	0.173	3929	tags=28%, list=18%, signal=33%
15	HALLMARK_ESTROGEN_RESPONSE_LATE	Details ...	184	0.38	1.54	0.000	0.017	0.185	3535	tags=31%, list=16%, signal=37%
16	HALLMARK_UV_RESPONSE_DN	Details ...	140	0.40	1.54	0.003	0.017	0.196	2872	tags=29%, list=13%, signal=34%
17	HALLMARK_INTERFERON_ALPHA_RESPONSE	Details ...	96	0.40	1.51	0.006	0.021	0.264	3916	tags=34%, list=18%, signal=42%
18	HALLMARK_COAGULATION	Details ...	104	0.40	1.50	0.007	0.022	0.281	3029	tags=32%, list=14%, signal=37%

19	HALLMARK_UV_RESPONSE_UP	Details ...	151	0.38	1.50	0.003	0.021	0.282	3741	tags=28%, list=17%, signal=33%
20	HALLMARK_P53_PATHWAY	Details ...	197	0.36	1.48	0.001	0.024	0.332	3244	tags=29%, list=15%, signal=34%
21	HALLMARK_MITOTIC_SPINDLE		199	0.37	1.47	0.001	0.024	0.340	4395	tags=26%, list=20%, signal=32%
22	HALLMARK_MYC_TARGETS_V1		200	0.36	1.47	0.000	0.023	0.342	8225	tags=51%, list=38%, signal=81%
23	HALLMARK_REACTIVE_OXYGEN_SPECIES_PATHWAY		48	0.45	1.46	0.029	0.025	0.380	4323	tags=40%, list=20%, signal=49%
24	HALLMARK_MYOGENESIS		160	0.36	1.44	0.008	0.029	0.453	3785	tags=31%, list=17%, signal=37%
25	HALLMARK_IL6_JAK_STAT3_SIGNALING		78	0.40	1.44	0.021	0.029	0.464	4345	tags=40%, list=20%, signal=49%
26	HALLMARK_KRAS_SIGNALING_DN		134	0.37	1.41	0.024	0.037	0.558	1938	tags=19%, list=9%, signal=20%
27	HALLMARK_INTERFERON_GAMMA_RESPONSE		189	0.35	1.38	0.013	0.046	0.646	4946	tags=39%, list=23%, signal=50%
28	HALLMARK_TGF_BETA_SIGNALING		53	0.39	1.31	0.103	0.092	0.890	3535	tags=30%, list=16%, signal=36%
29	HALLMARK_HYPOXIA		180	0.33	1.30	0.035	0.093	0.904	3670	tags=23%, list=17%, signal=28%
30	HALLMARK_MYC_TARGETS_V2		58	0.37	1.28	0.119	0.108	0.939	7218	tags=59%, list=33%, signal=87%
31	HALLMARK_ANDROGEN_RESPONSE		97	0.34	1.25	0.114	0.128	0.974	4938	tags=36%, list=23%, signal=46%
32	HALLMARK_PEROXISOME		93	0.32	1.18	0.199	0.224	0.997	3843	tags=27%, list=18%, signal=32%
33	HALLMARK_PI3K_AKT_MTOR_SIGNALING		95	0.32	1.16	0.186	0.238	0.999	5209	tags=33%, list=24%, signal=43%
34	HALLMARK_XENOBIOTIC_METABOLISM		162	0.28	1.11	0.228	0.315	1.000	4276	tags=30%, list=20%, signal=37%
35	HALLMARK_HEME_METABOLISM		176	0.28	1.11	0.212	0.307	1.000	2857	tags=17%, list=13%, signal=19%
36	HALLMARK_CHOLESTEROL_HOMEOSTASIS		73	0.29	1.02	0.430	0.498	1.000	3839	tags=32%, list=18%, signal=38%
37	HALLMARK_PANCREAS_BETA_CELLS		21	0.36	1.02	0.425	0.493	1.000	5893	tags=38%, list=27%, signal=52%
38	HALLMARK_PROTEIN_SECRETION		94	0.26	0.98	0.520	0.565	1.000	6694	tags=37%, list=31%,

										signal=54%
39	HALLMARK_OXIDATIVE_PHOSPHORYLATION		199	0.23	0.95	0.598	0.634	1.000	7328	tags=37%, list=34%, signal=55%
40	HALLMARK_DNA_REPAIR		149	0.23	0.90	0.663	0.721	1.000	6622	tags=32%, list=30%, signal=46%
41	HALLMARK_ADIPOGENESIS		191	0.21	0.87	0.755	0.786	1.000	5166	tags=28%, list=24%, signal=36%
42	HALLMARK_G2M_CHECKPOINT		199	0.20	0.80	0.903	0.890	1.000	4747	tags=19%, list=22%, signal=24%
43	HALLMARK_E2F_TARGETS		200	0.15	0.62	1.000	0.994	1.000	4726	tags=17%, list=22%, signal=22%
