# Supplementary material for: Role of Forkhead Box P3 in IFNγ-Mediated PD-L1 Expression and Bladder Cancer Epithelial-to-Mesenchymal Transition
Source: Cancer Res Commun. 2024 Aug 26;4(8):2228–41. doi: 10.1158/2767-9764.CRC-23-0493 (PMC11345674; doi:10.1158/2767-9764.CRC-23-0493)
Supplement: Supplementary Table 2 — FDRs and p-values of IFNgamma induced genes [file crc-23-0493_supplementary_table_2_suppst2.pdf]

Fig2.E_IFNg_induced_genes

IFNg vs control	baseMean	log2FoldChange	lfcSE	pvalue	padj
UBD	169.9588662	12.70367742	3.201321602	1.95E-16	1.79E-14
CXCL9	99.79380204	11.79164172	3.166734845	2.56E-13	1.89E-11
GBP5	5349.010632	11.17768064	0.767525977	2.23E-48	6.73E-46
HLA-DOA	289.3719056	11.16591365	1.311539295	2.12E-23	3.03E-21
HLA-DRA	2451.176536	11.11024511	0.453590753	2.4E-130	3.45E-127
GBP1P1	230.3044797	10.82579219	1.311986497	5.8E-22	7.6E-20
HLA-DMB	223.3363013	10.78391376	1.311417547	7.85E-22	1.01E-19
CD74	8581.536499	10.7725795	0.252415828	0	0
OR2I1P	46.36752507	10.58609794	2.945625501	1.97E-13	1.46E-11
HLA-DQA1	431.7602209	10.36628384	0.833369835	5.92E-34	1.22E-31
HLA-DRB1	1222.626252	9.855863691	0.427620695	1.81E-116	2.12E-113
AC005515.1	109.8302162	9.729413816	1.309592318	5.6E-18	5.79E-16
Z84484.1	23.35131704	9.518286244	2.818546842	1.32E-11	8.2E-10
HLA-DRB5	24.00883835	9.479914409	2.841574986	4.94E-11	2.92E-09
CIITA	2124.520732	9.348852076	0.307895866	4.96E-209	1.55E-205
PYHIN1	22.84880043	9.34051195	2.842801504	1.62E-10	9.04E-09
IDO1	695.4618439	9.283091094	0.487197755	2.62E-80	1.58E-77
GBP7	21.49438335	9.246946954	2.837106136	2.77E-10	1.51E-08
SPTA1	184.4323646	9.097860448	0.826811315	2.69E-27	4.58E-25
ACKR4	18.10247479	9.014571022	2.782831868	2.41E-10	1.32E-08
AC007991.2	16.3604576	8.906111965	2.786373936	7E-10	3.68E-08
HLA-DRB6	15.81571126	8.703645603	2.785404289	2.69E-09	1.31E-07
FCRL1	13.91630432	8.531309304	2.755375794	3.75E-09	1.78E-07
ENSG00000285744	117.9622544	8.441588712	0.834011343	2.14E-23	3.04E-21
HLA-DPB1	436.1191514	8.431760967	0.457763965	1.08E-76	5.45E-74
AC015911.3	12.91315236	8.348683307	2.756648196	1.26E-08	5.66E-07
AL157871.4	12.40399372	8.337817326	2.745603001	1.05E-08	4.74E-07
C1S	1107.663103	8.083680197	0.315873419	4.12E-148	8.58E-145
FCGR2A	11.28316285	8.059071463	2.790776967	1.41E-07	5.48E-06

**TOMM20P2**	10.42037083	8.021197497	2.736427524	5.8E-08	2.38E-06
**GBP4**	4376.920408	7.833104064	1.027285589	9.56E-16	8.33E-14
**GBP6**	95.60605227	7.81794142	0.752555081	3.19E-24	4.78E-22
**CXCL10**	513.1367148	7.73206548	0.777682008	2.82E-24	4.29E-22
**SLFN12L**	8.45166973	7.615017852	2.797071396	1.28E-06	4.15E-05
**APOL4**	64.38748026	7.598581701	0.85543602	4.58E-18	4.8E-16
**ENSG00000286065**	9.553942694	7.516222157	2.883784819	4.23E-06	0.000122767
**GBP2**	1195.579019	7.417127718	0.248470094	4.64E-196	1.24E-192
**U62317.2**	7.522699104	7.372579631	2.723175181	1.1E-06	3.61E-05
**SERPING1**	525.6679861	7.281846573	0.295322371	7.56E-133	1.29E-129
**HLA-DPA1**	1029.530794	7.22002732	0.636394766	2.31E-31	4.36E-29
**UGT3A2**	8.012552544	7.155655709	2.901350391	1.49E-05	0.000376616
**GBP1**	5662.521897	7.125396845	0.176082571	0	0
**TRIM40**	20.02973586	7.093612908	1.416285889	4.88E-09	2.29E-07
**LINC00364**	7.337237374	7.091314889	2.808705491	7.85E-06	0.000214278
**BANCR**	6.164402411	7.000525021	2.758826155	7.45E-06	0.000205404
**CX3CL1**	7.349883779	6.969316394	2.902975027	2.45E-05	0.000594446
**AL591468.1**	6.487091756	6.956563729	2.741940404	6.37E-06	0.000177554
**CCDC194**	5.973549724	6.923061838	2.756621043	9.27E-06	0.000249384
**TRIM22**	1004.879827	6.904696903	0.676058226	3.55E-26	5.94E-24
**RPL32P1**	5.786576651	6.859457397	2.759094018	1.17E-05	0.000303012
**ZBP1**	72.21550529	6.829529069	0.702887447	4.48E-22	5.99E-20
**AC025252.2**	5.664514346	6.785634265	2.789912552	1.95E-05	0.000483866
**AC083837.1**	280.9362532	6.723076598	0.359967534	1.36E-76	6.7E-74
**RUFY4**	6.052114458	6.720373962	2.828461644	2.84E-05	0.000679879
**ITK**	27.12687977	6.710550763	1.062824696	6.06E-11	3.55E-09
**IRF8**	5.543459602	6.662977383	2.778512354	2.36E-05	0.00057397
**ZBTB20-AS1**	6.085577403	6.617726203	2.840822903	3.69E-05	0.000856509
**BATF2**	756.0584133	6.181875079	0.255892076	1.78E-131	2.78E-128
**CTSS**	1847.468091	6.168808306	0.487862238	9.3E-38	2.23E-35
**IFI44L**	422.6409284	6.113682841	0.900089006	3.92E-13	2.82E-11
**HLA-DMA**	332.2668487	6.058220134	0.279445248	6.09E-104	6.34E-101

**WARS**	33622.59242	6.035389759	0.152968622	0	0
**USP30-AS1**	49.13529395	5.989799052	0.637775093	1.94E-21	2.38E-19
**COL4A1**	57.78332936	5.955583111	0.61953993	4.8E-22	6.38E-20
**AIM2**	2095.890257	5.938735001	0.180696426	5.71E-237	2.67E-233
**CXCL11**	160.2880886	5.91581532	0.484820898	7.2E-36	1.61E-33
**ICAM1**	4367.212312	5.888679745	0.483761062	2.24E-35	4.83E-33
**IL7R**	10.92153594	5.799442984	1.496187383	1.43E-06	4.61E-05
**IL7**	14.19275506	5.722641424	1.140964426	1.01E-07	4.03E-06
**KLHDC7B**	516.9971705	5.722565505	0.247536042	2.85E-120	3.55E-117
**LRCH2**	23.23896272	5.669666945	0.967841643	2.25E-09	1.11E-07
**AC022034.2**	8.658981215	5.628773029	1.528061468	6.3E-06	0.000175887
**XAF1**	1843.395826	5.587165529	0.586468533	6.72E-23	9.13E-21
**C4B**	36.86293984	5.586300845	0.73563779	1.04E-15	8.99E-14
**NUGGC**	19.94744299	5.40708919	0.936625548	3.47E-09	1.66E-07
**EXOC3L1**	17.89841481	5.401141664	0.9860801	4.22E-09	1.99E-07
**NEXN**	131.5183357	5.38734622	0.94704272	3.53E-10	1.9E-08
**C1R**	240.9199229	5.345661034	0.28037041	8.74E-83	5.65E-80
**SP140**	85.36447374	5.316212712	0.45923379	6.64E-32	1.29E-29
**AC068299.1**	7.432017399	5.281916995	1.596525399	2.84E-05	0.000679879
**AL357054.4**	15.2673049	5.238326989	0.993828894	2.51E-08	1.08E-06
**ANO4**	13.49337763	5.208925123	1.044039777	2.45E-07	9.02E-06
**ETV7**	423.3886184	5.184073325	0.221190745	1.31E-123	1.76E-120
**OAS2**	1903.421196	5.132824778	0.766094633	8.91E-13	6.21E-11
**IL32**	3622.445901	5.122553179	0.26934409	1.45E-81	9.03E-79
**DGKG**	9.435231421	5.116337987	1.262994762	7.75E-06	0.000212385
**CD5L**	63.55447531	5.039574777	0.460067824	7.45E-29	1.35E-26
**CMPK2**	350.7475253	4.908840411	0.691086757	4.57E-14	3.54E-12
**HLA-DQB1**	1171.012049	4.887354532	0.268085651	6.15E-75	2.96E-72
**C4A**	34.20739061	4.866381509	0.702013681	3.4E-13	2.46E-11
**LINC02033**	10.93310756	4.753890102	1.525745499	7.54E-05	0.001586136
**APOBEC3G**	258.87272	4.7050513	0.242090854	3.06E-85	2.2E-82
**ICAM4**	10.44122256	4.672693655	1.343627695	1.95E-05	0.000483866

**GRIP2**	105.3063102	4.661010965	1.170570659	2.1E-06	6.57E-05
**CCL3**	10.83417929	4.654233636	1.284760904	3.47E-05	0.00080997
**FRMD3**	147.2288614	4.620321924	0.393720958	1.08E-33	2.17E-31
**TEX29**	24.13833071	4.578265937	0.726630005	4.82E-11	2.86E-09
**45539**	53.04283245	4.566071368	0.513741503	7.93E-21	9.46E-19
**HCP5**	3203.030715	4.539060367	0.677496945	9.3E-13	6.46E-11
**TMEM140**	173.3598939	4.503940067	0.318207628	1.74E-47	5.16E-45
**RSAD2**	721.9729934	4.461708901	0.572874751	2.45E-16	2.22E-14
**GPM6A**	7.35792465	4.460416199	1.321075416	6.11E-05	0.001331393
**MX2**	246.0100609	4.439331166	0.699612839	8.27E-12	5.24E-10
**L3MBTL4**	23.61424598	4.383309199	0.664967478	6.28E-12	4.05E-10
**TAP1**	6295.847401	4.373794831	0.171422024	1.06E-144	1.99E-141
**MMP25**	34.55250336	4.281016717	0.708567728	2.8E-11	1.7E-09
**BTN3A3**	738.356905	4.273169077	0.524409	1.53E-17	1.54E-15
**NLRC5**	1219.924248	4.260000617	0.270123312	1.25E-57	4.57E-55
**NEURL3**	30.45126865	4.247009992	0.742056576	9.05E-10	4.7E-08
**AC007012.2**	6.386220508	4.244031262	1.339261041	0.00012309	0.002419662
**RTP4**	209.3858753	4.197943452	0.377641448	2.09E-30	3.92E-28
**CFB**	13.18112716	4.126893664	0.96012745	9.55E-07	3.17E-05
**IL12RB1**	95.02223202	4.112320888	0.397903351	1.49E-25	2.37E-23
**TMEM229B**	64.69823681	4.073274767	0.424806874	2.84E-23	3.97E-21
**CTSO**	94.84025525	4.026451266	0.462577565	3.1E-20	3.54E-18
**AC083862.2**	39.3461502	3.977848515	0.512166678	2.02E-16	1.84E-14
**AC007728.2**	22.51842835	3.953685731	0.825057104	3.56E-08	1.5E-06
**NLRP5**	20.5629764	3.952493206	0.694414828	1.79E-09	8.97E-08
**AP005060.1**	7.84457479	3.940648691	1.207507306	0.00012552	0.002462335
**APOBEC3F**	313.8428144	3.935623841	0.251991512	6.85E-57	2.47E-54
**AP003716.1**	5.645671688	3.89484622	1.427389407	0.00035438	0.006043708
**RF02116**	8.830046292	3.871828019	1.543022717	0.00052201	0.008481315
**HLA-B**	37300.81125	3.869334263	0.445965685	2.66E-19	2.9E-17
**ACSL5**	1166.189716	3.860013247	0.195750238	3.46E-88	2.59E-85
**SAA1**	81.8965883	3.856398726	0.490507838	2.5E-16	2.26E-14

**IRF1**	6432.027658	3.835619012	0.120376323	7.23E-224	2.71E-220
**HLA-H**	898.7867463	3.829346659	0.281609563	1.54E-43	4.38E-41
**MMP25-AS1**	484.6510837	3.814651331	0.17747755	1.65E-103	1.63E-100
**IFIT3**	4051.996526	3.809653616	0.183929641	1.15E-96	1.03E-93
**SECTM1**	741.6236409	3.790690354	0.169107424	6.91E-113	7.62E-110
**HLA-A**	23968.51654	3.780482548	0.234775339	1.69E-59	6.47E-57
**TMPRSS3**	184.1836611	3.763369699	0.856175524	4.32E-07	1.52E-05
**HLA-F**	4597.147598	3.761277968	0.245399969	2.65E-54	9.01E-52
**APOBEC3A**	81.09324972	3.753150588	0.503832769	6.44E-15	5.27E-13
**TYMP**	126.2335307	3.743849057	0.366342507	6.32E-26	1.03E-23
**TRIM17**	29.64335051	3.715463079	0.54299811	1.15E-12	7.92E-11
**RARRES3**	734.9462372	3.712247021	0.501515127	7.3E-15	5.95E-13
**LINC00623**	10.46258126	3.708942983	1.031718819	1.71E-05	0.000429746
**AC008760.2**	8.681813728	3.68865916	1.536349771	0.00079581	0.012120351
**SLFN5**	866.8035307	3.651593143	0.214604669	3.7E-66	1.58E-63
**CASP1**	1209.767948	3.622346367	0.193031138	3.71E-80	2.17E-77
**STAT1**	16141.31493	3.611246846	0.131591059	7.91E-167	1.85E-163
**RASGRP3**	17.96643884	3.601806529	0.792042189	3.79E-07	1.36E-05
**IL3RA**	11.11330502	3.591671784	0.959645224	1.41E-05	0.000359585
**AL671883.2**	30.26736764	3.575967062	0.519497714	6.98E-13	4.9E-11
**PSMB9**	1467.316697	3.569348217	0.218846179	4.13E-61	1.65E-58
**IL12RB2**	9.815552133	3.555081362	1.161960861	0.00018223	0.00341047
**LAMP3**	406.0161626	3.544721325	0.288797066	4.23E-36	9.55E-34
**PLSCR4**	24.34473798	3.541577672	0.650713158	2.34E-09	1.15E-07
**CACNA1F**	12.56840612	3.53228345	0.896571406	3.51E-06	0.000105198
**SLC15A3**	356.7756309	3.486663179	0.319553925	5.18E-29	9.51E-27
**HLA-DOB**	33.24996745	3.484498133	0.535432125	2.52E-12	1.67E-10
**AC067945.1**	72.00557478	3.459345033	0.398041107	1.03E-19	1.15E-17
**CDH15**	5.715418848	3.457858478	1.450369271	0.0010880	0.015450569
**LINC01225**	10.63442365	3.424423512	0.949552	1.23E-05	0.000318219
**CCRL2**	19.77269194	3.41710346	0.707767668	8.71E-08	3.5E-06
**AC105749.1**	11.91238399	3.370482097	1.100399196	6.49E-05	0.001396488

**OAS1**	973.7176303	3.319656095	0.490071502	5.83E-13	4.15E-11
**IFI27**	638.2470245	3.3163274	1.093251595	7.49E-05	0.001578584
**AC026333.3**	41.3297893	3.303777621	0.443342854	6.01E-15	4.98E-13
**SAMD9L**	6793.291932	3.299140938	0.18752575	1.4E-70	6.4E-68
**HAPLN3**	85.22215398	3.298311736	0.318009161	5.53E-26	9.18E-24
**APOL3**	1476.649633	3.291559887	0.166017303	7.07E-89	5.52E-86
**TRIM31**	8.482749384	3.289442581	1.570806578	0.00114317	0.015956676
**FGD2**	9.961320937	3.288518122	1.037168362	5.99E-05	0.001313936
**TRIM69**	1400.519919	3.271449525	0.160828731	2.02E-93	1.65E-90
**SAA2**	56.35718759	3.244281333	0.466105382	3.28E-13	2.38E-11
**TVP23A**	41.9790946	3.175293365	0.632505305	1.55E-08	6.85E-07
**UBA7**	204.5505831	3.154958979	0.278092702	1.81E-31	3.45E-29
**MDGA1**	96.00257753	3.144770724	0.339975886	1.86E-21	2.29E-19
**MDS2**	12.52492155	3.126551731	0.772833055	3.52E-06	0.000105383
**ACY3**	17.41526227	3.125214853	0.695380292	3.6E-07	1.29E-05
**CPA2**	9.184852878	3.091147323	1.098849481	0.00018142	0.00340207
**GLRX**	93.21757142	3.087050227	0.351986843	1.39E-19	1.54E-17
**OASL**	281.0074189	3.082010094	0.313032327	2.65E-24	4.07E-22
**HLA-C**	12701.12139	3.071535749	0.196949816	4.02E-56	1.39E-53
**ODF3B**	17.31017499	3.056252968	0.651403008	1.6E-07	6.16E-06
**CSF1**	286.3455754	3.04660822	0.332799615	3.09E-21	3.75E-19
**PARP8**	136.2949456	3.018924144	0.413053587	7.76E-15	6.3E-13
**TRANK1**	670.9342212	3.015108217	0.298628713	2.56E-25	3.99E-23
**AC093844.1**	79.88644797	3.01494121	0.389385133	7.18E-16	6.34E-14
**LINC01436**	13.76099204	3.013333331	0.898943853	5.77E-05	0.001275694
**SP110**	1338.068326	3.009720649	0.163113683	1.75E-77	9.08E-75
**BTN3A2**	1278.879245	3.004395651	0.180321711	1.02E-63	4.24E-61
**APOL1**	2251.614905	2.995014869	0.167124107	4.2E-73	1.97E-70
**LAP3**	2167.668079	2.993067315	0.139793131	5.23E-103	4.9E-100
**IFI35**	858.0625894	2.981103604	0.173305842	8.61E-68	3.84E-65
**IL15RA**	145.5061176	2.975077398	0.259139577	6.67E-32	1.29E-29
**PSMB8-AS1**	200.2980897	2.940926552	0.322288537	1.84E-21	2.29E-19

**EIF4E3**	55.58633021	2.9396842	0.368137456	7.84E-17	7.53E-15
**NRIR**	9.647511776	2.929694087	1.305131185	0.00055501	0.008901678
**PARP14**	15346.08966	2.927037519	0.1709959	6.21E-67	2.71E-64
**AL050403.2**	9.08278391	2.925379007	1.02367667	0.00025965	0.004610546
**MX1**	2517.499575	2.918306838	0.572521919	1.68E-08	7.4E-07
**CCL5**	38.72423907	2.884674843	0.593668296	4.96E-08	2.06E-06
**AL590094.1**	13.5498027	2.879415313	0.795245181	1.22E-05	0.000314795
**UBE2L6**	1943.313971	2.875258984	0.15329415	8.4E-80	4.77E-77
**ACTA2**	34.09486468	2.869820429	0.500422203	3.65E-10	1.96E-08
**B2M**	33026.99071	2.860827565	0.172604336	4.39E-63	1.79E-60
**HLA-E**	17273.98422	2.856083925	0.148317218	7.61E-84	5.09E-81
**PSMB10**	177.1909874	2.840333991	0.262396795	2.74E-28	4.85E-26
**Z95114.3**	39.67560287	2.824483997	0.449555403	1.49E-11	9.21E-10
**RNF213**	16753.78317	2.812253165	0.151331804	2.69E-78	1.44E-75
**HCG4P11**	8.745529992	2.804661429	0.955145958	0.00015911	0.003029203
**SLC7A11-AS1**	25.63267756	2.801633511	0.604040357	2.66E-07	9.74E-06
**PIGR**	117.1722468	2.793026365	0.2985886	6.3E-22	8.19E-20
**CYP4F11**	10.74997028	2.786551813	0.877896185	7.97E-05	0.001669447
**AC124319.1**	63.75179394	2.773831904	0.333400186	5.02E-18	5.22E-16
**LINC01226**	16.19439028	2.763598016	0.723407549	9.45E-06	0.000252961
**PDCD1LG2**	661.6046955	2.744015783	0.210775181	1.01E-39	2.55E-37
**LINC01675**	20.14714536	2.72805496	0.763315441	1.35E-05	0.000346029
**HCAR3**	15.20152942	2.726516564	0.68933487	4.14E-06	0.000120296
**BTN3A1**	1270.111293	2.717224633	0.133215721	8.4E-94	7.16E-91
**HLA-J**	20.14675498	2.696678306	0.756898875	1.19E-05	0.00030787
**AC124319.4**	45.56695292	2.689213409	0.4471849	6.02E-11	3.54E-09
**MARCKSL1**	225.2238839	2.686201012	0.900877747	9.84E-05	0.002002901
**AP001978.1**	6.460449197	2.676979458	1.30485868	0.00138969	0.018568526
**ISG15**	519.6197854	2.673164011	0.289528986	1.13E-21	1.44E-19
**PXYLP1**	154.568703	2.672599082	0.275566717	1.1E-23	1.6E-21
**RF00019**	6.818918976	2.6721957	1.216731492	0.00113761	0.015915611
**APOL6**	10883.40326	2.671544008	0.164889729	4.97E-60	1.94E-57

**DDX58**	367.3920837	2.668280396	0.231902051	5.94E-32	1.17E-29
**GVINP1**	76.48835562	2.663305096	0.358604279	5.56E-15	4.63E-13
**EPSTI1**	1498.402773	2.662387721	0.196306876	2.99E-43	8.36E-41
**HCG4P5**	41.16496586	2.659938141	0.65454999	1.66E-06	5.28E-05
**FAM46C**	28.44556494	2.659830092	0.750864327	1.39E-05	0.000354489
**AC241585.2**	76.91551382	2.656321593	0.520439727	1.46E-08	6.5E-07
**PML**	941.6259846	2.641547165	0.203308653	7.46E-40	1.94E-37
**LINC02100**	70.61168786	2.637372428	0.335020947	1.43E-16	1.32E-14
**VASH2**	104.8242755	2.636890597	0.34736669	1.42E-15	1.22E-13
**GBP3**	520.5928244	2.632603104	0.173212915	1.89E-53	6.22E-51
**AC011816.1**	9.69007797	2.631527462	0.936223622	0.00022932	0.004162798
**TMCC3**	318.9204954	2.628027427	0.32724424	4.81E-17	4.64E-15
**ENSG00000285761**	121.9705657	2.623432557	0.343522025	7.04E-16	6.25E-14
**AP006295.1**	31.38440146	2.62183244	0.521850208	2.33E-08	1.01E-06
**BEND7**	19.58437148	2.615472961	0.69332363	6.09E-06	0.000170753
**STARD8**	24.61293635	2.595996925	0.740718476	2.2E-05	0.000540552
**PSMB8**	2047.520855	2.590729192	0.138524625	2.69E-79	1.48E-76
**SLC28A3**	25.28402831	2.584671602	0.690271132	7.77E-06	0.00021241
**RASGRF1**	269.692864	2.584061446	0.30037111	4.03E-19	4.34E-17
**IFITM4P**	86.14488862	2.580492478	0.483932	3.68E-09	1.74E-07
**CARD17**	113.7792588	2.579452291	0.392537222	2.32E-12	1.55E-10
**PARP3**	198.1940911	2.557565749	0.250556086	5.76E-26	9.46E-24
**IFIH1**	2026.292049	2.547626736	0.181543728	5.18E-46	1.52E-43
**TLR3**	554.4640045	2.542386933	0.263652139	2.25E-23	3.17E-21
**AC010486.1**	35.58937252	2.540831669	0.481311973	4.89E-09	2.29E-07
**ENSG00000287839**	290.7215469	2.536145116	0.21022817	7.71E-35	1.64E-32
**PARP9**	3793.787228	2.53607777	0.131100177	1.25E-84	8.7E-82
**SEC16B**	22.37965106	2.530484111	0.638359583	3.88E-06	0.000114337
**LMO2**	60.09967035	2.530250544	0.420355702	1.17E-10	6.7E-09
**IFIT1**	755.2730943	2.520527722	0.277190351	4.56E-21	5.52E-19
**SSPN**	32.90835468	2.519619975	0.575603401	8.17E-07	2.73E-05
**CRYZL2P-SEC16B**	30.41154006	2.515699207	0.460407783	2.56E-09	1.25E-07

**AL021707.1**	10.8742038	2.493881504	0.931195687	0.00032727	0.005635016
**BST2**	862.4453711	2.48242558	0.158950059	3.3E-56	1.17E-53
**MMP1**	167.8559202	2.481004811	0.307163592	4.33E-17	4.2E-15
**IL18R1**	32.58050173	2.47907998	0.496030044	4.1E-08	1.71E-06
**RF00019**	17.37896307	2.478354641	0.670578776	1.44E-05	0.000365348
**IFI44**	808.1998452	2.476495612	0.641992701	5.18E-06	0.000147339
**AC009133.1**	86.09157295	2.475219761	0.389460077	7.35E-12	4.69E-10
**TNFRSF14**	211.4782956	2.464443674	0.344911191	3.85E-14	3.01E-12
**PTGES3P1**	37.75764899	2.456213328	0.440470852	1.07E-09	5.49E-08
**TLR4**	146.6179896	2.45518019	0.384857905	6.86E-12	4.39E-10
**HVCN1**	7.031517285	2.438019847	1.127077799	0.00114396	0.015956676
**GIMAP2**	204.840631	2.437451464	0.280636706	1.69E-19	1.85E-17
**LINC01117**	6.53254819	2.426450414	1.188089452	0.00139688	0.018638113
**LINC02577**	198.3502623	2.420085236	0.35516529	4.27E-13	3.06E-11
**STAT2**	1710.572708	2.415199342	0.150685932	4.44E-59	1.66E-56
**CA2**	23.57508167	2.414064798	0.577573012	1.43E-06	4.61E-05
**IFITM1**	511.0556397	2.409805903	0.251772397	4.49E-23	6.18E-21
**ENSG00000287077**	6.351083511	2.395430468	1.97364157	0.00333403	0.037715242
**ENSG00000287188**	33.42833248	2.393330756	0.526267413	2.06E-07	7.74E-06
**HNF4G**	42.18636518	2.385125215	0.518181591	3.02E-07	1.1E-05
**DDX60**	1895.541529	2.368853848	0.221176578	4.99E-28	8.65E-26
**SFMBT2**	7.646182429	2.360857648	1.548455564	0.00391278	0.042764395
**VWA3B**	22.67511416	2.356252	0.595565059	3.66E-06	0.00010909
**P2RY6**	107.622856	2.343132571	0.413956927	9.21E-10	4.77E-08
**AC067930.4**	17.28638861	2.333121548	0.619024876	8.82E-06	0.000238508
**PATL2**	78.36847595	2.319472717	0.314253201	6.3E-15	5.17E-13
**AC005840.4**	10.96998185	2.318964637	1.343664179	0.00232656	0.028319324
**HRASLS2**	27.32694025	2.318080838	0.478399871	8.54E-08	3.45E-06
**GSDMB**	383.3198878	2.317511153	0.245799808	1.91E-22	2.57E-20
**CASP1P2**	74.9157295	2.314478195	0.316552966	1.25E-14	9.93E-13
**FAS**	317.5240668	2.312051844	0.1893035	1.01E-35	2.2E-33
**LINC00944**	444.8861156	2.305220047	0.175571239	1.21E-40	3.19E-38

**SAMHD1**	1689.517135	2.30085643	0.173904528	3.03E-41	8.12E-39
**CD40**	588.6115613	2.291617945	0.183466827	7.84E-37	1.86E-34
**MTND1P11**	19.76793493	2.28818565	0.627532432	1.47E-05	0.000372608
**TNFSF10**	571.2985986	2.282335891	1.286260372	0.00171344	0.022106072
**MUC4**	362.1217831	2.276792167	0.317195847	4.73E-14	3.65E-12
**ERAP2**	1105.974145	2.275218321	0.149154433	7.63E-54	2.55E-51
**APOL2**	2319.396768	2.262989733	0.16994166	1.07E-41	2.9E-39
**LINC01752**	38.52543312	2.25790574	0.437399049	1.54E-08	6.83E-07
**GSAP**	245.4280194	2.247239926	0.187669517	2.48E-34	5.17E-32
**TPK1**	68.15485592	2.246967588	0.349182217	4.96E-12	3.21E-10
**OAS3**	3997.91774	2.24180809	0.207761851	2.32E-28	4.15E-26
**AC018868.2**	7.528340246	2.237877883	1.423777264	0.00251308	0.030177905
**LINC02328**	46.15884594	2.235207219	0.368096194	9.39E-11	5.43E-09
**ASPHD2**	53.74342297	2.214755574	0.479050338	2.46E-07	9.02E-06
**LINC02450**	11.53400137	2.20238845	0.999597713	0.00102506	0.014714515
**IFI16**	3362.546823	2.197002365	0.177737535	2.5E-36	5.77E-34
**VNN 2.00**	16.61906721	2.190259596	0.960724769	0.00069077	0.01075671
**IL15**	168.3739049	2.188914646	0.277417653	1.25E-16	1.17E-14
**STX11**	19.48453748	2.178012054	0.674994466	5.83E-05	0.001286963
**AC087683.1**	7.397007651	2.171102441	1.190940949	0.00204611	0.025502297
**ENSG00000286288**	34.18913377	2.167790528	0.603347143	1.15E-05	0.000301219
**ABCA6**	14.29740758	2.163846383	0.985854403	0.00081393	0.012326199
**KDR**	6.473599665	2.148631237	1.316834957	0.00298823	0.03449079
**CASC17**	19.46256625	2.143905988	0.726553428	0.00012742	0.002491603
**AC091614.1**	18.30113845	2.134490661	0.743777048	0.00019998	0.003690978
**TNFSF14**	6.224431783	2.116983877	1.783801953	0.00438659	0.046716383
**HLA-L**	14.95710828	2.108717833	1.011490295	0.00115769	0.016124252
**APOBEC3C**	178.063622	2.106322976	0.212507682	3.05E-24	4.6E-22
**PRKX**	8.683202486	2.102673529	1.067309756	0.00189432	0.02397726
**UBQLNL**	10.12087757	2.083364037	1.732602849	0.00355724	0.039736383
**TRIM21**	624.0875625	2.079839927	0.164400042	5.04E-38	1.23E-35
**GSDMC**	101.0822423	2.064965484	0.261026079	1.96E-16	1.79E-14

**RPLP0P2**	76.76616395	2.06132622	0.35606165	4.84E-10	2.58E-08
**MUC20**	16.65822872	2.058605391	0.618327352	4.55E-05	0.001033868
**TAPBPL**	119.9688789	2.055010091	0.244684828	2.86E-18	3.01E-16
**AC018644.1**	40.90713251	2.053051191	0.423910619	8.36E-08	3.38E-06
**AL160272.1**	9.689012728	2.049006223	1.049321678	0.0016603	0.021554622
**ZEB1**	30.80401901	2.048243027	0.567299197	1.62E-05	0.000408878
**CARD16**	316.7547861	2.046300676	0.18633685	2.28E-29	4.23E-27
**AL135838.1**	36.16185536	2.041797984	0.531406079	7.9E-06	0.000215214
**SELPLG**	25.7207	2.031521296	0.638791754	5.95E-05	0.001306189
**MICB**	761.8724015	2.028902924	0.279462864	2.47E-14	1.93E-12
**OR10A3**	14.15041776	2.026488452	0.973067776	0.001536	0.020192196
**CPQ**	10.26762908	2.01867119	1.080189413	0.0023693	0.028797872
**CHD5**	16.85109223	2.018301565	0.902753349	0.0007600	0.01163215
**ENSG00000286635**	14.82303488	2.009842352	0.672462962	0.0001335	0.002597666
**RBFOX1**	8.236767241	1.994139231	0.947500628	0.0014158	0.018837294
**ISG20**	317.9698813	1.989808915	0.21495234	1.36E-21	1.71E-19
**INPP1**	526.7987341	1.988394977	0.155244337	8.27E-39	2.04E-36
**LGALS3BP**	2893.946856	1.986328071	0.141529896	6.3E-46	1.81E-43
**USP18**	333.457759	1.98457097	0.249631332	1.02E-16	9.74E-15
**LGALS9**	76.05138651	1.981529358	0.326187592	7.64E-11	4.45E-09
**C5orf56**	652.8126316	1.978943537	0.163920632	8.32E-35	1.75E-32
**C6orf141**	74.08267703	1.973408646	0.379849378	1.27E-08	5.66E-07
**PSME1**	2048.919381	1.967309384	0.135932252	1.04E-48	3.26E-46
**AC009549.1**	12.53074705	1.966412573	0.940456759	0.0013640	0.018277682
**ENTPD3**	294.1934525	1.960164572	0.216861872	1.19E-20	1.38E-18
**RN7SL834P**	14.34730512	1.958681199	0.691853524	0.0002609	0.004629369
**SOCS1**	164.1698949	1.958669524	0.281669934	2.75E-13	2.02E-11
**CADPS2**	221.9284012	1.955520562	0.389776868	2.72E-08	1.16E-06
**PSME2**	1747.932065	1.954080064	0.13415979	3.01E-49	9.55E-47
**SERPINB9**	61.35093202	1.950910329	0.425297569	2.11E-07	7.91E-06
**ENKUR**	94.65487021	1.949134804	0.559064849	2.14E-05	0.000526617
**IFI30**	23.01328308	1.947346172	0.539194675	1.5E-05	0.00037868

**WDR25**	180.8366436	1.946342853	0.230552713	1.6E-18	1.72E-16
**AC083902.2**	106.6447414	1.942136769	0.303430632	1.18E-11	7.4E-10
**NUB1**	2322.699302	1.928904116	0.133944244	2.15E-48	6.6E-46
**NID1**	49.73569728	1.924552321	0.416752835	2.29E-07	8.48E-06
**FYB1**	847.7992882	1.917398236	0.234729719	1.83E-17	1.83E-15
**IFITM3**	1514.13237	1.915088506	0.148171742	2.11E-39	5.28E-37
**ENSG00000287263**	260.3051328	1.911024272	0.238982797	1.17E-16	1.1E-14
**RILP**	23.81697968	1.911020134	0.56657137	4.54E-05	0.001032507
**MLKL**	484.9921186	1.910951322	0.177692263	3.24E-28	5.67E-26
**CCL22**	13.07653616	1.90943467	0.883245508	0.00128480	0.017504239
**ZNF114**	88.13442877	1.908547373	0.307912481	2.75E-11	1.67E-09
**TAP2**	1246.448719	1.90393411	0.126979468	6.45E-52	2.08E-49
**AKAP7**	34.36487944	1.893562259	0.510599464	1.03E-05	0.000272486
**SOD2**	1952.766764	1.88985221	0.223512544	1.79E-18	1.9E-16
**AL445437.1**	83.08659668	1.877692888	0.284917376	2.85E-12	1.86E-10
**IRF9**	61.86963866	1.874942096	0.359069092	9.92E-09	4.49E-07
**AF117829.1**	1121.663933	1.869924029	0.271006406	3.15E-13	2.3E-11
**AC115522.1**	71.01869437	1.869895556	0.30075746	3.46E-11	2.08E-09
**OR10A6**	15.58709941	1.865008135	0.869727689	0.00136352	0.018277682
**MTND5P14**	12.17947504	1.856513821	0.754277857	0.00062520	0.009817253
**SP100**	1987.221575	1.855752052	0.189748194	8.89E-24	1.31E-21
**IL18BP**	218.4103513	1.854095312	0.219370857	2.23E-18	2.36E-16
**C1orf228**	11.20010656	1.842456522	1.184844082	0.00272174	0.032087072
**MUC1**	2937.447315	1.835385579	0.27202837	9.93E-13	6.87E-11
**NCF2**	492.4296715	1.83251862	0.204970967	2.71E-20	3.11E-18
**ACSS1**	148.8490422	1.820936189	0.236539927	9.38E-16	8.21E-14
**DTX3L**	7478.706671	1.810168515	0.135598415	8.1E-42	2.23E-39
**DHX58**	133.3425398	1.796265222	0.253818312	8.14E-14	6.2E-12
**MVP**	2498.815703	1.796234562	0.176573257	1.87E-25	2.95E-23
**AL139807.1**	12.33722947	1.795192993	0.81246216	0.00117534	0.016321494
**HERC6**	645.8105999	1.788811081	0.249307175	4.31E-14	3.35E-12
**ENSG00000287483**	26.35387423	1.786394882	0.546839203	6.38E-05	0.001376148

**LINC00920**	25.90506927	1.785010568	0.733629561	0.00053588	0.008654073
**RPL17P10**	12.08077845	1.774020577	0.828165589	0.0013607	0.018272984
**THEMIS2**	60.55961755	1.769513648	0.375033679	1.21E-07	4.79E-06
**NMI**	835.8538541	1.762394247	0.172789747	1.27E-25	2.04E-23
**LINC01215**	40.98111389	1.760227697	0.469679036	9.12E-06	0.0002461
**FAP**	25.12144671	1.758378301	0.758665876	0.0008495	0.012711839
**RAB19**	87.63791662	1.746245105	0.32637679	5.25E-09	2.44E-07
**FBXO6**	45.13344522	1.730335537	0.379901944	3.57E-07	1.28E-05
**CLMP**	121.5432034	1.729224916	0.433914803	3.95E-06	0.000115763
**AL021707.6**	44.85378722	1.721595966	0.422242371	2.17E-06	6.75E-05
**NOV**	17.79922543	1.719481714	1.032412167	0.0031378	0.035885661
**CAPN3**	23.88567205	1.712163491	0.530322213	6.04E-05	0.001324286
**OPTN**	1847.540925	1.704383299	0.213342641	9.6E-17	9.17E-15
**TRAF5**	69.46063562	1.696574512	0.343534796	5.77E-08	2.37E-06
**BISPR**	99.57767732	1.69305925	0.255299	2.6E-12	1.72E-10
**SCNN1G**	83.90389314	1.690195078	0.592565861	0.0002124	0.003879458
**TWF1P1**	10.44409438	1.689445671	0.912581346	0.0022975	0.028020388
**C1RL-AS1**	242.2840726	1.688957941	0.192027637	8.13E-20	9.12E-18
**ALPK1**	726.1094382	1.686558342	0.182313295	1.42E-21	1.78E-19
**ENSG00000287887**	11.96123476	1.682784699	0.874179471	0.0018475	0.023480573
**AC016831.1**	600.7116423	1.680080806	0.276019272	8.67E-11	5.03E-09
**CA13**	48.66766604	1.679945648	0.427121399	4.11E-06	0.000119791
**MRPL45P1**	27.43518184	1.670436916	0.560460522	0.0001757	0.003311666
**AC040162.1**	44.58303386	1.67020847	0.432711759	6.76E-06	0.000188062
**AC016831.7**	410.9349845	1.666592214	0.172756783	4.85E-23	6.63E-21
**ERAP1**	5508.968278	1.666182804	0.144153816	5.17E-32	1.03E-29
**ANO3**	51.8848022	1.665253747	0.384945984	8.97E-07	2.99E-05
**SOCS3**	131.4568016	1.664748006	0.252281491	3.37E-12	2.19E-10
**AL078599.2**	16.73115838	1.66109897	0.995856706	0.0027772	0.032618408
**GDPD2**	55.63089381	1.659362938	0.347003672	1.28E-07	5.05E-06
**TNFRSF1B**	236.9832911	1.659300489	0.183111012	9.63E-21	1.13E-18
**PARP12**	1298.745201	1.658034999	0.168827156	6.1E-24	9.07E-22

**MDK**	17.36866364	1.654814784	0.786766113	0.00117200	0.016287223
**BTN2A2**	433.6867691	1.648359915	0.250152539	2.66E-12	1.74E-10
**DDX60L**	2681.952146	1.647373643	0.133317029	3.36E-36	7.67E-34
**IL12A**	18.53540533	1.647122083	0.903734125	0.00245415	0.029653767
**SRGN**	1545.507175	1.64488541	0.180109258	5.4E-21	6.48E-19
**LYPD5**	1045.840597	1.63643535	0.164205194	1.84E-24	2.85E-22
**CBR3**	156.197381	1.632529721	0.214215652	1.99E-15	1.69E-13
**PTN**	14.13466268	1.617597068	0.919835953	0.00292361	0.033912166
**AC012020.1**	50.26063816	1.61707893	0.343341891	1.56E-07	6.04E-06
**ENSG00000287070**	11.85029369	1.611699854	1.035892457	0.00351528	0.039332599
**ABCA10**	40.0161987	1.601335746	0.494533362	6.32E-05	0.001371013
**CYP4V2**	195.8216107	1.599978246	0.240982586	2.27E-12	1.52E-10
**SLC8A1-AS1**	19.43714127	1.59969548	0.811921087	0.00178981	0.02285523
**ERO1B**	38.00713756	1.596556208	0.422629752	1.13E-05	0.000295587
**MUC13**	15.84052813	1.595705143	0.749953696	0.0012325	0.016939412
**PROX1**	42.50816103	1.593889073	0.61713293	0.00044818	0.007449692
**PRICKLE1**	99.69455852	1.593129839	0.412405396	6.8E-06	0.000188936
**GOLGA5P1**	10.05292138	1.579870255	1.011811393	0.00396563	0.04314071
**IFIT5**	1489.273969	1.578144619	0.128444907	7.89E-36	1.74E-33
**RFX5**	2011.405639	1.575958606	0.121048502	8.03E-40	2.06E-37
**HELZ2**	1304.214755	1.573187238	0.144765169	1.21E-28	2.17E-26
**PLSCR1**	1482.057858	1.572899739	0.191680378	1.61E-17	1.61E-15
**IRF7**	232.4361989	1.569309274	0.269668389	3.56E-10	1.91E-08
**WDR64**	16.86923663	1.565989364	0.668897675	0.00096717	0.014066872
**CXCL2**	36.15057172	1.557795493	0.440522854	2.26E-05	0.000552725
**IFI6**	507.0624293	1.557131144	0.250704805	3.58E-11	2.15E-09
**HDAC9**	4086.510114	1.556426234	0.20768325	5.03E-15	4.21E-13
**AC099343.3**	79.98781263	1.553768049	0.327206732	1.68E-07	6.41E-06
**CCDC68**	161.5436268	1.551461956	0.271057067	8.59E-10	4.47E-08
**PCGF5**	922.634151	1.546464762	0.20564584	4.29E-15	3.62E-13
**ELMO1**	49.8083828	1.540386924	0.48337016	9.6E-05	0.001970431
**SLC38A4**	64.52031007	1.539088604	0.37129097	2.05E-06	6.41E-05

**AL160408.1**	43.4402752	1.537749353	0.602713947	0.00049050	0.008067273
**AC008079.2**	60.78598851	1.537051936	0.406094563	7.95E-06	0.000216036
**AC004067.1**	20.50643115	1.535652626	0.673033112	0.00111719	0.015728422
**CASP4**	3444.129951	1.531687813	0.123444002	1.75E-36	4.1E-34
**RNF19B**	1399.154647	1.530635839	0.184127482	6.96E-18	7.16E-16
**DDIT3**	139.5172747	1.530421717	0.290312699	1.07E-08	4.82E-07
**BEST3**	26.97098738	1.52953158	0.517156522	0.00017169	0.003239001
**AC069528.2**	29.25212104	1.515861568	0.769251201	0.00173370	0.02229752
**REC8**	43.40373984	1.508117714	0.491977004	0.00010488	0.002101436
**RIPK2**	1279.455327	1.507199278	0.191500006	2.59E-16	2.32E-14
**AL078459.1**	129.9793424	1.506511921	0.316556062	1.24E-07	4.9E-06
**AL592078.1**	13.93475009	1.496954928	0.865991283	0.00297491	0.034384656
**RASAL1**	30.25381599	1.489957145	0.478603782	0.00012417	0.002438284
**AVIL**	16.97992555	1.483720838	0.734382898	0.00166533	0.021604419
**AL356234.1**	104.3541053	1.478066307	0.496976738	0.00015536	0.002967048
**TMEM173**	18.93546034	1.477292417	0.793233636	0.00247873	0.029861224
**AC022916.1**	34.47738249	1.46102741	0.515427139	0.00023431	0.004232827
**TRAFD1**	689.5711816	1.460174515	0.139868942	1.54E-26	2.6E-24
**CLSTN3**	179.2465552	1.456448194	0.202727451	5.03E-14	3.86E-12
**RN7SKP116**	16.73742713	1.452153475	0.623866635	0.00099763	0.014398115
**HSPA5**	24611.3416	1.450316737	0.155868509	1.2E-21	1.52E-19
**CYLD**	1457.644935	1.450231556	0.20509927	1.18E-13	8.98E-12
**LINC01232**	315.56478	1.449232328	0.224230768	7.57E-12	4.81E-10
**RTEL1P1**	41.96776265	1.448166897	0.499707773	0.00019483	0.003617343
**AL390957.1**	156.9948632	1.448133467	0.253227877	6.96E-10	3.67E-08
**HMCN2**	20.29285503	1.440640626	0.872444352	0.00321305	0.036622082
**C1RL**	206.0224396	1.437149982	0.203995553	1.35E-13	1.01E-11
**THSD7B**	266.6901109	1.428863461	0.331602417	1.21E-06	3.96E-05
**RBM11**	46.88966417	1.424314793	0.460113096	0.00010400	0.002092676
**HMGN2P46**	21.36013498	1.419152109	0.824890052	0.00295252	0.034176224
**LINC01138**	213.7152313	1.416915403	0.258393963	2.87E-09	1.39E-07
**ZNFX1**	2455.577167	1.416564643	0.137760965	7.34E-26	1.19E-23

**ZNF334**	24.77938966	1.415341787	0.531921118	0.00041261	0.006913914
**ZP4**	187.1981284	1.413228931	0.410310245	3.54E-05	0.000823928
**SLC4A9**	20.48881253	1.409746018	0.61061041	0.00099019	0.014312674
**AC108673.2**	21.15669652	1.406754453	0.588126932	0.00097894	0.014215912
**TAPBP**	6297.215262	1.405209379	0.169447924	7.36E-18	7.54E-16
**AC122718.1**	20.63705315	1.396891818	0.579434673	0.00096431	0.014039992
**C5orf30**	728.5469082	1.394727204	0.175371935	1.35E-16	1.25E-14
**AL645939.2**	71.1146447	1.393663163	0.633434004	0.00124019	0.017032664
**PRDM9**	149.5870989	1.392436004	0.253466882	2.71E-09	1.31E-07
**AC244669.1**	11.57261093	1.389562501	0.895483837	0.00401348	0.043560051
**TMEM154**	217.2873504	1.386093823	0.315319554	8.62E-07	2.87E-05
**ERVV-1**	22.63038344	1.380357206	0.796336063	0.00291245	0.033835746
**BTC**	65.06694124	1.379137184	0.480878459	0.00023930	0.004310468
**DRAM1**	336.7750123	1.377997921	0.177738166	8.03E-16	7.07E-14
**SLC2A12**	120.5777268	1.376160245	0.361358327	8.64E-06	0.000233986
**PKN2-AS1**	29.64633703	1.370651958	0.679866445	0.00173423	0.02229752
**MR1**	367.7712692	1.369529035	0.238849985	7.04E-10	3.69E-08
**FMO4**	46.01574819	1.356944841	0.458829357	0.00017946	0.003372013
**ARNTL2**	822.5122741	1.354262072	0.172593619	3.84E-16	3.42E-14
**ENSG00000286912**	16.44864011	1.350599696	0.830797184	0.00446251	0.047274813
**ETV6**	281.5659973	1.342105342	0.247349445	4.41E-09	2.07E-07
**FIRRE**	178.9715342	1.340732169	0.236479565	1.05E-09	5.4E-08
**SAMD9**	17323.51415	1.333031822	0.175210174	2.5E-15	2.12E-13
**TRIM5**	840.9421509	1.332932643	0.210949992	2.09E-11	1.28E-09
**JAK2**	533.4719186	1.329310438	0.163501816	3.51E-17	3.46E-15
**AF165147.1**	27.05095185	1.329065791	0.575814498	0.00115589	0.016111191
**IFNAR2**	81.82453443	1.31934023	0.275588744	1.41E-07	5.48E-06
**NOS3**	63.56273185	1.318566306	0.348433317	1.16E-05	0.000302142
**HNRNPA1P22**	31.85777577	1.318084268	0.599051427	0.00127776	0.017437957
**PMAIP1**	2300.730023	1.315829667	0.187417035	1.86E-13	1.38E-11
**LINC00520**	36.26692188	1.315483945	0.576069755	0.00120329	0.016647883
**DPPA2**	42.91176569	1.311855885	0.405699348	9.58E-05	0.001967242

**ANK2**	63.46228954	1.311484198	0.524179869	0.00080536	0.012225966
**CD47**	2403.364956	1.309290897	0.14400405	8.48E-21	1.01E-18
**DGLUCY**	364.2126407	1.306592517	0.195955868	2.01E-12	1.36E-10
**FAM167B**	33.08399398	1.293353878	0.555477653	0.00110074	0.015562419
**HSH2D**	38.1630252	1.292670718	0.439978518	0.00021673	0.003953402
**PRDM1**	845.2931975	1.291611198	0.273072268	1.84E-07	6.95E-06
**LACTB**	241.0271901	1.290206255	0.313788481	2.87E-06	8.72E-05
**PDP1**	1875.281566	1.285379087	0.119591001	5.82E-28	1E-25
**PARP11**	92.98553186	1.282100352	0.370743063	4.3E-05	0.000983484
**LINC01359**	17.4683208	1.272233472	0.791525973	0.00413800	0.044652751
**MTMR9LP**	22.26518164	1.271374582	0.578420768	0.00144070	0.019127395
**HSP90B1**	22324.98596	1.270544598	0.10674211	1.06E-33	2.16E-31
**OTOGL**	13.05905196	1.269132445	0.819495445	0.00457867	0.048213824
**RHBDF2**	458.3519094	1.266584409	0.155563721	3.7E-17	3.63E-15
**TMEM38A**	47.71634795	1.265589794	0.3568337	3.16E-05	0.000743516
**LINC00973**	74.725303	1.259909931	0.525843791	0.00098542	0.014280136
**MAGEA1**	53.51387415	1.258031508	0.38493486	8.84E-05	0.001833921
**RBM43**	40.57547998	1.256109394	0.533074613	0.00096458	0.014039992
**AL359924.1**	94.00382592	1.253202256	0.306748982	3.55E-06	0.00010597
**AP003170.1**	18.69441987	1.2516563	0.654087725	0.00268322	0.031752956
**HERPUD1**	1240.222045	1.248661764	0.202092495	6.09E-11	3.55E-09
**AL031316.1**	14.02946375	1.248124708	0.777835708	0.00434412	0.046343134
**STAT5A**	59.34769336	1.24009201	0.388848613	9.27E-05	0.001915261
**AC245297.3**	146.7062373	1.239498609	0.221249467	1.73E-09	8.7E-08
**RYR3**	94.98802367	1.238564016	0.537172612	0.00124965	0.01714095
**LPCAT4**	266.5009653	1.236317944	0.275578829	6.08E-07	2.09E-05
**AC009720.1**	24.09240709	1.236241467	0.546870919	0.00134286	0.018097722
**ZMYND15**	48.76110756	1.228491409	0.439034799	0.00032711	0.005635016
**ADGRF4**	768.7635944	1.227447613	0.140816119	2.82E-19	3.05E-17
**ADGRE1**	177.7653001	1.223122659	0.24823297	7.97E-08	3.23E-06
**IGFN1**	167.2967519	1.22009552	0.244260504	4.98E-08	2.06E-06
**KYNU**	267.0694362	1.219699447	0.362773137	5.9E-05	0.001299304

**VPS13C**	1740.019294	1.219254984	0.146523432	8.6E-18	8.75E-16
**CYP27B1**	34.09518797	1.211200687	0.503226315	0.00113174	0.015857054
**NLRP3**	325.4171733	1.20764283	0.234018138	2.46E-08	1.06E-06
**IDUA**	17.71073666	1.195956199	0.658826518	0.00392028	0.042796387
**XRN1**	2807.610904	1.194653158	0.12771885	8.42E-22	1.08E-19
**XCR1**	21.83450349	1.193127359	0.590201409	0.00281956	0.032986087
**GSTK1**	1161.008588	1.192729869	0.121667729	1.13E-23	1.63E-21
**RBCK1**	1047.153075	1.191563497	0.126380996	4.84E-22	6.39E-20
**NOS2**	41.67316467	1.184556273	0.562404422	0.00216342	0.02672519
**NT5C3A**	809.4356571	1.182441802	0.182626872	8.78E-12	5.54E-10
**IFIT2**	1635.852725	1.178723636	0.171993057	6.96E-13	4.9E-11
**CPEB3**	78.90652651	1.177740962	0.331391337	3.19E-05	0.000750637
**ABHD16A**	26.12640566	1.17647404	0.548620653	0.00201721	0.02519205
**STOML1**	66.82444716	1.169434674	0.367400617	0.00011996	0.002365509
**LIPH**	363.3520786	1.168011153	0.302240275	9.97E-06	0.00026405
**AC008269.1**	64.38547732	1.166193392	0.332023079	3.6E-05	0.000836951
**SORBS1**	131.3400444	1.151656074	0.402415944	0.00030663	0.005353369
**ZNF192P1**	82.28847419	1.150269881	0.36694023	0.00012246	0.002412246
**AC090502.1**	24.38045913	1.150173867	0.550343432	0.00219424	0.026965851
**PROS1**	32.87298585	1.148716422	0.479475367	0.00105562	0.015061008
**TRIM38**	1413.610669	1.145385188	0.203772185	1.91E-09	9.53E-08
**CALCOCO2**	2165.242065	1.14303051	0.125816147	1.1E-20	1.29E-18
**EIF2AK3**	1268.173661	1.139844954	0.169642367	2.08E-12	1.4E-10
**IL22RA1**	248.960195	1.139294759	0.280018846	4.26E-06	0.000123381
**AC025171.2**	354.9692231	1.138702148	0.192141322	3.37E-10	1.83E-08
**AL354872.2**	34.21264245	1.134692256	0.521658291	0.00174791	0.022427219
**FAM129A**	243.9155648	1.133410566	0.309978231	2.44E-05	0.000594357
**HDX**	18.17666816	1.1328958	0.643007394	0.00408598	0.044218852
**CLIC5**	2617.674184	1.1291412	0.221194007	3.35E-08	1.41E-06
**AL365184.1**	79.0609683	1.12898144	0.416051826	0.00056790	0.009061756
**AC005323.2**	21.86117035	1.127275375	0.554303916	0.00283098	0.033062918
**PHF11**	432.0573764	1.125954274	0.156199673	5.87E-14	4.49E-12

**TXNIP**	1596.494281	1.125244624	0.14917074	4.82E-15	4.05E-13
**AC092944.1**	67.07985876	1.123171874	0.356837511	0.00015479	0.002961971
**AC010328.1**	61.46890661	1.120866976	0.36400925	0.00015537	0.002967048
**ITPRIP**	268.6979389	1.119143471	0.298898651	1.72E-05	0.000429746
**PLA2G4C**	88.92539481	1.116216702	0.400033651	0.00043678	0.007292647
**GCH1**	384.2859478	1.115812539	0.241295757	3.52E-07	1.27E-05
**AC105233.5**	58.39199707	1.110223929	0.41109272	0.00056508	0.009024547
**LINC-PINT**	847.4135922	1.109834988	0.164045351	1.52E-12	1.04E-10
**ILDR1**	101.2877822	1.109648725	0.32826713	6.67E-05	0.001425397
**NUDCD1**	1071.464807	1.109438055	0.169503995	6.76E-12	4.34E-10
**MANF**	795.8892737	1.106338088	0.171991729	1.48E-11	9.16E-10
**C19orf66**	314.1410684	1.102800487	0.198011831	2.54E-09	1.25E-07
**TRIM14**	579.2891611	1.102339645	0.185973881	3.38E-10	1.83E-08
**SP140L**	826.092821	1.098338947	0.146219665	6.18E-15	5.1E-13
**AL138828.1**	63.31654813	1.089984762	0.628056043	0.00470320	0.04922079
**AL049629.1**	26.64412594	1.089408841	0.487020859	0.00189283	0.023974677
**TDRD7**	788.1270997	1.085726689	0.198944894	5.03E-09	2.34E-07
**MIR29B2CHG**	240.09387	1.082179699	0.247864863	1.2E-06	3.92E-05
**TNF**	239.6547053	1.079882904	0.343067849	0.00015016	0.002888158
**RAG1**	170.5163024	1.079061081	0.25646269	2.52E-06	7.75E-05
**ZG16B**	65.17895865	1.076324685	0.411510037	0.00066284	0.010347479
**MXD1**	1736.289587	1.074384081	0.527395416	0.00289514	0.033707102
**UCA1**	3695.570614	1.073931985	0.233199148	4.33E-07	1.52E-05
**RGS7**	107.1298025	1.069479157	0.31380933	6.92E-05	0.001472462
**PCDH7**	1104.582074	1.067991933	0.149747411	1.22E-13	9.22E-12
**NFKBIZ**	685.5010103	1.065512427	0.189689239	2.22E-09	1.1E-07
**CT83**	140.6394007	1.06511351	0.23638718	7.59E-07	2.55E-05
**AC016831.5**	313.2298097	1.063810815	0.209004804	4.35E-08	1.82E-06
**PSMA4**	2508.370106	1.062953768	0.109756493	4.48E-23	6.18E-21
**ZBED6CL**	58.74184864	1.061900932	0.372218312	0.00042651	0.007133903
**NOD1**	217.0634519	1.061808287	0.189102201	2.2E-09	1.09E-07
**LINC02407**	215.0406058	1.058707739	0.262576165	6.1E-06	0.000170878

**ZNF516**	25.19279017	1.057464309	0.524167935	0.00295368	0.034176224
**MIR762HG**	70.34280787	1.05266689	0.309839762	6.26E-05	0.001360705
**PPP1R15A**	1339.997922	1.049765796	0.175217244	2.49E-10	1.36E-08
**MSRB1**	295.2774221	1.049358728	0.187688173	2.96E-09	1.43E-07
**HELB**	163.5838101	1.048555909	0.226688413	4.44E-07	1.56E-05
**CASP7**	601.4298298	1.044796373	0.149585112	3.17E-13	2.31E-11
**WHAMMP3**	44.13538687	1.044659692	0.469649967	0.00176527	0.022581592
**STEAP4**	1903.137994	1.042903557	0.202695128	2.99E-08	1.27E-06
**BACH1**	3915.775212	1.040444775	0.147014912	1.75E-13	1.31E-11
**PODXL**	743.8868805	1.038495735	0.229585023	6.46E-07	2.2E-05
**MOV10**	2346.85636	1.037874205	0.135105246	1.82E-15	1.56E-13
**NAV3**	82.77979891	1.037560575	0.462063887	0.00200890	0.025155594
**CCNL1**	2030.994894	1.032449577	0.133880652	1.51E-15	1.3E-13
**HERC5**	582.1444772	1.02902308	0.32353872	0.00013756	0.002667662
**ATF3**	862.2201134	1.027215852	0.218569768	2.85E-07	1.04E-05
**ZSCAN12P1**	244.9118366	1.025528018	0.222083741	4.78E-07	1.66E-05
**SALL4**	94.86125173	1.024461633	0.268808311	1.37E-05	0.000348977
**ZC3HAV1**	3579.071447	1.023417058	0.123769113	1.89E-17	1.88E-15
**ADAM20**	33.70199585	1.022344631	0.450917265	0.00191503	0.024157793
**SMIM14**	407.2605553	1.021004643	0.247895092	3.86E-06	0.000114291
**SLFN12**	370.6802318	1.019883217	0.170323415	2.69E-10	1.47E-08
**MAST4-IT1**	83.24829372	1.017748903	0.274850233	2.35E-05	0.000572273
**GSDMD**	401.56113	1.016983784	0.175024454	7.4E-10	3.87E-08
**ACTR3C**	63.31271546	1.015955346	0.348102809	0.00033051	0.005675032
**KLK10**	236.8054441	1.01548138	0.298977524	6.76E-05	0.001440439
**NKX3-1**	109.2284451	1.013842051	0.26920502	1.74E-05	0.000434484
**PLAUR**	1913.82542	1.00895474	0.247641177	5.13E-06	0.000146331
**IRF2**	573.2866567	1.007120102	0.159599976	3.38E-11	2.03E-09
**TNFSF15**	7892.141052	1.001756952	0.187272104	1.04E-08	4.68E-07
**PDIA4**	4640.449057	1.000857351	0.148315971	1.93E-12	1.31E-10
**ZNF165**	1203.505872	1.000056949	0.205257273	1.37E-07	5.34E-06
**ZBTB20**	72.86544579	0.999879887	0.407630217	0.00119053	0.016508017

**FEZ1**	218.8853063	0.999817955	0.244182543	5.24E-06	0.000148597
**HCG27**	289.7949719	0.999199449	0.212960865	3.02E-07	1.1E-05
**PARP10**	456.1399174	0.994268466	0.19602822	4.7E-08	1.95E-06
**AP000662.1**	90.16718854	0.993484581	0.356095124	0.00051736	0.008427664
**ZNF267**	1074.481827	0.993402568	0.21832496	6.12E-07	2.1E-05
**DENND3**	517.0180069	0.993241637	0.163370553	1.59E-10	8.89E-09
**GPR137B**	28.39562637	0.992196668	0.512566816	0.00367042	0.040685292
**AC092349.1**	64.98109434	0.992004837	0.457014996	0.00254709	0.030382822
**GSTO1**	1428.170474	0.987744046	0.133490991	1.78E-14	1.4E-12
**BTN2A3P**	51.32100299	0.985133505	0.364864103	0.00070702	0.010951942
**AC008632.1**	82.35053537	0.979959531	0.456081105	0.00248394	0.029904771
**OSMR-AS1**	189.4958033	0.97749929	0.298426129	0.00010433	0.00209486
**CDKL1**	54.55170629	0.973852747	0.353824377	0.00061238	0.009640154
**LINC01772**	72.6497421	0.968390543	0.352159379	0.00055741	0.008932412
**CNDP2**	2031.870316	0.967431715	0.120351016	1.26E-16	1.17E-14
**ITPKC**	628.6272304	0.963062826	0.211897514	6.89E-07	2.34E-05
**ADAR**	9089.427038	0.960934551	0.115369935	1.15E-17	1.16E-15
**IGFBP6**	144.8010744	0.96027136	0.242756063	8.37E-06	0.000227292
**DOC2A**	50.77416877	0.958565887	0.52639377	0.00463480	0.048668019
**OGFR**	883.9145475	0.95726659	0.127768782	9.24E-15	7.43E-13
**ARMCX1**	61.22160458	0.957044162	0.416929316	0.00192148	0.024222797
**FGF1**	47.59872328	0.951602035	0.493354155	0.00473021	0.049475771
**CREBRF**	676.001482	0.94884906	0.282765386	8.74E-05	0.001814134
**AL137058.1**	83.03862235	0.94878407	0.28549613	9.98E-05	0.002025505
**SERPINB1**	1123.976649	0.947510113	0.226109645	3.34E-06	0.000100449
**SQOR**	369.2757792	0.947468777	0.206473162	5.22E-07	1.81E-05
**LINC01844**	110.7982634	0.945272227	0.484755836	0.00394974	0.042992742
**E2F5**	141.6010373	0.941505167	0.272716677	6.49E-05	0.001396488
**LONRF1**	135.7584869	0.939526499	0.361137622	0.00090094	0.013362955
**SEMA3D**	1459.494412	0.936770661	0.209445183	1.01E-06	3.32E-05
**AL136985.3**	143.1831931	0.933999664	0.250771982	2.52E-05	0.000609744
**CREM**	78.5881027	0.926280619	0.282631058	0.00012657	0.002480204

**SIX1**	125.4158369	0.924366239	0.265396568	5.71E-05	0.001264695
**THBS3**	183.5387516	0.922577046	0.228954091	7.01E-06	0.00019446
**ST6GALNAC2**	313.0780678	0.9185353	0.184204025	7.82E-08	3.18E-06
**HAS3**	1195.62535	0.9173192	0.178231529	3.58E-08	1.5E-06
**MAFF**	1074.513143	0.914456259	0.188851782	1.76E-07	6.68E-06
**NABP1**	1905.167401	0.910324065	0.17078551	1.42E-08	6.33E-07
**MAX**	564.0286359	0.905925661	0.149648139	2.19E-10	1.21E-08
**SLC25A28**	315.1849963	0.902594899	0.170896913	1.77E-08	7.76E-07
**CD274**	1740.52696	0.902367423	0.293883973	0.00025535	0.004547046
**TBC1D9**	141.5755988	0.901361167	0.244500277	2.74E-05	0.000657459
**PAXIP1-AS2**	162.3533	0.899373952	0.231009227	1.27E-05	0.000327346
**SCAMP1-AS1**	115.1624081	0.896905832	0.244235498	3.2E-05	0.000750637
**TRIM26**	2967.064581	0.896523535	0.11998595	9.92E-15	7.94E-13
**TRIM25**	3927.310507	0.895863263	0.121101761	2.03E-14	1.6E-12
**OAF**	342.0681198	0.893251283	0.182148791	1.33E-07	5.23E-06
**RNF149**	1014.815407	0.88832924	0.130678427	1.6E-12	1.09E-10
**PCYT1A**	1381.173193	0.881017011	0.135122515	1.09E-11	6.85E-10
**DUXAP8**	104.7384238	0.877761111	0.252491548	6.97E-05	0.001480437
**LTF**	140.3526979	0.875436266	0.235263887	2.51E-05	0.000609744
**C1orf74**	1486.775195	0.875266686	0.246757862	5.07E-05	0.001138455
**ZNF841**	1285.33409	0.87463918	0.289279902	0.00030748	0.005363164
**TRIM56**	2305.92761	0.872890196	0.116078496	8.54E-15	6.9E-13
**RNU6-26P**	58.8835074	0.872515718	0.442269165	0.00436799	0.046571219
**XDH**	3235.686861	0.871180558	0.155866868	3.48E-09	1.66E-07
**GPR158**	467.3311639	0.869995085	0.155266541	3.26E-09	1.56E-07
**PCAT1**	257.5329506	0.869373449	0.204211418	3.13E-06	9.49E-05
**BACH1-IT2**	192.684315	0.868560032	0.21882062	1.02E-05	0.000270614
**MUC16**	25985.485	0.867704882	0.125124549	6.35E-13	4.49E-11
**FNBP4**	2170.296234	0.862997989	0.157497627	6.57E-09	3E-07
**MYO1B**	1015.053734	0.857629351	0.155677074	5.62E-09	2.59E-07
**NFE2L3**	646.3636249	0.855826587	0.156276795	6.5E-09	2.98E-07
**BAK1**	255.5567889	0.854100438	0.204733493	4.64E-06	0.000133973

**ASS1**	132.1011767	0.853571564	0.271028066	0.00021147	0.003865003
**MAST4**	4626.068437	0.851516051	0.160008389	1.6E-08	7.04E-07
**RHEBL1**	100.6366475	0.851124559	0.403039398	0.00351690	0.039332599
**PAIP1P1**	111.7661627	0.849192652	0.266143456	0.00019317	0.003594513
**TMSB10**	7106.767755	0.847690139	0.10391322	4.15E-17	4.05E-15
**Z82243.1**	56.09504204	0.846064967	0.359019532	0.00205180	0.025556166
**OR2A20P**	43.11683996	0.846017746	0.400778066	0.00364443	0.040445042
**CASP8**	1309.195305	0.845853531	0.137807656	1.36E-10	7.74E-09
**SUSD6**	488.6065898	0.838354573	0.206195316	7.28E-06	0.000201266
**BACH1-IT3**	39.75859974	0.837048402	0.394490826	0.00360263	0.040058656
**CTH**	794.516881	0.832587062	0.17272237	2.29E-07	8.48E-06
**GTPBP1**	1108.456176	0.830402188	0.128116049	1.55E-11	9.55E-10
**BLVRA**	221.9907444	0.829671964	0.203101143	6.29E-06	0.000175734
**LINC01619**	58.08759365	0.824624037	0.324987402	0.00136056	0.018272984
**AL137782.1**	1067.66329	0.824401712	0.141648204	9.83E-10	5.07E-08
**PRRG4**	940.1859622	0.823293172	0.179707148	7.08E-07	2.4E-05
**DNAJB9**	400.9189637	0.822649704	0.219448827	2.58E-05	0.000621698
**SCYL3**	256.429372	0.818294716	0.189140856	2.35E-06	7.28E-05
**EIF2AK2**	5396.02637	0.814491682	0.147422846	5.48E-09	2.53E-07
**TMEM219**	603.0418462	0.813987166	0.157493391	3.87E-08	1.62E-06
**DUSP6**	596.7614532	0.8138166	0.193464748	3.95E-06	0.000115675
**DNPEP**	561.7375758	0.812012408	0.182674962	1.47E-06	4.7E-05
**KLF6**	2369.038762	0.811153577	0.165612284	1.6E-07	6.16E-06
**AL645933.2**	74.38523019	0.810940152	0.348206578	0.00238009	0.028858433
**ABCD1**	86.30231193	0.810190751	0.295758624	0.00079821	0.012147054
**FAM122C**	78.59970506	0.808421206	0.308841813	0.00114189	0.015951567
**BIRC3**	6336.297649	0.808191382	0.291606318	0.00074472	0.011425786
**CARD6**	387.1007793	0.807188798	0.201490374	9.5E-06	0.00025376
**C6orf62**	3344.415935	0.80166691	0.120304889	4.54E-12	2.94E-10
**SESN2**	284.2176692	0.800436857	0.322935785	0.00180067	0.022946933
**PROCR**	283.9936919	0.795005335	0.184958317	2.68E-06	8.19E-05
**CIR1**	411.8391901	0.79066105	0.187714477	3.94E-06	0.000115559

**MITD1**	498.6776277	0.790314415	0.158173043	9.53E-08	3.81E-06
**LINC00900**	89.69054168	0.789418753	0.291109487	0.00097089	0.014110036
**RWDD2A**	136.9230491	0.788572662	0.258646422	0.00032853	0.005651499
**BACH1-IT1**	143.8211924	0.78669604	0.223517379	7.2E-05	0.001518975
**TFAP2A-AS1**	159.4292753	0.78616118	0.253141087	0.00027960	0.00492741
**AC019117.1**	113.4270835	0.786121978	0.285232612	0.00082864	0.012508397
**PDIA3P1**	119.1957799	0.785558651	0.280857502	0.00072479	0.011184155
**L3HYPDH**	223.2973414	0.785220245	0.20913281	3.01E-05	0.00071393
**ARHGEF35**	121.754657	0.783558358	0.265829819	0.00048068	0.007926705
**LGALS8**	1459.426914	0.781606808	0.231356217	0.00011398	0.002257439
**VEGFC**	455.2991898	0.77972909	0.156332452	1.08E-07	4.28E-06
**CALR**	17466.29569	0.779562247	0.112406993	7.04E-13	4.92E-11
**LIF**	819.1006814	0.778220851	0.269043291	0.00057516	0.009138754
**UGDH**	509.0437093	0.774823554	0.164577212	4.63E-07	1.62E-05
**HMGA1P4**	101.1779774	0.774301197	0.269197585	0.00058716	0.009297865
**ANKRD10-IT1**	649.5588793	0.773335998	0.178806813	2.62E-06	8.02E-05
**TEC**	188.2092231	0.770422393	0.283329391	0.00093146	0.013716739
**N4BP2L2-IT2**	585.0317774	0.770398173	0.159358126	2.27E-07	8.44E-06
**PSMA5**	2464.630011	0.770252748	0.133889983	1.66E-09	8.4E-08
**LAT2**	191.9491913	0.770073295	0.191357848	9.8E-06	0.00026074
**STAT3**	3356.326693	0.769825643	0.12470899	1.26E-10	7.19E-09
**ATXN7**	1170.702442	0.768680405	0.125867895	1.86E-10	1.03E-08
**CAMK2D**	1528.820958	0.768096598	0.128103844	3.92E-10	2.09E-08
**MYCBP2**	5325.571255	0.767316066	0.113548688	2.66E-12	1.74E-10
**LINS1**	215.5208213	0.765423688	0.209451533	4.15E-05	0.000949835
**SAT1**	1024.174978	0.757049196	0.162728659	5.88E-07	2.03E-05
**S100P**	189.9547114	0.756800507	0.25511705	0.00050324	0.008240599
**TCIRG1**	290.0649288	0.753863605	0.168910575	1.47E-06	4.7E-05
**MORC3**	1010.497322	0.747513357	0.16311682	8.29E-07	2.77E-05
**SLC25A22**	543.0437759	0.746814856	0.170439391	2.28E-06	7.1E-05
**XBP1**	2388.611994	0.740042364	0.213998462	9.8E-05	0.002000587
**USF1**	640.3040372	0.733803571	0.149845176	1.97E-07	7.44E-06

**OTUD4**	2331.989102	0.733222501	0.164702653	1.66E-06	5.27E-05
**MYO6**	3292.393531	0.731909824	0.115518218	4.97E-11	2.93E-09
**AC139887.2**	195.2559061	0.729588234	0.192622198	2.94E-05	0.000699954
**ZFP36**	576.7303891	0.729086617	0.170964728	3.87E-06	0.000114337
**SQSTM1**	8138.145264	0.724879352	0.213372884	0.00011680	0.002308112
**NUCB1**	912.9407159	0.724799375	0.152188039	3.8E-07	1.36E-05
**PPA1**	1974.03327	0.724007324	0.144114496	1.02E-07	4.06E-06
**BBS12**	78.93870545	0.723348592	0.274831899	0.00142309	0.018907017
**MTHFR**	469.9324831	0.720651851	0.16928296	3.91E-06	0.000115003
**APOBEC3B**	752.1573654	0.71956055	0.229365311	0.00030498	0.005329477
**MAT2B**	1322.455106	0.719498813	0.142451958	9.9E-08	3.96E-06
**CXorf38**	250.9906083	0.719160834	0.2079072	9.8E-05	0.002000587
**MLLT3**	128.8565351	0.71900453	0.309199255	0.00304448	0.034986915
**SYNPO2**	109.6539957	0.716921309	0.268446317	0.00132324	0.017897699
**CDCP1**	3373.715672	0.715444338	0.153663515	6.62E-07	2.26E-05
**SDF2L1**	349.7887095	0.710401963	0.225395906	0.00031135	0.005415582
**LRRN3**	164.2250747	0.708777648	0.204589172	0.00010446	0.002095152
**PPP1R26-AS1**	180.2102841	0.703976133	0.230535204	0.00039499	0.0066661
**PDIA3**	7812.326596	0.702376777	0.107541727	1.58E-11	9.68E-10
**GABARAPL1**	482.3959384	0.702190609	0.193325342	5.34E-05	0.001192617
**MAP3K8**	454.9916226	0.701518308	0.194906951	6.08E-05	0.001328779
**SERPINB8**	505.0453082	0.701442702	0.225909323	0.00036106	0.00614336
**GCNT2**	159.0144514	0.697140416	0.214061146	0.00022048	0.004010111
**LINC00467**	245.4362947	0.696950053	0.239009476	0.00061895	0.009727262
**NAMPT**	5010.209454	0.69568083	0.172291949	1.12E-05	0.000293995
**PHLDA1**	4265.172905	0.693378929	0.1989443	9.85E-05	0.002002901
**KARS**	3932.975009	0.693182026	0.108121707	3.25E-11	1.97E-09
**SMG1**	7874.181968	0.692204399	0.102209366	2.39E-12	1.59E-10
**TPRN**	237.881758	0.690922661	0.215674919	0.00027547	0.004863825
**NBN**	2411.437272	0.690669306	0.123839654	5.39E-09	2.49E-07
**CEBPB**	370.0162669	0.690461569	0.188111685	5.18E-05	0.001161417
**FAM46A**	1578.460952	0.690022461	0.187982373	4.89E-05	0.001102697

**UBR2**	1734.581753	0.689821246	0.124687443	7.01E-09	3.2E-07
**CRELD2**	310.8155437	0.689776128	0.188631664	5.03E-05	0.001132126
**MOB3C**	609.8869488	0.687371036	0.182433183	3.32E-05	0.00077579
**GFPT1**	2653.413605	0.685614452	0.140688149	2.45E-07	9E-06
**CFLAR**	2496.05803	0.682118025	0.158718775	3.71E-06	0.000110377
**CEACAM1**	5643.038628	0.681767758	0.232978202	0.00069487	0.0108026
**SLC30A7**	1383.278344	0.681279045	0.155911273	2.79E-06	8.49E-05
**IGFBP7**	345.2727005	0.681134443	0.182995569	4.11E-05	0.000944253
**ETS1**	3978.668835	0.680419705	0.16625198	9.45E-06	0.000252961
**RNASE7**	1652.13974	0.679711953	0.223398614	0.00046376	0.00768819
**C4orf50**	189.2128521	0.679524719	0.278122166	0.00268737	0.031753647
**NRK**	1509.838627	0.676117878	0.297795088	0.0039197	0.042796387
**PDIA6**	8184.785916	0.676102862	0.106393537	4.77E-11	2.84E-09
**ZNF699**	140.8064418	0.675985946	0.234567685	0.00074237	0.01140844
**DUSP5**	1382.92182	0.674295213	0.185700187	5.91E-05	0.00129985
**ANKRD10**	4738.331634	0.673771492	0.127371637	2.82E-08	1.2E-06
**LGMN**	440.5012487	0.673634299	0.197444873	0.00012921	0.002520633
**FBXO7**	1969.073237	0.672609957	0.116927448	2.03E-09	1.01E-07
**C5orf15**	1201.931259	0.671556251	0.125016096	1.83E-08	7.99E-07
**SLC5A3**	3475.261247	0.669663536	0.2276206	0.00061553	0.009681669
**RNF152**	424.3670218	0.669079198	0.188530453	7.86E-05	0.001648655
**ECM1**	3173.471521	0.667280511	0.117323982	2.99E-09	1.43E-07
**PALM2-AKAP2**	6731.940195	0.665160843	0.137440068	3.05E-07	1.11E-05
**SH2B3**	156.3110401	0.663948669	0.241517203	0.00122318	0.016848521
**AC099329.2**	118.1506509	0.662500523	0.234649227	0.00095330	0.01390826
**EID3**	161.8264713	0.660054083	0.261220153	0.00221973	0.027213598
**MICA**	1498.847881	0.659894717	0.181182932	5.91E-05	0.00129985
**TNFAIP3**	8550.053203	0.655660753	0.180522482	6.34E-05	0.001371043
**NFKBIA**	1243.018184	0.654662743	0.19762757	0.00019948	0.003685441
**ZC3H12A**	260.4301922	0.65371116	0.212776587	0.00044189	0.007358198
**ACP2**	592.4848226	0.651916991	0.160149077	1.12E-05	0.0002928
**GTPBP2**	931.0481175	0.651512006	0.147529653	2.38E-06	7.34E-05

**ANKRD13A**	2422.037219	0.650748567	0.190613602	0.00014425	0.002787585
**MCL1**	7830.528722	0.650672228	0.12997519	1.34E-07	5.23E-06
**LINC00649**	1329.563147	0.650073788	0.195358202	0.00019322	0.003594513
**RNF114**	1138.962583	0.650054995	0.126452184	6.75E-08	2.76E-06
**RBM7**	382.6883558	0.649524722	0.168725961	2.63E-05	0.000632601
**UBE2Z**	2015.615305	0.647298327	0.130658177	1.77E-07	6.73E-06
**RBMXL1**	685.1605535	0.646262191	0.178531408	6.5E-05	0.00139703
**ZNF140**	230.5827053	0.644788507	0.178400278	6.95E-05	0.001478855
**PHC2**	1009.041478	0.644379923	0.175814888	5.87E-05	0.001296122
**MAST3**	260.5247814	0.643733148	0.218492773	0.00069463	0.0108026
**ZBTB43**	1052.595586	0.640555298	0.165520047	2.53E-05	0.00061072
**KIAA1551**	311.3723502	0.634465529	0.17842597	8.9E-05	0.001845138
**ENSG00000285820**	224.1172915	0.63269952	0.25419044	0.00270925	0.031968864
**KRT6A**	453.2092774	0.632069445	0.26022131	0.00319731	0.036521556
**SPATS2L**	2085.113127	0.631089808	0.17038778	5.07E-05	0.001138455
**CASZ1**	475.0564454	0.630489137	0.15460033	1.16E-05	0.000302142
**GPD2**	1486.608495	0.629386451	0.131045949	3.99E-07	1.42E-05
**AC025031.5**	143.5227055	0.628800525	0.241745812	0.00190869	0.024126526
**MBOAT1**	2554.267929	0.627973034	0.143887745	3.18E-06	9.63E-05
**ST5**	1419.539826	0.62751175	0.152128804	9.44E-06	0.000252961
**VMP1**	6668.920013	0.627047618	0.130704224	4.21E-07	1.49E-05
**AC234772.3**	144.3734944	0.626248839	0.22795593	0.00136597	0.018277682
**SLC9A7**	907.8343681	0.626142736	0.248598497	0.00252246	0.030220311
**UBXN4**	2122.155366	0.626060179	0.129102641	3.87E-07	1.38E-05
**PRKD2**	406.8734441	0.62560146	0.175632208	8.97E-05	0.001857728
**UBE2D1**	181.0641423	0.62103623	0.212407756	0.00080248	0.012192135
**ZNF311**	157.3961216	0.619795886	0.223473712	0.00123248	0.016939412
**LRCH1**	365.268598	0.618696492	0.18895568	0.00025375	0.004522877
**SETX**	5444.163568	0.618417883	0.125512223	2.19E-07	8.17E-06
**NDUFA9**	130.3329623	0.617807923	0.267300565	0.00438208	0.046694823
**PSMA6**	138.1792881	0.614986805	0.23075896	0.00175221	0.022466968
**MAP3K13**	288.1833785	0.614872095	0.24558524	0.00280332	0.032903982

**USP33**	1899.767932	0.614492857	0.122713884	1.45E-07	5.61E-06
**ELOVL7**	322.4199518	0.613852031	0.18954455	0.00028212	0.004962494
**NUBP1**	915.1220762	0.61313799	0.140085979	3.22E-06	9.73E-05
**KIF13B**	424.5366379	0.612230027	0.189075042	0.00029265	0.005133267
**KLF5**	1935.380404	0.611553314	0.136718921	2.02E-06	6.34E-05
**RGS20**	341.6308086	0.610520442	0.176241054	0.00012869	0.002513908
**USP43**	203.039895	0.610188232	0.220209728	0.00129139	0.017568461
**RICTOR**	5217.108766	0.608708631	0.166191792	6.34E-05	0.001371043
**UBC**	13409.6968	0.606979502	0.146363469	9.81E-06	0.00026074
**PTPN6**	206.0226381	0.605404715	0.214439758	0.00108997	0.015454724
**CPNE1**	738.464803	0.605236771	0.144452009	7.63E-06	0.000209614
**ABCA5**	1193.373147	0.604556011	0.155232646	2.52E-05	0.000610683
**KIAA0040**	1073.292343	0.60433591	0.132575643	1.36E-06	4.39E-05
**HYOU1**	3824.40752	0.604265999	0.176229223	0.00015690	0.002993174
**ID1**	408.9202517	0.603930138	0.2177955	0.00136270	0.018277682
**GDF15**	782.554018	0.603394398	0.210309266	0.00101065	0.014552385
**MAP2**	1032.430943	0.603173351	0.146888838	1.06E-05	0.000280055
**CCDC18-AS1**	237.6566875	0.601413049	0.256264965	0.00407403	0.044114968
**UBA6**	4920.210617	0.600766406	0.222444879	0.00165361	0.021508865
**RAPGEF2**	706.8972998	0.599972572	0.152269201	2.14E-05	0.000526617
**PSMA1**	510.7463797	0.598894723	0.158196099	4.06E-05	0.00093467
**GALT**	90.09024944	0.597690103	0.257745223	0.00469556	0.049168275
**N4BP2L2**	3649.460553	0.597317539	0.117010692	9.08E-08	3.64E-06
**NUP50-AS1**	242.3406655	0.596944137	0.21858325	0.00158739	0.020794852
**RCAN1**	326.9860908	0.596803095	0.216893483	0.00148717	0.019646875
**DCLRE1C**	564.7799523	0.596469498	0.160358284	5.43E-05	0.001209131
**KLHL21**	973.2317121	0.596426509	0.16818072	0.00010534	0.002106158
**TGM2**	17749.30727	0.595452193	0.167414069	0.00010059	0.002035101
**USO1**	2739.813237	0.593210335	0.129400849	1.25E-06	4.07E-05
**TVP23C**	117.4093797	0.592776576	0.245448803	0.00380566	0.041813221
**PPIB**	2519.027352	0.592154752	0.12948191	1.37E-06	4.41E-05
**STARD4**	890.8855673	0.588413885	0.155336525	4.08E-05	0.000938693

**ZNF224**	345.3172633	0.587634196	0.173822908	0.0001956	0.003625704
**CTSC**	1281.971603	0.587151697	0.177833395	0.0002594	0.004610546
**MAGEA4**	985.4817045	0.585697294	0.123688147	6.32E-07	2.16E-05
**SPART**	995.7121652	0.58509571	0.124615331	7.72E-07	2.59E-05
**ALOX12-AS1**	133.9537282	0.5830771	0.237648573	0.0033445	0.037765906
**LMO7**	20893.56058	0.582627649	0.116341475	1.64E-07	6.27E-06
**TMEM106B**	1725.950061	0.581775357	0.135781574	5.25E-06	0.000148597
**ME3**	130.945974	0.581622229	0.243993312	0.0042777	0.045731286
**AASDH**	163.1943148	0.581347955	0.217572786	0.0018679	0.02370779
**RAB27B**	5404.640825	0.581213362	0.196073101	0.0008010	0.012180173
**CGAS**	459.3665346	0.580861824	0.247202529	0.0044667	0.047274813
**BCL6**	278.4761054	0.578198717	0.236700648	0.0034603	0.038862912
**GLA**	230.8939767	0.577278292	0.215444945	0.0018799	0.023843848
**ZNF320**	413.4637495	0.577050983	0.175241464	0.0002699	0.004775241
**JADE2**	1393.471759	0.576806141	0.129855824	2.65E-06	8.09E-05
**RAB2B**	294.6080348	0.576018412	0.218294213	0.0020924	0.026010855
**GRAMD2B**	3000.243345	0.575205101	0.160167815	9.35E-05	0.001927113
**FOXC1**	1044.060246	0.573099535	0.147058512	2.87E-05	0.000684251
**TPT1**	16474.99024	0.569940166	0.144089458	2.23E-05	0.000547695
**DNAJA1**	5522.797522	0.568489613	0.125764024	1.86E-06	5.89E-05
**KDSR**	1749.490788	0.564419322	0.117856559	5.08E-07	1.76E-05
**LYN**	819.3121978	0.561766739	0.187844938	0.0007720	0.011797299
**MUC20-OT1**	519.2273694	0.561057178	0.168814579	0.0002498	0.004462251
**ERP44**	1522.442228	0.560681187	0.140481006	2.14E-05	0.000526944
**INAVA**	242.4725847	0.55934321	0.196442109	0.0012529	0.017169508
**TEP1**	824.1279407	0.556566691	0.169530844	0.0002936	0.005145473
**USP42**	567.5382758	0.556244296	0.153080588	8.62E-05	0.001792902
**ENSG00000287158**	213.2235279	0.555585001	0.206831792	0.0020129	0.025180229
**VCPIP1**	1918.934959	0.554021061	0.123157984	2.15E-06	6.69E-05
**EHF**	3092.239399	0.552171847	0.217233298	0.0030273	0.03481327
**UBR1**	1278.519	0.551097304	0.144778997	4.33E-05	0.000988744
**TYK2**	612.1917699	0.550672863	0.148540817	6.47E-05	0.001394114

**CLCN3P1**	185.2094106	0.54970902	0.232708281	0.0047459	0.04961223
**CLDN1**	3941.441012	0.547601723	0.153440385	0.0001112	0.002214618
**TNFRSF10B**	1468.683488	0.547051937	0.141838866	3.58E-05	0.000833901
**DOCK10**	1212.645928	0.546084559	0.158340754	0.0001704	0.003220554
**TXNL4B**	226.846543	0.545975362	0.185244767	0.0009385	0.013768876
**STAP2**	476.9753815	0.545131833	0.166350888	0.0003198	0.005543345
**RNF115**	788.9659544	0.544033886	0.130978438	1.3E-05	0.000335699
**TMEM179B**	200.4148998	0.543558402	0.206893693	0.0024628	0.029688885
**AL138724.1**	145.3252432	0.543355125	0.225385447	0.0044806	0.04736809
**NECAP2**	911.7416545	0.543046944	0.143151146	4.73E-05	0.001069409
**ANKFY1**	1899.400678	0.542027355	0.117572238	1.31E-06	4.25E-05
**LYST**	2404.519661	0.54185737	0.128090936	7.63E-06	0.000209614
**SPG11**	2012.354225	0.538675387	0.11817248	1.67E-06	5.31E-05
**UPP1**	1634.375434	0.538320252	0.200738328	0.0021561	0.026660711
**TMEM50B**	341.4621019	0.536659023	0.175172873	0.0006849	0.010674499
**PTER**	664.9372974	0.535811559	0.142841888	5.68E-05	0.001259806
**PSMA3**	1404.720014	0.535368358	0.123275236	4.65E-06	0.000134063
**FTL**	1468.717591	0.534387028	0.186740387	0.0012865	0.017514823
**BCL2L11**	592.4698648	0.533851962	0.17842341	0.0008390	0.012624606
**ADGRF1**	3562.664645	0.531897149	0.182937558	0.0011175	0.015728422
**IRF3**	610.054716	0.530409162	0.144413117	7.82E-05	0.001642105
**FOXP1**	687.5527882	0.530318831	0.20668062	0.0030098	0.034654646
**GULP1**	1049.191631	0.530210188	0.139244449	4.63E-05	0.001048231
**MIER1**	1746.212015	0.529829063	0.119837838	3.26E-06	9.83E-05
**GUK1**	1228.433785	0.529589947	0.135218283	2.96E-05	0.000703851
**TMEM170A**	504.2593727	0.529033848	0.148086026	0.0001125	0.002236671
**PCMTD1**	948.1694484	0.528686861	0.210469762	0.0034860	0.039104102
**PTPN12**	2868.844858	0.524863392	0.110471829	6.96E-07	2.36E-05
**F2RL1**	1727.541761	0.524357099	0.191665821	0.0019665	0.024707848
**SEC24A**	1201.171233	0.522282843	0.158873282	0.0003303	0.005675032
**ADPRHL2**	379.3089397	0.521295515	0.155385135	0.0002567	0.004567595
**RALA**	942.1849361	0.520785402	0.166150255	0.0005461	0.008767595

**SLC35F6**	1570.770553	0.518466402	0.202937174	0.00333757	0.037716137
**LSR**	3087.932867	0.517678856	0.150576915	0.00019795	0.003660782
**DNAJC1**	387.5234342	0.517117418	0.191631161	0.00218106	0.026844827
**BET1**	320.830654	0.516757592	0.212405105	0.00447631	0.04734883
**EPPK1**	2759.984205	0.516561083	0.150805543	0.00020660	0.003792618
**DAPP1**	1393.924808	0.515497525	0.1898578	0.00210090	0.02608099
**ZFYVE26**	1032.25741	0.51510773	0.145568839	0.00013706	0.002660707
**PAGE2**	2349.50832	0.51366298	0.14487718	0.0001333	0.002595741
**EFL1**	599.9334094	0.512441004	0.137865523	7E-05	0.001484751
**LINC00431**	297.6901929	0.511318962	0.20340991	0.00378637	0.04165012
**ANXA4**	755.3779741	0.510837507	0.154933129	0.00032482	0.005603055
**PARP4**	2192.351064	0.510716864	0.14060054	9.65E-05	0.001977961
**USP15**	1089.756747	0.510358282	0.131676101	3.72E-05	0.000862468
**AL390728.4**	598.9459457	0.509573299	0.19935855	0.00328746	0.037323628
**MTMR11**	441.9898261	0.509205439	0.192978641	0.00258725	0.030792326
**GRIPAP1**	861.6819901	0.508960508	0.135836272	6.25E-05	0.001359367
**ZNF277**	451.7797183	0.508540206	0.150549542	0.00024842	0.004440873
**RARS**	2576.087082	0.508535752	0.110183847	1.42E-06	4.58E-05
**MCUB**	747.8118115	0.507734391	0.166117467	0.00073829	0.011355116
**CTSL**	4617.140478	0.507476295	0.139715595	9.99E-05	0.002025505
**POMP**	2453.475697	0.505270837	0.112103039	2.41E-06	7.42E-05
**PHLDB2**	12453.99302	0.503411201	0.114617874	4.09E-06	0.000119218
**WDR45**	316.4847193	0.501776804	0.176840256	0.00156957	0.020587536
**E2F7**	1732.008702	0.501713172	0.141356109	0.00013667	0.002655906
**ENO1**	30272.75564	0.501712177	0.137005636	9.08E-05	0.00187715
**HSBP1L1**	171.9837776	0.50017595	0.197782869	0.00369906	0.040881809
**SMURF1**	1423.9902	0.499187072	0.152804718	0.00038587	0.006532265
**MYD88**	958.2134949	0.499032486	0.151584928	0.00035456	0.006043708
**FAM111A**	3798.095024	0.496660893	0.118818121	1.08E-05	0.000282924
**SCO1**	808.3859921	0.496369536	0.168761357	0.00114118	0.015951567
**PDCD10**	1049.439171	0.49466381	0.149912151	0.00034239	0.005846936
**GMDS-AS1**	362.4212756	0.49439203	0.185464799	0.00254580	0.030382822

**LITAF**	4641.661361	0.494177901	0.111779422	3.72E-06	0.000110377
**IRF6**	9094.170572	0.494148402	0.184699677	0.00254099	0.030382822
**FBXL5**	855.1787788	0.493185623	0.142958789	0.00020670	0.003792618
**GCLM**	639.2622815	0.491667554	0.162842161	0.00091578	0.013550806
**BCL10**	1167.515612	0.488692159	0.146898955	0.00031737	0.005510043
**MIS12**	573.7920075	0.488651743	0.154186451	0.00054052	0.008698952
**N4BP1**	991.9465795	0.486222318	0.140348448	0.00019511	0.003618847
**GALNT3**	1184.409861	0.482591896	0.158437356	0.00087037	0.01298142
**CLIP1**	3243.403748	0.482234437	0.118447294	1.82E-05	0.000454053
**DNAJB11**	750.1051354	0.481910694	0.182592928	0.00300966	0.034654646
**CDKN1A**	1995.872359	0.480939097	0.188110837	0.00383911	0.042131253
**EDEM1**	2149.202938	0.479132532	0.140602737	0.00024782	0.004438368
**SNX6**	944.9145666	0.478536253	0.12888791	7.79E-05	0.001638666
**ADGRE5**	1474.579881	0.47766545	0.132931023	0.00014936	0.002878693
**CLIP4**	4638.650602	0.476949995	0.147835427	0.00047499	0.007853487
**ZNF672**	409.949983	0.476013538	0.160375086	0.00108870	0.015450569
**TNIP1**	4439.272301	0.474858702	0.173996704	0.00231765	0.028229329
**LRIF1**	1145.326755	0.473750525	0.133914497	0.00016137	0.003066015
**RIOK3**	1945.159831	0.471932628	0.15604713	0.00098021	0.014223401
**LMF2**	292.4395013	0.471788422	0.188707671	0.00448431	0.047380006
**ITM2B**	2112.275649	0.471461278	0.130042021	0.00011333	0.002249012
**PUS3**	269.0211874	0.470665058	0.178832985	0.00304615	0.034986915
**CHD2**	1912.793825	0.467829668	0.179109	0.00330432	0.037469685
**GRB7**	777.1626988	0.464037414	0.131570375	0.00017007	0.003218271
**NFAT5**	9200.070987	0.463527791	0.172834507	0.00281763	0.032986087
**TMX1**	902.1164625	0.463014815	0.141658642	0.00042376	0.007094114
**TNFRSF10A**	715.1210756	0.462237453	0.17767127	0.00349982	0.039225878
**FAM193B**	383.7633686	0.461183008	0.165041739	0.00197459	0.024792273
**AGPAT3**	1124.614974	0.460899932	0.143173072	0.00051840	0.008437196
**VAMP8**	861.9467385	0.459163649	0.129993255	0.00017054	0.003220554
**MOSPD2**	650.5333425	0.457986112	0.144083725	0.00059374	0.009386211
**RAB27A**	458.5142746	0.45739796	0.181151395	0.00429797	0.045876874

**PPP1R18**	6462.318508	0.457128544	0.164210491	0.00216687	0.02672519
**CTTNBP2NL**	1504.728709	0.456757834	0.123060211	8.49E-05	0.001768501
**TUBB6**	569.0771613	0.454970311	0.153671272	0.0012077	0.016696561
**CMTR1**	801.1961185	0.454594888	0.137179506	0.00037497	0.00636847
**TMEM62**	492.4665339	0.45428493	0.156640826	0.00146964	0.019442731
**EGFR**	15839.03804	0.453397495	0.128426266	0.00017611	0.003315791
**PHC3**	2286.255114	0.451858026	0.120233198	7.2E-05	0.001518975
**RP2**	472.8749611	0.451619952	0.15290114	0.00125970	0.017250038
**SHISA5**	1141.275263	0.450976476	0.129515571	0.00020727	0.003797739
**RCN1**	1140.650546	0.45013198	0.140458748	0.00056210	0.008984551
**DCP1A**	397.2828885	0.449391776	0.156332801	0.00166962	0.021630124
**MFSD12**	1179.930784	0.449018686	0.155537764	0.00159160	0.020835486
**TBK1**	995.2565538	0.447478137	0.142479492	0.00070624	0.010951942
**TPT1-AS1**	528.1035241	0.447095019	0.166640998	0.00292262	0.033912166
**FOXK1**	2531.755433	0.445928981	0.175454269	0.00441019	0.04688773
**FZD5**	296.0378217	0.444589113	0.173985572	0.00427945	0.045731286
**SPRY2**	827.4873724	0.444089949	0.173075879	0.00415922	0.044855974
**COG3**	601.1238512	0.444016955	0.15919984	0.00217314	0.02678258
**MPZL2**	8316.196695	0.443729764	0.144185707	0.00087496	0.013029131
**NAPB**	467.3742999	0.442437401	0.152519289	0.00152468	0.020071631
**TMEM50A**	1317.72925	0.441902247	0.119955671	9.81E-05	0.002000587
**MMP28**	699.6118352	0.440510845	0.1362219	0.00052467	0.008512133
**TOP 1.00**	2445.822489	0.435123292	0.155064268	0.00213467	0.026412688
**DMTF1**	1587.226863	0.434692339	0.151692408	0.00173691	0.022316576
**OSGIN2**	933.9480007	0.434145772	0.160387284	0.00286494	0.033376201
**DNAJC3**	4881.23071	0.434030138	0.167958804	0.00411176	0.044420768
**OMA1**	535.9706905	0.433962508	0.144967746	0.00117155	0.016287223
**DENND5A**	810.7103164	0.433290343	0.148680395	0.00151158	0.019941208
**OTUD1**	529.8340583	0.430153291	0.142658351	0.00112292	0.015768917
**LY6E**	2012.739115	0.429131013	0.15750054	0.00276422	0.032506138
**TIA1**	954.6124576	0.428896282	0.132327909	0.00052637	0.008529978
**BAZ2A**	2178.401188	0.428596516	0.119408401	0.00014991	0.00288627

**XIAP**	1592.183865	0.42685289	0.117686446	0.00012930	0.002520633
**ELK3**	1533.602801	0.426534011	0.119687346	0.00016397	0.003112236
**BBS10**	441.8562288	0.426302595	0.154917803	0.00257775	0.030698693
**SLC50A1**	691.6152779	0.426297252	0.145371521	0.00145216	0.019252277
**TPP1**	1080.953381	0.424327675	0.142723923	0.00130939	0.017774584
**HERPUD2**	575.716408	0.42359246	0.163481892	0.00407340	0.044114968
**FKBP15**	1512.075746	0.422385925	0.143836295	0.00146704	0.019422061
**PRR3**	475.1835454	0.421554356	0.157598331	0.00325804	0.037079585
**CDKN2A**	666.8636032	0.421142921	0.145194787	0.00165452	0.021508865
**NFKB1**	1137.034689	0.419396217	0.150841085	0.00245835	0.029653767
**UHMK1**	5857.262005	0.418897341	0.105359506	3.13E-05	0.000739734
**STX17**	1673.034379	0.418615845	0.128778299	0.00052482	0.008512133
**DCBLD2**	9441.291728	0.418448081	0.110030714	6.75E-05	0.001440439
**ZNF75A**	334.6668084	0.416736149	0.160432837	0.00416296	0.044870404
**KDM6A**	1139.695657	0.415833161	0.14009273	0.00135343	0.018211756
**ALS2CL**	927.1828809	0.415404347	0.161759926	0.00449338	0.047422276
**ARAP2**	1782.505299	0.413720186	0.12040375	0.00027747	0.004894428
**AKR1A1**	608.5272907	0.409642918	0.142244145	0.00182618	0.023240431
**ZFPM2-AS1**	348.6684651	0.408081331	0.156411483	0.00419214	0.045107092
**MYDGF**	858.1199802	0.407090175	0.142466743	0.00200248	0.025091973
**SEC24D**	1361.34735	0.406633938	0.128954895	0.00084484	0.012681418
**SHISA2**	2067.952834	0.405166865	0.135481636	0.00131251	0.017803977
**PTPRK**	3898.098932	0.403562538	0.131370312	0.00084937	0.012711839
**TMED7**	2095.509968	0.403165611	0.140280413	0.0019231	0.024226927
**KCNK1**	3183.374107	0.398827049	0.131762669	0.00119582	0.01656913
**CSNK1G1**	407.1093921	0.398428388	0.149432277	0.00359833	0.040052097
**UEVLD**	596.0919398	0.396705088	0.145262602	0.00299730	0.03455291
**TMEM87B**	788.2141293	0.394381335	0.152644894	0.00460938	0.048476729
**AARS**	4432.784749	0.392889818	0.137082845	0.00201490	0.025180229
**UGCG**	3642.890048	0.392827812	0.129442581	0.00118850	0.016492018
**CCDC82**	852.7275631	0.392579286	0.148464204	0.00397422	0.043209003
**AC239868.1**	508.7507629	0.390718569	0.145064183	0.00341397	0.038457052

**TXN**	5674.85155	0.388704742	0.118743475	0.00054155	0.008708173
**RNF169**	1586.042058	0.385440059	0.133550217	0.00195778	0.02461419
**PATL1**	1760.347161	0.385094593	0.134729612	0.00213037	0.026376899
**CARHSP1**	2296.832022	0.384296486	0.144299757	0.0038548	0.042253932
**AVL9**	1172.385551	0.382846272	0.1204593	0.00075651	0.011587786
**GOSR2**	825.5349821	0.381365426	0.142521247	0.00376369	0.041473705
**MYL12A**	3533.770915	0.377399389	0.119535517	0.00082058	0.01241674
**NT5C2**	2390.004354	0.377036046	0.138174178	0.00326812	0.037126589
**DDX23**	1733.5797	0.375319079	0.114233948	0.00053730	0.008669566
**NT5E**	5764.833022	0.373553642	0.130900805	0.00225479	0.027535288
**CANX**	27260.87765	0.373531552	0.098733871	7.99E-05	0.001672244
**RIPK1**	1098.726821	0.373433261	0.133688052	0.00268837	0.031753647
**FCHO2**	1547.293378	0.373424903	0.140422117	0.00403323	0.043749079
**IBTK**	1871.210552	0.37213146	0.118773293	0.00092587	0.013674982
**LSM 12.00**	607.9103679	0.371705466	0.139967249	0.00410218	0.044368461
**DIS3**	2501.452981	0.370135789	0.120742499	0.00113416	0.015879218
**ASCC3**	4188.555584	0.369391964	0.107502339	0.00031950	0.00554189
**ILK**	1260.30805	0.366526638	0.131416272	0.00281485	0.032986087
**NFE2L2**	1649.827722	0.360288746	0.123350157	0.00223984	0.027388428
**LEMD3**	784.947582	0.35658174	0.127317139	0.00281497	0.032986087
**COPG1**	2699.80703	0.353591964	0.116380267	0.00133606	0.01804489
**AIDA**	1247.891562	0.353189747	0.118135641	0.00156667	0.020566615
**HERC4**	4451.316139	0.35213566	0.132567288	0.00441777	0.04692668
**EFR3A**	1191.11661	0.351901935	0.12809886	0.00336806	0.038008375
**IDI1**	1282.351182	0.347926926	0.12935456	0.00373109	0.041162897
**KRR1**	2152.688818	0.346632359	0.108629101	0.00081348	0.012326199
**MED13**	4993.703653	0.342385696	0.125025009	0.00356363	0.039784011
**IGF2BP2**	2564.84431	0.3398801	0.114571009	0.00176597	0.022581592
**ACBD3**	1637.369055	0.339009638	0.12156881	0.00306918	0.035186695
**TNKS2**	1683.611531	0.334592239	0.122157072	0.00362583	0.04026243
**ECPAS**	5575.989897	0.329232685	0.112024285	0.00201443	0.025180229
**BIRC2**	2630.469282	0.329164033	0.119451294	0.00350107	0.039225878

**DYNC1H1**	8345.787965	0.325918199	0.101932508	0.000832355	0.012554224
**TAX1BP1**	8522.49471	0.325593193	0.101168249	0.000792655	0.012081971
**OGT**	4746.345391	0.320195987	0.108165412	0.00191063	0.024134747
**TRAM1**	3777.997525	0.306742473	0.111994686	0.00394188	0.042945732
**NCOA7**	4183.746982	0.305914775	0.107623025	0.00289944	0.033736216
**AFF4**	5371.021087	0.298253185	0.100758885	0.00191371	0.024157454
**CXCL17**	6.882862853	0.252851844	0.50863114	0.00329774	0.037417748
**HIPK1**	2253.687898	-0.301494693	0.108685847	0.00332000	0.037579214
**PTEN**	1789.534423	-0.313220527	0.114572537	0.00388778	0.042540774
**ARHGEF12**	4404.660115	-0.32178516	0.112660731	0.00262014	0.031144128
**ICMT**	2148.035216	-0.323711062	0.11332542	0.00265661	0.031477754
**TRIO**	5753.47929	-0.325960867	0.113787274	0.00250293	0.030079341
**FAF1**	1668.969864	-0.326901094	0.112740171	0.00243208	0.0294127
**CGA**	6027.424484	-0.327531285	0.11897423	0.00352685	0.039420388
**LMNA**	6079.934283	-0.328861772	0.123148356	0.00464070	0.048702734
**FAM20B**	2019.901936	-0.33108474	0.113419946	0.00209601	0.026037534
**ITSN1**	1710.607788	-0.335340361	0.113222226	0.00170825	0.022069409
**QSER1**	3586.422491	-0.336316328	0.10538878	0.00082443	0.012459807
**TUBG1**	1021.374128	-0.341007272	0.128068981	0.00439907	0.046803161
**DOCK5**	6957.307471	-0.343586313	0.116542419	0.00169047	0.021869994
**CBX5**	7369.707663	-0.344304948	0.118071112	0.00202691	0.025279728
**DIAPH3**	2252.029395	-0.346644126	0.112314068	0.00110662	0.015612305
**LSM 4.00**	1199.037555	-0.347523076	0.124306212	0.00292628	0.033922027
**CHML**	4695.563433	-0.349611157	0.104889412	0.00048775	0.00803614
**RAP1GAP2**	1510.694577	-0.350449285	0.130442063	0.00400442	0.043512114
**C12orf75**	1158.875203	-0.350454	0.130126708	0.00393834	0.042945732
**CHP1**	1064.68956	-0.350571546	0.131670908	0.00427208	0.045704702
**DOT1L**	752.844384	-0.351436991	0.134201421	0.00464984	0.048734201
**RRM1**	2681.869546	-0.351626497	0.110833893	0.00084839	0.012711839
**FAM199X**	1110.600541	-0.352830007	0.132624652	0.00426789	0.045685944
**RNPEP**	1053.689497	-0.356131152	0.132114557	0.00385014	0.042227588
**DST**	36260.50927	-0.356739766	0.113680347	0.00093982	0.013776013

**JUP**	5966.71272	-0.360783049	0.117209715	0.00112741	0.015820174
**APPL1**	1041.762489	-0.36090317	0.131289315	0.00320998	0.036621581
**PACSIN2**	974.5868525	-0.36292169	0.132041075	0.00321393	0.036622082
**BRI3BP**	2062.373172	-0.364343288	0.115329744	0.00085343	0.012749074
**SBNO1**	2377.489674	-0.365296035	0.11113463	0.0004932	0.008104485
**CREB3L2**	1305.371484	-0.36582776	0.116918303	0.00076226	0.011656734
**IMPDH2**	1558.306531	-0.36671816	0.114772488	0.00075480	0.011570992
**MID1**	2059.512234	-0.368522363	0.117289624	0.00089010	0.013223044
**POLD2**	1057.309654	-0.368603951	0.1323461	0.00282002	0.032986087
**GLG1**	3336.95886	-0.368706367	0.113902957	0.00064457	0.010079133
**TPD52**	1488.619163	-0.369519309	0.128725849	0.00211027	0.02614541
**PBK**	932.7267021	-0.370241825	0.13630158	0.00346395	0.038879691
**ITGA6**	12726.10474	-0.370628091	0.125742184	0.00162707	0.021225635
**KIF1C**	2309.354173	-0.370936891	0.123585412	0.00142230	0.018907017
**APLP2**	6122.881963	-0.372405266	0.102073329	0.00015176	0.002912849
**LEPROTL1**	1016.733755	-0.373446908	0.125576119	0.00153251	0.020160536
**EXT2**	984.2783714	-0.373889742	0.135650043	0.0030214	0.034766515
**RACGAP1**	4606.570013	-0.373918965	0.13387304	0.00264604	0.031372376
**CDK6**	2593.223163	-0.374966791	0.12808676	0.00176825	0.022595289
**TMTC3**	1124.910128	-0.377991443	0.1298214	0.00179621	0.022905653
**AP2M1**	1377.949353	-0.37800286	0.122774773	0.00107616	0.015307415
**OPHN1**	1378.199561	-0.379133959	0.123603417	0.00109917	0.015552052
**NIN**	1747.235775	-0.382886026	0.116232398	0.00050774	0.008299874
**CLSTN1**	3097.263567	-0.383242444	0.131202185	0.00174547	0.022411248
**RRP1B**	1075.218701	-0.386402186	0.147996578	0.00446366	0.047274813
**KNL1**	2368.66371	-0.387551554	0.136772001	0.00227193	0.027726488
**OLFML3**	10120.20932	-0.387842457	0.128625876	0.00121160	0.016738201
**CDK16**	1323.176026	-0.38936489	0.119729888	0.00056999	0.009079671
**PRELID1**	1441.676779	-0.39035249	0.125406568	0.00092933	0.013707985
**GNG12**	3257.643518	-0.390453065	0.116383062	0.00039681	0.006690821
**KIAA1958**	599.3989298	-0.390455121	0.143957446	0.0032371	0.036863587
**AHCY**	2100.898172	-0.395255596	0.144441123	0.00298198	0.034439932

**FKBP4**	1204.071091	-0.398381661	0.154123698	0.00451582	0.047632269
**APMAP**	1169.645093	-0.39886732	0.147783402	0.00331882	0.037579214
**ACAT1**	652.4345402	-0.399563042	0.142518908	0.00237048	0.028797872
**SLC3A2**	4224.626167	-0.400239721	0.149024872	0.00333813	0.037716137
**PLXNB1**	557.3307761	-0.402454707	0.14613372	0.00272767	0.032116605
**PPP1R14C**	2085.77303	-0.405595884	0.156805397	0.00441886	0.04692668
**SNX12**	431.4655963	-0.406013674	0.155656859	0.00421701	0.045334268
**CIC**	588.402793	-0.407231704	0.149352587	0.00293944	0.034053599
**UBE4B**	1893.353863	-0.407834239	0.123706184	0.00047900	0.007905878
**PLXNB2**	1471.239907	-0.408410806	0.126073304	0.00053000	0.008573852
**AL161431.1**	5958.521611	-0.40895495	0.154890909	0.00380142	0.041791092
**LSS**	426.5806554	-0.40974529	0.155158154	0.00366656	0.040666535
**ZDHHC3**	1149.068417	-0.411390913	0.137587292	0.00129912	0.017660757
**PRKACA**	639.4159257	-0.411668431	0.149559534	0.00268659	0.031753647
**ANKRD50**	2281.195946	-0.412213851	0.130072909	0.00070106	0.010880658
**KDM5B**	3390.384989	-0.412541818	0.160729801	0.00452109	0.047661084
**TMX4**	415.1575079	-0.415788234	0.150895986	0.00259176	0.030826347
**KIF4A**	1659.067737	-0.416610697	0.142756659	0.00159693	0.020882204
**TCTN3**	437.5135406	-0.417044197	0.160825297	0.00426596	0.045685944
**CEP78**	2162.413571	-0.418438128	0.12396712	0.00033588	0.005751538
**SDC3**	639.9448437	-0.41853255	0.136574492	0.00094311	0.013785631
**SERTAD4**	1211.530963	-0.419247751	0.129251607	0.00052908	0.008566472
**MDC 1.00**	4345.109856	-0.422175882	0.130298338	0.00054609	0.008767595
**NDRG3**	694.5465252	-0.422566692	0.137190712	0.00087474	0.013029131
**MAPKAPK3**	669.7754672	-0.422749795	0.143799427	0.00145403	0.019263366
**SIGMAR1**	975.8034251	-0.423668403	0.142675155	0.00133078	0.017986749
**PDZD8**	1007.091244	-0.423878581	0.161071114	0.00371144	0.040970277
**ZFYVE9**	464.457671	-0.424341376	0.154293878	0.00254750	0.030382822
**ZNF185**	1562.804485	-0.425001224	0.138378371	0.00093209	0.013716739
**MIR205HG**	2815.785583	-0.425459405	0.163147769	0.00388381	0.042522208
**DESI2**	1534.572789	-0.425590765	0.120290295	0.00018012	0.003381018
**ANKS6**	729.1392255	-0.427838477	0.132442584	0.00052043	0.008462892

**TTL**	730.9869028	-0.428836625	0.146030064	0.00144973	0.019233579
**SLC7A6**	1229.550367	-0.428864467	0.163426692	0.00368474	0.040795735
**PLEKHG3**	681.5490232	-0.429690753	0.15101205	0.00193265	0.024330937
**UQCRC1**	943.3138313	-0.431553229	0.137182766	0.00072359	0.011184116
**LIMS1**	1214.330669	-0.43181244	0.149314757	0.00163901	0.021354008
**MBOAT2**	3569.579642	-0.431907014	0.137230322	0.00070953	0.010975875
**HMCES**	724.0594404	-0.432839578	0.163962553	0.00345776	0.038856857
**TSPAN3**	550.0443833	-0.434104192	0.143646551	0.00107019	0.015253755
**RABEP1**	1268.381842	-0.434900773	0.134799602	0.00053919	0.008685017
**RFC3**	650.9875827	-0.435868012	0.15074206	0.00163919	0.021354008
**PHF19**	883.2270512	-0.437361699	0.129624012	0.00032173	0.005568574
**ATAD2B**	554.6259097	-0.437553441	0.14751306	0.00127901	0.017437957
**PGM2**	829.9271499	-0.437786593	0.130272636	0.00033428	0.005729393
**KIAA1549L**	1254.819257	-0.438610596	0.127378447	0.00024596	0.004409174
**FSTL1**	1610.16347	-0.438950219	0.14348711	0.00094140	0.013777608
**CDS1**	1066.348775	-0.439332335	0.145325657	0.00103658	0.014857151
**C6orf132**	1003.375136	-0.439779632	0.173608128	0.00464881	0.048734201
**PSD4**	1043.876138	-0.439937959	0.135802611	0.00051423	0.008383869
**RAP1GDS1**	523.6392466	-0.440690408	0.17260859	0.00439974	0.046803161
**REEP5**	910.2252314	-0.440710469	0.13775773	0.00058386	0.00925348
**PINK1**	1434.83952	-0.441803714	0.169324939	0.00359521	0.040052097
**OXTR**	1121.054449	-0.442832671	0.160775893	0.00239490	0.02901923
**RUVBL2**	621.0532257	-0.443052483	0.162310773	0.00262599	0.031176859
**EXTL3**	1086.465151	-0.443157481	0.138820048	0.00059752	0.009430009
**SCRIB**	911.1610027	-0.444124247	0.163725048	0.00273721	0.032208648
**MARVELD2**	1092.751278	-0.444149777	0.138750913	0.00058069	0.009210987
**SGO2**	972.1707456	-0.4452821	0.173553629	0.00411082	0.044420768
**REXO2**	447.4743899	-0.445556697	0.164331765	0.00272509	0.032106447
**ACSL1**	863.417206	-0.445864167	0.129318983	0.00023555	0.004251176
**PPP2R5D**	656.4224789	-0.445931073	0.138446955	0.00050945	0.008320447
**PTPA**	1104.992318	-0.446921653	0.129834521	0.00024401	0.004378437
**CELSR1**	963.4486503	-0.447722976	0.176126705	0.00421829	0.045334268

**LAPTM4B**	1250.568451	-0.449845686	0.143634106	0.00069955	0.010866279
**TTLL12**	451.5121852	-0.449954789	0.175174443	0.00401181	0.043560051
**GJB2**	1732.386007	-0.450812097	0.116903558	4.87E-05	0.001099436
**RCC2**	4578.991415	-0.451315359	0.131073933	0.00023294	0.004212168
**ISOC1**	333.5216501	-0.451805349	0.177601471	0.00424883	0.045533999
**COLGALT1**	1351.741424	-0.452015417	0.138972731	0.00046839	0.007758088
**C7orf50**	368.6897142	-0.453611772	0.166447951	0.00254798	0.030382822
**RIPK4**	343.3644116	-0.45366362	0.161894329	0.00201853	0.02519205
**VSIR**	568.4000601	-0.454063091	0.157806224	0.00159740	0.020882204
**PCBD1**	347.7857919	-0.454105483	0.173684726	0.00342442	0.038528328
**PTTG1**	1248.761065	-0.454385473	0.165730019	0.00239852	0.029044374
**PPDPF**	721.956896	-0.454886507	0.157201046	0.00151640	0.019990736
**NHP2**	773.560685	-0.45647235	0.154322267	0.00121976	0.016826038
**KAT6B**	851.3139984	-0.458115264	0.171397722	0.00284089	0.033157884
**EID1**	912.017132	-0.458555477	0.129095633	0.00015417	0.002953185
**ITGAV**	2197.095795	-0.459800953	0.131852868	0.00019239	0.003586238
**DLG5**	2784.851752	-0.460444091	0.133772251	0.00023195	0.004202236
**STIM1**	418.6665422	-0.461043862	0.173063605	0.00299439	0.034540682
**POLR2L**	375.6571381	-0.461182756	0.163043137	0.00181261	0.023083425
**R3HDM1**	615.7265621	-0.46128594	0.153547601	0.00105173	0.015016811
**POLDIP2**	864.8528946	-0.463893126	0.148462324	0.00070740	0.010951942
**TMEM64**	432.2347823	-0.464465296	0.163014396	0.00167515	0.02168666
**CDK14**	539.2232519	-0.46447476	0.168509313	0.00216706	0.02672519
**GINS2**	511.8748769	-0.464684209	0.146837047	0.00060208	0.00948597
**GLRX5**	324.3291003	-0.4671345	0.187377306	0.00461140	0.048476729
**NT5C3B**	272.6694774	-0.468665942	0.18107665	0.00358504	0.039999218
**UHRF1BP1L**	399.3952737	-0.470375556	0.157852075	0.00107076	0.015253755
**EIF2B3**	536.6762995	-0.473435852	0.166734023	0.00169853	0.021958998
**PLS1**	536.0514824	-0.473844551	0.167843098	0.00175389	0.02247317
**SYT16**	1554.132922	-0.473890068	0.185354677	0.00378052	0.04161023
**TRPS1**	2006.21047	-0.474240623	0.173109766	0.00223965	0.027388428
**FUT9**	1063.908418	-0.475036181	0.144299354	0.00037450	0.006366169

**TSPAN13**	1366.549745	-0.475484789	0.155768931	0.00084149	0.01265153
**PTDSS1**	1004.608225	-0.475831292	0.163420792	0.00131723	0.017855169
**NTSR1**	1328.843825	-0.476161548	0.177708684	0.00267421	0.03166631
**C1QTNF6**	1267.6548	-0.476443225	0.150471367	0.00057412	0.009129889
**CENPL**	665.8994633	-0.477264759	0.168362496	0.00166957	0.021630124
**ETV4**	331.929823	-0.477279426	0.17216212	0.00205494	0.025578278
**TRIM24**	580.4002475	-0.477703789	0.148169007	0.00047193	0.007809818
**CUX1**	1383.548952	-0.477972032	0.155964583	0.00081392	0.012326199
**LUZP2**	343.4297839	-0.478143431	0.191587049	0.00452921	0.047719807
**CDC42EP3**	1690.23188	-0.478383898	0.177426223	0.00252176	0.030220311
**CLTB**	1078.03323	-0.479195748	0.150596509	0.00057216	0.009106543
**MISP**	846.0860093	-0.479312448	0.192439382	0.00448880	0.04740062
**CEBPZOS**	704.81025	-0.47952662	0.15886256	0.00093781	0.013768876
**TPX2**	5857.758196	-0.480891264	0.185286896	0.00326436	0.037126589
**P3H2**	1751.035941	-0.48110416	0.155658404	0.00073399	0.011307468
**IFT172**	316.8220996	-0.481267084	0.175758242	0.00220288	0.027024705
**KLF9**	559.8994545	-0.48162611	0.174168182	0.00198161	0.02486379
**KRT7**	7271.415458	-0.482178511	0.171182974	0.00172328	0.022195939
**ANKRD13B**	213.1919971	-0.482384427	0.187844669	0.00359795	0.040052097
**AFAP1L1**	714.9340435	-0.48262768	0.161250623	0.00100518	0.014484733
**UXS1**	661.3879254	-0.482923874	0.160503348	0.00091319	0.013531205
**LYPD3**	405.2102123	-0.483574345	0.189339703	0.00368299	0.040795735
**S100A16**	2115.151104	-0.484001735	0.163699656	0.00112197	0.015767384
**DLAT**	1181.405296	-0.485720141	0.134613855	0.00011540	0.00228287
**LAMC1**	6669.807299	-0.487455415	0.109911277	3.5E-06	0.000105123
**IGF1R**	2163.063228	-0.487500199	0.164954619	0.00109792	0.01554605
**TRAM2**	4112.235302	-0.488630655	0.149580853	0.00038670	0.006538014
**IFT122**	268.4632769	-0.490122326	0.172575008	0.00157046	0.020587536
**SLC35F2**	1298.965296	-0.490504074	0.192605109	0.00370725	0.040948063
**TIMM21**	377.6699642	-0.491248023	0.174604052	0.00171613	0.022125558
**OAT**	336.7849972	-0.491542729	0.159678389	0.00072469	0.011184155
**PEA15**	3377.70557	-0.492249917	0.163881869	0.00094342	0.013785631

**USF2**	478.0927579	-0.493616497	0.154295361	0.00049609	0.008138386
**CHD3**	2259.216649	-0.49416265	0.161096259	0.00074404	0.011424745
**SNRNP25**	415.5652385	-0.495160838	0.149263047	0.00032444	0.005601715
**SMG9**	459.353349	-0.495710333	0.162232428	0.00078264	0.011939217
**VWA1**	636.6785239	-0.497236673	0.169539512	0.00112029	0.015755564
**EPB41L4B**	821.3999837	-0.497265204	0.154500665	0.00046238	0.007672101
**CHKA**	318.8991844	-0.498468908	0.197591379	0.00387644	0.042466344
**TMEM19**	281.8955411	-0.49941491	0.172377839	0.00127489	0.01741988
**AHCYL2**	437.5052568	-0.499436668	0.166336798	0.00089289	0.013254048
**IGF2**	543.3719759	-0.500089899	0.187419345	0.00254336	0.030382822
**KIF22**	2361.749916	-0.502556708	0.141830434	0.00013958	0.002703995
**NUDT3**	530.0531595	-0.503274513	0.198029725	0.00359131	0.040045262
**CPD**	1520.129832	-0.503383226	0.144208652	0.00016969	0.003214319
**VPS26B**	528.0391315	-0.503669252	0.165826089	0.00083301	0.012554224
**C2CD2**	557.4178713	-0.503831739	0.15201975	0.00032193	0.005568574
**NACC2**	356.9659721	-0.506169473	0.168354341	0.00086628	0.012930803
**DPYSL2**	2798.188793	-0.506690113	0.198962123	0.00340805	0.038413394
**SNRPD3**	524.1239836	-0.507057679	0.150184224	0.00025232	0.004501702
**TUFT1**	1152.258146	-0.50705888	0.183452001	0.00182994	0.023272461
**CNR1**	185.1946806	-0.507210354	0.201101965	0.00376681	0.041483696
**ELOVL1**	2191.779091	-0.507258393	0.158795048	0.00047591	0.007861828
**LINC00346**	737.1810758	-0.507742907	0.161247066	0.00056122	0.008984551
**KIF3C**	418.6851581	-0.508188284	0.184482842	0.00186197	0.023647644
**BOK**	200.0596334	-0.508319911	0.190962134	0.00250326	0.030079341
**PRPS1**	291.5165314	-0.508634926	0.206521537	0.00446633	0.047274813
**RPS6KB2**	331.0025912	-0.508916338	0.172986083	0.00110367	0.015592172
**MSI2**	1165.832072	-0.510182313	0.153409532	0.00029541	0.005171983
**FNBP1**	323.8108896	-0.510537524	0.189037695	0.00216509	0.02672519
**RNF217**	5245.323403	-0.511567076	0.136672857	6.09E-05	0.001328779
**FBXO3**	1441.636269	-0.512004246	0.151173857	0.00024101	0.004337109
**CCNY**	428.6079414	-0.512889485	0.150821103	0.00023062	0.00418234
**CCNF**	988.9987205	-0.513255848	0.173935119	0.00104665	0.01496709

**HSDL2**	876.3532388	-0.514847064	0.149467814	0.00019075	0.003562744
**TYRO3**	181.2868998	-0.515993864	0.202458684	0.00330664	0.037473254
**S100A14**	932.1849295	-0.517177328	0.175386918	0.00101365	0.014584337
**LGALS3**	315.4708997	-0.517898007	0.174451482	0.00095859	0.013974549
**CLCN5**	1091.89872	-0.518298514	0.143264077	0.00010099	0.002038645
**IDH3A**	697.8124298	-0.518563384	0.203023661	0.00326726	0.037126589
**THOC3**	305.3437525	-0.518672145	0.176143829	0.00104491	0.014953673
**WNT7A**	537.6157778	-0.519757774	0.178914557	0.00120216	0.016644576
**CCDC34**	384.987482	-0.519888949	0.199475314	0.00282606	0.033026049
**NBPF1**	576.588379	-0.519939736	0.167961563	0.00064218	0.010062427
**ABCD3**	1813.156835	-0.520021748	0.14324549	9.34E-05	0.001927113
**FAM168B**	1848.182981	-0.520286252	0.11618743	2.6E-06	7.96E-05
**WSB2**	1299.032776	-0.522151318	0.155822086	0.00026674	0.004727391
**MGLL**	1695.066761	-0.523466991	0.185928623	0.00152299	0.020063485
**TMEM184A**	220.8784043	-0.523557138	0.206610649	0.00340141	0.038361602
**ATP11A**	5083.725226	-0.52419127	0.116624051	2.33E-06	7.22E-05
**MAST2**	1002.021068	-0.525757535	0.131367746	2.13E-05	0.000525593
**PHTF2**	1338.179627	-0.526158888	0.161305369	0.00035584	0.006060073
**BTD**	219.4617042	-0.526281211	0.2098985	0.00342346	0.038528328
**PAFAH1B3**	240.3211693	-0.526832515	0.204947556	0.00314241	0.035916275
**ACOT11**	180.2306893	-0.526971671	0.212888561	0.00374242	0.041263557
**PPP3CA**	1482.661213	-0.527210135	0.156089275	0.00023762	0.004284401
**PTPRF**	6772.204173	-0.529128499	0.121751433	4.61E-06	0.000133293
**ACOX1**	898.2746595	-0.530268817	0.127195366	9.96E-06	0.00026405
**FAM173B**	264.1097714	-0.531889872	0.185190564	0.00127124	0.017382687
**C1orf123**	212.4317783	-0.53305893	0.18368672	0.00112835	0.015821488
**GSN**	3084.741227	-0.534817467	0.150027723	0.00010751	0.002147169
**SCCPDH**	777.11018	-0.535539074	0.224474994	0.00477422	0.049852561
**MYBL2**	1851.717204	-0.536925744	0.128067325	7.68E-06	0.00021053
**RAB4A**	1200.150727	-0.53735664	0.169350524	0.00045654	0.007581941
**AMFR**	899.005095	-0.537407175	0.16135824	0.00027314	0.004827155
**MAZ**	2741.343008	-0.537488935	0.118654828	1.92E-06	6.07E-05

**EPHB4**	1031.336664	-0.537493619	0.128668531	9.59E-06	0.00025587
**DDHD1**	278.4894687	-0.537551212	0.207318745	0.00285465	0.033277056
**KNSTRN**	946.797557	-0.538767872	0.167552312	0.00040453	0.006802651
**CDKN2AIPNL**	293.5628841	-0.53897993	0.18365121	0.00098674	0.014280136
**SLC16A1**	4302.448118	-0.539982874	0.163793826	0.00030033	0.005253121
**LAMA1**	1169.92467	-0.540049797	0.190845801	0.00135043	0.01818673
**TEX261**	474.159646	-0.541707046	0.162706314	0.00026732	0.004733259
**ZHX1**	289.5231496	-0.54228014	0.177458445	0.00065974	0.010307746
**SRSF8**	444.7801214	-0.544353611	0.166980603	0.00034102	0.005831994
**SIPA1L3**	335.8571649	-0.546988087	0.19308783	0.00139276	0.018596327
**COMT**	1499.759828	-0.547220519	0.133868672	1.36E-05	0.000346934
**PDE7A**	1082.307163	-0.547914846	0.141158388	3.14E-05	0.000739734
**GPAA1**	440.5269185	-0.548157706	0.173640782	0.00048886	0.008047394
**SH2D3A**	288.8332289	-0.548729744	0.206120159	0.00225028	0.027498051
**TEX2**	2724.619987	-0.549538977	0.138399806	2.22E-05	0.000545354
**MAL**	2345.47183	-0.550283283	0.1409666	2.9E-05	0.000691661
**ADI1**	1320.224465	-0.550662948	0.121930645	1.95E-06	6.12E-05
**KIF20B**	2157.401134	-0.551044668	0.22536383	0.00389318	0.042575034
**DGKD**	574.2910235	-0.551630986	0.207025584	0.00219783	0.026980349
**MDFIC**	758.3642	-0.551809992	0.135160622	1.34E-05	0.000343241
**WDR91**	129.234678	-0.55241504	0.228324564	0.00422051	0.045334268
**PXMP4**	208.9258808	-0.55251924	0.208342493	0.00210736	0.026143905
**KRT14**	7029.210945	-0.554904479	0.225916471	0.00350334	0.039227801
**RASA1**	1431.189158	-0.556273906	0.134941478	1.16E-05	0.000302142
**PIMREG**	291.4383583	-0.556330661	0.209718781	0.00222701	0.027284282
**EPAS1**	2324.504366	-0.558563421	0.151299797	6.58E-05	0.001409567
**SYDE2**	246.1024137	-0.559572202	0.226718278	0.00369067	0.040813097
**ATP2B1**	869.5754715	-0.56027715	0.143098723	2.63E-05	0.000632601
**CRYBG2**	554.368758	-0.560713673	0.208559265	0.00189873	0.024016935
**ARL4A**	239.9763707	-0.561183057	0.214075097	0.00237791	0.028850685
**RAB3B**	2730.462133	-0.561934351	0.143186766	2.57E-05	0.00062086
**DLGAP5**	1512.078975	-0.562063943	0.195316449	0.00110677	0.015612305

**RPP40**	156.9417564	-0.562642052	0.236356826	0.00444172	0.04714267
**ALDH1B1**	234.6542707	-0.563479969	0.221208367	0.00290420	0.033770584
**GAS2L3**	822.7201363	-0.564311459	0.193504312	0.00098642	0.014280136
**HACD2**	3808.007402	-0.564702406	0.171656325	0.00028242	0.004963026
**ANLN**	9159.968291	-0.564705372	0.114716706	2.19E-07	8.17E-06
**SLC35D1**	530.6440501	-0.565209626	0.181585561	0.00053857	0.008682505
**GPATCH11**	192.2827938	-0.567553577	0.218629722	0.00252306	0.030220311
**PDGFB**	564.9912615	-0.567718305	0.16899251	0.00022015	0.004007866
**SEMA5A**	744.5294577	-0.569833821	0.208537091	0.00161532	0.021087074
**PHACTR2**	1820.290888	-0.570144983	0.160094059	0.00010505	0.002102498
**ATP6V0E2**	245.969103	-0.572546469	0.181459603	0.00044176	0.007358198
**NME4**	168.0682343	-0.572949533	0.226143928	0.00276813	0.032531652
**EEF1A2**	1492.781368	-0.574536771	0.160272096	9.39E-05	0.001932506
**LTBP3**	381.6402949	-0.575227588	0.203718472	0.00124990	0.01714095
**VAV2**	713.239381	-0.575241448	0.196962185	0.00093261	0.013716739
**COX20**	621.2897182	-0.575931207	0.161796918	0.00010059	0.002035101
**UBE2S**	686.9024083	-0.57729979	0.195141319	0.00083396	0.012558454
**DEPDC1**	1257.281331	-0.580109727	0.224717438	0.00257685	0.030698693
**PCYOX1**	1150.556989	-0.580114026	0.175047758	0.00024844	0.004440873
**BUB1B**	1057.53965	-0.581084395	0.221173147	0.00217569	0.02679638
**CYTH3**	737.2595765	-0.581462232	0.188839806	0.00056166	0.008984551
**ARL4C**	1119.140489	-0.581676807	0.231047144	0.00291341	0.033835746
**KIAA1522**	2856.154562	-0.582702207	0.138909649	7.54E-06	0.000207719
**GALNT1**	1121.817309	-0.583624515	0.128152626	1.51E-06	4.83E-05
**SPAG5**	1202.303244	-0.583920877	0.185466261	0.00044053	0.007348679
**MAD2L2**	220.206064	-0.584291237	0.207459169	0.00122070	0.016826675
**CASP6**	152.0923772	-0.586286538	0.225712925	0.00237781	0.028850685
**TRMT9B**	477.4048276	-0.586781233	0.219044121	0.00179477	0.022902911
**PUSL1**	157.9611484	-0.589141706	0.235607767	0.00307368	0.035216749
**TMA7**	579.2403508	-0.589524811	0.164924224	9.43E-05	0.001940054
**CMTM4**	1043.076671	-0.590531262	0.143759438	1.1E-05	0.000290008
**GPSM2**	2482.374541	-0.593597238	0.159680806	5.35E-05	0.001193599

**ZNF362**	433.0649279	-0.594089559	0.205853711	0.00095142	0.013891703
**SELENOW**	352.6356259	-0.594911634	0.18337045	0.00028590	0.005019526
**TMEM25**	117.1104351	-0.596591185	0.253955678	0.00423066	0.045417233
**NDST1**	1512.801493	-0.597002324	0.163586694	7.05E-05	0.001493274
**PPIC**	477.4907183	-0.597075652	0.161672788	6.07E-05	0.001327676
**SPRR1B**	3188.628165	-0.597457982	0.120817585	2.07E-07	7.76E-06
**FZD3**	305.5629747	-0.600235438	0.17586865	0.00016054	0.003053186
**PLCD3**	1049.338365	-0.600273896	0.190433745	0.00041182	0.006912796
**LRP6**	318.6701076	-0.60089394	0.169451091	0.00010083	0.002037627
**SPDL1**	893.301308	-0.601046394	0.206927551	0.00092255	0.013640281
**CETN2**	232.514885	-0.601177751	0.21969233	0.00148454	0.019625984
**EFEMP1**	2193.834903	-0.601927897	0.165431878	7.08E-05	0.001499519
**NSMF**	645.4749701	-0.605449273	0.14599444	7.9E-06	0.000215214
**TTC9**	251.7698024	-0.605805875	0.209784749	0.00093860	0.013768876
**SH3RF2**	1536.000804	-0.606671703	0.211428434	0.00098717	0.014280136
**DUSP14**	637.5740178	-0.607551874	0.163452847	5.31E-05	0.001188243
**PDE4D**	448.0328918	-0.608696593	0.162167843	4.6E-05	0.001044122
**PIK3C2B**	307.7619042	-0.609007275	0.217030035	0.00116910	0.01627103
**HSPA4L**	740.2737765	-0.609341383	0.167822686	7.2E-05	0.001518975
**RCN2**	636.8824073	-0.609537638	0.143799097	6.08E-06	0.000170753
**TNS3**	748.007427	-0.615626791	0.168257488	6.16E-05	0.001342805
**CAVIN1**	2666.91365	-0.618188619	0.167308723	5.4E-05	0.001202043
**COTL1**	3582.885865	-0.618417402	0.165066544	4.48E-05	0.001019837
**OIP5**	186.2283221	-0.618743616	0.201556759	0.00051137	0.008344616
**CDC25B**	3725.259539	-0.618963423	0.197540946	0.00040384	0.00679721
**SLC7A2**	852.4460548	-0.620210163	0.251781555	0.00297536	0.034384656
**TAGLN2**	2578.375419	-0.621848479	0.143218494	3.54E-06	0.000105843
**CALHM2**	221.2443059	-0.623203362	0.206558064	0.00058057	0.009210987
**KSR1**	300.3119881	-0.625217602	0.227650121	0.00135424	0.018211756
**GALNT10**	742.5681676	-0.625522749	0.162669013	2.96E-05	0.000702752
**LGALSL**	322.4131575	-0.626151318	0.175530029	8.5E-05	0.001770119
**DHRS13**	132.0809403	-0.629778258	0.237563744	0.00172396	0.022195939

**FYN**	743.4868834	-0.631045635	0.157915692	1.58E-05	0.000397206
**SESN3**	320.8840438	-0.631979147	0.179733123	9.96E-05	0.002023034
**EFNA5**	165.8921853	-0.63213218	0.231173919	0.00140244	0.018698919
**POMT2**	262.6074425	-0.633394271	0.189924126	0.00020319	0.003735436
**AFAP1L2**	140.4935353	-0.634824415	0.252831437	0.00245744	0.029653767
**BCORL1**	148.0536808	-0.636876242	0.272532941	0.00393961	0.042945732
**TMEM97**	435.4932272	-0.640031792	0.192811427	0.00020987	0.003839426
**AGO1**	3018.018499	-0.640723331	0.144031015	2.07E-06	6.47E-05
**AGAP1**	1428.995788	-0.642208044	0.121642529	3.22E-08	1.36E-06
**PTK7**	332.672136	-0.642411949	0.168185877	3E-05	0.000711978
**NRM**	628.7357878	-0.64249396	0.150761889	4.8E-06	0.000137639
**STON2**	429.5739665	-0.642545843	0.227722356	0.00102093	0.014677807
**TAF9B**	312.1573174	-0.642887999	0.227064986	0.00102440	0.014714515
**PRR11**	2087.408908	-0.644470688	0.192089667	0.00017771	0.003342438
**PLEC**	5680.423546	-0.644474578	0.128818933	1.39E-07	5.43E-06
**CCNA2**	1690.63958	-0.644747926	0.225844925	0.00092636	0.013674982
**SLC39A4**	215.7502525	-0.645179981	0.204778509	0.00037595	0.006379295
**LINC00689**	136.3036977	-0.64641833	0.265529996	0.00312533	0.035764763
**CBX2**	194.9006336	-0.646557371	0.222574047	0.00073597	0.011328632
**XKR8**	117.2742365	-0.646766545	0.280359421	0.00424698	0.045533999
**CTSV**	386.3184744	-0.647111739	0.171022824	3.4E-05	0.000793706
**S100A6**	9583.68423	-0.647942356	0.161477775	1.4E-05	0.000356275
**PAQR7**	607.0624868	-0.649886514	0.14569063	1.95E-06	6.12E-05
**USP30**	109.5932051	-0.651192781	0.266585487	0.00271000	0.031968864
**MGAT4A**	296.9418958	-0.651780187	0.182951508	8.03E-05	0.001678134
**FAM216A**	135.2426565	-0.652048969	0.240211778	0.001365	0.018277682
**CCNB2**	523.6863182	-0.652310915	0.15014072	3.32E-06	0.000100089
**HSPA12A**	144.0989265	-0.652528793	0.280042309	0.00360320	0.040058656
**NYNRIN**	185.2777382	-0.652672866	0.269610568	0.00305950	0.035097179
**CDCA3**	187.4732097	-0.652932567	0.283215041	0.00418206	0.045039602
**KIRREL1**	527.6725858	-0.654577123	0.165186434	1.64E-05	0.000412653
**SLC27A4**	726.963044	-0.655506185	0.197591488	0.00020182	0.003717547

**COL1A1**	309.6214676	-0.657593007	0.179733186	5.63E-05	0.001251324
**GPC1**	338.9910458	-0.65889865	0.180180554	5.39E-05	0.001202043
**CCDC85C**	317.9758013	-0.659943291	0.212519219	0.00041262	0.006913914
**GAS2L1**	196.3501082	-0.660391907	0.223119646	0.00064296	0.010062427
**AL035071.1**	112.9660082	-0.665789456	0.265064682	0.00230926	0.028145326
**C12orf49**	716.1727977	-0.667182646	0.215128118	0.00040806	0.006855904
**CCSAP**	1692.85399	-0.667562573	0.148052076	1.45E-06	4.64E-05
**CHST3**	1020.934903	-0.667717065	0.182956284	5.68E-05	0.001259854
**KLK5**	3362.593769	-0.670597494	0.124793028	1.75E-08	7.67E-07
**BIRC5**	1129.566693	-0.670735302	0.206334854	0.00024294	0.004363397
**IGSF3**	1489.573566	-0.672306621	0.202859489	0.00018474	0.003453993
**PBX3**	526.903143	-0.672475448	0.179563795	3.68E-05	0.000854542
**SIDT2**	185.8091792	-0.674822631	0.225542893	0.00059014	0.009337066
**ABCC2**	1245.883636	-0.675110778	0.227401132	0.00059514	0.009400387
**NLGN2**	547.4393973	-0.675147974	0.163385243	7.26E-06	0.000200865
**MIR100HG**	420.8012057	-0.675274882	0.237883014	0.00082475	0.012459807
**TPM1**	929.2739975	-0.675850909	0.20516757	0.00020226	0.0037221
**RTL10**	1444.963872	-0.676212146	0.171982124	1.84E-05	0.000458681
**GCAT**	146.7751335	-0.677528645	0.23055636	0.00067697	0.010559344
**POMGNT1**	598.0298063	-0.677773166	0.22405207	0.00050555	0.008271281
**BLMH**	203.1792357	-0.678513745	0.200992671	0.00014562	0.002809505
**LAYN**	430.7614492	-0.682074147	0.201138881	0.00014434	0.002787585
**CDC42BPG**	519.5945665	-0.683012934	0.159375714	3.9E-06	0.000114906
**FZD2**	118.5277486	-0.683388361	0.252928665	0.00123126	0.016939412
**OSR2**	331.2026914	-0.684273406	0.217880318	0.00034120	0.005831994
**TSC22D1**	918.8841365	-0.688800701	0.161564825	4.08E-06	0.000119218
**KIF20A**	863.7711265	-0.689005319	0.248543506	0.00103882	0.014877896
**INPP5A**	150.5516044	-0.690781567	0.244527894	0.00091373	0.013531205
**CHPT1**	336.7109243	-0.690861215	0.169221437	9.29E-06	0.000249671
**TMEM9**	220.1578953	-0.692641202	0.199445106	0.00010420	0.002094491
**HSPG2**	1481.59472	-0.693167998	0.135056679	6.1E-08	2.5E-06
**TIAM1**	1126.673489	-0.693400128	0.150923588	9.51E-07	3.16E-05

**ZBTB10**	629.269925	-0.694776126	0.159808194	2.73E-06	8.32E-05
**MBD3**	368.1489736	-0.694915747	0.171861318	1.12E-05	0.0002928
**RBM38**	307.8695089	-0.6953817	0.189987412	4.95E-05	0.001114295
**AKT3**	1767.631149	-0.695798977	0.131389229	2.46E-08	1.06E-06
**CENPF**	7602.215611	-0.696843794	0.203433804	0.00014022	0.002713695
**FAM172A**	324.3468611	-0.697621526	0.183481986	3.03E-05	0.000715572
**SLC22A23**	519.6441201	-0.698456682	0.202813133	0.00011152	0.002217784
**TMEM178B**	397.9007208	-0.698518154	0.208102406	0.00015038	0.002889386
**GCNT1**	1491.000611	-0.698721227	0.213058767	0.00019633	0.003634322
**TRIM29**	1633.963883	-0.698783708	0.145497601	3.39E-07	1.22E-05
**H2AFV**	3881.588196	-0.699102176	0.120684048	1.48E-09	7.49E-08
**ST3GAL4**	535.2256331	-0.699791815	0.147432085	4.45E-07	1.56E-05
**EVPL**	663.4438784	-0.701235828	0.141851374	1.63E-07	6.25E-06
**PCDH9**	349.2047094	-0.70162102	0.210985136	0.00015864	0.003023347
**TFAP2C**	1146.685606	-0.702616829	0.189278025	4.14E-05	0.000948743
**BZW2**	1898.209877	-0.703354823	0.204775415	0.00011399	0.002257439
**OSBP2**	2472.57687	-0.711260501	0.134208405	2.42E-08	1.05E-06
**TP53I3**	131.0256654	-0.71188557	0.243350949	0.00060081	0.009473961
**CAT**	527.4608325	-0.713360364	0.209325962	0.00012704	0.002486785
**GM2A**	1673.129022	-0.716689952	0.150924887	4.19E-07	1.49E-05
**DYNC2H1**	635.2047742	-0.719487082	0.231200249	0.00033196	0.005694839
**PSORS1C1**	329.3514322	-0.720049289	0.170568782	4.68E-06	0.000134442
**TLL1**	216.0840791	-0.720076995	0.260528157	0.00099185	0.014325684
**IRX2**	113.5332771	-0.726522672	0.30458777	0.00262622	0.031176859
**GLRB**	87.35145745	-0.726812137	0.301415543	0.00249934	0.030070789
**CRISPLD2**	2350.443797	-0.72876532	0.173289	4.84E-06	0.000138762
**SYT7**	129.0419996	-0.729636342	0.238442497	0.00039979	0.006735091
**DDAH2**	237.3957506	-0.730473317	0.194583195	3.17E-05	0.000745758
**TET3**	1419.312399	-0.73116044	0.173649594	4.95E-06	0.000141552
**RNF182**	244.4104589	-0.731309442	0.297021759	0.00219521	0.026965851
**ENAH**	913.6999781	-0.733929964	0.199187626	4.1E-05	0.000942871
**SPC24**	557.4378566	-0.735064813	0.199608809	4.38E-05	0.000998924

**TFRC**	8789.331009	-0.736245171	0.124066734	3.7E-10	1.98E-08
**RHOD**	1596.822407	-0.738387184	0.173273324	3.87E-06	0.000114337
**CDKN3**	448.092975	-0.738857863	0.160135387	7.74E-07	2.59E-05
**FAM198B-AS1**	417.8377346	-0.744288895	0.332362203	0.00361323	0.040146344
**KLF8**	138.1472478	-0.745899213	0.230364857	0.00019442	0.003613316
**DANCR**	274.5777783	-0.747159926	0.220864126	0.00012295	0.002419342
**KDELC1**	198.4829583	-0.748880735	0.211784383	6.75E-05	0.001440439
**SLC7A5**	14551.215	-0.751736221	0.153637842	1.73E-07	6.59E-06
**KCTD12**	2043.219978	-0.751800013	0.172250041	2.29E-06	7.1E-05
**FHOD1**	334.5845659	-0.752867054	0.184436565	8.54E-06	0.000231443
**SERPINB7**	1303.022761	-0.754962882	0.157396595	3E-07	1.09E-05
**C5orf38**	98.43357297	-0.755290639	0.315492918	0.00245531	0.029653767
**RGS4**	3744.885219	-0.757262126	0.274530158	0.00088028	0.013097989
**UCP2**	224.5041239	-0.757928905	0.212819213	6.65E-05	0.001422928
**MMP15**	108.4333537	-0.764578117	0.28565268	0.00121422	0.016762056
**WFS1**	318.4310688	-0.766316288	0.194703669	1.46E-05	0.000370413
**TUBB2A**	256.360285	-0.766540187	0.253455459	0.00038601	0.006532265
**ANXA9**	94.09020457	-0.766730841	0.263312922	0.00056933	0.00907682
**LIMK2**	1236.457457	-0.76691904	0.146278974	2.87E-08	1.22E-06
**SPTBN2**	193.3481502	-0.770626758	0.247453234	0.00030780	0.005363793
**FAM126A**	1493.599394	-0.772001248	0.167778262	7.46E-07	2.51E-05
**ZMAT3**	499.9068915	-0.773838679	0.21810395	6.53E-05	0.001402084
**SOGA1**	2792.260513	-0.774899916	0.217168758	6.27E-05	0.001360705
**SLC16A7**	1135.374833	-0.776646241	0.201464386	1.92E-05	0.000478769
**ALDH4A1**	102.1062653	-0.778739088	0.315749994	0.00199025	0.024955439
**SLC39A10**	491.9743064	-0.783112426	0.201601562	1.7E-05	0.000427502
**PRSS27**	70.07249513	-0.784704397	0.308704865	0.00154344	0.020275803
**SEMA6B**	159.3510021	-0.787853034	0.28362765	0.00072795	0.011223702
**PPARG**	205.0054207	-0.788541235	0.246484042	0.0002185	0.003981666
**DAAM1**	347.6590819	-0.788546243	0.162858529	2.18E-07	8.15E-06
**ERMP1**	725.675439	-0.793074635	0.142499622	4.24E-09	2E-07
**CASTOR2**	446.3815254	-0.793827352	0.159342361	1.11E-07	4.39E-06

**ANTXR1**	1843.284121	-0.794150481	0.176660191	1.15E-06	3.77E-05
**ANKH**	812.8781091	-0.79709276	0.154778278	4.39E-08	1.83E-06
**MYH10**	2047.791122	-0.797310489	0.180594714	1.64E-06	5.23E-05
**ERVMER34-1**	135.6314432	-0.799043731	0.273020464	0.00053048	0.00857431
**SLC12A2**	1449.775028	-0.799551314	0.173797276	7.1E-07	2.4E-05
**ZNF385A**	54.12039819	-0.799965113	0.370719855	0.00368972	0.040813097
**SDC1**	1796.260909	-0.800096157	0.130005199	1.37E-10	7.75E-09
**MIR600HG**	147.354437	-0.801129975	0.253539448	0.00024175	0.004346163
**HECW2**	588.3026367	-0.801423047	0.265373504	0.00037698	0.006391063
**SERPINE1**	76.20223401	-0.80283901	0.312491457	0.00130075	0.017670079
**PLA2G12A**	328.5541872	-0.803648164	0.192857391	5.18E-06	0.000147339
**CYFIP2**	365.9658388	-0.803868432	0.165892306	2.04E-07	7.69E-06
**DAG1**	3298.042314	-0.80473177	0.142230801	2.64E-09	1.29E-07
**CAB39L**	83.94456241	-0.806569598	0.39211193	0.00476955	0.049831644
**HEG1**	1427.158873	-0.808569984	0.205455729	1.32E-05	0.000338462
**GPR37**	90.84399294	-0.809869049	0.313850797	0.00133701	0.01804489
**PRSS3**	174.5421884	-0.811764926	0.221394899	3.81E-05	0.000878924
**SCEL**	479.6897415	-0.812071473	0.314391902	0.00127820	0.017437957
**PRKAR2A**	1133.210196	-0.816303363	0.14208419	1.6E-09	8.1E-08
**KLK6**	644.0462268	-0.821743806	0.269023473	0.00030847	0.005370466
**MVK**	205.1586839	-0.821979176	0.213938285	1.91E-05	0.000475676
**SAPCD2**	842.508608	-0.82361649	0.145655817	2.62E-09	1.28E-07
**KLK7**	1472.089757	-0.825339551	0.169374606	1.64E-07	6.27E-06
**ADAP1**	89.31897772	-0.826007151	0.335025342	0.00171050	0.0220833
**ZHX3**	772.9859988	-0.826481974	0.163596724	7.17E-08	2.93E-06
**SYNE1**	1870.500226	-0.830202453	0.20364765	7.02E-06	0.000194473
**TGFBI**	701.2925293	-0.834033114	0.328868393	0.00132043	0.01788552
**LGALS1**	964.3228167	-0.836584772	0.203669877	5.52E-06	0.000155888
**CASC10**	75.69536076	-0.838390008	0.308668334	0.00085111	0.012724545
**PRTG**	345.125911	-0.843147591	0.25914416	0.00015391	0.002951121
**E2F2**	671.2611999	-0.844078102	0.204170803	5.12E-06	0.000146223
**FAM198B**	756.1198768	-0.847978889	0.225179431	2.32E-05	0.000567685

**CABYR**	66.10822859	-0.857336103	0.323865037	0.00103584	0.014857151
**KRT13**	892.7329076	-0.858194493	0.219674356	1.33E-05	0.000342457
**AC000068.1**	40.05482449	-0.860394188	0.414190391	0.00405414	0.043950365
**SPRR3**	6384.717264	-0.861238703	0.146552539	6.3E-10	3.33E-08
**ADAMTS12**	801.0246735	-0.861891959	0.25266825	8.3E-05	0.001733396
**HYI**	244.9889576	-0.864975831	0.193644339	1.24E-06	4.04E-05
**SLCO2A1**	424.680173	-0.865975611	0.331174693	0.00107589	0.015307415
**ONECUT2**	158.7949134	-0.868672263	0.273753425	0.00020148	0.003714923
**XAGE2**	45.18001062	-0.869798809	0.395637698	0.00310022	0.035499087
**PSG1**	179.1643049	-0.873973888	0.349645668	0.00140531	0.018723899
**PLPPR2**	336.4951813	-0.876992412	0.186235973	3.39E-07	1.22E-05
**AL109918.1**	346.5104774	-0.87860891	0.243501359	3.82E-05	0.000882235
**NEK2**	709.5178161	-0.88026598	0.164945834	1.44E-08	6.41E-07
**GRIK2**	87.21225866	-0.881539405	0.30690172	0.00044339	0.007376612
**TRPC4**	121.2920032	-0.885256896	0.266876971	0.00011843	0.002337949
**BAD**	223.7272916	-0.889450775	0.216380876	5.25E-06	0.000148597
**PALLD**	87.38802423	-0.892182186	0.379522532	0.00210969	0.02614541
**TGFB2**	583.6063546	-0.892656782	0.144857778	1.01E-10	5.8E-09
**FSTL3**	183.6820869	-0.897094856	0.336541562	0.00084754	0.012711839
**AC093904.4**	57.96562207	-0.901788431	0.343696985	0.00100435	0.014483839
**DNAH5**	420.2923754	-0.910506719	0.168228186	8.98E-09	4.07E-07
**KIAA1549**	2191.112012	-0.916078348	0.150782666	1.79E-10	9.94E-09
**ACO1**	423.6624209	-0.917716068	0.19703122	4.65E-07	1.62E-05
**ABCA1**	1485.119917	-0.9193475	0.170355165	8.76E-09	3.98E-07
**UNC13D**	356.8272399	-0.919998931	0.201051176	6.31E-07	2.16E-05
**NRAV**	261.3697379	-0.923185596	0.247150078	2.24E-05	0.000549132
**DIRC3-AS1**	32.84824815	-0.927170098	0.474593598	0.00462676	0.048610848
**SCARA3**	431.4347994	-0.93164087	0.204019672	6.04E-07	2.08E-05
**TRIM2**	159.2772247	-0.933862961	0.295088964	0.00018191	0.003407824
**ZNF704**	1742.836751	-0.941474563	0.218494737	1.89E-06	5.98E-05
**AL590644.1**	121.8055694	-0.947809612	0.260607766	3.21E-05	0.000753724
**FAM46B**	121.3038728	-0.964413522	0.316479713	0.00023262	0.004210451

**TPD52L1**	727.3028289	-0.964537366	0.192582563	7.27E-08	2.96E-06
**AC098934.1**	63.57484298	-0.965024979	0.433816028	0.00193405	0.024332144
**MPZL3**	1219.344526	-0.969656692	0.160331951	1.71E-10	9.56E-09
**AC005392.2**	51.60865311	-0.970682436	0.441353702	0.00242512	0.029347481
**RETREG1**	508.0668445	-0.976201817	0.171044553	1.37E-09	6.98E-08
**ENDOD1**	1039.253776	-0.978288064	0.132205587	1.69E-14	1.34E-12
**FAM98C**	45.32988757	-0.980715881	0.385277954	0.00111402	0.015702756
**KRT16**	75.44453266	-0.986339749	0.378663283	0.00088552	0.013165446
**CCDC80**	109.3393389	-0.989971539	0.303911998	0.00010927	0.002177794
**AL513327.3**	47.6536064	-0.991049562	0.442001005	0.00207118	0.025763326
**SLC35F3**	572.0721196	-0.992232752	0.193488172	3.16E-08	1.34E-06
**FRMD4A**	238.5832314	-0.993562499	0.334775131	0.00031486	0.005471584
**LMCD1**	122.4230034	-0.995615291	0.326010434	0.00022390	0.004068297
**NKD1**	35.11242095	-0.998280247	0.458194217	0.00236940	0.028797872
**NT5DC2**	318.7403991	-1.004029627	0.211926099	2.43E-07	8.95E-06
**THBS1**	9008.496973	-1.004705015	0.266867812	2.04E-05	0.000506027
**GDPD5**	47.55614603	-1.013245582	0.498955553	0.00263642	0.031278109
**FOXI1**	44.68030318	-1.014551738	0.441333075	0.00188858	0.023937004
**LY6G6C**	83.35443941	-1.01938111	0.31218538	0.00010347	0.002084322
**CHP2**	275.9485911	-1.019605755	0.185564111	5.01E-09	2.33E-07
**USP13**	176.1088689	-1.022770008	0.306366592	8.34E-05	0.001739199
**THEM6**	140.9697842	-1.024106857	0.239988665	2.45E-06	7.53E-05
**BCYRN1**	76.85735851	-1.029171367	0.392311488	0.00084473	0.012681418
**ODC1**	948.3615753	-1.034561417	0.224367847	4.64E-07	1.62E-05
**NOTCH3**	190.2938636	-1.039363137	0.37937534	0.00049807	0.008163146
**FAM49A**	129.87278	-1.042963568	0.445180985	0.00160568	0.020975797
**MTSS1L**	458.6422608	-1.043163774	0.163823077	2.03E-11	1.24E-09
**PKP1**	535.2060496	-1.047376497	0.172568279	1.37E-10	7.75E-09
**SUN2**	717.6367336	-1.050979498	0.171172138	1.01E-10	5.8E-09
**UBL3**	839.6097088	-1.053536597	0.154615667	1.14E-12	7.83E-11
**PRADC1**	54.03748924	-1.054578912	0.344358918	0.00020739	0.003797739
**CKB**	223.8186523	-1.054666803	0.259637455	4.14E-06	0.000120296

**IVL**	152.015399	-1.05658437	0.306892429	4.81E-05	0.001086346
**MMP16**	83.95492874	-1.057108078	0.381551403	0.00043298	0.007235658
**BLK**	623.8858809	-1.06000685	0.228539594	3.37E-07	1.22E-05
**COL1A2**	233.824641	-1.061901828	0.394316262	0.00064285	0.010062427
**CDR2L**	123.245747	-1.069063314	0.262915207	5.66E-06	0.000159468
**NOG**	147.3544099	-1.069976147	0.424819677	0.00093285	0.013716739
**PTPRS**	1075.826158	-1.071528967	0.155095701	5.95E-13	4.22E-11
**ANK1**	454.7543043	-1.073013134	0.188426333	1.41E-09	7.16E-08
**MAPK4**	407.7078671	-1.073818804	0.245868358	1.15E-06	3.77E-05
**ATP7B**	151.293672	-1.077220378	0.331103416	9.81E-05	0.002000587
**EXPH5**	1676.180137	-1.079007631	0.298453748	2.85E-05	0.000679879
**FAM13A**	217.7941565	-1.093329074	0.247091358	9.67E-07	3.2E-05
**ILDR2**	20.71562955	-1.09444599	0.62507918	0.00460294	0.048442098
**CERK**	309.9031262	-1.094452653	0.198916842	3.31E-09	1.58E-07
**MEIOC**	43.27978811	-1.096184304	0.439958141	0.00108463	0.015416148
**GJA5**	1419.099789	-1.099553514	0.536094606	0.00254181	0.030382822
**TRPV6**	30.16465969	-1.105533093	0.473852799	0.00149063	0.019678737
**ALPP**	263.7481957	-1.114793215	0.221172915	5.57E-08	2.3E-06
**ULBP3**	207.9741926	-1.12647032	0.248905732	6E-07	2.07E-05
**PTGES**	955.5759841	-1.127953337	0.183875889	9.81E-11	5.65E-09
**CDK15**	24.18073621	-1.130459282	0.614997718	0.00444949	0.047198414
**SPTB**	30.70913929	-1.132957185	0.631471123	0.00394313	0.042945732
**SORL1**	1142.639741	-1.140102785	0.190853034	2.33E-10	1.28E-08
**GOLIM4**	1981.941874	-1.144909999	0.176978352	1.02E-11	6.43E-10
**RTN4RL1**	54.1081744	-1.15039326	0.354049203	0.00010228	0.002062457
**GDF11**	88.23757784	-1.151287963	0.316024515	1.97E-05	0.00048736
**CACNG4**	310.2060471	-1.151766665	0.265244404	1.21E-06	3.96E-05
**NALCN**	159.7773623	-1.152333373	0.250956081	4.21E-07	1.49E-05
**LOXL2**	50.80242826	-1.174462306	0.698548421	0.00423908	0.045481529
**EVA1A**	422.2561333	-1.176797968	0.208673855	1.77E-09	8.89E-08
**PRR16**	34.2701706	-1.182857694	0.564280387	0.00251966	0.030220311
**PROM2**	484.2727649	-1.190046697	0.196657971	1.44E-10	8.09E-09

**CDSN**	124.6477793	-1.202084291	0.334289956	2.68E-05	0.000642524
**AC132872.4**	68.06474813	-1.20700893	0.49705758	0.00094077	0.013777608
**GJB6**	428.4402166	-1.238370657	0.257435594	1.45E-07	5.63E-06
**SYNE3**	29.68819511	-1.247231776	0.56581978	0.00165972	0.021554622
**CDKN2C**	185.5191565	-1.249073695	0.242069952	1.86E-08	8.09E-07
**CAMK4**	363.2258125	-1.251240977	0.242242433	2.13E-08	9.25E-07
**HRK**	52.71969246	-1.276523921	0.450860848	0.00032340	0.005588875
**SLC25A29**	23.56578165	-1.279937061	0.554468296	0.00126707	0.017338219
**AC110619.1**	178.8063555	-1.29282348	0.386550458	6.35E-05	0.001371043
**EPN3**	195.5590188	-1.300615285	0.234967128	2.69E-09	1.31E-07
**ELF5**	25.16428936	-1.307985837	0.622585391	0.00222841	0.027284282
**FBN1**	3244.098178	-1.313863955	0.164152042	1.08E-16	1.02E-14
**PADI2**	519.6673496	-1.316849159	0.212285148	4.13E-11	2.46E-09
**PADI3**	62.67113904	-1.329263825	0.531552511	0.0006404	0.010047411
**GTF2IRD2P1**	15.86396514	-1.34652574	0.769955646	0.00418346	0.045039602
**GJC2**	51.23318021	-1.355280947	0.392732838	3.76E-05	0.000869525
**IGSF9**	160.1154589	-1.363989739	0.239496599	1.08E-09	5.55E-08
**ABAT**	551.1953776	-1.366307644	0.152163432	2.1E-20	2.43E-18
**DAW1**	106.8263899	-1.370270465	0.311355131	7.12E-07	2.4E-05
**SLC19A3**	85.34555076	-1.390448039	0.404101879	4.51E-05	0.00102585
**AGFG2**	63.6370533	-1.390653161	0.351406564	5.58E-06	0.000157479
**WNT3**	23.14856126	-1.393030555	0.580819055	0.00109065	0.015454724
**MOXD1**	76.4200903	-1.404925104	0.346338435	4.68E-06	0.000134442
**AP000812.2**	1003.996515	-1.424596643	0.29225979	8.67E-08	3.49E-06
**ZNF488**	93.15646297	-1.438660723	0.319506815	4.82E-07	1.68E-05
**DBN1**	585.9785435	-1.439655644	0.163756094	1.42E-19	1.57E-17
**PLXND1**	182.1277138	-1.448455857	0.253763542	8.44E-10	4.4E-08
**LINC01949**	21.57806273	-1.453849834	0.663886268	0.00140996	0.018772436
**S100A4**	1237.472586	-1.455366279	0.206487591	1.31E-13	9.85E-12
**RAB15**	41.1564912	-1.457388334	0.459839055	9.73E-05	0.001993046
**RDH10**	5014.773746	-1.467218925	0.253372396	5.38E-10	2.85E-08
**MAN1C1**	20.39461489	-1.468833223	0.795384358	0.00219435	0.026965851

**MGAT3**	17.50302335	-1.491586223	0.869172844	0.00284601	0.033197001
**CLDN9**	35.92244532	-1.4930091	0.544613416	0.00039026	0.006592263
**AC098934.2**	26.58948428	-1.511396386	0.566952133	0.00049613	0.008138386
**RHOV**	63.05195389	-1.531419905	0.403690469	1E-05	0.000265775
**AGR2**	143.4841394	-1.589402732	0.309649845	2.53E-08	1.09E-06
**AC068756.1**	12.03897117	-1.602578027	0.904125982	0.00320686	0.036608332
**ENSG00000287963**	93.19856392	-1.609419354	0.417330583	9.6E-06	0.00025587
**KCNMB4**	71.63955837	-1.621120163	0.354471375	2.29E-07	8.48E-06
**DSG1**	3733.366063	-1.633006519	0.531647644	0.00010858	0.002166189
**SLIT3**	52.33500098	-1.643292136	0.474720656	3.23E-05	0.000756213
**SPRR2E**	122.7687267	-1.644776823	0.425208751	6.03E-06	0.000169698
**ENSG00000287119**	11.31333649	-1.715170042	1.089797071	0.00344887	0.038780199
**NMU**	110.1703905	-1.72350598	0.405216665	1.02E-06	3.35E-05
**RBBP8NL**	13.48521832	-1.745187888	1.057951612	0.00305618	0.035080607
**LINC00390**	9.172013404	-1.781846581	1.095600962	0.00383685	0.042131211
**GUCY1A2**	330.6563172	-1.817977823	0.267801393	5.61E-13	4.01E-11
**WNT10A**	142.4027461	-1.837052985	0.343889089	6.08E-09	2.79E-07
**INSL4**	13.4703929	-1.848246998	0.817677388	0.00138259	0.018486923
**PRR15L**	16.66580627	-1.871406522	0.876117436	0.00164781	0.021451413
**CRIP2**	27.99245659	-1.876566698	0.834985379	0.00077932	0.01189815
**FBN2**	1480.558093	-1.89673821	0.667397687	0.0001913	0.00356935
**MFAP5**	16.49834415	-2.02377499	0.955322747	0.00175742	0.022502909
**TM4SF1**	7.78079617	-2.038299618	1.186013467	0.00413313	0.044625959
**CD24**	605.185644	-2.05289075	0.231584273	6.44E-20	7.27E-18
**P2RY2**	388.5799402	-2.06876114	0.232644599	4.04E-20	4.59E-18
**APLP1**	11.27503058	-2.130256063	1.022222821	0.00134257	0.018097722
**ADORA1**	27.31391618	-2.143281343	0.58394888	1.26E-05	0.000325473
**CTAGE1**	12.32841476	-2.149078421	0.904640218	0.00104792	0.014973865
**GCC2-AS1**	6.214221961	-2.202380978	1.760059911	0.00465151	0.048734201
**AC105052.2**	6.565031776	-2.392361082	1.434837432	0.00429704	0.045876874
**ENSG00000287364**	7.070983949	-2.624558369	1.357495362	0.00282088	0.032986087
**KRT3**	7.952769877	-2.795792885	1.225782759	0.00132262	0.017897699

**COL11A1**	26.08611681	-2.902501758	0.760312446	9.23E-06	0.000248716
**FREM2**	182.7452351	-2.930339962	0.297166221	1.02E-23	1.49E-21
**TEK**	49.03683045	-3.194642792	0.486085176	2.22E-12	1.49E-10
**AC108136.1**	8.997660255	-3.598477566	1.253453074	0.00028208[illegible]	0.004962494
**KRT78**	8.165515319	-4.346135216	1.49442507	0.00016589[illegible]	0.003145484
