# Supplementary material for: Role of Forkhead Box P3 in IFNγ-Mediated PD-L1 Expression and Bladder Cancer Epithelial-to-Mesenchymal Transition
Source: Cancer Res Commun. 2024 Aug 26;4(8):2228–41. doi: 10.1158/2767-9764.CRC-23-0493 (PMC11345674; doi:10.1158/2767-9764.CRC-23-0493)
Supplement: Supplementary Table 3 — FDRs and p-values of FOXP3-dependent genes [file crc-23-0493_supplementary_table_3_suppst3.pdf]

Fig2.E_FOXP3_dependent_genes

KO_IFN vs WT_IFN DE Genes (7207)	baseMean	log2FoldChange	lfcSE	pvalue	padj
**SEMA6D**	129.166492	11.38116791	3.010992683	2.25E-16	6.1E-15
**HPGD**	106.684514	11.09154843	2.965772216	4.26E-16	1.13E-14
**SH3BGRL**	62.38764933	10.18768153	2.880913733	1.19E-13	2.56E-12
**PTGIS**	62.87217677	10.17624413	2.905054327	5.04E-13	1.02E-11
**ATOH8**	228.4791	9.903008603	1.308364547	1.08E-18	3.56E-17
**AC008514.1**	51.69811936	9.884123417	2.85777826	8.07E-13	1.6E-11
**FGD5**	390.8462588	9.861442563	1.285199475	1.31E-15	3.34E-14
**FLT1**	1841.183749	9.796283177	1.349003356	1.21E-14	2.83E-13
**PRLR**	1620.223591	9.765924761	0.545566562	1.35E-70	4.82E-68
**ZNF208**	47.34338682	9.740212546	2.84134948	1.59E-12	3.08E-11
**ENSG00000287227**	47.92173378	9.707600016	2.883313263	2.03E-11	3.5E-10
**SIGLEC6**	4997.547351	9.675200435	0.413499475	2.47E-121	4.54E-118
**VIT**	47.283055	9.667165239	2.881401148	2.65E-11	4.5E-10
**SPAG17**	43.59841021	9.58024387	2.83926045	8.31E-12	1.49E-10
**ADAMTS15**	1131.217329	9.570115224	0.549053816	1.25E-65	3.78E-63
**CAPN6**	1104.789271	9.540682976	0.599709148	1.81E-55	3.33E-53
**TRPA1**	298.0820799	9.481061789	0.985641976	4.75E-23	2.14E-21
**ACKR3**	35.63883305	9.241685934	2.796888288	2.68E-11	4.54E-10
**WNT6**	35.28846524	9.233565622	2.811763398	6.36E-11	1.03E-09
**MXRA5**	33.83741486	9.074569704	2.842368627	7.96E-10	1.14E-08
**HSD3B1**	224.1460754	9.062272672	1.048828859	5.67E-19	1.9E-17
**BCHE**	123.5403118	8.986943518	1.336067061	6.65E-15	1.6E-13
**GPLD1**	121.7306976	8.973312796	1.315016009	2.59E-15	6.45E-14
**PPIAP46**	31.36642196	8.906678436	2.863730297	5.18E-09	6.63E-08
**SLC27A6**	204.043018	8.884284844	0.983141386	9.7E-21	3.78E-19
**SLC10A1**	29.6683785	8.85408731	2.82158308	2.06E-09	2.8E-08
**MTUS2**	27.80209101	8.813416529	2.783673176	7.78E-10	1.12E-08
**AC010615.2**	27.00597428	8.745830909	2.760099991	5.55E-10	8.12E-09
**SHOX**	27.11679948	8.727203028	2.777536704	1.21E-09	1.69E-08
**LINC00560**	102.4402035	8.72030323	1.31562216	1.68E-14	3.89E-13
**DPPA4**	25.47642814	8.671001998	2.75396704	8.52E-10	1.21E-08
**BOC**	313.237398	8.647628664	0.728043762	3.33E-31	2.35E-29
**L1CAM**	319.6817366	8.641242979	0.72936561	1.52E-31	1.09E-29

**MS4A15**	92.64459084	8.479278825	1.341121993	1.68E-13	3.54E-12
**ALDH1A1**	288.3106337	8.478713813	0.755848457	9.33E-29	5.72E-27
**MPPED1**	22.74897973	8.471928456	2.740911096	2.55E-09	3.42E-08
**ENSG00000286454**	24.2523685	8.451066009	2.820707549	2.82E-08	3.31E-07
**PIP5K1B**	88.85531564	8.450724375	1.356825861	4.6E-13	9.34E-12
**PDE3B**	281.2485366	8.436491278	0.77182015	1.47E-27	8.54E-26
**AC074135.1**	22.33360693	8.406011174	2.734418547	3.29E-09	4.34E-08
**A2M**	21.77420328	8.386909998	2.723661502	2.69E-09	3.6E-08
**ZNF98**	21.23760394	8.343314776	2.722683659	3.64E-09	4.77E-08
**LAMB4**	622.7896967	8.339718826	0.806048392	7.74E-25	3.87E-23
**LINC02518**	21.37176358	8.332565621	2.725678407	4.31E-09	5.6E-08
**AC024230.1**	20.86201496	8.31278482	2.724630499	4.9E-09	6.31E-08
**PTPRQ**	425.7330616	8.302691278	0.57752666	3.1E-46	3.93E-44
**CSF3R**	22.75406287	8.294920999	2.874839929	2.12E-07	2.19E-06
**SLC12A3**	20.47659397	8.211671533	2.761484504	2.79E-08	3.28E-07
**KANK4**	225.5889676	8.150111102	0.754271733	2.36E-26	1.29E-24
**MCC**	625.2567985	8.144000955	0.567361043	3.08E-48	4.28E-46
**AL139005.1**	19.77499534	8.09502682	2.781318212	8.71E-08	9.5E-07
**TP53I11**	409.0148281	8.092430251	0.573257273	3.28E-44	3.83E-42
**PIWIL1**	217.7932174	8.089961407	0.74220195	6.03E-27	3.41E-25
**APBA1**	18.55495201	8.083819466	2.70592766	1.42E-08	1.72E-07
**ZNF730**	18.34756396	8.065123403	2.702876215	1.48E-08	1.79E-07
**GALNT18**	454.6525511	8.04775836	0.575723359	3.44E-44	3.99E-42
**PBX1**	1099.392173	8.034263346	0.38529711	2.57E-98	1.92E-95
**ST8SIA1**	17.73049731	7.999675413	2.729064248	4.67E-08	5.32E-07
**ENSG00000285969**	19.50455562	7.985474723	2.8484962	5.38E-07	5.21E-06
**RNGTTP1**	17.72990061	7.972035506	2.716310548	3.91E-08	4.5E-07
**AC091885.1**	18.09548521	7.963427757	2.745799175	8.29E-08	9.08E-07
**ZNF492**	17.00007892	7.93455564	2.717926516	5.31E-08	6E-07
**IGFL2**	16.50418134	7.832434865	2.743118959	1.68E-07	1.76E-06
**ITLN2**	16.7255928	7.829903304	2.729021264	1.22E-07	1.31E-06
**ZNF429**	57.28250693	7.829338063	1.311096744	5.08E-12	9.3E-11
**FGD3**	56.43231007	7.806732902	1.314631707	6.61E-12	1.2E-10
**CLVS2**	16.21239448	7.795364325	2.731697278	1.6E-07	1.68E-06
**ZNF737**	94.07362355	7.763481714	0.996173933	1.06E-15	2.73E-14

**LINC01666**	15.75486275	7.756464449	2.697953437	9.35E-08	1.02E-06
**AL354714.1**	15.82007407	7.743366682	2.721279755	1.73E-07	1.81E-06
**AC008132.1**	14.97970533	7.675731581	2.690504351	1.29E-07	1.38E-06
**MAB21L3**	14.50457645	7.653621562	2.674767661	1.03E-07	1.11E-06
**ZSCAN4**	14.37379994	7.608760592	2.676204076	1.36E-07	1.45E-06
**KCNJ16**	48.50188556	7.587356039	1.337321602	5.38E-11	8.77E-10
**FOXN3**	445.6064317	7.559815756	0.48252791	1.47E-56	2.74E-54
**CD244**	48.45329734	7.53762501	1.391612149	3.43E-10	5.13E-09
**AGR3**	13.72935246	7.509127667	2.676354982	2.42E-07	2.47E-06
**GSTM5**	13.60863778	7.471446967	2.686450047	3.59E-07	3.57E-06
**TPT1P5**	13.37276947	7.459319246	2.674233199	3.04E-07	3.06E-06
**EMILIN2**	13.6825021	7.417386308	2.734407494	1.08E-06	9.99E-06
**VCAN**	116.6186873	7.382947167	1.655745661	7E-07	6.66E-06
**LINC00276**	12.94184951	7.358652081	2.692919535	7.17E-07	6.79E-06
**CLEC1A**	41.67358003	7.353922269	1.338752825	2.09E-10	3.21E-09
**SPP1**	41.179154	7.33142706	1.353895873	3.68E-10	5.5E-09
**ENSG00000286826**	13.16385766	7.330476288	2.741872795	1.79E-06	1.59E-05
**AOC1**	43.52343277	7.323984893	1.366896741	4.17E-10	6.2E-09
**ZNF100**	100.5376952	7.272582685	0.857976212	6.95E-18	2.13E-16
**CBLN2**	66.75728987	7.265185728	1.013328952	1.37E-13	2.92E-12
**AC007207.2**	12.3093506	7.259168894	2.745383669	2.68E-06	2.31E-05
**JAM2**	467.2672854	7.243977506	0.477919506	3.37E-51	5.19E-49
**RNU6-171P**	11.28801496	7.127659623	2.676028973	1.64E-06	1.47E-05
**FCGR2B**	11.50440462	7.109514783	2.694913271	2.3E-06	2.01E-05
**CST8**	12.86031283	7.105601076	2.872308311	1.74E-05	0.0001275
**LPL**	413.1185388	7.090813905	1.063791447	2.1E-12	4.04E-11
**MFAP5**	317.4967543	7.085305029	0.829675317	1.09E-17	3.29E-16
**AL357568.2**	10.66714085	7.060250173	2.653090612	1.57E-06	1.41E-05
**ZNF729**	10.49899738	7.022336229	2.659329913	2.06E-06	1.82E-05
**AC092279.2**	34.35598544	7.011443669	1.319533432	6.71E-10	9.7E-09
**ZNF835**	10.66941542	6.976796872	2.679950906	3.28E-06	2.77E-05
**ZNF716**	124.3176882	6.968404578	0.673181901	1.24E-24	6.13E-23
**DLGAP2**	33.06549986	6.961233407	1.355657477	2.51E-09	3.38E-08
**PLCE1-AS1**	10.38038491	6.957427022	2.66631067	2.97E-06	2.54E-05
**ITLN1**	10.37868878	6.943306523	2.677989689	3.71E-06	3.11E-05

**AP000550.1**	10.05965425	6.924296587	2.64456267	2.51E-06	2.18E-05
**IL9R**	10.00151859	6.887636385	2.653759647	3.34E-06	2.82E-05
**OLFML1**	9.914969247	6.886825016	2.68609054	5.4E-06	4.37E-05
**TTC28**	9.881864143	6.874310542	2.690216956	5.34E-06	4.33E-05
**RNF150**	9.740562263	6.864643151	2.652641066	3.69E-06	3.09E-05
**COL4A3**	430.1587052	6.85867734	0.382510764	3.55E-71	1.29E-68
**STXBP6**	54.26531182	6.84702911	1.122593129	9.9E-11	1.57E-09
**CAP2P1**	9.567793948	6.801428321	2.650362025	4.57E-06	3.75E-05
**PAGE4**	218.4235832	6.79713587	0.514248098	1.73E-40	1.76E-38
**AL031767.1**	9.444274594	6.793070693	2.642717634	4.3E-06	3.55E-05
**TRIL**	9.312919641	6.784749549	2.645178457	4.66E-06	3.82E-05
**FAAH2**	31.38946399	6.778507337	1.346974938	3.42E-09	4.49E-08
**WHAMMP2**	29.0799964	6.748843976	1.326646045	3.19E-09	4.22E-08
**LUM**	325.1083955	6.74880674	0.47117063	1.55E-48	2.17E-46
**APOB**	10.34167044	6.726824114	2.850433229	4.35E-05	0.0002955
**C14orf132**	9.081609588	6.696757371	2.655127192	7.44E-06	5.89E-05
**PTGER3**	84.19595195	6.669202834	0.76638601	5.35E-18	1.66E-16
**AC099796.1**	8.857962769	6.657712532	2.647738427	7.92E-06	6.22E-05
**MAFB**	202.1803939	6.638355471	1.084896118	4.47E-11	7.35E-10
**AL391001.1**	27.84937629	6.61013341	1.343852055	7.49E-09	9.37E-08
**NCMAP**	8.531035181	6.606702055	2.652803408	1.04E-05	8E-05
**NAV2-AS2**	8.772455401	6.587242848	2.669029339	1.3E-05	9.76E-05
**LINC00842**	8.564016249	6.580402957	2.643377263	1E-05	7.73E-05
**GSTM3**	43.87737281	6.540783024	1.02810359	2.33E-11	4E-10
**ENSG00000288013**	8.641630562	6.528696471	2.728773168	2.91E-05	0.0002051
**CASQ1**	8.505880587	6.521896636	2.666131104	1.58E-05	0.0001169
**ENSG00000286236**	43.16218089	6.510289791	1.096552282	3.17E-10	4.77E-09
**WNK2**	265.2515009	6.487314943	1.071983611	8.66E-11	1.38E-09
**MPDZ**	25.21954734	6.457234192	1.493133946	3.25E-07	3.25E-06
**ENPP1**	279.0481067	6.442638842	0.468450357	3.73E-43	4.2E-41
**CSF1R**	713.7985845	6.432018594	0.405615809	6.69E-58	1.36E-55
**ENSG00000287492**	9.307937052	6.377624298	2.907167128	0.0001357	0.0008211
**HAPLN1**	7.799508238	6.358510583	2.712601526	4.3E-05	0.0002926
**COL14A1**	268.1148419	6.352886932	0.817754563	1.95E-15	4.9E-14
**NPY1R**	84.37973531	6.344889227	0.723432263	7.92E-19	2.64E-17

**AL161637.1**	9.434848655	6.299191146	2.989713766	0.00023278	0.00133829
**SLAMF6**	37.79711046	6.291251702	1.026480429	8.52E-11	1.36E-09
**PPFIBP2**	512.4899005	6.255909645	0.305475323	2.33E-93	1.59E-90
**BCL11A**	34.93764663	6.244439058	1.053442685	4.39E-10	6.49E-09
**AC092447.5**	7.187984363	6.235543477	2.653109316	3.71E-05	0.00025636
**NRP2**	66.1848387	6.204244695	0.799664035	1.7E-15	4.29E-14
**BFSP2-AS1**	6.951585936	6.160741545	2.635403612	3.96E-05	0.00027184
**ZNF254**	76.29690862	6.156767158	0.71447332	2E-18	6.43E-17
**IPO5P1**	46.09734018	6.154471731	0.874576754	1.07E-12	2.1E-11
**A2MP1**	6.845964019	6.149366615	2.639403864	4.32E-05	0.00029353
**AC092634.4**	6.800694876	6.118529763	2.633704814	4.46E-05	0.00030203
**ENSG00000287388**	7.153828159	6.116259245	2.725499759	9.02E-05	0.00057104
**AC027312.1**	6.970534474	6.099834355	2.682664693	6.91E-05	0.00044971
**HMGN5**	7.119844319	6.099418253	2.692239362	7.27E-05	0.00047113
**AP002833.3**	30.67634821	6.069230705	1.040510516	1.1E-09	1.55E-08
**AC008592.5**	6.669397378	6.057625928	2.658022351	6.56E-05	0.00043031
**AL392046.2**	6.613487955	6.055223241	2.668514857	7.26E-05	0.00047046
**SEMA6A**	418.3617767	6.038283086	0.831336846	8.73E-15	2.07E-13
**ENSG00000287364**	41.24496535	6.033878481	0.884015247	5.93E-12	1.08E-10
**KRT1**	507.3640726	6.023736094	0.381686693	3.56E-55	6.51E-53
**KCNE4**	6.713491384	6.012397381	2.66908661	7.87E-05	0.00050601
**TRPC6**	6.428498145	6.00085142	2.648437974	7.22E-05	0.00046784
**COL4A4**	482.904713	5.965864454	0.326939511	1.65E-72	6.16E-70
**AL445430.1**	6.589914575	5.956217962	2.703760668	0.00011723	0.00072074
**NSG1**	7.186412275	5.933721562	2.895234476	0.00033200	0.00183711
**MAP2K6**	238.0226223	5.932574721	0.385966122	1.9E-54	3.37E-52
**GLDC**	339.4222431	5.920339274	0.36081636	1.11E-60	2.59E-58
**THAP12P5**	6.69331938	5.899922469	2.790756166	0.00022066	0.00127225
**GSDME**	284.2058878	5.883051646	0.354424452	5.65E-63	1.5E-60
**IL17RB**	7.124345125	5.871159373	2.854195099	0.00029696	0.00166131
**DUXA**	6.275853506	5.846923281	2.732402974	0.00018433	0.0010845
**TNIP3**	5.972432717	5.834184589	2.661438956	0.00012504	0.00076370
**LINC00379**	6.02658856	5.833681257	2.665622333	0.00012746	0.00077689
**AC009558.2**	6.084194108	5.833657416	2.653963454	0.00011599	0.00071459
**AC068134.1**	6.077997178	5.828610322	2.685296355	0.00014602	0.00087595

**DCN**	715.9166119	5.824359452	0.299344248	3.68E-87	2.2E-84
**GABRG1**	5.956231404	5.815407011	2.659700125	0.00012881	0.00078429
**MBNL3**	889.9585255	5.790806875	0.292044857	2.57E-89	1.62E-86
**GABRP**	56.59391248	5.780821914	0.741444959	4.54E-15	1.11E-13
**AC010745.4**	5.851718044	5.758166154	2.665458638	0.00015350	0.0009171
**ST3GAL1**	454.4033286	5.738173658	0.420725833	2.67E-42	2.92E-40
**LINC02438**	5.715332172	5.736452857	2.659236088	0.00015780	0.00094117
**EDN3**	6.08706537	5.732500366	2.771143105	0.00028815	0.00161808
**LINC01681**	5.69573861	5.726069289	2.652482648	0.00015423	0.00092103
**AC078785.2**	6.029335786	5.723230977	2.731838256	0.00023773	0.00136412
**CCR2**	15.29448618	5.722880457	1.4155258	1.68E-06	1.5E-05
**AFF3**	99.75582174	5.711971887	0.532115032	1.4E-26	7.73E-25
**KCNT2**	24.28107921	5.707244871	1.097722432	3.55E-08	4.12E-07
**ENSG00000286258**	5.976818612	5.706446624	2.74980736	0.00027311	0.00154157
**SMAD9**	44.42822654	5.691601529	0.92686744	3.32E-10	4.98E-09
**ENSG00000287430**	5.994243391	5.687970671	2.72695083	0.00024713	0.00141159
**PTHLH**	6.562070211	5.686110704	2.853204497	0.00041669	0.00225971
**C5orf46**	20.22946007	5.685858242	1.596243602	5.05E-06	4.12E-05
**CABLES1**	72.46207537	5.675638725	0.778326692	8.52E-15	2.03E-13
**SEMA3E**	15.56322977	5.675217924	1.554532797	9.7E-06	7.49E-05
**PCAT14**	6.096945717	5.672217995	2.797326117	0.00035844	0.00197245
**ZNF723**	5.567775906	5.631825203	2.706238625	0.00026316	0.00149138
**AC008938.1**	57.3129344	5.618381568	0.685739827	4.33E-16	1.14E-14
**AC092506.1**	5.488995582	5.615499805	2.684737243	0.00024318	0.00139202
**SDK1**	522.3461817	5.610081383	0.254651593	4.83E-108	4.81E-105
**FREM2**	891.2365993	5.571161623	0.252295827	6.41E-107	6.13E-104
**HOXC13-AS**	30.43308203	5.569363473	0.961999386	2.79E-09	3.72E-08
**PRKAR2B**	534.0505778	5.568913005	0.750664159	3.15E-15	7.82E-14
**ZNF703**	140.5678994	5.544778667	0.439965755	1.4E-37	1.25E-35
**AC092329.4**	74.06039864	5.541946698	0.603591633	2.93E-20	1.09E-18
**CDH10**	607.4574862	5.540908243	0.431879394	3.6E-39	3.48E-37
**RN7SL359P**	6.00652234	5.535134891	2.8217609	0.00048917	0.00260551
**C11orf44**	30.18590104	5.530837828	0.936966372	1.51E-09	2.08E-08
**AC091987.1**	6.419372499	5.522665629	2.985158339	0.00084253	0.00422240
**CYP1A2**	5.269849568	5.510796194	2.666832114	0.00027569	0.00155468

**LINC01859**	5.262013969	5.501209389	2.691151923	0.00031960	0.00177549
**MOXD1**	824.2733072	5.490210306	0.263941401	1.24E-94	8.75E-92
**FMO5**	21.89043899	5.448436668	1.118140498	1.34E-07	1.42E-06
**LINC00836**	7.477005749	5.447466966	3.273043917	0.00155050	0.00721960
**AC104435.2**	12.94332922	5.445302988	1.407251789	4.1E-06	3.4E-05
**ZNF66**	116.6970289	5.429893115	0.484885259	1.36E-30	9.28E-29
**CTAGE3P**	14.00594533	5.40139212	1.473983501	7.57E-06	5.97E-05
**MS4A7**	5.056967065	5.400855142	2.709962448	0.00043624	0.00235288
**SHROOM2**	20.89614469	5.400054624	1.115570587	1.64E-07	1.72E-06
**AL158847.1**	5.41231746	5.363807003	2.803564013	0.00063470	0.00329122
**IQGAP2**	12.0323735	5.344583054	1.436288037	8.25E-06	6.45E-05
**SNTG1**	4.916658831	5.335007732	2.68133157	0.00043017	0.00232524
**HNRNPCP6**	13.35828158	5.324196248	1.679763364	6.64E-05	0.00043483
**CYP46A1**	5.397869727	5.318064274	2.80703065	0.00068144	0.00350469
**SMAD6**	54.33597696	5.284631689	0.634473904	6.47E-17	1.82E-15
**LINC00885**	62.73735625	5.282889855	0.619898267	6.61E-18	2.03E-16
**MGAT3**	119.4137637	5.253333833	0.667010238	6.02E-17	1.7E-15
**RF00019**	5.227394232	5.237005228	2.893003915	0.00104303	0.00510419
**AC010501.1**	11.36610621	5.226721519	1.421091424	1.01E-05	7.8E-05
**BMPR1B**	34.78191	5.217359322	0.89756948	1.19E-09	1.67E-08
**FOXF2**	20.84548714	5.214946276	1.197166982	9.59E-07	8.91E-06
**RPL7AP34**	4.679094392	5.207614641	2.720879063	0.00065077	0.00336580
**HPSE2**	4.830630476	5.20296552	2.779991942	0.00080613	0.00406294
**GLUL**	1166.844937	5.191164752	0.309979595	8.45E-65	2.43E-62
**AC079465.1**	5.622567647	5.179481386	2.975827173	0.00128448	0.00610818
**ENSG00000287701**	5.198789934	5.174049517	2.891360561	0.00110840	0.00537881
**AC021915.2**	4.693069467	5.163869444	2.713333196	0.00066991	0.00345359
**CRIP2**	134.0146084	5.144108304	0.499503985	5.34E-27	3.02E-25
**GPC5**	4.487965108	5.130239135	2.703999967	0.00070831	0.00362807
**ENSG00000287692**	4.608092213	5.127173785	2.734308628	0.00077909	0.00394830
**RHOU**	504.0441945	5.123155991	0.307247269	4.57E-64	1.27E-61
**ZNF676**	16.69715237	5.116450285	1.139440604	1.17E-06	1.07E-05
**AC138649.1**	11.36159628	5.112030477	1.550355903	4.44E-05	0.00030086
**TRPM6**	573.3571632	5.111022253	0.267437997	1.22E-80	5.97E-78
**AP001527.1**	4.746116754	5.110775859	2.79397208	0.00095577	0.00472550

**AC104237.2**	10.48468186	5.103158499	1.443530755	1.92E-05	0.00013932
**ZDHHC8P1**	85.80002752	5.087225469	0.568880875	5.52E-19	1.86E-17
**AC015712.2**	10.45894882	5.084296403	1.468120459	2.55E-05	0.00018148
**OR52K1**	10.95484666	5.079331214	1.419863473	1.33E-05	0.0001001
**ACTG2**	10.37344145	5.073324717	1.445362575	2.14E-05	0.00015374
**COL1A2**	2133.210453	5.060134125	0.316637913	8.4E-58	1.65E-55
**NTF3**	5.042982865	5.058652656	2.885989659	0.00125826	0.0060026
**AC064807.2**	4.485217882	5.043939057	2.778731724	0.00104202	0.00510130
**GLULP5**	10.54614319	5.039730283	1.49036319	3.42E-05	0.00023773
**MTMR8**	18.76380056	5.038744851	1.125207952	5.06E-07	4.92E-06
**ZNF43**	49.74619429	5.021215917	0.676926296	1.26E-14	2.93E-13
**DHRS9**	4.554523937	5.020672688	2.83230918	0.00124817	0.00596282
**CLMN**	505.7929749	4.998208599	0.335014169	6.7E-51	1.02E-48
**LINC00456**	21.9566665	4.991202363	0.980417722	8.3E-08	9.08E-07
**S1PR3**	252.0842035	4.986293736	0.30423465	4.82E-60	1.11E-57
**ZNF727**	16.78395032	4.982566352	1.111292771	7.57E-07	7.14E-06
**PRKAA2**	71.69728365	4.968642794	0.691645916	2.17E-14	4.97E-13
**CPVL**	96.06061701	4.962205944	0.578979614	6.64E-20	2.39E-18
**FAM43B**	29.95174828	4.94300295	0.882161553	2.51E-09	3.38E-08
**ENSG00000286721**	10.4048507	4.931387208	1.456237444	2.83E-05	0.00019993
**ENSG00000285634**	4.423102403	4.928879465	2.782915407	0.00121568	0.00582504
**COL5A1**	1055.554785	4.921898268	0.795988069	4.19E-11	6.91E-10
**RN7SKP276**	4.86132785	4.892447122	2.943327331	0.00175289	0.00803365
**TRPC3**	9.334513902	4.890124795	1.452256271	3.95E-05	0.00027108
**CPEB2-AS1**	4.163254633	4.874230551	2.734186712	0.00118150	0.00568634
**RASSF6**	116.7947329	4.871774831	0.514456342	9.65E-22	4.04E-20
**GPR155**	35.44741277	4.865082672	0.828471361	3.45E-10	5.16E-09
**AC124856.1**	4.363915641	4.864457443	2.802924956	0.00140200	0.00660401
**AUTS2**	19.00320032	4.85648905	1.190424601	1.54E-06	1.39E-05
**EDNRB**	610.4729944	4.85338161	0.238525728	3.14E-93	2.09E-90
**SLC9A2**	20.2229914	4.852066509	0.974503622	1.47E-07	1.55E-06
**ALPPL2**	31.3120962	4.845436092	0.793538156	2.7E-10	4.09E-09
**CYP11A1**	9.619564595	4.837648201	1.543147852	8.75E-05	0.00055551
**OTOGL**	255.9570417	4.837331622	0.380463944	1.29E-38	1.22E-36
**HS6ST2**	4.086700547	4.833243118	2.777939241	0.00144174	0.00677250

**PIK3AP1**	1578.017534	4.831622404	0.344125416	1.35E-46	1.73E-44
**NANOGP4**	9.809064604	4.831312395	1.459656895	3.93E-05	0.00027045
**AC093534.2**	4.540539737	4.821758903	2.8969276	0.00179187	0.00818723
**COL18A1**	56.58713487	4.813219615	0.621243589	5.4E-16	1.42E-14
**AC010267.1**	4.760621942	4.802784647	3.041379023	0.00235765	0.01041794
**SMPDL3A**	401.2727002	4.795769255	0.28684974	2.53E-64	7.11E-62
**OR8R1P**	4.575747043	4.790085153	2.927479966	0.00195788	0.00885100
**PRKAG2**	100.0093116	4.788906941	0.61300382	2.94E-16	7.88E-15
**ALDH5A1**	272.6307325	4.782841067	0.289244799	7.09E-62	1.75E-59
**ANKRD6**	83.25528275	4.77284073	0.480715074	2E-23	9.22E-22
**HSPD1P11**	389.6069272	4.7671303	0.286512832	1.47E-62	3.78E-60
**GSTA3**	4.325258631	4.760426508	2.897684119	0.00198538	0.00895499
**SLC24A3**	30.38493031	4.753497811	0.781481152	2.36E-10	3.61E-09
**HUNK**	476.6787703	4.746971682	0.328241892	4.48E-50	6.53E-48
**PDZD2**	4748.086137	4.745944672	0.296891097	2.1E-58	4.37E-56
**FOXO6**	10.52184879	4.736483579	1.524661169	5.21E-05	0.00034770
**SCARA5**	13.73037681	4.734155972	1.245348378	1.84E-05	0.00013391
**ALOX5**	15.11893625	4.726211812	1.180979993	5.32E-06	4.32E-05
**SCN11A**	386.2722583	4.722554723	0.406471484	4.34E-32	3.17E-30
**MSC-AS1**	14.6601236	4.721436675	1.144036287	3.5E-06	2.94E-05
**LINC02476**	17.70842522	4.701371201	1.010573536	8.36E-07	7.82E-06
**LY9**	3.912881131	4.694408004	2.7757124	0.00173212	0.00795833
**IQCA1**	4.019090616	4.68684916	2.83397907	0.00196425	0.00887476
**AC020914.1**	4.07160779	4.682188901	2.822314827	0.00189826	0.00861405
**COL21A1**	28.86635456	4.679703434	0.819822614	2.43E-09	3.28E-08
**PCSK5**	13.30584466	4.668617765	1.1504301	6.17E-06	4.95E-05
**AC005162.3**	9.180875884	4.664381374	1.497039081	8.07E-05	0.00051677
**DEPTOR**	51.31109307	4.659582094	0.704953748	2.76E-12	5.2E-11
**SLAMF7**	97.16092819	4.650101366	1.238836473	7.73E-06	6.08E-05
**AC092279.1**	252.6920051	4.64282175	0.281363061	1.36E-61	3.28E-59
**AC107398.3**	3.991073887	4.624043636	2.834936347	0.00209547	0.00938431
**FBN2**	5802.8319	4.603004847	0.333010794	1.94E-44	2.29E-42
**GAS1**	130.0862481	4.594307418	0.377896268	6.66E-34	5.25E-32
**AL160153.1**	8.264234828	4.575985964	1.554721709	0.00018208	0.00107182
**CR381670.1**	13.11470625	4.570820862	1.186546146	1.29E-05	9.69E-05

**ENSG00000287172**	12.73795126	4.570054526	1.160639725	9.88E-06	7.63E-05
**NEBL**	1528.957052	4.568537453	0.311434456	5.39E-50	7.76E-48
**ZNF728**	16.31566191	4.562777427	1.019509903	1.81E-06	1.61E-05
**GOLGA8N**	8.244641266	4.558701053	1.561346388	0.00019582	0.00114284
**ATP10A**	12.41323139	4.554840571	1.223355154	2.57E-05	0.00018297
**AMOT**	2191.74766	4.546533244	0.301965505	1.44E-52	2.37E-50
**WISP1**	3.713916247	4.546266242	2.814840487	0.00230544	0.01021746
**ZNF491**	7.56780028	4.506550755	1.51081801	0.00017201	0.00101705
**RHOBTB3**	11826.53094	4.502530336	0.538170376	4.6E-18	1.43E-16
**AC005324.1**	4.769681008	4.498668938	3.232967512	0.0037276	0.01542522
**AC010387.1**	4.263845631	4.495351567	3.02187255	0.00310132	0.01316865
**RAB39B**	22.51510897	4.493008795	0.90226958	8.61E-08	9.4E-07
**CR381653.2**	9.49735985	4.483615982	1.543196657	0.00010769	0.00066950
**AC096721.1**	3.703796955	4.469852791	2.847054397	0.00264403	0.01146723
**ZNF681**	53.58233318	4.464901199	0.52876255	8.23E-18	2.51E-16
**GABRB3**	17.44882632	4.455039093	1.021317038	1.48E-06	1.34E-05
**HPRT1P1**	8.012174595	4.429849131	1.531659937	0.00018527	0.00108922
**AC002472.2**	7.610255616	4.429239747	1.559577931	0.00026467	0.00149890
**DUSP9**	42.23433479	4.427897892	0.609342852	1.88E-13	3.94E-12
**AC139769.3**	3.556298144	4.411514039	2.821315508	0.00274200	0.01184058
**NALCN**	926.6111401	4.397188101	0.225396816	1.11E-85	6.03E-83
**KRT73**	3.641805512	4.377221573	2.93005682	0.00336308	0.01412956
**GPR27**	3.749711121	4.362898117	2.995904591	0.00367263	0.01523998
**AL691403.1**	3.938026599	4.352727748	2.993813	0.00351437	0.01468396
**SOX6**	10.70308134	4.346410004	1.248448998	6.51E-05	0.00042763
**RN7SKP245**	3.839131726	4.340074004	3.009178749	0.00371967	0.01540041
**SLC2A5**	262.2103472	4.33805959	0.763448361	6.2E-10	9.01E-09
**ABCA4**	30.03385183	4.336548113	0.920865285	2.18E-07	2.25E-06
**AC006504.1**	10.58610754	4.330048038	1.221256699	5.33E-05	0.00035503
**SOWAHA**	69.06190293	4.326283319	0.479019861	9.02E-21	3.53E-19
**AC063944.1**	210.6772365	4.325693764	0.365089098	7E-33	5.33E-31
**FOXO4**	897.1129391	4.325297608	0.328136182	6.9E-41	7.08E-39
**AC237221.1**	24.15421064	4.3252147	0.973538484	3.32E-07	3.31E-06
**ITIH5**	71.40695033	4.325095794	0.452581882	3.81E-22	1.64E-20
**ZNF257**	106.0629675	4.320761533	0.385358166	2.75E-30	1.84E-28

**AC107884.1**	50.41529543	4.306411141	0.566372739	1.03E-14	2.43E-13
**PTCHD4**	44.02787414	4.304091157	0.559041667	4.36E-15	1.07E-13
**MAFTRR**	3.540039377	4.298716122	2.905360833	0.00350322	0.01464116
**AC099520.1**	3.431082666	4.289990524	2.872492791	0.00343645	0.01439734
**OVCH2**	111.2537321	4.285529093	0.391858217	6.11E-29	3.78E-27
**COL3A1**	87.46672584	4.281327606	0.551904337	1.59E-15	4.02E-14
**AC004593.2**	7.307489244	4.280772766	1.524889551	0.00025856	0.00146954
**AC007182.1**	3.785439415	4.276400758	2.981973485	0.00376393	0.01555404
**EGFEM1P**	22.92640044	4.27607733	0.846100698	6.4E-08	7.14E-07
**CD69**	14.87918301	4.264075184	1.009979766	3.35E-06	2.83E-05
**MAN1A1**	36.08028774	4.226593828	0.624826596	2.42E-12	4.6E-11
**MT-TM**	7.127530354	4.210532766	1.541899001	0.00032972	0.00182576
**METTL7A**	24.93370991	4.208353104	0.856356612	4.83E-08	5.48E-07
**GRIK4**	3.398680041	4.206843249	2.872650822	0.00369497	0.01532204
**PNPLA3**	12.88300529	4.201599644	1.36247338	0.00010312	0.00064427
**MS4A12**	3.562380164	4.201390515	2.98475699	0.00419101	0.01703912
**YWHAEP3**	3.463542746	4.198204105	2.969799822	0.00422719	0.01716303
**MGMT**	81.40729976	4.193315302	0.465547537	4.29E-21	1.7E-19
**OR5P1P**	25.86256493	4.192792175	0.812360209	2.74E-08	3.23E-07
**SNURF**	4.324785973	4.185263386	3.31918018	0.00510649	0.02014126
**PNPLA4**	20.98070306	4.175126598	0.820037495	7E-08	7.74E-07
**RN7SL810P**	39.66997917	4.168570264	0.591136499	6.11E-13	1.23E-11
**AL122017.1**	9.665668426	4.164964915	1.213475538	8.18E-05	0.00052366
**ENSG00000287481**	3.433887347	4.163582973	2.910031389	0.00400012	0.01636217
**TGM6**	3.697069911	4.163171803	3.064275656	0.00458850	0.01841408
**AC015920.1**	7.746120769	4.159862792	1.669809881	0.00059105	0.00309372
**AC008554.1**	3.291362	4.152022995	2.904561592	0.00415391	0.01690554
**FHIT**	277.7022403	4.1474203	0.325314195	1.18E-37	1.07E-35
**TMEM131L**	105.6329336	4.146745624	0.454088958	6.08E-21	2.4E-19
**CACNA1C**	60.23824242	4.143511859	0.49613904	1.52E-17	4.51E-16
**AC092634.3**	3.794913684	4.137271691	3.104363992	0.00475778	0.01896363
**ENPP2**	48.28434063	4.13706419	1.506602795	0.00030572	0.00170672
**COLCA1**	3.772515441	4.136658382	3.125930705	0.00490055	0.01943800
**SLIT2**	31.16059362	4.132621196	0.732668242	5.46E-09	6.96E-08
**AL139002.1**	179.5676641	4.123348841	0.419094643	6.49E-23	2.89E-21

**GLYAT**	6.803349992	4.117042135	1.571859359	0.00046988	0.00251226
**ZNF680**	152.3053706	4.107726517	0.341745387	6.88E-35	5.63E-33
**MAP6**	15.19937629	4.104860916	0.936491797	3.52E-06	2.96E-05
**SMIM3**	72.74933437	4.103919149	1.365454591	0.00017044	0.00100877
**GPR143**	7.046466337	4.101705747	1.580121496	0.00048183	0.00257213
**IL12RB2**	178.2311633	4.100592591	0.428591206	6.23E-22	2.65E-20
**AC092427.1**	61.60983362	4.094227505	0.48104932	4.15E-18	1.3E-16
**CRYAB**	1176.286666	4.092142709	0.284974558	1.09E-47	1.49E-45
**FAM47E**	16.50336455	4.091894799	0.934097959	2.31E-06	2.02E-05
**MBNL1-AS1**	58.9466135	4.080517479	0.483386121	5.17E-18	1.6E-16
**VWDE**	82.17708057	4.069049741	0.530009499	3.61E-16	9.62E-15
**CEBPA**	178.1188646	4.063545813	0.346783296	4.82E-32	3.5E-30
**LINC01209**	3.376281799	4.055068855	3.0202527	0.00502679	0.01988800
**Z84485.1**	16.65992243	4.047810879	0.993096592	7.21E-06	5.73E-05
**ENSG00000286213**	6.863048348	4.0461121	1.763316472	0.00123668	0.00591536
**AC010183.1**	11.52160289	4.045316058	1.248729988	6.72E-05	0.00043910
**HRASLS**	3.270129768	4.043478268	2.921952859	0.00461392	0.01850366
**PSD2**	6.387919472	4.041389431	1.656554129	0.00091384	0.00453979
**RNU6-880P**	4.302445185	4.04065239	3.463268506	0.00603596	0.02324742
**PDGFA**	15.1604375	4.038132097	0.977708713	7.91E-06	6.22E-05
**ROBO2**	13.06699446	4.037611587	1.125037617	4.32E-05	0.00029353
**EFHD1**	472.2709395	4.036834601	0.413879149	1.49E-23	6.94E-22
**C9orf170**	14.73149544	4.034882579	0.991426004	1.14E-05	8.7E-05
**SCIN**	2043.0501	4.02536963	0.554830277	3.26E-14	7.37E-13
**TCIM**	6.749715136	4.02188865	1.597189661	0.00061670	0.00320692
**ROR1**	247.5048932	4.020497054	0.854175424	2.06E-07	2.13E-06
**LINC01951**	3.729472536	4.004653139	3.259813729	0.00597368	0.02304837
**AC025458.1**	6.599875177	3.988651196	1.580343616	0.00061755	0.00321063
**CYP4F23P**	3.133743896	3.988185191	2.919441808	0.00495106	0.01961488
**AC044810.2**	110.4558877	3.982542918	0.389174418	4.36E-25	2.22E-23
**HNRNPA1P33**	3.111403109	3.977793558	2.924917279	0.00505299	0.01995985
**ALG10B**	240.9700185	3.977159256	0.280994088	2.34E-46	2.98E-44
**AC091563.1**	5.904018804	3.956138462	1.644902772	0.00106609	0.00520210
**PLCE1**	8.611657245	3.952269237	1.308219579	0.00030133	0.00168339
**TMEM254-AS1**	78.74692422	3.937712548	0.418542222	1.23E-21	5.14E-20

**TPRG1LP1**	8.958766231	3.936238746	1.211352423	0.00013050	0.00079357
**MIR483**	3.578581476	3.935169768	3.242055962	0.00628726	0.02405690
**ADAMTS5**	6.815098828	3.930549519	1.665385892	0.00091176	0.00453135
**ALPP**	1163.311044	3.930289208	0.23619168	1.38E-62	3.59E-60
**AL451064.2**	5.861439432	3.927885901	1.636848028	0.00107989	0.00525978
**HRK**	200.418789	3.922720161	0.372727376	8E-27	4.46E-25
**ZNF85**	74.72379592	3.918008118	0.499132606	2.81E-15	6.99E-14
**CLDN8**	26.30845452	3.913700584	0.74428893	2.6E-08	3.08E-07
**EPB41L1**	379.6639841	3.909927641	0.210898327	2.27E-77	9.51E-75
**LGI2**	28.30156096	3.900053816	0.723157374	1.48E-08	1.79E-07
**DSC1**	66.07361208	3.898437239	0.494958375	2.17E-16	5.89E-15
**WNT5A**	19.48982184	3.898030502	0.796153861	2.33E-07	2.39E-06
**MIR4636**	3.22819542	3.885914629	3.012543625	0.00572038	0.02219281
**SULT2A1**	9.156507648	3.885025674	1.342715746	0.00034961	0.00192830
**SLC16A12**	8.608207541	3.883517077	1.407695948	0.00059956	0.00313141
**XAGE2**	205.9247945	3.880046418	0.283092312	1.94E-43	2.22E-41
**ARSD**	3.353998466	3.870334767	3.140694731	0.00630853	0.02411850
**BCAT1**	366.8095436	3.869215259	0.307902521	1E-36	8.83E-35
**AC060834.1**	11.23199394	3.865644912	1.365647193	0.00021810	0.00126026
**TMEM191B**	15.73686776	3.863175796	0.91107393	5.97E-06	4.81E-05
**SPRY3**	142.5931401	3.858636052	1.296019165	0.00017072	0.00101015
**MIR550A2**	5.699435433	3.858547508	1.640585897	0.00123910	0.00592538
**FAM78A**	77.03865504	3.855443244	0.496428943	2.2E-16	5.96E-15
**ATP1A4**	9.455266542	3.855278711	1.234259365	0.00013835	0.00083577
**HOXC13**	41.17099841	3.855097606	0.628802003	1.16E-10	1.83E-09
**AC084759.3**	8.6837014	3.852962543	1.37326522	0.00051683	0.00273818
**KCNC4**	97.24863925	3.851628793	0.365366833	1.22E-26	6.77E-25
**CYP2J2**	354.0830409	3.849784978	1.05374304	1.35E-05	0.00010106
**CYP7B1**	21.3781498	3.846843643	0.797809854	1.56E-07	1.65E-06
**DNM3**	51.48217146	3.844862337	0.597682532	2.61E-12	4.93E-11
**AC116049.2**	3.130409102	3.844672225	2.997214194	0.00592748	0.02288860
**VAV3**	1034.443574	3.844056096	0.287444022	9.54E-42	1.01E-39
**F5**	45.13267903	3.840266966	0.801309754	2.67E-07	2.72E-06
**IGHE**	3.023091061	3.839624105	2.990195393	0.00607518	0.02337211
**HOMER1**	291.8774537	3.837099359	0.344370046	4.9E-31	3.39E-29

**LINC01905**	3.018060142	3.831788079	2.959012773	0.00591957	0.02287283
**LINC01814**	26.03519737	3.827982844	0.84364295	7.02E-07	6.67E-06
**CR381653.1**	26.32051316	3.820215123	0.683486451	5.91E-09	7.48E-08
**FPR3**	12.89167007	3.817229876	1.136757842	7.13E-05	0.00046248
**AC078906.1**	8.352793995	3.810066041	1.345351421	0.00049973	0.00265641
**TSPAN12**	114.8729255	3.806638837	0.429785925	6.41E-20	2.31E-18
**AC023824.6**	10.6189965	3.798613643	1.039642597	4.34E-05	0.00029514
**AC089998.3**	3.053267448	3.797250871	2.99031746	0.00617890	0.02371008
**AC063944.2**	25.2987057	3.774998918	0.892593936	7.59E-07	7.16E-06
**CYB5A**	6.193512848	3.773239554	1.855341742	0.00237875	0.01049178
**AL138999.1**	6.413189243	3.771737644	1.699571407	0.00133786	0.00633432
**AL445430.2**	10.09239216	3.766194003	1.101375223	0.00010756	0.00066885
**LY86-AS1**	6.701780515	3.76484377	1.614440595	0.00071024	0.00363481
**DPPA2**	427.3304317	3.759479632	0.226872559	1.03E-61	2.51E-59
**LINC02541**	87.99438094	3.757209864	0.52585259	1.55E-14	3.59E-13
**AC244258.1**	5.394203609	3.753663174	1.676011932	0.00168909	0.00779053
**PINCR**	15.34829938	3.74609812	1.035301186	2.95E-05	0.00020754
**BMP6**	274.4224977	3.733541312	0.352995948	2.74E-27	1.57E-25
**OLR1**	1315.516749	3.730242686	0.262940241	3.63E-46	4.56E-44
**ITGBL1**	218.5104503	3.726458275	0.336994603	4.37E-30	2.87E-28
**LINC01483**	16.73972657	3.72452074	0.908079595	9.66E-06	7.46E-05
**RIMKLB**	597.0057766	3.723828048	0.216982204	1.65E-66	5.26E-64
**SYNE3**	97.25680869	3.722223741	0.459965427	6.3E-17	1.78E-15
**CPS1**	425.266701	3.721879754	0.220529543	5.81E-64	1.6E-61
**GAS1RR**	22.19436523	3.721030476	0.792780936	3.37E-07	3.36E-06
**RNU6-371P**	3.019756267	3.720975969	3.048804711	0.00687367	0.02590983
**ATP7B**	570.5991447	3.705780523	0.335341402	1.41E-29	9.07E-28
**ECEL1P2**	25.87014236	3.702625097	0.808907254	2.3E-07	2.36E-06
**FSTL4**	36.25733275	3.702501452	0.64545711	9.04E-10	1.28E-08
**SNORD123**	2.999054149	3.699279293	3.102939022	0.00730201	0.02729692
**CLCA2**	244.8398217	3.696787437	0.339647115	4.65E-28	2.76E-26
**SEMA5A**	3522.954935	3.691241891	0.173007175	1.59E-102	1.27E-99
**TNPO1P3**	37.70232662	3.691199923	0.581905063	2.3E-11	3.95E-10
**SLC40A1**	5.105288008	3.689924814	1.641840639	0.00173145	0.00795677
**NNMT**	49.43274607	3.689261284	0.569410675	2.54E-11	4.33E-10

**RFESD**	273.6684043	3.688717848	0.245129125	2.1E-52	3.44E-50
**LIFR**	655.4272379	3.684695036	0.305945898	1.99E-34	1.6E-32
**LRMP**	18.70103972	3.684540872	1.057177317	4.24E-05	0.00028900
**WISP2**	10.94029621	3.682267899	1.371291249	0.00072311	0.00369597
**AC009955.3**	5.864310961	3.678504592	1.67625064	0.00153066	0.00714391
**OVAAL**	7.69677943	3.674581945	1.247813605	0.0003568	0.00196387
**SPARC**	7.579936659	3.669178285	1.811236139	0.00115132	0.00555898
**SNED1**	7.362412927	3.660973693	1.296466258	0.00057282	0.00300551
**HOTAIR**	2.894540789	3.660103987	3.047982214	0.00734580	0.02742057
**TTC23L**	86.3026693	3.658980697	0.585920046	2.53E-11	4.3E-10
**FPGT-TNNI3K**	5.64920222	3.658222639	1.622929584	0.00137767	0.00650215
**AL354714.4**	2.905711182	3.657016106	3.052129584	0.00735847	0.02744363
**AC007325.2**	2.905711182	3.657016106	3.052129584	0.00735847	0.02744363
**ZNF675**	107.3441367	3.655788254	0.406644016	3.06E-20	1.14E-18
**ADAM12**	32.9614807	3.650164935	0.742412227	1.05E-07	1.13E-06
**RNU4ATAC16P**	2.952088881	3.64631451	3.066937293	0.00739090	0.02753467
**CLEC7A**	29.10342206	3.640700525	0.6711946	1.8E-08	2.16E-07
**MIR548X**	2.896236913	3.64048557	3.063575761	0.00751813	0.02795188
**AL110292.1**	152.5667979	3.63509889	0.335474249	1.58E-28	9.64E-27
**ZNF138**	72.37784551	3.6348852	0.514875037	7.42E-13	1.48E-11
**A2M-AS1**	9.057197839	3.634841407	1.370639113	0.00058119	0.00304679
**POM121L7P**	3.430552552	3.629588641	3.360394859	0.00796785	0.02934555
**PZP**	2.955481131	3.625478714	3.083258684	0.00757006	0.02812527
**MEF2C**	8.817994731	3.601973036	1.360063325	0.00060053	0.00313374
**DOCK11**	25.00201864	3.601290904	0.721891196	3.95E-08	4.54E-07
**LINC00987**	6.345197528	3.598515085	1.65247639	0.00105947	0.00517547
**TMPRSS13**	20.24664872	3.591134905	0.899164354	1.19E-05	9.06E-05
**AC002044.1**	5.218745524	3.5907839	1.725664897	0.00244865	0.01075053
**AC046130.2**	2.867169082	3.588688061	3.062999867	0.00778827	0.02879504
**ALDH3B2**	5.397132594	3.58839616	1.621231534	0.00157608	0.00733153
**AC098613.1**	42.269431	3.585386746	0.753517103	1.24E-07	1.33E-06
**CYP4B1**	14.42569912	3.583507445	0.900942108	1.22E-05	9.22E-05
**AC010183.2**	9.819735589	3.580972308	1.109353401	0.00016958	0.00100388
**ACPP**	2.902376388	3.575350761	3.078273169	0.00785322	0.02898589
**ZNF91**	432.0005056	3.568054027	0.212324724	9.18E-65	2.61E-62

**XAGE3**	99.28695393	3.567458504	0.438275765	3.35E-17	9.67E-16
**GREB1L**	156.0520323	3.565720759	0.317932825	9.45E-30	6.1E-28
**TBC1D4**	210.3880554	3.556900905	0.276506401	6.26E-39	5.99E-37
**MIR4307**	16.92725472	3.542865406	0.837397565	3.33E-06	2.82E-05
**AC100802.1**	13.74394939	3.53651211	1.000821849	3.79E-05	0.00026148
**ZNF738**	68.87768045	3.529539453	0.415242869	7.65E-18	2.33E-16
**STAT4**	70.27892482	3.520896965	0.441646493	1.88E-16	5.14E-15
**MUC15**	409.0830951	3.515067385	0.24809589	1.31E-46	1.69E-44
**AC005162.2**	2.790027428	3.511506694	3.077383231	0.00836949	0.03053320
**CHN2**	16.76810735	3.505409053	0.826048029	3.1E-06	2.64E-05
**C10orf25**	2.9264133	3.477998956	3.167707956	0.00868127	0.03145941
**FCRLB**	25.25117483	3.473884938	0.846712264	4.94E-06	4.03E-05
**MIR4307HG**	241.6066529	3.472591263	0.248537884	2.82E-45	3.44E-43
**AC009902.3**	7.97177768	3.466895935	1.33925055	0.00076713	0.00389514
**AL391840.1**	4.668228573	3.465193813	1.684038263	0.00282474	0.01215616
**AC005476.2**	26.44761347	3.463614727	0.740298713	4.71E-07	4.61E-06
**AC108097.1**	18.29416886	3.460214623	0.777917514	1.32E-06	1.2E-05
**ID2**	618.9403158	3.452590936	0.282562221	1.15E-35	9.68E-34
**DTX4**	491.5954249	3.449146831	0.259546729	3.86E-41	4.01E-39
**LINC01554**	26.82556063	3.448157591	0.623400763	8.44E-09	1.05E-07
**GLIS3**	25.03901184	3.443639502	0.72062674	3.37E-07	3.36E-06
**TCAF2P1**	6.673878696	3.436868811	1.772456729	0.00160247	0.00743406
**XCR1**	185.8517576	3.432812278	0.407750432	6.77E-18	2.08E-16
**TGFBI**	2719.322046	3.429980953	0.650450291	1.18E-08	1.45E-07
**AC100860.1**	31.80245812	3.419796245	0.669280062	1.28E-08	1.56E-07
**PPM1L**	7.914425978	3.417362863	1.363979405	0.00079689	0.00402574
**ZNF724**	40.3335465	3.409048217	0.510362628	2.42E-12	4.6E-11
**KIRREL3**	45.24138527	3.402080495	0.55629687	2.62E-10	3.97E-09
**FOS**	236.2747085	3.398387845	0.411470365	7.38E-18	2.25E-16
**KIF21A**	114.4004837	3.397395402	0.33264493	2.89E-25	1.48E-23
**FAM49A**	394.5249508	3.387891999	0.297675029	1.82E-30	1.23E-28
**SIPA1L2**	614.4394313	3.386756125	0.341033922	1.46E-24	7.22E-23
**CU104787.1**	24.20788957	3.386078957	0.669184621	5.6E-08	6.3E-07
**TMEM52B**	72.81530885	3.373920752	0.563292173	2.97E-10	4.48E-09
**AC017100.1**	13.57194138	3.370216305	0.9125782	4.72E-05	0.00031756

**LINC02246**	32.23110481	3.369150261	0.604828418	5.04E-09	6.46E-08
**ADGRV1**	105.9661221	3.368192295	0.383412957	1.2E-19	4.27E-18
**IGKV2OR22-3**	2.760959597	3.36370013	3.155971638	0.00952774	0.03403680
**AC115220.1**	2.823538609	3.360594306	3.216955327	0.00967009	0.03447661
**C15orf59**	4.124597623	3.358960523	4.45490093	0.00996309	0.03532813
**DLK2**	161.5362982	3.358956774	0.334897014	6.75E-25	3.39E-23
**OR5P3**	35.42092402	3.357739149	0.566280659	4.75E-10	7E-09
**RASGRF2**	17.06941533	3.347663362	0.949430145	5.72E-05	0.00037874
**ARHGAP28**	39.85359799	3.337758212	0.553879567	1.25E-10	1.95E-09
**TMEM191C**	23.58098363	3.33425224	0.644253423	4.99E-08	5.66E-07
**AC244505.1**	2.732479335	3.331780984	3.227820391	0.01010819	0.03575854
**ZNF736**	344.8874264	3.330919422	0.221073635	1.93E-51	3.04E-49
**AC114284.1**	3.049932654	3.329326384	3.382124116	0.00992828	0.03523082
**AP002833.1**	23.19731071	3.320213878	0.680287155	2.28E-07	2.35E-06
**AC026877.1**	11.82725485	3.314402439	1.036787292	0.00023539	0.00135230
**WNT8B**	9.420430392	3.310979409	1.215942445	0.00056497	0.00296892
**AL031123.1**	10.91624893	3.309800389	1.005786701	0.00013524	0.00081866
**AC073316.3**	2.784996509	3.306056486	3.211091657	0.00999714	0.03543312
**ENSG00000287291**	10.51480437	3.299744895	1.153749117	0.00030851	0.00172071
**ROR1-AS1**	2.80962099	3.299577272	3.315384987	0.01043256	0.03670972
**LINC01535**	35.20216969	3.299194571	0.618619817	2.78E-08	3.27E-07
**CCDC173**	7.104973421	3.297929161	1.3238641	0.00108879	0.00529667
**PRICKLE2**	8.110106528	3.293329045	1.112220043	0.00043294	0.00233779
**LRRK1**	80.0430958	3.288570499	0.463428886	2.85E-14	6.49E-13
**ADGRF5**	9.766414165	3.287505482	1.157957632	0.00067369	0.00346854
**MYCN**	56.05925612	3.283886538	0.587378897	2.89E-09	3.85E-08
**CD200**	294.8378927	3.282741684	0.405081326	3.27E-17	9.45E-16
**KRT17P8**	3.412655115	3.27494003	3.734841013	0.01033286	0.03640711
**LINC00645**	4.713034451	3.274389787	1.680483854	0.00314176	0.01330770
**LINC02256**	2.780553159	3.269619392	3.292879635	0.01055385	0.03707103
**MGAT4EP**	13.38140719	3.268292304	0.973460676	8.53E-05	0.00054300
**FPR1**	22.25697009	3.265249474	0.64906174	6.75E-08	7.48E-07
**BMP7**	56.05440037	3.250338088	0.442176324	3.47E-14	7.84E-13
**HSPD1P6**	2.921912495	3.244581047	3.486995378	0.01098180	0.03833772
**LINC00525**	6.059136801	3.237960133	1.447825877	0.00261975	0.01137636

**LINC00475**	18.200087	3.232931355	0.73547617	2.38E-06	2.07E-05
**ZMAT3**	1635.970464	3.227634796	0.225334286	2.57E-47	3.44E-45
**ISM1**	16.25102437	3.227460475	0.775309971	4.07E-06	3.38E-05
**RNY1P16**	4.23789618	3.225260516	1.741905308	0.00461902	0.01852103
**LIFR-AS1**	25.12467421	3.21965153	0.640107464	1.08E-07	1.16E-06
**ENSG00000286626**	7.872599544	3.218255483	1.147956148	0.00067223	0.00346249
**ENSG00000286125**	41.18050529	3.21411772	0.609235819	2.62E-08	3.09E-07
**AC016730.1**	43.30516834	3.20989684	0.511529007	2.82E-11	4.77E-10
**YWHAZP1**	2.834709003	3.206287564	3.36874841	0.01096574	0.03829841
**HEYL**	6.087559608	3.20472853	1.486936998	0.00306337	0.01302601
**NPY5R**	5.839990787	3.203275739	1.379223329	0.00225846	0.01003901
**ENSG00000286042**	7.586306865	3.201531782	1.193057624	0.00100378	0.00493531
**CFAP45**	33.39342522	3.200392918	0.547634607	9.28E-10	1.32E-08
**AC127070.2**	18.92815324	3.198772824	0.723692468	9.68E-07	8.98E-06
**POM121L8P**	4.1965494	3.192011697	1.797923252	0.00536069	0.02098121
**RAB6C**	4.948835916	3.185879921	2.011248608	0.00643948	0.02449381
**PLPP3**	113.5694352	3.179946148	0.360610949	3.44E-19	1.17E-17
**MYB**	72.42171553	3.172872353	0.462214146	4.23E-13	8.63E-12
**ERVE-1**	19.93926744	3.170630793	0.877612127	1.51E-05	0.00011201
**SLC1A6**	104.8782504	3.168803256	1.040851249	0.00013226	0.00080313
**KIAA0825**	7.80598326	3.168208131	1.172981058	0.00087051	0.00434802
**SCARB1**	1184.602284	3.164608563	0.267998307	4.1E-33	3.16E-31
**ZNF680P1**	6.69587326	3.158756522	1.371070916	0.00178159	0.00814804
**TGFBR3**	2354.184152	3.1526624	0.322025198	1.73E-23	8E-22
**SEZ6L**	4.895731173	3.151938944	2.018244003	0.00672881	0.02543652
**AC012676.4**	6.828279314	3.12874351	1.38907799	0.00195076	0.00882348
**LFNG**	44.18407405	3.121990642	0.524918299	1.95E-10	2.99E-09
**CALD1**	1369.707163	3.110718565	0.215479292	7.76E-48	1.06E-45
**LINC02298**	19.18036982	3.100048995	0.843493315	4.49E-05	0.00030343
**SEMA6A-AS2**	11.94950668	3.099869065	1.01666384	0.00037590	0.00205720
**LINC01205**	4.757252493	3.089943804	1.862991661	0.00538209	0.02105116
**GSTA4**	22.60442834	3.087672231	0.645480532	3.08E-07	3.1E-06
**KCNJ2**	498.9064615	3.084078813	0.273663825	1.85E-30	1.24E-28
**AGFG2**	137.7308954	3.077375949	0.293365225	1.1E-26	6.13E-25
**TNS1**	79.97062189	3.075093373	0.364652871	3.98E-18	1.25E-16

**AL139383.1**	72.37372701	3.072532807	0.402118262	1.57E-15	3.97E-14
**MYCL**	268.0096819	3.071897047	0.270174794	2.4E-31	1.7E-29
**KCNK12**	2.659781031	3.062638135	3.37194843	0.01221081	0.04188817
**CPS1-IT1**	5.907194908	3.045855108	1.383345927	0.00267395	0.01157810
**PHF24**	3.934417936	3.035027502	1.795633138	0.00660245	0.02501358
**RAB15**	81.36431893	3.031444617	0.371670995	4.01E-17	1.15E-15
**AC104237.3**	11.77212265	3.027465519	1.11638638	0.00093167	0.00462355
**IGF2**	1760.986117	3.020743508	0.196640966	5.23E-54	9.19E-52
**ARHGAP6**	7.627091859	3.01605013	1.459193136	0.00205255	0.00921971
**PDGFRB**	8.495881917	3.011301991	1.220563632	0.00126145	0.00601425
**ZNF117**	150.6808953	3.007932441	0.343959826	8.54E-20	3.05E-18
**KLF8**	387.9278533	2.991639183	0.196517995	1.41E-53	2.42E-51
**Z96811.1**	4.070803808	2.97378785	1.884369248	0.00772797	0.02862490
**EIF4E3**	389.9949544	2.973173076	0.200260493	1.24E-50	1.83E-48
**TBX5-AS1**	3.056659697	2.967001399	3.857036815	0.01263223	0.04309906
**ZNF273**	77.04227703	2.965544918	0.430607284	4.89E-13	9.9E-12
**AL589669.1**	2.793362222	2.965487687	3.667415391	0.01307578	0.04435896
**P2RX6**	16.83694587	2.965225524	1.228083284	0.00054760	0.00288653
**ID4**	3657.05422	2.964570825	0.219615019	2.06E-42	2.26E-40
**CPAMD8**	240.7020537	2.963085766	0.356108325	1.85E-17	5.48E-16
**KCNJ2-AS1**	16.94514848	2.958914482	0.728427302	6.97E-06	5.55E-05
**MXRA7**	437.5040332	2.95675911	0.375903986	4.53E-16	1.2E-14
**ZNF626**	20.28811315	2.949205108	0.901769793	0.00017605	0.00103909
**SLC41A2**	407.0011487	2.947031895	0.361188583	2.49E-17	7.27E-16
**LCA5**	50.68540638	2.943885036	0.54763949	6.98E-09	8.78E-08
**AC103706.1**	5.502356338	2.938079033	1.547809912	0.00541384	0.02116151
**AL591806.1**	5.300114116	2.9355329	1.450195332	0.00447685	0.01803869
**COBL**	13.28774379	2.930905091	1.082989552	0.00052661	0.00278263
**CMAHP**	300.6692676	2.927061533	0.347002755	4.09E-18	1.28E-16
**MAP7D2**	18.19723176	2.925972548	1.117225704	0.00051125	0.00271223
**OR52K3P**	17.49698703	2.925722299	0.774660978	1.25E-05	9.42E-05
**ERV3-1**	92.00722709	2.918558061	0.317301988	3.72E-21	1.48E-19
**THSD7A**	1289.110139	2.915246379	0.650467086	7.24E-07	6.86E-06
**ECEL1P3**	6.499248036	2.913528393	1.24730468	0.00255465	0.01114413
**AL355478.1**	178.9438516	2.913028095	0.322747975	4.78E-20	1.74E-18

**NPTX1**	31.4823874	2.912302225	0.645753641	1.06E-06	9.79E-06
**AL358852.1**	4.23293211	2.912077719	1.809508353	0.00651769	0.02474013
**LCP1**	75.83536563	2.911740539	1.663563408	0.00388731	0.01597555
**AL359644.1**	23.45939006	2.906919665	0.709452282	4.31E-06	3.55E-05
**AC091544.4**	22.08413935	2.904372409	0.90777627	0.00012202	0.00074717
**RN7SL308P**	15.88970041	2.897829239	0.834351712	6.16E-05	0.00040558
**MIR548XHG**	141.3295546	2.886440899	0.314064378	1.17E-20	4.54E-19
**PAIP2B**	148.0740722	2.886178876	0.291951245	6.01E-24	2.87E-22
**WNT10A**	221.9217849	2.885409531	0.336018714	1.26E-18	4.13E-17
**AC073062.1**	36.18152654	2.882691104	0.633206214	3.04E-07	3.06E-06
**ATP6V1B1**	5.935096727	2.874326133	1.590481088	0.00569285	0.02209676
**ID2-AS1**	19.39264152	2.873497926	0.731790314	1.24E-05	9.4E-05
**ENSG00000286763**	6.712130539	2.873082455	1.329944053	0.00352290	0.01471305
**ADGRL2**	114.3891883	2.871653765	0.432494633	8.51E-12	1.52E-10
**BASP1**	79.85822535	2.871315362	0.535552092	4.12E-09	5.37E-08
**TUBA1A**	87.29261182	2.867364902	0.361068015	3.28E-16	8.76E-15
**HYAL4**	10.35994727	2.865486302	1.04810807	0.00100046	0.00492100
**TP53TG1**	94.82069383	2.86243592	0.331007028	9.32E-19	3.08E-17
**AL139082.1**	2.537957802	2.859440058	3.48794844	0.01411480	0.04730450
**ENSG00000286315**	2.537957802	2.859440058	3.48794844	0.01411480	0.04730450
**MAFA**	8.392078404	2.857535979	1.393628474	0.00433749	0.01754815
**RGMA**	13.21610299	2.838279892	0.850307425	0.00011232	0.00069430
**PLSCR4**	155.4583997	2.837185576	0.350090783	2.11E-17	6.19E-16
**CROT**	409.5181927	2.835848235	0.252403335	5.14E-30	3.36E-28
**AMD1P3**	2.549128195	2.834736154	3.512618463	0.01426400	0.04774636
**AC130456.7**	10.78870811	2.834595507	0.927716173	0.00035425	0.00195079
**OTOF**	5.238984377	2.833672024	2.120863433	0.00823856	0.03016139
**GAB2**	143.0536072	2.832587487	0.279646936	3.34E-25	1.7E-23
**ENSG00000287474**	49.09969164	2.831558688	0.485830597	1.19E-09	1.67E-08
**EDARADD**	30.44795798	2.827758365	0.710345123	7.94E-06	6.23E-05
**TMEM200A**	27.28295157	2.823570718	0.657984631	3.24E-06	2.75E-05
**APH1B**	83.43418147	2.819830134	0.424514141	1.56E-12	3.02E-11
**ENSG00000286021**	5.103896458	2.816692043	1.496600465	0.00600134	0.02313641
**CTAGE1**	14.28276252	2.815340414	0.795603768	7.53E-05	0.00048547
**ZIK1**	7.274488689	2.811274296	1.540193199	0.00351469	0.01468396

**SLC7A8**	137.0655045	2.810508597	0.301623	8.38E-22	3.53E-20
**RF00568**	2.910211987	2.809808499	3.82251397	0.01369743	0.04612756
**DNAH7**	10.81959637	2.805915195	0.901989839	0.00028444	0.00160006
**AL359715.4**	4.012668147	2.803207854	1.980250432	0.01007334	0.03566088
**FLVCR2**	148.6521716	2.802793754	0.410709532	6.84E-13	1.37E-11
**KCNJ15**	75.10491843	2.800489853	0.415899765	4.85E-12	8.9E-11
**ZNF92P3**	5.905565632	2.792084301	1.478368649	0.00477801	0.01903086
**ISPD**	56.99177523	2.79195404	0.617497706	6.18E-07	5.92E-06
**AL807742.1**	7.436250493	2.791363701	1.16498047	0.00243766	0.01071113
**LARGE1**	48.67932033	2.791318344	0.469930652	2.73E-10	4.14E-09
**FAM129A**	1221.807352	2.789147916	0.243224959	3.85E-31	2.69E-29
**LGALS9B**	26.35982418	2.787709765	0.639170723	2.18E-06	1.91E-05
**PCDH10**	8.158494319	2.785947516	1.124212023	0.00173030	0.00795300
**AL031667.3**	12.71033058	2.785264188	0.823388498	9.78E-05	0.00061374
**AL354877.1**	6.503228122	2.785001817	1.20863101	0.00260485	0.01131991
**RPS2P36**	14.1086945	2.780765048	1.024728224	0.00094827	0.00469232
**AL359715.3**	8.599142662	2.779183177	1.073167351	0.00110182	0.00535244
**AC015878.2**	2.457481353	2.774975016	3.417865411	0.01476155	0.04914353
**TAPT1-AS1**	15.04089723	2.766649692	0.841283901	7.69E-05	0.00049509
**OR5P2**	27.69539314	2.765660908	0.700247021	6.68E-06	5.34E-05
**45353**	51.49438965	2.763513359	0.497618136	4.16E-09	5.42E-08
**SYTL4**	101.5374258	2.762677861	0.469893142	6.75E-10	9.76E-09
**DAB2**	103.9496589	2.761919462	0.423343161	1.56E-11	2.73E-10
**PMEL**	12.63593615	2.754388274	0.837948533	0.00013353	0.00080974
**AC015522.1**	2.658142361	2.748696895	3.61297004	0.01455239	0.04854859
**PPIL6**	6.198641321	2.744111581	1.210784827	0.00297733	0.01270761
**AC027335.1**	87.47764138	2.740153026	0.438186372	3.82E-11	6.35E-10
**ZNF385A**	122.2195476	2.739367603	0.3071132	6.22E-20	2.25E-18
**AC116096.1**	8.378357979	2.735121373	1.037651941	0.00101334	0.00497515
**PTN**	87.31515463	2.730732594	0.492654295	4.79E-09	6.17E-08
**AC002456.1**	22.54953601	2.726959354	0.69901346	1.17E-05	8.93E-05
**AC098934.1**	128.4099555	2.722759585	0.364973825	3.92E-15	9.67E-14
**MIR3167**	3.667785667	2.721874727	1.969992435	0.01145292	0.03972142
**RAB30**	312.4313064	2.718045445	0.247012299	5.13E-29	3.19E-27
**ENSG00000286134**	6.15985237	2.716545533	1.53857409	0.00576016	0.02232541

**AC093259.1**	2.646914513	2.715437428	3.716009464	0.0149481	0.0496677
**AC007431.3**	11.08863515	2.711329415	0.869091716	0.0003149	0.0017514
**TACR1**	17.40231772	2.709962774	1.024699443	0.0010947	0.0053200
**CSNK2A3**	23.88608839	2.709390121	0.73151625	2.49E-05	0.0001774
**NRXN3**	229.4328889	2.706938914	0.314889441	7.94E-19	2.64E-17
**CYB5R2**	71.91095309	2.703609361	0.353774854	4.49E-15	1.1E-13
**LONRF2**	33.0648874	2.702767333	0.777749306	8.86E-05	0.0005621
**AC007743.1**	183.5356818	2.701205087	0.289345921	6.51E-22	2.76E-20
**FP325330.3**	40.60095436	2.688614698	0.548050822	1.18E-07	1.27E-06
**GRB10**	35.51574175	2.686028185	0.53403369	5.75E-08	6.45E-07
**VTCN1**	6.565682829	2.685560106	1.483231053	0.0068389	0.0258039
**MIR181B2**	18.58128893	2.682791947	0.82616074	0.0001367	0.0008270
**ZNF488**	157.1080078	2.681816172	0.321487885	1.03E-17	3.11E-16
**STOM**	289.638119	2.672078418	0.302796748	3.23E-19	1.1E-17
**AC068620.3**	5.106767987	2.671939392	1.460738279	0.0063619	0.0242906
**ENSG00000287248**	7.423328276	2.668980511	1.317900153	0.0039279	0.0161066
**THEM7P**	4.208240781	2.665033051	2.188681592	0.0126234	0.0430873
**PRDM5**	34.50758451	2.664281362	0.569690067	2E-07	2.07E-06
**AC139769.1**	86.61653113	2.664215069	1.64605333	0.0047475	0.0189343
**AC020928.2**	8.56679023	2.662864818	1.134920533	0.0026425	0.0114629
**CFAP69**	50.72759349	2.661553732	0.420978908	3.38E-11	5.66E-10
**SMIM2-AS1**	64.46223769	2.661480414	0.518143732	2.97E-08	3.48E-07
**RBBP8NL**	15.2312636	2.656731109	0.752680652	5.03E-05	0.0003366
**KIAA1324**	34.99124757	2.655776903	0.770133336	5.26E-05	0.0003502
**ACSS3**	17.65184858	2.654719508	0.799699172	0.0001631	0.0009693
**RASL11B**	3.447588552	2.653581989	1.893726083	0.0118844	0.0409920
**KRT18P3**	3.614680925	2.64733472	2.000025242	0.0126304	0.0430990
**DGCR9**	5.554143373	2.640888971	1.426173505	0.0046801	0.0187253
**TNNT3**	855.2088652	2.640826068	0.364283319	4.59E-14	1.02E-12
**AC008906.2**	5.945288642	2.635565822	1.497059637	0.0050295	0.0198880
**HNMT**	8.271080737	2.632874963	1.186634443	0.0037015	0.0153384
**AC103764.1**	11.76331499	2.628818209	1.030611017	0.0015914	0.0073899
**HTR7**	10.21201675	2.627070274	1.038109466	0.0010876	0.0052920
**PTP4A1P3**	4.641691554	2.625679658	1.544956674	0.0089841	0.0323902
**DNMT3B**	84.30140467	2.624058266	0.441476391	2.15E-10	3.3E-09

**AC002074.1**	14.31394596	2.623212545	0.852177498	0.00034654	0.00191316
**ZNF682**	61.43459763	2.623148501	0.426681951	8.88E-11	1.42E-09
**MRAS**	83.07228118	2.611851091	0.356383631	6.54E-14	1.43E-12
**ACSM3**	16.06302981	2.607729112	0.804941403	0.00016195	0.00096327
**LOXL4**	56.73411125	2.6046155	0.511908054	2.53E-08	2.99E-07
**DLX3**	55.29582382	2.601210221	0.431839947	1.66E-10	2.57E-09
**AL109613.1**	4.907736255	2.599312626	1.60860328	0.00972201	0.03462220
**ARID5B**	672.0254744	2.598312138	0.216072188	4.84E-34	3.84E-32
**AC105924.1**	5.845146277	2.597939498	1.567359017	0.00738878	0.02753467
**KRT18P55**	5.812990504	2.597446693	1.231107663	0.00440648	0.01779412
**MAGI2-AS3**	118.1476817	2.597041367	0.328854588	6.85E-16	1.78E-14
**EPAS1**	5717.494919	2.596622474	0.163172601	7.61E-58	1.52E-55
**CLEC2B**	6.265654289	2.594769647	1.46743513	0.00772908	0.02862490
**CRYBG3**	568.3086338	2.594580662	0.365999859	1.68E-13	3.54E-12
**SSBP2**	347.1938094	2.59409452	0.263532133	3.53E-24	1.72E-22
**NR2F1**	259.8654136	2.592585738	0.414599623	6.43E-11	1.04E-09
**BBS5**	4.698668466	2.590212207	1.870203906	0.00630726	0.02411749
**AP000705.1**	6.846728177	2.586733792	1.115696088	0.00307476	0.01306283
**MAN1C1**	27.00431502	2.584876716	0.619341516	2.4E-06	2.08E-05
**CYP24A1**	32.14583446	2.583731481	0.844790534	0.00015795	0.00094183
**CDYL2**	425.1429089	2.583571026	0.274701015	9.32E-22	3.92E-20
**SERPINB9**	436.0207727	2.582721686	1.0853059	0.00139853	0.00658894
**ZDHHC20P4**	26.06543559	2.575984662	0.570410575	6.96E-07	6.61E-06
**TBL1X**	199.6622617	2.575532377	0.360297494	7.87E-14	1.71E-12
**AC026124.1**	15.78642029	2.574890613	0.860708069	0.00026813	0.00151599
**FDX1P2**	3.032035217	2.573806658	4.216708724	0.01472574	0.04904821
**DCLK1**	8.845072546	2.573559269	1.391578503	0.00548689	0.02140855
**ENSG00000285877**	7.669595256	2.572365347	1.856234243	0.00677283	0.02557866
**MORC4**	747.1993065	2.572207437	0.182490795	7.91E-46	9.8E-44
**ADRA1B**	10.40990688	2.569237923	1.148215789	0.00341866	0.01432781
**AL033397.2**	5.842341597	2.568118419	1.61208793	0.00828082	0.03027901
**AC009271.1**	23.02266227	2.560565022	0.624179085	4.71E-06	3.86E-05
**RN7SL449P**	6.822103696	2.554006988	1.110592778	0.00320988	0.01355738
**FSTL3**	521.741231	2.553129547	1.125127065	0.00186906	0.00849444
**CCDC80**	209.3123715	2.550378976	0.268185463	1.77E-22	7.73E-21

**PALMD**	50.45818212	2.545609572	0.53738011	3.97E-07	3.92E-06
**AP001318.2**	14.4487505	2.542776596	0.812402108	0.00016253	0.00096633
**HTRA1**	1386.208063	2.542066244	0.197927481	1.37E-38	1.29E-36
**SHC3**	22.82353215	2.540773382	1.017928217	0.00147162	0.00689930
**DUSP16**	336.9646262	2.537281003	0.205104962	2.93E-36	2.53E-34
**KBTBD8**	50.47586354	2.53684722	0.395481818	3.45E-11	5.77E-10
**CPEB2**	104.9476885	2.535322119	0.401288614	4.92E-11	8.04E-10
**FBN1**	5474.171804	2.53444183	0.187974119	2.8E-42	3.04E-40
**KAT2B**	318.6281973	2.527706193	0.292192778	3.08E-19	1.05E-17
**COL12A1**	14715.57596	2.526692594	0.252377746	2.41E-24	1.18E-22
**AC104063.1**	5.07535874	2.522618282	1.813107948	0.0129508	0.0440292
**KDELC1P1**	17.54618288	2.522078685	0.695015021	5.91E-05	0.0003913
**PLCL2**	472.9177502	2.519558026	0.226589997	2.03E-29	1.3E-27
**AF127577.4**	49.51810728	2.513354061	0.409599487	1.73E-10	2.67E-09
**PKP2**	756.3863319	2.51287266	0.313101992	1.37E-16	3.78E-15
**ZNF850**	301.1914823	2.511747025	0.214407242	4.02E-32	2.95E-30
**CYP3A5**	10.35510602	2.511512812	1.20587938	0.0047230	0.0188653
**AC011503.2**	29.41674124	2.511318805	0.558306591	1.1E-06	1.01E-05
**AC105411.1**	118.0282964	2.508062662	0.3456395	5.71E-14	1.25E-12
**AL162411.1**	10.16777831	2.506067782	1.162891035	0.0031580	0.0133692
**SH3BGRL2**	14.58595616	2.504098408	0.758778331	0.0001115	0.0006905
**DIAPH2-AS1**	4.603273758	2.504036664	1.480576023	0.0090421	0.0325404
**ZNF726**	128.6550227	2.499943657	0.281651738	6.2E-20	2.25E-18
**AC099689.1**	6.710342305	2.499622925	1.185932407	0.0048666	0.0193325
**NDUFB10P2**	8.592117725	2.495100395	1.037600419	0.0021342	0.0095381
**PRR16**	55.53664209	2.492749084	0.461450626	1.62E-08	1.96E-07
**FCGR3B**	8.757693975	2.491362536	1.003948789	0.0021069	0.0094302
**ST3GAL5**	95.57112522	2.488407001	0.381902335	8.04E-12	1.44E-10
**AC012377.1**	4.493605175	2.48803133	1.562512747	0.0111057	0.0387083
**TRIM29**	3557.747014	2.484671634	0.17901915	1.52E-44	1.8E-42
**AK7**	21.25936642	2.479856657	0.65344522	1.64E-05	0.0001206
**BX004987.1**	81.24584649	2.47856812	0.415705932	2E-10	3.07E-09
**MATN2**	57.68055076	2.46748531	0.542422839	8.34E-07	7.81E-06
**SNHG18**	91.17465126	2.451022115	0.454314022	1.25E-08	1.52E-07
**C4BPB**	7.768704726	2.449487077	1.031292418	0.0026506	0.0114905

**C5orf38**	197.0307421	2.446126871	0.267583904	1.19E-20	4.61E-19
**AL022068.1**	238.9654082	2.442835248	0.305236022	2.37E-16	6.4E-15
**FGF14-IT1**	8.42234322	2.441810155	1.009603231	0.00256391	0.01117245
**ZNF175**	412.9038547	2.440788702	0.311214751	6.42E-16	1.67E-14
**FN1**	22100.9495	2.437637912	0.703686418	6.72E-05	0.00043909
**MS4A13**	20.95864064	2.429715012	0.720007569	7.91E-05	0.00050830
**SATB2**	36.43959236	2.427783422	0.561686969	1.97E-06	1.74E-05
**IGSF9**	245.957202	2.427427992	0.268740049	3.09E-20	1.15E-18
**RPL37P18**	7.23419969	2.42035417	1.124631538	0.00394801	0.01617496
**AL365295.1**	28.12855976	2.416196707	0.555986472	2.13E-06	1.87E-05
**TGFB1**	97.85054423	2.415322649	0.44378775	4.92E-09	6.32E-08
**AC022075.1**	24.87197003	2.415296127	0.593417927	8.71E-06	6.78E-05
**CSRP2**	610.2381739	2.414139381	0.253739107	1.8E-22	7.84E-21
**AC009152.1**	15.01316959	2.413826899	0.764129979	0.00024915	0.00142109
**SLC16A5**	88.22753561	2.410673124	0.3461287	2.8E-13	5.79E-12
**AL360182.1**	11.67022847	2.409782873	1.310090695	0.00371046	0.01536761
**AASS**	180.5250518	2.40348037	0.260326403	5.95E-21	2.35E-19
**PGAM1P6**	7.185022816	2.400609577	1.149451319	0.00443320	0.01789597
**AL031590.1**	5.387101462	2.399833438	1.262264758	0.00720962	0.0270149
**TMEM178B**	812.6950109	2.39910743	0.204371458	1.22E-32	9.16E-31
**STS**	456.170122	2.383377766	0.220762517	7.31E-28	4.29E-26
**AC008543.3**	14.04395391	2.382051708	0.733210168	0.00020828	0.00120844
**AL359220.1**	50.22513685	2.380837272	0.617109457	1.89E-05	0.00013795
**UBXN10**	297.8693904	2.376729466	0.332328135	9.61E-14	2.07E-12
**ADAMTSL1**	6.713552795	2.372506551	1.44127096	0.01102938	0.03848694
**PES1P1**	9.995045934	2.372500762	0.925240815	0.00151216	0.00706307
**C2CD4A**	9.020279578	2.3692439	1.290767387	0.00784787	0.02897062
**B4GALT6**	197.0837603	2.368500433	0.227243595	3.25E-26	1.77E-24
**KRT16P6**	758.8225054	2.363182815	0.265245627	9.42E-20	3.36E-18
**AC021491.4**	10.62008189	2.362266154	0.863918078	0.00084411	0.00422765
**SNHG14**	271.1395894	2.362091879	0.274026487	6.47E-19	2.16E-17
**MSRB3**	61.02131154	2.359990568	0.442158189	1.63E-08	1.97E-07
**AC004009.1**	5.746431674	2.359711389	1.390917973	0.00964035	0.03439284
**NEBL-AS1**	5.373539459	2.358674014	1.53751094	0.00843802	0.03074100
**SLC25A27**	115.1217584	2.356156295	0.384737283	6.94E-11	1.12E-09

**AC004943.3**	3.710365307	2.355453831	1.928766346	0.01391857	0.04677346
**LINC01019**	46.55636627	2.34926672	0.413679674	2.02E-09	2.76E-08
**LINC00898**	7.091185879	2.344834675	1.72760937	0.00841494	0.0306756
**AC112487.1**	6.39217887	2.335993413	1.349565329	0.00826213	0.03023379
**LRRC6**	26.87041676	2.332173691	0.735001602	0.00011630	0.00071615
**AL158209.1**	7.031898256	2.329405671	1.179185059	0.00716360	0.02686773
**ADAMTS3**	7.888243726	2.327482986	1.458127446	0.01198722	0.04124411
**RPL39P36**	9.722266552	2.327345393	0.961752347	0.00189979	0.00861935
**ZNF493**	190.1590811	2.326724681	0.236008632	6.42E-24	3.05E-22
**SLC7A7**	14.66056732	2.325820686	1.227369713	0.00339722	0.01426048
**EFHB**	46.65557194	2.325679626	0.445149362	1.83E-08	2.19E-07
**LINC00954**	4.573560904	2.321711078	1.573821369	0.01303253	0.04423735
**LINC00662**	117.745239	2.321530215	0.344952836	3.28E-12	6.13E-11
**JAG2**	268.7181111	2.320376553	0.294707752	2.97E-16	7.93E-15
**ZNF90**	117.9036577	2.319124637	0.323949726	9.57E-14	2.06E-12
**BEX4**	44.59549837	2.314375015	0.450188477	5.86E-08	6.57E-07
**DRAM1**	1286.223859	2.313791908	0.144942404	5.3E-58	1.08E-55
**AC116337.3**	8.795194635	2.311829437	1.034788453	0.00360259	0.01499877
**AC099795.2**	37.27767763	2.307170122	0.573855469	8.83E-06	6.87E-05
**PLXND1**	246.7793546	2.302966716	0.220972418	2.74E-26	1.49E-24
**TFCP2L1**	60.34287198	2.300928827	0.409693601	3.89E-09	5.08E-08
**AC009262.1**	63.88551494	2.299764225	0.369340089	6.53E-11	1.06E-09
**CYP1A1**	1105.006637	2.298395641	0.30468369	8.47E-15	2.02E-13
**GNPTAB**	1689.47696	2.296042691	0.169166547	1.39E-42	1.53E-40
**CALHM3**	10.10579729	2.291469635	0.998855873	0.00341536	0.01432095
**LINC00871**	11.33986148	2.28856168	1.098939954	0.00371423	0.01538054
**PPARG**	376.0366719	2.283906128	0.243165326	1.34E-21	5.53E-20
**AC011447.6**	37.90634269	2.280497845	0.434012069	3.33E-08	3.87E-07
**AC004540.1**	8.786953226	2.279060935	0.972098853	0.00274948	0.01186644
**RPS3AP54**	5.605196642	2.276580266	1.285988733	0.00882830	0.03191485
**TPT1P10**	18.57753311	2.260357435	0.72918557	0.00021677	0.00125347
**AGR2**	177.279917	2.259593862	0.327423838	1.28E-12	2.51E-11
**NAV2**	2743.60187	2.259331257	0.255160555	1.45E-19	5.1E-18
**IRX2**	206.6808234	2.259220932	0.278832092	8.79E-17	2.44E-15
**FAM234B**	137.7252775	2.25736595	0.292440927	1.99E-15	5E-14

**FABP5**	1006.809278	2.252992125	0.340250814	5.55E-12	1.01E-10
**RHOV**	77.34029451	2.252911247	0.376011978	4.23E-10	6.27E-09
**TPPP3**	30.92838029	2.251136781	0.548486305	8.43E-06	6.58E-05
**AC068756.1**	13.1735829	2.247106588	0.783359037	0.00061600	0.00320538
**XIST**	53.10901774	2.246289929	0.383120967	7.86E-10	1.13E-08
**H19**	7777.000303	2.245821569	0.294523148	4.64E-15	1.13E-13
**ZNF704**	2912.021824	2.23980149	0.205673826	1.97E-28	1.19E-26
**HMGB1P41**	32.09627022	2.239505095	0.501450068	1.52E-06	1.37E-05
**MORN3**	14.23815688	2.23653611	0.755471435	0.00045102	0.00242498
**CLCN1**	7.139690712	2.233962597	1.282304368	0.00924561	0.03319522
**COBLL1**	2900.647604	2.232851313	0.171042441	9.23E-40	9.19E-38
**SOD3**	31.64167698	2.229502866	0.524928068	5.14E-06	4.18E-05
**AC011477.2**	21.78656987	2.227563916	0.714689578	0.00016917	0.00100220
**AL354822.1**	68.41244735	2.223207061	0.345998559	3.47E-11	5.79E-10
**TSC22D1**	1738.426591	2.222553977	0.242684602	8.7E-21	3.41E-19
**CMTM3**	44.76480496	2.222493153	0.474807704	3.13E-07	3.14E-06
**FRMD4A**	377.5098397	2.219890869	0.267944176	3.41E-17	9.82E-16
**ZNF454**	17.46771342	2.211254901	0.638348284	9.33E-05	0.00058858
**AC087588.2**	8.793081551	2.208553679	1.149485562	0.00786219	0.02900558
**LINC01133**	196.5756637	2.203247054	0.27617884	3.64E-16	9.67E-15
**EMB**	7.035199245	2.20143812	1.320604593	0.00756171	0.02810079
**AP002884.1**	28.05090737	2.19743677	0.568356065	2.4E-05	0.00017169
**CSF2RB**	9.881826143	2.196398607	0.941507714	0.00310972	0.01319495
**SPATA6**	127.4683218	2.194249159	0.323334063	1.02E-12	2.02E-11
**SNRPN**	15.56084423	2.192214247	0.893274401	0.00229873	0.01019528
**RNU6-943P**	4.641352593	2.191401907	1.546013419	0.01435427	0.04798808
**AC011447.7**	96.03851893	2.188947246	0.306840485	1.92E-13	4.01E-12
**TRIM24**	1167.439689	2.188249133	0.182154018	5.61E-34	4.44E-32
**ZNF662**	195.1656466	2.1864485	0.33051038	8.06E-12	1.44E-10
**LINC01698**	12.04944588	2.185112238	0.7979156	0.00087439	0.00436649
**CYFIP2**	648.1680361	2.183802062	0.277323027	5.81E-16	1.52E-14
**FRG1CP**	114.5021683	2.176883036	0.337818921	2.43E-11	4.14E-10
**BZW2**	3419.968857	2.175366138	0.223609317	4.11E-23	1.87E-21
**XYLT1**	643.5797653	2.174522378	0.227260326	1.68E-22	7.35E-21
**AP001318.1**	4.914834721	2.172686484	1.295475747	0.01208205	0.04151795

**ASNSP1**	118.7256861	2.161763035	0.346318585	1.24E-10	1.94E-09
**TAGLN**	36.14640283	2.160730731	0.521379125	7.34E-06	5.82E-05
**AC119674.1**	5.770185324	2.159681632	1.302838659	0.0131760	0.0446356
**CASC10**	124.4590893	2.158043306	0.314286953	1.34E-12	2.61E-11
**MIR5007**	8.377265497	2.157423005	1.001226644	0.0045712	0.0183541
**SRGAP3**	169.0325471	2.15393797	0.304005564	1.57E-13	3.31E-12
**CNOT6LP1**	52.06976502	2.153730677	0.446572861	2.73E-07	2.77E-06
**ZNF717**	304.3997354	2.153050117	0.188745916	7E-31	4.81E-29
**CHMP1B2P**	45.83937608	2.152562922	0.397899948	1.54E-08	1.86E-07
**LRRC23**	46.29501931	2.150391711	0.442109187	2.73E-07	2.77E-06
**AC098934.2**	30.67338099	2.143685172	0.531162262	1.15E-05	8.75E-05
**ID1**	1212.36179	2.142146378	0.202073154	6.44E-27	3.61E-25
**AL590004.4**	19.92298072	2.138571108	0.799187335	0.0010906	0.0053044
**SH3KBP1**	1207.333141	2.138253644	0.348929273	1.66E-10	2.57E-09
**RBKS**	33.06439354	2.136693912	0.489271078	2.48E-06	2.15E-05
**CASK**	213.8815363	2.135592746	0.267557316	1.37E-16	3.78E-15
**FAM171B**	52.43129894	2.135531152	0.533175889	1.05E-05	8.08E-05
**AC010542.1**	7.721472851	2.131134406	1.036722275	0.0051787	0.0203759
**ANK3**	220.5034244	2.12819181	0.256623463	3.11E-17	9.03E-16
**FRG1EP**	122.1520783	2.125560885	0.298454226	1.24E-13	2.64E-12
**RPEP3**	7.600220803	2.123253857	1.007587918	0.0057568	0.0223162
**NOTCH3**	272.5943303	2.122189747	0.331947589	2.37E-11	4.06E-10
**LINC01257**	32.5571672	2.121104422	0.492313828	2.68E-06	2.31E-05
**LINC00390**	8.632742272	2.121054387	1.008452857	0.0047745	0.0190201
**CU459211.1**	123.9866736	2.120060231	0.291150381	4.89E-14	1.08E-12
**GSPT2**	103.4248843	2.119115072	0.290945279	7.22E-14	1.57E-12
**LINC00643**	105.6906154	2.116547443	0.352794163	6.24E-10	9.05E-09
**AC133552.5**	13.69483051	2.115483902	0.80730266	0.0012875	0.0061195
**ZNF331**	1027.208492	2.11459071	0.187911386	4.01E-30	2.64E-28
**AC007216.1**	8.993043325	2.109131001	1.25972524	0.0100886	0.0357045
**ENSG00000288048**	63.19018238	2.108647946	0.385688014	9.31E-09	1.15E-07
**SLC29A3**	116.063601	2.108643259	0.325694004	2.97E-11	5E-10
**AL158206.1**	104.5722769	2.10702223	0.304880941	1.19E-12	2.34E-11
**MAGI1**	85.67301366	2.105084854	0.350548203	5.35E-10	7.85E-09
**TGM1**	86.66785884	2.104077291	0.567232746	2.72E-05	0.0001926

**ZNF540**	9.242915663	2.093060697	0.942181097	0.00453654	0.01824536
**AJ003147.3**	16.44761186	2.09017149	0.658489703	0.00024298	0.00139123
**METTL8P1**	4.762247536	2.090030843	1.315138096	0.01461808	0.04873374
**SAMD15**	42.94464097	2.085294364	0.416181462	1.06E-07	1.14E-06
**ZIC1**	76.27596986	2.084445525	0.349793792	4.11E-10	6.1E-09
**GJA1**	1001.727913	2.082810922	0.353158332	7.52E-10	1.08E-08
**MAGI2**	47.05725626	2.079225189	0.412423293	7.14E-08	7.89E-07
**AL356056.2**	72.78100752	2.078545013	0.395865096	2.44E-08	2.89E-07
**SMC1B**	50.1170398	2.075393303	0.429230018	2.24E-07	2.3E-06
**RAB40B**	13.866721	2.071925477	0.790657483	0.00158614	0.00737116
**SLC27A1**	185.7003101	2.068740726	0.309338394	4.47E-12	8.24E-11
**KAZALD1**	206.2231538	2.063888109	0.341384549	1.74E-10	2.68E-09
**C11orf70**	76.38256231	2.063777573	0.487270051	2.66E-06	2.3E-05
**ACER2**	154.3973766	2.060545748	0.229781937	7.81E-20	2.79E-18
**AC099552.3**	15.66517144	2.060341364	1.049685505	0.00629271	0.02406954
**BEX3**	800.3358299	2.057813378	0.190352352	5.9E-28	3.49E-26
**ENSG00000286724**	38.97182554	2.05346416	0.479490594	3.66E-06	3.07E-05
**MRPS30-DT**	11.61923138	2.050117549	0.982904434	0.00389063	0.01598642
**SLC19A3**	100.316696	2.043226402	0.341372561	6.09E-10	8.86E-09
**IGHV3-35**	8.793006097	2.043138744	0.908027019	0.0039096	0.01604781
**SEPT7-AS1**	10.87925548	2.042237964	0.941314132	0.00395180	0.01618568
**DPY19L2P1**	17.39920825	2.03790178	0.6879343	0.00043434	0.00234428
**PEG10**	5480.106798	2.036068897	0.300624643	2.89E-12	5.43E-11
**RCSD1**	543.6847925	2.035462271	0.345427745	7.99E-10	1.14E-08
**CYP2U1**	92.74403981	2.033614205	0.305224777	5.23E-12	9.55E-11
**ENSG00000286977**	23.75114288	2.032757822	0.565440039	6.84E-05	0.00044624
**RPS12P16**	13.56324451	2.026205228	0.750743379	0.00120369	0.00577686
**DICER1-AS1**	37.55889886	2.025466203	0.465973501	2.59E-06	2.24E-05
**TMEM240**	6.266984703	2.013376888	1.060738571	0.00969936	0.03455698
**DSEL**	446.3063759	2.009635432	0.247694503	7.73E-17	2.15E-15
**ZNF823**	212.1956175	2.008741936	0.267608903	1.24E-14	2.89E-13
**RN7SL653P**	19.19586113	2.00758411	0.697356283	0.00075561	0.00383990
**GDPD2**	192.1672256	2.00448809	0.247114622	1.06E-16	2.95E-15
**RNU6-1060P**	12.24893854	2.000268902	0.903994992	0.00518764	0.02040747
**NREP**	55.09792359	-2.000026544	0.626126172	0.00018659	0.00109593

**AP002800.1**	111.7603685	-2.006644814	0.341792997	6.63E-10	9.6E-09
**STAT5A**	46.29191231	-2.009213414	0.419429213	2.72E-07	2.76E-06
**AC002075.1**	11.73995302	-2.009284792	1.013766869	0.00672581	0.02542917
**CPED1**	28.59922597	-2.009423735	0.534760128	3.51E-05	0.00024355
**HMSD**	29.98057847	-2.010335393	0.647867003	0.00023623	0.00135648
**FOXD1**	342.1095204	-2.011907027	0.250093359	1.28E-16	3.55E-15
**AF165147.1**	21.76838685	-2.014523857	0.612926598	0.00016830	0.00099780
**AC007991.2**	17.02645009	-2.014544123	0.667101055	0.00050315	0.00267281
**FSTL5**	380.0875543	-2.014895615	0.251550513	2.82E-16	7.56E-15
**RF00017**	4.202135834	-2.015516008	1.311827091	0.01390629	0.04674532
**ITPR1**	82.66987043	-2.018137343	0.369975933	6.95E-09	8.76E-08
**PLEKHG4**	11.91653377	-2.018209451	0.816879922	0.00222197	0.00989892
**RPS6KA1**	382.7064675	-2.018583955	0.206810588	2.53E-23	1.16E-21
**ADGRE2**	24.41535499	-2.024646782	0.743688846	0.00074671	0.00379952
**ADARB1**	654.4938979	-2.025045841	0.377214404	1.4E-08	1.7E-07
**MIR2052HG**	11.35024452	-2.025365673	0.743295128	0.00098002	0.00483144
**AL133410.1**	24.3638688	-2.026287571	0.58154623	7.64E-05	0.00049153
**HLA-DQB1**	1226.282267	-2.026722337	0.208684509	4.67E-23	2.11E-21
**FOXI1**	13.23332839	-2.029386358	0.727280526	0.00091312	0.00453714
**PKN2-AS1**	24.29749402	-2.029773299	0.608002919	0.00010929	0.00067861
**SH3BP1**	99.04450006	-2.030219784	0.295985204	1.3E-12	2.55E-11
**RNU7-181P**	13.10341007	-2.031547051	0.787311581	0.00160523	0.00744399
**AC021504.1**	96.75327546	-2.032130795	0.31389668	2.04E-11	3.5E-10
**AL160272.1**	8.386635363	-2.033232858	1.291790928	0.00982224	0.03491154
**TNFRSF10D**	153.358122	-2.037107614	0.410408854	9.95E-08	1.08E-06
**ZSCAN12P1**	179.9049066	-2.040729228	0.217398827	1.66E-21	6.83E-20
**AC107419.1**	12.30465137	-2.041606879	0.805950898	0.00207509	0.00930523
**ETS1**	2600.930728	-2.045082753	0.61817469	0.00013416	0.00081272
**SAMD9L**	6662.468274	-2.04709878	0.150555081	7.91E-43	8.84E-41
**FBXO16**	50.99302464	-2.047742604	0.356240268	1.84E-09	2.52E-08
**SPERT**	10.90309818	-2.050735425	0.892008985	0.00299682	0.01277938
**AC124319.1**	59.50569551	-2.052456231	0.361386835	2.73E-09	3.65E-08
**LRG1**	19.46414786	-2.054445161	0.869816736	0.00257690	0.01121822
**SEMA3B**	12.14523586	-2.055942806	0.961123542	0.00373973	0.01546781
**CASP4**	2769.973333	-2.055964937	0.129245618	1.13E-57	2.2E-55

**AC009226.1**	9.237193076	-2.056181451	0.994558415	0.00453829	0.01824629
**AC016831.7**	338.6190639	-2.056365334	0.187334749	1.03E-28	6.31E-27
**FAM46C**	26.25563373	-2.057288124	0.761976056	0.00088313	0.00440551
**NBPF14**	153.03191	-2.058935083	0.24408492	8.37E-18	2.55E-16
**B3GALNT1**	22.6825138	-2.060006524	0.581159934	8.43E-05	0.00053782
**TRPM2**	69.22798552	-2.060812246	0.397978416	5.75E-08	6.45E-07
**AC108673.2**	17.13555668	-2.061242699	0.613969398	0.00014101	0.00085076
**GPRC5D-AS1**	25.30597984	-2.064932809	0.586069979	5.98E-05	0.00039512
**PLA2G4C**	68.22547654	-2.066432296	0.408405358	6.27E-08	7E-07
**SRCIN1**	20.5703797	-2.06704143	0.696771995	0.00034987	0.00192931
**AC004784.1**	11.79494985	-2.067679213	0.837604128	0.00225733	0.01003708
**VPS9D1-AS1**	16.43399699	-2.068896937	0.810941356	0.00170511	0.00785384
**APBB2**	923.8763339	-2.069541224	0.219800101	9.92E-22	4.15E-20
**IL27RA**	8.741417859	-2.070325889	1.158001124	0.01003097	0.03554247
**LYST**	1547.298937	-2.071392628	0.188339563	6.6E-29	4.08E-27
**EMP2**	1159.817321	-2.072016655	0.235480982	2.31E-19	7.99E-18
**DKK 3.00**	241.347856	-2.073947039	0.380647487	1.06E-08	1.3E-07
**LINC01694**	295.4719065	-2.075228445	0.244214236	4.78E-18	1.49E-16
**ANKRD18B**	593.1096859	-2.075931945	0.213410461	5.68E-23	2.53E-21
**PRNP**	3803.172077	-2.077172287	0.1829902	1.47E-30	1E-28
**HLA-DOA**	310.3844328	-2.080381467	0.254454189	4.4E-17	1.26E-15
**AHNAK2**	2171.616953	-2.080909087	0.666056648	0.00024939	0.00142213
**HMOX1**	91.30852358	-2.081956607	0.369849156	2.95E-09	3.92E-08
**CTH**	550.3498523	-2.081963284	0.229289398	2.47E-20	9.3E-19
**AC009962.1**	44.21606962	-2.082991038	0.460422517	1.41E-06	1.28E-05
**CARD6**	268.9065978	-2.083067945	0.20906046	3.43E-24	1.68E-22
**OVOL1**	193.1543493	-2.084413541	0.276858853	1.17E-14	2.73E-13
**ATP2C2-AS1**	169.1071108	-2.084502637	0.277953957	9.67E-15	2.28E-13
**IFNLR1**	406.3228028	-2.086456954	0.253421154	2.89E-17	8.39E-16
**AL442128.1**	2.94495627	-2.086508732	2.192799246	0.01197731	0.04122325
**MYH14**	1695.700853	-2.090368423	0.192474183	3.77E-28	2.24E-26
**TICAM1**	382.2435709	-2.091176715	0.217417061	1.78E-22	7.76E-21
**ABLIM2**	10.22796916	-2.093179001	0.773906118	0.00103346	0.00506355
**AC025180.1**	12.87981669	-2.093227943	0.791520068	0.00106848	0.00521108
**OASL**	268.9582153	-2.096153101	0.337284062	7.83E-11	1.26E-09

**ASRGL1**	24.28990958	-2.099770832	0.507175318	6.05E-06	4.87E-05
**MAST1**	15.42065999	-2.101424986	0.711549902	0.00038219	0.00208638
**PPIF**	1110.8349	-2.102202045	0.195389481	1.05E-27	6.15E-26
**HELZ2**	1049.994435	-2.103497557	0.161237477	1.16E-39	1.15E-37
**SLC38A5**	12.22930377	-2.104423444	1.224230673	0.00668724	0.02530336
**FAT3**	8.8478176	-2.111490309	0.898089905	0.00301245	0.01283916
**DNAH11**	222.1995239	-2.111500908	0.325442504	1.56E-11	2.74E-10
**FAM198B**	275.4079592	-2.114117049	0.286891509	3.62E-14	8.14E-13
**TH**	30.7120171	-2.118431644	2.028911161	0.01219516	0.04184649
**MPZL2**	5181.06881	-2.118435631	0.172417185	2.01E-35	1.69E-33
**NAPRT**	262.5526949	-2.118485317	0.290095945	5.94E-14	1.3E-12
**MTCL1**	564.7037646	-2.118960485	0.188029838	3.69E-30	2.44E-28
**CHST11**	150.0379304	-2.119108073	0.327857623	1.81E-11	3.13E-10
**ARL 10.00**	13.94219261	-2.120048683	1.346037497	0.00815550	0.02992605
**TANC2**	1066.444242	-2.12102629	0.340409025	7.61E-11	1.23E-09
**TMEM255A**	11.59297194	-2.121449433	1.061511868	0.00424352	0.01720872
**SPRY4-AS1**	74.8356295	-2.121753419	0.428435745	1.66E-07	1.75E-06
**KLF7**	399.7803018	-2.122288605	0.337897609	4.91E-11	8.03E-10
**SPRY1**	46.20894212	-2.122826566	0.511770612	4.37E-06	3.6E-05
**CAMKK1**	32.72440153	-2.124456034	0.461587906	6.3E-07	6.03E-06
**HAUS6P2**	32.14795805	-2.125373928	0.48461456	1.64E-06	1.47E-05
**GVINP1**	70.76551159	-2.125600886	0.368440532	1.2E-09	1.68E-08
**SYTL3**	175.3369173	-2.125813061	0.313661184	1.48E-12	2.89E-11
**GSAP**	216.4735707	-2.130460162	0.227992605	1.56E-21	6.44E-20
**TMEM238**	9.07739951	-2.132085928	1.094365382	0.00534294	0.02092718
**AL136982.6**	11.49915735	-2.132491833	0.777253146	0.00090828	0.00451591
**FAM178B**	32.63221078	-2.133138333	0.496486747	3.23E-06	2.74E-05
**GFY**	8.001685504	-2.134653747	0.972134157	0.00355560	0.01482638
**RAB3D**	122.9690481	-2.136854192	0.285656412	1.1E-14	2.59E-13
**PTPRN2**	11.64850275	-2.136901832	0.884915982	0.00185982	0.00845569
**SH3BGRL3**	897.8309047	-2.137763057	0.164138885	1.69E-39	1.66E-37
**ATP2C2**	103.9601916	-2.138025349	0.438663057	1.52E-07	1.6E-06
**SLC45A3**	23.4303854	-2.138156019	0.510382483	5.03E-06	4.1E-05
**LINC02154**	52.00217294	-2.141091382	0.522374363	6.48E-06	5.2E-05
**HMCN2**	16.84845625	-2.141328094	0.81047398	0.00093426	0.00463449

**PLEKHG2**	223.9335457	-2.143594855	0.239267767	4.38E-20	1.62E-18
**SACS**	89.68659204	-2.146053195	0.386921086	6.28E-09	7.95E-08
**GPSM1**	78.57182137	-2.149831302	0.349327439	9.65E-11	1.53E-09
**SNX18P7**	94.40988569	-2.150091843	0.280266185	4.17E-15	1.03E-13
**DZIP1**	13.35978503	-2.150127747	0.75816779	0.00071558	0.00365901
**GAMT**	11.03744446	-2.150255295	1.059473	0.00366851	0.01522549
**AC093388.1**	15.08059003	-2.150328997	0.748828228	0.0005341	0.00281906
**TMC7**	79.53357253	-2.152583728	0.308660824	5.52E-13	1.11E-11
**SEMA7A**	206.4646814	-2.153316867	0.450085608	2.41E-07	2.46E-06
**ENSG00000287064**	14.91301714	-2.154071052	0.686173919	0.00029212	0.00163728
**SLC39A14**	1100.170664	-2.154435512	0.217321475	5.87E-24	2.81E-22
**ENSG00000286381**	18.21423295	-2.154521201	0.839804831	0.00130497	0.00619209
**HLA-DPA1**	1088.400688	-2.156249851	0.18204703	3.32E-33	2.58E-31
**TGIF2**	60.72026655	-2.156529161	0.331839939	1.26E-11	2.22E-10
**NEXN**	135.5940268	-2.158065129	0.398157939	8.95E-09	1.11E-07
**RN7SL749P**	9.556662307	-2.160338007	1.152847109	0.00584354	0.02261560
**RTP4**	210.7303195	-2.16170869	0.288114991	7.47E-15	1.79E-13
**AP000695.2**	9.649482876	-2.164919772	1.202118487	0.00550515	0.02147364
**FOXA3**	3.908400222	-2.165354601	1.422597121	0.01216258	0.04175867
**GOLM1**	840.222142	-2.165776924	0.158153116	1.53E-43	1.76E-41
**RFLNB**	32.18907088	-2.166593698	0.503474521	3.13E-06	2.66E-05
**SOCS3**	106.0158415	-2.167426022	0.493808129	1.73E-06	1.54E-05
**MAFF**	755.9852453	-2.168055659	0.231882314	1.33E-21	5.53E-20
**ZNF792**	5.350246708	-2.170529663	1.151713712	0.00737836	0.02750495
**AL121772.1**	36.43451647	-2.170652048	0.569581437	2.59E-05	0.00018455
**HLA-DQA1**	457.5318476	-2.17207435	0.243170476	6.24E-20	2.26E-18
**FXYD5**	2456.857813	-2.172297076	0.154677892	1.63E-45	2.01E-43
**TMSB10**	4874.827053	-2.172798301	0.176298508	1.14E-35	9.67E-34
**RFTN1**	25.2337968	-2.174193423	0.651335261	8.83E-05	0.00056058
**RAET1L**	11.46486715	-2.175160396	1.009011835	0.00369997	0.01533477
**MIR503HG**	42.96421357	-2.178058147	0.549816927	1.34E-05	0.00010026
**PFN2**	495.1047268	-2.179913522	0.171756932	8.83E-38	8.03E-36
**SBK1**	44.41363405	-2.18032409	0.489932172	1.15E-06	1.05E-05
**TGFB1I1**	149.5359584	-2.181427943	0.266463386	4.16E-17	1.19E-15
**EMBP1**	37.78639327	-2.182503091	0.425478728	3.72E-08	4.3E-07

**LURAP1L-AS1**	26.01881807	-2.182549401	0.517020711	3.51E-06	2.95E-05
**MIR2355**	4.008542586	-2.183391384	1.366588885	0.01293029	0.04399734
**POTEF**	103.797254	-2.189030253	0.40167213	1.08E-08	1.33E-07
**UPP1**	1046.183621	-2.190579503	0.248740511	1.89E-19	6.6E-18
**FAM167B**	26.09859913	-2.193510905	0.678421316	0.00013330	0.00080873
**HLA-F**	4537.216761	-2.193944938	0.196418414	9.29E-30	6.02E-28
**NAV3**	61.91490929	-2.193986472	0.426403401	5.11E-08	5.78E-07
**ERG**	3.982670929	-2.194926765	1.511360708	0.01316105	0.04460393
**WDR72**	4.012506598	-2.195604738	1.532138051	0.01342688	0.04534098
**FAP**	21.13675367	-2.19781721	0.702589049	0.00026432	0.00149724
**NAMPTP1**	9.400136589	-2.198226233	0.896556335	0.00180012	0.00822019
**PML**	860.8021626	-2.199853336	0.189223108	4.79E-32	3.49E-30
**PICSAR**	100.6427452	-2.201248776	0.386831223	1.57E-09	2.17E-08
**HAP1**	36.41679911	-2.201894593	0.463959367	2.77E-07	2.8E-06
**COL4A6**	1922.849975	-2.202846175	0.145389388	1.42E-52	2.36E-50
**ADGRE5**	913.7148765	-2.204988078	0.187575038	1.43E-32	1.06E-30
**CD274**	1234.049354	-2.206694536	0.222094922	5.62E-24	2.71E-22
**AL159169.3**	7.827239141	-2.207883913	0.932506854	0.00229704	0.01018969
**AC013652.2**	10.19848832	-2.209810726	0.873782134	0.00182317	0.00831274
**ADD3**	99.75918657	-2.210686008	0.301575352	3.9E-14	8.71E-13
**TBX1**	4.916010266	-2.210812753	1.170672305	0.00696561	0.02620479
**NOD2**	83.19005521	-2.214555179	1.079493037	0.00366313	0.01521112
**KYNU**	203.0626695	-2.214668373	0.361293731	1.31E-10	2.06E-09
**ANKRD30B**	39.01302051	-2.214677672	0.52143887	3.97E-06	3.31E-05
**CXCL9**	103.5906472	-2.215853371	0.589708949	2.12E-05	0.00015289
**CD9**	417.8690332	-2.216716033	0.267208896	2.08E-17	6.14E-16
**CTSL**	2890.332731	-2.220064635	0.155040149	3.13E-47	4.16E-45
**SLC7A2**	325.591304	-2.221935467	0.686234079	0.00016130	0.00096011
**ACSL5**	1150.803741	-2.223191263	0.169198405	4.02E-40	4.04E-38
**RFPL4AP5**	7.719116022	-2.224945609	1.199356786	0.00769930	0.02854114
**FAM43A**	11.82144125	-2.226607279	0.74899943	0.00039507	0.00215075
**AC093904.3**	6.055274914	-2.226622875	1.10336498	0.00559191	0.02177567
**PARP11**	70.23689306	-2.22711603	0.512002985	2.56E-06	2.22E-05
**WFDC21P**	178.6677399	-2.229917125	0.28711826	2.05E-15	5.15E-14
**IFI44**	724.3805058	-2.233212227	0.613326012	3.54E-05	0.00024576

**C3orf67**	80.78487596	-2.234044832	0.377444918	5.27E-10	7.74E-09
**ENSG00000287923**	28.21123664	-2.235166112	0.537973861	7.05E-06	5.61E-05
**DAGLA**	5.560739672	-2.235428995	1.467637595	0.01293492	0.0439973
**AL390066.1**	17.07411693	-2.236630819	0.61911578	4.88E-05	0.0003274
**45541**	73.47195402	-2.238996132	0.351437853	2.03E-11	3.5E-10
**CRYZL2P**	86.76120408	-2.241281251	0.314074275	1.22E-13	2.61E-12
**AFAP1**	208.0665751	-2.241920874	0.397686899	3.17E-09	4.2E-08
**LRRC2**	12.73089128	-2.2453283	0.869514342	0.0011391	0.0055067
**CRYZL2P-SEC16B**	27.39769515	-2.251182787	0.51528501	1.52E-06	1.37E-05
**ELL2**	949.3868032	-2.251236594	0.3826746	5.81E-10	8.48E-09
**SAMD9**	13132.46833	-2.251366637	0.175211523	1.41E-38	1.32E-36
**SLC9A3R2**	25.82416586	-2.25352789	0.569201898	1.07E-05	8.17E-05
**TRPS1**	861.0570595	-2.255874421	0.16819101	7.89E-42	8.38E-40
**TOR4A**	64.70669548	-2.256257383	0.373548723	3.23E-10	4.85E-09
**CXCL2**	28.9830538	-2.256476101	0.527464462	2.43E-06	2.11E-05
**AC007728.3**	6.004355718	-2.256886651	1.707744961	0.0102101	0.0360596
**CDK15**	5.483650865	-2.258689082	1.274879279	0.0087580	0.0317088
**SAA2**	53.7233996	-2.265816297	0.391333658	1.06E-09	1.5E-08
**HHIPL1**	4.946357154	-2.266819699	1.615703029	0.0131900	0.0446704
**CLDN23**	21.99704569	-2.267055797	0.618607432	3.33E-05	0.0002323
**ENSG00000285601**	7.808344614	-2.268099205	1.11502732	0.0053180	0.0208517
**LTF**	97.76660935	-2.269484084	0.4092041	2.97E-09	3.95E-08
**AC009948.1**	11.99407963	-2.269517513	1.004703065	0.0023217	0.0102803
**AL158071.3**	8.204278873	-2.270801381	1.196667812	0.0053355	0.0209100
**RETNLB**	3.306340294	-2.271108841	2.375874461	0.0122647	0.0420491
**ARNT2**	379.4660159	-2.273622371	0.4367226	2.6E-08	3.08E-07
**PREX1**	142.1772523	-2.275448289	0.29680391	3.24E-15	8.02E-14
**RNF130**	22.3747252	-2.279105244	0.689105047	0.0001212	0.0007430
**PLA2G2F**	7.062616014	-2.280532605	1.000657412	0.0032654	0.0137678
**AL160162.1**	127.6360445	-2.280768283	0.27873993	5.99E-17	1.7E-15
**ZNF503**	19.75033893	-2.280843531	0.605578197	2.63E-05	0.0001867
**AFAP1L2**	53.79731737	-2.28127402	0.417199398	9.4E-09	1.16E-07
**KCNH5**	52.36183399	-2.281673418	0.53490858	3.03E-06	2.58E-05
**CRYBB3**	8.272294691	-2.283659939	0.989052999	0.0023704	0.0104610
**HERC5**	421.1963403	-2.286720863	0.318084482	8.62E-14	1.87E-12

**AL589740.1**	4.108359726	-2.288676336	1.427937938	0.01219382	0.04184649
**ITM2C**	75.9570388	-2.290028129	0.335625722	1.1E-12	2.18E-11
**NETO2**	903.8912088	-2.292335288	0.155616894	5.18E-50	7.51E-48
**COL9A2**	19.62299735	-2.295008152	0.670199047	7.87E-05	0.00050587
**ENSG00000287763**	5.396424067	-2.29539295	1.315116477	0.00942563	0.03373757
**AC092902.5**	6.974793388	-2.29671227	1.315743603	0.00724752	0.02713989
**AC004067.1**	16.54397025	-2.301321338	0.747527935	0.00031021	0.00172937
**BTBD16**	24.8080721	-2.304463044	0.67386448	7.6E-05	0.00048932
**SH3BP4**	276.6933068	-2.305529315	0.324831461	1.88E-13	3.94E-12
**ZFPM2-AS1**	209.6388917	-2.305718033	0.25467832	2.29E-20	8.61E-19
**FLNC**	22.72471551	-2.307819299	0.620238645	2.94E-05	0.00020691
**LINC01592**	12.47349564	-2.308577786	0.78801614	0.00056012	0.00294600
**CXCL11**	163.192342	-2.308650378	0.439585157	2.02E-08	2.42E-07
**AC127521.1**	7.993907942	-2.31171022	0.972714274	0.00240896	0.01060940
**AC116424.1**	6.624579757	-2.312873179	1.260206735	0.00566275	0.02200137
**AL078459.1**	101.9980747	-2.314518319	0.501661131	4.49E-07	4.4E-06
**PLEK2**	364.6681684	-2.31658208	0.286063798	7.39E-17	2.06E-15
**KCNK6**	348.1332833	-2.316790773	0.217463668	1.78E-27	1.03E-25
**LINC00351**	391.1049558	-2.316898599	0.184834475	6.32E-37	5.58E-35
**HLA-DRB1**	1273.8671	-2.31709072	0.201598294	1.98E-31	1.41E-29
**SLC9A7**	591.622679	-2.318191168	0.250643166	3.27E-21	1.31E-19
**TCN2**	50.74835988	-2.322145892	0.355299488	8.83E-12	1.57E-10
**PLK3**	471.3779352	-2.323476481	0.311635363	1.7E-14	3.92E-13
**AC105285.1**	20.86088988	-2.325635234	0.568827253	6.97E-06	5.55E-05
**AC005515.1**	114.5573949	-2.328827711	0.289368338	8.08E-17	2.25E-15
**AC087664.3**	3.075146968	-2.330606121	2.148184039	0.00931052	0.03340556
**AOX1**	33.07674011	-2.330854615	0.546449025	1.98E-06	1.75E-05
**FSIP2**	57.7553708	-2.331778762	0.454794454	3.55E-08	4.12E-07
**RN7SKP202**	6.94203049	-2.332117773	1.555875994	0.00879505	0.03181388
**ARHGAP42**	472.2701785	-2.333224492	0.181871044	1.82E-38	1.7E-36
**STX2**	33.34634641	-2.334278903	0.478411957	1.6E-07	1.68E-06
**RUFY4**	5.844431246	-2.336178165	1.284831044	0.00651274	0.02472920
**ERC2**	19.53441032	-2.337552632	0.709097349	0.00012348	0.00075532
**AC091489.1**	3.194758591	-2.337897767	2.165385841	0.00906343	0.03260709
**TXK**	21.3537765	-2.339509131	0.690610725	0.00010152	0.00063559

**ZNF296**	16.02575376	-2.340091351	0.707148752	0.00014367	0.00086418
**SEC16B**	20.11184153	-2.34161356	0.701882252	9.05E-05	0.00057269
**NOTCH2**	357.1994993	-2.341806155	0.240989431	4.51E-23	2.04E-21
**SATB1**	4.932306341	-2.34365579	1.687300997	0.01363138	0.04594036
**GNG4**	3.466914024	-2.345392512	2.576234807	0.01196541	0.04119410
**ARL8A**	85.7519846	-2.347526427	0.327761508	8.98E-14	1.94E-12
**ZNF697**	229.6116124	-2.347592549	0.217322112	8.26E-28	4.83E-26
**C1orf74**	1021.694835	-2.348059681	0.281083317	9.97E-18	3.01E-16
**GRIN1**	9.263609243	-2.349011408	0.858798017	0.00074806	0.00380397
**MAP3K7CL**	85.18891814	-2.351146041	0.324581283	4.31E-14	9.6E-13
**CD74**	8914.585138	-2.353852781	0.164750994	3.78E-47	4.97E-45
**TMEM154**	165.999993	-2.355878806	0.336914593	4.82E-13	9.79E-12
**UNC93B4**	6.782896265	-2.356285403	1.451099939	0.00973221	0.03464303
**PATJ**	1325.825289	-2.359414883	0.249165181	3.76E-22	1.62E-20
**SECTM1**	717.114701	-2.363971742	0.211116997	6.5E-30	4.23E-28
**SLC25A30**	384.5985016	-2.364683186	0.208099311	1.52E-30	1.03E-28
**PTGER4**	482.7636285	-2.365236395	0.2330504	6.54E-25	3.29E-23
**AL583808.1**	7.808806667	-2.366057688	1.045873494	0.00234560	0.01037045
**ZDHHC2**	619.1555885	-2.367187579	0.148023884	2.37E-58	4.89E-56
**PSG8**	14.44268301	-2.368229335	0.700851201	8.33E-05	0.00053218
**KPNA7**	12.91948205	-2.369217971	0.918303586	0.00109467	0.00532001
**NUP210L**	9.228847403	-2.371415964	0.885194541	0.00087977	0.00439246
**IGFBP4**	52.66451218	-2.371816056	0.514300786	6.26E-07	5.99E-06
**ANKFN1**	28.92908063	-2.372576985	0.545881221	2.46E-06	2.14E-05
**AC005592.1**	13.03568614	-2.372768576	1.120303094	0.00367731	0.01525672
**U62317.2**	7.131422661	-2.372867562	1.194137897	0.00592193	0.02287517
**B3GNT5**	449.0610434	-2.374412469	0.343299128	5.95E-13	1.2E-11
**TNFRSF10B**	915.2398713	-2.374832176	0.148049235	8.3E-59	1.77E-56
**LINC01234**	116.8367735	-2.374929709	0.465348038	3.46E-08	4.01E-07
**DPEP2NB**	8.923470454	-2.377035188	1.09864379	0.00369570	0.01532242
**CACNA1A**	22.39010781	-2.37835474	0.574475752	3.88E-06	3.24E-05
**AIF1L**	92.92234007	-2.379006983	0.378763841	3.63E-11	6.05E-10
**ZIC2**	97.55573723	-2.380278595	0.349342144	1.99E-12	3.82E-11
**DENND2C**	338.4702556	-2.384883163	0.192019852	1.94E-36	1.7E-34
**PCAT7**	11.35197083	-2.386074414	0.768262894	0.00029970	0.00167467

**MIR31HG**	43.9825089	-2.386429476	0.447249937	1.14E-08	1.4E-07
**CYP27B1**	25.74344963	-2.38674668	0.571669472	4.75E-06	3.88E-05
**HLA-G**	7434.851754	-2.387019974	0.686484806	5.85E-05	0.00038725
**EIF4HP2**	35.6839825	-2.388854135	0.525745898	9.54E-07	8.86E-06
**CD44**	6028.30377	-2.391087428	0.54300347	1.23E-06	1.12E-05
**F2RL1**	1068.781783	-2.39186369	0.288428163	1.85E-17	5.47E-16
**MCTP1**	7.330174881	-2.392681375	1.180041582	0.00476028	0.01896658
**NFATC2**	177.4396173	-2.393748286	0.314807012	4.38E-15	1.07E-13
**AC025259.2**	6.405754638	-2.399001585	1.118240533	0.0034796	0.01455266
**SCG2**	12.85407109	-2.402754951	0.871853046	0.00072637	0.00370943
**PRKCQ**	102.2100624	-2.402760018	0.501928301	2.02E-07	2.09E-06
**SLC2A6**	35.69431524	-2.404399543	0.507097122	2.57E-07	2.61E-06
**PECR**	18.73249681	-2.409003862	0.686702835	5.07E-05	0.00033919
**RTKN2**	713.9316684	-2.410281752	0.211017357	6.04E-31	4.17E-29
**LMCD1-AS1**	16.28230855	-2.4123105	0.791109838	0.00026223	0.0014872
**MMP25-AS1**	468.2305503	-2.412687812	0.209383098	9.7E-32	6.96E-30
**KF456478.1**	14.66674458	-2.414947272	0.875176973	0.00044594	0.00239930
**LINC00511**	222.3084163	-2.42269999	0.33165943	3.13E-14	7.09E-13
**AC099518.1**	12.79492271	-2.423876376	0.857402478	0.00072349	0.00369708
**TMC6**	65.73648691	-2.424313566	0.41613727	1.33E-09	1.85E-08
**OCIAD2**	198.4561673	-2.430695845	0.213027332	6.67E-31	4.6E-29
**TNFAIP8L3**	3.136551157	-2.431314677	2.224618425	0.01079075	0.0377700
**PROX1**	34.25602817	-2.433655981	0.565142783	1.87E-06	1.65E-05
**PLAU**	2895.54904	-2.437245898	0.263834599	3.76E-21	1.5E-19
**NFKBIE**	211.1919188	-2.437787077	0.26829559	1.87E-20	7.1E-19
**CFLAR-AS1**	21.98284841	-2.44058687	0.579359179	3.1E-06	2.64E-05
**S100A2**	81.86938099	-2.440760861	0.479007694	3.3E-08	3.84E-07
**RN7SKP115**	3.210867479	-2.443273063	1.7232976	0.01256882	0.04293150
**RN7SL368P**	15.93542094	-2.444040613	0.807000258	0.00031333	0.00174437
**GIPR**	22.08374914	-2.444140733	0.662237596	2.34E-05	0.00016780
**TRIM10**	9.254645116	-2.444147601	1.413111401	0.00604217	0.02326758
**DMKN**	270.0924667	-2.444262226	0.234852016	5.4E-26	2.88E-24
**ENSG00000287232**	112.0588923	-2.445431576	0.3895395	5.64E-11	9.18E-10
**CLMP**	96.83403718	-2.446396	0.535417727	6.96E-07	6.61E-06
**DPP4**	25.95860584	-2.446969569	0.66909163	2.68E-05	0.00019005

**MCPH1-AS1**	30.06074994	-2.448590934	0.618725706	1.34E-05	0.00010016
**RNF213**	15107.15581	-2.449142115	0.135220919	3.8E-74	1.44E-71
**AC011294.1**	7.146222369	-2.451133705	1.17950327	0.00333580	0.01403220
**URAHP**	3.646744347	-2.45384607	2.69936036	0.01115009	0.03883447
**CT62**	8.829674208	-2.453852816	0.923551297	0.00116812	0.00562877
**DNAH8**	1069.666317	-2.458264806	0.285386038	8.69E-19	2.87E-17
**CFHR3**	12.84187076	-2.461064259	1.168997502	0.00387786	0.01594216
**DEF8**	100.2077688	-2.467449199	0.336521638	2.28E-14	5.22E-13
**TNFRSF1B**	184.6870682	-2.470404933	0.24799051	5.82E-24	2.79E-22
**MAP1B**	29.74829133	-2.473465382	0.511421319	1.78E-07	1.86E-06
**ISG20**	262.3880499	-2.474436457	0.253249312	1.46E-23	6.85E-22
**SP140**	84.85640144	-2.474896675	0.320486493	1.39E-15	3.54E-14
**TTC39A**	12.33506954	-2.477148032	1.310261553	0.00423311	0.01717815
**CASZ1**	299.5074416	-2.477825026	0.181930115	4.15E-43	4.66E-41
**AC093716.1**	21.03335232	-2.478355469	0.648004539	1.83E-05	0.00013329
**DENND2A**	5.011446938	-2.483066157	1.608368938	0.00857145	0.03113222
**ENSG00000286498**	6.323168479	-2.484722782	1.294280762	0.00641066	0.02441550
**JCAD**	139.1910915	-2.488684842	0.970472293	0.00094031	0.00465968
**PDP1**	1368.474791	-2.489739696	0.144161247	8.75E-68	2.86E-65
**ENSG00000285906**	10.27157412	-2.490295205	0.881123358	0.00072598	0.00370826
**CRYM**	5.605208001	-2.495202456	1.125124842	0.00293349	0.01255187
**RIN1**	111.6935602	-2.495519805	0.267255036	1.46E-21	6.03E-20
**PIK3CD**	76.82430401	-2.495622723	0.502822701	6.25E-08	6.98E-07
**AC009133.1**	74.87514221	-2.500368047	0.454585614	3.8E-09	4.97E-08
**HMGN2P46**	17.06620793	-2.502950012	0.912442272	0.00043685	0.00235518
**BEND7**	17.03959794	-2.509146062	0.632180173	1.12E-05	8.58E-05
**ULBP2**	29.44142547	-2.509691985	0.550647268	5.83E-07	5.62E-06
**HLA-DRB5**	24.03817365	-2.509935247	0.663376413	1.59E-05	0.00011738
**FAM81A**	88.97398233	-2.512454627	0.450963259	4.53E-09	5.86E-08
**CADM3**	2.761125792	-2.512883465	2.482710631	0.01491474	0.04958601
**GJA3**	20.00988965	-2.517695141	0.684089084	2.44E-05	0.00017413
**XKRX**	7.616427935	-2.518434268	1.024082173	0.00197682	0.00892313
**MAT1A**	28.79581743	-2.518636874	0.531637468	3.42E-07	3.41E-06
**TRIM72**	3.898388645	-2.522432652	1.712881737	0.01255442	0.04290070
**AL591468.1**	6.115179055	-2.524634359	1.16449044	0.00357219	0.01488776

**GAS6-AS2**	19.75310687	-2.525668939	0.718271563	4.36E-05	0.00029630
**ZNF711**	88.20592054	-2.526046978	0.35866156	2.42E-13	5.01E-12
**TPK1**	57.42813784	-2.527213058	0.382634541	5.3E-12	9.68E-11
**INAVA**	149.0701463	-2.530786199	0.363663609	6.42E-13	1.29E-11
**ERC2-IT1**	7.009038535	-2.533102486	1.587375158	0.00548374	0.02139977
**ZBP1**	72.5877682	-2.535086167	0.446121463	1.52E-09	2.1E-08
**GRB14**	30.3422737	-2.535222107	0.550870839	7.48E-07	7.06E-06
**TYMP**	118.9338302	-2.536756819	0.298972162	5.07E-18	1.57E-16
**IL17RD**	33.5503475	-2.537374247	0.466431475	8.75E-09	1.09E-07
**LGALS9**	62.15421428	-2.53873709	0.357262522	1.99E-13	4.15E-12
**DSG3**	92.05039985	-2.538937659	0.615298554	5.22E-06	4.25E-05
**AC087477.2**	47.10180357	-2.540531637	0.50129637	4.12E-08	4.72E-07
**LINC00659**	9.74486672	-2.543092361	0.956520915	0.00099806	0.00491022
**ELK3**	903.6741784	-2.543492497	0.1488624	6.56E-66	2.04E-63
**BACE2**	1609.392002	-2.54354956	0.218222565	3.36E-32	2.47E-30
**AC009549.1**	10.7394426	-2.543619858	0.902131396	0.00045601	0.00244798
**CHST15**	97.60529512	-2.545192233	0.303233643	9.15E-18	2.78E-16
**IRAK2**	206.2690734	-2.545948107	0.40631201	3.79E-11	6.31E-10
**AC002076.1**	7.199327693	-2.546250961	1.251380941	0.00382360	0.01575433
**ENSG00000286251**	16.86980939	-2.548663837	0.736645326	7.34E-05	0.00047522
**RNU4-80P**	10.45323859	-2.548990844	0.942270109	0.0006929	0.00355485
**AL138828.1**	47.3197458	-2.551832278	0.559391267	5.77E-07	5.57E-06
**TTC39C**	100.3639918	-2.554163419	0.336858026	6.35E-15	1.53E-13
**MOB1AP1**	18.39889877	-2.55599577	0.741801891	4.87E-05	0.00032718
**AC007513.1**	5.592528669	-2.55948676	1.859617357	0.00994223	0.03526461
**RAB31**	2365.686461	-2.560901539	0.187676935	3.59E-43	4.07E-41
**MMP25**	32.56014709	-2.564936616	0.610866959	3.9E-06	3.25E-05
**GSDMA**	18.05865028	-2.565038146	0.895137734	0.00037776	0.00206547
**ATP9A**	779.9525793	-2.567474105	0.176142645	4.67E-49	6.61E-47
**NEURL1B**	128.5185811	-2.568600589	0.292877421	1.42E-19	5.02E-18
**SSUH2**	46.78882879	-2.57194916	0.47209918	5.04E-09	6.46E-08
**FGF12**	31.45980392	-2.57657369	0.530671706	2.14E-07	2.2E-06
**ENSG00000287001**	32.69293222	-2.578001969	0.593677847	2.7E-06	2.32E-05
**LINC02421**	11.97948654	-2.578070191	0.767866217	0.00010288	0.00064314
**USP2**	28.0484144	-2.579533917	0.780234424	0.00014247	0.00085799

**FMO4**	34.69937006	-2.579679777	0.579153451	1.05E-06	9.74E-06
**OR51K1P**	3.454927324	-2.580793891	1.860070835	0.01218307	0.04181701
**ENSG00000285789**	10.02254249	-2.582710554	0.881321031	0.00051480	0.00272804
**KCTD14**	13.62464141	-2.583311444	1.053877863	0.00177837	0.0081364
**AC012636.1**	3.918999435	-2.584363338	1.572686757	0.00979483	0.03482446
**CBS**	47.63674704	-2.585840545	0.422881042	1.59E-10	2.47E-09
**PEAR1**	6.909926934	-2.586324004	1.152273775	0.00254885	0.01112303
**PECAM1**	4.587835187	-2.587406761	1.370834258	0.00512068	0.02018723
**COL4A1**	57.54011321	-2.587730201	0.414303002	6.88E-11	1.11E-09
**MTMR7**	17.83680031	-2.58953681	0.620276636	3.3E-06	2.79E-05
**GLRX**	83.7425656	-2.589655034	0.445651542	1.22E-09	1.71E-08
**ST8SIA4**	427.1527035	-2.591159796	0.205158841	1.38E-37	1.24E-35
**ZNF415P1**	46.31730198	-2.592357733	0.392297427	5.57E-12	1.01E-10
**RND1**	6.408589442	-2.595318524	1.044084402	0.00154234	0.00719011
**SMPDL3B**	6.174581449	-2.595636408	1.176115343	0.00330150	0.01390506
**AL158071.2**	9.694514325	-2.596921125	1.03768076	0.00106858	0.00521108
**AL022067.1**	12.27799249	-2.599129944	1.440241976	0.00330272	0.01390529
**TMPRSS4**	89.66538499	-2.601436405	0.412001802	4.04E-11	6.68E-10
**FBXO47**	3.354784959	-2.602484791	2.088326841	0.00670920	0.02537843
**GATA4**	23.27699606	-2.602684802	0.637333752	4.03E-06	3.35E-05
**DLC1**	1095.002344	-2.605240971	0.203859966	2.73E-38	2.52E-36
**KHDC1L**	8.21768101	-2.605273676	1.399748972	0.00341582	0.01432095
**LCE3E**	4.701770086	-2.608881122	1.554331856	0.00939912	0.03365751
**CYP4F26P**	165.0568272	-2.610981804	0.331274262	7.41E-16	1.92E-14
**CYP4F11**	9.597408898	-2.611912616	0.880458918	0.00032338	0.00179399
**PCDH18**	6.808599081	-2.612117117	1.179568236	0.00224698	0.00999916
**LINC01679**	8.395066926	-2.613703823	1.005455056	0.00103930	0.00509111
**STARD9**	123.062136	-2.613763576	0.311956884	3.59E-18	1.13E-16
**KIAA1671**	1730.882244	-2.614256723	0.204135059	2.09E-38	1.94E-36
**LINC00622**	4.448449219	-2.614897893	1.927427261	0.01256149	0.04291873
**ATAD3C**	14.78514869	-2.61618423	0.767488772	9.77E-05	0.00061307
**SLC5A2**	2.531665315	-2.618115543	2.247254976	0.01341702	0.04531729
**AC124319.4**	39.43841631	-2.619533378	0.463266536	2.36E-09	3.2E-08
**MDGA2**	33.21063672	-2.619823852	0.814801465	0.00010874	0.0006755
**SH2D2A**	7.717865363	-2.620697536	1.087587726	0.00170436	0.00785191

**CDC42EP1**	317.7470651	-2.622609584	0.711702635	2.26E-05	0.00016225
**AL365181.2**	7.766252088	-2.622819399	1.300720271	0.00251313	0.01099543
**CPNE4**	2.501560701	-2.623664446	2.330469908	0.01408097	0.04722626
**ADAMTS6**	56.37408951	-2.625055819	0.468349893	2.69E-09	3.6E-08
**ARTN**	50.63294606	-2.626587842	0.434656014	2.28E-10	3.49E-09
**PAGE2B**	566.6321118	-2.627254243	0.182870309	5.9E-48	8.11E-46
**AC117394.2**	3.96055286	-2.627821253	1.932767157	0.01099177	0.03836131
**ENSG00000286048**	5.077733341	-2.629963462	1.308756889	0.00468516	0.01873910
**SERPINA1**	32.56119075	-2.630212917	0.74114715	4E-05	0.00027389
**CD180**	12.50687825	-2.632913487	0.863008474	0.00024684	0.00141031
**RGS5**	198.8001452	-2.63526746	0.307572502	1.66E-18	5.34E-17
**NDN**	73.34327867	-2.635435891	0.398090918	8.49E-12	1.52E-10
**TRPC2**	4.687708607	-2.635557556	1.310987617	0.00514461	0.02027153
**MORC2-AS1**	3.978401829	-2.636088126	1.639870999	0.01013975	0.03585352
**AL359976.1**	4.055478313	-2.638441499	2.494793502	0.00854832	0.03106668
**LINC01168**	3.764613648	-2.638819675	2.196520336	0.00625251	0.02394645
**AC108206.1**	6.859480475	-2.641916059	1.129397926	0.00210684	0.00943021
**P2RY1**	4.483972277	-2.643723119	2.259648396	0.01198072	0.04122900
**AC037450.1**	7.32148562	-2.646752271	1.425564317	0.00420757	0.01709192
**RAB38**	32.169506	-2.649190387	0.743729747	2.68E-05	0.00019001
**LINC02428**	35.13267158	-2.649921437	0.479559443	6.75E-09	8.52E-08
**WDR64**	13.37388842	-2.651568284	0.760212792	5.85E-05	0.00038743
**AC096661.1**	5.795090176	-2.652491143	1.434877196	0.00643086	0.02446491
**AL451060.1**	3.94856616	-2.654068035	1.601163518	0.00948154	0.03389203
**HCG4P8**	24.54432935	-2.654726281	0.689459149	8.28E-06	6.46E-05
**AC073343.1**	2.701454453	-2.654766803	2.263008932	0.01255384	0.04290070
**HAR1B**	4.313356169	-2.657014812	1.62056193	0.00599426	0.02311658
**VAV1**	7.812812613	-2.657536892	1.205147658	0.00316366	0.01339055
**ACSS1**	117.4090734	-2.657694179	0.271972991	1.64E-23	7.58E-22
**C1orf68**	56.5276777	-2.659600306	0.576671945	6.53E-07	6.24E-06
**AL137145.2**	15.58527707	-2.661513404	1.001567681	0.00051348	0.00272226
**CCND1**	4584.972193	-2.662315953	0.197538702	3.04E-42	3.29E-40
**TARSL2**	316.461617	-2.663963586	0.323401293	2.4E-17	7.02E-16
**SPRY2**	488.5476638	-2.664015068	0.174126362	1.12E-53	1.94E-51
**MLPH**	651.5269528	-2.664796777	0.200890364	5.11E-41	5.26E-39

**MARCKSL1**	201.9583687	-2.665777787	0.456829718	6.03E-10	8.79E-09
**AP000695.3**	7.053204627	-2.666083469	1.66920912	0.00526671	0.02067768
**MDFI**	84.27311715	-2.672323415	0.345366063	2.5E-15	6.24E-14
**BTBD19**	141.6637371	-2.672706892	0.251849419	5.11E-27	2.9E-25
**SNRPEP10**	20.51701297	-2.674283467	0.645349437	3.01E-06	2.57E-05
**CGA**	2653.790928	-2.674491823	0.307386185	4.28E-19	1.45E-17
**AP001476.1**	4.083850077	-2.674892315	2.254905995	0.00584149	0.02261130
**PCAT19**	39.27606163	-2.679335502	0.705094087	1.76E-05	0.00012897
**CBX1P3**	9.877121513	-2.681762604	1.283369903	0.00190795	0.00865309
**OR10Y1P**	14.61881113	-2.682408655	0.839721648	9.51E-05	0.00059827
**SH3BP2**	335.5770081	-2.683808759	0.279388491	6.93E-23	3.08E-21
**AL596223.1**	8.454580088	-2.683823807	1.24564797	0.00191136	0.00866469
**NCALD**	26.34350179	-2.683964867	0.582853538	4.53E-07	4.45E-06
**AC083967.1**	9.848427976	-2.684382127	0.990835934	0.00093591	0.00464171
**PLEKHN1**	177.2811632	-2.684912933	0.257715681	5.54E-26	2.94E-24
**MIPOL1**	73.02705934	-2.686369783	0.311235007	8.34E-19	2.76E-17
**TRIM46**	14.95518721	-2.686393994	0.686364593	1.24E-05	9.39E-05
**TMEM51**	224.6911887	-2.687094774	0.291391491	2.02E-21	8.21E-20
**NET1**	1050.215728	-2.687414416	0.179474004	1.77E-51	2.8E-49
**LINC00514**	15.23575951	-2.689196601	0.820918162	8.35E-05	0.00053318
**MUC20**	13.70145036	-2.689626728	0.871661342	0.00017874	0.00105392
**MYPN**	1612.294761	-2.693171321	0.312023807	7.78E-19	2.59E-17
**MX2**	235.5769685	-2.69711914	0.433197327	4.86E-11	7.95E-10
**C1orf158**	88.61450927	-2.697572729	0.454465634	3.5E-10	5.22E-09
**AC099518.4**	5.668207	-2.699306267	2.299971572	0.00972407	0.03462435
**AP006248.3**	4.734098631	-2.699796786	1.679425339	0.00848310	0.03086893
**AL445250.1**	4.981945966	-2.701954619	1.428794052	0.00623747	0.02390416
**B3GNT3**	449.2634953	-2.704601998	0.296885926	1.52E-20	5.84E-19
**LRCH2**	22.63233477	-2.705613021	0.756299273	2.87E-05	0.00020222
**TSPAN1**	1334.931929	-2.705784395	0.166041491	2.17E-60	5.05E-58
**CXCL1**	9.916609184	-2.708520105	1.077771751	0.00104091	0.00509794
**CBLC**	30.17193707	-2.71094055	0.732543743	3.49E-05	0.00024234
**ENSG00000286023**	20.07889661	-2.718631878	0.789969326	4.29E-05	0.00029214
**NEURL3**	28.8198863	-2.719774178	0.731595421	1.59E-05	0.00011736
**LINC02432**	15.10416237	-2.721832976	0.788735545	8.41E-05	0.00053619

**AC055813.1**	3.800795436	-2.721930216	1.751446651	0.00861564	0.03124526
**AC233702.1**	4.683921116	-2.722444908	1.649522403	0.00802452	0.02953609
**GNA15**	155.5148809	-2.725888684	0.405370293	1.8E-12	3.47E-11
**TNFRSF10A**	419.6086378	-2.727493201	0.262365696	4.98E-26	2.67E-24
**IDO1**	693.9689303	-2.728474745	0.230413149	3.76E-33	2.91E-31
**MX1**	2250.946903	-2.729406542	0.349085747	6.03E-16	1.58E-14
**ABLIM1**	1030.689715	-2.732102575	0.266970127	2.36E-25	1.21E-23
**AC007342.9**	16.74167071	-2.736519343	0.677513213	7.51E-06	5.94E-05
**SNX18P3**	19.34866854	-2.73824127	0.663487523	5.58E-06	4.51E-05
**DNAH3**	23.11200194	-2.738531095	0.55815633	1.67E-07	1.75E-06
**USP18**	268.236618	-2.738736998	0.268281627	1.6E-25	8.25E-24
**ENSG00000286511**	16.50054591	-2.741244978	0.631499068	2.19E-06	1.92E-05
**COL6A1**	152.823795	-2.742057547	0.359804541	1.86E-15	4.68E-14
**AC015921.1**	4.535334087	-2.74221253	1.760884789	0.00730692	0.02730673
**LRRC75A**	37.69268056	-2.743634072	0.524486641	1.23E-08	1.5E-07
**ENSG00000287320**	62.72150351	-2.745379353	0.365416555	6.37E-15	1.53E-13
**ENSG00000286850**	3.930671575	-2.747461501	3.116604612	0.01178885	0.04069758
**AL450990.1**	13.74108068	-2.748862143	0.709827562	1.7E-05	0.00012533
**NOV**	14.49772139	-2.75138196	1.010997324	0.00069648	0.00357128
**PROCR**	180.3383127	-2.753442883	0.487439302	2.37E-09	3.21E-08
**PHYHD1**	4.374169039	-2.753748338	1.393553708	0.00490516	0.01945305
**AL591742.2**	105.4636214	-2.760106123	2.170794578	0.00566568	0.02200917
**TMCC2**	9.811327984	-2.76093687	1.111844128	0.00114986	0.00555420
**PCDH7**	750.5665081	-2.766035192	0.228866727	1.1E-34	8.92E-33
**AC073316.1**	5.352163892	-2.766208183	1.720956792	0.00509159	0.02008579
**SNX29P1**	10.96803273	-2.76706642	0.861823062	0.00012261	0.00075015
**AC133785.1**	18.48907861	-2.769778415	0.696657397	8.96E-06	6.95E-05
**CD177**	25.13176124	-2.773087343	0.62047421	1.1E-06	1.01E-05
**DOCK6**	75.96190039	-2.775408312	0.491765419	1.22E-09	1.7E-08
**AC068587.4**	16.25997448	-2.778395327	0.860003645	7.79E-05	0.00050127
**MUC16**	16827.5931	-2.781151483	0.176738235	1.07E-56	2.02E-54
**VOPP1**	109.5006109	-2.783773725	0.365241607	3.04E-15	7.55E-14
**CSTA**	165.1506606	-2.785826559	0.2862322	5.67E-23	2.53E-21
**ADGRE1**	125.4647613	-2.786821442	0.262143566	3.87E-27	2.21E-25
**EPHB6**	40.6785632	-2.78738144	0.578796243	2.65E-07	2.7E-06

**LINC02081**	5.481550608	-2.790647812	1.470362923	0.00554365	0.02159831
**ZFPM2**	4.044251638	-2.799665028	2.175629369	0.00510815	0.02014448
**TMEM173**	14.72810113	-2.80030921	0.86883956	0.00013196	0.00080182
**AC022784.1**	23.83310548	-2.802876703	0.664113458	2.08E-06	1.83E-05
**PLEKHH1**	135.8465675	-2.808060754	0.371466747	4.25E-15	1.04E-13
**KDR**	5.530799255	-2.809276821	1.655110958	0.00386241	0.01588959
**RPLP0P2**	61.69391735	-2.810174059	0.493126645	1.57E-09	2.17E-08
**PDCD1LG2**	573.7117325	-2.810277268	0.184421564	3.81E-53	6.45E-51
**IL7**	13.77368255	-2.814423831	0.765191751	2.25E-05	0.00016173
**GJB4**	578.278717	-2.817103811	0.312313158	1.72E-20	6.54E-19
**NCAM1**	4.435248003	-2.820085949	1.654108201	0.00577570	0.02238203
**INPP4B**	1100.312176	-2.820784178	0.263105293	6.26E-28	3.69E-26
**FOXQ1**	41.73211282	-2.822819238	0.560171545	8.34E-08	9.12E-07
**ENSG00000287047**	1.997890151	-2.825698333	4.466462863	0.01334560	0.04512067
**AL359834.1**	27.25347464	-2.825846158	0.596484692	2.45E-07	2.5E-06
**RN7SL734P**	3.799710185	-2.828876254	1.671688567	0.00696602	0.02620479
**LINC01271**	2.841138976	-2.829876151	2.239714401	0.01063690	0.03733535
**ANKRD18A**	52.32521103	-2.831767952	0.395586301	1.86E-13	3.9E-12
**AP000695.1**	16.21755191	-2.833046919	0.894493174	9.95E-05	0.00062329
**SLC4A9**	15.23150273	-2.83341843	0.797352645	4.5E-05	0.00030424
**ATP6V0A4**	15.62126694	-2.835019604	0.865464246	0.00016179	0.00096255
**RPS26P28**	10.99078405	-2.836204283	0.81507028	7.41E-05	0.00047861
**TEC**	121.3352749	-2.84274672	0.33929388	7.28E-18	2.23E-16
**FAM83E**	22.09513988	-2.843314719	0.835308222	6.96E-05	0.00045273
**AIM2**	2043.305523	-2.846430579	0.168114316	3.34E-65	9.87E-63
**SMPX**	4.801521472	-2.846492349	1.391370276	0.00372119	0.01540404
**AP003174.1**	19.40935877	-2.848729297	0.632640636	8.66E-07	8.09E-06
**TNFSF8**	3.79997913	-2.849745661	1.593574285	0.00592209	0.02287517
**AC002480.3**	4.068121502	-2.850520476	1.649144129	0.00526121	0.02065948
**LINC01783**	6.72839337	-2.850762083	1.252429355	0.00271629	0.01174181
**AL078590.3**	16.34557661	-2.851474002	0.691977425	4.08E-06	3.38E-05
**GRK5-IT1**	13.61382832	-2.851596547	0.829322241	6.14E-05	0.00040492
**HAR1A**	13.32876075	-2.864144339	0.816262877	4.16E-05	0.00028385
**DOC2A**	35.68699125	-2.870625709	0.612456235	1.98E-07	2.05E-06
**PLAT**	238.8599665	-2.871407498	0.244460866	1.41E-32	1.05E-30

**ILDR1**	69.79254043	-2.875209661	0.45246494	4.29E-11	7.06E-10
**OGDHL**	11.57611106	-2.882088432	0.922726807	0.00025972	0.00147507
**MUC1**	2287.641918	-2.882304242	0.278025101	3.59E-26	1.95E-24
**BRDT**	3.141397228	-2.883639812	2.597750783	0.01154739	0.03999104
**LIPH**	253.8341371	-2.883721756	0.335586709	1.34E-18	4.38E-17
**KLK11**	4.903609351	-2.884989669	1.397050412	0.00367936	0.01526259
**RNU6-26P**	40.21939695	-2.885573983	0.562713468	1.91E-08	2.29E-07
**F3**	6482.114967	-2.885826939	0.729461892	6.69E-06	5.34E-05
**KATNAL1**	272.3183501	-2.888865795	0.372521973	6.05E-16	1.58E-14
**AL049629.1**	18.61769233	-2.891385203	1.03991789	0.00027934	0.00157376
**SRXN1**	4.888104105	-2.892056411	1.597136538	0.00597250	0.02304755
**AL513328.1**	2.027725821	-2.893235357	4.459282092	0.01299834	0.04415889
**GUCY2D**	14.18672031	-2.893578268	0.710500821	7.06E-06	5.62E-05
**AC087894.1**	6.943948751	-2.894044955	1.204611889	0.00115850	0.00558915
**DNAH17-AS1**	2.087666104	-2.896986438	4.343881192	0.01264183	0.04311931
**LINC00520**	27.17442147	-2.90364548	0.680517827	1.29E-06	1.18E-05
**AGMO**	9.338096746	-2.903830666	1.066274148	0.00070990	0.00363385
**SAA1**	75.38178134	-2.906688919	0.453786802	1.59E-11	2.78E-10
**KCNK9**	16.48439802	-2.906837048	0.724359889	5.73E-06	4.63E-05
**ZIC5**	12.76267884	-2.907845866	0.797071738	4.19E-05	0.00028603
**ADAMTS17**	4.29009942	-2.907995392	1.832784474	0.00733528	0.02739132
**DPYSL4**	3.495933387	-2.911435543	2.100033068	0.00633100	0.02418893
**ENSG00000286288**	27.97382505	-2.912093848	0.610494336	1.63E-07	1.72E-06
**SOX7**	127.7616265	-2.917451662	0.263643815	2.65E-29	1.68E-27
**MRGPRE**	3.026128271	-2.919378222	8.47542519	0.01035149	0.03646738
**RHOB**	209.9830221	-2.921007459	0.370431535	2.7E-16	7.24E-15
**CT83**	94.40168821	-2.921169963	0.31389164	2.74E-21	1.11E-19
**LINC02241**	169.6651701	-2.921430557	0.269490833	3.63E-28	2.17E-26
**ENSG00000286751**	27.08146074	-2.924416919	0.621552721	4.75E-07	4.64E-06
**SH3PXD2A-AS1**	25.63818908	-2.924628159	0.620751817	1.79E-07	1.87E-06
**AKAP7**	26.89707781	-2.927280803	0.612189447	2.66E-07	2.71E-06
**KLHDC7B**	498.0316974	-2.927357866	0.24000118	5.15E-35	4.23E-33
**C1orf116**	2562.573174	-2.92788282	0.26998743	2.12E-28	1.28E-26
**LPAR5**	25.45777644	-2.930564801	0.726691115	3.81E-06	3.19E-05
**AC140479.7**	167.74439	-2.933196122	0.26512309	4.31E-29	2.69E-27

**SLCO2A1**	133.98294	-2.933606174	0.487897235	2.71E-10	4.1E-09
**45539**	49.92424131	-2.938220785	0.453682323	4.91E-12	9.01E-11
**RASSF10**	15.9482487	-2.941323674	0.720569861	5.89E-06	4.75E-05
**NKD1**	9.907662721	-2.943176736	0.866427791	7.44E-05	0.00048006
**SYT5**	98.29317044	-2.94354066	0.311759358	4.2E-22	1.8E-20
**SPOCK1**	601.0342893	-2.946436343	0.344108634	1.43E-18	4.65E-17
**CCDC152**	54.02177151	-2.947088731	0.447133463	4.33E-12	7.98E-11
**LSAMP**	3.964842097	-2.947843167	1.786037992	0.0072728	0.0272092
**HOXB13**	5.708667361	-2.948517285	1.430274169	0.0030865	0.0131083
**MEGF10**	3.466097717	-2.949546859	2.047187852	0.0058508	0.0226398
**RNU6-1331P**	7.797898685	-2.952133217	1.045226046	0.0006641	0.0034275
**ATP1B2**	2.89131649	-2.952578506	2.136305355	0.0088461	0.0319695
**LIVAR**	3.090941297	-2.959734573	2.220017523	0.0089514	0.0322869
**PTPRZ1**	219.5196595	-2.960430857	0.751896823	7.65E-06	6.03E-05
**DCBLD2**	5336.836468	-2.962643227	0.123647283	9.92E-128	2.64E-124
**GUCY1B1**	6.194618206	-2.962728289	1.292111862	0.0026273	0.0114032
**PPP1R1C**	36.71161961	-2.964212038	0.511509003	6.6E-10	9.57E-09
**ITGA2**	13622.91312	-2.97028288	0.191644998	4.18E-55	7.58E-53
**TSPO**	68.97244508	-2.97223461	0.360516252	2.09E-17	6.15E-16
**CMPK2**	333.0706806	-2.975189831	0.314212043	2.48E-22	1.07E-20
**STC1**	4794.287603	-2.975226234	0.80501847	1.78E-05	0.0001305
**FOXG1-AS1**	10.53243661	-2.977650686	1.374107342	0.0013182	0.0062538
**HCG4P7**	4.588671631	-2.977845307	1.53327566	0.0051527	0.0202970
**SIRPG**	18.74540072	-2.979368049	0.651287812	5.08E-07	4.94E-06
**RHBDF2**	319.0340175	-2.981012464	0.201386259	3.12E-50	4.58E-48
**LINC00887**	43.70629039	-2.981831884	2.018020073	0.0042277	0.0171630
**TTC34**	9.740141471	-2.982190487	0.982789587	0.0001691	0.0010020
**CCNJL**	27.54996142	-2.982639596	0.924587294	0.0001248	0.0007626
**NCS1**	84.23861014	-2.986257147	0.358886653	1.22E-17	3.66E-16
**CAMK4**	100.6680303	-2.991287929	0.33401142	5.5E-20	2E-18
**AL138902.1**	3.705008062	-2.99488038	1.770146593	0.0072834	0.0272431
**45355**	641.6225885	-3.000813024	0.36655773	3.81E-17	1.1E-15
**APOBEC3G**	244.3934164	-3.000826921	0.227310369	4.01E-41	4.15E-39
**BACE2-IT1**	63.28359814	-3.003954404	0.383459722	6.12E-16	1.6E-14
**SERPINE2**	861.7131078	-3.004756271	0.358210619	4.36E-18	1.36E-16

**AL356234.2**	13.07360123	-3.004827372	0.917833314	8.61E-05	0.00054753
**AL355312.3**	5.830510145	-3.00764617	1.603540368	0.00257723	0.01121822
**AC037487.3**	2.881553721	-3.01136791	2.142140662	0.00854951	0.03106668
**MALL**	127.174384	-3.011620262	0.308691961	3.72E-23	1.69E-21
**RGS2**	350.4925612	-3.012052741	0.249943972	1.18E-34	9.59E-33
**ICAM4**	9.487524483	-3.013433834	1.096800821	0.00049776	0.00264773
**EDN1**	445.7182455	-3.013770403	0.316872199	1.59E-22	6.95E-21
**AC132825.3**	20.26054343	-3.016991652	0.6467925	2.49E-07	2.54E-06
**AL592293.1**	9.280705618	-3.017721045	0.973349944	0.00026709	0.00151151
**AP000997.1**	5.167213678	-3.020378708	1.810668739	0.00717264	0.02689743
**ENSG00000285517**	14.16951183	-3.022550651	0.902114243	0.00011643	0.00071679
**PLCB1-IT1**	7.168874035	-3.023174192	1.180146508	0.00135854	0.00642585
**TUBB2A**	88.43048795	-3.025230149	0.323912357	6.23E-22	2.65E-20
**RSAD2**	675.195008	-3.030026024	0.383046771	2.26E-16	6.11E-15
**SYT7**	43.68015077	-3.030380315	0.590583631	5.64E-08	6.35E-07
**GALNT5**	39.45487865	-3.034910131	0.688200707	1.14E-06	1.04E-05
**CACNA1G**	3.301145179	-3.03493479	2.280067239	0.00843348	0.03072914
**GSDMC**	79.86917876	-3.035057094	0.334967841	3.01E-20	1.12E-18
**MEIS3P1**	3.286545811	-3.039364444	1.983461514	0.00527705	0.02070807
**AC126603.1**	4.388174237	-3.041850839	1.603034342	0.00362437	0.01507110
**AADACL3**	1523.290728	-3.04633118	0.330096691	2.83E-21	1.14E-19
**SERPINB7**	461.3610102	-3.04905469	0.258292913	6.45E-33	4.93E-31
**PTAFR**	246.1556107	-3.052855206	0.326367912	1.25E-21	5.2E-20
**ANP32C**	3.201068567	-3.053167511	2.466356144	0.00981790	0.03490130
**AL357060.1**	239.5875745	-3.053286441	0.381354353	7.14E-17	2E-15
**CYP4A22-AS1**	5.738781447	-3.054929072	1.449132992	0.00286331	0.01229339
**PIWIL2**	9.199774232	-3.055905597	1.264842829	0.00125583	0.00599220
**TMSB4XP8**	14.3091306	-3.057621785	0.799328174	1.86E-05	0.00013531
**PURPL**	393.1870677	-3.058335572	0.260485672	1.4E-32	1.05E-30
**LINC01363**	3.546110901	-3.058418429	1.999555836	0.00495171	0.01961488
**AL512274.1**	52.06056889	-3.061535971	0.407780182	6.1E-15	1.48E-13
**PA2G4P2**	2.587549164	-3.063161593	5.677091973	0.01110039	0.03869522
**ENSG00000287043**	2.587549164	-3.063161593	5.677091973	0.01110039	0.03869522
**STEAP2**	27.91927487	-3.064572159	0.657499357	3.82E-07	3.78E-06
**LINC00704**	6.898884593	-3.0676314	1.731002813	0.00274036	0.01183563

**NLRP4**	4.253436022	-3.072347961	1.919322969	0.00740186	0.02756676
**BX072579.2**	5.117026692	-3.073687559	1.587755005	0.00471890	0.01885195
**ETV4**	130.3644579	-3.075685918	0.377025935	6.42E-17	1.8E-15
**NAALADL2**	47.14764687	-3.078073615	0.435566023	1.59E-13	3.36E-12
**FABP6**	10.71194636	-3.079590647	1.058931546	0.00050775	0.00269605
**TFPI**	174.5329324	-3.080004631	0.305786582	4.82E-25	2.44E-23
**NUGGC**	18.97460398	-3.08162618	0.735802835	1.76E-06	1.57E-05
**AL160408.1**	31.64368566	-3.08632805	1.90411963	0.00352350	0.01471305
**ENSG00000287963**	18.53876558	-3.087211207	0.911454719	5.63E-05	0.00037350
**CMTM7**	60.46531753	-3.089647792	0.529178543	5.59E-10	8.18E-09
**HSD11B1**	13.7902677	-3.093138175	0.90993301	7.09E-05	0.00046031
**SEZ6L2**	7.977136031	-3.095937578	1.246913842	0.00144207	0.00677273
**HTR4**	3.17095448	-3.099716131	2.130288436	0.00732624	0.02736611
**LINC00239**	3.446572179	-3.10005084	2.002796642	0.00492362	0.01951979
**ENSG00000286176**	5.638102386	-3.101685755	1.897624734	0.00519757	0.02043980
**FREM1**	37.48990908	-3.103066723	0.532208082	7.6E-10	1.09E-08
**TNF**	163.6116478	-3.103670953	0.342974368	1.54E-20	5.91E-19
**QPCT**	17.83285593	-3.104448737	0.770239035	3.59E-06	3.01E-05
**AC025580.2**	45.07854305	-3.105923317	0.736795601	1.56E-06	1.41E-05
**LTA**	170.9464869	-3.107231683	0.425618843	3.16E-14	7.15E-13
**AC083837.1**	270.6959815	-3.107524058	0.346730286	1.62E-20	6.17E-19
**BATF2**	725.1669578	-3.108060248	0.166738963	2.05E-78	8.89E-76
**IL36B**	4.269210213	-3.108618884	1.588489227	0.00394355	0.01615945
**FAR2P1**	16.46667167	-3.109756688	0.778728335	7.65E-06	6.03E-05
**45352**	26.51364087	-3.110706681	0.57264865	6.85E-09	8.64E-08
**ARL4C**	408.032903	-3.115922408	0.691543578	5.95E-07	5.72E-06
**HRH2**	16.36670643	-3.118540883	0.778034651	5.3E-06	4.3E-05
**UBE2SP1**	4.773920399	-3.125985617	1.915470224	0.00625245	0.02394645
**ENSG00000287579**	2.137843619	-3.134015863	4.033525648	0.01124974	0.03911317
**MICB**	599.0269029	-3.136075555	0.249655342	1.95E-37	1.73E-35
**AL033504.1**	45.96624753	-3.137758904	0.426832733	2.24E-14	5.14E-13
**ZCCHC24**	12.05656597	-3.139402332	0.830646302	2.35E-05	0.00016799
**OAF**	217.2369847	-3.140325038	0.340694654	5.31E-21	2.1E-19
**EREG**	3309.049493	-3.142441877	0.261361876	3.13E-34	2.5E-32
**TSSC2**	16.15521517	-3.143233363	0.888785159	2.48E-05	0.00017677

**45538**	31.58555365	-3.144238968	0.635165414	5.41E-08	6.1E-07
**AC078820.1**	26.38610784	-3.146902082	0.549679665	1.56E-09	2.15E-08
**SLC8A1-AS1**	14.94216665	-3.146926985	0.876498444	3.92E-05	0.0002698
**OR51I1**	4.797790262	-3.147018345	1.42270316	0.0030373	0.0129381
**NCF2**	373.8524917	-3.151915778	0.305051049	6.84E-26	3.61E-24
**AC016687.3**	26.57697398	-3.15361011	0.72765101	1.61E-06	1.45E-05
**ENSG00000285864**	2.367304095	-3.157341826	4.31892549	0.0107965	0.0377848
**CD33**	27.25209221	-3.160234486	0.744841333	1.23E-06	1.12E-05
**GAS6**	149.3899368	-3.160417261	0.304668416	6E-26	3.18E-24
**LMCD1**	35.99624734	-3.162389952	0.568189089	2.55E-09	3.42E-08
**TRIM7**	90.68635664	-3.168893379	0.298559843	3.95E-27	2.26E-25
**SORCS2**	59.96481365	-3.172678778	0.539061681	4.44E-10	6.57E-09
**COL27A1**	32.48503514	-3.173897192	0.741358696	2.67E-06	2.3E-05
**MROH6**	294.5854821	-3.174342647	0.199279839	7.39E-58	1.48E-55
**LINC02484**	329.8370849	-3.177686613	0.261990988	1.42E-34	1.14E-32
**ENSG00000287554**	56.32132361	-3.17965506	0.531240533	4.02E-10	5.98E-09
**RYR3**	68.06179778	-3.187974013	0.543809616	4.33E-10	6.41E-09
**MIR9-3HG**	8.901761707	-3.188654179	1.123463651	0.0005963	0.0031166
**TFF2**	4.964104809	-3.194287294	2.450340838	0.0052426	0.0205966
**TMSB4X**	3707.566312	-3.194926782	0.551091329	6.1E-10	8.87E-09
**ALOX12B**	7.467981283	-3.199483394	1.071749292	0.0003375	0.0018666
**CD86**	16.15197683	-3.20040353	0.786840157	7.55E-06	5.96E-05
**RAP1GAP**	41.37893907	-3.205825846	0.569344086	1.46E-09	2.02E-08
**MLKL**	371.6398901	-3.206292028	0.23833766	1.02E-42	1.13E-40
**PLAC1**	4.354876302	-3.212134869	1.798957787	0.0054473	0.0212787
**EVC**	508.5705426	-3.212334934	0.175327045	5.78E-76	2.34E-73
**LAMC2**	17392.316	-3.214550194	0.193474502	5.89E-63	1.55E-60
**TESMIN**	76.2079082	-3.21486953	0.41717723	1.14E-15	2.92E-14
**EPHX2**	12.74287262	-3.216394938	0.827150089	1.13E-05	8.6E-05
**SLC37A2**	841.1041796	-3.22024529	0.183339201	3.79E-70	1.33E-67
**LOXL1**	8.415585873	-3.222270458	1.282361363	0.0008340	0.0041850
**E2F6P4**	5.07855746	-3.224859901	1.6404287	0.0032573	0.0137432
**BAMBI**	21.19447808	-3.237595029	0.730080078	5.02E-07	4.89E-06
**FGD2**	8.599751049	-3.245270529	1.066110186	0.0002185	0.0012623
**PADI3**	14.90663232	-3.245651722	0.890455293	1.37E-05	0.0001026

**EVC2**	37.4270149	-3.246734167	0.487428773	4.19E-12	7.74E-11
**WNT5B**	3.111014197	-3.247738775	2.02010534	0.00586999	0.02270695
**AC003659.1**	4.38416461	-3.250963911	1.64539695	0.0039080	0.0160441
**AL353726.2**	15.18430654	-3.252062494	1.034806456	0.0001713	0.0010136
**MEGF6**	589.40957	-3.252215778	0.319000784	1.32E-25	6.87E-24
**AC007336.1**	7.064184154	-3.255552687	1.164735179	0.0007340	0.0037428
**AC002401.4**	15.69385263	-3.256791038	0.753914297	2.16E-06	1.9E-05
**AC209154.2**	38.33738028	-3.270613878	0.485848061	2.36E-12	4.49E-11
**DGAT2**	39.63037423	-3.272671251	0.491984117	5.15E-12	9.42E-11
**PLCB1**	312.4371375	-3.273623606	0.257706827	1.04E-37	9.45E-36
**MYOZ2**	9.689681858	-3.274717879	1.149768725	0.0005274	0.0027862
**GABBR1**	266.7897589	-3.275602522	0.267105801	1.27E-35	1.07E-33
**MGAT5B**	5.187008161	-3.290498234	1.443344769	0.0025002	0.0109490
**ABCA6**	11.64512586	-3.290860395	1.043887978	0.0001322	0.0008031
**RCN3**	9.366156191	-3.294634165	0.953907465	8.85E-05	0.0005616
**TNFAIP3**	5091.928872	-3.296063673	0.255322317	4.04E-39	3.9E-37
**C15orf48**	159.3839901	-3.298622608	0.846293772	6.62E-06	5.3E-05
**PRKCQ-AS1**	45.07875532	-3.304789617	0.563401298	3.19E-10	4.8E-09
**RPL23AP32**	6.927305407	-3.304937224	1.393591029	0.0013238	0.0062779
**COL6A3**	81.34408008	-3.306525666	0.344876492	8.99E-23	3.98E-21
**LAPTM5**	60.42998079	-3.310994265	0.561573562	5.49E-10	8.04E-09
**CTSS**	1746.650274	-3.312357571	0.240288785	2.37E-44	2.77E-42
**IL18R1**	26.87403606	-3.313919421	0.604576365	5.54E-09	7.04E-08
**CGREF1**	12.3854738	-3.314938799	1.165075502	0.0001913	0.0011197
**PAK1**	242.2100932	-3.321970071	0.224191571	1.03E-50	1.53E-48
**GOLT1A**	42.72354835	-3.324537432	0.456113776	6.29E-14	1.37E-12
**TMEM45B**	23.69109344	-3.325336825	0.883742111	2.5E-05	0.0001784
**ENSG00000287382**	4.738710241	-3.32587958	1.538063976	0.0024132	0.0106225
**AL138760.1**	2.337199481	-3.332185257	4.170221072	0.0103016	0.0363218
**MTND6P8**	2.337199481	-3.332185257	4.170221072	0.0103016	0.0363218
**AC084026.1**	2.337199481	-3.332185257	4.170221072	0.0103016	0.0363218
**DPYD**	1521.385206	-3.332795564	0.177737205	3.94E-79	1.78E-76
**NT5DC4**	69.70958684	-3.333628987	0.373626844	3.63E-20	1.34E-18
**MAGEA11**	41.06310209	-3.33393618	0.504381579	2.89E-12	5.43E-11
**RPL10P13**	6.501389626	-3.334672818	1.367186369	0.0017330	0.0079610

**CPM**	67.28821028	-3.335520964	0.450661251	9.84E-15	2.32E-13
**AC005150.1**	6.689887996	-3.3412183	1.302445829	0.00124719	0.00595934
**AL358176.4**	11.43919332	-3.345421723	0.855832442	8.95E-06	6.95E-05
**RP1**	2.197514958	-3.345795452	3.890434162	0.01017727	0.03594896
**ENSG00000287211**	10.05126788	-3.34897241	0.947720713	4.04E-05	0.00027678
**DPYD-IT1**	10.49808957	-3.354660296	1.026559931	6.1E-05	0.00040264
**TRERF1**	194.083845	-3.355770316	0.326991445	1.5E-25	7.77E-24
**AC007952.2**	2.167679288	-3.361324544	3.798923163	0.00999669	0.03543312
**HRH1**	266.7576634	-3.362517121	0.406326345	1.22E-17	3.66E-16
**LINC00377**	3.056232885	-3.362535741	5.436191205	0.00945109	0.03380844
**ICAM1**	4101.669705	-3.366913115	0.26374047	2.25E-38	2.09E-36
**BX284668.5**	7.878670235	-3.367852145	1.109010313	0.00021437	0.00124108
**TENM4**	28.37808064	-3.374612604	0.533355659	2.42E-11	4.13E-10
**CCDC26**	4.583520472	-3.376042382	1.639160979	0.00326312	0.01376036
**ENSG00000287148**	3.226290967	-3.376284263	5.429236964	0.00901331	0.03245606
**DIRC3-AS1**	8.722532174	-3.37664435	0.99816767	0.00011189	0.00069252
**RIPPLY3**	3.271040565	-3.377839024	1.985316434	0.00479362	0.01908031
**AC011155.1**	3.271040565	-3.377839024	1.985316434	0.00479362	0.01908031
**ARHGEF4**	434.2083853	-3.378378514	0.703970592	1.34E-07	1.43E-06
**AC092306.1**	7.729288696	-3.380946899	1.050538569	0.00019001	0.00111353
**ITK**	25.69775257	-3.381554899	0.623147813	3.18E-09	4.22E-08
**HCP5**	2942.712947	-3.391038503	0.156382881	2.63E-105	2.25E-102
**AF131216.5**	2.237929703	-3.392392364	3.974111478	0.01004804	0.03558713
**TNNI3**	8.20848574	-3.39363549	1.137829376	0.00023803	0.00136552
**PDGFC**	84.03565413	-3.393730293	0.422361119	2.2E-16	5.98E-15
**FZD4**	39.5179685	-3.395348103	0.507234899	2.39E-12	4.55E-11
**MIR4635**	2.827588715	-3.398381235	5.112147164	0.00973958	0.03466409
**LINC00861**	50.43932554	-3.399363205	0.522583429	3.74E-12	6.95E-11
**AL022315.1**	22.36451508	-3.406535854	0.614565655	2.87E-09	3.83E-08
**MYO10**	3109.357784	-3.407042717	0.255776809	1.87E-41	1.96E-39
**ENSG00000287927**	4.343759337	-3.407207906	1.675406917	0.00363514	0.01511061
**AJ003147.2**	5.417553889	-3.409485661	1.359961539	0.00148903	0.00696999
**IL22RA1**	166.6713653	-3.412497104	0.333566612	1.15E-25	6.03E-24
**KCTD17**	63.55274155	-3.413608636	0.408718634	1.57E-17	4.67E-16
**MFGE8**	212.5196538	-3.413720711	0.294445209	7.71E-32	5.57E-30

**HBEGF**	1229.325087	-3.414787046	0.641350227	8.41E-09	1.05E-07
**LINC01748**	72.49269371	-3.417813019	0.348505667	6.85E-24	3.24E-22
**CKB**	67.61100207	-3.419169657	0.416634096	1.12E-17	3.36E-16
**DUSP4**	2940.48807	-3.424243537	0.182649607	2.82E-79	1.3E-76
**PDE4B**	169.2542943	-3.439702963	0.448190289	2.69E-15	6.7E-14
**RGS20**	199.870381	-3.439817185	0.248454643	1.2E-44	1.43E-42
**HYDIN**	47.94129308	-3.439842621	0.438165901	5.86E-16	1.54E-14
**ENSG00000285888**	12.71475036	-3.443314736	0.895619703	1.4E-05	0.00010428
**PDPN**	29.24384237	-3.443599272	0.839332395	1.79E-06	1.59E-05
**ROR2**	8.670547166	-3.445734977	1.436178882	0.0009369	0.0046456
**PTGS2**	8217.147812	-3.446665645	0.230828173	2.02E-51	3.15E-49
**CNKSR3**	63.50491105	-3.448265414	0.428680041	1.61E-16	4.4E-15
**HR**	14.54306974	-3.450772216	0.942140129	3.92E-05	0.0002701
**AC013268.4**	164.022635	-3.461951262	0.263228384	5.49E-40	5.49E-38
**AC123788.1**	8.74000745	-3.462400676	1.483206199	0.0009893	0.0048713
**GNB4**	350.987902	-3.469517615	0.210864185	2.37E-62	6.02E-60
**BOK**	75.21297741	-3.471054695	0.353109634	2.65E-23	1.21E-21
**GRIA1**	9.588407434	-3.471222007	1.091130025	0.0001251	0.0007640
**AC025459.1**	3.311186365	-3.477031428	2.035605247	0.0047560	0.0189624
**SPRED1**	172.5789519	-3.477503985	0.303038469	3.83E-31	2.69E-29
**PHLDA1**	2539.992886	-3.480684153	0.264101935	1.46E-40	1.48E-38
**AC097374.1**	107.3344465	-3.483353594	0.423053483	1.48E-17	4.41E-16
**AC105046.1**	2.617106416	-3.48382284	4.055641387	0.0088557	0.0319946
**IL23A**	39.69592794	-3.485952585	0.587606507	2.26E-10	3.45E-09
**SORD2P**	39.76416827	-3.489630209	0.526934006	5.77E-12	1.05E-10
**MUC13**	11.57229524	-3.498674886	0.983448552	6.1E-05	0.0004026
**RBFOX1**	6.50717606	-3.499160692	1.217842702	0.0004238	0.0022960
**RSPO4**	13.81008171	-3.499605737	0.819148949	2.89E-06	2.48E-05
**LY6E-DT**	18.09153976	-3.501144163	0.743712129	3.55E-07	3.54E-06
**ABCC2**	433.7543814	-3.501919105	0.289078065	7.6E-35	6.2E-33
**LINC02539**	14.19231822	-3.503268019	0.837621274	5E-06	4.08E-05
**CSF2**	3.330442959	-3.503944395	2.005484685	0.0044413	0.0179259
**RGS10**	202.6452892	-3.506015168	0.243558257	1.98E-47	2.66E-45
**FUT3**	319.3645923	-3.518008395	0.296847536	7.98E-34	6.28E-32
**PYCARD**	26.16956527	-3.519724216	0.572029507	1.17E-10	1.84E-09

**APOBEC3F**	278.6472602	-3.521205355	0.23301376	5.14E-53	8.66E-51
**KCNS3**	14.5597453	-3.522083742	0.972781035	1.96E-05	0.00014248
**ZEB1**	23.98333538	-3.526648732	0.745370036	1.45E-07	1.54E-06
**BX284668.2**	18.06219293	-3.527401783	0.698560483	4.29E-08	4.9E-07
**LINC00668**	56.97402387	-3.532052295	0.416080761	3.19E-18	1.01E-16
**AC091564.1**	2.647220503	-3.534226007	4.523306812	0.00947949	0.03388976
**LPAR3**	23.00623373	-3.535357138	0.716107132	6.65E-08	7.39E-07
**AP001148.1**	5.687094412	-3.544795201	1.476251846	0.00102139	0.00501262
**GABRG3**	2.187752189	-3.546742749	3.728906841	0.00919904	0.03304029
**TRBJ2-1**	8.132837018	-3.547097501	1.111423142	0.00023092	0.00132791
**PALD1**	5.947272617	-3.549826207	1.457259436	0.00163041	0.00754757
**WNT16**	177.5193915	-3.559805344	0.480743055	1.11E-14	2.62E-13
**AP005233.2**	20.90321828	-3.564641678	0.694595914	3.3E-08	3.84E-07
**TNFSF15**	5029.023949	-3.572005527	0.179638216	5.67E-89	3.48E-86
**SETP21**	3.470396427	-3.573732478	1.949454174	0.00364735	0.01515875
**CES1**	3.925566031	-3.576296441	1.829571462	0.00206638	0.00927488
**PLA2G7**	3.749765472	-3.579792575	2.12335028	0.00454950	0.01827907
**LINC01907**	19.41428194	-3.582384982	0.732802109	1.26E-07	1.34E-06
**OR10V2P**	5.353826114	-3.583765392	1.876407537	0.00380594	0.01569238
**PRR36**	45.86576847	-3.58650635	0.729675729	3.9E-08	4.49E-07
**AC008632.1**	54.58666356	-3.587350235	0.479410687	3.84E-15	9.49E-14
**ACY3**	14.62973909	-3.589926071	0.774846413	5.68E-07	5.49E-06
**TMEM51-AS1**	114.6912581	-3.593905807	0.422490758	2.76E-18	8.77E-17
**SULT1C2**	42.46254609	-3.593943224	0.47325465	2.07E-15	5.2E-14
**HPCAL1**	444.9333509	-3.59581348	0.223837831	3.07E-59	6.67E-57
**LAMP3**	352.7898099	-3.603173835	0.321899605	2.8E-30	1.86E-28
**KDELR3**	264.1027614	-3.604648872	0.242459918	7.51E-51	1.14E-48
**AL139161.1**	6.112170068	-3.605509675	1.887023852	0.00326284	0.01376036
**P3H3**	11.21973789	-3.611065139	0.917341128	8.92E-06	6.93E-05
**LINC01225**	8.977762412	-3.616802794	0.99566457	4.47E-05	0.00030214
**EHF**	1770.858633	-3.623348749	0.285276297	6.93E-38	6.33E-36
**AC105460.1**	1309.633445	-3.626826324	0.212020962	5.17E-67	1.67E-64
**MERTK**	89.50881312	-3.629376241	0.464159003	1.54E-16	4.23E-15
**NLRP5**	18.05598898	-3.639084555	0.795507458	8.71E-07	8.14E-06
**RHBDL2**	31.27988331	-3.640768042	0.701040905	1.35E-08	1.65E-07

**SAMD4A**	186.2862708	-3.646659056	0.329781368	6.72E-29	4.14E-27
**KIF21B**	14.79926875	-3.650157642	0.811364051	1.11E-06	1.02E-05
**TRPC4**	37.33616182	-3.650943628	0.548498113	7.2E-12	1.29E-10
**GAS6-AS1**	185.6545212	-3.65134287	0.375516148	4.88E-23	2.19E-21
**MYHAS**	17.04120146	-3.653847023	0.736162892	1.49E-07	1.58E-06
**AC011632.1**	96.72857175	-3.6549719	0.325557647	1.04E-30	7.12E-29
**FAM25A**	6.466671743	-3.656838912	1.34070233	0.00076392	0.0038796
**INHBA**	398.0886163	-3.657437573	0.801321192	3.18E-07	3.19E-06
**TRIB2**	292.246509	-3.658462118	0.356977903	5E-26	2.68E-24
**TESPA1**	3.64100187	-3.659085472	1.974905332	0.0033877	0.0142283
**HIP1**	61.81279566	-3.668962245	0.411683882	3.35E-20	1.24E-18
**AOX2P**	30.56032149	-3.673096003	0.611741062	1.42E-10	2.22E-09
**VIPR1**	19.1323097	-3.674473231	0.668275717	4.5E-09	5.83E-08
**C11orf91**	11.29979503	-3.676612511	0.966540592	1.06E-05	8.12E-05
**MAGEA1**	36.9171426	-3.677423726	0.499483579	1.59E-14	3.69E-13
**LINC01127**	305.3606042	-3.681528796	0.83434747	6.1E-07	5.86E-06
**CIB2**	5.093435245	-3.682325912	1.497006836	0.0013422	0.0063540
**LDLRAD3**	97.83656133	-3.683983635	0.415937	2.68E-19	9.19E-18
**NDRG4**	42.20475438	-3.684774237	0.822667783	9.27E-07	8.63E-06
**NBL1**	124.9913356	-3.691232369	0.336286166	1.59E-28	9.64E-27
**AL158839.1**	8.851601478	-3.697400983	1.123231177	0.0001649	0.0009796
**IQCF1**	2.267765373	-3.700696604	3.634565209	0.0082477	0.0301905
**LINC01029**	51.4609874	-3.70292449	0.492094162	9.1E-15	2.16E-13
**KLK8**	69.16554531	-3.703463364	0.429046426	2.76E-19	9.46E-18
**PADI2**	136.9041158	-3.706347803	0.413412069	1.24E-20	4.8E-19
**BEST3**	19.54707522	-3.707142903	0.696696224	1.35E-08	1.64E-07
**OXTR**	434.4902372	-3.714196139	0.218094798	1.07E-65	3.29E-63
**GRAMD1B**	528.7866721	-3.714352509	0.270300366	9.39E-44	1.09E-41
**ABCA3**	9.417157825	-3.715296311	0.979952185	2.54E-05	0.0001806
**CNTN5**	2.587818108	-3.720780327	3.954657894	0.0082728	0.0302545
**TRBV29OR9-2**	7.928207964	-3.722454823	1.490467559	0.0004657	0.0024923
**MARCO**	11.55357368	-3.724120453	0.871842066	2.67E-06	2.3E-05
**LINC01204**	10.8671912	-3.730993955	1.114850352	4.86E-05	0.0003262
**PLAUR**	1220.325444	-3.742137533	0.24844874	3.36E-52	5.42E-50
**MMP2**	3.690094133	-3.742448848	1.910169494	0.0027349	0.0118145

**ATP12A**	31.17785334	-3.742665468	0.66579293	1.5E-09	2.08E-08
**LINC01795**	13.04272508	-3.744537088	1.1110088	6.78E-05	0.0004428[illegible]
**KCNJ18**	5.012818419	-3.74630691	1.841321836	0.0011033[illegible]	0.0053576[illegible]
**ENSG00000288547**	2.727233686	-3.748554808	4.223189057	0.0084864[illegible]	0.0308752[illegible]
**COL13A1**	48.76989486	-3.759602707	0.586317015	7.86E-12	1.41E-10
**LINC01842**	5.608276189	-3.760247779	1.37929442	0.0005818[illegible]	0.0030497[illegible]
**PHLDA2**	82.88061648	-3.770803029	0.364040618	5.41E-26	2.88E-24
**SPRY4**	801.188503	-3.771451517	0.340441637	2.42E-29	1.54E-27
**AL355607.1**	13.2005025	-3.771838022	0.925010904	9.53E-06	7.37E-05
**RNU6-313P**	3.63015385	-3.772589975	1.904107998	0.0026647[illegible]	0.0115403[illegible]
**MYLK**	833.3088906	-3.777700546	0.355190806	1.17E-27	6.8E-26
**ELFN1**	3.60031818	-3.77871714	1.875201001	0.0024883[illegible]	0.0109085[illegible]
**FCMR**	39.06512765	-3.779197533	0.489229813	1.11E-15	2.84E-14
**TMEM270**	5.262955438	-3.78046463	1.470933527	0.0010322[illegible]	0.0050586[illegible]
**GREM2**	173.8615678	-3.780689449	0.33442366	4.38E-31	3.04E-29
**RAB29**	110.9481046	-3.781831363	0.392612578	1.46E-23	6.85E-22
**APOBEC3A**	70.42506563	-3.78436833	0.552498769	1.17E-12	2.3E-11
**AP000864.1**	10.87956663	-3.786595641	0.940829198	6.64E-06	5.31E-05
**THBD**	661.6323131	-3.789155171	0.257224006	6.23E-50	8.92E-48
**SRMS**	12.86073644	-3.795171688	1.010168661	9.55E-06	7.39E-05
**AC139712.3**	2.347778556	-3.79581029	3.606773358	0.007688	0.0285036[illegible]
**RF00019**	3.725895609	-3.800589444	4.909765528	0.0073851[illegible]	0.0275260[illegible]
**ANKRD20A8P**	91.16456667	-3.8057134	0.482706509	2.07E-16	5.64E-15
**CARD17**	91.60077109	-3.813171261	0.44672248	1.61E-18	5.21E-17
**AC055839.1**	9.392168227	-3.81607742	1.115017385	0.0001082[illegible]	0.0006726[illegible]
**AC090833.1**	25.28579837	-3.817583519	0.766761881	1.36E-07	1.44E-06
**SELENOP**	34.97138922	-3.818245052	0.751203278	6.78E-08	7.51E-07
**45361**	5.571891208	-3.819153667	1.632143267	0.0016516[illegible]	0.0076398[illegible]
**LY75**	6.122480199	-3.820246899	1.655170478	0.0015578[illegible]	0.0072524[illegible]
**AL031316.1**	9.901257221	-3.821960684	1.071685548	6.12E-05	0.0004034[illegible]
**THEMIS2**	44.86258701	-3.822297914	0.540199489	5.45E-14	1.2E-12
**AC027613.1**	3.880225115	-3.827481556	1.843677635	0.0020259[illegible]	0.0091155[illegible]
**GPSM3**	18.28737541	-3.828335081	0.744592832	2.96E-08	3.47E-07
**TMEM52**	4.97321998	-3.828404721	1.792286409	0.0008936[illegible]	0.0044512[illegible]
**SUN3**	75.06647906	-3.834798542	0.403102057	5.27E-23	2.36E-21

**TEX29**	21.67033618	-3.837718757	0.729321173	1.41E-08	1.72E-07
**ALCAM**	2924.817461	-3.838667086	0.166632009	3.33E-118	4.69E-115
**TRPM8**	5.402305263	-3.840431573	1.863957237	0.00096825	0.00478030
**GRHL3**	411.1273862	-3.844895785	0.335464386	4.06E-31	2.83E-29
**ADAM8**	1075.368265	-3.84979427	0.275791398	5.08E-45	6.13E-43
**ENSG00000287550**	2.297601042	-3.849802422	3.518001348	0.00736084	0.0274482
**LINC01186**	20.98216861	-3.852797189	0.818147158	2.02E-07	2.09E-06
**HVCN1**	5.668494889	-3.856699565	1.400327472	0.00054742	0.00288620
**CYP4F32P**	17.8426228	-3.86329415	0.982218715	5.75E-06	4.64E-05
**AC073464.2**	7.174319237	-3.863687162	1.413084918	0.00072243	0.00369324
**AC002351.1**	41.66969501	-3.865898534	0.480642282	1.45E-16	3.98E-15
**KCNH2**	4.823503743	-3.8730698	1.771487404	0.00082464	0.00414316
**LINC01338**	6.841881592	-3.877585748	1.431535394	0.00045142	0.00242549
**AC009081.1**	4.040798845	-3.878805863	1.995852707	0.00267989	0.01159962
**LINC00384**	2.556897188	-3.883548692	3.699245284	0.00734655	0.02742057
**AC126773.2**	2.357272381	-3.890698679	3.562349294	0.00729948	0.02729175
**AC093844.1**	65.47610381	-3.890746878	0.574473009	3.39E-12	6.34E-11
**AC026616.1**	4.802892954	-3.890903825	1.791133756	0.00085716	0.00428580
**FGFBP1**	585.3953966	-3.892786389	0.343697211	7.94E-31	5.44E-29
**MIR3681HG**	72.36790655	-3.896049835	0.443193689	2.65E-19	9.12E-18
**PHLDB1**	79.7381201	-3.906252606	0.368086972	1.49E-27	8.61E-26
**ETNK2**	43.25683769	-3.906685312	0.463824096	4.9E-18	1.52E-16
**45627**	160.9086447	-3.909003827	0.322029938	2.77E-35	2.3E-33
**LINC01897**	2.327436712	-3.909343945	3.499965844	0.00707233	0.02656291
**CNGA1**	2.357541326	-3.913949282	3.408125672	0.00659740	0.02499904
**CLIP2**	208.3231917	-3.918655282	0.317191202	2.13E-35	1.78E-33
**ESRRG**	57.92804465	-3.920604115	0.538149311	6.6E-14	1.44E-12
**FOSL1**	687.6302328	-3.921242112	0.252231716	7.21E-56	1.34E-53
**HCG22**	5.961646997	-3.922131115	1.591015808	0.00118764	0.00571041
**ENSG00000287771**	4.049745308	-3.922297247	1.859890356	0.00185285	0.00842879
**AC134978.1**	8.438523188	-3.926121533	1.124998568	9.39E-05	0.00059207
**AL354872.2**	22.90524578	-3.926206283	0.695943184	3.22E-09	4.25E-08
**ECM1**	1816.898564	-3.92655713	0.223163214	5.26E-70	1.82E-67
**AC021785.1**	13.11382021	-3.928059997	1.065779446	2.78E-05	0.00019625
**TMEM239**	4.120264667	-3.941680782	1.840058691	0.00167573	0.00774087

**GRK5**	246.7777832	-3.943605381	0.371021563	4.48E-27	2.55E-25
**ANK2**	43.78375393	-3.946137623	0.628715244	4.11E-11	6.79E-10
**ENSG00000286190**	17.34687246	-3.952336729	0.933028838	1.01E-06	9.33E-06
**EGR3**	4.485389374	-3.953015945	1.763282231	0.00098073	0.00483334
**OSBPL5**	53.69071184	-3.953559145	0.438096014	2.66E-20	9.94E-19
**PIK3CG**	40.94479573	-3.954101146	1.841156872	0.00100644	0.00494636
**AC078923.1**	65.39687719	-3.960054218	0.44486457	1.08E-19	3.86E-18
**LINC02450**	9.187382789	-3.960663449	1.148706539	9.43E-05	0.00059435
**AL691515.2**	2.976757591	-3.964211784	3.755589335	0.00641277	0.02441550
**AC026358.1**	14.73521695	-3.971444344	0.825851782	2.73E-07	2.77E-06
**AC010275.1**	12.23769548	-3.974123041	0.970864679	7.95E-06	6.24E-05
**FLG**	10.7932168	-3.976842718	1.041363862	1.15E-05	8.75E-05
**IGFBP6**	91.1365898	-3.979404712	0.364465927	3.82E-29	2.39E-27
**TSPAN5**	19.80203164	-3.979526179	0.783364875	1.01E-07	1.1E-06
**AC037198.1**	16.04339042	-3.980583562	1.170008144	6.7E-05	0.00043828
**PDE10A**	931.3689995	-3.984121515	0.166941798	8.84E-127	2.11E-123
**SCNN1B**	59.17128417	-3.984567331	0.581440948	8.11E-13	1.61E-11
**STK32B**	44.75223956	-3.984639643	0.541184478	5.26E-14	1.16E-12
**ENSG00000286123**	2.377614226	-3.99001222	3.445468611	0.00651493	0.02473359
**AC244502.1**	2.527330463	-3.990093324	3.407511032	0.00602691	0.02322002
**TNFSF18**	84.39155177	-3.995163795	0.484330102	6.1E-17	1.72E-15
**DPCR1**	17.07355453	-4.001909134	0.799104582	7.94E-08	8.72E-07
**TMEM229B**	56.41647938	-4.00258497	0.469013249	1.35E-18	4.39E-17
**RNU6-154P**	9.273147922	-4.012421346	1.068236341	2.99E-05	0.00020976
**OR10A3**	11.03506443	-4.012945968	1.063460751	2.63E-05	0.00018662
**POM121L2**	9.436916165	-4.014889466	1.149206822	8.13E-05	0.00052064
**LANCL3**	27.81141305	-4.016400849	0.751291893	3.87E-09	5.07E-08
**AP000941.1**	7.040534767	-4.017148345	2.054051496	0.00094428	0.00467548
**RGS4**	1201.198591	-4.018745319	0.786260112	1.98E-08	2.37E-07
**PPIAP78**	3.676534401	-4.029765458	4.449947608	0.00674901	0.02550482
**BVES**	12.13114125	-4.031670536	1.257110918	4.77E-05	0.00032068
**ANGPT2**	3.980042255	-4.042638723	1.794149811	0.00136355	0.00644575
**SALL4**	59.98561279	-4.043127346	0.430425368	1.7E-21	6.99E-20
**NLRP10**	284.7423369	-4.043340876	0.37320973	6.5E-28	3.83E-26
**LINC01122**	36.07433839	-4.058484553	0.584597494	7.84E-13	1.56E-11

**ELFN1-AS1**	4.269443015	-4.059219561	1.831868881	0.00140853	0.00662822
**AC103563.7**	7.744443239	-4.061060608	1.223853943	0.00014483	0.00087012
**IL32**	3249.759896	-4.061874892	0.285550148	5.68E-47	7.42E-45
**ZSCAN5B**	8.467151572	-4.079457758	1.386292276	0.00013562	0.00082073
**SLC43A3**	69.52309154	-4.082508194	0.404451747	1.65E-24	8.11E-23
**SLC22A1**	21.99354952	-4.089249587	0.722915496	2.52E-09	3.39E-08
**AL590666.2**	12.05927278	-4.099255949	0.951609619	2.62E-06	2.27E-05
**PAX9**	56.64201418	-4.111208091	0.512498766	4.1E-17	1.17E-15
**SDCBP2**	240.1920969	-4.112196039	0.314161234	1.02E-40	1.05E-38
**FRMPD4-AS1**	2.896753879	-4.113508747	3.878452814	0.00675723	0.02552782
**CPNE2**	110.0588484	-4.116710649	0.399339601	1.84E-26	1.02E-24
**TNNT1**	179.5719416	-4.120903694	0.305272692	1.02E-42	1.13E-40
**PRDM1**	564.0219803	-4.121879631	0.319379223	1.7E-39	1.67E-37
**LAMA1**	425.6071945	-4.124908382	0.331306237	2.12E-36	1.84E-34
**LINC02454**	2.437285565	-4.128077759	3.389776757	0.00582687	0.02256203
**CEMIP**	283.995507	-4.130706184	0.321408441	9.5E-39	9.05E-37
**ARHGEF10L**	169.0450112	-4.134677541	0.332460374	8.02E-36	6.82E-34
**SULT2B1**	19.93244567	-4.136876232	0.805624644	8.59E-08	9.38E-07
**ABI3BP**	31.14208099	-4.137302351	0.645940393	1.06E-11	1.88E-10
**ENSG00000288523**	2.527061518	-4.138794494	3.385775749	0.00562539	0.02188468
**AC112178.1**	23.489983	-4.138871908	0.692655605	2.79E-10	4.21E-09
**GDPD4**	56.57839839	-4.139335215	0.556855313	5.78E-15	1.4E-13
**TMEM108**	2.767370015	-4.151725982	3.462610971	0.00544199	0.02126113
**TRBC2**	321.2932845	-4.158392119	0.77163796	5.29E-09	6.76E-08
**NES**	35.9289653	-4.160960918	0.776329469	3.41E-09	4.48E-08
**KLHL5**	203.1872659	-4.164562677	0.328897573	4.53E-38	4.15E-36
**FRMPD4**	2.467390179	-4.165658632	3.281707295	0.00517203	0.02035611
**BID**	47.75081997	-4.167261587	0.525463911	1.24E-16	3.42E-15
**CASC9**	72.64779793	-4.171894614	0.402192126	2.36E-26	1.29E-24
**MAGEA10**	109.409366	-4.175625878	0.344385918	2.81E-35	2.34E-33
**OR10A6**	12.10162357	-4.177565965	1.008471729	3.33E-06	2.81E-05
**LIPE**	7.954656593	-4.180365414	1.259555085	0.00014866	0.00089064
**IGFBP1**	41.80648533	-4.180645403	0.555136821	7.87E-15	1.88E-13
**SPATA13**	28.3714981	-4.181260758	0.625827862	1.3E-12	2.55E-11
**ENSG00000286872**	6.223426546	-4.194026945	1.898857984	0.00080244	0.00404778

**PRR9**	3681.277053	-4.20130449	0.641753816	4.03E-12	7.47E-11
**ENSG00000286966**	431.6697936	-4.205018365	0.228855493	9.43E-77	3.89E-74
**AL121781.1**	7.545309513	-4.207483934	1.43327047	0.00021888	0.00126323
**CYP2S1**	26.79741245	-4.211374213	0.855031594	6.95E-08	7.69E-07
**NLRP1**	623.4093635	-4.215148767	0.222910636	2.9E-81	1.44E-78
**FHAD1**	284.4693252	-4.215243119	0.26125242	1.53E-58	3.21E-56
**DMTN**	17.62873557	-4.222339545	0.991874096	9.28E-07	8.64E-06
**AC078777.1**	2.487463079	-4.222940949	3.326248566	0.00520744	0.02046915
**PMEPA1**	49.93916192	-4.223940445	0.633852314	2.57E-12	4.86E-11
**ST8SIA6**	14.97427254	-4.227202433	1.119764544	7.38E-06	5.85E-05
**PALM2-AKAP2**	3813.209795	-4.236198613	0.26185209	7.39E-60	1.67E-57
**POF1B**	723.0011359	-4.23933028	0.278296855	2.05E-53	3.5E-51
**AC107959.3**	5.288994144	-4.239517393	1.936471031	0.00126028	0.00601107
**ZFHX4**	327.4241501	-4.247902157	0.283005361	1.72E-51	2.75E-49
**SHANK2**	6.341649489	-4.255627057	1.42037306	0.00032711	0.00181301
**ENSG00000287887**	8.903409946	-4.259509426	1.208636569	6.93E-05	0.00045100
**ALDH1A3**	12.57858234	-4.264308479	0.969299382	2.74E-06	2.35E-05
**AC068446.3**	11.12934228	-4.269168296	0.989465311	2.82E-06	2.42E-05
**LAMA3**	10053.74368	-4.270581978	0.341397416	4.91E-37	4.34E-35
**AC005186.1**	5.04439641	-4.27089749	1.675632156	0.00040741	0.00221341
**OR2I1P**	42.2098254	-4.27092502	0.697759659	8.54E-11	1.37E-09
**AL365181.3**	59.25018931	-4.271926415	0.45614319	9.06E-22	3.81E-20
**MYEOV**	463.8300928	-4.272407137	0.265369784	1.24E-58	2.62E-56
**PNMA8A**	10.4654327	-4.276461844	1.086710757	1.91E-05	0.00013877
**CLDN3**	6.532900206	-4.276692581	1.901581455	0.00070492	0.00361225
**AC090124.2**	20.15933743	-4.27776108	0.728062471	8.37E-10	1.19E-08
**LINC01152**	2.576970088	-4.283287892	3.373081987	0.00515226	0.02029701
**AC009478.1**	3.037245236	-4.284199306	3.563527151	0.00516588	0.02033860
**LINC00165**	23.64513663	-4.29128125	0.707223773	1.52E-10	2.35E-09
**CALB2**	634.1342138	-4.295246392	0.660962151	6.38E-12	1.15E-10
**SQOR**	226.0412887	-4.310166826	0.267145364	9.05E-60	1.98E-57
**ENSG00000285831**	9.297119081	-4.319400633	1.336729968	5.26E-05	0.00035052
**KLK6**	201.3343157	-4.320007765	0.348341337	7.12E-36	6.08E-34
**CD83**	120.0215689	-4.322662285	0.542287395	4.68E-17	1.33E-15
**KIRREL1**	183.3666109	-4.334388512	0.286596723	6E-52	9.63E-50

**CCDC9B**	582.416356	-4.343489001	0.194710344	1.26E-111	1.31E-108
**FAM201A**	20.51371827	-4.349849255	0.804070833	6.82E-09	8.6E-08
**ENSG00000286427**	2.867456099	-4.350678378	3.417583853	0.00472650	0.01887603
**AC087667.1**	3.026944577	-4.355214996	3.676533258	0.00550537	0.02147364
**AC105233.5**	37.8028906	-4.363727856	0.705776496	4.77E-11	7.81E-10
**AL139161.2**	2.627416547	-4.36642049	3.225588169	0.00417889	0.01699565
**LY6E**	1066.906042	-4.366482991	0.237743581	7.65E-76	3E-73
**PLPP2**	33.03503755	-4.367550019	0.597676113	1.23E-13	2.62E-12
**SYT6**	40.94214364	-4.377077318	0.586305516	2.46E-14	5.62E-13
**AC023310.4**	30.71252807	-4.384762069	0.661366102	2.91E-12	5.47E-11
**AC007741.1**	2.607074702	-4.388394155	3.220195249	0.00415386	0.01690554
**SPANXC**	5.832216324	-4.400207612	1.687656371	0.00024166	0.00138434
**AC096582.3**	15.29375291	-4.400767299	0.877205465	6.62E-08	7.36E-07
**HAS3**	719.4019897	-4.402073987	0.2712127	7.56E-60	1.69E-57
**SPANXB1**	32.74502592	-4.404237871	0.593607487	3.78E-14	8.47E-13
**POU2F2**	120.5174924	-4.405366324	0.343325117	2.44E-39	2.38E-37
**MTND2P32**	6.891431645	-4.40706008	1.324019204	0.00012094	0.00074150
**AC103718.1**	110.6943652	-4.415881412	0.419839332	6.22E-26	3.29E-24
**BCL2A1**	2.916817307	-4.417775751	3.286175782	0.00396061	0.01620992
**ESR1**	94.60215189	-4.421518289	0.404319149	1.75E-28	1.06E-26
**SCNN1A**	339.9715575	-4.423439526	0.263752307	4.49E-65	1.31E-62
**MN1**	2.727502631	-4.427312906	3.311687012	0.00431650	0.01747503
**SEC14L2**	40.61916961	-4.430838392	0.762849438	1.21E-10	1.9E-09
**AC073172.2**	2.916548362	-4.431795174	3.368420331	0.00432795	0.01751248
**SLC22A2**	2.776863839	-4.432239064	3.242912166	0.00393815	0.01614287
**KIRREL1-IT1**	5.008818264	-4.433353225	1.7064898	0.00047013	0.00251305
**LINC01844**	70.88537787	-4.435753073	0.589752674	7.11E-15	1.7E-13
**CD3E**	7.320785873	-4.439707644	1.326406027	0.00010698	0.00066581
**MACROD2**	248.2813962	-4.446336855	0.24011463	1.05E-77	4.49E-75
**AC087664.1**	3.665686381	-4.447034364	3.838457569	0.00503028	0.01988800
**ENSG00000285744**	107.481937	-4.450995706	0.389944653	2.99E-32	2.21E-30
**LINC01510**	14.85293151	-4.462410258	1.130923958	7.32E-06	5.81E-05
**HHLA2**	3.805639848	-4.474070828	3.86398778	0.00489383	0.01942423
**CRCT1**	652.749219	-4.479801057	0.320713497	8.29E-45	9.91E-43
**APOBEC3C**	132.2869037	-4.482441335	0.320588318	2.37E-45	2.91E-43

**AL117335.1**	2.826503464	-4.48451701	3.384405376	0.00442415	0.01786246
**MUC5B**	2.746759225	-4.484813969	3.25771152	0.00395198	0.01618568
**AC010327.3**	5.269199661	-4.496691562	1.720929093	0.00043337	0.00233958
**TRIM15**	16.08915905	-4.498539052	1.051235835	2.75E-06	2.36E-05
**RAP1GAP2**	595.2723632	-4.49864839	0.233156317	2.35E-83	1.2E-80
**CDHR3**	17.52891356	-4.498894269	0.805242407	5.62E-09	7.14E-08
**AC068299.1**	6.66108911	-4.502831413	1.684972696	0.00017510	0.00103428
**RPL5P13**	18.22551863	-4.503711002	0.90409108	4.33E-08	4.94E-07
**RAB3IL1**	38.04143058	-4.509095388	0.656853268	1.26E-13	2.68E-12
**AC027338.2**	12.79833347	-4.511085891	0.995190627	1.27E-06	1.15E-05
**ABCA13**	5.028891164	-4.511305848	1.666857748	0.00034588	0.00190993
**FER1L6**	12.43320877	-4.513403343	1.084602284	7.4E-06	5.86E-05
**SLC16A7**	366.7513424	-4.51519174	0.269155341	1.47E-63	3.96E-61
**AC025252.2**	4.998517605	-4.522688733	1.740787185	0.00049292	0.00262431
**CDH3**	1079.165795	-4.530199628	0.621074425	2.12E-14	4.86E-13
**ABR**	186.2882905	-4.540268335	0.280757055	8.53E-60	1.89E-57
**NRG1**	3.285971778	-4.542424127	3.682446779	0.00489712	0.01942763
**ACVR1C**	7.909628578	-4.546270874	1.379564843	0.00011733	0.00072112
**AC016723.1**	18.97150406	-4.549275587	0.894956712	1.23E-07	1.32E-06
**AP000997.2**	60.71231766	-4.549294604	0.462282959	8.42E-24	3.98E-22
**AP003097.1**	2.716923556	-4.553622541	3.168342359	0.00342143	0.01433693
**GNGT2**	7.64138597	-4.556062222	1.345724898	9.38E-05	0.00059162
**CARD10**	362.7033452	-4.558091928	0.401439069	3.12E-30	2.07E-28
**MYOM3**	6.802506482	-4.558414108	1.749459746	0.00026813	0.00151599
**AGPAT4**	837.3246466	-4.563424798	0.196513253	3.55E-119	5.65E-116
**CATSPER1**	10.07134206	-4.57590964	1.170177224	1.98E-05	0.00014314
**AC008691.1**	15.00944537	-4.580479771	1.140528701	2.03E-06	1.79E-05
**TMEM171**	7.890371985	-4.592845221	1.287993707	5.2E-05	0.00034729
**KLK7**	471.9009683	-4.596875954	0.38890159	9.14E-33	6.93E-31
**OR7E122P**	15.64791822	-4.597277105	1.159377188	2.32E-06	2.02E-05
**LINC01828**	11.0447253	-4.59774692	1.075069053	3.27E-06	2.77E-05
**AC131254.1**	60.98735814	-4.606336515	0.513858062	1.36E-19	4.8E-18
**AC021351.1**	7.881156577	-4.626262481	1.351401791	8.28E-05	0.00052920
**ME3**	70.87482422	-4.632884384	2.358226695	0.00080346	0.00405121
**NRIP3**	97.73643382	-4.636103112	0.541033274	7.18E-20	2.58E-18

**LINC01162**	56.49455858	-4.636144712	0.487867825	1.43E-21	5.91E-20
**AC129502.1**	7.940280554	-4.63665916	1.274273018	4.15E-05	0.00028374
**FCGRT**	12.14482751	-4.637493335	1.102465799	3.63E-06	3.04E-05
**PKNOX2**	3.166350683	-4.647164097	3.290828227	0.0033168	0.0139572
**LINC00508**	46.30022224	-4.654226535	0.517037928	1.25E-19	4.42E-18
**AC073648.6**	10.482256	-4.654757275	1.097971449	5.97E-06	4.81E-05
**HAPLN3**	70.08090023	-4.659062044	0.520446935	2.19E-19	7.63E-18
**AC110995.1**	10.67884719	-4.662663026	1.526262252	0.0001174	0.0007218
**PLCB4**	27.15258516	-4.664810279	0.708389133	5.46E-12	9.95E-11
**DMBX1**	2.907054538	-4.664984149	3.11949574	0.0027699	0.0119397
**HSH2D**	25.58828219	-4.668411088	0.746408412	2.94E-11	4.95E-10
**TLR10**	6.143422834	-4.670338318	1.595637898	0.0001126	0.00069588
**CTSC**	706.971117	-4.675816957	0.21421214	3.82E-106	3.51E-103
**STEAP1**	46.21962212	-4.680336094	0.57965228	1.22E-16	3.38E-15
**AC005537.1**	18.28065192	-4.681195959	0.880199306	1.54E-08	1.87E-07
**AC091804.1**	6.611458957	-4.688296632	1.599626622	8.37E-05	0.00053394
**SFTA1P**	8.010262024	-4.692048117	1.28267906	3.95E-05	0.00027125
**CASC15**	390.4995859	-4.695225232	0.278252857	2.95E-65	8.82E-63
**TEX15**	280.6721214	-4.697117011	0.293496501	6.55E-59	1.41E-56
**AL121904.1**	17.28453915	-4.701032733	0.835698774	4.55E-09	5.88E-08
**GNG11**	11.67598455	-4.720676371	1.03759151	1.26E-06	1.15E-05
**BMP2**	31.26231992	-4.730596764	0.764683528	3.13E-10	4.71E-09
**AC025884.1**	45.16466249	-4.738828544	0.667976554	1.99E-14	4.58E-13
**AC110056.1**	13.88389921	-4.74089246	1.105965587	1.6E-06	1.43E-05
**SPRR2E**	24.74163461	-4.745068445	0.814668186	9.56E-10	1.35E-08
**PPIAP26**	15.53304972	-4.753007536	1.025415518	5.75E-07	5.55E-06
**VWA2**	6.810545874	-4.754847027	1.619358747	8.02E-05	0.00051429
**GPAT3**	668.2301924	-4.755189565	0.220990098	4.54E-103	3.75E-100
**KIF13B**	236.5313962	-4.759895809	0.287995189	2.6E-62	6.54E-60
**CASR**	11.20970745	-4.759921554	1.223109987	2.02E-05	0.00014597
**ENSG00000285914**	3.805908793	-4.762942912	3.471281886	0.0032291	0.0136363
**MAGEA9B**	11.59022889	-4.763934928	1.105594027	4.17E-06	3.45E-05
**SDR16C5**	78.26777725	-4.767504513	0.519561925	2.7E-21	1.09E-19
**SSX6**	42.5869447	-4.776632586	0.71809661	1.42E-12	2.77E-11
**SERPINB2**	5311.031557	-4.777946361	0.328213583	4.49E-49	6.39E-47

**LINC02267**	9.134556463	-4.783617104	1.21134483	9.94E-06	7.66E-05
**CASP1**	1008.866469	-4.785520093	0.212606661	2.83E-112	3.23E-109
**CCDC88A**	93.50494809	-4.788566495	0.466171678	1.24E-25	6.45E-24
**LINC02474**	98.65768587	-4.791131199	0.655412147	4.47E-15	1.09E-13
**DLGAP1-AS5**	3.416153004	-4.7960984	3.272307428	0.00277344	0.01195262
**NBPF13P**	3.606283986	-4.802199629	3.3286008	0.00280898	0.01209488
**TRIM17**	24.99106052	-4.803790787	0.710590222	3.42E-12	6.38E-11
**PRR5L**	38.64606263	-4.806578752	0.79044604	1.58E-11	2.76E-10
**PLEKHG5**	375.4317364	-4.809930428	0.319449412	2.96E-51	4.59E-49
**MYRF**	17.25355248	-4.809944638	0.91172958	1.39E-08	1.69E-07
**ENSG00000286662**	30.53458706	-4.810176642	0.914078159	2.55E-09	3.43E-08
**PTPRH**	168.0545629	-4.8116156	0.380537666	4.21E-38	3.87E-36
**ADARB2-AS1**	3.356491138	-4.823021411	3.42751576	0.00343820	0.01439963
**TMPRSS3**	156.8818258	-4.826971202	0.447145101	2.68E-28	1.6E-26
**TNC**	2287.023035	-4.838953245	1.074127684	3.19E-07	3.2E-06
**OPRM1**	3.246094922	-4.843953881	3.238076834	0.00272487	0.01177298
**ENTPD3**	211.173503	-4.846124646	0.410628924	5.88E-33	4.5E-31
**SPRR3**	2008.826029	-4.846279255	0.305241652	1.7E-57	3.25E-55
**IL3RA**	9.358543407	-4.855091372	1.276521436	2.09E-05	0.00015084
**RPS26P30**	9.844364988	-4.855388216	1.222624062	8.42E-06	6.57E-05
**LINC02323**	3.186692528	-4.862385085	3.092672018	0.00211049	0.00944272
**RN7SL815P**	12.31034754	-4.865467854	1.070407589	1.61E-06	1.44E-05
**ADGRF1**	1930.753026	-4.867891381	0.169982247	1.4E-182	1.12E-178
**PNMA8B**	2.926858493	-4.874472481	3.104657076	0.00234277	0.01036179
**CDH13**	63.02810116	-4.875111661	0.509148578	1.81E-23	8.34E-22
**LINC00454**	18.57583424	-4.879078406	0.899535089	1.83E-08	2.19E-07
**THY1**	19.47376248	-4.896220746	0.858653116	3.23E-09	4.26E-08
**BRSK1**	6.097812974	-4.896279208	1.590656072	9.35E-05	0.00059011
**KCNQ2**	7.43084739	-4.900912752	1.583200039	4.4E-05	0.00029811
**NOS3**	42.127565	-4.907542952	0.607254019	7.07E-17	1.98E-15
**IGHV1OR15-6**	2.956963107	-4.908204284	3.032397685	0.00195344	0.00883259
**MCF2L**	84.04932397	-4.915997491	0.426315529	3.38E-31	2.37E-29
**LINC00917**	3.216528198	-4.921527088	3.073359453	0.00193321	0.00875107
**AC109439.2**	15.44252936	-4.922482003	1.008755834	3.92E-07	3.87E-06
**TSPEAR-AS2**	9.197988622	-4.923389878	1.32987474	3.16E-05	0.00022106

**VNN 1.00**	180.9225967	-4.936322727	0.482807525	8.93E-26	4.7E-24
**KISS1**	27.67312651	-4.937297138	0.815802786	7.04E-10	1.02E-08
**UBD**	153.1530313	-4.948679439	0.514574384	9.1E-24	4.29E-22
**SHANK3**	62.46920962	-4.965759884	0.47856924	1.23E-25	6.42E-24
**AL160408.4**	6.94622077	-4.971124941	1.745535735	0.00018802	0.00110320
**IL24**	963.6491666	-4.971993802	0.816585293	7.13E-11	1.15E-09
**CASC8**	7.056607513	-4.972436216	1.704632585	0.00014349	0.00086337
**DBNDD1**	32.60064351	-4.972735023	0.649404809	3.28E-15	8.11E-14
**GRIK2**	26.52653275	-4.976099386	0.864368693	2.06E-10	3.16E-09
**YWHAEP7**	3.016634446	-4.976818103	3.033256721	0.00182312	0.00831274
**LINC01049**	29.2186545	-4.977871812	0.695991052	5.76E-13	1.16E-11
**FSD1**	16.51062539	-4.978663559	0.960635389	8.99E-08	9.79E-07
**BTC**	44.11687787	-4.991865049	0.59631981	8.43E-18	2.56E-16
**CD5L**	55.18741356	-4.994392543	0.536414043	1.72E-21	7.04E-20
**RPL7P30**	12.78552429	-4.99565117	1.261890192	4E-06	3.32E-05
**GBP7**	19.1671414	-4.998589818	0.992338673	6.72E-08	7.46E-07
**PPP2R2C**	80.03092235	-4.999845224	0.601528619	1.74E-17	5.17E-16
**AREG**	4579.256207	-5.006109281	0.247498377	1.01E-91	6.51E-89
**LDOC1**	71.27465705	-5.007943851	0.576914931	5.55E-20	2.02E-18
**AL157770.1**	3.71613284	-5.02343288	3.200537866	0.00197243	0.00890666
**SLC10A2**	3.726721388	-5.023923769	3.423853555	0.00284289	0.01221886
**LINC01967**	7.017296963	-5.031208704	1.837046572	0.00028187	0.00158652
**BARX2**	101.1611716	-5.032827674	0.465375362	2.71E-29	1.71E-27
**ELMO1**	34.25139605	-5.033941337	0.694028786	1.98E-13	4.14E-12
**SLIT3**	9.887993191	-5.033983217	1.215720513	6.31E-06	5.07E-05
**ENSG00000287175**	3.775804179	-5.041323084	3.237937938	0.00206464	0.00926881
**MIR135B**	3.066811961	-5.044550419	2.999090415	0.00156004	0.00725975
**LINC02261**	3.066811961	-5.044550419	2.999090415	0.00156004	0.00725975
**CCDC78**	3.806734572	-5.04458865	3.442767633	0.00283088	0.01217601
**RGS19**	32.30916086	-5.045121764	0.67647349	2.7E-14	6.14E-13
**TGFBR2**	38.93320791	-5.055933945	0.847598378	3.2E-10	4.81E-09
**COL8A1**	286.0826704	-5.078000676	1.013968348	3.22E-08	3.76E-07
**ATP8B2**	22.19257812	-5.086651227	0.810392368	1.22E-10	1.91E-09
**SVEP1**	919.0175086	-5.087472776	0.181458175	1.94E-173	9.28E-170
**MIR99AHG**	27.63859393	-5.087529764	0.744536511	1.2E-12	2.35E-11

**OR7E99P**	27.31945062	-5.090055123	0.802414542	1.41E-10	2.2E-09
**TNFRSF25**	149.4070166	-5.094022108	0.351948016	1.75E-47	2.37E-45
**TLR6**	128.3284271	-5.097913441	0.378897448	5.24E-42	5.59E-40
**PPP1R3G**	9.649611264	-5.100002872	1.801074962	0.0001092	0.0006782
**AL356234.1**	72.39405487	-5.114801302	0.67405658	1.08E-15	2.77E-14
**BVES-AS1**	3.176391869	-5.117486696	3.070569826	0.0016865	0.0077804
**AL691420.1**	66.46627161	-5.122567558	0.517813087	2.18E-24	1.07E-22
**TRPV3**	43.59095181	-5.12614612	0.601413217	1.4E-17	4.17E-16
**CRISPLD1**	3.456567749	-5.126249153	3.044831665	0.0014311	0.0067279
**KLK10**	145.7267382	-5.126573173	0.425409813	2.15E-33	1.68E-31
**SLC7A11-AS1**	20.65166055	-5.130385989	0.934551566	4.64E-09	5.99E-08
**NTSR1**	481.3190727	-5.135793638	0.911281996	1.1E-09	1.56E-08
**PRICKLE1**	68.80902733	-5.147631286	0.576800138	3.9E-19	1.32E-17
**LVRN**	322.8593156	-5.155949179	0.342409666	8.83E-51	1.32E-48
**ICA1**	146.6333408	-5.167974321	0.367915279	1.32E-46	1.7E-44
**CARD11**	52.03246962	-5.171181847	0.553624994	1.41E-20	5.42E-19
**ITPRIPL1**	11.5769381	-5.175079894	1.28556436	8.11E-06	6.35E-05
**GAS7**	101.3690435	-5.177865761	0.518060812	2.09E-23	9.61E-22
**LINC00431**	159.1040642	-5.181627137	0.367955879	7.29E-45	8.76E-43
**ZP4**	125.8565447	-5.18583837	0.924081278	8.09E-10	1.16E-08
**IFNG-AS1**	3.316614282	-5.197547075	2.967357343	0.0011484	0.0055484
**CPNE5**	7.616430855	-5.20344385	1.590115703	4E-05	0.0002737
**LMO2**	46.78520143	-5.214878028	0.63325532	2.94E-17	8.53E-16
**AC005392.2**	13.42510135	-5.216444348	1.212656548	1.09E-06	1E-05
**FNDC11**	13.88174209	-5.219216979	1.197072304	6.85E-07	6.53E-06
**PRSS23**	187.4815997	-5.225933424	0.325464176	7.97E-58	1.57E-55
**SPRR2D**	864.9958673	-5.240933125	0.685094593	1.29E-15	3.3E-14
**LINC01522**	11.44567162	-5.241966701	1.236592521	3.9E-06	3.25E-05
**SULT1C3**	3.905994877	-5.243377814	3.12323187	0.0013729	0.0064851
**LINC01640**	3.905994877	-5.243377814	3.12323187	0.0013729	0.0064851
**C1orf167**	3.396627466	-5.244318978	2.972643278	0.0010857	0.0052842
**AC109445.1**	3.346449951	-5.254453144	2.949745857	0.0010227	0.0050182
**AC099506.1**	3.306582568	-5.260821915	3.018144997	0.0012550	0.0059906
**CHST4**	3.575910427	-5.262793071	3.034963102	0.0012088	0.0057971
**ROBO3**	66.94463244	-5.263896737	0.508915689	3.9E-26	2.11E-24

**ARAP3**	66.40297545	-5.267740389	0.495888106	2.78E-27	1.59E-25
**SCNN1G**	60.27169254	-5.269325399	0.732324297	9.44E-14	2.03E-12
**FBXL7**	87.2192696	-5.271877774	0.447456995	1.28E-32	9.59E-31
**PRKG1-AS1**	12.40046481	-5.28245919	1.274473845	3.85E-06	3.21E-05
**HSPE1P5**	8.096509959	-5.283979268	1.640923563	4.75E-05	0.00031973
**GOLGA7B**	27.75788152	-5.288047411	0.859682286	1.02E-10	1.61E-09
**SIRPB3P**	45.99331442	-5.294339726	0.602437772	1.37E-19	4.84E-18
**TLR1**	35.74698539	-5.297538355	0.760081987	6.25E-13	1.25E-11
**AC009090.5**	3.316345337	-5.301138031	2.959736126	0.00101783	0.00499617
**AC090124.3**	19.50747483	-5.306062673	0.923062287	5.8E-09	7.36E-08
**CLCN3P1**	101.5801625	-5.309159259	0.42098165	1.2E-37	1.08E-35
**GEM**	26.61580162	-5.312486957	0.913052248	1.25E-09	1.75E-08
**PRUNE2**	2184.847563	-5.314938021	0.88920271	1.23E-10	1.94E-09
**UNC5A**	240.2540227	-5.343922695	0.368495711	1.12E-46	1.46E-44
**LINC00603**	8.25547002	-5.349520669	1.787019394	0.00011735	0.00072112
**PIGR**	92.53962883	-5.350782001	0.565621284	1.52E-23	7.05E-22
**ACP7**	36.81497631	-5.351346729	0.689179452	1.12E-14	2.64E-13
**GLRB**	27.07034257	-5.354469479	0.782481138	1.81E-12	3.49E-11
**TMPRSS6**	3.596252272	-5.356190832	2.952820972	0.00085787	0.00428849
**OLFML3**	3852.531984	-5.36176321	0.347951957	1.07E-54	1.91E-52
**CDC42EP5**	13.40996528	-5.363260409	1.172151892	6.27E-07	6E-06
**ACTBP7**	9.298556315	-5.368224365	1.583175411	1.28E-05	9.67E-05
**TMEM92**	10.77620262	-5.368964927	1.66155649	1.91E-05	0.00013870
**AC010638.1**	12.62606543	-5.374028802	1.21022913	1.62E-06	1.45E-05
**RNU6-1039P**	3.965397271	-5.375767601	3.113610823	0.00120749	0.00579279
**F2R**	3.50593843	-5.397978215	3.029837946	0.00108049	0.00526162
**THBS1**	2546.106057	-5.400317175	0.348907101	8.16E-55	1.47E-52
**LAT2**	109.7626692	-5.407637184	0.409838102	2.21E-41	2.31E-39
**LPCAT2**	93.9991899	-5.410830312	0.461252567	1.75E-31	1.25E-29
**AC090409.1**	56.05216696	-5.413377283	0.649056389	5.45E-18	1.68E-16
**MAL**	833.0621498	-5.415484756	0.296435323	3.69E-75	1.42E-72
**MRPL35P1**	4.175870098	-5.418570801	3.120569739	0.00110952	0.00537998
**CARD16**	228.2698959	-5.430429223	0.32057837	4.24E-66	1.34E-63
**SULT1A1**	18.43017893	-5.432386905	0.997226444	1.67E-08	2.02E-07
**ENSG00000285755**	3.746515871	-5.434764328	3.001945348	0.00088559	0.00441502

**EPB41L2**	2994.883623	-5.444487372	0.254654304	4.09E-102	3.15E-99
**AC090791.1**	24.79622385	-5.451778033	0.844305283	1.77E-11	3.06E-10
**SPRR1A**	179.0120408	-5.460036627	0.359007557	6.52E-53	1.09E-50
**C1QTNF2**	8.414939554	-5.461032432	1.537022216	1.3E-05	9.78E-05
**MEG8**	9.404107652	-5.462212735	1.701836121	4.54E-05	0.00030659
**NAALADL2-AS2**	4.545284042	-5.465200055	3.210704074	0.00121293	0.00581535
**CCNB3**	30.31641132	-5.472566315	0.79254855	9.94E-13	1.97E-11
**PDE2A**	13.1961989	-5.473986369	1.225997899	1.46E-06	1.32E-05
**PRSS51**	9.527738374	-5.474155753	1.501563708	4.74E-06	3.88E-05
**AL358176.2**	8.15619077	-5.475487057	1.526925146	1.18E-05	8.97E-05
**ZFP41**	8.38483494	-5.478404227	1.537268171	1.26E-05	9.53E-05
**DYRK3**	122.0656525	-5.485492652	0.459589348	9.36E-33	7.06E-31
**TRIM58**	437.1477057	-5.491818797	0.334918242	6.21E-60	1.42E-57
**LINC00589**	134.05453	-5.495315703	0.458259492	1.31E-34	1.06E-32
**CD19**	3.556384889	-5.500860815	2.921411105	0.00066539	0.00343247
**TGFA**	2515.073914	-5.505829572	0.308662358	1.09E-71	4.03E-69
**LINC00880**	3.656470973	-5.506649654	2.994844652	0.00083017	0.00416657
**ENSG00000285673**	3.656470973	-5.506649654	2.994844652	0.00083017	0.00416657
**AC068875.1**	34.42009496	-5.509702797	0.718320188	8.33E-15	1.99E-13
**AL355607.2**	36.57873206	-5.521905975	0.799435568	4.06E-12	7.52E-11
**SEMA6B**	49.51535285	-5.540907749	0.654702282	2.03E-18	6.52E-17
**ENSG00000287897**	56.82337028	-5.543354272	0.578698116	2.66E-22	1.15E-20
**ELFN2**	23.18102242	-5.545288822	0.920731363	1.23E-09	1.71E-08
**SNX18P14**	8.74421717	-5.548336027	1.550811245	1.18E-05	8.96E-05
**CD6**	4.185094978	-5.55120648	3.083477794	0.00090522	0.00450163
**ENSG00000286281**	4.185363923	-5.559680358	3.026219174	0.00074561	0.00379471
**AC034154.1**	4.406156353	-5.566289769	3.265893234	0.00136139	0.00643682
**SYT14**	856.93704	-5.575840718	0.238916359	2.16E-119	3.69E-116
**SOX9**	878.373792	-5.576753836	0.243001535	2.2E-118	3.28E-115
**FBXL2**	53.28464553	-5.587148861	0.717419981	2.61E-16	7.02E-15
**MUC4**	269.3198105	-5.591521781	0.395486535	3.66E-45	4.45E-43
**FAM187B2P**	4.015574786	-5.592416269	2.953943534	0.00058889	0.00308510
**AL592463.1**	4.106167045	-5.592697699	3.014090977	0.00070876	0.00362959
**CASP1P2**	55.76824943	-5.599132929	0.5955475	7.42E-21	2.92E-19
**MUSK**	264.3990819	-5.600943277	0.905981077	1.79E-11	3.1E-10

**AC087392.1**	25.07176264	-5.626510575	0.895539463	2.59E-10	3.93E-09
**RN7SL591P**	9.65418839	-5.62651167	1.545799467	8.16E-06	6.38E-05
**LGALS1**	299.2651404	-5.630541024	0.300137704	2.6E-80	1.22E-77
**AL138789.1**	486.510761	-5.638662798	0.283642058	7.55E-87	4.3E-84
**LINC02511**	3.815681035	-5.646356337	2.912348606	0.0004996	0.0026564
**OR2W3**	26.65873766	-5.652628071	0.844960534	6.89E-12	1.24E-10
**KIAA1549L**	467.0211287	-5.664860716	0.240938242	1.32E-122	2.63E-119
**AC005323.1**	9.844050428	-5.666524778	1.536818666	6.6E-06	5.28E-05
**ANXA6**	186.2862763	-5.675425675	0.434001812	1.42E-41	1.5E-39
**AL365184.1**	50.15386715	-5.678445567	0.661798385	2.3E-17	6.75E-16
**LINC01792**	175.0663223	-5.70232402	0.867678296	1.41E-12	2.75E-11
**CACNA1I**	3.855826835	-5.704025938	2.955885982	0.0005484	0.0028905
**TMPRSS11E**	193.9613767	-5.734200434	0.908441516	2.59E-11	4.4E-10
**AC024597.1**	36.55414765	-5.753101528	0.763917615	6.21E-15	1.5E-13
**HCG21**	4.37522596	-5.755777708	2.956556565	0.0004428	0.0023842
**SPRR1B**	1111.818441	-5.770019328	0.363991227	5.69E-57	1.08E-54
**AC010343.3**	152.3951345	-5.770398717	0.436349573	3.78E-42	4.05E-40
**ROBO4**	54.78837978	-5.792859806	0.653550282	2.11E-18	6.74E-17
**PACSIN1**	9.774356847	-5.802130123	1.478058225	2.4E-06	2.08E-05
**PSG10P**	4.236088799	-5.810480423	2.941741106	0.0004051	0.0022018
**CCDC169**	4.11566087	-5.811780602	2.869004345	0.0002988	0.0016704
**AC025419.1**	4.205167878	-5.821471231	2.918681006	0.0003658	0.0020073
**MAP2**	556.1471947	-5.835093307	0.23974764	2E-129	5.98E-126
**AL606537.1**	4.345121346	-5.842933217	2.919939052	0.0003427	0.0018936
**SLC12A7**	1695.59227	-5.851471476	0.298090223	8.32E-86	4.62E-83
**BTF3P7**	4.495653889	-5.855607147	2.950980169	0.0003696	0.0020248
**AL356234.3**	51.26178035	-5.859873882	0.803662986	6.57E-14	1.43E-12
**MSLN**	33.41366921	-5.88574379	0.844259914	3.78E-13	7.75E-12
**OR2T8**	16.4208762	-5.89448807	1.134848317	5.42E-08	6.11E-07
**SH3GL3**	46.08886815	-5.895938027	0.772047619	5.77E-15	1.4E-13
**ENSG00000287218**	4.13573377	-5.896331569	2.855249328	0.0002435	0.0013936
**LCE3D**	4.255345393	-5.911718228	2.863761124	0.0002410	0.0013812
**Z99916.1**	4.285181062	-5.912069342	2.881764313	0.0002628	0.0014902
**KLK5**	1133.058161	-5.930462153	0.609220655	2.17E-23	9.96E-22
**TRIM40**	17.54837462	-5.939996366	1.186750039	1.2E-07	1.29E-06

**OLFM4**	162.041192	-5.940357323	0.93415635	2.02E-11	3.48E-10
**FXYD7**	4.525489559	-5.943833447	2.897792519	0.00025286	0.00143950
**SUSD4**	10.8435476	-5.950700826	1.463189289	1.14E-06	1.05E-05
**FAM131C**	4.844725988	-5.978882231	2.952112663	0.00028865	0.00161935
**AL137161.1**	4.564819053	-5.985849461	2.907022286	0.00024851	0.00141794
**RAB3B**	957.3218711	-5.996705544	0.240882972	1.14E-135	3.91E-132
**AFAP1-AS1**	181.1403829	-6.002760958	0.973242258	6.05E-11	9.82E-10
**SFTA2**	19.2969814	-6.005152746	1.29941695	5.73E-07	5.53E-06
**SPRR2A**	605.8363407	-6.00912369	0.657730776	7.55E-21	2.96E-19
**BNIP3P4**	4.894903503	-6.009196327	2.940175831	0.00025744	0.00146351
**AC079921.1**	4.725114366	-6.011088611	2.898380312	0.00021738	0.00125668
**FLI1**	19.15866054	-6.030872852	1.157682316	4.19E-08	4.8E-07
**LINC01910**	12.14713447	-6.044041495	1.453669525	4.96E-07	4.83E-06
**ACTBP1**	4.355431477	-6.045674487	2.840081903	0.00016406	0.00097439
**CADM1**	1713.037957	-6.046780328	0.254186746	3.18E-124	6.92E-121
**NGEF**	19.21752505	-6.067717089	1.134053102	2.18E-08	2.6E-07
**ENSG00000287544**	20.24254971	-6.067729895	1.106404634	7.17E-09	9E-08
**ANO4**	11.67134461	-6.074251424	1.45202119	6.2E-07	5.94E-06
**AC064875.1**	25.91450033	-6.0816257	0.939078851	9.65E-11	1.53E-09
**AC024681.1**	12.0217709	-6.089263316	1.441375614	4.83E-07	4.72E-06
**AC034223.2**	63.9318603	-6.089803864	0.699371706	1.67E-19	5.84E-18
**C8orf34**	4.834963219	-6.092046705	2.881130697	0.00016939	0.00100323
**AC018554.1**	4.465011386	-6.122800438	2.869835511	0.00016776	0.00099482
**TGFA-IT1**	21.51711722	-6.135202481	1.086910395	2.08E-09	2.83E-08
**MIR137HG**	336.1206941	-6.135335614	0.877344674	2.98E-13	6.13E-12
**PRSS3**	53.63032001	-6.138493339	0.677858523	3.21E-20	1.19E-18
**CDH15**	4.82520045	-6.139792807	2.869099994	0.00014609	0.00087615
**IGFBP5**	66.44380648	-6.141432667	0.728156972	2E-17	5.9E-16
**AL513304.1**	5.454179484	-6.143645866	3.007521466	0.00025912	0.00147198
**OR2AJ1**	5.484015153	-6.146376478	3.021723057	0.00027464	0.00154950
**OR7E163P**	12.29219349	-6.158450462	1.447686217	4.2E-07	4.13E-06
**XACT**	4196.221854	-6.164140654	0.696328824	4.51E-20	1.65E-18
**LINC01793**	4.745465683	-6.177545755	2.929108944	0.00020061	0.00116848
**AC078788.1**	4.575129184	-6.179297549	2.831889642	0.00011476	0.00070792
**BTNL9**	5.864008173	-6.195955122	3.094937713	0.00032770	0.00181586

**AL136982.5**	31.17733374	-6.197253659	0.916513885	4.09E-12	7.57E-11
**AC003080.1**	28.11310054	-6.204585146	0.916429711	1.63E-11	2.84E-10
**AC105245.1**	4.935049303	-6.21707355	2.852666034	0.0001119	0.0006925
**HNF4G**	32.25628003	-6.229291285	0.921454857	3.5E-12	6.53E-11
**EFNA5**	54.67116861	-6.259783672	0.672518886	2.05E-20	7.73E-19
**ENSG00000287976**	4.964884973	-6.259798887	2.835271849	9.12E-05	0.0005767
**AC090204.1**	86.74369114	-6.26287769	0.549332321	1.77E-29	1.13E-27
**NBEAP1**	196.9749058	-6.279773078	0.381563708	4.88E-61	1.15E-58
**AC097451.1**	5.304463247	-6.280485064	2.883803	0.0001112	0.0006894
**RAC2**	697.6943861	-6.281080874	0.277153477	6.51E-112	7.07E-109
**PTPRR**	432.3139411	-6.281868991	0.367757066	7.3E-69	2.43E-66
**AC013565.1**	4.845004405	-6.313915771	2.823130566	7.77E-05	0.0005001
**DOCK4**	1253.252261	-6.315512544	0.211597677	4.82E-195	5.76E-191
**AC005291.2**	80.10273617	-6.320402547	0.606029805	7.3E-25	3.65E-23
**AC234064.1**	4.975195104	-6.325489249	2.846391049	8.72E-05	0.0005542
**PTK6**	158.0141401	-6.326270175	0.416096612	2.34E-52	3.81E-50
**AC025627.1**	4.984957873	-6.328027949	2.823765941	7.3E-05	0.0004725
**ADD2**	5.533654778	-6.338304995	2.94233357	0.0001385	0.0008366
**AC108174.1**	22.45251248	-6.349177829	1.148932988	8.46E-09	1.05E-07
**CEACAM1**	3139.541485	-6.358395448	0.335373298	2.27E-80	1.09E-77
**LINP1**	177.5700144	-6.361514395	0.430859669	7.83E-51	1.18E-48
**LINC01873**	5.215234655	-6.363552561	2.890522797	0.0001033	0.0006450
**AP002371.1**	5.44414777	-6.371205568	2.869762824	8.29E-05	0.0005298
**LINC02582**	935.5067745	-6.373340034	0.281873853	5.6E-113	6.69E-110
**PTPRB**	160.831113	-6.380474617	0.461441681	3.01E-43	3.43E-41
**CUBN**	6.203048558	-6.396720317	3.055643643	0.0002020	0.0011755
**CRNDE**	5.235038611	-6.403867025	2.920833559	0.0001171	0.0007204
**ENSG00000288543**	24.94678434	-6.406886737	1.092828207	8.5E-10	1.21E-08
**AL162388.1**	5.165057141	-6.409138151	2.848389867	7.12E-05	0.0004615
**AC129507.1**	5.054939343	-6.410025552	2.849042509	7.32E-05	0.0004738
**BX005019.1**	15.42414084	-6.420154558	1.405877823	4.96E-08	5.63E-07
**LPAR1**	791.4543119	-6.428212036	0.311207413	8.17E-96	5.92E-93
**CDH12**	25.06966119	-6.43907759	1.093312646	7.34E-10	1.06E-08
**SIRPA**	62.59950476	-6.439288654	0.731121024	1.25E-18	4.1E-17
**RNA5SP237**	5.124373451	-6.45818727	2.822851457	5.34E-05	0.0003553

**CSMD2**	5.324267202	-6.465018021	2.813794559	4.68E-05	0.00031564
**FSCN1**	994.6578632	-6.469910571	0.326050566	5.18E-87	3.02E-84
**RN7SKP172**	5.354102872	-6.474045457	2.821595474	4.85E-05	0.00032573
**DOCK2**	26.22428689	-6.482334213	1.121006424	1.2E-09	1.68E-08
**LINC02192**	5.264326919	-6.491913551	2.809333854	4.24E-05	0.00028905
**ENSG00000287424**	53.99422771	-6.500207985	0.731560809	2.06E-18	6.59E-17
**KCNN2**	54.11391952	-6.503510507	0.771545794	4.83E-17	1.37E-15
**CHRDL1**	6.283061741	-6.50425192	2.987380232	0.00011715	0.00072046
**PKIA-AS1**	24.57029267	-6.508069798	1.123911116	2.05E-09	2.79E-08
**AL162718.1**	6.053601265	-6.511097966	2.914060548	7.58E-05	0.00048817
**PLA2G4D**	15.7183782	-6.518400222	1.423288976	6.23E-08	6.97E-07
**ENSG00000287972**	45.66413675	-6.519624117	0.792451337	2.41E-16	6.48E-15
**PAQR5**	71.96084409	-6.520150055	0.71254425	2.01E-19	6.99E-18
**CD300C**	5.772887496	-6.524103999	2.954355132	0.00010560	0.00065823
**PRDM9**	96.29795326	-6.527394607	0.55177652	8.69E-32	6.26E-30
**AGMAT**	5.534471085	-6.529329151	2.824577826	4.17E-05	0.00028474
**LINC01630**	5.473723967	-6.535895561	2.915861782	8.54E-05	0.00054374
**AC010127.1**	82.74779164	-6.539686603	1.631946186	9.85E-07	9.13E-06
**RELN**	170.0295336	-6.541112513	0.456377805	3.93E-48	5.43E-46
**SYNM**	209.8369889	-6.569978354	0.396278307	3.36E-62	8.38E-60
**PRAME**	82.95032414	-6.570305685	0.717539487	1.27E-20	4.91E-19
**AC007012.2**	5.394248672	-6.582955732	2.812897003	3.44E-05	0.00023943
**PART1**	7.442297393	-6.601263271	3.194129336	0.00023896	0.00137016
**RNU2-3P**	7.442297393	-6.601263271	3.194129336	0.00023896	0.00137016
**DNAJA4**	27.67229465	-6.607711562	1.06452839	1.06E-10	1.68E-09
**GALNT14**	131.6169265	-6.617202521	0.578805403	6.32E-30	4.13E-28
**LINC02407**	129.9828527	-6.630737415	0.528539187	4.76E-35	3.92E-33
**PNMA2**	6.332970311	-6.631639797	2.91677993	5.83E-05	0.00038631
**RN7SKP179**	5.88435949	-6.637760397	2.849537872	3.68E-05	0.00025420
**HECW1**	81.64253711	-6.687207604	0.768516362	2.23E-19	7.72E-18
**AC110058.1**	6.10324089	-6.690278565	2.844141185	2.98E-05	0.00020959
**AL021937.3**	5.853448042	-6.693026937	2.899165148	5.18E-05	0.00034597
**ENSG00000287597**	5.92450529	-6.698543173	2.858699036	3.5E-05	0.00024292
**CDK18**	55.43568156	-6.700946867	0.813920166	6.4E-17	1.8E-15
**AC036108.3**	5.814387492	-6.703302736	2.853824019	3.4E-05	0.00023699

**LEMD1**	17.02626379	-6.708250069	1.384927781	1.29E-08	1.57E-07
**ADAMTS12**	238.834075	-6.716196632	0.454714393	2.21E-48	3.09E-46
**PAPPA**	6.043300607	-6.731791863	2.826173845	2.25E-05	0.00016186
**FST**	87.8806183	-6.752696118	0.625570987	7.88E-27	4.4E-25
**MYH16**	20.14615869	-6.764168746	1.418135827	1.07E-08	1.32E-07
**LINC02220**	6.033816254	-6.764250037	2.891086768	3.94E-05	0.00027090
**NR5A2**	40.41861157	-6.776325909	0.924890804	4.16E-13	8.51E-12
**MUC22**	116.8809086	-6.7970734	0.738422533	1.61E-20	6.16E-19
**AC107029.1**	5.943492939	-6.800087596	2.814939111	1.78E-05	0.00013023
**SLC25A21**	104.8786178	-6.846665364	0.574083626	5.97E-32	4.33E-30
**MTND1P6**	6.313992134	-6.849640726	2.833133143	1.75E-05	0.00012806
**LINC02516**	6.87353706	-6.871601378	2.901123356	2.81E-05	0.00019821
**LINC01807**	20.1773675	-6.873807985	1.382847794	3.47E-09	4.55E-08
**AC010967.1**	33.17936951	-6.874697358	1.056783179	1.57E-11	2.74E-10
**RN7SKP89**	6.243472775	-6.8953911	2.79640978	1.05E-05	8.07E-05
**AC112236.1**	6.822274295	-6.903301845	2.86544679	1.9E-05	0.00013831
**COCH**	21.33523949	-6.903427892	1.387690204	3.18E-09	4.22E-08
**EEF1A2**	515.6646326	-6.911765907	0.31973386	2.5E-105	2.21E-102
**SLC14A1**	6.592006984	-6.912060732	2.888819882	2.47E-05	0.00017594
**AC087821.1**	6.79216968	-6.929684137	2.861383008	1.73E-05	0.00012678
**AXL**	725.5721177	-6.936492518	0.69825578	3.92E-24	1.9E-22
**TBKBP1**	19.56229071	-6.95397358	1.427926949	1.11E-08	1.36E-07
**ENSG00000285551**	6.751764408	-6.981379927	2.893699803	2.13E-05	0.00015319
**ABHD11-AS1**	6.603123949	-6.991792787	2.79253795	6.95E-06	5.54E-05
**LRAT**	49.06079989	-7.00192956	0.913781659	3.53E-14	7.97E-13
**AC004988.1**	7.881423862	-7.013227187	2.966446205	3.01E-05	0.00021147
**LINC01968**	7.082655691	-7.021786126	2.82102453	8.11E-06	6.35E-05
**NKILA**	7.650618663	-7.027755278	3.000354255	4.09E-05	0.00027952
**ENSG00000285743**	34.65251566	-7.047072078	1.098464358	4.77E-11	7.82E-10
**SULF2**	102.8231956	-7.04769147	0.675240282	1.5E-24	7.42E-23
**B4GALNT1**	36.47493535	-7.048592838	1.033806453	2.54E-12	4.82E-11
**IL1R2**	2031.136228	-7.057857394	0.851424649	3.98E-18	1.25E-16
**LINC00911**	7.102728591	-7.071997705	2.807076322	5.87E-06	4.73E-05
**CLDN14**	25.76689998	-7.076097011	1.508429144	1.32E-08	1.6E-07
**PSD3**	314.3399554	-7.088652815	0.812157417	7.98E-19	2.65E-17

**RTL1**	7.820945689	-7.093377866	2.940922588	2.06E-05	0.00014897
**CCDC190**	38.8044518	-7.110106849	1.085098534	9.28E-12	1.65E-10
**ROCR**	7.79111002	-7.121919802	2.904189639	1.36E-05	0.00010189
**RFLNA**	67.59295092	-7.12796165	0.799315373	2.07E-18	6.63E-17
**MMP1**	125.8582621	-7.132267594	0.600701653	2.62E-31	1.85E-29
**MECOM**	8.350654946	-7.150667579	2.975071614	2.24E-05	0.00016089
**LINC02448**	22.59925753	-7.172914279	1.376895942	1.11E-09	1.57E-08
**NAPGP2**	7.331382234	-7.179455297	2.845100026	6.59E-06	5.27E-05
**ENSG00000288011**	7.471604646	-7.197403455	2.818928543	4.31E-06	3.55E-05
**FRMD5**	159.6825084	-7.223644516	0.595022529	2.04E-34	1.63E-32
**TEX48**	7.921031774	-7.240673842	2.854359093	5.45E-06	4.41E-05
**PLEKHB1**	7.341692364	-7.244424664	2.833117872	4.55E-06	3.74E-05
**ST6GALNAC5**	259.3593122	-7.259484126	0.913733535	6.33E-16	1.65E-14
**AL365356.5**	63.1654632	-7.317956259	0.879220286	9.47E-17	2.63E-15
**CAVIN2**	8.101399986	-7.323068339	2.816950084	2.42E-06	2.1E-05
**PDE1C**	45.34900412	-7.325046822	1.11306706	6.18E-12	1.12E-10
**LIF**	467.6120441	-7.3351636	0.921982649	3.46E-16	9.23E-15
**AL049775.1**	7.962541242	-7.342907364	2.874255042	5.14E-06	4.18E-05
**RN7SL93P**	7.811730282	-7.346247567	2.792461111	1.63E-06	1.46E-05
**ADAMTS20**	8.042554426	-7.35305231	2.880427791	5.32E-06	4.31E-05
**FLRT3**	154.2054118	-7.360641891	0.55234565	3.13E-40	3.16E-38
**HDGFL1**	44.87581517	-7.374137254	1.075634586	1.5E-12	2.91E-11
**BLACAT1**	263.4388659	-7.405568249	0.45682639	1.54E-57	2.97E-55
**TLR4**	109.5249027	-7.41003066	0.737641623	2E-24	9.82E-23
**LINC00707**	1153.503678	-7.41177309	0.318519552	1.76E-117	2.33E-114
**LINC01035**	10.10958132	-7.440061554	3.053959027	1.86E-05	0.00013578
**IGFN1**	104.473918	-7.445553638	0.702062538	1.21E-25	6.37E-24
**RAB6B**	223.4511681	-7.448545854	0.480602285	5.79E-54	1.01E-51
**C4orf50**	104.7885056	-7.467966995	0.715961264	5.31E-25	2.68E-23
**DKK 1.00**	242.8748045	-7.471785243	0.495034714	3.68E-51	5.65E-49
**ST6GAL1**	8.211527257	-7.474899371	2.801225355	1.05E-06	9.74E-06
**KCNJ4**	9.719556591	-7.527305522	2.914496458	3.64E-06	3.05E-05
**C8orf48**	29.37351534	-7.542925179	1.350436626	6.86E-11	1.11E-09
**LINC02487**	10.13807227	-7.560902993	3.011212304	9.64E-06	7.45E-05
**LINC00900**	51.59797497	-7.577439143	1.021761375	3.58E-14	8.05E-13

**LINC02315**	9.369946602	-7.593087995	2.828655241	9.03E-07	8.42E-06
**DNAH5**	124.276153	-7.666926972	0.682451237	2.9E-29	1.83E-27
**LINC02475**	9.490921893	-7.676537851	2.832417854	6.94E-07	6.6E-06
**KCNJ12**	9.928953639	-7.688981154	2.855300554	8.95E-07	8.35E-06
**AC132825.4**	9.898849025	-7.707087589	2.849320011	7.61E-07	7.17E-06
**FLRT2**	498.4046751	-7.722513111	0.418003926	6.63E-76	2.64E-73
**LINC01479**	9.730145139	-7.723987701	2.812506275	3.82E-07	3.78E-06
**RPS23P5**	9.770031467	-7.732926138	2.914228551	1.94E-06	1.72E-05
**LRRC38**	84.13535418	-7.76518283	0.924700263	6.33E-17	1.79E-15
**AC107308.1**	9.579891012	-7.792088285	2.813383521	2.73E-07	2.76E-06
**THAP12P8**	9.78928806	-7.797026448	2.874549022	8.22E-07	7.72E-06
**ENSG00000286954**	106.8192539	-7.810853054	0.763703959	1.47E-23	6.86E-22
**LINC01468**	295.1958042	-7.854599093	0.474976965	2.95E-61	7.05E-59
**AL035409.1**	35.84644859	-7.856010548	1.340200078	7.77E-12	1.39E-10
**DNER**	137.9780538	-7.863556827	0.678976465	3.9E-30	2.57E-28
**AL451142.1**	10.40769749	-7.878545914	2.908462826	9.78E-07	9.08E-06
**LRRN3**	90.4787527	-7.899354888	0.845171473	2.55E-20	9.55E-19
**MFNG**	10.32930745	-7.923248711	2.851697218	2.95E-07	2.97E-06
**BLK**	171.6355124	-7.925927856	0.649485675	1.82E-33	1.42E-31
**ZNF365**	378.0308859	-7.930726642	0.442152427	5.64E-70	1.93E-67
**PCNPP3**	10.86823211	-7.953123786	2.809469844	1.13E-07	1.21E-06
**HDAC9**	2691.418725	-7.964069089	0.252992517	1.1E-218	2.64E-214
**TLL1**	67.03338429	-7.975853422	1.01595055	1.14E-15	2.93E-14
**ENSG00000286635**	10.8381275	-7.980131795	2.817993425	1.04E-07	1.12E-06
**BMP5**	11.69873751	-8.006717959	2.874057215	2.79E-07	2.82E-06
**MAP9**	72.44265319	-8.026948333	1.02313442	7.31E-16	1.89E-14
**ZG16B**	41.01093943	-8.028841908	1.373657984	8.71E-12	1.55E-10
**AL359692.1**	11.70823133	-8.047140665	2.839602619	1.13E-07	1.22E-06
**AC113346.1**	11.90596406	-8.078141021	2.892549231	2.72E-07	2.76E-06
**RIMS2**	70.51415992	-8.082914714	1.116324317	1.48E-13	3.14E-12
**SCHLAP1**	11.59867037	-8.116784625	2.875971818	1.71E-07	1.79E-06
**HMGA2**	1271.283106	-8.130812479	0.780335579	6.12E-27	3.45E-25
**FAM167A**	589.6855704	-8.139968799	0.437237871	1.73E-78	7.66E-76
**FCRL1**	12.12701596	-8.176086416	2.8261919	3.73E-08	4.31E-07
**SEMA3D**	850.7853336	-8.247604501	0.362566804	6.26E-114	7.87E-111

**VEGFC**	254.3773941	-8.284008318	0.57529327	4.19E-46	5.25E-44
**IL12A**	13.53443079	-8.298315547	2.899988674	9.66E-08	1.05E-06
**LINC00939**	13.38633769	-8.310801532	2.852182819	2.96E-08	3.47E-07
**ANTXR2**	13.15471619	-8.33165104	2.869514016	4.22E-08	4.83E-07
**GAD1**	14.03349754	-8.3448397	2.931009508	1.43E-07	1.52E-06
**CST4**	13.57484554	-8.350097504	2.861671729	2.98E-08	3.5E-07
**FGFR1**	13.53336448	-8.360750044	2.923726993	1.23E-07	1.32E-06
**PAX3**	51.18681718	-8.399600459	1.34140057	2.09E-13	4.36E-12
**AC005291.1**	53.58980689	-8.44823665	1.332319237	9.78E-14	2.1E-12
**AC100757.2**	15.09400131	-8.4747407	2.895908149	3.05E-08	3.57E-07
**AC005323.2**	13.90521788	-8.50087794	2.849609891	8.4E-09	1.05E-07
**CASC17**	14.41512318	-8.507533173	2.844152555	6.47E-09	8.18E-08
**AC015574.1**	101.4856353	-8.543955944	1.006759894	3.57E-18	1.12E-16
**SPATC1L**	14.44442096	-8.549734214	2.845228441	5.03E-09	6.46E-08
**LARP6**	15.44254499	-8.599830635	2.87916935	9.35E-09	1.16E-07
**USH2A**	110.2805196	-8.698552416	1.062796044	5.24E-17	1.49E-15
**PID1**	15.82336379	-8.731204712	2.860343895	2.06E-09	2.81E-08
**SGSM1**	16.2426863	-8.774939702	2.861139087	1.51E-09	2.1E-08
**STAC**	66.37133056	-8.781057123	1.352336568	3.64E-14	8.16E-13
**AC073648.4**	16.32324685	-8.78773143	2.877140059	2.38E-09	3.22E-08
**RGS7**	65.06486548	-8.795675099	1.325061597	1.07E-14	2.52E-13
**DOCK10**	636.0600465	-8.858659832	0.454641105	5.32E-84	2.76E-81
**AP000997.3**	18.15078155	-8.87214679	2.888550708	1.65E-09	2.26E-08
**DPP6**	125.9903438	-8.885891138	1.030939777	1.36E-18	4.43E-17
**ARL14EPL**	535.2752828	-8.89781716	0.498655377	2.06E-69	6.93E-67
**ATP8A2**	367.7521492	-9.004624497	0.623998147	3.29E-47	4.35E-45
**AC092327.2**	20.0955358	-9.093291136	2.955638305	3.02E-09	4.01E-08
**AC074237.1**	20.67919727	-9.1004659	2.915698971	6.95E-10	1E-08
**POPDC3**	20.23657452	-9.154848747	2.921727133	6.11E-10	8.89E-09
**SEMA3A**	1761.287674	-9.246651936	0.331547519	1.45E-174	8.65E-171
**ENSG00000287014**	21.81784108	-9.277946214	2.902832615	9.34E-11	1.49E-09
**LHX1-DT**	22.88756972	-9.318971049	2.92588624	1.56E-10	2.43E-09
**AC069120.1**	24.49378358	-9.379254681	2.95104319	2.54E-10	3.86E-09
**ZPLD1**	1899.316727	-9.489017259	0.343557084	8.5E-171	3.39E-167
**NRP1**	25.87791712	-9.508806547	3.010676552	8.31E-10	1.19E-08

**NBPF21P**	25.93160986	-9.599234045	2.931176089	1.46E-11	2.56E-10
**CST1**	134.0901128	-9.733926953	1.412814657	4.23E-16	1.12E-14
**ICAM2**	32.58513291	-9.847787646	3.02577086	8.88E-11	1.42E-09
**SHISA2**	1037.588724	-9.949784913	0.507902599	1.53E-85	8.1E-83
**C7**	33.02322149	-10.00270482	2.969492238	1.57E-12	3.04E-11
**GNG2**	41.99581056	-10.39695452	3.000104912	7.91E-14	1.71E-12
**LINC00973**	49.07519155	-10.61551785	3.05899589	2E-13	4.17E-12
**COL19A1**	52.37337502	-10.75023643	3.043996058	1.52E-14	3.52E-13
**LHX1**	52.1181983	-10.7816555	3.065964342	4.03E-14	9E-13
**ADARB2**	54.59098182	-10.8547806	3.04789696	4.24E-15	1.04E-13
**AL359924.1**	59.31663408	-10.97704573	3.056727012	1.86E-15	4.68E-14
**CYTH4**	69.04646291	-11.19002813	3.109112454	3.8E-15	9.38E-14
**RNF182**	74.10637138	-11.37547086	3.115269763	3.97E-16	1.05E-14
**THSD7B**	172.6778038	-12.75611674	3.255257878	2.5E-20	9.39E-19
