# Supplementary material for: Role of Forkhead Box P3 in IFNγ-Mediated PD-L1 Expression and Bladder Cancer Epithelial-to-Mesenchymal Transition
Source: Cancer Res Commun. 2024 Aug 26;4(8):2228–41. doi: 10.1158/2767-9764.CRC-23-0493 (PMC11345674; doi:10.1158/2767-9764.CRC-23-0493)
Supplement: Supplementary Table 4 — Genes and level of expression by z score of 271 IFNgamma induced and FOXP3-dependent genes [file crc-23-0493_supplementary_table_4_suppst4.pdf]

Fig2F_zscore_271genes_4group

	HT1376 WT	HT1376 IFNg	HT1376 KO	HT1376 KO IFNg
**ABCA6**	-0.356302842247622	1.32631270533814	-0.459935512822846	-0.510074350267676
**AC004067.1**	-0.432471602257916	1.49780638810171	-0.393160480942931	-0.672174304900866
**AC005323.2**	0.0218073760345578	1.48960796664946	-0.678074975592633	-0.833340367091383
**AC005515.1**	-0.69218735485361	1.62320356881129	-0.688924112555748	-0.242092101401933
**AC007012.2**	-0.49886785974336	1.63002233279537	-0.565577236526007	-0.565577236526007
**AC007991.2**	-0.654413493345366	1.52664742883841	-0.63696361570097	-0.235270319792077
**AC008632.1**	0.0711860711841846	1.39718053503894	-0.755232738564646	-0.71313386765848
**AC009133.1**	-0.43405746305703	1.444135129232	-0.563679592828839	-0.44639807334613
**AC009549.1**	-0.452329859897094	1.40580095613065	-0.454626679358722	-0.498844416874836
**AC016831.7**	-0.315211108538134	1.61353133161215	-0.776220915362537	-0.522099307711483
**AC025252.2**	-0.480976110271633	1.41859795530275	-0.480976110271633	-0.456645734759484
**AC068299.1**	-0.478234820358037	1.39902179810041	-0.493233839005	-0.427553138737373
**AC083837.1**	-0.618349018421183	1.61739642518266	-0.616366235497582	-0.382681171263898
**AC093844.1**	-0.396087546565952	1.5143447426522	-0.578905727798191	-0.539351468288053
**AC105233.5**	0.0508590187199656	1.40207607954104	-0.6885909682574	-0.764344130003606
**AC108673.2**	-0.3241945703387	1.5212426214343	-0.625269726961379	-0.571778324134224
**AC124319.1**	-0.660797614751559	1.62908238060873	-0.516757184018489	-0.451527581838678
**AC124319.4**	-0.498021093763241	1.6231048019897	-0.603053587054436	-0.522030121172025
**ACSL5**	-0.611593175724308	1.62414127389489	-0.732885574757489	-0.279662523413093
**ACSS1**	-0.24535813917616	1.60179462595793	-0.796734964416246	-0.559701522365521
**ACY3**	-0.458516057632182	1.61746776291355	-0.60741604680898	-0.551535658472393
**ADGRE1**	0.0198839708245536	1.52658626961985	-0.885115021029491	-0.661355219414918
**ADGRE5**	0.493590706281414	1.31417529084235	-0.93539574706406	-0.8723702500597
**ADGRF1**	0.517759121293135	1.28720093212983	-0.900217554852775	-0.904742498570196
**AF165147.1**	-0.273497519507606	1.45269888355043	-0.651333740437651	-0.527867623605177
**AIM2**	-0.624256801013862	1.624755770079	-0.647212531323701	-0.353286437741434
**AKAP7**	-0.316658149795314	1.47810479208454	-0.548781623946799	-0.61266501834243
**AL031316.1**	-0.133607891462432	1.47139705602413	-0.665996322784592	-0.67179284177711
**AL049629.1**	-0.0437488723800887	1.53455881227745	-0.740034336473863	-0.750775603423504
**AL078459.1**	-0.215967990618973	1.43166664055474	-0.677633867311924	-0.538064782623839

**AL138828.1**	-0.0918771500055634	1.34448581492565	-0.732486304324533	-0.520122360595559
**AL160272.1**	-0.350307202305456	1.27137487721254	-0.558446165285324	-0.362621509621759
**AL160408.1**	-0.135286445033585	1.26826996235714	-0.60630329000926	-0.526680227314299
**AL354872.2**	0.00292238166978634	1.45173742413912	-0.744214375943107	-0.710445429865796
**AL356234.1**	-0.0829873780461409	1.21550019233128	-0.56521601503572	-0.567296799249414
**AL359924.1**	0.0689340950080849	1.51092744374167	-0.763425179999495	-0.816436358750258
**AL365184.1**	0.0778696319622936	1.45678263001184	-0.761456053740813	-0.773196208233316
**AL591468.1**	-0.575836930072385	1.51775132258746	-0.533714778327382	-0.40819961418769
**ANK2**	-0.0438954333404167	1.29608911935839	-0.647264252702901	-0.604929433315075
**ANO4**	-0.529309317569343	1.60149491527324	-0.52611874762842	-0.54606685007548
**APOBEC3A**	-0.454136337997913	1.46045703032375	-0.540198094589792	-0.466122597736048
**APOBEC3C**	-0.226029918499245	1.61816178528441	-0.738335281416243	-0.653796585368926
**APOBEC3F**	-0.515113090731871	1.63318996780909	-0.647643398946367	-0.47043347813085
**APOBEC3G**	-0.582019158785594	1.63235226831442	-0.663209172120234	-0.387123937408596
**BATF2**	-0.617025299607642	1.64857247905946	-0.646358064028963	-0.385189115422861
**BEND7**	-0.5200522328929	1.53143193650417	-0.484609479546613	-0.526770224064659
**BEST3**	-0.175923461916241	1.50889558728909	-0.657180249156552	-0.675791876216295
**BTC**	-0.0386087573518162	1.48810545569845	-0.746454916297137	-0.703041782049493
**C1orf74**	0.0995101882379716	1.36209534715167	-0.73810672264568	-0.723498812743959
**C4orf50**	0.393066698822751	1.30723359505979	-0.851238742176753	-0.849061551705791
**CARD16**	-0.188169984335286	1.60041883785119	-0.723368178517466	-0.688880674998441
**CARD17**	-0.347130421210375	1.45010357088656	-0.566534945746	-0.53643820393018
**CARD6**	0.125191808329591	1.47409522987429	-0.788379461817116	-0.810907576386762
**CASC17**	-0.2655248897731	1.58822681685619	-0.661350963541544	-0.661350963541544
**CASP1**	-0.458676630452281	1.64615273267597	-0.622517018071974	-0.564959084151719
**CASP1P2**	-0.270236866122473	1.59901038228975	-0.661526569041335	-0.667246947125946
**CASP4**	-0.226122053056596	1.60479020408718	-0.85663597155589	-0.5220321794747
**CASZ1**	0.404089753572651	1.36844609291721	-1.00917478184184	-0.763361064648016
**CD274**	0.0943695382511735	1.42372245368338	-0.886199148602832	-0.631892843331721
**CD5L**	-0.524690344572025	1.64085627612271	-0.585314333283296	-0.530851598267389
**CD74**	-0.687225430684205	1.62346014600722	-0.686936531186923	-0.249298184136089
**CDH15**	-0.456229000817006	1.49667176403906	-0.520221381611027	-0.520221381611027
**CEACAM1**	0.404314353277287	1.31800567559502	-0.869155052667851	-0.853164976204453

**CLCN3P1**	0.467024288983015	1.30778156189398	-0.875811901019627	-0.898993949857363
**CLMP**	-0.283051782369588	1.34706783512265	-0.54050695203577	-0.523509100717291
**CMPK2**	-0.561541549808174	1.49035911476214	-0.556834248039736	-0.37198331691423
**COL4A1**	-0.608938047363428	1.52056022731668	-0.611036871472573	-0.300585308480684
**CRYZL2P-SE**	-0.433623337913555	1.60848481818282	-0.831992308729536	-0.342869171539725
**CT83**	0.0984240514685947	1.49586447190088	-0.824346596988405	-0.769941926381066
**CTH**	0.195937891135567	1.4327063298867	-0.959941567979516	-0.668702653042748
**CTSC**	0.479820605955692	1.30316585000404	-0.888576600883511	-0.89440985507622
**CTSL**	0.436983412281059	1.34049659055726	-0.843012905570162	-0.934467097268157
**CTSS**	-0.588247767305715	1.58943961477932	-0.598166153056005	-0.403025694417605
**CXCL11**	-0.602061859841661	1.45282114231966	-0.596211375528035	-0.254547906949962
**CXCL2**	-0.286175411175047	1.53621096511076	-0.696255777633539	-0.553779776302176
**CXCL9**	-0.593381738295282	1.40638427288271	-0.591427399614833	-0.22157513497259
**CYP27B1**	-0.143845254021271	1.49535605619958	-0.755228260174343	-0.596282542003968
**CYP4F11**	-0.484078618049163	1.54228288487128	-0.628107407344423	-0.430096859477696
**DCBLD2**	0.593104825161044	1.25913943655211	-0.901554291755868	-0.950689969957288
**DOC2A**	-0.0293302404998785	1.29556767845223	-0.653356087716539	-0.612881350235816
**DOCK10**	0.542193076152307	1.28073890505476	-0.912780562840486	-0.910151418366577
**ECM1**	0.442174984622482	1.3549835390001	-0.914362918474463	-0.88279560514812
**EHF**	0.445535020702148	1.28505633057841	-0.861534058381389	-0.869057292899171
**ELK3**	0.561305807442434	1.28016235517108	-0.915593881917492	-0.925874280696026
**ELMO1**	-0.104708253928206	1.44302386954448	-0.634376635220213	-0.703938980396057
**ENSG000002**	-0.58632904569051	1.65317507907522	-0.582723925984856	-0.484122107399853
**ENSG000002**	-0.303587737354608	1.38516232005666	-0.623996334563094	-0.457578248138955
**ENSG000002**	-0.240365648859437	1.58580826669501	-0.672721308917788	-0.672721308917788
**ENSG000002**	-0.293601858135709	1.39810196686063	-0.44243728838389	-0.662062820341036
**ENTPD3**	-0.174064960746084	1.58561615623131	-0.718349997476752	-0.693201198008472
**ETS1**	0.306476541348855	1.3592476311014	-0.906550765638976	-0.759173406811283
**F2RL1**	0.424164268765494	1.30705177768246	-0.883780984698098	-0.847435061749855
**FAM167B**	-0.236137275098079	1.37728932918017	-0.6006953894857	-0.540456664596385
**FAM46C**	-0.507483768774389	1.22416769502961	-0.330504624262069	-0.38617930199315
**FAP**	-0.48903336939442	1.35709152891127	-0.272632204662888	-0.595425954853962
**FCRL1**	-0.537709814234767	1.6131294427043	-0.537709814234767	-0.537709814234767

**FGD2**	-0.451794147936426	1.54360141039449	-0.618209726904103	-0.47359753555396
**FMO4**	-0.185667944499321	1.39068060537998	-0.60549124795617	-0.599521412924491
**GBP7**	-0.477928168931397	1.39380986062392	-0.477928168931397	-0.437953522761126
**GLRX**	-0.597138486951059	1.60750903932897	-0.492256894076431	-0.51811365830148
**GSAP**	-0.450862953184695	1.63459540288411	-0.764052774527755	-0.419679675171657
**GSDMC**	-0.324579660986269	1.63020874595757	-0.690382131677388	-0.615246953293912
**GVINP1**	-0.52896810191004	1.57504497384526	-0.683362440935557	-0.362714430999659
**HAPLN3**	-0.438730278092399	1.65205590025578	-0.633520442754272	-0.579805179409104
**HAS3**	0.271916154568388	1.43041495297633	-0.865533824836736	-0.836797282707985
**HCP5**	-0.559799614308468	1.63731863474556	-0.643258540122787	-0.434260480314304
**HDAC9**	-0.0125649717977943	1.52732224703905	-0.759539715118434	-0.755217560122817
**HELZ2**	-0.187900931593883	1.57681797037081	-0.926848721934454	-0.462068316842478
**HERC5**	0.0337804016952674	1.33285434814845	-0.826642376092507	-0.539992373751208
**HLA-DOA**	-0.71352421307513	1.61034679860479	-0.712850189749711	-0.183972395779949
**HLA-DPA1**	-0.695878407517268	1.61056299596183	-0.709069831424381	-0.205614757020185
**HLA-DQA1**	-0.686055510770174	1.57593988541715	-0.684265555961196	-0.205618818685784
**HLA-DQB1**	-0.672632366619163	1.60800267323516	-0.745223530854826	-0.190146775761176
**HLA-DRB1**	-0.68653432789488	1.6162048247089	-0.687776215052454	-0.241894281761566
**HLA-DRB5**	-0.58306920843339	1.45401216505425	-0.58306920843339	-0.287873748187465
**HLA-F**	-0.600725107985944	1.61683719557246	-0.74733050885818	-0.268781578728332
**HMCN2**	-0.267000874405143	1.1954880343554	-0.545744186818412	-0.382742973131846
**HMGN2P46**	-0.222716816942514	1.30337588717919	-0.639809979772262	-0.440849090464419
**HNF4G**	-0.338032491452544	1.61536796083323	-0.614381830925812	-0.66295363845487
**HSH2D**	-0.0149965216374585	1.5027656850373	-0.748772937995618	-0.738996225404222
**HVCN1**	-0.370832647599033	1.49174258784711	-0.57725443765622	-0.543655502591856
**ICAM1**	-0.579297513545822	1.58377336469809	-0.594447586568388	-0.410028264583876
**ICAM4**	-0.47567362562618	1.41503301777025	-0.544890718999416	-0.394468673144657
**IDO1**	-0.634351486658853	1.58731742708931	-0.637645262664152	-0.315320677766304
**IFI44**	-0.412873599443729	1.40536150586907	-0.641635040459381	-0.350852865965962
**IGFBP6**	0.193540878473626	1.44226469436714	-0.867652742930556	-0.768152829910213
**IGFN1**	0.0977375845604565	1.4806109916934	-0.762207029993508	-0.816141546260346
**IL12A**	-0.207019953942082	1.36012233092366	-0.568380176521777	-0.584722200459799
**IL18R1**	-0.38271313203471	1.59992563013103	-0.690032539361076	-0.527179958735248

**IL22RA1**	0.0802430793563701	1.43776277451454	-0.825326195072508	-0.692679658798403
**IL32**	-0.53517361582678	1.59359728558944	-0.58719052923902	-0.471233140523637
**IL3RA**	-0.44261750471899	1.50485384565373	-0.54512486911865	-0.51711147181609
**IL7**	-0.598041983087158	1.57365878591017	-0.627619231909385	-0.347997570913632
**ILDR1**	0.0304078728888251	1.48159083075619	-0.809883910846527	-0.702114792798488
**INAVA**	0.386411782427389	1.35893939281424	-0.814413480954037	-0.930937694287588
**ISG20**	-0.318811051356192	1.58700363522094	-0.776309628203604	-0.491882955661141
**ITK**	-0.59788328978667	1.61930581169921	-0.619478723794369	-0.401943798118175
**KDR**	-0.483960982531323	1.51623683294999	-0.495444397917787	-0.536831452500876
**KIF13B**	0.445692086261048	1.32878378197191	-0.864060118754673	-0.910415749478287
**KLHDC7B**	-0.609861565581054	1.62070936809025	-0.640605104465857	-0.370242698043342
**KLK10**	0.179721243936991	1.41561262100534	-0.816207329512526	-0.779126535429807
**KYNU**	-0.151082656733063	1.34740756841601	-0.613563887122313	-0.582761024560634
**LAMP3**	-0.441508009340233	1.49544759849063	-0.602317718236942	-0.451621870913451
**LAT2**	0.376622634090578	1.39549996788717	-0.901107897486153	-0.871014704491595
**LGALS9**	-0.378701528328804	1.62285938928542	-0.661080096301715	-0.583077764654903
**LIF**	0.321406151725838	1.33693459528957	-0.828803626173407	-0.829537120842001
**LINC00431**	0.526764610590304	1.27541099416176	-0.869622252545023	-0.932553352207042
**LINC00520**	-0.147302279458667	1.39666724033704	-0.688187951607784	-0.561177009270587
**LINC00900**	0.313569847168615	1.40285817633333	-0.858882128265346	-0.857545895236599
**LINC00973**	-0.0102881481376707	1.34671744977838	-0.664399389090253	-0.672029912550459
**LINC01225**	-0.483731013427591	1.63605733950087	-0.590355743201944	-0.561970582871339
**LINC01844**	0.090968221669111	1.31599145263014	-0.716297720767391	-0.690661953531858
**LINC02407**	0.189627036058206	1.43663276337486	-0.812434001734492	-0.813825797698576
**LINC02450**	-0.331885147294845	1.4553699681598	-0.590636278080597	-0.532848542784353
**LIPH**	-0.0366910875075175	1.44702524404551	-0.672393281291765	-0.737940875246228
**LMO2**	-0.342436344517482	1.53890433753553	-0.582765228963602	-0.613702764054449
**LRCH2**	-0.559690479336808	1.40651957394099	-0.515082758989719	-0.331746335614458
**LRRN3**	0.422689712258718	1.36559588213358	-0.8856055127047	-0.902680081687597
**LTF**	0.0826590287765241	1.45471815518808	-0.785495253045167	-0.751881930919441
**LY6E**	0.600875679229972	1.2321216587799	-0.924872546084219	-0.90812479192565
**LYST**	0.404060571121117	1.36594234325452	-0.889628891273202	-0.880374023102434
**MAFF**	0.108149003383573	1.46873014932961	-0.872592904829278	-0.704286247883909

**MAGEA1**	-0.0451242165513004	1.55669269902005	-0.75827064368011	-0.753297838788642
**MAP2**	0.504989926130137	1.31406795158554	-0.910603483186449	-0.908454394529228
**MARCKSL1**	-0.390902019482228	1.29289469394231	-0.564395381220199	-0.337597293239888
**ME3**	0.487401064079102	1.2955436888917	-0.901111699790781	-0.881833053180024
**MICB**	-0.266253785449947	1.59532591406575	-0.765877834739953	-0.563194293875853
**MLKL**	-0.209003526307159	1.59875009702157	-0.80607171997838	-0.583674850736028
**MMP1**	-0.289291935937886	1.5882998584255	-0.642518051628157	-0.656489870859455
**MMP25**	-0.569582520852593	1.51034592279678	-0.560414572514578	-0.380348829429608
**MMP25-AS1**	-0.626106197338838	1.63644307911463	-0.660707904954769	-0.349628976821019
**MPZL2**	0.502935593402253	1.29149383073507	-0.940546984490402	-0.853882439646925
**MUC1**	-0.241709478227076	1.54953888595014	-0.731598054622304	-0.576231353100756
**MUC13**	-0.182437762400982	1.53990494301947	-0.711785156277354	-0.645682024341135
**MUC16**	0.256748438419532	1.44492395968137	-0.94065634399259	-0.761016054108312
**MUC20**	-0.456944231537743	1.59779561732653	-0.512569144644836	-0.628282241143953
**MUC4**	-0.247563261809147	1.53360748508502	-0.651223128698614	-0.634821094577256
**MX1**	-0.440103966388928	1.46547248012824	-0.641372455088645	-0.38399605865067
**MX2**	-0.497180241697374	1.31201541288088	-0.50440811885648	-0.310427052327028
**NAV3**	-0.140477570322388	1.39857576541518	-0.60532233547862	-0.652775859614172
**NCF2**	-0.208146811978618	1.56856023861076	-0.74987027177718	-0.610543154854958
**NEURL3**	-0.483906280728054	1.2760853817964	-0.464102842443416	-0.328076258624934
**NEXN**	-0.624769846353344	1.50548300265486	-0.65859257903805	-0.222120577263462
**NLRP5**	-0.510304764595453	1.59635707227087	-0.591275316196576	-0.494776991478843
**NOS3**	0.0123143007940263	1.51854442128296	-0.778890172816828	-0.751968549260157
**NOV**	-0.282893622991194	1.2956227224474	-0.578488993539285	-0.434240105916921
**NUGGC**	-0.552565060373562	1.48224214527324	-0.563390676649626	-0.366286408250049
**OAF**	0.23841239607774	1.44412996399048	-0.895531902909538	-0.787010457158684
**OASL**	-0.50552560646841	1.43752433670302	-0.655635709562911	-0.276363020671699
**OR10A3**	-0.289722289113397	1.34461824427184	-0.53186965876677	-0.523026296391669
**OR10A6**	-0.338592865774041	1.56013090594285	-0.591570798711946	-0.629967241456864
**OR2I1P**	-0.475292923397419	1.34922268314568	-0.475292923397419	-0.398636836350836
**PALM2-AKAI**	0.441848658296659	1.34752634836209	-0.898446106763078	-0.890928899895667
**PARP11**	-0.115734260597452	1.50708051460333	-0.811019951695691	-0.580326302310187
**PCDH7**	0.101341445132187	1.5048541720739	-0.851246035296728	-0.754949581909358

**PDCD1LG2**	-0.44110695192085	1.63179702519653	-0.73312257696737	-0.457567496308307
**PDP1**	-0.0518348911273157	1.57340830248968	-0.840856708639734	-0.680716702722629
**PHLDA1**	0.378920180055479	1.36265125003619	-0.915909869241076	-0.825661560850588
**PIGR**	-0.356786158131478	1.60755547750705	-0.651557796739816	-0.599211522635755
**PKN2-AS1**	-0.302328921129938	1.34851330797884	-0.559234060918024	-0.486950325930875
**PLA2G4C**	-0.0612377273125785	1.42603643494461	-0.882286621228868	-0.482512086403164
**PLAUR**	0.170228695190869	1.42327427340257	-0.838876942585403	-0.75462602600803
**PML**	-0.520203172415055	1.61060771746264	-0.704775188588002	-0.385629356459588
**PRDM1**	0.0352242313783167	1.41881730229369	-0.76778440253245	-0.686257131139558
**PRDM9**	0.0422313037346045	1.53207970110502	-0.785118495193502	-0.789192509646125
**PRICKLE1**	-0.105024815865441	1.49036785662349	-0.684496636696131	-0.700846404061917
**PROCR**	0.219973407260842	1.41644033862973	-0.738012536380617	-0.898401209509952
**PROX1**	-0.290403462068315	1.43667980058757	-0.624773517509563	-0.521502821009695
**RBFOX1**	-0.42150962578903	1.61173697815741	-0.504933049342396	-0.685294303025987
**RF00019**	-1.45189361119522	0.008099473968759	0.660577823420765	0.783216313805693
**RGS20**	0.426366760422953	1.34553684650463	-0.905678732224876	-0.866224874702707
**RGS7**	0.170663778216873	1.45105706268432	-0.810475992101297	-0.811244848799901
**RHBDF2**	-0.00751012470908163	1.55606214527819	-0.796854041380129	-0.751697979188982
**RNF213**	-0.556304145606314	1.64439805985292	-0.62830255011953	-0.459791364127073
**RNU6-26P**	0.0407635634885912	1.3802994678401	-0.724760603648049	-0.696302427680645
**RPLP0P2**	-0.302137330274242	1.5491063701028	-0.712567909292925	-0.534401130535637
**RSAD2**	-0.507918801201383	1.42905989067903	-0.560784585334441	-0.360356504143212
**RTP4**	-0.610182482893392	1.5571023714861	-0.701405252062599	-0.245514636530105
**RUFY4**	-0.526686138339512	1.35407438352197	-0.496405249471224	-0.33098299571123
**RYR3**	-0.0913678101635226	1.31931208073793	-0.62634536115716	-0.601598909417245
**SAA1**	-0.494735732817867	1.45363317179643	-0.581094908112533	-0.377802530866035
**SAA2**	-0.561475584161086	1.56682300344944	-0.674333614632489	-0.331013804655867
**SALL4**	0.145040436465079	1.47716349472431	-0.808226966757734	-0.813976964431659
**SAMD9**	-0.0984553086498567	1.54384314917269	-0.858529810469767	-0.58685803005307
**SAMD9L**	-0.582506349823746	1.61588366153938	-0.780676466371149	-0.252700845344483
**SCNN1G**	-0.153657144162353	1.28011416251029	-0.558732582685274	-0.56772443566266
**SEC16B**	-0.420314211938996	1.42857849452336	-0.654449153905239	-0.353815128679128
**SECTM1**	-0.591069938283785	1.6319728942123	-0.725827422938647	-0.315075532989867

**SEMA3D**	0.276309629044053	1.40702203228005	-0.841476142525162	-0.841855518798943
**SHISA2**	0.649485046125963	1.21440503298357	-0.933189412555275	-0.930700666554255
**SLC4A9**	-0.229167416088423	1.55008688149707	-0.611481378743543	-0.709438086665102
**SLC7A11-AS**	-0.370775700384249	1.52741964410478	-0.604372538693756	-0.552271405026774
**SLC8A1-AS1**	-0.207761593945071	1.33448250794969	-0.63241818429638	-0.494302729708239
**SLC9A7**	0.29108253419475	1.34084059362781	-0.862104807659741	-0.769818320162816
**SOCS3**	-0.305727500174941	1.58948833206311	-0.708748906227659	-0.575011925660509
**SP140**	-0.667503100080974	1.60426919352966	-0.598600872582936	-0.33816522086575
**SPRY2**	0.528468322492406	1.27834441719237	-0.934359262508863	-0.872453477175912
**SQOR**	0.234701894779114	1.43363829820927	-0.852738324507177	-0.815601868481206
**STAT5A**	-0.143358448026393	1.45199819597974	-0.795861090608345	-0.512778657345006
**TEC**	0.197996511226951	1.37168208442554	-0.784760508013685	-0.784918087638811
**TEX29**	-0.523891395744157	1.52846504373016	-0.543629204274766	-0.460944443711242
**THEMIS2**	-0.173782114104953	1.49180601249352	-0.718245877141984	-0.599778021246589
**THSD7B**	0.0152428267744113	1.44498988908092	-0.729579179288716	-0.730653536566611
**TLR4**	-0.269895037287453	1.54228454467196	-0.638930607153297	-0.633458900231206
**TMEM154**	-0.155442500864456	1.49741634303648	-0.756800297350353	-0.585173544821671
**TMEM173**	-0.276527124548488	1.45025399511961	-0.597612543849731	-0.576114326721392
**TMEM229B**	-0.484148459334165	1.57903595526685	-0.609538544567791	-0.485348951364894
**TMPRSS3**	-0.443793796970931	1.4733038927051	-0.533740242622731	-0.495769853111437
**TMSB10**	0.217467656735682	1.46363912838665	-0.985746841379381	-0.695359943742946
**TNF**	0.051932050201499	1.4281422113872	-0.80126939099678	-0.678804870591914
**TNFAIP3**	0.401079785416144	1.34004265453602	-0.918276112745255	-0.822846327206912
**TNFRSF10A**	0.513562700722378	1.27545811076671	-0.893833204055435	-0.895187607433649
**TNFRSF10B**	0.43476663988965	1.3488441122879	-0.920997524524115	-0.862613227653436
**TNFRSF1B**	-0.20235837450345	1.60595293468107	-0.835726879539663	-0.567867680637954
**TNFSF15**	0.181086708811939	1.45486614895338	-0.839476034340883	-0.796476823424438
**TPK1**	-0.371720092383419	1.56225058818808	-0.718482063755923	-0.472048432048738
**TRIM17**	-0.487494538753993	1.64609373794935	-0.594419499546971	-0.564179699648383
**TRIM40**	-0.499827316382483	1.49671066175288	-0.505695634681828	-0.491187710688568
**TYMP**	-0.563472856890928	1.6232786586367	-0.697363037509073	-0.362442764236697
**U62317.2**	-0.616274079598462	1.62532051801546	-0.587134564490263	-0.421911873926731
**UBD**	-0.519989781548421	1.49359144340462	-0.518833535189013	-0.454768126667188

**UPP1**	0.378201238329809	1.32110492565813	-0.881363755917398	-0.817942408070546
**USP18**	-0.289509641777241	1.53878033090525	-0.724610289533253	-0.524660399594756
**VEGFC**	0.388520547100159	1.38351830193807	-0.887268797915195	-0.884770051123032
**WDR64**	-0.265048776849014	1.55171439415078	-0.75267553909671	-0.533990078205055
**ZBP1**	-0.610036198056472	1.52053631491563	-0.624119748236454	-0.286380368622704
**ZEB1**	-0.247987239668872	1.39026083388448	-0.624850288650402	-0.517423305565207
**ZFPM2-AS1**	0.564811219943569	1.27119212496893	-0.975466408814376	-0.86053693609812
**ZG16B**	0.075001260103141	1.36166125833041	-0.703217989860823	-0.73344452857273
**ZP4**	-0.0250519105669178	1.36091635183134	-0.684766063152717	-0.651098378111706
**ZSCAN12P1**	0.00639172498176871	1.53060923186096	-0.877605047320453	-0.659395909522274
