# Supplementary material for: Role of Forkhead Box P3 in IFNγ-Mediated PD-L1 Expression and Bladder Cancer Epithelial-to-Mesenchymal Transition
Source: Cancer Res Commun. 2024 Aug 26;4(8):2228–41. doi: 10.1158/2767-9764.CRC-23-0493 (PMC11345674; doi:10.1158/2767-9764.CRC-23-0493)
Supplement: Supplementary Table 6 — Exposure times of antibodies used for immunofluorescence [file crc-23-0493_supplementary_table_6_suppst6.pdf]

Supplementary Table 6

Table 6: Exposure Times for Immunofluorescence

Specificity	Antibody Target	Exposure
Human	FOXP3	1200 milliseconds (ms)
Human	PD-L1	1500 ms
Human	E-cadherin	1200 ms
Human	N-cadherin	1500 ms
Human	ICAM-1	1200 ms
Mouse	PD-L1	500 ms
Mouse	CD8	200 ms
Mouse	CD4	500 ms
Mouse	IFNγ	300 ms
DAPI		40 ms
Mouse	E-cadherin	600 ms
Mouse	N-cadherin	200 ms
Mouse	chromogranin A	150 ms
Mouse	NSE	600 ms
