# Supplementary material for: Role of Forkhead Box P3 in IFNγ-Mediated PD-L1 Expression and Bladder Cancer Epithelial-to-Mesenchymal Transition
Source: Cancer Res Commun. 2024 Aug 26;4(8):2228–41. doi: 10.1158/2767-9764.CRC-23-0493 (PMC11345674; doi:10.1158/2767-9764.CRC-23-0493)
Supplement: Supplementary Table 5 — Normalized gene expression of RNA sequencing [file crc-23-0493_supplementary_table_5_suppst5.pdf]

Fig 2F. normalized_count_matrix

ID	NAME	WT_IFN_11	WT_IFN_12	WT_IFN_13	WT_UTR_11	WT_UTR_12	WT_UTR_13	KO_IFN_11	KO_IFN_12	KO_IFN_13	KO_UTR_11	KO_UTR_12	KO_UTR_13
**ENSG00000000003**	TSPAN6	61.8741523	94.5865099	83.05178838	79.53719702	86.68417112	88.77533027	110.3106098	167.7925762	124.2750443	108.2049615	182.1077785	153.9401895
**ENSG00000000419**	DPM1	901.3939006	896.820242	885.8857428	984.5867758	963.9279829	891.926502	900.8699798	865.5438834	859.1391744	920.0585612	950.5474194	965.0865723
**ENSG00000000457**	SCYL3	327.6517611	399.365264	351.7487508	210.1456469	168.7451864	200.6929475	427.9643101	418.6507843	393.9284424	311.9593333	394.5668533	307.8803789
**ENSG00000000460**	C1orf112	985.7677446	1054.464425	1098.400613	1215.663264	1195.085773	1206.054594	952.961101	1260.936291	1038.751786	1005.483531	1080.230231	1171.467925
**ENSG00000000938**	FGR	18.28099954	42.03844885	15.47043117	16.74467306	8.090522638	9.105162079	7.149761744	0	7.503398903	6.327775524	2.759208765	5.074951301
**ENSG00000000971**	CFH	66.09284451	17.51602035	34.19779522	27.62871054	11.55788948	11.76083435	72.51901198	169.4538889	209.6262069	43.66165111	93.813098	151.4027138
**ENSG00000001036**	FUCA2	1157.327894	1156.057343	1277.531921	1315.294069	1356.896225	1360.842349	820.1798115	755.8972494	962.3109093	941.5729979	980.8987158	1005.686183
**ENSG00000001084**	GCLC	764.9895194	707.6472222	767.8219259	646.34438	683.0712684	670.7469398	793.6235536	739.2841231	802.8636826	864.3741366	822.2442119	858.512595
**ENSG00000001167**	NFYA	374.0573753	623.5703245	504.0103628	518.2476311	509.7029262	554.2767416	459.6275407	526.6361057	506.9483884	501.792599	618.0627633	590.3860013
**ENSG00000001460**	STPG1	323.4330689	353.8236111	330.5786871	367.5455736	357.138785	350.9281218	320.717884	348.8756536	266.3706611	275.2582353	297.9945466	293.5013502
**ENSG00000001461**	NIPAL3	2154.345485	1576.441832	2152.018399	2660.728549	2478.011505	2959.557058	1444.251872	1453.648557	1660.59597	1674.962181	1452.723415	1867.582079
**ENSG00000001497**	LAS1L	281.2461468	213.6954483	264.6257963	293.8690122	204.5746438	309.5755107	1032.629875	848.9307571	972.1591204	1050.410737	972.6210895	1127.485014
**ENSG00000001561**	ENPP4	492.180757	585.0350798	478.769133	535.8295378	496.9892478	516.717948	213.4714578	214.3093301	285.5981208	302.46767	296.6149422	408.5335797
**ENSG00000001617**	SEMA3F	35.15576835	59.5544692	85.49448804	108.8403749	114.4231059	84.60213099	136.8668677	172.7765142	215.253756	174.0138269	333.8642605	216.5312555
**ENSG00000001626**	CFTR	0	0	2.442699658	0	1.155788948	1.13814526	1.021394535	0	0	0.632777552	0	0.845825217
**ENSG00000001629**	ANKIB1	1537.010192	1516.887362	1583.683612	1455.112089	1389.258316	1523.597121	1368.668677	1443.680681	1405.949369	1230.752339	1313.383372	1361.778599
**ENSG00000001630**	CYP51A1	15.46853808	3.50320407	10.58503185	22.60530863	11.55788948	15.93403364	17.36370709	19.93575163	16.4136851	7.593330629	9.657230676	16.07067912
**ENSG00000001631**	KRIT1	167.3414574	171.6569995	122.9492161	144.0041883	109.7999501	135.8186677	197.1291452	206.0027669	203.9986577	187.3021555	284.1985028	214.8396051
**ENSG00000002016**	RAD52	74.53022891	108.5993262	87.9371877	106.3286739	70.50312584	95.98358359	145.038024	157.8247004	159.4472267	148.7027248	122.78479	179.314946
**ENSG00000002079**	MYH16	28.12461468	73.56728548	46.41129351	28.4659442	31.2063016	25.79795922	0	1.661312636	0	0	0	0
**ENSG00000002330**	BAD	168.7476881	164.6505913	157.1470114	254.5190305	357.138785	344.478632	111.3320043	154.5020752	159.4472267	132.883286	175.2097566	138.7153356
**ENSG00000002549**	LAP3	3819.322674	4820.408801	3836.66693	503.1774254	530.5071273	515.5798027	1036.715453	1209.435599	1056.103396	456.2326153	386.289227	519.3366831
**ENSG00000002586**	CD99	1182.640047	1457.332893	1292.188119	1302.735564	1492.123532	1345.667079	1070.421473	1463.616432	1068.765381	1179.497358	1483.074711	1086.885404
**ENSG00000002587**	HS3ST1	2.812461468	0	18.72736405	9.209570182	10.40210053	11.76083435	12.25673442	19.93575163	9.379248629	20.24888168	22.07367012	21.99145564
**ENSG00000002726**	AOC1	1.406230734	0	0	0	1.155788948	0	117.4603715	146.195512	52.52379232	161.3582759	226.2551187	60.89941561
**ENSG00000002745**	WNT16	607.4916772	290.7659378	322.4363549	856.4900269	495.8334588	416.9405469	8.171156279	46.51675381	40.3307691	18.98332657	45.52694462	62.59106604
**ENSG00000002746**	HECW1	84.37384405	224.2050605	291.4954926	133.9573845	224.223056	264.8084638	0	4.983937908	1.406887294	1.898332657	2.759208765	0
**ENSG00000002822**	MAD1L1	36.56199909	66.56087734	56.18209214	49.39678552	30.05051265	51.9753002	49.02693767	53.16200436	39.39284424	50.62220419	34.49010956	38.06213475
**ENSG00000002834**	LASP1	6808.969215	6218.187225	6955.994394	7248.768967	7117.348343	7373.663757	4295.985414	4847.710272	5317.096048	3829.569747	4436.807694	4654.576168
**ENSG00000002919**	SNX11	272.8087624	196.1794279	300.452058	276.2871054	231.1577897	283.3981697	241.0491102	235.9063943	230.7295163	203.7543719	219.3570968	196.2314503
**ENSG00000003056**	M6PR	479.5246803	406.3716722	497.4964971	569.3188839	551.3113283	564.1406672	919.2550814	832.3176307	938.3938253	920.6913387	860.8731346	960.011621
**ENSG00000003096**	KLHL13	4.218692202	10.50961221	8.142332194	10.88403749	8.090522638	10.24330734	0	4.983937908	2.344812157	5.694997971	2.759208765	0
**ENSG00000003137**	CYP26B1	32.34330689	17.51602035	21.17006371	40.18721534	57.78944741	58.04540826	76.60459012	48.17806645	90.04078684	89.22163489	60.70259282	131.1029086
**ENSG00000003147**	ICA1	281.2461468	420.3844885	357.4483833	221.866918	310.9072271	309.5755107	11.23533988	11.62918845	7.034436472	18.35054902	27.59208765	25.3747565
**ENSG00000003249**	DBNDD1	70.31153671	91.08330583	74.09522297	77.86272972	70.50312584	75.87635066	3.064183605	1.661312636	1.875849726	3.796665314	2.759208765	2.53747565
**ENSG00000003393**	ALS2	1161.546586	1170.07016	1290.559653	1214.82603	1111.868968	1171.910236	974.4103863	1149.628344	875.0838971	936.5107775	1106.442715	1015.836085
**ENSG00000003400**	CASP10	44.99938349	66.56087734	67.58135721	15.07020575	30.05051265	38.31755708	28.59904698	19.93575163	24.38604644	19.61610412	17.93485697	12.68737825
**ENSG00000003402**	CFLAR	2718.244009	3832.505253	3530.51524	1980.894823	1865.443362	2220.14202	2630.090927	2204.561868	2153.944448	1844.546565	1546.536513	1623.984416
**ENSG00000003436**	TFPI	312.183223	350.320407	496.6822639	460.4785091	509.7029262	554.2767416	51.06972674	51.50069172	31.88944534	55.68442461	96.57230676	29.60388259
**ENSG00000003509**	NDUFAF7	333.276684	297.772346	329.7644539	359.1732371	286.6356592	313.3693282	363.6164544	398.7150327	360.1631474	322.7165517	353.1787219	356.0924163
**ENSG00000003756**	RBM5	843.7384405	830.2593647	936.3682024	955.2835979	859.9069775	835.7780025	1582.140135	1724.442516	1375.935774	1528.157789	1684.496951	1386.30753
**ENSG00000003987**	MTMR7	30.93707615	49.04485699	35.82626166	51.90848648	26.58314581	56.14849949	5.106972674	6.645250545	4.689624315	1.898332657	1.379604382	3.383300867
**ENSG00000003989**	SLC7A2	800.1452877	511.4677943	746.6518622	1427.483378	748.9512385	1270.549492	112.3533988	36.54887799	203.5296953	172.7482718	55.18417529	204.6897025
**ENSG00000004059**	ARF5	458.4312193	458.9197332	350.1202844	440.3849014	436.8882224	442.7385061	506.6116893	576.4754847	532.7413221	550.5164706	627.719994	611.5316317
**ENSG00000004139**	SARM1	122.3420739	73.56728548	73.28098975	98.79357104	109.7999501	52.35468196	129.7171059	144.5341993	128.9646686	153.1321677	220.7367012	141.2528112
**ENSG00000004142**	POLDIP2	812.8013643	658.6023652	827.260951	1122.730328	1021.71743	1145.732895	913.1267142	807.3979412	1090.806616	995.9918674	838.7994644	1043.748318
**ENSG00000004399**	PLXND1	98.43615139	112.1025303	96.89375311	329.8700592	213.8209554	325.8889261	427.9643101	511.6842919	559.0032183	676.4392035	754.6435971	872.0457985
**ENSG00000004455**	AK2	4398.689736	4203.844885	3773.156739	4240.588452	3977.069771	3852.621705	3660.678013	3516.998851	3628.831295	3720.732008	3635.257547	3884.875221
**ENSG00000004468**	CD38	4.218692202	3.50320407	5.699632536	9.209570182	8.090522638	2.27629052	7.149761744	0	4.220661883	3.163887762	4.138813147	6.766601734
**ENSG00000004478**	FKBP4	1161.546586	886.3106298	1232.749094	1433.344014	1517.550889	1549.774462	1115.362832	822.3497549	1241.812518	1170.638472	815.3461899	1388.845006
**ENSG00000004487**	KDM1A	1639.665036	1198.095792	1449.335131	1820.145961	1778.759191	1611.613688	1817.060878	1770.95927	2166.606433	1954.017082	1844.531059	2589.916814
**ENSG00000004534**	RBM6	985.7677446	910.8330583	1085.372882	1020.587823	859.9069775	954.1451096	1503.492755	1422.083617	1276.046776	1492.722246	1458.241832	1341.478794
**ENSG00000004660**	CAMKK1	54.84299863	73.56728548	71.65252331	72.00209415	85.52838217	66.77118858	9.192550814	19.93575163	13.13094808	10.12444084	20.69406573	9.304077385
**ENSG00000004700**	RECQL	278.4336854	511.4677943	328.1359874	247.8211612	268.143036	268.2228996	642.4571624	812.3818791	651.3888173	546.7198053	670.4877298	607.3025056
**ENSG00000004766**	VPS50	746.7085198	886.3106298	877.7434106	807.930475	851.8164549	846.0213099	905.9769524	907.0766993	828.6566164	908.0357877	939.5105844	883.8873515
**ENSG00000004776**	HSPB6	0	0	1.628466439	0	0	1.517527013	2.04278907	1.661312636	2.813774589	2.53111021	0	0
**ENSG00000004777**	ARHGAP33	39.37446056	70.06408141	26.05546302	52.74572013	71.65891479	54.63097248	58.21948849	64.79119281	67.99955256	75.30052873	55.18417529	49.05786257
**ENSG00000004779**	NDUFAB1	731.2399818	574.5254675	587.8763844	724.2071097	641.4628663	701.8562436	402.4294467	428.6186601	415.5007143	501.792599	415.2609191	598.8442535
**ENSG00000004799**	PDK4	324.8392996	101.592918	338.7210193	112.1893095	52.01050267	232.181633	557.681416	26.58100218	524.2999984	540.3920297	63.46180159	603.9192048
**ENSG00000004838**	ZMYND10	0	10.50961221	1.628466439	11.72127114	23.11577897	7.208253313	7.149761744	14.95181373	4.220661883	14.5538837	28.97169203	5.920776517
**ENSG00000004846**	ABCB5	16.87476881	3.50320407	3.256932878	46.88508456	3.467366845	7.208253313	17.36370709	16.61312636	16.88264753	46.82553888	23.4532745	32.98718345
**ENSG00000004864**	SLC25A13	853.5820556	683.1247937	776.7784914	938.5389249	984.7321839	1014.087427	796.6877372	634.621427	858.2012496	866.9052468	778.0968716	962.5490967
**ENSG00000004866**	ST7	513.274218	514.9709984	521.1092604	446.245537	493.5218809	469.2952288	460.6489352	400.3763453	420.1903386	410.0398539	397.3260621	445.7498892
**ENSG00000004897**	CDC27	2413.09194	1989.819912	2482.597086	2663.24025	2268.813705	2516.818551	2312.437227	2061.688981	2257.116183	2152.076456	2070.786178	2419.905945

**ENSG00000004961**	HCCS	489.3682955	420.3844885	573.2201865	587.7380243	471.5618909	636.2232003	440.2210445	375.4566558	426.2868502	423.9609601	426.2977541	421.220958
**ENSG00000004975**	DVL2	556.8673707	500.9581821	544.7220238	504.014659	520.1050267	545.930343	398.3438686	556.5397331	550.0929321	466.9898337	601.5075107	488.0411501
**ENSG00000005001**	PRSS22	130.7794583	63.05767327	125.3919158	90.42123451	135.2273069	148.3382655	36.77020326	14.95181373	29.54463318	41.1305409	22.07367012	22.83728085
**ENSG00000005007**	UPF1	1535.603962	1229.624629	1726.988658	1891.310822	1707.100277	2035.762488	1732.285131	1538.375501	2022.634967	1877.450998	1730.023895	1884.498583
**ENSG00000005020**	SKAP2	545.6175249	746.182467	581.3625187	519.0848648	841.4143543	648.3634164	627.1362444	740.9454357	734.3951677	759.9658404	847.0770907	700.3432795
**ENSG00000005022**	SLC25A5	2595.901935	2347.146727	2344.177439	2971.342234	2729.973496	2818.047664	2308.351649	2377.338382	2735.926825	2446.318017	2534.33325	3075.420488
**ENSG00000005059**	MCUB	914.0499772	1082.490058	889.1426756	707.4624367	696.9407358	545.1715795	310.5039386	332.2625272	313.7358666	240.4554699	299.374151	309.5720293
**ENSG00000005075**	POLR2J	255.9339936	220.7018564	267.068496	365.0338727	318.9977497	341.0641962	237.9849266	309.0041503	273.87406	270.8287924	266.2636458	296.8846511
**ENSG00000005100**	DHX33	736.8649047	364.3332233	613.1176142	781.1389981	611.4123536	799.3573542	626.1148499	426.9573475	714.2297831	679.6030913	498.037182	767.1634716
**ENSG00000005108**	THSD7A	126.5607661	476.4357536	378.618447	69.49039319	210.3535886	277.7074434	3710.726345	1540.036814	3196.447933	3585.950389	1874.882356	3345.238732
**ENSG00000005156**	LIG3	1082.797665	763.6984874	947.7674674	1181.336684	837.9469875	1029.262697	1029.565691	873.8504466	1119.413324	1144.694592	889.8448266	1202.763458
**ENSG00000005175**	RPAP3	764.9895194	655.0991612	721.4106324	644.6699127	693.473369	668.4706493	786.4737919	824.0110675	777.5397113	767.559171	858.1139258	845.8252168
**ENSG00000005187**	ACSM3	9.843615139	0	2.442699658	9.209570182	20.80420107	7.587635066	49.02693767	26.58100218	29.54463318	31.63887762	27.59208765	28.75805737
**ENSG00000005189**	REXO5	222.184456	203.1858361	207.629471	232.7509555	220.7556891	252.2888659	244.1132938	151.1794499	206.3434698	236.6588046	216.597888	174.2399947
**ENSG00000005194**	CIAPIN1	400.7757592	297.772346	494.2395642	580.2029214	461.1597904	586.5241906	401.4080522	413.6668464	537.8999089	510.0187072	416.6405235	478.7370727
**ENSG00000005206**	SPPL2B	161.7165344	171.6569995	105.0360853	111.3520758	151.4083522	109.6413267	207.3430906	162.8086383	189.9297847	191.7315984	229.0143275	152.248539
**ENSG00000005238**	FAM214B	314.9956845	553.5062431	470.6268008	378.4296111	507.3913483	541.377762	188.957989	255.842146	228.3847041	193.629931	269.0228546	209.7646538
**ENSG00000005243**	COPZ2	0	0	0	0	0	0.758763507	4.08557814	3.322625272	4.220661883	7.593330629	9.657230676	1.691650434
**ENSG00000005249**	PRKAR2B	8.437384405	49.04485699	19.54159727	11.72127114	35.8294574	32.62683078	1093.913547	1937.090534	789.2637721	906.7702326	1549.295721	735.8679386
**ENSG00000005302**	MSL3	371.2449138	427.3908966	358.2626166	303.915816	369.8524634	303.8847844	547.4674707	546.5718573	495.2243276	511.2842623	444.2326111	546.40309
**ENSG00000005339**	CREBBP	1639.665036	1306.695118	1602.410976	1579.022669	1419.308828	1543.324972	2166.377808	2106.544423	2004.345432	2066.018709	2281.865648	1905.644213
**ENSG00000005436**	GCFC2	521.7116024	437.9005088	504.0103628	532.4806032	434.5766445	485.988026	465.7559079	310.665463	411.7490148	453.7015051	293.8557334	483.812024
**ENSG00000005448**	WDR54	293.9022234	308.2819582	223.9141353	352.4753679	362.9177298	298.9528216	173.6370709	367.1500926	223.6950798	232.8621393	347.6603043	209.7646538
**ENSG00000005469**	CROT	134.9981505	101.592918	124.5776826	133.1201508	142.1620406	158.9609546	1096.97773	636.2827396	900.8768308	1250.368444	619.4423677	1098.726957
**ENSG00000005471**	ABCB4	0	3.50320407	0	3.348934611	0	1.896908767	0	0	0	0	1.379604382	0
**ENSG00000005483**	KMT2E	1449.823887	1720.073199	1468.876728	1493.624837	1732.527633	1371.84442	1696.536322	2350.75738	1633.865111	1707.866614	2306.698527	1744.091597
**ENSG00000005486**	RHBDD2	219.3719945	164.6505913	208.4437042	243.634993	291.258815	303.8847844	226.7495867	325.6172767	336.2460634	285.3826761	354.5583263	335.7926111
**ENSG00000005700**	IBTK	2286.531174	2214.024973	2375.932534	1784.982148	1627.350839	1806.995291	2775.128951	2442.129575	2449.859742	2127.398131	2217.024242	2436.82245
**ENSG00000005801**	ZNF195	1068.735358	928.3490787	1358.14101	1078.356945	772.0670174	1240.578333	977.4745699	890.463573	902.7526805	982.7035388	812.5869812	885.579002
**ENSG00000005810**	MYCBP2	6973.498211	7770.106628	7087.085942	3945.882206	4328.429611	4334.436532	7391.832249	7030.675076	6473.088441	4632.564461	4803.782459	4545.464715
**ENSG00000005812**	FBXL3	1365.450043	1352.236771	1216.46443	1392.319565	1541.822457	1424.957865	2216.426141	2752.795038	2179.268419	2007.803174	2421.205691	2330.248472
**ENSG00000005844**	ITGAL	5.624922937	3.50320407	2.442699658	0	2.311577897	0.758763507	0	4.983937908	0	0	0	0
**ENSG00000005882**	PDK2	300.9333771	252.2306931	254.0407645	325.683891	401.058765	351.3075036	177.7226491	144.5341993	182.8953483	168.3188289	143.4788558	185.2357225
**ENSG00000005884**	ITGA3	12390.299	9122.343399	10466.1538	8734.021467	8082.432115	8360.056316	3442.099583	2550.114896	4679.776103	2947.477839	2379.817559	3826.513281
**ENSG00000005889**	ZFX	1416.074349	1383.765608	1552.742749	1511.206743	1262.121532	1418.508376	1612.781971	1721.119891	1574.306882	1530.688899	1552.05493	1632.442668
**ENSG00000005893**	LAMP2	943.5808226	1040.451609	1027.562323	1059.100571	1252.87522	1229.196881	1508.599728	1746.039581	1502.086668	1635.729973	1879.021169	1822.753342
**ENSG00000005961**	ITGA2B	14.06230734	14.01281628	24.42699658	25.95424324	36.98524634	16.31341539	5.106972674	6.645250545	1.406887294	14.5538837	24.83287888	5.074951301
**ENSG00000006007**	GDE1	746.7085198	819.7497525	756.4226609	889.979373	1165.03526	1024.330734	788.5165809	1345.663235	787.3879224	765.0280608	1218.19067	901.6496811
**ENSG00000006015**	REX1BD	116.7171509	154.1409791	91.19412058	108.0031412	92.46311586	133.1629954	52.09112128	31.56494009	56.74445421	73.40219608	57.94338406	63.43689126
**ENSG00000006016**	CRLF1	37.96822982	3.50320407	15.47043117	56.93188839	13.86946738	34.1443578	15.32091802	6.645250545	12.66198565	8.226108181	9.657230676	13.53320347
**ENSG00000006025**	OSBPL7	1.406230734	24.52242849	0.814233219	0.837233653	0	2.655672273	0	0	0.937924863	5.694997971	0	2.53747565
**ENSG00000006042**	TMEM98	7.031153671	3.50320407	14.65619795	10.88403749	13.86946738	29.59177676	0	3.322625272	0	0	0	0
**ENSG00000006047**	YBX2	78.74892111	105.0961221	70.83829009	77.02549606	83.21680428	94.46605657	41.87717593	43.19412854	22.04123428	46.82553888	34.49010956	22.83728085
**ENSG00000006062**	MAP3K14	563.8985244	535.9902228	626.1453458	514.0614629	465.7829462	598.285025	748.6821941	843.9468192	715.6366704	580.8897931	786.3744979	462.6663936
**ENSG00000006118**	TMEM132A	224.9969175	371.3396315	267.068496	236.9371238	242.7156791	317.5425275	174.6584655	159.4860131	145.3783538	217.675478	194.5242179	180.1607712
**ENSG00000006125**	AP2B1	4750.24742	4746.841515	4716.85304	4945.539187	5516.58065	5372.80439	3427.800059	3422.30403	3447.342834	3389.789348	3716.654206	3122.7867
**ENSG00000006194**	ZNF263	632.8038304	507.9645902	619.63148	502.3401917	484.2755693	586.5241906	685.3557329	584.7820479	542.5895332	547.3525828	560.1193792	607.3025056
**ENSG00000006210**	CX3CL1	1.406230734	35.0320407	11.39926507	0	0	0	20.4278907	38.21019063	43.14454369	3.796665314	0	0
**ENSG00000006282**	SPATA20	105.4673051	224.2050605	96.07951989	108.0031412	115.5788948	120.2640158	106.2250316	109.646634	103.6406974	112.6344043	133.8216251	99.80737558
**ENSG00000006283**	CACNA1G	8.437384405	14.01281628	1.628466439	0.837233653	10.40210053	4.931962793	0	0	0.468962431	0.632777552	5.518417529	0.845825217
**ENSG00000006327**	TNFRSF12A	1802.787801	1471.34571	1972.887091	2107.317104	1441.268818	2233.041	735.4040651	1013.400708	987.6348806	726.4286301	852.5955083	901.6496811
**ENSG00000006377**	DLX6	1.406230734	0	0	0	0	0.379381753	7.149761744	1.661312636	1.406887294	0.632777552	1.379604382	3.383300867
**ENSG00000006432**	MAP3K9	499.2119106	525.4806106	647.3154095	591.0869589	659.9554895	875.9924684	473.9270642	654.5571786	509.762163	479.0126072	687.0429824	545.5572648
**ENSG00000006451**	RALA	1092.64128	1485.358526	1077.230549	814.6283442	878.3996007	746.2439087	943.7685502	980.1744553	765.3466881	857.4135835	844.317882	806.9172568
**ENSG00000006453**	BAIAP2L1	1101.078665	1166.566955	1220.535596	1442.553584	1426.243562	1437.856845	1190.946028	1066.562712	1276.046776	1263.023995	1150.590055	1301.725009
**ENSG00000006459**	KDM7A	264.371378	875.8010176	488.5399317	147.3531229	321.3093276	272.3960989	189.9793835	486.7646024	212.9089439	175.9121596	244.1899757	206.3813529
**ENSG00000006468**	ETV1	49.2180757	70.06408141	26.05546302	34.32657977	67.035759	52.35468196	31.66323058	34.88756536	26.73085859	20.88165923	23.4532745	32.14135824
**ENSG00000006530**	AGK	576.554601	546.499835	558.5639885	694.0666982	478.4966246	642.6726901	739.4896433	581.4594226	630.2855079	687.1964219	580.813445	702.8807551
**ENSG00000006534**	ALDH3B1	49.2180757	59.5544692	35.82626166	51.07125282	61.25681426	38.31755708	42.89857046	49.83937908	30.95152048	29.10776741	68.98021912	24.52893129
**ENSG00000006555**	TTC22	25.31215321	42.03844885	39.89742775	10.04680383	35.8294574	22.7629052	9.192550814	9.967875817	10.78613592	8.858885733	5.518417529	8.458252168
**ENSG00000006576**	PHTF2	1281.076199	1033.445201	1151.325772	1969.173552	1478.254065	1732.257086	1189.924633	892.1248856	1191.633538	1295.928427	1145.071637	1434.519568
**ENSG00000006607**	FARP2	448.5876042	357.3268152	378.618447	487.269986	458.8482125	457.5343945	504.5689002	531.6200436	563.2238802	525.838146	500.7963908	628.4481361
**ENSG00000006625**	GGCT	293.9022234	262.7403053	243.4557326	358.3360034	328.2440613	290.606423	308.4611495	254.1808333	393.9284424	317.0215537	286.9577115	382.312998
**ENSG00000006634**	DBF4	786.0829804	381.8492437	587.0621512	838.9081202	632.2165547	689.3366458	650.6283187	495.0711656	738.6158295	729.5925179	544.943731	860.2042455
**ENSG00000006638**	TBXA2R	1.406230734	7.006408141	5.699632536	0.837233653	5.778944741	5.6907263	0	0	0	0	0	1.691650434
**ENSG00000006652**	IFRD1	323.4330689	315.2883663	333.0213868	288.8456102	217.2883223	343.7198685	376.8945834	373.7953431	315.6117164	361.94876	300.7537553	351.8632902
**ENSG00000006659**	LGALS14	0	0	0	0	0	0	1.021394535	0	2.344812157	1.898332657	0	0.845825217

**ENSG00000006695**	COX10	331.8704533	276.7531216	309.4086234	374.2434428	327.0882724	326.2683078	377.9159779	328.939902	362.5079595	417.000407	412.5017103	351.017465
**ENSG00000006704**	GTF2IRD1	544.2112941	483.4421617	460.8560022	393.4998168	290.103026	371.7941182	218.5784305	162.8086383	227.9157417	161.9910534	153.1360864	190.3106738
**ENSG00000006712**	PAF1	620.1477538	595.544692	575.6628861	687.368829	710.8102032	624.4623659	355.4452981	483.4419771	378.9216446	404.344856	435.9549848	453.3623162
**ENSG00000006715**	VPS41	1340.13789	1643.002709	1503.074523	1152.87074	1418.15304	1340.355734	1309.427794	1255.952353	1402.19767	1390.212283	1394.780031	1491.189857
**ENSG00000006740**	ARHGAP44	2.812461468	0	5.699632536	6.697869223	3.467366845	4.931962793	13.27812895	16.61312636	20.16538455	27.20943475	49.66575776	16.07067912
**ENSG00000006744**	ELAC2	686.2405983	388.8556518	718.9679328	880.7698028	664.5786453	880.9244312	777.281241	647.9119281	757.8432892	877.6624652	704.9778394	828.9087124
**ENSG00000006747**	SCIN	112.4984587	283.7595297	409.5593094	97.11910373	316.6861718	426.4250907	4042.679569	3914.052571	6160.2905	3400.546566	3712.515393	5718.624291
**ENSG00000006756**	ARSD	0	0	0	0	2.311577897	0	2.04278907	11.62918845	10.78613592	3.796665314	4.138813147	0
**ENSG00000006757**	PNPLA4	4.218692202	0	1.628466439	3.348934611	4.623155793	3.035054026	39.83438686	64.79119281	42.67558126	35.43554293	44.14734023	33.83300867
**ENSG00000006831**	ADIPOR2	804.3639799	896.820242	832.1463503	874.0719336	844.8817212	870.3017421	871.2495382	1137.999156	952.4626983	949.7991061	1110.581528	1103.801908
**ENSG00000006837**	CDKL3	22.49969175	17.51602035	15.47043117	51.07125282	23.11577897	33.38559429	59.24088302	33.22625272	36.57906965	34.80276538	41.38813147	48.21203736
**ENSG00000007047**	MARK4	172.9663803	171.6569995	184.8309408	226.8903199	216.1325333	192.7259307	106.2250316	137.8889488	121.9302322	148.7027248	166.9321303	131.9487338
**ENSG00000007062**	PROM1	0	0	0	0	3.467366845	1.896908767	0	3.322625272	1.406887294	8.226108181	4.138813147	3.383300867
**ENSG00000007080**	CCDC124	457.0249886	315.2883663	429.9151399	584.3890897	500.4566146	574.7633563	323.7820676	332.2625272	409.4042027	371.4404232	373.8727876	461.8205684
**ENSG00000007168**	PAFAH1B1	3405.890838	3646.835437	3203.193485	4122.538507	4082.246565	3981.990883	2973.279491	2955.47518	2816.588363	3055.6828	3196.543354	3278.41854
**ENSG00000007171**	NOS2	26.71838395	80.57369362	94.45105346	28.4659442	13.86946738	25.79795922	41.87717593	58.14594226	37.51699452	23.41276944	26.21248326	34.67883389
**ENSG00000007202**	KIAA0100	3133.082076	3082.819582	3288.687973	3543.172819	3794.455117	3795.33506	2854.797725	2845.828546	2982.601064	3031.637253	3163.432849	3003.525345
**ENSG00000007237**	GAS7	357.1826065	210.1922442	169.3605096	331.5445265	208.0420107	143.026921	3.064183605	1.661312636	10.78613592	15.81943881	4.138813147	14.37902869
**ENSG00000007255**	TRAPPC6A	4.218692202	24.52242849	13.02773151	15.9074394	4.623155793	12.14021611	31.66323058	14.95181373	9.379248629	7.593330629	23.4532745	14.37902869
**ENSG00000007314**	SCN4A	2.812461468	0	4.885399317	2.511700959	0	1.896908767	0	0	0	0	0	0
**ENSG00000007341**	ST7L	584.9919854	585.0350798	569.1490204	530.8061359	539.7534388	522.7880561	631.2218226	599.7338616	608.2442736	533.4314767	517.3516434	554.015517
**ENSG00000007372**	PAX6	426.0879124	367.8364274	398.1600443	467.1763783	446.134534	454.4993405	721.1045416	636.2827396	736.7399798	659.3542096	615.3035545	757.8593942
**ENSG00000007376**	RPUSD1	172.9663803	101.592918	172.6174425	204.2850113	153.7199301	259.4971193	124.6101333	132.9050109	177.7367615	135.4143962	135.2012295	128.5654329
**ENSG00000007384**	RHBDF1	227.8093789	276.7531216	244.2699658	310.6136852	255.4293576	269.3610448	161.3803365	124.5984477	168.3575129	149.9682799	150.3768777	131.9487338
**ENSG00000007392**	LUC7L	455.6187579	318.7915704	412.8162423	452.9434062	426.4861219	465.8807931	585.2590685	549.8944826	450.2039342	595.4436768	618.0627633	515.9533822
**ENSG00000007402**	CACNA2D2	0	0	0.814233219	0	3.467366845	2.27629052	2.04278907	3.322625272	0.937924863	0	1.379604382	2.53747565
**ENSG00000007516**	BAIAP3	68.90530597	73.56728548	47.22552673	53.58295378	64.7241811	46.2845739	72.51901198	66.45250545	92.385599	91.11996754	78.63744979	62.59106604
**ENSG00000007520**	TSR3	237.6529941	290.7659378	306.1516905	308.9392179	351.3598403	354.7219393	231.8565594	159.4860131	217.1296058	224.6360311	230.3939318	279.1223215
**ENSG00000007541**	PIGQ	182.8099954	98.08971397	203.5583049	162.4233287	171.0567643	182.4826233	188.957989	156.1633878	195.5573339	194.8954861	182.1077785	183.544072
**ENSG00000007545**	CRAMP1	348.7452221	420.3844885	384.3180796	302.2413487	346.7366845	379.7611351	350.3383255	453.5383497	409.4042027	334.7393252	371.1135788	375.5463962
**ENSG00000007866**	TEAD3	545.6175249	585.0350798	489.3541649	632.1114079	618.3470873	626.7386565	729.2756979	767.5264379	796.2982086	713.7730791	682.9041692	779.0050247
**ENSG00000007923**	DNAJC11	1098.266203	931.8522827	1046.289687	1207.290927	1003.224807	1188.603033	844.6932803	882.1570098	929.0145767	990.2968695	918.8165186	1069.123074
**ENSG00000007933**	FMO3	14.06230734	28.02563256	37.45472809	5.023401917	13.86946738	25.03919572	7.149761744	4.983937908	7.034436472	2.53111021	2.759208765	2.53747565
**ENSG00000007944**	MYLIP	112.4984587	154.1409791	144.9335131	107.1659076	157.187297	92.94852956	272.7123408	430.2799728	224.6330047	202.4888168	313.1701948	208.9188285
**ENSG00000007968**	E2F2	592.0231391	504.4613861	411.1877758	1118.54416	773.2228064	937.0729307	598.5371974	571.4915468	863.8287987	851.085808	651.1732684	893.1914289
**ENSG00000008018**	PSMB1	2916.522543	2785.047236	2941.824622	2671.612586	2481.478872	2651.119692	1961.077507	2187.948742	2130.027364	2054.628713	1957.658618	2230.441097
**ENSG00000008083**	JARID2	1233.264354	980.8971397	1328.014381	1182.173918	1205.487873	1202.260776	647.5641351	598.072549	649.5129676	672.0097606	687.0429824	655.514543
**ENSG00000008086**	CDKL5	91.40499772	122.6121425	109.1072514	66.97869223	60.10102531	80.80831345	31.66323058	48.17806645	48.77209287	26.5766572	37.24931832	51.59533822
**ENSG00000008128**	CDK11A	11.24984587	14.01281628	10.58503185	16.74467306	18.49262317	10.24330734	14.29952349	6.645250545	25.3239713	20.88165923	27.59208765	16.91650434
**ENSG00000008130**	NADK	555.46114	483.4421617	550.4216563	619.5529031	567.4923736	614.5984404	658.799475	584.7820479	718.450445	759.3330629	558.7397748	692.7308525
**ENSG00000008256**	CYTH3	767.8019808	465.9261414	609.8606814	1003.005916	925.7869476	990.1863761	1473.872314	1543.359439	1549.920836	1639.526638	1409.955679	1634.134319
**ENSG00000008277**	ADAM22	0	0	0	0	1.155788948	3.793817533	1.021394535	8.306563181	1.406887294	7.593330629	2.759208765	9.304077385
**ENSG00000008282**	SYPL1	1843.568492	1639.499505	1458.291696	1362.179153	1354.584647	1453.032115	1124.555383	1257.613666	1284.4881	1111.79016	1105.06311	1311.874911
**ENSG00000008283**	CYB561	694.6779827	588.5382838	605.7895153	678.1592588	842.5701433	781.5264118	657.7780805	614.6856754	655.6094792	613.7942258	598.7483019	674.1226978
**ENSG00000008294**	SPAG9	7633.020425	8001.318097	7735.215585	6447.536361	6191.561396	6806.108654	5796.413985	5992.354679	5852.651145	4531.95283	4780.329185	5348.152846
**ENSG00000008300**	CELSR3	499.2119106	532.4870187	447.0140375	491.4561542	580.206052	512.165367	922.319265	1076.530588	839.4427523	918.1602285	1059.536166	720.6430847
**ENSG00000008311**	AASS	71.71776744	56.05126513	74.90945619	58.6063557	60.10102531	45.5258104	301.3113878	438.5865359	377.9837198	244.2521352	366.9747657	378.0838719
**ENSG00000008323**	PLEKHG6	175.7788418	168.1537954	143.3050466	200.098843	176.8357091	226.111525	317.6537003	337.2464651	343.2804998	328.4115497	365.5951613	323.1052328
**ENSG00000008324**	SS18L2	130.7794583	63.05767327	117.2495836	119.7244124	136.3830959	128.9897961	126.6529223	154.5020752	155.2265648	194.2627086	131.0624163	190.3106738
**ENSG00000008382**	MPND	71.71776744	31.52883663	59.43902502	76.18826241	55.47786952	76.63511417	55.15530488	84.72694444	68.93747742	76.56608384	102.0907243	67.66601734
**ENSG00000008394**	MGST1	594.8356005	514.9709984	514.5953947	574.3422859	520.1050267	533.7901269	1640.359623	1206.112974	1330.446418	1503.479464	1094.026275	1107.185209
**ENSG00000008405**	CRY1	203.9034565	259.2371012	179.1313083	178.3307681	277.3893476	226.111525	483.119615	774.1716884	589.4857763	445.4753969	631.8588071	537.0990127
**ENSG00000008441**	NFIX	684.8343675	525.4806106	687.2128372	421.1285274	424.174544	639.6376361	508.6544784	310.665463	556.1894437	190.4660433	205.561053	245.2893129
**ENSG00000008513**	ST3GAL1	9.843615139	14.01281628	30.94086234	10.88403749	16.18104528	31.48868552	869.2067492	1649.683448	741.8985666	809.3224895	1683.117346	598.8442535
**ENSG00000008516**	MMP25	102.6548436	45.54165292	63.51019112	5.023401917	5.778944741	0.379381753	18.38510163	1.661312636	9.379248629	3.163887762	5.518417529	3.383300867
**ENSG00000008517**	IL32	5205.866178	8022.337321	9583.524993	138.9807864	194.1725433	305.4023114	283.9476807	441.9091612	578.6996404	7.593330629	35.86971394	53.28698866
**ENSG00000008710**	PKD1	613.1166001	683.1247937	605.7895153	524.9455003	725.8354595	628.2561835	643.478557	593.0886111	692.6575113	660.6197647	787.7541023	555.7071674
**ENSG00000008735**	MAPK8IP2	16.87476881	31.52883663	14.65619795	6.697869223	16.18104528	7.587635066	25.53486337	11.62918845	26.73085859	26.5766572	8.277626294	10.1499026
**ENSG00000008838**	MED24	563.8985244	500.9581821	593.576017	555.0859119	606.7891978	656.3304332	510.6972674	543.249232	573.0720912	554.3131359	565.6377967	556.5529926
**ENSG00000008853**	RHOBTB2	1240.295508	858.2849973	902.9846404	1286.828124	1301.418356	1121.073081	884.5276672	563.1849836	1114.7237	920.0585612	502.1759952	1056.435696
**ENSG00000008869**	HEATR5B	812.8013643	907.3298542	876.9291773	853.9783259	863.3743444	997.3946294	944.7899448	1216.08085	918.2284408	1015.607972	1150.590055	984.5405523
**ENSG00000008952**	SEC62	1420.293041	1411.79124	1554.371216	1378.086593	1437.801452	1429.131065	1269.593407	1260.936291	1252.129692	1236.447337	1280.272867	1328.791416
**ENSG00000008988**	RPS20	8700.349552	9171.388256	8052.76654	8949.190515	8789.774952	8657.870992	4339.905379	4977.292658	4449.51555	4347.181785	4405.076793	4621.588984
**ENSG00000009307**	CSDE1	12186.39554	11970.44831	11510.00079	11830.94875	11804.07253	11644.36415	10939.13547	12313.64926	10881.3353	11155.86825	12037.04824	12660.31184
**ENSG00000009335**	UBE3C	1690.289342	1369.752792	1621.952573	1746.4694	1533.731934	1800.925183	1183.796266	1109.756841	1280.267438	1267.453437	1131.275593	1344.016269
**ENSG00000009413**	REV3L	2150.126793	1741.092423	1818.997012	2323.323387	2081.575896	2007.688238	3200.029078	2649.793655	2794.547129	3032.902809	2750.931138	3018.750199
**ENSG00000009694**	TENM1	146.2479964	185.6698157	161.2181775	77.02549606	127.1367843	147.2001203	77.62598465	116.2918845	70.81332715	62.64497769	30.35129641	65.12854169

ENSG00000009724	MASP2	0	7.006408141	1.628466439	1.674467306	4.623155793	2.27629052	0	3.322625272	0	0.632777552	0	0.845825217
ENSG00000009765	IYD	1.406230734	3.50320407	4.885399317	4.186168264	1.155788948	7.587635066	0	0	0	0.632777552	0	0
ENSG00000009780	FAM76A	274.2149932	259.2371012	261.3688634	313.1253862	286.6356592	243.9424674	384.0443451	277.4392102	336.2460634	359.4176498	326.9662386	384.0046484
ENSG00000009790	TRAF3IP3	0	3.50320407	0	2.511700959	2.311577897	0	0	1.661312636	0.468962431	3.163887762	5.518417529	1.691650434
ENSG00000009830	POMT2	241.8716863	175.1602035	221.4714357	343.2657977	372.1640413	342.961105	544.4032871	421.9734096	457.2383707	558.1098012	481.4819294	557.3988179
ENSG00000009844	VTA1	1574.978422	1411.79124	1437.121632	1758.190671	1499.058266	1599.852854	1682.236799	1687.893638	1765.174592	1661.673853	1776.930444	1815.98674
ENSG00000009950	MLXIPL	14.06230734	14.01281628	8.956565414	12.55850479	11.55788948	15.17527013	14.29952349	3.322625272	1.875849726	5.694997971	4.138813147	3.383300867
ENSG00000009954	BAZ1B	3214.643458	3082.819582	3271.589076	3347.260144	3520.533136	3499.417292	3703.576583	3912.391258	3730.596142	3622.01871	3840.8186	4103.943952
ENSG00000010017	RANBP9	1738.101187	1944.278259	1786.427683	2036.152244	1990.268569	2121.882146	1207.28834	1265.920229	1271.357152	1232.650672	1255.439988	1364.316075
ENSG00000010030	ETV7	950.6119763	823.2529565	887.5142092	20.93084132	32.36209055	22.00414169	274.7551299	260.8260839	312.3289793	5.062220419	9.657230676	5.920776517
ENSG00000010072	SPRTN	555.46114	315.2883663	570.7774868	644.6699127	447.290323	696.9242808	539.2963144	523.3134804	577.2927531	549.2509155	474.5839075	642.8271647
ENSG00000010165	METTL13	473.8997574	451.9133251	613.9318475	519.9220984	448.4461119	645.7077441	611.8153264	598.072549	622.782109	586.5847911	538.0457091	652.9770674
ENSG00000010219	DYRK4	19.68723028	14.01281628	35.01202844	12.55850479	10.40210053	23.14228695	30.64183605	58.14594226	22.97915914	43.02887356	40.00852709	38.90795997
ENSG00000010244	ZNF207	4052.756976	3937.601375	4057.324133	3708.945082	3505.50788	3790.782479	4971.127201	4194.814406	4192.055175	4364.899556	4145.711169	4421.974233
ENSG00000010256	UQCRC1	815.6138258	837.2657728	905.42734	1127.75373	1115.336335	1297.106215	1394.20354	1496.842685	1731.409297	1630.667752	1597.581875	1746.629073
ENSG00000010270	STARD3NL	271.4025317	343.3139989	267.8827292	241.9605257	295.8819708	276.9486799	232.877954	318.9720261	227.9157417	236.6588046	246.9491844	283.3514476
ENSG00000010278	CD9	946.3932841	830.2593647	794.6916222	871.5602326	776.6901732	984.1162681	112.3533988	172.7765142	237.2949903	155.6632779	158.654504	263.0516424
ENSG00000010292	NCAPD2	1610.134191	987.9035479	1344.299045	1724.701325	1720.969744	1801.304565	3487.040942	3782.808872	3916.774227	3650.4937	4374.725496	3851.888037
ENSG00000010295	IFFO1	18.28099954	0	10.58503185	0	4.623155793	3.035054026	1.021394535	14.95181373	0.937924863	3.163887762	0	0.845825217
ENSG00000010310	GIPR	36.56199909	77.07048955	29.3123959	56.09465474	42.76419109	25.79795922	11.23533988	4.983937908	4.689624315	8.226108181	16.55525259	6.766601734
ENSG00000010318	PHF7	66.09284451	42.03844885	61.88172468	82.04889798	82.06101533	91.43100255	97.03248081	101.3400708	104.1096598	128.4538431	103.4703287	122.6446564
ENSG00000010319	SEMA3G	1.406230734	7.006408141	2.442699658	0	2.311577897	4.931962793	0	3.322625272	0.937924863	0	0	0
ENSG00000010322	NISCH	310.7769922	308.2819582	357.4483833	344.1030313	387.1892977	369.8972095	507.6330838	465.1675381	546.3412326	494.1992684	575.2950274	524.4116344
ENSG00000010327	STAB1	0	0	0	1.674467306	0	0	1.021394535	0	2.344812157	0	0	2.53747565
ENSG00000010361	FUZ	59.06169083	66.56087734	50.48245961	43.53614995	79.74943743	48.56086442	68.43343384	64.79119281	82.53738794	109.4705166	100.7111199	84.58252168
ENSG00000010404	IDS	64.68661377	189.1730198	63.51019112	70.32762684	92.46311586	85.36089449	50.04833221	86.38825708	49.2410553	48.09109398	84.15586732	32.98718345
ENSG00000010438	PRSS3	122.3420739	126.1153465	143.3050466	272.9381708	247.3388349	215.8682176	0	4.983937908	1.406887294	1.265555105	2.759208765	14.37902869
ENSG00000010539	ZNF200	710.1465207	819.7497525	579.7340522	592.7614262	465.7829462	533.7901269	591.3874357	486.7646024	538.3688713	541.0248073	384.9096227	590.3860013
ENSG00000010610	CD4	0	0	0	2.511700959	1.155788948	2.27629052	0	0	0	0	0	0
ENSG00000010626	LRRC23	23.90592248	7.006408141	22.79853014	49.39678552	57.78944741	35.66188481	85.79714093	121.2758224	77.84776362	79.09719405	108.9887462	60.05359039
ENSG00000010671	BTK	0	0	0	0	0	0	0	1.661312636	1.406887294	3.163887762	1.379604382	1.691650434
ENSG00000010704	HFE	163.1227652	231.2114686	176.6886086	133.9573845	142.1620406	120.6433976	133.8026841	127.921073	115.8337206	119.5949574	151.7564821	100.6532008
ENSG00000010803	SCMH1	306.5583	311.7851623	334.6498532	290.5200775	303.9724934	310.713656	362.5950599	320.6333388	298.2601064	425.8592928	360.0767438	321.4135824
ENSG00000010810	FYN	711.5527515	560.5126513	571.5917201	1048.216533	959.3048271	952.6275825	245.1346884	212.6480174	305.7635053	187.9349331	198.6630311	265.5891181
ENSG00000010818	HIVEP2	2877.148082	3885.053314	3121.770163	2147.50432	2718.415606	2508.472153	2347.164641	3505.369662	2432.977094	2113.477025	3184.126914	2225.366145
ENSG00000011007	ELOA	1850.599646	1467.842506	1794.570016	1857.821476	1512.927733	1776.644751	1406.460275	1216.08085	1488.017795	1449.060595	1389.261613	1669.658978
ENSG00000011009	LYPLA2	864.8319015	683.1247937	713.2683002	795.3719702	648.3976	797.4604454	476.9912478	466.8288508	691.250624	606.2008952	547.7029398	603.9192048
ENSG00000011021	CLCN6	361.4012987	301.2755501	382.6896131	323.17219	354.8272071	253.4270112	363.6164544	466.8288508	366.7286214	398.0170804	470.4450944	329.8718345
ENSG00000011028	MRC2	1.406230734	10.50961221	9.770798633	0.837233653	11.55788948	5.311344546	0	0	0.937924863	0	2.759208765	0
ENSG00000011083	SLC6A7	0	0	0	0.837233653	0	1.13814526	3.064183605	0	0.468962431	3.163887762	5.518417529	1.691650434
ENSG00000011105	TSPAN9	80.15515185	42.03844885	45.59706029	63.62975762	64.7241811	47.42271916	15.32091802	33.22625272	60.02719123	12.0227735	45.52694462	58.36193996
ENSG00000011114	BTBD7	646.8661377	819.7497525	742.5806961	704.1135021	803.273319	775.4563038	915.1695032	1048.288273	945.4282618	1082.682392	1231.986713	1053.89822
ENSG00000011132	APBA3	73.12399818	112.1025303	83.8660216	61.95529031	75.12628164	94.46605657	80.69016826	93.03350762	95.19937358	74.66775118	77.25784541	82.89087124
ENSG00000011143	MKS1	215.1533023	189.1730198	201.9298384	219.3552171	249.6504128	203.3486198	304.3755714	259.1647712	283.722271	295.507117	347.6603043	257.9766911
ENSG00000011198	ABHD5	368.4324523	539.4934268	475.5122002	467.1763783	469.250313	495.0931881	588.3232521	521.6521677	455.8314834	610.630338	591.85028	562.4737692
ENSG00000011243	AKAP8L	469.6810652	472.9325495	542.2793242	564.295482	570.9597404	620.2891667	571.9809395	699.4126198	588.5478515	541.0248073	641.5160378	518.4908579
ENSG00000011258	MBTD1	732.6462125	648.092753	600.904116	689.0432963	621.8144542	639.2582543	1011.18059	1287.517293	944.9592994	1094.072388	1045.740122	932.0993889
ENSG00000011260	UTP18	1188.26497	704.1440182	977.0798633	1239.105806	1054.079521	1150.285476	1170.518137	1104.772903	1041.56556	1075.721839	1087.128253	1276.350252
ENSG00000011275	RNF216	1109.516049	868.7946095	1164.353504	912.5846816	953.5258823	946.5574745	894.7416126	1044.965648	853.5116252	759.9658404	902.261266	752.7844429
ENSG00000011295	TTC19	645.459907	430.8941007	604.975282	724.2071097	577.8944741	646.4665076	576.0665177	481.7806645	499.9139519	589.7486788	413.8813147	614.0691074
ENSG00000011304	PTBP1	4557.593809	3384.095132	4220.170776	4603.947857	4318.027511	4843.566844	4409.360207	4103.442211	5149.67646	4719.254986	4249.181498	5063.109748
ENSG00000011332	DPF1	11.24984587	10.50961221	18.72736405	33.48934611	17.33683422	22.38352344	3.064183605	6.645250545	15.47576024	17.08499391	2.759208765	9.304077385
ENSG00000011347	SYT7	94.21745919	87.58010176	113.9926507	174.1445998	193.0167544	171.1011707	4.08557814	3.322625272	20.63434698	15.81943881	6.898021912	37.21630954
ENSG00000011376	LARS2	625.7726767	567.5190594	682.3274379	599.4592955	559.401851	592.5942987	736.4254596	692.7673693	764.8777257	682.1342015	740.8475533	729.1013369
ENSG00000011405	PIK3C2A	2137.470716	2385.681972	2217.157057	2237.925554	2150.923233	2193.964679	2269.538656	2830.876732	2423.128883	2390.633593	2505.361558	2548.471378
ENSG00000011422	PLAUR	2002.472565	2991.736276	3455.605783	1022.26229	1116.492124	1797.131365	160.358942	194.3735784	249.4880135	75.93330629	86.91507609	137.8695103
ENSG00000011426	ANLN	8156.138258	7889.215567	7560.969676	11358.74897	12808.45312	11436.84234	6897.477294	10860.0007	7398.820281	7428.175687	10868.52332	8844.794292
ENSG00000011451	WIZ	409.2131436	420.3844885	394.9031114	483.9210514	403.3703429	491.2993705	442.2638336	378.779281	483.9692293	429.0231805	419.3997322	450.8248405
ENSG00000011454	RABGAP1	2027.784719	2308.611482	1981.843656	2134.945815	2443.337837	1996.686168	2360.44277	2526.85652	2071.40706	2279.264744	2612.9707	2244.820125
ENSG00000011465	DCN	39.37446056	38.53524477	19.54159727	21.76807497	15.02525633	6.828871559	1583.161529	1473.584308	2071.876022	1306.052868	1443.066184	2003.759939
ENSG00000011478	QPCTL	44.99938349	56.05126513	46.41129351	42.6989163	40.45261319	53.11344546	50.04833221	36.54887799	62.37200338	58.84831237	60.70259282	54.13281387
ENSG00000011485	PPP5C	611.7103694	592.0414879	702.6832684	790.3485683	782.469118	756.1078343	540.3177089	528.2974183	503.6656514	536.5953644	460.7878637	592.923477
ENSG00000011523	CEP68	163.1227652	224.2050605	166.1035768	226.8903199	305.1282823	248.1156667	250.241661	204.3414542	242.4535771	261.9699067	234.532745	292.655525
ENSG00000011566	MAP4K3	542.8050634	413.3780803	557.7497553	648.856081	449.6019009	612.3221498	550.5316543	539.9266067	533.2102846	607.4664503	503.5555995	591.2318265
ENSG00000011590	ZBTB32	12.65607661	0	2.442699658	0.837233653	0	0.379381753	0	1.661312636	1.406887294	0.632777552	1.379604382	0
ENSG00000011638	TMEM159	195.466072	343.3139989	182.3882412	189.2148055	263.5198802	221.1795622	142.9952349	192.7122658	158.5093018	150.6010575	157.2748996	156.4776651
ENSG00000011677	GABRA3	89.99876699	73.56728548	129.4630819	126.4222816	125.9809954	138.8537217	101.118059	111.3079466	116.7716454	99.34607572	91.05388923	106.5739773

**ENSG00000012048**	BRCA1	2032.003411	2291.095462	2177.259629	2336.719125	2643.289325	2170.443011	2737.337353	2638.164466	2520.204107	2736.130136	2692.987754	2686.340888
**ENSG00000012061**	ERCC1	912.6437465	721.6600385	797.9485551	895.8400086	765.1322838	907.4811539	279.8621026	412.0055338	392.0525927	325.8804395	336.6234693	367.0881441
**ENSG00000012124**	CD22	91.40499772	56.05126513	65.95289078	58.6063557	87.83996007	57.28664475	199.1719343	139.5502614	76.90983876	206.2854821	144.8584601	70.20349299
**ENSG00000012171**	SEMA3B	12.65607661	56.05126513	9.770798633	10.88403749	19.64841212	9.484543833	2.04278907	4.983937908	4.689624315	8.858885733	11.03683506	0.845825217
**ENSG00000012174**	MBTPS2	1226.2332	1019.432384	1016.163058	1108.497356	1207.799451	1193.914378	973.3889917	754.2359368	944.4903369	889.0524611	754.6435971	888.9623028
**ENSG00000012211**	PRICKLE3	212.3408409	238.2178768	258.9261638	225.2158526	307.4398602	283.7775515	253.3058446	304.0202124	272.4671727	242.9865801	264.8840414	237.6768859
**ENSG00000012223**	LTF	185.6224569	245.2242849	174.245909	94.60740277	101.7094274	106.6062727	33.70601965	64.79119281	20.63434698	30.37332251	70.3598235	10.99572782
**ENSG00000012232**	EXTL3	1060.297974	875.8010176	988.4791284	1324.503639	1306.041512	1466.689858	356.4666927	328.939902	359.2252225	357.5193171	339.382678	455.0539666
**ENSG00000012504**	NR1H4	5.624922937	0	1.628466439	0	2.311577897	4.173199286	1.021394535	0	2.813774589	0.632777552	0	4.229126084
**ENSG00000012660**	ELOVL5	1373.887427	1432.810465	1319.057816	1522.090781	1400.816205	1638.170411	2835.391229	1558.311253	2048.427901	2894.957302	1782.448862	2260.890804
**ENSG00000012779**	ALOX5	2.812461468	0	0	0	0	0	48.00554314	39.87150327	19.69642212	56.94997971	66.22101035	32.98718345
**ENSG00000012822**	CALCOCO1	642.6474455	1131.534915	744.2091626	509.0380609	656.4881226	548.965397	721.1045416	1008.41677	733.9262052	795.4013833	829.1422338	579.3902735
**ENSG00000012963**	UBR7	437.3377583	539.4934268	468.1841012	564.295482	644.9302331	607.7695688	761.960323	995.126269	799.5809456	868.8035794	867.7711565	962.5490967
**ENSG00000012983**	MAP4K5	1178.421355	910.8330583	1007.206492	965.3304018	896.8922239	942.763657	1081.656812	1031.675147	1057.04132	999.1557552	1009.870408	1083.502103
**ENSG00000013016**	EHD3	327.6517611	217.1986524	365.5907155	496.4795562	377.9429861	541.377762	264.5411845	272.4552723	341.8736125	253.7437985	215.2182836	299.4221267
**ENSG00000013275**	PSMC4	1179.827586	1117.522098	1390.710339	1169.615413	1078.351089	1305.452613	685.3557329	676.1542429	823.9669921	737.1858485	767.0600366	819.6046351
**ENSG00000013288**	MAN2B2	465.462373	525.4806106	604.1610488	504.8518927	642.6186552	684.404683	721.1045416	732.6388725	803.8016075	679.6030913	790.5133111	751.0927925
**ENSG00000013297**	CLDN11	4.218692202	7.006408141	1.628466439	5.023401917	3.467366845	10.62268909	5.106972674	3.322625272	0	2.53111021	0	3.383300867
**ENSG00000013306**	SLC25A39	888.737824	749.6856711	854.1306472	1054.914403	902.6711686	1007.637937	503.5475057	380.4405937	696.4092107	568.8670196	540.8049179	672.4310473
**ENSG00000013364**	MVP	4155.411819	4056.710314	4389.531286	910.9102143	1472.47512	1172.668999	1882.430128	1802.52421	1905.863321	875.1313549	902.261266	655.514543
**ENSG00000013374**	NUB1	4056.975668	3976.13662	3893.663255	921.7942518	1170.814205	998.5327747	2797.599631	2751.133725	2686.216807	1276.312323	1180.941351	1289.03763
**ENSG00000013375**	PGM3	2030.59718	2350.649931	2349.877071	1668.60667	1923.23281	1923.086108	1458.551396	1447.003306	1420.487205	1452.224483	1440.306975	1514.872963
**ENSG00000013392**	RWDD2A	189.8411491	234.7146727	156.3327781	82.04889798	112.111528	110.779472	73.54040651	121.2758224	121.4612697	114.532737	128.3032076	142.9444616
**ENSG00000013441**	CLK1	1359.82512	1811.156504	1420.022735	1065.79844	1526.797201	1177.600962	1504.51415	1770.95927	1423.300979	1387.681172	1730.023895	1463.277625
**ENSG00000013503**	POLR3B	393.7446056	266.2435094	311.0370898	452.9434062	359.4503629	437.4271616	733.361276	518.3295425	748.933003	782.7458323	671.8673342	726.5638612
**ENSG00000013523**	ANGEL1	206.7159179	168.1537954	223.9141353	288.0083766	254.2735686	274.6723894	458.6061462	252.5195207	462.8659198	494.1992684	387.6688314	539.6364883
**ENSG00000013561**	RNF14	878.8942088	1110.51569	987.6648952	1303.572798	1225.136285	1266.755674	730.2970924	712.7031209	751.2778152	911.832453	823.6238162	948.170068
**ENSG00000013563**	DNASE1L1	267.1838395	297.772346	299.6378248	313.9626198	307.4398602	302.7466391	159.3375474	194.3735784	166.0127007	163.2566085	191.7650091	198.7689259
**ENSG00000013573**	DDX11	198.2785335	196.1794279	187.2736405	225.2158526	156.031508	176.0331335	400.3866577	412.0055338	373.2940954	414.4692968	415.2609191	356.9382415
**ENSG00000013583**	HEBP1	144.8417656	105.0961221	122.1349829	133.9573845	124.8252064	102.4330734	224.7067977	294.0523366	309.9841672	228.4326964	229.0143275	300.267952
**ENSG00000013588**	GPRC5A	6408.193456	11623.63111	9309.128398	6563.074605	10081.947	10292.62697	2772.064768	6459.183529	3121.413944	2564.64742	5780.542362	2922.326124
**ENSG00000013619**	MAMLD1	0	0	3.256932878	0	0	2.27629052	0	0	0.468962431	1.265555105	1.379604382	0.845825217
**ENSG00000013725**	CD6	1.406230734	17.51602035	12.21349829	3.348934611	3.467366845	5.6907263	0	0	0	0	0	0
**ENSG00000013810**	TACC3	902.8001313	728.6664466	970.5659976	1026.448458	1106.090023	1091.481304	1022.415929	777.4943137	1041.56556	992.8279797	1015.388825	1119.872587
**ENSG00000014123**	UFL1	939.3621304	1040.451609	946.9532342	946.9112614	995.1342845	1025.468879	1533.113197	1443.680681	1460.817974	1517.400571	1323.040603	1510.643837
**ENSG00000014138**	POLA2	203.9034565	315.2883663	212.5148703	182.5169363	128.2925733	180.5857146	185.8938053	192.7122658	172.5781748	219.5738107	191.7650091	162.3984416
**ENSG00000014164**	ZC3H3	386.7134519	294.2691419	468.9983344	375.9179101	275.0777697	355.1013211	231.8565594	320.6333388	381.7354192	278.422123	222.1163056	274.8931955
**ENSG00000014216**	CAPN1	3450.890222	3058.297153	3454.79155	3749.969531	3916.968746	4416.003608	3941.56151	3676.484864	3943.505086	4133.302972	3712.515393	3776.609593
**ENSG00000014257**	ACPP	0	0	0	0	0	0	5.106972674	9.967875817	6.096511609	8.858885733	0	5.074951301
**ENSG00000014641**	MDH1	1430.136657	1317.20473	1355.69831	1579.859903	1606.546638	1553.947662	1349.262181	1398.82524	1376.404736	1365.533958	1565.850974	1428.598791
**ENSG00000014824**	SLC30A9	836.7072868	875.8010176	761.3080602	752.6730539	846.0375101	751.5552533	974.4103863	762.5425	779.8845235	806.7913793	812.5869812	854.2834689
**ENSG00000014914**	MTMR11	541.3988327	711.1504263	468.1841012	385.1274803	414.9282324	338.0291422	690.4627056	792.4461274	634.9751322	476.4814969	554.6009617	384.0046484
**ENSG00000014919**	COX15	430.3066047	546.499835	477.1406666	515.7359302	530.5071273	470.4333741	566.8739669	707.719183	548.2170824	549.883693	629.0995983	556.5529926
**ENSG00000015133**	CCDC88C	14.06230734	7.006408141	13.02773151	7.535102876	8.090522638	14.41650663	0	0	5.158586746	3.796665314	26.21248326	7.612426951
**ENSG00000015153**	YAF2	208.1221487	227.7082646	223.0999021	234.4254228	253.1177797	280.3631157	440.2210445	403.6989706	375.6389076	397.3843029	358.6971394	416.9918319
**ENSG00000015171**	ZMYND11	968.8929758	791.7241199	956.7240329	1054.914403	1082.974245	1041.023531	687.398522	691.1060566	721.733182	697.3208627	706.3574437	797.6131794
**ENSG00000015285**	WAS	0	0	4.071166097	0.837233653	8.090522638	2.655672273	1.021394535	13.29050109	1.875849726	1.898332657	1.379604382	0.845825217
**ENSG00000015475**	BID	68.90530597	133.1217547	134.3484812	125.5850479	152.5641412	135.4392859	5.106972674	8.306563181	4.689624315	6.327775524	12.41643944	15.2248539
**ENSG00000015479**	MATR3	503.4306028	409.8748762	447.8282707	458.8040418	322.4651166	419.5962192	721.1045416	624.6535512	701.098835	725.7958526	688.4225868	648.7479413
**ENSG00000015520**	NPC1L1	0	0	0.814233219	0	0	0.379381753	1.021394535	3.322625272	1.406887294	0	6.898021912	3.383300867
**ENSG00000015532**	XYLT2	268.5900702	262.7403053	297.1951251	257.0307314	325.9324834	358.5157569	222.6640086	234.2450817	294.508407	236.026027	303.5129641	245.2893129
**ENSG00000015568**	RGPD5	0	0	0.814233219	1.674467306	1.155788948	1.13814526	1.021394535	0	0.468962431	0.632777552	0	0
**ENSG00000015676**	NUDCD3	953.4244378	896.820242	1055.246252	1016.401655	1037.898476	1170.392709	990.7526988	830.6563181	1055.165471	1114.954047	960.2046501	1046.285793
**ENSG00000016082**	ISL1	25.31215321	0	10.58503185	53.58295378	18.49262317	17.45156065	6.128367209	8.306563181	3.751699452	5.062220419	4.138813147	10.1499026
**ENSG00000016391**	CHDH	2.812461468	7.006408141	3.256932878	5.023401917	13.86946738	7.967016819	25.53486337	21.59706427	9.848211061	20.88165923	16.55525259	11.84155303
**ENSG00000016864**	GLT8D1	307.9645308	248.727489	280.0962275	395.1742842	360.6061519	353.9631758	683.3129438	716.0257462	645.7612681	723.2647424	716.0146744	716.4139586
**ENSG00000017260**	ATP2C1	2878.554313	3366.579112	3173.066856	3055.902833	3120.63016	3286.204747	1712.878635	1839.073088	1767.050442	1721.154942	1946.621783	1950.47295
**ENSG00000017483**	SLC38A5	28.12461468	21.01922442	29.3123959	20.93084132	60.10102531	41.35261111	0	11.62918845	0.468962431	1.898332657	5.518417529	0
**ENSG00000017797**	RALBP1	1434.355349	1576.441832	1328.014381	1624.233287	1558.003502	1498.178544	1110.255859	1141.321781	1163.495792	1183.294023	1220.949878	1419.294714
**ENSG00000018189**	RUFY3	469.6810652	381.8492437	487.7256984	475.5487148	449.6019009	474.9859551	489.2479822	476.7967266	476.9347928	532.7986991	463.5470725	510.8784309
**ENSG00000018280**	SLC11A1	7.031153671	10.50961221	0	3.348934611	26.58314581	1.13814526	10.21394535	13.29050109	3.28273702	6.960553076	15.17564821	5.920776517
**ENSG00000018408**	WWTR1	662.3346758	616.5639164	566.7063207	816.3028115	784.7806959	699.5799531	617.9436936	935.3190141	592.2995509	720.1008546	751.8843884	701.1891047
**ENSG00000018510**	AGPS	1154.515433	1012.425976	1073.973616	1119.381394	1107.245812	1134.351442	1244.058543	1078.191901	1101.123789	1157.350143	1040.221704	1176.542877
**ENSG00000018607**	ZNF806	0	0	0	3.348934611	0	1.896908767	0	0	0	0	1.379604382	0
**ENSG00000018610**	CXorf56	336.0891455	301.2755501	367.219182	330.7072929	361.7619408	347.1343043	357.4880872	295.7136492	365.7906965	367.0109804	384.9096227	432.2166858
**ENSG00000018699**	TTC27	725.6150588	627.0735286	829.7036506	763.5570914	684.2270574	813.7738608	579.1307013	604.7177995	685.6230748	665.6819851	601.5075107	673.2768726
**ENSG00000018869**	ZNF582	2.812461468	3.50320407	2.442699658	10.04680383	3.467366845	4.173199286	4.08557814	0	1.406887294	0	0	0

**ENSG00000019144**	PHLDB1	175.7788418	161.1473872	217.4002696	184.1914036	389.5008756	298.5734399	12.25673442	14.95181373	9.848211061	11.38999594	13.79604382	14.37902869
**ENSG00000019169**	MARCO	29.53084542	28.02563256	23.61276336	21.76807497	55.47786952	34.1443578	1.021394535	1.661312636	1.875849726	2.53111021	4.138813147	4.229126084
**ENSG00000019186**	CYP24A1	0	24.52242849	7.328098975	7.535102876	1.155788948	13.27836137	77.62598465	31.56494009	94.26144872	92.38552265	28.97169203	91.34912341
**ENSG00000019485**	PRDM11	11.24984587	7.006408141	8.956565414	5.86063557	10.40210053	13.65774312	14.29952349	16.61312636	10.78613592	11.38999594	8.277626294	6.766601734
**ENSG00000019505**	SYT13	1.406230734	14.01281628	4.885399317	0	1.155788948	1.896908767	0	0	0.937924863	0	0	2.53747565
**ENSG00000019549**	SNAI2	338.9016069	206.6890402	239.3845665	406.0583216	412.6166545	355.1013211	319.6964894	388.7471569	308.5772799	267.0321271	318.6886123	286.7347485
**ENSG00000019582**	CD74	17099.76573	19957.75359	18434.24009	7.535102876	5.778944741	14.03712487	4022.251678	3626.645485	2890.215465	12.65555105	16.55525259	5.074951301
**ENSG00000019995**	ZRANB1	876.0817474	837.2657728	810.1620533	953.6091306	946.5911486	956.0420183	974.4103863	953.5934531	905.5664551	880.1935754	914.6777055	993.8446297
**ENSG00000020129**	NCDN	376.8698368	266.2435094	414.4447087	457.1295745	434.5766445	625.6005112	314.5895167	240.8903322	331.0874766	323.3493293	309.0313816	285.8889233
**ENSG00000020181**	ADGRA2	0	10.50961221	4.071166097	5.023401917	0	6.828871559	9.192550814	1.661312636	14.06887294	3.163887762	0	5.920776517
**ENSG00000020256**	ZFP64	420.4629895	346.817203	477.9548998	397.6859851	431.1092777	436.2890163	395.279685	330.6012146	407.5283529	366.3782028	332.4846561	373.8547458
**ENSG00000020426**	MNAT1	293.9022234	343.3139989	263.8115631	311.4509189	301.6609155	263.2909368	328.8890402	357.1822168	328.7426644	299.3037823	321.4478211	336.6384363
**ENSG00000020577**	SAMD4A	462.6499115	332.8043867	491.7968645	313.9626198	242.7156791	361.9301927	20.4278907	26.58100218	46.89624315	27.20943475	45.52694462	30.4497078
**ENSG00000020633**	RUNX3	0	0	0	0.837233653	2.311577897	4.173199286	0	0	0	0	0	0
**ENSG00000020922**	MRE11	1799.97534	1432.810465	1527.50152	1805.075756	1788.005503	1723.531305	2057.088593	1800.862898	1735.160996	2069.815374	1935.584948	2006.297414
**ENSG00000021300**	PLEKHB1	15.46853808	10.50961221	28.49816268	13.39573845	35.8294574	22.7629052	0	0	0	0	1.379604382	0.845825217
**ENSG00000021355**	SERPINB1	1144.671818	2277.082646	1470.505194	740.9517828	849.504877	794.8047732	1203.202762	1888.912467	950.5868486	1163.677919	1677.598929	1009.915309
**ENSG00000021488**	SLC7A9	0	7.006408141	1.628466439	0.837233653	4.623155793	0	0	0	0	0	1.379604382	1.691650434
**ENSG00000021574**	SPAST	537.1801404	441.4037129	477.1406666	708.2996703	646.0860221	588.0417176	649.6069242	556.5397331	653.264667	709.9764138	674.6265429	887.2706524
**ENSG00000021645**	NRXN3	98.43615139	73.56728548	48.85399317	119.7244124	120.2020506	88.77533027	556.6600215	543.249232	357.3493728	583.4209033	713.2554657	465.2038692
**ENSG00000021762**	OSBPL5	108.2797665	157.6441832	111.5499511	92.93293547	101.7094274	114.9526713	8.171156279	4.983937908	7.972361335	16.45221636	9.657230676	10.99572782
**ENSG00000021776**	AQR	1119.359664	1092.99967	1089.444048	1212.314329	988.1995508	1174.94529	1233.844598	1048.288273	1138.171821	1138.999594	1223.709087	1156.243071
**ENSG00000021826**	CPS1	75.93645964	49.04485699	83.05178838	62.79252397	80.90522638	97.12172885	1066.335894	915.3832625	917.2905159	1149.124035	1294.068911	1283.116854
**ENSG00000022267**	FHL1	1001.236283	886.3106298	904.6131068	723.3698761	642.6186552	792.1491009	370.7662162	416.9894717	738.6158295	368.2765355	386.289227	701.1891047
**ENSG00000022277**	RTF2	1005.454975	1036.948405	1025.119623	1022.26229	1018.250063	1070.615308	1097.999125	1245.984477	1121.289174	1114.32127	1178.182142	1260.279573
**ENSG00000022556**	NLRP2	12.65607661	10.50961221	10.58503185	1.674467306	5.778944741	6.828871559	13.27812895	14.95181373	12.19302322	6.327775524	12.41643944	5.074951301
**ENSG00000022567**	SLC45A4	23.90592248	31.52883663	30.12662912	38.51274803	27.73893476	58.42479001	13.27812895	6.645250545	12.19302322	6.960553076	6.898021912	11.84155303
**ENSG00000022840**	RNF10	1480.760963	1635.996301	1481.090226	1659.3971	1683.984498	1734.912758	2013.168628	2262.70781	2035.296953	2127.398131	2044.573695	2084.113334
**ENSG00000022976**	ZNF839	143.4355349	175.1602035	153.0758453	127.2595152	116.7346838	117.2289618	271.6909463	182.74439	248.0811262	235.3932495	208.3202617	217.3770807
**ENSG00000023041**	ZDHHC6	1141.859356	1247.140649	1205.065165	1144.498403	1161.567893	1187.464888	927.4262377	867.2051961	1022.807063	946.6352184	892.6040354	1113.95181
**ENSG00000023171**	GRAMD1B	831.0823639	1485.358526	1345.927512	1111.846291	1111.868968	1465.551713	91.92550814	64.79119281	105.5165471	93.65107775	77.25784541	114.1864043
**ENSG00000023191**	RNH1	776.2393653	669.1119775	765.3792263	774.4411289	757.0417611	818.3264419	725.1901198	772.5103758	923.3870275	774.5197241	756.0232015	856.8209446
**ENSG00000023228**	NDUFS1	1822.475031	1692.047566	1685.462764	1982.56929	1864.287574	1896.150003	1875.280366	1631.409009	1721.561086	1973.000408	1701.052203	1980.076832
**ENSG00000023287**	RB1CC1	3687.136985	4592.700536	3674.634519	3862.996074	4623.155793	4130.329148	2944.680444	3016.943747	2675.430671	3012.021149	3203.441376	3211.598348
**ENSG00000023318**	ERP44	1807.006493	2101.922442	2023.36955	1164.592011	1287.548888	1462.137277	1759.862784	1842.395713	1685.450979	1561.694999	1534.120073	1633.288494
**ENSG00000023330**	ALAS1	722.8025974	896.820242	771.893092	687.368829	703.8754695	806.1862258	315.6109113	581.4594226	344.6873871	342.3326558	558.7397748	412.7627058
**ENSG00000023445**	BIRC3	5241.021946	11616.6247	10184.42911	3027.436889	4809.237814	6161.539055	2969.193913	3754.566558	2568.507237	1581.943881	1848.669872	1715.33354
**ENSG00000023516**	AKAP11	1663.570959	1706.060382	1682.205831	1468.507827	1539.510879	1631.720921	1397.267724	1149.628344	1420.487205	1443.365597	1103.683506	1559.7017
**ENSG00000023572**	GLRX2	369.8386831	290.7659378	303.7089909	354.9870688	342.1135287	384.3137161	331.9532238	227.5998311	345.1563495	340.4343232	335.2438649	408.5335797
**ENSG00000023608**	SNAPC1	123.7483046	98.08971397	93.63682024	108.8403749	82.06101533	82.70522222	77.62598465	225.9385185	96.60626088	119.5949574	224.8755143	137.8695103
**ENSG00000023697**	DERA	191.2473798	224.2050605	182.3882412	239.4488247	334.023006	256.841447	321.7392785	455.1996623	337.1839882	432.8198458	390.4280402	405.9961041
**ENSG00000023734**	STRAP	1019.517282	973.8907316	1037.333122	1235.756872	1175.43736	1203.398921	1136.812117	1086.498464	1173.812966	1252.899554	1060.91577	1409.144811
**ENSG00000023839**	ABCC2	1192.483663	651.5959571	1117.94221	2016.89587	1553.380346	1512.59505	109.2892152	78.0816939	65.18577797	168.3188289	108.9887462	95.5782495
**ENSG00000023892**	DEF6	80.15515185	52.54806106	83.8660216	54.42018744	87.83996007	93.70729307	92.94690267	96.3561329	117.2406079	119.5949574	84.15586732	93.88659906
**ENSG00000023902**	PLEKHO1	14.06230734	31.52883663	12.21349829	15.07020575	11.55788948	10.62268909	32.68462512	38.21019063	33.29633263	43.02887356	56.56377967	56.67028952
**ENSG00000023909**	GCLM	793.1141341	721.6600385	929.8543366	498.9912571	575.5828962	613.4602951	707.8264127	526.6361057	762.5329135	692.2586423	560.1193792	772.2384229
**ENSG00000024048**	UBR2	2435.591632	2266.573034	2269.267983	1311.945134	1510.616155	1419.267139	2121.436449	1933.767908	1920.870119	1524.993901	1440.306975	1481.88578
**ENSG00000024422**	EHD2	680.6156753	472.9325495	468.1841012	740.1145491	797.4943743	707.5469699	868.1853546	1657.990011	1038.282823	911.1996754	1582.406227	906.7246324
**ENSG00000024526**	DEPDC1	1333.106736	662.1055693	1122.013376	1757.353437	1531.420356	1714.046761	1666.915881	1219.403475	1843.022356	1817.969908	1481.695107	2286.265561
**ENSG00000024862**	CCDC28A	109.6859973	101.592918	140.0481137	115.5382441	169.9009754	141.8887757	132.7812895	167.7925762	159.9161891	176.5449371	131.0624163	160.7067912
**ENSG00000025039**	RRAGD	0	3.50320407	0	0.837233653	12.71367843	3.035054026	4.08557814	4.983937908	3.28273702	8.226108181	6.898021912	4.229126084
**ENSG00000025156**	HSF2	431.7128354	262.7403053	344.4206518	396.0115178	407.9934987	385.4518614	389.1513178	350.5369662	378.4526822	317.6543313	307.6517773	416.9918319
**ENSG00000025293**	PHF20	1549.666269	1523.893771	1338.599413	1570.650333	1547.601402	1531.564138	1508.599728	1593.198818	1470.197223	1408.562832	1576.887809	1489.498207
**ENSG00000025423**	HSD17B6	25.31215321	17.51602035	9.770798633	6.697869223	11.55788948	6.828871559	15.32091802	3.322625272	14.06887294	8.858885733	22.07367012	16.91650434
**ENSG00000025434**	NR1H3	97.02992066	84.07689769	123.7634494	74.51379511	94.77469376	85.74027625	144.0166294	86.38825708	149.5990156	141.1093942	139.3400426	98.11572515
**ENSG00000025708**	TYMP	233.4343019	231.2114686	295.5666587	6.697869223	31.2063016	18.21032416	49.02693767	24.91968954	46.89624315	6.327775524	4.138813147	2.53747565
**ENSG00000025770**	NCAPH2	374.0573753	399.365264	353.3772172	319.8232554	410.3050766	399.868368	302.3327823	377.1179684	385.9560811	341.6998783	373.8727876	317.1844563
**ENSG00000025772**	TOMM34	1030.767128	833.7625688	1066.645517	1249.15261	1232.071019	1372.982565	1980.484003	1852.363589	2086.88282	2050.19927	1959.038223	2056.201102
**ENSG00000025796**	SEC63	3708.230446	3261.48299	3621.70936	3292.002723	2704.546139	2973.973564	2401.298551	2581.679837	2516.452407	2543.132983	2603.313469	2552.700504
**ENSG00000025800**	KPNA6	3644.950063	3201.92852	3784.556004	3914.904561	3804.857218	4111.739442	3170.408636	2877.393486	3544.887019	3206.283858	2905.446829	3422.208827
**ENSG00000026036**	RTEL1-TNFF	12.65607661	24.52242849	14.65619795	15.9074394	20.80420107	23.90105046	8.171156279	14.95181373	17.8205724	12.65555105	20.69406573	10.99572782
**ENSG00000026103**	FAS	558.2736015	634.0799367	521.9234937	103.816973	132.9157291	102.8124551	165.4659146	237.567707	218.0675306	68.33997566	96.57230676	73.58679386
**ENSG00000026297**	RNASET2	66.09284451	73.56728548	52.11092604	41.86168264	55.47786952	28.4536315	133.8026841	124.5984477	106.9234344	115.7982921	139.3400426	85.42834689
**ENSG00000026508**	CD44	12661.70153	12054.52521	13668.53305	9568.743419	11557.88948	12213.81617	1504.51415	1151.289657	3614.293459	1530.056122	1196.116999	3468.729214
**ENSG00000026559**	KCNG1	624.366446	364.3332233	600.0898827	512.3869956	453.0692677	635.4644368	239.0063212	119.6145098	194.1504466	226.5343638	165.5525259	211.4563042
**ENSG00000026652**	AGPAT4	1801.38157	2045.871177	2128.405636	1997.639496	1977.55489	2138.574943	73.54040651	68.11381808	97.54418574	74.03497363	53.80457091	51.59533822
**ENSG00000026751**	SLAMF7	1.406230734	10.50961221	8.142332194	2.511700959	0	3.035054026	160.358942	483.4419771	45.02039342	133.5160636	426.2977541	46.52038692

**ENSG00000026950**	BTN3A1	2269.656405	2406.701196	2458.984323	332.3817602	396.4356093	349.7899765	996.881066	897.1088235	873.2080474	275.2582353	213.8386793	225.8353329
**ENSG00000027001**	MIPEP	212.3408409	241.7210809	220.6572025	335.7306948	331.7114282	257.6002105	351.35972	375.4566558	327.3357772	415.1020744	406.9832928	344.2508632
**ENSG00000027075**	PRKCH	175.7788418	178.6634076	179.1313083	267.0775353	238.0925233	206.004292	347.2741419	309.0041503	347.0321993	423.9609601	442.8530067	399.2295023
**ENSG00000027697**	IFNGR1	684.8343675	868.7946095	624.5168793	910.0729807	777.8459622	852.8501814	521.9326073	586.4433606	482.562342	589.7486788	620.821972	559.0904683
**ENSG00000027847**	B4GALT7	185.6224569	196.1794279	165.2893435	146.5158893	154.8757191	201.0723293	187.9365944	171.1152015	216.6606433	186.669378	115.8867681	195.3856251
**ENSG00000027869**	SH2D2A	30.93707615	3.50320407	17.09889761	14.2329721	18.49262317	18.58970591	2.04278907	1.661312636	1.875849726	1.898332657	0	1.691650434
**ENSG00000028116**	VRK2	791.7079033	763.6984874	672.5566393	735.0911472	655.3323337	603.9757513	773.1956629	704.3965577	709.0711964	639.1053279	652.5528728	685.1184256
**ENSG00000028137**	TNFRSF1B	372.6511446	406.3716722	393.274645	128.9339825	110.955739	120.2640158	67.4120393	46.51675381	82.0684255	22.14721433	13.79604382	39.75378519
**ENSG00000028203**	VEZT	1499.041963	1422.300853	1752.229888	1212.314329	1221.668918	1392.331035	1550.476904	1443.680681	1281.674325	1466.145589	1357.530712	1300.879183
**ENSG00000028277**	POU2F2	269.996301	276.7531216	306.9659237	228.5647872	250.8062018	255.7033017	18.38510163	13.29050109	8.910286198	9.491663286	6.898021912	4.229126084
**ENSG00000028310**	BRD9	1174.202663	1029.941997	1174.124302	1214.82603	852.9722438	1125.24628	1066.335894	893.7861982	892.9044695	997.8902001	997.4539684	960.011621
**ENSG00000028528**	SNX1	985.7677446	1036.948405	1090.258281	990.4474113	1004.380596	964.3884169	878.3993	812.3818791	784.1051854	794.1358282	899.5020573	828.9087124
**ENSG00000028839**	TBPL1	258.7464551	276.7531216	242.6414994	209.3084132	223.067267	240.1486498	232.877954	186.0670152	255.5845251	220.2065882	223.4959099	252.0559146
**ENSG00000029153**	ARNTL2	1051.860589	1471.34571	1336.156713	431.1753312	485.4313583	546.3097248	355.4452981	327.2785893	291.2256699	125.2899554	132.4420207	149.7110634
**ENSG00000029363**	BCLAF1	4350.877891	4540.152475	3991.371242	4213.796975	3789.831961	3923.945474	4396.082078	4238.008535	3896.139881	4211.767389	4187.0993	4495.561027
**ENSG00000029364**	SLC39A9	1203.733508	1138.541323	1209.136331	1392.319565	1275.990999	1332.768099	1736.370709	1621.441133	1845.367168	1874.28711	1776.930444	1890.419359
**ENSG00000029534**	ANK1	367.0262216	259.2371012	289.8670261	640.4837444	758.1975501	623.7036024	877.3779055	764.2038126	733.4572428	1024.466857	1059.536166	865.2791968
**ENSG00000029639**	TFB1M	272.8087624	301.2755501	334.6498532	304.7530496	315.5303829	320.9569633	224.7067977	242.5516449	217.1296058	227.7999189	266.2636458	292.655525
**ENSG00000029725**	RABEP1	1162.952817	1121.025303	1150.511539	1730.56196	1599.611904	1432.166119	2117.350871	1847.379651	2208.34409	2403.289144	1941.103366	2604.295842
**ENSG00000029993**	HMGB3	1091.23505	907.3298542	1002.321093	1278.455788	1204.332084	1088.44625	2157.185258	2074.979483	1856.622266	2171.059782	2326.012989	2101.875664
**ENSG00000030066**	NUP160	2670.432164	2466.255666	2683.712691	2607.982829	2224.893725	2561.585598	2983.493436	2684.68122	2870.988005	2881.668974	2679.19171	3017.058548
**ENSG00000030110**	BAK1	375.463606	343.3139989	364.7764823	186.7031046	154.8757191	226.8702885	106.2250316	112.9692593	134.5922178	77.83163894	103.4703287	93.88659906
**ENSG00000030304**	MUSK	476.7122189	819.7497525	633.4734447	313.9626198	577.8944741	464.7426478	3.064183605	26.58100218	4.689624315	0.632777552	9.657230676	7.612426951
**ENSG00000030419**	IKZF2	1254.357815	1671.028342	1126.898776	1389.807864	1316.443612	1073.27098	2105.094136	1701.184139	1593.06538	2025.520945	1855.567894	1645.130047
**ENSG00000030582**	GRN	1248.732892	1551.919403	1671.6208	925.9804201	1242.473119	1534.599192	1128.640961	1242.661852	1380.625398	1063.066288	1346.493877	1053.052395
**ENSG00000031003**	FAM13B	1127.797049	802.2337321	1039.775821	1087.566515	887.6459123	1020.157535	1220.566469	1154.612282	1002.172716	1145.32737	1084.369044	1046.285793
**ENSG00000031081**	ARHGAP31	710.1465207	1215.611812	1120.38491	796.2092039	985.8879729	1062.648291	364.637849	533.2813562	496.1622525	349.2932089	409.7425015	489.7328005
**ENSG00000031691**	CENPQ	528.742756	599.047896	538.2081581	532.4806032	499.3008257	468.9158471	261.4770009	382.1019063	318.425491	304.3660027	351.7991175	311.2636798
**ENSG00000031698**	SARS	1999.660104	1874.214178	2178.073862	1744.794933	1578.807703	1963.300573	2373.720899	1975.300724	2487.845699	2014.763727	1596.20227	1972.464406
**ENSG00000031823**	RANBP3	736.8649047	690.1312019	819.932852	774.4411289	700.4081026	820.2233506	631.2218226	526.6361057	671.5542018	706.812526	638.756829	598.8442535
**ENSG00000032219**	ARID4A	275.6212239	420.3844885	311.851323	202.610544	354.8272071	253.0476295	684.3343384	770.8490632	574.947941	609.3647829	718.7738832	654.6687178
**ENSG00000032389**	EIPR1	527.3365253	472.9325495	478.769133	425.3146957	345.5808955	448.0498507	447.3708063	337.2464651	539.3067962	423.9609601	426.2977541	434.7541614
**ENSG00000032444**	PNPLA6	513.274218	420.3844885	518.6665608	568.4816503	744.3280827	763.3160876	686.3771274	862.2212581	900.4078684	924.488004	1041.601309	990.4613288
**ENSG00000032742**	IFT88	374.0573753	308.2819582	260.5546302	430.3380976	443.8229561	306.5404567	442.2638336	407.0215959	399.0870292	454.3342826	434.5753804	403.4586284
**ENSG00000033011**	ALG1	192.6536106	210.1922442	189.7163401	219.3552171	165.2778196	206.3836738	138.9096567	74.75906863	152.8817527	153.1321677	146.2380645	135.3320347
**ENSG00000033030**	ZCCHC8	892.9565162	816.2465484	919.2693048	835.5591856	668.0460121	788.7346651	1375.818438	1373.90555	1308.405184	1143.429037	1109.201923	1293.266756
**ENSG00000033050**	ABCF2	257.3402244	119.1089384	262.1830967	313.1253862	234.6251565	331.9590341	222.6640086	207.6640795	242.9225395	231.5965842	209.6998661	241.906012
**ENSG00000033100**	CHPF2	444.368912	458.9197332	512.152695	473.0370139	481.9639914	536.0664174	517.8470292	458.5222876	443.6384602	459.396503	624.9607852	451.6706658
**ENSG00000033122**	LRRC7	16.87476881	10.50961221	20.35583049	17.58190671	34.67366845	17.83094241	64.3478557	53.16200436	55.80652934	29.74054496	34.49010956	28.75805737
**ENSG00000033170**	FUT8	208.1221487	175.1602035	249.9695984	208.4711796	258.8967244	240.9074133	614.87951	445.2317865	469.4313939	578.9914604	429.0569629	422.0667832
**ENSG00000033178**	UBA6	5671.328551	8498.773075	5512.358896	3317.119733	5382.509132	3457.6853	2437.04736	2877.393486	2501.914572	1806.579912	2037.675673	1994.455861
**ENSG00000033327**	GAB2	33.74953762	56.05126513	42.34012741	32.65211246	53.16629162	64.11551631	331.9532238	313.9880882	268.2465108	373.9715335	241.4307669	304.497078
**ENSG00000033627**	ATP6V0A1	1053.26682	910.8330583	989.2933616	1196.40689	1225.136285	1217.056665	685.3557329	742.6067483	694.533361	754.90362	779.476476	848.3626924
**ENSG00000033800**	PIAS1	1243.107969	1128.031711	1169.238903	1249.989844	1094.532134	1141.939077	1374.797044	2216.191057	1220.709209	1348.448964	1827.975807	1226.446564
**ENSG00000033867**	SLC4A7	1085.610127	711.1504263	918.4550715	1095.938852	818.2985754	1006.12041	580.1520958	689.444744	725.9538439	622.020334	678.7653561	702.0349299
**ENSG00000034152**	MAP2K3	628.5851382	655.0991612	757.2368941	678.1592588	499.3008257	801.254263	131.759895	159.4860131	212.9089439	187.3021555	155.8952952	212.3021294
**ENSG00000034510**	TMSB10	9742.366526	10313.43278	9648.66365	5409.366631	5469.193303	5367.493046	1792.547409	2638.164466	1956.980226	852.9841406	1429.27014	969.3156984
**ENSG00000034533**	ASTE1	194.0598413	101.592918	237.7561001	198.4243757	199.951488	196.1403665	230.8351649	292.391024	236.3570655	232.2293617	213.8386793	205.5355277
**ENSG00000034677**	RNF19A	923.8935923	1015.92918	888.3284424	698.2528665	775.5343843	710.5820239	929.4690267	1199.467723	1106.282376	614.4270034	866.3915521	804.3797812
**ENSG00000034693**	PEX3	326.2455303	241.7210809	280.9104607	343.2657977	335.178795	305.0229297	473.9270642	576.4754847	360.6321098	385.3615294	408.3628972	392.4629006
**ENSG00000034713**	GABARAPL2	881.7066703	973.8907316	874.4864777	1003.005916	1097.999501	1036.091568	726.2115143	747.5906863	725.9538439	761.2313955	865.0119477	851.7459933
**ENSG00000035115**	SH3YL1	1012.486129	868.7946095	958.3524993	828.0240827	958.1490381	847.1594551	1417.695614	1275.888105	1341.701516	1289.600652	1249.92157	1242.517243
**ENSG00000035141**	FAM136A	696.0842134	578.0286716	859.8302797	1028.960159	717.7449369	997.0152477	499.4619276	473.4741013	495.2243276	580.8897931	491.1391601	564.1654196
**ENSG00000035403**	VCL	2186.688792	1800.646892	2291.25228	2588.726455	2327.758942	3085.132418	2410.491102	2869.086923	2564.286575	2548.195203	2984.084279	2690.570015
**ENSG00000035499**	DEPDC1B	532.9614482	339.8107948	523.5519601	679.8337261	627.5933989	589.1798629	605.6869592	455.1996623	694.0643986	669.4786504	553.2213573	803.5339559
**ENSG00000035664**	DAPK2	1.406230734	3.50320407	4.885399317	5.86063557	5.778944741	4.173199286	13.27812895	26.58100218	11.25509835	10.12444084	52.42496653	12.68737825
**ENSG00000035681**	NSMAF	679.2094446	493.9517739	711.6398338	615.3667349	587.1407857	729.5511116	653.6925023	491.7485403	606.3684239	632.7775524	549.0825442	550.6322161
**ENSG00000035687**	ADSS	1134.828202	753.1888751	929.8543366	1236.594105	935.0332591	1059.613237	925.3834486	652.895866	988.1038431	1027.630745	721.5330919	1052.20657
**ENSG00000035862**	TIMP2	4920.401339	5342.386207	6126.290743	6038.129104	7846.65117	8024.303464	1240.99436	1147.967032	1809.726023	1261.125662	1280.272867	1897.185961
**ENSG00000035928**	RFC1	2057.315564	2508.294114	2150.389933	1907.218261	1952.127534	1953.436648	1908.986386	2148.077239	1801.753662	1866.69378	1855.567894	2004.605764
**ENSG00000036054**	TBC1D23	735.458674	1001.916364	947.7674674	723.3698761	850.6606659	855.1264719	1156.218613	1058.256149	1025.151875	947.2679959	1096.785484	1079.272977
**ENSG00000036257**	CUL3	1655.133574	1607.970668	1502.26029	1777.447045	1878.157041	1808.133436	1347.219392	1450.325931	1294.336311	1339.590078	1494.111546	1567.314127
**ENSG00000036530**	CYP46A1	0	0	0	0	0	1.896908767	7.149761744	26.58100218	5.627549177	10.75721839	20.69406573	6.766601734
**ENSG00000036549**	AC118549.1	2026.378488	1471.34571	1749.787189	2135.783048	1762.578146	1899.943821	2322.651172	2104.88311	1923.214931	2253.953642	2085.961826	2083.267509
**ENSG00000036672**	USP2	49.2180757	66.56087734	70.83829009	81.21166433	49.69892478	103.5712187	3.064183605	0	18.28953483	10.12444084	11.03683506	18.60815477
**ENSG00000036828**	CASR	18.28099954	10.50961221	52.92515926	8.372336529	12.71367843	40.21446585	0	0	1.406887294	1.265555105	0	0
**ENSG00000037042**	TUBG2	158.904073	182.1666117	127.8346155	179.1680017	138.6946738	152.8908466	160.358942	220.9545806	147.2542035	201.8560392	182.1077785	144.6361121

**ENSG00000037241**	RPL26L1	440.1502198	357.3268152	368.8476484	493.1306215	403.3703429	430.59829	336.038802	400.3763453	349.3770114	339.8015456	362.8359525	447.4415397
**ENSG00000037474**	NSUN2	4407.127121	3534.732907	4195.74378	4430.640491	3556.362594	4363.648927	3500.319071	3126.590381	3615.700347	3714.404232	3215.857815	3690.335421
**ENSG00000037637**	FBXO42	641.2412148	861.7882013	641.6157769	656.3911838	906.1385354	667.3325041	702.71944	1113.079466	677.181751	675.8064259	987.7967377	622.5273595
**ENSG00000037749**	MFAP3	1593.259422	1460.836097	1353.255611	1645.164128	1612.325583	1501.972361	1386.032384	1432.051492	1359.991051	1412.992274	1492.731942	1556.318399
**ENSG00000037757**	MRI1	337.4953762	322.2947745	363.9622491	410.2444899	493.5218809	387.7281519	326.8462512	357.1822168	391.1146678	331.5754374	404.224084	381.4671728
**ENSG00000037897**	METTL1	130.7794583	105.0961221	126.206149	155.7254594	88.99574902	129.3691779	100.0966644	54.82331699	109.737209	141.1093942	41.38813147	121.7988312
**ENSG00000037965**	HOXC8	23.90592248	10.50961221	13.02773151	15.07020575	17.33683422	20.86599643	4.08557814	4.983937908	3.751699452	3.796665314	6.898021912	5.920776517
**ENSG00000038002**	AGA	109.6859973	133.1217547	131.9057816	87.90953355	88.99574902	97.5011106	131.759895	149.5181373	135.0611803	125.9227329	128.3032076	121.7988312
**ENSG00000038210**	PI4K2B	741.0835969	739.1760589	559.3782218	505.6891263	567.4923736	445.3941784	1004.030828	918.7058878	928.0766518	784.0113874	790.5133111	916.0287098
**ENSG00000038219**	BOD1L1	1555.291192	1937.271851	1646.37957	1525.439716	1527.95299	1483.762037	2126.543422	2081.624733	1850.056792	1966.039855	2481.908284	2038.438772
**ENSG00000038274**	MAT2B	1989.816489	1758.608443	1615.438707	1111.846291	1078.351089	992.8420484	1925.328698	1702.845452	2003.87647	1524.993901	1172.663725	1456.511023
**ENSG00000038295**	TLL1	199.6847643	129.6185506	166.1035768	390.1508822	255.4293576	254.1857747	0	1.661312636	0.468962431	5.062220419	1.379604382	0
**ENSG00000038358**	EDC4	420.4629895	413.3780803	405.4881433	405.221088	440.3555893	500.7839144	363.6164544	468.4901634	418.7834513	465.091501	452.5102374	425.450084
**ENSG00000038382**	TRIO	5242.428177	5377.418248	5740.344197	6735.544737	6623.826463	7475.717449	3153.044929	4434.043426	3004.173336	3572.029283	4875.521887	3466.191738
**ENSG00000038427**	VCAN	0	0	2.442699658	3.348934611	3.467366845	3.035054026	231.8565594	44.85544118	572.1341664	233.4949168	92.43349361	668.2019213
**ENSG00000038532**	CLEC16A	1957.473182	2052.877585	2415.829962	2101.456469	2094.289574	2527.061859	2504.4594	2428.839074	2392.646325	2466.566899	2404.650438	2330.248472
**ENSG00000038945**	MSR1	22.49969175	7.006408141	33.383562	29.30317785	34.67366845	24.28043221	16.34231256	21.59706427	28.13774589	17.08499391	33.11050518	10.99572782
**ENSG00000039068**	CDH1	5613.673091	6007.994981	5783.498558	5529.928277	5150.195553	5726.767566	3498.276282	4419.091612	4081.849003	3077.197237	4096.045411	3613.365326
**ENSG00000039123**	MTREX	1968.723028	1898.736606	1887.392603	2494.956286	2160.169544	2209.519331	2010.104445	1769.297957	1948.06994	2088.7987	1837.633037	2212.678767
**ENSG00000039139**	DNAH5	348.7452221	269.7467134	298.8235915	619.5529031	601.0102531	577.7984103	1.021394535	3.322625272	0.937924863	1.898332657	9.657230676	0
**ENSG00000039319**	ZFYVE16	1174.202663	1271.663078	1279.160388	1337.062144	1255.186798	1245.130914	1790.50462	1724.442516	1557.424235	1662.939408	1888.678399	1707.721113
**ENSG00000039523**	RIPOR1	409.2131436	455.4165292	400.602744	522.4337994	542.0650167	508.3715494	406.5150249	468.4901634	400.9628789	458.1309479	467.6858856	355.246591
**ENSG00000039537**	C6	7.031153671	14.01281628	7.328098975	1.674467306	2.311577897	12.51959786	6.128367209	13.29050109	3.28273702	0	1.379604382	3.383300867
**ENSG00000039560**	RAI14	1037.798282	721.6600385	613.9318475	949.4229624	745.4838716	669.6087946	2553.486337	3028.572936	2745.306074	2749.418465	3271.04199	2975.613113
**ENSG00000039600**	SOX30	0	0	0	0	0	0.758763507	2.04278907	4.983937908	0.937924863	0.632777552	6.898021912	0
**ENSG00000039650**	PNKP	364.2137601	318.7915704	320.8078885	366.70834	285.4798702	303.5054026	280.8834971	318.9720261	259.805187	306.8971129	353.1787219	244.4434876
**ENSG00000040199**	PHLPP2	371.2449138	416.8812844	369.6618816	475.5487148	426.4861219	482.1942085	532.1465527	626.3148638	533.2102846	503.0581541	622.2015764	552.3238666
**ENSG00000040275**	SPDL1	847.9571327	518.4742024	837.0317496	1368.877022	1050.612154	1146.491658	868.1853546	706.0578704	851.1668131	906.7702326	813.9665856	1003.994532
**ENSG00000040341**	STAU2	696.0842134	728.6664466	508.0815289	961.9814671	1013.626908	761.7985606	335.0174074	287.407086	268.2465108	334.1065477	434.5753804	389.9254249
**ENSG00000040487**	PQLC2	229.2156097	189.1730198	219.028736	193.4009738	224.223056	247.3569032	159.3375474	161.1473257	239.17084	211.3477025	252.467602	242.7518372
**ENSG00000040531**	CTNS	194.0598413	129.6185506	204.3725381	215.1690488	187.2378096	252.2888659	133.8026841	132.9050109	177.7367615	158.1943881	146.2380645	156.4776651
**ENSG00000040608**	RTN4R	112.4984587	161.1473872	96.07951989	105.4914403	137.5388848	153.2702283	51.06972674	99.67875817	62.37200338	77.19886139	84.15586732	74.43261908
**ENSG00000040633**	PHF23	409.2131436	476.4357536	525.1804265	453.7806399	463.4713683	460.1900668	289.0546534	242.5516449	357.8183352	263.2354618	306.2721729	343.405038
**ENSG00000040731**	CDH10	40.78069129	31.52883663	20.35583049	72.00209415	43.91998003	25.79795922	1237.930176	2387.306258	714.6987455	1187.090688	2400.511625	703.7265804
**ENSG00000040933**	INPP4A	563.8985244	476.4357536	643.2442434	662.2518194	587.1407857	682.1283924	855.9286202	862.2212581	861.4839866	685.9308668	782.2356848	790.8465777
**ENSG00000041353**	RAB27B	8762.223705	5997.485369	6574.119014	5558.394221	3747.06777	4266.147816	2968.172518	2390.628883	3086.710724	1643.956081	1507.90759	1767.774703
**ENSG00000041357**	PSMA4	3719.480292	3734.415539	3559.827635	1713.817287	1733.683422	1756.158136	1374.797044	1566.617816	1376.873699	924.488004	880.1875959	1009.069484
**ENSG00000041515**	MYO16	63.28038304	35.0320407	38.26896131	30.97764516	48.54313583	40.97322936	168.5300983	74.75906863	121.4612697	106.3066288	110.3683506	129.4112582
**ENSG00000041802**	LSG1	908.4250543	581.5318757	908.6842729	951.0974297	772.0670174	1017.881244	952.961101	827.3336928	878.8355965	927.0191142	867.7711565	922.7953115
**ENSG00000041880**	PARP3	358.5888372	413.3780803	326.507521	51.07125282	83.21680428	49.31962793	242.0705048	225.9385185	210.5641317	112.6344043	100.7111199	66.82019213
**ENSG00000041982**	TNC	5851.326085	6435.385877	4272.281702	6421.582117	7811.977501	5176.284642	20.4278907	64.79119281	339.0598379	38.5994307	102.0907243	351.017465
**ENSG00000041988**	THAP3	104.0610743	56.05126513	152.261612	115.5382441	84.37259322	155.1671371	88.86132453	112.9692593	127.5577814	89.22163489	91.05388923	103.1906764
**ENSG00000042062**	RIPOR3	0	0	1.628466439	1.674467306	1.155788948	0.758763507	0	0	1.406887294	0	5.518417529	0
**ENSG00000042088**	TDP1	310.7769922	213.6954483	276.0250614	360.8477044	394.1240314	344.8580138	523.9753964	443.5704738	524.7689608	560.0081339	491.1391601	593.7693022
**ENSG00000042286**	AIFM2	493.5869877	585.0350798	649.7581091	542.5274071	536.286072	582.7303731	287.0118643	295.7136492	339.5288004	296.7726721	311.7905904	303.6512528
**ENSG00000042317**	SPATA7	53.4367679	143.6313669	61.88172468	66.97869223	78.59364848	55.01035423	198.1505398	210.9867048	167.419588	186.669378	189.0058004	179.314946
**ENSG00000042429**	MED17	313.5894537	224.2050605	265.4400295	256.1934978	246.183046	251.9094842	304.3755714	318.9720261	226.039892	317.0215537	361.4563482	302.8054276
**ENSG00000042445**	RETSAT	348.7452221	357.3268152	310.2228566	297.2179468	340.9577397	303.1260209	316.6323058	259.1647712	358.2872976	242.9865801	304.8925685	281.6597972
**ENSG00000042493**	CAPG	551.2424478	609.5575083	506.4530625	445.4083033	602.166042	514.8210392	400.3866577	604.7177995	409.4042027	322.0837742	321.4478211	362.0131928
**ENSG00000042753**	AP2S1	541.3988327	472.9325495	504.0103628	628.7624733	598.6986752	687.439737	385.0657396	368.8114052	491.4726282	484.0748276	440.093798	510.8784309
**ENSG00000042781**	USH2A	464.0561423	175.1602035	177.5028418	946.0740277	636.8397105	427.1838542	1.021394535	0	0.468962431	0	0	1.691650434
**ENSG00000042832**	TG	0	0	1.628466439	0.837233653	0	0	3.064183605	8.306563181	2.813774589	6.960553076	0	0
**ENSG00000042980**	ADAM28	1.406230734	24.52242849	8.956565414	24.27977593	21.95999002	6.449489806	11.23533988	43.19412854	4.689624315	27.8422123	84.15586732	5.074951301
**ENSG00000043039**	BARX2	285.464839	203.1858361	238.5703333	347.4519659	330.5556392	261.7734098	12.25673442	8.306563181	2.813774589	3.163887762	0	9.304077385
**ENSG00000043093**	DCUN1D1	1030.767128	1310.198322	984.4079623	1169.615413	1103.778446	1092.240068	985.6457262	1114.740779	1029.372537	1075.089061	957.4454413	1136.789091
**ENSG00000043143**	JADE2	1700.132958	2010.839136	1739.202157	1119.381394	1243.628908	1198.84634	1971.291452	1465.277745	2174.578795	1547.773893	1136.794011	1655.279949
**ENSG00000043355**	ZIC2	246.0903785	213.6954483	157.1470114	201.7733103	177.991498	177.9300423	30.64183605	24.91968954	50.17898017	29.10776741	41.38813147	30.4497078
**ENSG00000043462**	LCP2	0	0	1.628466439	0.837233653	4.623155793	0.758763507	3.064183605	0	0.468962431	0	0	0
**ENSG00000043514**	TRIT1	554.0549093	472.9325495	481.2118327	627.9252397	521.2608157	434.3921075	1087.78518	1084.837151	849.7599258	1013.709639	1237.505131	828.9087124
**ENSG00000043591**	ADRB1	29.53084542	35.0320407	39.08319453	41.02444899	21.95999002	52.35468196	46.9841486	13.29050109	24.38604644	34.80276538	8.277626294	32.98718345
**ENSG00000044090**	CUL7	254.5277629	301.2755501	281.7246939	200.098843	280.8567144	239.7692681	416.7289702	478.4580392	365.3217341	361.3159824	384.9096227	276.5848459
**ENSG00000044115**	CTNNA1	7953.641032	8250.045586	7963.200886	7589.523063	7560.015511	7551.973181	6998.595353	8444.452129	7355.206775	7018.135833	8421.10515	7245.338807
**ENSG00000044446**	PHKA2	458.4312193	210.1922442	339.5352525	394.3370505	433.4208556	397.2126957	814.0514443	784.1395643	812.2429313	836.5319242	837.4198601	779.0050247
**ENSG00000044459**	CNTLN	479.5246803	392.3588559	357.4483833	423.6402284	448.4461119	285.2950785	122.5673442	152.8407625	131.7784432	161.9910534	133.8216251	148.8652382
**ENSG00000044524**	EPHA3	2.812461468	10.50961221	4.071166097	1.674467306	16.18104528	5.6907263	0	0	0.468962431	0	0	0
**ENSG00000044574**	HSPA5	41330.52751	35757.20395	39985.36494	14381.16246	11436.53164	16181.76992	11487.62433	11594.30089	11342.32537	8153.97154	7103.582965	9451.250972
**ENSG00000046604**	DSG2	6863.812213	8509.282687	7478.732121	7317.422126	9286.764199	8467.800734	5008.918799	5160.037048	5137.952399	5333.049211	5959.890932	5968.14273

**ENSG00000046647**	GEMIN8	126.5607661	122.6121425	121.3207497	149.8648239	165.2778196	126.3341239	170.5728873	167.7925762	187.5849726	174.6466045	168.3117346	198.7689259
**ENSG00000046651**	OFD1	1043.423205	910.8330583	1182.266635	1014.727187	1102.622657	985.2544133	1395.224935	1523.423687	1137.233896	1133.937374	1507.90759	1168.084624
**ENSG00000046774**	MAGEC2	319.2143767	308.2819582	357.4483833	352.4753679	314.3745939	390.3838242	686.3771274	519.9908551	694.533361	690.9930872	662.2101035	707.1098812
**ENSG00000047056**	WDR37	509.0555258	458.9197332	607.4179817	436.1987331	342.1135287	438.1859251	421.8359429	515.0069172	506.9483884	403.0793009	402.8444796	401.766978
**ENSG00000047188**	YTHDC2	1153.109202	893.317038	1104.914479	1191.383488	1118.803702	1077.444179	1411.567247	1292.501231	1309.812071	1481.965028	1176.802538	1344.862095
**ENSG00000047230**	CTPS2	466.8686037	469.4293454	446.1998043	498.1540235	587.1407857	479.9179179	648.5855296	519.9908551	547.2791575	574.5620176	664.9693123	619.1440587
**ENSG00000047249**	ATP6V1H	907.0188235	798.7305281	914.3839054	820.4889798	811.3638417	899.5141371	464.7345134	390.4084695	479.2796049	477.747052	503.5555995	497.3452275
**ENSG00000047315**	POLR2B	2120.595947	1730.582811	2156.089565	2061.269253	1827.302327	1996.686168	1756.7986	1651.34476	1828.015558	1856.569339	1724.505478	2018.138967
**ENSG00000047346**	FAM214A	358.5888372	599.047896	569.9632536	331.5445265	472.7176798	427.9426177	674.120393	589.7659858	508.8242381	577.0931278	623.5811808	563.3195944
**ENSG00000047365**	ARAP2	2103.721178	2336.637115	2203.315092	1508.695042	1672.426608	1701.906545	1180.732082	1113.079466	1076.26878	830.2041487	797.411333	802.6881307
**ENSG00000047410**	TPR	6571.316221	7083.47863	6587.146745	6932.294646	6648.09803	5891.039865	6591.058934	5919.256923	5966.140053	6634.039859	7064.954042	6925.616875
**ENSG00000047457**	CP	0	3.50320407	0	0	0	0.379381753	1.021394535	4.983937908	0	0.632777552	6.898021912	0.845825217
**ENSG00000047578**	KIAA0556	1025.142205	1026.438793	1092.70098	1030.634627	1148.854215	1104.759666	1361.518915	1578.247004	1514.279691	1455.38837	1684.496951	1364.316075
**ENSG00000047579**	DTNBP1	354.370145	378.3460396	496.6822639	298.8924141	339.8019508	361.1714291	304.3755714	310.665463	305.2945429	311.9593333	270.4024589	301.9596024
**ENSG00000047597**	XK	144.8417656	192.6762239	245.8984323	236.9371238	248.4946239	323.6126356	127.6743169	109.646634	90.50974927	159.4599432	75.87824103	113.340579
**ENSG00000047617**	ANO2	14.06230734	7.006408141	21.98429693	11.72127114	13.86946738	10.24330734	84.77574639	38.21019063	81.13050064	41.1305409	38.6289227	40.59961041
**ENSG00000047621**	C12orf4	343.1202991	423.8876925	326.507521	362.5221717	359.4503629	327.4064531	365.6592435	385.4245316	397.2111794	404.344856	354.5583263	435.5999866
**ENSG00000047634**	SCML1	213.7470716	164.6505913	210.0721706	150.7020575	136.3830959	149.4764108	292.118837	224.2772059	290.2877451	282.8515659	212.4590749	235.9852355
**ENSG00000047644**	WWC3	1619.977806	1618.480281	1492.489491	1901.357626	2156.702177	1942.434577	4225.509191	4219.734096	3691.67226	3835.897523	4336.096574	3545.699309
**ENSG00000047648**	ARHGAP6	1.406230734	3.50320407	0	0	0	0.379381753	30.64183605	6.645250545	13.59991051	20.88165923	20.69406573	33.83300867
**ENSG00000047662**	FAM184B	7.031153671	3.50320407	1.628466439	0	1.155788948	3.41443578	3.064183605	4.983937908	1.406887294	0	4.138813147	0
**ENSG00000047849**	MAP4	3256.83038	4028.684681	3310.67227	3253.489975	3610.684674	3435.301776	3406.350774	4312.767603	3671.037913	3325.878815	3689.062118	3510.17465
**ENSG00000047932**	GOPC	1582.009576	1057.967629	1260.433024	1610.000314	1170.814205	1509.939378	1413.610036	1403.809178	1587.43783	1325.668972	1180.941351	1575.772379
**ENSG00000047936**	ROS1	8.437384405	10.50961221	0	5.023401917	3.467366845	3.035054026	3.064183605	6.645250545	6.096511609	1.898332657	1.379604382	7.612426951
**ENSG00000048028**	USP28	891.5502855	1029.941997	818.3043855	807.930475	1025.184797	809.9800433	950.918312	1194.483785	870.8632352	828.3058161	983.6579246	973.5448245
**ENSG00000048052**	HDAC9	5217.116024	8095.904607	6606.688343	1630.093922	2224.893725	2678.81456	21.44928523	29.90362745	26.73085859	15.18666126	15.17564821	10.1499026
**ENSG00000048140**	TSPAN17	729.833751	609.5575083	862.2729794	605.319931	728.1470374	793.2872462	389.1513178	402.0376579	386.8940059	346.7620987	264.8840414	323.951058
**ENSG00000048162**	NOP16	646.8661377	325.7979785	625.3311125	765.2315587	432.2650667	706.4088247	443.2852281	294.0523366	531.8033973	544.188695	233.1531406	596.3067778
**ENSG00000048342**	CC2D2A	126.5607661	105.0961221	177.5028418	208.4711796	159.4988749	213.5919271	182.8296217	181.0830773	198.8400709	225.2688086	248.3287888	240.2143616
**ENSG00000048392**	RRM2B	403.5882207	514.9709984	456.7848361	336.5679285	381.4103529	358.1363751	339.1029856	245.8742701	323.1151153	291.7104516	213.8386793	329.8718345
**ENSG00000048405**	ZNF800	1064.516666	1219.115017	1061.760118	1000.494215	975.4858723	1034.19466	1015.266168	941.9642647	1007.331303	952.3302163	1037.462495	1075.043851
**ENSG00000048471**	SNX29	642.6474455	697.13761	799.5770215	759.3709232	709.6544142	677.1964296	2026.446757	2920.587614	2733.582013	2090.697033	2797.837687	2636.437201
**ENSG00000048544**	MRPS10	1099.672434	851.2785891	1100.843313	1086.729281	972.0185055	1166.598891	853.8858312	755.8972494	843.6634142	886.5213509	757.4028059	957.4741454
**ENSG00000048649**	RSF1	1871.693107	2872.627338	1994.871388	1811.773625	2252.63266	1727.704505	1339.048235	1536.714188	1284.4881	1337.691746	1538.258886	1306.79996
**ENSG00000048707**	VPS13D	3654.793678	3562.75854	3447.463451	3573.31323	3426.914232	3252.060389	3920.112225	3821.019063	3789.216446	4024.465233	3857.373853	3910.249977
**ENSG00000048740**	CELF2	7.031153671	0	2.442699658	5.86063557	4.623155793	11.00207085	0	3.322625272	1.875849726	1.898332657	0	0
**ENSG00000048828**	FAM120A	843.7384405	595.544692	867.1583787	1041.518664	947.7469376	1105.518429	1768.03394	1795.87896	1924.152856	1731.279383	1939.723762	1824.444993
**ENSG00000048991**	R3HDM1	611.7103694	479.9389576	544.7220238	820.4889798	740.8607158	780.7676483	681.2701548	536.6039815	567.4445421	689.7275321	580.813445	722.3347351
**ENSG00000049089**	COL9A2	36.56199909	24.52242849	61.88172468	31.81487881	56.63365846	54.63097248	10.21394535	8.306563181	3.751699452	13.92110615	8.277626294	10.1499026
**ENSG00000049130**	KITLG	859.2069786	395.86206	390.8319453	963.6559345	805.5848969	658.6067237	578.1093067	297.3749619	617.6235222	614.4270034	431.8161717	656.3603682
**ENSG00000049167**	ERCC8	369.8386831	234.7146727	368.0334152	368.3828073	384.8777198	374.4497905	518.8684237	466.8288508	435.6660988	499.2614888	389.0484358	436.4458119
**ENSG00000049192**	ADAMTS6	64.68661377	126.1153465	173.4316757	68.65315954	72.81470374	145.6825933	12.25673442	21.59706427	19.69642212	4.429442867	31.73090079	14.37902869
**ENSG00000049239**	H6PD	596.2418313	802.2337321	758.8653605	446.245537	642.6186552	531.1344546	1570.904795	1618.118508	1598.692929	1501.581132	1645.868028	1368.545201
**ENSG00000049245**	VAMP3	2176.845176	2371.669156	2160.974964	1815.122559	1855.041262	1684.454985	1821.146456	1759.330082	1954.166452	1429.444491	1543.777304	1620.601115
**ENSG00000049246**	PER3	1497.635732	2042.367973	1742.45909	1490.275902	2094.289574	1626.409576	1523.920646	1094.805027	1839.739619	1124.445711	980.8987158	1264.508699
**ENSG00000049247**	UTS2	1.406230734	0	0	0.837233653	2.311577897	1.517527013	0	4.983937908	0.468962431	1.898332657	1.379604382	0
**ENSG00000049249**	TNFRSF9	4.218692202	42.03844885	17.09889761	0.837233653	25.42735686	12.89897961	0	33.22625272	8.441323766	1.265555105	20.69406573	7.612426951
**ENSG00000049283**	EPN3	115.3109202	112.1025303	122.1349829	338.2423958	335.178795	240.5280316	243.0918993	119.6145098	188.5228974	253.7437985	219.3570968	238.5227111
**ENSG00000049323**	LTBP1	1513.10427	879.3042217	1221.349829	1829.355532	1240.161541	1718.599342	3360.38802	2066.672919	3581.935051	2845.600653	2226.681473	3330.859704
**ENSG00000049449**	RCN1	1458.261271	1348.733567	1493.303724	892.491074	1095.687923	1085.790578	1960.056112	1775.943208	1979.021461	1382.618952	1164.386099	1556.318399
**ENSG00000049540**	ELN	0	0	0	0	1.155788948	0	1.021394535	0	0.937924863	3.796665314	0	0.845825217
**ENSG00000049541**	RFC2	739.6773662	749.6856711	709.1971341	850.6293913	858.7511886	743.9676182	560.7455996	599.7338616	542.1205708	547.9853604	593.2298844	673.2768726
**ENSG00000049618**	ARID1B	2722.462701	2515.300523	2700.811589	2675.798755	2729.973496	2680.332087	4040.63678	3485.433911	3739.037466	3676.437579	3494.5379	3434.896205
**ENSG00000049656**	CLPTM1L	2157.157946	2045.871177	2342.548972	2458.118005	2511.529385	2942.484879	1890.601284	1744.378268	2274.467793	2229.275317	1832.11462	2381.84381
**ENSG00000049759**	NEDD4L	2141.689408	2728.995971	2662.542628	2944.550757	2717.259817	3340.456338	666.9706313	847.2694444	790.6706594	756.8019526	779.476476	919.4120106
**ENSG00000049769**	PPP1R3F	32.34330689	14.01281628	35.82626166	52.74572013	33.5178795	56.14849949	32.68462512	39.87150327	29.07567075	31.63887762	11.03683506	27.91223215
**ENSG00000049860**	HEXB	2486.215938	2539.822951	2365.347502	2488.258416	2549.67042	2462.946342	2224.597297	2342.450817	2364.039617	2315.965842	2225.301869	2795.452341
**ENSG00000049883**	PTCD2	757.9583657	697.13761	702.6832684	783.6506991	655.3323337	680.6108654	713.9547799	691.1060566	662.6439156	591.0142339	608.4055326	613.2232822
**ENSG00000050130**	JKAMP	448.5876042	486.9453658	522.7377269	422.8029947	507.3913483	420.3549827	407.5364194	466.8288508	433.3212867	429.0231805	423.5385454	444.904064
**ENSG00000050165**	DKK 3.00	700.3029056	364.3332233	404.6739101	626.2507723	612.5681426	473.4684281	91.92550814	58.14594226	166.4816632	79.09719405	96.57230676	164.9359173
**ENSG00000050327**	ARHGEF5	584.9919854	595.544692	594.3902502	451.2689389	483.1197804	468.9158471	446.3494117	794.1074401	505.5415011	430.9215132	533.906896	465.2038692
**ENSG00000050344**	NFE2L3	847.9571327	956.3747112	918.4550715	415.2678918	513.170293	529.2375459	306.4183605	177.7604521	235.4191406	226.5343638	51.04536215	175.0858199
**ENSG00000050393**	MCUR1	794.5203648	826.7561606	736.0668304	889.979373	1200.864717	894.5821743	563.8097833	599.7338616	566.5066172	563.8047992	630.4792027	658.0520187
**ENSG00000050405**	LIMA1	16192.7469	9812.474601	13449.50432	17103.00906	12729.85948	14745.43061	16710.01459	14514.8885	15372.11954	14729.79586	13082.78836	15553.03409
**ENSG00000050426**	LETMD1	1385.137273	1173.573364	1296.259285	1275.944087	1218.201551	1047.473021	2269.538656	1501.826623	2107.048205	2055.26149	1517.564821	1755.087325
**ENSG00000050438**	SLC4A8	63.28038304	21.01922442	43.15436063	36.83828073	33.5178795	32.24744903	85.79714093	59.8072549	83.94427523	73.40219608	51.04536215	70.20349299
**ENSG00000050628**	PTGER3	2.812461468	0	1.628466439	3.348934611	0	0	165.4659146	259.1647712	185.2401604	202.4888168	246.9491844	222.452032

**ENSG00000050730**	TNIP3	0	0	0	0	0	0	12.25673442	11.62918845	19.69642212	15.81943881	5.518417529	49.90368779
**ENSG00000050748**	MAPK9	1026.548436	942.3618949	988.4791284	1255.850479	1086.441611	1100.586466	1092.892152	941.9642647	1084.241142	1104.196829	1164.386099	1160.472197
**ENSG00000050820**	BCAR1	2768.868316	1954.787871	2591.704338	2084.711796	1917.453865	2442.83911	1066.335894	1550.00469	1367.49445	990.929647	1390.641217	1014.144435
**ENSG00000051009**	FAM160A2	279.8399161	308.2819582	271.9538953	254.5190305	292.4146039	284.9156967	468.8200915	383.7632189	402.8387286	489.7698255	478.7227207	394.154551
**ENSG00000051108**	HERPUD1	1560.916115	1968.800688	2182.145028	583.551856	783.6249069	945.0399475	1291.042692	1762.652707	1388.128797	1140.265149	1360.289921	1286.500155
**ENSG00000051128**	HOMER3	123.7483046	136.6249587	154.7043117	189.2148055	154.8757191	221.9383257	84.77574639	78.0816939	69.87540229	69.60553076	80.01705417	71.89514343
**ENSG00000051180**	RAD51	393.7446056	542.9966309	383.5038464	349.9636669	465.7829462	429.8395265	367.7020326	533.2813562	450.6728966	436.6165111	510.4536215	530.3324109
**ENSG00000051341**	POLQ	1881.536722	1579.945036	1829.582044	2018.570337	1733.683422	2026.277944	1983.548187	1928.783971	2127.213589	2102.087029	2103.896683	2249.049251
**ENSG00000051382**	PIK3CB	2923.553696	2939.188215	2688.598091	2711.799802	2428.31258	2573.346433	1833.40319	2116.512298	1877.725576	1936.932088	1967.315849	2002.068288
**ENSG00000051523**	CYBA	1.406230734	14.01281628	3.256932878	2.511700959	15.02525633	10.24330734	3.064183605	0	1.875849726	1.265555105	0	0
**ENSG00000051596**	THOC3	299.5271464	238.2178768	250.7838316	401.8721534	376.7871971	405.938476	355.4452981	435.2639107	349.8459739	332.208215	411.1221059	415.3001814
**ENSG00000051620**	HEBP2	1756.382187	1635.996301	1652.079202	1193.057955	1470.163542	1293.312397	1224.652047	1144.644406	1196.323163	1070.026841	1055.397352	1241.671418
**ENSG00000051825**	MPHOSPH9	845.1446712	511.4677943	678.2562718	880.7698028	772.0670174	788.7346651	1304.320821	1154.612282	1336.073967	1508.541685	1266.476823	1514.027138
**ENSG00000052126**	PLEKHA5	42.18692202	77.07048955	56.18209214	55.25742109	46.23155793	54.63097248	116.438977	159.4860131	92.385599	127.188288	176.5893609	71.89514343
**ENSG00000052344**	PRSS8	1704.35165	1996.82632	1778.285351	1319.480237	1913.986498	1759.572572	1492.257415	2834.199357	1746.885057	1369.963401	2828.188984	1380.386754
**ENSG00000052723**	SIKE1	1189.671201	1478.352118	1222.978296	1158.731376	1273.679421	1165.840128	1330.877079	1227.710038	1328.101606	1203.542905	1222.329483	1357.549473
**ENSG00000052749**	RRP12	898.5814391	578.0286716	746.6518622	760.2081568	668.0460121	937.0729307	609.7725373	287.407086	729.2365809	685.2980892	413.8813147	719.7972595
**ENSG00000052795**	FNIP2	140.6230734	227.7082646	181.5740079	197.5871421	286.6356592	232.181633	176.7012545	166.1312636	143.502504	203.7543719	237.2919538	181.8524216
**ENSG00000052802**	MSMO1	1129.20328	1156.057343	924.1547041	837.2336529	963.9279829	831.984185	310.5039386	578.1367974	324.5220026	289.812119	704.9778394	362.859018
**ENSG00000052841**	TTC17	1359.82512	1541.409791	1373.611441	1269.246218	1319.910979	1279.275272	2107.136925	2069.995545	1791.905451	1990.71818	2232.199891	1881.115282
**ENSG00000053108**	FSTL4	11.24984587	3.50320407	2.442699658	3.348934611	8.090522638	6.449489806	65.36925023	66.45250545	115.8337206	58.21553482	70.3598235	94.73242428
**ENSG00000053254**	FOXN3	12.65607661	3.50320407	1.628466439	17.58190671	10.40210053	5.6907263	921.2978705	1312.436983	999.8279039	953.5957714	1284.41168	1024.294338
**ENSG00000053371**	AKR7A2	310.7769922	392.3588559	309.4086234	369.2200409	433.4208556	415.4230199	416.7289702	470.151476	412.6869397	425.2265152	449.7510286	468.5871701
**ENSG00000053372**	MRTO4	1005.454975	588.5382838	964.0521318	1203.104759	962.7721939	1263.72062	665.9492367	448.5544118	728.298656	824.5091508	484.2411382	927.0244376
**ENSG00000053438**	NNAT	8.437384405	0	2.442699658	3.348934611	0	0.758763507	1.021394535	4.983937908	2.344812157	3.163887762	0	0.845825217
**ENSG00000053501**	USE1	126.5607661	147.134571	135.9769476	180.0052354	143.3178296	119.5052523	96.01108628	91.37219499	94.26144872	105.6738512	100.7111199	81.19922081
**ENSG00000053524**	MCF2L2	28.12461468	24.52242849	9.770798633	10.04680383	6.93473369	5.311344546	16.34231256	0	4.689624315	8.858885733	6.898021912	5.920776517
**ENSG00000053747**	LAMA3	14335.1161	26536.77083	30261.79183	15827.90221	21887.17531	27666.41436	602.6227756	1536.714188	1355.770389	331.5754374	949.167815	987.078028
**ENSG00000053770**	AP5M1	530.1489868	381.8492437	512.9669283	565.1327157	517.7934488	520.8911473	823.2439951	970.2065795	888.2148452	910.5668979	834.6606513	1008.223658
**ENSG00000053900**	ANAPC4	569.5234473	557.0094472	466.5556347	430.3380976	405.6819208	432.8745805	552.5744434	498.3937908	528.0516978	477.1142745	475.9635119	565.85707
**ENSG00000054116**	TRAPPC3	932.3309767	872.2978135	1051.98932	960.3069998	877.2438117	1063.407055	701.6980455	824.0110675	794.8913213	767.559171	830.5218381	872.8916237
**ENSG00000054118**	THRAP3	5360.551559	4774.867148	5130.483516	4845.071149	4639.336838	4318.123116	5039.560635	4440.688676	4771.69274	4797.086625	4707.210152	5025.047613
**ENSG00000054148**	PHPT1	201.090995	234.7146727	188.0878737	181.6797027	245.027257	204.486765	208.3644851	295.7136492	245.2673516	222.7376984	289.7169203	226.6811581
**ENSG00000054179**	ENTPD2	75.93645964	140.1281628	30.94086234	74.51379511	113.2673169	54.25159072	61.28367209	106.3240087	49.71001773	34.80276538	142.0992514	51.59533822
**ENSG00000054219**	LY75	9.843615139	31.52883663	3.256932878	0	2.311577897	7.587635066	0	0	0.937924863	0	2.759208765	0
**ENSG00000054267**	ARID4B	1407.636965	1758.608443	1544.600417	1311.945134	1384.63516	1246.26906	1517.792279	1679.587075	1390.942572	1304.787313	1626.553567	1413.373937
**ENSG00000054277**	OPN3	527.3365253	500.9581821	531.6942923	666.4379877	678.4481126	635.085055	680.2487602	563.1849836	717.0435577	708.0780811	641.5160378	882.1957011
**ENSG00000054282**	SDCCAG8	351.5576835	423.8876925	445.385571	305.5902833	361.7619408	287.1919873	548.4888652	564.8462963	438.4798734	494.832046	531.1476872	422.9126084
**ENSG00000054392**	HHAT	165.9352266	238.2178768	174.245909	132.2829172	188.3935986	141.509394	172.6156764	157.8247004	92.385599	154.3977228	161.4137127	89.65747298
**ENSG00000054523**	KIF1B	1763.413341	2448.739645	1752.229888	1961.638449	2146.300077	1899.185057	1786.419041	1957.026285	1681.699279	1848.343231	1979.732289	1925.944019
**ENSG00000054598**	FOXC1	1437.16781	1247.140649	1395.595738	903.3751114	784.7806959	979.9430688	1422.802587	805.7366285	1125.040873	1098.501831	601.5075107	949.0158932
**ENSG00000054611**	TBC1D22A	291.089762	427.3908966	292.3097258	269.5892362	329.3998503	297.0559128	265.5625791	337.2464651	293.1015197	289.1793414	366.9747657	284.1972728
**ENSG00000054654**	SYNE2	9798.615756	10110.24695	8461.511616	8388.243968	8072.030015	6605.036325	17132.87193	14190.93254	14149.53448	14631.08257	14360.30202	13368.26755
**ENSG00000054690**	PLEKHH1	232.0280711	465.9261414	194.6017394	203.4477776	211.5093775	191.5877854	32.68462512	38.21019063	44.55143099	25.94387965	31.73090079	34.67883389
**ENSG00000054793**	ATP9A	1442.792733	1839.182137	1685.462764	1134.4516	1437.801452	1169.254564	233.8993485	295.7136492	282.3153837	239.8226924	267.6432502	244.4434876
**ENSG00000054965**	FAM168A	648.2723684	819.7497525	586.247918	814.6283442	861.0627665	836.536766	655.7352914	845.6081318	699.2229853	756.8019526	869.1507609	782.3883255
**ENSG00000054967**	RELT	281.2461468	385.3524477	300.452058	261.2168997	432.2650667	364.2064832	91.92550814	184.4057026	124.7440068	117.0638472	193.1446135	102.3448512
**ENSG00000054983**	GALC	240.4654555	283.7595297	236.1276336	238.6115911	279.7009255	278.4662069	591.3874357	578.1367974	592.7685134	649.2297687	557.3601705	587.0027004
**ENSG00000055044**	NOP58	1811.225186	1268.159873	1650.450736	1699.584315	1220.513129	1587.333256	1484.086259	955.2547658	1340.294629	1383.884507	1069.193396	1376.157628
**ENSG00000055070**	SZRD1	2238.719329	2164.980116	2281.481481	2174.295796	2242.23056	2281.981246	1944.735194	2060.027669	2122.992927	2018.560392	2090.100639	2003.759939
**ENSG00000055118**	KCNH2	15.46853808	10.50961221	8.142332194	5.023401917	5.778944741	11.00207085	0	1.661312636	0	0.632777552	0	0.845825217
**ENSG00000055130**	CUL1	1705.757881	1618.480281	1831.210511	1830.192765	1709.411854	1792.578784	1630.145678	1676.26445	1528.817527	1618.012201	1747.958752	1695.033734
**ENSG00000055147**	FAM114A2	555.46114	451.9133251	521.1092604	544.2018744	518.9492378	499.2663873	560.7455996	589.7659858	566.9755796	583.4209033	662.2101035	636.9063882
**ENSG00000055163**	CYFIP2	277.0274546	287.2627338	279.2819943	516.5731638	540.9092278	464.363266	1099.02052	1902.202968	898.5320187	1191.520131	2103.896683	1048.823269
**ENSG00000055208**	TAB2	2242.938021	2466.255666	2421.529595	2044.52458	2304.643163	2068.389319	1523.920646	1976.962037	1488.486757	1438.936154	1738.301522	1458.202674
**ENSG00000055211**	GINM1	597.648062	784.7177118	587.8763844	601.9709964	722.3680927	658.6067237	762.9817176	845.6081318	849.2909634	797.299716	739.4679489	956.6283202
**ENSG00000055332**	EIF2AK2	7360.211663	8446.225014	6657.170802	3812.762055	4741.046266	3880.316573	4765.8269	4859.339461	4581.762955	2962.6645	2690.228546	2799.681468
**ENSG00000055483**	USP36	818.4262873	458.9197332	898.0992411	884.1187374	688.8502132	928.7265321	685.3557329	465.1675381	674.3679764	759.3330629	538.0457091	667.356096
**ENSG00000055609**	KMT2C	2384.967325	3751.931559	2323.821608	2976.365636	3311.335337	2681.090851	4419.574152	5625.204586	4092.166177	4921.111025	5726.737791	4427.049185
**ENSG00000055732**	MCOLN3	5.624922937	10.50961221	13.02773151	5.023401917	10.40210053	10.24330734	21.44928523	13.29050109	7.503398903	25.3111021	8.277626294	7.612426951
**ENSG00000055813**	CCDC85A	30.93707615	28.02563256	19.54159727	14.2329721	31.2063016	9.863925586	107.2464262	107.9853213	86.75804982	87.95607978	120.0255813	70.20349299
**ENSG00000055917**	PUM2	4318.534585	3867.537294	3982.414676	4043.838543	4044.10553	4128.811621	4495.157348	4505.479869	4271.309826	4378.187885	4458.881364	4616.514033
**ENSG00000055950**	MRPL43	399.3695285	402.8684681	427.4724402	452.1061725	389.5008756	446.9117054	303.3541769	272.4552723	377.5147573	315.1232211	309.0313816	377.2380467
**ENSG00000056050**	HPF1	253.1215321	276.7531216	257.2976973	255.3562641	278.5451365	238.6311228	183.8510163	186.0670152	191.336672	208.8165923	173.8301522	181.8524216
**ENSG00000056097**	ZFR	3384.797377	3289.508622	3463.748116	3560.754726	3139.122783	3587.054477	3514.618595	3174.768448	3414.984426	3218.939409	2968.908631	3556.695037
**ENSG00000056277**	ZNF280C	164.5289959	115.6057343	176.6886086	143.1669546	116.7346838	130.8867049	170.5728873	152.8407625	161.7920389	155.6632779	106.2295374	145.4819373
**ENSG00000056291**	NPFFR2	660.9284451	648.092753	727.9244982	656.3911838	659.9554895	744.7263817	179.7654381	189.3896405	253.7086754	164.5221636	147.6176689	241.0601868

ENSG00000056558	TRAF1	30.93707615	150.637775	102.5933857	46.04785091	65.87997005	61.83922579	13.27812895	33.22625272	23.917084	18.35054902	30.35129641	7.612426951
ENSG00000056586	RC3H2	4480.251119	4477.094802	4297.522932	4025.419403	3903.099278	4299.154028	2934.466499	3066.783126	3071.703926	2839.905655	2535.712855	3193.836019
ENSG00000056736	IL17RB	0	0	0	6.697869223	4.623155793	2.27629052	4.08557814	39.87150327	7.972361335	12.65555105	15.17564821	10.1499026
ENSG00000056972	TRAF3IP2	826.8636717	784.7177118	933.9255027	810.442176	649.5533889	777.7325943	466.7773024	433.602598	550.5618945	421.4298499	442.8530067	406.8419293
ENSG00000056998	GYG2	0	0	0	0	0	0	0	1.661312636	1.875849726	0	4.138813147	1.691650434
ENSG00000057019	DCBLD2	12058.42855	11280.31711	11808.82438	8547.318362	8213.036266	9121.854876	1366.625888	1516.778437	1522.721015	1538.28223	1638.970006	1912.410815
ENSG00000057252	SOAT1	2424.341786	1888.226994	2603.103603	1920.614	1623.883472	2187.894571	869.2067492	817.365817	1025.151875	818.8141528	728.4311139	1002.302882
ENSG00000057294	PKP2	251.7153014	283.7595297	273.5823617	283.8222083	258.8967244	298.1940581	1063.271711	2500.275517	1158.806168	1339.590078	2673.673293	1334.712192
ENSG00000057468	MSH4	12.65607661	17.51602035	22.79853014	22.60530863	20.80420107	23.90105046	40.8557814	51.50069172	22.97915914	34.16998783	55.18417529	35.5246591
ENSG00000057608	GDI2	4298.847354	4074.226334	3919.718718	3472.007958	3669.629911	3489.173985	2853.77633	2940.523366	3159.868863	2627.292397	2730.237073	3015.366898
ENSG00000057657	PRDM1	876.0817474	1933.768647	1160.282338	526.6199677	376.7871971	601.320079	53.11251581	98.01744553	67.99955256	21.51443678	30.35129641	21.99145564
ENSG00000057663	ATG5	821.2387487	931.8522827	771.0788588	792.8602693	867.9975001	855.8852355	624.0720608	759.2198747	558.0652934	516.9792603	762.9212234	519.3366831
ENSG00000057704	TMCC3	397.9632978	767.2016914	626.1453458	46.88508456	129.4483622	98.25987411	105.2036371	215.9706427	132.2474057	100.6116308	113.1275593	79.50757038
ENSG00000057757	PITHD1	570.9296781	602.5511001	567.520554	587.7380243	633.3723436	659.3654872	605.6869592	651.2345534	747.9950782	651.760879	551.8417529	743.4803655
ENSG00000057935	MTA3	469.6810652	532.4870187	441.3144049	565.9699493	582.5176299	512.5447487	656.7566859	629.6374891	624.6579587	706.1797485	613.9239501	684.2726004
ENSG00000058056	USP13	175.7788418	94.5865099	79.79485551	246.9839276	291.258815	249.2538119	132.7812895	91.37219499	144.9093913	207.5510372	108.9887462	169.1650434
ENSG00000058063	ATP11B	562.4922937	581.5318757	705.125968	648.856081	729.3028264	829.3285127	1053.057765	1214.419537	1142.861445	1187.723466	1383.743195	1374.465977
ENSG00000058085	LAMC2	29561.78249	40647.67683	46572.51169	35471.9154	41559.859	50360.27208	3632.078966	4108.426149	4447.170737	3945.368039	4330.578156	4915.090335
ENSG00000058091	CDK14	530.1489868	486.9453658	416.8874084	739.2773155	701.5638916	609.6664776	1546.391326	1363.937674	1504.43148	1450.32615	1245.782757	1643.438396
ENSG00000058262	SEC61A1	4176.50528	4210.851293	4826.774525	4075.653422	3988.62766	4731.269845	3301.147137	3123.267756	3968.360095	3627.713708	3123.424322	3980.45347
ENSG00000058272	PPP1R12A	1269.826353	1464.339301	1272.646522	1297.712162	1223.980496	1273.963928	1831.360401	2031.785354	1762.82978	1851.507118	1869.363938	1872.65703
ENSG00000058335	RASGRF1	402.18199	623.5703245	481.2118327	41.86168264	106.3325832	91.05162079	195.0863562	252.5195207	224.6330047	117.6966247	140.719647	82.04504603
ENSG00000058453	CROCC	115.3109202	94.5865099	113.9926507	103.816973	87.83996007	85.74027625	80.69016826	66.45250545	80.66153821	124.6571778	71.73942788	95.5782495
ENSG00000058600	POLR3E	690.4592905	399.365264	735.2525972	742.6262501	548.9997504	695.4067538	650.6283187	383.7632189	642.0095687	636.5742177	549.0825442	635.2147378
ENSG00000058668	ATP2B4	4443.68912	4701.299863	4065.466465	5327.317733	4713.307331	5097.752619	5653.418751	5500.606138	5147.80061	6560.004886	6346.180159	5953.763701
ENSG00000058673	ZC3H11A	32.34330689	49.04485699	21.17006371	45.21061725	41.60840214	27.69486799	39.83438686	34.88756536	23.917084	43.66165111	40.00852709	47.36621214
ENSG00000058729	RIOK2	586.3982161	469.4293454	583.8052183	679.8337261	505.0797704	564.5200489	563.8097833	513.3456046	576.3548283	542.2903624	535.2865003	638.5980387
ENSG00000058799	YIPF1	592.0231391	616.5639164	578.919819	513.2242292	523.5723936	539.1014714	594.4516193	521.6521677	689.8437367	587.2175686	525.6292697	703.7265804
ENSG00000058804	NDC1	1873.099338	1054.464425	1454.22053	2391.976546	2000.670669	2096.084187	2027.468152	1643.038197	2085.944895	2174.22367	1779.689653	2440.20575
ENSG00000058866	DGKG	12.65607661	17.51602035	30.12662912	0	0	0.758763507	5.106972674	3.322625272	8.441323766	0	0	0
ENSG00000059122	FLYWCH1	483.7433725	357.3268152	541.4650909	402.709387	401.058765	452.9818134	502.5261112	390.4084695	529.4585851	490.4026031	398.7056665	428.8333849
ENSG00000059145	UNKL	362.8075294	322.2947745	427.4724402	304.7530496	301.6609155	387.3487701	439.19965	375.4566558	498.5070646	432.1870683	400.0852709	427.9875597
ENSG00000059377	TBXAS1	0	7.006408141	1.628466439	0	0	1.517527013	0	3.322625272	0	0	0	1.691650434
ENSG00000059378	PARP12	1889.974107	2525.810135	2001.385253	588.575258	775.5343843	622.1860754	1094.934941	1146.305719	1168.654379	456.2326153	390.4280402	365.3964936
ENSG00000059573	ALDH18A1	870.4568244	581.5318757	754.7941944	809.6049423	795.1827964	846.0213099	961.1322573	908.738012	1112.84785	1055.472957	865.0119477	1098.726957
ENSG00000059588	TARBP1	1544.041346	1117.522098	1275.903455	1455.949322	1225.136285	1267.135056	1218.52368	858.8986329	1053.289621	1201.644572	819.4850031	1060.664822
ENSG00000059691	GATB	337.4953762	297.772346	365.5907155	324.8466573	321.3093276	318.301291	266.5839736	197.6962037	198.3711085	218.9410331	288.3373159	260.5141668
ENSG00000059728	MXD1	1515.916731	4361.489068	2296.951912	807.0932414	1213.578396	1058.854473	1154.175824	1407.131803	1099.247939	730.2252954	1030.564474	647.9021161
ENSG00000059758	CDK17	1465.292425	1376.7592	1574.727046	1132.777132	1128.050014	1272.825782	825.2867842	968.5452669	1012.958852	832.7352589	1004.35199	1098.726957
ENSG00000059769	DNAJC25	292.4959927	339.8107948	341.1637189	308.9392179	287.7914481	284.9156967	283.9476807	333.9238399	215.253756	261.9699067	317.3090079	317.1844563
ENSG00000059804	SLC2A3	764.9895194	4554.165292	718.9679328	656.3911838	4135.412857	799.736736	4202.017116	13591.19868	3741.382278	3560.639287	12539.22423	3506.791349
ENSG00000059915	PSD	0	3.50320407	0	0	6.93473369	2.27629052	8.171156279	6.645250545	2.344812157	7.593330629	1.379604382	10.1499026
ENSG00000060069	CTDP1	268.5900702	409.8748762	299.6378248	239.4488247	301.6609155	290.2270413	347.2741419	471.8127887	361.5700347	331.5754374	411.1221059	351.017465
ENSG00000060138	YBX3	3569.013603	2932.181807	3018.362544	3934.998168	3892.697178	3800.646405	4161.161335	4111.748774	4224.882545	4145.958523	4151.229586	4725.625486
ENSG00000060140	STYK1	234.8405326	430.8941007	276.8392946	269.5892362	265.8314581	316.4043823	216.5356414	220.9545806	205.405545	175.279382	217.9774924	230.064459
ENSG00000060237	WNK1	1726.851342	1835.678933	1879.25027	2000.151197	2215.647414	2247.836888	3031.49898	2997.007996	3081.552137	2971.523386	3044.786872	3472.95834
ENSG00000060339	CCAR1	2356.84271	2228.037789	2285.552647	2651.518979	2506.906229	2425.766931	2662.775552	2339.128192	2380.453302	2402.023589	2535.712855	2575.537785
ENSG00000060491	OGFR	1313.419506	1222.618221	1256.361858	625.4135387	653.0207558	643.0520719	1023.437324	1204.451661	1147.082107	566.3359094	670.4877298	632.6772621
ENSG00000060558	GNA15	352.9639143	329.3011826	327.3217542	291.3573112	257.7409355	295.5383858	16.34231256	74.75906863	51.58586746	28.47498986	51.04536215	38.90795997
ENSG00000060566	CREB3L3	1.406230734	0	1.628466439	0	0	0.379381753	3.064183605	3.322625272	14.06887294	6.960553076	0	16.91650434
ENSG00000060642	PIGV	482.3371418	378.3460396	403.0454436	378.4296111	451.9134788	351.3075036	482.0982205	624.6535512	511.6380127	534.0642542	469.06549	482.1203736
ENSG00000060656	PTPRU	1784.506802	1558.925811	1895.534935	1790.842783	1940.569644	2113.915129	1409.524458	1355.631111	1600.568779	1597.130542	1807.281741	1544.476846
ENSG00000060688	SNRNP40	781.8642882	651.5959571	697.7978691	784.4879327	762.8207059	705.2706794	816.0942334	853.914695	872.2701225	839.0630345	660.8304991	789.1549273
ENSG00000060709	RIMBP2	0	0	0	0	0	2.27629052	5.106972674	3.322625272	0.468962431	0	6.898021912	0
ENSG00000060718	COL11A1	2.812461468	3.50320407	8.142332194	82.04889798	52.01050267	19.72785117	5.106972674	23.25837691	36.11010722	18.98332657	59.32298844	65.97436691
ENSG00000060749	QSER1	3332.76684	3475.178438	3364.411663	4259.007592	4472.90323	4280.564323	3926.240592	3610.032358	4321.957768	4304.785689	4127.776312	4900.711306
ENSG00000060762	MPC1	63.28038304	45.54165292	78.16638907	89.58400086	75.12628164	76.63511417	157.2947584	176.0991394	152.8817527	194.2627086	164.1729215	181.0065964
ENSG00000060971	ACAA1	400.7757592	385.3524477	450.2709704	472.1997802	539.7534388	597.5262615	473.9270642	387.0858442	432.8523242	441.045954	480.102325	436.4458119
ENSG00000060982	BCAT1	57.6554601	38.53524477	66.76712399	19.25637402	43.91998003	45.5258104	1021.394535	538.2652941	957.621285	523.9398134	306.2721729	645.3646404
ENSG00000061273	HDAC7	660.9284451	546.499835	683.1416711	682.3454271	714.27757	758.3841249	743.5752214	787.4621895	842.2565269	772.6213915	896.7428485	826.3712368
ENSG00000061455	PRDM6	1.406230734	14.01281628	1.628466439	3.348934611	0	0.758763507	10.21394535	1.661312636	4.689624315	5.694997971	5.518417529	5.920776517
ENSG00000061656	SPAG4	8.437384405	21.01922442	6.513865756	2.511700959	39.29682424	6.828871559	4.08557814	94.69482026	9.848211061	10.12444084	64.84140597	3.383300867
ENSG00000061676	NCKAP1	3962.758209	4063.716722	4006.841673	4248.960788	4336.520134	4452.044875	3636.164544	3568.499542	3544.418057	3575.825948	3642.155569	3993.140848
ENSG00000061794	MRPS35	158.904073	150.637775	160.4039442	162.4233287	152.5641412	176.791897	251.2630556	245.8742701	246.6742389	271.46157	240.0511625	313.8011554
ENSG00000061918	GUCY1B1	8.437384405	14.01281628	21.17006371	9.209570182	23.11577897	14.79588838	0	0	2.344812157	0	0	0
ENSG00000061936	SFSWAP	817.0200565	798.7305281	955.0955664	905.0495787	747.7954495	849.8151274	1581.11874	1398.82524	1376.404736	1592.701099	1418.233305	1411.682287
ENSG00000061938	TNK2	542.8050634	392.3588559	448.6425039	623.7390714	589.4523636	654.8129062	402.4294467	305.681525	420.1903386	382.1976416	398.7056665	376.3922215

ENSG00000061987	MON2	1050.454358	1138.541323	1129.341475	1141.986703	1106.090023	1214.400992	2019.296995	1777.604521	1658.251158	1902.129322	1883.159982	1896.340136
ENSG00000062038	CDH3	1874.505569	3093.329194	2752.922515	1666.094969	1908.207554	2211.795622	39.83438686	79.74300653	173.0471372	49.98942664	84.15586732	157.3234903
ENSG00000062194	GPBP1	2985.427849	3293.011826	2932.868056	2451.420136	2504.594651	2451.185508	2640.304873	2815.924918	2451.266629	2324.824727	2386.715581	2527.325748
ENSG00000062282	DGAT2	74.53022891	91.08330583	105.0360853	85.39783259	120.2020506	111.5382355	12.25673442	1.661312636	9.379248629	15.18666126	1.379604382	26.22058172
ENSG00000062370	ZNF112	54.84299863	154.1409791	107.478785	86.23506625	145.6294075	99.77740112	132.7812895	217.6319553	123.8060819	150.6010575	183.4873828	112.4947538
ENSG00000062485	CS	1884.349184	1565.932219	1875.993338	2156.71389	1974.087524	2045.247032	1987.633765	1852.363589	2083.131121	2132.460352	1985.250706	2304.873716
ENSG00000062582	MRPS24	8.437384405	24.52242849	17.91313083	25.95424324	6.93473369	18.58970591	18.38510163	9.967875817	11.72406079	8.858885733	12.41643944	17.76232955
ENSG00000062598	ELMO2	531.5552175	627.0735286	582.1767519	515.7359302	572.1155294	580.0747008	584.2376739	697.7513072	610.1201233	558.1098012	582.1930493	622.5273595
ENSG00000062650	WAPL	3085.270231	2809.569664	3135.612128	2920.270981	2941.482873	3023.672574	2789.428475	2736.181912	2697.002943	2534.906875	2625.38714	2753.161081
ENSG00000062716	VMP1	8309.417408	9437.631766	8669.955321	5321.457098	5092.406106	6296.978341	3885.384811	4754.676765	3653.217341	2821.555106	3756.662733	2756.544381
ENSG00000062725	APPBP2	863.4256708	903.8266502	822.3755516	640.4837444	844.8817212	697.6830443	847.7574639	882.1570098	788.3258473	718.8352995	840.1790688	745.172016
ENSG00000062822	POLD1	279.8399161	294.2691419	225.5426018	241.123292	300.5051265	285.6744602	259.4342119	290.7297113	284.6601959	265.766572	263.504437	249.5184389
ENSG00000063046	EIF4B	7472.710121	7192.077957	6892.484203	7983.02288	7910.219562	7548.938127	7600.196734	8598.954205	7935.782265	8798.771866	9673.785929	8873.552349
ENSG00000063127	SLC6A16	5.624922937	0	10.58503185	7.535102876	6.93473369	11.00207085	2.04278907	8.306563181	0.468962431	0.632777552	4.138813147	2.53747565
ENSG00000063169	BICRA	151.8729193	42.03844885	139.2338805	145.6786556	137.5388848	153.6496101	181.8082272	132.9050109	132.7163681	169.584384	129.6828119	146.3277625
ENSG00000063176	SPHK2	74.53022891	108.5993262	72.46675653	75.35102876	58.94523636	112.296999	80.69016826	58.14594226	70.81332715	93.65107775	81.39665856	69.35766778
ENSG00000063177	RPL18	3154.175537	2662.435094	2943.453088	3270.234648	2832.838712	3242.955227	2525.908685	2739.504537	3107.814033	2668.422938	2981.32507	2924.017774
ENSG00000063180	CA11	153.27915	115.6057343	123.7634494	95.44463643	112.111528	89.53409378	109.2892152	91.37219499	76.44087633	110.7360717	135.2012295	65.12854169
ENSG00000063241	ISOC2	208.1221487	105.0961221	184.0167076	172.4701325	138.6946738	157.0640459	103.160848	36.54887799	101.2958852	97.44774307	77.25784541	124.3363069
ENSG00000063244	U2AF2	1319.044429	1306.695118	1407.809236	1592.418408	1322.222557	1564.190969	1320.663134	1219.403475	1426.583716	1319.973974	1419.612909	1463.277625
ENSG00000063245	EPN1	646.8661377	641.0863449	611.4891478	672.2986233	768.5996506	736.0006014	521.9326073	430.2799728	548.2170824	537.8609195	550.4621485	516.7992075
ENSG00000063322	MED29	412.0256051	420.3844885	453.5279032	455.4551072	438.0440114	521.270529	452.477779	450.2157244	457.7073331	416.3676295	409.7425015	434.7541614
ENSG00000063438	AHRR	4.218692202	10.50961221	4.885399317	2.511700959	2.311577897	3.035054026	4.08557814	11.62918845	9.848211061	3.796665314	13.79604382	3.383300867
ENSG00000063587	ZNF275	171.5601496	210.1922442	147.3762127	136.4690854	162.9662417	201.451711	91.92550814	99.67875817	74.09606417	101.2444084	93.813098	106.5739773
ENSG00000063601	MTMR1	984.3615139	984.4003438	1053.617786	938.5389249	831.0122538	927.5883868	1185.839055	1254.29104	1013.427814	1075.721839	1194.737395	1005.686183
ENSG00000063660	GPC1	300.9333771	297.772346	235.3134004	425.3146957	469.250313	462.845739	469.841486	639.6053649	728.7676185	696.6880852	641.5160378	740.0970647
ENSG00000063761	ADCK1	73.12399818	59.5544692	71.65252331	62.79252397	57.78944741	50.07839144	98.05387535	76.42038126	114.8957957	93.65107775	115.8867681	133.6403843
ENSG00000063854	HAGH	344.5265299	451.9133251	337.0925529	376.7551438	450.7576898	443.8766514	299.2685987	269.1326471	315.1427539	291.7104516	318.6886123	344.2508632
ENSG00000063978	RNF4	836.7072868	858.2849973	985.2221955	780.3017645	720.0565148	907.1017722	556.6600215	566.5076089	673.899014	641.0036606	528.3884784	679.1976491
ENSG00000064012	CASP8	1860.443261	1723.576403	1893.906468	1098.450553	908.4501133	976.9080148	1253.251094	890.463573	1042.034523	868.8035794	648.4140597	768.0092968
ENSG00000064042	LIMCH1	1231.858123	1579.945036	1078.859016	1520.416314	2337.005253	1497.040399	1740.456287	3176.42976	1178.50259	1837.586012	2920.622477	1397.303258
ENSG00000064102	INTS13	201.090995	353.8236111	192.1590398	209.3084132	242.7156791	166.9279715	390.1727123	415.328159	324.0530401	278.422123	387.6688314	359.4757171
ENSG00000064115	TM7SF3	302.3396078	245.2242849	298.0093583	286.3339093	325.9324834	268.6022813	510.6972674	385.4245316	547.7481199	564.4375767	489.7595557	548.0947405
ENSG00000064195	DLX3	15.46853808	28.02563256	15.47043117	36.00104707	20.80420107	31.10930377	89.88271907	107.9853213	147.7231659	124.6571778	100.7111199	189.4648486
ENSG00000064199	SPA17	219.3719945	238.2178768	144.1192798	235.2626565	196.4841212	165.7898262	240.0277157	264.1487091	218.0675306	208.1838147	241.4307669	224.1436824
ENSG00000064205	WISP2	0	0	2.442699658	0	0	0	11.23533988	58.14594226	7.972361335	8.226108181	51.04536215	7.612426951
ENSG00000064225	ST3GAL6	74.53022891	143.6313669	67.58135721	79.53719702	161.8104528	94.08667482	319.6964894	455.1996623	282.7843462	382.1976416	495.2779732	367.9339693
ENSG00000064270	ATP2C2	213.7470716	178.6634076	241.8272662	266.2403016	279.7009255	260.6352645	20.4278907	81.40431917	33.29633263	31.00610007	106.2295374	16.91650434
ENSG00000064300	NGFR	5.624922937	0	0.814233219	8.372336529	6.93473369	4.173199286	10.21394535	8.306563181	4.220661883	5.062220419	5.518417529	2.53747565
ENSG00000064309	CDON	22.49969175	21.01922442	20.35583049	19.25637402	18.49262317	30.72992202	26.55625791	41.5328159	13.59991051	20.88165923	30.35129641	34.67883389
ENSG00000064313	TAF2	1226.2332	1191.089384	1125.270309	1129.428198	1074.883722	1147.250422	1149.068852	1103.11159	1043.910372	1118.117935	1004.35199	1135.097441
ENSG00000064393	HIPK2	53.4367679	168.1537954	262.1830967	23.44254228	83.21680428	130.1279414	55.15530488	79.74300653	47.83416801	39.23220825	38.6289227	35.5246591
ENSG00000064419	TNPO3	1825.287493	1807.6533	1886.578369	2187.691535	1874.689674	2235.696672	1786.419041	1608.150632	1837.863769	1893.903214	1843.151455	1986.843434
ENSG00000064489	BORCS8-ME	0	0	0.814233219	3.348934611	1.155788948	2.27629052	9.192550814	4.983937908	3.28273702	8.858885733	4.138813147	1.691650434
ENSG00000064490	RFXANK	337.4953762	280.2563256	275.2108282	272.1009372	238.0925233	285.6744602	199.1719343	161.1473257	274.8119848	187.9349331	172.4505478	235.9852355
ENSG00000064545	TMEM161A	95.62368992	38.53524477	80.60908873	109.6776085	86.68417112	116.84958	68.43343384	46.51675381	98.95107304	68.33997566	78.63744979	102.3448512
ENSG00000064547	LPAR2	116.7171509	119.1089384	91.19412058	97.95633739	112.111528	111.1588537	93.96829721	84.72694444	90.04078684	74.03497363	97.95191114	99.80737558
ENSG00000064601	CTSA	1750.757264	1765.614851	2055.124646	1457.62379	1844.639161	1701.527164	1648.530779	1802.52421	1882.884162	1814.173243	1924.548113	1838.824021
ENSG00000064607	SUGP2	1005.454975	574.5254675	960.7951989	1099.287786	1131.51738	1139.662787	870.2281437	860.5599455	930.8904264	1013.076861	1065.054583	956.6283202
ENSG00000064651	SLC12A2	1327.481813	886.3106298	1113.056811	2201.087273	1895.493875	1941.675813	4500.264321	4357.623045	3787.340596	5394.428634	5323.893311	5284.715954
ENSG00000064652	SNX24	490.7745262	444.9069169	450.2709704	533.3178369	381.4103529	487.8849347	564.8311778	564.8462963	587.6099266	461.9276132	449.7510286	608.1483309
ENSG00000064655	EYA2	2.812461468	3.50320407	8.956565414	0	0	0	0	8.306563181	0.468962431	0.632777552	4.138813147	0
ENSG00000064666	CNN2	2105.127409	1474.848914	2192.73006	2260.530863	1942.881222	2531.993822	1675.087037	2457.081389	2022.166004	1790.127696	2552.268107	1984.305959
ENSG00000064687	ABCA7	254.5277629	371.3396315	294.7524254	241.123292	366.3850966	288.3301325	217.5570359	456.8609749	193.6814842	241.721025	487.000347	201.3064016
ENSG00000064692	SNCAIP	0	3.50320407	7.328098975	1.674467306	2.311577897	0.758763507	0	0	1.406887294	3.163887762	0	0
ENSG00000064703	DDX20	696.0842134	500.9581821	626.1453458	681.5081934	550.1555394	651.7778522	774.2170574	637.9440523	640.6026814	651.760879	580.813445	650.4395917
ENSG00000064726	BTBD1	1150.296741	959.8779153	988.4791284	983.7495421	1040.210053	1070.615308	546.4460762	599.7338616	536.4930216	522.0414807	540.8049179	630.9856117
ENSG00000064763	FAR2	497.8056799	332.8043867	461.6702354	451.2689389	344.4251066	385.0724796	331.9532238	272.4552723	355.9424855	315.7559986	269.0228546	306.1887285
ENSG00000064787	BCAS1	8.437384405	10.50961221	15.47043117	0.837233653	11.55788948	8.725780326	2.04278907	1.661312636	3.751699452	4.429442867	2.759208765	10.1499026
ENSG00000064886	CHI3L2	0	0	4.071166097	6.697869223	8.090522638	8.725780326	0	4.983937908	1.875849726	0.632777552	0	1.691650434
ENSG00000064932	SBNO2	944.9870534	774.2080996	1131.784175	751.8358203	670.35759	986.3925586	636.3287952	636.2827396	687.9678869	585.319236	543.5641266	476.199597
ENSG00000064933	PMS1	361.4012987	350.320407	338.7210193	381.7785457	354.8272071	362.3095744	352.3811145	320.6333388	366.259659	357.5193171	310.410986	356.9382415
ENSG00000064961	HMG20B	558.2736015	585.0350798	644.0584766	482.2465841	461.1597904	514.0622757	437.1568609	335.5851525	489.127816	423.9609601	382.1504139	423.7584336
ENSG00000064989	CALCRL	4.218692202	0	8.956565414	0	1.155788948	5.6907263	8.171156279	0	2.344812157	10.75721839	2.759208765	11.84155303
ENSG00000064995	TAF11	404.9944514	322.2947745	326.507521	526.6199677	379.098775	500.7839144	468.8200915	598.072549	484.4381917	552.4148032	491.1391601	550.6322161
ENSG00000064999	ANKS1A	655.3035221	567.5190594	710.0113674	531.6433696	551.3113283	621.4273119	619.9864827	493.4098529	596.9891752	600.5058972	669.1081254	595.4609526
ENSG00000065000	AP3D1	2040.440795	1790.13728	2257.054484	1851.123606	1818.056016	2110.880075	1816.039483	1835.750463	1912.428795	1823.032128	1765.893609	1808.374313

**ENSG00000065029**	ZNF76	299.5271464	217.1986524	311.851323	318.1487881	299.3493376	289.8476595	328.8890402	295.7136492	316.0806788	322.7165517	310.410986	242.7518372
**ENSG00000065054**	SLC9A3R2	84.37384405	42.03844885	35.01202844	113.0265431	119.0462617	82.32584047	9.192550814	11.62918845	8.910286198	12.65555105	6.898021912	16.91650434
**ENSG00000065057**	NTHL1	70.31153671	91.08330583	85.49448804	100.4680383	105.1767943	110.0207085	51.06972674	29.90362745	91.44767413	43.02887356	52.42496653	83.73669646
**ENSG00000065060**	UHRF1BP1	1967.316797	1590.454648	2105.607105	1679.490708	1384.63516	1862.385027	2031.55373	1422.083617	2414.68756	1809.7438	1116.099945	1816.832566
**ENSG00000065135**	GNAI3	2570.589782	2588.867808	2397.916831	2276.438302	2383.236811	2381.379265	2107.136925	2626.535278	2086.88282	2010.967061	2332.91101	2237.207698
**ENSG00000065150**	IPO5	5232.584562	3902.569334	4744.53697	5992.918487	5068.134538	5627.748929	4758.677138	4002.10214	4878.147212	5021.089878	4304.365673	5573.142353
**ENSG00000065154**	OAT	277.0274546	318.7915704	294.7524254	426.1519293	435.7324335	424.9075637	251.2630556	313.9880882	188.5228974	281.5860108	398.7056665	256.2850407
**ENSG00000065183**	WDR3	1295.138506	662.1055693	1113.056811	1333.713209	958.1490381	1194.673141	1053.057765	745.9293736	1077.675667	1088.37739	765.6804322	1233.213166
**ENSG00000065243**	PKN2	2804.024084	2865.62093	2851.444735	2379.418041	2423.689425	2594.971193	2533.058446	2262.70781	2386.549814	2311.536399	2292.902483	2405.526917
**ENSG00000065268**	WDR18	212.3408409	129.6185506	199.4871388	232.7509555	198.7956991	239.7692681	155.2519693	147.8568246	229.7915914	206.9182596	209.6998661	232.6019346
**ENSG00000065308**	TRAM2	3630.887756	3871.040498	3373.368228	4711.113765	6244.727687	4755.929659	3003.921327	9545.902407	3522.845785	3485.338759	9781.395071	3502.562223
**ENSG00000065320**	NTN1	0	0	4.885399317	5.86063557	5.778944741	6.070108053	11.23533988	14.95181373	11.72406079	9.491663286	2.759208765	1.691650434
**ENSG00000065325**	GLP2R	0	0	0	0	0	0	5.106972674	4.983937908	3.28273702	1.265555105	1.379604382	10.99572782
**ENSG00000065328**	MCM10	817.0200565	952.8715072	807.7193537	1059.100571	909.6059023	1005.741028	729.2756979	837.3015686	769.56735	885.2557958	727.0515095	1000.611231
**ENSG00000065357**	DGKA	1482.167194	1324.211139	1494.117958	1602.465212	1549.91298	1608.958016	1561.712244	1176.209346	1287.301874	1536.383897	1187.839373	1260.279573
**ENSG00000065361**	ERBB3	1058.891743	1362.746383	1072.34515	1362.179153	926.9427365	1145.732895	1547.41272	1265.920229	1453.314575	1456.653926	1364.428734	1470.044227
**ENSG00000065413**	ANKRD44	43.59315276	31.52883663	17.91313083	36.83828073	26.58314581	47.42271916	134.8240786	53.16200436	190.3987472	135.4143962	93.813098	210.610479
**ENSG00000065427**	KARS	5203.053716	5268.818922	5331.599121	3224.186797	3075.554391	3323.763541	2801.685209	2879.054798	3035.124856	2212.190323	2228.061077	2342.090025
**ENSG00000065457**	ADAT1	929.5185153	721.6600385	938.810902	943.5623268	944.2795707	1043.679203	1055.100555	717.6870588	1018.586401	1022.568525	802.9297505	1062.356472
**ENSG00000065485**	PDIA5	302.3396078	297.772346	270.3254289	228.5647872	204.5746438	239.7692681	178.7440436	159.4860131	204.4676201	192.9971535	151.7564821	197.9231007
**ENSG00000065491**	TBC1D22B	362.8075294	364.3332233	377.8042138	380.1040784	349.0482624	411.6292023	335.0174074	397.05372	350.3149363	356.8865395	338.0030737	331.563485
**ENSG00000065518**	NDUFB4	968.8929758	1089.496466	907.8700397	987.0984767	968.5511386	967.8028527	894.7416126	970.2065795	869.9253103	958.6579919	935.3717712	1054.744045
**ENSG00000065526**	SPEN	4664.467345	4897.47929	5270.531629	4768.045653	4642.804205	4946.758681	4601.38238	4661.643257	4796.547749	4592.066698	4784.467998	4923.548587
**ENSG00000065534**	MYLK	1234.670585	2045.871177	2499.695984	1848.611906	2938.015506	3345.767682	78.64737919	215.9706427	107.8613592	104.4082961	140.719647	108.2656277
**ENSG00000065548**	ZC3H15	1258.576507	1047.458017	1210.764797	1264.222816	1252.87522	1243.992769	1073.485656	985.1583932	957.621285	1058.636845	903.6408704	1067.431424
**ENSG00000065559**	MAP2K4	638.4287533	585.0350798	658.7146745	693.2294646	661.1112784	697.6830443	697.6124673	745.9293736	655.6094792	641.0036606	660.8304991	664.8186204
**ENSG00000065600**	TMEM206	289.6835312	210.1922442	268.6969624	321.4977227	308.5956492	292.5033318	159.3375474	222.6158932	176.7988367	188.5677106	193.1446135	193.6939746
**ENSG00000065609**	SNAP91	0	0	0	0	0	4.931962793	0	0	0	0	0	0
**ENSG00000065613**	SLK	8361.447945	10499.1026	8218.055884	11448.33297	10300.39111	9854.06166	6291.790335	6273.116514	5829.203023	5823.451815	6259.265083	6730.23125
**ENSG00000065615**	CYB5R4	632.8038304	795.227324	677.4420386	632.9486416	632.2165547	583.4891366	648.5855296	634.621427	525.7068857	609.9975605	688.4225868	655.514543
**ENSG00000065618**	COL17A1	10888.44457	13059.94477	11482.31686	11045.62358	14905.05428	12401.23075	8331.515221	17648.12413	7440.088975	7532.583984	16592.50191	6878.250663
**ENSG00000065621**	GSTO2	296.7146849	171.6569995	188.9021069	324.0094237	316.6861718	328.9239801	490.2693767	363.8274673	508.8242381	556.2114685	411.1221059	509.1867805
**ENSG00000065665**	SEC61A2	101.2486129	63.05767327	77.35215585	129.7712162	73.97049269	107.3650362	177.7226491	196.0348911	146.3162786	143.6405044	162.7933171	163.2442668
**ENSG00000065675**	PRKCQ	106.8735358	231.2114686	308.5943902	140.6552537	235.7809454	268.9816631	15.32091802	61.46856754	33.76529506	6.960553076	27.59208765	38.06213475
**ENSG00000065717**	TLE2	25.31215321	52.54806106	26.86969624	54.42018744	61.25681426	34.9031213	82.73295732	49.83937908	60.02719123	77.19886139	75.87824103	82.89087124
**ENSG00000065802**	ASB1	212.3408409	248.727489	223.9141353	288.0083766	325.9324834	325.8889261	213.4714578	214.3093301	211.5020566	240.4554699	211.0794705	209.7646538
**ENSG00000065809**	FAM107B	3016.364925	3335.050275	2529.00838	2906.875243	3355.255317	2626.459878	2548.379365	2786.021291	2229.916362	2303.310291	2566.064151	2522.250796
**ENSG00000065833**	ME1	254.5277629	399.365264	357.4483833	252.0073295	309.7514381	352.0662671	109.2892152	69.77513072	77.84776362	84.79219202	106.2295374	129.4112582
**ENSG00000065882**	TBC1D1	525.9302946	490.4485699	487.7256984	662.2518194	634.5281326	653.2953792	210.4072742	161.1473257	187.1160101	181.6071575	150.3768777	168.3192181
**ENSG00000065883**	CDK13	1794.350417	1965.297484	1744.087556	1713.817287	1785.693925	1795.613838	2453.389673	2570.050648	2084.538008	2297.615293	2550.888503	2238.053524
**ENSG00000065911**	MTHFD2	3889.634211	3555.752131	4304.851031	3177.301713	2864.045014	3778.642263	2638.262084	2136.44805	2932.422084	2212.823101	1819.69818	2360.69818
**ENSG00000065923**	SLC9A7	856.3945171	1418.797649	1402.109604	494.8050888	858.7511886	845.6419281	183.8510163	279.1005229	242.4535771	163.2566085	219.3570968	192.8481494
**ENSG00000065970**	FOXJ2	472.4935267	1005.419568	520.2950272	550.8997436	938.500626	715.8933685	713.9547799	1317.42092	740.0227168	723.2647424	1327.179416	738.4054142
**ENSG00000065978**	YBX1	10169.86067	9160.878644	9598.995424	11185.4416	11055.12129	10614.72208	7177.339397	7489.197364	7482.295594	7135.832458	7637.48986	8877.781475
**ENSG00000065989**	PDE4A	21.09346101	70.06408141	30.94086234	9.209570182	39.29682424	23.90105046	11.23533988	14.95181373	3.751699452	2.53111021	11.03683506	2.53747565
**ENSG00000066027**	PPP2R5A	714.365213	714.6536304	771.893092	879.9325692	1000.913229	881.3038129	722.1259362	699.4126198	706.7263842	709.3436362	713.2554657	762.0885203
**ENSG00000066044**	ELAVL1	1424.511734	1015.92918	1221.349829	1429.157845	1275.990999	1602.129144	1358.454731	1123.047342	1493.645344	1288.335097	1107.822319	1508.106362
**ENSG00000066056**	TIE1	4.218692202	0	8.142332194	4.186168264	3.467366845	7.587635066	0	0	0	0	0	1.691650434
**ENSG00000066084**	DIP2B	3006.52131	2648.422277	2993.121315	3630.245119	3270.882724	3542.666812	6328.560538	5236.457429	5656.624848	6282.848318	5975.06658	5621.354391
**ENSG00000066117**	SMARCD1	1217.795816	756.6920792	1135.041108	1136.126067	970.8627165	1073.650362	1622.995916	1696.200201	1671.851068	1590.169989	1578.267413	1620.601115
**ENSG00000066135**	KDM4A	582.1795239	392.3588559	545.536257	585.2263234	588.2965747	591.8355352	644.4999515	651.2345534	674.8369389	663.1508749	778.0968716	664.8186204
**ENSG00000066136**	NFYC	1012.486129	896.820242	973.0086972	1076.682478	938.500626	1058.09571	1265.507829	922.028513	1133.951159	1262.391217	971.2414851	1081.810452
**ENSG00000066185**	ZMYND12	1.406230734	3.50320407	0.814233219	1.674467306	2.311577897	2.27629052	0	9.967875817	8.910286198	0	1.379604382	0.845825217
**ENSG00000066230**	SLC9A3	2.812461468	0	3.256932878	1.674467306	1.155788948	2.27629052	0	4.983937908	0.937924863	0.632777552	1.379604382	1.691650434
**ENSG00000066248**	NGEF	28.12461468	66.56087734	47.22552673	72.00209415	94.77469376	66.77118858	0	0	0.937924863	1.265555105	0	0
**ENSG00000066279**	ASPM	5257.896715	2231.540993	4478.282707	6999.273338	6352.21606	6189.992687	5742.280075	3595.080545	5886.885402	6131.614483	4934.844876	6935.766778
**ENSG00000066294**	CD84	0	0	0	0	0	0	0	3.322625272	0.937924863	2.53111021	0	5.920776517
**ENSG00000066322**	ELOVL1	2224.657021	1551.919403	1942.760462	2939.527355	2640.977747	2860.53842	1006.073617	1211.096912	1304.653484	1320.606752	1233.366318	1503.877235
**ENSG00000066379**	ZNRD1	444.368912	336.3075908	412.002009	411.9189572	386.0335087	457.5343945	371.7876107	322.2946514	438.9488358	413.2037417	289.7169203	474.5079466
**ENSG00000066422**	ZBTB11	1026.548436	1068.477241	1175.752769	875.7464009	952.3700934	1089.963777	1216.480891	1412.115741	1392.349459	1338.324523	1400.298448	1503.03141
**ENSG00000066427**	ATXN3	341.7140684	332.8043867	251.5980648	288.8456102	380.254564	280.7424974	530.1037636	500.0551035	448.3280845	487.2387153	544.943731	532.8698866
**ENSG00000066455**	GOLGA5	714.365213	921.3426705	821.5613184	573.5050522	732.7701932	691.6129363	817.1156279	1043.304335	826.3118042	770.7230588	1015.388825	864.4333715
**ENSG00000066468**	FGFR2	769.2082116	402.8684681	583.8052183	946.9112614	950.0585155	816.8089149	1387.053778	2118.173611	1533.976113	1652.182189	2154.942045	1675.579754
**ENSG00000066557**	LRRC40	465.462373	357.3268152	423.4012741	518.2476311	531.6629162	509.5096947	495.3763494	476.7967266	491.9415906	482.1764949	478.7227207	466.8955197
**ENSG00000066583**	ISOC1	254.5277629	301.2755501	331.3929203	384.2902467	451.9134788	433.2539623	411.6219976	450.2157244	420.1903386	384.7287518	372.4931832	482.1203736
**ENSG00000066629**	EML1	0	0	0	0.837233653	0	4.173199286	0	1.661312636	1.406887294	2.53111021	6.898021912	3.383300867
**ENSG00000066651**	TRMT11	957.64313	840.7689769	883.4430431	812.9538769	671.5133789	763.3160876	1021.394535	877.1730719	927.6076894	870.0691345	878.8079915	814.5296838

**ENSG00000066654**	THUMPD1	1019.517282	826.7561606	1073.973616	915.9336162	774.3785953	865.749161	1224.652047	988.4810185	1093.151428	1108.626272	1087.128253	1173.159576
**ENSG00000066697**	MSANTD3	566.7109859	826.7561606	653.8292752	616.2039685	656.4881226	688.957264	367.7020326	676.1542429	376.10787	430.2887356	576.6746318	458.4372675
**ENSG00000066739**	ATG2B	496.3994492	423.8876925	466.5556347	612.0178002	613.7239315	599.0437885	1063.271711	729.3162473	952.9316607	1114.32127	889.8448266	1016.681911
**ENSG00000066777**	ARFGEF1	2522.777937	2623.899849	2358.833637	2934.503953	3095.202803	2674.641361	1881.408733	1897.21903	1634.334074	1948.322084	1927.307322	2019.830618
**ENSG00000066827**	ZFAT	244.6841477	213.6954483	264.6257963	277.1243391	260.0525134	239.7692681	352.3811145	446.8930991	339.5288004	438.5148438	431.8161717	364.5506684
**ENSG00000066855**	MTFR1	736.8649047	420.3844885	733.6241307	971.1910373	835.6354096	897.6172283	498.440533	390.4084695	587.1409642	587.8503462	528.3884784	665.6644456
**ENSG00000066923**	STAG3	344.5265299	444.9069169	301.2662912	234.4254228	270.4546139	243.1837039	352.3811145	420.3120969	318.425491	339.1687681	445.6122155	251.2100894
**ENSG00000066926**	FECH	229.2156097	231.2114686	181.5740079	271.2637035	231.1577897	268.6022813	308.4611495	335.5851525	394.3974049	387.2598621	249.7083932	367.9339693
**ENSG00000066933**	MYO9A	1216.389585	1380.262404	1309.287017	1552.231192	1666.647663	1539.151773	1136.812117	1129.692593	1135.827009	1071.292396	950.5474194	1218.834137
**ENSG00000067057**	PFKP	1774.663187	2396.191584	1915.890765	1728.05026	2451.428359	1992.512968	706.8050181	1895.557718	824.9049169	686.5636443	1696.91339	673.2768726
**ENSG00000067064**	IDI1	1456.855041	1758.608443	1487.604092	1154.545207	1191.618406	1243.992769	468.8200915	750.9133115	562.2859553	494.1992684	749.1251796	595.4609526
**ENSG00000067066**	SP100	3030.427232	4060.213518	3038.718375	802.9070731	1144.231059	782.2851753	1624.01731	2066.672919	1544.293287	729.5925179	802.9297505	707.1098812
**ENSG00000067082**	KLF6	2865.898236	3478.681642	3533.772172	1455.112089	1987.956991	2003.515039	1491.236021	1937.090534	1357.646239	981.4379837	1670.700907	999.7654062
**ENSG00000067113**	PLPP1	15.46853808	115.6057343	27.68392946	33.48934611	45.07576898	36.80003007	150.1449966	142.8728867	174.4540245	132.2505084	135.2012295	164.0900921
**ENSG00000067141**	NEO1	28.12461468	24.52242849	26.86969624	0.837233653	9.246311586	20.86599643	62.30506663	56.48462963	40.79973154	57.58275727	48.28615338	38.06213475
**ENSG00000067167**	TRAM1	4664.467345	4501.61723	4431.871413	3349.771845	3718.173047	3758.53503	2401.298551	2683.019907	2437.666719	1955.282637	2302.559714	2274.424008
**ENSG00000067177**	PHKA1	4.218692202	10.50961221	14.65619795	10.04680383	10.40210053	12.14021611	26.55625791	14.95181373	9.379248629	6.327775524	12.41643944	7.612426951
**ENSG00000067182**	TNFRSF1A	698.8966749	641.0863449	910.3127393	612.0178002	543.2208057	723.1016218	1088.806574	717.6870588	1177.564665	968.7824327	833.2810469	1071.66055
**ENSG00000067191**	CACNB1	30.93707615	14.01281628	28.49816268	52.74572013	55.47786952	45.14642864	13.27812895	29.90362745	28.60670832	22.77999189	30.35129641	30.4497078
**ENSG00000067208**	EVI5	824.0512102	893.317038	934.7397359	1088.403749	1094.532134	1108.553483	1287.978508	1108.095528	1187.881839	1275.679546	993.3151553	1494.573158
**ENSG00000067221**	STOML1	95.62368992	108.5993262	106.6645517	57.76912205	24.27156791	39.07632059	48.00554314	11.62918845	24.38604644	41.76331846	22.07367012	27.06640694
**ENSG00000067225**	PKM	18160.0637	22819.87131	16847.29954	18166.2958	22055.9205	17936.41053	8996.443063	14828.87659	10315.7666	8829.145188	13906.41217	10378.27541
**ENSG00000067248**	DHX29	936.5496689	952.8715072	956.7240329	1109.33459	972.0185055	961.3533629	1206.266946	1157.934907	1200.074862	1232.650672	1042.980913	1371.082676
**ENSG00000067334**	DNTTIP2	2446.841477	2326.127503	2737.452084	2641.472175	1897.805453	2223.935838	1842.595741	1732.749079	1666.692481	1900.23099	1783.828466	2084.959159
**ENSG00000067365**	METTL22	503.4306028	437.9005088	525.9946598	450.4317052	441.5113782	476.5034822	420.8145484	515.0069172	458.1762955	497.3631562	550.4621485	444.0582388
**ENSG00000067369**	TP53BP1	1591.853191	1408.288036	1519.359187	1682.002409	1772.980247	1455.308406	1534.134591	1297.485169	1452.37665	1428.811713	1476.176689	1587.613932
**ENSG00000067445**	TRO	0	0	7.328098975	3.348934611	2.311577897	3.793817533	0	0	0.468962431	0	0	0
**ENSG00000067533**	RRP15	804.3639799	539.4934268	651.3865756	1005.517617	811.3638417	938.2110759	653.6925023	451.877037	612.9338979	662.5180973	601.5075107	810.3005577
**ENSG00000067560**	RHOA	4270.72274	4578.68772	4124.90549	4495.107482	4415.113782	4703.574977	4539.077313	5043.745163	5138.421361	4616.112245	5014.86193	5484.330706
**ENSG00000067596**	DHX8	1482.167194	1348.733567	1697.676263	1531.300351	1429.710929	1509.180615	1574.990373	1294.162544	1600.099816	1480.066695	1483.074711	1503.03141
**ENSG00000067601**	PMS2P4	63.28038304	59.5544692	79.79485551	45.21061725	60.10102531	49.31962793	110.3106098	114.6305719	82.53738794	91.11996754	106.2295374	93.04077385
**ENSG00000067606**	PRKCZ	60.46792157	140.1281628	89.56565414	51.07125282	63.56839215	52.73406371	59.24088302	79.74300653	90.50974927	60.11386748	111.747955	62.59106604
**ENSG00000067704**	IARS2	2466.528708	2129.948075	2432.92886	2619.7041	2747.31033	2679.952705	2210.297773	2113.189673	2235.543911	2328.621393	2326.012989	2476.576235
**ENSG00000067715**	SYT1	163.1227652	245.2242849	227.9853014	240.2860584	180.3030759	179.0681876	149.1236021	99.67875817	141.6266543	161.3582759	104.8499331	98.96155036
**ENSG00000067798**	NAV3	73.12399818	140.1281628	170.9889761	35.16381342	33.5178795	83.08460397	20.4278907	21.59706427	32.35840777	20.88165923	23.4532745	37.21630954
**ENSG00000067829**	IDH3G	320.6206074	350.320407	294.7524254	313.1253862	325.9324834	375.9673175	284.9690752	383.7632189	317.4875661	309.4282231	333.8642605	299.4221267
**ENSG00000067836**	ROGDI	28.12461468	38.53524477	31.75509556	39.34998168	31.2063016	43.24951988	44.94135953	19.93575163	24.38604644	25.94387965	56.56377967	32.98718345
**ENSG00000067900**	ROCK1	1860.443261	2193.005748	2174.002696	2118.201142	2548.514631	2333.956546	2164.335019	2149.738551	1923.683894	2133.093129	2018.361211	2236.361873
**ENSG00000067955**	CBFB	1396.387119	1537.906587	1378.496841	1090.078216	1047.144787	1320.248501	771.1528738	787.4621895	1007.331303	772.6213915	881.5672003	983.6947271
**ENSG00000067992**	PDK3	15.46853808	157.6441832	21.98429693	20.93084132	61.25681426	29.59177676	92.94690267	210.9867048	90.9787117	65.80886545	249.7083932	93.04077385
**ENSG00000068001**	HYAL2	137.8106119	101.592918	94.45105346	120.561646	142.1620406	174.1362248	79.66877372	86.38825708	132.2474057	124.6571778	86.91507609	144.6361121
**ENSG00000068024**	HDAC4	137.8106119	101.592918	137.6054141	133.9573845	154.8757191	152.5114648	200.1933288	152.8407625	234.9501782	244.2521352	177.9689653	224.9895077
**ENSG00000068028**	RASSF1	136.4043812	119.1089384	185.645174	152.3765248	160.6546638	207.5218191	166.4873092	204.3414542	196.4952588	148.7027248	235.9123494	183.544072
**ENSG00000068078**	FGFR3	7.031153671	0	4.885399317	6.697869223	26.58314581	7.967016819	13.27812895	14.95181373	18.28953483	14.5538837	16.55525259	10.99572782
**ENSG00000068079**	IFI35	1529.979039	1720.073199	1679.763132	174.1445998	257.7409355	188.5527314	577.0879122	632.9601144	681.8713753	122.7588452	102.0907243	136.1778599
**ENSG00000068097**	HEATR6	510.4617565	413.3780803	622.8884129	485.5955187	391.8124535	462.4663573	551.5530488	546.5718573	567.4445421	547.9853604	554.6009617	561.6279439
**ENSG00000068120**	COASY	798.739057	714.6536304	817.4901523	686.5315954	791.7154296	890.7883568	827.3295732	770.8490632	801.4567954	774.5197241	746.3659708	780.6966751
**ENSG00000068137**	PLEKHH3	67.49907524	94.5865099	56.99632536	115.5382441	94.77469376	109.6413267	152.1877857	88.04956971	123.8060819	143.6405044	154.5156908	117.5697051
**ENSG00000068305**	MEF2A	884.5191318	1334.720751	972.194464	884.9559711	1150.010004	829.3285127	455.5419626	1039.98171	535.5550967	556.2114685	896.7428485	521.0283335
**ENSG00000068308**	OTUD5	954.8306685	1033.445201	939.6251352	799.5581385	980.1090281	858.9202895	792.6021591	1005.094145	797.7050959	808.6897119	1015.388825	775.6217238
**ENSG00000068323**	TFE3	797.3328263	931.8522827	991.7360613	872.3974663	887.6459123	1012.190518	1144.983274	1162.918845	1165.840605	1044.715739	1276.134054	1012.452784
**ENSG00000068354**	TBC1D25	71.71776744	108.5993262	63.51019112	88.7467672	84.37259322	110.0207085	86.81853546	106.3240087	98.95107304	115.1655145	102.0907243	97.26989993
**ENSG00000068366**	ACSL4	36.56199909	154.1409791	111.5499511	25.95424324	87.83996007	97.5011106	271.6909463	347.214341	463.3348823	314.4904435	418.0201278	551.4780413
**ENSG00000068383**	INPP5A	122.3420739	105.0961221	126.206149	185.8658709	181.4588649	252.2888659	185.8938053	201.018829	179.6126112	206.2854821	190.3854048	201.3064016
**ENSG00000068394**	GPKOW	530.1489868	571.0222635	596.8329499	554.2486782	567.4923736	568.3138665	387.1085287	505.0390414	467.0865817	394.8531927	434.5753804	438.9832875
**ENSG00000068400**	GRIPAP1	1060.297974	1170.07016	1081.301715	787.8368673	686.5386353	785.3202293	855.9286202	862.2212581	746.5881909	697.3208627	667.728521	603.9192048
**ENSG00000068438**	FTSJ1	596.2418313	634.0799367	627.7738122	689.88053	690.0060021	738.6562737	394.2582905	443.5704738	379.390607	340.4343232	391.8076446	390.7712502
**ENSG00000068489**	PRR11	1975.754181	1191.089384	1924.033098	2866.688027	2777.360843	2749.758948	1350.283575	1206.112974	1515.686578	1475.637252	1401.678052	1912.410815
**ENSG00000068615**	REEP1	0	0	0	1.674467306	8.090522638	4.55258104	6.128367209	4.983937908	2.813774589	10.12444084	1.379604382	14.37902869
**ENSG00000068650**	ATP11A	4201.817434	4596.20374	4546.678297	6071.618451	6434.277075	7018.941818	4315.39191	5253.070555	4453.736212	5041.33876	5861.93902	5229.737315
**ENSG00000068654**	POLR1A	1740.913649	1068.477241	1661.035768	1959.126748	1530.264568	1879.457206	1564.776427	983.4970806	1703.271551	1706.601059	1174.043329	1755.93315
**ENSG00000068697**	LAPTM4A	2019.347334	2203.51536	1932.989663	1582.371604	1619.260317	1671.935387	3345.067102	3151.510071	3534.569846	2891.160637	2868.197511	3232.743979
**ENSG00000068724**	TTC7A	1143.265587	1219.115017	1231.934861	989.6101777	990.5111287	1253.477313	1367.647282	1380.550801	1467.85241	1399.071168	1302.346537	1231.521516
**ENSG00000068745**	IP6K2	357.1826065	360.8300193	383.5038464	419.4540601	379.098775	383.5549526	697.6124673	832.3176307	666.3956151	748.5758445	768.4396409	640.2896891
**ENSG00000068784**	SRBD1	510.4617565	413.3780803	462.4844686	512.3869956	396.4356093	420.3549827	498.440533	554.8784205	437.5419485	496.7303786	630.4792027	517.6450327
**ENSG00000068796**	KIF2A	3888.22798	3734.415539	3273.217542	3376.563322	3165.705929	2889.371433	3066.226394	2776.053415	2961.028792	2736.130136	2472.251053	2754.852731
**ENSG00000068831**	RASGRP2	4.218692202	0	0.814233219	0.837233653	2.311577897	2.27629052	1.021394535	0	4.220661883	3.163887762	1.379604382	2.53747565

**ENSG00000068878**	PSME4	3119.019768	2679.951114	3152.711026	3129.579394	3132.18805	3209.569633	2130.629	2119.834924	2245.392122	2365.322491	2272.208418	2466.426332
**ENSG00000068885**	IFT80	337.4953762	266.2435094	256.4834641	346.6147323	478.4966246	297.0559128	370.7662162	333.9238399	353.1287109	427.7576254	413.8813147	407.6877545
**ENSG00000068903**	SIRT2	57.6554601	129.6185506	74.90945619	108.8403749	161.8104528	112.6763807	123.5887387	132.9050109	130.3715559	146.8043922	148.9972733	136.1778599
**ENSG00000068912**	ERLEC1	1132.015741	1229.624629	1262.06149	916.7708499	1057.546888	1067.200872	634.2860062	644.5893028	725.015919	607.4664503	607.0259282	762.0885203
**ENSG00000068971**	PPP2R5B	184.2162262	210.1922442	236.1276336	210.9828805	283.1682923	277.7074434	100.0966644	201.018829	123.8060819	87.32330223	246.9491844	126.0279573
**ENSG00000068976**	PYGM	11.24984587	10.50961221	4.071166097	2.511700959	17.33683422	2.27629052	2.04278907	13.29050109	3.28273702	1.265555105	16.55525259	6.766601734
**ENSG00000068985**	PAGE1	1290.919814	1131.534915	1069.088217	1098.450553	1252.87522	1104.759666	251.2630556	284.0844608	344.6873871	313.857666	336.6234693	367.0881441
**ENSG00000069011**	PITX1	179.997534	122.6121425	131.9057816	146.5158893	108.6441611	207.9012008	108.2678207	81.40431917	140.6887294	126.5555105	70.3598235	126.8737825
**ENSG00000069020**	MAST4	6094.604002	6677.106958	6687.297431	3677.967437	2764.647164	4010.823896	4381.782555	5689.995779	4397.929682	3484.705981	5176.275642	3478.033291
**ENSG00000069122**	ADGRF5	0	0	3.256932878	15.07020575	5.778944741	17.83094241	12.25673442	18.274439	37.51699452	43.66165111	20.69406573	64.28271647
**ENSG00000069188**	SDK2	25.31215321	14.01281628	34.19779522	16.74467306	28.89472371	25.79795922	5.106972674	29.90362745	0.468962431	2.53111021	4.138813147	1.691650434
**ENSG00000069248**	NUP133	1210.764662	1313.701526	1071.530917	1473.531229	1415.841462	1223.885536	1578.054556	1510.133186	1484.735058	1554.734446	1553.434534	1696.725385
**ENSG00000069275**	NUCKS1	11325.78233	8197.497525	8590.974698	12194.30815	11772.86623	10761.16343	13732.64952	11463.05719	13704.48913	13654.7068	11871.49571	14362.95801
**ENSG00000069329**	VPS35	2410.279478	2490.778094	2374.304068	2309.090415	2726.506129	2400.727735	2266.474473	2580.018524	2290.881478	2158.404231	2367.40112	2281.19061
**ENSG00000069345**	DNAJA2	1881.536722	1667.525138	1955.788193	1568.138632	1551.068769	1591.127073	1265.507829	1119.724717	1257.288279	1123.812933	976.7599027	1338.941318
**ENSG00000069399**	BCL3	109.6859973	73.56728548	82.23755516	59.44358935	87.83996007	81.94645871	59.24088302	48.17806645	67.53059013	54.4188695	108.9887462	44.82873649
**ENSG00000069424**	KCNAB2	191.2473798	101.592918	179.1313083	153.2137585	165.2778196	168.8248802	216.5356414	156.1633878	213.3779063	235.3932495	160.0341083	208.0730033
**ENSG00000069482**	GAL	4.218692202	10.50961221	8.142332194	15.07020575	16.18104528	10.62268909	1.021394535	0	0.937924863	1.898332657	0	0.845825217
**ENSG00000069493**	CLEC2D	106.8735358	122.6121425	75.72368941	71.16486049	121.3578396	79.67016819	252.2844501	254.1808333	198.3711085	189.8332657	224.8755143	192.0023242
**ENSG00000069509**	FUNDC1	140.6230734	210.1922442	155.5185449	135.6318518	175.6799201	162.3753904	135.8454731	157.8247004	137.8749548	129.7193982	117.2663725	123.4904816
**ENSG00000069667**	RORA	15.46853808	297.772346	21.17006371	11.72127114	153.7199301	22.7629052	67.4120393	478.4580392	43.14454369	99.97885328	285.5781071	60.89941561
**ENSG00000069696**	DRD4	0	0	4.885399317	0.837233653	6.93473369	3.41443578	6.128367209	9.967875817	7.503398903	8.226108181	8.277626294	9.304077385
**ENSG00000069702**	TGFBR3	845.1446712	283.7595297	568.3347872	1294.363227	591.7639415	957.9389271	4987.469514	4128.361901	6394.771715	4721.153318	3789.773238	6401.205241
**ENSG00000069812**	HES2	402.18199	224.2050605	211.7006371	468.8508456	146.7851964	259.876501	237.9849266	104.6626961	285.5981208	261.9699067	106.2295374	213.1479546
**ENSG00000069849**	ATP1B3	1979.972874	1618.480281	1663.478467	2185.179834	1925.544388	1991.374823	1833.40319	1809.169461	2081.255271	2171.69256	1989.389519	2726.094674
**ENSG00000069869**	NEDD4	2327.311865	2245.553809	2393.031432	2293.182975	2126.651665	2203.828605	2006.018866	1255.952353	1956.980226	1898.965435	1247.162362	2059.584403
**ENSG00000069943**	PIGB	194.0598413	255.7338971	164.4751103	176.6563008	242.7156791	200.3135657	197.1291452	141.2115741	174.9229869	199.9577066	234.532745	217.3770807
**ENSG00000069956**	MAPK6	3014.958694	2883.13695	2422.343828	2541.004136	2496.504128	1998.583076	1216.480891	1390.518676	1105.813413	1058.004068	1073.332209	1228.98404
**ENSG00000069966**	GNB5	313.5894537	392.3588559	351.7487508	266.2403016	435.7324335	358.1363751	136.8668677	207.6640795	155.6955272	160.7254983	255.2268107	153.9401895
**ENSG00000069974**	RAB27A	478.1184496	718.1568344	559.3782218	380.9413121	439.1998003	390.7632059	297.2258096	458.5222876	333.9012512	384.7287518	405.6036884	356.0924163
**ENSG00000069998**	HDHD5	421.8692202	420.3844885	379.4326803	486.4327523	565.1807957	503.8189684	252.2844501	342.230403	280.9084964	333.4737701	383.5300183	269.8182442
**ENSG00000070010**	UFD1	3014.958694	3408.617561	3568.784201	2791.336999	2987.714431	2983.458108	2115.308082	2671.390719	2090.165557	2234.970315	2425.344504	2299.798764
**ENSG00000070018**	LRP6	289.6835312	248.727489	258.1119306	417.7795928	440.3555893	404.8003308	431.0284937	397.05372	442.7005353	519.5103705	551.8417529	575.1611474
**ENSG00000070047**	PHRF1	780.4580575	770.7048955	874.4864777	760.2081568	844.8817212	935.5554036	832.4365459	885.4796351	918.2284408	1004.850753	1011.250012	955.782495
**ENSG00000070061**	ELP1	3487.452221	3948.110987	3378.253627	3254.327209	3629.177298	2991.425125	2475.860353	2417.209886	2345.28112	2326.72306	2583.999008	2357.314879
**ENSG00000070081**	NUCB2	1172.796432	1289.179098	1057.688952	961.1442335	956.9932492	912.4131167	898.8271907	1005.094145	750.3398903	895.3802366	983.6579246	937.1743402
**ENSG00000070087**	PFN2	946.3932841	1064.974037	1006.392259	1121.055861	1326.845713	1174.565908	239.0063212	219.293268	189.9297847	245.5176903	246.9491844	206.3813529
**ENSG00000070159**	PTPN3	2892.61662	2672.944706	3033.832976	3342.236742	3076.71018	3368.530588	3578.96645	2457.081389	3179.096323	3658.08703	2742.653512	3279.264365
**ENSG00000070182**	SPTB	16.87476881	21.01922442	13.84196473	21.76807497	56.63365846	68.66809735	7.149761744	1.661312636	5.158586746	0	2.759208765	0
**ENSG00000070190**	DAPP1	1566.541038	2073.89681	1779.913818	1223.198367	914.2290581	1458.722841	496.3977439	651.2345534	411.2800524	208.8165923	244.1899757	300.267952
**ENSG00000070214**	SLC44A1	3265.267765	3240.463765	3171.43839	3125.393226	3382.994252	2821.841481	4323.563066	4517.109058	4714.010361	3999.154131	4354.031431	4331.470935
**ENSG00000070269**	TMEM260	322.0268381	322.2947745	314.2940227	293.8690122	324.7766945	295.1590041	607.7297483	563.1849836	569.3203918	667.5803178	589.0910712	648.7479413
**ENSG00000070366**	SMG6	614.5228308	721.6600385	653.8292752	674.8103242	709.6544142	635.085055	655.7352914	750.9133115	592.2995509	675.8064259	703.598235	652.1312421
**ENSG00000070367**	EXOC5	1307.794583	1191.089384	1247.405292	1404.87807	1489.811954	1375.638237	1750.670233	1892.235093	1703.740513	1690.148842	1883.159982	1952.1646
**ENSG00000070371**	CLTCL1	160.3103037	308.2819582	258.9261638	204.2850113	264.6756692	196.89913	150.1449966	179.4217647	103.1717349	160.7254983	227.6347231	112.4947538
**ENSG00000070404**	FSTL3	77.34269038	143.6313669	163.6608771	174.1445998	291.258815	338.7879057	248.198872	2734.520599	442.7005353	363.8470926	2532.953646	465.2038692
**ENSG00000070413**	DGCR2	1034.98582	1124.528507	1244.962593	1136.126067	1310.664667	1510.698142	1156.218613	1259.274978	1401.259745	1243.40789	1289.930097	1181.617828
**ENSG00000070423**	RNF126	279.8399161	185.6698157	180.7597747	265.403068	204.5746438	261.0146463	183.8510163	154.5020752	199.3090334	212.6132576	183.4873828	186.9273729
**ENSG00000070444**	MNT	627.1789074	725.1632426	670.1139396	563.4582484	711.9659921	651.7778522	631.2218226	616.346988	646.699193	637.2069952	713.2554657	542.173964
**ENSG00000070476**	ZXDC	521.7116024	655.0991612	617.1887803	646.34438	570.9597404	604.355133	651.6497133	717.6870588	695.9402483	666.3147627	818.1053987	690.1933769
**ENSG00000070495**	JMJD6	258.7464551	308.2819582	369.6618816	302.2413487	382.5661419	347.513686	203.2575124	358.8435294	261.2120743	158.1943881	360.0767438	298.5763015
**ENSG00000070501**	POLB	406.4006822	616.5639164	416.8874084	362.5221717	431.1092777	407.8353848	162.401731	134.5663235	141.6266543	112.6344043	122.78479	150.5568886
**ENSG00000070526**	ST6GALNAC	0	3.50320407	2.442699658	2.511700959	4.623155793	0.758763507	1.021394535	0	0	1.898332657	2.759208765	2.53747565
**ENSG00000070540**	WIPI1	194.0598413	259.2371012	271.1396621	128.0967489	253.1177797	189.6908767	243.0918993	244.2129575	173.9850621	201.8560392	224.8755143	197.9231007
**ENSG00000070601**	FRMPD1	0	0	7.328098975	0	1.155788948	0	12.25673442	6.645250545	0.937924863	22.14721433	2.759208765	6.766601734
**ENSG00000070610**	GBA2	359.9950679	315.2883663	388.3892457	343.2657977	343.2693176	314.8868552	325.8248566	318.9720261	302.4807683	344.2309885	336.6234693	356.0924163
**ENSG00000070614**	NDST1	1347.169043	1005.419568	1441.192798	1911.404429	1922.077021	2149.197632	1004.030828	1486.874809	933.704201	1246.571778	1847.290268	1151.16812
**ENSG00000070669**	ASNS	57.6554601	70.06408141	103.4076189	22.60530863	13.86946738	51.9753002	131.759895	56.48462963	133.1853305	79.09719405	31.73090079	43.13708606
**ENSG00000070718**	AP3M2	364.2137601	395.86206	461.6702354	419.4540601	392.9682424	353.5837941	191.000778	166.1312636	181.9574234	186.669378	205.561053	202.998052
**ENSG00000070729**	CNGB1	4.218692202	3.50320407	4.885399317	2.511700959	1.155788948	1.896908767	4.08557814	0	1.406887294	2.53111021	1.379604382	1.691650434
**ENSG00000070731**	ST6GALNAC	437.3377583	497.454978	409.5593094	257.8679651	213.8209554	208.2805826	491.2907713	470.151476	431.9143994	395.4859702	509.0740171	346.7883389
**ENSG00000070748**	CHAT	0	0	0	2.511700959	8.090522638	0.758763507	0	0	0	0.632777552	2.759208765	1.691650434
**ENSG00000070756**	PABPC1	11199.22157	9994.641213	10795.91826	11905.46254	12291.81546	13123.19423	7325.441604	7238.339156	7562.019207	7047.876378	7227.747359	7724.07588
**ENSG00000070759**	TESK2	42.18692202	101.592918	90.37988736	68.65315954	79.74943743	59.56293527	123.5887387	167.7925762	115.3647581	129.7193982	129.6828119	93.88659906
**ENSG00000070761**	CFAP20	341.7140684	423.8876925	414.4447087	337.4051621	337.4903729	385.4518614	236.9635321	215.9706427	272.9361351	216.4099229	215.2182836	241.0601868
**ENSG00000070770**	CSNK2A2	1376.699889	1268.159873	1420.836968	1553.90566	1415.841462	1638.549793	1330.877079	1348.985861	1358.584164	1342.753966	1350.63269	1494.573158
**ENSG00000070778**	PTPN21	416.2442973	311.7851623	558.5639885	470.5253129	365.2293077	529.6169276	443.2852281	543.249232	421.5972259	476.4814969	473.2043031	443.2124136

**ENSG00000070785**	EIF2B3	499.2119106	395.86206	515.4096279	756.0219885	646.0860221	654.8129062	400.3866577	267.4713344	462.8659198	437.2492887	358.6971394	482.9661988
**ENSG00000070808**	CAMK2A	52.03053716	14.01281628	27.68392946	61.95529031	49.69892478	39.07632059	119.5031606	69.77513072	92.385599	51.8877593	16.55525259	60.89941561
**ENSG00000070814**	TCOF1	2043.253257	1509.880954	2198.429693	2134.945815	1905.895976	2279.704956	1845.659925	1382.212113	2095.324144	1965.407078	1724.505478	2157.700128
**ENSG00000070831**	CDC42	4582.905963	4613.719761	4466.069209	4779.766924	4758.3831	4567.376928	4780.126423	5455.750697	5202.669215	5197.002038	5674.312824	5246.65382
**ENSG00000070882**	OSBPL3	2546.68386	2354.153135	2564.834641	2571.981782	2419.066269	2577.519632	2596.384908	2158.045114	2601.80357	2347.604719	1894.196817	2487.571963
**ENSG00000070915**	SLC12A3	0	0	0	1.674467306	0	0.379381753	20.4278907	73.09775599	55.80652934	21.51443678	93.813098	32.98718345
**ENSG00000070950**	RAD18	918.2686694	714.6536304	864.7156791	976.2144392	785.9364848	932.1409679	839.5863077	963.561329	789.7327346	908.0357877	933.9921668	891.4997785
**ENSG00000070961**	ATP2B1	645.459907	819.7497525	776.7784914	1109.33459	1160.412104	1110.829774	1426.888165	1151.289657	1410.638994	1689.516065	1356.151108	2011.372365
**ENSG00000070985**	TRPM5	1.406230734	0	0	6.697869223	6.93473369	1.896908767	0	0	0	0	13.79604382	0.845825217
**ENSG00000071051**	NCK2	298.1209156	269.7467134	367.219182	374.2434428	402.214554	494.3344246	613.8581155	639.6053649	646.2302305	532.7986991	549.0825442	647.9021161
**ENSG00000071054**	MAP4K4	3436.827914	3583.777764	3364.411663	3528.102613	3712.394102	3436.06054	1904.900808	2634.841841	2092.041407	1806.579912	2036.296068	2216.062068
**ENSG00000071073**	MGAT4A	208.1221487	269.7467134	253.2265312	362.5221717	420.7071772	405.938476	931.5118158	830.6563181	1006.393378	957.3924368	958.8250457	1098.726957
**ENSG00000071082**	RPL31	5035.712259	4907.988903	4603.674623	5570.115493	4995.319834	4921.719486	4537.034524	5311.216498	5063.387372	4609.151692	4963.816568	5083.409553
**ENSG00000071127**	WDR1	2314.655788	2284.089054	2785.491844	2500.816921	2306.954741	2984.216871	1717.985608	1845.718339	1857.091229	1757.856041	2000.426354	1859.969652
**ENSG00000071189**	SNX13	2593.089474	2207.018564	2350.691305	2551.05094	2393.638912	2429.94013	3039.670136	2907.297113	2889.27754	3238.555513	3021.333597	3234.435629
**ENSG00000071203**	MS4A12	0	0	0	0	0	0	16.34231256	4.983937908	4.689624315	4.429442867	5.518417529	3.383300867
**ENSG00000071205**	ARHGAP10	552.6486785	500.9581821	527.6231262	579.3656878	580.206052	570.9695387	437.1568609	526.6361057	376.5768325	422.0626274	571.1562143	427.1417345
**ENSG00000071242**	RPS6KA2	5.624922937	0	8.142332194	3.348934611	13.86946738	7.208253313	5.106972674	3.322625272	8.910286198	4.429442867	1.379604382	4.229126084
**ENSG00000071243**	ING3	354.370145	280.2563256	313.4797895	359.1732371	329.3998503	325.5095443	392.2155014	284.0844608	273.4050975	372.7059784	340.7622824	342.5592128
**ENSG00000071246**	VASH1	0	10.50961221	0	3.348934611	11.55788948	1.517527013	0	0	0	0	0	0.845825217
**ENSG00000071282**	LMCD1	109.6859973	66.56087734	66.76712399	200.9360767	217.2883223	129.3691779	15.32091802	3.322625272	5.158586746	3.163887762	2.759208765	10.99572782
**ENSG00000071462**	BUD23	552.6486785	409.8748762	636.7303776	628.7624733	539.7534388	656.3304332	492.3121658	495.0711656	572.6031288	564.4375767	473.2043031	521.0283335
**ENSG00000071537**	SEL1L	2022.159796	2059.883993	2342.548972	1342.922779	1612.325583	1817.997362	2582.085384	2820.908856	2353.722443	2365.322491	2741.273908	2429.210023
**ENSG00000071539**	TRIP13	1456.855041	1001.916364	1486.789859	1682.839642	1547.601402	1863.523172	874.3137219	729.3162473	1120.351249	1090.9085	800.1705417	1289.883456
**ENSG00000071553**	ATP6AP1	658.1159836	567.5190594	696.1694026	787.8368673	694.6291579	842.6068741	584.2376739	637.9440523	831.0014285	765.6608384	750.504784	846.671042
**ENSG00000071564**	TCF3	459.8374501	329.3011826	482.0260659	610.3433329	611.4123536	601.6994607	458.6061462	493.4098529	481.6244171	492.9337133	593.2298844	527.7949353
**ENSG00000071575**	TRIB2	794.5203648	644.589549	573.2201865	766.906026	804.429108	445.7735601	24.51346884	86.38825708	44.08246856	10.12444084	45.52694462	38.06213475
**ENSG00000071626**	DAZAP1	2096.690025	1551.919403	1963.930525	2258.856395	1895.493875	2149.956396	1895.708257	1262.597603	1816.291497	1936.29931	1433.408953	2003.759939
**ENSG00000071655**	MBD3	331.8704533	248.727489	298.8235915	493.1306215	513.170293	493.1962793	347.2741419	327.2785893	417.376564	522.6742583	524.2496653	442.3665884
**ENSG00000071794**	HLTF	1472.323579	1586.951444	1285.674254	1540.509921	1785.693925	1612.372452	1355.390548	1272.565479	1409.232107	1348.448964	1353.391899	1480.194129
**ENSG00000071859**	FAM50A	793.1141341	893.317038	801.2054879	720.0209415	811.3638417	776.2150673	559.7242051	451.877037	583.8582272	573.92924	525.6292697	566.7028952
**ENSG00000071889**	FAM3A	216.5595331	276.7531216	175.8743754	283.8222083	270.4546139	252.2888659	265.5625791	255.842146	321.708228	317.0215537	273.1616677	288.4263989
**ENSG00000071894**	CPSF1	1071.547819	809.2401403	955.9097996	1144.498403	1167.346838	1207.951503	700.6766509	632.9601144	740.9606417	730.2252954	756.0232015	661.4353195
**ENSG00000071909**	MYO3B	7.031153671	0	0.814233219	0	0	1.896908767	10.21394535	6.645250545	5.627549177	0	12.41643944	4.229126084
**ENSG00000071967**	CYBRD1	2.812461468	17.51602035	2.442699658	3.348934611	8.090522638	4.55258104	11.23533988	11.62918845	17.35160996	12.65555105	6.898021912	22.83728085
**ENSG00000071991**	CDH19	208.1221487	241.7210809	184.8309408	246.9839276	288.9472371	246.9775214	199.1719343	186.0670152	183.3643107	172.7482718	176.5893609	237.6768859
**ENSG00000071994**	PDCD2	1071.547819	987.9035479	1044.661221	1270.083451	1110.713179	1166.21951	983.6029371	1013.400708	1001.234791	961.8218796	931.2329581	1035.290065
**ENSG00000072042**	RDH11	641.2412148	465.9261414	542.2793242	650.5305483	564.0250068	553.1385963	394.2582905	574.8141721	408.4662778	408.7742988	549.0825442	488.8869753
**ENSG00000072062**	PRKACA	587.8044469	546.499835	603.3468156	727.5560443	895.7364349	772.4212497	613.8581155	699.4126198	656.0784416	718.8352995	838.7994644	765.4718212
**ENSG00000072071**	ADGRL1	773.4269038	550.0030391	522.7377269	1006.354851	952.3700934	767.8686687	475.9698533	350.5369662	378.4526822	583.4209033	527.008874	454.2081414
**ENSG00000072110**	ACTN1	4142.755743	3650.338641	4751.050835	4265.705461	3769.02776	5384.944606	3126.488671	3457.191596	4270.371901	2971.523386	2891.650785	3635.356782
**ENSG00000072121**	ZFYVE26	1175.608894	1390.772016	1410.251936	806.2560077	911.9174802	981.4605958	1332.919868	1621.441133	1312.156883	1247.204556	1469.278667	1161.318023
**ENSG00000072133**	RPS6KA6	57.6554601	56.05126513	30.94086234	89.58400086	31.2063016	31.10930377	174.6584655	179.4217647	149.5990156	246.7832454	150.3768777	152.248539
**ENSG00000072134**	EPN2	620.1477538	616.5639164	569.1490204	745.9751847	878.3996007	652.1572339	405.4936303	358.8435294	426.7558126	504.9564868	503.5555995	439.8291127
**ENSG00000072135**	PTPN18	549.8362171	402.8684681	494.2395642	590.2497253	657.6439116	602.4582242	655.7352914	518.3295425	615.2787101	638.4725504	640.1364334	588.6943509
**ENSG00000072163**	LIMS2	5.624922937	0	8.956565414	0.837233653	15.02525633	15.93403364	3.064183605	0	0	2.53111021	0	0
**ENSG00000072195**	SPEG	1.406230734	17.51602035	6.513865756	3.348934611	8.090522638	4.55258104	0	0	2.344812157	0	1.379604382	0.845825217
**ENSG00000072201**	LNX1	35.15576835	91.08330583	64.32442434	29.30317785	63.56839215	47.80210092	54.13391035	41.5328159	65.18577797	41.76331846	52.42496653	41.44543562
**ENSG00000072210**	ALDH3A2	2345.592865	1706.060382	1880.878737	2052.896917	2301.175796	1616.925033	1267.550618	895.4475109	1039.220748	1240.244003	1218.19067	1092.80618
**ENSG00000072274**	TFRC	7008.653979	7700.042547	6289.95162	12104.72415	12324.17756	11388.66085	10051.54362	9740.275986	11110.18896	11146.37659	10307.02434	13036.70407
**ENSG00000072310**	SREBF1	603.272985	728.6664466	517.0380943	675.6475579	780.1575401	609.6664776	406.5150249	840.6241939	488.1898911	399.2826356	876.0487828	432.2166858
**ENSG00000072364**	AFF4	6395.537379	6519.462775	6355.090278	5108.79975	5146.728187	5197.53002	5010.961588	5060.35829	4799.361523	4501.579508	4498.889891	4723.933836
**ENSG00000072401**	UBE2D1	246.0903785	234.7146727	243.4557326	121.3988797	168.7451864	156.3052824	212.4500633	192.7122658	170.702325	183.5054902	189.0058004	201.3064016
**ENSG00000072415**	MPP5	949.2057456	1047.458017	1043.846987	1177.150516	1064.481621	1107.035956	721.1045416	858.8986329	726.4228063	739.7169587	836.0402557	912.6454089
**ENSG00000072422**	RHOBTB1	0	0	2.442699658	1.674467306	0	0.758763507	10.21394535	9.967875817	9.379248629	8.858885733	6.898021912	18.60815477
**ENSG00000072501**	SMC1A	4530.875425	5387.92786	4757.564701	5142.289096	5511.957494	4873.538003	5838.291161	6460.844842	5602.694169	5808.265153	6663.489167	6102.628939
**ENSG00000072506**	HSD17B10	606.0854464	465.9261414	577.2913526	712.4858386	614.8797205	724.6191488	545.4246816	461.8449128	583.3892647	580.8897931	551.8417529	590.3860013
**ENSG00000072518**	MARK2	1209.358431	1128.031711	1350.812911	1265.897283	1062.170043	1287.621671	1156.218613	1054.933524	1260.571016	1120.649045	1153.349264	1159.626372
**ENSG00000072571**	HMMR	1687.476881	973.8907316	1485.161392	1495.299304	1789.161292	1448.858916	1839.531557	1368.921612	1599.630854	1682.555512	1374.085965	1806.682663
**ENSG00000072609**	CHFR	423.275451	539.4934268	523.5519601	466.3391446	442.6671672	503.4395866	963.1750464	1073.207963	896.1872065	914.9963407	939.5105844	821.2962855
**ENSG00000072657**	TRHDE	5.624922937	0	1.628466439	1.674467306	0	4.55258104	0	0	0	0	0	0
**ENSG00000072682**	P4HA2	369.8386831	1071.980446	506.4530625	354.9870688	811.3638417	612.3221498	524.9967909	1855.686215	516.327637	561.273689	1474.797085	691.0392021
**ENSG00000072694**	FCGR2B	0	0	0	0	0	0	21.44928523	46.51675381	15.94472267	6.327775524	35.86971394	3.383300867
**ENSG00000072736**	NFATC3	687.646829	500.9581821	498.3107303	658.0656512	614.8797205	627.1180382	557.681416	466.8288508	538.8378337	581.5225706	573.915423	603.0733796
**ENSG00000072756**	TRNT1	345.9327606	140.1281628	306.1516905	322.3349564	236.9367344	294.4002406	622.0292717	333.9238399	489.127816	497.9959337	322.8274255	487.1953249
**ENSG00000072778**	ACADVL	1799.97534	1877.717382	2152.018399	1815.122559	1959.062267	1985.304715	1370.711466	1111.418154	1503.024593	1495.886134	1212.672252	1512.335488
**ENSG00000072786**	STK10	1608.72796	1614.977076	1529.944219	1443.390818	1315.287823	1561.155915	762.9817176	624.6535512	937.4559005	695.4225301	659.4508947	836.5211394

**ENSG00000072803**	FBXW11	1372.481197	1355.739975	1367.097575	1355.481284	1385.790949	1604.405435	1144.983274	1360.615049	1354.832464	1291.498984	1233.366318	1425.21549
**ENSG00000072818**	ACAP1	2.812461468	3.50320407	6.513865756	5.023401917	13.86946738	4.55258104	2.04278907	38.21019063	1.406887294	0.632777552	24.83287888	0.845825217
**ENSG00000072832**	CRMP1	0	3.50320407	3.256932878	3.348934611	0	1.13814526	0	0	0	0	1.379604382	0
**ENSG00000072840**	EVC	1071.547819	1177.076568	1164.353504	778.6272972	1158.100526	839.9512018	103.160848	129.5823856	125.2129692	142.3749493	155.8952952	131.9487338
**ENSG00000072849**	DERL2	684.8343675	711.1504263	806.9051205	554.2486782	544.3765946	646.8458894	476.9912478	566.5076089	485.3761166	570.7653522	578.0542362	480.4287231
**ENSG00000072858**	SIDT1	0	0	10.58503185	0	0	0.379381753	2.04278907	4.983937908	8.910286198	1.898332657	4.138813147	0.845825217
**ENSG00000072864**	NDE1	527.3365253	557.0094472	570.7774868	609.5060993	668.0460121	625.2211294	780.3454246	888.8022603	796.2982086	766.2936159	1092.646671	822.1421107
**ENSG00000072952**	MRVI1	134.9981505	73.56728548	87.12295448	128.9339825	80.90522638	84.98151274	40.8557814	159.4860131	39.39284424	36.70109804	161.4137127	32.98718345
**ENSG00000072954**	TMEM38A	80.15515185	73.56728548	71.65252331	24.27977593	26.58314581	32.24744903	23.4920743	33.22625272	15.94472267	12.0227735	34.49010956	31.29553302
**ENSG00000072958**	AP1M1	295.3084542	283.7595297	409.5593094	335.7306948	295.8819708	398.7302227	329.9104348	294.0523366	349.8459739	322.7165517	299.374151	336.6384363
**ENSG00000073008**	PVR	763.5832886	686.6279978	936.3682024	648.0188473	524.7281825	718.5490408	512.7400565	495.0711656	469.4313939	508.1203746	423.5385454	456.7456171
**ENSG00000073050**	XRCC1	219.3719945	269.7467134	236.9418669	303.915816	282.0125034	273.5342441	247.1774774	305.681525	261.6810368	279.0549006	322.8274255	317.1844563
**ENSG00000073060**	SCARB1	269.996301	276.7531216	312.6655563	287.1711429	417.2398103	299.7115851	2014.190023	3791.115436	1991.683446	2278.631966	4060.175697	2156.008478
**ENSG00000073111**	MCM2	1455.44881	1467.842506	1555.185449	1532.137585	1746.397101	1632.100303	1920.221726	1882.267217	2137.0618	2097.024809	2052.851321	2002.068288
**ENSG00000073150**	PANX2	178.5913032	161.1473872	111.5499511	86.23506625	146.7851964	116.84958	107.2464262	117.9531972	54.86860448	87.32330223	92.43349361	74.43261908
**ENSG00000073169**	SELENOO	88.59253625	108.5993262	91.19412058	68.65315954	104.0210053	109.6413267	66.39064477	124.5984477	109.737209	117.0638472	96.57230676	127.7196077
**ENSG00000073282**	TP63	1861.849492	1292.682302	1560.070848	1308.596199	1301.418356	960.9739811	1950.863562	1417.099679	2036.70384	2023.622613	1546.536513	1950.47295
**ENSG00000073331**	ALPK1	1049.048128	1474.848914	1087.001348	311.4509189	406.8377098	369.8972095	732.3398815	691.1060566	576.8237907	422.695405	448.3714243	400.0753275
**ENSG00000073350**	LLGL2	805.7702107	802.2337321	859.0160465	878.2581019	840.2585654	1015.225572	446.3494117	445.2317865	687.4989245	535.3298093	473.2043031	559.0904683
**ENSG00000073417**	PDE8A	482.3371418	872.2978135	525.9946598	595.2731272	907.2943244	665.814977	481.0768259	553.2171078	340.9356877	492.9337133	529.7680828	372.1630954
**ENSG00000073464**	CLCN4	61.8741523	24.52242849	19.54159727	27.62871054	73.97049269	34.1443578	9.192550814	11.62918845	10.78613592	12.65555105	12.41643944	5.920776517
**ENSG00000073536**	NLE1	184.2162262	126.1153465	233.684934	243.634993	209.1977996	314.5074735	158.3161529	107.9853213	187.5849726	183.5054902	121.4051856	186.0815477
**ENSG00000073578**	SDHA	285.464839	364.3332233	316.7367224	319.8232554	278.5451365	275.8105347	369.7448216	505.0390414	307.1703926	341.6998783	488.3799513	317.1844563
**ENSG00000073584**	SMARCE1	416.2442973	273.2499175	319.179422	360.0104707	266.987247	319.818818	527.03958	478.4580392	467.0865817	494.832046	527.008874	513.4159066
**ENSG00000073605**	GSDMB	545.6175249	805.7369362	724.6675653	102.1425056	186.0820207	116.84958	357.4880872	473.4741013	363.9148468	163.8893861	240.0511625	146.3277625
**ENSG00000073614**	KDM5A	915.4562079	1012.425976	969.7517644	888.3049057	886.4901233	775.8356855	1653.637752	1574.924379	1500.679781	1578.779993	1678.978533	1603.684611
**ENSG00000073670**	ADAM11	0	0	2.442699658	0.837233653	3.467366845	1.517527013	12.25673442	0	1.406887294	2.53111021	6.898021912	1.691650434
**ENSG00000073711**	PPP2R3A	1217.795816	977.3939356	909.4985061	1352.132349	1356.896225	1243.613387	2257.281922	1388.857364	2136.123875	2205.22977	1386.502404	2274.424008
**ENSG00000073712**	FERMT2	1175.608894	1285.675894	1244.962593	1404.040836	1358.052014	1535.737337	1900.815229	2322.515065	1971.987024	1733.810494	1880.400773	1934.402271
**ENSG00000073734**	ABCB11	4.218692202	0	1.628466439	1.674467306	0	0	2.04278907	8.306563181	0.937924863	2.53111021	0	0.845825217
**ENSG00000073737**	DHRS9	0	0	0	0	0	1.896908767	7.149761744	4.983937908	21.10330942	12.0227735	15.17564821	18.60815477
**ENSG00000073754**	CD5L	150.4666886	112.1025303	135.9769476	2.511700959	5.778944741	3.793817533	8.171156279	0	2.813774589	1.265555105	0	0
**ENSG00000073756**	PTGS2	15844.00168	19127.49422	21025.13019	12466.40909	7423.632415	15913.92641	1158.261403	1638.054259	2150.192748	1063.699066	1238.884735	1935.248096
**ENSG00000073792**	IGF2BP2	3293.392379	3012.755501	3019.176778	2352.626565	2506.906229	2393.898863	796.6877372	1039.98171	788.3258473	604.9353401	611.1647414	567.5487205
**ENSG00000073803**	MAP3K13	305.1520693	339.8107948	513.7811615	195.0754411	230.0020007	279.9837339	219.599825	277.4392102	244.3294268	246.7832454	191.7650091	231.7561094
**ENSG00000073849**	ST6GAL1	23.90592248	17.51602035	19.54159727	6.697869223	8.090522638	12.14021611	0	0	0	3.796665314	0	0
**ENSG00000073905**	VDAC1P1	7.031153671	0	0	1.674467306	2.311577897	1.13814526	2.04278907	0	3.751699452	0.632777552	0	2.53747565
**ENSG00000073910**	FRY	2.812461468	0	4.885399317	5.86063557	6.93473369	8.346398573	3.064183605	9.967875817	4.689624315	3.796665314	9.657230676	4.229126084
**ENSG00000073921**	PICALM	6281.632689	8092.401403	7285.758848	5689.839905	6604.17805	6954.826302	3332.810367	4131.684526	3498.928701	3196.159417	3366.234693	3675.956392
**ENSG00000073969**	NSF	590.6169083	725.1632426	571.5917201	669.7869223	696.9407358	707.1675882	428.9857046	491.7485403	414.5627894	433.4526234	488.3799513	486.3494996
**ENSG00000074047**	GLI2	0	14.01281628	1.628466439	3.348934611	2.311577897	0.379381753	0	0	0	0	0	0
**ENSG00000074054**	CLASP1	1139.046895	1117.522098	1238.448727	1296.037695	1341.870969	1330.871191	1222.609258	1217.742162	1183.192215	1105.462384	1185.080164	1191.76773
**ENSG00000074071**	MRPS34	645.459907	532.4870187	691.2840033	733.4166799	787.0922738	818.7058236	533.1679472	524.974793	584.3271896	582.7881257	456.6490505	614.9149326
**ENSG00000074181**	NOTCH3	154.6853808	150.637775	65.95289078	396.8487515	276.2335586	184.3795321	520.9112128	433.602598	670.1473145	646.6986585	542.1845222	603.9192048
**ENSG00000074201**	CLNS1A	1340.13789	1092.99967	896.4707746	1141.149469	1139.607903	1023.57197	1139.876301	1078.191901	1107.689263	1095.337943	954.6862326	1183.309478
**ENSG00000074211**	PPP2R2C	195.466072	287.2627338	96.89375311	165.7722633	151.4083522	72.46191488	0	6.645250545	8.441323766	3.163887762	9.657230676	9.304077385
**ENSG00000074219**	TEAD2	28.12461468	35.0320407	14.65619795	42.6989163	48.54313583	28.07424974	6.128367209	1.661312636	19.69642212	6.327775524	8.277626294	18.60815477
**ENSG00000074266**	EED	809.9889029	1121.025303	993.3645277	1005.517617	825.2333091	937.4523124	738.4682487	852.2533823	804.2705699	776.4180568	808.448168	806.9172568
**ENSG00000074276**	CDHR2	5.624922937	10.50961221	7.328098975	9.209570182	16.18104528	12.89897961	3.064183605	8.306563181	1.406887294	3.163887762	19.31446135	0
**ENSG00000074319**	TSG101	1057.485512	1289.179098	1102.471779	839.7453538	857.5953996	924.5533328	1027.522902	1167.902783	1130.19946	995.9918674	1040.221704	1028.523464
**ENSG00000074356**	NCBP3	1056.079281	1142.044527	1064.202818	1183.848385	1015.938486	1051.266838	936.6187885	1074.869276	926.6697646	923.8552265	1040.221704	1062.356472
**ENSG00000074370**	ATP2A3	1.406230734	14.01281628	0	1.674467306	2.311577897	0.758763507	0	0	0	0	0	0
**ENSG00000074416**	MGLL	1514.510501	1159.560547	1687.905464	2125.736245	1848.106528	2619.251625	1428.930954	1003.432832	1997.779958	1649.018302	1153.349264	1849.819749
**ENSG00000074527**	NTN4	1144.671818	658.6023652	1313.358183	1095.101618	849.504877	1360.842349	1362.54031	825.6723802	1482.859208	1180.762913	718.7738832	1321.178989
**ENSG00000074582**	BCS1L	158.904073	129.6185506	184.8309408	223.5413853	167.5893975	182.4826233	122.5673442	116.2918845	138.3439173	140.4766166	102.0907243	126.8737825
**ENSG00000074590**	NUAK1	154.6853808	231.2114686	355.0056837	190.0520392	347.8924734	462.845739	237.9849266	350.5369662	260.2741495	235.3932495	241.4307669	321.4135824
**ENSG00000074603**	DPP8	919.6749001	882.8074257	831.3321171	889.979373	880.7111786	824.7759317	585.2590685	531.6200436	534.6171719	597.3420094	659.4508947	614.0691074
**ENSG00000074621**	SLC24A1	132.185689	136.6249587	111.5499511	108.8403749	109.7999501	97.88049235	153.2091802	103.0013834	120.0543825	118.3294023	106.2295374	96.42407471
**ENSG00000074657**	ZNF532	44.99938349	126.1153465	227.1710682	77.02549606	130.6041512	217.0063629	117.4603715	244.2129575	201.6538455	153.1321677	238.6715581	201.3064016
**ENSG00000074660**	SCARF1	11.24984587	3.50320407	4.071166097	0	0	0.379381753	1.021394535	4.983937908	3.751699452	0.632777552	0	0
**ENSG00000074695**	LMAN1	4272.12897	4645.248597	4078.494196	3429.309042	3762.093027	3898.147515	3577.945056	3932.32701	3916.305265	3691.624241	3462.807	4371.22472
**ENSG00000074696**	HACD3	1227.639431	1159.560547	1182.266635	1245.803675	1311.820456	1290.656725	676.1631821	490.0872277	701.098835	721.3664097	543.5641266	753.6302681
**ENSG00000074706**	IPCEF1	22.49969175	38.53524477	12.21349829	17.58190671	15.02525633	9.863925586	7.149761744	4.983937908	7.034436472	2.53111021	2.759208765	9.304077385
**ENSG00000074755**	ZZEF1	1167.171509	1261.153465	1213.207497	1047.3793	1056.391099	1159.390638	1628.102889	1702.845452	1609.010102	1846.444898	1496.870755	1492.035682
**ENSG00000074800**	ENO1	41341.77735	40546.08391	33995.86538	26438.16429	29342.01403	24031.93716	19623.0318	38381.30583	22440.79027	18479.00286	32336.54712	21034.82732
**ENSG00000074842**	MYDGF	1064.516666	1078.986854	1059.317419	707.4624367	750.1070274	888.1326845	314.5895167	337.2464651	407.5283529	360.0504273	344.9010956	416.9918319
**ENSG00000074855**	ANO8	109.6859973	178.6634076	131.0915483	164.097796	188.3935986	147.579502	188.957989	204.3414542	140.6887294	188.5677106	186.2465916	110.8031034

ENSG00000074935	TUBE1	494.9932184	248.727489	508.8957622	445.4083033	307.4398602	495.8519516	731.318487	481.7806645	603.0856868	460.6620581	375.252392	554.8613422
ENSG00000074964	ARHGEF10L	382.4947597	402.8684681	407.9308429	406.0583216	344.4251066	485.2292625	10.21394535	19.93575163	30.95152048	7.593330629	20.69406573	12.68737825
ENSG00000074966	TXK	16.87476881	66.56087734	54.5536257	24.27977593	31.2063016	27.31548624	8.171156279	3.322625272	8.910286198	5.062220419	5.518417529	8.458252168
ENSG00000075043	KCNQ2	21.09346101	21.01922442	11.39926507	25.95424324	15.02525633	11.00207085	0	1.661312636	0	0	0	0
ENSG00000075073	TACR2	1.406230734	3.50320407	0	0.837233653	0	1.13814526	0	6.645250545	0	0.632777552	0	0
ENSG00000075089	ACTR6	389.5259134	276.7531216	345.234885	358.3360034	317.8419608	292.12395	603.6441701	463.5062255	619.9683344	557.4770236	449.7510286	581.9277491
ENSG00000075131	TIPIN	226.4031482	150.637775	230.4280011	251.1700959	230.0020007	206.7630556	118.481766	83.06563181	136.4680676	139.8438391	92.43349361	149.7110634
ENSG00000075142	SRI	451.4000657	434.3973047	387.5750125	550.8997436	496.9892478	455.6374857	437.1568609	573.1528595	470.8382812	512.5498174	627.719994	532.8698866
ENSG00000075151	EIF4G3	3106.363692	2977.72346	3053.374573	3405.029266	3247.766945	3089.684999	2776.150346	2638.164466	2635.099902	2934.18951	3003.39874	2927.401075
ENSG00000075188	NUP37	556.8673707	574.5254675	554.4928224	688.2060627	625.281821	572.8664475	1135.790723	1098.127652	1047.193109	1056.105735	1081.609836	1092.80618
ENSG00000075213	SEMA3A	4315.722123	4421.043537	4319.507229	3869.693944	3033.945989	3083.614891	10.21394535	9.967875817	4.220661883	5.062220419	4.138813147	5.920776517
ENSG00000075218	GTSE1	507.649295	318.7915704	495.8680306	658.9028848	572.1155294	647.2252711	450.4349899	333.9238399	589.0168139	481.5437174	572.5358187	624.21901
ENSG00000075223	SEMA3C	3303.235995	2795.556848	2944.267322	3036.646459	2758.86822	3193.256218	1517.792279	1427.067554	1642.775397	1415.523385	1270.615636	1599.455485
ENSG00000075234	TTC38	101.2486129	87.58010176	75.72368941	123.9105806	112.111528	111.5382355	113.3747934	81.40431917	123.3371195	134.7816187	95.19270238	135.3320347
ENSG00000075239	ACAT1	648.2723684	602.5511001	542.2793242	806.2560077	814.8312085	802.77179	551.5530488	506.700354	552.9067067	630.2464422	506.3148083	704.5724056
ENSG00000075240	GRAMD4	171.5601496	115.6057343	127.0203822	179.1680017	168.7451864	187.4145861	118.481766	146.195512	91.44767413	123.3916227	110.3683506	89.65747298
ENSG00000075275	CELSR1	742.4898276	1050.961221	791.4346893	1116.869693	1421.620406	1107.79472	867.1639601	1606.489319	791.1396219	1071.925174	1942.48297	815.375509
ENSG00000075290	WNT8B	0	3.50320407	1.628466439	0	0	0	11.23533988	14.95181373	37.51699452	15.81943881	19.31446135	54.97863909
ENSG00000075292	ZNF638	3583.075911	4088.23915	3453.977317	3741.597195	3956.26557	3565.429718	4494.135953	4307.783665	3905.988092	4314.277352	4285.051211	4315.400256
ENSG00000075303	SLC25A40	511.8679872	458.9197332	465.7414015	609.5060993	508.5471372	489.7818435	492.3121658	475.1354139	525.2379232	593.5453441	558.7397748	536.2531874
ENSG00000075336	TIMM21	322.0268381	304.7787541	358.2626166	547.550809	468.094524	439.7034521	384.0443451	410.3442211	326.8668147	389.7909723	449.7510286	449.9790153
ENSG00000075340	ADD2	2.812461468	24.52242849	13.84196473	10.04680383	5.778944741	9.105162079	0	0	0	0.632777552	0	0
ENSG00000075391	RASAL2	3564.794911	2956.704235	3729.188145	3491.264332	3028.167044	3320.728487	2804.749393	2702.955659	2676.837559	2717.779587	2527.435228	2786.148264
ENSG00000075399	VPS9D1	80.15515185	63.05767327	62.6959579	48.55955187	61.25681426	49.31962793	80.69016826	46.51675381	74.5650266	79.7299716	68.98021912	75.27844429
ENSG00000075407	ZNF37A	651.0848299	658.6023652	689.6555369	623.7390714	800.9617411	577.7984103	890.6560344	990.1423311	815.9946307	837.1647018	859.4935302	843.2877411
ENSG00000075413	MARK3	2348.405326	2143.960891	2315.679276	2122.38731	1919.765443	1996.686168	2542.250997	2450.436138	2213.971639	2267.24197	2539.851668	2207.603816
ENSG00000075415	SLC25A3	3481.827298	3012.755501	3107.113965	3992.767291	3474.301578	3978.576447	4302.113781	4314.428916	4718.231023	4962.241566	4563.731297	5376.065078
ENSG00000075420	FNDC3B	3360.891455	4466.58519	4641.129351	3163.068741	3609.528885	4779.071946	4332.755617	4776.273829	4114.207411	4151.653521	4823.096921	4065.035992
ENSG00000075426	FOSL2	1822.475031	2946.194623	1773.399952	1844.425737	3206.158542	2123.399673	2500.373821	4802.854831	2597.113945	2653.869055	5097.638193	2655.891181
ENSG00000075461	CACNG4	262.9651473	206.6890402	127.8346155	509.8752946	484.2755693	412.3879658	422.8573374	613.0243627	509.762163	726.4286301	713.2554657	688.5017265
ENSG00000075539	FRYL	3009.333771	3100.335602	2790.377243	2762.871054	2712.636662	2747.103276	3425.75727	3718.01768	3247.564838	3351.822695	3828.402161	3473.804165
ENSG00000075568	TMEM131	2531.215321	2399.694788	2549.36421	2987.249673	2764.647164	2708.406337	2180.677332	2050.059793	2277.75053	1869.857667	2114.933518	2302.33624
ENSG00000075618	FSCN1	2690.119394	2326.127503	2292.880746	3359.818649	2954.196552	2920.480737	9.192550814	26.58100218	39.86180667	15.18666126	37.24931832	53.28698866
ENSG00000075624	ACTB	72229.63543	48431.79627	77727.51736	81698.09708	59822.48017	94062.77377	44516.45941	36206.64759	55978.63856	43414.23509	34901.23166	51910.83103
ENSG00000075643	MOCOS	168.7476881	94.5865099	100.150686	137.3063191	134.071518	90.67223904	267.6053681	151.1794499	213.3779063	239.1899148	155.8952952	170.0108686
ENSG00000075651	PLD1	286.8710698	308.2819582	424.2155073	279.6360401	394.1240314	396.4539322	795.6663427	888.8022603	688.4368494	930.183002	975.3802983	763.7801707
ENSG00000075673	ATP12A	74.53022891	80.57369362	62.6959579	118.8871787	75.12628164	123.6784516	11.23533988	0	2.344812157	6.327775524	1.379604382	6.766601734
ENSG00000075702	WDR62	397.9632978	374.8428355	437.2432388	514.8986965	503.9239814	558.0705591	366.680638	405.3602832	393.45948	369.5420906	358.6971394	414.4543562
ENSG00000075711	DLG1	2029.190949	2031.858361	2245.655219	2488.258416	2252.63266	2624.183588	1951.884956	1842.395713	1742.195433	1940.095976	1899.715234	2020.676443
ENSG00000075785	RAB7A	3531.045373	3871.040498	4058.952599	3611.825978	3595.659418	3870.073265	2679.117865	2658.100218	2696.533981	2602.614073	2771.625204	2722.711373
ENSG00000075790	BCAP29	43.59315276	42.03844885	48.03975995	61.95529031	38.14103529	44.00828338	70.47622291	71.43644335	77.37880119	52.52053685	51.04536215	64.28271647
ENSG00000075826	SEC31B	66.09284451	87.58010176	50.48245961	84.56059894	99.39784955	59.56293527	107.2464262	129.5823856	89.10286198	123.3916227	121.4051856	73.58679386
ENSG00000075856	SART3	1134.828202	1047.458017	1343.484812	1209.802628	1063.325832	1159.390638	1616.867549	1643.038197	1634.334074	1723.686053	1578.267413	1719.562666
ENSG00000075886	TUBA3D	4.218692202	0	0	0.837233653	1.155788948	0.758763507	1.021394535	4.983937908	0	0	0	0
ENSG00000075914	EXOSC7	223.5906867	154.1409791	215.7718032	246.9839276	171.0567643	196.89913	280.8834971	222.6158932	282.3153837	387.2598621	264.8840414	319.7219319
ENSG00000075945	KIFAP3	420.4629895	542.9966309	387.5750125	508.2008273	602.166042	482.1942085	659.8208695	613.0243627	622.3131465	673.2753157	681.5245649	672.4310473
ENSG00000075975	MKRN2	476.7122189	472.9325495	381.0611467	439.5476678	439.1998003	460.1900668	558.7028106	521.6521677	544.4653829	480.2781623	638.756829	554.015517
ENSG00000076003	MCM6	1061.704204	1324.211139	992.5502945	1090.91545	1293.327833	1184.429834	1296.149665	1247.64579	1295.743198	1345.917854	1400.298448	1425.21549
ENSG00000076043	REXO2	364.2137601	371.3396315	456.7848361	584.3890897	582.5176299	533.0313634	373.8303998	284.0844608	341.4046501	413.2037417	358.6971394	427.1417345
ENSG00000076053	RBM7	494.9932184	592.0414879	453.5279032	307.2647506	302.8167044	324.3713991	436.1354664	483.4419771	422.0661883	382.8304192	371.1135788	357.7840667
ENSG00000076067	RBMS2	2431.372939	2000.329524	2660.914161	2638.960474	2068.862217	2780.868252	1554.562482	1030.013834	1770.333179	1090.9085	718.7738832	1244.208894
ENSG00000076108	BAZ2A	2573.402244	2778.040828	2796.891109	1821.820429	2065.394851	2050.558377	2939.573471	2482.001078	2919.291136	2179.28589	2299.800505	2241.436824
ENSG00000076201	PTPN23	610.3041386	641.0863449	601.7183492	487.269986	485.4313583	605.4932783	589.3446466	634.621427	694.533361	585.319236	631.8588071	576.0069726
ENSG00000076242	MLH1	599.0542928	514.9709984	698.6121023	663.9262867	637.9954994	657.0891967	1137.833512	928.6737636	1013.427814	1030.161855	1100.924297	1054.744045
ENSG00000076248	UNG	419.0567588	497.454978	460.8560022	600.2965291	559.401851	545.1715795	1000.966644	1174.548034	1133.013234	1268.718993	1114.720341	1212.067536
ENSG00000076258	FMO4	50.62430643	119.1089384	56.99632536	17.58190671	26.58314581	27.31548624	4.08557814	11.62918845	15.00679781	5.694997971	4.138813147	20.2998052
ENSG00000076321	KLHL20	340.3078377	441.4037129	507.2672957	418.6168264	372.1640413	512.5447487	384.0443451	325.6172767	390.6457054	428.390403	437.3345892	368.7797945
ENSG00000076351	SLC46A1	402.18199	392.3588559	385.1323128	445.4083033	382.5661419	565.2788124	1029.565691	458.5222876	1235.247044	928.2846693	527.008874	1251.821321
ENSG00000076356	PLXNA2	2674.650856	2809.569664	3014.291378	2576.16795	2664.093526	2944.002406	3343.024313	3319.302647	4085.600703	2827.882882	2819.911357	2984.91719
ENSG00000076382	SPAG5	1143.265587	721.6600385	1143.18344	1558.091828	1576.496125	1625.271431	1046.929398	1054.933524	1075.330855	1264.922327	1096.785484	1432.827917
ENSG00000076513	ANKRD13A	3801.041674	2501.287706	3418.151055	2103.130936	1626.19505	2195.861588	3097.889624	2955.47518	2849.884696	2190.043109	2406.030043	2241.436824
ENSG00000076554	TPD52	1449.823887	1464.339301	1240.891426	1805.912989	1887.403353	1774.36846	1189.924633	1701.184139	1306.529334	1232.017894	1411.335283	1398.994909
ENSG00000076555	ACACB	39.37446056	59.5544692	42.34012741	22.60530863	45.07576898	33.38559429	73.54040651	78.0816939	83.4753128	111.3688492	194.5242179	71.04931821
ENSG00000076604	TRAF4	687.646829	686.6279978	795.5058554	890.8166067	566.3365846	1005.741028	994.838277	657.8798039	987.1659182	988.3985368	604.2667195	927.8702628
ENSG00000076641	PAG1	5773.983394	7279.658058	3857.022761	5841.379196	8514.697182	5131.517595	1133.747934	3603.387108	1279.798475	997.8902001	2779.90283	1206.992584
ENSG00000076650	GPATCH1	234.8405326	227.7082646	326.507521	262.891367	264.6756692	306.1610749	311.5253331	397.05372	331.0874766	337.2704354	335.2438649	259.6683416
ENSG00000076662	ICAM3	46.40561423	42.03844885	68.39559043	37.67551438	55.47786952	62.5979893	22.47067977	19.93575163	36.57906965	32.27165517	41.38813147	35.5246591

**ENSG00000076685**	NT5C2	2698.556779	2935.685011	3194.23692	2132.434114	1994.891725	2498.228846	1297.171059	1245.984477	1275.108851	1080.784059	1020.907243	1196.842682
**ENSG00000076706**	MCAM	9.843615139	0	4.071166097	5.86063557	3.467366845	4.931962793	1.021394535	4.983937908	0.468962431	3.163887762	0	0.845825217
**ENSG00000076716**	GPC4	4.218692202	0	4.885399317	0	0	0	0	4.983937908	0	0	0	0
**ENSG00000076770**	MBNL3	39.37446056	45.54165292	34.19779522	52.74572013	80.90522638	48.94024618	2782.278713	1538.375501	2058.745074	3158.825541	2085.961826	2386.918762
**ENSG00000076826**	CAMSAP3	157.4978422	161.1473872	205.1867713	246.9839276	228.8462118	278.4662069	135.8454731	169.4538889	190.3987472	156.928833	177.9689653	181.0065964
**ENSG00000076864**	RAP1GAP	53.4367679	150.637775	77.35215585	96.28187008	175.6799201	115.332053	14.29952349	4.983937908	6.56547404	14.5538837	5.518417529	9.304077385
**ENSG00000076924**	XAB2	489.3682955	539.4934268	539.8366245	453.7806399	486.5871472	554.2767416	530.1037636	501.7164161	557.1273686	502.4253766	525.6292697	531.1782361
**ENSG00000076928**	ARHGEF1	926.7060538	952.8715072	973.8229305	859.8389615	815.9869975	949.9719103	437.1568609	488.425915	558.5342559	466.9898337	554.6009617	487.1953249
**ENSG00000076944**	STXBP2	209.5283794	273.2499175	249.9695984	195.0754411	208.0420107	235.9754506	195.0863562	265.8100218	264.4948113	217.0427005	186.2465916	166.6275677
**ENSG00000076984**	MAP2K7	403.5882207	448.410121	458.4133025	497.3167898	490.0545141	515.5798027	487.2051931	388.7471569	474.5899806	438.5148438	522.8700609	433.9083362
**ENSG00000077044**	DGKD	541.3988327	332.8043867	566.7063207	672.2986233	767.4438616	830.466658	1112.298648	1013.400708	1082.834254	921.9568938	927.0941449	1046.285793
**ENSG00000077092**	RARB	4.218692202	0	2.442699658	2.511700959	1.155788948	7.587635066	0	1.661312636	1.875849726	0.632777552	1.379604382	5.074951301
**ENSG00000077097**	TOP2B	1676.227035	1944.278259	1791.313083	2181.830899	2209.868469	2003.135657	3024.349218	3020.266372	2855.981208	3770.088657	4050.518466	4007.519877
**ENSG00000077147**	TM9SF3	4611.030577	4585.694128	4378.132021	4835.024345	5136.326086	5377.356971	4841.410095	4610.142565	4628.190236	4776.204965	4451.983342	5442.039445
**ENSG00000077150**	NFKB2	1361.231351	2161.476911	2091.765141	882.4442701	1440.11303	1520.562067	478.0126423	774.1716884	543.9964205	375.8698661	507.6944127	416.9918319
**ENSG00000077152**	UBE2T	608.8979079	486.9453658	525.9946598	659.7401185	613.7239315	576.2808833	450.4349899	304.0202124	463.3348823	455.5998377	328.345843	514.2617318
**ENSG00000077157**	PPP1R12B	763.5832886	742.6792629	865.5299123	833.0474846	889.9574902	846.7800734	2008.061656	2648.132342	1713.588725	2397.594146	2624.007535	1755.93315
**ENSG00000077232**	DNAJC10	1681.851958	1940.775055	1955.788193	1497.811005	1627.350839	1800.166419	1304.320821	1171.225408	1292.460461	1257.961774	1063.674979	1364.316075
**ENSG00000077235**	GTF3C1	3269.486457	2679.951114	3701.504216	4001.139627	4036.015007	4457.35622	3110.146359	2987.04012	3135.482817	3358.783248	3322.087353	3358.771936
**ENSG00000077238**	IL4R	3695.574369	3674.86107	4220.170776	3900.671589	3081.333336	3772.951537	1680.19401	1676.26445	1883.353125	1676.860514	1688.635764	1660.354901
**ENSG00000077254**	USP33	2498.872015	2644.919073	2348.248605	1568.138632	1699.009754	1523.976503	2277.709813	2252.739935	2149.723786	1693.31273	1794.865301	1846.436448
**ENSG00000077264**	PAK3	4.218692202	0	0.814233219	0	0	0	0	3.322625272	3.28273702	0.632777552	2.759208765	0
**ENSG00000077274**	CAPN6	2.812461468	0	4.885399317	0	0	1.13814526	2274.645629	4043.634956	1733.285147	2338.745834	3580.073372	1865.044603
**ENSG00000077312**	SNRPA	680.6156753	613.0607123	714.8967667	678.1592588	730.4586153	784.5614658	591.3874357	553.2171078	712.3539334	611.2631156	554.6009617	730.7929873
**ENSG00000077348**	EXOSC5	116.7171509	56.05126513	65.95289078	116.3754777	110.955739	127.8516509	82.73295732	69.77513072	113.4889084	94.2838553	114.5071637	109.9572782
**ENSG00000077380**	DYNC1I2	1321.85689	1635.996301	1297.887752	1419.948275	1435.489874	1332.009336	1178.689293	1485.213497	1127.385685	1143.429037	1254.060384	1146.093169
**ENSG00000077420**	APBB1IP	111.092228	112.1025303	116.4353504	138.1435527	167.5893975	172.239316	224.7067977	387.0858442	195.0883715	168.9516065	362.8359525	178.4691207
**ENSG00000077454**	LRCH4	67.49907524	101.592918	67.58135721	72.00209415	84.37259322	88.39594852	116.438977	68.11381808	102.7027725	88.58885733	104.8499331	75.27844429
**ENSG00000077458**	FAM76B	490.7745262	521.9774065	452.71367	455.4551072	405.6819208	450.3261412	618.9650881	558.2010457	557.596331	553.6803583	564.2581924	542.173964
**ENSG00000077463**	SIRT6	99.84238213	150.637775	122.9492161	113.0265431	112.111528	145.3032115	105.2036371	111.3079466	133.1853305	109.4705166	151.7564821	137.8695103
**ENSG00000077514**	POLD3	786.0829804	917.8394665	824.0040181	920.1197845	925.7869476	831.2254215	1082.678207	1229.371351	1018.117439	1097.869053	1096.785484	1032.75259
**ENSG00000077522**	ACTN2	2.812461468	3.50320407	3.256932878	0	0	2.655672273	3.064183605	8.306563181	3.751699452	4.429442867	2.759208765	0
**ENSG00000077549**	CAPZB	2285.124943	1982.813504	2387.331799	2492.444585	2371.678922	2575.243341	2360.44277	2305.901939	2423.597846	2403.289144	2539.851668	2673.65351
**ENSG00000077585**	GPR137B	46.40561423	52.54806106	32.56932878	14.2329721	23.11577897	14.79588838	78.64737919	154.5020752	192.7435593	95.54941041	157.2748996	201.3064016
**ENSG00000077616**	NAALAD2	1.406230734	3.50320407	0	0.837233653	3.467366845	0.758763507	0	0	0	1.898332657	1.379604382	0
**ENSG00000077684**	JADE1	517.4929102	325.7979785	508.0815289	611.1805666	671.5133789	689.7160275	205.3003015	250.8582081	214.7847936	194.8954861	277.3004808	290.1180494
**ENSG00000077713**	SLC25A43	189.8411491	161.1473872	98.52221955	161.586095	164.1220307	143.4063027	251.2630556	227.5998311	242.9225395	330.9426599	245.5695801	357.7840667
**ENSG00000077721**	UBE2A	1006.861206	1068.477241	1100.029079	1051.565468	1078.351089	1163.943219	836.5221241	927.012451	918.2284408	927.0191142	873.289574	959.1657958
**ENSG00000077782**	FGFR1	12.65607661	21.01922442	66.76712399	36.00104707	46.23155793	94.84543833	0	0	0	1.265555105	0	0
**ENSG00000077935**	SMC1B	22.49969175	24.52242849	20.35583049	9.209570182	17.33683422	10.24330734	126.6529223	106.3240087	66.59266527	94.2838553	99.33151553	73.58679386
**ENSG00000077942**	FBLN1	1245.92043	1254.147057	1174.124302	1205.61646	1389.258316	1339.217589	3841.464846	3638.274673	5131.386925	3430.287111	3513.852362	4249.425889
**ENSG00000078018**	MAP2	1442.792733	1324.211139	1297.887752	854.8155596	977.7974502	775.8356855	21.44928523	14.95181373	27.66878346	22.14721433	26.21248326	11.84155303
**ENSG00000078043**	PIAS2	343.1202991	304.7787541	369.6618816	429.5008639	347.8924734	413.1467293	323.7820676	279.1005229	308.5772799	366.3782028	303.5129641	358.6298919
**ENSG00000078053**	AMPH	0	0	2.442699658	0	1.155788948	1.13814526	4.08557814	0	0	0	0	0
**ENSG00000078061**	ARAF	452.8062964	280.2563256	473.8837337	534.1550705	510.8587151	572.4870657	460.6489352	393.7310948	530.39651	517.6120378	567.0174011	510.8784309
**ENSG00000078070**	MCCC1	291.089762	252.2306931	288.2385597	368.3828073	395.2798203	344.0992502	463.7131188	441.9091612	392.5215551	456.2326153	536.6661047	448.2873649
**ENSG00000078081**	LAMP3	594.8356005	1254.147057	582.1767519	65.30422492	72.81470374	62.21860754	70.47622291	61.46856754	56.74445421	5.694997971	6.898021912	2.53747565
**ENSG00000078098**	FAP	19.68723028	63.05767327	52.11092604	1.674467306	12.71367843	13.65774312	12.25673442	1.661312636	7.972361335	21.51443678	5.518417529	13.53320347
**ENSG00000078114**	NEBL	209.5283794	143.6313669	96.07951989	210.1456469	246.183046	139.2331035	4065.150249	4321.074167	2329.336397	3005.060596	4133.294729	1789.766159
**ENSG00000078124**	ACER3	749.5209813	700.6408141	606.6037485	709.9741376	760.509128	740.9325642	401.4080522	407.0215959	316.0806788	320.1854415	361.4563482	378.0838719
**ENSG00000078140**	UBE2K	1555.291192	1317.20473	1415.951569	1614.186483	1425.087773	1654.863208	1431.995138	1390.518676	1372.184074	1457.919481	1303.726141	1627.367717
**ENSG00000078142**	PIK3C3	1660.758497	2014.34234	1637.423004	1401.529135	1679.361342	1381.328964	1472.850919	1643.038197	1386.252947	1473.738919	1670.700907	1418.448889
**ENSG00000078177**	N4BP2	426.0879124	581.5318757	532.5085255	677.3220252	584.8292078	566.0375759	898.8271907	875.5117592	719.3883698	819.4469303	1005.731595	856.8209446
**ENSG00000078237**	TIGAR	44.99938349	10.50961221	72.46675653	61.95529031	35.8294574	67.52995209	46.9841486	68.11381808	64.24785311	39.8649858	55.18417529	60.05359039
**ENSG00000078246**	TULP3	302.3396078	458.9197332	336.2783196	353.3126015	342.1135287	365.3446284	383.0229506	403.6989706	404.7145783	382.8304192	487.000347	370.4714449
**ENSG00000078269**	SYNJ2	1655.133574	2000.329524	1796.198482	1375.574892	1820.367594	1420.784666	956.0252846	1561.633878	847.8840761	828.3058161	1329.938625	788.309102
**ENSG00000078295**	ADCY2	0	0	0	0.837233653	0	0.758763507	3.064183605	4.983937908	0.937924863	0.632777552	2.759208765	0.845825217
**ENSG00000078304**	PPP2R5C	1442.792733	1541.409791	1389.896106	1656.048165	1774.136036	1634.376593	1992.740738	1888.912467	1850.525755	2111.578692	1989.389519	2226.211971
**ENSG00000078319**	PMS2P1	241.8716863	248.727489	276.0250614	308.9392179	308.5956492	256.0826835	378.9373724	290.7297113	346.5632368	375.8698661	333.8642605	304.497078
**ENSG00000078328**	RBFOX1	15.46853808	14.01281628	16.28466439	2.511700959	2.311577897	2.655672273	2.04278907	0	0.468962431	5.062220419	0	0.845825217
**ENSG00000078369**	GNB1	4440.876658	4410.533925	4762.450101	4537.806399	4715.618909	4843.187463	3282.762035	3116.622505	3328.226376	3080.993903	3151.016409	3279.264365
**ENSG00000078401**	EDN1	719.9901359	959.8779153	1273.460755	665.600754	538.5976499	968.9409979	72.51901198	171.1152015	106.9234344	74.66775118	96.57230676	67.66601734
**ENSG00000078403**	MLLT10	642.6474455	472.9325495	597.6471831	605.319931	678.4481126	638.1201091	975.4317808	848.9307571	908.3802297	898.5441244	953.3066282	884.7331767
**ENSG00000078487**	ZCWPW1	87.18630552	77.07048955	62.6959579	43.53614995	50.85471372	53.11344546	96.01108628	149.5181373	98.01314817	120.227735	100.7111199	73.58679386
**ENSG00000078549**	ADCYAP1R1	2.812461468	0	1.628466439	0	0	4.55258104	0	0	0	0	0	0
**ENSG00000078579**	FGF20	4.218692202	7.006408141	1.628466439	0.837233653	0	2.655672273	0	0	0	0	0	0
**ENSG00000078596**	ITM2A	0	0	0	0	4.623155793	0.379381753	3.064183605	1.661312636	2.813774589	3.163887762	0	3.383300867

**ENSG00000078618**	NRDC	6017.261311	5619.139329	5714.288734	5317.270929	5086.627161	4986.214384	4635.088399	4399.15586	4330.399092	4337.690122	4418.872837	4460.036368
**ENSG00000078668**	VDAC3	1486.385886	1485.358526	1482.718693	1898.008691	1742.929734	1764.504535	579.1307013	707.719183	616.6855974	562.5392441	746.3659708	720.6430847
**ENSG00000078674**	PCM1	4481.65735	4235.373721	4199.814946	3506.334538	3979.381349	3512.695654	2686.267627	2467.049265	2293.22629	2159.037009	2215.644638	2228.749446
**ENSG00000078687**	TNRC6C	71.71776744	49.04485699	71.65252331	65.30422492	86.68417112	65.63304332	46.9841486	73.09775599	53.46171719	37.96665314	40.00852709	47.36621214
**ENSG00000078699**	CBFA2T2	374.0573753	374.8428355	407.9308429	327.3583583	431.1092777	391.9013512	610.7939319	659.5411165	602.6167244	641.6364381	622.2015764	601.3817291
**ENSG00000078747**	ITCH	5710.703011	5699.713023	5759.071561	6568.098007	5502.711183	6446.454752	5013.004377	5590.317021	4496.880755	5141.950391	5881.253482	4962.456547
**ENSG00000078804**	TP53INP2	130.7794583	294.2691419	246.7126655	199.2616094	290.103026	276.5692982	105.2036371	367.1500926	103.1717349	175.9121596	404.224084	159.860966
**ENSG00000078808**	SDF4	1281.076199	1257.650261	1263.689957	1244.966442	1459.761442	1411.300122	1209.331129	1388.857364	1183.661177	1187.723466	1448.584601	1310.183261
**ENSG00000078814**	MYH7B	0	7.006408141	0	1.674467306	3.467366845	3.41443578	3.064183605	8.306563181	3.28273702	8.226108181	15.17564821	1.691650434
**ENSG00000078900**	TP73	260.1526858	332.8043867	254.0407645	240.2860584	352.5156292	297.8146763	227.7709813	333.9238399	282.3153837	325.8804395	324.2070298	297.7304763
**ENSG00000078902**	TOLLIP	282.6523776	245.2242849	300.452058	226.0530863	327.0882724	338.4085239	343.1885637	405.3602832	409.4042027	349.9259865	445.6122155	377.2380467
**ENSG00000078967**	UBE2D4	277.0274546	185.6698157	232.0564675	308.9392179	266.987247	341.8229597	153.2091802	176.0991394	142.5645792	182.2399351	154.5156908	170.0108686
**ENSG00000079101**	CLUL1	0	0	0	3.348934611	1.155788948	0	2.04278907	1.661312636	0.468962431	2.53111021	4.138813147	0
**ENSG00000079112**	CDH17	2.812461468	0	0	0	2.311577897	3.793817533	0	0	0	0	0	0
**ENSG00000079134**	THOC1	665.1471373	535.9902228	608.2322149	730.904979	622.9702431	598.6644067	706.8050181	702.7352451	676.7127886	795.4013833	802.9297505	726.5638612
**ENSG00000079150**	FKBP7	4.218692202	14.01281628	6.513865756	3.348934611	10.40210053	6.449489806	4.08557814	8.306563181	4.689624315	13.2883286	2.759208765	9.304077385
**ENSG00000079156**	OSBPL6	1.406230734	7.006408141	2.442699658	3.348934611	4.623155793	5.6907263	2.04278907	26.58100218	5.158586746	5.062220419	2.759208765	6.766601734
**ENSG00000079215**	SLC1A3	1420.293041	1460.836097	1205.879398	1525.439716	1462.07302	1182.532925	3744.432365	2927.232865	2930.077272	4278.209032	3531.787219	3703.868624
**ENSG00000079246**	XRCC5	5925.856314	5993.982164	5408.137044	6535.445894	6047.087777	5874.72645	5235.668386	5376.007691	5455.439965	5446.949171	5332.170938	5985.905059
**ENSG00000079257**	LXN	64.68661377	91.08330583	63.51019112	32.65211246	67.035759	36.80003007	80.69016826	79.74300653	41.26869397	44.29442867	63.46180159	67.66601734
**ENSG00000079263**	SP140	163.1227652	224.2050605	152.261612	0	8.090522638	4.931962793	36.77020326	24.91968954	27.66878346	6.960553076	11.03683506	10.99572782
**ENSG00000079277**	MKNK1	748.1147506	746.182467	866.3441455	864.0251298	862.2185554	805.0480805	823.2439951	915.3832625	779.8845235	835.8991467	961.5842545	721.4889099
**ENSG00000079308**	TNS1	25.31215321	17.51602035	17.09889761	9.209570182	5.778944741	4.55258104	142.9952349	201.018829	180.5505361	135.4143962	179.3485697	120.1071808
**ENSG00000079313**	REXO1	509.0555258	525.4806106	497.4964971	564.295482	537.4418609	605.1138965	408.557814	338.9077778	467.5555442	489.7698255	412.5017103	433.9083362
**ENSG00000079332**	SAR1A	3203.393612	2865.62093	2652.771829	2495.793519	2275.748439	2345.717381	2938.552077	3171.445822	2908.036037	2641.213504	2457.075405	2899.488843
**ENSG00000079335**	CDC14A	95.62368992	98.08971397	71.65252331	117.2127114	141.0062517	92.18976605	272.7123408	325.6172767	270.1223605	292.9760068	320.0682167	315.4928059
**ENSG00000079337**	RAPGEF3	686.2405983	507.9645902	740.1379965	432.8497985	396.4356093	569.07263	597.5158029	292.391024	452.5487464	480.9109398	182.1077785	304.497078
**ENSG00000079385**	CEACAM1	5856.951008	7749.087404	9445.919579	3228.372965	4234.810706	5998.02552	69.45482837	46.51675381	145.8473162	9.491663286	30.35129641	54.13281387
**ENSG00000079387**	SENP1	880.3004396	791.7241199	822.3755516	863.1878961	761.6649169	879.4069042	1135.790723	1099.788965	1124.571911	1092.806833	1030.564474	1146.938994
**ENSG00000079393**	DUSP13	0	0	0	2.511700959	0	0	4.08557814	0	0.937924863	0.632777552	0	0
**ENSG00000079432**	CIC	499.2119106	507.9645902	594.3902502	687.368829	777.8459622	737.1387467	624.0720608	742.6067483	643.416456	692.2586423	671.8673342	620.8357091
**ENSG00000079435**	LIPE	25.31215321	7.006408141	25.2412298	29.30317785	24.27156791	26.93610448	0	0	1.406887294	3.163887762	1.379604382	0.845825217
**ENSG00000079459**	FDFT1	3048.708232	2438.230033	2852.258968	3029.111356	2899.874471	3014.567412	934.5759994	1315.759608	1103.468601	936.5107775	1289.930097	1147.784819
**ENSG00000079462**	PAFAH1B3	205.3096872	164.6505913	237.7561001	277.9615728	324.7766945	342.961105	132.7812895	136.2276362	154.2886399	174.0138269	126.9236032	206.3813529
**ENSG00000079482**	OPHN1	1316.231967	1324.211139	1195.294366	1741.445998	1612.325583	1718.978724	1851.788292	1817.476024	1798.470925	1910.988208	2029.398046	2027.443045
**ENSG00000079557**	AFM	0	0	0	0	0	0.758763507	5.106972674	0	6.096511609	3.796665314	2.759208765	8.458252168
**ENSG00000079616**	KIF22	2209.188483	1765.614851	2226.927855	3211.628292	2879.07027	2967.903456	2908.931635	2729.536661	3134.075929	3046.191137	3149.636805	3165.077961
**ENSG00000079691**	CARMIL1	829.6761332	665.6087734	709.1971341	946.9112614	776.6901732	854.7470902	730.2970924	627.9761765	855.8564374	840.9613671	641.5160378	940.5576411
**ENSG00000079739**	PGM1	312.183223	655.0991612	219.8429693	375.9179101	647.241811	363.4477197	836.5221241	1888.912467	1019.055364	1076.987394	1607.239105	1107.185209
**ENSG00000079785**	DDX1	3795.416751	3212.438133	3326.956935	3718.154652	3423.446865	3495.244093	3595.308763	3925.681759	3573.96269	3740.348112	3706.996975	4125.935407
**ENSG00000079805**	DNM2	1701.539188	1516.887362	1798.641182	1764.051307	1867.75494	1939.778905	1459.57279	1611.473257	1525.065827	1512.33835	1721.746269	1565.622476
**ENSG00000079819**	EPB41L2	4966.806953	7430.295833	9361.239324	2713.474269	4934.06302	5554.52825	150.1449966	131.2436983	196.9642212	71.50386342	122.78479	159.860966
**ENSG00000079841**	RIMS1	2.812461468	7.006408141	0	0.837233653	0	3.41443578	0	0	1.406887294	0	1.379604382	0
**ENSG00000079931**	MOXD1	35.15576835	24.52242849	59.43902502	123.9105806	125.9809954	124.8165968	2102.029953	1712.813328	2083.600083	2512.75966	2008.703981	2703.257393
**ENSG00000079950**	STX7	1177.015124	1383.765608	1325.571681	1092.589917	1210.111029	1220.091719	2752.658271	2364.047881	2341.52942	2473.527452	2381.197164	2342.93585
**ENSG00000079974**	RABL2B	206.7159179	217.1986524	227.9853014	194.2382075	231.1577897	180.9650963	347.2741419	348.8756536	284.6601959	310.6937782	286.9577115	271.5098946
**ENSG00000079999**	KEAP1	645.459907	606.0543042	727.9244982	616.2039685	587.1407857	758.3841249	530.1037636	516.6682298	586.6720017	438.5148438	520.1108521	532.0240614
**ENSG00000080031**	PTPRH	298.1209156	567.5190594	341.1637189	227.7275536	382.5661419	307.2992202	8.171156279	19.93575163	13.59991051	6.960553076	23.4532745	19.45397999
**ENSG00000080166**	DCT	1.406230734	38.53524477	0.814233219	5.86063557	8.090522638	11.3814526	2.04278907	0	5.158586746	3.163887762	4.138813147	1.691650434
**ENSG00000080189**	SLC35C2	615.9290616	462.4229373	604.975282	529.9689023	632.2165547	716.652132	619.9864827	762.5425	672.0231643	697.9536403	709.1166525	680.8892995
**ENSG00000080200**	CRYBG3	164.5289959	217.1986524	192.1590398	141.4924873	265.8314581	211.3156366	923.3406595	2013.510915	644.8233432	917.527451	1718.98706	650.4395917
**ENSG00000080224**	EPHA6	0	0	2.442699658	0	4.623155793	4.173199286	0	0	0.937924863	0.632777552	0	0
**ENSG00000080298**	RFX3	352.9639143	262.7403053	336.2783196	216.0062824	279.7009255	191.2084037	179.7654381	132.9050109	190.8677096	172.7482718	155.8952952	163.2442668
**ENSG00000080345**	RIF1	4731.96642	5317.863779	4518.180135	5051.030628	5011.50088	4376.168524	4672.879997	4066.893333	4276.468412	4522.461167	4519.583956	4810.208008
**ENSG00000080371**	RAB21	933.7372075	1078.986854	915.1981387	905.8868124	941.9679928	1002.705974	1240.99436	1189.499847	1277.922626	1204.175682	1219.570274	1399.840734
**ENSG00000080493**	SLC4A4	0	0	3.256932878	0	0	0	2.04278907	4.983937908	6.096511609	1.898332657	6.898021912	2.53747565
**ENSG00000080503**	SMARCA2	1012.486129	1597.461056	1316.615116	1384.784462	2050.369594	1533.081665	1779.26928	2066.672919	1694.830227	1914.152096	2417.066878	1846.436448
**ENSG00000080546**	SESN1	149.0604578	98.08971397	115.6211172	107.1659076	208.0420107	158.5815729	400.3866577	508.3616667	321.2392655	427.1248479	594.6094888	395.0003762
**ENSG00000080561**	MID2	14.06230734	42.03844885	34.19779522	10.04680383	28.89472371	44.00828338	64.3478557	49.83937908	36.11010722	67.07442055	55.18417529	61.74524082
**ENSG00000080573**	COL5A3	1.406230734	0	0	5.86063557	3.467366845	1.896908767	1.021394535	3.322625272	8.910286198	7.593330629	1.379604382	7.612426951
**ENSG00000080603**	SRCAP	475.3059881	455.4165292	457.5990693	519.0848648	355.9829961	434.0127258	522.9540019	412.0055338	479.2796049	461.2948357	518.7312477	432.2166858
**ENSG00000080608**	PUM3	1063.110435	578.0286716	1011.277659	1171.28988	844.8817212	1114.623591	675.1417876	594.7499237	675.3059013	696.6880852	478.7227207	718.9514343
**ENSG00000080618**	CPB2	0	3.50320407	6.513865756	0	2.311577897	1.13814526	0	1.661312636	0.937924863	0	0	0
**ENSG00000080709**	KCNN2	199.6847643	84.07689769	114.8068839	159.074394	87.83996007	109.261945	1.021394535	0	1.875849726	0	9.657230676	0
**ENSG00000080802**	CNOT4	600.4605235	872.2978135	775.9642581	678.9964925	717.7449369	763.3160876	809.9658662	931.9963889	745.650266	786.5424976	975.3802983	800.1506551
**ENSG00000080815**	PSEN1	1108.109819	1415.294444	1305.215851	885.7932047	900.3595907	1063.786436	1325.770106	1378.889488	1366.087563	1178.231803	1254.060384	1352.474522
**ENSG00000080819**	CPOX	192.6536106	343.3139989	235.3134004	225.2158526	362.9177298	268.9816631	416.7289702	662.8637418	414.5627894	341.0671007	489.7595557	417.8376571

**ENSG00000080822**	CLDND1	859.2069786	935.3554868	982.7794959	912.5846816	1007.847963	1135.489588	961.1322573	1227.710038	921.9801402	1042.817406	1180.941351	946.4784176
**ENSG00000080823**	MOK	203.9034565	143.6313669	115.6211172	193.4009738	179.147287	137.3361947	261.4770009	287.407086	188.9918599	301.8348925	293.8557334	238.5227111
**ENSG00000080824**	HSP90AA1	22066.57268	23481.97688	23502.84188	21788.16858	22801.40437	21647.52284	22728.07119	23796.6422	25109.65547	21260.69298	22221.28779	29151.3661
**ENSG00000080839**	RBL1	1525.760347	1341.727159	1328.828614	1471.019528	1412.374095	1327.077373	1516.770884	1415.438366	1406.418332	1415.523385	1461.001041	1485.269081
**ENSG00000080845**	DLGAP4	1399.19958	1303.191914	1395.595738	1131.939899	1183.527883	1354.772241	601.601381	681.1381808	703.4436472	573.2964625	732.569927	674.968523
**ENSG00000080854**	IGSF9B	2.812461468	0	4.071166097	1.674467306	5.778944741	2.655672273	2.04278907	1.661312636	5.158586746	0.632777552	4.138813147	0.845825217
**ENSG00000080947**	CROCCP3	147.6542271	231.2114686	181.5740079	141.4924873	226.5346339	139.991867	233.8993485	385.4245316	182.8953483	249.3143556	373.8727876	159.0151408
**ENSG00000080986**	NDC80	1278.263737	767.2016914	1197.737066	1732.236428	1448.203552	1562.29406	1250.186911	1051.610899	1289.646686	1306.685646	1114.720341	1541.93937
**ENSG00000081014**	AP4E1	952.018207	798.7305281	863.0872126	918.4453172	983.576395	1009.914227	1129.662356	956.9160784	990.4486552	908.0357877	980.8987158	932.9452141
**ENSG00000081019**	RSBN1	493.5869877	472.9325495	447.8282707	456.2923408	706.1870474	515.200421	568.9167559	717.6870588	616.2166349	597.3420094	722.9126963	611.5316317
**ENSG00000081026**	MAGI3	101.2486129	161.1473872	118.87805	142.329721	177.991498	125.5753603	174.6584655	159.4860131	161.3230764	192.9971535	209.6998661	186.9273729
**ENSG00000081041**	CXCL2	57.6554601	80.57369362	43.96859385	15.07020575	18.49262317	18.21032416	16.34231256	8.306563181	7.972361335	8.226108181	8.277626294	5.920776517
**ENSG00000081051**	AFP	0	0	0	0	0	1.13814526	5.106972674	8.306563181	4.220661883	6.327775524	9.657230676	12.68737825
**ENSG00000081052**	COL4A4	18.28099954	0	25.2412298	18.41914036	58.94523636	39.8350841	1058.164738	1327.388796	1095.027277	1151.655145	1415.474096	1337.249668
**ENSG00000081059**	TCF7	8.437384405	17.51602035	6.513865756	15.07020575	27.73893476	17.0721789	11.23533988	14.95181373	18.75849726	14.5538837	22.07367012	40.59961041
**ENSG00000081087**	OSTM1	722.8025974	812.7433443	844.3598486	684.0198944	644.9302331	905.5842451	443.2852281	446.8930991	483.5002668	480.9109398	402.8444796	537.9448379
**ENSG00000081148**	IMPG2	5.624922937	0	0.814233219	0.837233653	11.55788948	3.41443578	7.149761744	1.661312636	3.28273702	0.632777552	1.379604382	7.612426951
**ENSG00000081154**	PCNP	1794.350417	1579.945036	1607.296375	1846.100205	1868.910729	1750.088028	2233.789848	2151.399864	2054.524412	2028.052055	2124.590749	2381.84381
**ENSG00000081177**	EXD2	262.9651473	234.7146727	256.4834641	269.5892362	337.4903729	277.7074434	458.6061462	473.4741013	358.7562601	485.9731602	553.2213573	457.5914423
**ENSG00000081181**	ARG2	98.43615139	108.5993262	81.42332194	102.1425056	114.4231059	72.08253313	7.149761744	31.56494009	28.13774589	37.33387559	35.86971394	43.13708606
**ENSG00000081189**	MEF2C	2.812461468	0	0	0	0	0.758763507	16.34231256	34.88756536	10.31717349	5.694997971	31.73090079	18.60815477
**ENSG00000081237**	PTPRC	8.437384405	3.50320407	5.699632536	20.93084132	6.93473369	6.828871559	7.149761744	0	3.28273702	3.796665314	2.759208765	0.845825217
**ENSG00000081277**	PKP1	371.2449138	385.3524477	350.1202844	874.9091672	810.2080527	666.5737406	113.3747934	149.5181373	254.6466003	170.2171616	182.1077785	268.1265937
**ENSG00000081307**	UBA5	895.7689777	721.6600385	765.3792263	920.9570182	940.8122039	874.4749414	799.7519208	694.4286819	809.4291567	819.4469303	809.8277724	840.7502655
**ENSG00000081320**	STK17B	196.8723028	133.1217547	160.4039442	254.5190305	239.2483123	240.5280316	300.2899933	232.5837691	302.9497307	257.5404638	211.0794705	269.8182442
**ENSG00000081377**	CDC14B	359.9950679	416.8812844	407.9308429	297.2179468	369.8524634	396.4539322	142.9952349	177.7604521	183.3643107	139.2110615	117.2663725	135.3320347
**ENSG00000081386**	ZNF510	462.6499115	434.3973047	432.3578395	411.0817236	394.1240314	452.6024317	562.7883887	603.0564869	534.6171719	561.273689	554.6009617	566.7028952
**ENSG00000081479**	LRP2	0	10.50961221	1.628466439	0	1.155788948	1.13814526	4.08557814	1.661312636	12.66198565	7.593330629	2.759208765	7.612426951
**ENSG00000081665**	ZNF506	151.8729193	171.6569995	203.5583049	128.0967489	142.1620406	164.2722992	617.9436936	588.1046732	620.9062592	592.279789	601.5075107	638.5980387
**ENSG00000081692**	JMJD4	229.2156097	171.6569995	254.0407645	237.7743574	179.147287	281.1218792	207.3430906	196.0348911	287.005008	229.6982515	189.0058004	172.5483442
**ENSG00000081721**	DUSP12	331.8704533	217.1986524	328.1359874	388.4764149	293.5703929	342.2023415	427.9643101	307.3428377	330.6185142	351.8243191	376.6319964	355.246591
**ENSG00000081760**	AACS	351.5576835	430.8941007	458.4133025	469.6880793	525.8839715	481.0560632	644.4999515	669.5089924	519.6103741	634.0431075	819.4850031	588.6943509
**ENSG00000081791**	KIAA0141	719.9901359	620.0671205	620.4457132	832.210251	692.31758	664.6768318	703.7408345	664.5250545	688.4368494	729.5925179	813.9665856	647.9021161
**ENSG00000081803**	CADPS2	277.0274546	581.5318757	306.9659237	43.53614995	144.4736185	84.60213099	189.9793835	245.8742701	173.9850621	115.7982921	132.4420207	109.9572782
**ENSG00000081818**	PCDHB4	0	0	0.814233219	0.837233653	1.155788948	0	2.04278907	0	0.937924863	1.265555105	5.518417529	1.691650434
**ENSG00000081842**	PCDHA6	8.437384405	0	2.442699658	2.511700959	0	0.758763507	0	0	0	2.53111021	0	0
**ENSG00000081853**	PCDHGA2	4.218692202	17.51602035	12.21349829	16.74467306	12.71367843	13.65774312	23.4920743	14.95181373	23.44812157	39.23220825	42.76773585	35.5246591
**ENSG00000081870**	HSPB11	590.6169083	917.8394665	564.2636211	514.8986965	565.1807957	514.4416575	694.5482837	767.5264379	784.5741478	652.3936565	607.0259282	845.8252168
**ENSG00000081913**	PHLPP1	334.6829147	514.9709984	405.4881433	591.9241926	596.3870973	510.6478399	394.2582905	430.2799728	407.5283529	451.1703948	582.1930493	406.8419293
**ENSG00000081923**	ATP8B1	1889.974107	994.909956	1450.963597	1876.240616	1502.525633	1711.391089	994.838277	774.1716884	1133.951159	821.345263	682.9041692	1014.99026
**ENSG00000081985**	IL12RB2	15.46853808	7.006408141	38.26896131	0	0	2.655672273	380.9801615	563.1849836	296.3842567	89.22163489	48.28615338	43.13708606
**ENSG00000082014**	SMARCD3	0	0	1.628466439	3.348934611	9.246311586	12.51959786	2.04278907	0	2.344812157	0	2.759208765	0
**ENSG00000082068**	WDR70	691.8655212	784.7177118	757.2368941	805.4187741	888.8017012	855.5058537	1124.555383	1044.965648	1035.938011	1056.105735	1082.98944	1058.127346
**ENSG00000082074**	FYB1	1447.011425	1702.557178	1218.092896	280.4732737	521.2608157	315.266237	2977.365069	1501.826623	1700.457776	2422.272471	1455.482623	1661.200726
**ENSG00000082126**	MPP4	21.09346101	14.01281628	12.21349829	49.39678552	27.73893476	26.55672273	0	9.967875817	0	2.53111021	0	0
**ENSG00000082146**	STRADB	158.904073	164.6505913	144.9335131	103.816973	164.1220307	154.4083736	99.07526988	144.5341993	131.3094808	122.1260676	122.78479	129.4112582
**ENSG00000082153**	BZW1	9105.344004	13115.99604	9331.926928	9631.535943	11114.06653	9643.125405	4885.33006	7215.080779	4893.622972	4668.000004	6413.780773	5378.602553
**ENSG00000082196**	C1QTNF3	18.28099954	10.50961221	19.54159727	14.2329721	43.91998003	25.41857747	20.4278907	13.29050109	19.22745969	34.16998783	46.906549	21.14563042
**ENSG00000082212**	ME2	1245.92043	963.3811194	1246.591059	1069.147375	976.6416613	1067.200872	994.838277	789.1235022	982.9452563	746.6775118	687.0429824	815.375509
**ENSG00000082213**	C5orf22	1492.010809	1685.041158	1431.422	1364.690854	1212.422607	1347.563988	1356.411942	1211.096912	1404.07352	1352.245629	989.1763421	1387.153356
**ENSG00000082258**	CCNT2	878.8942088	847.775385	681.5132047	632.1114079	552.4671173	594.8705892	1087.78518	887.1409477	897.1251314	872.6002447	805.6889593	895.7289046
**ENSG00000082269**	FAM135A	811.3951336	602.5511001	754.7941944	808.7677087	535.130283	649.8809434	1305.342216	1141.321781	1238.060819	1187.723466	1099.544693	1260.279573
**ENSG00000082293**	COL19A1	92.81122845	175.1602035	121.3207497	96.28187008	124.8252064	146.061975	0	0	0	0	1.379604382	1.691650434
**ENSG00000082397**	EPB41L3	0	0	8.956565414	2.511700959	0	1.517527013	13.27812895	13.29050109	7.972361335	9.491663286	13.79604382	7.612426951
**ENSG00000082438**	COBLL1	1189.671201	1359.243179	1196.108599	1085.054814	1211.266818	1267.89382	5609.498786	6919.367129	4956.9329	5127.396507	7378.124237	5458.955949
**ENSG00000082458**	DLG3	947.7995148	1040.451609	740.1379965	727.5560443	755.8859722	601.6994607	963.1750464	820.6884422	971.690158	846.0235875	807.0685637	888.1164776
**ENSG00000082482**	KCNK2	0	0	0	2.511700959	0	0.758763507	0	0	1.406887294	0.632777552	0	0.845825217
**ENSG00000082497**	SERTAD4	1171.390202	1121.025303	1018.605758	1556.417361	1512.927733	1449.997061	1288.999903	1264.258916	1141.923521	1127.609598	1147.830846	1123.255888
**ENSG00000082512**	TRAF5	122.3420739	84.07689769	140.862347	24.27977593	41.60840214	35.66188481	79.66877372	58.14594226	59.55822879	42.39609601	27.59208765	8.458252168
**ENSG00000082515**	MRPL22	601.8667542	711.1504263	626.1453458	686.5315954	649.5533889	745.4851452	461.6703298	543.249232	505.0725387	483.44205	536.6661047	608.9941561
**ENSG00000082516**	GEMIN5	992.7988983	521.9774065	927.411637	1240.780274	902.6711686	1141.939077	849.800253	657.8798039	969.8143082	1032.060188	749.1251796	1113.95181
**ENSG00000082641**	NFE2L1	2991.052772	3405.114356	4046.739101	2994.784776	3826.817208	4039.277527	3171.430031	3134.896944	3436.556698	3150.599433	3453.149769	3357.926111
**ENSG00000082684**	SEMA5B	2.812461468	3.50320407	0.814233219	2.511700959	2.311577897	0	3.064183605	19.93575163	9.379248629	12.0227735	44.14734023	7.612426951
**ENSG00000082701**	GSK3B	4605.405654	4879.96327	5036.846695	4850.094551	4950.244065	5309.068256	3761.796072	4297.81579	3878.319308	3885.886949	4296.088047	3944.928811
**ENSG00000082781**	ITGB5	383.9009904	532.4870187	715.7109999	574.3422859	1021.71743	1094.136977	655.7352914	1309.114357	1015.772627	680.2358688	1251.301175	1125.793364
**ENSG00000082805**	ERC1	853.5820556	819.7497525	941.2536017	791.185802	858.7511886	789.1140469	747.6607995	857.2373202	598.865025	674.5408708	791.8929154	674.1226978
**ENSG00000082898**	XPO1	8445.821789	8337.625688	8076.379304	8374.010996	8799.021263	7966.258056	8502.088108	7595.521372	8608.274392	8143.847099	7645.767487	8844.794292

**ENSG00000082996**	RNF13	871.8630552	795.227324	911.1269726	856.4900269	819.4543643	844.1244011	915.1695032	857.2373202	880.7114463	860.5774712	716.0146744	748.5553168
**ENSG00000083093**	PALB2	822.6449795	679.6215897	816.6759191	713.3230722	538.5976499	649.5015617	720.0831471	523.3134804	606.3684239	593.5453441	597.3686975	587.8485257
**ENSG00000083097**	DOPEY1	305.1520693	322.2947745	220.6572025	401.0349197	402.214554	380.5198986	957.0466792	956.9160784	770.0363124	1004.850753	1030.564474	977.7739506
**ENSG00000083099**	LYRM2	1023.735974	819.7497525	1002.321093	1137.800534	1150.010004	1141.939077	1262.443645	1010.078083	1063.137832	1195.316796	972.6210895	1166.392974
**ENSG00000083123**	BCKDHB	106.8735358	70.06408141	96.07951989	114.7010104	144.4736185	130.1279414	342.1671692	315.6494009	352.190786	370.8076457	368.3543701	345.0966884
**ENSG00000083168**	KAT6A	1987.004027	2298.10187	2211.457424	2144.155385	2332.382098	2333.577165	1278.785958	1342.34061	1260.571016	1257.961774	1226.468296	1363.470249
**ENSG00000083223**	ZCCHC6	2186.688792	2399.694788	2339.292039	1959.126748	2138.209554	1952.677884	3047.841292	2967.104368	2650.1067	2736.130136	2595.035843	2772.615061
**ENSG00000083290**	ULK2	507.649295	458.9197332	445.385571	469.6880793	420.7071772	433.2539623	367.7020326	412.0055338	389.2388181	409.4070764	325.5866342	386.5421241
**ENSG00000083307**	GRHL2	1476.542271	1492.364934	1310.915483	1719.677923	1977.55489	1632.859066	2130.629	2192.93268	2112.675754	1862.897114	2272.208418	1847.282273
**ENSG00000083312**	TNPO1	4782.590727	4165.30964	4851.201521	4767.208419	4088.02551	5144.037193	4037.572596	3816.035125	4554.563134	4056.736888	3664.229239	4778.06665
**ENSG00000083444**	PLOD1	1459.667502	2658.931889	1494.117958	1856.984242	2741.531385	2027.036708	973.3889917	1722.781204	1076.26878	1188.356243	1975.593475	1276.350252
**ENSG00000083454**	P2RX5	1.406230734	0	6.513865756	16.74467306	6.93473369	3.41443578	0	0	0	0.632777552	0	0
**ENSG00000083457**	ITGAE	132.185689	73.56728548	103.4076189	198.4243757	136.3830959	113.4351442	90.9041136	76.42038126	84.41323766	120.8605125	111.747955	126.8737825
**ENSG00000083520**	DIS3	2931.991081	3377.088724	2897.041795	2278.950003	2437.558892	2252.768851	2998.814354	3286.076394	2712.478704	2948.743394	3068.240146	3031.437577
**ENSG00000083535**	PIBF1	935.1434382	907.3298542	963.2378986	905.0495787	931.5658923	789.8728104	1312.491977	1181.193284	1196.323163	1270.617325	1096.785484	1286.500155
**ENSG00000083544**	TDRD3	538.5863712	434.3973047	512.152695	565.9699493	498.1450367	503.4395866	451.4563844	362.1661547	356.4114479	449.2720622	398.7056665	356.9382415
**ENSG00000083635**	NUFIP1	165.9352266	154.1409791	181.5740079	229.4020209	180.3030759	210.1774913	170.5728873	139.5502614	205.8745074	220.8393658	146.2380645	237.6768859
**ENSG00000083642**	PDS5B	2079.815256	2164.980116	1947.645861	2256.344694	2377.457867	2171.581156	2143.907129	2284.304875	2104.703392	2219.783654	2457.075405	2562.850407
**ENSG00000083720**	OXCT1	1954.66072	2059.883993	1786.427683	2606.308361	2465.297827	2303.606006	2229.70427	2098.237859	2091.572444	2341.276944	2269.449209	2432.593323
**ENSG00000083750**	RRAGB	220.7782253	178.6634076	249.1553652	220.1924507	238.0925233	189.3114949	304.3755714	279.1005229	257.9293373	225.2688086	281.439294	211.4563042
**ENSG00000083799**	CYLD	1759.194648	3030.271521	2190.28736	697.4156328	937.344837	822.4996412	959.0894682	1516.778437	869.4563479	652.3936565	738.0883445	612.3774569
**ENSG00000083807**	SLC27A5	36.56199909	0	19.54159727	24.27977593	16.18104528	25.03919572	27.57765244	41.5328159	40.79973154	45.55998377	24.83287888	22.83728085
**ENSG00000083812**	ZNF324	91.40499772	112.1025303	81.42332194	103.816973	76.28207059	105.0887457	176.7012545	201.018829	166.4816632	134.7816187	175.2097566	181.0065964
**ENSG00000083817**	ZNF416	67.49907524	59.5544692	67.58135721	77.02549606	46.23155793	78.91140469	88.86132453	169.4538889	121.9302322	106.9394064	136.5808338	122.6446564
**ENSG00000083828**	ZNF586	182.8099954	206.6890402	182.3882412	185.8658709	204.5746438	185.1382956	395.279685	310.665463	305.2945429	368.909313	387.6688314	323.1052328
**ENSG00000083838**	ZNF446	68.90530597	35.0320407	52.11092604	66.97869223	67.035759	43.24951988	85.79714093	76.42038126	109.737209	89.22163489	103.4703287	66.82019213
**ENSG00000083844**	ZNF264	818.4262873	637.5831408	849.2452479	738.4400818	736.23756	681.3696289	913.1267142	1074.869276	910.7250419	790.9719405	918.8165186	860.2042455
**ENSG00000083845**	RPS5	1735.288726	1821.666117	1755.486821	1778.284279	1710.567643	2012.999583	1601.546631	1805.846835	2019.821192	1776.839367	1754.856774	1870.96538
**ENSG00000083857**	FAT1	10565.01151	6214.684021	7841.880136	8849.559711	7332.325088	7613.053644	3501.340466	3679.807489	5019.304904	3079.728347	3177.228892	4502.327629
**ENSG00000083896**	YTHDC1	2200.751099	2105.425646	2077.923176	1970.848019	1797.251815	1803.580855	2165.356414	2271.014374	2032.483178	2037.543719	2193.570968	2119.637993
**ENSG00000083937**	CHMP2B	524.5240638	756.6920792	499.9391967	547.550809	553.6229062	534.5488904	689.441311	679.4768682	647.6371178	644.8003259	724.2923007	679.1976491
**ENSG00000084070**	SMAP2	331.8704533	434.3973047	426.658207	489.7816869	480.8082025	495.0931881	411.6219976	455.1996623	422.0661883	439.1476214	433.195776	504.1118292
**ENSG00000084072**	PPIE	1172.796432	1043.954813	1143.18344	1198.918591	1076.039511	1098.689558	1234.865993	1159.59622	1264.791678	1266.187882	1295.448515	1265.354524
**ENSG00000084073**	ZMPSTE24	2730.900086	1947.781463	2398.731064	2931.155019	2514.996751	2793.008468	1835.445979	1714.47464	2068.593285	1944.525418	1465.139854	2404.681091
**ENSG00000084090**	STARD7	1974.347951	1709.563586	1875.179104	2264.717031	2051.525383	2021.345982	2006.018866	1657.990011	1926.966631	2077.408704	1942.48297	2160.237604
**ENSG00000084092**	NOA1	241.8716863	329.3011826	198.6729055	252.0073295	240.4041012	272.7754806	243.0918993	227.5998311	272.4671727	295.507117	317.3090079	362.0131928
**ENSG00000084093**	REST	1288.107352	1429.307261	1342.670579	1284.316423	1273.679421	1420.784666	1754.755811	1727.765142	1638.554735	1530.056122	1734.162709	1809.220139
**ENSG00000084110**	HAL	5.624922937	3.50320407	2.442699658	0.837233653	5.778944741	4.55258104	8.171156279	3.322625272	8.441323766	7.593330629	5.518417529	8.458252168
**ENSG00000084112**	SSH1	1752.163495	1534.403383	1700.933195	1631.768389	1358.052014	1664.347752	1177.667899	1451.987244	1458.942124	1345.917854	1567.230578	1400.686559
**ENSG00000084207**	GSTP1	1333.106736	1145.547731	1281.603087	1202.267526	1267.900476	1297.106215	411.6219976	524.974793	562.7549177	522.6742583	624.9607852	612.3774569
**ENSG00000084234**	APLP2	5588.360938	5825.828369	5727.316466	7591.197531	7407.451369	7439.676182	4756.634349	5618.559335	5726.50025	5948.74177	7041.500767	6598.282516
**ENSG00000084444**	FAM234B	43.59315276	66.56087734	63.51019112	55.25742109	83.21680428	64.11551631	223.6854031	279.1005229	331.556439	262.6026842	329.7254474	375.5463962
**ENSG00000084453**	SLCO1A2	0	3.50320407	2.442699658	0.837233653	0	0.379381753	0	3.322625272	5.158586746	5.062220419	5.518417529	2.53747565
**ENSG00000084463**	WBP11	1127.797049	1156.057343	997.4356938	1134.4516	1210.111029	1188.982415	1591.332685	1643.038197	1622.610013	1568.022775	1558.952952	1782.999557
**ENSG00000084623**	EIF3I	3256.83038	3019.761909	3743.844343	3217.488928	3222.339588	3449.718283	2469.731985	2196.255305	2682.93407	2492.510779	2259.791978	2659.274482
**ENSG00000084628**	NKAIN1	0	7.006408141	0	0	0	0	7.149761744	0	1.875849726	1.265555105	0	0.845825217
**ENSG00000084636**	COL16A1	19.68723028	17.51602035	45.59706029	15.07020575	19.64841212	23.90105046	24.51346884	1.661312636	13.13094808	4.429442867	11.03683506	17.76232955
**ENSG00000084652**	TXLNA	2996.677694	2714.983155	3050.931873	2815.616775	2686.053516	2954.625095	2295.07352	2350.75738	2563.817613	2155.873121	2274.967626	2251.586727
**ENSG00000084674**	APOB	0	0	0	0	0	0	60.26227756	4.983937908	10.31717349	36.70109804	11.03683506	6.766601734
**ENSG00000084676**	NCOA1	262.9651473	318.7915704	420.9585745	291.3573112	346.7366845	401.0065132	815.0728388	920.3672004	812.2429313	970.6807654	1002.972386	899.9580306
**ENSG00000084693**	AGBL5	594.8356005	500.9581821	482.0260659	602.8082301	584.8292078	580.4540826	533.1679472	671.170305	619.9683344	642.9019932	813.9665856	613.2232822
**ENSG00000084710**	EFR3B	44.99938349	28.02563256	65.13865756	72.8393278	57.78944741	121.4021611	10.21394535	8.306563181	23.44812157	11.38999594	6.898021912	25.3747565
**ENSG00000084731**	KIF3C	407.8069129	399.365264	287.4243265	534.1550705	553.6229062	523.9262013	150.1449966	199.3575163	174.4540245	169.584384	306.2721729	250.3642642
**ENSG00000084733**	RAB10	3256.83038	3415.623969	3003.706347	2930.317785	3024.699678	2990.666361	2394.14879	2410.564635	2660.423874	2319.762507	2327.392593	2443.589051
**ENSG00000084734**	GCKR	0	7.006408141	9.770798633	0	0	0	0	0	0	0	0	0
**ENSG00000084754**	HADHA	2463.716246	2238.547401	2411.758796	2685.008325	2814.346089	2729.651715	3006.985511	2917.264989	2856.45017	2974.687274	2683.330524	2888.493115
**ENSG00000084764**	MAPRE3	15.46853808	70.06408141	43.96859385	32.65211246	49.69892478	53.49282722	26.55625791	23.25837691	14.06887294	21.51443678	13.79604382	17.76232955
**ENSG00000084774**	CAD	1574.978422	1173.573364	1525.873053	1573.162034	1445.891974	1726.945741	1450.38024	1030.013834	1662.002857	1659.142742	1240.26434	1622.292766
**ENSG00000085063**	CD59	6799.1256	6691.119775	7199.450126	7484.031623	7651.322838	8561.887409	4838.345912	5254.731868	5817.478962	6380.296061	5777.783153	7253.797059
**ENSG00000085117**	CD82	410.6193744	392.3588559	336.2783196	318.1487881	286.6356592	280.3631157	472.9056696	682.7994934	438.9488358	494.1992684	716.0146744	532.8698866
**ENSG00000085185**	BCORL1	97.02992066	105.0961221	146.5619795	173.3073661	255.4293576	179.8269511	37.79159779	44.85544118	49.2410553	62.01220013	44.14734023	57.51611474
**ENSG00000085224**	ATRX	2449.653939	2637.912665	2331.149707	2454.76907	2560.07252	2366.203995	2700.56715	2462.065327	2264.150619	2628.557953	2814.39294	2470.655458
**ENSG00000085231**	AK6	269.996301	294.2691419	282.5389271	305.5902833	275.0777697	308.8167472	290.0760479	257.5034586	234.9501782	255.6421312	242.8103713	270.6640694
**ENSG00000085274**	MYNN	397.9632978	315.2883663	426.658207	398.5232188	365.2293077	376.3466993	341.1457746	410.3442211	376.10787	328.4115497	340.7622824	412.7627058
**ENSG00000085276**	MECOM	2.812461468	45.54165292	13.84196473	5.023401917	12.71367843	23.90105046	0	0	0	0.632777552	0	0
**ENSG00000085365**	SCAMP1	912.6437465	858.2849973	841.9171489	874.0719336	1079.506878	877.8893771	736.4254596	739.2841231	762.0639511	789.0736078	749.1251796	870.3541481
**ENSG00000085377**	PREP	1282.48243	938.8586909	1216.46443	1048.216533	961.616405	968.9409979	767.0672957	759.2198747	737.6779047	629.6136646	732.569927	719.7972595

**ENSG00000085382**	HACE1	662.3346758	823.2529565	707.5686677	833.8847183	848.349088	843.7450193	810.9872607	998.4488943	837.5669026	847.9219202	1074.711814	810.3005577
**ENSG00000085415**	SEH1L	1231.858123	742.6792629	1092.70098	1470.182294	1014.782697	1253.856695	1171.539532	1001.77152	1219.302322	1215.565678	924.3349361	1371.928502
**ENSG00000085433**	WDR47	617.3352923	735.6728548	593.576017	682.3454271	799.8059522	674.5407574	453.4991735	983.4970806	509.762163	429.0231805	739.4679489	514.2617318
**ENSG00000085449**	WDFY1	749.5209813	1075.48365	703.4975016	507.3635936	666.8902231	591.8355352	715.9975689	862.2212581	719.8573323	473.9503867	555.9805661	551.4780413
**ENSG00000085465**	OVGP1	18.28099954	7.006408141	5.699632536	4.186168264	16.18104528	7.587635066	14.29952349	29.90362745	20.63434698	8.858885733	15.17564821	10.1499026
**ENSG00000085491**	SLC25A24	1209.358431	1415.294444	1151.325772	1022.26229	1404.283572	1131.316388	1126.598172	1407.131803	1026.0898	1077.620172	1207.153835	1159.626372
**ENSG00000085511**	MAP3K4	641.2412148	760.1952833	749.9087951	832.210251	810.2080527	794.4253914	732.3398815	843.9468192	692.6575113	739.0841812	841.5586732	739.2512395
**ENSG00000085514**	PILRA	36.56199909	7.006408141	24.42699658	19.25637402	31.2063016	11.00207085	12.25673442	33.22625272	7.972361335	18.35054902	19.31446135	11.84155303
**ENSG00000085552**	IGSF9	105.4673051	84.07689769	88.75142092	263.7286007	211.5093775	281.1218792	458.6061462	398.7150327	664.0508029	534.0642542	376.6319964	541.3281387
**ENSG00000085644**	ZNF213	201.090995	199.682632	168.5462764	159.9116277	165.2778196	207.5218191	166.4873092	142.8728867	225.5709295	182.2399351	223.4959099	167.4733929
**ENSG00000085662**	AKR1B1	344.5265299	507.9645902	464.1129351	329.0328256	464.6271572	520.8911473	924.3620541	694.4286819	974.0349701	1134.570151	779.476476	1382.078404
**ENSG00000085719**	CPNE3	2940.428465	2743.008787	2728.495518	3247.629339	3439.62791	3085.5118	2717.930857	2659.76153	2677.306521	2823.453439	2672.293689	3202.294271
**ENSG00000085721**	RRN3	641.2412148	518.4742024	741.7664629	727.5560443	740.8607158	846.7800734	679.2273657	470.151476	726.4228063	658.721432	459.4082593	717.2597838
**ENSG00000085733**	CTTN	3831.978751	3482.184846	4054.0672	3250.14104	3363.345839	3660.654538	2341.036274	2269.353061	2391.239438	2220.416431	2401.89123	2282.88226
**ENSG00000085741**	WNT11	0	7.006408141	1.628466439	0	0	0	0	0	1.406887294	0	0	6.766601734
**ENSG00000085760**	MTIF2	1040.610743	966.8843234	1117.94221	1183.011152	1018.250063	1147.629804	1148.047457	1015.062021	1015.772627	1039.020741	958.8250457	1086.039578
**ENSG00000085788**	DDHD2	587.8044469	420.3844885	595.2044834	742.6262501	688.8502132	663.1593048	427.9643101	415.328159	361.1010722	444.2098418	477.3431163	339.1759119
**ENSG00000085831**	TTC39A	11.24984587	42.03844885	30.12662912	29.30317785	31.2063016	39.8350841	8.171156279	0	0	5.694997971	1.379604382	6.766601734
**ENSG00000085832**	EPS15	2588.870782	2298.10187	2208.200491	2422.116958	2171.727434	2241.008017	2010.104445	1875.621966	1907.739171	1884.411551	1685.876555	1869.273729
**ENSG00000085840**	ORC1	523.1178331	634.0799367	543.0935574	557.5976128	680.7596905	528.4787824	526.0181855	747.5906863	651.3888173	584.6864584	640.1364334	636.060563
**ENSG00000085871**	MGST2	64.68661377	105.0961221	95.26528668	77.02549606	105.1767943	141.8887757	94.98969174	91.37219499	68.46851499	125.9227329	88.29468047	93.88659906
**ENSG00000085872**	CHERP	516.0866794	409.8748762	555.3070557	523.271033	501.6124035	574.0045927	516.8256346	604.7177995	559.4721807	603.669785	518.7312477	635.2147378
**ENSG00000085978**	ATG16L1	584.9919854	662.1055693	634.2876779	636.2975762	679.6039016	720.4459495	398.3438686	554.8784205	414.093827	456.8653928	513.2128302	406.8419293
**ENSG00000085982**	USP40	521.7116024	683.1247937	689.6555369	460.4785091	573.2713183	567.5551029	791.5807645	724.3323094	717.9814826	797.299716	823.6238162	725.718036
**ENSG00000085998**	POMGNT1	538.5863712	315.2883663	562.6351546	803.7443068	703.8754695	939.7286029	466.7773024	388.7471569	530.8654724	602.4042299	543.5641266	614.9149326
**ENSG00000085999**	RAD54L	673.5845217	795.227324	698.6121023	783.6506991	657.6439116	658.9861055	809.9658662	799.091378	812.2429313	885.2557958	1040.221704	830.6003629
**ENSG00000086015**	MAST2	902.8001313	795.227324	911.1269726	1254.176012	1328.001502	1284.965998	865.121171	819.0271296	908.8491922	968.7824327	837.4198601	941.4034663
**ENSG00000086061**	DNAJA1	6650.065142	7286.664466	7576.440107	4227.192713	4921.349342	5052.985572	4782.169212	5459.073322	5425.895332	3463.191544	3486.260274	4745.079466
**ENSG00000086062**	B4GALT1	2285.124943	3429.636785	3113.627831	2302.392545	2746.154541	3102.96336	2993.707382	2045.075855	3244.751063	2870.911755	1903.854048	2870.730786
**ENSG00000086065**	CHMP5	1933.567259	2207.018564	1832.838977	1443.390818	1531.420356	1658.657025	1084.720996	1078.191901	1079.551517	943.4713306	939.5105844	1086.885404
**ENSG00000086102**	NFX1	1150.296741	1068.477241	1351.627144	1285.990891	1130.361591	1190.12056	1141.91909	1083.175839	1180.847402	1149.124035	1037.462495	1093.652005
**ENSG00000086189**	DIMT1	407.8069129	196.1794279	355.0056837	560.1093138	344.4251066	501.1632961	427.9643101	285.7457734	395.8042921	471.4192765	318.6886123	481.2745483
**ENSG00000086200**	IPO11	1122.172126	889.8138339	1096.772147	1246.640909	1141.919481	1236.405134	1309.427794	1182.854597	1267.605452	1259.227329	1259.578801	1437.902869
**ENSG00000086232**	EIF2AK1	2258.406559	1961.794279	2125.148703	2351.789331	2459.518882	2266.426594	2852.754936	2496.952892	2607.431119	2707.022369	2778.523226	2826.747874
**ENSG00000086300**	SNX10	61.8741523	112.1025303	77.35215585	44.3733836	110.955739	78.91140469	101.118059	91.37219499	51.11690503	74.03497363	103.4703287	80.35339559
**ENSG00000086475**	SEPHS1	620.1477538	539.4934268	483.6545324	714.1603059	589.4523636	676.4376661	592.4088302	546.5718573	612.4649355	636.5742177	589.0910712	707.1098812
**ENSG00000086504**	MRPL28	427.4941432	329.3011826	456.7848361	384.2902467	375.6314082	451.4642864	253.3058446	252.5195207	319.8323783	273.9926802	253.8472063	286.7347485
**ENSG00000086544**	ITPKC	708.74029	980.8971397	1046.289687	383.453013	392.9682424	555.0355051	350.3383255	365.4887799	395.3353297	325.2476619	331.1050518	311.2636798
**ENSG00000086548**	CEACAM6	14.06230734	10.50961221	6.513865756	1.674467306	5.778944741	5.311344546	0	0	4.220661883	0.632777552	4.138813147	5.074951301
**ENSG00000086570**	FAT2	91.40499772	77.07048955	40.71166097	155.7254594	93.61890481	76.25573241	140.9524458	162.8086383	370.0113584	186.669378	281.439294	395.0003762
**ENSG00000086589**	RBM22	1375.293658	1558.925811	1398.852671	1296.037695	1375.388848	1349.460897	1385.010989	1709.490703	1430.335416	1352.878407	1575.508205	1445.515295
**ENSG00000086598**	TMED2	3689.949446	3538.236111	3634.737092	3424.28564	3787.520383	3639.029778	4536.013129	4473.914929	4624.438537	4409.193985	4205.034157	5231.428966
**ENSG00000086619**	ERO1B	75.93645964	49.04485699	65.13865756	21.76807497	12.71367843	20.86599643	145.038024	147.8568246	170.702325	151.8666126	142.0992514	184.3898973
**ENSG00000086666**	ZFAND6	676.3969831	872.2978135	759.6795937	736.7656145	763.9764948	726.1366758	443.2852281	481.7806645	404.7145783	403.0793009	358.6971394	421.220958
**ENSG00000086696**	HSD17B2	0	0	0	0	0	0	12.25673442	1.661312636	4.220661883	9.491663286	4.138813147	14.37902869
**ENSG00000086712**	TXLNG	572.3359088	514.9709984	591.9475505	723.3698761	617.1912984	657.8479602	503.5475057	402.0376579	553.3756691	541.6575848	466.3062812	681.7351247
**ENSG00000086730**	LAT2	272.8087624	262.7403053	258.9261638	139.81802	156.031508	150.6145561	6.128367209	9.967875817	4.220661883	4.429442867	1.379604382	4.229126084
**ENSG00000086758**	HUWE1	10452.51305	10716.30125	11984.69876	10011.64002	11720.85572	11371.96806	9083.261599	8726.875278	9343.607484	9018.345677	9722.072082	9238.948843
**ENSG00000086827**	ZW10	791.7079033	581.5318757	731.9956643	956.9580652	858.7511886	946.9368562	737.4468542	679.4768682	672.9610891	806.7913793	699.4594218	748.5553168
**ENSG00000086848**	ALG9	189.8411491	206.6890402	127.0203822	182.5169363	190.7051765	156.6846641	172.6156764	194.3735784	128.0267438	108.837739	200.0426354	126.8737825
**ENSG00000087008**	ACOX3	151.8729193	126.1153465	100.9649192	144.8414219	143.3178296	158.9609546	135.8454731	147.8568246	138.8128797	160.7254983	125.5439988	126.0279573
**ENSG00000087053**	MTMR2	1421.699272	1061.470833	1357.326777	1751.492802	1591.521382	1663.588988	1340.06963	1129.692593	1385.315023	1428.811713	1281.652471	1605.376261
**ENSG00000087074**	PPP1R15A	1743.72611	1842.685341	2316.493509	828.0240827	839.1027764	1095.275122	689.441311	1021.707271	674.8369389	517.6120378	718.7738832	494.8077518
**ENSG00000087076**	HSD17B14	5.624922937	10.50961221	17.09889761	19.25637402	13.86946738	11.76083435	5.106972674	6.645250545	6.56547404	5.062220419	13.79604382	5.920776517
**ENSG00000087077**	TRIP6	413.4318358	353.8236111	384.3180796	380.1040784	332.8672171	484.0911172	341.1457746	292.391024	327.3357772	275.2582353	226.2551187	246.1351381
**ENSG00000087085**	ACHE	0	0	2.442699658	1.674467306	0	2.27629052	0	0	0.937924863	0	0	0.845825217
**ENSG00000087086**	FTL	1570.75973	1947.781463	2207.386258	982.9123085	1347.649914	1444.685717	715.9975689	1216.08085	1065.482644	685.2980892	1062.295374	893.1914289
**ENSG00000087087**	SRRT	1386.543504	1345.230363	1414.323102	1452.600388	1496.746688	1408.265068	1270.614801	1415.438366	1373.590962	1340.855633	1434.788558	1491.189857
**ENSG00000087088**	BAX	293.9022234	213.6954483	275.2108282	269.5892362	218.4441112	284.1569332	144.0166294	161.1473257	234.0122533	232.8621393	212.4590749	181.0065964
**ENSG00000087095**	NLK	376.8698368	479.9389576	417.7016416	340.7540967	275.0777697	363.4477197	441.2424391	383.7632189	422.0661883	417.000407	336.6234693	345.9425137
**ENSG00000087111**	PIGS	451.4000657	423.8876925	477.1406666	548.3880426	595.2313084	630.1530922	790.55937	940.302952	846.9461512	899.1769019	954.6862326	880.5040507
**ENSG00000087128**	TMPRSS11E	271.4025317	581.5318757	567.520554	552.5742109	959.3048271	766.3511417	1.021394535	4.983937908	14.53783538	6.327775524	20.69406573	23.68310607
**ENSG00000087152**	ATXN7L3	793.1141341	819.7497525	855.7591136	855.6527932	932.7216812	836.9161478	812.0086552	794.1074401	805.6774572	756.1691751	698.0798174	820.4504603
**ENSG00000087157**	PGS1	331.8704533	220.7018564	271.1396621	297.2179468	282.0125034	328.1652166	323.7820676	307.3428377	294.9773694	263.8682393	311.7905904	311.2636798
**ENSG00000087191**	PSMC5	1709.976573	1800.646892	1998.942554	2029.454375	1886.247564	2038.418161	1196.053	1206.112974	1329.977456	1359.83896	1295.448515	1429.444616
**ENSG00000087206**	UIMC1	877.4879781	816.2465484	890.7711421	786.1624	743.1722937	757.2459796	739.4896433	754.2359368	646.2302305	616.325336	711.8758613	667.356096
**ENSG00000087245**	MMP2	4.218692202	10.50961221	12.21349829	8.372336529	26.58314581	17.83094241	0	0	0.468962431	0.632777552	4.138813147	0

**ENSG00000087253**	LPCAT2	219.3719945	241.7210809	223.9141353	271.2637035	372.1640413	352.4456488	0	4.983937908	7.972361335	5.062220419	13.79604382	10.1499026
**ENSG00000087258**	GNAO1	9.843615139	45.54165292	15.47043117	30.1404115	39.29682424	36.80003007	61.28367209	26.58100218	30.01359561	101.8771859	45.52694462	54.13281387
**ENSG00000087263**	OGFOD1	850.7695942	637.5831408	934.7397359	973.7027383	722.3680927	965.9059439	771.1528738	493.4098529	669.2093897	670.111428	372.4931832	701.1891047
**ENSG00000087266**	SH3BP2	513.274218	1026.438793	628.5880454	348.2891996	671.5133789	442.3591244	104.1822426	119.6145098	96.60626088	129.7193982	126.9236032	71.89514343
**ENSG00000087269**	NOP14	835.3010561	599.047896	888.3284424	1033.146328	898.0480128	932.5203496	738.4682487	579.79811	751.2778152	742.2480689	644.2752465	797.6131794
**ENSG00000087274**	ADD1	1607.321729	1671.028342	1529.129986	1457.62379	1688.607653	1504.628034	1467.743947	1814.153399	1506.776292	1575.616105	1888.678399	1596.072184
**ENSG00000087299**	L2HGDH	284.0586083	301.2755501	281.7246939	318.1487881	345.5808955	291.7445683	423.878732	465.1675381	417.8455264	429.0231805	498.037182	469.4329953
**ENSG00000087301**	TXNDC16	196.8723028	234.7146727	186.4594073	179.1680017	220.7556891	198.7960387	317.6537003	285.7457734	289.8187826	282.8515659	358.6971394	265.5891181
**ENSG00000087302**	RTRAF	1874.505569	1828.672525	1775.028418	2062.106487	1630.818206	2016.034637	1354.369153	1385.534739	1456.597312	1415.523385	1332.697833	1651.896648
**ENSG00000087303**	NID2	37.96822982	52.54806106	49.66822639	77.02549606	92.46311586	59.94231702	23.4920743	9.967875817	13.59991051	17.71777147	16.55525259	14.37902869
**ENSG00000087338**	GMCL1	499.2119106	567.5190594	494.2395642	580.2029214	544.3765946	540.6189985	601.601381	546.5718573	578.230678	588.4831237	587.7114669	677.5059986
**ENSG00000087365**	SF3B2	3621.04414	3993.65264	3795.955269	3740.759961	3817.570896	3771.813391	3052.948265	3380.771215	3236.778702	3146.802768	3243.449903	3218.36495
**ENSG00000087448**	KLHL42	312.183223	360.8300193	246.7126655	296.3807131	329.3998503	322.4744903	523.9753964	574.8141721	479.2796049	499.2614888	605.6463238	494.8077518
**ENSG00000087460**	GNAS	5193.210101	6078.059062	5078.37259	5591.046334	6241.260321	5338.660033	4764.805505	5834.529978	4985.070646	4974.897117	5935.058053	5396.364883
**ENSG00000087470**	DNM1L	907.0188235	711.1504263	992.5502945	988.772944	735.0817711	922.2770423	979.5173589	945.28689	1102.530676	971.9463205	838.7994644	1157.934722
**ENSG00000087494**	PTHLH	0	0	0	0	0	1.896908767	6.128367209	36.54887799	5.158586746	13.92110615	40.00852709	1.691650434
**ENSG00000087495**	PHACTR3	4.218692202	0	4.071166097	2.511700959	6.93473369	11.76083435	3.064183605	1.661312636	1.875849726	1.898332657	0	0
**ENSG00000087502**	ERGIC2	336.0891455	413.3780803	311.0370898	371.7317419	388.3450866	395.3157869	642.4571624	674.4929303	551.9687818	698.5864178	583.5726537	735.8679386
**ENSG00000087510**	TFAP2C	1088.422588	676.1183856	963.2378986	1711.305586	1427.399351	1540.289918	1981.505398	1460.293807	2056.869224	2066.018709	1564.47137	2143.321099
**ENSG00000087586**	AURKA	1442.792733	791.7241199	1434.678933	1853.635307	1532.576145	2006.929475	1082.678207	772.5103758	1234.30912	1135.202929	784.9948935	1409.990636
**ENSG00000087589**	CASS4	7.031153671	7.006408141	6.513865756	3.348934611	1.155788948	10.62268909	7.149761744	11.62918845	4.689624315	1.898332657	4.138813147	5.074951301
**ENSG00000087842**	PIR	449.9938349	385.3524477	489.3541649	296.3807131	332.8672171	358.5157569	271.6909463	247.5355828	317.9565285	249.3143556	259.3656239	291.8096998
**ENSG00000087884**	AAMDC	52.03053716	63.05767327	56.18209214	58.6063557	70.50312584	48.18148267	51.06972674	33.22625272	20.63434698	34.80276538	53.80457091	39.75378519
**ENSG00000087903**	RFX2	243.277917	213.6954483	167.7320432	205.9594786	257.7409355	173.756843	226.7495867	410.3442211	272.9361351	285.3826761	386.289227	268.1265937
**ENSG00000087995**	METTL2A	478.1184496	627.0735286	503.1961296	531.6433696	465.7829462	571.7283022	346.2527473	342.230403	340.4667252	349.2932089	358.6971394	370.4714449
**ENSG00000088002**	SULT2B1	47.81184496	49.04485699	46.41129351	59.44358935	33.5178795	45.14642864	0	0	4.689624315	0.632777552	1.379604382	2.53747565
**ENSG00000088035**	ALG6	435.9315276	521.9774065	370.4761148	492.2933879	450.7576898	492.058134	514.7828456	453.5383497	505.5415011	541.6575848	533.906896	578.5444483
**ENSG00000088038**	CNOT3	510.4617565	483.4421617	525.1804265	478.0604158	512.0145041	514.8210392	364.637849	428.6186601	413.1559021	449.9048397	420.7793366	430.5250353
**ENSG00000088053**	GP6	11.24984587	7.006408141	17.09889761	1.674467306	12.71367843	15.93403364	8.171156279	8.306563181	13.13094808	8.226108181	20.69406573	8.458252168
**ENSG00000088179**	PTPN4	587.8044469	378.3460396	469.8125676	709.9741376	665.7344342	688.957264	648.5855296	430.2799728	610.1201233	689.0947545	554.6009617	746.8636664
**ENSG00000088205**	DDX18	1327.481813	1467.842506	1318.243582	1491.113136	1404.283572	1373.361947	1346.197997	1332.372734	1422.832017	1335.793413	1238.884735	1472.581702
**ENSG00000088247**	KHSRP	2678.869549	2150.967299	2669.056493	2794.685933	2632.887224	2972.456037	2842.540991	2518.549956	3111.565733	3013.919482	2766.106787	3043.27913
**ENSG00000088256**	GNA11	1044.829435	991.4067519	952.6528668	940.2133922	935.0332591	1151.04424	757.8747449	682.7994934	901.3457933	754.90362	620.821972	873.7374489
**ENSG00000088280**	ASAP3	667.9595987	283.7595297	316.7367224	802.9070731	547.8439615	405.1797125	2030.532335	936.9803268	1941.504466	2024.25539	1069.193396	1933.556446
**ENSG00000088298**	EDEM2	412.0256051	364.3332233	468.9983344	329.0328256	290.103026	375.208554	412.6433921	402.0376579	513.5138624	455.5998377	401.4648753	497.3452275
**ENSG00000088305**	DNMT3B	15.46853808	49.04485699	24.42699658	43.53614995	45.07576898	29.97115851	216.5356414	111.3079466	200.2469582	267.6649047	146.2380645	337.4842615
**ENSG00000088325**	TPX2	6008.823927	3674.86107	5663.806274	7774.551701	6980.965248	7737.87024	6626.807742	5548.784205	7201.85606	7239.607977	6533.806355	8397.352752
**ENSG00000088340**	FER1L4	16.87476881	38.53524477	2.442699658	5.023401917	25.42735686	8.725780326	8.171156279	39.87150327	7.972361335	16.45221636	97.95191114	7.612426951
**ENSG00000088356**	PDRG1	277.0274546	164.6505913	306.1516905	248.6583949	241.5598902	341.443578	288.0332588	285.7457734	367.1975838	285.3826761	252.467602	352.7091154
**ENSG00000088367**	EPB41L1	53.4367679	56.05126513	61.88172468	66.14145858	93.61890481	53.11344546	892.6988235	885.4796351	823.9669921	1180.762913	1299.587328	840.7502655
**ENSG00000088386**	SLC15A1	0	0	2.442699658	0	3.467366845	1.13814526	0	4.983937908	0.937924863	1.898332657	1.379604382	0
**ENSG00000088387**	DOCK9	3213.237228	2928.678603	3519.930208	2244.623423	2970.377597	3046.056097	4771.955267	7882.928458	4102.952313	3021.512813	5677.072033	2781.919138
**ENSG00000088448**	ANKRD10	6983.341826	5776.783512	6206.899832	3704.758914	3907.722434	4044.20949	3826.143928	3993.795577	3635.396769	3649.228145	3889.104754	3179.45699
**ENSG00000088451**	TGDS	444.368912	322.2947745	380.2469135	332.3817602	298.1935487	292.12395	389.1513178	352.1982789	406.5904281	359.4176498	275.9208765	342.5592128
**ENSG00000088538**	DOCK3	37.96822982	42.03844885	37.45472809	35.16381342	39.29682424	35.28250306	12.25673442	29.90362745	7.972361335	15.81943881	28.97169203	8.458252168
**ENSG00000088543**	C3orf18	15.46853808	7.006408141	20.35583049	20.09360767	26.58314581	25.03919572	4.08557814	3.322625272	1.875849726	4.429442867	6.898021912	4.229126084
**ENSG00000088682**	COQ9	323.4330689	294.2691419	359.0768498	424.477462	322.4651166	338.7879057	326.8462512	403.6989706	370.9492833	406.2431886	369.7339745	383.1588232
**ENSG00000088726**	TMEM40	1120.765895	851.2785891	1050.360853	1130.265431	1022.873219	1186.706124	462.6917243	662.8637418	558.5342559	479.6453847	431.8161717	405.9961041
**ENSG00000088727**	KIF9	30.93707615	21.01922442	30.94086234	48.55955187	49.69892478	38.31755708	46.9841486	51.50069172	57.68237907	62.01220013	42.76773585	51.59533822
**ENSG00000088756**	ARHGAP28	2.812461468	17.51602035	8.142332194	5.023401917	18.49262317	6.449489806	84.77574639	73.09775599	105.0475846	77.19886139	82.77626294	106.5739773
**ENSG00000088766**	CRLS1	97.02992066	87.58010176	74.09522297	78.69996337	63.56839215	75.11758715	122.5673442	151.1794499	138.3439173	111.3688492	117.2663725	98.11572515
**ENSG00000088808**	PPP1R13B	206.7159179	245.2242849	242.6414994	266.2403016	288.9472371	286.053842	219.599825	284.0844608	239.6398025	230.3310291	289.7169203	258.8225163
**ENSG00000088812**	ATRN	804.3639799	924.8458746	1011.277659	776.1155962	1092.220556	1135.868969	1388.075173	1744.378268	1344.984253	1508.541685	1827.975807	1535.172768
**ENSG00000088826**	SMOX	49.2180757	63.05767327	70.83829009	90.42123451	117.8904727	119.5052523	22.47067977	4.983937908	12.19302322	14.5538837	16.55525259	24.52893129
**ENSG00000088832**	FKBP1A	7167.558052	7104.497855	8123.60483	6840.198944	7222.525138	8789.895842	2881.353983	2736.181912	3159.868863	3098.078896	3095.832234	3171.844563
**ENSG00000088833**	NSFL1C	3009.333771	3307.024642	3398.609458	2947.899692	3131.032261	3414.056398	2353.293008	2438.80695	2192.868329	2183.715333	2408.789252	2203.37469
**ENSG00000088836**	SLC4A11	30.93707615	31.52883663	38.26896131	14.2329721	26.58314581	22.38352344	9.192550814	6.645250545	11.25509835	25.3111021	12.41643944	10.1499026
**ENSG00000088854**	C20orf194	4.218692202	84.07689769	20.35583049	19.25637402	36.98524634	20.10723293	13.27812895	23.25837691	8.910286198	15.81943881	49.66575776	25.3747565
**ENSG00000088876**	ZNF343	268.5900702	189.1730198	220.6572025	240.2860584	226.5346339	216.2475994	295.1830206	260.8260839	280.439534	316.3887762	299.374151	274.0473702
**ENSG00000088888**	MAVS	5518.049401	5146.206779	5952.044834	6278.415163	6032.062521	6650.562135	5448.118449	4959.018219	5724.624401	5616.533555	5431.502453	5591.750508
**ENSG00000088899**	LZTS3	11.24984587	10.50961221	13.02773151	16.74467306	33.5178795	12.89897961	23.4920743	33.22625272	41.7376564	27.20943475	22.07367012	32.98718345
**ENSG00000088930**	XRN2	3802.447905	3377.088724	3668.934887	3615.174913	3726.263569	3672.03599	3983.438686	3910.729945	3554.266268	4157.348519	3803.569282	4125.089582
**ENSG00000088970**	KIZ	191.2473798	329.3011826	229.6137679	270.4264699	313.218805	270.4991901	448.3922008	402.0376579	386.8940059	423.3281825	527.008874	429.6792101
**ENSG00000088986**	DYNLL1	1975.754181	3671.357866	2245.655219	2535.980735	3327.516382	2592.694902	1759.862784	3176.42976	2357.943105	2093.228143	3179.988101	2410.601868
**ENSG00000089006**	SNX5	3175.268998	1912.749422	2825.389271	3384.935659	2480.323083	2977.008618	3807.758826	2840.844608	3510.652762	3792.868649	3244.829507	3727.55173
**ENSG00000089009**	RPL6	8922.534008	7633.481669	8349.147432	9639.071045	9442.795707	9003.867151	9593.958866	9007.637113	9597.31616	10744.56284	10087.66724	11264.70024
**ENSG00000089012**	SIRPG	33.74953762	38.53524477	52.92515926	28.4659442	67.035759	49.31962793	7.149761744	3.322625272	3.28273702	3.163887762	0	4.229126084

**ENSG00000089022**	MAPKAPK5	679.2094446	528.9838146	689.6555369	622.0646041	590.6081526	616.4953491	1099.02052	907.0766993	960.4350596	1084.580725	927.0941449	921.9494863
**ENSG00000089041**	P2RX7	1.406230734	0	0	0	0	2.655672273	1.021394535	1.661312636	0.937924863	0.632777552	1.379604382	3.383300867
**ENSG00000089048**	ESF1	1115.140972	683.1247937	1120.38491	1408.227004	1012.471119	1159.77002	896.7844016	895.4475109	960.4350596	1111.157382	812.5869812	1118.180937
**ENSG00000089050**	RBBP9	442.9626813	521.9774065	471.4410341	531.6433696	581.361841	547.0684883	543.3818926	549.8944826	619.4993719	609.3647829	595.9890932	661.4353195
**ENSG00000089053**	ANAPC5	1549.666269	1159.560547	1388.267639	1739.771531	1433.178296	1407.885687	2480.967325	2163.029052	2102.35858	2505.799107	2288.76367	2231.286922
**ENSG00000089057**	SLC23A2	828.2699024	742.6792629	788.9919896	1107.660123	848.349088	1152.941148	1224.652047	995.126269	1279.798475	1449.693373	1109.201923	1363.470249
**ENSG00000089060**	SLC8B1	161.7165344	203.1858361	240.1987997	157.3999267	164.1220307	190.0702584	281.9048916	410.3442211	315.1427539	328.4115497	364.2155569	317.1844563
**ENSG00000089063**	TMEM230	1514.510501	1464.339301	1475.390594	1666.094969	1754.487623	1842.657176	1564.776427	1554.988627	1677.478617	1726.84994	1724.505478	1782.999557
**ENSG00000089091**	DZANK1	81.56138258	80.57369362	64.32442434	84.56059894	102.8652164	47.42271916	210.4072742	182.74439	150.5369405	154.3977228	229.0143275	158.1693155
**ENSG00000089094**	KDM2B	403.5882207	528.9838146	411.1877758	460.4785091	619.5028763	452.6024317	674.120393	843.9468192	575.8858658	613.7942258	889.8448266	615.7607578
**ENSG00000089101**	CFAP61	1.406230734	0	0.814233219	2.511700959	5.778944741	1.896908767	4.08557814	0	0	3.796665314	9.657230676	0.845825217
**ENSG00000089116**	LHX5	18.28099954	38.53524477	42.34012741	30.1404115	86.68417112	34.9031213	18.38510163	89.71088235	10.78613592	15.81943881	89.67428485	16.07067912
**ENSG00000089123**	TASP1	379.6822982	374.8428355	319.179422	408.5700226	404.5261319	340.6848145	472.9056696	609.7017375	484.4381917	544.188695	533.906896	548.9405657
**ENSG00000089127**	OAS1	1451.230118	2911.162583	1420.836968	118.8871787	263.5198802	144.544448	1211.373918	1785.911084	1255.412429	246.1504679	303.5129641	274.8931955
**ENSG00000089154**	GCN1	3176.675228	2707.976746	3336.727733	3111.160254	3082.489125	3657.998865	4152.990179	3585.112669	4535.335675	4178.230178	4096.045411	4303.558703
**ENSG00000089157**	RPLP0	10400.48251	9227.439521	9524.085968	11496.05529	11154.51914	11852.26535	11128.09346	12135.88881	13202.69933	11715.87638	11941.85553	12182.4206
**ENSG00000089159**	PXN	5111.648719	4953.530556	5855.965314	5713.282447	5829.799455	6504.120779	7851.45979	14237.44929	7915.61688	7883.142748	15109.4272	7383.208317
**ENSG00000089163**	SIRT4	1.406230734	7.006408141	8.956565414	15.07020575	31.2063016	12.89897961	14.29952349	14.95181373	13.13094808	16.45221636	19.31446135	11.84155303
**ENSG00000089177**	KIF16B	554.0549093	847.775385	732.8098975	632.9486416	584.8292078	608.5283323	544.4032871	533.2813562	538.8378337	641.6364381	616.6831589	565.0112448
**ENSG00000089195**	TRMT6	829.6761332	714.6536304	832.9605835	889.1421393	766.2880727	888.1326845	832.4365459	694.4286819	810.836044	899.8096795	729.8107182	890.6539533
**ENSG00000089220**	PEBP1	1157.327894	1138.541323	1035.704655	1360.504686	1399.660416	1407.885687	1404.417485	1603.166694	1656.375308	1769.246036	1680.358138	2112.871392
**ENSG00000089225**	TBX5	70.31153671	87.58010176	61.88172468	53.58295378	83.21680428	64.87427982	121.5459497	264.1487091	124.2750443	100.6116308	360.0767438	110.8031034
**ENSG00000089234**	BRAP	447.1813735	518.4742024	529.2515926	486.4327523	436.8882224	494.7138063	847.7574639	863.8825708	795.8292462	795.4013833	885.7060134	762.0885203
**ENSG00000089248**	ERP29	1448.417656	1625.486689	1755.486821	1162.08031	1523.329834	1407.506305	2266.474473	2432.161699	2458.770028	2262.812527	2537.092459	2315.023618
**ENSG00000089280**	FUS	9459.714149	7048.44659	8648.785257	9159.336162	6623.826463	7558.043289	9903.44141	7849.702206	9112.877968	10098.49696	8441.799215	9131.52904
**ENSG00000089289**	IGBP1	814.2075951	938.8586909	994.9929942	761.0453905	939.6564149	825.5346952	960.1108628	1164.580158	934.1731635	909.3013428	1197.496604	909.262108
**ENSG00000089327**	FXYD5	4515.406887	5177.735616	5277.045495	3301.212293	4247.524385	4203.549827	978.4959644	1016.723333	1225.398833	954.8613265	826.383025	1116.489286
**ENSG00000089335**	ZNF302	423.275451	455.4165292	473.0695005	370.0572746	307.4398602	301.9878756	622.0292717	622.9922385	564.161805	523.3070358	586.3318625	508.3409553
**ENSG00000089351**	GRAMD1A	2.812461468	28.02563256	32.56932878	12.55850479	50.85471372	31.10930377	9.192550814	14.95181373	9.848211061	17.08499391	26.21248326	21.14563042
**ENSG00000089356**	FXYD3	88.59253625	73.56728548	49.66822639	46.04785091	45.07576898	40.21446585	35.74880872	41.5328159	36.11010722	40.49776335	42.76773585	15.2248539
**ENSG00000089505**	CMTM1	33.74953762	7.006408141	36.64049488	39.34998168	52.01050267	43.62890163	18.38510163	23.25837691	46.42728071	25.94387965	49.66575776	27.91223215
**ENSG00000089597**	GANAB	4049.944514	4210.851293	4247.854706	4216.308676	4366.570647	4491.121196	4549.291258	4744.708889	5165.15222	4857.200492	5559.805661	5107.092659
**ENSG00000089639**	GMIP	120.9358431	133.1217547	114.8068839	150.7020575	130.6041512	144.9238298	80.69016826	116.2918845	113.9578708	136.6799513	150.3768777	110.8031034
**ENSG00000089682**	RBM41	458.4312193	406.3716722	479.5833663	494.8050888	388.3450866	464.7426478	472.9056696	426.9573475	456.7694082	452.43595	462.1674681	468.5871701
**ENSG00000089685**	BIRC5	1061.704204	623.5703245	1023.491157	1612.512015	1341.870969	1633.61783	1177.667899	1234.355289	1232.43327	1394.641725	1349.253086	1655.279949
**ENSG00000089692**	LAG3	0	0	0	3.348934611	3.467366845	1.517527013	0	1.661312636	6.56547404	1.898332657	0	2.53747565
**ENSG00000089693**	MLF 2.00	705.9278285	550.0030391	749.0945619	791.185802	729.3028264	842.2274923	713.9547799	691.1060566	798.1740583	771.9886139	757.4028059	795.921529
**ENSG00000089723**	OTUB2	89.99876699	115.6057343	72.46675653	62.79252397	91.30732691	77.01449592	26.55625791	41.5328159	26.26189616	34.16998783	22.07367012	39.75378519
**ENSG00000089737**	DDX24	1134.828202	1226.121425	1139.112274	1074.170777	1057.546888	1151.803003	1284.914325	1611.473257	1364.680676	1373.127289	1361.669525	1303.416659
**ENSG00000089775**	ZBTB25	393.7446056	613.0607123	432.3578395	412.7561909	506.2355593	369.5178277	619.9864827	850.5920697	595.5822879	615.6925585	851.2159039	589.5401761
**ENSG00000089818**	NECAP1	199.6847643	199.682632	228.7995347	239.4488247	225.3788449	241.6661769	217.5570359	272.4552723	250.4259384	237.2915821	204.1814486	235.1394103
**ENSG00000089820**	ARHGAP4	2.812461468	0	8.142332194	0	1.155788948	0.379381753	12.25673442	14.95181373	7.034436472	8.226108181	16.55525259	11.84155303
**ENSG00000089847**	ANKRD24	1.406230734	3.50320407	2.442699658	0.837233653	4.623155793	2.27629052	4.08557814	1.661312636	2.344812157	1.898332657	4.138813147	0.845825217
**ENSG00000089876**	DHX32	975.9241295	1166.566955	973.0086972	864.0251298	968.5511386	850.9532727	1001.988039	1327.388796	894.7803192	919.4257836	1156.108472	953.2450193
**ENSG00000089902**	RCOR1	766.3957501	777.7113036	845.1740818	836.3964192	863.3743444	913.551262	767.0672957	928.6737636	764.8777257	793.5030507	964.3434632	902.4955063
**ENSG00000089916**	GPATCH2L	1245.92043	1716.569995	1445.263965	1085.054814	1251.719431	1283.448471	1236.908782	1621.441133	1159.744093	1268.086215	1662.423281	1168.084624
**ENSG00000090006**	LTBP4	64.68661377	73.56728548	47.22552673	43.53614995	63.56839215	49.69900968	41.87717593	34.88756536	42.67558126	40.49776335	40.00852709	37.21630954
**ENSG00000090013**	BLVRB	150.4666886	238.2178768	216.5860364	146.5158893	198.7956991	210.5568731	280.8834971	292.391024	267.3085859	241.721025	314.5497992	274.8931955
**ENSG00000090020**	SLC9A1	808.5826721	1043.954813	824.8182513	934.3527566	1239.005753	958.3183088	686.3771274	1094.805027	806.1464197	803.6274915	1049.878935	880.5040507
**ENSG00000090054**	SPTLC1	1988.410258	2073.89681	2058.381579	2085.549029	1792.628659	2020.207836	2113.265293	2109.867048	2036.234877	2035.012608	2011.463189	2170.387506
**ENSG00000090060**	PAPOLA	3495.889605	3583.777764	3275.660242	3845.414168	4046.417108	3862.48563	4318.456093	4495.511993	4053.711257	4172.53518	4508.547121	4454.961417
**ENSG00000090061**	CCNK	857.8007478	917.8394665	959.1667325	787.8368673	901.5153796	889.6502115	1031.60848	1113.079466	1008.73819	1033.958521	1105.06311	1062.356472
**ENSG00000090097**	PCBP4	54.84299863	84.07689769	62.6959579	81.21166433	93.61890481	97.88049235	33.70601965	61.46856754	43.14454369	55.05164706	68.98021912	49.05786257
**ENSG00000090238**	YPEL3	576.554601	1033.445201	604.1610488	662.2518194	766.2880727	597.9056432	1103.106098	790.7848148	930.8904264	1066.230176	876.0487828	978.6197758
**ENSG00000090263**	MRPS33	563.8985244	574.5254675	514.5953947	514.0614629	476.1850467	449.1879959	412.6433921	485.1032897	426.2868502	413.8365193	460.7878637	471.1246457
**ENSG00000090266**	NDUFB2	502.0243721	571.0222635	428.2866734	609.5060993	673.8249568	550.482924	426.9429156	440.2478486	508.3552757	521.4087032	438.7141936	552.3238666
**ENSG00000090273**	NUDC	1867.474415	1359.243179	1818.997012	2257.181928	2159.013755	2119.226474	1643.423807	1245.984477	1596.348117	1656.611632	1414.094492	1785.537033
**ENSG00000090316**	MAEA	552.6486785	490.4485699	593.576017	604.4826974	490.0545141	638.8788726	435.1140719	348.8756536	432.8523242	448.0065071	387.6688314	436.4458119
**ENSG00000090339**	ICAM1	6017.261311	10779.35892	11041.81669	73.67656145	194.1725433	177.9300423	683.3129438	1109.756841	796.2982086	69.60553076	93.813098	90.5032982
**ENSG00000090372**	STRN4	620.1477538	458.9197332	672.5566393	648.856081	558.246062	696.9242808	524.9967909	490.0872277	564.6307675	562.5392441	624.9607852	553.1696918
**ENSG00000090432**	MUL1	428.9003739	290.7659378	555.3070557	604.4826974	453.0692677	647.2252711	521.9326073	513.3456046	543.0584956	520.7759256	539.4253135	540.4823135
**ENSG00000090447**	TFAP4	157.4978422	77.07048955	132.7200148	157.3999267	131.7599401	126.7135056	180.7868327	73.09775599	201.1848831	187.9349331	131.0624163	181.0065964
**ENSG00000090470**	PDCD7	314.9956845	283.7595297	298.8235915	315.6370871	284.3240813	330.8208889	237.9849266	204.3414542	272.4671727	264.5010169	249.7083932	268.9724189
**ENSG00000090487**	SPG21	950.6119763	630.5767327	850.0594811	854.8155596	814.8312085	879.4069042	590.3660412	627.9761765	596.5202128	531.533144	611.1647414	560.7821187
**ENSG00000090520**	DNAJB11	1068.735358	753.1888751	1047.10392	724.2071097	564.0250068	688.5778822	643.478557	684.4608061	676.7127886	622.6531115	489.7595557	733.3304629
**ENSG00000090530**	P3H2	1785.913032	1334.720751	1504.70299	2357.649966	1990.268569	2340.406036	1778.247885	1515.117124	2264.619581	1907.191543	1666.562094	2610.216619
**ENSG00000090534**	THPO	4.218692202	0	0	0	0	2.27629052	0	1.661312636	0.937924863	1.265555105	0	0

**ENSG00000090539**	CHRD	0	3.50320407	13.02773151	0.837233653	0	0	1.021394535	1.661312636	0	0	4.138813147	0
**ENSG00000090554**	FLT3LG	7.031153671	45.54165292	3.256932878	1.674467306	4.623155793	3.41443578	6.128367209	6.645250545	7.034436472	1.898332657	1.379604382	1.691650434
**ENSG00000090565**	RAB11FIP3	217.9657638	217.1986524	205.1867713	238.6115911	273.9219807	287.571369	326.8462512	292.391024	321.708228	386.6270845	384.9096227	364.5506684
**ENSG00000090581**	GNPTG	269.996301	290.7659378	335.4640864	238.6115911	290.103026	286.4332237	281.9048916	317.3107135	256.0534876	276.5237904	266.2636458	268.9724189
**ENSG00000090612**	ZNF268	670.7720602	546.499835	546.3504902	505.6891263	537.4418609	467.7777018	1270.614801	1064.9014	1072.517081	1107.993494	1222.329483	1115.643461
**ENSG00000090615**	GOLGA3	2213.407176	1586.951444	2315.679276	1972.522486	1893.182297	2218.245112	2558.59331	2141.431988	2586.327809	2560.850754	2327.392593	2539.167301
**ENSG00000090621**	PABPC4	3869.94698	2876.130542	3530.51524	4738.742475	4122.699178	4493.018104	3100.953808	2362.386569	3465.632368	3701.115904	3119.285508	4284.950548
**ENSG00000090661**	CERS4	56.24922937	0	13.02773151	8.372336529	1.155788948	0.379381753	9.192550814	8.306563181	37.04803208	5.062220419	4.138813147	19.45397999
**ENSG00000090674**	MCOLN1	113.9046895	42.03844885	129.4630819	134.7946181	121.3578396	185.5176774	128.6957114	98.01744553	152.8817527	170.8499391	182.1077785	151.4027138
**ENSG00000090686**	USP48	1965.910566	2126.444871	1823.068178	1887.961887	1843.483372	1759.19319	1855.87387	2068.334232	1854.746416	1881.247663	1793.485697	1955.547901
**ENSG00000090776**	EFNB1	326.2455303	332.8043867	349.3060511	386.8019476	350.2040513	368.7590642	344.2099583	506.700354	288.4118953	379.6665314	702.2186306	276.5848459
**ENSG00000090857**	PDPR	847.9571327	966.8843234	840.2886825	859.8389615	1154.633159	957.9389271	1093.913547	1049.949586	982.4762939	1052.30907	1085.748649	936.328515
**ENSG00000090861**	AARS	5426.644403	5041.110657	5970.772198	3693.874876	3900.7877	4624.284191	5252.010698	4743.047576	5600.818319	4013.075237	4017.407961	4226.588608
**ENSG00000090863**	GLG1	3026.20854	3121.354827	3180.394955	3785.970578	4330.741189	4129.191003	4087.620929	4769.628578	4287.723511	4043.44856	5264.570323	4653.730343
**ENSG00000090889**	KIF4A	1631.227652	1257.650261	1617.067174	2095.595833	2065.394851	2053.593431	1833.40319	1918.816095	1895.077186	1765.449371	1843.151455	2336.169249
**ENSG00000090905**	TNRC6A	3990.882824	3517.216887	3896.920188	4253.146957	3668.474122	3883.351627	4132.562288	3960.569325	3806.568056	4191.518507	4301.606464	3882.337745
**ENSG00000090924**	PLEKHG2	531.5552175	423.8876925	402.2312104	373.4062092	508.5471372	344.478632	99.07526988	119.6145098	81.13050064	92.38552265	78.63744979	76.12426951
**ENSG00000090932**	DLL3	5.624922937	0	5.699632536	11.72127114	4.623155793	11.3814526	1.021394535	4.983937908	0	1.898332657	0	0.845825217
**ENSG00000090971**	NAT14	59.06169083	73.56728548	78.98062229	82.88613163	70.50312584	71.70315137	62.30506663	13.29050109	47.36520558	47.45831643	42.76773585	34.67883389
**ENSG00000090975**	PITPNM2	281.2461468	161.1473872	262.1830967	236.9371238	227.6904228	274.2930076	58.21948849	68.11381808	76.44087633	81.62830426	52.42496653	61.74524082
**ENSG00000090989**	EXOC1	992.7988983	868.7946095	959.9809657	747.649652	780.1575401	756.8665978	1027.522902	799.091378	830.0635037	883.9902407	841.5586732	828.0628872
**ENSG00000091009**	RBM27	642.6474455	465.9261414	557.7497553	586.063557	635.6839215	623.3242207	752.7677722	646.2506155	760.6570638	778.949167	663.5897079	872.0457985
**ENSG00000091010**	POU4F3	0	0	0.814233219	1.674467306	2.311577897	1.13814526	0	3.322625272	2.813774589	1.898332657	0	0
**ENSG00000091039**	OSBPL8	1830.912416	1975.807096	1920.776165	1630.931156	1693.230809	1933.708797	1944.735194	1589.876193	1739.850621	1985.023182	1745.199544	2217.753718
**ENSG00000091073**	DTX2	144.8417656	143.6313669	184.8309408	140.6552537	125.9809954	201.8310928	112.3533988	93.03350762	108.7992841	66.441643	77.25784541	81.19922081
**ENSG00000091127**	PUS7	666.553368	511.4677943	574.8486529	743.4634837	611.4123536	629.014947	660.8422641	556.5397331	616.6855974	583.4209033	583.5726537	730.7929873
**ENSG00000091128**	LAMB4	2.812461468	0	7.328098975	15.9074394	12.71367843	11.00207085	1574.990373	770.8490632	2190.054555	2264.71086	1386.502404	2702.411568
**ENSG00000091129**	NRCAM	0	0	3.256932878	0	0	0.758763507	14.29952349	4.983937908	3.28273702	14.5538837	8.277626294	2.53747565
**ENSG00000091136**	LAMB1	4705.248036	2904.156174	3872.493192	4794.83713	4387.374848	4064.696105	4139.71205	4728.095762	4817.182096	4686.98333	5976.446184	5212.820811
**ENSG00000091137**	SLC26A4	12.65607661	0	11.39926507	14.2329721	8.090522638	10.62268909	6.128367209	11.62918845	12.19302322	11.38999594	5.518417529	8.458252168
**ENSG00000091140**	DLD	1333.106736	1289.179098	1161.096571	1459.298257	1322.222557	1259.547421	1562.733638	1528.407625	1507.714217	1670.532738	1782.448862	1675.579754
**ENSG00000091157**	WDR7	538.5863712	525.4806106	591.9475505	542.5274071	565.1807957	596.3881162	629.1790335	634.621427	606.8373863	667.5803178	593.2298844	607.3025056
**ENSG00000091164**	TXNL1	1214.983354	1212.108608	1330.457081	1157.056908	1337.247813	1314.178393	908.0197415	980.1744553	942.1455248	893.481904	859.4935302	1017.527736
**ENSG00000091262**	ABCC6	0	0	0	0.837233653	2.311577897	1.517527013	0	0	0	2.53111021	0	0
**ENSG00000091317**	CMTM6	898.5814391	952.8715072	946.139001	751.8358203	873.7764449	860.0584347	999.9452496	1074.869276	1056.103396	987.7657593	1105.06311	1140.172392
**ENSG00000091409**	ITGA6	13193.25675	11101.6537	11141.15314	16424.01257	15117.71944	15257.59597	14082.98785	16172.87851	14391.5191	15885.88045	17203.66665	18472.82273
**ENSG00000091428**	RAPGEF4	2.812461468	10.50961221	0	2.511700959	12.71367843	3.41443578	12.25673442	64.79119281	1.406887294	8.226108181	22.07367012	10.99572782
**ENSG00000091436**	MAP3K20	970.2992066	770.7048955	966.4948315	1126.079263	899.2038017	1170.772091	606.7083537	614.6856754	807.553307	697.3208627	713.2554657	920.2578359
**ENSG00000091482**	SMPX	7.031153671	10.50961221	15.47043117	0	3.467366845	4.173199286	0	1.661312636	0.937924863	2.53111021	0	0
**ENSG00000091483**	FH	1222.014508	1005.419568	1253.104925	1583.208838	1457.449864	1384.364018	1234.865993	1337.356672	1340.294629	1375.658399	1105.06311	1511.489662
**ENSG00000091490**	SEL1L3	44.99938349	150.637775	122.9492161	51.90848648	98.2420606	81.56707696	186.9151999	252.5195207	180.0815737	100.6116308	153.1360864	133.6403843
**ENSG00000091513**	TF	0	0	1.628466439	3.348934611	0	0	2.04278907	0	1.875849726	1.898332657	2.759208765	7.612426951
**ENSG00000091527**	CDV3	5318.364637	4060.213518	5068.601791	5344.89964	4890.14304	5510.519967	4981.341147	4879.275212	5062.91841	4474.370073	4515.445143	5277.103527
**ENSG00000091536**	MYO15A	0	14.01281628	8.142332194	2.511700959	19.64841212	6.070108053	1.021394535	4.983937908	1.406887294	0.632777552	4.138813147	3.383300867
**ENSG00000091542**	ALKBH5	940.7683612	1173.573364	1029.190789	1110.171824	1515.239311	1201.122631	878.3993	1510.133186	1017.179514	987.7657593	1550.675326	1073.3522
**ENSG00000091592**	NLRP1	1522.947885	1205.1022	1661.850001	1058.263337	1087.5974	1282.310326	63.32646116	98.01744553	72.68917688	66.441643	31.73090079	45.67456171
**ENSG00000091622**	PITPNM3	64.68661377	105.0961221	73.28098975	65.30422492	80.90522638	44.38766514	59.24088302	96.3561329	89.10286198	77.83163894	85.5354717	38.06213475
**ENSG00000091640**	SPAG7	265.7776088	206.6890402	282.5389271	360.8477044	325.9324834	299.7115851	217.5570359	254.1808333	271.5292478	275.8910128	216.597888	235.1394103
**ENSG00000091651**	ORC6	403.5882207	402.8684681	397.3458111	447.9200043	398.7471872	448.4292324	393.2368959	342.230403	362.5079595	435.9837336	401.4648753	376.3922215
**ENSG00000091656**	ZFHX4	1025.142205	602.5511001	686.398604	1054.914403	895.7364349	758.0047431	42.89857046	28.24231481	43.14454369	30.37332251	24.83287888	29.60388259
**ENSG00000091664**	SLC17A6	39.37446056	7.006408141	13.02773151	216.8435161	35.8294574	69.0474791	0	1.661312636	7.034436472	0	4.138813147	4.229126084
**ENSG00000091732**	ZC3HC1	309.3707615	241.7210809	344.4206518	318.1487881	248.4946239	355.8600846	322.760673	304.0202124	294.508407	323.9821068	322.8274255	312.9553302
**ENSG00000091831**	ESR1	305.1520693	168.1537954	199.4871388	384.2902467	225.3788449	301.2291121	7.149761744	9.967875817	11.72406079	2.53111021	9.657230676	9.304077385
**ENSG00000091844**	RGS17	2.812461468	0	3.256932878	0	4.623155793	1.896908767	2.04278907	0	0	0	0	0
**ENSG00000091879**	ANGPT2	9.843615139	7.006408141	12.21349829	3.348934611	3.467366845	1.517527013	0	0	0.468962431	0	0	0
**ENSG00000091947**	TMEM101	271.4025317	189.1730198	316.7367224	257.8679651	313.218805	324.3713991	282.9262862	323.955964	343.7494623	273.9926802	280.0596896	251.2100894
**ENSG00000091972**	CD200	21.09346101	105.0961221	74.90945619	11.72127114	45.07576898	31.86806728	750.7249831	511.6842919	692.1885488	597.3420094	335.2438649	587.8485257
**ENSG00000091986**	CCDC80	50.62430643	98.08971397	78.16638907	130.6084498	183.7704428	166.1692079	441.2424391	486.7646024	376.5768325	522.6742583	578.0542362	536.2531874
**ENSG00000092010**	PSME1	3462.140067	3797.473212	3318.814602	835.5591856	1004.380596	832.7429485	3203.093261	3307.673459	3080.14525	1156.084588	1190.598582	1168.084624
**ENSG00000092020**	PPP2R3C	257.3402244	227.7082646	245.0841991	308.1019843	275.0777697	276.9486799	205.3003015	262.4873965	191.336672	265.1337944	180.7281741	222.452032
**ENSG00000092036**	HAUS4	105.4673051	56.05126513	114.8068839	142.329721	120.2020506	103.5712187	148.1022076	186.0670152	129.9025935	141.1093942	176.5893609	143.7902869
**ENSG00000092068**	SLC7A8	28.12461468	59.5544692	41.52589419	47.72231821	41.60840214	45.5258104	292.118837	325.6172767	255.5845251	299.3037823	420.7793366	313.8011554
**ENSG00000092094**	OSGEP	247.4966092	224.2050605	223.9141353	221.0296844	202.2630659	253.4270112	224.7067977	275.7778976	245.2673516	299.3037823	230.3939318	238.5227111
**ENSG00000092096**	SLC22A17	1.406230734	10.50961221	1.628466439	12.55850479	3.467366845	8.725780326	2.04278907	0	2.344812157	4.429442867	1.379604382	3.383300867
**ENSG00000092098**	RNF31	19.68723028	7.006408141	17.91313083	7.535102876	5.778944741	9.484543833	36.77020326	29.90362745	36.11010722	14.5538837	22.07367012	15.2248539
**ENSG00000092108**	SCFD1	1299.357198	1460.836097	1083.744415	1068.310141	1025.184797	1072.891598	1436.080716	1516.778437	1261.039978	1281.374544	1340.97546	1215.450837
**ENSG00000092140**	G2E3	836.7072868	462.4229373	788.1777564	1126.916497	813.6754196	1141.939077	1032.629875	716.0257462	1102.061714	1090.275723	827.7626294	1190.921905

ENSG00000092148	HECTD1	4793.840573	4371.99868	5329.156421	5661.373961	4937.530387	5897.868737	3841.464846	3718.01768	3976.801419	4031.425786	3869.790292	4401.674428
ENSG00000092199	HNRNPC	7230.838435	7272.65165	6755.693022	6984.203132	6752.119036	6612.244578	7354.040651	7088.821018	7483.233519	7313.010173	7013.90868	8319.536832
ENSG00000092200	RPGRIP1	4.218692202	10.50961221	1.628466439	5.023401917	3.467366845	1.13814526	7.149761744	0	1.875849726	9.491663286	1.379604382	3.383300867
ENSG00000092201	SUPT16H	3885.415518	3604.796988	3532.143706	4167.749124	3867.269821	3507.384309	5265.288827	5037.099913	5077.925208	5153.340387	4860.346239	5738.078271
ENSG00000092203	TOX4	1029.360897	1142.044527	1187.966267	951.0974297	1182.372094	1074.788507	1434.037927	1651.34476	1385.783985	1339.590078	1587.924644	1407.453161
ENSG00000092208	GEMIN2	91.40499772	70.06408141	96.89375311	182.5169363	97.08627165	141.1300122	210.4072742	152.8407625	224.6330047	277.1565679	161.4137127	220.7603816
ENSG00000092295	TGM1	19.68723028	63.05767327	30.12662912	16.74467306	25.42735686	22.00414169	72.51901198	312.3267756	136.4680676	99.34607572	411.1221059	118.4155303
ENSG00000092330	TINF2	258.7464551	395.86206	316.7367224	269.5892362	248.4946239	267.8435178	375.8731888	428.6186601	436.1350613	407.5087437	529.7680828	335.7926111
ENSG00000092345	DAZL	2.812461468	0	4.071166097	0.837233653	2.311577897	2.27629052	4.08557814	0	1.406887294	3.163887762	2.759208765	5.074951301
ENSG00000092421	SEMA6A	2.812461468	28.02563256	13.02773151	5.023401917	8.090522638	15.17527013	824.2653896	1495.181373	689.3747742	610.630338	1356.151108	669.8935717
ENSG00000092439	TRPM7	2375.12371	2511.797318	2401.173764	2154.202189	2070.018006	2009.205766	2367.592532	2116.512298	2206.937202	2298.880848	2272.208418	2357.314879
ENSG00000092445	TYRO3	140.6230734	168.1537954	160.4039442	213.4945815	238.0925233	251.5301024	115.4175824	184.4057026	131.3094808	223.370476	235.9123494	230.9102842
ENSG00000092470	WDR76	1112.328511	1362.746383	1110.614111	1283.47919	1348.805703	1336.561917	981.560148	1106.434216	904.1595678	896.0130142	989.1763421	916.0287098
ENSG00000092529	CAPN3	42.18692202	45.54165292	35.01202844	12.55850479	11.55788948	7.587635066	15.32091802	8.306563181	4.220661883	5.694997971	6.898021912	6.766601734
ENSG00000092531	SNAP23	590.6169083	627.0735286	675.8135721	745.9751847	900.3595907	751.5552533	744.5966159	729.3162473	652.3267422	801.0963813	728.4311139	746.8636664
ENSG00000092621	PHGDH	1660.758497	1888.226994	1907.748433	1234.082404	1770.668669	2097.981096	2218.46893	2011.849602	2243.04731	1628.76942	1513.426007	1293.266756
ENSG00000092758	COL9A3	12.65607661	0	13.84196473	16.74467306	18.49262317	17.0721789	2.04278907	0	4.220661883	4.429442867	1.379604382	4.229126084
ENSG00000092820	EZR	16434.61859	12373.31678	16028.18092	18100.15434	14138.7662	18657.23586	12017.7281	16873.95245	12972.90774	11423.53315	15383.96847	12831.16854
ENSG00000092841	MYL6	2300.593481	2315.617891	2572.16274	2584.540286	2561.228309	2763.037309	2484.031509	2518.549956	2539.900529	2602.614073	2688.848941	2490.109438
ENSG00000092847	AGO1	2646.526242	2084.406422	2747.222882	4130.07361	3926.215057	3967.194994	2424.790626	2015.172228	2500.976647	2819.656773	2618.489118	3067.808061
ENSG00000092853	CLSPN	3450.890222	4606.713353	3778.042138	3800.20355	3772.495127	3564.291572	2857.861909	3643.258611	2920.698023	2646.275724	3466.945813	3177.765339
ENSG00000092871	RFFL	539.9926019	672.6151815	581.3625187	449.5944716	493.5218809	462.4663573	465.7559079	383.7632189	384.0802314	364.4798702	372.4931832	357.7840667
ENSG00000092929	UNC13D	260.1526858	241.7210809	268.6969624	407.7327889	609.1007757	519.753002	312.5467277	297.3749619	352.190786	424.5937376	470.4450944	340.8675624
ENSG00000092931	MFSD11	224.9969175	241.7210809	205.1867713	218.5179834	195.3283323	191.2084037	364.637849	322.2946514	349.8459739	389.7909723	353.1787219	373.0089206
ENSG00000092964	DPYSL2	1998.253873	3215.941337	2054.310413	3117.02089	4227.875973	3489.173985	1372.754255	2320.853753	1602.913591	1632.566085	2428.103713	1809.220139
ENSG00000092969	TGFB2	424.6816817	465.9261414	416.8874084	819.6517462	784.7806959	860.8171982	640.4143734	516.6682298	583.3892647	532.1659216	492.5187645	623.3731848
ENSG00000092978	GPATCH2	788.8954419	774.2080996	779.221191	811.2794096	761.6649169	788.7346651	801.7947099	775.8330011	778.0086738	751.1069547	876.0487828	765.4718212
ENSG00000093000	NUP50	2979.802926	2827.085685	2645.44373	2395.325481	2118.561142	2324.472002	1600.525236	1696.200201	1658.251158	1588.271656	1484.454315	1869.273729
ENSG00000093009	CDC45	1074.360281	1415.294444	1069.088217	1157.894142	1174.281571	1220.850482	981.560148	1058.256149	1004.986491	1026.36519	1146.451242	992.1529793
ENSG00000093010	COMT	1445.605195	1233.127833	1200.993999	1949.079944	1952.127534	1911.325273	1129.662356	1334.034047	1508.652142	1485.761693	1581.026622	1584.230631
ENSG00000093072	ADA2	12.65607661	14.01281628	10.58503185	11.72127114	31.2063016	21.24537819	1.021394535	9.967875817	2.813774589	0	4.138813147	1.691650434
ENSG00000093100	AC016026.1	18.28099954	0	8.956565414	14.2329721	11.55788948	20.86599643	19.40649616	8.306563181	11.72406079	16.45221636	26.21248326	12.68737825
ENSG00000093144	ECHDC1	1105.297357	1219.115017	1305.215851	984.5867758	972.0185055	1017.122481	979.5173589	1025.029896	1052.351696	908.6685652	1058.156561	977.7739506
ENSG00000093167	LRRFIP2	1029.360897	1135.038119	1006.392259	1044.030365	1032.119531	929.1059138	1103.106098	1292.501231	1036.875936	1009.912974	1117.47955	1067.431424
ENSG00000093183	SEC22C	511.8679872	437.9005088	456.7848361	617.0412022	566.3365846	504.9571136	622.0292717	608.0404248	638.7268316	642.2692157	604.2667195	586.1568752
ENSG00000093217	XYLB	140.6230734	206.6890402	198.6729055	202.610544	167.5893975	221.5589439	329.9104348	232.5837691	292.6325572	304.9987802	223.4959099	268.1265937
ENSG00000094631	HDAC6	511.8679872	630.5767327	501.5676632	521.5965657	585.9849968	478.7797727	610.7939319	779.1556263	493.8174403	596.7092319	834.6606513	518.4908579
ENSG00000094755	GABRP	2.812461468	0	2.442699658	2.511700959	0	0	183.8510163	127.921073	96.13729845	96.81496551	63.46180159	35.5246591
ENSG00000094796	KRT31	0	0	2.442699658	3.348934611	0	1.896908767	0	0	0	0	0	0
ENSG00000094804	CDC6	1702.945419	2252.560217	1678.948898	1538.835454	1724.437111	1553.56828	1212.395313	1486.874809	1204.295524	1207.33957	1364.428734	1395.611608
ENSG00000094841	UPRT	84.37384405	129.6185506	87.12295448	100.4680383	109.7999501	102.8124551	98.05387535	156.1633878	98.95107304	129.0866207	132.4420207	113.340579
ENSG00000094880	CDC23	745.3022891	714.6536304	820.7470852	953.6091306	831.0122538	855.5058537	912.1053196	1108.095528	1003.110641	1004.850753	956.0658369	977.7739506
ENSG00000094914	AAAS	284.0586083	318.7915704	276.8392946	293.8690122	279.7009255	276.5692982	424.9001265	235.9063943	408.4662778	389.1581947	412.5017103	344.2508632
ENSG00000094916	CBX5	7107.09013	6656.087734	7017.876118	8168.051517	9732.898733	8959.100104	7431.666636	8765.085468	7519.343626	7211.765764	8863.958156	7771.442092
ENSG00000094975	SUCO	3431.202991	4606.713353	4128.162423	3433.49521	3569.076272	4150.436381	3369.580571	4159.926841	3182.37906	3083.525013	3920.835655	3522.016203
ENSG00000095002	MSH2	1807.006493	2164.980116	1692.790863	1892.148055	1952.127534	1800.925183	1697.557717	1548.343377	1624.0169	1576.881661	1521.703634	1781.307907
ENSG00000095015	MAP3K1	441.5564505	746.182467	504.8245961	509.8752946	768.5996506	557.3117956	1028.544297	1684.571013	1033.124236	928.9174469	1539.638491	1008.223658
ENSG00000095059	DHPS	39.37446056	28.02563256	32.56932878	25.95424324	27.73893476	18.58970591	23.4920743	34.88756536	33.76529506	31.00610007	37.24931832	28.75805737
ENSG00000095066	HOOK2	396.557067	518.4742024	448.6425039	376.7551438	550.1555394	365.7240102	307.439755	375.4566558	310.9220921	311.9593333	378.0116008	252.9017398
ENSG00000095139	ARCN1	4401.502198	4273.908966	5203.764505	4106.631067	4303.002254	4584.449107	2914.038608	2819.247544	3137.358666	2798.142337	2799.217292	3326.630578
ENSG00000095203	EPB41L4B	767.8019808	599.047896	781.6638907	1072.496309	985.8879729	1100.965848	363.6164544	363.8274673	414.5627894	399.2826356	357.317535	466.0496944
ENSG00000095209	TMEM38B	499.2119106	423.8876925	373.7330477	622.9018377	559.401851	586.9035724	535.2107363	398.7150327	550.5618945	580.2570155	478.7227207	760.3968699
ENSG00000095261	PSMD5	1057.485512	1317.20473	1103.286012	1219.849432	1094.532134	1195.052523	771.1528738	754.2359368	747.0571533	765.6608384	751.8843884	739.2512395
ENSG00000095303	PTGS1	136.4043812	24.52242849	32.56932878	221.866918	143.3178296	96.36296534	24.51346884	151.1794499	71.75125201	43.66165111	259.3656239	88.81164776
ENSG00000095319	NUP188	2297.78102	2231.540993	2287.995347	2975.528402	2493.036761	3146.21288	1889.57989	1774.281895	2270.247131	2101.454251	2048.712508	2461.351381
ENSG00000095321	CRAT	258.7464551	259.2371012	276.8392946	371.7317419	386.0335087	342.961105	299.2685987	279.1005229	329.6805893	338.5359905	282.8188984	295.1930007
ENSG00000095370	SH2D3C	5.624922937	3.50320407	2.442699658	10.04680383	13.86946738	7.967016819	4.08557814	1.661312636	7.972361335	6.327775524	1.379604382	10.1499026
ENSG00000095380	NANS	780.4580575	858.2849973	963.2378986	744.3007174	709.6544142	792.5284827	491.2907713	508.3616667	537.8999089	544.8214726	513.2128302	491.4244509
ENSG00000095383	TBC1D2	430.3066047	385.3524477	492.6110978	399.3604524	346.7366845	470.0539923	243.0918893	220.9545806	268.7154732	212.6132576	238.6715581	258.8225163
ENSG00000095397	WHRN	300.9333771	336.3075908	285.79586	260.379666	402.214554	283.3981697	82.73295732	98.01744553	106.4544719	87.32330223	136.5808338	111.6489286
ENSG00000095485	CWF19L1	662.3346758	476.4357536	711.6398338	610.3433329	564.0250068	640.3963996	567.8953614	573.1528595	616.2166349	599.8731197	580.813445	647.9021161
ENSG00000095539	SEMA4G	8.437384405	3.50320407	6.513865756	7.535102876	6.93473369	5.6907263	1.021394535	3.322625272	7.972361335	11.38999594	12.41643944	10.1499026
ENSG00000095564	BTAF1	1662.164728	1734.086015	1703.375895	1532.974818	1457.449864	1560.01777	1784.376252	1942.074472	1548.044986	1587.006101	1883.159982	1659.509075
ENSG00000095574	IKZF5	451.4000657	497.454978	420.1443412	403.5466207	375.6314082	427.1838542	339.1029856	363.8274673	353.1287109	381.5648641	355.9379306	356.0924163
ENSG00000095585	BLNK	343.1202991	535.9902228	256.4834641	273.7754045	480.8082025	237.1135958	1257.336672	1598.182756	944.9592994	859.9446937	1129.895989	741.7887151
ENSG00000095587	TLL2	2.812461468	0	1.628466439	4.186168264	1.155788948	0.758763507	1.021394535	3.322625272	3.28273702	10.12444084	5.518417529	4.229126084
ENSG00000095637	SORBS1	106.8735358	322.2947745	186.4594073	50.23401917	100.5536385	84.98151274	80.69016826	147.8568246	98.48211061	53.1533144	131.0624163	41.44543562

**ENSG00000095739**	BAMBI	57.6554601	31.52883663	52.92515926	55.25742109	67.035759	88.77533027	3.064183605	9.967875817	1.875849726	5.062220419	0	7.612426951
**ENSG00000095752**	IL11	1.406230734	0	6.513865756	6.697869223	15.02525633	11.00207085	5.106972674	3.322625272	4.689624315	8.226108181	13.79604382	2.53747565
**ENSG00000095777**	MYO3A	4.218692202	0	0	0	0	0.379381753	0	4.983937908	4.689624315	2.53111021	0	0
**ENSG00000095787**	WAC	2421.529324	2455.746053	2480.96862	2320.811686	2429.468369	2410.971042	2072.409511	2282.643562	2060.151961	2004.639286	1936.964553	2083.267509
**ENSG00000095794**	CREM	112.4984587	115.6057343	115.6211172	50.23401917	50.85471372	63.3567528	64.3478557	48.17806645	71.75125201	57.58275727	62.0821972	90.5032982
**ENSG00000095906**	NUBP2	250.3090707	280.2563256	260.5546302	268.7520026	193.0167544	342.961105	221.6426141	201.018829	228.8536665	282.2187884	231.7735362	253.747565
**ENSG00000095951**	HIVEP1	2133.252024	1727.079607	2248.097919	1696.235381	1398.504627	1905.634547	2102.029953	2066.672919	2125.337739	1597.130542	1571.369391	1735.633345
**ENSG00000096060**	FKBP5	447.1813735	283.7595297	302.8947576	584.3890897	532.8187051	352.8250306	385.0657396	262.4873965	260.7431119	496.7303786	362.8359525	362.0131928
**ENSG00000096063**	SRPK1	3076.832846	2599.37742	3199.936552	3690.525942	3066.30808	3377.256368	3050.905476	2241.110746	3093.74516	2937.986176	2334.290615	3338.472131
**ENSG00000096070**	BRPF3	767.8019808	886.3106298	857.3875801	635.4603425	760.509128	788.7346651	769.1100848	574.8141721	662.1749532	632.1447748	703.598235	641.9813395
**ENSG00000096080**	MRPS18A	430.3066047	413.3780803	397.3458111	386.8019476	427.6419109	433.2539623	305.3969659	289.0683987	316.5496412	320.8182191	346.2807	299.4221267
**ENSG00000096092**	TMEM14A	312.183223	350.320407	289.8670261	339.9168631	375.6314082	355.8600846	230.8351649	292.391024	267.7775484	268.9304598	338.0030737	274.0473702
**ENSG00000096093**	EFHC1	338.9016069	634.0799367	294.7524254	412.7561909	569.8039515	366.1033919	827.3295732	965.2226416	730.1745058	830.8369263	1034.703287	694.422503
**ENSG00000096384**	HSP90AB1	22660.00205	19299.15122	22315.68985	25863.822	24825.19082	25333.59596	12928.81202	10747.03144	14821.08868	14495.66817	12203.98037	17559.3315
**ENSG00000096401**	CDC5L	2005.285027	2224.534585	1940.317762	1920.614	2072.329584	1891.21804	1704.707479	1898.880343	1657.313233	1686.352177	1928.686926	1885.344408
**ENSG00000096433**	ITPR3	3456.515145	3786.9636	3410.822956	2481.560547	3109.072271	3016.843702	1409.524458	1427.067554	1673.726918	1435.139489	1364.428734	1447.206946
**ENSG00000096654**	ZNF184	282.6523776	304.7787541	324.0648213	411.9189572	336.3345839	314.1280917	283.9476807	420.3120969	254.1776378	242.3538026	306.2721729	280.813972
**ENSG00000096696**	DSP	18345.68616	18318.25408	15648.74824	23114.34669	22796.78122	19767.68626	35621.1344	76068.18298	32191.45714	32919.61938	75912.73114	33690.06421
**ENSG00000096717**	SIRT1	728.4275203	823.2529565	729.5529646	727.5560443	672.6691679	796.7016819	601.601381	616.346988	609.6511609	620.7547789	691.1817955	768.0092968
**ENSG00000096746**	HNRNPH3	3367.922608	3282.502214	3134.797895	3956.766243	3548.272071	3332.109939	3175.515609	2923.91024	2950.711619	3300.567713	3145.497992	3670.881441
**ENSG00000096872**	IFT74	413.4318358	413.3780803	328.1359874	423.6402284	423.0187551	334.2353247	615.9009045	433.602598	470.8382812	541.6575848	515.972039	467.7413449
**ENSG00000096968**	JAK2	742.4898276	963.3811194	785.7350568	342.428564	298.1935487	318.6806728	263.51979	245.8742701	243.8604644	152.4993901	169.691339	151.4027138
**ENSG00000096996**	IL12RB1	257.3402244	126.1153465	199.4871388	6.697869223	8.090522638	15.55465189	134.8240786	121.2758224	131.7784432	4.429442867	4.138813147	5.074951301
**ENSG00000097007**	ABL1	520.3053716	592.0414879	572.4059533	663.0890531	620.6586652	634.7056733	589.3446466	571.4915468	656.547404	632.7775524	544.943731	647.0562908
**ENSG00000097021**	ACOT7	1767.632033	1306.695118	1694.41933	1872.054448	1551.068769	1873.007716	597.5158029	506.700354	875.5528595	519.5103705	416.6405235	767.1634716
**ENSG00000097033**	SH3GLB1	1382.324812	1632.493097	1237.634494	1257.524947	1380.012004	1177.600962	1314.534766	1583.230942	1116.130587	1169.372917	1369.947152	1234.058991
**ENSG00000097046**	CDC7	804.3639799	651.5959571	610.6749146	828.8613163	854.1280328	751.5552533	1186.86045	1292.501231	1067.358494	1163.677919	1281.652471	1283.116854
**ENSG00000097096**	SYDE2	171.5601496	175.1602035	264.6257963	308.1019843	303.9724934	367.620919	690.4627056	438.5865359	560.879068	675.8064259	474.5839075	696.1141534
**ENSG00000099139**	PCSK5	1.406230734	0	0.814233219	1.674467306	3.467366845	0.758763507	39.83438686	19.93575163	35.17218236	27.8422123	66.22101035	29.60388259
**ENSG00000099194**	SCD	13229.81875	16994.04295	11359.36764	10928.41087	16053.90849	10965.6502	9472.412916	33232.89797	12847.22581	7897.063854	27273.39903	11943.89789
**ENSG00000099203**	TMED1	182.8099954	213.6954483	180.7597747	169.9584315	212.6651665	223.8352344	136.8668677	134.5663235	178.6746864	165.1549412	153.1360864	188.6190233
**ENSG00000099204**	ABLIM1	2854.64839	1558.925811	2261.939884	3334.701639	2528.866219	2825.635299	282.9262862	254.1808333	420.1903386	301.8348925	240.0511625	511.7242561
**ENSG00000099219**	ERMP1	551.2424478	511.4677943	615.5603139	1003.84315	996.2900734	1011.811136	671.0562094	622.9922385	857.2633247	783.3786098	682.9041692	1119.872587
**ENSG00000099246**	RAB18	1027.954667	1373.255996	984.4079623	963.6559345	794.0270075	933.2791131	714.9761744	809.0592538	718.9194074	739.7169587	700.8390262	759.5510447
**ENSG00000099250**	NRP1	16.87476881	49.04485699	126.206149	15.07020575	60.10102531	100.9155464	0	0	0	0	0	0
**ENSG00000099251**	HSD17B7P2	5.624922937	7.006408141	26.86969624	18.41914036	17.33683422	7.967016819	40.8557814	33.22625272	38.45491938	44.92720622	37.24931832	54.97863909
**ENSG00000099256**	PRTFDC1	194.0598413	220.7018564	171.8032093	220.1924507	264.6756692	200.3135657	199.1719343	144.5341993	180.5505361	184.1382677	183.4873828	144.6361121
**ENSG00000099260**	PALMD	28.12461468	3.50320407	14.65619795	33.48934611	48.54313583	36.42064832	106.2250316	63.12988017	153.3507151	129.7193982	120.0255813	230.064459
**ENSG00000099282**	TSPAN15	206.7159179	210.1922442	239.3845665	232.7509555	276.2335586	182.8620051	487.2051931	581.4594226	374.7009827	424.5937376	433.195776	345.0966884
**ENSG00000099284**	H2AFY2	0	0	5.699632536	0	8.090522638	2.27629052	0	0	1.875849726	1.265555105	1.379604382	0
**ENSG00000099290**	WASHC2A	721.3963666	753.1888751	819.932852	670.6241559	640.3070773	702.9943889	554.6172324	601.3951743	584.3271896	592.279789	591.85028	512.5700814
**ENSG00000099308**	MAST3	295.3084542	381.8492437	376.1757474	161.586095	235.7809454	234.8373053	185.8938053	159.4860131	215.253756	161.3582759	189.0058004	187.7731981
**ENSG00000099326**	MZF1	333.276684	343.3139989	267.068496	293.0317785	342.1135287	264.0497003	253.3058446	212.6480174	202.122808	280.9532333	212.4590749	187.7731981
**ENSG00000099330**	OCEL1	57.6554601	42.03844885	47.22552673	40.18721534	56.63365846	56.14849949	58.21948849	56.48462963	39.39284424	50.62220419	67.60061473	51.59533822
**ENSG00000099331**	MYO9B	1259.982738	1534.403383	1428.165067	1484.415267	1386.946738	1423.440338	1327.812895	1355.631111	1492.238457	1450.958928	1629.312776	1377.849278
**ENSG00000099337**	KCNK6	641.2412148	837.2657728	680.6989715	527.4572013	662.2670674	613.0809133	116.438977	172.7765142	130.3715559	134.1488411	136.5808338	150.5568886
**ENSG00000099338**	CATSPERG	33.74953762	52.54806106	32.56932878	26.79147689	42.76419109	21.62475994	38.81299233	63.12988017	34.2342575	32.90443272	38.6289227	21.14563042
**ENSG00000099341**	PSMD8	1088.422588	987.9035479	1081.301715	1136.126067	1125.738436	1198.84634	608.7511428	544.9105446	664.0508029	640.370883	640.1364334	753.6302681
**ENSG00000099364**	FBXL19	624.366446	672.6151815	543.0935574	599.4592955	739.7049269	748.5201993	520.9112128	534.9426688	649.5129676	558.7425788	544.943731	520.1825083
**ENSG00000099365**	STX1B	21.09346101	73.56728548	32.56932878	16.74467306	30.05051265	18.96908767	48.00554314	76.42038126	46.42728071	38.5994307	33.11050518	16.07067912
**ENSG00000099377**	HSD3B7	388.1196826	546.499835	433.1720727	227.7275536	416.0840214	333.8559429	268.6267627	496.7324782	360.1631474	205.019927	191.7650091	194.5397999
**ENSG00000099381**	SETD1A	1129.20328	875.8010176	1108.985645	1054.077169	1064.481621	1028.124551	1154.175824	1184.51591	1184.599102	1182.661245	1099.544693	1155.397246
**ENSG00000099385**	BCL7C	793.1141341	844.272181	850.0594811	998.8197479	908.4501133	1042.92044	789.5379755	837.3015686	944.9592994	970.0479878	791.8929154	969.3156984
**ENSG00000099399**	MAGEB2	0	21.01922442	13.02773151	1.674467306	2.311577897	6.449489806	6.128367209	0	5.158586746	1.265555105	2.759208765	13.53320347
**ENSG00000099622**	CIRBP	1116.547203	907.3298542	965.6805983	1068.310141	982.420606	855.1264719	1375.818438	1013.400708	1208.985148	1340.855633	1189.218978	1195.996857
**ENSG00000099624**	ATP5F1D	275.6212239	262.7403053	220.6572025	262.891367	243.8714681	289.088896	204.278907	215.9706427	229.7915914	236.6588046	231.7735362	195.3856251
**ENSG00000099625**	CBARP	9.843615139	10.50961221	19.54159727	20.09360767	12.71367843	14.79588838	3.064183605	0	7.503398903	6.327775524	6.898021912	5.074951301
**ENSG00000099783**	HNRNPM	3992.289054	3622.313009	3763.38594	4624.878698	3913.501379	4160.300307	4076.385589	4299.477102	4320.081919	4199.111838	4304.365673	4734.083738
**ENSG00000099785**	2-Mar	102.6548436	133.1217547	105.8503185	83.72336529	98.2420606	108.8825632	83.75435186	129.5823856	150.0679781	106.3066288	118.6459769	125.1821321
**ENSG00000099795**	NDUFB7	168.7476881	199.682632	116.4353504	153.2137585	107.4883722	177.5506605	140.9524458	71.43644335	152.8817527	125.2899554	117.2663725	124.3363069
**ENSG00000099797**	TECR	691.8655212	655.0991612	718.1536996	722.5326424	851.8164549	816.0501514	634.2860062	745.9293736	717.9814826	722.6319648	742.2271577	682.5809499
**ENSG00000099800**	TIMM13	330.4642225	171.6569995	309.4086234	462.99021	342.1135287	468.1570836	193.0435671	182.74439	256.0534876	270.1960149	212.4590749	321.4135824
**ENSG00000099804**	CDC34	471.0872959	434.3973047	436.4290056	546.7135753	411.4608656	570.2107752	353.4025091	412.0055338	422.5351507	396.1187478	380.7708095	434.7541614
**ENSG00000099810**	MTAP	1507.479347	1068.477241	1209.950564	1298.549396	1166.191049	1176.842199	823.2439951	581.4594226	707.6643091	676.4392035	593.2298844	760.3968699
**ENSG00000099812**	MISP	748.1147506	588.5382838	872.043778	1025.611225	926.9427365	1307.349522	161.3803365	279.1005229	249.4880135	169.584384	217.9774924	222.452032
**ENSG00000099814**	CEP170B	646.8661377	690.1312019	682.3274379	515.7359302	518.9492378	619.1510214	629.1790335	686.1221187	779.4155611	669.4786504	618.0627633	662.2811447
**ENSG00000099817**	POLR2E	759.3645964	571.0222635	625.3311125	905.0495787	730.4586153	971.2172885	688.4199165	598.072549	915.4146662	849.1874753	746.3659708	920.2578359

**ENSG00000099821**	POLRMT	313.5894537	171.6569995	338.7210193	315.6370871	233.4693675	353.9631758	211.4286687	172.7765142	311.3910545	273.3599026	194.5242179	262.2058172
**ENSG00000099822**	HCN2	0	10.50961221	0	7.535102876	2.311577897	3.035054026	7.149761744	4.983937908	0.937924863	1.898332657	5.518417529	4.229126084
**ENSG00000099840**	IZUMO4	5.624922937	7.006408141	0.814233219	7.535102876	6.93473369	2.655672273	8.171156279	48.17806645	4.220661883	7.593330629	57.94338406	1.691650434
**ENSG00000099849**	RASSF7	104.0610743	252.2306931	175.8743754	134.7946181	194.1725433	155.5465189	153.2091802	244.2129575	202.5917704	129.7193982	213.8386793	155.6318399
**ENSG00000099860**	GADD45B	476.7122189	560.5126513	634.2876779	462.1529764	397.5913982	643.8108354	679.2273657	819.0271296	682.3403378	661.8853198	881.5672003	735.8679386
**ENSG00000099866**	MADCAM1	0	0	0.814233219	0	2.311577897	4.55258104	0	0	5.627549177	3.163887762	12.41643944	5.920776517
**ENSG00000099869**	IGF2-AS	12.65607661	14.01281628	24.42699658	26.79147689	19.64841212	15.93403364	33.70601965	48.17806645	31.88944534	24.04554699	13.79604382	15.2248539
**ENSG00000099875**	MKNK2	968.8929758	1727.079607	890.7711421	767.7432597	1278.302577	810.7388068	1048.972187	1237.677914	1085.179066	834.000814	1109.201923	851.7459933
**ENSG00000099889**	ARVCF	298.1209156	437.9005088	271.9538953	390.9881159	434.5766445	337.2703787	167.5087037	201.018829	208.2193196	222.1049209	242.8103713	250.3642642
**ENSG00000099899**	TRMT2A	629.9913689	423.8876925	667.6712399	691.5549973	639.1512884	852.4707997	589.3446466	534.9426688	659.3611786	630.8792197	509.0740171	609.8399813
**ENSG00000099901**	RANBP1	3434.015453	3128.361235	2840.045469	4026.256637	3171.484874	3556.324555	3169.387242	2601.615588	3493.770114	3346.127697	2848.883049	4157.23094
**ENSG00000099904**	ZDHHC8	317.8081459	311.7851623	288.2385597	301.404115	373.3198303	317.5425275	154.2305748	98.01744553	128.9646686	164.5221636	182.1077785	121.7988312
**ENSG00000099910**	KLHL22	611.7103694	742.6792629	720.5963992	611.1805666	714.27757	706.7882064	767.0672957	1106.434216	779.4155611	899.1769019	1117.47955	780.6966751
**ENSG00000099917**	MED15	2612.776704	2543.326155	3184.466121	2474.862678	2248.009504	2741.412549	2541.229603	3231.253077	2824.560725	2372.915821	3111.007882	2145.858575
**ENSG00000099940**	SNAP29	1009.673667	1166.566955	997.4356938	1286.828124	1301.418356	1157.493729	779.3240301	895.4475109	815.0567059	886.5213509	900.8816616	885.579002
**ENSG00000099942**	CRKL	3246.986765	3927.091763	3577.740766	3106.974086	3814.103529	3457.6853	3528.918118	5510.574014	3901.76743	3726.427006	5379.077487	3863.72959
**ENSG00000099949**	LZTR1	185.6224569	143.6313669	170.1747429	210.1456469	165.2778196	193.8640759	185.8938053	162.8086383	188.5228974	224.6360311	209.6998661	169.1650434
**ENSG00000099953**	MMP11	0	0	0	5.86063557	5.778944741	3.41443578	0	9.967875817	2.344812157	7.593330629	4.138813147	0
**ENSG00000099954**	CECR2	50.62430643	63.05767327	48.85399317	63.62975762	50.85471372	64.87427982	22.47067977	29.90362745	81.13050064	18.35054902	28.97169203	63.43689126
**ENSG00000099956**	SMARCB1	856.3945171	651.5959571	878.5576438	952.771897	815.9869975	1039.506004	751.7463777	681.1381808	653.264667	772.6213915	859.4935302	712.1848325
**ENSG00000099957**	P2RX6	0	14.01281628	0	0	0	3.41443578	39.83438686	33.22625272	36.11010722	16.45221636	15.17564821	9.304077385
**ENSG00000099958**	DERL3	63.28038304	45.54165292	57.81055858	25.95424324	33.5178795	23.5216687	25.53486337	8.306563181	26.73085859	20.24888168	12.41643944	19.45397999
**ENSG00000099968**	BCL2L13	2453.872631	2315.617891	2397.916831	1898.845925	1974.087524	2015.655255	1531.070408	1332.372734	1538.196775	1417.421717	1369.947152	1377.849278
**ENSG00000099974**	DDTL	9.843615139	42.03844885	18.72736405	10.04680383	24.27156791	16.69279715	10.21394535	26.58100218	16.88264753	19.61610412	11.03683506	19.45397999
**ENSG00000099977**	DDT	4.218692202	0	0	0.837233653	1.155788948	2.27629052	1.021394535	1.661312636	1.875849726	2.53111021	6.898021912	0
**ENSG00000099984**	GSTT2	8.437384405	0	9.770798633	3.348934611	4.623155793	2.27629052	0	3.322625272	0	5.062220419	0	5.074951301
**ENSG00000099991**	CABIN1	1053.26682	1149.050935	1143.18344	1044.030365	1346.494125	1086.16996	937.640183	1214.419537	1008.73819	1064.964621	1240.26434	988.7696784
**ENSG00000099992**	TBC1D10A	172.9663803	241.7210809	207.629471	205.9594786	191.8609654	228.7671972	87.83993	122.9371351	121.4612697	125.9227329	93.813098	131.1029086
**ENSG00000099994**	SUSD2	0	7.006408141	0	7.535102876	1.155788948	3.41443578	1.021394535	0	2.344812157	0.632777552	0	0
**ENSG00000099995**	SF3A1	3467.76499	3422.630377	3765.014407	3724.015288	3747.06777	3878.799046	2522.844501	2437.145637	2585.389885	2567.811308	2939.936939	2910.484571
**ENSG00000099999**	RNF215	139.2168427	217.1986524	148.1904459	145.6786556	176.8357091	195.7609847	89.88271907	66.45250545	64.24785311	92.38552265	121.4051856	81.19922081
**ENSG00000100003**	SEC14L2	57.6554601	168.1537954	62.6959579	77.02549606	130.6041512	68.28871559	2.04278907	9.967875817	1.406887294	8.858885733	4.138813147	6.766601734
**ENSG00000100014**	SPECC1L	431.7128354	395.86206	411.1877758	442.0593687	349.0482624	496.6107151	312.5467277	289.0683987	331.0874766	299.9365598	314.5497992	359.4757171
**ENSG00000100023**	PPIL2	1238.889277	1082.490058	1285.674254	1241.617507	1024.029008	1265.238147	1340.06963	1031.675147	1127.854648	1319.341197	1151.969659	1108.031034
**ENSG00000100024**	UPB1	0	0	0.814233219	0	1.155788948	1.896908767	0	1.661312636	0	1.265555105	5.518417529	1.691650434
**ENSG00000100027**	YPEL1	85.78007478	262.7403053	69.20982365	66.97869223	201.107277	92.94852956	21.44928523	86.38825708	29.54463318	40.49776335	49.66575776	32.98718345
**ENSG00000100028**	SNRPD3	426.0879124	455.4165292	490.1683981	685.6943617	701.5638916	629.014947	679.2273657	692.7673693	574.0100161	682.766979	757.4028059	695.2683282
**ENSG00000100029**	PES 1.00	1521.541654	1163.063751	1408.62347	1373.900424	1334.936235	1443.926953	801.7947099	727.6549346	843.6634142	836.5319242	830.5218381	836.5211394
**ENSG00000100030**	MAPK1	3739.167522	3321.037459	3817.125333	3809.413121	3966.66767	4258.560181	3669.870564	3698.081928	4130.152134	3650.4937	4021.546774	4319.629382
**ENSG00000100031**	GGT1	1.406230734	0	4.885399317	1.674467306	3.467366845	0.758763507	0	0	0.937924863	0	0	0
**ENSG00000100034**	PPM1F	857.8007478	753.1888751	937.1824356	803.7443068	774.3785953	1055.819419	488.2265877	438.5865359	673.899014	605.5681176	462.1674681	648.7479413
**ENSG00000100036**	SLC35E4	175.7788418	126.1153465	152.261612	150.7020575	186.0820207	171.8599342	28.59904698	54.82331699	58.1513415	66.441643	64.84140597	57.51611474
**ENSG00000100038**	TOP3B	458.4312193	346.817203	450.2709704	371.7317419	374.4756192	399.1096045	509.6758729	528.2974183	439.8867607	508.1203746	601.5075107	375.5463962
**ENSG00000100053**	CRYBB3	19.68723028	17.51602035	16.28466439	17.58190671	10.40210053	10.24330734	0	4.983937908	2.813774589	1.265555105	2.759208765	0
**ENSG00000100055**	CYTH4	108.2797665	287.2627338	118.0638168	139.81802	219.5999002	110.779472	0	0	0	0	0	0
**ENSG00000100056**	ESS2	531.5552175	535.9902228	585.4336848	530.8061359	546.6881725	619.1510214	344.2099583	352.1982789	385.9560811	373.9715335	431.8161717	345.9425137
**ENSG00000100058**	CRYBB2P1	334.6829147	357.3268152	336.2783196	266.2403016	262.3640913	259.1177375	287.0118643	352.1982789	291.2256699	261.9699067	335.2438649	270.6640694
**ENSG00000100060**	MFNG	35.15576835	10.50961221	30.94086234	24.27977593	21.95999002	18.58970591	0	0	0	0	0	0
**ENSG00000100065**	CARD10	984.3615139	739.1760589	870.4153116	1136.963301	826.389098	1097.172031	21.44928523	13.29050109	62.37200338	18.98332657	30.35129641	78.66174516
**ENSG00000100068**	LRP5L	109.6859973	119.1089384	95.26528668	74.51379511	55.47786952	92.94852956	64.3478557	81.40431917	79.25465092	59.48108992	95.19270238	61.74524082
**ENSG00000100075**	SLC25A1	1330.294275	1285.675894	1222.164062	1320.317471	1495.590899	1514.871341	952.961101	1197.806411	1043.44141	962.4546572	1251.301175	1074.198025
**ENSG00000100077**	GRK3	32.34330689	59.5544692	30.12662912	12.55850479	19.64841212	14.03712487	8.171156279	16.61312636	13.13094808	10.75721839	35.86971394	9.304077385
**ENSG00000100083**	GGA1	596.2418313	557.0094472	639.9873105	648.0188473	642.6186552	624.8417477	412.6433921	357.1822168	401.4318413	480.2781623	422.158941	415.3001814
**ENSG00000100084**	HIRA	174.372611	140.1281628	175.0601422	159.074394	143.3178296	154.7877553	169.5514928	187.7283279	175.3919494	191.0988208	158.654504	200.4605764
**ENSG00000100092**	SH3BP1	236.2467633	203.1858361	157.1470114	225.2158526	174.5241312	191.2084037	53.11251581	39.87150327	43.61350613	38.5994307	38.6289227	59.20776517
**ENSG00000100095**	SEZ6L	0	0	0.814233219	0	1.155788948	0	4.08557814	26.58100218	4.220661883	6.960553076	11.03683506	5.074951301
**ENSG00000100097**	LGALS1	600.4605235	858.2849973	719.782166	1011.378253	1508.304577	1540.6693	13.27812895	18.274439	12.66198565	6.327775524	34.49010956	14.37902869
**ENSG00000100099**	HPS4	642.6474455	805.7369362	734.4383639	661.4145858	641.4628663	627.8768017	622.0292717	661.2024292	617.1545598	515.7137052	594.6094888	549.7863909
**ENSG00000100100**	PIK3IP1	16.87476881	84.07689769	29.3123959	22.60530863	56.63365846	17.0721789	53.11251581	109.646634	45.48935585	79.7299716	120.0255813	60.05359039
**ENSG00000100104**	SRRD	667.9595987	413.3780803	657.9004413	664.7635204	708.4986253	743.5882365	460.6489352	403.6989706	484.4381917	393.5876376	467.6858856	448.2873649
**ENSG00000100105**	PATZ1	303.7458386	339.8107948	377.8042138	281.3105074	317.8419608	300.0909669	326.8462512	320.6333388	327.3357772	316.3887762	339.382678	290.9638746
**ENSG00000100106**	TRIOBP	1548.260038	1404.784832	1402.923837	1319.480237	1390.414105	1246.648441	1308.406399	985.1583932	1178.50259	1119.38349	1056.776957	850.0543429
**ENSG00000100109**	TFIP11	611.7103694	585.0350798	745.0233958	680.6709598	737.393349	724.6191488	624.0720608	684.4608061	653.7336294	627.715332	572.5358187	646.2104656
**ENSG00000100116**	GCAT	130.7794583	91.08330583	122.9492161	191.7265065	194.1725433	217.7651264	90.9041136	69.77513072	117.7095703	141.1093942	107.6091418	104.8823269
**ENSG00000100121**	GGTLC2	0	0	0	0	0	0	1.021394535	0	5.158586746	1.898332657	0	0.845825217
**ENSG00000100124**	ANKRD54	209.5283794	213.6954483	268.6969624	315.6370871	264.6756692	281.8806427	228.7923758	214.3093301	272.9361351	257.5404638	211.0794705	265.5891181
**ENSG00000100129**	EIF3L	1666.38342	1404.784832	1563.327781	1663.583268	1699.009754	1667.762188	1482.04347	1686.232326	1570.555183	1615.481091	1702.431808	1676.42558

**ENSG00000100138**	SNU13	843.7384405	588.5382838	679.8847382	1016.401655	730.4586153	896.8584648	607.7297483	524.974793	593.7064382	612.5286707	511.8332258	635.2147378
**ENSG00000100139**	MICALL1	2061.534256	1944.278259	2148.761466	1604.976913	1585.742437	1725.807596	2015.211417	1468.60037	2379.515377	1757.223263	1374.085965	1812.60344
**ENSG00000100142**	POLR2F	319.2143767	378.3460396	440.5001717	429.5008639	373.3198303	456.0168675	221.6426141	225.9385185	301.073881	263.2354618	273.1616677	292.655525
**ENSG00000100147**	CCDC134	449.9938349	434.3973047	434.8005392	414.4306582	413.7724435	376.3466993	303.3541769	302.3588998	336.2460634	320.8182191	244.1899757	289.2722241
**ENSG00000100150**	DEPDC5	350.1514528	434.3973047	307.780157	315.6370871	298.1935487	299.3322034	356.4666927	350.5369662	283.722271	294.8743394	349.0399087	270.6640694
**ENSG00000100151**	PICK1	365.6199909	252.2306931	375.3615142	390.1508822	346.7366845	451.0849047	203.2575124	219.293268	217.1296058	270.1960149	216.597888	181.0065964
**ENSG00000100154**	TTC28	0	0	0	0	1.155788948	1.13814526	34.72741419	11.62918845	25.79293373	89.22163489	34.49010956	58.36193996
**ENSG00000100162**	CENPM	129.3732275	112.1025303	70.02405687	142.329721	101.7094274	156.3052824	114.3961879	53.16200436	126.6198565	141.7421717	113.1275593	137.8695103
**ENSG00000100167**	3-Sep	35.15576835	122.6121425	56.18209214	105.4914403	113.2673169	97.12172885	4.08557814	9.967875817	6.56547404	6.327775524	9.657230676	13.53320347
**ENSG00000100170**	SLC5A1	0	7.006408141	12.21349829	4.186168264	17.33683422	12.89897961	0	0	1.406887294	0.632777552	0	0
**ENSG00000100181**	TPTEP1	9.843615139	7.006408141	6.513865756	6.697869223	0	1.517527013	0	9.967875817	5.158586746	2.53111021	5.518417529	7.612426951
**ENSG00000100191**	SLC5A4	8.437384405	0	9.770798633	0.837233653	0	2.27629052	0	0	0.468962431	0	0	0
**ENSG00000100196**	KDELR3	492.180757	714.6536304	611.4891478	405.221088	628.7491879	490.540607	41.87717593	46.51675381	52.99275475	32.90443272	56.56377967	76.12426951
**ENSG00000100197**	CYP2D6	1.406230734	0	10.58503185	2.511700959	2.311577897	0	6.128367209	0	0.468962431	1.265555105	0	0
**ENSG00000100201**	DDX17	16466.9619	17232.26082	16136.47394	14947.96964	13952.68418	13939.24438	14029.87533	14104.54428	13452.65631	13493.34853	13880.19969	12944.50912
**ENSG00000100206**	DMC1	19.68723028	31.52883663	17.91313083	22.60530863	62.41260321	29.59177676	36.77020326	24.91968954	26.73085859	41.1305409	63.46180159	33.83300867
**ENSG00000100207**	TCF20	2126.22087	2028.355157	1852.380574	1877.07785	1824.990749	1914.739709	1438.123505	1569.940441	1599.630854	1457.919481	1432.029349	1580.84733
**ENSG00000100209**	HSCB	108.2797665	126.1153465	124.5776826	117.2127114	142.1620406	145.6825933	76.60459012	141.2115741	110.2061714	85.42496957	114.5071637	86.27417211
**ENSG00000100211**	CBY1	217.9657638	203.1858361	249.1553652	227.7275536	245.027257	231.0434878	239.0063212	237.567707	261.2120743	267.6649047	256.6064151	280.813972
**ENSG00000100216**	TOMM22	1341.54412	837.2657728	1328.014381	1599.116277	1339.559391	1500.834216	781.3668192	712.7031209	970.7522331	913.7307856	765.6804322	971.8531741
**ENSG00000100218**	RSPH14	4.218692202	3.50320407	4.071166097	0.837233653	1.155788948	2.655672273	1.021394535	4.983937908	2.813774589	6.960553076	1.379604382	0
**ENSG00000100219**	XBP1	3359.485224	2792.053644	3700.689982	1743.957699	1374.233059	2469.016451	1546.391326	1335.695359	1727.657597	1268.086215	809.8277724	1387.999181
**ENSG00000100220**	RTCB	1400.605811	1306.695118	1609.739075	1591.581174	1301.418356	1645.378664	1114.341438	1098.127652	1111.440963	1058.636845	1014.009221	1163.009673
**ENSG00000100221**	JOSD1	1691.695573	2350.649931	1704.190128	1516.230145	1694.386598	1755.019991	1181.753477	1119.724717	1175.219853	1153.553478	1178.182142	1114.797636
**ENSG00000100225**	FBXO7	2571.996013	2725.492767	2581.119306	1547.20779	1717.502377	1588.471401	1850.766897	1732.749079	1796.126112	1364.90118	1367.187943	1426.907141
**ENSG00000100226**	GTPBP1	1591.853191	1422.300853	1597.525577	855.6527932	858.7511886	838.4336748	961.1322573	789.1235022	983.4142188	717.5697444	718.7738832	641.1355143
**ENSG00000100227**	POLDIP3	701.7091363	588.5382838	714.8967667	711.6486049	641.4628663	727.6542028	698.6338619	732.6388725	751.7467776	704.9141934	724.2923007	757.013569
**ENSG00000100228**	RAB36	478.1184496	332.8043867	369.6618816	360.0104707	374.4756192	293.2620953	588.3232521	476.7967266	621.8441841	488.5042704	393.187249	515.9533822
**ENSG00000100234**	TIMP3	18558.027	12583.50902	16930.35133	17085.42715	15368.52565	18908.0072	25340.79841	26004.52669	24585.35547	24356.87355	25937.94199	22526.863
**ENSG00000100239**	PPP6R2	448.5876042	479.9389576	466.5556347	459.6412754	449.6019009	462.4663573	397.3224741	360.504842	318.8944534	351.8243191	402.8444796	339.1759119
**ENSG00000100241**	SBF1	697.4904441	518.4742024	822.3755516	720.0209415	815.9869975	964.0090351	468.8200915	440.2478486	604.4925741	495.4648235	478.7227207	482.1203736
**ENSG00000100242**	SUN2	527.3365253	416.8812844	525.1804265	931.003822	1029.807953	1208.710266	974.4103863	843.9468192	1172.406079	1242.775113	1307.864954	1313.566562
**ENSG00000100243**	CYB5R3	908.4250543	865.2914054	969.7517644	993.796346	1044.833209	1118.417409	515.8042401	461.8449128	619.0304095	530.9003664	580.813445	587.0027004
**ENSG00000100246**	DNAL4	74.53022891	119.1089384	74.90945619	97.95633739	135.2273069	92.18976605	83.75435186	96.3561329	110.6751338	100.6116308	82.77626294	93.88659906
**ENSG00000100258**	LMF2	338.9016069	399.365264	385.946546	216.0062824	254.2735686	297.0559128	215.5142469	222.6158932	283.722271	191.7315984	189.0058004	224.1436824
**ENSG00000100263**	RHBDD3	213.7470716	143.6313669	188.0878737	182.5169363	136.3830959	174.5156065	119.5031606	68.11381808	106.4544719	137.9455064	88.29468047	123.4904816
**ENSG00000100266**	PACSIN2	870.4568244	889.8138339	959.9809657	1172.127114	1121.11528	1286.862907	943.7685502	1204.451661	1154.116544	978.9068735	1096.785484	1138.480742
**ENSG00000100271**	TTLL1	28.12461468	14.01281628	12.21349829	5.023401917	26.58314581	13.27836137	21.44928523	19.93575163	15.00679781	18.98332657	9.657230676	17.76232955
**ENSG00000100276**	RASL10A	1.406230734	7.006408141	2.442699658	10.04680383	3.467366845	1.13814526	4.08557814	16.61312636	1.875849726	1.898332657	0	4.229126084
**ENSG00000100280**	AP1B1	1430.136657	1233.127833	1620.324107	1671.955605	1494.43511	1827.102524	1180.732082	1059.917462	1445.342214	1388.946727	1165.765703	1371.082676
**ENSG00000100281**	HMGXB4	1537.010192	1261.153465	1419.208501	1417.436574	1363.830959	1407.885687	892.6988235	825.6723802	891.0286198	923.2224489	972.6210895	943.0951167
**ENSG00000100284**	TOM1	226.4031482	234.7146727	311.851323	267.9147689	320.1535387	314.1280917	326.8462512	342.230403	268.2465108	340.4343232	325.5866342	307.8803789
**ENSG00000100288**	CHKB	1.406230734	0	6.513865756	1.674467306	0	2.655672273	8.171156279	3.322625272	7.034436472	1.898332657	8.277626294	1.691650434
**ENSG00000100290**	BIK	7.031153671	52.54806106	26.86969624	24.27977593	65.87997005	33.76497604	14.29952349	29.90362745	9.848211061	11.38999594	22.07367012	11.84155303
**ENSG00000100292**	HMOX1	142.0293041	220.7018564	192.1590398	120.561646	130.6041512	182.1032416	21.44928523	54.82331699	45.02039342	51.8877593	37.24931832	59.20776517
**ENSG00000100294**	MCAT	102.6548436	45.54165292	115.6211172	141.4924873	159.4988749	146.061975	89.88271907	86.38825708	127.0888189	113.8999594	88.29468047	117.5697051
**ENSG00000100296**	THOC5	1213.577124	984.4003438	1288.116953	1475.205696	1249.407853	1455.687787	875.3351164	632.9601144	847.8840761	898.5441244	765.6804322	885.579002
**ENSG00000100297**	MCM5	1248.732892	1516.887362	1404.552304	1446.739752	1555.691924	1607.440489	1280.828747	1199.467723	1333.729155	1308.583978	1412.714887	1311.874911
**ENSG00000100299**	ARSA	144.8417656	182.1666117	160.4039442	97.95633739	147.9409854	132.4042319	167.5087037	154.5020752	162.2610013	140.4766166	161.4137127	106.5739773
**ENSG00000100300**	TSPO	187.0286876	122.6121425	146.5619795	130.6084498	88.99574902	144.1650663	23.4920743	14.95181373	16.4136851	11.38999594	20.69406573	8.458252168
**ENSG00000100302**	RASD2	0	3.50320407	0.814233219	0.837233653	3.467366845	0.379381753	0	0	1.406887294	0	0	0.845825217
**ENSG00000100304**	TTLL12	472.4935267	357.3268152	371.2903481	565.1327157	524.7281825	626.7386565	415.7075757	378.779281	513.5138624	466.9898337	412.5017103	502.4201788
**ENSG00000100307**	CBX7	300.9333771	308.2819582	277.6535278	287.1711429	343.2693176	321.7157268	327.8676457	187.7283279	282.3153837	236.026027	288.3373159	305.3429033
**ENSG00000100311**	PDGFB	409.2131436	504.4613861	527.6231262	671.4613896	713.1217811	827.8109857	126.6529223	106.3240087	126.1508941	88.58885733	111.747955	113.340579
**ENSG00000100316**	RPL3	12443.73577	12100.06686	10852.10035	13031.54181	12633.92899	12859.90329	8823.827387	10137.32971	9852.900685	9412.566092	10416.01309	9848.788824
**ENSG00000100319**	ZMAT5	91.40499772	136.6249587	121.3207497	102.1425056	142.1620406	136.5774312	59.24088302	109.646634	97.54418574	87.32330223	41.38813147	80.35339559
**ENSG00000100320**	RBFOX2	3318.704533	3783.460396	3364.411663	3925.788598	3913.501379	4146.263182	2351.250219	3056.81525	2536.148829	2512.126883	3413.141242	2828.439525
**ENSG00000100321**	SYNGR1	73.12399818	63.05767327	48.03975995	88.7467672	91.30732691	75.11758715	10.21394535	26.58100218	37.51699452	13.2883286	38.6289227	62.59106604
**ENSG00000100324**	TAB1	281.2461468	210.1922442	337.0925529	397.6859851	407.9934987	340.6848145	269.6481572	245.8742701	286.5360456	233.4949168	307.6517773	277.4306711
**ENSG00000100325**	ASCC2	748.1147506	921.3426705	1021.86269	823.0006808	965.0837718	971.5966702	769.1100848	682.7994934	816.4635932	886.5213509	698.0798174	713.876483
**ENSG00000100330**	MTMR3	1151.702971	1376.7592	1202.622465	1179.662217	1221.668918	1188.982415	922.319265	1058.256149	1083.772179	975.7429858	1117.47955	954.0908445
**ENSG00000100335**	MIEF1	1168.57774	847.775385	1143.997673	1365.528088	1158.100526	1360.083586	840.6077022	787.4621895	1016.241589	1066.230176	933.9921668	1134.251616
**ENSG00000100336**	APOL4	153.27915	133.1217547	128.6488487	0	0	1.13814526	150.1449966	294.0523366	120.0543825	10.12444084	9.657230676	4.229126084
**ENSG00000100341**	PNPLA5	0	0	0	0	0	1.13814526	1.021394535	0	1.875849726	2.53111021	1.379604382	0
**ENSG00000100342**	APOL1	3801.041674	4925.504923	4238.083907	437.0359668	628.7491879	539.860235	2814.963338	2947.168616	2628.065466	820.7124854	888.4652222	690.1933769
**ENSG00000100344**	PNPLA3	0	3.50320407	0.814233219	3.348934611	1.155788948	1.13814526	15.32091802	63.12988017	11.25509835	36.06832049	24.83287888	15.2248539
**ENSG00000100345**	MYH9	15984.62476	15312.50499	17817.86554	15263.60673	16097.82847	18661.40906	10624.54595	15308.99594	12167.69925	10512.33348	15295.67379	11586.95964

**ENSG00000100346**	CACNA1I	11.24984587	3.50320407	13.84196473	0.837233653	1.155788948	1.13814526	0	0	0	0	0	0
**ENSG00000100347**	SAMM50	338.9016069	416.8812844	438.0574721	412.7561909	371.0082524	404.8003308	434.0926773	491.7485403	452.0797839	485.9731602	509.0740171	538.7906631
**ENSG00000100348**	TXN2	521.7116024	444.9069169	468.1841012	568.4816503	575.5828962	547.44787	470.8628806	463.5062255	487.2519663	504.3237092	455.2694462	536.2531874
**ENSG00000100350**	FOXRED2	717.1776744	672.6151815	583.8052183	848.954924	820.6101533	937.8316942	603.6441701	569.8302342	675.3059013	714.4058566	729.8107182	696.9599786
**ENSG00000100351**	GRAP2	0	3.50320407	1.628466439	2.511700959	0	1.13814526	0	0	0.468962431	0	0	0
**ENSG00000100353**	EIF3D	3822.135135	3800.976416	3821.196499	3852.112037	3619.930986	3628.407089	2334.907907	2383.983633	2401.087649	2340.644166	2319.114967	2432.593323
**ENSG00000100354**	TNRC6B	3076.832846	3208.934929	3067.216538	3039.995394	3566.764694	2977.008618	3149.980746	3319.302647	2977.442477	2931.025623	3171.710475	2994.221267
**ENSG00000100359**	SGSM3	5.624922937	10.50961221	3.256932878	8.372336529	3.467366845	16.69279715	7.149761744	4.983937908	13.13094808	10.75721839	1.379604382	4.229126084
**ENSG00000100360**	IFT27	175.7788418	227.7082646	198.6729055	211.8201142	232.3135786	202.2104745	175.67986	186.0670152	165.5437383	186.0366004	183.4873828	187.7731981
**ENSG00000100362**	PVALB	0	0	0	0	0	0	3.064183605	6.645250545	0.468962431	0.632777552	6.898021912	5.074951301
**ENSG00000100364**	KIAA0930	407.8069129	402.8684681	430.7293731	452.1061725	488.8987251	567.5551029	386.0871342	313.9880882	426.2868502	333.4737701	292.476129	404.3044536
**ENSG00000100365**	NCF4	9.843615139	7.006408141	19.54159727	6.697869223	3.467366845	12.89897961	22.47067977	9.967875817	17.8205724	31.00610007	20.69406573	18.60815477
**ENSG00000100368**	CSF2RB	0	3.50320407	5.699632536	9.209570182	4.623155793	4.55258104	23.4920743	16.61312636	22.97915914	24.04554699	13.79604382	17.76232955
**ENSG00000100372**	SLC25A17	689.0530597	437.9005088	577.2913526	803.7443068	543.2208057	687.439737	487.2051931	272.4552723	489.127816	502.4253766	452.5102374	506.6493048
**ENSG00000100376**	FAM118A	165.9352266	98.08971397	171.8032093	153.2137585	150.2525633	152.1320831	155.2519693	129.5823856	158.0403394	177.8104922	136.5808338	186.0815477
**ENSG00000100379**	KCTD17	163.1227652	129.6185506	143.3050466	173.3073661	225.3788449	216.2475994	17.36370709	3.322625272	14.53783538	13.92110615	16.55525259	19.45397999
**ENSG00000100380**	ST13	3059.958078	3114.348419	3193.422687	3318.7942	3259.324834	3272.926386	2521.823107	2752.795038	2729.361351	2626.026842	2577.100986	3106.716021
**ENSG00000100385**	IL2RB	22.49969175	10.50961221	19.54159727	23.44254228	17.33683422	16.69279715	8.171156279	11.62918845	3.751699452	6.327775524	4.138813147	9.304077385
**ENSG00000100387**	RBX1	715.7714437	798.7305281	603.3468156	846.443223	774.3785953	849.4357456	651.6497133	742.6067483	785.9810351	668.2130953	811.2073768	776.467549
**ENSG00000100393**	EP300	3711.042907	3580.27456	3670.563353	3600.104707	3788.676172	3720.596855	3544.239036	3570.160855	3584.279864	3639.736481	3542.824054	3487.337369
**ENSG00000100395**	L3MBTL2	627.1789074	392.3588559	649.7581091	665.600754	597.5428863	723.8603853	546.4460762	415.328159	558.5342559	510.0187072	513.2128302	482.1203736
**ENSG00000100399**	CHADL	0	0	3.256932878	0	9.246311586	2.655672273	1.021394535	3.322625272	0.468962431	0.632777552	0	0.845825217
**ENSG00000100401**	RANGAP1	3275.11138	2322.624299	3456.420017	3338.887808	2661.781948	3824.547455	1946.777983	1634.731634	2231.323249	1911.620986	1732.783104	2233.824398
**ENSG00000100403**	ZC3H7B	2268.250174	1734.086015	2492.367885	1641.815193	1594.988749	1961.024283	1156.218613	900.4314488	1187.412876	940.3074428	869.1507609	957.4741454
**ENSG00000100410**	PHF5A	746.7085198	462.4229373	677.4420386	740.1145491	590.6081526	680.9902472	423.878732	362.1661547	537.4309464	456.2326153	445.6122155	510.8784309
**ENSG00000100412**	ACO2	936.5496689	858.2849973	828.0751842	946.9112614	944.2795707	933.6584949	902.9127688	779.1556263	931.3593889	909.9341203	830.5218381	927.0244376
**ENSG00000100413**	POLR3H	412.0256051	189.1730198	365.5907155	456.2923408	390.6566645	453.740577	351.35972	217.6319553	380.7974943	402.4465233	331.1050518	389.9254249
**ENSG00000100416**	TRMU	337.4953762	287.2627338	306.9659237	364.196639	345.5808955	357.7569934	297.2258096	290.7297113	333.9012512	313.857666	397.3260621	320.5677572
**ENSG00000100417**	PMM1	94.21745919	73.56728548	127.0203822	83.72336529	92.46311586	99.39801937	79.66877372	99.67875817	101.2958852	113.8999594	151.7564821	94.73242428
**ENSG00000100418**	DESI1	617.3352923	448.410121	797.1343218	569.3188839	381.4103529	586.5241906	400.3866577	289.0683987	427.6937375	430.9215132	355.9379306	429.6792101
**ENSG00000100422**	CERK	208.1221487	252.2306931	171.8032093	477.2231821	473.8734688	419.9756009	138.9096567	167.7925762	138.8128797	230.9638066	297.9945466	248.6726137
**ENSG00000100425**	BRD1	472.4935267	360.8300193	462.4844686	419.4540601	381.4103529	486.7467895	446.3494117	450.2157244	469.9003563	454.9670602	418.0201278	450.8248405
**ENSG00000100426**	ZBED4	614.5228308	497.454978	552.864356	674.8103242	646.0860221	668.8500311	454.520568	500.0551035	553.8446315	557.4770236	583.5726537	620.8357091
**ENSG00000100427**	MLC1	1.406230734	24.52242849	3.256932878	3.348934611	0	3.793817533	0	3.322625272	0.468962431	0	4.138813147	1.691650434
**ENSG00000100429**	HDAC10	2.812461468	14.01281628	4.885399317	5.86063557	8.090522638	3.41443578	8.171156279	1.661312636	1.875849726	6.327775524	5.518417529	0.845825217
**ENSG00000100439**	ABHD4	348.7452221	557.0094472	619.63148	406.0583216	479.6524135	493.1962793	498.440533	598.072549	469.4313939	429.0231805	572.5358187	373.8547458
**ENSG00000100441**	KHNYN	2117.783486	2056.380789	2084.437042	1786.656615	2053.836961	1821.411798	2950.808811	3000.330621	2913.194624	2660.829608	2793.698874	2673.65351
**ENSG00000100442**	FKBP3	596.2418313	574.5254675	486.097232	683.1826607	676.1365347	535.3076539	766.0459012	735.9614978	699.2229853	721.9991873	787.7541023	861.0500707
**ENSG00000100445**	SDR39U1	94.21745919	143.6313669	90.37988736	172.4701325	151.4083522	114.5732895	189.9793835	219.293268	207.2813947	224.6360311	249.7083932	215.6854303
**ENSG00000100461**	RBM23	764.9895194	788.2209158	736.8810636	753.5102876	694.6291579	743.9676182	981.560148	1103.11159	1085.179066	1021.935747	1060.91577	1121.564237
**ENSG00000100462**	PRMT5	2567.777321	2270.076238	2935.310756	2721.846605	2127.807454	2783.144542	1506.556939	1382.212113	1586.030943	1495.253356	1116.099945	1692.496259
**ENSG00000100473**	COCH	37.96822982	70.06408141	48.85399317	50.23401917	60.10102531	55.01035423	0	1.661312636	0	0.632777552	0	0
**ENSG00000100478**	AP4S1	108.2797665	52.54806106	57.81055858	99.63080469	52.01050267	98.25987411	183.8510163	137.8889488	126.6198565	98.08052062	117.2663725	140.406986
**ENSG00000100479**	POLE2	205.3096872	192.6762239	142.4908134	236.9371238	182.6146538	164.2722992	212.4500633	144.5341993	211.5020566	256.9076863	201.4222398	246.9809633
**ENSG00000100483**	VCPKMT	140.6230734	164.6505913	139.2338805	138.9807864	82.06101533	128.6104144	164.4445201	146.195512	163.1989261	194.2627086	155.8952952	176.7774703
**ENSG00000100485**	SOS 2	649.6785992	830.2593647	734.4383639	590.2497253	723.5238816	715.134605	1317.59895	1279.21073	1286.363949	1266.187882	1347.873482	1470.890052
**ENSG00000100490**	CDKL1	99.84238213	56.05126513	84.68025482	36.00104707	39.29682424	36.42064832	57.19809395	56.48462963	49.71001773	65.1760879	40.00852709	49.05786257
**ENSG00000100503**	NIN	1610.134191	1646.505913	1599.154043	2093.084132	2272.281072	2073.700664	1655.680541	2069.995545	1911.490871	1735.708826	2193.570968	2407.218567
**ENSG00000100504**	PYGL	2352.624018	2350.649931	1705.004362	2700.07853	2751.933486	2439.045292	2754.701061	4706.498698	2990.573425	3218.306631	5323.893311	3478.879117
**ENSG00000100505**	TRIM9	18.28099954	10.50961221	4.885399317	2.511700959	5.778944741	5.6907263	0	0	4.220661883	1.898332657	13.79604382	6.766601734
**ENSG00000100519**	PSMC6	1688.883112	1842.685341	1573.09858	1783.307681	1690.919231	1772.09217	1380.925411	1628.086383	1489.424682	1507.908907	1618.27594	1691.650434
**ENSG00000100522**	GNPNAT1	1120.765895	644.589549	913.5696722	1169.615413	1040.210053	1210.607175	1016.287562	900.4314488	1098.778977	1060.535178	867.7711565	1228.138215
**ENSG00000100523**	DDHD1	250.3090707	168.1537954	279.2819943	347.4519659	381.4103529	372.9322635	848.7788585	1048.288273	623.2510714	837.1647018	1111.961132	671.5852221
**ENSG00000100526**	CDKN3	372.6511446	325.7979785	360.7053162	658.0656512	568.6481625	609.6664776	505.5902948	400.3763453	528.9896227	537.228142	404.224084	696.1141534
**ENSG00000100528**	CNIH1	1212.170893	1033.445201	1001.50686	1543.021622	1304.885723	1448.479534	1190.946028	1038.320398	1321.536132	1256.063441	1080.230231	1542.785195
**ENSG00000100532**	CGRRF1	115.3109202	98.08971397	77.35215585	140.6552537	101.7094274	122.5403063	107.2464262	162.8086383	121.9302322	132.2505084	135.2012295	109.111453
**ENSG00000100554**	ATP6V1D	646.8661377	893.317038	683.1416711	705.7879694	725.8354595	763.3160876	702.71944	817.365817	720.7952571	778.3163894	800.1705417	842.4419159
**ENSG00000100558**	PLEK2	632.8038304	942.3618949	694.5409362	661.4145858	738.5491379	739.0356554	88.86132453	189.3896405	153.8196775	74.03497363	128.3032076	146.3277625
**ENSG00000100564**	PIGH	136.4043812	126.1153465	135.1627144	166.6094969	114.4231059	153.6496101	120.5245551	112.9692593	143.9714665	150.6010575	81.39665856	153.9401895
**ENSG00000100567**	PSMA3	1882.942953	1699.053974	1832.024744	1193.057955	1193.929984	1278.89589	1889.57989	1742.716955	1843.491318	1356.042295	1267.856427	1657.817425
**ENSG00000100568**	VTI1B	531.5552175	571.0222635	525.9946598	605.319931	668.0460121	581.9716096	891.6774289	951.9321405	843.6634142	843.4924773	895.3632441	915.1828846
**ENSG00000100575**	TIMM9	285.464839	196.1794279	263.8115631	307.2647506	292.4146039	319.0600545	332.9746184	239.2290196	327.3357772	338.5359905	213.8386793	402.6128032
**ENSG00000100577**	GSTZ1	102.6548436	70.06408141	100.9649192	71.16486049	123.6694175	116.84958	294.161626	282.4231481	231.1984787	253.7437985	300.7537553	219.0687311
**ENSG00000100578**	KIAA0586	312.183223	255.7338971	306.1516905	425.3146957	388.3450866	377.1054628	548.4888652	368.8114052	566.5066172	630.8792197	522.8700609	653.8228926
**ENSG00000100580**	TMED8	763.5832886	658.6023652	708.3829009	817.1400452	865.6859222	707.9263517	818.1370224	734.3001852	800.9878329	858.6791386	742.2271577	910.9537585
**ENSG00000100583**	SAMD15	25.31215321	14.01281628	16.28466439	44.3733836	40.45261319	30.72992202	83.75435186	98.01744553	76.90983876	120.8605125	124.1643944	98.11572515
**ENSG00000100591**	AHSA1	863.4256708	707.6472222	939.6251352	1069.147375	1062.170043	1089.963777	947.8541284	799.091378	1099.716902	922.5896714	914.6777055	1199.380157

**ENSG00000100592**	DAAM1	261.5589166	276.7531216	270.3254289	448.7572379	499.3008257	490.9199888	558.7028106	740.9454357	529.4585851	708.0780811	805.6889593	649.5937665
**ENSG00000100593**	ISM2	0	0	0	0	0	0	0	4.983937908	0.468962431	0	9.657230676	0.845825217
**ENSG00000100596**	SPTLC2	1777.475648	2042.367973	1455.848996	1303.572798	1521.018256	1361.980494	994.838277	1280.872042	1072.048118	877.0296876	1118.859154	899.1122054
**ENSG00000100599**	RIN3	47.81184496	42.03844885	65.13865756	44.3733836	57.78944741	70.56500611	62.30506663	64.79119281	59.55822879	63.91053279	77.25784541	102.3448512
**ENSG00000100600**	LGMN	476.7122189	718.1568344	593.576017	363.3594053	394.1240314	303.8847844	462.6917243	410.3442211	527.5827354	467.6226112	462.1674681	528.6407605
**ENSG00000100601**	ALKBH1	185.6224569	129.6185506	215.7718032	164.097796	152.5641412	196.89913	219.599825	270.7939597	208.688282	238.5571372	227.6347231	245.2893129
**ENSG00000100603**	SNW1	1411.855657	1741.092423	1403.73807	1360.504686	1343.026758	1351.737187	1636.274045	1709.490703	1556.017348	1425.015048	1652.76605	1626.521892
**ENSG00000100604**	CHGA	2.812461468	0	5.699632536	4.186168264	13.86946738	7.587635066	4.08557814	0	4.220661883	1.265555105	0	6.766601734
**ENSG00000100605**	ITPK1	648.2723684	665.6087734	660.343141	753.5102876	604.4776199	864.6110158	428.9857046	353.8595915	592.7685134	485.3403827	411.1221059	556.5529926
**ENSG00000100612**	DHRS7	149.0604578	248.727489	206.8152377	234.4254228	250.8062018	259.876501	211.4286687	230.9224564	161.3230764	221.4721433	136.5808338	172.5483442
**ENSG00000100614**	PPM1A	975.9241295	977.3939356	874.4864777	1051.565468	1104.934235	1048.231784	1381.946806	1295.823856	1311.687921	1366.166736	1310.624163	1517.410439
**ENSG00000100625**	SIX4	459.8374501	553.5062431	591.1333173	436.1987331	438.0440114	546.3097248	422.8573374	358.8435294	472.2451685	356.253762	365.5951613	477.0454223
**ENSG00000100628**	ASB2	1.406230734	0	4.071166097	0	0	0.379381753	1.021394535	23.25837691	0.468962431	0.632777552	11.03683506	0.845825217
**ENSG00000100629**	CEP128	310.7769922	318.7915704	280.0962275	370.0572746	405.6819208	312.231183	511.718662	754.2359368	430.5075121	583.4209033	711.8758613	457.5914423
**ENSG00000100632**	ERH	1143.265587	903.8266502	1047.918153	1198.081357	1087.5974	1191.638087	1165.411164	1189.499847	1362.804826	1376.923954	1161.62689	1471.735877
**ENSG00000100644**	HIF1A	1563.728576	1075.48365	1671.6208	1373.900424	1155.788948	1448.858916	1607.674998	1207.774286	1702.802589	1499.050022	1214.051856	1706.875287
**ENSG00000100647**	SUSD6	586.3982161	735.6728548	745.0233958	313.1253862	316.6861718	463.9838843	325.8248566	398.7150327	418.3144889	298.0382272	404.224084	334.9467858
**ENSG00000100650**	SRSF5	2441.216554	2101.922442	2349.877071	2415.419089	1887.403353	1998.203695	5982.307791	6864.543812	4650.700433	5837.372921	7209.812502	4712.938108
**ENSG00000100652**	SLC10A1	0	0	0	0	0	0.379381753	103.160848	88.04956971	25.3239713	85.42496957	81.39665856	14.37902869
**ENSG00000100664**	EIF5	3875.571903	3646.835437	3766.642873	4104.9566	4012.899228	4266.147816	6090.575611	5889.353295	5522.970555	5816.491261	5704.664121	6182.982335
**ENSG00000100678**	SLC8A3	1.406230734	0	0	0	0	0.379381753	0	0	1.875849726	0.632777552	2.759208765	0.845825217
**ENSG00000100697**	DICER1	2615.589166	2063.387197	2475.268987	2994.784776	2708.013506	2913.272484	5265.288827	4955.695594	5171.717694	5093.859297	4842.411382	5545.230121
**ENSG00000100711**	ZFYVE21	182.8099954	231.2114686	216.5860364	174.9818334	176.8357091	216.2475994	185.8938053	255.842146	215.253756	204.3871494	230.3939318	271.5098946
**ENSG00000100714**	MTHFD1	1784.506802	1338.223955	1661.850001	1697.072614	1522.174045	1570.261077	1923.285909	1350.647173	1986.055897	1864.162669	1695.533786	2002.068288
**ENSG00000100722**	ZC3H14	1309.200813	1240.134241	1244.148359	1416.599341	1450.51513	1442.409426	2072.409511	2109.867048	1934.001067	2005.904841	1935.584948	1983.460133
**ENSG00000100726**	TELO2	220.7782253	213.6954483	227.1710682	348.2891996	240.4041012	364.9652467	209.3858796	96.3561329	253.7086754	258.8060189	143.4788558	228.3728085
**ENSG00000100731**	PCNX1	731.2399818	606.0543042	997.4356938	956.9580652	1052.923732	1359.704204	1417.695614	1701.184139	1547.576024	1549.672226	1731.4035	1660.354901
**ENSG00000100739**	BDKRB1	5.624922937	0	0.814233219	0.837233653	0	1.517527013	4.08557814	9.967875817	5.158586746	0	1.379604382	4.229126084
**ENSG00000100744**	GSKIP	219.3719945	248.727489	262.9973299	190.8892729	250.8062018	218.5238899	271.6909463	325.6172767	279.0326467	260.071574	249.7083932	276.5848459
**ENSG00000100749**	VRK1	1251.545353	1152.554139	1283.231554	1321.991938	1227.447863	1398.780524	1609.717787	1684.571013	1595.879154	1756.590485	1758.995587	1925.944019
**ENSG00000100764**	PSMC1	1798.569109	1895.233402	1925.661564	1794.191718	1744.085523	1785.749913	1638.316834	1932.106596	1616.513501	1747.098822	1702.431808	2157.700128
**ENSG00000100767**	PAPLN	56.24922937	87.58010176	100.9649192	25.11700959	56.63365846	33.00621254	31.66323058	16.61312636	21.10330942	37.33387559	27.59208765	11.84155303
**ENSG00000100784**	RPS6KA5	549.8362171	770.7048955	529.2515926	360.0104707	508.5471372	397.5920775	1045.908004	1159.59622	923.3870275	666.9475402	808.448168	658.0520187
**ENSG00000100796**	PPP4R3A	1468.104886	1537.906587	1525.05882	1570.650333	1484.03301	1456.825933	2053.003015	2382.32232	1982.77316	1879.349331	2063.888156	2078.192558
**ENSG00000100802**	C14orf93	95.62368992	150.637775	74.09522297	118.0499451	132.9157291	109.261945	110.3106098	89.71088235	136.93703	143.0077268	113.1275593	104.8823269
**ENSG00000100804**	PSMB5	825.4574409	798.7305281	961.6094322	982.0750748	893.424857	1083.893669	701.6980455	684.4608061	875.0838971	828.9385936	667.728521	877.1207498
**ENSG00000100811**	YY1	2006.691258	1877.717382	1779.099584	1957.45228	1971.775946	1972.785117	2648.476029	2784.359978	2620.562067	2562.749087	2868.197511	2800.527293
**ENSG00000100813**	ACIN1	2304.812173	2189.502544	2571.348507	2441.373332	2122.028509	2338.888509	2853.77633	3021.927685	2957.746055	2941.150063	3167.571662	2851.276806
**ENSG00000100814**	CCNB1IP1	431.7128354	241.7210809	305.3374573	360.8477044	288.9472371	323.9920173	380.9801615	365.4887799	438.4798734	425.2265152	355.9379306	398.3836771
**ENSG00000100815**	TRIP11	1206.54597	1551.919403	1514.473788	1443.390818	1614.637161	1330.871191	1742.499076	1536.714188	1584.624056	1671.165516	1723.125874	1790.611984
**ENSG00000100823**	APEX1	950.6119763	620.0671205	826.4467177	1012.215486	815.9869975	953.0069643	1099.02052	1054.933524	1158.337206	1201.644572	1156.108472	1288.191805
**ENSG00000100836**	PABPN1	105.4673051	112.1025303	84.68025482	134.7946181	107.4883722	87.63718501	193.0435671	161.1473257	127.5577814	186.0366004	164.1729215	140.406986
**ENSG00000100842**	EFS	42.18692202	73.56728548	26.86969624	42.6989163	80.90522638	62.21860754	21.44928523	28.24231481	24.85500887	22.77999189	11.03683506	21.14563042
**ENSG00000100852**	ARHGAP5	4529.469195	5384.424656	4755.122002	4480.037276	4185.111782	4162.197216	6552.245941	6520.652097	5945.974668	6063.274507	6355.837389	6437.575725
**ENSG00000100865**	CINP	196.8723028	171.6569995	218.2145028	236.9371238	253.1177797	247.7362849	246.1560829	230.9224564	236.3570655	279.0549006	201.4222398	273.201545
**ENSG00000100867**	DHRS2	260.1526858	220.7018564	571.5917201	139.81802	225.3788449	256.841447	180.7868327	132.9050109	89.57182441	132.2505084	120.0255813	99.80737558
**ENSG00000100883**	SRP54	1158.734125	1145.547731	1257.176091	1092.589917	1160.412104	1127.143189	1181.753477	1325.727484	1158.337206	1188.989021	1271.99524	1230.67569
**ENSG00000100888**	CHD8	2354.030249	2683.454318	2607.989002	2337.556359	2339.316831	2369.239049	2895.653506	2877.393486	2718.106253	2698.796261	2741.273908	2805.602244
**ENSG00000100889**	PCK2	801.5515185	658.6023652	773.5215585	418.6168264	525.8839715	693.509845	1045.908004	744.268061	1126.44776	710.6091913	567.0174011	662.2811447
**ENSG00000100890**	KIAA0391	113.9046895	94.5865099	123.7634494	105.4914403	108.6441611	113.4351442	191.000778	142.8728867	156.1644897	196.1610412	142.0992514	172.5483442
**ENSG00000100897**	DCAF11	458.4312193	430.8941007	446.1998043	539.1784724	529.3513383	494.3344246	786.4737919	843.9468192	794.4223589	678.9703137	772.5784541	696.9599786
**ENSG00000100902**	PSMA6	156.0916115	192.6762239	206.0010045	123.9105806	107.4883722	107.3650362	269.6481572	247.5355828	274.8119848	203.1215943	142.0992514	215.6854303
**ENSG00000100906**	NFKBIA	1251.545353	2087.909626	1683.020065	851.466625	1116.492124	1054.681274	1813.996694	1953.70366	1823.794896	1356.675072	1463.76025	1587.613932
**ENSG00000100908**	EMC9	129.3732275	70.06408141	74.90945619	112.1893095	99.39784955	116.0908165	126.6529223	103.0013834	146.785241	136.0471738	108.9887462	173.3941694
**ENSG00000100911**	PSME2	3200.581151	2900.65297	2897.041795	725.881577	851.8164549	723.1016218	2528.972868	2058.366356	2577.886486	763.1297282	671.8673342	716.4139586
**ENSG00000100916**	BRMS1L	118.1233817	252.2306931	168.5462764	150.7020575	196.4841212	146.4413568	231.8565594	204.3414542	241.5156522	248.0488005	252.467602	266.4349433
**ENSG00000100918**	REC8	66.09284451	94.5865099	55.36785892	22.60530863	31.2063016	11.00207085	68.43343384	58.14594226	45.02039342	3.796665314	4.138813147	14.37902869
**ENSG00000100926**	TM9SF1	29.53084542	31.52883663	35.82626166	44.3733836	61.25681426	46.2845739	51.06972674	58.14594226	45.02039342	62.64497769	46.906549	40.59961041
**ENSG00000100934**	SEC23A	750.927212	784.7177118	780.0354242	627.088006	676.1365347	699.5799531	1033.651269	1028.352522	955.2764729	846.6563651	776.7172672	1063.202297
**ENSG00000100938**	GMPR2	403.5882207	392.3588559	417.7016416	401.8721534	420.7071772	400.2477497	592.4088302	564.8462963	549.6239697	550.5164706	564.2581924	521.8741588
**ENSG00000100941**	PNN	1791.537955	1607.970668	1698.490496	1966.661851	1497.902477	1656.760117	2314.480016	2000.220414	2167.075396	2500.736887	2164.599276	2513.792544
**ENSG00000100949**	RABGGTA	132.185689	133.1217547	144.9335131	124.7478143	120.2020506	131.2660866	119.5031606	99.67875817	166.9506256	122.1260676	148.9972733	124.3363069
**ENSG00000100968**	NFATC4	99.84238213	94.5865099	76.53792263	133.9573845	175.6799201	128.2310326	130.7385005	142.8728867	113.4889084	189.8332657	147.6176689	129.4112582
**ENSG00000100979**	PLTP	11.24984587	14.01281628	9.770798633	22.60530863	31.2063016	20.10723293	0	0	4.689624315	0.632777552	0	10.1499026
**ENSG00000100982**	PCIF1	201.090995	287.2627338	253.2265312	225.2158526	263.5198802	292.12395	266.5839736	317.3107135	302.4807683	272.7271251	364.2155569	256.2850407
**ENSG00000100983**	GSS	887.3315933	840.7689769	1000.692627	828.0240827	910.7616912	1005.361646	787.4951864	699.4126198	832.4083158	859.9446937	822.2442119	845.8252168
**ENSG00000100985**	MMP9	12.65607661	10.50961221	34.19779522	34.32657977	28.89472371	83.84336748	0	14.95181373	11.25509835	7.593330629	4.138813147	10.99572782

**ENSG00000100991**	TRPC4AP	981.5490524	977.3939356	1070.716684	972.028271	941.9679928	982.2193593	1094.934941	1076.530588	1184.599102	1157.350143	1100.924297	1067.431424
**ENSG00000100994**	PYGB	4923.2138	3902.569334	5071.858724	5209.267788	4649.738939	5736.631492	1367.647282	1442.019368	1762.360817	1482.597805	1535.499678	1623.138591
**ENSG00000100997**	ABHD12	734.0524432	735.6728548	661.1573742	791.185802	839.1027764	845.6419281	961.1322573	850.5920697	976.3797823	935.2452224	1209.913043	1045.439968
**ENSG00000101000**	PROCR	369.8386831	448.410121	367.219182	236.0998901	218.4441112	196.5197482	26.55625791	19.93575163	105.0475846	48.72387153	28.97169203	145.4819373
**ENSG00000101003**	GINS1	1313.419506	1040.451609	1092.70098	1264.222816	1176.593149	1104.759666	1366.625888	1274.226792	1433.618153	1351.612852	1433.408953	1550.397622
**ENSG00000101004**	NINL	23.90592248	3.50320407	13.84196473	5.86063557	11.55788948	5.311344546	10.21394535	28.24231481	9.379248629	7.593330629	12.41643944	25.3747565
**ENSG00000101017**	CD40	1042.016974	1015.92918	1116.313744	175.8190671	194.1725433	261.7734098	1093.913547	855.5760076	1319.19132	357.5193171	355.9379306	341.7133876
**ENSG00000101019**	UQCC1	724.2088281	539.4934268	714.0825335	720.0209415	648.3976	635.8438185	710.8905963	621.3309259	634.5061698	705.5469709	692.5613999	674.1226978
**ENSG00000101040**	ZMYND8	2683.088241	2925.175399	2568.091574	2587.051987	2736.908229	2189.412098	2710.781096	2943.845991	2229.916362	2893.058969	3530.407614	2353.931578
**ENSG00000101049**	SGK2	1.406230734	10.50961221	5.699632536	0.837233653	1.155788948	0.758763507	8.171156279	14.95181373	1.875849726	3.163887762	5.518417529	5.920776517
**ENSG00000101052**	IFT52	202.4972257	206.6890402	214.9575699	221.866918	211.5093775	224.2146162	338.081591	418.6507843	333.4322888	381.5648641	380.7708095	366.2423189
**ENSG00000101057**	MYBL2	1632.633882	1744.595627	1450.149364	2433.838229	2286.15054	2422.731877	1684.279588	2199.57793	2098.137918	1967.30541	2244.61633	2282.88226
**ENSG00000101079**	NDRG3	597.648062	665.6087734	630.2165119	812.1166433	923.4753697	861.5759618	664.9278422	797.4300653	619.4993719	771.9886139	816.7257943	711.3390073
**ENSG00000101082**	SLA2	0	0	3.256932878	0	1.155788948	1.13814526	0	3.322625272	0	0	2.759208765	3.383300867
**ENSG00000101084**	C20orf24	59.06169083	80.57369362	52.92515926	56.93188839	39.29682424	57.28664475	53.11251581	33.22625272	73.62710174	45.55998377	40.00852709	65.12854169
**ENSG00000101096**	NFATC2	255.9339936	385.3524477	473.0695005	489.7816869	439.1998003	733.3449291	45.96275407	78.0816939	75.9719139	56.31720216	64.84140597	82.04504603
**ENSG00000101104**	PABPC1L	447.1813735	350.320407	453.5279032	412.7561909	293.5703929	344.0992502	566.8739669	543.249232	579.1686028	520.7759256	510.4536215	376.3922215
**ENSG00000101109**	STK4	2069.971641	2038.864769	2081.180109	1964.987383	1945.1928	1984.925333	2156.163863	2103.221797	2302.136576	2184.980888	2157.701254	2159.391778
**ENSG00000101115**	SALL4	119.5296124	171.6569995	132.7200148	60.28082301	65.87997005	63.73613456	6.128367209	4.983937908	10.31717349	6.327775524	11.03683506	5.074951301
**ENSG00000101126**	ADNP	3480.421067	2743.008787	3190.979987	3779.272709	3586.413106	3259.648024	3064.183605	2732.859286	2859.732907	3299.302158	2858.54028	3282.647666
**ENSG00000101132**	PFDN4	413.4318358	434.3973047	449.4567371	468.8508456	388.3450866	453.3611952	401.4080522	343.8917157	357.3493728	463.8259459	338.0030737	466.8955197
**ENSG00000101138**	CSTF1	838.1135176	704.1440182	727.9244982	812.9538769	716.5891479	819.8439689	750.7249831	802.4140032	839.4427523	791.604718	823.6238162	828.0628872
**ENSG00000101144**	BMP7	19.68723028	3.50320407	10.58503185	30.1404115	26.58314581	11.76083435	112.3533988	142.8728867	120.5233449	105.0410737	146.2380645	140.406986
**ENSG00000101146**	RAE1	667.9595987	623.5703245	778.4069578	720.0209415	710.8102032	772.4212497	711.9119908	724.3323094	793.484434	841.5941447	794.6521242	806.9172568
**ENSG00000101150**	TPD52L2	1664.977189	1562.429015	1893.092235	1605.814146	1725.5929	1945.469631	1284.914325	1252.629728	1430.335416	1356.042295	1313.383372	1428.598791
**ENSG00000101152**	DNAJC5	1231.858123	1261.153465	1449.335131	1171.28988	1265.588898	1502.351743	1040.801031	1147.967032	1272.764039	1158.615698	1327.179416	1140.172392
**ENSG00000101158**	NELFCD	1295.138506	980.8971397	1100.843313	1399.854668	1188.151039	1272.067019	1282.871536	1523.423687	1365.6186	1514.236683	1434.788558	1452.281897
**ENSG00000101160**	CTSZ	393.7446056	514.9709984	491.7968645	506.52636	626.43761	612.7015316	756.8533503	837.3015686	698.7540229	820.0797079	918.8165186	831.4461881
**ENSG00000101161**	PRPF6	1819.66257	1842.685341	2065.709678	1892.985289	2075.796951	2030.451144	1793.568803	1830.766525	1897.421998	1718.623832	1981.111893	1974.156056
**ENSG00000101166**	PRELID3B	3128.863383	2816.576073	3001.263647	3127.904927	2615.55039	3024.431337	2261.3675	2413.88726	2558.659026	2380.509152	1927.307322	2701.565742
**ENSG00000101181**	MTG2	389.5259134	290.7659378	371.2903481	348.2891996	381.4103529	387.3487701	460.6489352	407.0215959	436.1350613	461.2948357	471.8246987	477.0454223
**ENSG00000101182**	PSMA7	3082.457769	2721.989563	2931.23959	3014.04115	3003.895477	3043.779807	2248.089371	2141.431988	2594.300171	2238.134203	2273.588022	2700.719917
**ENSG00000101187**	SLCO4A1	1234.670585	1884.72379	1365.469109	962.8187008	1584.586648	1296.347451	398.3438686	1400.486552	643.8854184	369.5420906	1258.199197	652.9770674
**ENSG00000101188**	NTSR1	1473.729809	970.3875275	1049.54662	1666.932203	1803.030759	1621.477614	6.128367209	13.29050109	58.62030393	6.960553076	35.86971394	40.59961041
**ENSG00000101189**	MRGBP	544.2112941	599.047896	499.9391967	579.3656878	533.9744941	559.9674679	367.7020326	531.6200436	355.473523	344.863766	542.1845222	371.3172702
**ENSG00000101190**	TCFL5	317.8081459	472.9325495	253.2265312	335.7306948	329.3998503	298.9528216	355.4452981	420.3120969	391.1146678	448.6392846	440.093798	411.0710554
**ENSG00000101191**	DIDO1	2176.845176	2070.393606	2403.616464	1998.476729	2073.485373	2211.795622	2398.234368	2417.209886	2479.404375	2493.776334	2571.582569	2457.122255
**ENSG00000101193**	GID8	755.1459042	525.4806106	609.0464481	786.9996337	805.5848969	713.2376962	894.7416126	1006.755457	1049.068959	921.9568938	1009.870408	1051.360744
**ENSG00000101199**	ARFGAP1	513.274218	490.4485699	661.9716074	508.2008273	490.0545141	598.6644067	593.4302248	503.3777287	648.1060803	511.2842623	531.1476872	548.0947405
**ENSG00000101203**	COL20A1	2.812461468	3.50320407	0	0	2.311577897	0	0	0	0.937924863	3.796665314	0	0.845825217
**ENSG00000101210**	EEF1A2	1511.698039	1198.095792	1086.187115	2046.199048	1865.443362	1937.881996	8.171156279	14.95181373	9.379248629	6.327775524	17.93485697	10.1499026
**ENSG00000101213**	PTK6	381.088529	406.3716722	372.1045813	374.2434428	358.294574	456.0168675	2.04278907	6.645250545	5.158586746	5.062220419	5.518417529	4.229126084
**ENSG00000101216**	GMEB2	227.8093789	196.1794279	280.0962275	313.9626198	209.1977996	270.4991901	290.0760479	249.1968954	313.2669042	287.9137863	321.4478211	299.4221267
**ENSG00000101220**	C20orf27	254.5277629	168.1537954	196.2302059	251.1700959	292.4146039	354.7219393	145.038024	116.2918845	282.3153837	296.7726721	217.9774924	284.1972728
**ENSG00000101222**	SPEF1	1.406230734	0	4.885399317	11.72127114	11.55788948	11.00207085	8.171156279	3.322625272	15.94472267	10.75721839	22.07367012	8.458252168
**ENSG00000101224**	CDC25B	3760.260983	2129.948075	3289.502207	5148.986965	4793.056768	4941.067955	2648.476029	1701.184139	3185.192834	2825.984549	2188.05255	3192.144368
**ENSG00000101230**	ISM1	4.218692202	3.50320407	2.442699658	3.348934611	0	0.379381753	39.83438686	26.58100218	42.20661883	30.37332251	28.97169203	43.13708606
**ENSG00000101236**	RNF24	537.1801404	1019.432384	469.8125676	513.2242292	958.1490381	483.3323537	1167.453953	2231.14287	1178.50259	1057.37129	1822.457389	1080.964627
**ENSG00000101246**	ARFRP1	333.276684	287.2627338	311.851323	324.8466573	236.9367344	294.4002406	302.3327823	232.5837691	313.7358666	315.7559986	282.8188984	234.293585
**ENSG00000101247**	NDUFAF5	230.6218404	234.7146727	291.4954926	325.683891	264.6756692	302.3672574	355.4452981	358.8435294	342.342575	380.299309	313.1701948	395.8462015
**ENSG00000101255**	TRIB3	704.5215978	658.6023652	824.8182513	553.4114445	533.9744941	619.5304031	1110.255859	880.4956971	1096.434165	950.4318837	903.6408704	837.3669646
**ENSG00000101265**	RASSF2	8.437384405	0	3.256932878	0	2.311577897	5.311344546	2.04278907	0	1.875849726	6.960553076	9.657230676	4.229126084
**ENSG00000101266**	CSNK2A1	4252.44174	3871.040498	4173.759483	4224.681012	4055.663419	4183.063212	3905.812701	3719.678992	3603.976286	4018.770235	3829.781765	3808.750951
**ENSG00000101276**	SLC52A3	165.9352266	122.6121425	227.9853014	180.0052354	234.6251565	252.2888659	110.3106098	121.2758224	166.4816632	107.5721839	78.63744979	161.5526164
**ENSG00000101278**	RPS10P5	1.406230734	0	0.814233219	2.511700959	1.155788948	2.655672273	5.106972674	3.322625272	1.875849726	6.960553076	8.277626294	2.53747565
**ENSG00000101280**	ANGPT4	0	0	0	0	0	0	3.064183605	4.983937908	0.468962431	0	4.138813147	0
**ENSG00000101282**	RSPO4	21.09346101	31.52883663	43.96859385	28.4659442	16.18104528	26.55672273	3.064183605	0	2.813774589	0.632777552	1.379604382	1.691650434
**ENSG00000101290**	CDS2	1241.701738	1614.977076	1182.266635	1126.916497	1339.559391	1086.16996	1584.182924	1599.844069	1554.61046	1644.588859	1670.700907	1702.646161
**ENSG00000101294**	HM13	1953.25449	2427.720421	2596.589737	1919.776766	2380.925233	2682.228996	1507.578333	1566.617816	1689.202678	1597.76332	1651.386446	1689.112958
**ENSG00000101298**	SNPH	23.90592248	70.06408141	55.36785892	28.4659442	70.50312584	71.70315137	11.23533988	4.983937908	17.8205724	20.24888168	1.379604382	9.304077385
**ENSG00000101306**	MYLK2	16.87476881	10.50961221	17.91313083	17.58190671	8.090522638	17.83094241	24.51346884	3.322625272	17.8205724	20.88165923	15.17564821	15.2248539
**ENSG00000101307**	SIRPB1	143.4355349	217.1986524	139.2338805	159.074394	169.9009754	188.1733496	178.7440436	242.5516449	103.6406974	118.3294023	171.0709434	93.04077385
**ENSG00000101310**	SEC23B	1774.663187	1905.743014	2026.626483	1740.608764	1619.260317	2089.634697	1431.995138	1496.842685	1589.782643	1449.693373	1281.652471	1535.172768
**ENSG00000101311**	FERMT1	1587.634499	1692.047566	2445.142358	1397.342967	1348.805703	2499.746373	1425.866771	966.8839542	1407.825219	1272.515658	916.0573099	1261.125398
**ENSG00000101323**	HAO1	0	14.01281628	6.513865756	1.674467306	12.71367843	3.41443578	0	0	0	0	0	0
**ENSG00000101331**	CCM2L	0	7.006408141	0.814233219	0	5.778944741	0.758763507	1.021394535	0	0.468962431	0	0	0
**ENSG00000101333**	PLCB4	54.84299863	59.5544692	80.60908873	28.4659442	78.59364848	65.25366157	5.106972674	0	1.406887294	3.796665314	9.657230676	2.53747565

**ENSG00000101335**	MYL9	25.31215321	21.01922442	50.48245961	29.30317785	38.14103529	75.87635066	36.77020326	6.645250545	27.19982102	38.5994307	30.35129641	68.51184256
**ENSG00000101337**	TM9SF4	1231.858123	1205.1022	1283.231554	1353.806817	1355.740436	1388.916599	1601.546631	1548.343377	1616.513501	1612.949981	1757.615983	1671.350628
**ENSG00000101342**	TLDC2	1.406230734	7.006408141	3.256932878	0	0	2.655672273	1.021394535	1.661312636	0	0	0	0
**ENSG00000101343**	CRNKL1	2455.278862	2347.146727	2064.081211	2159.225591	1937.102277	1916.257236	2085.68764	2118.173611	1992.152409	1885.044329	2043.19409	2114.563042
**ENSG00000101346**	POFUT1	772.0206731	721.6600385	956.7240329	1037.332496	1034.431109	1155.596821	993.8168824	875.5117592	1149.426919	1119.38349	918.8165186	1260.279573
**ENSG00000101347**	SAMHD1	2881.366774	3559.255336	2673.127659	524.9455003	731.6144043	560.7262314	999.9452496	1136.337843	875.5528595	296.1398945	238.6715581	242.7518372
**ENSG00000101350**	KIF3B	1663.570959	1678.03475	1577.169746	2075.502225	2080.420107	1840.380885	1673.044248	1553.327315	1701.395701	1879.982108	2023.879629	1996.147512
**ENSG00000101353**	MROH8	1.406230734	24.52242849	4.885399317	0.837233653	2.311577897	9.863925586	18.38510163	14.95181373	20.16538455	15.81943881	5.518417529	23.68310607
**ENSG00000101361**	NOP56	3294.79861	2059.883993	2987.421682	3067.624104	2209.868469	2816.150755	2255.239133	1614.795882	2385.142926	2452.013015	1702.431808	2485.880312
**ENSG00000101363**	MANBAL	535.7739097	469.4293454	493.425331	506.52636	475.0292577	492.058134	557.681416	604.7177995	613.4028603	654.2919892	635.9976202	663.12697
**ENSG00000101365**	IDH3B	956.2368992	893.317038	942.0678349	1028.122926	1028.652164	1096.792649	950.918312	883.8183224	991.3865801	1088.37739	945.0290019	1047.977444
**ENSG00000101367**	MAPRE1	2517.153014	2385.681972	2713.025087	3045.856029	2719.571395	3203.499525	2628.048138	2448.774826	2751.871548	2816.492886	2737.135095	3281.801841
**ENSG00000101384**	JAG1	2306.218404	1783.130872	2248.912152	2110.666039	2049.213805	2306.64106	1090.849363	1365.598987	1279.798475	1045.348517	1398.918844	1164.701324
**ENSG00000101391**	CDK5RAP1	516.0866794	434.3973047	666.0427735	474.7114812	451.9134788	531.1344546	624.0720608	460.1836002	542.5895332	589.7486788	500.7963908	510.8784309
**ENSG00000101400**	SNTA1	19.68723028	35.0320407	32.56932878	35.16381342	31.2063016	22.38352344	48.00554314	83.06563181	44.08246856	81.62830426	100.7111199	51.59533822
**ENSG00000101407**	TTI1	1175.608894	1043.954813	1161.096571	1090.078216	1107.245812	1138.904023	1411.567247	1653.006073	1368.901337	1297.82676	1770.032423	1383.770055
**ENSG00000101412**	E2F1	413.4318358	613.0607123	428.2866734	452.9434062	551.3113283	533.0313634	346.2527473	425.2960348	417.8455264	430.2887356	430.4365673	451.6706658
**ENSG00000101413**	RPRD1B	1146.078048	1117.522098	1258.804557	1048.216533	870.309078	1044.817349	1792.547409	2066.672919	1569.148296	1488.925581	1921.788905	1557.164224
**ENSG00000101417**	PXMP4	184.2162262	203.1858361	147.3762127	262.0541333	297.0377597	256.841447	462.6917243	450.2157244	492.410553	557.4770236	510.4536215	505.8034796
**ENSG00000101421**	CHMP4B	1091.23505	1100.006078	1073.159383	1085.054814	1029.807953	1003.464737	1401.353302	1209.435599	1310.281033	1309.216756	1194.737395	1164.701324
**ENSG00000101439**	CST3	2954.490772	2350.649931	2201.686625	2414.581855	2271.125283	2199.655406	4539.077313	4525.415621	5673.038533	3546.718181	3632.498339	3449.275234
**ENSG00000101441**	CST4	16.87476881	49.04485699	35.01202844	9.209570182	21.95999002	24.65981396	0	0	0	0	0	0
**ENSG00000101442**	ACTR5	208.1221487	168.1537954	177.5028418	195.9126748	226.5346339	256.841447	181.8082272	154.5020752	172.5781748	225.9015862	224.8755143	192.8481494
**ENSG00000101443**	WFDC2	1.406230734	3.50320407	4.885399317	5.023401917	6.93473369	9.484543833	0	0	1.875849726	0	1.379604382	2.53747565
**ENSG00000101444**	AHCY	2015.128642	1660.518729	2086.879741	2604.633894	2358.965243	2851.433258	1857.916659	1832.427838	2192.868329	2081.838147	2070.786178	2353.931578
**ENSG00000101445**	PPP1R16B	0	0	2.442699658	3.348934611	0	1.13814526	1.021394535	0	0	0	0	2.53747565
**ENSG00000101447**	FAM83D	600.4605235	182.1666117	456.7848361	807.930475	650.7091779	780.7676483	520.9112128	456.8609749	679.0576007	609.9975605	430.4365673	866.9708472
**ENSG00000101448**	EPPIN	1.406230734	0	6.513865756	0	0	1.13814526	7.149761744	0	2.813774589	5.694997971	0	0
**ENSG00000101452**	DHX35	378.2760675	262.7403053	481.2118327	375.9179101	302.8167044	391.5219694	562.7883887	513.3456046	590.4237012	597.3420094	533.906896	511.7242561
**ENSG00000101457**	DNTTIP1	404.9944514	448.410121	494.2395642	357.4987698	365.2293077	477.2622457	428.9857046	443.5704738	457.7073331	419.5315172	517.3516434	464.358044
**ENSG00000101463**	SYNDIG1	12.65607661	0	0.814233219	0	3.467366845	1.517527013	0	0	0	0	0	0
**ENSG00000101464**	PIGU	555.46114	476.4357536	591.9475505	656.3911838	618.3470873	659.744869	612.8367209	549.8944826	626.5338084	620.7547789	539.4253135	708.8015317
**ENSG00000101470**	TNNC2	0	0	6.513865756	4.186168264	1.155788948	0.758763507	0	1.661312636	2.344812157	0.632777552	2.759208765	3.383300867
**ENSG00000101473**	ACOT8	118.1233817	168.1537954	179.9455415	158.2371604	160.6546638	186.2764409	292.118837	313.9880882	307.639355	255.0093536	307.6517773	268.9724189
**ENSG00000101474**	APMAP	1081.391435	935.3554868	1181.452401	1353.806817	1391.569894	1616.925033	1291.042692	1510.133186	1558.831122	1785.065475	1712.089038	1876.886156
**ENSG00000101489**	CELF4	1.406230734	0	0	2.511700959	5.778944741	0	1.021394535	0	0	0	12.41643944	0
**ENSG00000101493**	ZNF516	37.96822982	45.54165292	35.01202844	10.04680383	18.49262317	15.93403364	15.32091802	6.645250545	19.22745969	1.898332657	8.277626294	7.612426951
**ENSG00000101544**	ADNP2	804.3639799	770.7048955	817.4901523	850.6293913	671.5133789	827.8109857	610.7939319	375.4566558	594.6443631	551.1492481	390.4280402	559.9362935
**ENSG00000101546**	RBFA	149.0604578	140.1281628	157.1470114	210.9828805	147.9409854	140.3712487	231.8565594	137.8889488	235.888103	242.3538026	229.0143275	262.2058172
**ENSG00000101557**	USP14	3190.737536	2788.55044	2820.503872	2961.29543	2962.287074	3082.476746	2033.596519	2066.672919	2097.199993	2152.076456	2004.565167	2209.295466
**ENSG00000101558**	VAPA	4857.120956	4435.056353	4335.791894	4503.479819	4563.054768	4732.028609	3237.820676	3161.477947	3528.942297	3203.11997	2922.002082	3409.521449
**ENSG00000101574**	METTL4	606.0854464	455.4165292	627.7738122	595.2731272	542.0650167	621.4273119	461.6703298	446.8930991	513.9828249	499.2614888	389.0484358	545.5572648
**ENSG00000101577**	LPIN2	494.9932184	728.6664466	559.3782218	373.4062092	678.4481126	546.6891065	626.1148499	880.4956971	638.2578692	487.8714929	707.7370481	623.3731848
**ENSG00000101596**	SMCHD1	3831.978751	3419.127173	3590.768498	3590.895137	3354.099528	3408.365672	3911.941069	3227.930452	3414.515463	3529.633187	3242.070298	3737.701633
**ENSG00000101605**	MYOM1	26.71838395	77.07048955	39.89742775	20.09360767	42.76419109	30.35054026	14.29952349	29.90362745	22.04123428	19.61610412	23.4532745	10.99572782
**ENSG00000101608**	MYL12A	4170.880358	4480.598006	4372.432388	3376.563322	2966.91023	3479.689441	1600.525236	1976.962037	1695.768152	1260.492884	1397.539239	1648.513347
**ENSG00000101624**	CEP76	255.9339936	262.7403053	281.7246939	296.3807131	303.9724934	298.9528216	232.877954	270.7939597	232.1364036	203.7543719	244.1899757	320.5677572
**ENSG00000101639**	CEP192	1764.819571	2140.457687	1695.233563	2089.735198	1917.453865	1804.339619	2514.673345	2988.701432	2390.770476	2621.5974	2886.132368	2398.760315
**ENSG00000101654**	RNMT	1160.140356	1996.82632	1382.568007	1180.499451	1732.527633	1360.842349	1052.036371	1533.391563	1102.530676	1138.366817	1585.165435	1165.547149
**ENSG00000101665**	SMAD7	108.2797665	210.1922442	231.2422343	159.074394	176.8357091	197.6578935	224.7067977	350.8274673	312.7979418	332.208215	361.4563482	282.5056224
**ENSG00000101670**	LIPG	2680.275779	1653.512321	2648.700663	2083.874562	1524.485623	2310.055496	514.7828456	350.5369662	914.4767413	415.7348519	264.8840414	824.6795864
**ENSG00000101680**	LAMA1	756.552135	1078.986854	1162.725037	1265.060049	1663.180297	1641.205465	26.55625791	56.48462963	77.37880119	22.77999189	53.80457091	95.5782495
**ENSG00000101695**	RNF125	7.031153671	10.50961221	4.071166097	10.88403749	41.60840214	14.41650663	10.21394535	0	6.096511609	5.062220419	12.41643944	9.304077385
**ENSG00000101745**	ANKRD12	1892.786568	2855.111317	2327.078541	1855.309775	2473.388349	1839.622122	2076.495089	2372.354444	1847.243017	1836.953235	2433.62213	1749.166548
**ENSG00000101746**	NOL4	0	0	0	0	0	0	0	3.322625272	0.468962431	0	1.379604382	5.920776517
**ENSG00000101751**	POLI	566.7109859	672.6151815	524.3661933	407.7327889	545.5323836	418.4580739	1065.3145	890.463573	809.4291567	1021.30297	1059.536166	828.9087124
**ENSG00000101752**	MIB1	1590.44696	1646.505913	1284.86002	1795.866185	2029.565393	1639.308556	2244.003793	2300.918001	2175.047757	2207.128103	2469.491844	2474.038759
**ENSG00000101773**	RBBP8	1106.703588	1089.496466	979.522563	1111.009057	956.9932492	1024.710116	949.8969174	960.2387037	1012.020927	973.8446531	1037.462495	1118.180937
**ENSG00000101782**	RIOK3	2206.376022	2904.156174	2305.908477	1492.787603	1911.67492	1762.607626	2436.025966	2879.054798	2522.548919	2053.995935	2192.191363	2163.620905
**ENSG00000101811**	CSTF2	572.3359088	550.0030391	548.7931899	628.7624733	579.0502631	644.9489806	367.7020326	373.7953431	385.0181562	443.5770642	433.195776	390.7712502
**ENSG00000101825**	MXRA5	0	0	0	0	0	0.379381753	72.51901198	142.8728867	31.42048291	20.88165923	31.73090079	12.68737825
**ENSG00000101843**	PSMD10	365.6199909	472.9325495	443.7571046	324.8466573	332.8672171	473.8478099	499.4619276	513.3456046	503.6656514	478.3798296	452.5102374	548.0947405
**ENSG00000101844**	ATG4A	80.15515185	112.1025303	100.9649192	108.0031412	127.1367843	164.2722992	138.9096567	152.8407625	127.5577814	109.4705166	115.8867681	137.8695103
**ENSG00000101846**	STS	184.2162262	157.6441832	188.0878737	191.7265065	190.7051765	223.4558527	1164.38977	872.189134	771.9121622	1019.404637	707.7370481	855.1292942
**ENSG00000101849**	TBL1X	85.78007478	87.58010176	37.45472809	71.16486049	60.10102531	55.01035423	450.4349899	289.0683987	510.7000879	455.5998377	291.0965247	396.6920267
**ENSG00000101850**	GPR143	1.406230734	0	0	0	0	0	11.23533988	26.58100218	12.19302322	14.5538837	26.21248326	8.458252168
**ENSG00000101856**	PGRMC1	866.2381322	942.3618949	672.5566393	843.9315221	820.6101533	878.6481407	992.7954879	1137.999156	1055.165471	1004.217976	1040.221704	1150.322295

**ENSG00000101868**	POLA1	1522.947885	1979.3103	1429.793533	1728.887493	1729.060267	1452.273352	1852.809686	2043.414542	1781.119315	1945.158196	1976.97308	2046.897025
**ENSG00000101871**	MID1	1915.28626	2007.335932	1839.352843	2360.998901	2546.203053	2646.187729	2207.23359	3390.73909	2154.882373	1971.734853	3239.31109	2238.053524
**ENSG00000101882**	NKAP	345.9327606	290.7659378	304.5232241	345.7774986	339.8019508	310.3342742	290.0760479	299.0362745	277.156797	250.5799107	347.6603043	285.0430981
**ENSG00000101888**	NXT2	343.1202991	350.320407	412.8162423	252.8445632	232.3135786	300.4703486	263.51979	232.5837691	274.3430224	265.1337944	224.8755143	241.0601868
**ENSG00000101901**	ALG13	891.5502855	781.2145077	854.1306472	698.2528665	726.9912485	725.3779123	755.8319558	631.2988017	640.6026814	673.2753157	641.5160378	591.2318265
**ENSG00000101911**	PRPS2	949.2057456	1103.509282	984.4079623	1054.077169	1071.416355	971.2172885	427.9643101	480.1193518	591.3616261	505.5892644	659.4508947	719.7972595
**ENSG00000101928**	MOSPD1	139.2168427	178.6634076	210.0721706	180.842469	173.3683422	219.6620352	127.6743169	162.8086383	151.9438278	146.8043922	110.3683506	192.8481494
**ENSG00000101935**	AMMECR1	511.8679872	606.0543042	556.1212889	584.3890897	646.0860221	611.9427681	663.9064477	569.8302342	542.1205708	722.6319648	557.3601705	643.67299
**ENSG00000101938**	CHRDL1	2.812461468	35.0320407	8.956565414	18.41914036	73.97049269	29.212395	0	0	0	0.632777552	0	0
**ENSG00000101940**	WDR13	544.2112941	441.4037129	499.1249635	471.3625466	493.5218809	501.9220596	442.2638336	460.1836002	367.6665463	418.2659621	522.8700609	407.6877545
**ENSG00000101945**	SUV39H1	281.2461468	290.7659378	342.7921854	382.6157794	361.7619408	439.3240703	329.9104348	493.4098529	361.5700347	325.8804395	678.7653561	341.7133876
**ENSG00000101951**	PAGE4	0	14.01281628	4.885399317	1.674467306	2.311577897	2.27629052	605.6869592	460.1836002	509.762163	741.6152914	762.9212234	857.6667698
**ENSG00000101955**	SRPX	4.218692202	0	1.628466439	0	0	1.517527013	0	0	0	0.632777552	0	0
**ENSG00000101966**	XIAP	1934.97349	2115.935259	1906.119967	1480.229098	1415.841462	1441.271281	1444.251872	1415.438366	1483.328171	1140.897927	1243.023548	1372.774327
**ENSG00000101972**	STAG2	3148.550614	3433.139989	2761.064847	3450.239883	3259.324834	2967.524074	3642.292911	3796.099374	3645.713942	3564.435953	3552.481284	3957.616189
**ENSG00000101974**	ATP11C	23.90592248	14.01281628	28.49816268	19.25637402	3.467366845	7.587635066	31.66323058	23.25837691	11.25509835	13.2883286	1.379604382	12.68737825
**ENSG00000101986**	ABCD1	105.4673051	143.6313669	119.6922833	51.07125282	78.59364848	59.94231702	50.04833221	89.71088235	53.93067962	31.00610007	40.00852709	23.68310607
**ENSG00000101997**	CCDC22	85.78007478	161.1473872	159.589711	133.9573845	127.1367843	146.061975	80.69016826	107.9853213	114.4268333	79.09719405	97.95191114	103.1906764
**ENSG00000102001**	CACNA1F	23.90592248	38.53524477	13.84196473	1.674467306	2.311577897	1.13814526	19.40649616	3.322625272	13.13094808	0	0	0
**ENSG00000102003**	SYP	22.49969175	42.03844885	24.42699658	26.79147689	24.27156791	27.31548624	22.47067977	28.24231481	35.64114479	30.37332251	22.07367012	21.14563042
**ENSG00000102007**	PLP2	1432.949118	1565.932219	1302.773151	1813.448092	2132.43061	1663.209606	1169.496742	1302.469107	1028.903575	1223.159009	1434.788558	1197.688507
**ENSG00000102010**	BMX	1.406230734	0	1.628466439	0	0	0	3.064183605	1.661312636	1.406887294	0	0	0.845825217
**ENSG00000102021**	LUZP4	5.624922937	24.52242849	25.2412298	27.62871054	18.49262317	16.69279715	9.192550814	6.645250545	12.19302322	5.694997971	9.657230676	9.304077385
**ENSG00000102024**	PLS3	6610.690681	7447.811854	6044.053188	7970.464375	7637.45337	7316.377112	4473.708063	5417.540506	4445.76385	4499.681175	5047.972435	4956.53577
**ENSG00000102030**	NAA10	164.5289959	133.1217547	151.4473788	163.2605623	132.9157291	149.4764108	151.1663912	59.8072549	146.785241	183.5054902	115.8867681	143.7902869
**ENSG00000102034**	ELF4	928.1122845	865.2914054	983.5937291	1032.309094	1005.536385	1097.551412	1254.272489	1295.823856	1450.969763	1369.963401	1111.961132	1409.144811
**ENSG00000102038**	SMARCA1	804.3639799	896.820242	666.0427735	811.2794096	834.4796206	643.4314536	551.5530488	923.6898257	507.8863133	608.0992278	1009.870408	625.0648352
**ENSG00000102043**	MTMR8	1.406230734	3.50320407	0	0.837233653	0	0.379381753	40.8557814	59.8072549	31.42048291	46.19276132	46.906549	22.83728085
**ENSG00000102048**	ASB9	1.406230734	3.50320407	4.885399317	0	8.090522638	6.070108053	0	1.661312636	0	1.265555105	0	0.845825217
**ENSG00000102053**	ZC3H12B	0	0	0	0	0	0	10.21394535	1.661312636	2.344812157	13.92110615	6.898021912	5.074951301
**ENSG00000102054**	RBBP7	1992.62895	2157.973707	1990.800222	2170.946862	2263.034761	2106.706876	1799.69717	1947.05841	1811.601873	1907.82432	2063.888156	2013.064016
**ENSG00000102057**	KCND1	14.06230734	7.006408141	5.699632536	6.697869223	3.467366845	2.27629052	11.23533988	29.90362745	12.19302322	12.0227735	2.759208765	24.52893129
**ENSG00000102078**	SLC25A14	153.27915	150.637775	140.0481137	150.7020575	188.3935986	136.5774312	187.9365944	157.8247004	172.1092123	186.669378	162.7933171	179.314946
**ENSG00000102081**	FMR1	3666.043524	3384.095132	3120.141697	3435.169678	2933.392351	3255.095443	4759.698533	4334.364668	4664.300343	4503.47784	4134.674334	4754.383543
**ENSG00000102096**	PIM2	106.8735358	115.6057343	82.23755516	77.02549606	69.3473369	102.8124551	63.32646116	252.5195207	78.31672605	83.52663691	286.9577115	76.12426951
**ENSG00000102098**	SCML2	91.40499772	84.07689769	115.6211172	114.7010104	95.93048271	119.5052523	150.1449966	127.921073	135.0611803	158.1943881	165.5525259	177.6232955
**ENSG00000102100**	SLC35A2	499.2119106	550.0030391	523.5519601	505.6891263	473.8734688	595.6293527	538.2749199	669.5089924	622.3131465	585.9520135	670.4877298	575.1611474
**ENSG00000102103**	PQBP1	527.3365253	658.6023652	602.5325824	617.0412022	591.7639415	591.0767716	386.0871342	598.072549	440.8246856	427.1248479	489.7595557	442.3665884
**ENSG00000102109**	PCSK1N	9.843615139	21.01922442	13.02773151	10.04680383	6.93473369	14.41650663	0	6.645250545	0	3.796665314	0	0.845825217
**ENSG00000102119**	EMD	345.9327606	420.3844885	352.562984	401.0349197	339.8019508	464.363266	333.9960129	397.05372	347.0321993	391.6893049	394.5668533	362.859018
**ENSG00000102125**	TAZ	164.5289959	136.6249587	159.589711	173.3073661	190.7051765	154.7877553	165.4659146	149.5181373	186.6470477	189.2004882	136.5808338	204.6897025
**ENSG00000102128**	RAB40AL	9.843615139	21.01922442	8.142332194	2.511700959	9.246311586	8.725780326	9.192550814	18.274439	12.19302322	24.04554699	24.83287888	14.37902869
**ENSG00000102144**	PGK 1.00	3332.76684	9851.009846	3229.248948	3034.971992	6141.862471	3104.480887	2730.187592	10162.2494	3184.723872	2997.467266	8502.501808	3299.564171
**ENSG00000102158**	MAGT1	1459.667502	1741.092423	1413.508869	1299.386629	1330.313079	1316.454684	1461.615579	1508.471874	1635.271998	1537.649452	1534.120073	1586.768107
**ENSG00000102172**	SMS	2872.92939	2781.544032	2589.261638	3041.669861	3150.680673	2736.101205	1103.106098	1453.648557	1227.274683	1240.244003	1514.805612	1416.757238
**ENSG00000102174**	PHEX	14.06230734	24.52242849	13.02773151	36.83828073	33.5178795	19.34846942	23.4920743	28.24231481	17.8205724	32.27165517	24.83287888	32.14135824
**ENSG00000102178**	UBL4A	454.2125271	409.8748762	479.5833663	598.6220618	528.1955494	614.2190586	379.958767	365.4887799	465.6796944	425.2265152	409.7425015	499.8827031
**ENSG00000102181**	CD99L2	299.5271464	273.2499175	408.7450762	410.2444899	372.1640413	393.7982599	754.8105613	667.8476797	666.3956151	773.254169	641.5160378	659.7436691
**ENSG00000102189**	EEA1	2188.095022	1720.073199	2106.421339	2090.572431	1934.790699	1918.154145	2937.530682	2450.436138	2302.136576	2572.24075	2490.18591	2611.908269
**ENSG00000102195**	GPR50	0	0	0	0	5.778944741	1.13814526	0	0	0	0	0	3.383300867
**ENSG00000102218**	RP2	556.8673707	658.6023652	579.7340522	384.2902467	434.5766445	444.2560331	502.5261112	531.6200436	577.7617156	582.1553482	515.972039	622.5273595
**ENSG00000102221**	JADE3	178.5913032	248.727489	293.123959	179.1680017	193.0167544	267.0847543	584.2376739	466.8288508	609.1821985	534.6970318	555.9805661	663.9727952
**ENSG00000102225**	CDK16	1195.296124	1275.166282	1198.551299	1686.188577	1626.19505	1571.778604	1229.75902	1249.307102	1251.66073	1386.415617	1243.023548	1304.262484
**ENSG00000102226**	USP11	592.0231391	865.2914054	648.9438759	432.0125649	538.5976499	566.4169577	451.4563844	415.328159	443.6384602	354.3554293	452.5102374	359.4757171
**ENSG00000102230**	PCYT1B	1.406230734	3.50320407	10.58503185	7.535102876	21.95999002	7.208253313	4.08557814	13.29050109	0.468962431	4.429442867	1.379604382	0
**ENSG00000102241**	HTATSF1	1965.910566	2021.348749	1680.577365	2271.4149	2206.401102	1877.939679	2254.217738	2134.786737	2314.329599	2069.815374	2189.432155	2857.197582
**ENSG00000102243**	VGLL1	1042.016974	655.0991612	749.9087951	1407.38977	999.7574402	1226.92059	1868.130604	1631.409009	2192.399367	1669.267183	1329.938625	2046.051199
**ENSG00000102265**	TIMP1	423.275451	504.4613861	464.9271683	349.9636669	494.6776699	485.2292625	85.79714093	104.6626961	151.0059029	80.9955267	103.4703287	116.7238799
**ENSG00000102271**	KLHL4	0	7.006408141	11.39926507	3.348934611	15.02525633	4.173199286	23.4920743	38.21019063	26.26189616	18.98332657	37.24931832	49.05786257
**ENSG00000102287**	GABRE	4.218692202	7.006408141	0.814233219	2.511700959	5.778944741	1.896908767	20.4278907	1.661312636	24.85500887	12.65555105	15.17564821	19.45397999
**ENSG00000102302**	FGD1	12.65607661	17.51602035	26.05546302	25.95424324	34.67366845	26.17734098	12.25673442	3.322625272	2.344812157	8.858885733	20.69406573	4.229126084
**ENSG00000102309**	PIN4	289.6835312	371.3396315	292.3097258	327.3583583	359.4503629	287.9507508	255.3486337	313.9880882	225.5709295	284.7498986	233.1531406	254.5933902
**ENSG00000102312**	PORCN	44.99938349	17.51602035	43.96859385	64.46699127	76.28207059	69.0474791	39.83438686	61.46856754	37.98595695	46.82553888	46.906549	45.67456171
**ENSG00000102313**	ITIH6	0	7.006408141	0	0	0	0.758763507	8.171156279	3.322625272	0.468962431	0	0	1.691650434
**ENSG00000102316**	MAGED2	849.3633634	1054.464425	979.522563	663.0890531	842.5701433	771.6624862	682.2915493	892.1248856	813.1808561	747.9430669	852.5955083	821.2962855
**ENSG00000102317**	RBM3	1501.854424	935.3554868	1221.349829	1339.573845	978.9532392	1236.405134	1225.673442	915.3832625	1119.413324	1273.781213	958.8250457	1227.29239
**ENSG00000102349**	KLF8	101.2486129	122.6121425	100.150686	198.4243757	195.3283323	175.27437	903.9341634	837.3015686	771.4431997	990.2968695	1000.213177	829.7545377

**ENSG00000102359**	SRPX2	15.46853808	17.51602035	10.58503185	10.88403749	38.14103529	14.03712487	27.57765244	8.306563181	61.90304095	31.00610007	33.11050518	39.75378519
**ENSG00000102362**	SYTL4	8.437384405	28.02563256	50.48245961	42.6989163	36.98524634	43.24951988	186.9151999	254.1808333	214.3158312	249.3143556	244.1899757	304.497078
**ENSG00000102384**	CENPI	682.0219061	595.544692	649.7581091	682.3454271	686.5386353	775.8356855	450.4349899	498.3937908	535.5550967	532.7986991	631.8588071	617.4524082
**ENSG00000102385**	DRP2	25.31215321	31.52883663	28.49816268	13.39573845	21.95999002	18.96908767	14.29952349	29.90362745	19.69642212	18.98332657	16.55525259	28.75805737
**ENSG00000102390**	PBDC1	372.6511446	374.8428355	341.9779522	426.989163	343.2693176	335.7528517	221.6426141	264.1487091	235.4191406	224.0032535	229.0143275	272.3557198
**ENSG00000102393**	GLA	243.277917	360.8300193	317.5509556	188.3775719	166.4336086	217.3857446	172.6156764	194.3735784	164.136851	161.9910534	96.57230676	186.0815477
**ENSG00000102401**	ARMCX3	378.2760675	620.0671205	534.136992	370.8945082	533.9744941	471.5715194	549.5102598	657.8798039	505.0725387	461.2948357	491.1391601	505.8034796
**ENSG00000102409**	BEX4	12.65607661	14.01281628	22.79853014	9.209570182	16.18104528	12.14021611	118.481766	69.77513072	88.63389954	113.8999594	70.3598235	78.66174516
**ENSG00000102445**	RUBCNL	2.812461468	0	6.513865756	0.837233653	10.40210053	7.967016819	1.021394535	1.661312636	6.56547404	0.632777552	2.759208765	1.691650434
**ENSG00000102452**	NALCN	126.5607661	87.58010176	90.37988736	274.6126381	241.5598902	211.3156366	2567.785861	2078.302108	1817.229422	2733.599026	2281.865648	1892.11101
**ENSG00000102466**	FGF14	2.812461468	0	2.442699658	0	0	1.13814526	0	9.967875817	7.034436472	5.694997971	12.41643944	7.612426951
**ENSG00000102471**	NDFIP2	5412.582096	4221.360905	4063.837998	5798.68028	4956.02301	5702.487134	5921.024119	6060.468497	6342.247923	6125.286707	6020.593524	6649.877854
**ENSG00000102524**	TNFSF13B	32.34330689	7.006408141	35.01202844	19.25637402	5.778944741	4.931962793	48.00554314	36.54887799	32.35840777	13.92110615	16.55525259	7.612426951
**ENSG00000102531**	FNDC3A	3130.269614	3394.604744	3098.1574	2481.560547	2744.998752	2917.445683	3019.242245	2930.55549	3105.938184	2564.014642	2308.078132	2703.257393
**ENSG00000102543**	CDADC1	136.4043812	119.1089384	126.206149	101.305272	120.2020506	119.1258705	171.5942819	114.6305719	121.4612697	138.578284	155.8952952	139.5611608
**ENSG00000102547**	CAB39L	44.99938349	42.03844885	84.68025482	97.11910373	172.2125533	101.6743099	142.9952349	157.8247004	146.3162786	201.8560392	173.8301522	184.3898973
**ENSG00000102554**	KLF5	2289.343635	2718.486359	2638.929864	1546.370557	1518.706678	1805.098382	1819.103667	1955.364973	1981.366273	2019.825947	2309.457736	2032.517996
**ENSG00000102572**	STK24	6160.696846	6281.244898	6125.47651	5857.286635	6094.475124	5923.287314	6033.377518	10602.49724	6238.138263	5412.146406	9182.646769	5672.103904
**ENSG00000102575**	ACP5	0	0	3.256932878	1.674467306	0	1.896908767	0	4.983937908	2.813774589	2.53111021	0	0
**ENSG00000102580**	DNAJC3	5636.172782	5913.408471	6877.828005	4138.445946	3609.528885	5387.220897	1900.815229	1834.08915	1792.843375	1607.887761	1625.173962	1961.468678
**ENSG00000102595**	UGGT2	1573.572192	1303.191914	1539.715018	1494.46207	1477.098276	1451.135206	1958.013323	1613.13457	1730.00241	1881.247663	1810.04095	1819.370041
**ENSG00000102606**	ARHGEF7	2050.28441	1856.698157	2236.698654	1928.149103	1947.504378	2104.430586	1573.968978	1947.05841	1624.0169	1551.570558	1869.363938	1574.926554
**ENSG00000102678**	FGF9	0	7.006408141	2.442699658	0.837233653	0	1.896908767	2.04278907	0	0	0	4.138813147	1.691650434
**ENSG00000102699**	PARP4	2621.214088	3009.252296	2795.262642	1652.699231	2150.923233	1949.64283	1421.781193	1493.52006	1034.531124	1138.999594	1154.728868	905.8788072
**ENSG00000102710**	SUPT20H	881.7066703	749.6856711	825.6324845	803.7443068	757.0417611	816.4295331	972.3675972	819.0271296	794.8913213	859.3119161	782.2356848	873.7374489
**ENSG00000102738**	MRPS31	329.0579918	280.2563256	329.7644539	241.123292	271.6104028	274.2930076	355.4452981	328.939902	241.0466898	232.8621393	244.1899757	246.9809633
**ENSG00000102743**	SLC25A15	205.3096872	199.682632	209.2579374	301.404115	280.8567144	267.0847543	157.2947584	89.71088235	148.6610908	208.8165923	118.6459769	175.9316451
**ENSG00000102753**	KPNA3	1486.385886	1100.006078	1428.165067	1370.55149	1308.353089	1382.087727	1185.839055	1113.079466	1204.295524	1142.79626	1107.822319	1358.395298
**ENSG00000102755**	FLT1	0	7.006408141	5.699632536	5.86063557	2.311577897	3.035054026	2259.324711	9821.680305	1329.977456	1809.7438	8741.173366	1305.108309
**ENSG00000102763**	VWA8	662.3346758	423.8876925	709.1971341	737.6028482	773.2228064	761.7985606	734.3826706	501.7164161	681.4024129	691.6258648	555.9805661	727.4096864
**ENSG00000102780**	DGKH	2181.063869	1996.82632	2024.183784	2225.367049	1910.519131	2119.226474	1455.487212	1302.469107	1610.41699	1488.925581	1292.689306	1645.130047
**ENSG00000102781**	KATNAL1	502.0243721	791.7241199	495.8680306	612.8550339	776.6901732	661.262396	52.09112128	129.5823856	48.30313044	94.2838553	155.8952952	82.04504603
**ENSG00000102786**	INTS6	1504.666886	1807.6533	1689.53393	1850.286373	1791.47287	1825.964379	1514.728095	1574.924379	1349.204915	1468.043922	1398.918844	1582.538981
**ENSG00000102796**	DHRS12	7.031153671	0	1.628466439	4.186168264	1.155788948	5.6907263	12.25673442	28.24231481	10.31717349	7.593330629	5.518417529	10.99572782
**ENSG00000102804**	TSC22D1	760.7708272	798.7305281	673.3708725	1181.336684	1433.178296	1092.61945	3255.184383	4779.596454	2455.018329	3172.746648	5108.675028	2711.715645
**ENSG00000102805**	CLN5	525.9302946	676.1183856	582.1767519	570.9933513	626.43761	599.0437885	693.5268892	626.3148638	687.0299621	723.2647424	700.8390262	773.0842481
**ENSG00000102837**	OLFM4	118.1233817	479.9389576	591.1333173	138.1435527	856.4396107	828.1903675	2.04278907	3.322625272	9.379248629	3.163887762	5.518417529	12.68737825
**ENSG00000102854**	MSLN	77.34269038	77.07048955	89.56565414	121.3988797	93.61890481	133.5423772	4.08557814	0	0	0	1.379604382	0
**ENSG00000102858**	MGRN1	343.1202991	290.7659378	496.6822639	275.4498718	293.5703929	388.8662971	355.4452981	277.4392102	333.4322888	304.3660027	351.7991175	307.8803789
**ENSG00000102870**	ZNF629	333.276684	402.8684681	442.1286382	277.1243391	406.8377098	291.7445683	209.3858796	257.5034586	129.4336311	146.8043922	238.6715581	133.6403843
**ENSG00000102871**	TRADD	161.7165344	227.7082646	142.4908134	109.6776085	150.2525633	133.9217589	141.9738403	184.4057026	131.3094808	130.9849533	136.5808338	89.65747298
**ENSG00000102878**	HSF4	16.87476881	35.0320407	32.56932878	29.30317785	42.76419109	18.21032416	32.68462512	28.24231481	37.51699452	39.23220825	52.42496653	26.22058172
**ENSG00000102879**	CORO1A	5.624922937	3.50320407	8.956565414	22.60530863	20.80420107	22.00414169	4.08557814	4.983937908	7.503398903	20.24888168	1.379604382	6.766601734
**ENSG00000102882**	MAPK3	500.6181414	833.7625688	722.2248656	752.6730539	856.4396107	851.7120362	958.0680737	888.8022603	1057.979245	1210.503458	1100.924297	992.1529793
**ENSG00000102886**	GDPD3	23.90592248	84.07689769	20.35583049	29.30317785	31.2063016	29.97115851	93.96829721	88.04956971	101.2958852	114.532737	133.8216251	89.65747298
**ENSG00000102890**	ELMO3	106.8735358	161.1473872	135.9769476	180.842469	202.2630659	158.5815729	168.5300983	146.195512	152.8817527	156.928833	180.7281741	113.340579
**ENSG00000102893**	PHKB	1612.946652	1208.605404	1534.015385	1570.650333	1626.19505	1572.916749	1513.706701	1309.114357	1505.838367	1541.446118	1582.406227	1547.860147
**ENSG00000102897**	LYRM1	219.3719945	336.3075908	285.79586	217.6807497	184.9262317	242.8043221	158.3161529	119.6145098	166.9506256	175.279382	164.1729215	154.7860147
**ENSG00000102898**	NUTF2	914.0499772	798.7305281	819.1186188	986.2612431	929.2543144	1070.615308	727.2329088	872.189134	912.1319292	857.4135835	836.0402557	965.9323976
**ENSG00000102900**	NUP93	877.4879781	907.3298542	964.0521318	1054.077169	995.1342845	975.011106	891.6774289	647.9119281	1008.73819	904.2391224	761.541619	998.0737558
**ENSG00000102901**	CENPT	230.6218404	203.1858361	196.2302059	320.660489	212.6651665	251.5301024	304.3755714	347.214341	320.3013407	370.8076457	373.8727876	300.267952
**ENSG00000102904**	TSNAXIP1	12.65607661	3.50320407	0.814233219	4.186168264	4.623155793	0.379381753	10.21394535	21.59706427	6.56547404	22.77999189	11.03683506	2.53747565
**ENSG00000102908**	NFAT5	12647.63922	9308.013215	13058.67237	8312.892939	6856.140041	9262.984889	9922.847906	7382.873355	10149.28494	7409.192361	5711.562143	8498.851778
**ENSG00000102910**	LONP2	1714.195265	1912.749422	1706.632828	1601.627978	1907.051765	1698.49211	1776.205096	1706.168077	1737.036846	1775.573812	1815.559367	1884.498583
**ENSG00000102921**	N4BP1	1137.640664	1443.320077	1214.02173	848.954924	856.4396107	915.4481707	688.4199165	988.4810185	823.9669921	618.8564462	717.3942788	716.4139586
**ENSG00000102931**	ARL2BP	590.6169083	564.0158553	533.3227587	720.0209415	636.8397105	647.2252711	516.8256346	466.8288508	576.8237907	539.7592522	398.7056665	653.8228926
**ENSG00000102934**	PLLP	8.437384405	14.01281628	7.328098975	40.18721534	38.14103529	10.62268909	8.171156279	8.306563181	16.4136851	12.0227735	13.79604382	14.37902869
**ENSG00000102962**	CCL22	8.437384405	24.52242849	39.08319453	5.023401917	2.311577897	5.311344546	5.106972674	13.29050109	14.06887294	0	1.379604382	0
**ENSG00000102967**	DHODH	129.3732275	98.08971397	125.3919158	200.098843	136.3830959	149.8557926	100.0966644	136.2276362	143.0335416	144.2732819	135.2012295	154.7860147
**ENSG00000102974**	CTCF	1257.170276	1208.605404	1363.840643	1321.991938	1254.031009	1284.965998	1442.209083	1413.777053	1455.190425	1429.444491	1542.397699	1456.511023
**ENSG00000102977**	ACD	149.0604578	115.6057343	198.6729055	145.6786556	159.4988749	199.5548022	115.4175824	136.2276362	131.7784432	150.6010575	150.3768777	144.6361121
**ENSG00000102978**	POLR2C	762.1770579	644.589549	918.4550715	784.4879327	836.7911985	859.679053	502.5261112	578.1367974	574.0100161	577.7259053	584.9522581	630.1397865
**ENSG00000102981**	PARD6A	42.18692202	24.52242849	22.79853014	46.04785091	39.29682424	42.87013812	11.23533988	19.93575163	28.13774589	19.61610412	12.41643944	31.29553302
**ENSG00000102984**	ZNF821	33.74953762	80.57369362	24.42699658	37.67551438	56.63365846	39.45570234	43.919965	109.646634	38.45491938	46.19276132	74.49863664	55.82446431
**ENSG00000102996**	MMP15	74.53022891	56.05126513	106.6645517	156.5626931	146.7851964	160.0990999	94.98969174	78.0816939	128.9646686	144.2732819	122.78479	199.6147512
**ENSG00000103005**	USB1	500.6181414	290.7659378	517.8523276	488.1072196	505.0797704	619.9097849	299.2685987	302.3588998	425.8178878	346.7620987	340.7622824	412.7627058
**ENSG00000103018**	CYB5B	2992.459002	2392.68838	2816.432706	2241.274489	2071.173795	2457.634998	1954.94914	1623.102445	2417.032372	1707.233836	1339.595855	2101.875664

**ENSG00000103021**	CCDC113	223.5906867	164.6505913	166.91781	205.122245	241.5598902	166.9279715	287.0118643	196.0348911	260.2741495	256.9076863	260.7452283	257.9766911
**ENSG00000103024**	NME3	144.8417656	80.57369362	118.87805	95.44463643	124.8252064	92.18976605	85.79714093	78.0816939	120.9923073	110.1032941	92.43349361	74.43261908
**ENSG00000103034**	NDRG4	19.68723028	122.6121425	154.7043117	41.02444899	156.031508	208.2805826	2.04278907	1.661312636	12.66198565	2.53111021	5.518417529	10.99572782
**ENSG00000103035**	PSMD7	2680.275779	2249.057013	2364.533269	2214.483012	2179.817956	2170.063629	1765.991151	1732.749079	1692.016453	1700.906061	1685.876555	1914.102466
**ENSG00000103037**	SETD6	340.3078377	196.1794279	215.7718032	346.6147323	312.063016	284.1569332	309.4825441	269.1326471	304.8255804	378.4009763	332.4846561	305.3429033
**ENSG00000103042**	SLC38A7	292.4959927	252.2306931	331.3929203	330.7072929	261.2083023	317.9219093	303.3541769	174.4378268	297.3221815	287.2810088	271.7820633	279.9681468
**ENSG00000103043**	VAC14	973.111668	809.2401403	976.2656301	939.3761585	1043.67742	1039.506004	774.2170574	613.0243627	887.7458827	740.3497363	773.9580585	792.5382281
**ENSG00000103044**	HAS3	1961.691874	1625.486689	1522.61612	1014.727187	873.7764449	726.8954393	46.9841486	74.75906863	106.4544719	22.77999189	75.87824103	67.66601734
**ENSG00000103047**	TANGO6	223.5906867	196.1794279	243.4557326	247.8211612	175.6799201	214.3506906	230.8351649	245.8742701	209.1572444	201.8560392	169.691339	232.6019346
**ENSG00000103051**	COG4	1193.889893	1215.611812	1293.816586	1168.778179	1274.83521	1146.112277	1029.565691	1207.774286	1087.054916	1135.835707	1274.754449	1099.572782
**ENSG00000103056**	SMPD3	21.09346101	24.52242849	15.47043117	10.88403749	12.71367843	13.27836137	15.32091802	24.91968954	13.59991051	8.226108181	12.41643944	9.304077385
**ENSG00000103061**	SLC7A6OS	433.1190661	353.8236111	478.769133	384.2902467	394.1240314	422.2518914	378.9373724	583.1207353	378.9216446	429.0231805	444.2326111	418.6834823
**ENSG00000103064**	SLC7A6	1258.576507	879.3042217	1165.98197	1598.279043	1418.15304	1623.753904	1783.354858	2035.107979	1794.250263	1950.853194	2019.740816	1761.008101
**ENSG00000103066**	PLA2G15	75.93645964	133.1217547	161.2181775	133.9573845	145.6294075	189.3114949	117.4603715	172.7765142	209.1572444	200.5904841	230.3939318	224.9895077
**ENSG00000103067**	ESRP2	1079.985204	924.8458746	991.7360613	1255.013246	1250.563642	1215.918519	1194.010211	1171.225408	1208.047223	1287.702319	1011.250012	1073.3522
**ENSG00000103089**	FA2H	88.59253625	213.6954483	123.7634494	82.04889798	139.8504627	103.1918369	29.62044151	53.16200436	51.58586746	24.67832454	34.49010956	35.5246591
**ENSG00000103091**	WDR59	769.2082116	669.1119775	653.015042	680.6709598	535.130283	637.7407273	616.9222991	574.8141721	560.4101056	604.3025625	724.2923007	653.8228926
**ENSG00000103111**	MON1B	1207.952201	1226.121425	1354.069844	1089.240982	1034.431109	1169.633945	1079.614023	993.4649564	1037.344898	982.0707613	1135.414407	1039.519191
**ENSG00000103121**	CMC2	718.5839052	578.0286716	731.1814311	766.0687924	671.5133789	693.509845	648.5855296	584.7820479	583.8582272	601.7714523	567.0174011	642.8271647
**ENSG00000103126**	AXIN1	537.1801404	399.365264	508.8957622	495.6423225	535.130283	559.5880861	436.1354664	506.700354	540.244721	452.43595	487.000347	439.8291127
**ENSG00000103145**	HCFC1R1	105.4673051	154.1409791	92.0083538	95.44463643	194.1725433	102.8124551	75.58319558	147.8568246	82.53738794	105.0410737	113.1275593	89.65747298
**ENSG00000103148**	NPRL3	340.3078377	269.7467134	327.3217542	300.5668814	314.3745939	353.9631758	382.001556	300.6975871	392.9905176	397.3843029	357.317535	326.4885337
**ENSG00000103150**	MLYCD	268.5900702	192.6762239	293.123959	210.9828805	223.067267	248.1156667	395.279685	267.4713344	400.4939165	494.1992684	366.9747657	416.9918319
**ENSG00000103152**	MPG	149.0604578	175.1602035	203.5583049	194.2382075	166.4336086	166.1692079	188.957989	172.7765142	189.4608223	159.4599432	182.1077785	137.8695103
**ENSG00000103154**	NECAB2	5.624922937	0	0.814233219	1.674467306	1.155788948	0.379381753	1.021394535	0	1.875849726	0	1.379604382	0
**ENSG00000103160**	HSDL1	525.9302946	535.9902228	501.5676632	504.8518927	525.8839715	481.8148267	315.6109113	436.9252233	370.9492833	363.8470926	478.7227207	458.4372675
**ENSG00000103168**	TAF1C	282.6523776	308.2819582	277.6535278	325.683891	255.4293576	283.018788	222.6640086	240.8903322	256.52245	268.9304598	264.8840414	208.0730033
**ENSG00000103174**	NAGPA	71.71776744	115.6057343	78.98062229	92.93293547	95.93048271	124.8165968	107.2464262	93.03350762	95.19937358	101.2444084	115.8867681	101.499026
**ENSG00000103175**	WFDC1	0	0	0	0.837233653	4.623155793	0.758763507	1.021394535	0	0.937924863	0.632777552	1.379604382	1.691650434
**ENSG00000103184**	SEC14L5	0	0	0	2.511700959	1.155788948	1.517527013	0	0	3.28273702	0.632777552	12.41643944	1.691650434
**ENSG00000103187**	COTL1	3256.83038	2378.675564	3286.245274	4997.447674	4202.448616	5027.946377	916.1908978	978.5131427	1492.238457	1079.518504	1125.757176	1716.179365
**ENSG00000103194**	USP10	2529.809091	1888.226994	2351.505538	2507.51479	1976.399102	2419.317441	2155.142469	1769.297957	2158.16511	2110.313137	1678.978533	2155.162652
**ENSG00000103196**	CRISPLD2	1618.571575	2171.986524	1811.668913	2613.006231	3751.690926	3236.126356	983.6029371	2817.586231	1371.24615	1159.881254	2477.769471	1617.217814
**ENSG00000103197**	TSC2	719.9901359	679.6215897	819.1186188	730.0677453	768.5996506	888.1326845	815.0728388	855.5760076	918.6974032	882.7246856	809.8277724	796.7673542
**ENSG00000103199**	ZNF500	102.6548436	77.07048955	109.9214846	92.09570182	91.30732691	109.261945	98.05387535	129.5823856	119.58542	120.8605125	133.8216251	116.7238799
**ENSG00000103202**	NME4	144.8417656	157.6441832	121.3207497	253.6817968	204.5746438	204.1073833	40.8557814	16.61312636	47.83416801	62.01220013	52.42496653	78.66174516
**ENSG00000103222**	ABCC1	725.6150588	550.0030391	675.8135721	752.6730539	893.424857	781.9057936	227.7709813	375.4566558	315.1427539	268.2976822	448.3714243	327.3343589
**ENSG00000103226**	NOMO3	43.59315276	45.54165292	43.96859385	35.16381342	36.98524634	65.63304332	45.96275407	33.22625272	43.61350613	34.16998783	84.15586732	47.36621214
**ENSG00000103227**	LMF1	8.437384405	35.0320407	21.98429693	11.72127114	8.090522638	9.105162079	18.38510163	28.24231481	15.00679781	27.8422123	28.97169203	13.53320347
**ENSG00000103245**	NARFL	236.2467633	98.08971397	225.5426018	234.4254228	219.5999002	245.0806126	240.0277157	186.0670152	233.0743284	255.0093536	223.4959099	177.6232955
**ENSG00000103248**	MTHFSD	374.0573753	336.3075908	464.1129351	340.7540967	374.4756192	435.5302528	412.6433921	412.0055338	353.1287109	392.95486	445.6122155	366.2423189
**ENSG00000103249**	CLCN7	687.646829	711.1504263	842.7313821	673.1358569	699.2523137	947.6956198	651.6497133	569.8302342	706.7263842	705.5469709	736.7087402	684.2726004
**ENSG00000103253**	HAGHL	11.24984587	14.01281628	5.699632536	9.209570182	8.090522638	13.65774312	1.021394535	3.322625272	0.937924863	0	6.898021912	0
**ENSG00000103254**	FAM173A	70.31153671	56.05126513	69.20982365	75.35102876	57.78944741	71.70315137	52.09112128	16.61312636	48.77209287	45.55998377	20.69406573	35.5246591
**ENSG00000103257**	SLC7A5	12237.01985	9514.702255	12598.6306	17802.09916	19996.30459	21904.74367	9723.675972	9231.914319	10788.48074	10838.84669	10469.81766	9611.957763
**ENSG00000103260**	METRN	57.6554601	24.52242849	13.84196473	36.00104707	26.58314581	46.2845739	18.38510163	33.22625272	30.95152048	47.45831643	20.69406573	31.29553302
**ENSG00000103264**	FBXO31	323.4330689	262.7403053	341.1637189	303.915816	279.7009255	341.8229597	300.2899933	212.6480174	294.9773694	312.5921109	233.1531406	244.4434876
**ENSG00000103266**	STUB1	440.1502198	479.9389576	493.425331	483.9210514	515.4818709	544.412816	386.0871342	315.6494009	399.5559916	360.0504273	369.7339745	384.8504736
**ENSG00000103269**	RHBDL1	5.624922937	0	2.442699658	11.72127114	10.40210053	7.967016819	9.192550814	6.645250545	3.28273702	3.163887762	2.759208765	10.99572782
**ENSG00000103274**	NUBP1	1203.733508	1163.063751	1246.591059	807.930475	681.9154795	812.2563338	724.1687252	828.9950054	706.7263842	635.3086626	674.6265429	617.4524082
**ENSG00000103275**	UBE2I	1991.22272	1867.20777	1917.519232	2062.943721	2006.449614	1976.578935	1653.637752	1744.378268	1803.160549	1728.115496	1645.868028	1789.766159
**ENSG00000103313**	MEFV	0	0	9.770798633	0.837233653	3.467366845	2.27629052	13.27812895	6.645250545	2.344812157	1.898332657	2.759208765	6.766601734
**ENSG00000103316**	CRYM	11.24984587	14.01281628	12.21349829	3.348934611	16.18104528	1.896908767	1.021394535	1.661312636	1.406887294	0	8.277626294	0
**ENSG00000103319**	EEF2K	451.4000657	238.2178768	383.5038464	553.4114445	517.7934488	474.9859551	529.0823691	701.0739324	541.6516083	563.1720216	975.3802983	631.8314369
**ENSG00000103326**	CAPN15	423.275451	364.3332233	402.2312104	403.5466207	354.8272071	473.8478099	284.9690752	171.1152015	364.8527717	290.4448965	222.1163056	331.563485
**ENSG00000103335**	PIEZO1	1454.042579	1713.06679	1736.759457	1609.163081	2034.188549	2102.533677	1219.545075	1687.893638	1514.279691	1137.101262	1615.516732	1136.789091
**ENSG00000103342**	GSPT1	11317.34495	8204.503933	10959.57913	11887.0434	10305.01426	12273.75848	9074.069048	9095.686683	10647.79201	9418.893867	9317.847998	11640.24663
**ENSG00000103343**	ZNF174	36.56199909	28.02563256	55.36785892	58.6063557	36.98524634	35.66188481	66.39064477	79.74300653	62.37200338	100.6116308	64.84140597	68.51184256
**ENSG00000103351**	CLUAP1	272.8087624	255.7338971	123.7634494	187.5403382	181.4588649	124.8165968	234.920743	144.5341993	216.1916809	237.2915821	164.1729215	203.8438772
**ENSG00000103353**	UBFD1	1075.766512	1243.637445	1239.26296	991.284645	1005.536385	1091.860686	914.1481087	996.7875817	906.50438	869.436357	876.0487828	798.4590046
**ENSG00000103356**	EARS2	793.1141341	528.9838146	788.1777564	882.4442701	710.8102032	791.7697191	659.8208695	431.9412854	664.0508029	603.669785	568.3970055	757.8593942
**ENSG00000103363**	ELOB	731.2399818	868.7946095	903.7988736	840.5825875	861.0627665	1061.889527	588.3232521	737.6228104	761.1260262	728.3269628	845.6974864	773.0842481
**ENSG00000103365**	GGA2	1244.5142	1271.663078	1275.089222	1538.835454	1620.416105	1534.978574	1459.57279	1746.039581	1523.189977	1499.682799	1823.836993	1620.601115
**ENSG00000103381**	CPPED1	2781.524392	2073.89681	2924.725724	3343.91121	2797.009255	3585.157569	2417.640864	2219.513682	2492.066361	2483.019116	2077.6842	2586.533513
**ENSG00000103404**	USP31	1355.606428	1110.51569	1567.398947	1140.312235	1113.024757	1423.060957	1179.710688	1521.762375	1159.744093	1323.77064	1441.68658	1174.851226
**ENSG00000103415**	HMOX2	371.2449138	199.682632	370.4761148	483.0838177	420.7071772	435.5302528	154.2305748	184.4057026	215.7227185	220.8393658	219.3570968	223.2978572
**ENSG00000103423**	DNAJA3	752.3334428	493.9517739	765.3792263	725.0443434	694.6291579	800.4954995	612.8367209	460.1836002	624.1889963	620.7547789	513.2128302	634.3689126

**ENSG00000103429**	BFAR	663.7409065	889.8138339	914.3839054	734.2539136	844.8817212	787.5965199	728.2543034	787.4621895	795.8292462	627.0825544	772.5784541	734.1762882
**ENSG00000103479**	RBL2	2337.15548	2242.050605	2276.596082	1767.400241	2124.340087	1829.758196	1666.915881	1883.928529	1533.507151	1628.76942	1888.678399	1627.367717
**ENSG00000103485**	QPRT	1026.548436	893.317038	876.9291773	915.0963826	815.9869975	855.8852355	354.4239036	539.9266067	543.9964205	356.8865395	601.5075107	503.266004
**ENSG00000103489**	XYLT1	299.5271464	322.2947745	237.7561001	303.0785823	325.9324834	291.3651865	1348.240786	965.2226416	1539.1347	1545.87556	1124.377572	1733.095869
**ENSG00000103490**	PYCARD	74.53022891	45.54165292	60.25325824	13.39573845	36.98524634	45.14642864	3.064183605	4.983937908	5.627549177	2.53111021	16.55525259	5.920776517
**ENSG00000103494**	RPGRIP1L	440.1502198	388.8556518	315.1082559	421.1285274	502.7681925	341.8229597	502.5261112	546.5718573	437.0729861	591.0142339	529.7680828	531.1782361
**ENSG00000103495**	MAZ	2521.371706	2203.51536	2412.573029	3443.542014	3589.880473	3546.840012	3603.479919	3613.354984	3852.057412	3944.102484	4000.852709	3934.778908
**ENSG00000103496**	STX4	1001.236283	1411.79124	1113.056811	738.4400818	1010.159541	883.2007217	895.7630071	960.2387037	885.870033	673.2753157	776.7172672	664.8186204
**ENSG00000103502**	CDIPT	860.6132093	753.1888751	848.4310147	873.2346999	981.2648171	1048.611166	1121.491199	737.6228104	1283.081212	1113.055715	790.5133111	1177.388702
**ENSG00000103507**	BCKDK	705.9278285	959.8779153	845.988315	934.3527566	1012.471119	1009.534846	544.4032871	661.2024292	585.7340769	592.279789	688.4225868	577.6986231
**ENSG00000103510**	KAT8	1034.98582	1012.425976	1066.645517	1131.102665	1132.673169	1189.361797	1198.095789	1074.869276	1142.861445	1230.119562	1125.757176	1003.994532
**ENSG00000103512**	NOMO1	641.2412148	672.6151815	710.0113674	720.8581751	831.0122538	892.3058838	646.5427406	662.8637418	707.1953466	707.4453036	840.1790688	770.5467725
**ENSG00000103528**	SYT17	49.2180757	28.02563256	54.5536257	29.30317785	43.91998003	47.42271916	110.3106098	79.74300653	110.2061714	146.1716146	75.87824103	135.3320347
**ENSG00000103534**	TMC5	208.1221487	224.2050605	280.9104607	260.379666	276.2335586	331.9590341	222.6640086	127.921073	202.5917704	250.5799107	142.0992514	257.1308659
**ENSG00000103540**	CCP110	1192.483663	1474.848914	1084.558648	1327.852573	2229.516881	1268.273201	1827.274823	2339.128192	1659.189082	1751.528265	2297.041297	1887.881884
**ENSG00000103544**	VPS35L	483.7433725	332.8043867	499.1249635	440.3849014	403.3703429	512.5447487	732.3398815	701.0739324	646.2302305	597.974787	615.3035545	594.6151274
**ENSG00000103546**	SLC6A2	0	0	0	0	0	2.27629052	0	0	0.468962431	3.796665314	0	0
**ENSG00000103549**	RNF40	2504.496938	2382.178768	2774.906812	2690.031727	2890.62816	3025.190101	2296.094914	2563.405398	2567.569312	2282.428631	2658.497645	2511.255069
**ENSG00000103550**	KNOP1	1084.203896	847.775385	1089.444048	1262.548349	1077.1953	1187.085506	768.0886902	795.7687527	914.0077789	929.5502244	776.7172672	929.5619132
**ENSG00000103591**	AAGAB	1258.576507	1029.941997	1073.159383	1175.476049	1085.285822	1111.588537	841.6290967	1147.967032	919.1663656	867.5380243	932.6125624	1044.594143
**ENSG00000103599**	IQCH	95.62368992	59.5544692	96.07951989	92.09570182	98.2420606	76.25573241	94.98969174	74.75906863	90.9787117	58.84831237	137.9604382	82.89087124
**ENSG00000103642**	LACTB	262.9651473	493.9517739	377.8042138	102.9797393	209.1977996	113.814526	133.8026841	275.7778976	149.1300532	108.2049615	195.9038223	182.6982468
**ENSG00000103647**	CORO2B	1.406230734	0	5.699632536	0	3.467366845	1.517527013	0	0	0.468962431	0	4.138813147	0
**ENSG00000103653**	CSK	395.1508363	315.2883663	449.4567371	392.6625832	371.0082524	469.6746106	254.3272392	260.8260839	258.8672622	235.3932495	234.532745	203.8438772
**ENSG00000103657**	HERC1	838.1135176	1061.470833	915.1981387	1010.541019	1113.024757	1085.790578	1029.565691	1236.016601	960.4350596	1109.259049	1278.893262	1058.127346
**ENSG00000103671**	TRIP4	489.3682955	441.4037129	495.8680306	401.0349197	351.3598403	356.2394664	283.9476807	249.1968954	253.7086754	230.3310291	153.1360864	213.1479546
**ENSG00000103707**	MTFMT	71.71776744	91.08330583	78.98062229	134.7946181	114.4231059	108.8825632	68.43343384	39.87150327	45.48935585	55.68442461	75.87824103	80.35339559
**ENSG00000103723**	AP3B2	0	0	0.814233219	2.511700959	1.155788948	1.13814526	8.171156279	3.322625272	0.468962431	0	0	0.845825217
**ENSG00000103740**	ACSBG1	0	3.50320407	17.09889761	0.837233653	10.40210053	13.27836137	2.04278907	3.322625272	2.344812157	1.898332657	1.379604382	0
**ENSG00000103742**	IGDCC4	0	3.50320407	3.256932878	1.674467306	1.155788948	0.758763507	0	0	0.937924863	0	0	0
**ENSG00000103769**	RAB11A	1306.388352	1278.669486	1349.184445	1472.693995	1354.584647	1504.628034	799.7519208	1026.691209	727.3607312	728.9597403	769.8192453	810.3005577
**ENSG00000103811**	CTSH	111.092228	238.2178768	218.2145028	106.3286739	183.7704428	179.0681876	249.2202665	219.293268	234.4812157	318.9198864	263.504437	352.7091154
**ENSG00000103832**	GOLGA8UP	9.843615139	3.50320407	30.12662912	7.535102876	11.55788948	14.41650663	19.40649616	23.25837691	26.26189616	27.8422123	17.93485697	16.07067912
**ENSG00000103852**	TTC23	298.1209156	360.8300193	296.3808919	303.0785823	291.258815	287.1919873	135.8454731	139.5502614	151.4748654	137.3127289	176.5893609	155.6318399
**ENSG00000103855**	CD276	307.9645308	465.9261414	381.8753799	488.9444533	460.0040014	547.0684883	261.4770009	290.7297113	309.9841672	298.0382272	255.2268107	354.4007658
**ENSG00000103876**	FAH	108.2797665	136.6249587	119.6922833	142.329721	172.2125533	141.8887757	44.94135953	78.0816939	64.71681554	69.60553076	74.49863664	78.66174516
**ENSG00000103888**	CEMIP	402.18199	893.317038	706.7544345	533.3178369	1359.207803	907.4811539	32.68462512	33.22625272	40.79973154	68.97275321	68.98021912	75.27844429
**ENSG00000103932**	RPAP1	322.0268381	329.3011826	394.0888782	344.1030313	405.6819208	401.0065132	312.5467277	260.8260839	307.639355	299.3037823	257.9860195	307.8803789
**ENSG00000103942**	HOMER2	25.31215321	21.01922442	16.28466439	15.9074394	28.89472371	9.484543833	15.32091802	29.90362745	15.94472267	24.04554699	16.55525259	10.99572782
**ENSG00000103966**	EHD4	414.8380666	672.6151815	521.1092604	423.6402284	462.3155793	509.8890764	264.5411845	433.602598	354.0666357	270.8287924	303.5129641	335.7926111
**ENSG00000103978**	TMEM87A	966.0805144	970.3875275	1049.54662	872.3974663	743.1722937	921.138897	888.6132453	913.7219499	777.5397113	739.7169587	768.4396409	827.217062
**ENSG00000103994**	ZNF106	1972.94172	2077.400014	1874.364871	2125.736245	2060.771695	2058.146012	2014.190023	2227.820245	2066.717435	2140.68646	2079.063804	2356.469054
**ENSG00000103995**	CEP152	1275.451276	1110.51569	1181.452401	1521.253547	1448.203552	1341.49388	1723.09258	1551.666002	1388.59776	1757.223263	1743.819939	1849.819749
**ENSG00000104043**	ATP8B4	0	0	2.442699658	0	1.155788948	3.035054026	10.21394535	0	3.28273702	1.898332657	0	0
**ENSG00000104047**	DTWD1	309.3707615	266.2435094	226.356835	299.7296477	295.8819708	270.8785719	219.599825	179.4217647	227.4467793	255.6421312	173.8301522	252.0559146
**ENSG00000104055**	TGM5	0	0	0	0	1.155788948	0	1.021394535	9.967875817	0.937924863	4.429442867	0	3.383300867
**ENSG00000104064**	GABPB1	576.554601	542.9966309	669.2997064	549.2252763	427.6419109	589.5592446	459.6275407	400.3763453	423.0041132	336.0048803	332.4846561	442.3665884
**ENSG00000104067**	TJP1	4934.463646	4547.158883	4955.423374	5780.261139	5128.235563	5688.829391	3590.20179	3649.903862	3634.927806	3614.425379	3277.940012	3823.12998
**ENSG00000104081**	BMF	26.71838395	66.56087734	27.68392946	31.81487881	63.56839215	29.212395	127.6743169	98.01744553	83.00635037	180.97438	107.6091418	81.19922081
**ENSG00000104093**	DMXL2	1403.418273	2038.864769	1527.50152	1391.482331	1619.260317	1390.434126	1123.533988	1136.337843	996.0762044	1037.122408	1089.887462	1095.343656
**ENSG00000104112**	SCG3	1.406230734	17.51602035	1.628466439	0.837233653	0	1.13814526	0	0	0	0.632777552	0	0
**ENSG00000104129**	DNAJC17	125.1545353	136.6249587	135.1627144	144.8414219	112.111528	119.1258705	129.7171059	122.9371351	131.7784432	136.6799513	157.2748996	112.4947538
**ENSG00000104131**	EIF3J	1659.352266	949.3683031	1479.46176	2151.690488	1605.390849	1992.133587	1100.041914	769.1877505	1089.399728	1154.819033	815.3461899	1385.461705
**ENSG00000104133**	SPG11	2571.996013	2764.028012	2435.371559	1724.701325	1807.653915	1707.597272	2327.758145	2214.529744	2065.779511	1616.746646	1741.06073	1540.24772
**ENSG00000104140**	RHOV	36.56199909	24.52242849	31.75509556	110.5148422	136.3830959	67.52995209	209.3858796	106.3240087	157.571377	191.7315984	99.33151553	192.0023242
**ENSG00000104142**	VPS18	233.4343019	126.1153465	256.4834641	224.378619	180.3030759	295.1590041	199.1719343	151.1794499	239.6398025	220.2065882	242.8103713	216.5312555
**ENSG00000104147**	OIP5	164.5289959	140.1281628	152.261612	266.2403016	241.5598902	238.6311228	156.2733638	164.469951	196.9642212	187.3021555	200.0426354	223.2978572
**ENSG00000104154**	SLC30A4	352.9639143	395.86206	407.1166097	316.4743208	453.0692677	379.3817533	296.2044151	232.5837691	283.722271	224.6360311	231.7735362	278.2764963
**ENSG00000104164**	BLOC1S6	880.3004396	910.8330583	775.9642581	910.9102143	762.8207059	835.3986208	576.0665177	425.2960348	518.6724492	589.7486788	579.4338406	619.1440587
**ENSG00000104177**	MYEF2	707.3340593	648.092753	942.8820681	745.1379511	858.7511886	927.2090051	366.680638	232.5837691	327.3357772	310.6937782	259.3656239	325.6427085
**ENSG00000104205**	SGK3	212.3408409	126.1153465	158.7754778	132.2829172	128.2925733	121.7815428	94.98969174	51.50069172	72.68917688	65.80886545	59.32298844	71.04931821
**ENSG00000104213**	PDGFRL	25.31215321	17.51602035	13.02773151	19.25637402	8.090522638	17.0721789	5.106972674	11.62918845	10.31717349	8.858885733	20.69406573	5.074951301
**ENSG00000104218**	CSPP1	1186.85874	858.2849973	933.1112695	1298.549396	1153.47737	1142.697841	1069.400078	910.3993246	866.1736109	1093.439611	887.0856178	1021.756862
**ENSG00000104219**	ZDHHC2	1272.638814	1240.134241	1342.670579	1491.950369	1649.310829	1646.137428	244.1132938	245.8742701	239.6398025	260.7043516	180.7281741	232.6019346
**ENSG00000104221**	BRF2	255.9339936	126.1153465	228.7995347	197.5871421	169.9009754	223.4558527	98.05387535	121.2758224	115.3647581	75.93330629	122.78479	81.19922081
**ENSG00000104228**	TRIM35	303.7458386	217.1986524	366.4049488	292.1945449	262.3640913	325.8889261	158.3161529	162.8086383	176.7988367	189.2004882	184.8669872	188.6190233
**ENSG00000104231**	ZFAND1	1394.980888	1271.663078	1490.861025	1240.780274	1061.014255	1239.81957	1364.583099	1013.400708	1162.088905	1124.445711	927.0941449	1124.101713

**ENSG00000104237**	RP1	2.812461468	7.006408141	6.513865756	0.837233653	5.778944741	5.311344546	0	0	0	0	0	0
**ENSG00000104267**	CA2	25.31215321	52.54806106	53.73939248	5.023401917	9.246311586	6.828871559	33.70601965	21.59706427	17.8205724	23.41276944	15.17564821	15.2248539
**ENSG00000104290**	FZD3	258.7464551	266.2435094	242.6414994	431.1753312	411.4608656	364.5858649	130.7385005	91.37219499	98.48211061	170.2171616	102.0907243	133.6403843
**ENSG00000104299**	INTS9	302.3396078	287.2627338	357.4483833	344.1030313	312.063016	377.1054628	167.5087037	124.5984477	203.5296953	174.0138269	124.1643944	170.8566938
**ENSG00000104312**	RIPK2	1736.694957	2550.332563	1889.835302	588.575258	842.5701433	672.6438486	929.4690267	1227.710038	904.1595678	526.4709236	756.0232015	559.0904683
**ENSG00000104313**	EYA1	8.437384405	24.52242849	26.86969624	5.023401917	40.45261319	31.10930377	74.56180105	61.46856754	56.74445421	62.64497769	53.80457091	75.27844429
**ENSG00000104320**	NBN	3379.172454	3271.992602	3046.860707	2093.921366	1944.037011	1851.382956	3004.942722	2314.208502	2882.712066	2238.134203	2005.944772	2276.961484
**ENSG00000104321**	TRPA1	0	0	1.628466439	1.674467306	0	0.758763507	812.0086552	730.9775599	630.7544703	867.5380243	878.8079915	756.1677438
**ENSG00000104324**	CPQ	14.06230734	17.51602035	26.86969624	0	2.311577897	5.6907263	8.171156279	1.661312636	3.751699452	3.796665314	2.759208765	3.383300867
**ENSG00000104325**	DECR1	1216.389585	959.8779153	944.5105346	979.5633739	825.2333091	741.311946	1954.94914	1872.299341	1833.174145	1536.383897	1575.508205	1685.729657
**ENSG00000104327**	CALB1	849.3633634	1569.435424	459.2275358	746.8124184	1517.550889	539.4808532	124.6101333	343.8917157	144.4404289	127.8210656	280.0596896	139.5611608
**ENSG00000104331**	IMPAD1	2834.96116	2827.085685	2836.788537	3228.372965	3385.305829	3550.254447	2057.088593	3352.5289	2172.233982	2146.381458	3145.497992	2512.100894
**ENSG00000104341**	LAPTM4B	1136.234433	1145.547731	1082.115949	1777.447045	1541.822457	1398.401143	726.2115143	682.7994934	1008.73819	985.8674266	907.7796836	1241.671418
**ENSG00000104343**	UBE2W	1072.95405	879.3042217	997.4356938	1023.936757	899.2038017	1026.607024	512.7400565	468.4901634	501.3208392	534.0642542	471.8246987	552.3238666
**ENSG00000104356**	POP1	722.8025974	406.3716722	635.1019112	771.9294279	558.246062	685.92221	323.7820676	189.3896405	314.6737915	367.0109804	281.439294	417.8376571
**ENSG00000104361**	NIPAL2	130.7794583	171.6569995	153.8900785	120.561646	242.7156791	193.1053124	70.47622291	79.74300653	40.3307691	47.45831643	49.66575776	38.90795997
**ENSG00000104365**	IKBKB	798.739057	700.6408141	864.7156791	857.3272605	694.6291579	813.3944791	421.8359429	347.214341	420.1903386	442.3115091	333.8642605	376.3922215
**ENSG00000104368**	PLAT	466.8686037	441.4037129	656.2719749	462.99021	477.3408356	660.8830143	66.39064477	64.79119281	74.09606417	48.09109398	106.2295374	54.97863909
**ENSG00000104369**	JPH1	26.71838395	73.56728548	21.98429693	68.65315954	69.3473369	48.18148267	7.149761744	0	19.22745969	24.04554699	15.17564821	18.60815477
**ENSG00000104375**	STK3	2084.033948	1912.749422	2052.681946	1829.355532	1678.205553	1918.154145	1128.640961	1088.159777	1117.537474	1009.280196	954.6862326	1072.506375
**ENSG00000104381**	GDAP1	461.2436808	343.3139989	392.4604118	351.6381342	282.0125034	314.5074735	312.5467277	194.3735784	278.5636843	214.5115903	172.4505478	271.5098946
**ENSG00000104388**	RAB2A	2407.467017	2357.656339	2287.181113	2179.319198	2305.798952	2369.239049	1820.125061	1739.39433	1903.049547	1679.391624	1569.989787	1814.29509
**ENSG00000104408**	EIF3E	4681.342114	4196.838476	4253.554338	5094.566778	4578.080024	4710.024467	3513.5972	3158.155321	3243.344176	3256.906062	3108.248673	3678.493868
**ENSG00000104412**	EMC2	527.3365253	697.13761	491.7968645	400.1976861	475.0292577	410.4910571	382.001556	380.4405937	365.3217341	308.162668	373.8727876	345.9425137
**ENSG00000104413**	ESRP1	2663.40101	2466.255666	2486.668252	2870.036962	3181.886975	2827.532207	2189.869883	1722.781204	2283.378079	2090.064256	1881.780377	2385.227111
**ENSG00000104415**	WISP1	0	0	0	0	0	0	6.128367209	8.306563181	12.66198565	3.163887762	6.898021912	10.1499026
**ENSG00000104419**	NDRG1	680.6156753	4375.501884	800.3912547	504.014659	3674.253067	573.625211	2463.603618	19332.69515	1644.182285	2527.946322	20009.78196	1638.363445
**ENSG00000104427**	ZC2HC1A	81.56138258	126.1153465	125.3919158	122.2361133	121.3578396	114.5732895	117.4603715	116.2918845	112.0820211	135.4143962	118.6459769	105.7281521
**ENSG00000104432**	IL7	25.31215321	24.52242849	40.71166097	0.837233653	0	0.379381753	2.04278907	6.645250545	2.813774589	0	0	0
**ENSG00000104442**	ARMC1	1680.445727	1429.307261	1411.880403	1716.328988	1670.11503	1682.178694	908.0197415	784.1395643	965.1246839	909.3013428	783.6152892	1014.99026
**ENSG00000104447**	TRPS1	2030.59718	1583.44824	1678.948898	3041.669861	2475.699927	2148.438869	353.4025091	388.7471569	339.0598379	286.0154537	271.7820633	324.7968832
**ENSG00000104450**	SPAG1	354.370145	220.7018564	259.740397	370.0572746	352.5156292	385.0724796	192.0221726	385.4245316	213.8468687	158.1943881	270.4024589	206.3813529
**ENSG00000104472**	CHRAC1	597.648062	679.6215897	582.1767519	609.5060993	713.1217811	596.3881162	679.2273657	805.7366285	623.2510714	696.0553076	662.2101035	821.2962855
**ENSG00000104490**	NCALD	30.93707615	77.07048955	64.32442434	38.51274803	47.38734688	49.69900968	5.106972674	9.967875817	7.972361335	5.062220419	2.759208765	5.074951301
**ENSG00000104497**	SNX16	396.557067	542.9966309	409.5593094	310.6136852	310.9072271	359.6539021	201.2147234	234.2450817	262.1499992	223.370476	219.3570968	227.5269833
**ENSG00000104517**	UBR5	2289.343635	2361.159543	2211.457424	2609.657296	2587.811455	2493.676264	2200.083828	2055.043731	2115.958491	2322.293617	2371.539933	2216.907893
**ENSG00000104518**	GSDMD	592.0231391	606.0543042	560.192455	231.0764882	300.5051265	306.9198384	435.1140719	390.4084695	422.0661883	255.6421312	231.7735362	206.3813529
**ENSG00000104522**	TSTA3	473.8997574	308.2819582	438.8717053	393.4998168	361.7619408	417.3199286	288.0332588	201.018829	358.2872976	226.5343638	238.6715581	296.8846511
**ENSG00000104524**	PYCR3	194.0598413	98.08971397	109.1072514	168.2839642	136.3830959	161.9960087	144.0166294	59.8072549	154.7576024	170.8499391	97.95191114	175.0858199
**ENSG00000104529**	EEF1D	3671.668447	3293.011826	3695.804583	3575.824931	3162.238562	3667.104027	2459.51804	2606.599526	2906.160188	2553.257424	2553.647712	2483.342836
**ENSG00000104549**	SQLE	1184.046278	1198.095792	1224.606762	1374.737658	1284.081522	1323.662937	745.6180105	1362.276362	1018.586401	906.137455	1449.964206	1283.116854
**ENSG00000104611**	SH2D4A	1241.701738	1555.422607	1410.251936	1054.914403	1396.19305	1209.089648	764.0031121	892.1248856	831.0014285	644.8003259	744.9863664	769.7009473
**ENSG00000104613**	INTS10	859.2069786	774.2080996	850.8737143	785.3251664	742.0165048	826.2934587	453.4991735	382.1019063	453.4866712	467.6226112	416.6405235	488.8869753
**ENSG00000104626**	ERI1	984.3615139	1156.057343	964.0521318	853.9783259	960.460616	783.4233206	484.1410095	415.328159	394.3974049	486.6059378	409.7425015	421.220958
**ENSG00000104635**	SLC39A14	2117.783486	2466.255666	2113.749438	1927.311869	1967.15279	2160.958467	344.2099583	604.7177995	499.4449895	381.5648641	584.9522581	537.0990127
**ENSG00000104643**	MTMR9	523.1178331	455.4165292	618.0030136	549.2252763	520.1050267	613.0809133	175.67986	159.4860131	183.3643107	183.5054902	213.8386793	217.3770807
**ENSG00000104660**	LEPROTL1	974.5178988	914.3362624	939.6251352	1258.36218	1186.99525	1297.864978	312.5467277	232.5837691	406.5904281	323.9821068	333.8642605	402.6128032
**ENSG00000104671**	DCTN6	392.3383748	311.7851623	429.9151399	444.5710697	404.5261319	430.9776718	91.92550814	117.9531972	133.654293	141.7421717	114.5071637	157.3234903
**ENSG00000104679**	R3HCC1	178.5913032	189.1730198	177.5028418	216.8435161	218.4441112	244.7012309	81.71156279	91.37219499	77.37880119	90.48718999	118.6459769	82.04504603
**ENSG00000104687**	GSR	514.6804487	479.9389576	589.5048509	507.3635936	540.9092278	600.1819337	364.637849	230.9224564	370.0113584	395.4859702	244.1899757	417.8376571
**ENSG00000104689**	TNFRSF10A	916.8624387	742.6792629	1060.131652	673.1358569	544.3765946	684.0253012	163.4231256	81.40431917	143.9714665	144.9060595	100.7111199	144.6361121
**ENSG00000104691**	UBXN8	144.8417656	119.1089384	109.1072514	159.074394	122.5136285	136.9568129	41.87717593	36.54887799	51.11690503	54.4188695	33.11050518	66.82019213
**ENSG00000104695**	PPP2CB	1468.104886	1558.925811	1561.699315	1558.091828	1474.786698	1729.601413	437.1568609	536.6039815	566.0376548	453.7015051	613.9239501	590.3860013
**ENSG00000104714**	ERICH1	198.2785335	185.6698157	150.6331456	133.1201508	151.4083522	134.6805224	171.5942819	217.6319553	153.8196775	209.4493698	260.7452283	157.3234903
**ENSG00000104722**	NEFM	1.406230734	0	1.628466439	3.348934611	1.155788948	1.13814526	0	1.661312636	0	0	0	0
**ENSG00000104723**	TUSC3	1537.010192	1425.804057	1308.472784	1545.533323	1463.228809	1446.203244	796.6877372	767.5264379	792.0775467	796.0341609	874.6691784	940.5576411
**ENSG00000104728**	ARHGEF10	46.40561423	21.01922442	52.11092604	44.3733836	35.8294574	48.56086442	14.29952349	36.54887799	22.97915914	22.14721433	34.49010956	37.21630954
**ENSG00000104731**	KLHDC4	534.367679	332.8043867	548.7931899	576.8539868	506.2355593	627.1180382	550.5316543	430.2799728	536.0240592	593.5453441	470.4450944	482.1203736
**ENSG00000104738**	MCM4	3301.829764	3818.492437	3021.619477	3420.099472	3608.373096	3337.041902	2941.61626	3254.511454	3151.896502	3178.441646	3195.163749	3214.981649
**ENSG00000104756**	KCTD9	1520.135424	2256.063421	1569.027414	1499.485472	1621.571894	1572.537367	518.8684237	687.7834314	605.430499	449.9048397	699.4594218	676.6601734
**ENSG00000104763**	ASAH1	1196.702355	1208.605404	1213.207497	1095.938852	1315.287823	1174.94529	1641.381018	1247.64579	1678.885505	1684.453844	1156.108472	1765.237227
**ENSG00000104765**	BNIP3L	1462.479964	4746.841515	1687.905464	1342.922779	3576.011006	1531.184756	772.1742684	3028.572936	574.0100161	584.0536808	2411.54846	660.5894943
**ENSG00000104774**	MAN2B1	161.7165344	206.6890402	216.5860364	146.5158893	183.7704428	152.8908466	171.5942819	240.8903322	230.7295163	201.8560392	252.467602	224.9895077
**ENSG00000104783**	KCNN4	381.088529	304.7787541	359.891083	389.3136486	349.0482624	418.8374556	90.9041136	58.14594226	105.9855095	142.3749493	51.04536215	118.4155303
**ENSG00000104805**	NUCB1	1091.23505	1296.185506	1332.89978	640.4837444	766.2880727	777.7325943	984.6243316	1064.9014	1026.0898	863.1085814	956.0658369	839.058615
**ENSG00000104808**	DHDH	7.031153671	0	11.39926507	8.372336529	3.467366845	6.828871559	0	3.322625272	11.25509835	2.53111021	0	3.383300867
**ENSG00000104812**	GYS1	592.0231391	514.9709984	543.9077906	684.857128	762.8207059	699.9593348	757.8747449	981.835768	923.85599	778.3163894	942.2697931	932.9452141

ENSG00000104814	MAP4K1	4.218692202	0	9.770798633	2.511700959	1.155788948	1.896908767	1.021394535	4.983937908	3.751699452	7.593330629	1.379604382	0.845825217
ENSG00000104823	ECH1	146.2479964	140.1281628	172.6174425	206.7967123	196.4841212	217.3857446	295.1830206	161.1473257	308.5772799	323.9821068	140.719647	293.5013502
ENSG00000104824	HNRNPL	2231.688175	1905.743014	2247.283686	2632.262605	2313.889474	2497.849464	2104.072742	2216.191057	2473.776826	2400.125256	2370.160329	2567.079533
ENSG00000104825	NFKBIB	215.1533023	252.2306931	258.1119306	232.7509555	301.6609155	309.1961289	203.2575124	240.8903322	255.1155627	168.9516065	195.9038223	219.9145564
ENSG00000104833	TUBB4A	1.406230734	10.50961221	0	1.674467306	5.778944741	0.379381753	0	0	0.468962431	0	0	0
ENSG00000104835	SARS2	23.90592248	0	17.09889761	14.2329721	12.71367843	13.65774312	11.23533988	11.62918845	19.22745969	21.51443678	16.55525259	21.99145564
ENSG00000104852	SNRNP70	1109.516049	1145.547731	1073.159383	1128.590964	1036.742687	1076.685416	974.4103863	862.2212581	1014.834702	1121.9146	987.7967377	1144.401518
ENSG00000104853	CLPTM1	795.9265955	714.6536304	927.411637	982.0750748	959.3048271	1139.662787	642.4571624	631.2988017	748.933003	717.5697444	721.5330919	746.8636664
ENSG00000104856	RELB	239.0592248	451.9133251	297.1951251	154.0509921	176.8357091	255.32392	94.98969174	136.2276362	95.19937358	51.8877593	97.95191114	68.51184256
ENSG00000104859	CLASRP	257.3402244	224.2050605	217.4002696	236.0998901	249.6504128	237.1135958	359.5308763	222.6158932	245.7363141	297.4054496	282.8188984	229.2186337
ENSG00000104863	LIN7B	8.437384405	7.006408141	22.79853014	18.41914036	24.27156791	15.93403364	28.59904698	21.59706427	22.51019671	8.226108181	24.83287888	16.07067912
ENSG00000104866	PPP1R37	205.3096872	192.6762239	130.2773151	175.8190671	197.6399102	177.1712788	195.0863562	410.3442211	218.0675306	173.3810494	482.8615338	213.1479546
ENSG00000104870	FCGRT	30.93707615	42.03844885	14.65619795	14.2329721	17.33683422	11.76083435	0	1.661312636	0.937924863	0	1.379604382	0
ENSG00000104872	PIH1D1	323.4330689	273.2499175	306.1516905	335.7306948	321.3093276	320.9569633	290.0760479	333.9238399	263.5568865	274.6254577	237.2919538	225.8353329
ENSG00000104880	ARHGEF18	871.8630552	1054.464425	1001.50686	811.2794096	1009.003752	1148.009185	1360.49752	1819.137337	1462.224861	1335.160636	2000.426354	1347.39957
ENSG00000104881	PPP1R13L	1092.64128	1061.470833	1188.7805	1174.638815	1262.121532	1261.44433	994.838277	867.2051961	1020.931213	954.8613265	938.13098	910.1079333
ENSG00000104883	PEX11G	16.87476881	28.02563256	5.699632536	3.348934611	13.86946738	2.655672273	11.23533988	14.95181373	7.503398903	14.5538837	9.657230676	4.229126084
ENSG00000104884	ERCC2	81.56138258	115.6057343	79.79485551	98.79357104	65.87997005	92.18976605	104.1822426	94.69482026	124.7440068	115.1655145	106.2295374	109.111453
ENSG00000104885	DOT1L	712.9589822	732.1696507	669.2997064	852.3038586	936.1890481	964.3884169	982.5815426	1290.839918	946.8351491	1058.004068	1426.510931	989.6155036
ENSG00000104886	PLEKHJ1	182.8099954	210.1922442	206.0010045	179.1680017	190.7051765	220.4207987	184.8724108	141.2115741	200.2469582	176.5449371	125.5439988	166.6275677
ENSG00000104888	SLC17A7	2.812461468	0	1.628466439	4.186168264	4.623155793	7.967016819	4.08557814	1.661312636	3.751699452	0	2.759208765	10.1499026
ENSG00000104889	RNASEH2A	21.09346101	17.51602035	14.65619795	18.41914036	20.80420107	13.27836137	21.44928523	23.25837691	14.53783538	13.2883286	23.4532745	11.84155303
ENSG00000104892	KLC3	129.3732275	150.637775	117.2495836	200.9360767	134.071518	200.6929475	40.8557814	33.22625272	37.04803208	24.67832454	38.6289227	44.82873649
ENSG00000104894	CD37	0	0	5.699632536	0	0	0.379381753	1.021394535	3.322625272	4.689624315	1.898332657	0	0
ENSG00000104897	SF3A2	925.2998231	721.6600385	916.0123719	955.2835979	829.8564649	1044.437967	959.0894682	872.189134	1019.993288	889.6852386	936.7513756	905.032982
ENSG00000104899	AMH	12.65607661	24.52242849	9.770798633	15.9074394	5.778944741	9.105162079	9.192550814	3.322625272	4.689624315	10.12444084	11.03683506	6.766601734
ENSG00000104904	OAZ1	2867.304467	3002.245888	3127.469796	2642.309408	2843.240813	3305.553217	2299.159098	2794.327854	2892.560277	2435.560799	2619.868722	2704.103218
ENSG00000104907	TRMT1	396.557067	262.7403053	459.2275358	410.2444899	376.7871971	445.0147966	303.3541769	239.2290196	287.005008	303.7332251	222.1163056	307.0345537
ENSG00000104915	STX10	177.1850725	213.6954483	248.3411319	299.7296477	276.2335586	299.3322034	136.8668677	147.8568246	167.8885505	162.623831	177.9689653	169.1650434
ENSG00000104936	DMPK	97.02992066	87.58010176	114.8068839	134.7946181	149.0967743	99.39801937	150.1449966	184.4057026	151.4748654	147.4371697	136.5808338	103.1906764
ENSG00000104938	CLEC4M	0	7.006408141	0.814233219	0	6.93473369	1.13814526	0	0	0	0	0	2.53747565
ENSG00000104941	RSPH6A	1.406230734	3.50320407	0	2.511700959	0	0.379381753	1.021394535	1.661312636	0	1.265555105	0	0
ENSG00000104946	TBC1D17	220.7782253	220.7018564	208.4437042	133.9573845	224.223056	178.3094241	259.4342119	247.5355828	239.6398025	273.3599026	296.6149422	253.747565
ENSG00000104951	IL4I1	7.031153671	17.51602035	7.328098975	1.674467306	0	0.379381753	1.021394535	1.661312636	1.875849726	0	0	2.53747565
ENSG00000104953	TLE6	19.68723028	24.52242849	12.21349829	24.27977593	26.58314581	15.55465189	77.62598465	68.11381808	66.59266527	62.64497769	44.14734023	77.81591994
ENSG00000104957	CCDC130	202.4972257	136.6249587	205.1867713	154.0509921	160.6546638	156.3052824	195.0863562	197.6962037	206.3434698	191.0988208	151.7564821	184.3898973
ENSG00000104960	PTOV1	473.8997574	469.4293454	460.8560022	541.6901734	548.9997504	559.9674679	437.1568609	423.6347222	506.0104635	408.7742988	547.7029398	485.5036744
ENSG00000104964	AES	1619.977806	1860.201361	1619.509873	1535.486519	1610.014005	1586.574492	1171.539532	1388.857364	1339.356704	1207.972347	1244.403153	1305.108309
ENSG00000104969	SGTA	566.7109859	511.4677943	582.1767519	619.5529031	637.9954994	677.1964296	390.1727123	490.0872277	542.1205708	461.9276132	518.7312477	488.0411501
ENSG00000104973	MED25	334.6829147	381.8492437	306.1516905	349.1264332	330.5556392	375.5879358	331.9532238	337.2464651	349.3770114	353.0898742	281.439294	309.5720293
ENSG00000104976	SNAPC2	68.90530597	77.07048955	75.72368941	87.90953355	110.955739	88.01656677	86.81853546	46.51675381	83.4753128	70.23830831	70.3598235	71.89514343
ENSG00000104979	C19orf53	375.463606	462.4229373	498.3107303	458.8040418	490.0545141	525.4437283	280.8834971	265.8100218	367.6665463	335.3721028	317.3090079	431.3708606
ENSG00000104980	TIMM44	420.4629895	273.2499175	470.6268008	530.8061359	487.7429362	591.0767716	432.0498883	332.2625272	419.2524137	414.4692968	390.4280402	431.3708606
ENSG00000104983	CCDC61	73.12399818	45.54165292	65.95289078	52.74572013	62.41260321	63.3567528	65.36925023	56.48462963	60.49615366	74.66775118	42.76773585	49.05786257
ENSG00000104998	IL27RA	16.87476881	21.01922442	20.35583049	36.83828073	38.14103529	42.11137462	0	0	6.56547404	2.53111021	0	29.60388259
ENSG00000105011	ASF1B	672.1782909	931.8522827	607.4179817	700.7645674	796.3385854	634.7056733	598.5371974	661.2024292	642.4785311	690.3603096	729.8107182	684.2726004
ENSG00000105048	TNNT1	521.7116024	423.8876925	316.7367224	469.6880793	461.1597904	337.2703787	20.4278907	26.58100218	22.97915914	14.5538837	35.86971394	15.2248539
ENSG00000105053	VRK3	337.4953762	392.3588559	443.7571046	302.2413487	337.4903729	360.4126656	340.1243801	398.7150327	334.3702136	313.2248884	444.2326111	351.8632902
ENSG00000105058	FAM32A	628.5851382	585.0350798	679.070505	601.9709964	655.3323337	651.0190887	568.9167559	664.5250545	645.2923057	538.4936971	579.4338406	644.5188152
ENSG00000105063	PPP6R1	833.8948254	858.2849973	876.9291773	875.7464009	937.344837	989.0482309	558.7028106	516.6682298	571.1962415	517.6120378	598.7483019	592.0776517
ENSG00000105072	C19orf44	105.4673051	73.56728548	83.05178838	102.9797393	98.2420606	94.84543833	120.5245551	114.6305719	130.8405184	126.5555105	67.60061473	103.1906764
ENSG00000105085	MED26	125.1545353	112.1025303	109.1072514	91.25846816	120.2020506	111.9176172	102.1394535	91.37219499	113.9578708	95.54941041	96.57230676	106.5739773
ENSG00000105088	OLFM2	1.406230734	0	0	0	1.155788948	1.13814526	0	3.322625272	0	0	15.17564821	0
ENSG00000105127	AKAP8	642.6474455	399.365264	660.343141	579.3656878	545.5323836	579.3159373	605.6869592	824.0110675	601.6787996	563.8047992	676.0061473	587.0027004
ENSG00000105131	EPHX3	7.031153671	0	2.442699658	5.023401917	1.155788948	2.655672273	1.021394535	0	5.158586746	0	2.759208765	0
ENSG00000105135	ILVBL	179.997534	346.817203	197.0444391	249.4956286	380.254564	267.8435178	249.2202665	322.2946514	246.2052765	283.4843435	438.7141936	188.6190233
ENSG00000105136	ZNF419	149.0604578	94.5865099	114.8068839	100.4680383	105.1767943	97.5011106	220.6212195	206.0027669	187.1160101	203.1215943	193.1446135	195.3856251
ENSG00000105137	SYDE1	71.71776744	143.6313669	67.58135721	66.14145858	211.5093775	98.25987411	48.00554314	129.5823856	39.86180667	34.80276538	160.0341083	36.37048432
ENSG00000105141	CASP14	0	14.01281628	0	2.511700959	6.93473369	0	0	0	2.344812157	6.327775524	1.379604382	1.691650434
ENSG00000105143	SLC1A6	9.843615139	45.54165292	5.699632536	20.93084132	145.6294075	9.863925586	195.0863562	397.05372	113.019946	277.7893455	475.9635119	109.111453
ENSG00000105146	AURKC	5.624922937	3.50320407	7.328098975	1.674467306	6.93473369	3.793817533	11.23533988	1.661312636	9.379248629	3.796665314	5.518417529	10.99572782
ENSG00000105171	POP4	399.3695285	500.9581821	403.0454436	390.9881159	423.0187551	470.0539923	332.9746184	398.7150327	400.024954	391.0565274	343.5214912	439.8291127
ENSG00000105173	CCNE1	358.5888372	507.9645902	333.0213868	479.7348831	451.9134788	469.6746106	287.0118643	234.2450817	276.6878346	287.2810088	280.0596896	315.4928059
ENSG00000105176	URI1	953.4244378	767.2016914	798.7627883	997.9825142	887.6459123	930.6234409	836.5221241	840.6241939	797.2361335	846.0235875	870.5303652	944.7867671
ENSG00000105185	PDCD5	648.2723684	609.5575083	617.1887803	604.4826974	672.6691679	612.7015316	450.4349899	390.4084695	435.1971364	465.7242786	415.2609191	471.1246457
ENSG00000105186	ANKRD27	544.2112941	602.5511001	628.5880454	560.9465474	572.1155294	566.7963394	606.7083537	606.3791122	624.1889963	634.675885	681.5245649	657.2061934
ENSG00000105193	RPS16	4078.069129	3832.505253	4012.541305	4062.257684	3623.398353	3876.522755	3576.923661	4174.878655	4045.269934	3871.333065	4298.847255	4263.804918

**ENSG00000105197**	TIMM50	407.8069129	332.8043867	376.1757474	449.5944716	419.5513882	489.7818435	300.2899933	252.5195207	351.7218236	361.3159824	273.1616677	349.3258145
**ENSG00000105198**	LGALS13	0	0	5.699632536	0	0	0	5.106972674	1.661312636	1.406887294	0	1.379604382	0
**ENSG00000105202**	FBL	1002.642513	798.7305281	908.6842729	1087.566515	933.8774702	1119.555554	850.8216476	1008.41677	1050.944809	925.7535591	967.102672	1083.502103
**ENSG00000105204**	DYRK1B	52.03053716	112.1025303	52.92515926	33.48934611	85.52838217	42.49075637	80.69016826	142.8728867	66.12370283	106.3066288	148.9972733	82.04504603
**ENSG00000105219**	CNTD2	4.218692202	0	6.513865756	5.023401917	5.778944741	7.208253313	0	1.661312636	1.875849726	0	2.759208765	0.845825217
**ENSG00000105220**	GPI	1618.571575	3391.10154	1593.45441	1756.516204	3085.956492	1648.413718	1140.897695	3639.935986	1291.053574	1407.297276	3632.498339	1393.074132
**ENSG00000105221**	AKT2	773.4269038	704.1440182	686.398604	859.8389615	799.8059522	800.4954995	760.9389285	591.4272985	776.6017865	775.7852792	769.8192453	775.6217238
**ENSG00000105223**	PLD3	282.6523776	269.7467134	243.4557326	216.8435161	346.7366845	328.1652166	335.0174074	343.8917157	378.4526822	337.2704354	475.9635119	321.4135824
**ENSG00000105227**	PRX	50.62430643	17.51602035	35.82626166	70.32762684	58.94523636	38.31755708	65.36925023	66.45250545	74.09606417	77.19886139	60.70259282	76.97009473
**ENSG00000105229**	PIAS4	343.1202991	255.7338971	372.9188145	322.3349564	340.9577397	334.2353247	306.4183605	292.391024	295.4463318	294.2415619	373.8727876	319.7219319
**ENSG00000105245**	NUMBL	194.0598413	241.7210809	227.1710682	211.8201142	223.067267	210.1774913	154.2305748	169.4538889	160.854114	137.3127289	176.5893609	140.406986
**ENSG00000105246**	EBI3	16.87476881	3.50320407	10.58503185	0.837233653	4.623155793	2.27629052	7.149761744	9.967875817	9.848211061	2.53111021	8.277626294	9.304077385
**ENSG00000105248**	CCDC94	157.4978422	185.6698157	190.5305734	200.098843	137.5388848	217.0063629	155.2519693	189.3896405	174.4540245	157.5616105	113.1275593	153.0943642
**ENSG00000105254**	TBCB	296.7146849	220.7018564	279.2819943	382.6157794	382.5661419	366.1033919	325.8248566	287.407086	361.5700347	314.4904435	355.9379306	252.9017398
**ENSG00000105255**	FSD1	42.18692202	52.54806106	26.05546302	32.65211246	41.60840214	40.21446585	0	0	1.875849726	2.53111021	0	1.691650434
**ENSG00000105258**	POLR2I	149.0604578	108.5993262	150.6331456	146.5158893	169.9009754	169.9630255	112.3533988	127.921073	134.1232554	155.6632779	133.8216251	137.8695103
**ENSG00000105261**	OVOL3	12.65607661	0	2.442699658	0	0	0	1.021394535	0	0.937924863	0	0	0
**ENSG00000105270**	CLIP3	7.031153671	10.50961221	8.956565414	8.372336529	11.55788948	0.379381753	10.21394535	14.95181373	7.972361335	15.81943881	20.69406573	10.99572782
**ENSG00000105281**	SLC1A5	5165.085487	3170.399684	5283.559361	4576.319147	4091.492877	5694.899499	3682.127298	2259.385185	4237.544531	2800.040669	1996.287541	3203.985921
**ENSG00000105287**	PRKD2	565.3047551	521.9774065	532.5085255	277.1243391	368.6966745	364.9652467	407.5364194	335.5851525	517.2655619	256.9076863	224.8755143	335.7926111
**ENSG00000105289**	TJP3	28.12461468	42.03844885	51.29669283	56.09465474	56.63365846	52.35468196	27.57765244	19.93575163	27.19982102	33.53721028	38.6289227	27.91223215
**ENSG00000105290**	APLP1	1.406230734	7.006408141	2.442699658	14.2329721	30.05051265	17.83094241	4.08557814	6.645250545	13.59991051	26.5766572	24.83287888	17.76232955
**ENSG00000105298**	CACTIN	296.7146849	210.1922442	256.4834641	231.9137218	198.7956991	244.7012309	211.4286687	222.6158932	246.6742389	221.4721433	223.4959099	252.0559146
**ENSG00000105321**	CCDC9	300.9333771	311.7851623	319.179422	205.9594786	226.5346339	276.9486799	187.9365944	201.018829	241.0466898	162.623831	189.0058004	223.2978572
**ENSG00000105323**	HNRNPUL1	2785.743084	2448.739645	2744.780183	3307.910162	3283.596402	3242.575845	2797.599631	2678.035969	2960.090867	3021.512813	3331.744583	3268.268638
**ENSG00000105325**	FZR1	329.0579918	343.3139989	356.6341501	449.5944716	394.1240314	505.7158772	422.8573374	392.0697821	483.5002668	470.1537214	451.130633	433.062511
**ENSG00000105327**	BBC3	42.18692202	63.05767327	65.13865756	36.00104707	63.56839215	35.66188481	45.96275407	63.12988017	58.62030393	37.33387559	60.70259282	48.21203736
**ENSG00000105329**	TGFB1	26.71838395	56.05126513	29.3123959	32.65211246	72.81470374	70.18562436	145.038024	318.9720261	139.7508046	229.6982515	368.3543701	171.702519
**ENSG00000105339**	DENND3	697.4904441	749.6856711	802.0197212	354.9870688	324.7766945	414.2848746	428.9857046	355.5209041	404.7145783	329.0443272	291.0965247	355.246591
**ENSG00000105341**	DMAC2	247.4966092	224.2050605	222.2856689	210.9828805	251.9619907	289.088896	156.2733638	172.7765142	195.5573339	177.8104922	238.6715581	170.0108686
**ENSG00000105355**	PLIN3	1501.854424	1422.300853	1858.89444	1573.162034	1764.889724	1975.061408	397.3224741	521.6521677	542.1205708	496.7303786	579.4338406	565.0112448
**ENSG00000105357**	MYH14	3352.45407	3152.883663	3730.002378	2993.110309	3259.324834	3960.366123	664.9278422	677.8155555	972.6280828	448.6392846	423.5385454	729.1013369
**ENSG00000105364**	MRPL4	298.1209156	248.727489	272.7681285	305.5902833	203.4188549	356.2394664	288.0332588	169.4538889	302.4807683	325.8804395	215.2182836	301.1137772
**ENSG00000105371**	ICAM4	8.437384405	35.0320407	21.98429693	0	2.311577897	0	4.08557814	0	0.937924863	0	0	0
**ENSG00000105372**	RPS19	2732.306316	2315.617891	2847.373568	2489.932884	2460.674671	2876.093072	2359.421376	2761.101601	3014.490509	2624.761287	2368.780724	2563.696232
**ENSG00000105373**	NOP53	857.8007478	774.2080996	912.755439	757.6964558	780.1575401	817.5676784	926.4048431	1137.999156	1019.993288	841.5941447	911.9184967	869.5083228
**ENSG00000105376**	ICAM5	47.81184496	42.03844885	27.68392946	38.51274803	65.87997005	37.93817533	14.29952349	0	16.88264753	27.20943475	15.17564821	15.2248539
**ENSG00000105379**	ETFB	133.5919197	213.6954483	144.9335131	200.098843	205.7304328	182.4826233	203.2575124	121.2758224	221.8192301	255.6421312	215.2182836	232.6019346
**ENSG00000105383**	CD33	22.49969175	80.57369362	80.60908873	15.9074394	43.91998003	45.90519215	4.08557814	11.62918845	2.813774589	1.898332657	0	10.99572782
**ENSG00000105388**	CEACAM5	0	0	5.699632536	0	0	0	0	0	0	1.265555105	0	1.691650434
**ENSG00000105393**	BABAM1	187.0286876	105.0961221	205.1867713	220.1924507	198.7956991	179.4475693	134.8240786	136.2276362	166.9506256	178.4432698	164.1729215	176.7774703
**ENSG00000105397**	TYK2	701.7091363	823.2529565	856.5733469	496.4795562	536.286072	544.7921977	553.5958379	576.4754847	547.7481199	515.7137052	529.7680828	509.1867805
**ENSG00000105398**	SULT2A1	1.406230734	0	0.814233219	0	0	0	38.81299233	6.645250545	19.22745969	67.07442055	15.17564821	27.91223215
**ENSG00000105401**	CDC37	1281.076199	938.8586909	1320.686282	1208.128161	1181.216305	1300.141269	831.4151514	835.640256	966.0626088	934.6124449	858.1139258	839.9044403
**ENSG00000105402**	NAPA	262.9651473	157.6441832	260.5546302	217.6807497	195.3283323	238.2517411	199.1719343	264.1487091	258.3982997	194.2627086	187.626196	203.8438772
**ENSG00000105404**	RABAC1	12.65607661	28.02563256	28.49816268	20.09360767	46.23155793	18.96908767	24.51346884	29.90362745	17.8205724	24.67832454	23.4532745	30.4497078
**ENSG00000105419**	MEIS3	2.812461468	7.006408141	2.442699658	1.674467306	4.623155793	4.55258104	2.04278907	0	0.937924863	1.265555105	2.759208765	1.691650434
**ENSG00000105426**	PTPRS	853.5820556	620.0671205	718.9679328	1616.698184	1559.159291	1582.780675	293.1402315	250.8582081	362.9769219	444.8426193	456.6490505	533.7157118
**ENSG00000105427**	CNFN	7.031153671	0	8.956565414	5.023401917	0	2.27629052	0	0	4.220661883	0	0	0.845825217
**ENSG00000105429**	MEGF8	158.904073	185.6698157	225.5426018	214.3318151	233.4693675	294.4002406	113.3747934	164.469951	155.2265648	168.9516065	155.8952952	131.1029086
**ENSG00000105438**	KDELR1	1081.391435	1043.954813	1063.388585	1093.427151	1280.614155	1202.260776	893.720218	1021.707271	1096.903127	1068.761286	1080.230231	1224.754914
**ENSG00000105443**	CYTH2	580.7732932	795.227324	729.5529646	646.34438	670.35759	621.0479302	342.1671692	392.0697821	332.9633263	342.3326558	430.4365673	341.7133876
**ENSG00000105447**	GRWD1	544.2112941	185.6698157	465.7414015	552.5742109	351.3598403	572.4870657	365.6592435	234.2450817	427.2247751	375.2370886	263.504437	427.9875597
**ENSG00000105464**	GRIN2D	291.089762	374.8428355	255.6692309	274.6126381	231.1577897	278.0868252	195.0863562	219.293268	278.0947219	253.111021	288.3373159	269.8182442
**ENSG00000105467**	SYNGR4	8.437384405	0	0	1.674467306	5.778944741	3.035054026	0	1.661312636	2.344812157	0.632777552	0	0.845825217
**ENSG00000105472**	CLEC11A	0	14.01281628	4.885399317	1.674467306	3.467366845	4.173199286	0	4.983937908	2.813774589	2.53111021	2.759208765	5.074951301
**ENSG00000105479**	CCDC114	7.031153671	17.51602035	8.142332194	14.2329721	13.86946738	10.62268909	15.32091802	4.983937908	6.096511609	12.0227735	6.898021912	9.304077385
**ENSG00000105483**	CARD8	163.1227652	129.6185506	152.261612	98.79357104	123.6694175	87.63718501	84.77574639	114.6305719	79.25465092	78.4644165	100.7111199	69.35766778
**ENSG00000105486**	LIG1	509.0555258	760.1952833	504.8245961	508.2008273	595.2313084	494.3344246	586.280463	764.2038126	658.8922162	658.0886545	898.1224529	595.4609526
**ENSG00000105492**	SIGLEC6	18.28099954	10.50961221	13.84196473	10.88403749	13.86946738	13.27836137	9307.968396	19350.96959	7751.480029	7888.837746	17137.44564	6736.152026
**ENSG00000105497**	ZNF175	130.7794583	157.6441832	170.9889761	109.6776085	180.3030759	139.2331035	694.5482837	1287.517293	578.6996404	596.0764543	1372.70636	636.9063882
**ENSG00000105499**	PLA2G4C	84.37384405	150.637775	177.5028418	32.65211246	55.47786952	75.11758715	28.59904698	41.5328159	22.51019671	3.163887762	16.55525259	5.920776517
**ENSG00000105501**	SIGLEC5	0	0	0	0	0	0	1.021394535	4.983937908	1.875849726	0	8.277626294	1.691650434
**ENSG00000105514**	RAB3D	232.0280711	325.7979785	194.6017394	313.9626198	409.1492877	267.4641361	56.17669942	49.83937908	52.52379232	68.33997566	59.32298844	60.05359039
**ENSG00000105516**	DBP	130.7794583	203.1858361	119.6922833	155.7254594	206.8862217	136.5774312	117.4603715	104.6626961	122.868157	136.6799513	117.2663725	130.2570834
**ENSG00000105518**	TMEM205	118.1233817	119.1089384	143.3050466	149.8648239	142.1620406	142.2681575	111.3320043	112.9692593	130.8405184	146.1716146	122.78479	130.2570834
**ENSG00000105519**	CAPS	16.87476881	3.50320407	14.65619795	26.79147689	24.27156791	17.83094241	17.36370709	14.95181373	37.04803208	29.74054496	33.11050518	42.29126084

**ENSG00000105520**	PLPPR2	286.8710698	252.2306931	210.8864038	489.7816869	470.4061019	464.363266	74.56180105	73.09775599	103.6406974	124.0244003	103.4703287	163.2442668
**ENSG00000105523**	FAM83E	11.24984587	94.5865099	43.96859385	14.2329721	42.76419109	22.38352344	3.064183605	3.322625272	7.972361335	8.226108181	2.759208765	9.304077385
**ENSG00000105538**	RASIP1	0	14.01281628	5.699632536	10.04680383	6.93473369	2.27629052	1.021394535	4.983937908	0.468962431	0	1.379604382	1.691650434
**ENSG00000105550**	FGF21	0	0	0.814233219	0	0	0	7.149761744	0	0.937924863	0	0	0
**ENSG00000105552**	BCAT2	88.59253625	94.5865099	120.5065165	164.097796	129.4483622	132.7836137	225.7281922	132.9050109	279.0326467	240.4554699	169.691339	298.5763015
**ENSG00000105556**	MIER2	219.3719945	192.6762239	210.0721706	161.586095	179.147287	199.1754205	230.8351649	162.8086383	192.7435593	201.2232617	193.1446135	177.6232955
**ENSG00000105559**	PLEKHA4	108.2797665	108.5993262	84.68025482	41.02444899	80.90522638	33.76497604	56.17669942	61.46856754	52.52379232	40.49776335	41.38813147	38.90795997
**ENSG00000105568**	PPP2R1A	1490.604578	1544.912995	1455.848996	1625.907754	1489.811954	1715.184907	1519.835068	1744.378268	1812.070835	1709.764947	1793.485697	1728.020918
**ENSG00000105576**	TNPO2	824.0512102	493.9517739	750.7230283	905.8868124	782.469118	1015.604954	750.7249831	669.5089924	895.2492816	825.7747059	649.7936641	933.7910393
**ENSG00000105607**	GCDH	95.62368992	66.56087734	100.150686	131.4456835	92.46311586	134.3011407	120.5245551	144.5341993	120.5233449	113.2671819	106.2295374	172.5483442
**ENSG00000105612**	DNASE2	542.8050634	630.5767327	624.5168793	469.6880793	611.4123536	531.1344546	654.7138969	641.2666775	698.2850604	525.2053685	546.3233354	572.6236718
**ENSG00000105613**	MAST1	32.34330689	38.53524477	24.42699658	15.9074394	53.16629162	22.00414169	3.064183605	11.62918845	4.220661883	12.65555105	8.277626294	3.383300867
**ENSG00000105617**	LENG1	84.37384405	108.5993262	114.8068839	74.51379511	68.19154795	80.4289317	74.56180105	44.85544118	62.37200338	56.94997971	60.70259282	65.97436691
**ENSG00000105618**	PRPF31	399.3695285	294.2691419	359.891083	482.2465841	421.8629661	467.7777018	370.7662162	357.1822168	404.7145783	369.5420906	378.0116008	429.6792101
**ENSG00000105619**	TFPT	106.8735358	59.5544692	78.16638907	62.79252397	91.30732691	94.84543833	43.919965	81.40431917	72.22021444	48.72387153	49.66575776	52.44116344
**ENSG00000105639**	JAK3	150.4666886	182.1666117	121.3207497	151.5392912	156.031508	127.0928874	126.6529223	78.0816939	95.66833602	121.4932901	108.9887462	90.5032982
**ENSG00000105640**	RPL18A	4865.55834	3765.944376	4705.453775	4049.699179	3907.722434	4638.321316	3685.191482	3709.711116	4569.569932	3706.810902	3404.863616	3936.470559
**ENSG00000105641**	SLC5A5	0	3.50320407	0	0	4.623155793	1.517527013	0	0	0	1.265555105	0	0
**ENSG00000105642**	KCNN1	0	0	4.071166097	5.86063557	4.623155793	1.896908767	0	1.661312636	2.813774589	0	11.03683506	0
**ENSG00000105643**	ARRDC2	177.1850725	248.727489	220.6572025	164.097796	169.9009754	137.3361947	73.54040651	94.69482026	109.2682465	88.58885733	80.01705417	95.5782495
**ENSG00000105647**	PIK3R2	0	3.50320407	4.885399317	3.348934611	1.155788948	2.27629052	4.08557814	3.322625272	7.972361335	4.429442867	9.657230676	5.920776517
**ENSG00000105649**	RAB3A	11.24984587	21.01922442	29.3123959	25.11700959	38.14103529	22.00414169	11.23533988	19.93575163	12.66198565	6.960553076	26.21248326	13.53320347
**ENSG00000105655**	ISYNA1	142.0293041	140.1281628	82.23755516	132.2829172	158.3430859	105.8475092	292.118837	332.2625272	415.5007143	366.3782028	350.4195131	565.85707
**ENSG00000105656**	ELL	316.4019152	472.9325495	409.5593094	318.9860217	317.8419608	389.6250606	307.439755	250.8582081	290.7567075	225.2688086	264.8840414	263.0516424
**ENSG00000105662**	CRTC1	92.81122845	38.53524477	78.98062229	140.6552537	119.0462617	106.9856544	102.1394535	41.5328159	65.18577797	83.52663691	104.8499331	73.58679386
**ENSG00000105669**	COPE	323.4330689	346.817203	393.274645	267.0775353	227.6904228	359.6539021	256.3700283	279.1005229	323.1151153	301.2021149	246.9491844	255.4392155
**ENSG00000105671**	DDX49	343.1202991	262.7403053	334.6498532	354.9870688	373.3198303	381.2786621	345.2313528	307.3428377	401.9008038	360.0504273	245.5695801	394.154551
**ENSG00000105672**	ETV2	0	10.50961221	3.256932878	1.674467306	3.467366845	4.173199286	1.021394535	0	2.344812157	2.53111021	1.379604382	2.53747565
**ENSG00000105676**	ARMC6	284.0586083	108.5993262	324.0648213	376.7551438	242.7156791	365.7240102	167.5087037	132.9050109	234.0122533	230.9638066	162.7933171	213.9937798
**ENSG00000105677**	TMEM147	379.6822982	266.2435094	330.5786871	323.17219	338.6461618	341.8229597	207.3430906	174.4378268	302.0118059	252.4782434	224.8755143	275.7390207
**ENSG00000105694**	ELOCP28	0	0	1.628466439	0	0	0.379381753	0	0	0.468962431	1.898332657	1.379604382	3.383300867
**ENSG00000105695**	MAG	11.24984587	10.50961221	27.68392946	15.07020575	23.11577897	22.00414169	57.19809395	6.645250545	25.3239713	74.03497363	11.03683506	27.06640694
**ENSG00000105697**	HAMP	5.624922937	0	0	0	1.155788948	1.517527013	2.04278907	4.983937908	0.937924863	3.163887762	1.379604382	0.845825217
**ENSG00000105698**	USF2	447.1813735	374.8428355	429.9151399	581.0401551	651.8649668	605.1138965	428.9857046	378.779281	417.8455264	434.7181785	529.7680828	446.5957145
**ENSG00000105699**	LSR	3414.328223	4610.216557	3895.291722	2385.278677	2719.571395	2952.348804	945.8113393	948.6095152	1035.000086	767.559171	714.63507	803.5339559
**ENSG00000105700**	KXD1	760.7708272	823.2529565	780.8496574	819.6517462	759.353339	818.3264419	598.5371974	662.8637418	724.0779942	646.6986585	760.1620147	623.3731848
**ENSG00000105701**	FKBP8	814.2075951	746.182467	743.3949294	937.7016912	976.6416613	972.7348155	642.4571624	822.3497549	852.104738	797.9324936	905.0204748	835.6753142
**ENSG00000105705**	SUGP1	306.5583	301.2755501	329.7644539	293.0317785	330.5556392	307.2992202	322.760673	292.391024	335.3081385	319.552664	347.6603043	329.8718345
**ENSG00000105708**	ZNF14	91.40499772	126.1153465	91.19412058	96.28187008	149.0967743	107.7444179	207.3430906	282.4231481	170.2333626	160.0927208	242.8103713	241.906012
**ENSG00000105717**	PBX4	39.37446056	56.05126513	56.18209214	56.09465474	57.78944741	64.87427982	50.04833221	44.85544118	57.68237907	47.45831643	37.24931832	56.67028952
**ENSG00000105722**	ERF	497.8056799	357.3268152	368.8476484	552.5742109	589.4523636	484.470499	450.4349899	312.3267756	573.5410537	396.1187478	321.4478211	518.4908579
**ENSG00000105723**	GSK3A	275.6212239	308.2819582	298.8235915	329.8700592	293.5703929	330.0621254	207.3430906	167.7925762	234.0122533	234.7604719	201.4222398	236.8310607
**ENSG00000105726**	ATP13A1	365.6199909	350.320407	328.9502207	375.0806765	306.2840713	384.6930979	400.3866577	383.7632189	410.81109	455.5998377	502.1759952	416.9918319
**ENSG00000105732**	ZNF574	64.68661377	115.6057343	78.98062229	102.1425056	122.5136285	131.6454684	92.94690267	69.77513072	82.53738794	108.2049615	107.6091418	100.6532008
**ENSG00000105737**	GRIK5	5.624922937	3.50320407	10.58503185	7.535102876	1.155788948	5.6907263	2.04278907	0	1.875849726	0.632777552	0	0
**ENSG00000105738**	SIPA1L3	299.5271464	231.2114686	318.3651888	414.4306582	401.058765	506.0952589	275.7765244	206.0027669	274.3430224	285.3826761	365.5951613	311.2636798
**ENSG00000105750**	ZNF85	5.624922937	0	19.54159727	20.93084132	13.86946738	20.10723293	177.7226491	156.1633878	186.6470477	162.623831	164.1729215	224.1436824
**ENSG00000105755**	ETHE1	592.0231391	353.8236111	537.3939248	503.1774254	392.9682424	613.0809133	220.6212195	176.0991394	269.1844357	215.1443678	122.78479	201.3064016
**ENSG00000105767**	CADM4	68.90530597	59.5544692	71.65252331	104.6542066	93.61890481	138.8537217	38.81299233	38.21019063	40.79973154	41.1305409	60.70259282	30.4497078
**ENSG00000105771**	SMG9	388.1196826	399.365264	417.7016416	662.2518194	558.246062	542.895289	412.6433921	461.8449128	532.7413221	497.3631562	561.4989836	540.4823135
**ENSG00000105778**	AVL9	1390.762196	1541.409791	1399.666904	1058.263337	1097.999501	1092.61945	1375.818438	1536.714188	1224.929871	1256.696219	1353.391899	1158.780547
**ENSG00000105784**	RUNDC3B	0	3.50320407	3.256932878	0	2.311577897	2.655672273	0	0	0	0	0	0
**ENSG00000105792**	CFAP69	21.09346101	14.01281628	13.02773151	38.51274803	28.89472371	17.0721789	124.6101333	109.646634	88.63389954	137.3127289	96.57230676	78.66174516
**ENSG00000105793**	GTPBP10	590.6169083	469.4293454	613.9318475	505.6891263	431.1092777	527.7200188	738.4682487	549.8944826	616.6855974	592.279789	630.4792027	596.3067778
**ENSG00000105808**	RASA4	2.812461468	38.53524477	8.142332194	5.023401917	6.93473369	6.070108053	27.57765244	28.24231481	24.85500887	16.45221636	13.79604382	10.99572782
**ENSG00000105810**	CDK6	2625.432781	2319.121095	2265.196817	3276.932517	2941.482873	3331.351176	652.6711078	1096.46634	838.5048274	579.624238	967.102672	780.6966751
**ENSG00000105819**	PMPCB	689.0530597	844.272181	622.0741797	739.2773155	678.4481126	670.3675581	830.3937569	782.4782516	776.6017865	789.7063854	858.1139258	792.5382281
**ENSG00000105821**	DNAJC2	860.6132093	630.5767327	782.4781239	786.1624	685.3828463	746.2439087	692.5054946	622.9922385	710.0091212	742.8808465	696.7002131	726.5638612
**ENSG00000105825**	TFPI2	0	0	1.628466439	2.511700959	6.93473369	2.27629052	10.21394535	3.322625272	9.379248629	6.327775524	6.898021912	10.1499026
**ENSG00000105829**	BET1	329.0579918	532.4870187	398.9742775	237.7743574	266.987247	311.0930377	336.038802	345.5530283	331.0874766	288.5465639	291.0965247	357.7840667
**ENSG00000105835**	NAMPT	5496.95594	8130.936647	6710.910195	3435.169678	4545.717934	4100.35799	1800.718565	2606.599526	1842.553393	1340.855633	1959.038223	1470.044227
**ENSG00000105849**	TWISTNB	919.6749001	690.1312019	817.4901523	1057.426104	804.429108	997.3946294	648.5855296	516.6682298	683.7472251	706.812526	565.6377967	725.718036
**ENSG00000105851**	PIK3CG	16.87476881	143.6313669	135.1627144	23.44254228	99.39784955	133.9217589	8.171156279	0	0.468962431	1.898332657	8.277626294	0
**ENSG00000105852**	PON3	0	0	0.814233219	0	0	4.173199286	0	0	0.937924863	0.632777552	0	0
**ENSG00000105854**	PON2	448.5876042	357.3268152	341.1637189	509.0380609	485.4313583	479.1591544	226.7495867	189.3896405	182.4263858	269.5632373	237.2919538	262.2058172
**ENSG00000105855**	ITGB8	2683.088241	3993.65264	2608.803235	2406.209518	2288.462118	2655.672273	1250.186911	817.365817	1197.73005	1130.773486	776.7172672	1343.170444
**ENSG00000105856**	HBP1	1140.453125	1401.281628	1179.009702	884.9559711	1321.066768	967.0440892	1158.261403	1737.733017	1199.136937	1239.611225	1449.964206	1032.75259

**ENSG00000105865**	DUS4L	130.7794583	63.05767327	106.6645517	66.14145858	95.93048271	102.0536916	147.080813	142.8728867	114.4268333	134.1488411	110.3683506	126.0279573
**ENSG00000105866**	SP4	608.8979079	655.0991612	591.9475505	696.5783992	595.2313084	661.262396	379.958767	486.7646024	401.4318413	352.4570967	471.8246987	391.6170754
**ENSG00000105875**	WDR91	106.8735358	98.08971397	116.4353504	162.4233287	187.2378096	164.2722992	303.3541769	220.9545806	252.7707506	268.2976822	240.0511625	269.8182442
**ENSG00000105877**	DNAH11	271.4025317	437.9005088	641.6157769	204.2850113	262.3640913	426.4250907	117.4603715	91.37219499	85.35116252	75.30052873	70.3598235	38.06213475
**ENSG00000105879**	CBLL1	728.4275203	620.0671205	661.9716074	664.7635204	607.9449868	677.1964296	737.4468542	898.7701361	822.0911423	758.7002853	779.476476	835.6753142
**ENSG00000105880**	DLX5	0	0	0	0	0	0	2.04278907	3.322625272	3.751699452	3.163887762	2.759208765	3.383300867
**ENSG00000105887**	MTPN	4099.16259	3825.498845	3903.434054	4156.865086	4074.156043	4460.011892	3620.843626	3387.416465	3914.898378	3632.775928	3515.231966	4087.027447
**ENSG00000105889**	STEAP1B	4.218692202	35.0320407	7.328098975	0	4.623155793	17.45156065	5.106972674	4.983937908	1.406887294	3.163887762	0	0.845825217
**ENSG00000105894**	PTN	7.031153671	24.52242849	43.96859385	4.186168264	5.778944741	6.070108053	220.6212195	199.3575163	143.0335416	168.3188289	187.626196	136.1778599
**ENSG00000105926**	MPP6	569.5234473	423.8876925	499.1249635	652.2050156	639.1512884	619.9097849	625.0934553	631.2988017	643.8854184	758.0675078	670.4877298	775.6217238
**ENSG00000105928**	GSDME	7.031153671	21.01922442	11.39926507	1.674467306	6.93473369	6.828871559	618.9650881	691.1060566	725.015919	675.1736484	554.6009617	724.8722108
**ENSG00000105929**	ATP6V0A4	23.90592248	24.52242849	57.81055858	56.09465474	49.69892478	57.28664475	2.04278907	0	7.503398903	1.898332657	0	0
**ENSG00000105939**	ZC3HAV1	5044.149643	5342.386207	5202.950272	2446.396734	2785.451365	2320.678185	3151.00214	3869.19713	3135.013854	2293.18585	2149.423628	2238.053524
**ENSG00000105948**	TTC26	310.7769922	276.7531216	265.4400295	379.2668447	345.5808955	278.4662069	420.8145484	370.4727179	438.010911	441.045954	423.5385454	446.5957145
**ENSG00000105953**	OGDH	4384.627429	3681.867478	4476.654241	3657.873829	3280.129035	4141.331219	1872.216182	1812.492086	2083.131121	1812.907688	1707.950225	2076.500907
**ENSG00000105963**	ADAP1	94.21745919	45.54165292	49.66822639	138.1435527	122.5136285	126.7135056	26.55625791	21.59706427	28.13774589	31.00610007	35.86971394	56.67028952
**ENSG00000105967**	TFEC	0	0	2.442699658	0.837233653	0	0.758763507	3.064183605	3.322625272	1.406887294	2.53111021	2.759208765	1.691650434
**ENSG00000105968**	H2AFV	3342.610455	3100.335602	3011.034446	5536.626146	5113.210307	4980.523657	4494.135953	4527.076933	4865.954189	5105.249293	4897.595557	5567.221577
**ENSG00000105971**	CAV2	3602.763141	3086.322786	3097.343167	3703.92168	3188.821708	3547.598775	1838.510163	2211.207119	2045.145164	1518.033348	1856.947499	1746.629073
**ENSG00000105974**	CAV1	17518.82249	12769.17884	13163.70846	16559.64442	16472.30409	15666.94888	5823.991638	8328.160245	6291.59998	4452.855636	5907.465965	4948.923343
**ENSG00000105976**	MET	16780.55135	17113.15188	18313.73357	15883.15963	16140.59266	17429.55651	8896.346399	9261.817946	8085.850243	7991.347709	8709.442466	8304.311978
**ENSG00000105983**	LMBR1	1188.26497	1131.534915	1043.032754	1201.430292	1317.599401	1166.21951	1253.251094	1249.307102	1256.350354	1309.849533	1201.635417	1313.566562
**ENSG00000105989**	WNT2	0	0	0	0	0	0	1.021394535	1.661312636	7.034436472	2.53111021	0	4.229126084
**ENSG00000105991**	HOXA1	63.28038304	66.56087734	131.9057816	87.90953355	80.90522638	139.6124852	13.27812895	31.56494009	29.54463318	32.90443272	16.55525259	54.97863909
**ENSG00000105993**	DNAJB6	2112.158563	1685.041158	1985.100589	2037.826711	1829.613905	2033.106816	1504.51415	1614.795882	1722.030048	1535.118342	1677.598929	1666.275677
**ENSG00000105997**	HOXA3	0	7.006408141	0.814233219	0	1.155788948	1.517527013	1.021394535	1.661312636	3.28273702	1.265555105	2.759208765	1.691650434
**ENSG00000106003**	LFNG	14.06230734	14.01281628	5.699632536	17.58190671	16.18104528	13.27836137	68.43343384	106.3240087	114.4268333	105.0410737	121.4051856	132.794559
**ENSG00000106004**	HOXA5	2.812461468	3.50320407	0	1.674467306	5.778944741	3.035054026	15.32091802	8.306563181	7.972361335	14.5538837	28.97169203	5.920776517
**ENSG00000106006**	HOXA6	5.624922937	0	0	4.186168264	8.090522638	3.793817533	3.064183605	13.29050109	7.503398903	5.062220419	2.759208765	3.383300867
**ENSG00000106009**	BRAT1	222.184456	101.592918	149.0046792	264.5658343	202.2630659	189.3114949	208.3644851	186.0670152	248.5500887	213.2460352	190.3854048	255.4392155
**ENSG00000106012**	IQCE	379.6822982	360.8300193	330.5786871	470.5253129	540.9092278	405.1797125	606.7083537	579.79811	628.8786206	663.1508749	662.2101035	607.3025056
**ENSG00000106013**	ANKRD7	0	0	3.256932878	0.837233653	0	1.896908767	0	0	0	0.632777552	0	0
**ENSG00000106018**	VIPR2	0	0	2.442699658	0	0	0	5.106972674	0	2.344812157	0	0	0
**ENSG00000106025**	TSPAN12	7.031153671	28.02563256	21.98429693	29.30317785	41.60840214	42.11137462	339.1029856	179.4217647	264.0258489	357.5193171	213.8386793	337.4842615
**ENSG00000106028**	SSBP1	1688.883112	1590.454648	1791.313083	1954.103346	1646.999251	1703.424072	1478.979287	1473.584308	1426.114754	1495.253356	1281.652471	1711.104414
**ENSG00000106031**	HOXA13	0	0	1.628466439	0	0	1.896908767	0	0	0.937924863	1.265555105	4.138813147	0
**ENSG00000106034**	CPED1	37.96822982	73.56728548	65.13865756	62.79252397	72.81470374	80.80831345	14.29952349	4.983937908	15.94472267	13.92110615	8.277626294	26.22058172
**ENSG00000106049**	HIBADH	1461.073733	1229.624629	1244.962593	1207.290927	1091.064767	1070.235926	854.9072257	737.6228104	896.1872065	838.4302569	746.3659708	934.6368645
**ENSG00000106052**	TAX1BP1	10093.92421	10569.16668	10164.07328	7921.06759	8329.77095	8022.406555	6716.690461	7583.892184	6566.880928	5835.474588	6449.650487	6609.278244
**ENSG00000106066**	CPVL	4.218692202	21.01922442	3.256932878	5.023401917	12.71367843	3.035054026	214.4928523	295.7136492	162.7299637	249.9471332	263.504437	251.2100894
**ENSG00000106069**	CHN2	5.624922937	0	1.628466439	8.372336529	8.090522638	10.24330734	30.64183605	49.83937908	34.70321993	55.05164706	56.56377967	59.20776517
**ENSG00000106070**	GRB10	11.24984587	14.01281628	8.956565414	6.697869223	1.155788948	3.41443578	88.86132453	44.85544118	91.91663656	70.87108587	73.11903226	84.58252168
**ENSG00000106077**	ABHD11	464.0561423	395.86206	333.0213868	313.9626198	292.4146039	370.655973	161.3803365	137.8889488	205.405545	186.0366004	147.6176689	170.0108686
**ENSG00000106078**	COBL	5.624922937	3.50320407	0	1.674467306	0	1.13814526	39.83438686	11.62918845	36.57906965	51.8877593	26.21248326	28.75805737
**ENSG00000106080**	FKBP14	565.3047551	448.410121	508.0815289	723.3698761	646.0860221	709.0644969	419.7931538	244.2129575	401.4318413	337.903213	275.9208765	560.7821187
**ENSG00000106086**	PLEKHA8	639.834984	949.3683031	708.3829009	880.7698028	848.349088	825.5346952	1161.325586	1588.21488	1282.143288	1302.256203	1543.777304	1442.131995
**ENSG00000106089**	STX1A	39.37446056	52.54806106	69.20982365	61.95529031	43.91998003	59.94231702	13.27812895	29.90362745	31.42048291	28.47498986	35.86971394	43.98291127
**ENSG00000106100**	NOD1	338.9016069	322.2947745	300.452058	138.9807864	144.4736185	158.2021911	287.0118643	323.955964	306.2324677	130.9849533	204.1814486	164.0900921
**ENSG00000106105**	GARS	5370.395174	5016.588229	5790.826657	3793.505681	4040.638163	5035.913393	4647.345134	3777.824935	4316.330219	3409.405452	2859.919885	3388.375818
**ENSG00000106123**	EPHB6	71.71776744	77.07048955	121.3207497	57.76912205	94.77469376	148.3382655	4.08557814	6.645250545	20.63434698	4.429442867	4.138813147	21.99145564
**ENSG00000106125**	MINDY4	44.99938349	56.05126513	36.64049488	46.88508456	48.54313583	33.00621254	43.919965	31.56494009	45.48935585	69.60553076	44.14734023	32.98718345
**ENSG00000106133**	NSUN5P2	60.46792157	52.54806106	73.28098975	82.88613163	92.46311586	69.80624261	74.56180105	78.0816939	84.41323766	100.6116308	103.4703287	79.50757038
**ENSG00000106144**	CASP2	1272.638814	1369.752792	1307.65855	1489.438668	1325.689924	1501.972361	1587.247107	1432.051492	1709.837025	1549.672226	1520.324029	1644.284221
**ENSG00000106153**	CHCHD2	1660.758497	1324.211139	1535.643852	2017.733103	1696.698176	2049.799613	1661.808908	1550.00469	1945.256166	1830.625459	1637.590402	2189.841486
**ENSG00000106211**	HSPB1	656.7097529	795.227324	804.4624208	830.5357836	935.0332591	1161.666929	229.8137703	139.5502614	370.4803208	322.7165517	251.0879976	402.6128032
**ENSG00000106244**	PDAP1	1500.448193	1257.650261	1455.034763	1716.328988	1610.014005	1680.281785	1324.748712	1297.485169	1392.818421	1297.193982	1312.003768	1557.164224
**ENSG00000106245**	BUD31	606.0854464	686.6279978	746.6518622	644.6699127	613.7239315	730.6892569	588.3232521	634.621427	613.8718228	499.8942664	567.0174011	592.0776517
**ENSG00000106246**	PTCD1	39.37446056	31.52883663	47.22552673	51.07125282	38.14103529	67.52995209	50.04833221	46.51675381	47.36520558	45.55998377	51.04536215	44.82873649
**ENSG00000106258**	CYP3A5	0	0	5.699632536	7.535102876	5.778944741	6.449489806	40.8557814	6.645250545	22.51019671	34.16998783	17.93485697	34.67883389
**ENSG00000106261**	ZKSCAN1	1933.567259	2340.140319	1958.230893	1622.558819	1908.207554	1648.034336	2034.617913	1988.591225	1876.787651	1879.982108	2200.46899	1975.001881
**ENSG00000106263**	EIF3B	3140.113229	2522.306931	3029.76181	3528.102613	3228.118533	3542.287431	2698.524361	2430.500387	2711.071816	2924.697847	2692.987754	3054.274858
**ENSG00000106266**	SNX8	437.3377583	311.7851623	466.5556347	377.5923774	429.9534888	459.810685	221.6426141	225.9385185	309.9841672	213.2460352	267.6432502	246.1351381
**ENSG00000106268**	NUDT1	97.02992066	66.56087734	131.0915483	153.2137585	121.3578396	141.509394	128.6957114	107.9853213	135.5301427	100.6116308	88.29468047	125.1821321
**ENSG00000106278**	PTPRZ1	618.741523	273.2499175	583.8052183	754.3475212	243.8714681	563.7612854	59.24088302	13.29050109	77.84776362	46.19276132	33.11050518	112.4947538
**ENSG00000106290**	TAF6	445.7751427	301.2755501	474.6979669	536.6667715	516.6376599	556.9324139	496.3977439	408.6829085	493.8174403	496.7303786	549.0825442	450.8248405
**ENSG00000106299**	WASL	2223.250791	2168.48332	1998.942554	1768.237475	1842.327584	1986.44286	1733.306526	1759.330082	2019.821192	1762.918261	1674.83972	2076.500907
**ENSG00000106302**	HYAL4	0	0	4.885399317	2.511700959	1.155788948	1.517527013	23.4920743	14.95181373	32.35840777	10.12444084	2.759208765	16.91650434

**ENSG00000106305**	AIMP2	487.9620648	322.2947745	480.3975995	505.6891263	453.0692677	528.4787824	427.9643101	333.9238399	519.6103741	487.8714929	419.3997322	514.2617318
**ENSG00000106327**	TFR2	2.812461468	28.02563256	7.328098975	19.25637402	56.63365846	22.7629052	18.38510163	13.29050109	17.8205724	27.20943475	33.11050518	17.76232955
**ENSG00000106328**	FSCN3	18.28099954	0	1.628466439	0.837233653	3.467366845	4.55258104	6.128367209	14.95181373	1.875849726	15.18666126	2.759208765	5.074951301
**ENSG00000106330**	MOSPD3	39.37446056	59.5544692	47.22552673	40.18721534	53.16629162	53.87220897	63.32646116	33.22625272	75.50295146	43.02887356	74.49863664	69.35766778
**ENSG00000106333**	PCOLCE	2.812461468	17.51602035	4.071166097	15.07020575	24.27156791	13.65774312	18.38510163	8.306563181	4.689624315	8.226108181	5.518417529	6.766601734
**ENSG00000106336**	FBXO24	2.812461468	3.50320407	0	4.186168264	3.467366845	3.793817533	2.04278907	0	2.813774589	5.694997971	2.759208765	5.074951301
**ENSG00000106344**	RBM28	1663.570959	1184.082976	1566.584714	1768.237475	1531.420356	1621.098232	1916.136147	1863.992778	1894.139261	2160.302564	1990.769124	2214.370418
**ENSG00000106346**	USP42	783.2705189	648.092753	774.3357917	488.1072196	457.6924235	516.3385662	735.4040651	712.7031209	728.298656	508.1203746	449.7510286	602.2275543
**ENSG00000106348**	IMPDH1	47.81184496	77.07048955	25.2412298	86.23506625	106.3325832	60.32169878	32.68462512	33.22625272	19.69642212	63.91053279	92.43349361	32.98718345
**ENSG00000106351**	AGFG2	43.59315276	31.52883663	30.12662912	82.88613163	110.955739	112.296999	345.2313528	262.4873965	294.0394445	361.94876	249.7083932	328.1801841
**ENSG00000106355**	LSM 5.00	772.0206731	669.1119775	609.8606814	807.0932414	717.7449369	734.8624562	768.0886902	832.3176307	810.3670816	803.6274915	600.1279063	1009.069484
**ENSG00000106366**	SERPINE1	52.03053716	66.56087734	51.29669283	89.58400086	127.1367843	106.2268909	72.51901198	435.2639107	78.31672605	87.32330223	282.8188984	49.05786257
**ENSG00000106367**	AP1S1	572.3359088	525.4806106	554.4928224	564.295482	583.6734189	552.7592146	358.5094817	338.9077778	330.1495517	374.604311	412.5017103	393.3087258
**ENSG00000106392**	C1GALT1	1085.610127	1639.499505	1126.084542	1378.086593	1505.993	1435.959936	1246.101333	1393.841302	1125.978798	1239.611225	1368.567547	1369.391026
**ENSG00000106397**	PLOD3	485.1496033	493.9517739	573.2201865	499.8284908	621.8144542	716.2727502	314.5895167	372.1340305	436.6040237	448.6392846	400.0852709	403.4586284
**ENSG00000106399**	RPA3	781.8642882	802.2337321	620.4457132	719.1837078	714.27757	647.6046529	791.5807645	814.0431917	754.5605522	802.3619364	825.0034206	979.465601
**ENSG00000106400**	ZNHIT1	392.3383748	423.8876925	389.2034789	460.4785091	461.1597904	475.3653369	354.4239036	355.5209041	366.7286214	422.695405	406.9832928	373.8547458
**ENSG00000106404**	CLDN15	35.15576835	35.0320407	39.89742775	43.53614995	10.40210053	35.28250306	51.06972674	39.87150327	67.53059013	60.11386748	78.63744979	41.44543562
**ENSG00000106415**	GLCCI1	1230.451892	1236.631037	1236.006027	1208.965395	867.9975001	1366.912457	1588.268502	933.6577015	1473.948922	1362.37007	962.9638589	1410.836462
**ENSG00000106443**	PHF14	1439.980272	1296.185506	1179.009702	1230.73347	1084.130033	1140.800932	1659.766119	1579.908317	1618.389351	1745.833267	1554.814139	1632.442668
**ENSG00000106459**	NRF1	319.2143767	154.1409791	359.0768498	363.3594053	273.9219807	327.4064531	367.7020326	330.6012146	376.10787	417.6331846	393.187249	397.5378519
**ENSG00000106460**	TMEM106B	2407.467017	2389.185176	1963.116292	1409.901471	1492.123532	1496.661017	1958.013323	1628.086383	1918.056345	1724.951608	1449.964206	1739.016646
**ENSG00000106462**	EZH2	1642.477497	1888.226994	1513.659555	1702.93325	1523.329834	1518.285777	1464.679763	1674.603137	1563.989709	1482.597805	1735.542313	1554.626748
**ENSG00000106477**	CEP41	333.276684	164.6505913	287.4243265	281.3105074	270.4546139	276.5692982	321.7392785	230.9224564	368.6044711	365.7454253	223.4959099	384.0046484
**ENSG00000106479**	ZNF862	14.06230734	7.006408141	24.42699658	15.9074394	35.8294574	16.69279715	54.13391035	33.22625272	46.42728071	42.39609601	34.49010956	37.21630954
**ENSG00000106484**	MEST	11.24984587	10.50961221	21.17006371	17.58190671	2.311577897	23.5216687	38.81299233	11.62918845	36.57906965	62.01220013	12.41643944	60.89941561
**ENSG00000106524**	ANKMY2	278.4336854	290.7659378	353.3772172	248.6583949	368.6966745	287.571369	511.718662	651.2345534	481.1554547	475.8487194	620.821972	505.8034796
**ENSG00000106526**	ACTR3C	75.93645964	115.6057343	95.26528668	30.1404115	43.91998003	48.94024618	155.2519693	204.3414542	120.0543825	82.89385936	115.8867681	62.59106604
**ENSG00000106536**	POU6F2	7.031153671	7.006408141	12.21349829	5.86063557	8.090522638	4.55258104	3.064183605	3.322625272	12.66198565	7.593330629	4.138813147	1.691650434
**ENSG00000106537**	TSPAN13	1445.605195	1103.509282	1070.716684	1711.305586	1781.070769	1718.219961	2443.175727	2149.738551	2545.528078	2503.900775	2439.140548	3044.97078
**ENSG00000106538**	RARRES2	11.24984587	0	0	0	0	0	0	6.645250545	1.406887294	0.632777552	0	0
**ENSG00000106541**	AGR2	105.4673051	31.52883663	72.46675653	248.6583949	242.7156791	225.7321432	314.5895167	425.2960348	347.5011617	341.6998783	470.4450944	363.7048432
**ENSG00000106546**	AHR	4315.722123	3878.046906	3440.949585	3224.186797	3300.933236	3050.229297	2423.769231	2206.223181	3125.165643	2132.460352	1913.511278	2924.017774
**ENSG00000106554**	CHCHD3	899.9876699	791.7241199	763.7507598	894.1655413	907.2943244	843.7450193	809.9658662	840.6241939	794.8913213	840.9613671	844.317882	914.3370593
**ENSG00000106560**	GIMAP2	327.6517611	430.8941007	366.4049488	41.02444899	95.93048271	63.73613456	149.1236021	116.2918845	109.2682465	31.00610007	28.97169203	16.07067912
**ENSG00000106571**	GLI3	2200.751099	1611.473872	1574.727046	1691.211979	1336.092024	1208.710266	3258.248566	3802.744624	3056.228166	3430.919889	4280.912398	3056.812333
**ENSG00000106588**	PSMA2	240.4654555	245.2242849	288.2385597	221.866918	202.2630659	191.2084037	249.2202665	164.469951	227.4467793	184.1382677	162.7933171	147.1735877
**ENSG00000106591**	MRPL32	631.3975996	564.0158553	714.0825335	564.295482	477.3408356	558.8293226	406.5150249	402.0376579	362.0389971	339.8015456	424.9181498	387.3879493
**ENSG00000106603**	COA1	1254.357815	1040.451609	1144.811907	1283.47919	1229.759441	1269.790728	1044.886609	963.561329	1019.993288	1015.607972	1069.193396	1113.95181
**ENSG00000106605**	BLVRA	293.9022234	343.3139989	297.1951251	163.2605623	193.0167544	144.9238298	69.45482837	86.38825708	111.6130587	51.25498174	97.95191114	75.27844429
**ENSG00000106608**	URGCP	728.4275203	781.2145077	864.7156791	749.3241193	747.7954495	791.0109556	667.9920258	654.5571786	697.816098	628.3481095	595.9890932	657.2061934
**ENSG00000106609**	TMEM248	1070.141589	1085.993262	1245.776826	1216.500498	1289.860466	1334.285626	1284.914325	1320.743546	1345.922178	1374.392844	1352.012295	1492.035682
**ENSG00000106610**	STAG3L4	189.8411491	227.7082646	150.6331456	166.6094969	177.991498	145.3032115	247.1774774	272.4552723	223.2261174	271.46157	259.3656239	230.064459
**ENSG00000106615**	RHEB	1508.885578	1523.893771	1594.268644	1377.249359	1418.15304	1578.607476	1215.459497	1226.048725	1240.405631	1245.306223	1066.434188	1231.521516
**ENSG00000106617**	PRKAG2	2.812461468	14.01281628	8.956565414	0.837233653	6.93473369	9.863925586	183.8510163	408.6829085	111.6130587	196.7938188	372.4931832	140.406986
**ENSG00000106628**	POLD2	1008.267436	945.865099	989.2933616	1357.992985	1176.593149	1355.531005	972.3675972	807.3979412	1140.047671	1118.117935	949.167815	1201.917633
**ENSG00000106635**	BCL7B	413.4318358	224.2050605	402.2312104	383.453013	330.5556392	329.6827436	300.2899933	471.8127887	309.5152048	331.5754374	395.9464577	304.497078
**ENSG00000106636**	YKT6	1386.543504	1285.675894	1653.707669	1554.742893	1363.830959	1715.564288	1115.362832	1353.969798	1377.342661	1226.955674	1288.550493	1211.22171
**ENSG00000106638**	TBL2	532.9614482	395.86206	523.5519601	541.6901734	518.9492378	507.2334042	445.3280172	456.8609749	511.6380127	439.1476214	470.4450944	476.199597
**ENSG00000106665**	CLIP2	521.7116024	550.0030391	388.3892457	351.6381342	401.058765	288.3301325	24.51346884	18.274439	43.14454369	31.63887762	30.35129641	61.74524082
**ENSG00000106682**	EIF4H	6728.814063	6365.321796	6679.969332	5940.172767	5628.692178	5812.887224	4169.332491	5251.409243	4510.480666	4091.539654	5045.213226	4579.297724
**ENSG00000106683**	LIMK1	347.3389913	269.7467134	355.0056837	292.1945449	250.8062018	289.8476595	201.2147234	149.5181373	237.7639527	151.233835	155.8952952	170.8566938
**ENSG00000106686**	SPATA6L	43.59315276	52.54806106	46.41129351	58.6063557	50.85471372	50.45777319	28.59904698	18.274439	25.3239713	13.92110615	40.00852709	12.68737825
**ENSG00000106688**	SLC1A1	4.218692202	7.006408141	0	0	6.93473369	0	0	0	0.468962431	4.429442867	2.759208765	0.845825217
**ENSG00000106689**	LHX2	15.46853808	10.50961221	15.47043117	15.07020575	18.49262317	8.725780326	1.021394535	3.322625272	9.848211061	3.796665314	17.93485697	8.458252168
**ENSG00000106692**	FKTN	1393.574658	1159.560547	1428.165067	1429.157845	1274.83521	1421.922811	1434.037927	1142.983094	1402.666632	1419.32005	1252.680779	1525.022866
**ENSG00000106701**	FSD1L	132.185689	255.7338971	146.5619795	138.9807864	198.7956991	143.7856845	116.438977	194.3735784	84.88220009	120.227735	169.691339	88.81164776
**ENSG00000106714**	CNTNAP3	458.4312193	374.8428355	465.7414015	447.0827706	407.9934987	440.4622156	297.2258096	270.7939597	371.4182457	224.6360311	262.1248326	280.813972
**ENSG00000106723**	SPIN1	2837.773622	2704.473542	3230.063182	3007.343281	2870.979747	3318.831578	2546.336575	2528.517832	2655.265287	2579.834081	2589.517426	2722.711373
**ENSG00000106733**	NMRK1	480.9309111	595.544692	514.5953947	353.3126015	456.5366346	355.1013211	407.5364194	470.151476	479.2796049	399.9154131	419.3997322	384.0046484
**ENSG00000106771**	TMEM245	3419.953145	3023.265113	3133.983662	3050.879431	3372.592151	3449.338901	2123.479238	2151.399864	2183.020118	1928.073202	2154.942045	2198.299738
**ENSG00000106772**	PRUNE2	8638.4754	3345.559887	3898.548655	4908.700907	3812.94774	2562.344362	45.96275407	44.85544118	231.6674411	24.67832454	59.32298844	235.1394103
**ENSG00000106780**	MEGF9	746.7085198	655.0991612	632.6592115	766.0687924	822.9217312	813.7738608	555.638627	446.8930991	614.8097476	668.2130953	553.2213573	829.7545377
**ENSG00000106785**	TRIM14	980.1428217	760.1952833	837.8459828	465.501911	344.4251066	354.3425576	739.4896433	533.2813562	711.8849709	386.6270845	274.5412721	296.8846511
**ENSG00000106789**	CORO2A	22.49969175	49.04485699	17.91313083	34.32657977	43.91998003	22.7629052	7.149761744	11.62918845	18.28953483	20.88165923	13.79604382	9.304077385
**ENSG00000106799**	TGFBR1	9241.748385	11070.12486	8306.807305	9284.083977	12904.38361	8956.444432	6876.028009	7381.212042	5464.350251	7445.260681	7969.974517	6316.622719
**ENSG00000106803**	SEC61B	1198.108586	1257.650261	1331.271314	1220.686666	1054.079521	1221.609246	730.2970924	850.5920697	873.6770098	809.955267	913.2981011	951.5533689

ENSG00000106804	C5	137.8106119	192.6762239	105.8503185	80.37443068	165.2778196	96.36296534	243.0918993	181.0830773	207.2813947	151.8666126	162.7933171	117.5697051
ENSG00000106823	ECM2	16.87476881	0	4.071166097	0.837233653	5.778944741	3.035054026	16.34231256	29.90362745	16.88264753	21.51443678	34.49010956	16.07067912
ENSG00000106829	TLE4	2120.595947	1758.608443	1991.614455	2531.794566	2404.041012	2138.574943	1276.743169	1644.69951	1336.073967	1195.949574	1480.315502	1188.38443
ENSG00000106852	LHX6	7.031153671	3.50320407	4.885399317	12.55850479	21.95999002	10.62268909	8.171156279	13.29050109	5.158586746	6.327775524	8.277626294	8.458252168
ENSG00000106853	PTGR1	826.8636717	1114.018894	790.6204561	900.0261768	1151.165792	901.4110459	726.2115143	1294.162544	813.6498186	828.9385936	1283.032076	861.8958959
ENSG00000106868	SUSD1	140.6230734	182.1666117	140.862347	193.4009738	179.147287	179.4475693	297.2258096	240.8903322	256.52245	261.9699067	260.7452283	274.8931955
ENSG00000106948	AKNA	624.366446	451.9133251	660.343141	525.782734	524.7281825	553.8973598	309.4825441	294.0523366	394.3974049	358.7848722	339.382678	356.9382415
ENSG00000106952	TNFSF8	5.624922937	10.50961221	10.58503185	7.535102876	5.778944741	3.793817533	1.021394535	0	0.468962431	2.53111021	1.379604382	5.074951301
ENSG00000106976	DNM1	49.2180757	70.06408141	29.3123959	88.7467672	100.5536385	39.8350841	25.53486337	19.93575163	18.28953483	38.5994307	28.97169203	34.67883389
ENSG00000106991	ENG	226.4031482	136.6249587	270.3254289	241.123292	226.5346339	237.1135958	68.43343384	66.45250545	60.02719123	51.25498174	45.52694462	62.59106604
ENSG00000106992	AK1	11.24984587	3.50320407	23.61276336	28.4659442	26.58314581	31.48868552	7.149761744	8.306563181	16.4136851	8.226108181	22.07367012	13.53320347
ENSG00000106993	CDC37L1	165.9352266	154.1409791	210.8864038	200.9360767	204.5746438	174.5156065	132.7812895	121.2758224	128.4957062	110.7360717	124.1643944	120.1071808
ENSG00000107014	RLN2	16.87476881	10.50961221	9.770798633	20.93084132	18.49262317	13.27836137	8.171156279	3.322625272	7.034436472	8.858885733	0	16.91650434
ENSG00000107018	RLN1	0	0	0.814233219	0.837233653	0	7.967016819	0	4.983937908	1.406887294	5.694997971	1.379604382	5.920776517
ENSG00000107020	PLGRKT	264.371378	154.1409791	204.3725381	277.9615728	279.7009255	271.6373354	149.1236021	96.3561329	131.3094808	149.3355024	133.8216251	198.7689259
ENSG00000107021	TBC1D13	345.9327606	448.410121	471.4410341	400.1976861	392.9682424	437.0477798	403.4508413	405.3602832	475.5279055	440.4131765	453.8898418	361.1673676
ENSG00000107036	RIC1	575.1483703	606.0543042	630.2165119	731.7422126	661.1112784	724.6191488	636.3287952	651.2345534	568.8514294	573.92924	669.1081254	586.1568752
ENSG00000107077	KDM4C	368.4324523	511.4677943	470.6268008	436.1987331	606.7891978	452.9818134	379.958767	435.2639107	386.8940059	391.6893049	430.4365673	313.8011554
ENSG00000107099	DOCK8	12.65607661	31.52883663	13.84196473	8.372336529	3.467366845	10.24330734	18.38510163	43.19412854	46.89624315	32.90443272	55.18417529	62.59106604
ENSG00000107104	KANK1	469.6810652	276.7531216	267.8827292	673.1358569	329.3998503	455.6374857	897.8057962	709.3804956	1071.579156	940.3074428	722.9126963	1063.202297
ENSG00000107105	ELAVL2	1.406230734	0	0	3.348934611	1.155788948	2.27629052	0	0	0	0	1.379604382	0
ENSG00000107130	NCS1	205.3096872	164.6505913	188.0878737	252.8445632	264.6756692	278.0868252	11.23533988	28.24231481	26.73085859	21.51443678	48.28615338	34.67883389
ENSG00000107140	TESK1	188.4349184	406.3716722	306.1516905	211.8201142	320.1535387	296.6765311	211.4286687	313.9880882	252.3017881	208.1838147	246.9491844	241.0601868
ENSG00000107159	CA9	0	24.52242849	0.814233219	0	4.623155793	0	0	34.88756536	0.468962431	1.898332657	24.83287888	0
ENSG00000107164	FUBP3	1579.197114	1278.669486	1652.079202	1525.439716	1332.624657	1515.250723	1369.690071	1340.679297	1337.480855	1366.799513	1383.743195	1528.406167
ENSG00000107175	CREB3	420.4629895	500.9581821	468.1841012	377.5923774	495.8334588	525.8231101	351.35972	338.9077778	351.7218236	287.9137863	368.3543701	330.7176598
ENSG00000107185	RGP1	1133.421972	1320.707935	1237.634494	1182.173918	1143.07527	1225.403063	1065.3145	1034.997772	1096.434165	969.4152102	1117.47955	1052.20657
ENSG00000107186	MPDZ	0	0	0.814233219	0	0	0	41.87717593	122.9371351	18.28953483	24.67832454	184.8669872	15.2248539
ENSG00000107201	DDX58	604.6792157	931.8522827	530.8800591	98.79357104	99.39784955	113.0557625	203.2575124	305.681525	174.9229869	82.26108181	77.25784541	72.74096864
ENSG00000107223	EDF1	2069.971641	2129.948075	2289.623813	1566.464165	1716.346588	2020.207836	1073.485656	1063.240087	1390.473609	1047.879627	1052.638144	1170.6221
ENSG00000107242	PIP5K1B	1.406230734	0	0	0	4.623155793	1.13814526	342.1671692	112.9692593	192.2745969	279.6876782	113.1275593	127.7196077
ENSG00000107249	GLIS3	0	3.50320407	8.142332194	0	0	2.655672273	50.04833221	74.75906863	46.42728071	41.76331846	96.57230676	18.60815477
ENSG00000107262	BAG1	621.5539845	679.6215897	550.4216563	768.5804933	842.5701433	746.6232905	746.639405	450.2157244	694.533361	732.1236281	555.9805661	632.6772621
ENSG00000107263	RAPGEF1	1593.259422	1583.44824	1861.33714	1620.047118	2009.916981	2085.082116	2621.919771	4063.570708	2605.086307	2560.850754	3770.458777	2457.96808
ENSG00000107281	NPDC1	140.6230734	234.7146727	123.7634494	216.0062824	232.3135786	144.544448	210.4072742	262.4873965	233.0743284	208.1838147	241.4307669	215.6854303
ENSG00000107282	APBA1	0	0	0	1.674467306	0	3.41443578	38.81299233	54.82331699	41.7376564	46.82553888	37.24931832	51.59533822
ENSG00000107290	SETX	7400.992354	6771.693468	7310.185844	4853.443486	4108.829711	4742.651298	4977.255568	5256.393181	4469.680934	4083.313546	4264.357146	4520.089958
ENSG00000107331	ABCA2	763.5832886	728.6664466	683.9559043	743.4634837	907.2943244	772.8006315	589.3446466	603.0564869	570.2583166	657.4558769	731.1903226	588.6943509
ENSG00000107338	SHB	194.0598413	315.2883663	228.7995347	246.9839276	246.183046	300.0909669	155.2519693	139.5502614	126.6198565	169.584384	180.7281741	137.0236851
ENSG00000107341	UBE2R2	967.4867451	1121.025303	1143.997673	1156.219675	1153.47737	1229.196881	851.8430421	905.4153867	966.0626088	927.6518918	983.6579246	903.3413315
ENSG00000107362	ABHD17B	509.0555258	273.2499175	376.9899806	502.3401917	401.058765	470.8127559	466.7773024	352.1982789	447.3901596	508.1203746	424.9181498	546.40309
ENSG00000107371	EXOSC3	458.4312193	476.4357536	435.6147724	503.1774254	416.0840214	442.3591244	449.4135953	407.0215959	481.1554547	408.1415213	474.5839075	507.4951301
ENSG00000107372	ZFAND5	5719.140396	5356.399024	6098.606814	5687.328204	5108.587151	6284.079362	4731.099486	3304.350833	4617.4041	5126.130952	3770.458777	5306.70741
ENSG00000107404	DVL1	873.2692859	683.1247937	885.8857428	1063.286739	1058.702677	1170.772091	385.0657396	393.7310948	403.7766535	406.2431886	372.4931832	420.3751327
ENSG00000107438	PDLIM1	625.7726767	627.0735286	530.8800591	773.6038952	742.0165048	734.1036926	1767.012545	1714.47464	2306.8262	2046.402604	2087.34143	2455.430604
ENSG00000107485	GATA3	2171.220254	1646.505913	2151.204166	2478.211612	2093.133785	2538.443311	1362.54031	1639.715572	1517.562428	1414.25783	1608.61871	1541.93937
ENSG00000107518	ATRNL1	1.406230734	0	0.814233219	2.511700959	2.311577897	3.035054026	1.021394535	1.661312636	2.344812157	0	0	0
ENSG00000107521	HPS1	361.4012987	399.365264	371.2903481	271.2637035	349.0482624	351.3075036	272.7123408	337.2464651	341.4046501	272.7271251	307.6517773	247.8267885
ENSG00000107537	PHYH	43.59315276	168.1537954	49.66822639	72.00209415	108.6441611	84.98151274	165.4659146	166.1312636	116.302683	214.5115903	190.3854048	131.9487338
ENSG00000107551	RASSF4	7.031153671	7.006408141	0	10.88403749	34.67366845	7.967016819	14.29952349	19.93575163	10.31717349	19.61610412	20.69406573	17.76232955
ENSG00000107554	DNMBP	2152.939254	1699.053974	2284.738414	1819.308728	1600.767693	2072.9419	656.7566859	850.5920697	735.8020549	610.630338	786.3744979	826.3712368
ENSG00000107560	RAB11FIP2	579.3670625	367.8364274	557.7497553	497.3167898	488.8987251	506.0952589	728.2543034	495.0711656	634.9751322	653.6592116	562.878588	679.1976491
ENSG00000107566	ERLIN1	646.8661377	711.1504263	740.9522297	923.4687191	805.5848969	912.0337349	635.3074007	636.2827396	792.0775467	743.513624	658.0712904	811.1463829
ENSG00000107581	EIF3A	9118.00008	7721.061771	8828.730798	9253.943565	8766.659173	8984.898064	7209.002627	6477.457968	7677.852928	7553.465643	6728.330573	8410.04013
ENSG00000107611	CUBN	1.406230734	35.0320407	9.770798633	5.023401917	75.12628164	48.94024618	0	0	0	0	5.518417529	0.845825217
ENSG00000107614	TRDMT1	81.56138258	49.04485699	81.42332194	61.95529031	86.68417112	80.80831345	43.919965	43.19412854	46.89624315	48.09109398	40.00852709	45.67456171
ENSG00000107625	DDX50	1101.078665	1548.416199	1141.554974	1170.452647	1254.031009	1129.040098	1185.839055	1227.710038	1181.785327	1116.85238	1277.513658	1340.632969
ENSG00000107643	MAPK8	420.4629895	599.047896	530.8800591	524.9455003	481.9639914	662.779923	434.0926773	446.8930991	400.4939165	439.7803989	389.0484358	425.450084
ENSG00000107651	SEC23IP	1036.392051	735.6728548	1045.475454	1033.146328	931.5658923	986.3925586	905.9769524	807.3979412	846.9461512	835.2663691	882.9468047	857.6667698
ENSG00000107669	ATE1	698.8966749	564.0158553	740.9522297	703.2762684	602.166042	713.9964597	538.2749199	486.7646024	579.6375653	574.5620176	491.1391601	636.060563
ENSG00000107672	NSMCE4A	399.3695285	248.727489	342.7921854	332.3817602	208.0420107	223.0764709	345.2313528	280.7618355	322.1771904	290.4448965	198.6630311	257.1308659
ENSG00000107679	PLEKHA1	2062.940487	2077.400014	1835.281677	2172.621329	2159.013755	2130.228545	1914.093358	2387.306258	2045.145164	1867.326557	2170.117693	2108.642265
ENSG00000107719	PALD1	25.31215321	3.50320407	13.84196473	26.79147689	64.7241811	26.93610448	0	0	1.406887294	1.265555105	1.379604382	0
ENSG00000107731	UNC5B	149.0604578	80.57369362	101.7791524	209.3084132	150.2525633	120.2640158	184.8724108	101.3400708	296.3842567	196.7938188	114.5071637	235.1394103
ENSG00000107736	CDH23	14.06230734	0	1.628466439	9.209570182	8.090522638	9.484543833	20.4278907	48.17806645	14.53783538	25.94387965	56.56377967	12.68737825
ENSG00000107738	VSIR	539.9926019	483.4421617	496.6822639	677.3220252	659.9554895	816.8089149	1071.442867	1804.185523	1024.682913	840.3285896	1434.788558	642.8271647
ENSG00000107742	SPOCK2	33.74953762	63.05767327	37.45472809	19.25637402	35.8294574	32.24744903	18.38510163	18.274439	15.94472267	22.14721433	17.93485697	19.45397999

**ENSG00000107745**	MICU1	814.2075951	959.8779153	848.4310147	960.3069998	1073.727933	997.0152477	759.9175339	797.4300653	730.1745058	820.7124854	791.8929154	835.6753142
**ENSG00000107758**	PPP3CB	1338.731659	1212.108608	1029.190789	1140.312235	1087.5974	1041.023531	862.0569874	734.3001852	761.5949887	786.5424976	826.383025	775.6217238
**ENSG00000107771**	CCSER2	1302.16966	1716.569995	1466.434028	1479.391865	1590.365593	1584.677584	1081.656812	1192.822473	1031.717349	1055.472957	1214.051856	1186.692779
**ENSG00000107779**	BMPR1A	846.550902	770.7048955	850.0594811	1021.425056	1039.054264	1014.466808	1221.587864	1069.885338	1261.977903	1145.32737	1200.255813	1305.954135
**ENSG00000107789**	MINPP1	358.5888372	350.320407	330.5786871	277.1243391	283.1682923	326.6476896	306.4183605	350.5369662	338.1219131	294.2415619	263.504437	379.7755223
**ENSG00000107796**	ACTA2	63.28038304	87.58010176	45.59706029	5.86063557	11.55788948	6.828871559	54.13391035	46.51675381	37.04803208	14.5538837	9.657230676	19.45397999
**ENSG00000107798**	LIPA	1482.167194	1558.925811	1353.255611	1592.418408	1680.517131	1598.714708	1299.213848	1554.988627	1348.735953	1480.066695	1466.519458	1545.322671
**ENSG00000107815**	TWNK	348.7452221	143.6313669	381.8753799	360.0104707	254.2735686	401.0065132	373.8303998	224.2772059	337.1839882	344.2309885	216.597888	345.0966884
**ENSG00000107816**	LZTS2	253.1215321	357.3268152	266.2542628	174.1445998	220.7556891	183.6207686	333.9960129	448.5544118	390.176743	336.0048803	342.1418868	301.1137772
**ENSG00000107819**	SFXN3	478.1184496	1068.477241	516.2238611	605.319931	1188.151039	571.3489205	480.0554314	2207.884493	404.7145783	402.4465233	1946.621783	349.3258145
**ENSG00000107821**	KAZALD1	68.90530597	164.6505913	64.32442434	77.02549606	114.4231059	86.49903975	366.680638	382.1019063	463.8038447	380.9320865	395.9464577	372.1630954
**ENSG00000107829**	FBXW4	278.4336854	381.8492437	312.6655563	280.4732737	290.103026	282.2600245	480.0554314	561.523671	466.1486569	460.0292806	371.1135788	418.6834823
**ENSG00000107833**	NPM3	160.3103037	59.5544692	162.0324107	236.0998901	173.3683422	195.381603	112.3533988	71.43644335	142.0956167	136.0471738	104.8499331	154.7860147
**ENSG00000107854**	TNKS2	1951.848259	2119.438463	2055.938879	1471.019528	1569.561392	1718.978724	1467.743947	1418.760991	1382.97021	1233.28345	1267.856427	1349.937046
**ENSG00000107862**	GBF1	2019.347334	2017.845545	2156.903798	2060.43202	2139.365343	2154.508977	2413.555286	2302.579314	2161.916809	2150.178123	2394.993208	2171.233331
**ENSG00000107863**	ARHGAP21	5835.857547	6291.75451	6349.390645	5028.425319	5494.62066	5043.501028	4953.763494	6198.357445	4815.306246	4566.755596	6015.075107	4623.280635
**ENSG00000107864**	CPEB3	91.40499772	157.6441832	119.6922833	44.3733836	38.14103529	59.56293527	102.1394535	71.43644335	91.91663656	77.19886139	46.906549	84.58252168
**ENSG00000107872**	FBXL15	23.90592248	52.54806106	35.01202844	31.81487881	36.98524634	46.2845739	25.53486337	29.90362745	39.86180667	33.53721028	41.38813147	28.75805737
**ENSG00000107874**	CUEDC2	264.371378	108.5993262	209.2579374	208.4711796	343.2693176	232.9403965	148.1022076	151.1794499	181.488461	175.279382	151.7564821	143.7902869
**ENSG00000107882**	SUFU	122.3420739	161.1473872	149.8189124	159.9116277	219.5999002	194.2434577	202.2361179	309.0041503	226.9778168	198.0593739	237.2919538	219.9145564
**ENSG00000107890**	ANKRD26	542.8050634	714.6536304	515.4096279	705.7879694	696.9407358	535.6870357	783.4096082	898.7701361	583.8582272	780.2147221	1000.213177	696.9599786
**ENSG00000107897**	ACBD5	620.1477538	735.6728548	591.1333173	451.2689389	594.0755194	498.1282421	460.6489352	559.8623584	401.9008038	370.1748681	456.6490505	467.7413449
**ENSG00000107902**	LHPP	109.6859973	143.6313669	135.9769476	129.7712162	158.3430859	169.5836437	234.920743	234.2450817	196.4952588	249.3143556	244.1899757	188.6190233
**ENSG00000107929**	LARP4B	2609.964243	2249.057013	2403.616464	2512.538192	2125.495876	2594.971193	1875.280366	1716.135953	1662.940782	1921.745427	1845.910664	1950.47295
**ENSG00000107937**	GTPBP4	2796.99293	2059.883993	2769.207179	2889.293336	2089.666418	2836.637369	1281.850141	1030.013834	1219.771284	1311.115089	1134.034802	1525.868691
**ENSG00000107938**	EDRF1	722.8025974	641.0863449	701.0548019	775.2783626	728.1470374	828.5697492	727.2329088	671.170305	707.6643091	788.4408303	707.7370481	795.0757038
**ENSG00000107949**	BCCIP	1012.486129	1064.974037	980.3367962	1153.707974	1017.094274	1039.506004	748.6821941	712.7031209	806.6153821	761.8641731	797.411333	898.2663802
**ENSG00000107951**	MTPAP	441.5564505	245.2242849	385.946546	479.7348831	342.1135287	464.7426478	415.7075757	345.5530283	485.845079	445.4753969	373.8727876	444.0582388
**ENSG00000107954**	NEURL1	4366.34643	4382.508292	4172.131016	3836.204597	4533.004255	3956.192923	2942.637655	4018.715267	3005.111261	2816.492886	4574.768132	2527.325748
**ENSG00000107957**	SH3PXD2A	942.1745919	861.7882013	818.3043855	1104.311188	974.3300834	1068.718399	521.9326073	925.3511383	769.0983876	697.3208627	1026.42566	893.1914289
**ENSG00000107959**	PITRM1	1065.922896	1142.044527	1249.847992	1044.030365	1113.024757	1150.664858	516.8256346	475.1354139	504.1346138	632.1447748	637.3772246	558.2446431
**ENSG00000107960**	STN 1.00	358.5888372	707.6472222	329.7644539	525.782734	640.3070773	578.5571738	353.4025091	410.3442211	342.8115374	414.4692968	430.4365673	351.8632902
**ENSG00000107968**	MAP3K8	471.0872959	763.6984874	626.959579	360.0104707	375.6314082	346.7549225	303.3541769	505.0390414	324.990965	263.8682393	406.9832928	268.1265937
**ENSG00000107984**	DKK 1.00	684.8343675	420.3844885	688.0270704	492.2933879	438.0440114	475.3653369	2.04278907	3.322625272	3.751699452	1.898332657	0	5.074951301
**ENSG00000108010**	GLRX3	1999.660104	1884.72379	2016.041451	1956.615047	1969.464368	2072.9419	1368.668677	1450.325931	1504.43148	1483.230583	1596.20227	1623.138591
**ENSG00000108018**	SORCS1	7.031153671	0	4.071166097	0	2.311577897	0	0	0	0	0	4.138813147	0
**ENSG00000108021**	FAM208B	3978.226747	2504.79091	3697.43305	3209.953825	2368.211555	3032.398354	3260.291355	2402.258072	3022.931833	2629.19073	2085.961826	2775.152536
**ENSG00000108039**	XPNPEP1	876.0817474	1159.560547	978.7083298	769.417727	925.7869476	827.0522222	943.7685502	1206.112974	987.1659182	767.559171	1065.054583	836.5211394
**ENSG00000108055**	SMC3	2791.368007	3033.774725	2606.360535	3034.971992	2822.436612	2566.896943	3044.777108	2688.003845	2842.381297	2955.703947	2761.967973	3281.801841
**ENSG00000108061**	SHOC2	811.3951336	984.4003438	1017.791524	833.8847183	929.2543144	937.4523124	1029.565691	1091.482402	975.4418574	908.6685652	968.4822764	1022.602687
**ENSG00000108064**	TFAM	746.7085198	507.9645902	609.0464481	736.7656145	702.7196805	677.1964296	779.3240301	583.1207353	740.9606417	727.0614077	667.728521	877.1207498
**ENSG00000108091**	CCDC6	1358.418889	1184.082976	1361.397943	1595.767342	1748.708679	1685.59313	2222.554508	1344.001923	1939.159654	2280.530299	1674.83972	2326.865171
**ENSG00000108094**	CUL2	1044.829435	900.3234461	1015.348825	805.4187741	834.4796206	847.5388369	784.4310028	819.0271296	741.8985666	685.9308668	641.5160378	779.0050247
**ENSG00000108100**	CCNY	351.5576835	381.8492437	388.3892457	526.6199677	569.8039515	552.7592146	585.2590685	632.9601144	563.6928426	593.5453441	553.2213573	588.6943509
**ENSG00000108106**	UBE2S	693.2717519	441.4037129	583.8052183	955.2835979	770.9112285	992.8420484	282.9262862	129.5823856	304.8255804	308.7954456	140.719647	399.2295023
**ENSG00000108107**	RPL28	2622.620319	2571.351788	2927.982657	2213.645778	2275.748439	2936.794152	2266.474473	2704.616972	2982.132102	2310.903621	2454.316196	2334.477598
**ENSG00000108175**	ZMIZ1	1574.978422	1303.191914	1436.307399	1756.516204	1767.201302	1813.065399	403.4508413	476.7967266	607.7753112	348.6604314	383.5300183	447.4415397
**ENSG00000108176**	DNAJC12	0	7.006408141	2.442699658	0	0	1.13814526	0	0	0	1.265555105	0	0
**ENSG00000108179**	PPIF	1733.882495	2361.159543	2622.6452	1761.539606	1768.357091	2520.612369	531.1251581	448.5544118	527.113773	597.974787	517.3516434	583.6193996
**ENSG00000108187**	PBLD	109.6859973	73.56728548	114.8068839	123.073347	134.071518	97.88049235	218.5784305	131.2436983	194.6194091	237.9243597	171.0709434	184.3898973
**ENSG00000108219**	TSPAN14	4190.567588	3937.601375	4882.142384	5076.984871	4620.844215	6129.670988	2685.246232	2535.163083	2898.187826	2990.506713	2964.769818	3029.745926
**ENSG00000108231**	LGI1	0	0	0	0	0	0	0	1.661312636	2.813774589	6.327775524	9.657230676	9.304077385
**ENSG00000108239**	TBC1D12	171.5601496	217.1986524	206.0010045	167.4467306	275.0777697	265.9466091	228.7923758	337.2464651	258.8672622	272.7271251	349.0399087	303.6512528
**ENSG00000108244**	KRT23	9.843615139	0	9.770798633	12.55850479	5.778944741	12.51959786	0	0	8.441323766	1.265555105	0	8.458252168
**ENSG00000108256**	NUFIP2	5086.336565	5037.607453	5219.234937	4730.370139	4699.437864	5217.637253	3993.652631	3653.226487	4147.972706	3678.96869	3581.452976	4217.284531
**ENSG00000108262**	GIT1	592.0231391	749.6856711	718.9679328	569.3188839	835.6354096	840.7099653	649.6069242	706.0578704	734.8641301	752.3725098	798.7909374	654.6687178
**ENSG00000108298**	RPL19	8957.689777	8940.176788	8823.031166	8372.336529	8356.354096	8346.019191	7375.489936	8386.306187	7766.486827	6963.084186	7751.997024	7726.613355
**ENSG00000108306**	FBXL20	500.6181414	711.1504263	539.0223913	512.3869956	703.8754695	514.0622757	643.478557	696.0899945	561.3480304	690.9930872	786.3744979	543.8656144
**ENSG00000108309**	RUNDC3A	0	0	0.814233219	2.511700959	5.778944741	1.13814526	1.021394535	0	0.468962431	0.632777552	0	0
**ENSG00000108312**	UBTF	1407.636965	1306.695118	1422.465434	1636.791791	1748.708679	1634.376593	1661.808908	1604.828006	1767.519404	1669.899961	1611.377919	1737.324995
**ENSG00000108344**	PSMD3	1766.225802	1635.996301	1877.621804	1820.145961	1755.643412	1945.469631	1197.074395	1169.564096	1167.716454	1274.41399	1364.428734	1305.954135
**ENSG00000108349**	CASC3	1238.889277	1450.326485	1484.347159	1635.954558	1479.409854	1613.889979	1351.30497	1179.531972	1412.983806	1298.459537	1336.836646	1346.553745
**ENSG00000108352**	RAPGEFL1	44.99938349	49.04485699	29.3123959	66.14145858	34.67366845	32.62683078	14.29952349	34.88756536	21.10330942	28.47498986	17.93485697	33.83300867
**ENSG00000108370**	RGS9	156.0916115	269.7467134	201.1156052	182.5169363	278.5451365	167.3073532	106.2250316	93.03350762	118.6474952	137.9455064	104.8499331	123.4904816
**ENSG00000108375**	RNF43	15.46853808	35.0320407	24.42699658	17.58190671	28.89472371	28.4536315	9.192550814	11.62918845	11.72406079	11.38999594	13.79604382	9.304077385
**ENSG00000108379**	WNT3	9.843615139	7.006408141	15.47043117	43.53614995	31.2063016	42.49075637	1.021394535	6.645250545	5.627549177	13.2883286	8.277626294	18.60815477
**ENSG00000108384**	RAD51C	359.9950679	413.3780803	312.6655563	437.8732004	416.0840214	434.0127258	267.6053681	247.5355828	264.0258489	275.2582353	357.317535	279.9681468

**ENSG00000108387**	4-Sep	98.43615139	133.1217547	96.89375311	3.348934611	9.246311586	2.27629052	14.29952349	19.93575163	7.503398903	0	0	0.845825217
**ENSG00000108389**	MTMR4	1037.798282	1121.025303	966.4948315	909.235747	969.7069276	847.9182186	1038.758242	1084.837151	1057.979245	1048.512404	1074.711814	955.782495
**ENSG00000108395**	TRIM37	817.0200565	991.4067519	816.6759191	848.1176904	929.2543144	912.4131167	695.5696783	838.9628812	731.5813931	675.8064259	762.9212234	772.2384229
**ENSG00000108406**	DHX40	867.644363	781.2145077	762.9365266	811.2794096	995.1342845	777.3532125	1073.485656	1147.967032	996.5451668	1042.184629	1151.969659	996.3821054
**ENSG00000108423**	TUBD1	205.3096872	189.1730198	188.0878737	226.8903199	224.223056	206.004292	196.1077507	186.0670152	189.9297847	230.3310291	183.4873828	242.7518372
**ENSG00000108424**	KPNB1	9612.993299	9136.356216	9780.569432	10268.67075	9456.665175	9821.814211	6869.899642	7439.357985	7098.684325	6668.842625	6855.254176	7698.701123
**ENSG00000108433**	GOSR2	926.7060538	1040.451609	1089.444048	694.9039319	753.5743943	833.501712	1017.308957	1011.739395	934.6421259	904.2391224	1012.629617	818.7588098
**ENSG00000108439**	PNPO	372.6511446	353.8236111	376.1757474	478.0604158	462.3155793	481.8148267	427.9643101	265.8100218	427.6937375	362.5815375	313.1701948	405.9961041
**ENSG00000108443**	RPS6KB1	742.4898276	784.7177118	681.5132047	753.5102876	544.3765946	661.262396	789.5379755	719.3483714	701.5677975	739.7169587	619.4423677	685.1184256
**ENSG00000108448**	TRIM16L	192.6536106	168.1537954	232.0564675	225.2158526	295.8819708	232.9403965	133.8026841	187.7283279	157.571377	175.279382	150.3768777	122.6446564
**ENSG00000108465**	CDK5RAP3	108.2797665	73.56728548	91.19412058	110.5148422	83.21680428	85.36089449	164.4445201	159.4860131	97.54418574	167.6860514	151.7564821	107.4198025
**ENSG00000108468**	CBX1	1567.947269	1390.772016	1500.631823	1904.70656	1657.401352	1997.444931	1587.247107	1980.284662	1855.684341	1722.420498	2034.916464	2144.166925
**ENSG00000108469**	RECQL5	518.8991409	465.9261414	511.3384618	411.9189572	439.1998003	453.740577	473.9270642	455.1996623	488.1898911	484.0748276	438.7141936	396.6920267
**ENSG00000108474**	PIGL	229.2156097	129.6185506	207.629471	244.4722266	190.7051765	201.8310928	352.3811145	189.3896405	200.2469582	275.2582353	233.1531406	229.2186337
**ENSG00000108479**	GALK1	32.34330689	10.50961221	17.09889761	46.88508456	43.91998003	34.52373955	41.87717593	59.8072549	53.46171719	53.1533144	128.3032076	45.67456171
**ENSG00000108506**	INTS2	634.2100611	546.499835	606.6037485	553.4114445	501.6124035	566.0375759	631.2218226	750.9133115	687.4989245	687.8291994	642.8956422	720.6430847
**ENSG00000108509**	CAMTA2	334.6829147	276.7531216	306.9659237	370.8945082	376.7871971	429.8395265	295.1830206	245.8742701	339.9977628	260.071574	257.9860195	301.1137772
**ENSG00000108510**	MED13	5508.205786	6396.850633	6315.19285	4321.800116	4601.195803	5151.624828	3831.2509	3649.903862	3818.292117	3257.53884	3291.736056	3626.052704
**ENSG00000108511**	HOXB6	1.406230734	7.006408141	13.84196473	0.837233653	5.778944741	0	3.064183605	1.661312636	2.344812157	1.898332657	0	0.845825217
**ENSG00000108515**	ENO3	2.812461468	31.52883663	18.72736405	33.48934611	35.8294574	26.55672273	51.06972674	44.85544118	62.84096581	67.7071981	56.56377967	49.05786257
**ENSG00000108518**	PFN1	6115.697463	5212.767657	6237.840694	6085.851423	5448.389102	7038.669669	4559.505204	3909.068633	5468.570913	4817.968284	4072.592137	5075.797126
**ENSG00000108523**	RNF167	995.6113598	1022.935589	1119.570677	1244.966442	1303.729934	1416.232085	879.4206945	815.7045043	988.1038431	986.5002042	1019.527639	1049.669094
**ENSG00000108528**	SLC25A11	562.4922937	357.3268152	569.1490204	648.0188473	530.5071273	714.3758415	341.1457746	382.1019063	540.7136835	515.0809276	328.345843	500.7285283
**ENSG00000108556**	CHRNE	0	3.50320407	0	0	3.467366845	3.41443578	5.106972674	1.661312636	0.937924863	5.694997971	2.759208765	5.074951301
**ENSG00000108557**	RAI1	637.0225226	528.9838146	562.6351546	713.3230722	698.0965248	711.7201692	369.7448216	465.1675381	397.2111794	403.7120784	553.2213573	382.312998
**ENSG00000108559**	NUP88	1144.671818	1050.961221	1214.02173	1429.995079	1293.327833	1348.322751	995.8596715	704.3965577	992.324505	935.2452224	716.0146744	1042.056667
**ENSG00000108561**	C1QBP	1722.632649	917.8394665	1382.568007	2100.619235	1686.296076	1748.191119	1371.73286	920.3672004	1343.577366	1587.006101	986.4171334	1667.121502
**ENSG00000108576**	SLC6A4	1.406230734	0	0	0	0	0.379381753	0	1.661312636	0.468962431	3.796665314	1.379604382	2.53747565
**ENSG00000108578**	BLMH	170.1539188	171.6569995	149.8189124	248.6583949	299.3493376	273.9136259	83.75435186	96.3561329	89.10286198	105.6738512	111.747955	126.8737825
**ENSG00000108582**	CPD	1258.576507	1229.624629	1502.26029	1741.445998	2041.123283	2054.731576	2091.816007	2158.045114	2023.103929	2332.418058	2510.879976	2634.74555
**ENSG00000108587**	GOSR1	881.7066703	819.7497525	1026.74809	853.9783259	766.2880727	959.0770724	726.2115143	883.8183224	835.2220904	820.0797079	838.7994644	795.921529
**ENSG00000108588**	CCDC47	1417.48058	1460.836097	1341.856346	1567.301398	1594.988749	1534.21981	1235.887387	1250.968415	1252.598654	1191.520131	1358.910317	1499.648109
**ENSG00000108590**	MED31	229.2156097	273.2499175	169.3605096	199.2616094	195.3283323	199.934184	163.4231256	189.3896405	152.8817527	149.3355024	148.9972733	171.702519
**ENSG00000108591**	DRG2	329.0579918	294.2691419	396.5315779	392.6625832	421.8629661	412.0085841	406.5150249	302.3588998	419.2524137	426.4920703	364.2155569	433.062511
**ENSG00000108592**	FTSJ3	863.4256708	784.7177118	908.6842729	858.1644942	776.6901732	863.8522523	725.1901198	877.1730719	697.816098	737.8186261	674.6265429	677.5059986
**ENSG00000108599**	AKAP10	769.2082116	872.2978135	845.1740818	689.88053	604.4776199	652.9159974	625.0934553	732.6388725	559.9411432	546.7198053	634.6180159	598.8442535
**ENSG00000108604**	SMARCD2	621.5539845	592.0414879	556.9355221	736.7656145	789.4038517	676.8170479	745.6180105	737.6228104	812.7118937	864.3741366	852.5955083	839.9044403
**ENSG00000108622**	ICAM2	33.74953762	168.1537954	40.71166097	9.209570182	49.69892478	34.9031213	0	0	0	0	0	0.845825217
**ENSG00000108639**	SYNGR2	561.0860629	535.9902228	566.7063207	650.5305483	533.9744941	775.8356855	391.1941069	313.9880882	507.4173508	499.2614888	412.5017103	460.1289179
**ENSG00000108641**	B9D1	99.84238213	154.1409791	113.1784175	111.3520758	134.071518	158.5815729	115.4175824	83.06563181	121.9302322	127.8210656	93.813098	102.3448512
**ENSG00000108651**	UTP6	1240.295508	1215.611812	1235.191794	1147.847338	1071.416355	1166.978273	949.8969174	908.738012	866.6425733	860.5774712	939.5105844	941.4034663
**ENSG00000108654**	DDX5	7818.642882	7048.44659	7500.716418	7329.143397	5996.233064	7457.886506	7078.264127	6337.907707	7154.959817	7033.955272	5759.848296	7338.379581
**ENSG00000108666**	C17orf75	324.8392996	287.2627338	282.5389271	298.8924141	265.8314581	333.8559429	273.7337353	242.5516449	283.2533086	293.6087843	222.1163056	338.3300867
**ENSG00000108669**	CYTH1	475.3059881	707.6472222	666.8570067	406.0583216	500.4566146	516.3385662	251.2630556	408.6829085	284.6601959	298.6710047	351.7991175	237.6768859
**ENSG00000108671**	PSMD11	3383.391146	3030.271521	3458.048483	3274.420816	2901.03026	3460.720354	1994.783527	2111.528361	2193.337292	2145.115903	2090.100639	2344.627501
**ENSG00000108679**	LGALS3BP	4736.185113	5191.748432	5056.388293	1051.565468	1407.750939	1273.584546	1669.980065	2008.526977	1944.318241	315.1232211	458.0286549	323.951058
**ENSG00000108691**	CCL2	4.218692202	0	10.58503185	0.837233653	0	0.379381753	0	0	0.468962431	1.265555105	0	0
**ENSG00000108733**	PEX12	133.5919197	140.1281628	144.1192798	106.3286739	121.3578396	94.84543833	162.401731	167.7925762	158.5093018	157.5616105	157.2748996	157.3234903
**ENSG00000108759**	KRT32	0	0	0	0	0	0.379381753	0	1.661312636	2.344812157	0	1.379604382	1.691650434
**ENSG00000108771**	DHX58	205.3096872	287.2627338	187.2736405	56.93188839	63.56839215	62.5979893	158.3161529	196.0348911	153.3507151	56.31720216	63.46180159	25.3747565
**ENSG00000108773**	KAT2A	337.4953762	241.7210809	324.8790546	358.3360034	276.2335586	350.1693583	279.8621026	245.8742701	327.3357772	337.903213	263.504437	315.4928059
**ENSG00000108774**	RAB5C	185.6224569	206.6890402	255.6692309	206.7967123	211.5093775	221.5589439	145.038024	162.8086383	192.2745969	170.2171616	140.719647	164.0900921
**ENSG00000108784**	NAGLU	132.185689	70.06408141	113.9926507	107.1659076	94.77469376	114.5732895	200.1933288	212.6480174	244.7983892	205.6527045	164.1729215	181.8524216
**ENSG00000108785**	HSD17B1P1	0	0	0.814233219	2.511700959	1.155788948	0.379381753	0	4.983937908	3.28273702	5.694997971	8.277626294	2.53747565
**ENSG00000108786**	HSD17B1	11.24984587	0	1.628466439	2.511700959	1.155788948	0.758763507	3.064183605	0	10.31717349	5.694997971	2.759208765	9.304077385
**ENSG00000108788**	MLX	729.833751	830.2593647	868.7868452	813.7911106	711.9659921	872.9574144	431.0284937	318.9720261	514.9207497	437.8820662	354.5583263	433.062511
**ENSG00000108797**	CNTNAP1	29.53084542	115.6057343	43.96859385	58.6063557	147.9409854	80.4289317	31.66323058	56.48462963	30.01359561	28.47498986	44.14734023	27.06640694
**ENSG00000108798**	ABI3	11.24984587	0	5.699632536	0	0	0.379381753	0	0	0	0	0	0
**ENSG00000108799**	EZH1	590.6169083	693.6344059	656.2719749	590.2497253	553.6229062	529.2375459	497.4191385	556.5397331	472.7141309	534.6970318	502.1759952	474.5079466
**ENSG00000108813**	DLX4	5.624922937	14.01281628	12.21349829	12.55850479	24.27156791	17.45156065	38.81299233	14.95181373	19.22745969	44.92720622	33.11050518	32.98718345
**ENSG00000108819**	PPP1R9B	1351.387736	1085.993262	1458.291696	1661.071567	1598.456115	1746.673592	1183.796266	1016.723333	1150.833807	1213.667345	1180.941351	1210.375885
**ENSG00000108821**	COL1A1	230.6218404	262.7403053	265.4400295	370.0572746	417.2398103	456.0168675	118.481766	88.04956971	83.94427523	125.9227329	113.1275593	109.9572782
**ENSG00000108823**	SGCA	0	35.0320407	0	0	0	2.655672273	0	0	0	0	0	1.691650434
**ENSG00000108826**	MRPL27	573.7421395	420.3844885	521.9234937	550.0625099	498.1450367	495.4725698	424.9001265	347.214341	382.6733441	436.6165111	420.7793366	449.1331901
**ENSG00000108828**	VAT1	930.924746	1071.980446	1108.985645	1205.61646	1519.862467	1402.574342	345.2313528	385.4245316	373.7630579	454.3342826	569.7766099	497.3452275
**ENSG00000108829**	LRRC59	3895.259134	3321.037459	4283.680968	4274.915032	3890.3856	4531.335661	2220.511719	2060.027669	2451.735592	2323.559172	2321.874175	2848.73933
**ENSG00000108830**	RND2	2.812461468	0	0.814233219	7.535102876	8.090522638	3.035054026	1.021394535	8.306563181	0.937924863	1.898332657	0	3.383300867

**ENSG00000108840**	HDAC5	376.8698368	472.9325495	420.1443412	349.9636669	469.250313	371.4147365	298.2472042	350.5369662	359.6941849	251.8454658	390.4280402	251.2100894
**ENSG00000108846**	ABCC3	475.3059881	641.0863449	796.3200886	443.733836	612.5681426	591.8355352	300.2899933	277.4392102	467.0865817	356.253762	354.5583263	415.3001814
**ENSG00000108848**	LUC7L3	3417.140684	3772.950784	3334.285034	3415.07607	3165.705929	3019.499375	3798.566275	3922.359134	3183.316985	3687.194798	3992.575082	3519.478727
**ENSG00000108852**	MPP2	140.6230734	175.1602035	140.862347	169.1211979	176.8357091	155.1671371	186.9151999	136.2276362	181.0194985	224.6360311	169.691339	167.4733929
**ENSG00000108854**	SMURF2	2774.493238	1905.743014	2789.56301	2829.012513	2038.811705	3222.847994	876.3565109	945.28689	930.421464	890.9507938	991.9355509	926.1786124
**ENSG00000108861**	DUSP3	1451.230118	1762.111647	1482.718693	1380.598294	1917.453865	1543.324972	1200.138578	1794.217647	1316.377545	1157.350143	1494.111546	1416.757238
**ENSG00000108878**	CACNG1	0	3.50320407	4.885399317	0	0	1.13814526	0	0	0.937924863	0	2.759208765	3.383300867
**ENSG00000108883**	EFTUD2	2095.283794	1727.079607	2289.623813	2422.116958	2249.165293	2405.280316	2019.296995	2048.39848	2192.868329	2113.477025	1965.936245	2161.083429
**ENSG00000108924**	HLF	1.406230734	0	0	1.674467306	0	0	7.149761744	0	1.406887294	2.53111021	0	0
**ENSG00000108932**	SLC16A6	1.406230734	17.51602035	4.071166097	6.697869223	6.93473369	5.311344546	34.72741419	11.62918845	21.10330942	58.84831237	15.17564821	45.67456171
**ENSG00000108946**	PRKAR1A	2749.181085	2718.486359	2439.442725	2679.147689	2903.341838	2748.241421	2286.902364	2364.047881	2327.92951	2312.169176	2116.313122	2595.83759
**ENSG00000108947**	EFNB3	0	3.50320407	0	0.837233653	2.311577897	2.655672273	0	0	0	0	0	0.845825217
**ENSG00000108953**	YWHAE	9416.120996	9406.102929	9674.719113	10820.40773	10372.05002	11020.28117	8768.672082	9677.146105	9475.385928	9095.544538	9891.763421	10045.8661
**ENSG00000108958**	AC130689.1	0	0	1.628466439	2.511700959	0	1.896908767	0	1.661312636	0.468962431	2.53111021	0	0.845825217
**ENSG00000108960**	MMD	412.0256051	560.5126513	476.3264334	439.5476678	609.1007757	613.8396768	478.0126423	388.7471569	523.3620735	566.9686869	449.7510286	680.0434743
**ENSG00000108961**	RANGRF	130.7794583	154.1409791	116.4353504	186.7031046	198.7956991	204.486765	92.94690267	83.06563181	132.2474057	118.3294023	125.5439988	146.3277625
**ENSG00000108963**	DPH1	191.2473798	91.08330583	205.1867713	239.4488247	220.7556891	242.4249404	213.4714578	210.9867048	229.7915914	214.5115903	176.5893609	207.2271781
**ENSG00000108984**	MAP2K6	7.031153671	17.51602035	8.142332194	15.07020575	48.54313583	12.51959786	513.761451	604.7177995	586.2030393	351.1915416	583.5726537	421.220958
**ENSG00000109016**	DHRS7B	255.9339936	238.2178768	294.7524254	209.3084132	221.9114781	226.4909067	152.1877857	181.0830773	147.2542035	176.5449371	129.6828119	198.7689259
**ENSG00000109046**	WSB1	1189.671201	1905.743014	1212.393264	1300.223863	1665.491874	1215.159756	1910.00778	2681.358595	1720.154199	2052.097602	2745.412721	1921.714893
**ENSG00000109062**	SLC9A3R1	199.6847643	161.1473872	250.7838316	324.0094237	279.7009255	299.3322034	282.9262862	224.2772059	298.7290688	285.3826761	256.6064151	281.6597972
**ENSG00000109063**	MYH3	11.24984587	3.50320407	6.513865756	10.04680383	9.246311586	4.173199286	26.55625791	11.62918845	9.848211061	10.12444084	15.17564821	18.60815477
**ENSG00000109065**	NAT9	227.8093789	178.6634076	202.7440716	217.6807497	214.9767444	229.146579	163.4231256	159.4860131	177.7367615	170.2171616	155.8952952	177.6232955
**ENSG00000109066**	TMEM104	209.5283794	164.6505913	309.4086234	301.404115	258.8967244	372.5528817	188.957989	215.9706427	282.7843462	230.3310291	277.3004808	251.2100894
**ENSG00000109079**	TNFAIP1	1364.043812	1579.945036	1634.980305	1269.246218	1148.854215	1516.009486	1282.871536	1390.518676	1503.962518	1095.970721	1149.21045	1349.091221
**ENSG00000109083**	IFT20	165.9352266	196.1794279	181.5740079	165.7722633	187.2378096	165.7898262	235.9421376	182.74439	190.8677096	201.8560392	215.2182836	192.8481494
**ENSG00000109084**	TMEM97	406.4006822	294.2691419	360.7053162	669.7869223	498.1450367	583.8685183	602.6227756	737.6228104	707.1953466	678.3375362	878.8079915	903.3413315
**ENSG00000109089**	CDR2L	89.99876699	52.54806106	94.45105346	176.6563008	191.8609654	190.8290219	83.75435186	151.1794499	141.1576919	125.9227329	182.1077785	156.4776651
**ENSG00000109099**	PMP22	2.812461468	21.01922442	25.2412298	10.88403749	15.02525633	7.587635066	5.106972674	6.645250545	6.096511609	6.960553076	17.93485697	1.691650434
**ENSG00000109101**	FOXN1	0	0	0	0.837233653	0	0	3.064183605	0	6.56547404	3.796665314	4.138813147	1.691650434
**ENSG00000109103**	UNC119	115.3109202	136.6249587	152.261612	144.8414219	177.991498	173.756843	115.4175824	137.8889488	121.4612697	91.7527451	106.2295374	153.0943642
**ENSG00000109107**	ALDOC	112.4984587	451.9133251	104.2218521	75.35102876	255.4293576	82.70522222	100.0966644	450.2157244	120.0543825	117.6966247	375.252392	127.7196077
**ENSG00000109111**	SUPT6H	2015.128642	2294.598666	2557.506542	2242.948956	2202.933735	2348.373053	2144.928523	2176.319553	2023.103929	2064.753153	2091.480244	2018.138967
**ENSG00000109113**	RAB34	88.59253625	105.0961221	88.75142092	80.37443068	84.37259322	103.5712187	51.06972674	36.54887799	57.21341664	73.40219608	45.52694462	61.74524082
**ENSG00000109118**	PHF12	551.2424478	760.1952833	746.6518622	570.1561176	692.31758	657.0891967	758.8961394	867.2051961	762.5329135	870.0691345	742.2271577	676.6601734
**ENSG00000109133**	TMEM33	2137.470716	1481.855322	2134.105268	1748.143867	1659.71293	2031.589289	1476.936497	1295.823856	1630.113412	1432.608379	1157.488077	1552.935098
**ENSG00000109158**	GABRA4	0	0	0	0	0	0	5.106972674	0	3.28273702	1.265555105	0	0
**ENSG00000109163**	GNRHR	0	0	0.814233219	2.511700959	11.55788948	3.41443578	0	0	0	0	0	0
**ENSG00000109171**	SLAIN2	1037.798282	1142.044527	1146.440373	1095.938852	1099.15529	1223.506154	871.2495382	990.1423311	955.7454353	902.9735672	905.0204748	1074.198025
**ENSG00000109180**	OCIAD1	1044.829435	1057.967629	1007.206492	986.2612431	1097.999501	987.1513221	1237.930176	1164.580158	1118.006437	1095.970721	1198.876208	1120.718412
**ENSG00000109184**	DCUN1D4	634.2100611	959.8779153	637.5446108	843.0942884	792.8712185	759.5222701	790.55937	897.1088235	780.3534859	821.345263	813.9665856	903.3413315
**ENSG00000109189**	USP46	298.1209156	318.7915704	306.9659237	345.7774986	399.9029761	360.4126656	352.3811145	392.0697821	421.5972259	477.747052	299.374151	444.0582388
**ENSG00000109220**	CHIC2	350.1514528	483.4421617	242.6414994	313.1253862	253.1177797	371.4147365	181.8082272	348.8756536	186.6470477	158.1943881	255.2268107	228.3728085
**ENSG00000109255**	NMU	37.96822982	77.07048955	43.15436063	96.28187008	232.3135786	226.4909067	28.59904698	24.91968954	53.93067962	36.70109804	49.66575776	98.11572515
**ENSG00000109265**	KIAA1211	5.624922937	0	6.513865756	5.86063557	6.93473369	5.6907263	1.021394535	1.661312636	2.344812157	5.694997971	1.379604382	0.845825217
**ENSG00000109270**	LAMTOR3	427.4941432	430.8941007	393.274645	399.3604524	419.5513882	415.0436381	342.1671692	475.1354139	361.5700347	382.1976416	386.289227	431.3708606
**ENSG00000109320**	NFKB1	1244.5142	1583.44824	1441.192798	903.3751114	1040.210053	1142.318459	845.7146749	918.7058878	925.2628773	761.8641731	880.1875959	855.9751194
**ENSG00000109321**	AREG	10982.66203	10408.01929	11614.22264	11288.42134	6681.61591	12175.49861	330.9318293	202.6801416	449.2660093	323.9821068	206.9406573	537.0990127
**ENSG00000109323**	MANBA	686.2405983	711.1504263	911.9412058	796.2092039	1171.969994	958.3183088	1729.220948	2403.919384	1768.926291	1715.459945	2312.216945	1978.385182
**ENSG00000109332**	UBE2D3	6468.661377	6687.61657	7392.423399	5328.154967	5619.445866	6021.547188	4671.858603	4982.276596	4502.508304	4063.697441	4224.348619	4308.633654
**ENSG00000109339**	MAPK10	516.0866794	371.3396315	361.5195494	607.831632	365.2293077	410.4910571	178.7440436	124.5984477	158.0403394	137.3127289	143.4788558	138.7153356
**ENSG00000109381**	ELF2	734.0524432	1026.438793	819.932852	807.930475	809.0522638	666.5737406	942.7471557	1026.691209	868.9873855	908.0357877	929.8533537	958.3199706
**ENSG00000109390**	NDUFC1	268.5900702	227.7082646	205.1867713	348.2891996	260.0525134	321.7157268	233.8993485	202.6801416	305.7635053	278.422123	219.3570968	298.5763015
**ENSG00000109436**	TBC1D9	168.7476881	245.2242849	198.6729055	86.23506625	115.5788948	101.6743099	156.2733638	157.8247004	151.4748654	130.9849533	113.1275593	128.5654329
**ENSG00000109445**	ZNF330	232.0280711	318.7915704	248.3411319	240.2860584	236.9367344	282.2600245	192.0221726	197.6962037	181.488461	153.1321677	157.2748996	180.1607712
**ENSG00000109452**	INPP4B	2257.000328	2620.396645	2301.023078	2161.737292	2490.725184	2104.809967	240.0277157	483.4419771	253.7086754	234.1276944	466.3062812	285.8889233
**ENSG00000109458**	GAB1	2339.967942	1930.265443	2077.108943	1947.405477	1240.161541	1721.255015	1422.802587	1344.001923	1572.899995	1435.139489	1393.400426	1527.560341
**ENSG00000109466**	KLHL2	573.7421395	609.5575083	563.4493879	604.4826974	567.4923736	543.6540525	416.7289702	423.6347222	342.342575	442.9442867	456.6490505	472.8162962
**ENSG00000109472**	CPE	4.218692202	3.50320407	4.885399317	14.2329721	12.71367843	16.69279715	0	6.645250545	1.875849726	1.265555105	2.759208765	5.074951301
**ENSG00000109475**	RPL34	3351.047839	3082.819582	3142.125994	3246.792106	3131.032261	3136.348955	2020.31839	2756.117663	2338.715646	2140.053682	2348.086659	2365.773131
**ENSG00000109501**	WFS1	209.5283794	245.2242849	284.1673936	398.5232188	428.7976998	492.8168975	273.7337353	259.1647712	347.9701241	318.9198864	415.2609191	327.3343589
**ENSG00000109519**	GRPEL1	476.7122189	374.8428355	542.2793242	534.1550705	478.4966246	606.6314235	328.8890402	272.4552723	350.7838987	358.7848722	262.1248326	350.1716397
**ENSG00000109534**	GAR1	644.0536762	311.7851623	643.2442434	624.576305	402.214554	577.4190285	336.038802	237.567707	366.7286214	365.7454253	333.8642605	395.0003762
**ENSG00000109536**	FRG1	316.4019152	465.9261414	330.5786871	335.7306948	312.063016	293.2620953	346.2527473	405.3602832	302.9497307	375.2370886	380.7708095	307.8803789
**ENSG00000109572**	CLCN3	1223.420739	1320.707935	1344.299045	1282.641956	1188.151039	1076.685416	948.8755229	998.4488943	840.8496396	930.183002	798.7909374	894.0372541
**ENSG00000109576**	AADAT	73.12399818	49.04485699	64.32442434	51.90848648	85.52838217	53.49282722	56.17669942	74.75906863	47.83416801	50.62220419	30.35129641	60.05359039
**ENSG00000109586**	GALNT7	413.4318358	441.4037129	332.2071535	422.8029947	419.5513882	405.5590943	90.9041136	111.3079466	94.26144872	107.5721839	77.25784541	98.96155036

**ENSG00000109606**	DHX15	3792.60429	3026.768317	3744.658576	3518.893043	3059.373346	3530.147214	3284.804824	3217.962576	3454.846233	3384.727128	3142.738783	3860.346289
**ENSG00000109610**	SOD3	14.06230734	3.50320407	16.28466439	25.95424324	24.27156791	20.10723293	57.19809395	53.16200436	87.22701225	53.78609195	70.3598235	64.28271647
**ENSG00000109618**	SEPSECS	136.4043812	119.1089384	144.1192798	118.0499451	117.8904727	121.7815428	370.7662162	275.7778976	323.5840777	325.8804395	328.345843	278.2764963
**ENSG00000109654**	TRIM2	149.0604578	77.07048955	100.9649192	293.0317785	216.1325333	191.9671672	41.87717593	23.25837691	45.95831828	42.39609601	34.49010956	63.43689126
**ENSG00000109667**	SLC2A9	9.843615139	7.006408141	6.513865756	8.372336529	10.40210053	6.449489806	1.021394535	0	3.751699452	0.632777552	2.759208765	0.845825217
**ENSG00000109670**	FBXW7	473.8997574	490.4485699	491.7968645	469.6880793	434.5766445	481.8148267	398.3438686	353.8595915	347.0321993	412.5709642	449.7510286	403.4586284
**ENSG00000109674**	NEIL3	274.2149932	161.1473872	210.8864038	329.8700592	263.5198802	251.9094842	153.2091802	182.74439	190.3987472	192.9971535	219.3570968	219.0687311
**ENSG00000109680**	TBC1D19	23.90592248	42.03844885	21.98429693	36.83828073	52.01050267	20.10723293	118.481766	99.67875817	83.00635037	98.08052062	74.49863664	95.5782495
**ENSG00000109685**	NSD2	3955.727055	3541.739315	4080.936896	3533.126015	3460.432111	3706.180348	3167.344453	3005.314559	3289.302494	3118.327778	3308.291309	3249.660483
**ENSG00000109686**	SH3D19	1071.547819	945.865099	1058.503185	1280.967489	1337.247813	1323.662937	1222.609258	918.7058878	1060.79302	1160.514031	1016.76843	1148.630644
**ENSG00000109689**	STIM2	267.1838395	206.6890402	330.5786871	330.7072929	345.5808955	330.8208889	445.3280172	428.6186601	399.5559916	432.1870683	431.8161717	466.8955197
**ENSG00000109736**	MFSD10	157.4978422	140.1281628	143.3050466	182.5169363	216.1325333	206.7630556	104.1822426	94.69482026	150.0679781	97.44774307	180.7281741	106.5739773
**ENSG00000109738**	GLRB	59.06169083	63.05767327	74.09522297	113.8637768	151.4083522	103.1918369	0	3.322625272	1.406887294	0	1.379604382	3.383300867
**ENSG00000109743**	BST1	32.34330689	45.54165292	74.90945619	48.55955187	55.47786952	68.28871559	17.36370709	33.22625272	13.13094808	19.61610412	13.79604382	17.76232955
**ENSG00000109756**	RAPGEF2	828.2699024	1064.974037	903.7988736	557.5976128	579.0502631	638.8788726	884.5276672	651.2345534	772.3811246	737.8186261	629.0995983	641.1355143
**ENSG00000109758**	HGFAC	0	0	0.814233219	5.023401917	1.155788948	0.379381753	0	0	0.468962431	0	5.518417529	0
**ENSG00000109762**	SNX25	217.9657638	630.5767327	319.9936552	229.4020209	473.8734688	279.6043522	115.4175824	320.6333388	87.22701225	149.9682799	206.9406573	138.7153356
**ENSG00000109771**	LRP2BP	8.437384405	17.51602035	12.21349829	14.2329721	23.11577897	8.725780326	8.171156279	13.29050109	11.25509835	13.2883286	11.03683506	1.691650434
**ENSG00000109775**	UFSP2	203.9034565	203.1858361	192.973273	220.1924507	257.7409355	168.4454985	132.7812895	199.3575163	176.3298742	149.3355024	194.5242179	162.3984416
**ENSG00000109787**	KLF3	3325.735686	3821.995641	3158.410658	3081.019843	3099.825959	3037.330317	2014.190023	2385.644945	2151.130673	1769.878814	1712.089038	1895.494311
**ENSG00000109790**	KLHL5	314.9956845	648.092753	469.8125676	259.5424324	499.3008257	562.6231402	28.59904698	24.91968954	22.51019671	15.81943881	23.4532745	24.52893129
**ENSG00000109794**	FAM149A	4.218692202	0	0	13.39573845	0	4.55258104	14.29952349	4.983937908	7.503398903	18.35054902	33.11050518	3.383300867
**ENSG00000109805**	NCAPG	1882.942953	1457.332893	1608.110608	2178.481965	1999.51488	1915.119091	1754.755811	1554.988627	1791.905451	1878.716553	1785.208071	2079.038383
**ENSG00000109814**	UGDH	715.7714437	616.5639164	764.5649931	432.0125649	358.294574	402.5240403	370.7662162	455.1996623	431.9143994	369.5420906	378.0116008	445.7498892
**ENSG00000109819**	PPARGC1A	0	0	2.442699658	0	0	0	3.064183605	0	3.28273702	12.65555105	1.379604382	10.99572782
**ENSG00000109832**	DDX25	0	0	0	0	2.311577897	3.035054026	1.021394535	0	0	1.265555105	0	0
**ENSG00000109846**	CRYAB	220.7782253	115.6057343	130.2773151	457.1295745	151.4083522	310.713656	1958.013323	3492.079161	2672.147934	1922.378204	2939.936939	2761.619333
**ENSG00000109854**	HTATIP2	1320.450659	1362.746383	1320.686282	967.0048691	1232.071019	1018.260626	1741.477682	1814.153399	1626.830675	1406.664499	1590.683853	1413.373937
**ENSG00000109861**	CTSC	1459.667502	1558.925811	2038.025748	977.0516729	993.9784955	1262.203093	65.36925023	56.48462963	67.99955256	83.52663691	52.42496653	66.82019213
**ENSG00000109881**	CCDC34	364.2137601	287.2627338	333.0213868	605.319931	475.0292577	421.8725097	832.4365459	893.7861982	892.9044695	1009.912974	979.5191114	1135.943266
**ENSG00000109906**	ZBTB16	0	0	0	0	0	0	5.106972674	0	0.937924863	11.38999594	2.759208765	1.691650434
**ENSG00000109911**	ELP4	409.2131436	469.4293454	390.0177121	401.0349197	481.9639914	400.6271315	623.0506663	566.5076089	569.7893542	604.9353401	648.4140597	675.8143482
**ENSG00000109917**	ZPR1	1011.079898	770.7048955	1111.428345	990.4474113	795.1827964	973.493579	623.0506663	593.0886111	712.8228958	650.4953238	589.0910712	678.3518239
**ENSG00000109919**	MTCH2	1465.292425	1369.752792	1312.54395	1785.819382	1718.658166	1601.749762	1318.620345	1493.52006	1363.742751	1467.411144	1713.468643	1671.350628
**ENSG00000109920**	FNBP4	3175.268998	2792.053644	3173.881089	1848.611906	1346.494125	1689.386947	2686.267627	1890.57378	2229.447399	2275.468078	1706.570621	1991.918386
**ENSG00000109927**	TECTA	29.53084542	38.53524477	18.72736405	14.2329721	17.33683422	7.587635066	15.32091802	3.322625272	13.13094808	14.5538837	17.93485697	8.458252168
**ENSG00000109929**	SC5D	826.8636717	1250.643853	707.5686677	895.8400086	960.460616	805.806844	517.8470292	936.9803268	524.2999984	491.6681582	786.3744979	626.7564856
**ENSG00000109943**	CRTAM	0	0	1.628466439	0	0	1.13814526	5.106972674	0	1.406887294	0.632777552	1.379604382	0.845825217
**ENSG00000109944**	JHY	367.0262216	469.4293454	411.1877758	416.1051255	438.0440114	362.6889562	151.1663912	104.6626961	86.28908739	106.9394064	144.8584601	91.34912341
**ENSG00000109971**	HSPA8	27607.12177	24102.044	33563.50754	26816.5939	28774.52166	35211.17929	11546.86522	10587.54543	16268.30675	11351.39651	10500.16895	18865.28563
**ENSG00000110002**	VWA5A	1279.669968	1478.352118	1188.7805	982.9123085	1006.692174	1132.833915	404.4722358	515.0069172	414.093827	473.9503867	615.3035545	484.6578492
**ENSG00000110011**	DNAJC4	71.71776744	94.5865099	52.92515926	71.16486049	88.99574902	73.97944189	125.6315278	136.2276362	127.0888189	98.08052062	229.0143275	85.42834689
**ENSG00000110013**	SIAE	22.49969175	70.06408141	35.82626166	8.372336529	19.64841212	22.00414169	79.66877372	91.37219499	82.0684255	132.2505084	147.6176689	91.34912341
**ENSG00000110025**	SNX15	15.46853808	14.01281628	17.09889761	10.04680383	11.55788948	14.03712487	10.21394535	26.58100218	13.13094808	11.38999594	6.898021912	5.920776517
**ENSG00000110031**	LPXN	29.53084542	91.08330583	77.35215585	62.79252397	60.10102551	50.07839144	69.45482837	54.82331699	54.86860448	86.05774712	99.33151553	68.51184256
**ENSG00000110042**	DTX4	130.7794583	80.57369362	85.49448804	176.6563008	84.37259322	133.1629954	1149.068852	838.9628812	1307.936221	1261.125662	1081.609836	1487.806556
**ENSG00000110046**	ATG2A	345.9327606	252.2306931	311.0370898	281.3105074	241.5598902	310.3342742	325.8248566	264.1487091	331.556439	296.1398945	271.7820633	293.5013502
**ENSG00000110047**	EHD1	1001.236283	777.7113036	1006.392259	1116.869693	872.6206559	1263.341239	983.6029371	1189.499847	1168.654379	884.6230182	910.5388923	961.7032715
**ENSG00000110048**	OSBP	1394.980888	1481.855322	1599.968276	1306.084498	1503.681422	1482.623892	1422.802587	1456.971182	1412.045881	1347.183409	1414.094492	1473.427528
**ENSG00000110057**	UNC93B1	150.4666886	178.6634076	99.33645277	96.28187008	158.3430859	110.4000902	62.30506663	106.3240087	96.13729845	77.83163894	81.39665856	87.11999733
**ENSG00000110060**	PUS3	323.4330689	385.3524477	323.2505881	216.0062824	260.0525134	231.8022513	164.4445201	182.74439	152.4127902	119.5949574	102.0907243	143.7902869
**ENSG00000110063**	DCPS	431.7128354	231.2114686	437.2432388	483.0838177	466.9387351	479.5385362	345.2313528	259.1647712	392.0525927	383.4631967	339.382678	452.516491
**ENSG00000110066**	KMT5B	556.8673707	381.8492437	412.002009	662.2518194	569.8039515	536.8251809	383.0229506	353.8595915	403.307691	382.1976416	390.4280402	482.9661988
**ENSG00000110074**	FOXRED1	525.9302946	637.5831408	585.4336848	657.2284175	606.7891978	635.8438185	567.8953614	594.7499237	559.9411432	580.2570155	645.6548509	543.8656144
**ENSG00000110075**	PPP6R3	3090.895154	3412.120765	3034.647209	3262.699545	3052.438612	3284.307838	3162.23748	2962.12043	3040.283443	3241.086623	2944.075752	3165.077961
**ENSG00000110076**	NRXN2	11.24984587	0	3.256932878	37.67551438	15.02525633	8.725780326	29.62044151	16.61312636	16.88264753	41.1305409	27.59208765	27.91223215
**ENSG00000110080**	ST3GAL4	420.4629895	409.8748762	460.041769	701.6018011	759.353339	708.3057334	510.6972674	423.6347222	552.4377443	630.2464422	593.2298844	628.4481361
**ENSG00000110090**	CPT1A	2951.678311	2028.355157	2953.223887	2650.681745	2778.516632	3652.687521	1262.443645	176.0991394	1251.191767	1261.758439	286.9577115	1222.217438
**ENSG00000110092**	CCND1	10847.66388	7910.234791	10703.9099	8719.788495	6071.359345	9739.867753	1529.027619	1211.096912	1756.733268	823.8763732	732.569927	1009.915309
**ENSG00000110104**	CCDC86	921.0811309	620.0671205	920.8977712	1126.916497	857.5953996	1221.229864	417.7503648	327.2785893	577.2927531	515.0809276	378.0116008	668.2019213
**ENSG00000110107**	PRPF19	1314.825736	1005.419568	1241.70566	1339.573845	1145.386848	1316.075302	1363.561704	1162.918845	1494.583269	1548.406671	1335.457042	1585.922281
**ENSG00000110108**	TMEM109	544.2112941	486.9453658	590.3190841	737.6028482	604.4776199	773.9387767	579.1307013	471.8127887	768.6294252	703.6486382	522.8700609	797.6131794
**ENSG00000110169**	HPX	4.218692202	7.006408141	7.328098975	2.511700959	3.467366845	4.55258104	10.21394535	3.322625272	0.937924863	4.429442867	1.379604382	0.845825217
**ENSG00000110171**	TRIM3	132.185689	241.7210809	161.2181775	174.1445998	228.8462118	182.8620051	138.9096567	146.195512	119.58542	148.0699473	183.4873828	142.0986364
**ENSG00000110172**	CHORDC1	2587.464551	1933.768647	2680.455758	2287.32234	2228.361092	2501.643281	2121.436449	1790.895022	2258.054107	1929.338757	1856.947499	2582.304387
**ENSG00000110195**	FOLR1	66.09284451	42.03844885	98.52221955	113.8637768	116.7346838	98.25987411	123.5887387	196.0348911	171.6402499	166.4204963	191.7650091	203.8438772
**ENSG00000110200**	ANAPC15	209.5283794	213.6954483	210.8864038	341.5913304	306.2840713	267.0847543	206.321696	202.6801416	226.039892	311.3265558	231.7735362	268.1265937

**ENSG00000110203**	FOLR3	26.71838395	10.50961221	20.35583049	23.44254228	12.71367843	41.35261111	6.128367209	13.29050109	7.034436472	14.5538837	6.898021912	16.07067912
**ENSG00000110218**	PANX1	1392.168427	1621.983485	1399.666904	984.5867758	1222.824707	1139.283405	769.1100848	1706.168077	801.4567954	720.7336322	1512.046403	726.5638612
**ENSG00000110237**	ARHGEF17	241.8716863	115.6057343	165.2893435	279.6360401	210.3535886	289.088896	317.6537003	363.8274673	330.1495517	324.6148844	408.3628972	294.3471754
**ENSG00000110274**	CEP164	509.0555258	476.4357536	555.3070557	609.5060993	553.6229062	629.7737105	506.6116893	446.8930991	496.6312149	566.9686869	509.0740171	499.8827031
**ENSG00000110315**	RNF141	1466.698656	1383.765608	1353.255611	1532.137585	1339.559391	1308.867049	1511.663912	2953.813867	1318.253395	1407.297276	2924.76129	1372.774327
**ENSG00000110318**	CEP126	170.1539188	157.6441832	122.9492161	205.122245	230.0020007	160.8578634	678.2059712	534.9426688	481.1554547	709.9764138	590.4706756	563.3195944
**ENSG00000110321**	EIF4G2	19545.20097	18580.99439	19712.58624	23944.88247	22659.24233	23865.38857	21951.81134	21161.80036	23512.83839	22207.96098	21839.13737	25936.38445
**ENSG00000110324**	IL10RA	1.406230734	3.50320407	5.699632536	0.837233653	2.311577897	1.896908767	3.064183605	16.61312636	3.751699452	3.796665314	16.55525259	3.383300867
**ENSG00000110328**	GALNT18	0	7.006408141	5.699632536	0.837233653	3.467366845	2.655672273	835.5007295	1628.086383	840.3806772	923.2224489	1474.797085	783.2341507
**ENSG00000110330**	BIRC2	3119.019768	3352.566295	3080.244269	2256.344694	2584.344088	2616.975334	2186.805699	2634.841841	2335.901871	1704.702726	1979.732289	1858.278001
**ENSG00000110344**	UBE4A	2816.680161	2963.710644	3045.232241	3248.466573	3084.800703	3321.48725	2277.709813	2196.255305	2170.827095	2327.355838	2385.335977	2413.985169
**ENSG00000110367**	DDX6	7050.840901	6466.914714	6930.753164	7818.08785	7972.632165	7534.901002	5494.081203	5342.781438	5582.528784	5580.465234	5459.094541	6029.88797
**ENSG00000110375**	UPK2	0	3.50320407	1.628466439	0.837233653	3.467366845	6.449489806	11.23533988	3.322625272	7.503398903	12.65555105	2.759208765	14.37902869
**ENSG00000110395**	CBL	2244.344252	2059.883993	2330.335474	2605.471128	2620.173546	2618.492861	1354.369153	1629.747696	1543.355362	1346.550631	1609.998314	1570.697428
**ENSG00000110400**	NECTIN1	2733.712547	3937.601375	2469.569355	2956.272028	4661.296828	2510.748443	1023.437324	822.3497549	1291.053574	1240.244003	1165.765703	1598.60966
**ENSG00000110422**	HIPK3	2057.315564	2476.765278	2252.169085	2072.153291	2441.026259	2510.369062	2647.454634	3596.741857	2841.912335	2677.281824	3174.469684	3099.103594
**ENSG00000110427**	KIAA1549L	1040.610743	1173.573364	1190.408967	1482.740799	1593.83296	1632.100303	26.55625791	14.95181373	21.57227185	29.74054496	16.55525259	35.5246591
**ENSG00000110429**	FBXO3	1134.828202	1324.211139	1320.686282	2050.385216	1579.963492	1908.669601	1445.273267	1473.584308	1493.645344	1477.535585	1448.584601	1659.509075
**ENSG00000110435**	PDHX	548.4299863	690.1312019	622.0741797	735.9283809	673.8249568	754.2109256	793.6235536	780.816939	770.0363124	772.6213915	753.2639927	892.3456037
**ENSG00000110436**	SLC1A2	2.812461468	0	1.628466439	2.511700959	0	1.13814526	0	3.322625272	0.468962431	4.429442867	0	1.691650434
**ENSG00000110442**	COMMD9	258.7464551	301.2755501	274.396595	298.8924141	293.5703929	309.5755107	389.1513178	413.6668464	388.7698557	383.4631967	437.3345892	401.766978
**ENSG00000110446**	SLC15A3	687.646829	865.2914054	574.0344197	25.95424324	83.21680428	72.84129663	270.6695517	345.5530283	293.5704821	24.04554699	49.66575776	36.37048432
**ENSG00000110455**	ACCS	157.4978422	154.1409791	131.0915483	85.39783259	130.6041512	80.4289317	122.5673442	94.69482026	105.5165471	130.9849533	115.8867681	76.97009473
**ENSG00000110492**	MDK	18.28099954	49.04485699	24.42699658	5.86063557	11.55788948	3.41443578	56.17669942	64.79119281	58.62030393	6.327775524	24.83287888	19.45397999
**ENSG00000110497**	AMBRA1	561.0860629	588.5382838	670.9281728	465.501911	477.3408356	578.5571738	750.7249831	734.3001852	778.0086738	739.7169587	641.5160378	679.1976491
**ENSG00000110514**	MADD	727.0212896	861.7882013	863.0872126	792.8602693	902.6711686	920.0007518	988.7099098	878.8343845	976.3797823	908.0357877	1011.250012	913.4912341
**ENSG00000110536**	PTPMT1	119.5296124	94.5865099	79.79485551	135.6318518	109.7999501	114.5732895	123.5887387	136.2276362	124.7440068	160.0927208	132.4420207	140.406986
**ENSG00000110583**	NAA40	648.2723684	493.9517739	676.6278054	673.9730906	674.9807458	701.4768619	619.9864827	584.7820479	718.450445	715.6714117	738.0883445	724.0263856
**ENSG00000110619**	CARS	1863.255723	1727.079607	2219.599756	1248.315376	1325.689924	1700.389018	1748.627444	1540.036814	1674.19588	1332.629525	1323.040603	1188.38443
**ENSG00000110628**	SLC22A18	33.74953762	66.56087734	44.78282707	41.86168264	43.91998003	35.66188481	35.74880872	54.82331699	49.2410553	43.02887356	81.39665856	34.67883389
**ENSG00000110651**	CD81	1182.640047	1173.573364	1009.649192	1247.478143	1233.226808	1333.147481	1071.442867	1221.064788	1333.260193	1221.893454	1323.040603	1376.157628
**ENSG00000110660**	SLC35F2	1271.232584	805.7369362	1297.073519	1651.024763	1509.460366	1858.211828	812.0086552	732.6388725	1040.158673	948.533551	903.6408704	1155.397246
**ENSG00000110675**	ELMOD1	0	24.52242849	2.442699658	0	1.155788948	0	0	4.983937908	0	0.632777552	0	2.53747565
**ENSG00000110693**	SOX6	0	0	1.628466439	1.674467306	2.311577897	1.517527013	14.29952349	23.25837691	38.92388181	22.14721433	20.69406573	40.59961041
**ENSG00000110696**	C11orf58	2493.247092	2221.031381	2295.323446	2452.257369	2521.931485	2456.117471	3153.044929	2809.279668	2907.098113	2935.455065	2684.710128	3000.142044
**ENSG00000110697**	PITPNM1	351.5576835	297.772346	258.1119306	356.6615361	345.5808955	341.0641962	314.5895167	230.9224564	303.8876556	315.7559986	364.2155569	347.6341641
**ENSG00000110700**	RPS13	4367.75266	4252.889741	4186.787214	4318.451181	3892.697178	4101.496135	4875.116115	5651.785588	5504.212058	4843.279386	4818.958107	5538.463519
**ENSG00000110711**	AIP	250.3090707	185.6698157	268.6969624	325.683891	276.2335586	314.1280917	179.7654381	174.4378268	210.0951693	188.5677106	208.3202617	257.9766911
**ENSG00000110713**	NUP98	2858.867083	2431.223625	2820.503872	3203.255956	2705.701928	3192.497454	3830.229506	3264.47933	3845.022975	3780.213098	3305.5321	4292.562975
**ENSG00000110717**	NDUFS8	355.7763757	413.3780803	420.1443412	508.2008273	498.1450367	594.4912074	407.5364194	312.3267756	512.1069751	517.6120378	453.8898418	489.7328005
**ENSG00000110719**	TCIRG1	410.6193744	406.3716722	376.9899806	213.4945815	231.1577897	236.7342141	221.6426141	199.3575163	214.7847936	194.2627086	144.8584601	176.7774703
**ENSG00000110721**	CHKA	338.9016069	213.6954483	267.068496	395.1742842	438.0440114	407.456003	260.4556064	260.8260839	275.7499097	290.4448965	249.7083932	290.9638746
**ENSG00000110723**	EXPH5	1589.04073	662.1055693	1012.906125	3310.421863	1781.070769	2462.946342	2748.572693	2181.303491	2658.079061	3036.699474	2199.089385	3368.921838
**ENSG00000110756**	HPS5	2692.931856	3412.120765	2923.097258	2111.503273	2677.962993	2580.175304	2398.234368	3345.883649	2370.605091	1943.892641	3093.073025	2067.19683
**ENSG00000110768**	GTF2H1	1378.106119	1310.198322	1540.529251	1479.391865	1443.580396	1596.059036	1464.679763	1744.378268	1516.155541	1425.015048	1554.814139	1574.080728
**ENSG00000110777**	POU2AF1	2.812461468	0	0	0	0	0.379381753	6.128367209	18.274439	5.627549177	21.51443678	15.17564821	7.612426951
**ENSG00000110799**	VWF	8.437384405	0	8.956565414	11.72127114	0	4.931962793	0	0	0.937924863	0.632777552	2.759208765	0.845825217
**ENSG00000110801**	PSMD9	85.78007478	56.05126513	93.63682024	82.04889798	72.81470374	76.25573241	122.5673442	83.06563181	115.8337206	109.4705166	135.2012295	109.111453
**ENSG00000110811**	P3H3	37.96822982	14.01281628	26.05546302	46.88508456	46.23155793	53.87220897	2.04278907	1.661312636	1.406887294	0.632777552	0	0
**ENSG00000110841**	PPFIBP1	412.0256051	486.9453658	381.8753799	515.7359302	529.3513383	526.5818736	1198.095789	1437.03543	1105.344451	1381.986174	1803.142928	1183.309478
**ENSG00000110844**	PRPF40B	220.7782253	175.1602035	199.4871388	216.8435161	280.8567144	217.3857446	333.9960129	292.391024	318.8944534	344.2309885	281.439294	328.1801841
**ENSG00000110848**	CD69	0	3.50320407	1.628466439	0	1.155788948	1.13814526	24.51346884	38.21019063	40.79973154	27.8422123	23.4532745	45.67456171
**ENSG00000110851**	PRDM4	795.9265955	634.0799367	844.3598486	646.34438	647.241811	752.3140168	1059.186133	998.4488943	952.4626983	959.923547	1052.638144	910.9537585
**ENSG00000110852**	CLEC2B	0	0	2.442699658	0	2.311577897	0	6.128367209	28.24231481	8.910286198	0.632777552	5.518417529	3.383300867
**ENSG00000110871**	COQ5	464.0561423	542.9966309	509.7099954	473.8742475	463.4713683	430.2189082	652.6711078	551.5557952	657.4853289	637.2069952	704.9778394	668.2019213
**ENSG00000110876**	SELPLG	49.2180757	42.03844885	46.41129351	15.07020575	10.40210053	3.035054026	0	21.59706427	5.627549177	2.53111021	0	2.53747565
**ENSG00000110880**	CORO1C	3936.039825	3804.47962	3966.130012	3939.184337	3416.512131	3773.330918	2498.331032	4483.882805	2803.926378	2557.686867	3867.031084	2874.959912
**ENSG00000110881**	ASIC1	36.56199909	35.0320407	30.94086234	66.14145858	65.87997005	34.9031213	14.29952349	18.274439	20.16538455	27.20943475	46.906549	21.14563042
**ENSG00000110888**	CAPRIN2	175.7788418	126.1153465	139.2338805	149.8648239	157.187297	111.1588537	153.2091802	229.2611438	184.3022356	193.629931	242.8103713	195.3856251
**ENSG00000110906**	KCTD10	815.6138258	788.2209158	797.1343218	758.5336895	826.389098	869.5429786	908.0197415	923.6898257	831.0014285	928.2846693	1124.377572	950.7075437
**ENSG00000110911**	SLC11A2	1473.729809	1107.012486	1437.121632	1570.650333	1258.654165	1495.14349	4202.017116	2854.135109	3402.32244	3896.644168	2953.732983	3865.421241
**ENSG00000110917**	MLEC	2971.365541	3107.34201	3226.806249	2919.433748	3533.246815	3490.691512	4494.135953	4138.329777	4651.638358	4387.046771	4370.586683	4926.086063
**ENSG00000110921**	MVK	157.4978422	133.1217547	166.1035768	266.2403016	336.3345839	266.7053726	366.680638	671.170305	393.9284424	327.7787721	691.1817955	361.1673676
**ENSG00000110925**	CSRNP2	520.3053716	472.9325495	609.8606814	574.3422859	545.5323836	600.9406972	634.2860062	744.268061	669.6783521	588.4831237	724.2923007	636.9063882
**ENSG00000110931**	CAMKK2	655.3035221	585.0350798	715.7109999	687.368829	663.4228563	616.1159674	912.1053196	772.5103758	954.8075104	933.3468898	793.2725198	926.1786124
**ENSG00000110934**	BIN2	4.218692202	0	4.071166097	21.76807497	3.467366845	14.79588838	22.47067977	14.95181373	11.25509835	6.960553076	6.898021912	16.91650434
**ENSG00000110944**	IL23A	60.46792157	105.0961221	106.6645517	18.41914036	30.05051265	73.60006014	1.021394535	13.29050109	7.972361335	3.163887762	2.759208765	0.845825217

**ENSG00000110955**	ATP5F1B	3137.300768	2504.79091	2962.994686	3800.20355	3496.261568	3690.625696	4018.1661	3350.867587	4176.579415	4720.520541	3726.311437	4794.983154
**ENSG00000110958**	PTGES3	6038.354772	5587.610492	5655.663942	5431.97194	5505.022761	5192.598057	5791.307013	5321.184374	5986.305437	5507.695816	5264.570323	6802.126393
**ENSG00000110975**	SYT10	0	0	0	0	0	0.379381753	6.128367209	14.95181373	0	0	8.277626294	3.383300867
**ENSG00000110987**	BCL7A	357.1826065	392.3588559	447.8282707	510.7125282	436.8882224	495.4725698	404.4722358	353.8595915	488.6588536	456.2326153	420.7793366	489.7328005
**ENSG00000111011**	RSRC2	1365.450043	1443.320077	1481.904459	1335.387676	1257.498376	1214.021611	2015.211417	2211.207119	1767.519404	1988.819847	1982.491497	2050.280325
**ENSG00000111012**	CYP27B1	36.56199909	59.5544692	68.39559043	18.41914036	11.55788948	26.17734098	4.08557814	9.967875817	12.19302322	7.593330629	1.379604382	6.766601734
**ENSG00000111052**	LIN7A	0	0	0.814233219	0	0	1.13814526	10.21394535	11.62918845	2.813774589	8.858885733	5.518417529	1.691650434
**ENSG00000111057**	KRT18	22525.0039	19323.67365	22466.32299	22126.41098	20002.08354	23062.2374	19178.72518	12612.68553	20228.22552	18398.00734	12216.39681	19404.92212
**ENSG00000111058**	ACSS3	2.812461468	0	8.956565414	2.511700959	5.778944741	2.655672273	60.26227756	23.25837691	33.76529506	48.09109398	17.93485697	28.75805737
**ENSG00000111077**	TNS2	40.78069129	17.51602035	21.98429693	45.21061725	24.27156791	20.10723293	18.38510163	23.25837691	21.57227185	32.90443272	35.86971394	23.68310607
**ENSG00000111110**	PPM1H	15.46853808	14.01281628	28.49816268	17.58190671	26.58314581	55.01035423	10.21394535	61.46856754	75.03398903	29.10776741	55.18417529	98.96155036
**ENSG00000111142**	METAP2	1144.671818	889.8138339	1240.077193	1370.55149	1059.858466	1367.671221	1315.556161	1137.999156	1317.784432	1401.602279	1295.448515	1402.378209
**ENSG00000111144**	LTA4H	696.0842134	651.5959571	621.2599464	657.2284175	624.1260321	612.7015316	686.3771274	684.4608061	587.6099266	637.8397728	858.1139258	727.4096864
**ENSG00000111145**	ELK3	1870.286876	1940.775055	1924.847331	1397.342967	1306.041512	1475.79502	289.0546534	320.6333388	348.908049	264.5010169	325.5866342	390.7712502
**ENSG00000111181**	SLC6A12	1.406230734	0	0.814233219	4.186168264	0	0	0	0	0.468962431	0	1.379604382	0.845825217
**ENSG00000111186**	WNT5B	5.624922937	10.50961221	6.513865756	1.674467306	0	4.173199286	0	0	0.468962431	0	0	0
**ENSG00000111196**	MAGOHB	329.0579918	297.772346	255.6692309	334.0562275	231.1577897	253.4270112	385.0657396	270.7939597	404.7145783	372.0732008	300.7537553	380.6213475
**ENSG00000111199**	TRPV4	4.218692202	7.006408141	17.09889761	10.88403749	35.8294574	17.0721789	4.08557814	11.62918845	7.972361335	11.38999594	31.73090079	15.2248539
**ENSG00000111203**	ITFG2	119.5296124	115.6057343	113.9926507	120.561646	123.6694175	104.7093639	235.9421376	259.1647712	243.3915019	247.416023	230.3939318	225.8353329
**ENSG00000111206**	FOXM1	826.8636717	539.4934268	752.3514948	941.8878595	906.1385354	1049.36993	1184.81766	1315.759608	1321.536132	1258.594552	1335.457042	1315.258212
**ENSG00000111215**	PRR4	0	0	3.256932878	6.697869223	10.40210053	4.173199286	15.32091802	18.274439	1.875849726	10.75721839	6.898021912	4.229126084
**ENSG00000111224**	PARP11	179.997534	133.1217547	127.8346155	72.00209415	26.58314581	61.08046228	10.21394535	18.274439	50.6479426	4.429442867	11.03683506	23.68310607
**ENSG00000111229**	ARPC3	1638.258805	1975.807096	1660.221534	1559.766295	1637.75294	1446.582625	1994.783527	2360.725256	2082.662158	2052.73038	2105.276287	2213.524592
**ENSG00000111231**	GPN3	278.4336854	325.7979785	270.3254289	337.4051621	324.7766945	336.8909969	282.9262862	317.3107135	369.0734336	348.6604314	270.4024589	425.450084
**ENSG00000111237**	VPS29	1081.391435	1191.089384	1078.859016	985.4240094	980.1090281	1070.615308	1407.481669	1373.90555	1332.322268	1306.052868	1185.080164	1508.106362
**ENSG00000111245**	MYL2	2.812461468	3.50320407	1.628466439	0.837233653	26.58314581	11.00207085	0	0	0	0	0	0
**ENSG00000111247**	RAD51AP1	316.4019152	192.6762239	214.9575699	312.2881525	255.4293576	294.7796223	376.8945834	481.7806645	405.1835408	434.0854009	478.7227207	539.6364883
**ENSG00000111252**	SH2B3	219.3719945	171.6569995	241.013033	150.7020575	114.4231059	112.6763807	60.26227756	34.88756536	60.02719123	42.39609601	24.83287888	44.82873649
**ENSG00000111254**	AKAP3	2.812461468	7.006408141	10.58503185	3.348934611	12.71367843	3.035054026	14.29952349	6.645250545	7.972361335	10.75721839	13.79604382	5.920776517
**ENSG00000111261**	MANSC1	120.9358431	154.1409791	113.1784175	160.7488614	182.6146538	173.756843	287.0118643	227.5998311	241.9846146	354.9882069	311.7905904	346.7883389
**ENSG00000111266**	DUSP16	112.4984587	143.6313669	113.1784175	97.95633739	136.3830959	123.2990698	702.71944	769.1877505	624.1889963	611.2631156	664.9693123	597.9984283
**ENSG00000111269**	CREBL2	161.7165344	157.6441832	131.9057816	105.4914403	132.9157291	146.061975	325.8248566	370.4727179	293.1015197	287.9137863	328.345843	259.6683416
**ENSG00000111271**	ACAD10	223.5906867	308.2819582	306.9659237	210.9828805	248.4946239	221.1795622	545.4246816	546.5718573	479.2796049	504.3237092	542.1845222	384.0046484
**ENSG00000111275**	ALDH2	0	0	0.814233219	1.674467306	0	1.517527013	2.04278907	0	0.468962431	0	2.759208765	1.691650434
**ENSG00000111276**	CDKN1B	464.0561423	427.3908966	569.9632536	404.3838543	418.3955993	501.1632961	693.5268892	651.2345534	746.1192284	616.9581136	565.6377967	680.0434743
**ENSG00000111291**	GPRC5D	0	0	0.814233219	0.837233653	0	7.208253313	0	0	0.468962431	0	0	0
**ENSG00000111300**	NAA25	1712.789034	1089.496466	1747.344489	1555.580127	1241.31733	1600.611617	2002.954683	1438.696743	1866.470477	1912.253763	1632.071984	1927.635669
**ENSG00000111319**	SCNN1A	732.6462125	1008.922772	671.742406	596.1103608	655.3323337	477.2622457	37.79159779	43.19412854	30.01359561	58.21553482	62.0821972	27.06640694
**ENSG00000111321**	LTBR	656.7097529	465.9261414	731.1814311	663.9262867	564.0250068	740.9325642	659.8208695	428.6186601	765.3466881	779.5819445	467.6858856	750.2469673
**ENSG00000111325**	OGFOD2	19.68723028	3.50320407	19.54159727	10.88403749	24.27156791	22.38352344	16.34231256	21.59706427	29.07567075	41.76331846	28.97169203	30.4497078
**ENSG00000111328**	CDK2AP1	779.0518267	865.2914054	723.0390989	1013.05272	987.0437618	956.0420183	1389.096567	2121.496236	1493.176382	1457.286703	1997.667146	1720.408491
**ENSG00000111331**	OAS3	5661.484936	9276.484378	6529.336187	1152.87074	1763.733935	1501.972361	4292.92123	5479.009074	4679.776103	1574.35055	1792.106093	1437.902869
**ENSG00000111335**	OAS2	3408.7033	5405.443881	3216.221217	35.16381342	202.2630659	59.56293527	852.8644366	1209.435599	855.8564374	28.47498986	53.80457091	22.83728085
**ENSG00000111339**	ART4	146.2479964	147.134571	140.862347	152.3765248	216.1325333	215.4888359	425.921521	505.0390414	341.8736125	344.863766	371.1135788	298.5763015
**ENSG00000111341**	MGP	0	0	0	0	1.155788948	0	0	0	0	1.265555105	0	8.458252168
**ENSG00000111344**	RASAL1	64.68661377	31.52883663	52.92515926	12.55850479	16.18104528	17.45156065	32.68462512	31.56494009	22.97915914	22.14721433	12.41643944	10.1499026
**ENSG00000111348**	ARHGDIB	2122.002178	1643.002709	1694.41933	2501.654155	2046.902227	2035.762488	2485.052903	1810.830773	2326.05366	2426.701913	1582.406227	2541.704776
**ENSG00000111358**	GTF2H3	641.2412148	479.9389576	512.9669283	532.4806032	493.5218809	450.7055229	901.8913743	637.9440523	783.1672605	774.5197241	634.6180159	753.6302681
**ENSG00000111361**	EIF2B1	750.927212	535.9902228	772.7073253	830.5357836	661.1112784	769.7655775	1249.165516	1051.610899	1179.440515	1254.165109	1117.47955	1297.495883
**ENSG00000111364**	DDX55	624.366446	641.0863449	605.7895153	547.550809	495.8334588	472.3302829	868.1853546	732.6388725	847.4151136	750.4741771	786.3744979	876.2749246
**ENSG00000111371**	SLC38A1	7834.11142	6071.052654	6991.820655	7947.021833	7127.750444	7577.012377	8606.270351	8899.651792	8412.248095	8385.568124	8363.161766	8739.06614
**ENSG00000111405**	ENDOU	15.46853808	3.50320407	14.65619795	9.209570182	1.155788948	4.55258104	9.192550814	3.322625272	3.751699452	4.429442867	2.759208765	1.691650434
**ENSG00000111412**	C12orf49	646.8661377	381.8492437	681.5132047	991.284645	863.3743444	1061.889527	1263.46504	1174.548034	1336.54293	1395.907281	1356.151108	1598.60966
**ENSG00000111424**	VDR	322.0268381	521.9774065	382.6896131	477.2231821	639.1512884	566.0375759	147.080813	186.0670152	109.737209	194.2627086	209.6998661	92.19494863
**ENSG00000111432**	FZD10	0	0	0.814233219	0	0	1.517527013	3.064183605	9.967875817	2.813774589	2.53111021	20.69406573	5.074951301
**ENSG00000111445**	RFC5	755.1459042	679.6215897	614.7460807	781.1389981	768.5996506	725.7572941	1304.320821	1216.08085	1265.729602	1321.239529	1420.992514	1431.982092
**ENSG00000111450**	STX2	75.93645964	52.54806106	79.79485551	51.90848648	57.78944741	66.39180683	7.149761744	18.274439	13.13094808	13.92110615	9.657230676	13.53320347
**ENSG00000111452**	ADGRD1	50.62430643	63.05767327	38.26896131	20.93084132	34.67366845	32.62683078	37.79159779	23.25837691	45.02039342	27.8422123	20.69406573	28.75805737
**ENSG00000111481**	COPZ1	1542.635115	1313.701526	1531.572686	1563.952464	1497.902477	1504.248652	1477.957892	1458.632495	1475.355809	1407.297276	1345.114273	1469.198402
**ENSG00000111490**	TBC1D30	16.87476881	14.01281628	8.956565414	5.023401917	10.40210053	3.035054026	6.128367209	8.306563181	8.441323766	5.694997971	0	1.691650434
**ENSG00000111530**	CAND1	3549.326373	3829.002049	3116.070531	3107.811319	3274.35009	2967.903456	3711.74774	3556.870354	3465.632368	3462.558767	3505.574735	3906.866676
**ENSG00000111540**	RAB5B	1236.076815	1355.739975	1344.299045	1136.963301	1193.929984	1200.743249	1610.739181	1739.39433	1791.436488	1635.097195	1709.32983	1737.324995
**ENSG00000111554**	MDM1	136.4043812	203.1858361	157.1470114	201.7733103	221.9114781	190.8290219	362.5950599	302.3588998	310.4531296	356.8865395	321.4478211	351.017465
**ENSG00000111581**	NUP107	1312.013275	1152.554139	1237.634494	1296.874928	1160.412104	1214.400992	1737.392104	1757.668769	1639.961623	1594.599432	1494.111546	1512.335488
**ENSG00000111596**	CNOT2	1102.484896	1071.980446	1329.642847	1147.010104	1113.024757	1123.349372	1388.075173	1322.404858	1309.812071	1457.286703	1444.445788	1405.76151
**ENSG00000111602**	TIMELESS	890.1440547	861.7882013	918.4550715	841.4198211	1028.652164	921.8976605	1555.583877	1905.525594	1535.383001	1520.564458	1785.208071	1541.093545
**ENSG00000111605**	CPSF6	1657.946036	1527.396975	1648.822269	1717.166222	1583.430859	1703.424072	2229.70427	1800.862898	2033.421103	2097.657586	1840.392246	2112.871392

**ENSG00000111615**	KRR1	2619.807858	2658.931889	2565.648874	2043.687347	2023.786448	2004.653184	1437.102111	1606.489319	1418.142393	1332.629525	1451.34381	1464.969275
**ENSG00000111639**	MRPL51	613.1166001	648.092753	624.5168793	810.442176	924.6311586	744.347	791.5807645	991.8036438	815.9946307	775.7852792	983.6579246	978.6197758
**ENSG00000111640**	GAPDH	17347.26234	21996.61836	15447.63264	17697.44495	22666.17706	17336.98736	18320.75377	39034.2017	22104.07524	18603.66004	34588.06147	20704.95548
**ENSG00000111641**	NOP2	8.437384405	0	5.699632536	10.88403749	8.090522638	9.863925586	11.23533988	4.983937908	12.66198565	8.226108181	2.759208765	4.229126084
**ENSG00000111642**	CHD4	202.4972257	122.6121425	153.8900785	243.634993	212.6651665	196.5197482	220.6212195	186.0670152	245.7363141	258.8060189	217.9774924	205.5355277
**ENSG00000111644**	ACRBP	5.624922937	0	0.814233219	2.511700959	1.155788948	2.27629052	0	4.983937908	6.096511609	0.632777552	5.518417529	4.229126084
**ENSG00000111647**	UHRF1BP1L	314.9956845	381.8492437	368.0334152	519.9220984	494.6776699	502.6808231	792.6021591	571.4915468	675.7748637	773.8869466	689.8021912	792.5382281
**ENSG00000111652**	COPS7A	239.0592248	276.7531216	267.8827292	330.7072929	270.4546139	320.1981998	425.921521	358.8435294	430.9764745	466.9898337	371.1135788	460.1289179
**ENSG00000111653**	ING4	77.34269038	84.07689769	95.26528668	73.67656145	94.77469376	98.63925586	167.5087037	197.6962037	154.2886399	136.0471738	246.9491844	136.1778599
**ENSG00000111664**	GNB3	0	0	0	3.348934611	3.467366845	1.896908767	0	0	0.937924863	0	1.379604382	0
**ENSG00000111665**	CDCA3	165.9352266	91.08330583	177.5028418	308.1019843	193.0167544	275.0517711	379.958767	244.2129575	377.9837198	442.9442867	373.8727876	395.8462015
**ENSG00000111666**	CHPT1	292.4959927	259.2371012	260.5546302	483.0838177	442.6671672	437.8065433	796.6877372	516.6682298	722.6711069	725.7958526	583.5726537	888.9623028
**ENSG00000111667**	USP5	542.8050634	514.9709984	650.5723423	725.0443434	706.1870474	748.1408175	813.0300498	800.7526906	928.0766518	965.6185449	960.2046501	946.4784176
**ENSG00000111669**	TPI1	2891.210389	4235.373721	2787.934543	3058.414534	4178.177048	3025.948864	3281.740641	8029.12397	3827.202403	3761.862549	7096.684943	3889.104347
**ENSG00000111670**	GNPTAB	676.3969831	616.5639164	796.3200886	745.9751847	844.8817212	934.4172584	3138.745406	3257.834079	3878.319308	2939.251731	3064.101333	3787.605321
**ENSG00000111671**	SPSB2	35.15576835	38.53524477	59.43902502	66.14145858	42.76419109	50.45777319	65.36925023	91.37219499	87.69597468	72.13664097	48.28615338	68.51184256
**ENSG00000111674**	ENO2	160.3103037	592.0414879	192.973273	138.1435527	346.7366845	154.0289918	92.94690267	402.0376579	67.0616277	91.7527451	467.6858856	80.35339559
**ENSG00000111676**	ATN1	739.6773662	686.6279978	780.8496574	823.0006808	839.1027764	891.926502	676.1631821	599.7338616	766.7535754	678.9703137	673.2469386	685.9642508
**ENSG00000111678**	C12orf57	104.0610743	129.6185506	101.7791524	72.8393278	85.52838217	99.39801937	128.6957114	149.5181373	188.053935	131.6177309	142.0992514	120.953006
**ENSG00000111679**	PTPN6	230.6218404	332.8043867	262.9973299	159.074394	191.8609654	155.5465189	260.4556064	287.407086	299.1980313	258.8060189	310.410986	242.7518372
**ENSG00000111684**	LPCAT3	132.185689	80.57369362	157.9612446	125.5850479	107.4883722	121.0227793	283.9476807	279.1005229	320.7703031	334.7393252	277.3004808	351.8632902
**ENSG00000111696**	NT5DC3	596.2418313	497.454978	601.7183492	462.99021	458.8482125	537.5839444	667.9920258	505.0390414	658.8922162	644.8003259	467.6858856	595.4609526
**ENSG00000111700**	SLCO1B3	2.812461468	0	0.814233219	0.837233653	0	0.379381753	1.021394535	1.661312636	0.468962431	1.898332657	0	0
**ENSG00000111707**	SUDS3	1392.168427	1243.637445	1342.670579	1501.15994	1488.656165	1379.432055	2265.453078	2119.834924	2363.570655	2306.474178	2415.687273	2474.884584
**ENSG00000111711**	GOLT1B	319.2143767	231.2114686	302.0805244	241.123292	243.8714681	278.0868252	514.7828456	471.8127887	541.1826459	506.2220419	429.0569629	543.8656144
**ENSG00000111716**	LDHB	70.31153671	63.05767327	35.82626166	61.95529031	62.41260321	48.18148267	90.9041136	38.21019063	77.37880119	87.95607978	51.04536215	92.19494863
**ENSG00000111725**	PRKAB1	359.9950679	318.7915704	417.7016416	382.6157794	332.8672171	474.6065734	756.8533503	717.6870588	726.4228063	698.5864178	634.6180159	714.7223082
**ENSG00000111726**	CMAS	175.7788418	178.6634076	158.7754778	159.9116277	182.6146538	144.544448	320.717884	323.955964	334.8391761	361.94876	293.8557334	391.6170754
**ENSG00000111727**	HCFC2	223.5906867	238.2178768	214.9575699	187.5403382	203.4188549	226.4909067	496.3977439	397.05372	467.5555442	479.0126072	535.2865003	549.7863909
**ENSG00000111728**	ST8SIA1	0	0	0	0	0	0	57.19809395	21.59706427	50.6479426	37.33387559	31.73090079	60.89941561
**ENSG00000111731**	C2CD5	189.8411491	185.6698157	149.8189124	198.4243757	182.6146538	163.8929174	468.8200915	405.3602832	413.1559021	485.9731602	418.0201278	466.8955197
**ENSG00000111737**	RAB35	961.8618222	1019.432384	1051.175086	955.2835979	836.7911985	977.6667783	1117.405621	887.1409477	1098.310014	957.3924368	921.5757274	958.3199706
**ENSG00000111752**	PHC1	87.18630552	66.56087734	83.05178838	83.72336529	85.52838217	80.4289317	275.7765244	184.4057026	277.156797	251.8454658	289.7169203	213.9937798
**ENSG00000111775**	COX6A1	389.5259134	518.4742024	416.0731751	490.6189206	420.7071772	421.4931279	547.4674707	554.8784205	594.1754007	554.3131359	627.719994	662.2811447
**ENSG00000111785**	RIC8B	455.6187579	465.9261414	415.2589419	396.8487515	475.0292577	439.7034521	512.7400565	752.5746242	533.2102846	513.182595	667.728521	614.9149326
**ENSG00000111786**	SRSF9	1835.131108	2431.223625	1675.691966	2239.600021	2164.7927	2021.725363	2304.266071	3186.397636	2540.369491	2517.821881	3298.634078	2681.265937
**ENSG00000111788**	AC009533.1	64.68661377	87.58010176	66.76712399	63.62975762	53.16629152	59.18355352	123.5887387	152.8407625	150.0679781	136.0471738	135.2012295	90.5032982
**ENSG00000111790**	FGFR1OP2	136.4043812	192.6762239	121.3207497	115.5382441	141.0062517	159.7197181	277.8193135	327.2785893	273.4050975	302.46767	335.2438649	274.8931955
**ENSG00000111799**	COL12A1	7728.644115	4224.864109	3861.90816	8185.633424	6574.127538	5080.68044	31710.21473	31194.46737	28931.69928	30959.90731	32895.28689	30858.24138
**ENSG00000111801**	BTN3A3	1366.856274	1748.098831	1444.449731	46.04785091	129.4483622	43.24951988	726.2115143	696.0899945	559.4721807	100.6116308	107.6091418	71.89514343
**ENSG00000111802**	TDP2	748.1147506	781.2145077	740.1379965	711.6486049	648.3976	645.7077441	540.3177089	629.6374891	517.7345243	535.3298093	514.5924346	584.4652248
**ENSG00000111816**	FRK	15.46853808	31.52883663	90.37988736	32.65211246	38.14103529	47.42271916	189.9793835	194.3735784	125.6819316	232.8621393	234.532745	140.406986
**ENSG00000111817**	DSE	770.6144423	1380.262404	1233.563327	925.1431864	1308.353089	1459.102223	228.7923758	307.3428377	301.5428434	284.7498986	307.6517773	355.246591
**ENSG00000111832**	RWDD1	781.8642882	679.6215897	686.398604	785.3251664	622.9702431	684.404683	691.4841001	639.6053649	631.2234327	700.4847505	637.3772246	817.9129846
**ENSG00000111834**	RSPH4A	1.406230734	0	1.628466439	0	0	0.758763507	0	3.322625272	0.937924863	0	2.759208765	1.691650434
**ENSG00000111837**	MAK	8.437384405	24.52242849	21.17006371	19.25637402	24.27156791	19.34846942	23.4920743	19.93575163	8.910286198	18.98332657	11.03683506	10.1499026
**ENSG00000111843**	TMEM14C	1226.2332	1436.313669	1245.776826	1156.219675	1270.212054	1363.498021	786.4737919	830.6563181	871.8011601	797.9324936	797.411333	760.3968699
**ENSG00000111845**	PAK1IP1	638.4287533	399.365264	609.8606814	797.0464375	512.0145041	703.7531524	487.2051931	378.779281	464.2728071	393.5876376	446.9918199	583.6193996
**ENSG00000111846**	GCNT2	220.7782253	220.7018564	210.0721706	133.1201508	107.4883722	135.8186677	61.28367209	36.54887799	77.84776362	40.49776335	46.906549	80.35339559
**ENSG00000111850**	SMIM8	99.84238213	150.637775	79.79485551	82.88613163	115.5788948	91.8103843	140.9524458	103.0013834	130.8405184	132.883286	96.57230676	125.1821321
**ENSG00000111859**	NEDD9	1276.857507	2830.588889	1946.017394	1580.697137	2956.50813	1954.574793	4702.500439	4937.421155	3925.215551	3939.040264	3973.260621	3206.523397
**ENSG00000111860**	CEP85L	11.24984587	10.50961221	17.91313083	22.60530863	55.47786952	39.07632059	37.79159779	83.06563181	43.61350613	79.09719405	114.5071637	50.74951301
**ENSG00000111863**	ADTRP	26.71838395	24.52242849	36.64049488	61.95529031	33.5178795	59.94231702	22.47067977	9.967875817	17.8205724	2.53111021	1.379604382	10.99572782
**ENSG00000111875**	ASF1A	250.3090707	206.6890402	219.8429693	313.9626198	246.183046	277.7074434	398.3438686	201.018829	378.9216446	441.045954	253.8472063	386.5421241
**ENSG00000111877**	MCM9	549.8362171	690.1312019	512.9669283	509.0380609	449.6019009	475.7447186	1021.394535	981.835768	837.5669026	886.5213509	867.7711565	846.671042
**ENSG00000111879**	FAM184A	4.218692202	35.0320407	5.699632536	8.372336529	16.18104528	12.14021611	15.32091802	6.645250545	13.13094808	27.20943475	8.277626294	28.75805737
**ENSG00000111880**	RNGTT	824.0512102	816.2465484	819.932852	980.4006075	941.9679928	992.4626666	808.9444716	825.6723802	794.4223589	799.1980487	851.2159039	1007.377833
**ENSG00000111885**	MAN1A1	1.406230734	7.006408141	4.885399317	0.837233653	4.623155793	2.655672273	98.05387535	81.40431917	70.81332715	89.22163489	74.49863664	94.73242428
**ENSG00000111886**	GABRR2	14.06230734	45.54165292	11.39926507	10.04680383	6.93473369	11.76083435	19.40649616	24.91968954	4.689624315	5.062220419	19.31446135	10.1499026
**ENSG00000111897**	SERINC1	3338.391763	4133.780803	3440.949585	3062.600702	3654.604654	3380.670804	3026.392007	2998.669308	3023.400796	3101.242784	2999.259927	3209.906698
**ENSG00000111906**	HDDC2	517.4929102	500.9581821	512.152695	628.7624733	633.3723436	589.5592446	631.2218226	594.7499237	657.9542913	652.3936565	535.2865003	741.7887151
**ENSG00000111907**	TPD52L1	627.1789074	374.8428355	533.3227587	1052.402702	1114.180546	996.6358659	1740.456287	1659.651323	1648.871909	1934.400978	1659.664072	2023.213919
**ENSG00000111911**	HINT3	862.01944	707.6472222	693.726703	659.7401185	790.5596406	853.6089449	799.7519208	757.5585621	903.221643	829.5713712	663.5897079	1020.065211
**ENSG00000111912**	NCOA7	5075.08672	4858.944046	5107.684986	3821.971625	4026.768696	4155.747726	3384.901489	3284.415082	3794.843995	2784.854008	2330.151802	3417.979701
**ENSG00000111913**	RIPOR2	19.68723028	63.05767327	15.47043117	4.186168264	18.49262317	8.725780326	59.24088302	101.3400708	94.26144872	74.03497363	63.46180159	71.04931821
**ENSG00000111961**	SASH1	3081.051539	2553.835767	2493.996351	3412.564369	3770.183549	3348.423355	3549.346009	4083.50646	3076.862513	3232.227738	3542.824054	3214.981649
**ENSG00000111962**	UST	0	0	2.442699658	2.511700959	2.311577897	1.896908767	0	8.306563181	0	0	2.759208765	0

**ENSG00000111981**	ULBP1	49.2180757	31.52883663	36.64049488	37.67551438	73.97049269	62.97737105	40.8557814	39.87150327	37.04803208	26.5766572	20.69406573	21.14563042
**ENSG00000112029**	FBXO5	1127.797049	1026.438793	951.0244003	1293.525994	1039.054264	1099.068939	935.5973939	1182.854597	1105.813413	1051.676292	1020.907243	1234.058991
**ENSG00000112031**	MTRF1L	462.6499115	507.9645902	491.7968645	496.4795562	399.9029761	510.6478399	462.6917243	445.2317865	430.5075121	389.1581947	435.9549848	394.154551
**ENSG00000112033**	PPARD	385.3072212	266.2435094	377.8042138	349.9636669	388.3450866	418.8374556	465.7559079	445.2317865	526.6448105	551.7820257	510.4536215	516.7992075
**ENSG00000112038**	OPRM1	1.406230734	10.50961221	12.21349829	9.209570182	8.090522638	11.3814526	0	0	0	0	0	0
**ENSG00000112039**	FANCE	132.185689	108.5993262	135.1627144	186.7031046	162.9662417	180.9650963	231.8565594	156.1633878	265.4327362	283.4843435	235.9123494	293.5013502
**ENSG00000112062**	MAPK14	825.4574409	746.182467	829.7036506	1015.564421	825.2333091	927.9677686	1001.988039	930.3350762	951.5247734	980.8052062	902.261266	972.6989993
**ENSG00000112078**	KCTD20	1905.442645	2052.877585	2191.101594	2195.226638	2141.676921	2396.175154	2045.853253	2242.772059	2071.876022	2164.099229	2299.800505	2223.674495
**ENSG00000112079**	STK38	701.7091363	746.182467	764.5649931	750.9985866	913.0732691	839.1924383	974.4103863	1194.483785	899.4699435	789.7063854	1077.471023	945.6325924
**ENSG00000112081**	SRSF3	5548.986477	4526.139659	4966.008405	5551.696352	4401.244315	5059.814444	5157.021007	5076.971416	5410.419572	5372.28142	4766.533141	5911.47244
**ENSG00000112096**	SOD2	2199.344868	4487.604414	3349.755465	868.211298	945.4353597	796.3223002	2478.924536	3098.348066	2347.625932	1857.202116	1931.446135	1818.524216
**ENSG00000112110**	MRPL18	824.0512102	945.865099	873.6722445	1039.844197	987.0437618	1051.64622	671.0562094	662.8637418	787.3879224	709.9764138	634.6180159	888.9623028
**ENSG00000112118**	MCM3	2349.811557	2441.733237	2278.224548	2740.265746	2609.771445	2628.356787	2790.449869	2913.942364	2818.464213	2977.218384	3002.019136	2856.351757
**ENSG00000112130**	RNF8	299.5271464	290.7659378	285.79586	296.3807131	327.0882724	343.3404867	320.717884	230.9224564	297.791144	322.0837742	244.1899757	330.7176598
**ENSG00000112137**	PHACTR1	0	3.50320407	4.885399317	0	0	0	0	4.983937908	0.468962431	1.265555105	8.277626294	0
**ENSG00000112139**	MDGA1	123.7483046	196.1794279	241.8272662	18.41914036	18.49262317	22.7629052	122.5673442	179.4217647	119.58542	18.35054902	23.4532745	30.4497078
**ENSG00000112144**	ICK	227.8093789	203.1858361	219.028736	221.0296844	258.8967244	237.4929776	972.3675972	631.2988017	836.1600153	1035.224076	706.3574437	913.4912341
**ENSG00000112146**	FBXO9	452.8062964	385.3524477	418.5158748	581.8773887	446.134534	491.6787523	878.3993	697.7513072	745.1813036	878.2952427	699.4594218	828.9087124
**ENSG00000112149**	CD83	98.43615139	392.3588559	359.891083	93.77016912	223.067267	297.0559128	11.23533988	21.59706427	7.972361335	5.062220419	15.17564821	9.304077385
**ENSG00000112159**	MDN1	3367.922608	2080.903218	3306.601104	3780.947176	2921.834461	3310.864561	3684.170087	2739.504537	3413.577539	4051.674668	2833.707401	3772.380467
**ENSG00000112167**	SAYSD1	150.4666886	164.6505913	117.2495836	133.1201508	161.8104528	177.9300423	181.8082272	245.8742701	154.2886399	161.3582759	189.0058004	111.6489286
**ENSG00000112175**	BMP5	35.15576835	42.03844885	9.770798633	105.4914403	50.85471372	31.86806728	0	0	0	0	0	1.691650434
**ENSG00000112182**	BACH2	92.81122845	154.1409791	150.6331456	190.8892729	136.3830959	135.0599042	91.92550814	98.01744553	205.8745074	143.0077268	128.3032076	221.6062068
**ENSG00000112183**	RBM24	0	0	0.814233219	0.837233653	1.155788948	1.896908767	0	0	1.875849726	1.265555105	0	0
**ENSG00000112186**	CAP2	306.5583	297.772346	377.8042138	475.5487148	377.9429861	430.9776718	756.8533503	671.170305	638.7268316	803.6274915	718.7738832	739.2512395
**ENSG00000112195**	TREML2	0	10.50961221	14.65619795	10.04680383	10.40210053	8.346398573	5.106972674	1.661312636	9.379248629	5.694997971	2.759208765	15.2248539
**ENSG00000112200**	ZNF451	2761.837162	2543.326155	2594.96127	2503.328622	2542.735686	2773.659998	2872.161432	2751.133725	2877.553479	2576.037416	2396.372812	2902.872144
**ENSG00000112208**	BAG2	25.31215321	24.52242849	33.383562	25.11700959	30.05051265	36.42064832	40.8557814	23.25837691	40.79973154	39.8649858	55.18417529	72.74096864
**ENSG00000112210**	RAB23	359.9950679	500.9581821	294.7524254	388.4764149	506.2355593	410.8704388	303.3541769	412.0055338	307.1703926	318.2871088	354.5583263	336.6384363
**ENSG00000112218**	GPR63	25.31215321	17.51602035	13.02773151	30.97764516	38.14103529	28.4536315	67.4120393	64.79119281	69.87540229	67.07442055	78.63744979	50.74951301
**ENSG00000112234**	FBXL4	648.2723684	749.6856711	673.3708725	536.6667715	598.6986752	545.1715795	1133.747934	955.2547658	986.2279933	1016.240749	909.1592879	979.465601
**ENSG00000112237**	CCNC	1853.412108	1495.868138	1621.13834	2016.89587	1651.622407	1894.632476	1306.36361	1237.677914	1208.985148	1201.011794	1258.199197	1355.857822
**ENSG00000112242**	E2F3	4603.999424	4196.838476	4430.242947	4369.522434	3793.299328	4525.644935	2971.236702	2520.211269	3520.03201	2885.465639	2559.166129	3458.579311
**ENSG00000112245**	PTP4A1	35.15576835	70.06408141	29.3123959	36.00104707	61.25681426	15.93403364	75.58319558	89.71088235	80.19257578	72.13664097	73.11903226	45.67456171
**ENSG00000112249**	ASCC3	5193.210101	5219.774065	4952.166441	3939.184337	4009.431862	3763.466993	5563.536031	5992.354679	5208.296764	4313.644575	4794.125229	4476.107047
**ENSG00000112273**	HDGFL1	126.5607661	52.54806106	152.261612	115.5382441	106.3325832	107.7444179	1.021394535	0	0.468962431	1.898332657	1.379604382	5.074951301
**ENSG00000112276**	BVES	16.87476881	31.52883663	36.64049488	21.76807497	31.2063016	31.86806728	0	4.983937908	0	0	1.379604382	0
**ENSG00000112282**	MED23	897.1752084	952.8715072	961.6094322	789.5113347	737.393349	845.6419281	982.5815426	988.4810185	994.6693171	1011.811306	985.037529	916.874535
**ENSG00000112290**	WASF1	243.277917	213.6954483	285.79586	316.4743208	294.7261818	360.0332839	780.3454246	408.6829085	703.4436472	730.858073	590.4706756	797.6131794
**ENSG00000112293**	GPLD1	0	0	0.814233219	0.837233653	4.623155793	1.517527013	237.9849266	372.1340305	277.156797	249.9471332	376.6319964	289.2722241
**ENSG00000112294**	ALDH5A1	29.53084542	14.01281628	21.98429693	15.07020575	31.2063016	20.86599643	588.3232521	613.0243627	723.6090317	630.8792197	624.9607852	630.9856117
**ENSG00000112297**	CRYBG1	6824.437753	5769.777104	6483.739126	6215.622639	5554.721685	5767.361414	3131.595644	2422.193823	3331.509113	2302.044736	1506.527985	2359.852355
**ENSG00000112299**	VNN 1.00	170.1539188	665.6087734	469.8125676	98.79357104	276.2335586	337.6497604	18.38510163	9.967875817	10.78613592	8.226108181	15.17564821	2.53747565
**ENSG00000112303**	VNN 2.00	14.06230734	42.03844885	37.45472809	0	10.40210053	3.793817533	7.149761744	19.93575163	9.379248629	6.960553076	2.759208765	6.766601734
**ENSG00000112304**	ACOT13	563.8985244	641.0863449	609.8606814	480.5721167	523.5723936	522.0292925	354.4239036	327.2785893	317.4875661	328.4115497	331.1050518	352.7091154
**ENSG00000112305**	SMAP1	538.5863712	683.1247937	591.1333173	645.5071464	772.0670174	715.5139867	667.9920258	915.3832625	647.1681554	773.8869466	871.9099696	869.5083228
**ENSG00000112306**	RPS12	6460.223993	6312.773735	5355.211884	6287.624733	6351.060271	5980.573959	5924.088302	6384.424461	6084.787548	5684.240753	5798.477219	6035.808747
**ENSG00000112308**	C6orf62	4691.185729	4655.75821	4483.982339	2350.114864	2757.712431	2684.884668	2972.258096	2410.564635	2979.787289	2159.037009	1930.066531	2393.685363
**ENSG00000112309**	B3GAT2	1.406230734	0	3.256932878	4.186168264	0	0.758763507	9.192550814	0	0	4.429442867	0	2.53747565
**ENSG00000112312**	GMNN	731.2399818	847.775385	725.4817985	717.5092405	747.7954495	723.8603853	662.8850531	749.2519989	639.6647565	669.4786504	713.2554657	750.2469673
**ENSG00000112319**	EYA4	4.218692202	0	0.814233219	0	0	2.27629052	7.149761744	0	0.937924863	3.796665314	0	1.691650434
**ENSG00000112335**	SNX3	1320.450659	1411.79124	1288.116953	1478.554631	1610.014005	1461.378514	1144.983274	1265.920229	1198.199012	1066.862953	1203.015021	1467.506751
**ENSG00000112339**	HBS1L	1816.850109	1814.659708	1659.407301	2119.038375	2000.670669	1939.778905	1625.038705	1746.039581	1523.189977	1612.317203	1765.893609	1842.207322
**ENSG00000112343**	TRIM38	1965.910566	2686.957522	1728.617125	813.7911106	1137.296325	814.1532426	1480.000681	1285.85598	1207.109299	834.000814	820.8646075	815.375509
**ENSG00000112357**	PEX7	244.6841477	129.6185506	154.7043117	130.6084498	175.6799201	179.0681876	264.5411845	164.469951	174.4540245	205.019927	128.3032076	207.2271781
**ENSG00000112365**	ZBTB24	547.0237556	357.3268152	466.5556347	596.1103608	371.0082524	477.2622457	550.5316543	453.5383497	502.2587641	552.4148032	412.5017103	508.3409553
**ENSG00000112367**	FIG4	551.2424478	742.6792629	657.0862081	582.7146224	657.6439116	602.837606	845.7146749	828.9950054	835.6910528	971.3135429	1012.629617	931.2535637
**ENSG00000112378**	PERP	22212.82068	23762.23321	19051.42887	25856.2869	29775.43489	25354.84134	19513.74259	19316.08202	17241.87275	20470.9866	20502.30073	20503.64908
**ENSG00000112379**	ARFGEF3	143.4355349	490.4485699	279.2819943	72.00209415	276.2335586	183.2413868	105.2036371	230.9224564	82.0684255	117.6966247	208.3202617	66.82019213
**ENSG00000112394**	SLC16A10	0	3.50320407	0.814233219	0.837233653	3.467366845	4.931962793	0	0	0	1.898332657	0	0
**ENSG00000112406**	HECA	1297.950968	2066.890402	1216.46443	1324.503639	1490.967743	1417.37023	1236.908782	1322.404858	1337.011892	1438.303377	1346.493877	1466.660926
**ENSG00000112414**	ADGRG6	2271.062636	1842.685341	1946.017394	2426.303126	2362.43261	2598.385628	1594.396869	1036.659085	1926.028706	1701.538838	1134.034802	2380.15216
**ENSG00000112419**	PHACTR2	1756.382187	1387.268812	1489.232558	2722.683839	2100.068519	2303.985388	826.3081787	875.5117592	915.8836286	942.2057755	849.8362995	1258.587923
**ENSG00000112425**	EPM2A	151.8729193	84.07689769	117.2495836	146.5158893	151.4083522	105.8475092	365.6592435	184.4057026	236.3570655	354.3554293	191.7650091	235.9852355
**ENSG00000112473**	SLC39A7	1392.168427	1282.17269	1455.848996	1579.859903	1537.199301	1652.207536	1290.021298	1275.888105	1542.886399	1430.077268	1441.68658	1678.11723
**ENSG00000112499**	SLC22A2	2.812461468	10.50961221	7.328098975	3.348934611	5.778944741	6.449489806	0	0	0	2.53111021	0	0
**ENSG00000112511**	PHF1	247.4966092	395.86206	328.9502207	176.6563008	295.8819708	243.5630856	117.4603715	217.6319553	158.5093018	140.4766166	216.597888	130.2570834

**ENSG00000112514**	CUTA	745.3022891	753.1888751	762.1222934	858.1644942	896.8922239	923.0358058	537.2535253	493.4098529	619.9683344	524.5725909	538.0457091	651.2854169
**ENSG00000112531**	QKI	7350.368047	7079.975426	7558.526976	7431.285903	6897.748443	7793.639358	4009.994944	4950.711656	4850.009466	3512.548193	4105.702642	4798.366455
**ENSG00000112541**	PDE10A	2169.814023	2150.967299	2188.658894	1680.327941	1762.578146	1923.844871	111.3320043	144.5341993	144.4404289	85.42496957	142.0992514	120.953006
**ENSG00000112559**	MDFI	212.3408409	147.134571	187.2736405	248.6583949	151.4083522	281.1218792	21.44928523	19.93575163	36.11010722	20.88165923	53.80457091	27.91223215
**ENSG00000112561**	TFEB	4.218692202	24.52242849	13.02773151	15.9074394	28.89472371	14.41650663	4.08557814	8.306563181	5.158586746	1.898332657	2.759208765	5.074951301
**ENSG00000112562**	SMOC2	8.437384405	14.01281628	26.86969624	1.674467306	4.623155793	8.346398573	2.04278907	3.322625272	9.379248629	3.796665314	8.277626294	2.53747565
**ENSG00000112576**	CCND3	257.3402244	381.8492437	406.3023765	356.6615361	372.1640413	412.7673476	275.7765244	300.6975871	349.8459739	303.1004476	295.2353378	357.7840667
**ENSG00000112578**	BYSL	323.4330689	196.1794279	343.6064186	336.5679285	243.8714681	375.9673175	211.4286687	117.9531972	208.688282	219.5738107	161.4137127	255.4392155
**ENSG00000112584**	FAM120B	800.1452877	844.272181	835.4032832	757.6964558	768.5996506	827.8109857	1029.565691	1086.498464	982.9452563	1103.564051	1124.377572	1001.457057
**ENSG00000112592**	TBP	412.0256051	283.7595297	418.5158748	374.2434428	349.0482624	414.2848746	392.2155014	388.7471569	400.9628789	429.6559581	387.6688314	366.2423189
**ENSG00000112599**	GUCA1B	9.843615139	45.54165292	14.65619795	19.25637402	15.02525633	9.484543833	34.72741419	41.5328159	12.66198565	29.74054496	24.83287888	14.37902869
**ENSG00000112624**	BICRAL	774.8331345	714.6536304	696.1694026	770.2549606	635.6839215	717.7902773	791.5807645	697.7513072	703.4436472	622.020334	562.878588	597.9984283
**ENSG00000112640**	PPP2R5D	552.6486785	655.0991612	570.7774868	820.4889798	824.0775201	820.9821142	611.8153264	523.3134804	653.264667	638.4725504	658.0712904	707.9557064
**ENSG00000112651**	MRPL2	36.56199909	24.52242849	24.42699658	35.16381342	42.76419109	34.1443578	28.59904698	33.22625272	37.51699452	28.47498986	23.4532745	27.06640694
**ENSG00000112655**	PTK7	255.9339936	259.2371012	300.452058	432.8497985	460.0040014	441.9797426	748.6821941	752.5746242	728.7676185	797.9324936	1047.119726	881.3498759
**ENSG00000112658**	SRF	379.6822982	346.817203	444.5713378	427.8263966	323.6209055	471.1921376	488.2265877	544.9105446	475.9968679	424.5937376	533.906896	427.1417345
**ENSG00000112659**	CUL9	426.0879124	416.8812844	422.5870409	282.147741	355.9829961	278.8455887	516.8256346	544.9105446	453.4866712	411.9381866	579.4338406	356.0924163
**ENSG00000112667**	DNPH1	170.1539188	126.1153465	170.1747429	178.3307681	154.8757191	177.9300423	114.3961879	162.8086383	200.7159207	144.9060595	146.2380645	206.3813529
**ENSG00000112679**	DUSP22	375.463606	472.9325495	458.4133025	514.0614629	535.130283	490.9199888	290.0760479	403.6989706	309.0462423	343.5982109	440.093798	330.7176598
**ENSG00000112685**	EXOC2	1157.327894	1071.980446	1040.590054	1085.892048	1051.767943	1055.060656	1058.164738	1068.224025	972.6280828	908.6685652	905.0204748	1005.686183
**ENSG00000112695**	COX7A2	1754.975956	1800.646892	1615.438707	1779.958746	1770.668669	1803.201473	1395.224935	1481.890871	1382.97021	1496.518911	1236.125527	1535.172768
**ENSG00000112697**	TMEM30A	2363.873864	2354.153135	2041.282681	2386.115911	2442.182048	2647.325875	2686.267627	2706.278284	2599.92772	2726.005696	2597.795052	3027.208451
**ENSG00000112699**	GMDS	476.7122189	444.9069169	513.7811615	462.1529764	460.0040014	495.8519516	284.9690752	309.0041503	285.1291583	255.6421312	331.1050518	292.655525
**ENSG00000112701**	SENP6	1933.567259	1702.557178	1950.088561	1983.406524	1865.443362	1897.288148	2695.460178	2239.449433	2296.509027	2693.101263	2210.12622	2443.589051
**ENSG00000112715**	VEGFA	3979.632978	5846.847594	4637.872418	2349.27763	4046.417108	3268.753186	1359.476126	4226.379346	1356.708314	977.0085409	3658.710822	907.5704576
**ENSG00000112739**	PRPF4B	2802.617853	2686.957522	2835.974303	2679.984923	2382.081022	2553.618581	3118.317515	3056.81525	2791.264392	2737.395692	2927.520499	3061.887285
**ENSG00000112742**	TTK	1877.31803	1387.268812	1575.54128	2217.831946	2078.108529	2160.958467	2033.596519	1834.08915	1983.711085	2014.130949	1870.743542	2538.321476
**ENSG00000112759**	SLC29A1	442.9626813	297.772346	368.0334152	551.7369772	389.5008756	474.2271916	358.5094817	227.5998311	430.5075121	551.1492481	343.5214912	586.1568752
**ENSG00000112761**	WISP3	4.218692202	0	7.328098975	5.023401917	4.623155793	3.793817533	5.106972674	1.661312636	4.220661883	5.062220419	1.379604382	0
**ENSG00000112763**	BTN2A1	593.4293698	739.1760589	792.2489225	518.2476311	520.1050267	540.6189985	339.1029856	368.8114052	381.2664568	321.4509966	360.0767438	306.1887285
**ENSG00000112769**	LAMA4	80.15515185	80.57369362	96.89375311	59.44358935	47.38734688	89.91347553	33.70601965	51.50069172	52.05482989	36.06832049	13.79604382	38.90795997
**ENSG00000112773**	FAM46A	1885.755415	2613.390237	1919.147698	1029.797393	1514.083522	1251.201022	1469.786736	2010.18829	1331.384343	1434.506711	1822.457389	1667.967327
**ENSG00000112782**	CLIC5	4314.315892	3233.457357	4229.941575	1995.965028	1150.010004	1988.339769	3105.039386	2297.595376	3734.816804	2067.284264	1609.998314	2858.889233
**ENSG00000112787**	FBRSL1	268.5900702	213.6954483	254.8549977	296.3807131	338.6461618	345.6167773	523.9753964	352.1982789	463.3348823	513.8153725	398.7056665	485.5036744
**ENSG00000112796**	ENPP5	111.092228	129.6185506	105.8503185	99.63080469	141.0062517	111.9176172	122.5673442	112.9692593	135.9991051	110.7360717	193.1446135	140.406986
**ENSG00000112812**	PRSS16	240.4654555	301.2755501	293.9381922	193.4009738	227.6904228	188.5527314	283.9476807	292.391024	274.3430224	296.7726721	304.8925685	249.5184389
**ENSG00000112818**	MEP1A	0	0	2.442699658	0	1.155788948	1.517527013	0	0	0	1.265555105	0	0.845825217
**ENSG00000112851**	ERBIN	7564.115119	8652.914054	7902.947628	7864.972935	7258.354595	8465.903825	4808.72547	4176.539967	4388.081471	4604.089471	4251.940706	4766.225097
**ENSG00000112852**	PCDHB2	4.218692202	21.01922442	9.770798633	21.76807497	12.71367843	16.69279715	4.08557814	8.306563181	5.158586746	11.38999594	2.759208765	10.99572782
**ENSG00000112855**	HARS2	665.1471373	735.6728548	661.1573742	596.9475945	648.3976	588.0417176	621.0078772	656.2184913	570.2583166	658.721432	673.2469386	604.76503
**ENSG00000112874**	NUDT12	278.4336854	266.2435094	255.6692309	267.0775353	233.4693675	237.1135958	283.9476807	403.6989706	285.5981208	298.6710047	299.374151	323.951058
**ENSG00000112877**	CEP72	419.0567588	413.3780803	458.4133025	432.0125649	443.8229561	465.1220296	398.3438686	460.1836002	460.5211077	484.7076051	513.2128302	384.0046484
**ENSG00000112893**	MAN2A1	2418.716863	2238.547401	2403.616464	2599.610492	2271.125283	3009.635449	2220.511719	1928.783971	2289.47459	1935.033755	1746.579148	2441.051576
**ENSG00000112902**	SEMA5A	525.9302946	774.2080996	583.8052183	732.5794463	1113.024757	1087.687487	7776.897989	8482.66232	7597.19139	8102.083781	8281.765107	8438.798188
**ENSG00000112936**	C7	60.46792157	80.57369362	104.2218521	78.69996337	142.1620406	165.4104444	0	0	0	0	4.138813147	5.074951301
**ENSG00000112941**	PAPD7	1791.537955	1814.659708	1922.404631	2009.360767	1917.453865	1822.170561	1841.574346	2387.306258	1903.518509	1885.677106	2363.262307	1958.931202
**ENSG00000112964**	GHR	5.624922937	17.51602035	8.956565414	0.837233653	20.80420107	27.31548624	2.04278907	0	1.875849726	1.898332657	0	0
**ENSG00000112972**	HMGCS1	3338.391763	3194.922112	2648.700663	2933.66672	2829.371345	2546.030946	2426.833415	2815.924918	2192.868329	2185.613666	2734.375886	2224.52032
**ENSG00000112977**	DAP	2034.815872	2119.438463	2323.007375	2237.925554	2493.036761	2712.200154	2391.084606	2995.346683	2347.156969	2239.399758	2774.384413	2486.726137
**ENSG00000112983**	BRD8	1213.577124	1138.541323	1160.282338	1158.731376	1056.391099	1128.660716	1485.107654	1199.467723	1505.369405	1532.587232	1292.689306	1535.172768
**ENSG00000112984**	KIF20A	939.3621304	406.3716722	676.6278054	1223.198367	1186.99525	1145.353513	1350.283575	888.8022603	1429.866454	1435.772266	1172.663725	1787.228683
**ENSG00000112992**	NNT	777.645596	809.2401403	569.1490204	780.3017645	815.9869975	712.4789327	1776.205096	2026.801416	1625.423787	1741.403824	1956.279014	1776.232955
**ENSG00000112996**	MRPS30	764.9895194	676.1183856	786.54929	911.747448	883.0227565	943.5224205	1667.937275	1362.276362	1523.189977	1683.821067	1517.564821	1727.175093
**ENSG00000113013**	HSPA9	8178.63795	6312.773735	8104.063233	7176.766872	6274.7782	7029.564507	6304.047069	4987.260534	6342.716885	6373.335508	4967.955381	6537.3831
**ENSG00000113048**	MRPS27	1649.508651	1376.7592	1603.225209	1979.220355	1723.281322	1809.650963	1261.422251	1294.162544	1378.280586	1380.087842	1329.938625	1398.994909
**ENSG00000113068**	PFDN1	548.4299863	525.4806106	648.9438759	677.3220252	624.1260321	702.6150071	440.2210445	453.5383497	526.1758481	451.8031724	413.8813147	480.4287231
**ENSG00000113070**	HBEGF	2393.40471	2431.223625	3617.638194	1163.754777	785.9364848	2358.61636	197.1291452	79.74300653	396.742217	176.5449371	78.63744979	307.0345537
**ENSG00000113073**	SLC4A9	39.37446056	38.53524477	24.42699658	11.72127114	11.55788948	6.828871559	0	4.983937908	5.627549177	5.062220419	2.759208765	6.766601734
**ENSG00000113083**	LOX	0	0	3.256932878	0	4.623155793	0.379381753	1.021394535	13.29050109	0	0	4.138813147	0
**ENSG00000113108**	APBB3	109.6859973	112.1025303	108.2930182	130.6084498	97.08627165	106.2268909	191.000778	162.8086383	162.2610013	200.5904841	157.2748996	123.4904816
**ENSG00000113119**	TMCO6	134.9981505	56.05126513	116.4353504	131.4456835	100.5536385	106.6062727	182.8296217	59.8072549	134.1232554	143.6405044	78.63744979	116.7238799
**ENSG00000113140**	SPARC	0	3.50320407	0	0.837233653	5.778944741	3.035054026	4.08557814	14.95181373	32.8273702	20.88165923	19.31446135	59.20776517
**ENSG00000113141**	IK	1522.947885	1688.544362	1547.85735	1561.440763	1560.31508	1533.840429	1512.685306	1628.086383	1508.18318	1480.699473	1701.052203	1512.335488
**ENSG00000113161**	HMGCR	3321.516994	2364.662748	2165.046131	3903.18329	3333.295327	2702.336229	2566.764466	3206.333388	2430.16332	2454.544126	3567.656933	2814.060496
**ENSG00000113163**	COL4A3BP	1333.106736	1509.880954	1300.330451	1475.205696	1761.422357	1555.085807	1312.491977	1533.391563	1227.743646	1419.32005	1666.562094	1346.553745
**ENSG00000113194**	FAF2	2705.587933	2669.441502	2962.994686	2530.120099	2328.914731	2569.552615	1651.594963	1765.975332	1654.968421	1504.74502	1741.06073	1599.455485
**ENSG00000113196**	HAND1	15.46853808	3.50320407	8.956565414	41.02444899	18.49262317	36.04126656	3.064183605	3.322625272	7.972361335	4.429442867	4.138813147	5.920776517

**ENSG00000113205**	PCDHB3	5.624922937	3.50320407	14.65619795	5.86063557	10.40210053	12.51959786	25.53486337	4.983937908	5.627549177	9.491663286	27.59208765	14.37902869
**ENSG00000113209**	PCDHB5	2.812461468	3.50320407	3.256932878	0	0	0.758763507	0	0	0	1.265555105	2.759208765	0
**ENSG00000113211**	PCDHB6	0	3.50320407	0	2.511700959	1.155788948	0.758763507	0	8.306563181	0.468962431	0	0	0
**ENSG00000113212**	PCDHB7	0	0	0	5.023401917	0	2.655672273	0	0	0	1.265555105	0	0
**ENSG00000113231**	PDE8B	0	7.006408141	0	7.535102876	3.467366845	1.896908767	1.021394535	0	0.937924863	0	0	0.845825217
**ENSG00000113240**	CLK4	430.3066047	525.4806106	525.1804265	442.0593687	432.2650667	436.6683981	596.4944084	727.6549346	540.7136835	603.0370074	682.9041692	491.4244509
**ENSG00000113248**	PCDHB15	8.437384405	0	4.885399317	5.86063557	12.71367843	4.173199286	12.25673442	1.661312636	4.689624315	12.65555105	8.277626294	9.304077385
**ENSG00000113249**	HAVCR1	9.843615139	3.50320407	10.58503185	10.04680383	15.02525633	10.62268909	5.106972674	3.322625272	1.406887294	0	4.138813147	5.074951301
**ENSG00000113263**	ITK	60.46792157	45.54165292	67.58135721	1.674467306	0	0	4.08557814	9.967875817	2.813774589	0	0	0
**ENSG00000113269**	RNF130	19.68723028	87.58010176	36.64049488	24.27977593	80.90522638	25.79795922	6.128367209	6.645250545	9.379248629	5.062220419	11.03683506	4.229126084
**ENSG00000113272**	THG1L	182.8099954	157.6441832	193.7875062	185.0286373	224.223056	203.7280015	327.8676457	378.779281	299.1980313	331.5754374	307.6517773	318.0302815
**ENSG00000113273**	ARSB	212.3408409	199.682632	176.6886086	241.123292	321.3093276	283.7775515	335.0174074	260.8260839	338.5908755	384.0959743	300.7537553	406.8419293
**ENSG00000113282**	CLINT1	2109.346101	1572.938628	2015.227218	2026.10544	1769.51288	1982.269661	2888.503745	2629.857903	2859.732907	2974.687274	2861.299489	3083.87874
**ENSG00000113296**	THBS4	2.812461468	10.50961221	8.142332194	4.186168264	6.93473369	3.41443578	2.04278907	26.58100218	6.096511609	5.062220419	4.138813147	5.074951301
**ENSG00000113300**	CNOT6	1773.256956	1478.352118	1558.442382	1615.023716	1544.134035	1547.11879	1211.373918	1051.610899	1239.467706	1276.312323	1056.776957	1438.748694
**ENSG00000113312**	TTC1	1136.234433	1047.458017	1262.875723	1105.148422	1101.466868	1190.879324	983.6029371	1149.628344	927.6076894	901.7080121	983.6579246	1102.956083
**ENSG00000113318**	MSH3	696.0842134	721.6600385	753.165728	798.7209048	829.8564649	809.6006616	1126.598172	1013.400708	994.2003547	977.6413184	1091.267066	1031.906764
**ENSG00000113319**	RASGRF2	2.812461468	0	4.071166097	6.697869223	0	0.758763507	32.68462512	71.43644335	13.59991051	59.48108992	91.05388923	16.07067912
**ENSG00000113328**	CCNG1	663.7409065	518.4742024	435.6147724	663.0890531	637.9954994	416.1817834	706.8050181	563.1849836	570.7272791	610.630338	549.0825442	649.5937665
**ENSG00000113356**	POLR3G	205.3096872	192.6762239	159.589711	218.5179834	202.2630659	175.6537518	48.00554314	28.24231481	51.58586746	63.27775524	38.6289227	49.90368779
**ENSG00000113360**	DROSHA	3380.578685	2900.65297	3176.323789	3437.681379	3121.785949	3236.505737	3155.087718	2852.473796	3059.510903	3206.916635	2692.987754	3343.547082
**ENSG00000113368**	LMNB1	2085.440179	1341.727159	1624.395273	2541.84137	2245.697926	2066.871792	3221.478363	2781.037353	3377.467431	3315.121597	3191.024936	3980.45347
**ENSG00000113369**	ARRDC3	966.0805144	3366.579112	1056.060486	821.3262135	4672.854718	851.3326544	3361.409414	12303.68138	3181.441135	3435.349332	13775.34976	3583.761443
**ENSG00000113384**	GOLPH3	2579.027166	2550.332563	2507.024083	2374.39464	2342.784198	2662.121763	2510.587767	2370.693132	2527.707506	2447.583573	2492.945119	2635.591375
**ENSG00000113387**	SUB1	3045.89577	3328.043867	3028.947576	3450.239883	3198.06802	3141.660299	2915.060003	3247.866204	3085.303837	3140.474992	3231.033463	3541.470183
**ENSG00000113389**	NPR 3.00	26.71838395	10.50961221	19.54159727	12.55850479	8.090522638	14.41650663	15.32091802	9.967875817	19.69642212	12.0227735	5.518417529	13.53320347
**ENSG00000113391**	FAM172A	241.8716863	227.7082646	301.2662912	463.8274437	439.1998003	422.2518914	508.6544784	569.8302342	442.7005353	570.7653522	499.4167864	619.9898839
**ENSG00000113396**	SLC27A6	1.406230734	3.50320407	0	10.04680383	12.71367843	5.311344546	499.4619276	531.6200436	453.0177088	551.7820257	521.4904565	547.2489153
**ENSG00000113407**	TARS	10835.00781	11248.78827	12403.21463	8924.073506	9151.536892	11245.25455	9384.572986	9987.811568	9360.021169	7566.121194	7798.903573	8362.673918
**ENSG00000113441**	LNPEP	3521.201758	4736.331903	3870.864725	4031.280039	4845.067271	4620.110992	3266.419723	3405.690904	3738.099541	4154.184631	4087.767785	4838.12024
**ENSG00000113448**	PDE4D	416.2442973	322.2947745	376.9899806	608.6688656	575.5828962	595.2499709	604.6655646	458.5222876	687.9678869	601.7714523	635.9976202	757.013569
**ENSG00000113456**	RAD1	1337.325428	1131.534915	1240.077193	1512.881211	1147.698426	1371.465038	1456.508607	1528.407625	1440.183627	1543.977228	1483.074711	1497.110634
**ENSG00000113460**	BRIX1	2397.623402	1730.582811	2220.413989	2426.303126	1673.582397	2280.463719	1642.402412	1152.950969	1597.755004	1629.402197	1196.116999	1772.003829
**ENSG00000113494**	PRLR	2.812461468	3.50320407	5.699632536	6.697869223	0	5.311344546	4686.158126	4856.016835	2269.309206	4774.93941	4973.473798	2893.568067
**ENSG00000113504**	SLC12A7	2984.021618	4774.867148	4635.429718	1604.139679	3415.356342	3256.233589	50.04833221	48.17806645	101.7648476	22.14721433	24.83287888	76.97009473
**ENSG00000113522**	RAD50	184.2162262	150.637775	149.0046792	172.4701325	158.3430859	159.3403364	177.7226491	149.5181373	172.1092123	172.7482718	147.6176689	192.8481494
**ENSG00000113525**	IL5	1.406230734	0	5.699632536	0	1.155788948	0.758763507	3.064183605	1.661312636	0	0	2.759208765	1.691650434
**ENSG00000113532**	ST8SIA4	954.8306685	882.8074257	885.8857428	880.7698028	826.389098	567.5551029	176.7012545	147.8568246	116.302683	118.3294023	115.8867681	106.5739773
**ENSG00000113552**	GNPDA1	592.0231391	662.1055693	670.1139396	586.9007907	492.366092	559.9674679	500.4833221	500.0551035	577.2927531	509.3859297	446.9918199	587.0027004
**ENSG00000113555**	PCDH12	4.218692202	31.52883663	12.21349829	6.697869223	1.155788948	4.173199286	0	1.661312636	4.220661883	0	0	0
**ENSG00000113558**	SKP1	2384.967325	2382.178768	2224.485156	2340.905293	2489.569395	2102.913059	2344.100458	2450.436138	2181.144269	2493.776334	2603.313469	2599.220891
**ENSG00000113569**	NUP155	6949.592288	6449.398694	6057.895153	7255.466836	6738.249568	6793.589056	5828.077216	5286.296808	6151.849176	6298.034979	5489.445837	6687.094164
**ENSG00000113575**	PPP2CA	2446.841477	2249.057013	2415.829962	2771.243391	2319.668419	2436.769001	2577.999806	2400.596759	2477.528525	2545.664093	2484.667493	2657.582831
**ENSG00000113578**	FGF1	80.15515185	35.0320407	100.9649192	22.60530863	24.27156791	44.38766514	8.171156279	3.322625272	33.76529506	6.327775524	2.759208765	21.14563042
**ENSG00000113580**	NR3C1	2678.869549	2648.422277	2524.937214	3048.36773	3540.181549	2711.441391	5247.92512	5032.115975	4753.403205	5211.555921	4987.269842	5196.750132
**ENSG00000113583**	C5orf15	1704.35165	1537.906587	1564.956248	964.4931681	989.3553397	1008.017319	1633.209861	2050.059793	1701.395701	1116.85238	1134.034802	1220.525788
**ENSG00000113593**	PPWD1	814.2075951	774.2080996	866.3441455	843.0942884	800.9617411	872.1986508	933.5546049	719.3483714	847.4151136	881.4591305	933.9921668	905.8788072
**ENSG00000113594**	LIFR	56.24922937	133.1217547	153.0758453	24.27977593	79.74943743	103.9506004	1292.064087	1490.197435	1663.878707	1235.81456	1598.961479	1875.194506
**ENSG00000113595**	TRIM23	500.6181414	683.1247937	573.2201865	544.2018744	520.1050267	577.4190285	565.8525723	576.4754847	535.0861343	505.5892644	533.906896	589.5401761
**ENSG00000113597**	TRAPPC13	542.8050634	525.4806106	535.7654584	437.0359668	384.8777198	461.7075938	553.5958379	413.6668464	525.2379232	540.3920297	397.3260621	543.8656144
**ENSG00000113600**	C9	0	7.006408141	1.628466439	2.511700959	9.246311586	1.13814526	3.064183605	3.322625272	1.875849726	0	1.379604382	0.845825217
**ENSG00000113615**	SEC24A	1340.13789	1832.175729	1482.718693	928.492121	1044.833209	1142.697841	1027.522902	1224.387413	1089.399728	973.2118756	1027.805265	1181.617828
**ENSG00000113621**	TXNDC15	434.5252969	357.3268152	507.2672957	509.0380609	506.2355593	543.6540525	514.7828456	476.7967266	531.8033973	556.2114685	538.0457091	622.5273595
**ENSG00000113638**	TTC33	444.368912	567.5190594	520.2950272	577.6912205	569.8039515	531.5138364	471.8842751	652.895866	505.5415011	482.8092725	493.8983689	638.5980387
**ENSG00000113643**	RARS	3394.640992	3254.476581	3195.865386	2242.948956	2272.281072	2292.603935	2350.228825	2473.694515	2355.598293	1976.797074	1781.069258	2134.862847
**ENSG00000113645**	WWC1	1351.387736	1408.288036	1648.008036	1630.093922	1643.531884	2006.170711	1720.028397	1280.872042	1730.940334	1836.320457	1327.179416	1774.541305
**ENSG00000113648**	H2AFY	4619.467962	4638.242189	4817.003726	4675.949951	4716.774698	4780.589473	4712.714384	4480.56018	4707.913849	4667.367226	4496.130682	4789.908203
**ENSG00000113649**	TCERG1	2902.460235	2157.973707	2600.660903	2978.877337	2318.51263	2748.241421	2821.091705	2357.402631	2681.527183	3048.08947	2435.001735	3003.525345
**ENSG00000113657**	DPYSL3	28.12461468	17.51602035	39.08319453	6.697869223	31.2063016	69.0474791	13.27812895	83.06563181	54.39964205	36.06832049	53.80457091	43.98291127
**ENSG00000113658**	SMAD5	1967.316797	2045.871177	1805.969281	2060.43202	2044.590649	1923.465489	2275.667024	2196.255305	2248.205896	2284.326964	2295.661692	2578.921086
**ENSG00000113712**	CSNK1A1	2884.179236	2263.069829	2687.783857	2978.040103	2556.605154	2676.917651	3443.120977	2902.313175	3236.778702	3287.279385	3035.129641	3161.69466
**ENSG00000113716**	HMGXB3	966.0805144	945.865099	1057.688952	985.4240094	858.7511886	1091.860686	902.9127688	920.3672004	991.3865801	1011.178529	903.6408704	943.0951167
**ENSG00000113719**	ERGIC1	2553.715013	2378.675564	2340.106273	2674.961521	2932.236562	2616.216571	2178.634543	3163.139259	2263.212694	2302.044736	3170.330871	2452.893129
**ENSG00000113721**	PDGFRB	4.218692202	0	0	0	6.93473369	2.655672273	21.44928523	23.25837691	13.13094808	29.10776741	35.86971394	17.76232955
**ENSG00000113732**	ATP6V0E1	1051.860589	1243.637445	1128.527242	1053.239935	1037.898476	1108.932865	832.4365459	1018.384646	1107.689263	898.5441244	982.2783202	1116.489286
**ENSG00000113734**	BNIP1	174.372611	175.1602035	206.0010045	190.0520392	214.9767444	187.0352044	173.6370709	117.9531972	175.8609118	205.019927	175.2097566	179.314946
**ENSG00000113739**	STC2	4298.847354	6810.228713	6386.03114	2436.34993	5548.942741	4577.620235	1437.102111	3138.21957	1474.417884	980.8052062	3061.342124	1174.851226

**ENSG00000113742**	CPEB4	3770.104598	3692.37709	4349.633858	3960.115178	2698.767194	4861.777169	1441.187689	1378.889488	1640.430585	1491.456691	1294.068911	1898.877612
**ENSG00000113749**	HRH2	16.87476881	56.05126513	38.26896131	49.39678552	87.83996007	79.29078644	2.04278907	4.983937908	3.28273702	0.632777552	4.138813147	3.383300867
**ENSG00000113758**	DBN1	367.0262216	290.7659378	337.0925529	1019.750589	883.0227565	888.1326845	96.01108628	114.6305719	141.6266543	164.5221636	165.5525259	185.2357225
**ENSG00000113761**	ZNF346	482.3371418	329.3011826	445.385571	513.2242292	440.3555893	450.7055229	509.6758729	579.79811	521.9551862	494.1992684	419.3997322	490.5786257
**ENSG00000113763**	UNC5A	552.6486785	539.4934268	656.2719749	563.4582484	587.1407857	762.9367059	12.25673442	1.661312636	21.10330942	10.75721839	4.138813147	26.22058172
**ENSG00000113790**	EHHADH	60.46792157	94.5865099	90.37988736	53.58295378	76.28207059	50.83715494	119.5031606	99.67875817	94.26144872	84.79219202	68.98021912	103.1906764
**ENSG00000113810**	SMC4	8122.38872	6214.684021	7591.096305	8448.524791	8251.177302	8097.144761	7318.291842	6361.166084	7542.791747	7144.691344	6925.613999	8713.691383
**ENSG00000113811**	SELENOK	375.463606	416.8812844	321.6221217	283.8222083	224.223056	265.1878456	347.2741419	388.7471569	342.342575	344.863766	322.8274255	355.246591
**ENSG00000113812**	ACTR8	233.4343019	290.7659378	338.7210193	327.3583583	291.258815	320.1981998	382.001556	465.1675381	387.3629684	425.2265152	440.093798	366.2423189
**ENSG00000113838**	TBCCD1	172.9663803	196.1794279	212.5148703	213.4945815	153.7199301	208.6599643	230.8351649	176.0991394	239.17084	196.1610412	211.0794705	234.293585
**ENSG00000113845**	TIMMDC1	749.5209813	707.6472222	826.4467177	718.3464742	854.1280328	725.3779123	824.2653896	966.8839542	910.7250419	869.436357	947.7882106	856.8209446
**ENSG00000113851**	CRBN	243.277917	241.7210809	254.8549977	247.8211612	279.7009255	206.004292	416.7289702	460.1836002	365.3217341	373.3387559	430.4365673	378.9296971
**ENSG00000113916**	BCL6	314.9956845	479.9389576	319.9936552	196.7499084	287.7914481	202.5898563	517.8470292	789.1235022	456.3004458	518.2448154	693.9410043	479.5828979
**ENSG00000113946**	CLDN16	25.31215321	49.04485699	24.42699658	10.88403749	9.246311586	19.72785117	22.47067977	11.62918845	22.97915914	18.98332657	16.55525259	21.99145564
**ENSG00000113966**	ARL 6.00	149.0604578	87.58010176	140.862347	176.6563008	189.5493875	126.3341239	262.4983955	235.9063943	174.4540245	173.3810494	187.626196	203.8438772
**ENSG00000113971**	NPHP3	172.9663803	98.08971397	97.70798633	89.58400086	131.7599401	97.12172885	161.3803365	154.5020752	165.5437383	158.8271656	248.3287888	155.6318399
**ENSG00000114013**	CD86	22.49969175	38.53524477	50.48245961	10.88403749	6.93473369	22.00414169	1.021394535	1.661312636	5.627549177	6.327775524	0	6.766601734
**ENSG00000114019**	AMOTL2	3919.165056	4357.985864	4808.861394	3811.924822	4078.779198	5026.42885	3378.773121	3315.980022	3393.881116	2792.447339	3301.393287	2702.411568
**ENSG00000114021**	NIT2	973.111668	844.272181	771.0788588	689.0432963	646.0860221	654.8129062	955.0038901	838.9628812	858.670212	948.533551	856.7343214	808.6089072
**ENSG00000114023**	FAM162A	181.4037647	416.8812844	205.1867713	298.0551804	453.0692677	276.1899164	124.6101333	493.4098529	180.0815737	198.0593739	464.9266768	198.7689259
**ENSG00000114026**	OGG1	213.7470716	171.6569995	176.6886086	251.1700959	225.3788449	262.1527915	283.9476807	378.779281	307.639355	357.5193171	326.9662386	268.1265937
**ENSG00000114030**	KPNA1	2079.815256	1797.143688	2099.09324	2252.158526	2127.807454	2464.843251	2190.891277	2103.221797	2114.551603	2141.952015	2250.134748	2306.565366
**ENSG00000114054**	PCCB	504.8368336	357.3268152	463.2987019	627.9252397	536.286072	544.0334342	686.3771274	501.7164161	676.7127886	713.1403015	682.9041692	727.4096864
**ENSG00000114062**	UBE3A	1809.818955	1933.768647	1676.506199	2006.849066	1826.146538	1950.022212	2227.661481	2207.884493	2010.910906	2267.24197	2217.024242	2485.034487
**ENSG00000114098**	ARMC8	614.5228308	585.0350798	552.0501228	627.9252397	524.7281825	590.3180081	795.6663427	850.5920697	791.6085843	837.1647018	797.411333	779.8508499
**ENSG00000114107**	CEP70	320.6206074	301.2755501	300.452058	384.2902467	379.098775	391.1425877	486.1837986	392.0697821	397.6801419	496.0976011	435.9549848	477.8912475
**ENSG00000114120**	SLC25A36	1375.293658	2014.34234	1319.872049	1178.824983	1703.63291	1305.073231	2102.029953	2530.179145	1921.808044	1926.174869	2346.707054	2013.064016
**ENSG00000114124**	GRK7	0	0	0	0	0	0	2.04278907	1.661312636	0	2.53111021	0	4.229126084
**ENSG00000114125**	RNF7	375.463606	423.8876925	425.8439738	406.8955553	424.174544	426.8044725	461.6703298	421.9734096	471.776206	514.4481501	413.8813147	501.5743535
**ENSG00000114126**	TFDP2	627.1789074	627.0735286	536.5796916	586.9007907	797.4943743	585.0066636	1339.048235	1164.580158	1169.592304	1534.485565	1318.901789	1378.695103
**ENSG00000114127**	XRN1	4234.160741	4540.152475	3923.789885	1839.402335	1944.037011	1672.69415	3163.258875	3104.993317	2852.698471	2383.67304	2377.058351	2309.948667
**ENSG00000114166**	KAT2B	102.6548436	178.6634076	74.09522297	101.305272	91.30732691	75.49696891	714.9761744	657.8798039	603.5546493	408.1415213	521.4904565	384.0046484
**ENSG00000114200**	BCHE	0	0	0.814233219	0	0	0	231.8565594	448.5544118	219.9433804	306.2643354	536.6661047	395.8462015
**ENSG00000114209**	PDCD10	1286.701122	1460.836097	1275.903455	786.9996337	1032.119531	944.6605657	910.0625306	1104.772903	806.1464197	784.644165	848.4566951	830.6003629
**ENSG00000114251**	WNT5A	2.812461468	0	3.256932878	0	0	4.173199286	37.79159779	36.54887799	61.90304095	36.70109804	26.21248326	88.81164776
**ENSG00000114268**	PFKFB4	84.37384405	788.2209158	85.49448804	113.0265431	577.8944741	75.49696891	218.5784305	1948.719722	228.3847041	215.7771454	1920.4093	223.2978572
**ENSG00000114270**	COL7A1	261.5589166	564.0158553	379.4326803	421.1285274	767.4438616	600.5613155	415.7075757	553.2171078	318.8944534	377.7681988	609.785137	316.3386311
**ENSG00000114279**	FGF12	43.59315276	70.06408141	91.19412058	93.77016912	137.5388848	152.1320831	10.21394535	4.983937908	13.13094808	10.12444084	12.41643944	38.90795997
**ENSG00000114302**	PRKAR2A	928.1122845	749.6856711	920.8977712	1503.671641	1621.571894	1599.852854	1478.979287	1501.826623	1640.430585	1779.370477	1648.627237	2018.984792
**ENSG00000114315**	HES1	271.4025317	493.9517739	327.3217542	223.5413853	286.6356592	302.7466391	171.5942819	171.1152015	161.3230764	168.3188289	166.9321303	167.4733929
**ENSG00000114316**	USP4	645.459907	606.0543042	676.6278054	553.4114445	503.9239814	583.8685183	794.6449481	735.9614978	857.2633247	780.2147221	801.5501461	745.172016
**ENSG00000114331**	ACAP2	1493.41704	1706.060382	1506.331456	1350.457882	1466.696175	1388.916599	1417.695614	1275.888105	1391.880497	1323.77064	1208.533439	1345.70792
**ENSG00000114346**	ECT2	2919.335004	2294.598666	2585.190472	3413.401603	2801.632411	3343.491392	2314.480016	1767.636645	2359.349993	2301.411958	2007.324376	2786.994089
**ENSG00000114353**	GNAI2	854.9882864	1089.496466	946.9532342	914.2591489	928.0985255	1006.879173	673.0989985	730.9775599	815.9946307	688.461977	764.3008278	717.2597838
**ENSG00000114354**	TFG	3656.199909	4007.665457	3739.773177	3106.974086	3474.301578	3346.905828	3816.951377	3915.713883	3589.907413	3511.282638	3878.067919	3851.042212
**ENSG00000114378**	HYAL1	43.59315276	45.54165292	81.42332194	122.2361133	124.8252064	84.22274923	44.94135953	26.58100218	40.3307691	45.55998377	44.14734023	31.29553302
**ENSG00000114383**	TUSC2	130.7794583	185.6698157	166.91781	200.098843	219.5999002	201.451711	252.2844501	244.2129575	308.5772799	240.4554699	179.3485697	298.5763015
**ENSG00000114388**	NPRL2	134.9981505	84.07689769	121.3207497	83.72336529	121.3578396	129.7485596	148.1022076	149.5181373	143.9714665	147.4371697	157.2748996	120.1071808
**ENSG00000114391**	RPL24	5000.556491	4571.681312	4759.193168	5374.202818	4830.042015	5079.921677	4617.724692	5596.962271	5414.640234	4994.513221	5390.114322	5831.119044
**ENSG00000114395**	CYB561D2	49.2180757	77.07048955	50.48245961	49.39678552	58.94523636	49.31962793	53.11251581	46.51675381	46.42728071	48.09109398	68.98021912	55.82446431
**ENSG00000114405**	C3orf14	75.93645964	31.52883663	69.20982365	45.21061725	53.16629162	57.6660265	51.06972674	41.5328159	21.10330942	32.90443272	11.03683506	28.75805737
**ENSG00000114416**	FXR1	2396.217171	2501.287706	2636.487165	2506.677557	2803.943988	2773.280617	2143.907129	2083.286046	2174.578795	2158.404231	2356.364285	2460.505556
**ENSG00000114423**	CBLB	262.9651473	290.7659378	436.4290056	267.0775353	417.2398103	372.5528817	298.2472042	297.3749619	290.2877451	304.9987802	393.187249	284.1972728
**ENSG00000114439**	BBX	4210.254818	5198.754841	4079.308429	3262.699545	3925.059268	3081.3386	5391.94175	6085.388186	5096.214743	4209.236278	4591.323384	4667.263546
**ENSG00000114446**	IFT57	195.466072	136.6249587	159.589711	187.5403382	201.107277	165.0310627	367.7020326	426.9573475	349.8459739	369.5420906	335.2438649	360.3215423
**ENSG00000114450**	GNB4	776.2393653	830.2593647	784.1065903	555.0859119	799.8059522	525.0643466	82.73295732	73.09775599	56.74445421	25.94387965	23.4532745	21.99145564
**ENSG00000114455**	HHLA2	0	21.01922442	7.328098975	3.348934611	4.623155793	6.449489806	0	0	0	0	0	0
**ENSG00000114473**	IQCG	215.1533023	280.2563256	195.4159727	177.4935344	277.3893476	169.5836437	250.241661	435.2639107	261.6810368	278.422123	307.6517773	270.6640694
**ENSG00000114480**	GBE1	959.0493607	1401.281628	999.0641603	1104.311188	1610.014005	1355.151623	861.0355929	2101.560485	1006.86234	992.1952021	2324.633384	1205.300934
**ENSG00000114491**	UMPS	741.0835969	707.6472222	805.276654	822.1634471	713.1217811	920.0007518	673.0989985	642.9279902	650.9198549	736.553071	644.2752465	756.1677438
**ENSG00000114503**	NCBP2	1065.922896	826.7561606	1051.98932	1147.010104	988.1995508	1052.025602	1027.522902	867.2051961	1001.234791	987.1329817	1000.213177	1064.893948
**ENSG00000114520**	SNX4	410.6193744	371.3396315	385.1323128	504.8518927	456.5366346	471.1921376	489.2479822	553.2171078	478.3416801	535.3298093	504.9352039	597.152603
**ENSG00000114529**	C3orf52	1095.453742	1310.198322	1322.314748	1019.750589	1055.23531	1125.24628	2976.343675	4246.315098	2369.667166	2773.464012	4109.841455	2357.314879
**ENSG00000114541**	FRMD4B	244.6841477	283.7595297	266.2542628	259.5424324	165.2778196	187.7939679	575.0451231	267.4713344	631.2234327	503.0581541	282.8188984	617.4524082
**ENSG00000114544**	SLC41A3	523.1178331	518.4742024	476.3264334	576.0167532	565.1807957	547.44787	272.7123408	362.1661547	326.3978523	316.3887762	372.4931832	369.6256197
**ENSG00000114554**	PLXNA1	814.2075951	686.6279978	974.6371637	904.2123451	982.420606	1250.062877	663.9064477	599.7338616	901.8147557	792.2374956	677.3857517	895.7289046
**ENSG00000114573**	ATP6V1A	1359.82512	1254.147057	1214.02173	1476.880164	1606.546638	1607.061107	1976.398425	1912.170844	2033.890065	2328.621393	2091.480244	2554.392155

**ENSG00000114626**	ABTB1	202.4972257	206.6890402	246.7126655	215.1690488	175.6799201	192.7259307	213.4714578	161.1473257	214.7847936	189.2004882	168.3117346	208.0730033
**ENSG00000114631**	PODXL2	433.1190661	381.8492437	398.9742775	479.7348831	496.9892478	509.5096947	191.000778	141.2115741	196.0262963	242.3538026	193.1446135	228.3728085
**ENSG00000114638**	UPK1B	6905.999135	5654.17137	5741.972664	8074.281348	7694.087029	7497.342209	5620.734125	6881.156939	5832.016798	5421.005291	6496.557036	6190.594762
**ENSG00000114646**	CSPG5	19.68723028	31.52883663	23.61276336	20.93084132	40.45261319	23.90105046	10.21394535	21.59706427	11.25509835	15.81943881	13.79604382	12.68737825
**ENSG00000114648**	KLHL18	194.0598413	171.6569995	264.6257963	216.0062824	221.9114781	294.7796223	283.9476807	299.0362745	358.7562601	309.4282231	271.7820633	352.7091154
**ENSG00000114650**	SCAP	417.650528	360.8300193	388.3892457	437.8732004	457.6924235	512.9241305	598.5371974	358.8435294	638.2578692	602.4042299	568.3970055	581.9277491
**ENSG00000114656**	KIAA1257	5.624922937	7.006408141	13.02773151	8.372336529	13.86946738	6.070108053	2.04278907	0	0	1.898332657	1.379604382	0
**ENSG00000114670**	NEK11	175.7788418	161.1473872	146.5619795	184.1914036	233.4693675	135.8186677	214.4928523	277.4392102	163.1989261	225.9015862	229.0143275	168.3192181
**ENSG00000114686**	MRPL3	2415.904401	1467.842506	2028.25495	2607.982829	2139.365343	2414.006096	1834.424585	1442.019368	1888.042749	1930.604312	1445.825393	2205.912165
**ENSG00000114698**	PLSCR4	30.93707615	80.57369362	35.82626166	2.511700959	4.623155793	3.41443578	302.3327823	400.3763453	287.005008	243.6193577	284.1985028	294.3471754
**ENSG00000114735**	HEMK1	309.3707615	287.2627338	304.5232241	250.3328622	305.1282823	243.5630856	443.2852281	511.6842919	460.9900701	413.8365193	470.4450944	318.0302815
**ENSG00000114737**	CISH	15.46853808	0	5.699632536	10.04680383	5.778944741	3.793817533	1.021394535	6.645250545	1.406887294	6.327775524	12.41643944	4.229126084
**ENSG00000114738**	MAPKAPK3	658.1159836	585.0350798	577.2913526	819.6517462	792.8712185	896.0997013	274.7551299	239.2290196	284.1912335	342.9654334	246.9491844	296.8846511
**ENSG00000114739**	ACVR2B	310.7769922	308.2819582	341.1637189	283.8222083	346.7366845	288.7095143	886.5704563	745.9293736	710.4780837	974.4774307	809.8277724	775.6217238
**ENSG00000114742**	WDR48	562.4922937	627.0735286	638.358844	438.7104341	579.0502631	595.6293527	870.2281437	935.3190141	766.7535754	737.1858485	943.6493975	816.2213342
**ENSG00000114744**	COMMD2	722.8025974	528.9838146	544.7220238	644.6699127	573.2713183	620.6685484	764.0031121	784.1395643	680.9334505	687.1964219	704.9778394	746.0178412
**ENSG00000114745**	GORASP1	322.0268381	332.8043867	344.4206518	374.2434428	397.5913982	394.5570234	680.2487602	760.8811873	731.5813931	681.5014239	818.1053987	636.9063882
**ENSG00000114757**	PEX5L	0	3.50320407	5.699632536	0.837233653	10.40210053	2.655672273	0	0	1.875849726	0	0	0
**ENSG00000114767**	RRP9	251.7153014	192.6762239	267.8827292	221.866918	188.3935986	277.3280617	139.9310513	91.37219499	234.9501782	176.5449371	125.5439988	237.6768859
**ENSG00000114770**	ABCC5	482.3371418	371.3396315	522.7377269	554.2486782	516.6376599	550.1035423	594.4516193	551.5557952	550.0929321	593.5453441	616.6831589	686.810076
**ENSG00000114779**	ABHD14B	119.5296124	143.6313669	132.7200148	101.305272	132.9157291	132.4042319	198.1505398	154.5020752	217.5985682	197.4265963	213.8386793	206.3813529
**ENSG00000114784**	EIF1B	246.0903785	343.3139989	293.9381922	293.8690122	258.8967244	284.9156967	333.9960129	380.4405937	336.2460634	341.6998783	297.9945466	377.2380467
**ENSG00000114790**	ARHGEF26	75.93645964	119.1089384	96.07951989	122.2361133	107.4883722	111.5382355	218.5784305	136.2276362	205.8745074	246.7832454	252.467602	266.4349433
**ENSG00000114796**	KLHL24	465.462373	1005.419568	524.3661933	405.221088	874.9322338	492.4375158	1027.522902	1091.482402	796.2982086	910.5668979	1048.499331	847.5168672
**ENSG00000114812**	VIPR1	52.03053716	35.0320407	45.59706029	102.9797393	72.81470374	35.66188481	3.064183605	3.322625272	2.813774589	3.163887762	6.898021912	8.458252168
**ENSG00000114841**	DNAH1	126.5607661	91.08330583	118.0638168	108.8403749	85.52838217	81.94645871	150.1449966	124.5984477	142.0956167	168.3188289	150.3768777	139.5611608
**ENSG00000114850**	SSR3	3677.29337	3443.649601	3748.729742	3174.790012	2766.958742	3320.349105	1312.491977	1177.870659	1424.707867	1318.708419	1233.366318	1575.772379
**ENSG00000114853**	ZBTB47	53.4367679	77.07048955	117.2495836	87.0722999	88.99574902	102.4330734	113.3747934	84.72694444	111.6130587	132.883286	81.39665856	132.794559
**ENSG00000114854**	TNNC1	11.24984587	0	0	0	3.467366845	1.517527013	3.064183605	0	0	3.163887762	5.518417529	1.691650434
**ENSG00000114857**	NKTR	1832.318647	1853.194953	1866.222539	1591.581174	1552.224558	1587.712638	2883.396772	2920.587614	2529.114393	2746.254577	2886.132368	2659.274482
**ENSG00000114859**	CLCN2	73.12399818	87.58010176	81.42332194	82.04889798	110.955739	74.35882365	94.98969174	94.69482026	74.5650266	77.83163894	110.3683506	104.0365017
**ENSG00000114861**	FOXP1	1057.485512	921.3426705	706.7544345	642.9954454	653.0207558	461.328212	1564.776427	1817.476024	1187.881839	1221.260676	1855.567894	951.5533689
**ENSG00000114867**	EIF4G1	9281.122845	7577.430404	9313.199564	10220.94843	9619.631416	10255.82694	6101.810951	5224.828241	6580.011876	6631.508749	5696.386495	6808.04717
**ENSG00000114902**	SPCS1	523.1178331	637.5831408	656.2719749	666.4379877	679.6039016	745.864527	764.0031121	822.3497549	922.4491027	841.5941447	830.5218381	948.170068
**ENSG00000114904**	NEK4	559.6798322	395.86206	442.1286382	495.6423225	451.9134788	468.1570836	648.5855296	506.700354	640.1337189	630.8792197	561.4989836	674.968523
**ENSG00000114923**	SLC4A3	32.34330689	17.51602035	21.98429693	36.00104707	56.63365846	39.45570234	45.96275407	43.19412854	32.35840777	39.8649858	60.70259282	37.21630954
**ENSG00000114933**	INO80D	1499.041963	1688.544362	1482.718693	1280.967489	1299.106778	1543.704354	1074.507051	913.7219499	1120.820211	995.9918674	895.3632441	1119.872587
**ENSG00000114942**	EEF1B2	2860.273313	2333.133911	2743.96595	2785.476363	2768.114531	2843.086859	2433.983177	2510.243393	2638.382639	2461.504679	2451.556987	2836.897777
**ENSG00000114956**	DGUOK	452.8062964	451.9133251	461.6702354	333.2189938	410.3050766	400.6271315	449.4135953	543.249232	506.9483884	433.4526234	453.8898418	528.6407605
**ENSG00000114978**	MOB1A	4024.632361	3937.601375	3697.43305	3498.799435	3346.009005	3385.223385	3932.368959	4336.02598	4110.455712	3914.994717	4018.787566	4279.029772
**ENSG00000114982**	KANSL3	997.0175905	1092.99967	1122.013376	963.6559345	1071.416355	1026.227643	1051.014976	1101.450278	895.2492816	1066.230176	1156.108472	947.3242428
**ENSG00000114988**	LMAN2L	243.277917	157.6441832	232.8707008	268.7520026	295.8819708	347.8930678	263.51979	294.0523366	312.3289793	324.6148844	357.317535	340.8675624
**ENSG00000114993**	RTKN	674.9907524	823.2529565	671.742406	896.6772422	753.5743943	866.5079245	479.0340369	393.7310948	441.7626104	484.0748276	376.6319964	428.8333849
**ENSG00000114999**	TTL	631.3975996	613.0607123	729.5529646	841.4198211	969.7069276	940.4873664	766.0459012	669.5089924	835.2220904	768.8247261	825.0034206	899.9580306
**ENSG00000115008**	IL1A	88.59253625	441.4037129	361.5195494	65.30422492	158.3430859	227.629052	83.75435186	104.6626961	48.77209287	73.40219608	100.7111199	61.74524082
**ENSG00000115009**	CCL20	0	0	0.814233219	0.837233653	2.311577897	0.758763507	0	3.322625272	0	3.163887762	0	1.691650434
**ENSG00000115020**	PIKFYVE	1174.202663	1226.121425	1213.207497	1087.566515	1011.31533	1158.252493	1316.577555	1229.371351	1218.833359	1266.82066	1179.561747	1291.575106
**ENSG00000115041**	KCNIP3	1.406230734	0	0	0	4.623155793	3.793817533	2.04278907	0	0.937924863	0	2.759208765	0.845825217
**ENSG00000115042**	FAHD2A	220.7782253	196.1794279	224.7283686	189.2148055	239.2483123	247.7362849	233.8993485	166.1312636	251.3638633	253.7437985	220.7367012	220.7603816
**ENSG00000115053**	NCL	11951.55501	9346.54846	10423.81368	13042.42584	10797.38035	11252.84218	8863.661774	8020.817407	9634.833154	9485.968288	8105.175746	10920.44937
**ENSG00000115073**	ACTR1B	282.6523776	378.3460396	240.1987997	291.3573112	317.8419608	333.8559429	299.2685987	332.2625272	360.6321098	301.8348925	383.5300183	265.5891181
**ENSG00000115084**	SLC35F5	1442.792733	1243.637445	1353.255611	1487.764201	1620.416105	1622.236377	2331.843723	2000.220414	2415.156522	2348.870274	2250.134748	2502.796816
**ENSG00000115091**	ACTR3	2826.523776	3086.322786	2952.409654	2666.589184	2676.807204	2980.423054	3472.741419	3468.820784	3355.426197	3292.341605	3357.957067	3423.900478
**ENSG00000115107**	STEAP3	94.21745919	77.07048955	121.3207497	69.49039319	205.7304328	140.3712487	9.192550814	29.90362745	43.61350613	32.27165517	55.18417529	51.59533822
**ENSG00000115109**	EPB41L5	410.6193744	416.8812844	443.7571046	435.3614995	344.4251066	429.8395265	183.8510163	204.3414542	149.5990156	179.0760473	180.7281741	134.4862095
**ENSG00000115112**	TFCP2L1	12.65607661	24.52242849	32.56932878	27.62871054	16.18104528	53.11344546	146.0594185	117.9531972	107.8613592	108.837739	124.1643944	118.4155303
**ENSG00000115128**	SF3B6	1442.792733	1334.720751	1256.361858	1387.296163	1328.001502	1329.353664	1075.528445	1129.692593	1111.440963	1118.117935	1142.312429	1261.125398
**ENSG00000115129**	TP53I3	112.4984587	101.592918	92.82258702	159.074394	205.7304328	175.27437	185.8938053	229.2611438	120.9923073	167.0532738	278.6800852	162.3984416
**ENSG00000115137**	DNAJC27	81.56138258	70.06408141	85.49448804	82.88613163	77.43785953	83.46398573	96.01108628	112.9692593	106.9234344	125.9227329	107.6091418	126.0279573
**ENSG00000115145**	STAM2	648.2723684	630.5767327	700.2405687	559.2720801	554.7786952	653.2953792	585.2590685	679.4768682	551.9687818	531.533144	613.9239501	530.3324109
**ENSG00000115155**	OTOF	1.406230734	0	0	0.837233653	1.155788948	0	4.08557814	29.90362745	2.813774589	0	2.759208765	0
**ENSG00000115159**	GPD2	2009.503719	2066.890402	1817.368546	1333.713209	1169.658416	1213.262847	2174.548965	1765.975332	2054.993375	1619.277757	1397.539239	1733.095869
**ENSG00000115163**	CENPA	732.6462125	357.3268152	688.0270704	798.7209048	621.8144542	704.5119159	622.0292717	493.4098529	747.9950782	648.5969912	515.972039	743.4803655
**ENSG00000115170**	ACVR1	251.7153014	234.7146727	328.1359874	195.9126748	217.2883223	229.9053425	127.6743169	162.8086383	158.0403394	170.8499391	198.6630311	154.7860147
**ENSG00000115183**	TANC1	1130.60951	1201.598996	964.866365	1147.010104	1104.934235	1083.893669	602.6227756	767.5264379	654.2025919	665.0492076	698.0798174	614.9149326
**ENSG00000115194**	SLC30A3	0	0	0	0	2.311577897	0.758763507	0	6.645250545	0.937924863	0	0	0
**ENSG00000115204**	MPV17	547.0237556	595.544692	504.0103628	621.2273704	573.2713183	589.9386264	556.6600215	644.5893028	587.1409642	727.6941852	685.663378	586.1568752

**ENSG00000115207**	GTF3C2	1095.453742	921.3426705	1254.733391	1424.134444	1312.976245	1451.135206	1155.197219	1231.032663	1194.916275	1345.917854	1335.457042	1277.196077
**ENSG00000115211**	EIF2B4	352.9639143	350.320407	358.2626166	425.3146957	448.4461119	386.9693884	301.3113878	315.6494009	332.4943639	284.7498986	274.5412721	385.6962988
**ENSG00000115216**	NRBP1	1403.418273	1621.983485	1509.588389	1602.465212	1700.165543	1616.545651	1333.941263	1785.911084	1528.817527	1326.30175	1683.117346	1525.022866
**ENSG00000115221**	ITGB6	952.018207	1607.970668	1232.749094	1069.147375	1759.110779	1858.591209	1292.064087	1559.972565	998.889979	1577.514438	1673.460116	1171.467925
**ENSG00000115226**	FNDC4	0	0	0.814233219	0	4.623155793	6.828871559	0	0	0	0	0	5.074951301
**ENSG00000115233**	PSMD14	1773.256956	1846.188545	2110.492505	1728.887493	1823.83496	1926.500543	1264.486434	1141.321781	1177.095703	1148.491258	1000.213177	1327.94559
**ENSG00000115234**	SNX17	947.7995148	910.8330583	962.4236654	919.2825508	1048.300576	1090.343159	1043.865215	873.8504466	1052.351696	966.8841	938.13098	1019.219386
**ENSG00000115239**	ASB3	369.8386831	332.8043867	401.4169772	307.2647506	349.0482624	368.7590642	284.9690752	289.0683987	266.3706611	291.0776741	369.7339745	324.7968832
**ENSG00000115241**	PPM1G	2082.627717	1730.582811	1941.946228	2168.435161	2029.565393	2198.51726	1651.594963	1523.423687	1650.747759	1858.467671	1514.805612	1925.944019
**ENSG00000115252**	PDE1A	0	10.50961221	0	0	2.311577897	0.758763507	2.04278907	1.661312636	0	0	1.379604382	0.845825217
**ENSG00000115255**	REEP6	15.46853808	24.52242849	13.84196473	4.186168264	23.11577897	14.41650663	16.34231256	9.967875817	17.35160996	20.24888168	22.07367012	13.53320347
**ENSG00000115257**	PCSK4	5.624922937	24.52242849	5.699632536	10.04680383	12.71367843	1.896908767	12.25673442	31.56494009	14.06887294	8.858885733	9.657230676	8.458252168
**ENSG00000115266**	APC2	1.406230734	0	1.628466439	4.186168264	5.778944741	7.587635066	1.021394535	0	0.468962431	1.265555105	0	0
**ENSG00000115267**	IFIH1	3282.142534	4350.979455	3592.396964	503.1774254	773.2228064	610.8046228	1701.643295	1619.77982	1744.071283	458.1309479	391.8076446	515.107557
**ENSG00000115268**	RPS15	3486.04599	3124.858031	3028.947576	3291.165489	3504.352091	3730.081398	2607.620248	2917.264989	3002.297486	2801.939002	2850.262654	2800.527293
**ENSG00000115271**	GCA	395.1508363	304.7787541	260.5546302	197.5871421	249.6504128	199.1754205	439.19965	368.8114052	465.6796944	309.4282231	244.1899757	348.4799893
**ENSG00000115274**	INO80B	1.406230734	14.01281628	0.814233219	15.07020575	4.623155793	5.6907263	11.23533988	4.983937908	2.813774589	5.062220419	4.138813147	1.691650434
**ENSG00000115275**	MOGS	756.552135	567.5190594	801.2054879	786.1624	685.3828463	917.3450795	657.7780805	571.4915468	765.8156506	663.7836524	728.4311139	735.0221134
**ENSG00000115282**	TTC31	295.3084542	287.2627338	277.6535278	312.2881525	336.3345839	330.0621254	341.1457746	294.0523366	382.6733441	398.649858	328.345843	353.5549406
**ENSG00000115286**	NDUFS7	134.9981505	171.6569995	131.9057816	198.4243757	142.1620406	164.6516809	201.2147234	117.9531972	160.854114	172.1154942	168.3117346	192.0023242
**ENSG00000115289**	PCGF1	168.7476881	122.6121425	182.3882412	157.3999267	153.7199301	201.8310928	151.1663912	131.2436983	141.1576919	143.0077268	126.9236032	121.7988312
**ENSG00000115290**	GRB14	88.59253625	38.53524477	69.20982365	111.3520758	69.3473369	73.97944189	13.27812895	3.322625272	11.72406079	5.062220419	5.518417529	5.074951301
**ENSG00000115295**	CLIP4	6036.948542	5535.062431	6089.650248	4161.888488	3430.381598	4730.1317	2441.132938	1797.540272	2381.860189	1728.748273	1251.301175	1631.596843
**ENSG00000115297**	TLX2	0	0	0	0	5.778944741	0.379381753	0	0	0	3.163887762	0	0
**ENSG00000115306**	SPTBN1	12768.57507	13203.57614	14625.25709	12901.77059	14514.39761	15721.57986	6822.915493	8951.152483	6801.362143	6289.808871	7765.793068	6317.468544
**ENSG00000115307**	AUP1	973.111668	924.8458746	968.9375311	999.6569815	937.344837	1096.792649	796.6877372	800.7526906	876.4907844	882.7246856	914.6777055	883.8873515
**ENSG00000115310**	RTN4	4813.527803	4564.674904	4466.883442	4345.242658	4341.14329	4559.789293	3156.109113	5862.772293	3460.004819	3060.745021	5246.635466	3385.838343
**ENSG00000115317**	HTRA2	251.7153014	196.1794279	312.6655563	262.0541333	299.3493376	296.2971493	226.7495867	199.3575163	250.8949008	294.8743394	255.2268107	218.2229059
**ENSG00000115318**	LOXL3	44.99938349	45.54165292	26.86969264	23.44254228	61.25681426	37.17941182	49.02693767	59.8072549	40.79973154	55.05164706	59.32298844	49.05786257
**ENSG00000115325**	DOK1	26.71838395	31.52883663	24.42699658	20.09360767	24.27156791	18.21032416	32.68462512	19.93575163	33.76529506	36.70109804	22.07367012	31.29553302
**ENSG00000115339**	GALNT3	1364.043812	1667.525138	1503.888756	970.3538037	924.6311586	1230.335026	705.7836236	725.993622	714.2297831	514.4481501	492.5187645	565.85707
**ENSG00000115350**	POLE4	164.5289959	185.6698157	211.7006371	226.0530863	276.2335586	264.0497003	193.0435671	277.4392102	177.2677991	161.9910534	215.2182836	231.7561094
**ENSG00000115353**	TACR1	0	0	9.770798633	5.86063557	18.49262317	17.83094241	16.34231256	68.11381808	32.8273702	22.14721433	57.94338406	21.14563042
**ENSG00000115355**	CCDC88A	130.7794583	262.7403053	279.2819943	195.9126748	406.8377098	346.7549225	11.23533988	3.322625272	7.034436472	8.858885733	20.69406573	9.304077385
**ENSG00000115363**	EVA1A	348.7452221	206.6890402	248.3411319	664.7635204	665.7344342	593.3530622	462.6917243	739.2841231	429.5695872	510.0187072	790.5133111	489.7328005
**ENSG00000115364**	MRPL19	1139.046895	917.8394665	916.8266051	1178.824983	1168.502627	1192.396851	971.3462027	1018.384646	979.6625193	1020.670192	900.8816616	1208.684235
**ENSG00000115365**	LANCL1	625.7726767	641.0863449	585.4336848	802.9070731	817.1427864	762.5573241	1285.935719	1368.921612	1171.468154	1235.181782	1187.839373	1301.725009
**ENSG00000115368**	WDR75	864.8319015	528.9838146	810.1620533	761.0453905	691.1617911	781.1470301	741.5324323	463.5062255	653.7336294	572.0309074	455.2694462	706.264056
**ENSG00000115380**	EFEMP1	2200.751099	1586.951444	1711.518227	3121.207058	2962.287074	2589.659848	1397.267724	971.8678921	1276.984701	1877.450998	1223.709087	1952.1646
**ENSG00000115392**	FANCL	946.3932841	826.7561606	824.0040181	803.7443068	680.7596905	759.5222701	674.120393	524.974793	591.3616261	592.9125666	613.9239501	590.3860013
**ENSG00000115414**	FN1	1954.66072	8912.151155	10865.12808	3287.816555	16787.83447	21725.2961	36195.15813	73237.30625	30446.44794	37352.85892	74607.62539	32339.28134
**ENSG00000115415**	STAT1	33116.73379	34411.97358	29011.12961	2340.06806	2866.356592	2642.393912	28132.26967	28685.88529	26136.68319	2903.18341	2781.282435	3058.503984
**ENSG00000115419**	GLS	2010.90995	1755.105239	2064.081211	1518.741846	1363.830959	1865.420081	2275.667024	2505.259455	2588.203659	2238.76698	2833.707401	3015.366898
**ENSG00000115421**	PAPOLG	658.1159836	490.4485699	604.1610488	542.5274071	554.7786952	501.9220596	603.6441701	573.1528595	559.4721807	479.6453847	629.0995983	641.1355143
**ENSG00000115423**	DNAH6	61.8741523	136.6249587	71.65252331	51.07125282	112.111528	49.31962793	75.58319558	68.11381808	54.39964205	55.68442461	66.22101035	49.05786257
**ENSG00000115425**	PECR	35.15576835	63.05767327	22.79853014	46.88508456	72.81470374	34.1443578	3.064183605	8.306563181	6.56547404	5.062220419	5.518417529	5.920776517
**ENSG00000115446**	UNC50	461.2436808	371.3396315	461.6702354	461.3157427	399.9029761	468.9158471	523.9753964	456.8609749	484.4381917	420.7970723	365.5951613	543.8656144
**ENSG00000115457**	IGFBP2	0	17.51602035	0.814233219	1.674467306	3.467366845	1.517527013	0	1.661312636	3.28273702	0	0	1.691650434
**ENSG00000115459**	ELMOD3	279.8399161	245.2242849	288.2385597	236.9371238	233.4693675	247.7362849	263.51979	237.567707	287.005008	296.7726721	269.0228546	249.5184389
**ENSG00000115461**	IGFBP5	251.7153014	171.6569995	65.13865756	727.5560443	418.3955993	222.6970892	2.04278907	0	2.813774589	6.960553076	1.379604382	8.458252168
**ENSG00000115464**	USP34	3751.823599	3517.216887	3641.250957	4017.8843	4053.351842	3872.728938	4205.0813	3967.214575	3715.589344	3963.718588	4599.601011	4284.104723
**ENSG00000115468**	EFHD1	50.62430643	66.56087734	74.09522297	130.6084498	86.68417112	119.5052523	679.2273657	1945.397097	631.6923952	759.3330629	1694.154181	616.606583
**ENSG00000115484**	CCT4	3976.820516	3387.598336	3777.227905	4113.328937	3953.953992	4328.366423	2775.128951	2668.068094	3113.441582	2864.58398	2539.851668	3376.534265
**ENSG00000115486**	GGCX	809.9889029	914.3362624	996.6214606	766.0687924	1021.71743	959.8358359	945.8113393	1044.965648	1068.296419	875.7641325	1051.258539	957.4741454
**ENSG00000115488**	NEU2	0	0	0	0.837233653	4.623155793	1.896908767	0	0	0	0	0	0
**ENSG00000115504**	EHBP1	1245.92043	1418.797649	1271.018056	1310.270667	1534.887723	1541.048682	480.0554314	471.8127887	563.6928426	634.675885	565.6377967	618.2982335
**ENSG00000115507**	OTX1	19.68723028	7.006408141	9.770798633	17.58190671	12.71367843	13.27836137	19.40649616	14.95181373	14.53783538	25.3111021	9.657230676	27.06640694
**ENSG00000115514**	TXNDC9	915.4562079	826.7561606	872.043778	778.6272972	717.7449369	753.8315438	574.0237286	536.6039815	545.8722702	581.5225706	634.6180159	628.4481361
**ENSG00000115520**	COQ10B	527.3365253	514.9709984	525.1804265	347.4519659	344.4251066	478.4003909	346.2527473	358.8435294	331.556439	302.46767	302.1333597	312.109505
**ENSG00000115524**	SF3B1	4782.590727	4985.059392	4463.626509	4054.722581	4164.307581	4201.273536	5308.187398	5311.216498	5277.234241	4976.795449	4959.677754	5381.985854
**ENSG00000115525**	ST3GAL5	43.59315276	35.0320407	24.42699658	38.51274803	42.76419109	31.10930377	183.8510163	269.1326471	143.0335416	141.1093942	186.2465916	98.96155036
**ENSG00000115526**	CHST10	0	0	0	1.674467306	1.155788948	1.13814526	0	0	1.875849726	0	0	0
**ENSG00000115539**	PDCL3	265.7776088	161.1473872	232.8707008	273.7754045	245.027257	212.0744001	218.5784305	217.6319553	239.6398025	246.1504679	212.4590749	249.5184389
**ENSG00000115540**	MOB4	397.9632978	367.8364274	374.5472809	414.4306582	396.4356093	411.2498206	345.2313528	310.665463	392.0525927	343.5982109	322.8274255	405.9961041
**ENSG00000115541**	HSPE1	431.7128354	388.8556518	365.5907155	491.4561542	471.5618909	386.5900066	243.0918993	191.0509532	317.0186037	336.6376579	223.4959099	417.8376571
**ENSG00000115548**	KDM3A	1274.045045	4533.146067	1406.995003	1010.541019	3487.015257	1035.332805	1454.465818	3937.310948	1356.708314	1207.33957	3530.407614	1346.553745
**ENSG00000115556**	PLCD4	0	0	0	0.837233653	2.311577897	0.758763507	2.04278907	1.661312636	0	1.265555105	2.759208765	0

**ENSG00000115561**	CHMP3	577.9608317	924.8458746	578.1055858	653.0422492	661.1112784	638.4994908	643.478557	819.0271296	762.5329135	690.3603096	727.0515095	706.264056
**ENSG00000115568**	ZNF142	584.9919854	423.8876925	703.4975016	687.368829	599.8544641	776.594449	537.2535253	498.3937908	596.9891752	556.2114685	499.4167864	605.6108552
**ENSG00000115590**	IL1R2	2960.115695	6677.106958	5360.097284	2511.700959	5384.82071	4499.467594	12.25673442	71.43644335	14.53783538	8.226108181	33.11050518	25.3747565
**ENSG00000115594**	IL1R1	92.81122845	217.1986524	237.7561001	133.9573845	226.5346339	237.1135958	73.54040651	139.5502614	81.13050064	91.7527451	118.6459769	138.7153356
**ENSG00000115596**	WNT6	0	0	0	0	2.311577897	3.035054026	87.83993	54.82331699	114.8957957	146.8043922	35.86971394	152.248539
**ENSG00000115598**	IL1RL2	14.06230734	0	20.35583049	15.9074394	25.42735686	19.72785117	2.04278907	3.322625272	3.751699452	3.796665314	5.518417529	8.458252168
**ENSG00000115602**	IL1RL1	0	10.50961221	4.071166097	0.837233653	1.155788948	1.13814526	0	0	0	0	0	0
**ENSG00000115604**	IL18R1	53.4367679	77.07048955	53.73939248	8.372336529	4.623155793	13.65774312	1.021394535	6.645250545	7.503398903	0	1.379604382	0.845825217
**ENSG00000115616**	SLC9A2	1.406230734	0	1.628466439	0	0	3.035054026	67.4120393	44.85544118	32.35840777	79.09719405	33.11050518	67.66601734
**ENSG00000115641**	FHL2	2358.248941	2431.223625	2286.36688	2407.883986	3173.796452	2914.410629	3252.120199	5422.524444	3078.738362	3173.379425	5297.680828	2919.788648
**ENSG00000115648**	MLPH	1492.010809	1317.20473	1378.496841	1219.849432	1137.296325	1170.013327	159.3375474	240.8903322	238.7018776	180.3416024	259.3656239	157.3234903
**ENSG00000115649**	CNPPD1	277.0274546	231.2114686	319.9936552	332.3817602	418.3955993	377.4848445	371.7876107	348.8756536	369.542396	358.1520946	320.0682167	347.6341641
**ENSG00000115652**	UXS1	538.5863712	707.6472222	516.2238611	823.8379144	871.464867	819.0852054	658.799475	760.8811873	753.6226273	754.2708424	813.9665856	883.8873515
**ENSG00000115657**	ABCB6	1.406230734	0	3.256932878	5.023401917	13.86946738	8.725780326	7.149761744	16.61312636	10.78613592	10.75721839	12.41643944	5.074951301
**ENSG00000115661**	STK16	132.185689	80.57369362	122.1349829	138.9807864	158.3430859	177.9300423	152.1877857	141.2115741	133.654293	158.8271656	137.9604382	192.8481494
**ENSG00000115677**	HDLBP	6599.440835	6635.068509	6542.363918	6373.022566	6911.617911	6516.640377	5065.095498	5523.864515	5564.708212	5266.607568	5592.916166	5615.433614
**ENSG00000115685**	PPP1R7	528.742756	641.0863449	649.7581091	735.9283809	713.1217811	680.9902472	534.1893417	534.9426688	506.9483884	511.2842623	550.4621485	641.1355143
**ENSG00000115687**	PASK	374.0573753	367.8364274	295.5666587	369.2200409	360.6061519	324.7507808	500.4833221	363.8274673	527.5827354	522.6742583	405.6036884	409.3794049
**ENSG00000115694**	STK25	516.0866794	378.3460396	407.9308429	396.8487515	401.058765	389.2456789	349.3169309	318.9720261	331.0874766	400.5481907	376.6319964	411.9168806
**ENSG00000115705**	TPO	0	0	0.814233219	2.511700959	2.311577897	1.517527013	0	0	0.468962431	0.632777552	1.379604382	0
**ENSG00000115738**	ID2	95.62368992	157.6441832	127.0203822	116.3754777	102.8652164	119.884634	1090.849363	1922.13872	1128.792573	878.9280203	1901.094839	1124.947538
**ENSG00000115750**	TAF1B	750.927212	697.13761	735.2525972	802.9070731	818.2985754	684.7840647	708.8478072	815.7045043	605.8994614	704.9141934	830.5218381	687.6559012
**ENSG00000115756**	HPCAL1	1147.484279	1078.986854	828.8894174	1070.821842	1037.898476	909.3780627	65.36925023	94.69482026	84.88220009	67.07442055	114.5071637	80.35339559
**ENSG00000115758**	ODC1	791.7079033	399.365264	731.9956643	1364.690854	1386.946738	1451.89397	659.8208695	274.116585	668.2714648	1010.545751	498.037182	1141.018217
**ENSG00000115760**	BIRC6	4686.967037	4266.902558	4648.45745	4603.110623	4589.637914	4904.267925	5741.258681	5763.093535	5259.882631	5340.009765	5583.258935	5354.073622
**ENSG00000115761**	NOL10	1161.546586	980.8971397	1248.219525	1289.339825	1124.582647	1314.178393	1129.662356	787.4621895	1019.524326	1008.647418	819.4850031	1001.457057
**ENSG00000115762**	PLEKHB2	1210.764662	1121.025303	1340.227879	1053.239935	1221.668918	1269.790728	510.6972674	488.425915	700.6298726	576.4603502	441.4734023	603.9192048
**ENSG00000115806**	GORASP2	1257.170276	1170.07016	1369.540275	1115.195226	1017.094274	1328.5949	1043.865215	1018.384646	1094.089353	1059.9024	945.0290019	1082.656277
**ENSG00000115808**	STRN	1875.911799	2224.534585	1713.960927	2092.246899	2183.285323	2073.321282	1808.889721	2129.8028	1715.464574	1754.059375	2248.755143	1996.993337
**ENSG00000115816**	CEBPZ	1537.010192	1303.191914	1500.631823	1733.910895	1433.178296	1596.8178	1215.459497	1025.029896	1173.812966	1257.961774	1113.340737	1354.166172
**ENSG00000115825**	PRKD3	1594.665653	1660.518729	1352.441378	1643.489661	1499.058266	1494.005345	1248.144122	1759.330082	1250.722805	1190.254576	1582.406227	1374.465977
**ENSG00000115827**	DCAF17	554.0549093	451.9133251	503.1961296	497.3167898	434.5766445	523.5468196	589.3446466	416.9894717	503.6656514	560.6409114	493.8983689	566.7028952
**ENSG00000115828**	QPCT	22.49969175	49.04485699	48.03975995	30.1404115	71.65891479	75.49696891	2.04278907	8.306563181	2.344812157	1.265555105	2.759208765	9.304077385
**ENSG00000115839**	RAB3GAP1	1309.200813	1397.778424	1365.469109	1063.286739	1279.458366	1215.539138	1317.59895	1383.873426	1178.50259	1238.34567	1294.068911	1190.07608
**ENSG00000115840**	SLC25A12	264.371378	217.1986524	184.8309408	313.9626198	295.8819708	249.6331937	308.4611495	212.6480174	270.1223605	313.857666	234.532745	246.9809633
**ENSG00000115841**	RMDN2	40.78069129	42.03844885	87.9371877	48.55955187	32.36209055	50.45777319	40.8557814	36.54887799	34.2342575	37.33387559	35.86971394	27.06640694
**ENSG00000115844**	DLX2	2.812461468	45.54165292	5.699632536	6.697869223	10.40210053	11.3814526	4.08557814	9.967875817	6.56547404	5.694997971	13.79604382	3.383300867
**ENSG00000115866**	DARS	1487.792117	2238.547401	1458.291696	1372.225957	1653.933985	1270.17011	1448.33745	2757.778976	1538.665738	1423.749493	2430.862922	1495.418983
**ENSG00000115875**	SRSF7	2532.621552	1660.518729	2089.322441	2377.743574	1890.870719	2133.263599	2158.206652	1950.381035	2265.557506	2221.049209	1909.372465	2397.91449
**ENSG00000115884**	SDC1	1513.10427	1289.179098	1372.797208	2498.30522	2350.874721	2584.348504	1849.745503	1817.476024	2538.962604	2546.929648	2385.335977	3017.058548
**ENSG00000115896**	PLCL1	1.406230734	0	2.442699658	0	0	0.758763507	5.106972674	0	4.689624315	4.429442867	2.759208765	0.845825217
**ENSG00000115902**	SLC1A4	452.8062964	553.5062431	608.2322149	417.7795928	634.5281326	642.6726901	480.0554314	523.3134804	548.2170824	454.9670602	456.6490505	452.516491
**ENSG00000115904**	SOS 1	2286.531174	2525.810135	2060.824278	1643.489661	1981.022257	1609.337398	3538.110669	2820.908856	2866.298381	2635.518506	2364.641911	2556.083805
**ENSG00000115919**	KYNU	215.1533023	651.5959571	390.8319453	110.5148422	153.7199301	209.4187278	85.79714093	78.0816939	83.94427523	80.9955267	70.3598235	80.35339559
**ENSG00000115935**	WIPF1	8.437384405	0	14.65619795	0.837233653	1.155788948	0.758763507	9.192550814	8.306563181	1.406887294	6.960553076	0	0.845825217
**ENSG00000115942**	ORC2	719.9901359	599.047896	695.3551694	765.2315587	596.3870973	694.2686085	653.6925023	569.8302342	593.7064382	638.4725504	529.7680828	634.3689126
**ENSG00000115944**	COX7A2L	937.9558997	952.8715072	1112.242578	1051.565468	1085.285822	1089.205014	794.6449481	945.28689	996.0762044	866.9052468	856.7343214	946.4784176
**ENSG00000115946**	PNO1	548.4299863	276.7531216	518.6665608	606.9943983	416.0840214	618.0128761	407.5364194	315.6494009	420.659301	501.792599	300.7537553	490.5786257
**ENSG00000115947**	ORC4	499.2119106	486.9453658	461.6702354	478.8976494	491.210303	443.4972696	592.4088302	581.4594226	557.596331	482.8092725	576.6746318	550.6322161
**ENSG00000115963**	RND3	4047.132053	4308.941007	4696.49721	3403.354799	2833.994501	3702.007149	1172.560926	1991.913851	1182.723252	975.1102082	1578.267413	981.1572515
**ENSG00000115966**	ATF2	496.3994492	416.8812844	565.0778543	458.8040418	483.1197804	506.8540224	602.6227756	722.6709967	609.1821985	641.0036606	689.8021912	615.7607578
**ENSG00000115970**	THADA	1293.732275	1005.419568	1106.542945	1352.969583	1221.668918	1290.277343	1564.776427	1217.742162	1472.542035	1554.101669	1345.114273	1555.472574
**ENSG00000115977**	AAK1	3400.265915	2504.79091	3191.79422	3703.084447	3408.421608	3698.592713	3909.89828	4789.56433	3942.567161	3866.903623	5043.833622	4106.481427
**ENSG00000115993**	TRAK2	704.5215978	728.6664466	760.493827	770.2549606	906.1385354	931.3822044	448.3922008	563.1849836	509.762163	649.2297687	666.3489167	693.5766778
**ENSG00000115998**	C2orf42	132.185689	185.6698157	166.91781	131.4456835	151.4083522	163.8929174	167.5087037	220.9545806	167.8885505	177.8104922	169.691339	186.9273729
**ENSG00000116001**	TIA1	1236.076815	1236.631037	1108.985645	875.7464009	809.0522638	904.8254816	1393.182146	1437.03543	1361.397939	1102.298496	1160.247286	1185.846954
**ENSG00000116005**	PCYOX1	971.7054373	879.3042217	1049.54662	1304.410031	1863.131785	1369.568129	1411.567247	2036.769292	1603.851516	1763.551038	2302.559714	2104.413139
**ENSG00000116016**	EPAS1	2272.468866	1776.124464	1907.748433	3350.609079	2933.392351	2777.074434	13100.4063	10391.51054	12380.60819	14925.95691	11693.52674	13506.98289
**ENSG00000116017**	ARID3A	753.7396735	1268.159873	778.4069578	885.7932047	1450.51513	910.5162079	777.281241	1702.845452	861.4839866	751.1069547	1696.91339	877.1207498
**ENSG00000116030**	SUMO1	1663.570959	1674.531546	1461.548629	1639.303492	1687.451864	1555.84457	1286.957114	1442.019368	1193.97835	1150.38959	1241.643944	1337.249668
**ENSG00000116035**	VAX2	7.031153671	0	1.628466439	2.511700959	2.311577897	5.311344546	22.47067977	3.322625272	17.35160996	21.51443678	11.03683506	15.2248539
**ENSG00000116039**	ATP6V1B1	1.406230734	0	0.814233219	0.837233653	0	1.13814526	18.38510163	1.661312636	21.10330942	32.27165517	9.657230676	20.2998052
**ENSG00000116044**	NFE2L2	1828.099954	2070.393606	2142.2476	1540.509921	1489.811954	1596.8178	1810.93251	1613.13457	1816.760459	1616.113869	1422.372118	1913.25664
**ENSG00000116062**	MSH6	2771.680777	3867.537294	2745.594416	2886.781635	3448.874222	2698.921793	2930.380921	3568.499542	2967.594266	2876.606753	3322.087353	3088.953692
**ENSG00000116095**	PLEKHA3	576.554601	392.3588559	474.6979669	536.6667715	569.8039515	600.9406972	554.6172324	531.6200436	576.3548283	653.6592116	593.2298844	647.0562908
**ENSG00000116096**	SPR	88.59253625	94.5865099	123.7634494	72.8393278	117.8904727	136.9568129	101.118059	74.75906863	124.7440068	140.4766166	157.2748996	151.4027138
**ENSG00000116106**	EPHA4	379.6822982	98.08971397	224.7283686	441.2221351	305.1282823	336.1322334	595.4730138	466.8288508	491.9415906	670.7442055	612.5443457	619.1440587

**ENSG00000116117**	PARD3B	7.031153671	0	14.65619795	10.88403749	2.311577897	9.484543833	10.21394535	9.967875817	16.88264753	21.51443678	53.80457091	21.99145564
**ENSG00000116120**	FARSB	1143.265587	819.7497525	1021.86269	1275.106853	968.5511386	1143.456604	1007.095011	709.3804956	990.9176177	1121.281823	808.448168	1004.840358
**ENSG00000116127**	ALMS1	1614.352883	1699.053974	1501.446057	1591.581174	1659.71293	1445.823862	2080.580668	2020.156166	1898.828885	1902.129322	1907.992861	1925.944019
**ENSG00000116128**	BCL9	300.9333771	329.3011826	326.507521	466.3391446	420.7071772	379.7611351	300.2899933	340.5690904	333.4322888	318.2871088	285.5781071	306.1887285
**ENSG00000116133**	DHCR24	2259.81279	2077.400014	2088.508208	2268.903199	2785.451365	2367.342141	662.8850531	1543.359439	1243.688368	827.040261	1626.553567	1519.947915
**ENSG00000116138**	DNAJC16	676.3969831	952.8715072	701.8690352	732.5794463	792.8712185	742.4500912	898.8271907	860.5599455	791.6085843	908.0357877	826.383025	830.6003629
**ENSG00000116141**	MARK1	798.739057	805.7369362	653.015042	1156.219675	959.3048271	957.1801636	1208.309735	1295.823856	1246.03318	1368.697846	1438.927371	1355.011997
**ENSG00000116151**	MORN1	316.4019152	248.727489	233.684934	237.7743574	268.143036	192.7259307	395.279685	398.7150327	405.6525032	387.8926396	466.3062812	413.608531
**ENSG00000116161**	CACYBP	2300.593481	2231.540993	2127.591402	2496.630753	2363.588399	2214.071912	1558.64806	1525.085	1708.8991	1625.605532	1638.970006	2168.695856
**ENSG00000116171**	SCP2	922.4873616	977.3939356	924.9689373	1058.263337	1212.422607	1177.221581	1354.369153	1252.629728	1263.853753	1435.139489	1433.408953	1396.457433
**ENSG00000116183**	PAPPA2	30.93707615	63.05767327	35.82626166	27.62871054	27.73893476	35.28250306	14.29952349	26.58100218	13.59991051	18.35054902	13.79604382	10.1499026
**ENSG00000116191**	RALGPS2	1847.787185	1828.672525	1682.205831	1697.909848	1578.807703	1556.223952	598.5371974	757.5585621	605.430499	634.675885	929.8533537	765.4718212
**ENSG00000116194**	ANGPTL1	0	0	0	0	3.467366845	0	6.128367209	0	0.468962431	0	0	0
**ENSG00000116198**	CEP104	781.8642882	812.7433443	833.7748167	709.136904	862.2185554	890.408975	773.1956629	868.8665087	797.7050959	828.3058161	891.224431	896.5747298
**ENSG00000116199**	FAM20B	1885.755415	1842.685341	1997.314087	2480.723313	2451.428359	2398.072063	1836.467374	1586.553568	1768.926291	1841.382677	1743.819939	2106.950615
**ENSG00000116205**	TCEANC2	327.6517611	462.4229373	380.2469135	323.17219	491.210303	400.6271315	421.8359429	468.4901634	453.0177088	439.1476214	467.6858856	497.3452275
**ENSG00000116209**	TMEM59	1965.910566	2161.476911	1985.914822	1603.302445	2057.304328	2027.036708	2509.566372	2983.717494	2559.596951	2480.488005	2719.200238	2334.477598
**ENSG00000116212**	LRRC42	721.3963666	735.6728548	561.8209214	626.2507723	589.4523636	531.5138364	692.5054946	812.3818791	823.9669921	661.8853198	706.3574437	764.625996
**ENSG00000116213**	WRAP73	371.2449138	413.3780803	315.9224891	316.4743208	297.0377597	324.3713991	269.6481572	315.6494009	331.556439	308.162668	293.8557334	246.1351381
**ENSG00000116221**	MRPL37	1202.327278	1247.140649	1147.254606	1406.552537	1178.904727	1460.999132	992.7954879	1071.54665	1141.923521	1079.518504	1120.238758	1254.358796
**ENSG00000116237**	ICMT	2130.439562	1954.787871	2024.998017	2559.423277	2563.539887	2650.74031	2256.260528	2187.948742	2616.341405	2381.14193	2243.236726	2908.79292
**ENSG00000116251**	RPL22	1143.265587	858.2849973	943.6963013	1121.055861	1191.618406	1181.774162	1342.112419	1290.839918	1400.32182	1378.822287	1383.743195	1357.549473
**ENSG00000116254**	CHD5	18.28099954	49.04485699	26.05546302	10.04680383	4.623155793	1.13814526	35.74880872	31.56494009	25.3239713	41.1305409	57.94338406	21.99145564
**ENSG00000116260**	QSOX1	856.3945171	1411.79124	895.6565414	1006.354851	1842.327584	1282.310326	764.0031121	2066.672919	804.2705699	848.5546977	2040.434881	786.6174516
**ENSG00000116266**	STXBP3	1019.517282	1163.063751	948.5817007	990.4474113	935.0332591	982.9781228	1220.566469	1393.841302	1061.261982	1134.570151	1240.26434	1161.318023
**ENSG00000116273**	PHF13	582.1795239	826.7561606	670.1139396	686.5315954	776.6901732	693.1304633	612.8367209	694.4286819	736.2710174	677.071981	813.9665856	809.4547325
**ENSG00000116285**	ERRFI1	2809.649007	6263.728878	2679.641525	2027.779907	4802.30308	2171.581156	4724.971118	7244.984406	4756.21698	4212.400166	7029.084328	4481.181998
**ENSG00000116288**	PARK7	1742.31988	1611.473872	1740.830623	2153.364955	2054.99275	2081.288299	1683.258193	1583.230942	1876.787651	1738.239936	1644.488424	2019.830618
**ENSG00000116299**	KIAA1324	1.406230734	17.51602035	12.21349829	5.86063557	10.40210053	14.79588838	135.8454731	58.14594226	30.95152048	88.58885733	64.84140597	44.82873649
**ENSG00000116329**	OPRD1	0	0	4.071166097	0.837233653	1.155788948	0	0	0	0.937924863	0	0	2.53747565
**ENSG00000116337**	AMPD2	390.9321441	367.8364274	333.0213868	385.964714	479.6524135	484.0911172	337.0601965	234.2450817	404.2456159	346.1293212	369.7339745	378.9296971
**ENSG00000116350**	SRSF4	2136.064485	1905.743014	2003.827953	2071.316057	1848.106528	1800.545801	2383.934844	2383.983633	2193.806254	2269.77308	2396.372812	2201.683039
**ENSG00000116353**	MECR	357.1826065	241.7210809	304.5232241	350.8009006	337.4903729	365.3446284	394.2582905	284.0844608	401.9008038	417.000407	339.382678	439.8291127
**ENSG00000116396**	KCNC4	8.437384405	17.51602035	18.72736405	15.9074394	60.10102531	29.59177676	215.5142469	235.9063943	214.3158312	332.208215	300.7537553	332.4093102
**ENSG00000116406**	EDEM3	963.2680529	914.3362624	1081.301715	1100.12502	1206.643662	1336.182535	1192.988817	1157.934907	1299.96386	1314.911754	1146.451242	1574.080728
**ENSG00000116455**	WDR77	368.4324523	164.6505913	292.3097258	420.2912937	307.4398602	482.1942085	285.9904698	189.3896405	316.0806788	358.7848722	227.6347231	355.246591
**ENSG00000116459**	ATP5PB	1964.504336	1961.794279	1886.578369	2505.003089	2406.35259	2346.476144	1883.451522	2018.494853	2047.489976	2285.592519	2159.080858	2542.550602
**ENSG00000116473**	RAP1A	866.2381322	795.227324	744.2091626	786.1624	907.2943244	855.8852355	723.1473307	689.444744	696.4092107	713.1403015	794.6521242	795.921529
**ENSG00000116478**	HDAC1	2145.9081	2319.121095	2182.959261	2083.874562	2024.942237	1901.081966	1840.552952	1618.118508	1783.933089	1814.80602	1505.148381	1970.772755
**ENSG00000116489**	CAPZA1	5782.420779	6326.786551	5400.808945	5202.569919	5247.281825	5116.342325	4123.369737	4091.813023	3930.374138	3896.644168	3871.169897	4123.397932
**ENSG00000116497**	S100PBP	1525.760347	1772.62126	1469.690961	1352.969583	1542.978246	1248.924732	1645.466596	1880.605904	1620.734163	1663.572185	1971.454662	1574.080728
**ENSG00000116514**	RNF19B	1842.162262	2778.040828	2160.160731	632.1114079	844.8817212	798.5985907	1276.743169	1242.661852	1112.84785	792.2374956	797.411333	901.6496811
**ENSG00000116521**	SCAMP3	666.553368	634.0799367	829.7036506	776.9528299	828.7006759	978.8049235	490.2693767	318.9720261	597.4581377	482.1764949	451.130633	498.1910527
**ENSG00000116525**	TRIM62	132.185689	105.0961221	144.9335131	172.4701325	158.3430859	171.4805525	241.0491102	257.5034586	282.3153837	208.8165923	253.8472063	214.8396051
**ENSG00000116539**	ASH1L	3294.79861	3138.870847	3321.257302	3667.0834	3735.509881	3473.239952	4268.407761	4201.459657	3892.857143	4208.603501	4623.054285	4204.597153
**ENSG00000116544**	DLGAP3	0	0	0	0	0	2.27629052	6.128367209	0	0.937924863	2.53111021	2.759208765	0
**ENSG00000116560**	SFPQ	8475.352635	6708.635795	7738.472518	9240.547827	7707.956496	7947.288968	9787.002433	8912.942293	9542.916518	9778.944294	8543.88994	10159.20668
**ENSG00000116574**	RHOU	28.12461468	45.54165292	33.383562	16.74467306	43.91998003	34.52373955	1456.508607	819.0271296	1298.556973	1413.625052	916.0573099	1387.999181
**ENSG00000116580**	GON4L	1476.542271	1135.038119	1488.418325	1350.457882	1347.649914	1365.015548	1502.471361	1550.00469	1241.812518	1380.720619	1357.530712	1356.703648
**ENSG00000116584**	ARHGEF2	1590.44696	1527.396975	2167.48883	1580.697137	1191.618406	1866.178845	914.1481087	747.5906863	1007.800265	933.3468898	769.8192453	992.1529793
**ENSG00000116586**	LAMTOR2	227.8093789	269.7467134	262.1830967	242.7977593	249.6504128	296.6765311	283.9476807	229.2611438	233.5432909	227.7999189	179.3485697	262.2058172
**ENSG00000116604**	MEF2D	864.8319015	872.2978135	984.4079623	857.3272605	950.0585155	1035.332805	542.360498	480.1193518	548.6860448	539.1264746	506.3148083	529.4865857
**ENSG00000116641**	DOCK7	1354.200197	1671.028342	1390.710339	1442.553584	1923.23281	1551.291989	1674.065643	2339.128192	1579.934432	1554.734446	1839.012642	1666.275677
**ENSG00000116649**	SRM	541.3988327	290.7659378	539.0223913	695.7411655	591.7639415	746.6232905	308.4611495	126.2597603	450.6728966	353.7226518	190.3854048	438.9832875
**ENSG00000116652**	DLEU2L	40.78069129	38.53524477	39.89742775	33.48934611	49.69892478	21.24537819	74.56180105	46.51675381	48.77209287	56.31720216	42.76773585	41.44543562
**ENSG00000116661**	FBXO2	52.03053716	136.6249587	65.95289078	52.74572013	109.7999501	76.63511417	38.81299233	66.45250545	52.52379232	75.93330629	120.0255813	90.5032982
**ENSG00000116663**	FBXO6	63.28038304	73.56728548	92.0083538	17.58190671	23.11577897	22.38352344	33.70601965	41.5328159	51.58586746	11.38999594	13.79604382	10.99572782
**ENSG00000116667**	C1orf21	1109.516049	1212.108608	842.7313821	929.3293547	923.4753697	587.6623359	3286.847613	3920.697821	2766.878346	3437.247665	3953.94616	2773.460886
**ENSG00000116668**	SWT1	143.4355349	185.6698157	162.8466439	56.93188839	137.5388848	81.94645871	132.7812895	196.0348911	98.01314817	101.8771859	165.5525259	125.1821321
**ENSG00000116670**	MAD2L2	196.8723028	189.1730198	166.91781	243.634993	303.9724934	323.2332538	193.0435671	166.1312636	237.7639527	217.0427005	212.4590749	266.4349433
**ENSG00000116675**	DNAJC6	313.5894537	276.7531216	354.1914505	262.0541333	391.8124535	412.7673476	218.5784305	237.567707	332.9633263	286.6482312	235.9123494	319.7219319
**ENSG00000116678**	LEPR	684.8343675	756.6920792	670.1139396	589.4124916	646.0860221	533.4107451	207.3430906	187.7283279	235.4191406	163.2566085	175.2097566	193.6939746
**ENSG00000116679**	IVNS1ABP	1465.292425	1905.743014	1502.26029	1520.416314	1913.986498	1455.308406	1627.081494	2252.739935	1560.706972	1704.069949	2353.605076	1865.044603
**ENSG00000116685**	KIAA2013	509.0555258	504.4613861	510.5242286	519.0848648	721.2123037	585.0066636	435.1140719	486.7646024	517.7345243	496.0976011	582.1930493	511.7242561
**ENSG00000116688**	MFN2	2951.678311	3457.662418	2928.79689	2593.749857	3063.996502	2841.948714	2601.49188	3998.779515	2716.230403	2520.352991	3592.489812	2716.790596
**ENSG00000116690**	PRG4	4.218692202	0	0	2.511700959	2.311577897	0.758763507	3.064183605	0	1.406887294	3.796665314	0	0
**ENSG00000116691**	MIIP	146.2479964	45.54165292	157.9612446	128.9339825	116.7346838	154.7877553	60.26227756	103.0013834	123.3371195	84.79219202	122.78479	92.19494863

**ENSG00000116698**	SMG7	4404.314659	3478.681642	4282.866734	3923.276897	3454.653166	3885.248536	4081.492561	3879.165005	4155.945068	3266.397725	3458.668186	3744.468235
**ENSG00000116701**	NCF2	614.5228308	977.3939356	916.8266051	210.9828805	213.8209554	253.806393	54.13391035	88.04956971	121.9302322	18.35054902	30.35129641	71.89514343
**ENSG00000116704**	SLC35D1	464.0561423	343.3139989	526.808893	718.3464742	659.9554895	716.2727502	1185.839055	913.7219499	1203.357599	1357.30785	985.037529	1415.911413
**ENSG00000116711**	PLA2G4A	322.0268381	192.6762239	270.3254289	381.7785457	182.6146538	374.8291723	515.8042401	377.1179684	789.7327346	556.8442461	246.9491844	882.1957011
**ENSG00000116717**	GADD45A	1102.484896	732.1696507	1195.294366	993.796346	522.4166046	1209.46903	357.4880872	466.8288508	397.2111794	275.2582353	237.2919538	365.3964936
**ENSG00000116726**	PRAMEF12	4.218692202	3.50320407	4.885399317	4.186168264	2.311577897	0.758763507	3.064183605	0	0.468962431	0	0	0
**ENSG00000116729**	WLS	5248.0531	5002.575413	5178.523276	4896.142402	4244.057018	4583.310962	1954.94914	1960.348911	2524.424769	1750.26271	1734.162709	2289.648862
**ENSG00000116731**	PRDM2	1409.043196	1534.403383	1597.525577	1621.721586	1665.491874	1619.960087	1641.381018	1704.506765	1423.300979	1558.531112	1696.91339	1457.356849
**ENSG00000116741**	RGS2	597.648062	1012.425976	712.454067	1162.917544	412.6166545	959.8358359	88.86132453	106.3240087	82.0684255	63.91053279	82.77626294	159.0151408
**ENSG00000116745**	RPE65	5.624922937	7.006408141	1.628466439	8.372336529	8.090522638	3.41443578	0	0	0	0	0	0.845825217
**ENSG00000116747**	TROVE2	2535.434014	2844.601705	2158.532265	2635.611539	2806.255566	2741.412549	2517.737528	2477.01714	2557.252139	2346.339164	2481.908284	2751.46943
**ENSG00000116750**	UCHL5	1667.789651	1299.68871	1494.117958	1771.586409	1408.906728	1571.778604	1292.064087	1139.660468	1210.860998	1275.679546	1171.284121	1306.79996
**ENSG00000116752**	BCAS2	833.8948254	896.820242	878.5576438	870.722999	797.4943743	858.5409077	541.3391035	706.0578704	614.3407852	551.7820257	554.6009617	558.2446431
**ENSG00000116754**	SRSF11	3917.758825	3321.037459	3406.75179	3860.484373	3410.733186	3242.196464	4415.488574	3826.003001	3875.036571	4366.165111	3818.74493	4159.768416
**ENSG00000116761**	CTH	1354.200197	973.8907316	996.6214606	611.1805666	585.9849968	611.1840046	221.6426141	210.9867048	315.6117164	107.5721839	92.43349361	191.156499
**ENSG00000116771**	AGMAT	15.46853808	17.51602035	8.142332194	6.697869223	9.246311586	3.41443578	0	0	0	0	1.379604382	0
**ENSG00000116774**	OLFML3	10169.86067	8116.923831	9658.434449	12576.92393	12633.92899	12241.13165	85.79714093	250.8582081	309.5152048	61.37942258	306.2721729	313.8011554
**ENSG00000116783**	TNNI3K	0	0	0	5.023401917	0	0	0	1.661312636	2.344812157	1.265555105	6.898021912	0
**ENSG00000116785**	CFHR3	47.81184496	28.02563256	11.39926507	43.53614995	27.73893476	12.89897961	0	0	7.972361335	0	0	2.53747565
**ENSG00000116786**	PLEKHM2	892.9565162	984.4003438	893.2138417	899.1889432	1037.898476	1134.351442	1055.100555	927.012451	1110.034075	1058.636845	1015.388825	1058.127346
**ENSG00000116791**	CRYZ	111.092228	119.1089384	61.06749146	85.39783259	112.111528	58.04540826	103.160848	117.9531972	83.00635037	94.91663286	129.6828119	110.8031034
**ENSG00000116793**	PHTF1	532.9614482	588.5382838	590.3190841	442.0593687	617.1912984	532.6519816	579.1307013	626.3148638	493.3484779	503.6909317	549.0825442	509.1867805
**ENSG00000116809**	ZBTB17	188.4349184	206.6890402	175.0601422	192.5637402	209.1977996	233.3197783	279.8621026	230.9224564	252.3017881	248.0488005	257.9860195	214.8396051
**ENSG00000116815**	CD58	233.4343019	336.3075908	271.9538953	277.1243391	376.7871971	321.7157268	100.0966644	174.4378268	138.3439173	116.4310696	129.6828119	119.2613556
**ENSG00000116819**	TFAP2E	11.24984587	7.006408141	0.814233219	4.186168264	4.623155793	1.13814526	0	4.983937908	5.158586746	5.694997971	2.759208765	4.229126084
**ENSG00000116830**	TTF2	1662.164728	1029.941997	1726.988658	2125.736245	1709.411854	1867.696372	2063.21696	1412.115741	2096.262069	2286.225297	1786.587675	2517.175845
**ENSG00000116833**	NR5A2	97.02992066	63.05767327	138.4196473	128.9339825	138.6946738	155.5465189	0	0	1.406887294	0	0	0
**ENSG00000116852**	KIF21B	32.34330689	21.01922442	50.48245961	10.88403749	24.27156791	32.62683078	3.064183605	0	2.813774589	0	0	0.845825217
**ENSG00000116857**	TMEM9	194.0598413	161.1473872	170.1747429	267.0775353	305.1282823	325.5095443	213.4714578	229.2611438	234.9501782	198.0593739	338.0030737	202.1522268
**ENSG00000116863**	ADPRHL2	452.8062964	525.4806106	488.5399317	320.660489	344.4251066	320.9569633	350.3383255	508.3616667	385.9560811	267.0321271	384.9096227	250.3642642
**ENSG00000116871**	MAP7D1	2504.496938	2305.108278	2822.132339	2627.239203	2466.453616	2967.524074	1445.273267	1375.566863	1775.960728	1491.456691	1463.76025	1634.980144
**ENSG00000116874**	WARS2	185.6224569	171.6569995	162.8466439	278.7988064	138.6946738	195.381603	283.9476807	215.9706427	258.8672622	311.3265558	259.3656239	301.1137772
**ENSG00000116885**	OSCP1	66.09284451	59.5544692	70.02405687	67.81592588	87.83996007	55.01035423	222.6640086	264.1487091	155.2265648	211.3477025	318.6886123	189.4648486
**ENSG00000116898**	MRPS15	1191.077432	1156.057343	1240.891426	1368.039789	1315.287823	1306.97014	994.838277	903.7540741	947.3041115	990.2968695	871.9099696	1177.388702
**ENSG00000116903**	EXOC8	798.739057	819.7497525	866.3441455	753.5102876	841.4143543	865.3697793	666.9706313	722.6709967	743.7744163	649.8625463	691.1817955	760.3968699
**ENSG00000116906**	GNPAT	795.9265955	795.227324	810.1620533	821.3262135	970.8627165	762.1779424	790.55937	819.0271296	766.284613	777.6836119	848.4566951	844.1335663
**ENSG00000116918**	TSNAX	610.3041386	784.7177118	656.2719749	590.2497253	529.3513383	567.1757212	516.8256346	694.4286819	529.4585851	610.630338	518.7312477	623.3731848
**ENSG00000116922**	C1orf109	569.5234473	416.8812844	478.769133	587.7380243	447.290323	567.9344847	505.5902948	393.7310948	572.1341664	523.3070358	376.6319964	616.606583
**ENSG00000116954**	RRAGC	527.3365253	532.4870187	555.3070557	384.2902467	480.8082025	410.4910571	327.8676457	501.7164161	413.1559021	314.4904435	346.2807	354.4007658
**ENSG00000116962**	NID1	80.15515185	98.08971397	83.05178838	30.97764516	10.40210053	18.96908767	12.25673442	38.21019063	41.26869397	17.08499391	17.93485697	28.75805737
**ENSG00000116977**	LGALS8	1618.571575	2655.428685	1840.981309	915.9336162	1466.696175	948.4543833	2545.315181	3475.466035	2588.203659	1815.438798	2651.599623	2089.188285
**ENSG00000116983**	HPCAL4	1.406230734	0	2.442699658	7.535102876	5.778944741	6.449489806	1.021394535	0	0	1.265555105	0	1.691650434
**ENSG00000116984**	MTR	1669.195881	2329.630707	1695.233563	1977.545888	2072.329584	1762.228244	2958.979968	3266.140643	2705.444267	3057.581133	3338.642605	2906.255445
**ENSG00000116985**	BMP8B	122.3420739	192.6762239	120.5065165	158.2371604	181.4588649	169.9630255	208.3644851	191.0509532	174.9229869	206.2854821	204.1814486	221.6062068
**ENSG00000116990**	MYCL	59.06169083	98.08971397	60.25325824	117.2127114	130.6041512	99.39801937	598.5371974	463.5062255	680.464488	764.3952833	662.2101035	832.2920133
**ENSG00000116991**	SIPA1L2	167.3414574	168.1537954	57.81055858	264.5658343	154.8757191	124.8165968	1192.988817	1550.00469	1353.89454	1135.835707	1307.864954	1377.003453
**ENSG00000116996**	ZP4	137.8106119	476.4357536	300.452058	66.14145858	143.3178296	89.53409378	1.021394535	13.29050109	6.56547404	0	0	5.920776517
**ENSG00000117000**	RLF	1494.82327	2970.717052	1599.968276	1528.78865	2653.691425	1364.636167	1635.25265	3945.617511	1606.66529	1554.101669	3264.143969	1772.003829
**ENSG00000117009**	KMO	12.65607661	21.01922442	24.42699658	7.535102876	20.80420107	16.69279715	9.192550814	4.983937908	1.875849726	0.632777552	6.898021912	12.68737825
**ENSG00000117010**	ZNF684	111.092228	84.07689769	118.87805	132.2829172	93.61890481	113.814526	141.9738403	119.6145098	119.1164576	132.2505084	150.3768777	148.0194129
**ENSG00000117013**	KCNQ4	7.031153671	0	0	8.372336529	2.311577897	4.173199286	2.04278907	3.322625272	0	4.429442867	0	0.845825217
**ENSG00000117016**	RIMS3	15.46853808	49.04485699	28.49816268	19.25637402	50.85471372	20.48661468	4.08557814	16.61312636	10.78613592	22.77999189	19.31446135	19.45397999
**ENSG00000117020**	AKT3	1510.291808	1520.390567	1280.788854	2365.185069	2454.895726	2294.121462	1982.526792	2013.510915	2399.680762	2070.448151	1992.148728	2728.632149
**ENSG00000117036**	ETV3	1247.326661	1495.868138	1456.66323	1302.735564	1269.056265	1432.5455	1146.004668	1237.677914	1158.337206	1109.891827	1067.813792	1245.054719
**ENSG00000117054**	ACADM	772.0206731	662.1055693	665.2285403	763.5570914	781.313329	716.652132	839.5863077	805.7366285	835.6910528	883.3574631	805.6889593	932.0993889
**ENSG00000117069**	ST6GALNAC	611.7103694	585.0350798	719.782166	710.8113713	570.9597404	741.311946	0	1.661312636	7.503398903	4.429442867	1.379604382	5.074951301
**ENSG00000117091**	CD48	0	0	0	0	0	0	0	9.967875817	0.468962431	2.53111021	0	0
**ENSG00000117114**	ADGRL2	25.31215321	10.50961221	52.11092604	59.44358935	63.56839215	64.11551631	302.3327823	166.1312636	279.9705716	312.5921109	223.4959099	399.2295023
**ENSG00000117115**	PADI2	229.2156097	395.86206	320.8078885	694.9039319	920.0080028	800.4954995	9.192550814	39.87150327	21.10330942	14.5538837	16.55525259	48.21203736
**ENSG00000117118**	SDHB	985.7677446	903.8266502	889.1426756	1064.123973	991.6669176	1101.724612	731.318487	780.816939	762.0639511	785.9097201	728.4311139	925.3327872
**ENSG00000117122**	MFAP2	5.624922937	0	0	4.186168264	0	2.27629052	0	0	0	0	0	0
**ENSG00000117133**	RPF1	891.5502855	595.544692	776.7784914	834.7219519	703.8754695	738.6562737	696.5910728	654.5571786	662.6439156	701.117528	503.5555995	696.9599786
**ENSG00000117139**	KDM5B	3089.488923	3524.223295	2619.388267	4437.33836	4691.347341	3554.427647	5667.718274	7063.901329	5563.770287	5591.85523	7400.197907	6113.624667
**ENSG00000117143**	UAP1	1421.699272	1748.098831	1527.50152	1214.82603	1181.216305	1314.557775	656.7566859	1149.628344	796.767171	688.461977	987.7967377	816.2213342
**ENSG00000117148**	ACTL8	0	0	4.071166097	1.674467306	0	3.793817533	0	0	0	0	0	0
**ENSG00000117151**	CTBS	254.5277629	252.2306931	198.6729055	150.7020575	172.2125533	147.579502	215.5142469	214.3093301	200.7159207	205.6527045	194.5242179	241.0601868
**ENSG00000117152**	RGS4	4041.50713	1734.086015	2699.997356	6640.100101	4269.484375	4786.2802	53.11251581	275.7778976	100.3579603	77.19886139	331.1050518	116.7238799

**ENSG00000117153**	KLHL12	862.01944	865.2914054	883.4430431	800.3953721	851.8164549	813.0150973	1060.207527	1020.045959	1140.516633	1063.699066	1154.728868	1113.95181
**ENSG00000117155**	SSX2IP	1521.541654	1324.211139	1194.480133	1570.650333	1243.628908	1366.153694	1009.1378	887.1409477	974.5039326	1081.416837	852.5955083	1179.926177
**ENSG00000117174**	ZNHIT6	1327.481813	686.6279978	1205.065165	1399.854668	963.9279829	1416.611467	1087.78518	833.9789433	999.3589414	1074.456284	880.1875959	1228.138215
**ENSG00000117215**	PLA2G2D	2.812461468	3.50320407	9.770798633	0	4.623155793	0	2.04278907	3.322625272	0.468962431	0	0	0
**ENSG00000117222**	RBBP5	738.2711354	830.2593647	921.7120044	826.3496154	791.7154296	871.0605056	719.0617526	692.7673693	749.4019655	700.4847505	805.6889593	784.9258012
**ENSG00000117226**	GBP3	846.550902	1107.012486	955.9097996	133.1201508	164.1220307	161.9960087	775.238452	817.365817	754.5605522	182.8727126	172.4505478	215.6854303
**ENSG00000117228**	GBP1	12722.16945	11332.86517	12294.92161	64.46699127	84.37259322	105.4681274	11344.6291	12240.5515	10123.49201	761.2313955	700.8390262	576.0069726
**ENSG00000117242**	PINK1-AS	306.5583	413.3780803	324.8790546	282.9849747	286.6356592	347.1343043	241.0491102	257.5034586	229.322629	200.5904841	165.5525259	254.5933902
**ENSG00000117245**	KIF17	19.68723028	3.50320407	16.28466439	18.41914036	27.73893476	10.62268909	5.106972674	14.95181373	0.468962431	5.694997971	13.79604382	4.229126084
**ENSG00000117262**	GPR89A	220.7782253	210.1922442	220.6572025	188.3775719	167.5893975	233.69916	228.7923758	242.5516449	242.4535771	256.9076863	241.4307669	283.3514476
**ENSG00000117266**	CDK18	97.02992066	192.6762239	118.0638168	66.97869223	135.2273069	77.39387767	0	3.322625272	0.937924863	1.265555105	9.657230676	2.53747565
**ENSG00000117280**	RAB29	260.1526858	304.7787541	203.5583049	237.7743574	256.5851465	225.7321432	25.53486337	19.93575163	9.379248629	11.38999594	19.31446135	10.99572782
**ENSG00000117298**	ECE1	1608.72796	2462.752462	1633.351838	1816.797027	2992.337587	2136.678035	651.6497133	1707.82939	549.6239697	674.5408708	1707.950225	640.2896891
**ENSG00000117305**	HMGCL	381.088529	364.3332233	349.3060511	255.3562641	374.4756192	270.1198084	414.6861812	483.4419771	366.7286214	358.7848722	475.9635119	348.4799893
**ENSG00000117308**	GALE	873.2692859	1019.432384	894.028075	745.1379511	844.8817212	783.4233206	453.4991735	446.8930991	475.9968679	458.7637255	492.5187645	433.062511
**ENSG00000117318**	ID3	1095.453742	781.2145077	1027.562323	849.7921577	476.1850467	732.2067839	3279.697851	2184.626117	4232.854906	3314.488819	2353.605076	3519.478727
**ENSG00000117322**	CR2	0	0	3.256932878	0.837233653	4.623155793	4.55258104	0	1.661312636	0	0	1.379604382	0
**ENSG00000117335**	CD46	3990.882824	4575.184516	4432.685647	4291.659705	4663.608406	4696.366724	5305.123214	4967.324782	5301.620288	5638.047992	4893.456744	5863.260403
**ENSG00000117360**	PRPF3	1577.790884	1401.281628	1650.450736	1432.50678	1329.157291	1402.19496	1255.293883	1505.149248	1193.040426	1282.640099	1267.856427	1279.733553
**ENSG00000117362**	APH1A	1151.702971	1075.48365	1110.614111	1038.16973	939.6564149	1021.29568	1200.138578	1152.950969	1208.047223	1048.512404	1214.051856	1135.097441
**ENSG00000117385**	P3H1	164.5289959	84.07689769	150.6331456	116.3754777	165.2778196	186.6558226	344.2099583	476.7967266	385.4871187	364.4798702	463.5470725	368.7797945
**ENSG00000117394**	SLC2A1	2847.617237	8036.350138	2623.459433	3531.451548	8693.844469	3186.047964	6517.518527	23886.35308	6212.814292	6356.883291	23834.04531	5471.643327
**ENSG00000117395**	EBNA1BP2	5392.894865	3198.425316	4146.075553	5992.918487	4757.227311	4975.591695	2770.021979	2121.496236	3029.028345	2565.280197	2123.211144	3086.416216
**ENSG00000117399**	CDC20	2732.306316	1194.592588	2562.391942	3397.494163	2512.685174	3516.11009	1968.227269	1596.521443	2494.411173	2192.574219	1856.947499	2628.824774
**ENSG00000117400**	MPL	0	0	1.628466439	0	4.623155793	3.035054026	0	0	4.220661883	1.898332657	0	0.845825217
**ENSG00000117407**	ARTN	142.0293041	59.5544692	123.7634494	162.4233287	87.83996007	122.9196881	14.29952349	18.274439	16.88264753	13.2883286	15.17564821	15.2248539
**ENSG00000117408**	IPO13	464.0561423	357.3268152	460.041769	514.8986965	423.0187551	524.6849648	336.038802	350.5369662	377.9837198	309.4282231	326.9662386	401.766978
**ENSG00000117410**	ATP6V0B	1046.235666	987.9035479	1080.487482	1105.148422	1030.963742	1289.897961	420.8145484	403.6989706	565.5686923	546.0870277	343.5214912	578.5444483
**ENSG00000117411**	B4GALT2	625.7726767	367.8364274	536.5796916	642.1582117	552.4671173	602.0788425	254.3272392	222.6158932	252.7707506	297.4054496	295.2353378	340.0217371
**ENSG00000117419**	ERI3	594.8356005	406.3716722	575.6628861	704.1135021	728.1470374	741.6913277	631.2218226	541.5879194	669.2093897	649.8625463	622.2015764	693.5766778
**ENSG00000117425**	PTCH2	4.218692202	0	1.628466439	11.72127114	5.778944741	2.27629052	3.064183605	4.983937908	8.441323766	4.429442867	4.138813147	0.845825217
**ENSG00000117448**	AKR1A1	756.552135	795.227324	721.4106324	504.8518927	595.2313084	561.4849949	656.7566859	518.3295425	708.1332715	544.188695	495.2779732	565.85707
**ENSG00000117450**	PRDX1	8506.289711	7693.036139	7950.987388	8870.490552	8176.05102	8795.207187	7307.056503	7902.86421	8699.722066	7680.653931	7934.104803	9284.623405
**ENSG00000117461**	PIK3R3	174.372611	182.1666117	138.4196473	166.6094969	108.6441611	145.6825933	254.3272392	252.5195207	272.9361351	191.0988208	204.1814486	222.452032
**ENSG00000117472**	TSPAN1	2957.303234	2757.021603	2901.927194	2709.288101	2788.918732	3004.703486	430.0070992	352.1982789	492.410553	431.5542907	357.317535	471.970471
**ENSG00000117475**	BLZF1	1392.168427	1727.079607	1806.783514	1142.823936	1176.593149	1472.380585	829.3723623	1071.54665	904.6285303	876.39691	838.7994644	892.3456037
**ENSG00000117477**	CCDC181	0	0	2.442699658	1.674467306	0	0.379381753	1.021394535	0	3.28273702	3.163887762	0	1.691650434
**ENSG00000117479**	SLC19A2	115.3109202	84.07689769	138.4196473	146.5158893	135.2273069	156.3052824	291.0974424	161.1473257	276.6878346	290.4448965	184.8669872	304.497078
**ENSG00000117480**	FAAH	74.53022891	91.08330583	100.150686	78.69996337	158.3430859	101.2949281	182.8296217	134.5663235	159.4472267	196.7938188	186.2465916	168.3192181
**ENSG00000117481**	NSUN4	617.3352923	434.3973047	610.6749146	564.295482	494.6776699	588.4210994	589.3446466	606.3791122	611.5270106	518.877593	495.2779732	535.4073622
**ENSG00000117500**	TMED5	2165.595331	2087.909626	2156.089565	2154.202189	2039.967494	2396.554536	1975.37703	1907.186906	2203.185503	2084.369258	1765.893609	2566.233708
**ENSG00000117505**	DR1	1888.567876	1450.326485	1610.553308	2030.291608	1873.533885	1994.030495	2807.813576	2568.389335	3074.986663	3000.631153	2465.353031	3478.879117
**ENSG00000117519**	CNN3	3570.419834	4172.316048	3431.178787	3156.370871	3625.709931	2951.590041	2122.457843	5130.13342	2395.929062	1814.80602	4556.833275	2183.074884
**ENSG00000117523**	PRRC2C	10954.53742	10243.3687	10567.93296	10587.65677	9605.761949	9610.119193	10606.16085	9856.56787	10221.03619	10347.17854	10119.39814	10843.47928
**ENSG00000117525**	F3	13533.56459	18199.14515	11637.02117	11990.86038	15322.29409	11109.0565	700.6766509	3036.879499	971.690158	572.6636849	2149.423628	905.8788072
**ENSG00000117528**	ABCD3	1766.225802	1555.422607	1415.137335	2420.44249	2432.935736	2126.814109	1847.702714	1850.702277	1585.561981	2067.917041	2125.970353	2271.040707
**ENSG00000117533**	VAMP4	437.3377583	462.4229373	342.7921854	407.7327889	424.174544	403.6621855	411.6219976	403.6989706	446.4522347	466.3570561	366.9747657	533.7157118
**ENSG00000117543**	DPH5	437.3377583	318.7915704	423.4012741	497.3167898	441.5113782	491.6787523	563.8097833	393.7310948	483.0313044	485.9731602	394.5668533	604.76503
**ENSG00000117569**	PTBP2	414.8380666	367.8364274	435.6147724	535.8295378	453.0692677	476.5034822	840.6077022	622.9922385	701.098835	689.7275321	699.4594218	764.625996
**ENSG00000117586**	TNFSF4	39.37446056	28.02563256	27.68392946	36.00104707	41.60840214	37.93817533	30.64183605	13.29050109	14.53783538	8.858885733	33.11050518	12.68737825
**ENSG00000117592**	PRDX6	4438.064197	3850.021273	3931.932217	5032.611487	4882.052517	5348.90334	3109.124964	3010.298497	3630.707144	2881.668974	2637.803579	3408.675624
**ENSG00000117593**	DARS2	1812.631416	1579.945036	1818.182779	1831.867232	1720.969744	1810.789109	1596.439658	1511.794499	1597.755004	1669.267183	1300.966933	1714.487714
**ENSG00000117594**	HSD11B1	14.06230734	49.04485699	31.75509556	3.348934611	42.76419109	36.04126656	0	3.322625272	4.220661883	0	4.138813147	0.845825217
**ENSG00000117595**	IRF6	8762.223705	14867.59807	11468.4749	7011.831843	8522.787705	8223.478885	7218.195178	5444.121509	7055.539781	6792.867025	5457.714936	6669.331834
**ENSG00000117597**	DIEXF	1290.919814	1135.038119	1401.295371	1186.360086	982.420606	1269.790728	981.560148	885.4796351	932.7662762	961.1891021	768.4396409	1025.140163
**ENSG00000117601**	SERPINC1	0	0	2.442699658	4.186168264	2.311577897	0.379381753	1.021394535	0	1.406887294	3.163887762	2.759208765	2.53747565
**ENSG00000117602**	RCAN3	561.0860629	718.1568344	467.369868	774.4411289	796.3385854	585.0066636	670.0348149	767.5264379	705.3194969	704.2814158	903.6408704	918.5661854
**ENSG00000117614**	SYF2	667.9595987	742.6792629	775.9642581	613.6922676	636.8397105	571.7283022	836.5221241	963.561329	778.0086738	802.3619364	911.9184967	806.9172568
**ENSG00000117616**	RSRP1	742.4898276	1240.134241	815.0474527	594.4358935	943.1237818	689.7160275	964.1964409	1352.308486	991.8555425	951.0646612	1418.233305	1038.673366
**ENSG00000117620**	SLC35A3	1250.139123	1544.912995	982.7794959	1055.751636	1082.974245	1004.223501	1482.04347	1812.492086	1317.31547	1378.189509	1767.273214	1248.43802
**ENSG00000117625**	RCOR3	649.6785992	662.1055693	603.3468156	695.7411655	739.7049269	633.1881463	890.6560344	948.6095152	753.6226273	873.8657998	985.037529	827.217062
**ENSG00000117632**	STMN1	4940.088569	5104.168331	4282.866734	6385.58107	6067.891978	5007.839144	4102.941847	4168.233404	4043.863046	4140.263525	4584.425362	5075.797126
**ENSG00000117640**	MTFR1L	385.3072212	521.9774065	364.7764823	389.3136486	359.4503629	363.0683379	368.7234271	461.8449128	312.7979418	339.1687681	434.5753804	309.5720293
**ENSG00000117643**	MAN1C1	7.031153671	17.51602035	4.885399317	32.65211246	49.69892478	20.10723293	63.32646116	63.12988017	41.7376564	67.7071981	60.70259282	56.67028952
**ENSG00000117650**	NEK2	615.9290616	437.9005088	517.0380943	1026.448458	1021.71743	965.1471804	848.7788585	569.8302342	781.7603732	814.3847099	756.0232015	910.1079333
**ENSG00000117676**	RPS6KA1	698.8966749	875.8010176	713.2683002	667.2752213	688.8502132	721.204713	150.1449966	219.293268	175.8609118	164.5221636	286.9577115	210.610479
**ENSG00000117682**	DHDDS	701.7091363	697.13761	861.4587462	694.0666982	636.8397105	711.3407874	613.8581155	581.4594226	611.5270106	529.6348113	542.1845222	597.9984283

**ENSG00000117691**	NENF	268.5900702	353.8236111	237.7561001	257.8679651	337.4903729	332.3384159	304.3755714	380.4405937	336.7150258	324.6148844	358.6971394	354.4007658
**ENSG00000117697**	NSL1	857.8007478	917.8394665	754.7941944	866.5368307	913.0732691	767.1099052	992.7954879	1093.143715	924.7939148	1027.630745	1136.794011	1049.669094
**ENSG00000117707**	PROX1	36.56199909	94.5865099	87.12295448	5.86063557	33.5178795	17.83094241	6.128367209	18.274439	11.25509835	5.062220419	8.277626294	12.68737825
**ENSG00000117713**	ARID1A	1267.013891	1068.477241	1284.045787	1295.200461	1126.894225	1201.881394	1713.90003	1397.163927	1528.817527	1680.657179	1458.241832	1597.763834
**ENSG00000117724**	CENPF	7184.432821	4151.296823	6846.887142	11092.50867	10403.25632	9423.842752	8686.960519	5708.270218	8540.743802	9598.602692	8142.425064	11155.58878
**ENSG00000117748**	RPA2	722.8025974	753.1888751	748.2803287	740.1145491	729.3028264	730.6892569	576.0665177	603.0564869	674.8369389	613.1614483	467.6858856	705.4182308
**ENSG00000117751**	PPP1R8	672.1782909	739.1760589	791.4346893	831.3730173	810.2080527	853.2295632	755.8319558	817.365817	745.650266	771.9886139	801.5501461	822.9879359
**ENSG00000117758**	STX12	669.3658295	774.2080996	681.5132047	580.2029214	614.8797205	564.5200489	503.5475057	541.5879194	490.0657409	460.0292806	449.7510286	485.5036744
**ENSG00000117791**	2-Mar	9.843615139	14.01281628	19.54159727	15.9074394	19.64841212	20.48661468	90.9041136	166.1312636	75.9719139	105.6738512	133.8216251	92.19494863
**ENSG00000117859**	OSBPL9	2138.876947	2301.605074	2147.133	2427.14036	2533.489375	2470.154596	2252.174949	2061.688981	1990.276559	2183.715333	2041.814486	2024.059744
**ENSG00000117862**	TXNDC12	70.31153671	28.02563256	52.11092604	45.21061725	47.38734688	56.52788124	58.21948849	41.5328159	61.43407852	45.55998377	77.25784541	63.43689126
**ENSG00000117868**	ESYT2	3659.01237	4974.54978	3360.340497	5148.986965	6099.09828	5044.639174	6140.623944	6778.155555	5161.400521	5914.571782	6862.152198	5497.863909
**ENSG00000117877**	CD3EAP	404.9944514	224.2050605	451.0852036	537.5040051	364.0735187	490.9199888	195.0863562	116.2918845	268.2465108	248.0488005	146.2380645	302.8054276
**ENSG00000117899**	MESD	1060.297974	1138.541323	1023.491157	880.7698028	922.3195807	840.3305836	548.4888652	490.0872277	634.9751322	500.5270439	513.2128302	565.85707
**ENSG00000117906**	RCN2	547.0237556	535.9902228	522.7377269	884.1187374	769.7554395	856.643999	468.8200915	315.6494009	472.7141309	497.3631562	430.4365673	614.0691074
**ENSG00000117971**	CHRNB4	0	0	3.256932878	0	0	2.27629052	0	0	1.875849726	0	0	0
**ENSG00000117983**	MUC5B	2.812461468	7.006408141	10.58503185	3.348934611	1.155788948	6.070108053	0	0	0	1.898332657	0	0
**ENSG00000117984**	CTSD	3605.575602	3836.008457	3635.551325	3744.108896	4418.581149	4387.170595	4443.066227	5067.00354	5348.047568	4693.943884	5526.695156	4727.317137
**ENSG00000118004**	COLEC11	7.031153671	14.01281628	18.72736405	10.88403749	3.467366845	4.931962793	10.21394535	14.95181373	4.689624315	6.327775524	9.657230676	4.229126084
**ENSG00000118007**	STAG1	2051.690641	2007.335932	1853.194807	2232.902152	2101.224308	1939.778905	2348.186036	2433.823012	2116.896416	2369.119156	2377.058351	2430.055848
**ENSG00000118046**	STK11	476.7122189	402.8684681	416.0731751	495.6423225	478.4966246	538.7220897	372.8090052	347.214341	429.5695872	438.5148438	423.5385454	464.358044
**ENSG00000118058**	KMT2A	6405.380994	6435.385877	7021.133051	6083.339722	6070.203556	6606.17447	3963.010795	4204.782282	4132.496946	3348.658807	4196.756531	3988.065897
**ENSG00000118096**	IFT46	74.53022891	108.5993262	81.42332194	108.0031412	73.97049269	93.70729307	69.45482837	96.3561329	52.52379232	62.01220013	70.3598235	39.75378519
**ENSG00000118160**	SLC8A2	0	3.50320407	3.256932878	0	1.155788948	3.035054026	0	3.322625272	0	0	0	0
**ENSG00000118162**	KPTN	57.6554601	17.51602035	38.26896131	61.11805666	30.05051265	42.11137462	37.79159779	39.87150327	47.83416801	30.37332251	28.97169203	40.59961041
**ENSG00000118181**	RPS25	5797.889317	5433.469513	5305.543658	5583.511231	5285.42286	5081.439204	3911.941069	4576.916313	4248.799629	4097.867429	4501.649099	4517.552483
**ENSG00000118193**	KIF14	2280.906251	1191.089384	2123.520236	2853.292289	2421.377847	2573.346433	1906.943597	1267.581541	1940.566541	1961.610412	1565.850974	2375.923034
**ENSG00000118194**	TNNT2	11.24984587	0	6.513865756	7.535102876	10.40210053	4.55258104	3.064183605	18.274439	14.06887294	5.694997971	12.41643944	2.53747565
**ENSG00000118197**	DDX59	788.8954419	693.6344059	718.1536996	632.1114079	564.0250068	640.3963996	837.5435186	970.2065795	732.9882804	773.8869466	802.9297505	652.1312421
**ENSG00000118200**	CAMSAP2	5689.60955	4659.261414	5139.440081	7046.158423	5990.454119	6100.458593	6592.080328	6342.891645	6160.759462	6201.852791	6075.7777	6493.400189
**ENSG00000118217**	ATF6	1462.479964	1671.028342	1752.229888	1580.697137	1702.477121	1734.153994	2180.677332	2153.061176	2103.296505	2110.313137	2148.044023	2123.021294
**ENSG00000118242**	MREG	160.3103037	168.1537954	184.8309408	185.8658709	142.1620406	179.0681876	122.5673442	239.2290196	133.1853305	142.3749493	175.2097566	126.0279573
**ENSG00000118246**	FASTKD2	860.6132093	525.4806106	786.54929	723.3698761	627.5933989	660.5036325	760.9389285	601.3951743	668.7404273	718.202522	500.7963908	618.2982335
**ENSG00000118257**	NRP2	1.406230734	7.006408141	0.814233219	0	1.155788948	0	194.0649616	171.1152015	108.7992841	147.4371697	165.5525259	76.97009473
**ENSG00000118260**	CREB1	1096.859973	1317.20473	1195.294366	823.8379144	1013.626908	926.4502416	920.2764759	1096.46634	1003.110641	804.8930466	1001.592782	1067.431424
**ENSG00000118263**	KLF7	659.5222143	1201.598996	577.2913526	620.3901368	1151.165792	572.4870657	147.080813	252.5195207	124.2750443	202.4888168	286.9577115	145.4819373
**ENSG00000118276**	B4GALT6	92.81122845	70.06408141	69.20982365	129.7712162	135.2273069	112.296999	402.4294467	398.7150327	408.9352402	488.5042704	452.5102374	534.561537
**ENSG00000118292**	C1orf54	16.87476881	24.52242849	15.47043117	16.74467306	2.311577897	7.967016819	8.171156279	11.62918845	5.158586746	11.38999594	4.138813147	6.766601734
**ENSG00000118307**	CASC1	5.624922937	0	0.814233219	0	1.155788948	1.13814526	16.34231256	14.95181373	2.813774589	4.429442867	2.759208765	3.383300867
**ENSG00000118308**	LRMP	7.031153671	0	0	0	0	2.27629052	60.26227756	14.95181373	54.39964205	40.49776335	38.6289227	39.75378519
**ENSG00000118322**	ATP10B	0	14.01281628	0	0.837233653	3.467366845	1.896908767	0	0	0	0	0	0
**ENSG00000118363**	SPCS2	933.7372075	896.820242	942.8820681	763.5570914	677.2923237	879.4069042	842.6504913	770.8490632	802.3947202	767.559171	928.4737493	748.5553168
**ENSG00000118369**	USP35	78.74892111	73.56728548	59.43902502	70.32762684	107.4883722	88.77533027	59.24088302	116.2918845	71.28228958	79.7299716	106.2295374	69.35766778
**ENSG00000118402**	ELOVL4	44.99938349	35.0320407	35.01202844	57.76912205	70.50312584	66.01242507	83.75435186	86.38825708	80.66153821	91.7527451	153.1360864	120.953006
**ENSG00000118407**	FILIP1	2.812461468	0	4.885399317	0	5.778944741	0.758763507	0	6.645250545	2.344812157	3.163887762	5.518417529	2.53747565
**ENSG00000118412**	CASP8AP2	1923.723644	2595.874216	1836.09591	1895.49699	2061.927484	1743.638538	2180.677332	2503.598143	2020.759117	2305.841401	2552.268107	2226.211971
**ENSG00000118418**	HMGN3	435.9315276	388.8556518	320.8078885	316.4743208	302.8167044	191.2084037	649.6069242	754.2359368	538.3688713	604.9353401	655.3120816	603.0733796
**ENSG00000118420**	UBE3D	151.8729193	77.07048955	135.1627144	184.1914036	157.187297	157.0640459	161.3803365	186.0670152	153.8196775	163.8893861	190.3854048	172.5483442
**ENSG00000118432**	CNR1	174.372611	133.1217547	165.2893435	235.2626565	251.9619907	236.7342141	177.7226491	164.469951	126.6198565	149.9682799	164.1729215	140.406986
**ENSG00000118454**	ANKRD13C	1528.572808	1142.044527	1378.496841	1454.274855	1226.292074	1324.042319	755.8319558	867.2051961	860.0770993	682.766979	586.3318625	866.125022
**ENSG00000118473**	SGIP1	3597.138218	2511.797318	2988.235915	2846.59442	2447.960992	2570.69076	1862.002237	2892.345299	1655.906345	1494.620579	2440.520152	1431.136267
**ENSG00000118482**	PHF3	4156.81805	5104.168331	3889.592089	4086.53746	4958.334588	3877.660901	5530.851406	6185.066944	4723.38961	5093.859297	5855.040999	4924.394412
**ENSG00000118491**	ZC2HC1B	8.437384405	0	8.956565414	7.535102876	0	3.035054026	4.08557814	1.661312636	5.158586746	6.960553076	6.898021912	3.383300867
**ENSG00000118495**	PLAGL1	5.624922937	3.50320407	0.814233219	0	0	1.896908767	0	4.983937908	0.468962431	0	0	0.845825217
**ENSG00000118496**	FBXO30	698.8966749	550.0030391	673.3708725	603.6454637	582.5176299	653.2953792	1097.999125	845.6081318	898.5320187	900.442457	682.9041692	934.6368645
**ENSG00000118503**	TNFAIP3	8940.815008	12611.53465	12840.45787	6159.527984	6393.824462	8372.575914	789.5379755	1554.988627	1027.496687	399.9154131	980.8987158	622.5273595
**ENSG00000118507**	AKAP7	32.34330689	80.57369362	68.39559043	9.209570182	18.49262317	13.65774312	5.106972674	3.322625272	9.848211061	4.429442867	2.759208765	16.07067912
**ENSG00000118508**	RAB32	71.71776744	154.1409791	107.478785	74.51379511	129.4483622	96.74234709	287.0118643	305.681525	297.791144	266.3993496	226.2551187	250.3642642
**ENSG00000118513**	MYB	9.843615139	28.02563256	16.28466439	10.88403749	25.42735686	21.24537819	154.2305748	215.9706427	105.0475846	131.6177309	280.0596896	81.19922081
**ENSG00000118514**	ALDH8A1	9.843615139	7.006408141	0	0.837233653	0	1.517527013	0	1.661312636	2.813774589	6.327775524	2.759208765	0
**ENSG00000118515**	SGK1	2113.564793	1579.945036	2110.492505	1694.560913	1604.23506	1673.452914	691.4841001	722.6709967	1060.79302	628.9808871	540.8049179	921.1036611
**ENSG00000118518**	RNF146	390.9321441	507.9645902	493.425331	365.8711063	359.4503629	394.5570234	542.360498	515.0069172	574.0100161	487.2387153	535.2865003	521.0283335
**ENSG00000118523**	CTGF	195.466072	122.6121425	197.8586723	189.2148055	125.9809954	305.4023114	86.81853546	73.09775599	113.9578708	80.36274915	64.84140597	112.4947538
**ENSG00000118557**	PMFBP1	11.24984587	0	3.256932878	1.674467306	3.467366845	1.13814526	4.08557814	3.322625272	3.751699452	1.265555105	4.138813147	4.229126084
**ENSG00000118564**	FBXL5	1089.828819	1040.451609	1137.483808	798.7209048	664.5786453	796.7016819	755.8319558	880.4956971	772.3811246	565.0703543	647.0344553	652.9770674
**ENSG00000118579**	MED28	959.0493607	984.4003438	902.1704071	874.9091672	822.9217312	910.8955897	1223.630653	1285.85598	1256.819316	1348.448964	1506.527985	1245.900544
**ENSG00000118596**	SLC16A7	1123.578357	725.1632426	764.5649931	1765.725774	1594.988749	1358.94544	32.68462512	31.56494009	43.14454369	43.66165111	5.518417529	40.59961041

**ENSG00000118600**	RXYLT1	191.2473798	283.7595297	241.013033	271.2637035	236.9367344	278.4662069	331.9532238	164.469951	314.2048291	291.0776741	222.1163056	298.5763015
**ENSG00000118620**	ZNF430	210.9346101	196.1794279	233.684934	305.5902833	176.8357091	253.0476295	313.5681222	393.7310948	306.2324677	298.0382272	365.5951613	341.7133876
**ENSG00000118640**	VAMP8	1099.672434	1033.445201	1120.38491	739.2773155	762.8207059	816.4295331	1085.742391	988.4810185	1063.137832	886.5213509	876.0487828	910.9537585
**ENSG00000118655**	DCLRE1B	485.1496033	441.4037129	463.2987019	475.5487148	535.130283	471.5715194	356.4666927	433.602598	388.3008932	370.1748681	324.2070298	460.1289179
**ENSG00000118680**	MYL12B	4764.309727	4305.437803	4399.302085	4156.865086	3906.566645	4010.444514	3430.864243	3749.58262	3648.996679	3167.05165	3535.926032	4033.740459
**ENSG00000118689**	FOXO3	3570.419834	4231.870517	4438.385279	2922.782682	3148.369095	3705.042203	1561.712244	2252.739935	1571.96207	1316.177309	1971.454662	1393.074132
**ENSG00000118690**	ARMC2	52.03053716	35.0320407	41.52589419	71.16486049	65.87997005	46.66395566	77.62598465	56.48462963	60.02719123	68.33997566	103.4703287	100.6532008
**ENSG00000118705**	RPN2	3851.665981	4326.457027	4429.428714	4009.511964	4375.816958	4680.812072	5067.138288	5809.610289	6001.781198	5662.093539	6514.491893	6421.505046
**ENSG00000118707**	TGIF2	125.1545353	126.1153465	119.6922833	79.53719702	130.6041512	108.5031814	31.66323058	24.91968954	22.04123428	32.27165517	15.17564821	18.60815477
**ENSG00000118762**	PKD2	652.4910606	539.4934268	511.3384618	541.6901734	570.9597404	611.1840046	923.3406595	706.0578704	982.0073315	739.7169587	633.2384115	876.2749246
**ENSG00000118777**	ABCG2	3261.049073	1954.787871	2672.313426	2763.708288	2489.569395	3158.353096	4484.943403	2516.888644	5409.481647	3847.920296	1938.344157	5200.133433
**ENSG00000118785**	SPP1	0	0	0.814233219	0	0	1.896908767	85.79714093	64.79119281	149.1300532	163.8893861	106.2295374	348.4799893
**ENSG00000118804**	STBD1	0	0	2.442699658	0	0	0.758763507	7.149761744	3.322625272	4.220661883	1.265555105	4.138813147	0
**ENSG00000118816**	CCNI	2683.088241	2122.941667	2443.513892	2764.545522	3147.213306	2862.81471	2409.469708	2689.665158	2604.148382	2394.430258	2986.843488	2723.557198
**ENSG00000118849**	RARRES1	40.78069129	147.134571	109.9214846	11.72127114	54.32208057	48.94024618	49.02693767	63.12988017	41.7376564	61.37942258	35.86971394	28.75805737
**ENSG00000118855**	MFSD1	458.4312193	578.0286716	492.6110978	576.8539868	545.5323836	550.8623058	703.7408345	578.1367974	626.5338084	673.2753157	551.8417529	703.7265804
**ENSG00000118873**	RAB3GAP2	1701.539188	1534.403383	1578.798213	1596.604576	1597.300327	1496.281635	1768.03394	1564.956503	1551.327723	1672.431071	1550.675326	1644.284221
**ENSG00000118894**	EEF2KMT	144.8417656	73.56728548	126.206149	135.6318518	144.4736185	135.8186677	84.77574639	58.14594226	143.502504	116.4310696	64.84140597	120.1071808
**ENSG00000118898**	PPL	2497.465784	2599.37742	2386.517566	2865.01356	2696.455616	2697.024884	615.9009045	593.0886111	742.8364914	601.7714523	613.9239501	829.7545377
**ENSG00000118900**	UBN1	772.0206731	1156.057343	942.0678349	1033.983561	1003.224807	999.2915382	912.1053196	830.6563181	832.8772783	825.7747059	888.4652222	939.7118158
**ENSG00000118922**	KLF12	8.437384405	0	5.699632536	0	6.93473369	5.6907263	3.064183605	1.661312636	10.31717349	1.265555105	0	2.53747565
**ENSG00000118939**	UCHL3	92.81122845	126.1153465	96.07951989	103.816973	100.5536385	119.1258705	120.5245551	167.7925762	147.2542035	166.4204963	140.719647	145.4819373
**ENSG00000118946**	PCDH17	4.218692202	3.50320407	0.814233219	6.697869223	13.86946738	6.828871559	0	0	2.344812157	1.265555105	0	0
**ENSG00000118960**	HS1BP3	361.4012987	427.3908966	438.8717053	282.147741	351.3598403	352.4456488	220.6212195	267.4713344	197.9021461	196.1610412	278.6800852	206.3813529
**ENSG00000118961**	LDAH	534.367679	542.9966309	533.3227587	516.5731638	567.4923736	591.0767716	548.4888652	503.3777287	549.6239697	516.3464827	511.8332258	659.7436691
**ENSG00000118965**	WDR35	402.18199	252.2306931	274.396595	461.3157427	394.1240314	368.0003007	604.6655646	302.3588998	485.845079	551.7820257	466.3062812	534.561537
**ENSG00000118971**	CCND2	1718.413957	616.5639164	687.2128372	1023.099524	377.9429861	549.3447788	28.59904698	36.54887799	317.9565285	29.10776741	11.03683506	208.9188285
**ENSG00000118976**	OTUD4P1	1.406230734	0	4.885399317	0.837233653	0	1.13814526	2.04278907	0	0.468962431	0	0	1.691650434
**ENSG00000118985**	ELL2	1382.324812	2953.201031	1560.885082	1574.836501	2115.093775	1588.850783	222.6640086	631.2988017	286.5360456	309.4282231	709.1166525	360.3215423
**ENSG00000118997**	DNAH7	4.218692202	0	2.442699658	9.209570182	18.49262317	15.17527013	28.59904698	16.61312636	27.19982102	36.06832049	38.6289227	25.3747565
**ENSG00000119004**	CYP20A1	264.371378	245.2242849	235.3134004	291.3573112	226.5346339	268.2228996	511.718662	510.0229793	524.2999984	506.8548195	517.3516434	575.1611474
**ENSG00000119013**	NDUFB3	437.3377583	437.9005088	445.385571	564.295482	496.9892478	537.9633262	278.840708	318.9720261	331.0874766	364.4798702	395.9464577	422.9126084
**ENSG00000119041**	GTF3C3	835.3010561	865.2914054	777.5927246	803.7443068	711.9659921	779.629503	964.1964409	824.0110675	910.7250419	887.1541284	951.9270238	902.4955063
**ENSG00000119042**	SATB2	2.812461468	21.01922442	16.28466439	14.2329721	13.86946738	20.48661468	59.24088302	84.72694444	82.53738794	77.83163894	103.4703287	76.97009473
**ENSG00000119048**	UBE2B	783.2705189	1057.967629	733.6241307	642.1582117	635.6839215	724.2397671	872.2709328	822.3497549	921.0422154	846.6563651	725.6719051	1069.123074
**ENSG00000119121**	TRPM6	43.59315276	17.51602035	45.59706029	19.25637402	27.73893476	19.34846942	1346.197997	1147.967032	1585.093018	1347.183409	1211.292648	1463.277625
**ENSG00000119125**	GDA	2691.525625	1968.800688	2719.538953	2266.391498	1835.39285	2132.504835	2042.78907	1596.521443	2057.338187	1839.484345	1596.20227	1999.530812
**ENSG00000119138**	KLF9	511.8679872	518.4742024	446.1998043	740.9517828	806.7406859	594.4912074	1027.522902	941.9642647	895.7182441	1056.105735	1073.332209	984.5405523
**ENSG00000119139**	TJP2	494.9932184	641.0863449	555.3070557	449.5944716	424.174544	455.258104	421.8359429	312.3267756	355.0045606	347.3948763	364.2155569	346.7883389
**ENSG00000119185**	ITGB1BP1	655.3035221	767.2016914	664.4143071	748.4868857	797.4943743	804.6686988	505.5902948	591.4272985	487.7209287	511.9170399	564.2581924	506.6493048
**ENSG00000119203**	CPSF3	1680.445727	1499.371342	1447.706664	1447.576986	1328.001502	1368.809366	1426.888165	1335.695359	1410.170031	1239.611225	1347.873482	1527.560341
**ENSG00000119227**	PIGZ	5.624922937	0	1.628466439	0	6.93473369	3.41443578	2.04278907	4.983937908	4.220661883	1.898332657	0	3.383300867
**ENSG00000119231**	SENP5	1577.790884	1208.605404	1778.285351	1777.447045	1548.757191	1932.570651	1511.663912	1596.521443	1505.369405	1427.546158	1396.159635	1634.980144
**ENSG00000119242**	CCDC92	236.2467633	273.2499175	213.3291035	186.7031046	246.183046	196.89913	386.0871342	373.7953431	310.4531296	367.6437579	445.6122155	298.5763015
**ENSG00000119280**	C1orf198	355.7763757	409.8748762	358.2626166	378.4296111	352.5156292	424.9075637	538.2749199	438.5865359	584.796152	552.4148032	506.3148083	625.9106604
**ENSG00000119283**	TRIM67	0	7.006408141	0	0	1.155788948	0.379381753	9.192550814	1.661312636	5.158586746	8.858885733	11.03683506	10.99572782
**ENSG00000119285**	HEATR1	3051.520693	1898.736606	2857.144367	3281.118686	2452.584148	3252.439771	3252.120199	2221.174995	3412.170651	3179.074423	2451.556987	3580.378143
**ENSG00000119314**	PTBP3	5353.520405	4872.956862	4615.888121	6633.402232	5699.195304	5990.058503	7774.855199	7580.569559	7483.702481	7959.076054	7905.133111	8354.215666
**ENSG00000119318**	RAD23B	11684.37117	9938.589948	10148.60285	12135.7018	10194.05852	10763.8191	9598.044444	8467.710506	9604.350596	9005.690125	7877.541023	10099.15309
**ENSG00000119321**	FKBP15	1650.914882	2122.941667	1899.606101	1249.989844	1507.148789	1349.840278	1466.722552	1847.379651	1416.266543	1316.177309	1568.610183	1316.104037
**ENSG00000119326**	CTNNAL1	4637.748961	3373.58552	3363.59743	5488.903828	4943.309332	4556.374857	3803.673248	4271.234787	4000.24954	3712.5059	3941.52972	4822.049561
**ENSG00000119328**	FAM206A	389.5259134	434.3973047	333.83562	413.5934245	405.6819208	314.8868552	542.360498	518.3295425	459.5831828	551.7820257	420.7793366	525.2574596
**ENSG00000119333**	WDR34	914.0499772	760.1952833	732.8098975	1158.731376	984.7321839	1102.483375	873.2923273	760.8811873	1050.006884	883.9902407	816.7257943	1080.118802
**ENSG00000119335**	SET	20016.28827	16352.9566	18146.00153	20229.23952	17987.5434	17652.63298	14630.45532	12976.513	15509.05657	13753.4201	11974.96604	16302.43523
**ENSG00000119383**	PTPA	1004.048744	924.8458746	1047.918153	1404.040836	1312.976245	1448.100152	949.8969174	853.914695	1133.951159	1046.614072	1084.369044	1059.818997
**ENSG00000119392**	GLE1	1819.66257	2010.839136	1840.167076	1738.934297	1844.639161	1804.339619	1862.002237	1995.236476	1823.325933	1800.252137	1971.454662	2024.059744
**ENSG00000119396**	RAB14	2718.244009	2785.047236	3066.402304	3120.369824	3200.379598	3314.658379	3167.344453	2985.378807	3189.882459	2989.241157	2999.259927	3315.63485
**ENSG00000119397**	CNTRL	1123.578357	711.1504263	1059.317419	1240.780274	1156.944737	1026.986406	1323.727317	1049.949586	1283.550175	1460.450591	1186.459769	1409.144811
**ENSG00000119401**	TRIM32	700.3029056	753.1888751	624.5168793	606.1571647	768.5996506	673.4026121	618.9650881	589.7659858	635.4440946	583.4209033	612.5443457	643.67299
**ENSG00000119402**	FBXW2	2112.158563	1678.03475	2159.346498	2527.608398	2223.737936	2420.076204	2490.159876	2275.998311	2530.990243	2512.126883	2401.89123	2698.182442
**ENSG00000119403**	PHF19	811.3951336	760.1952833	817.4901523	1045.704832	1150.010004	1124.487517	996.881066	888.8022603	1155.523431	1204.80846	1085.748649	1210.375885
**ENSG00000119408**	NEK6	139.2168427	192.6762239	133.534248	173.3073661	316.6861718	158.9609546	232.877954	380.4405937	156.6334521	253.7437985	456.6490505	179.314946
**ENSG00000119411**	BSPRY	375.463606	535.9902228	444.5713378	402.709387	492.366092	508.7509312	153.2091802	196.0348911	165.5437383	147.4371697	184.8669872	131.1029086
**ENSG00000119414**	PPP6C	1514.510501	1457.332893	1599.968276	1554.742893	1530.264568	1652.586917	1359.476126	1355.631111	1365.149638	1337.058968	1252.680779	1336.403843
**ENSG00000119421**	NDUFA8	592.0231391	620.0671205	660.343141	648.856081	702.7196805	696.9242808	466.7773024	543.249232	504.6035762	515.7137052	515.972039	581.9277491
**ENSG00000119431**	HDHD3	210.9346101	129.6185506	225.5426018	170.7956652	204.5746438	167.686735	156.2733638	196.0348911	167.419588	208.8165923	209.6998661	158.1693155
**ENSG00000119446**	RBM18	786.0829804	753.1888751	715.7109999	684.0198944	701.5638916	782.6645571	536.2321308	613.0243627	660.7680659	659.9869871	641.5160378	751.9386177

ENSG00000119471	HSDL2	874.6755166	714.6536304	698.6121023	1168.778179	1094.532134	1111.588537	1028.544297	973.5292048	1210.860998	1217.464011	1325.799811	1462.4318
ENSG00000119487	MAPKAP1	1579.197114	1481.855322	1577.169746	1897.171457	1894.338086	1919.29229	1355.390548	1387.196051	1439.245702	1618.012201	1648.627237	1722.100141
ENSG00000119508	NR4A3	9.843615139	21.01922442	14.65619795	34.32657977	43.91998003	23.5216687	39.83438686	23.25837691	73.62710174	65.1760879	44.14734023	76.97009473
ENSG00000119509	INVS	542.8050634	497.454978	641.6157769	498.9912571	481.9639914	473.0890464	667.9920258	697.7513072	600.7408747	709.9764138	706.3574437	631.8314369
ENSG00000119514	GALNT12	50.62430643	52.54806106	60.25325824	12.55850479	56.63365846	15.55465189	15.32091802	11.62918845	10.31717349	12.0227735	0	1.691650434
ENSG00000119522	DENND1A	400.7757592	409.8748762	448.6425039	395.1742842	419.5513882	456.0168675	718.040358	744.268061	566.5066172	623.2858891	561.4989836	555.7071674
ENSG00000119523	ALG2	705.9278285	627.0735286	783.2923571	639.6465108	588.2965747	702.9943889	499.4619276	528.2974183	501.7898017	524.5725909	569.7766099	548.0947405
ENSG00000119535	CSF3R	0	0	0	0	0	0	127.6743169	9.967875817	28.60670832	114.532737	12.41643944	26.22058172
ENSG00000119537	KDSR	2266.843943	2343.643523	2190.28736	1435.018481	1588.054015	1487.555855	1507.578333	1574.924379	1680.292392	1344.652299	1295.448515	1481.88578
ENSG00000119541	VPS4B	2415.904401	2504.79091	2386.517566	2148.341553	2192.531635	2082.047062	1825.232034	1847.379651	1837.863769	1654.080522	1816.938972	1901.415087
ENSG00000119547	ONECUT2	157.4978422	80.57369362	100.9649192	248.6583949	201.107277	236.7342141	98.05387535	71.43644335	106.9234344	131.6177309	88.29468047	166.6275677
ENSG00000119559	C19orf25	198.2785335	248.727489	222.2856689	210.1456469	246.183046	228.7671972	174.6584655	207.6640795	255.1155627	206.9182596	198.6630311	200.4605764
ENSG00000119574	ZBTB45	42.18692202	56.05126513	42.34012741	63.62975762	41.60840214	47.04333741	43.919965	48.17806645	62.84096581	44.92720622	44.14734023	63.43689126
ENSG00000119596	YLPM1	1445.605195	1341.727159	1623.58104	1839.402335	1695.542387	1832.034487	2263.410289	2249.417309	2143.627274	2240.032535	2368.780724	2315.023618
ENSG00000119599	DCAF4	108.2797665	112.1025303	120.5065165	133.9573845	112.111528	113.814526	189.9793835	132.9050109	193.6814842	242.9865801	189.0058004	223.2978572
ENSG00000119616	FCF1	621.5539845	465.9261414	615.5603139	740.1145491	751.2628164	731.4480204	987.6885152	1073.207963	1032.655274	1021.935747	956.0658369	1146.093169
ENSG00000119630	PGF	5.624922937	35.0320407	5.699632536	10.04680383	48.54313583	5.6907263	0	69.77513072	0.937924863	0.632777552	46.906549	5.920776517
ENSG00000119632	IFI27L2	30.93707615	52.54806106	55.36785892	10.88403749	55.47786952	37.17941182	11.23533988	21.59706427	11.25509835	6.327775524	5.518417529	10.1499026
ENSG00000119636	BBOF1	11.24984587	63.05767327	23.61276336	16.74467306	18.49262317	22.38352344	104.1822426	157.8247004	92.385599	96.18218796	110.3683506	80.35339559
ENSG00000119638	NEK9	939.3621304	980.8971397	911.1269726	762.7198578	985.8879729	823.6377864	1466.722552	1608.150632	1323.411982	1357.30785	1564.47137	1152.013945
ENSG00000119640	ACYP1	258.7464551	168.1537954	204.3725381	266.2403016	266.987247	243.1837039	271.6909463	260.8260839	225.5709295	298.6710047	217.9774924	256.2850407
ENSG00000119650	IFT43	156.0916115	150.637775	118.0638168	103.816973	97.08627165	121.7815428	256.3700283	176.0991394	242.9225395	279.6876782	197.2834267	253.747565
ENSG00000119655	NPC2	480.9309111	535.9902228	493.425331	362.5221717	447.290323	422.6312732	597.5158029	754.2359368	799.5809456	584.6864584	670.4877298	800.9964803
ENSG00000119661	DNAL1	137.8106119	168.1537954	93.63682024	219.3552171	248.4946239	166.9279715	264.5411845	345.5530283	255.1155627	299.9365598	314.5497992	298.5763015
ENSG00000119669	IRF2BPL	352.9639143	332.8043867	267.068496	421.1285274	391.8124535	357.7569934	823.2439951	712.7031209	1004.517528	787.1752752	704.9778394	838.2127898
ENSG00000119673	ACOT2	23.90592248	10.50961221	40.71166097	28.4659442	30.05051265	33.76497604	57.19809395	66.45250545	50.6479426	48.09109398	80.01705417	61.74524082
ENSG00000119681	LTBP2	433.1190661	322.2947745	539.0223913	504.8518927	644.9302331	747.0026723	408.557814	210.9867048	515.8586746	335.3721028	368.3543701	455.0539666
ENSG00000119682	AREL1	708.74029	644.589549	946.139001	750.161353	710.8102032	732.2067839	836.5221241	727.6549346	803.8016075	890.3180162	771.1988497	819.6046351
ENSG00000119684	MLH3	638.4287533	707.6472222	625.3311125	530.8061359	606.7891978	562.6231402	756.8533503	1068.224025	723.6090317	797.9324936	1045.740122	791.6924029
ENSG00000119685	TTLL5	862.01944	900.3234461	787.3635232	1010.541019	985.8879729	874.0955596	1313.513372	1456.971182	1225.398833	1478.168362	1787.967279	1388.845006
ENSG00000119686	FLVCR2	29.53084542	63.05767327	42.34012741	84.56059894	108.6441611	58.80417176	281.9048916	483.4419771	186.6470477	199.324929	533.906896	202.998052
ENSG00000119688	ABCD4	122.3420739	133.1217547	163.6608771	100.4680383	121.3578396	106.6062727	164.4445201	257.5034586	208.688282	242.3538026	286.9577115	172.5483442
ENSG00000119689	DLST	1023.735974	994.909956	1016.163058	1064.123973	953.5258823	1129.41948	1184.81766	1109.756841	1208.985148	1190.254576	1178.182142	1294.112582
ENSG00000119698	PPP4R4	2.812461468	17.51602035	9.770798633	0	6.93473369	1.896908767	6.128367209	9.967875817	8.441323766	2.53111021	12.41643944	9.304077385
ENSG00000119699	TGFB3	1.406230734	3.50320407	0	0.837233653	5.778944741	4.931962793	1.021394535	3.322625272	5.627549177	10.12444084	1.379604382	4.229126084
ENSG00000119703	ZC2HC1C	19.68723028	38.53524477	43.15436063	36.00104707	42.76419109	35.28250306	65.36925023	64.79119281	51.58586746	64.54331034	62.0821972	75.27844429
ENSG00000119705	SLIRP	420.4629895	346.817203	400.602744	526.6199677	417.2398103	443.1178879	443.2852281	435.2639107	407.0593905	464.4587234	355.9379306	555.7071674
ENSG00000119707	RBM25	2552.308782	2049.374381	2475.268987	2289.834041	2026.098026	2249.733797	2991.664593	2741.16585	2570.383087	2697.530706	2477.769471	2688.878364
ENSG00000119711	ALDH6A1	120.9358431	94.5865099	97.70798633	107.1659076	149.0967743	90.67223904	307.439755	340.5690904	254.1776378	294.2415619	357.317535	268.9724189
ENSG00000119715	ESRRB	0	3.50320407	2.442699658	2.511700959	0	0.379381753	6.128367209	3.322625272	0.468962431	0	0	2.53747565
ENSG00000119718	EIF2B2	257.3402244	269.7467134	296.3808919	277.9615728	247.3388349	301.6084939	365.6592435	378.779281	381.7354192	415.7348519	376.6319964	427.1417345
ENSG00000119720	NRDE2	277.0274546	238.2178768	266.2542628	311.4509189	223.067267	250.0125754	370.7662162	269.1326471	407.5283529	365.7454253	344.9010956	397.5378519
ENSG00000119723	COQ6	153.27915	227.7082646	194.6017394	150.7020575	151.4083522	175.6537518	267.6053681	225.9385185	263.5568865	262.6026842	282.8188984	278.2764963
ENSG00000119725	ZNF410	67.49907524	105.0961221	58.6247918	64.46699127	42.76419109	60.32169878	144.0166294	184.4057026	141.1576919	165.1549412	169.691339	171.702519
ENSG00000119729	RHOQ	497.8056799	542.9966309	651.3865756	606.1571647	619.5028763	671.8850851	364.637849	370.4727179	374.7009827	380.299309	384.9096227	383.1588232
ENSG00000119737	GPR75	71.71776744	31.52883663	98.52221955	82.04889798	67.035759	94.08667482	60.26227756	59.8072549	48.77209287	43.02887356	86.91507609	54.13281387
ENSG00000119760	SUPT7L	788.8954419	693.6344059	856.5733469	786.1624	805.5848969	786.4583746	994.838277	983.4970806	815.5256683	851.085808	946.4086063	866.9708472
ENSG00000119771	KLHL29	978.736591	655.0991612	783.2923571	901.7006441	625.281821	806.9449893	1166.432559	689.444744	1095.965202	1066.862953	742.2271577	1075.043851
ENSG00000119772	DNMT3A	232.0280711	290.7659378	298.8235915	226.0530863	396.4356093	303.1260209	123.5887387	134.5663235	132.2474057	149.3355024	153.1360864	175.0858199
ENSG00000119777	TMEM214	1423.105503	1331.217547	1353.255611	1201.430292	1122.271069	1369.568129	984.6243316	855.5760076	1065.013682	892.2163489	964.3434632	1031.906764
ENSG00000119778	ATAD2B	513.274218	465.9261414	512.9669283	636.2975762	742.0165048	714.7552232	1065.3145	1001.77152	923.3870275	966.8841	1124.377572	1091.960355
ENSG00000119782	FKBP1B	1.406230734	7.006408141	7.328098975	4.186168264	6.93473369	4.931962793	9.192550814	23.25837691	1.875849726	9.491663286	1.379604382	5.074951301
ENSG00000119787	ATL2	1254.357815	900.3234461	1100.029079	1310.270667	997.4458624	1328.974282	869.2067492	624.6535512	810.836044	902.3407897	698.0798174	998.919581
ENSG00000119801	YPEL5	1369.668735	1860.201361	1730.245591	1236.594105	1675.893975	1670.797242	1310.449188	1653.006073	1362.804826	1145.960147	1357.530712	1234.058991
ENSG00000119812	FAM98A	866.2381322	683.1247937	911.9412058	944.3995604	846.0375101	993.9801937	718.040358	566.5076089	664.9887278	706.812526	678.7653561	735.0221134
ENSG00000119820	YIPF4	942.1745919	959.8779153	839.4744492	827.186849	889.9574902	938.5904577	1320.663134	1571.601754	1245.564218	1375.658399	1524.462842	1284.808504
ENSG00000119844	AFTPH	867.644363	973.8907316	983.5937291	866.5368307	1032.119531	890.0295933	958.0680737	913.7219499	902.7526805	811.2208222	950.5474194	973.5448245
ENSG00000119862	LGALSL	292.4959927	266.2435094	241.8272662	399.3604524	457.6924235	426.4250907	106.2250316	136.2276362	110.6751338	134.7816187	150.3768777	165.7817425
ENSG00000119866	BCL11A	1.406230734	0	0.814233219	5.023401917	10.40210053	5.6907263	80.69016826	58.14594226	113.9578708	77.83163894	104.8499331	113.340579
ENSG00000119878	CRIPT	582.1795239	571.0222635	613.1176142	589.4124916	702.7196805	713.9964597	470.8628806	435.2639107	515.8586746	537.228142	480.102325	637.7522134
ENSG00000119888	EPCAM	549.8362171	1040.451609	871.2295448	957.7952989	1415.841462	1306.97014	241.0491102	229.2611438	256.9914124	309.4282231	357.317535	330.7176598
ENSG00000119899	SLC17A5	1011.079898	1022.935589	1089.444048	891.6538403	963.9279829	1115.002973	755.8319558	641.2666775	862.4219114	789.7063854	676.0061473	965.0865723
ENSG00000119900	OGFRL1	1545.447577	1720.073199	1186.337801	1884.612953	1391.569894	1396.883616	4694.329282	2940.523366	4259.585765	4895.167145	3022.713202	5124.854988
ENSG00000119906	SLF2	1705.757881	2056.380789	1893.092235	1568.975865	1872.378096	1579.366239	2008.061656	2216.191057	1751.105719	1701.538838	2010.083585	1735.633345
ENSG00000119912	IDE	1122.172126	1029.941997	1266.132656	1131.939899	1163.879471	1188.223651	1205.245551	1192.822473	1236.653932	1226.955674	1249.92157	1346.553745
ENSG00000119915	ELOVL3	5.624922937	3.50320407	5.699632536	7.535102876	6.93473369	6.449489806	1.021394535	13.29050109	0	3.796665314	8.277626294	2.53747565
ENSG00000119917	IFIT3	7424.898276	9283.490787	7783.255345	442.0593687	726.9912485	558.8293226	2403.341341	2942.184679	2234.137023	224.0032535	306.2721729	170.8566938

**ENSG00000119922**	IFIT2	2273.875097	2893.646562	2239.141353	1068.310141	1225.136285	881.3038129	1493.27881	2319.19244	1427.052679	577.0931278	1092.646671	806.9172568
**ENSG00000119927**	GPAM	461.2436808	273.2499175	479.5833663	522.4337994	502.7681925	520.8911473	547.4674707	534.9426688	589.0168139	635.3086626	575.2950274	728.2555116
**ENSG00000119929**	CUTC	343.1202991	262.7403053	283.3531604	408.5700226	330.5556392	311.0930377	194.0649616	207.6640795	184.3022356	206.2854821	289.7169203	166.6275677
**ENSG00000119938**	PPP1R3C	19.68723028	91.08330583	38.26896131	30.1404115	405.6819208	74.35882365	1.021394535	43.19412854	3.751699452	0	23.4532745	0.845825217
**ENSG00000119943**	PYROXD2	23.90592248	24.52242849	24.42699658	20.09360767	46.23155793	18.96908767	10.21394535	49.83937908	12.19302322	37.33387559	20.69406573	10.99572782
**ENSG00000119946**	CNNM1	0	0	0.814233219	0	0	1.517527013	2.04278907	1.661312636	4.220661883	0	5.518417529	8.458252168
**ENSG00000119950**	MXI1	317.8081459	620.0671205	254.0407645	185.8658709	559.401851	172.9980795	521.9326073	1554.988627	390.6457054	512.5498174	1485.83392	341.7133876
**ENSG00000119953**	SMNDC1	824.0512102	858.2849973	775.1500249	687.368829	738.5491379	709.0644969	656.7566859	787.4621895	669.6783521	643.5347708	582.1930493	662.2811447
**ENSG00000119965**	C10orf88	299.5271464	276.7531216	315.1082559	273.7754045	264.6756692	286.053842	204.278907	176.0991394	239.6398025	233.4949168	197.2834267	226.6811581
**ENSG00000119969**	HELLS	2396.217171	2714.983155	1993.242921	2857.478457	2779.672421	2299.432807	2403.341341	2109.867048	2085.944895	2476.058562	2204.607803	2410.601868
**ENSG00000119977**	TCTN3	412.0256051	332.8043867	432.3578395	565.1327157	537.4418609	547.44787	518.8684237	463.5062255	555.2515188	615.0597809	538.0457091	634.3689126
**ENSG00000119979**	FAM45A	314.9956845	287.2627338	310.2228566	349.9636669	339.8019508	377.4848445	325.8248566	262.4873965	335.3081385	286.0154537	332.4846561	335.7926111
**ENSG00000119986**	AVPI1	320.6206074	441.4037129	311.851323	406.0583216	491.210303	503.4395866	226.7495867	250.8582081	240.1087649	175.279382	289.7169203	202.998052
**ENSG00000120008**	WDR11	1349.981505	1166.566955	1336.970946	1077.519711	1040.210053	1214.400992	1499.407177	1518.439749	1368.901337	1476.902807	1328.55902	1256.896272
**ENSG00000120029**	C10orf76	410.6193744	458.9197332	388.3892457	488.9444533	454.2250567	386.2106249	560.7455996	654.5571786	512.5759376	559.3753563	500.7963908	527.7949353
**ENSG00000120049**	KCNIP2	11.24984587	21.01922442	14.65619795	35.16381342	33.5178795	25.41857747	36.77020326	61.46856754	25.79293373	25.3111021	40.00852709	30.4497078
**ENSG00000120051**	CFAP58	12.65607661	66.56087734	22.79853014	5.86063557	8.090522638	17.0721789	15.32091802	6.645250545	8.910286198	14.5538837	6.898021912	13.53320347
**ENSG00000120053**	GOT1	538.5863712	462.4229373	544.7220238	623.7390714	532.8187051	639.6376361	536.2321308	446.8930991	559.4721807	534.0642542	462.1674681	591.2318265
**ENSG00000120055**	C10orf95	0	0	5.699632536	0.837233653	4.623155793	3.793817533	1.021394535	1.661312636	5.158586746	3.163887762	2.759208765	1.691650434
**ENSG00000120063**	GNA13	2594.495705	2602.880624	2399.545298	2198.575572	2423.689425	2310.434878	1903.879413	2086.608671	2028.731478	1859.733226	1974.213871	2081.575858
**ENSG00000120071**	KANSL1	1598.884345	1450.326485	1612.181775	1636.791791	1562.626658	1474.656875	1778.247885	1923.800033	1614.637651	1745.833267	1935.584948	1686.575482
**ENSG00000120093**	HOXB3	1.406230734	0	8.142332194	5.86063557	3.467366845	0.758763507	4.08557814	11.62918845	2.813774589	2.53111021	1.379604382	2.53747565
**ENSG00000120129**	DUSP1	1262.795199	2052.877585	1261.247257	1033.983561	1398.504627	1302.796941	861.0355929	1216.08085	996.0762044	885.8885733	1328.55902	1033.598415
**ENSG00000120137**	PANK3	3476.202375	3405.114356	3417.336822	3474.519659	3402.642664	3526.353397	2428.876204	3090.041503	2729.361351	2548.195203	2705.404194	2981.533889
**ENSG00000120149**	MSX2	485.1496033	206.6890402	357.4483833	704.9507357	492.366092	524.6849648	1214.438102	721.0096841	1377.811624	1157.982921	760.1620147	1363.470249
**ENSG00000120156**	TEK	9.843615139	14.01281628	7.328098975	107.1659076	107.4883722	70.94438787	0	63.12988017	6.56547404	2.53111021	75.87824103	11.84155303
**ENSG00000120158**	RCL1	236.2467633	140.1281628	226.356835	344.940265	183.7704428	279.6043522	170.5728873	106.3240087	196.9642212	186.0366004	126.9236032	208.0730033
**ENSG00000120159**	CAAP1	397.9632978	406.3716722	378.618447	425.3146957	346.7366845	400.6271315	370.7662162	348.8756536	405.1835408	375.8698661	342.1418868	404.3044536
**ENSG00000120160**	EQTN	4.218692202	0	0.814233219	3.348934611	0	0	8.171156279	0	1.875849726	2.53111021	9.657230676	0
**ENSG00000120162**	MOB3B	981.5490524	486.9453658	691.2840033	813.7911106	575.5828962	596.0087344	1668.95867	1413.777053	1692.954378	1301.623425	1518.944425	1394.765782
**ENSG00000120211**	INSL4	4.218692202	0	7.328098975	23.44254228	28.89472371	23.14228695	27.57765244	1.661312636	17.35160996	17.71777147	4.138813147	27.91223215
**ENSG00000120217**	CD274	2143.095639	2228.037789	3193.422687	1369.714256	648.3976	1670.41786	491.2907713	438.5865359	641.0716438	227.7999189	245.5695801	356.0924163
**ENSG00000120251**	GRIA2	1.406230734	0	5.699632536	4.186168264	6.93473369	2.27629052	0	0	2.344812157	0	0	0
**ENSG00000120253**	NUP43	1653.727343	1432.810465	1507.145689	1574.836501	1562.626658	1755.399373	1549.455509	1377.228175	1647.465022	1654.080522	1423.751723	1816.832566
**ENSG00000120254**	MTHFD1L	1643.883728	1481.855322	1775.842652	1422.459976	1571.87297	1809.271582	2031.55373	2116.512298	1983.242123	1866.061002	1808.661345	1827.828293
**ENSG00000120256**	LRP11	181.4037647	140.1281628	140.862347	159.074394	196.4841212	135.0599042	173.6370709	237.567707	176.3298742	200.5904841	198.6630311	190.3106738
**ENSG00000120265**	PCMT1	967.4867451	1078.986854	1025.933857	1269.246218	1272.523632	1194.673141	981.560148	1174.548034	1071.579156	1144.694592	1163.006494	1302.570834
**ENSG00000120278**	PLEKHG1	0	7.006408141	0	3.348934611	3.467366845	0.758763507	1.021394535	1.661312636	1.406887294	1.265555105	0	0.845825217
**ENSG00000120279**	MYCT1	1.406230734	0	2.442699658	0	0	2.655672273	10.21394535	13.29050109	3.751699452	1.898332657	24.83287888	9.304077385
**ENSG00000120306**	CYSTM1	382.4947597	287.2627338	421.7728077	288.0083766	298.1935487	353.9631758	184.8724108	176.0991394	278.0947219	179.0760473	195.9038223	266.4349433
**ENSG00000120314**	WDR55	682.0219061	700.6408141	771.0788588	704.1135021	760.509128	743.5882365	628.1576389	659.5411165	717.9814826	725.7958526	702.2186306	637.7522134
**ENSG00000120318**	ARAP3	154.6853808	164.6505913	162.0324107	172.4701325	253.1177797	144.544448	7.149761744	1.661312636	2.813774589	6.327775524	1.379604382	1.691650434
**ENSG00000120327**	PCDHB14	2.812461468	7.006408141	4.885399317	5.023401917	6.93473369	2.655672273	2.04278907	0	2.344812157	8.226108181	5.518417529	1.691650434
**ENSG00000120329**	SLC25A2	2.812461468	0	0	1.674467306	3.467366845	0	10.21394535	3.322625272	5.158586746	4.429442867	0	1.691650434
**ENSG00000120332**	TNN	7.031153671	3.50320407	0	1.674467306	1.155788948	0	0	0	0.937924863	0	0	0.845825217
**ENSG00000120333**	MRPS14	406.4006822	413.3780803	371.2903481	380.9413121	427.6419109	373.3116453	368.7234271	413.6668464	401.9008038	435.350956	442.8530067	424.6042588
**ENSG00000120334**	CENPL	631.3975996	560.5126513	567.520554	926.8176537	696.9407358	920.0007518	619.9864827	627.9761765	702.5057223	710.6091913	597.3686975	873.7374489
**ENSG00000120337**	TNFSF18	220.7782253	122.6121425	251.5980648	156.5626931	108.6441611	206.004292	11.23533988	1.661312636	17.8205724	6.960553076	1.379604382	2.53747565
**ENSG00000120341**	SEC16B	21.09346101	66.56087734	40.71166097	4.186168264	4.623155793	7.967016819	3.064183605	11.62918845	6.096511609	1.265555105	1.379604382	0
**ENSG00000120370**	GORAB	320.6206074	329.3011826	356.6341501	237.7743574	264.6756692	264.0497003	357.4880872	315.6494009	287.005008	334.7393252	310.410986	318.0302815
**ENSG00000120437**	ACAT2	1089.828819	1159.560547	938.810902	1260.873881	1321.066768	1023.951352	721.1045416	1241.000539	894.3113568	826.4074834	1243.023548	990.4613288
**ENSG00000120438**	TCP1	4491.500965	4214.354497	4308.107964	4866.00199	4404.711682	4809.043105	3209.221629	2666.406781	3452.970383	3362.579913	2886.132368	4200.368027
**ENSG00000120451**	SNX19	2654.963626	2483.771686	3149.454093	2655.705147	2764.647164	2992.942652	2501.395216	2895.667925	2579.293373	2636.151283	2669.53448	2679.574287
**ENSG00000120458**	MSANTD2	350.1514528	308.2819582	371.2903481	391.8253495	298.1935487	312.9899465	346.2527473	416.9894717	306.2324677	300.5693374	427.6773585	306.1887285
**ENSG00000120471**	TP53AIP1	16.87476881	28.02563256	4.071166097	10.04680383	8.090522638	8.725780326	5.106972674	3.322625272	1.406887294	0.632777552	4.138813147	1.691650434
**ENSG00000120498**	TEX11	66.09284451	49.04485699	72.46675653	54.42018744	80.90522638	72.84129663	15.32091802	9.967875817	25.3239713	14.5538837	30.35129641	16.91650434
**ENSG00000120509**	PDZD11	638.4287533	917.8394665	859.8302797	782.8134654	746.6396606	822.1202594	595.4730138	618.0083006	632.63032	535.9625869	623.5811808	626.7564856
**ENSG00000120519**	SLC10A7	286.8710698	322.2947745	285.79586	315.6370871	283.1682923	284.1569332	239.0063212	209.3253922	226.5088544	209.4493698	223.4959099	286.7347485
**ENSG00000120526**	NUDCD1	1688.883112	1467.842506	1619.509873	764.3943251	572.1155294	812.2563338	607.7297483	515.0069172	608.2442736	406.8759662	393.187249	521.0283335
**ENSG00000120533**	ENY2	615.9290616	648.092753	599.2756495	523.271033	496.9892478	464.7426478	346.2527473	416.9894717	372.3561706	304.3660027	366.9747657	354.4007658
**ENSG00000120539**	MASTL	932.3309767	1159.560547	852.5021808	784.4879327	850.6606659	876.7512319	723.1473307	855.5760076	747.5261157	743.513624	783.6152892	820.4504603
**ENSG00000120549**	KIAA1217	2323.093173	2224.534585	2154.461099	2068.804356	2049.213805	1893.873712	2684.224838	2242.772059	2511.762783	2349.503052	2150.803232	2339.55255
**ENSG00000120555**	SEPT7P9	5.624922937	0	1.628466439	0.837233653	1.155788948	3.035054026	16.34231256	4.983937908	7.972361335	6.327775524	22.07367012	9.304077385
**ENSG00000120594**	PLXDC2	577.9608317	231.2114686	246.7126655	974.5399719	591.7639415	509.5096947	895.7630071	702.7352451	858.670212	942.838553	852.5955083	1055.589871
**ENSG00000120616**	EPC1	499.2119106	486.9453658	523.5519601	589.4124916	492.366092	527.7200188	554.6172324	538.2652941	536.0240592	475.2159418	584.9522581	523.5658092
**ENSG00000120647**	CCDC77	147.6542271	196.1794279	175.0601422	169.1211979	172.2125533	150.6145561	135.8454731	139.5502614	158.0403394	153.1321677	110.3683506	151.4027138
**ENSG00000120656**	TAF12	534.367679	535.9902228	478.769133	457.9668081	472.7176798	481.4354449	301.3113878	350.5369662	344.6873871	320.1854415	314.5497992	385.6962988

**ENSG00000120658**	ENOX1	0	0	2.442699658	0	1.155788948	0	5.106972674	3.322625272	4.220661883	1.265555105	9.657230676	0
**ENSG00000120662**	MTRF1	157.4978422	112.1025303	179.9455415	185.0286373	173.3683422	202.969238	182.8296217	227.5998311	217.5985682	199.324929	278.6800852	192.8481494
**ENSG00000120664**	SPART-AS1	8.437384405	31.52883663	16.28466439	5.023401917	15.02525633	3.035054026	18.38510163	14.95181373	13.13094808	8.226108181	16.55525259	13.53320347
**ENSG00000120669**	SOHLH2	21.09346101	31.52883663	26.86969624	27.62871054	25.42735686	35.66188481	13.27812895	26.58100218	19.22745969	20.24888168	9.657230676	22.83728085
**ENSG00000120675**	DNAJC15	637.0225226	809.2401403	559.3782218	428.6636303	555.9344841	416.5611651	162.401731	204.3414542	146.3162786	127.188288	117.2663725	111.6489286
**ENSG00000120685**	PROSER1	921.0811309	742.6792629	1043.032754	991.284645	926.9427365	1017.122481	997.9024606	988.4810185	979.6625193	1023.83408	893.9836397	1029.369289
**ENSG00000120686**	UFM1	894.3627469	1012.425976	991.7360613	654.7167165	834.4796206	828.1903675	893.720218	792.4461274	748.4640406	747.9430669	762.9212234	836.5211394
**ENSG00000120688**	WBP4	347.3389913	399.365264	381.8753799	319.8232554	299.3493376	331.9590341	285.9904698	292.391024	267.3085859	280.3204557	297.9945466	281.6597972
**ENSG00000120690**	ELF1	1079.985204	1355.739975	1184.709334	1065.79844	1141.919481	1139.283405	1327.812895	1456.971182	1232.43327	1149.756813	1360.289921	1261.125398
**ENSG00000120693**	SMAD9	1.406230734	0	2.442699658	7.535102876	9.246311586	3.793817533	85.79714093	192.7122658	41.7376564	105.0410737	226.2551187	40.59961041
**ENSG00000120694**	HSPH1	5963.824544	4876.460066	6732.894492	6745.591541	7042.222062	8029.235427	2715.888068	2712.923535	3944.911973	2877.872308	2654.358832	4656.267818
**ENSG00000120696**	KBTBD7	106.8735358	129.6185506	146.5619795	145.6786556	134.071518	155.9259006	214.4928523	215.9706427	267.3085859	248.0488005	215.2182836	239.3685363
**ENSG00000120697**	ALG5	499.2119106	465.9261414	484.4687656	459.6412754	419.5513882	455.6374857	528.0609745	458.5222876	461.927995	480.2781623	492.5187645	517.6450327
**ENSG00000120699**	EXOSC8	658.1159836	763.6984874	647.3154095	583.551856	562.8692178	634.3262915	612.8367209	513.3456046	545.8722702	573.2964625	558.7397748	561.6279439
**ENSG00000120705**	ETF1	3552.138834	2949.697827	3576.926533	4226.35548	3411.888975	4218.725097	3287.869008	2656.438905	3318.847127	3299.302158	2563.304942	3767.305516
**ENSG00000120708**	TGFBI	347.3389913	714.6536304	456.7848361	618.7156695	1538.35509	863.4728705	4665.730235	10718.78913	2958.68398	4299.723468	9951.08641	2545.088077
**ENSG00000120709**	FAM53C	658.1159836	606.0543042	639.1730773	620.3901368	768.5996506	722.72224	582.1948849	721.0096841	584.3271896	572.6636849	702.2186306	561.6279439
**ENSG00000120725**	SIL1	420.4629895	374.8428355	404.6739101	354.1498352	331.7114282	363.8271014	482.0982205	450.2157244	426.7558126	427.1248479	360.0767438	433.9083362
**ENSG00000120727**	PAIP2	1198.108586	1604.467464	1256.361858	1186.360086	1247.096275	1118.79679	1053.057765	1191.16116	955.7454353	1079.518504	1107.822319	1122.410063
**ENSG00000120733**	KDM3B	1866.068184	2291.095462	2257.868718	2053.73415	2265.346339	2186.377044	2597.406302	2609.922151	2552.093552	2399.492479	2607.452283	2352.239928
**ENSG00000120738**	EGR1	545.6175249	528.9838146	451.0852036	384.2902467	409.1492877	372.1735	292.118837	328.939902	467.5555442	167.6860514	274.5412721	318.8761067
**ENSG00000120742**	SERP1	1708.570342	1769.118056	1766.886086	1373.900424	1511.771944	1728.083886	1405.43888	1265.920229	1454.721462	1243.40789	1103.683506	1416.757238
**ENSG00000120756**	PLS1	552.6486785	427.3908966	434.8005392	733.4166799	636.8397105	678.3345749	284.9690752	202.6801416	269.1844357	235.3932495	237.2919538	304.497078
**ENSG00000120784**	ZFP30	177.1850725	136.6249587	127.8346155	174.9818334	171.0567643	154.0289918	307.439755	254.1808333	277.6257594	266.3993496	292.476129	293.5013502
**ENSG00000120798**	NR2C1	237.6529941	196.1794279	239.3845665	222.7041517	173.3683422	193.4846942	315.6109113	460.1836002	321.2392655	333.4737701	292.476129	321.4135824
**ENSG00000120800**	UTP20	2176.845176	1320.707935	2104.792872	2545.190305	2036.500127	2442.080346	2601.49188	1922.13872	2694.658131	2770.300124	2117.692727	3064.42476
**ENSG00000120802**	TMPO	6595.222143	5226.780473	5474.904168	6861.967019	5996.233064	6003.336864	9576.595159	7432.712734	9356.26947	8945.576258	7585.064894	10335.13832
**ENSG00000120805**	ARL 1.00	975.9241295	1054.464425	1164.353504	974.5399719	920.0080028	1076.685416	1430.973743	1312.436983	1386.72191	1453.490038	1265.097219	1659.509075
**ENSG00000120820**	GLT8D2	2.812461468	0	3.256932878	6.697869223	3.467366845	0.758763507	14.29952349	1.661312636	9.379248629	8.858885733	4.138813147	5.074951301
**ENSG00000120832**	MTERF2	122.3420739	77.07048955	129.4630819	146.5158893	124.8252064	137.3361947	258.4128173	219.293268	227.9157417	246.7832454	252.467602	249.5184389
**ENSG00000120833**	SOCS2	2.812461468	14.01281628	7.328098975	9.209570182	16.18104528	19.34846942	20.4278907	9.967875817	9.848211061	17.71777147	13.79604382	5.074951301
**ENSG00000120837**	NFYB	489.3682955	581.5318757	500.75343	545.8763417	520.1050267	532.6519816	1867.10921	1262.597603	1421.42513	1730.013828	1060.91577	1607.913737
**ENSG00000120860**	WASHC3	307.9645308	399.365264	309.4086234	270.4264699	346.7366845	264.8084638	534.1893417	624.6535512	493.8174403	513.182595	623.5811808	545.5572648
**ENSG00000120868**	APAF1	279.8399161	472.9325495	298.0093583	251.1700959	435.7324335	282.6394062	754.8105613	1121.386029	797.7050959	715.0386342	1074.711814	847.5168672
**ENSG00000120875**	DUSP4	6624.752989	6389.844224	6987.749489	6424.093818	6297.893979	7905.177594	501.5047166	551.5557952	748.933003	426.4920703	420.7793366	700.3432795
**ENSG00000120885**	CLU	449.9938349	388.8556518	464.1129351	623.7390714	484.2755693	512.165367	1481.022076	1397.163927	1336.54293	1486.394471	1398.918844	1348.245396
**ENSG00000120889**	TNFRSF10B	1731.070034	2049.374381	1927.29003	1132.777132	1239.005753	1419.646521	346.2527473	375.4566558	349.8459739	303.1004476	355.9379306	290.1180494
**ENSG00000120896**	SORBS3	523.1178331	788.2209158	552.0501228	552.5742109	736.23756	666.5737406	224.7067977	270.7939597	255.5845251	246.1504679	329.7254474	238.5227111
**ENSG00000120899**	PTK2B	628.5851382	620.0671205	793.0631557	884.9559711	691.1617911	932.5203496	719.0617526	578.1367974	760.6570638	727.6941852	551.8417529	704.5724056
**ENSG00000120910**	PPP3CC	281.2461468	294.2691419	293.123959	221.0296844	270.4546139	261.0146463	93.96829721	137.8889488	105.5165471	79.7299716	74.49863664	99.80737558
**ENSG00000120913**	PDLIM2	29.53084542	42.03844885	5.699632536	44.3733836	88.99574902	29.212395	29.62044151	117.9531972	34.2342575	43.02887356	144.8584601	38.06213475
**ENSG00000120915**	EPHX2	25.31215321	28.02563256	34.19779522	21.76807497	25.42735686	29.97115851	5.106972674	0	1.875849726	0	4.138813147	5.920776517
**ENSG00000120925**	RNF170	199.6847643	245.2242849	233.684934	158.2371604	234.6251565	184.0001504	212.4500633	189.3896405	196.0262963	230.3310291	182.1077785	214.8396051
**ENSG00000120942**	UBIAD1	319.2143767	206.6890402	337.9067861	293.8690122	253.1177797	379.3817533	319.6964894	227.5998311	317.9565285	295.507117	224.8755143	334.1009606
**ENSG00000120948**	TARDBP	3677.29337	3044.284337	3545.171437	3868.85671	2993.493376	3726.287581	3610.629681	3847.600065	3743.258128	3778.314765	3435.214912	4041.352886
**ENSG00000120949**	TNFRSF8	25.31215321	17.51602035	8.142332194	25.11700959	10.40210053	15.93403364	0	16.61312636	8.910286198	0	0	5.074951301
**ENSG00000120963**	ZNF706	707.3340593	956.3747112	854.9448804	1013.889954	847.1932991	884.338867	414.6861812	380.4405937	462.3969574	442.9442867	406.9832928	548.0947405
**ENSG00000120992**	LYPLA1	3985.257901	3173.902888	3733.259311	2829.012513	2555.449365	3195.153126	1714.921424	1378.889488	1952.290602	1705.335504	1420.992514	1795.686935
**ENSG00000121005**	CRISPLD1	8.437384405	14.01281628	3.256932878	5.023401917	9.246311586	11.76083435	0	0	0	0	0	0
**ENSG00000121022**	COPS5	794.5203648	711.1504263	851.6879475	951.0974297	922.3195807	922.656424	583.2162794	586.4433606	591.8305885	554.3131359	564.2581924	636.060563
**ENSG00000121039**	RDH10	4086.506513	2007.335932	2169.93153	9541.951942	7308.05352	7264.401812	3436.99261	3766.195746	3399.977628	3930.814155	4301.606464	4103.098127
**ENSG00000121057**	AKAP1	693.2717519	581.5318757	639.9873105	857.3272605	709.6544142	831.984185	687.398522	395.3924074	605.8994614	789.0736078	510.4536215	688.5017265
**ENSG00000121058**	COIL	811.3951336	858.2849973	761.3080602	796.2092039	754.7301832	742.4500912	669.0134203	634.621427	653.7336294	721.3664097	744.9863664	781.5425003
**ENSG00000121060**	TRIM25	5477.26871	5366.908636	5770.470826	2673.287054	2938.015506	3165.940731	2579.021201	2457.081389	2642.603301	1516.135016	1409.955679	1614.680339
**ENSG00000121064**	SCPEP1	216.5595331	287.2627338	235.3134004	167.4467306	227.6904228	194.2434577	285.9904698	317.3107135	283.722271	248.6815781	252.467602	266.4349433
**ENSG00000121067**	SPOP	1124.984587	1156.057343	1090.258281	853.9783259	975.4858723	916.2069342	1139.876301	1636.392947	1178.033628	1078.885727	1338.216251	1005.686183
**ENSG00000121073**	SLC35B1	704.5215978	648.092753	754.7941944	546.7135753	536.286072	588.0417176	464.7345134	519.9908551	479.7485674	487.2387153	514.5924346	512.5700814
**ENSG00000121075**	TBX4	1.406230734	10.50961221	12.21349829	0.837233653	4.623155793	4.931962793	0	0	2.344812157	0	1.379604382	0
**ENSG00000121089**	NACA3P	1.406230734	0	2.442699658	1.674467306	2.311577897	4.55258104	2.04278907	1.661312636	0.937924863	1.898332657	0	5.920776517
**ENSG00000121101**	TEX14	28.12461468	31.52883663	10.58503185	10.88403749	5.778944741	19.72785117	10.21394535	9.967875817	2.813774589	8.226108181	1.379604382	6.766601734
**ENSG00000121104**	FAM117A	12.65607661	45.54165292	12.21349829	17.58190671	30.05051265	20.48661468	9.192550814	11.62918845	9.379248629	6.327775524	0	5.920776517
**ENSG00000121152**	NCAPH	915.4562079	588.5382838	861.4587462	1196.40689	1052.923732	1101.34523	1023.437324	1113.079466	1012.020927	1145.32737	1022.286847	1176.542877
**ENSG00000121207**	LRAT	88.59253625	178.6634076	96.07951989	133.9573845	210.3535886	115.7114348	0	0	1.406887294	1.265555105	0	2.53747565
**ENSG00000121210**	TMEM131L	12.65607661	14.01281628	13.02773151	7.535102876	16.18104528	12.14021611	188.957989	370.4727179	172.1092123	214.5115903	227.6347231	173.3941694
**ENSG00000121211**	MND1	12.65607661	14.01281628	14.65619795	13.39573845	9.246311586	7.208253313	10.21394535	11.62918845	11.72406079	10.75721839	16.55525259	7.612426951
**ENSG00000121236**	TRIM6	30.93707615	42.03844885	46.41129351	46.88508456	42.76419109	54.63097248	82.73295732	33.22625272	71.75125201	68.97275321	48.28615338	59.20776517
**ENSG00000121270**	ABCC11	7.031153671	7.006408141	17.09889761	1.674467306	8.090522638	4.173199286	2.04278907	1.661312636	0.937924863	2.53111021	0	4.229126084

**ENSG00000121274**	PAPD5	413.4318358	336.3075908	441.3144049	512.3869956	550.1555394	568.3138665	432.0498883	438.5865359	360.6321098	358.1520946	386.289227	425.450084
**ENSG00000121281**	ADCY7	57.6554601	63.05767327	61.06749146	53.58295378	56.63365846	37.17941182	12.25673442	58.14594226	38.92388181	27.20943475	55.18417529	54.97863909
**ENSG00000121289**	CEP89	503.4306028	616.5639164	512.9669283	498.9912571	620.6586652	450.7055229	475.9698533	513.3456046	451.1418591	442.9442867	506.3148083	439.8291127
**ENSG00000121310**	ECHDC2	570.9296781	669.1119775	659.5289078	583.551856	603.321831	520.1323838	1000.966644	802.4140032	722.2021444	1011.811306	893.9836397	695.2683282
**ENSG00000121316**	PLBD1	479.5246803	413.3780803	452.71367	365.8711063	471.5618909	446.1529419	657.7780805	779.1556263	692.1885488	637.2069952	698.0798174	759.5510447
**ENSG00000121318**	TAS2R10	0	3.50320407	2.442699658	0	2.311577897	0.379381753	1.021394535	0	0.937924863	2.53111021	0	0
**ENSG00000121350**	PYROXD1	136.4043812	126.1153465	135.9769476	109.6776085	86.68417112	122.1609246	184.8724108	137.8889488	204.4676201	217.0427005	184.8669872	230.9102842
**ENSG00000121380**	BCL2L14	0	7.006408141	2.442699658	0	0	0	3.064183605	3.322625272	2.813774589	0	0	0
**ENSG00000121388**	AL139327.1	122.3420739	122.6121425	96.07951989	131.4456835	141.0062517	131.2660866	87.83993	73.09775599	88.16493711	102.5099635	82.77626294	112.4947538
**ENSG00000121390**	PSPC1	1476.542271	1306.695118	1433.050466	1369.714256	1227.447863	1345.667079	1500.428572	1229.371351	1353.425577	1469.309477	1229.227505	1322.870639
**ENSG00000121406**	ZNF549	0	0	0	0.837233653	0	0	0	3.322625272	2.344812157	0	0	3.383300867
**ENSG00000121413**	ZSCAN18	94.21745919	98.08971397	94.45105346	128.9339825	120.2020506	127.8516509	414.6861812	358.8435294	359.2252225	365.1126477	291.0965247	281.6597972
**ENSG00000121417**	ZNF211	75.93645964	98.08971397	75.72368941	77.86272972	102.8652164	90.67223904	308.4611495	304.0202124	289.8187826	309.4282231	270.4024589	327.3343589
**ENSG00000121454**	LHX4	403.5882207	217.1986524	202.7440716	558.4348465	331.7114282	373.691027	316.6323058	116.2918845	354.0666357	279.6876782	143.4788558	380.6213475
**ENSG00000121481**	RNF2	215.1533023	322.2947745	237.7561001	228.5647872	236.9367344	234.0785418	301.3113878	387.0858442	281.3774589	277.1565679	286.9577115	340.0217371
**ENSG00000121486**	TRMT1L	808.5826721	889.8138339	740.9522297	641.3209781	933.8774702	705.6500611	884.5276672	847.2694444	860.0770993	831.4697038	951.9270238	895.7289046
**ENSG00000121542**	SEC22A	260.1526858	255.7338971	236.9418669	267.9147689	234.6251565	253.4270112	277.8193135	317.3107135	257.4603749	261.9699067	264.8840414	253.747565
**ENSG00000121552**	CSTA	312.183223	371.3396315	397.3458111	321.4977227	303.9724934	416.9405469	50.04833221	29.90362745	63.30992825	35.43554293	37.24931832	79.50757038
**ENSG00000121570**	DPPA4	0	0	0	0	0	0.758763507	55.15530488	51.50069172	79.25465092	62.64497769	71.73942788	76.97009473
**ENSG00000121577**	POPDC2	0	0	0	6.697869223	2.311577897	3.41443578	3.064183605	4.983937908	5.627549177	10.12444084	2.759208765	1.691650434
**ENSG00000121578**	B4GALT4	1905.442645	1793.640484	1726.988658	2108.991572	1861.975996	2101.01615	1478.979287	2018.494853	1656.84427	1365.533958	1637.590402	1533.481118
**ENSG00000121579**	NAA50	3801.041674	3457.662418	3732.445078	3905.694991	3653.448865	3933.430018	4101.920452	3473.804722	4418.564029	4206.705168	3519.370779	4854.190919
**ENSG00000121621**	KIF18A	1213.577124	931.8522827	1034.076189	1123.567562	901.5153796	937.0729307	1037.736847	885.4796351	961.8419469	930.8157796	778.0968716	1050.514919
**ENSG00000121644**	DESI2	1376.699889	1478.352118	1341.856346	1943.219308	1814.588649	1965.576864	1182.774871	1207.774286	1188.819764	1144.694592	1092.646671	1438.748694
**ENSG00000121653**	MAPK8IP1	15.46853808	42.03844885	27.68392946	36.83828073	34.67366845	33.00621254	27.57765244	23.25837691	15.94472267	24.67832454	22.07367012	23.68310607
**ENSG00000121671**	CRY2	122.3420739	143.6313669	179.1313083	119.7244124	136.3830959	151.3733196	253.3058446	309.0041503	250.4259384	200.5904841	293.8557334	202.1522268
**ENSG00000121680**	PEX16	94.21745919	108.5993262	140.0481137	123.073347	134.071518	177.9300423	153.2091802	117.9531972	216.6606433	199.9577066	168.3117346	175.0858199
**ENSG00000121690**	DEPDC7	85.78007478	77.07048955	60.25325824	92.09570182	127.1367843	110.4000902	54.13391035	132.9050109	56.27549177	68.33997566	96.57230676	68.51184256
**ENSG00000121691**	CAT	485.1496033	297.772346	452.71367	838.9081202	668.0460121	663.9180683	559.7242051	485.1032897	671.0852394	650.4953238	540.8049179	680.8892995
**ENSG00000121716**	PILRB	208.1221487	147.134571	152.261612	159.9116277	122.5136285	135.4392859	175.67986	156.1633878	163.6678886	173.3810494	171.0709434	131.1029086
**ENSG00000121741**	ZMYM2	1711.382803	2087.909626	1555.185449	1771.586409	2078.108529	1687.490039	2193.955461	2556.760147	1721.092123	2036.278164	2370.160329	1982.614308
**ENSG00000121742**	GJB6	351.5576835	154.1409791	271.1396621	770.2549606	591.7639415	627.49742	274.7551299	172.7765142	365.3217341	352.4570967	321.4478211	424.6042588
**ENSG00000121743**	GJA3	44.99938349	59.5544692	25.2412298	34.32657977	77.43785953	28.83301325	2.04278907	9.967875817	6.56547404	0.632777552	6.898021912	0.845825217
**ENSG00000121749**	TBC1D15	880.3004396	1026.438793	961.6094322	834.7219519	879.5553896	846.4006916	1035.694058	907.0766993	860.0770993	902.3407897	833.2810469	866.125022
**ENSG00000121753**	ADGRB2	32.34330689	77.07048955	30.94086234	33.48934611	62.41260321	45.5258104	12.25673442	9.967875817	7.034436472	9.491663286	2.759208765	1.691650434
**ENSG00000121766**	ZCCHC17	437.3377583	514.9709984	448.6425039	617.8784358	642.6186552	568.3138665	547.4674707	598.072549	546.8101951	599.2403421	575.2950274	601.3817291
**ENSG00000121774**	KHDRBS1	3775.729521	3002.245888	3433.621486	3659.548297	3423.446865	3373.083169	3196.964894	2879.054798	3213.799543	3366.376579	3500.056318	3745.31406
**ENSG00000121775**	TMEM39B	198.2785335	238.2178768	165.2893435	194.2382075	189.5493875	235.5960688	235.9421376	222.6158932	226.9778168	257.5404638	226.2551187	253.747565
**ENSG00000121797**	CCRL2	36.56199909	52.54806106	30.12662912	0	4.623155793	4.173199286	7.149761744	24.91968954	7.034436472	2.53111021	8.277626294	5.920776517
**ENSG00000121807**	CCR2	0	0	0.814233219	0.837233653	0	0	55.15530488	29.90362745	25.79293373	68.97275321	53.80457091	38.90795997
**ENSG00000121851**	POLR3GL	116.7171509	119.1089384	100.150686	101.305272	105.1767943	69.0474791	93.96829721	88.04956971	88.63389954	99.97885328	108.9887462	126.8737825
**ENSG00000121858**	TNFSF10	705.9278285	1979.3103	639.1730773	48.55955187	292.4146039	40.97322936	3697.448216	9281.753698	2584.45196	1741.403824	5401.151157	1146.938994
**ENSG00000121864**	ZNF639	573.7421395	413.3780803	535.7654584	622.0646041	529.3513383	527.3406371	647.5641351	566.5076089	642.9474935	658.0886545	498.037182	721.4889099
**ENSG00000121879**	PIK3CA	1058.891743	1359.243179	1120.38491	886.6304384	993.9784955	1005.741028	2560.636099	1815.814711	2637.913677	2669.055716	1794.865301	2938.396803
**ENSG00000121892**	PDS5A	2760.430931	2392.68838	2732.566684	2854.129523	2818.969245	2924.653936	2755.722455	2505.259455	2684.80992	2688.67182	2502.60235	2996.758743
**ENSG00000121897**	LIAS	74.53022891	52.54806106	106.6645517	112.1893095	106.3325832	113.0557625	168.5300983	88.04956971	149.1300532	158.1943881	128.3032076	154.7860147
**ENSG00000121898**	CPXM2	7.031153671	7.006408141	14.65619795	0	16.18104528	9.484543833	5.106972674	6.645250545	1.406887294	3.163887762	1.379604382	0.845825217
**ENSG00000121900**	TMEM54	296.7146849	332.8043867	271.9538953	295.5434795	282.0125034	331.2002706	205.3003015	330.6012146	314.6737915	250.5799107	275.9208765	266.4349433
**ENSG00000121903**	ZSCAN20	255.9339936	280.2563256	338.7210193	288.8456102	270.4546139	294.4002406	284.9690752	385.4245316	274.3430224	299.3037823	273.1616677	280.813972
**ENSG00000121904**	CSMD2	9.843615139	17.51602035	12.21349829	5.023401917	17.33683422	18.21032416	0	0	0	1.265555105	0	0
**ENSG00000121905**	HPCA	7.031153671	0	0	3.348934611	0	0	0	0	1.875849726	0	4.138813147	8.458252168
**ENSG00000121931**	LRIF1	1447.011425	1362.746383	1534.015385	941.8878595	1007.847963	1110.829774	926.4048431	820.6884422	1057.510283	761.8641731	696.7002131	822.9879359
**ENSG00000121940**	CLCC1	1199.514816	1303.191914	1334.528247	1216.500498	1312.976245	1204.537067	1327.812895	1342.34061	1217.895434	1274.41399	1287.170889	1316.104037
**ENSG00000121957**	GPSM2	2494.653322	1870.710974	1892.278002	3524.753679	3252.3901	3002.427196	2165.356414	2104.88311	2119.71019	2346.339164	2259.791978	2515.484195
**ENSG00000121964**	GTDC1	172.9663803	94.5865099	116.4353504	169.1211979	161.8104528	165.0310627	148.1022076	154.5020752	144.4404289	152.4993901	179.3485697	160.7067912
**ENSG00000121966**	CXCR4	2.812461468	21.01922442	0.814233219	0	16.18104528	1.896908767	0	3.322625272	0	0	9.657230676	0
**ENSG00000121988**	ZRANB3	80.15515185	63.05767327	56.99632536	64.46699127	85.52838217	65.25366157	84.77574639	69.77513072	96.60626088	66.441643	40.00852709	87.96582254
**ENSG00000121989**	ACVR2A	29.53084542	59.5544692	69.20982365	59.44358935	64.7241811	65.63304332	118.481766	106.3240087	88.16493711	96.18218796	140.719647	104.8823269
**ENSG00000122008**	POLK	898.5814391	1050.961221	882.6288099	879.9325692	941.9679928	986.3925586	1153.15443	936.9803268	1025.620838	993.4607572	1034.703287	1043.748318
**ENSG00000122026**	RPL21	10448.29435	10972.03515	9571.311495	10455.37386	9914.357598	9514.514991	6775.931344	8015.833469	6700.066258	6851.715337	7627.83263	7839.108109
**ENSG00000122033**	MTIF3	430.3066047	578.0286716	483.6545324	381.7785457	540.9092278	435.5302528	377.9159779	426.9573475	335.7771009	372.0732008	318.6886123	338.3300867
**ENSG00000122034**	GTF3A	935.1434382	910.8330583	916.8266051	983.7495421	1108.401601	1075.926652	816.0942334	769.1877505	834.2841656	943.4713306	710.4962569	931.2535637
**ENSG00000122035**	RASL11A	1.406230734	14.01281628	3.256932878	10.04680383	12.71367843	3.41443578	2.04278907	3.322625272	5.627549177	6.327775524	5.518417529	4.229126084
**ENSG00000122042**	UBL3	624.366446	542.9966309	565.0778543	1272.595152	1079.506878	1341.49388	870.2281437	981.835768	875.5528595	966.8841	1054.017748	1081.810452
**ENSG00000122068**	FYTTD1	1915.28626	1586.951444	1734.316757	1846.100205	1614.637161	1830.896341	1359.476126	1476.906934	1336.54293	1189.621798	1321.660998	1426.061315
**ENSG00000122085**	MTERF4	227.8093789	245.2242849	253.2265312	322.3349564	230.0020007	288.3301325	327.8676457	360.504842	288.4118953	342.9654334	349.0399087	353.5549406
**ENSG00000122126**	OCRL	839.5197483	606.0543042	882.6288099	859.0017278	831.0122538	801.6336447	812.0086552	842.2855065	785.9810351	757.4347302	1000.213177	833.1378385

**ENSG00000122133**	PAEP	1.406230734	3.50320407	3.256932878	0	2.311577897	0.379381753	1.021394535	1.661312636	2.344812157	0.632777552	0	3.383300867
**ENSG00000122140**	MRPS2	596.2418313	472.9325495	589.5048509	559.2720801	488.8987251	652.1572339	341.1457746	358.8435294	395.8042921	389.7909723	259.3656239	411.0710554
**ENSG00000122188**	LAX1	0	0	0	6.697869223	0	2.27629052	0	3.322625272	1.406887294	2.53111021	0	0
**ENSG00000122203**	KIAA1191	1272.638814	1092.99967	1160.282338	1343.760013	1181.216305	1368.429984	939.6829721	1000.110207	1072.048118	1071.292396	918.8165186	1103.801908
**ENSG00000122218**	COPA	4713.685421	5086.65231	4970.893805	3557.405791	4111.141289	4153.092053	4354.204902	4739.724951	4418.564029	4157.348519	4431.289276	4513.323357
**ENSG00000122223**	CD244	0	0	0.814233219	0	0	0.379381753	139.9310513	167.7925762	45.02039342	154.3977228	153.1360864	36.37048432
**ENSG00000122224**	LY9	0	0	0	0	0	0	6.128367209	11.62918845	10.78613592	9.491663286	8.277626294	5.920776517
**ENSG00000122257**	RBBP6	3137.300768	3857.027682	2990.678615	2742.777447	2961.131285	2499.746373	3602.458525	3948.940136	3228.806341	3342.963809	3533.166823	3366.384363
**ENSG00000122299**	ZC3H7A	2614.182935	2301.605074	2517.609115	2355.975499	1993.735936	2180.686318	2497.309638	2299.256688	2482.687112	2554.522979	2526.055624	2519.713321
**ENSG00000122335**	SERAC1	213.7470716	196.1794279	267.8827292	246.1466939	323.6209055	266.7053726	279.8621026	358.8435294	296.3842567	304.3660027	344.9010956	276.5848459
**ENSG00000122359**	ANXA11	3065.583	3191.418908	3487.360879	3404.192033	3282.440613	4006.271315	2466.667802	2231.14287	2568.507237	2580.466859	2564.684547	2627.133123
**ENSG00000122367**	LDB3	0	0	0	0	4.623155793	1.13814526	0	0	2.344812157	5.694997971	1.379604382	0
**ENSG00000122376**	FAM35A	904.2063621	662.1055693	802.8339544	1055.751636	980.1090281	917.3450795	975.4317808	654.5571786	832.4083158	837.7974794	627.719994	965.0865723
**ENSG00000122378**	FAM213A	1078.578973	1173.573364	925.7831705	1192.220722	1370.765693	1144.59475	750.7249831	744.268061	682.3403378	982.7035388	951.9270238	947.3242428
**ENSG00000122386**	ZNF205	102.6548436	112.1025303	126.206149	87.90953355	125.9809954	129.7485596	57.19809395	88.04956971	88.63389954	62.01220013	57.94338406	60.89941561
**ENSG00000122390**	NAA60	243.277917	367.8364274	351.7487508	377.5923774	291.258815	332.7177976	327.8676457	330.6012146	398.6180667	372.7059784	292.476129	344.2508632
**ENSG00000122406**	RPL5	9611.587068	8582.849973	8758.706742	10514.81745	9982.549146	9863.546204	8702.281437	9012.621051	9170.560347	9527.731606	9144.017846	10307.22609
**ENSG00000122417**	ODF2L	891.5502855	931.8522827	728.7387314	726.7188107	831.0122538	587.6623359	1316.577555	1427.067554	1070.172269	1222.526231	1189.218978	1138.480742
**ENSG00000122432**	SPATA1	11.24984587	17.51602035	16.28466439	9.209570182	1.155788948	6.828871559	10.21394535	11.62918845	10.78613592	10.12444084	8.277626294	4.229126084
**ENSG00000122435**	TRMT13	471.0872959	287.2627338	389.2034789	499.8284908	412.6166545	437.8065433	608.7511428	505.0390414	603.5546493	615.6925585	634.6180159	573.469497
**ENSG00000122477**	LRRC39	19.68723028	0	4.071166097	8.372336529	17.33683422	4.173199286	23.4920743	21.59706427	10.78613592	20.88165923	8.277626294	10.99572782
**ENSG00000122481**	RWDD3	40.78069129	94.5865099	69.20982365	27.62871054	48.54313583	47.04333741	68.43343384	76.42038126	47.36520558	51.25498174	68.98021912	54.13281387
**ENSG00000122482**	ZNF644	1967.316797	2003.832728	2085.251275	1987.592692	1739.462367	1933.329415	2038.703492	2212.868431	1805.505361	1992.616512	2092.859848	2044.359549
**ENSG00000122483**	CCDC18	667.9595987	521.9774065	577.2913526	711.6486049	744.3280827	641.5345448	881.4634836	717.6870588	757.3743268	802.994714	775.3376629	917.7203602
**ENSG00000122484**	RPAP2	825.4574409	1121.025303	992.5502945	1076.682478	1126.894225	1023.951352	1094.934941	935.3190141	1049.537922	1097.869053	969.8618808	1057.281521
**ENSG00000122490**	PQLC1	118.1233817	150.637775	177.5028418	168.2839642	168.7451864	204.1073833	137.8882622	176.0991394	170.2333626	183.5054902	195.9038223	194.5397999
**ENSG00000122507**	BBS9	187.0286876	171.6569995	224.7283686	205.9594786	195.3283323	161.6166269	370.7662162	322.2946514	243.8604644	248.0488005	307.6517773	257.1308659
**ENSG00000122512**	PMS2	355.7763757	210.1922442	455.1563697	402.709387	342.1135287	340.3054327	443.2852281	430.2799728	473.6520558	518.2448154	538.0457091	426.2959093
**ENSG00000122515**	ZMIZ2	1355.606428	1296.185506	1373.611441	1313.619601	1423.931984	1530.046611	1039.779637	1021.707271	1111.440963	976.3757633	878.8079915	939.7118158
**ENSG00000122545**	7-Sep	3037.458386	3086.322786	2713.83932	3554.056856	3448.874222	3082.097364	5524.723039	5990.693366	5597.535582	5613.369667	5897.808734	6473.946209
**ENSG00000122547**	EEPD1	22.49969175	45.54165292	24.42699658	22.60530863	20.80420107	25.03919572	3.064183605	14.95181373	6.096511609	5.694997971	0	9.304077385
**ENSG00000122550**	KLHL7	306.5583	269.7467134	311.0370898	334.8934611	340.9577397	375.9673175	351.35972	322.2946514	274.8119848	253.7437985	342.1418868	314.6469806
**ENSG00000122557**	HERPUD2	665.1471373	819.7497525	686.398604	442.8966024	576.7386852	533.0313634	857.9714093	960.2387037	737.6779047	703.6486382	780.8560804	702.8807551
**ENSG00000122565**	CBX3	3682.918293	3243.966969	3038.718375	3986.906655	3614.152041	3442.510029	3570.795294	3031.895561	3571.617878	3655.55592	3393.82678	4265.496568
**ENSG00000122566**	HNRNPA2B1	21497.04923	18493.41429	20051.30726	22608.65756	19973.18881	19617.83046	20236.88992	18477.11914	19989.9926	20299.50388	19252.37916	22722.24862
**ENSG00000122574**	WIPF3	1.406230734	14.01281628	0.814233219	0.837233653	0	1.13814526	0	0	0.468962431	1.265555105	1.379604382	0
**ENSG00000122591**	FAM126A	1317.638198	994.909956	1166.796203	2301.555312	1754.487623	2113.915129	413.6647866	421.9734096	457.2383707	431.5542907	424.9181498	510.8784309
**ENSG00000122641**	INHBA	434.5252969	1387.268812	956.7240329	564.295482	1138.452114	1179.877253	27.57765244	116.2918845	34.2342575	32.90443272	104.8499331	42.29126084
**ENSG00000122642**	FKBP9	915.4562079	1057.967629	1048.732387	859.8389615	1134.984747	970.0791432	1699.600506	2069.995545	1825.670746	1641.424971	2121.83154	1827.828293
**ENSG00000122643**	NT5C3A	1220.608277	1488.86173	968.1232979	562.6210147	495.8334588	499.2663873	927.4262377	817.365817	814.118781	549.883693	495.2779732	631.8314369
**ENSG00000122644**	ARL4A	227.8093789	175.1602035	197.8586723	371.7317419	263.5198802	314.1280917	486.1837986	360.504842	383.1423065	479.6453847	390.4280402	464.358044
**ENSG00000122674**	CCZ1	549.8362171	504.4613861	534.9512252	497.3167898	417.2398103	520.8911473	456.5633571	368.8114052	387.3629684	355.6209844	292.476129	422.0667832
**ENSG00000122678**	POLM	210.9346101	189.1730198	193.7875062	123.9105806	164.1220307	158.9609546	253.3058446	232.5837691	226.9778168	217.675478	189.0058004	197.9231007
**ENSG00000122687**	MRM2	15.46853808	0	5.699632536	13.39573845	4.623155793	4.931962793	9.192550814	1.661312636	15.47576024	9.491663286	9.657230676	9.304077385
**ENSG00000122691**	TWIST1	0	0	3.256932878	1.674467306	1.155788948	1.13814526	2.04278907	3.322625272	3.751699452	5.062220419	0	0.845825217
**ENSG00000122692**	SMU1	1380.918581	1331.217547	1458.291696	1520.416314	1323.378346	1430.648592	1405.43888	1368.921612	1336.54293	1329.465638	1394.780031	1440.440344
**ENSG00000122694**	GLIPR2	1.406230734	0	0.814233219	1.674467306	3.467366845	1.896908767	2.04278907	4.983937908	5.158586746	0	5.518417529	0.845825217
**ENSG00000122696**	SLC25A51	220.7782253	196.1794279	141.6765802	205.9594786	173.3683422	222.6970892	208.3644851	137.8889488	158.0403394	174.0138269	117.2663725	173.3941694
**ENSG00000122705**	CLTA	1244.5142	1173.573364	1371.168742	1233.245171	1358.052014	1331.250572	882.4848781	1003.432832	975.9108199	972.579098	1139.55322	1075.043851
**ENSG00000122707**	RECK	9.843615139	59.5544692	14.65619795	16.74467306	15.02525633	23.5216687	18.38510163	43.19412854	24.85500887	35.43554293	31.73090079	26.22058172
**ENSG00000122728**	TAF1L	0	0	3.256932878	2.511700959	0	0.758763507	1.021394535	0	0	1.265555105	0	0
**ENSG00000122729**	ACO1	361.4012987	217.1986524	328.9502207	601.9709964	617.1912984	610.4252411	495.3763494	593.0886111	560.4101056	492.3009358	511.8332258	557.3988179
**ENSG00000122733**	PHF24	0	0	0.814233219	0	0	0	7.149761744	9.967875817	10.78613592	5.694997971	1.379604382	7.612426951
**ENSG00000122735**	DNAI1	1.406230734	7.006408141	1.628466439	10.04680383	15.02525633	7.208253313	0	11.62918845	1.875849726	10.75721839	5.518417529	1.691650434
**ENSG00000122741**	DCAF10	1039.204513	1166.566955	1065.831284	1036.495262	1064.481621	1057.716328	1211.373918	1113.079466	1278.391588	1030.794633	1056.776957	1188.38443
**ENSG00000122778**	KIAA1549	1701.539188	1334.720751	1770.957252	3096.927282	2916.055516	3339.697574	685.3557329	574.8141721	1028.434612	718.8352995	692.5613999	1047.977444
**ENSG00000122779**	TRIM24	520.3053716	497.454978	519.480794	708.2996703	815.9869975	690.0954093	2169.441992	2834.199357	2003.87647	1610.418871	2334.290615	1843.898973
**ENSG00000122783**	C7orf49	559.6798322	413.3780803	527.6231262	749.3241193	569.8039515	647.6046529	539.2963144	471.8127887	571.6652039	618.2236687	620.821972	636.9063882
**ENSG00000122786**	CALD1	395.1508363	234.7146727	391.6461786	403.5466207	308.5956492	581.2128461	2756.74385	3443.901095	2788.91958	2600.71574	3017.194784	3083.032915
**ENSG00000122787**	AKR1D1	0	0	0.814233219	0.837233653	0	1.896908767	4.08557814	0	1.406887294	0.632777552	0	0.845825217
**ENSG00000122852**	SFTPA1	0	0	0.814233219	1.674467306	0	0.379381753	0	0	0.937924863	0	0	3.383300867
**ENSG00000122861**	PLAU	4041.50713	5972.96294	8227.012449	2840.733784	3495.10578	5605.744787	1123.533988	825.6723802	1258.226204	720.1008546	525.6292697	915.1828846
**ENSG00000122862**	SRGN	2158.564177	2497.784502	2966.251618	738.4400818	679.6039016	955.6626366	873.2923273	913.7219499	1526.941677	567.6014645	590.4706756	1051.360744
**ENSG00000122863**	CHST3	727.0212896	812.7433443	943.6963013	1159.568609	1284.081522	1675.349823	832.4365459	780.816939	982.4762939	804.2602691	765.6804322	930.4077385
**ENSG00000122870**	BICC1	0	0	0.814233219	0	0	0	7.149761744	1.661312636	1.875849726	1.265555105	0	0.845825217
**ENSG00000122873**	CISD1	302.3396078	353.8236111	364.7764823	337.4051621	447.290323	408.2147666	524.9967909	561.523671	505.5415011	542.2903624	448.3714243	600.5359039
**ENSG00000122877**	EGR2	0	0	5.699632536	1.674467306	0	1.896908767	1.021394535	0	5.627549177	0	4.138813147	1.691650434

**ENSG00000122882**	ECD	776.2393653	742.6792629	881.0003434	907.5612797	787.0922738	840.7099653	737.4468542	730.9775599	696.4092107	703.6486382	633.2384115	728.2555116
**ENSG00000122884**	P4HA1	975.9241295	4673.27423	1141.554974	801.2326058	2907.964994	836.1573843	1150.090246	5482.331699	1065.482644	1152.287923	5042.454017	1297.495883
**ENSG00000122912**	SLC25A16	330.4642225	329.3011826	324.0648213	397.6859851	280.8557144	375.9673175	437.1568609	453.5383497	397.6801419	491.6681582	506.3148083	389.9254249
**ENSG00000122952**	ZWINT	764.9895194	798.7305281	875.3007109	1094.264384	953.5258823	932.5203496	702.71944	722.6709967	706.7263842	775.1525017	859.4935302	927.8702628
**ENSG00000122958**	VPS26A	1627.008959	1902.23981	1761.186454	1716.328988	1741.773945	1697.733346	1321.684528	1402.147865	1425.176829	1325.668972	1312.003768	1582.538981
**ENSG00000122965**	RBM19	1243.107969	1142.044527	1351.627144	1241.617507	1089.908978	1372.223802	1743.520471	1787.572396	1695.768152	1620.543312	1665.182489	1623.138591
**ENSG00000122966**	CIT	1901.223953	1544.912995	1929.73273	2405.372285	2204.089524	2264.150304	2949.787417	2367.370506	3008.393998	3137.943882	2755.069951	3298.718345
**ENSG00000122970**	IFT81	406.4006822	458.9197332	283.3531604	321.4977227	382.5661419	250.0125754	506.6116893	533.2813562	411.2800524	525.838146	593.2298844	473.6621214
**ENSG00000122971**	ACADS	25.31215321	63.05767327	42.34012741	33.48934611	55.47786952	48.94024618	69.45482837	73.09775599	82.0684255	96.18218796	81.39665856	68.51184256
**ENSG00000122986**	HVCN1	19.68723028	7.006408141	13.84196473	2.511700959	1.155788948	1.13814526	1.021394535	0	0.468962431	0	0	0.845825217
**ENSG00000123009**	NME2P1	1.406230734	0	1.628466439	0	5.778944741	0.758763507	0	0	1.406887294	1.898332657	4.138813147	0
**ENSG00000123064**	DDX54	725.6150588	465.9261414	682.3274379	785.3251664	798.6501633	901.7904276	817.1156279	872.189134	1015.303664	947.9007735	838.7994644	916.874535
**ENSG00000123066**	MED13L	982.9552832	1264.656669	1179.009702	1211.477096	1199.708928	1408.64445	2228.682875	1950.381035	1918.525307	2231.806427	2065.26776	2082.421684
**ENSG00000123080**	CDKN2C	130.7794583	129.6185506	87.12295448	309.7764516	282.0125034	259.4971193	237.9849266	383.7632189	260.7431119	346.1293212	382.1504139	383.1588232
**ENSG00000123091**	RNF11	1026.548436	1078.986854	1043.032754	1296.037695	1221.668918	1234.128843	830.3937569	995.126269	857.2633247	855.5152508	946.4086063	1153.705596
**ENSG00000123094**	RASSF8	191.2473798	227.7082646	239.3845665	176.6563008	234.6251565	188.5527314	638.3715843	679.4768682	684.2161875	543.5559175	616.6831589	789.1549273
**ENSG00000123095**	BHLHE41	44.99938349	59.5544692	23.61276336	52.74572013	160.6546638	43.62890163	85.79714093	179.4217647	104.5786222	70.23830831	103.4703287	115.0322295
**ENSG00000123096**	SSPN	91.40499772	56.05126513	39.08319453	5.023401917	5.778944741	15.17527013	171.5942819	131.2436983	195.0883715	68.97275321	12.41643944	61.74524082
**ENSG00000123104**	ITPR2	215.1533023	241.7210809	193.7875062	163.2605623	254.2735686	185.8970591	255.3486337	282.4231481	259.3362246	213.2460352	353.1787219	251.2100894
**ENSG00000123106**	CCDC91	137.8106119	143.6313669	144.9335131	110.5148422	143.3178296	130.8867049	230.8351649	269.1326471	206.3434698	247.416023	306.2721729	186.0815477
**ENSG00000123119**	NECAB1	5.624922937	7.006408141	38.26896131	5.023401917	28.89472371	46.66395566	46.9841486	21.59706427	29.54463318	27.8422123	24.83287888	14.37902869
**ENSG00000123124**	WWP1	1409.043196	1583.44824	1314.172416	1456.786556	1370.765693	1322.904174	875.3351164	814.0431917	797.7050959	984.6018715	1066.434188	1079.272977
**ENSG00000123130**	ACOT9	835.3010561	795.227324	887.5142092	808.7677087	632.2165547	759.1428884	657.7780805	724.3323094	623.7200338	632.7775524	600.1279063	639.4438639
**ENSG00000123131**	PRDX4	1504.666886	1450.326485	1390.710339	1539.672688	1604.23506	1478.450693	953.9824956	1272.565479	1010.145077	1253.532331	1168.524912	1322.870639
**ENSG00000123136**	DDX39A	738.2711354	725.1632426	633.4734447	712.4858386	587.1407857	744.347	492.3121658	478.4580392	637.3199443	635.9414401	498.037182	641.1355143
**ENSG00000123143**	PKN1	711.5527515	896.820242	849.2452479	728.393278	976.6416613	917.3450795	339.1029856	270.7939597	298.7290688	366.3782028	317.3090079	295.1930007
**ENSG00000123144**	TRIR	991.3926676	1012.425976	852.5021808	948.5857287	1054.079521	1104.380284	738.4682487	852.2533823	832.8772783	804.8930466	808.448168	925.3327872
**ENSG00000123146**	ADGRE5	1767.632033	1863.704565	1967.187458	1309.433433	1186.99525	1438.99499	441.2424391	295.7136492	430.0385496	418.8987397	241.4307669	378.9296971
**ENSG00000123154**	WDR83	95.62368992	70.06408141	83.05178838	104.6542066	112.111528	80.80831345	79.66877372	111.3079466	97.54418574	93.65107775	57.94338406	91.34912341
**ENSG00000123159**	GIPC1	1663.570959	1268.159873	1680.577365	1863.682111	1600.767693	2118.847092	1333.941263	1025.029896	1414.390693	1204.175682	1099.544693	1283.962679
**ENSG00000123178**	SPRYD7	156.0916115	126.1153465	135.9769476	152.3765248	188.3935986	176.791897	243.0918993	299.0362745	226.039892	241.0882475	244.1899757	274.0473702
**ENSG00000123179**	EBPL	298.1209156	241.7210809	192.973273	251.1700959	189.5493875	210.1774913	197.1291452	151.1794499	235.888103	225.2688086	142.0992514	238.5227111
**ENSG00000123191**	ATP7B	122.3420739	98.08971397	72.46675653	261.2168997	277.3893476	145.6825933	995.8596715	1978.62335	899.938906	1349.081742	2683.330524	1285.654329
**ENSG00000123200**	ZC3H13	1459.667502	1572.938628	1427.350834	1575.673735	1635.441362	1441.650663	2241.961004	2285.966187	2052.648562	2254.586419	2484.667493	2398.760315
**ENSG00000123213**	NLN	1688.883112	1226.121425	1367.097575	1660.234334	1734.839211	1557.362097	1376.839833	1071.54665	1414.859656	1415.523385	1190.598582	1547.014321
**ENSG00000123219**	CENPK	815.6138258	732.1696507	754.7941944	817.9772789	708.4986253	718.9284225	810.9872607	917.0445751	768.6294252	881.4591305	760.1620147	939.7118158
**ENSG00000123240**	OPTN	2597.308166	3632.822621	2993.121315	627.088006	1102.622657	1004.602883	1667.937275	2063.350294	1594.941229	768.1919486	949.167815	770.5467725
**ENSG00000123243**	ITIH5	5.624922937	7.006408141	9.770798633	2.511700959	5.778944741	10.62268909	168.5300983	186.0670152	144.4404289	148.7027248	271.7820633	128.5654329
**ENSG00000123268**	ATF1	642.6474455	686.6279978	539.8366245	659.7401185	633.3723436	640.3963996	1019.351746	1023.368584	951.055811	1084.580725	951.9270238	1041.210842
**ENSG00000123297**	TSFM	5.624922937	3.50320407	9.770798633	2.511700959	15.02525633	6.070108053	14.29952349	16.61312636	9.379248629	13.92110615	4.138813147	7.612426951
**ENSG00000123329**	ARHGAP9	2.812461468	0	2.442699658	0	1.155788948	3.035054026	3.064183605	0	0	0.632777552	0	1.691650434
**ENSG00000123338**	NCKAP1L	8.437384405	0	2.442699658	1.674467306	1.155788948	1.517527013	0	0	0	3.163887762	0	0
**ENSG00000123342**	MMP19	30.93707615	45.54165292	78.16638907	14.2329721	19.64841212	33.00621254	55.15530488	29.90362745	150.5369405	93.65107775	34.49010956	159.0151408
**ENSG00000123349**	PFDN5	63.28038304	38.53524477	39.89742775	46.04785091	36.98524634	41.73199286	73.54040651	93.03350762	64.24785311	70.23830831	108.9887462	102.3448512
**ENSG00000123352**	SPATS2	642.6474455	648.092753	556.1212889	671.4613896	788.2480627	648.3634164	785.4523973	794.1074401	922.9180651	909.3013428	834.6606513	1011.606959
**ENSG00000123353**	ORMDL2	445.7751427	441.4037129	455.1563697	478.0604158	520.1050267	553.1385963	478.0126423	426.9573475	471.3072436	465.091501	500.7963908	495.653577
**ENSG00000123358**	NR4A1	30.93707615	77.07048955	47.22552673	67.81592588	62.41260321	51.2165367	21.44928523	13.29050109	80.66153821	18.35054902	52.42496653	54.13281387
**ENSG00000123360**	PDE1B	1.406230734	3.50320407	1.628466439	1.674467306	13.86946738	6.449489806	13.27812895	6.645250545	2.813774589	0	0	0
**ENSG00000123364**	HOXC13	12.65607661	0	3.256932878	9.209570182	12.71367843	9.863925586	122.5673442	61.46856754	100.8269228	112.6344043	45.52694462	119.2613556
**ENSG00000123374**	CDK2	998.4238213	1247.140649	871.2295448	1124.404796	1069.104777	1147.629804	1667.937275	1799.201585	1816.760459	1876.185443	1636.210797	1925.098193
**ENSG00000123384**	LRP1	430.3066047	637.5831408	416.8874084	556.7603792	762.8207059	564.1406672	781.3668192	1875.621966	1091.74454	1039.020741	2043.19409	1086.039578
**ENSG00000123388**	HOXC11	1.406230734	7.006408141	4.885399317	0.837233653	3.467366845	1.517527013	4.08557814	3.322625272	2.344812157	7.593330629	1.379604382	0.845825217
**ENSG00000123395**	ATG101	292.4959927	185.6698157	290.6812593	228.5647872	210.3535886	309.1961289	170.5728873	230.9224564	231.6674411	180.3416024	271.7820633	227.5269833
**ENSG00000123405**	NFE2	0	0	0	0	1.155788948	1.896908767	1.021394535	0	1.875849726	0	6.898021912	1.691650434
**ENSG00000123411**	IKZF4	116.7171509	119.1089384	92.82258702	105.4914403	129.4483622	95.22482008	182.8296217	144.5341993	145.8473162	139.8438391	146.2380645	116.7238799
**ENSG00000123415**	SMUG1	255.9339936	276.7531216	340.3494857	270.4264699	300.5051265	290.2270413	467.798697	365.4887799	440.8246856	437.8820662	466.3062812	362.859018
**ENSG00000123416**	TUBA1B	14945.42024	13746.57277	15132.52438	16660.94969	15544.20557	17857.49913	12505.95469	13092.80489	15828.41999	14061.58277	12969.6608	15703.59097
**ENSG00000123427**	EEF1AKMT3	61.8741523	94.5865099	92.0083538	62.79252397	110.955739	63.3567528	74.56180105	93.03350762	66.59266527	95.54941041	115.8867681	77.81591994
**ENSG00000123444**	KBTBD4	305.1520693	357.3268152	289.0527929	349.1264332	328.2440613	324.7507808	295.1830206	385.4245316	269.6533981	312.5921109	442.8530067	339.1759119
**ENSG00000123472**	ATPAF1	845.1446712	830.2593647	817.4901523	828.0240827	879.5553896	762.9367059	923.3406595	830.6563181	921.0422154	759.9658404	800.1705417	842.4419159
**ENSG00000123473**	STIL	1760.600879	1755.105239	1706.632828	1568.138632	1484.03301	1621.477614	1478.979287	1456.971182	1645.589172	1448.427817	1233.366318	1601.147135
**ENSG00000123485**	HJURP	1070.141589	623.5703245	948.5817007	1193.057955	837.9469875	1207.572121	820.1798115	701.0739324	978.7245944	863.741359	747.7455752	1131.71414
**ENSG00000123505**	AMD 1.00	3088.082692	1769.118056	2645.44373	2798.034868	1897.805453	2933.379717	2459.51804	2045.075855	2750.46466	2639.315171	1821.077785	2847.893505
**ENSG00000123545**	NDUFAF4	241.8716863	59.5544692	177.5028418	296.3807131	175.6799201	285.6744602	210.4072742	117.9531972	225.1019671	192.9971535	148.9972733	212.3021294
**ENSG00000123552**	USP45	260.1526858	276.7531216	259.740397	221.0296844	263.5198802	254.5651565	284.9690752	245.8742701	311.8600169	384.7287518	313.1701948	384.0046484
**ENSG00000123560**	PLP1	2.812461468	0	0	9.209570182	2.311577897	1.13814526	3.064183605	0	2.813774589	1.265555105	0	5.920776517
**ENSG00000123562**	MORF4L2	5487.112325	5268.818922	5012.419699	5562.58039	5247.281825	5526.074619	4241.851503	4201.459657	3989.463404	4581.942257	4537.518813	5001.364507

**ENSG00000123570**	RAB9B	21.09346101	10.50961221	5.699632536	18.41914036	25.42735686	17.83094241	2.04278907	6.645250545	5.158586746	5.062220419	16.55525259	5.920776517
**ENSG00000123572**	NRK	3029.021001	1863.704565	1347.555978	1465.996126	1275.990999	764.8336147	5306.144609	3561.854292	3177.220473	4467.40952	3229.653859	3395.988245
**ENSG00000123575**	FAM199X	1096.859973	1019.432384	994.1787609	1343.760013	1465.540386	1258.409276	1131.705145	1039.98171	1080.489442	1053.574625	1078.850627	1230.67569
**ENSG00000123595**	RAB9A	452.8062964	507.9645902	420.9585745	428.6636303	456.5366346	427.563236	490.2693767	779.1556263	432.8523242	404.9776335	652.5528728	471.970471
**ENSG00000123600**	METTL8	502.0243721	346.817203	352.562984	438.7104341	395.2798203	331.2002706	433.0712828	392.0697821	416.9076016	412.5709642	315.9294035	426.2959093
**ENSG00000123607**	TTC21B	455.6187579	497.454978	495.8680306	475.5487148	503.9239814	502.3014414	777.281241	872.189134	761.5949887	778.949167	815.3461899	731.6388125
**ENSG00000123609**	NMI	1556.697423	1390.772016	1241.70566	380.1040784	479.6524135	353.9631758	1140.897695	1089.821089	977.7866696	464.4587234	423.5385454	411.0710554
**ENSG00000123612**	ACVR1C	7.031153671	28.02563256	22.79853014	3.348934611	30.05051265	18.21032416	0	0	0.937924863	0	0	0
**ENSG00000123636**	BAZ2B	240.4654555	430.8941007	348.4918179	264.5658343	324.7766945	333.4765612	326.8462512	197.6962037	244.7983892	280.9532333	215.2182836	228.3728085
**ENSG00000123643**	SLC36A1	397.9632978	210.1922442	433.986306	451.2689389	337.4903729	437.4271616	498.440533	460.1836002	703.4436472	529.0020338	528.3884784	667.356096
**ENSG00000123684**	LPGAT1	1520.135424	1457.332893	1406.995003	1342.085546	1562.626658	1440.891899	1953.927745	1666.296574	1775.022803	1899.598212	1734.162709	1910.719165
**ENSG00000123685**	BATF3	2.812461468	7.006408141	0	0	2.311577897	0.758763507	2.04278907	3.322625272	0	0	0	0
**ENSG00000123689**	G0S2	0	84.07689769	14.65619795	4.186168264	19.64841212	12.51959786	0	3.322625272	1.875849726	0	1.379604382	1.691650434
**ENSG00000123700**	KCNJ2	185.6224569	112.1025303	83.8660216	181.6797027	54.32208057	75.87635066	1099.02052	1108.095528	1058.448208	858.046361	702.2186306	855.9751194
**ENSG00000123728**	RAP2C	539.9926019	651.5959571	543.9077906	548.3880426	540.9092278	537.5839444	494.3549549	461.8449128	527.5827354	489.7698255	460.7878637	538.7906631
**ENSG00000123737**	EXOSC9	897.1752084	980.8971397	900.5419407	1005.517617	805.5848969	823.2584047	635.3074007	599.7338616	590.4237012	601.7714523	495.2779732	677.5059986
**ENSG00000123739**	PLA2G12A	291.089762	199.682632	252.412298	500.6657244	442.6671672	435.9096345	306.4183605	259.1647712	288.4118953	368.909313	354.5583263	361.1673676
**ENSG00000123810**	B9D2	12.65607661	14.01281628	25.2412298	18.41914036	26.58314581	12.89897961	20.4278907	14.95181373	18.75849726	26.5766572	34.49010956	34.67883389
**ENSG00000123815**	COQ8B	157.4978422	231.2114686	254.8549977	200.9360767	203.4188549	197.6578935	325.8248566	353.8595915	305.2945429	283.4843435	360.0767438	241.906012
**ENSG00000123836**	PFKFB2	1583.415807	1558.925811	1602.410976	1877.915083	1779.91498	2107.845021	982.5815426	1302.469107	1118.475399	978.9068735	1394.780031	1124.947538
**ENSG00000123843**	C4BPB	2.812461468	0	2.442699658	3.348934611	1.155788948	4.931962793	23.4920743	11.62918845	16.4136851	5.062220419	19.31446135	12.68737825
**ENSG00000123870**	ZNF137P	94.21745919	94.5865099	110.7357178	79.53719702	94.77469376	86.49903975	207.3430906	186.0670152	175.8609118	205.6527045	146.2380645	197.0772755
**ENSG00000123892**	RAB38	36.56199909	101.592918	71.65252331	51.90848648	64.7241811	67.15057033	2.04278907	21.59706427	5.158586746	2.53111021	8.277626294	3.383300867
**ENSG00000123901**	GPR83	0	3.50320407	6.513865756	2.511700959	2.311577897	7.587635066	6.128367209	3.322625272	5.158586746	5.062220419	6.898021912	6.766601734
**ENSG00000123908**	AGO2	4305.878508	3972.633416	4361.033123	5496.438931	4544.562145	5919.872879	5193.79121	4909.17884	5870.940679	5803.202933	5177.655247	6287.018836
**ENSG00000123933**	MXD4	40.78069129	38.53524477	65.95289078	37.67551438	85.52838217	46.2845739	136.8668677	237.567707	168.8264753	180.97438	248.3287888	142.9444616
**ENSG00000123965**	PMS2P5	9.843615139	10.50961221	23.61276336	22.60530863	28.89472371	14.41650663	42.89857046	16.61312636	28.60670832	25.3111021	17.93485697	21.14563042
**ENSG00000123975**	CKS2	1589.04073	921.3426705	1550.30005	1653.536464	1481.721432	1886.286077	990.7526988	852.2533823	1190.226651	1040.286296	790.5133111	1361.778599
**ENSG00000123977**	DAW1	64.68661377	73.56728548	48.03975995	212.6573478	136.3830959	154.7877553	41.87717593	49.83937908	32.35840777	73.40219608	34.49010956	72.74096864
**ENSG00000123983**	ACSL3	7063.496978	5247.799697	6701.953629	6456.745931	5449.544891	6739.337466	6572.673832	6646.911857	6724.452305	6360.679957	6362.735411	6796.205617
**ENSG00000123989**	CHPF	327.6517611	262.7403053	333.0213868	331.5445265	436.8882224	477.6416274	557.681416	455.1996623	672.9610891	572.6636849	587.7114669	775.6217238
**ENSG00000123992**	DNPEP	892.9565162	606.0543042	837.0317496	408.5700226	461.1597904	423.3900367	541.3391035	707.719183	594.1754007	323.9821068	278.6800852	299.4221267
**ENSG00000123999**	INHA	15.46853808	0	6.513865756	15.07020575	25.42735686	16.31341539	34.72741419	41.5328159	30.48255804	39.23220825	71.73942788	28.75805737
**ENSG00000124006**	OBSL1	298.1209156	311.7851623	279.2819943	375.0806765	384.8777198	341.443578	1179.710688	986.8197059	878.8355965	1097.236276	965.7230676	844.9793916
**ENSG00000124019**	FAM124B	4.218692202	0	0	0.837233653	0	1.13814526	0	0	0.937924863	0	0	0.845825217
**ENSG00000124067**	SLC12A4	1525.760347	1331.217547	1375.239908	1379.76106	1291.016255	1427.234156	789.5379755	892.1248856	955.2764729	806.7913793	829.1422338	862.7417211
**ENSG00000124074**	ENKD1	122.3420739	122.6121425	78.98062229	102.9797393	124.8252064	106.6062727	136.8668677	121.2758224	118.1785327	115.1655145	121.4051856	116.7238799
**ENSG00000124097**	HMGB1P1	9.843615139	0	4.885399317	10.88403749	8.090522638	5.311344546	3.064183605	6.645250545	1.875849726	3.163887762	2.759208765	6.766601734
**ENSG00000124098**	FAM210B	291.089762	357.3268152	188.9021069	339.0796294	409.1492877	317.5425275	791.5807645	440.2478486	718.9194074	731.4908506	560.1193792	736.7137638
**ENSG00000124102**	PI3	0	0	6.513865756	2.511700959	0	1.13814526	0	0	5.627549177	0	2.759208765	16.91650434
**ENSG00000124103**	FAM209A	1.406230734	0	4.885399317	0	0	0.758763507	1.021394535	0	0.468962431	0	1.379604382	0
**ENSG00000124104**	SNX21	105.4673051	101.592918	175.0601422	152.3765248	210.3535886	245.8393761	112.3533988	166.1312636	136.4680676	134.1488411	146.2380645	130.2570834
**ENSG00000124107**	SLPI	223.5906867	451.9133251	342.7921854	179.1680017	420.7071772	286.8126055	84.77574639	106.3240087	103.6406974	111.3688492	120.0255813	175.9316451
**ENSG00000124116**	WFDC3	7.031153671	3.50320407	15.47043117	6.697869223	5.778944741	9.105162079	4.08557814	9.967875817	1.406887294	3.163887762	11.03683506	10.1499026
**ENSG00000124120**	TTPAL	712.9589822	620.0671205	806.9051205	738.4400818	604.4776199	848.6769821	670.0348149	742.6067483	717.9814826	686.5636443	798.7909374	658.0520187
**ENSG00000124126**	PREX1	306.5583	262.7403053	311.0370898	210.1456469	226.5346339	268.2228996	34.72741419	68.11381808	69.40643986	35.43554293	37.24931832	50.74951301
**ENSG00000124140**	SLC12A5	14.06230734	21.01922442	8.142332194	1.674467306	3.467366845	3.035054026	4.08557814	1.661312636	1.406887294	4.429442867	1.379604382	4.229126084
**ENSG00000124143**	ARHGAP40	251.7153014	105.0961221	106.6645517	213.4945815	94.77469376	121.7815428	206.321696	215.9706427	196.4952588	144.2732819	128.3032076	118.4155303
**ENSG00000124145**	SDC4	4062.600591	7412.779813	4749.422369	4012.023665	5235.723936	5321.208472	5614.605758	7246.645719	5805.285939	5084.367633	6122.684249	5196.750132
**ENSG00000124151**	NCOA3	3819.322674	3478.681642	4074.42303	3106.974086	2888.316582	3368.151206	2497.309638	2144.754613	3078.738362	2207.128103	2085.961826	2793.760691
**ENSG00000124155**	PIGT	1103.891126	1061.470833	1250.662225	1097.613319	1397.348838	1352.116569	1503.492755	1809.169461	1737.974771	1621.176089	1782.448862	1616.371989
**ENSG00000124157**	SEMG2	0	0	1.628466439	3.348934611	6.93473369	4.55258104	0	0	1.875849726	0	0	0
**ENSG00000124159**	MATN4	1.406230734	7.006408141	1.628466439	4.186168264	0	2.27629052	1.021394535	1.661312636	1.406887294	3.796665314	0	0
**ENSG00000124160**	NCOA5	773.4269038	732.1696507	807.7193537	765.2315587	684.2270574	692.3716998	1027.522902	1011.739395	967.0005337	952.3302163	917.4369142	980.3114262
**ENSG00000124164**	VAPB	1455.44881	1555.422607	1652.079202	1499.485472	1540.666668	1514.491959	1997.84771	2187.948742	1881.008313	2102.087029	2334.290615	2010.52654
**ENSG00000124171**	PARD6B	1826.693724	2203.51536	2322.193142	1451.763154	1485.188799	1845.312848	1099.02052	1265.920229	1216.019585	1036.489631	1305.105746	1120.718412
**ENSG00000124172**	ATP5F1E	1963.098105	1832.175729	1595.89711	1912.241663	1764.889724	1811.16849	1345.176602	1653.006073	1682.637204	1570.553885	1492.731942	1722.945967
**ENSG00000124177**	CHD6	649.6785992	735.6728548	822.3755516	769.417727	840.2585654	797.0810637	1919.200331	1822.459962	1573.83792	1928.70598	2113.553914	1655.279949
**ENSG00000124181**	PLCG1	745.3022891	970.3875275	834.5890499	918.4453172	1022.873219	908.9986809	1077.571234	1898.880343	1148.020032	1161.779586	1821.077785	1148.630644
**ENSG00000124193**	SRSF6	2223.250791	2000.329524	1980.21519	2232.902152	1986.801202	1994.789259	2587.192357	2491.968954	2300.260726	2750.051243	2490.18591	2583.150212
**ENSG00000124194**	GDAP1L1	0	0	0	1.674467306	0	2.27629052	0	0	0.468962431	1.265555105	2.759208765	0
**ENSG00000124198**	ARFGEF2	2733.712547	2746.511991	2862.029766	2642.309408	2704.546139	2940.208588	2777.17174	2734.520599	2756.561172	2732.333471	2934.418521	2836.897777
**ENSG00000124201**	ZNFX1	3436.827914	4144.290415	4032.082903	1296.037695	1400.816205	1574.054894	2703.631334	2930.55549	2803.457415	1183.926801	1240.26434	1354.166172
**ENSG00000124203**	ZNF831	0	0	0	0	0	0.758763507	5.106972674	0	0.937924863	3.163887762	0	0
**ENSG00000124205**	EDN3	0	0	0	0	0	0	5.106972674	11.62918845	27.66878346	12.0227735	27.59208765	24.52893129
**ENSG00000124207**	CSE1L	4225.723356	4116.264783	4142.818621	5096.241245	4701.749442	4978.626749	4431.830887	3972.198513	4829.375119	4847.708829	4257.459124	5244.116344
**ENSG00000124209**	RAB22A	1625.602729	1993.323116	1678.134665	1828.518298	2190.220057	1827.481906	2041.767675	2556.760147	2049.834788	2048.933715	2385.335977	2216.907893

ENSG00000124212	PTGIS	0	0	0	0	0	0.379381753	155.2519693	217.6319553	85.82012496	99.97885328	216.597888	118.4155303
ENSG00000124214	STAU1	2006.691258	2087.909626	2046.982314	2052.896917	1942.881222	2039.556306	2171.484781	2236.126808	2447.51493	2290.021962	2178.39532	2399.60614
ENSG00000124215	CDH26	22.49969175	14.01281628	28.49816268	23.44254228	23.11577897	24.28043221	28.59904698	26.58100218	29.07567075	17.08499391	37.24931832	27.06640694
ENSG00000124216	SNAI1	21.09346101	17.51602035	13.02773151	21.76807497	18.49262317	18.96908767	5.106972674	1.661312636	6.096511609	0.632777552	1.379604382	3.383300867
ENSG00000124217	MOCS3	210.9346101	168.1537954	206.0010045	276.2871054	162.9662417	289.088896	270.6695517	294.0523366	329.6805893	327.7787721	281.439294	323.951058
ENSG00000124222	STX16	1299.357198	1275.166282	1319.872049	1086.729281	1026.340586	1163.184456	1525.963435	1344.001923	1498.803931	1357.30785	1298.207724	1344.862095
ENSG00000124224	PPP4R1L	140.6230734	164.6505913	112.3641843	123.9105806	167.5893975	99.39801937	152.1877857	269.1326471	150.5369405	180.3416024	269.0228546	186.9273729
ENSG00000124225	PMEPA1	49.2180757	91.08330583	213.3291035	68.65315954	272.7661918	394.9364052	4.08557814	6.645250545	6.096511609	2.53111021	6.898021912	10.99572782
ENSG00000124226	RNF114	1503.260655	1499.371342	1532.386919	891.6538403	921.1637918	1015.984335	1114.341438	1025.029896	1061.730945	851.7185855	853.9751126	949.8617184
ENSG00000124228	DDX27	1306.388352	970.3875275	1271.018056	1145.335637	1081.818456	1178.739108	1171.539532	1131.353905	1230.55742	1094.705166	1160.247286	1222.217438
ENSG00000124233	SEMG1	5.624922937	3.50320407	0	5.023401917	0	2.655672273	0	0	0	1.265555105	0	0.845825217
ENSG00000124243	BCAS4	181.4037647	77.07048955	156.3327781	247.8211612	217.2883223	176.4125153	408.557814	365.4887799	303.4186932	613.1614483	533.906896	426.2959093
ENSG00000124249	KCNK15	0	3.50320407	4.885399317	0	2.311577897	1.896908767	0	4.983937908	7.034436472	5.694997971	5.518417529	4.229126084
ENSG00000124251	TP53TG5	8.437384405	7.006408141	3.256932878	5.86063557	1.155788948	0.758763507	5.106972674	0	3.28273702	5.062220419	5.518417529	0.845825217
ENSG00000124256	ZBP1	82.96761332	185.6698157	196.2302059	0	1.155788948	1.896908767	34.72741419	21.59706427	16.88264753	0	0	0
ENSG00000124257	NEURL2	15.46853808	7.006408141	29.3123959	17.58190671	25.42735686	27.69486799	31.66323058	26.58100218	36.57906965	46.19276132	15.17564821	27.06640694
ENSG00000124260	MAGEA10	232.0280711	238.2178768	298.8235915	154.0509921	293.5703929	254.1857747	17.36370709	14.95181373	10.31717349	17.08499391	17.93485697	10.99572782
ENSG00000124275	MTRR	1328.888044	1047.458017	1312.54395	1374.737658	960.460616	1378.673292	1482.04347	1212.758224	1390.942572	1331.36397	1165.765703	1401.532384
ENSG00000124279	FASTKD3	496.3994492	388.8556518	444.5713378	473.8742475	271.6104028	394.5570234	408.557814	416.9894717	369.542396	430.9215132	405.6036884	411.0710554
ENSG00000124299	PEPD	232.0280711	259.2371012	217.4002696	262.0541333	233.4693675	282.2600245	210.4072742	199.3575163	215.253756	244.8849128	226.2551187	274.0473702
ENSG00000124313	IQSEC2	126.5607661	178.6634076	163.6608771	141.4924873	216.1325333	166.9279715	206.321696	217.6319553	158.0403394	208.8165923	201.4222398	172.5483442
ENSG00000124333	VAMP7	625.7726767	651.5959571	635.9161444	728.393278	751.2628164	770.1449592	1281.850141	1124.708655	1223.054021	1329.465638	1186.459769	1528.406167
ENSG00000124334	IL9R	0	0	0	1.674467306	0	0.379381753	21.44928523	33.22625272	18.28953483	12.0227735	27.59208765	13.53320347
ENSG00000124343	XG	18.28099954	24.52242849	18.72736405	20.09360767	18.49262317	33.76497604	52.09112128	44.85544118	47.36520558	45.55998377	26.21248326	65.12854169
ENSG00000124356	STAMBP	1008.267436	1008.922772	910.3127393	879.9325692	850.6606659	890.7883568	979.5173589	990.1423311	961.3729845	944.7368857	936.7513756	1018.373561
ENSG00000124357	NAGK	539.9926019	795.227324	666.8570067	614.5295012	703.8754695	679.8521019	751.7463777	707.719183	853.9805877	672.6425382	664.9693123	702.0349299
ENSG00000124370	MCEE	137.8106119	112.1025303	92.0083538	108.0031412	137.5388848	103.9506004	173.6370709	181.0830773	164.136851	184.1382677	184.8669872	149.7110634
ENSG00000124374	PAIP2B	54.84299863	35.0320407	35.82626166	68.65315954	98.2420606	57.6660265	364.637849	322.2946514	270.1223605	354.9882069	480.102325	336.6384363
ENSG00000124380	SNRNP27	326.2455303	255.7338971	332.2071535	302.2413487	293.5703929	284.536315	269.6481572	227.5998311	234.9501782	209.4493698	233.1531406	230.9102842
ENSG00000124383	MPHOSPH1(	690.4592905	535.9902228	696.1694026	750.9985866	737.393349	758.7635066	784.4310028	551.5557952	695.9402483	748.5758445	633.2384115	792.5382281
ENSG00000124399	NDUFB4P12	1.406230734	0	1.628466439	5.86063557	0	1.13814526	6.128367209	0	0	0	4.138813147	0
ENSG00000124406	ATP8A1	16.87476881	14.01281628	15.47043117	5.023401917	21.95999002	14.79588838	22.47067977	11.62918845	21.57227185	27.8422123	27.59208765	16.07067912
ENSG00000124422	USP22	2774.493238	2686.957522	2845.745102	3255.164442	3416.512131	3645.858649	3157.130507	2722.891411	3166.9033	3055.050023	3102.730256	3132.936603
ENSG00000124429	POF1B	2214.813406	1737.589219	1154.582705	2194.389404	1968.308579	1282.689708	110.3106098	66.45250545	81.13050064	91.7527451	117.2663725	76.97009473
ENSG00000124444	ZNF576	61.8741523	101.592918	85.49448804	92.09570182	71.65891479	80.80831345	83.75435186	74.75906863	96.13729845	78.4644165	86.91507609	81.19922081
ENSG00000124459	ZNF45	305.1520693	311.7851623	342.7921854	333.2189938	277.3893476	305.7816932	291.0974424	328.939902	241.0466898	226.5343638	270.4024589	234.293585
ENSG00000124466	LYPD3	331.8704533	371.3396315	362.3337827	434.5242658	487.7429362	632.8087645	58.21948849	139.5502614	102.7027725	75.30052873	102.0907243	120.953006
ENSG00000124467	PSG8	26.71838395	28.02563256	36.64049488	23.44254228	31.2063016	34.1443578	6.128367209	6.645250545	2.813774589	5.694997971	17.93485697	7.612426951
ENSG00000124486	USP9X	4557.593809	4554.165292	4448.156078	4306.72991	4670.54314	4351.50871	4415.488574	3947.278823	4768.878965	4277.576254	4245.042684	5008.976934
ENSG00000124493	GRM4	4.218692202	10.50961221	4.885399317	3.348934611	2.311577897	1.896908767	2.04278907	0	4.220661883	2.53111021	5.518417529	0.845825217
ENSG00000124496	TRERF1	503.4306028	507.9645902	309.4086234	658.0656512	753.5743943	548.965397	25.53486337	39.87150327	53.46171719	46.82553888	38.6289227	70.20349299
ENSG00000124507	PACSIN1	30.93707615	17.51602035	23.61276336	34.32657977	67.035759	76.63511417	0	0	0.468962431	0.632777552	5.518417529	2.53747565
ENSG00000124508	BTN2A2	614.5228308	956.3747112	582.9909851	251.1700959	243.8714681	157.8228094	228.7923758	387.0858442	192.2745969	161.9910534	142.0992514	122.6446564
ENSG00000124523	SIRT5	341.7140684	420.3844885	388.3892457	275.4498718	351.3598403	274.6723894	459.6275407	521.6521677	418.3144889	454.3342826	594.6094888	419.5293075
ENSG00000124532	MRS2	604.6792157	672.6151815	547.9789567	666.4379877	627.5933989	610.4252411	762.9817176	742.6067483	759.719139	832.7352589	735.3291358	915.1828846
ENSG00000124535	WRNIP1	880.3004396	963.3811194	948.5817007	776.1155962	1012.471119	921.5182788	928.4476322	1016.723333	911.6629667	882.091908	950.5474194	1017.527736
ENSG00000124541	RRP36	877.4879781	693.6344059	756.4226609	921.7942518	742.0165048	803.9099353	609.7725373	578.1367974	591.3616261	615.6925585	669.1081254	671.5852221
ENSG00000124549	BTN2A3P	94.21745919	56.05126513	78.16638907	37.67551438	28.89472371	36.42064832	32.68462512	106.3240087	51.11690503	32.27165517	42.76773585	27.91223215
ENSG00000124562	SNRPC	1200.921047	1152.554139	1282.417321	1175.476049	1130.361591	1349.460897	1030.587086	1113.079466	1163.02683	1025.732412	918.8165186	1233.213166
ENSG00000124570	SERPINB6	824.0512102	1289.179098	859.8302797	666.4379877	835.6354096	685.5428282	1342.112419	2463.726639	982.9452563	1157.982921	2476.389866	888.1164776
ENSG00000124571	XPO5	1611.540421	1012.425976	1520.173421	1693.72368	1411.218306	1681.799312	1456.508607	1144.644406	1464.100711	1529.423344	1205.77423	1538.556069
ENSG00000124574	ABCC10	376.8698368	371.3396315	311.0370898	330.7072929	399.9029761	342.961105	453.4991735	463.5062255	342.342575	375.2370886	543.5641266	357.7840667
ENSG00000124575	HIST1H1D	1627.008959	220.7018564	1236.006027	1918.939532	681.9154795	1580.504384	3051.92687	1556.64994	3211.454731	3134.147217	1142.312429	3210.752523
ENSG00000124587	PEX6	21.09346101	21.01922442	17.09889761	17.58190671	17.33683422	12.89897961	43.919965	41.5328159	41.26869397	37.96665314	48.28615338	18.60815477
ENSG00000124588	NQO2	381.088529	448.410121	412.8162423	463.8274437	443.8229561	463.2251208	318.6750949	380.4405937	363.4458844	320.1854415	395.9464577	328.1801841
ENSG00000124593	AL365205.1	7.031153671	0	14.65619795	4.186168264	15.02525633	10.62268909	6.128367209	14.95181373	10.78613592	5.062220419	9.657230676	15.2248539
ENSG00000124596	OARD1	357.1826065	472.9325495	482.0260659	391.8253495	398.7471872	371.4147365	476.9912478	546.5718573	429.5695872	451.8031724	506.3148083	465.2038692
ENSG00000124602	UNC5CL	16.87476881	24.52242849	8.142332194	5.023401917	10.40210053	9.863925586	19.40649616	28.24231481	22.51019671	30.37332251	24.83287888	14.37902869
ENSG00000124608	AARS2	295.3084542	273.2499175	317.5509556	246.1466939	322.4651166	300.8497304	277.8193135	191.0509532	243.8604644	260.7043516	245.5695801	221.6062068
ENSG00000124610	HIST1H1A	0	0	1.628466439	0	1.155788948	0.379381753	3.064183605	0	0.937924863	5.062220419	0	0
ENSG00000124613	ZNF391	182.8099954	189.1730198	190.5305734	159.074394	116.7346838	146.8207385	59.24088302	54.82331699	44.08246856	74.03497363	41.38813147	47.36621214
ENSG00000124614	RPS10	98.43615139	14.01281628	60.25325824	112.1893095	40.45261319	80.80831345	61.28367209	29.90362745	53.93067962	77.83163894	38.6289227	58.36193996
ENSG00000124615	MOCS1	0	0	0.814233219	0	0	0.758763507	3.064183605	4.983937908	0.937924863	3.163887762	4.138813147	3.383300867
ENSG00000124635	HIST1H2BJ	966.0805144	252.2306931	921.7120044	1184.685619	491.210303	1122.590608	1120.469805	664.5250545	1544.293287	1191.520131	517.3516434	1389.690831
ENSG00000124641	MED20	241.8716863	192.6762239	284.1673936	285.4966756	240.4041012	308.0579837	357.4880872	428.6186601	370.4803208	337.2704354	402.8444796	433.062511
ENSG00000124657	OR2B6	97.02992066	80.57369362	122.1349829	100.4680383	141.0062517	132.7836137	218.5784305	144.5341993	231.6674411	205.019927	102.0907243	188.6190233
ENSG00000124659	TBCC	209.5283794	213.6954483	215.7718032	170.7956652	183.7704428	228.3878155	189.9793835	230.9224564	232.1364036	174.0138269	212.4590749	206.3813529

**ENSG00000124688**	MAD2L1BP	237.6529941	315.2883663	236.1276336	208.4711796	173.3683422	259.876501	161.3803365	206.0027669	230.7295163	179.7088249	160.0341083	237.6768859
**ENSG00000124702**	KLHDC3	577.9608317	613.0607123	428.2866734	776.1155962	683.0712684	698.8211896	803.8374989	730.9775599	800.5188705	922.5896714	913.2981011	820.4504603
**ENSG00000124713**	GNMT	9.843615139	0	9.770798633	5.023401917	6.93473369	6.828871559	3.064183605	0	4.220661883	5.694997971	11.03683506	3.383300867
**ENSG00000124721**	DNAH8	2386.373556	2431.223625	1933.803896	2005.174599	1681.67292	1564.570351	417.7503648	533.2813562	221.8192301	206.9182596	418.0201278	219.0687311
**ENSG00000124731**	TREM1	0	0	0	0	0	0	6.128367209	8.306563181	3.28273702	22.14721433	8.277626294	10.99572782
**ENSG00000124733**	MEA1	752.3334428	816.2465484	795.5058554	780.3017645	852.9722438	842.9862558	537.2535253	742.6067483	630.2855079	534.0642542	619.4423677	591.2318265
**ENSG00000124743**	KLHL31	50.62430643	101.592918	35.82626166	35.16381342	69.3473369	31.48868552	60.26227756	83.06563181	59.08926636	63.27775524	55.18417529	43.13708606
**ENSG00000124749**	COL21A1	4.218692202	0	1.628466439	1.674467306	4.623155793	0.379381753	38.81299233	94.69482026	71.28228958	48.72387153	95.19270238	97.26989993
**ENSG00000124762**	CDKN1A	2110.752332	2700.970338	2858.772833	1414.924873	1631.973995	2194.723443	592.4088302	782.4782516	654.2025919	521.4087032	840.1790688	526.1032848
**ENSG00000124766**	SOX4	115.3109202	133.1217547	149.0046792	132.2829172	198.7956991	183.2413868	210.4072742	202.6801416	234.9501782	260.7043516	324.2070298	287.5805737
**ENSG00000124767**	GLO1	6060.854464	5303.850963	4887.842016	5466.29852	5110.898729	5101.546437	2154.121074	2224.49762	2153.944448	2243.196423	2044.573695	2391.993713
**ENSG00000124772**	CPNE5	14.06230734	31.52883663	10.58503185	4.186168264	5.778944741	13.27836137	0	0	0.468962431	1.265555105	1.379604382	0
**ENSG00000124782**	RREB1	1625.602729	1874.214178	1793.755782	1407.38977	1443.580396	1604.026053	915.1695032	1001.77152	958.5592099	906.137455	1149.21045	1005.686183
**ENSG00000124783**	SSR1	4366.34643	4606.713353	4385.46012	3770.063139	4283.353842	3864.761921	3901.727123	4915.82409	3968.360095	3939.040264	4478.195825	4455.807242
**ENSG00000124784**	RIOK1	559.6798322	283.7595297	488.5399317	667.2752213	465.7829462	590.6973899	664.9278422	689.444744	588.078889	661.2525422	589.0910712	712.1848325
**ENSG00000124785**	NRN1	5.624922937	0	0	0	1.155788948	0	0	4.983937908	0.468962431	0	4.138813147	0
**ENSG00000124786**	SLC35B3	466.8686037	476.4357536	486.9114652	546.7135753	429.9534888	511.0272217	521.9326073	508.3616667	498.0381022	469.5209439	398.7056665	459.2830927
**ENSG00000124787**	RPP40	153.27915	115.6057343	120.5065165	242.7977593	193.0167544	188.5527314	116.438977	106.3240087	155.6955272	179.7088249	118.6459769	181.8524216
**ENSG00000124788**	ATXN1	1409.043196	1856.698157	1209.136331	1921.451233	2127.807454	1613.131215	1648.530779	2925.571552	1310.749996	1727.482718	3208.959793	1503.03141
**ENSG00000124789**	NUP153	10825.16419	8912.151155	10238.1685	10612.77378	9086.812711	10504.70137	10711.36449	9225.269068	10733.61213	10678.1212	9356.476921	11317.98723
**ENSG00000124795**	DEK	8985.814391	8712.468523	7774.298779	8306.19507	7716.047019	7177.523391	10469.29398	10853.35545	10128.65059	9866.267597	9981.437706	10906.91617
**ENSG00000124802**	EEF1E1	219.3719945	185.6698157	177.5028418	272.9381708	173.3683422	225.7321432	176.7012545	122.9371351	165.5437383	191.7315984	117.2663725	181.0065964
**ENSG00000124813**	RUNX2	94.21745919	119.1089384	101.7791524	159.9116277	138.6946738	145.3032115	25.53486337	23.25837691	52.99275475	31.00610007	46.906549	50.74951301
**ENSG00000124818**	OPN5	8.437384405	0	8.142332194	1.674467306	0	3.035054026	0	0	0.937924863	0	0	5.920776517
**ENSG00000124831**	LRRFIP1	3415.734453	2921.672195	3375.810928	3207.442124	3106.760693	3166.699495	1902.858018	2357.402631	1915.711532	2088.165923	2237.718308	2034.209646
**ENSG00000124835**	AC105760.1	8.437384405	0	9.770798633	22.60530863	17.33683422	7.587635066	14.29952349	23.25837691	7.972361335	28.47498986	20.69406573	9.304077385
**ENSG00000124839**	RAB17	227.8093789	266.2435094	299.6378248	183.35417	257.7409355	257.2208287	137.8882622	210.9867048	125.2129692	101.2444084	135.2012295	117.5697051
**ENSG00000124882**	EREG	8265.824255	5062.129882	8792.904537	6628.37883	3626.86572	8085.763308	780.3454246	584.7820479	1031.248387	791.604718	509.0740171	1260.279573
**ENSG00000124920**	MYRF	30.93707615	49.04485699	43.96859385	39.34998168	41.60840214	28.07424974	2.04278907	1.661312636	0.468962431	2.53111021	8.277626294	9.304077385
**ENSG00000124942**	AHNAK	45500.00163	59585.99803	40513.8023	53447.32193	70944.63722	45728.77963	59154.06449	99045.79805	58960.77066	62755.08098	108366.5446	69130.98662
**ENSG00000125037**	EMC3	489.3682955	441.4037129	507.2672957	517.4103975	471.5618909	509.8890764	543.3818926	651.2345534	551.9687818	513.8153725	598.7483019	582.7735744
**ENSG00000125046**	SSUH2	82.96761332	136.6249587	81.42332194	72.00209415	61.25681426	55.76911774	7.149761744	23.25837691	15.47576024	10.75721839	16.55525259	17.76232955
**ENSG00000125089**	SH3TC1	409.2131436	301.2755501	358.2626166	367.5455736	354.8272071	355.4807028	164.4445201	93.03350762	266.3706611	176.5449371	153.1360864	222.452032
**ENSG00000125107**	CNOT1	4824.777649	4932.511331	5557.955956	5191.685881	5398.690177	5373.183772	4534.991735	4907.517527	4835.940593	4507.907283	4708.589757	4591.985102
**ENSG00000125122**	LRRC29	4.218692202	21.01922442	5.699632536	2.511700959	12.71367843	3.035054026	7.149761744	13.29050109	7.503398903	6.327775524	11.03683506	2.53747565
**ENSG00000125124**	BBS2	454.2125271	742.6792629	430.7293731	401.0349197	514.326082	361.1714291	570.959545	589.7659858	453.0177088	599.8731197	547.7029398	462.6663936
**ENSG00000125144**	MT1G	4.218692202	10.50961221	3.256932878	15.07020575	13.86946738	7.587635066	4.08557814	0	4.689624315	3.163887762	1.379604382	2.53747565
**ENSG00000125148**	MT2A	2074.190333	1425.804057	2336.035107	1373.063191	1503.681422	2044.86765	1227.716231	1177.870659	1822.388009	1015.607972	933.9921668	1025.140163
**ENSG00000125149**	C16orf70	306.5583	252.2306931	298.0093583	257.8679651	249.6504128	264.0497003	272.7123408	224.2772059	212.4399814	242.3538026	263.504437	252.9017398
**ENSG00000125166**	GOT2	2119.189716	2108.92885	2533.079546	2476.537145	2334.693675	2774.03938	1242.015754	1131.353905	1434.087115	1369.330623	1336.836646	1558.010049
**ENSG00000125170**	DOK4	105.4673051	133.1217547	113.1784175	103.816973	154.8757191	133.5423772	94.98969174	84.72694444	101.2958852	73.40219608	146.2380645	129.4112582
**ENSG00000125207**	PIWIL1	2.812461468	0	1.628466439	0.837233653	2.311577897	1.896908767	413.6647866	599.7338616	571.1962415	430.9215132	529.7680828	555.7071674
**ENSG00000125245**	GPR18	0	0	0	2.511700959	0	0	1.021394535	1.661312636	0	5.062220419	1.379604382	0
**ENSG00000125246**	CLYBL	33.74953762	14.01281628	11.39926507	25.95424324	11.55788948	10.24330734	22.47067977	36.54887799	20.63434698	27.20943475	26.21248326	27.06640694
**ENSG00000125247**	TMTC4	416.2442973	259.2371012	340.3494857	467.1763783	406.8377098	366.1033919	551.5530488	435.2639107	629.347583	654.2919892	580.813445	763.7801707
**ENSG00000125249**	RAP2A	727.0212896	1397.778424	830.5178838	951.0974297	1353.428858	1118.79679	892.6988235	1119.724717	884.9321082	893.481904	1045.740122	1006.532008
**ENSG00000125255**	SLC10A2	21.09346101	0	6.513865756	0.837233653	1.155788948	1.13814526	0	0	0	0	0	0
**ENSG00000125257**	ABCC4	1.406230734	10.50961221	17.91313083	1.674467306	5.778944741	16.69279715	28.59904698	11.62918845	25.3239713	39.8649858	38.6289227	35.5246591
**ENSG00000125266**	EFNB2	490.7745262	788.2209158	583.8052183	745.1379511	696.9407358	830.466658	103.160848	217.6319553	224.6330047	129.0866207	289.7169203	366.2423189
**ENSG00000125304**	TM9SF2	5293.052483	5804.809145	5176.080576	4754.649915	5520.048017	5016.185542	5494.081203	5530.509766	5373.840502	5480.486381	5690.868077	6083.174959
**ENSG00000125319**	C17orf53	215.1533023	182.1666117	219.028736	192.5637402	247.3388349	252.2888659	158.3161529	141.2115741	168.8264753	143.6405044	172.4505478	141.2528112
**ENSG00000125347**	IRF1	12649.04545	12979.37108	13252.45988	814.6283442	984.7321839	914.6894072	5722.873579	5766.41616	5598.942469	814.3847099	700.8390262	773.0842481
**ENSG00000125351**	UPF3B	412.0256051	346.817203	372.9188145	362.5221717	390.6566645	341.0641962	434.0926773	518.3295425	397.6801419	451.1703948	485.6207426	460.1289179
**ENSG00000125352**	RNF113A	73.12399818	150.637775	135.1627144	92.09570182	88.99574902	125.1959786	104.1822426	129.5823856	114.4268333	99.97885328	113.1275593	123.4904816
**ENSG00000125354**	6-Sep	160.3103037	175.1602035	117.2495836	81.21166433	109.7999501	40.21446585	28.59904698	39.87150327	22.97915914	17.71777147	22.07367012	16.91650434
**ENSG00000125355**	TMEM255A	21.09346101	49.04485699	4.885399317	20.93084132	43.91998003	7.967016819	2.04278907	6.645250545	2.344812157	0	1.379604382	1.691650434
**ENSG00000125356**	NDUFA1	464.0561423	665.6087734	434.8005392	475.5487148	477.3408356	437.0477798	424.9001265	531.6200436	508.8242381	508.7531521	550.4621485	565.0112448
**ENSG00000125375**	ATP5S	275.6212239	154.1409791	201.1156052	205.122245	181.4588649	176.791897	350.3383255	282.4231481	366.259659	365.7454253	340.7622824	353.5549406
**ENSG00000125378**	BMP4	5.624922937	28.02563256	39.08319453	6.697869223	20.80420107	44.00828338	0	13.29050109	1.875849726	1.898332657	0	0
**ENSG00000125384**	PTGER2	1.406230734	0	0	4.186168264	4.623155793	4.931962793	0	0	0	0	2.759208765	0
**ENSG00000125386**	FAM193A	379.6822982	458.9197332	543.9077906	416.9423591	480.8082025	441.2209791	529.0823691	667.8476797	530.8654724	535.3298093	555.9805661	454.2081414
**ENSG00000125388**	GRK4	47.81184496	28.02563256	17.91313083	30.1404115	33.5178795	41.73199286	26.55625791	56.48462963	30.01359561	37.33387559	49.66575776	45.67456171
**ENSG00000125398**	SOX9	1909.661337	2147.464095	2331.149707	1526.276949	1634.285573	1984.925333	26.55625791	61.46856754	45.02039342	15.81943881	53.80457091	40.59961041
**ENSG00000125409**	TEKT3	0	0	3.256932878	1.674467306	1.155788948	0.758763507	2.04278907	0	0	0	6.898021912	0
**ENSG00000125414**	MYH2	2.812461468	0	0.814233219	4.186168264	4.623155793	0.758763507	0	0	0	0	0	0
**ENSG00000125430**	HS3ST3B1	8.437384405	0	1.628466439	0.837233653	0	0.379381753	0	0	1.406887294	3.796665314	0	0
**ENSG00000125434**	SLC25A35	97.02992066	129.6185506	109.9214846	103.816973	121.3578396	112.296999	181.8082272	122.9371351	181.0194985	171.4827167	157.2748996	142.0986364

**ENSG00000125445**	MRPS7	705.9278285	623.5703245	613.1176142	750.9985866	646.0860221	763.6954694	455.5419626	392.0697821	485.845079	474.5831643	506.3148083	581.0819239
**ENSG00000125447**	GGA3	596.2418313	553.5062431	581.3625187	462.1529764	501.6124035	491.6787523	592.4088302	553.2171078	477.4037552	497.3631562	609.785137	423.7584336
**ENSG00000125449**	ARMC7	73.12399818	42.03844885	100.150686	94.60740277	75.12628164	110.779472	91.92550814	106.3240087	129.4336311	107.5721839	115.8867681	131.9487338
**ENSG00000125450**	NUP85	762.1770579	641.0863449	718.9679328	808.7677087	663.4228563	665.4355953	657.7780805	711.0418083	730.1745058	701.7503056	714.63507	687.6559012
**ENSG00000125454**	SLC25A19	174.372611	143.6313669	171.8032093	286.3339093	182.6146538	279.2249704	172.6156764	234.2450817	181.488461	221.4721433	233.1531406	195.3856251
**ENSG00000125457**	MIF4GD	164.5289959	56.05126513	122.9492161	166.6094969	143.3178296	138.8537217	150.1449966	171.1152015	153.3507151	152.4993901	137.9604382	184.3898973
**ENSG00000125458**	NT5C	153.27915	129.6185506	129.4630819	133.1201508	125.9809954	113.0557625	108.2678207	69.77513072	102.2338101	92.38552265	100.7111199	92.19494863
**ENSG00000125459**	MSTO1	191.2473798	119.1089384	238.5703333	208.4711796	107.4883722	202.969238	177.7226491	119.6145098	134.5922178	205.019927	103.4703287	130.2570834
**ENSG00000125462**	C1orf61	25.31215321	42.03844885	44.78282707	25.95424324	30.05051265	22.38352344	11.23533988	13.29050109	8.441323766	4.429442867	9.657230676	3.383300867
**ENSG00000125482**	TTF1	710.1465207	847.775385	688.0270704	746.8124184	640.3070773	660.5036325	658.799475	692.7673693	619.0304095	664.41643	725.6719051	724.0263856
**ENSG00000125484**	GTF3C4	1774.663187	1292.682302	1586.940545	2068.804356	1875.845463	1960.265519	1906.943597	1692.877576	1873.973876	1932.502645	1807.281741	2041.822073
**ENSG00000125485**	DDX31	722.8025974	437.9005088	705.9402013	720.0209415	521.2608157	676.4376661	571.9809395	473.4741013	579.1686028	632.1447748	509.0740171	535.4073622
**ENSG00000125503**	PPP1R12C	305.1520693	241.7210809	313.4797895	318.9860217	284.3240813	355.4807028	293.1402315	413.6668464	305.7635053	318.9198864	380.7708095	275.7390207
**ENSG00000125505**	MBOAT7	527.3365253	693.6344059	601.7183492	609.5060993	807.8964748	793.6666279	629.1790335	686.1221187	693.5954361	666.3147627	862.2527389	703.7265804
**ENSG00000125508**	SRMS	36.56199909	35.0320407	17.91313083	44.3733836	40.45261319	52.73406371	0	4.983937908	0.937924863	0	0	0
**ENSG00000125510**	OPRL1	0	0	4.071166097	2.511700959	1.155788948	6.449489806	0	0	0	0	0	0
**ENSG00000125520**	SLC2A4RG	267.1838395	311.7851623	224.7283686	239.4488247	343.2693176	267.0847543	325.8248566	289.0683987	289.8187826	318.9198864	325.5866342	334.9467858
**ENSG00000125531**	FNDC11	32.34330689	31.52883663	35.82626166	33.48934611	48.54313583	44.38766514	0	3.322625272	0	0	0	4.229126084
**ENSG00000125534**	PPDPF	717.1776744	553.5062431	652.2008088	915.9336162	834.4796206	992.0832849	377.9159779	309.0041503	506.0104635	411.9381866	474.5839075	456.7456171
**ENSG00000125538**	IL1B	0	3.50320407	3.256932878	1.674467306	1.155788948	5.6907263	14.29952349	0	25.79293373	31.00610007	17.93485697	25.3747565
**ENSG00000125551**	PLGLB2	8.437384405	17.51602035	17.09889761	7.535102876	6.93473369	9.863925586	8.171156279	11.62918845	5.158586746	10.12444084	5.518417529	16.07067912
**ENSG00000125571**	IL37	29.53084542	59.5544692	30.12662912	20.09360767	60.10102531	19.34846942	21.44928523	6.645250545	27.66878346	24.67832454	22.07367012	20.2998052
**ENSG00000125611**	CHCHD5	88.59253625	63.05767327	81.42332194	109.6776085	80.90522638	105.0887457	80.69016826	99.67875817	116.302683	110.1032941	114.5071637	101.499026
**ENSG00000125618**	PAX8	25.31215321	24.52242849	14.65619795	23.44254228	26.58314581	42.11137462	4.08557814	13.29050109	2.344812157	9.491663286	16.55525259	0.845825217
**ENSG00000125629**	INSIG2	514.6804487	1131.534915	571.5917201	493.9678552	796.3385854	539.1014714	222.6640086	725.993622	181.9574234	197.4265963	641.5160378	199.6147512
**ENSG00000125630**	POLR1B	821.2387487	325.7979785	762.1222934	852.3038586	535.130283	865.749161	632.2432171	310.665463	631.6923952	649.2297687	373.8727876	606.4566804
**ENSG00000125633**	CCDC93	1172.796432	1502.874546	1379.311074	906.7240461	1091.064767	1232.990698	870.2281437	1076.530588	818.3394429	754.90362	885.7060134	788.309102
**ENSG00000125637**	PSD4	1012.486129	840.7689769	959.9809657	1242.454741	1346.494125	1344.528934	1307.385005	1131.353905	1372.653037	1264.922327	1287.170889	1348.245396
**ENSG00000125648**	SLC25A23	555.46114	770.7048955	613.1176142	695.7411655	790.5596406	678.7139567	695.5696783	696.0899945	642.9474935	624.5514442	798.7909374	606.4566804
**ENSG00000125650**	PSPN	30.93707615	21.01922442	35.82626166	31.81487881	38.14103529	31.48868552	51.06972674	36.54887799	61.90304095	53.1533144	48.28615338	48.21203736
**ENSG00000125651**	GTF2F1	1072.95405	1015.92918	1122.82761	1106.822889	1144.231059	1104.000902	861.0355929	998.4488943	920.5732529	878.2952427	961.5842545	882.1957011
**ENSG00000125652**	ALKBH7	94.21745919	66.56087734	48.03975995	61.95529031	70.50312584	50.45777319	59.24088302	46.51675381	72.68917688	63.91053279	66.22101035	66.82019213
**ENSG00000125656**	CLPP	219.3719945	199.682632	191.3448066	252.0073295	228.8462118	229.5259607	103.160848	131.2436983	173.9850621	175.9121596	151.7564821	169.1650434
**ENSG00000125657**	TNFSF9	132.185689	150.637775	122.9492161	85.39783259	187.2378096	120.6433976	32.68462512	69.77513072	27.19982102	22.14721433	40.00852709	24.52893129
**ENSG00000125676**	THOC2	1909.661337	1926.762239	1840.167076	1830.192765	1722.125533	1757.675663	2205.190801	2143.093301	1938.690692	2117.906468	2011.463189	2036.747122
**ENSG00000125686**	MED1	1925.129875	2031.858361	2071.40931	2067.967123	2009.916981	2155.267741	1872.216182	1857.347527	1843.96028	1755.32493	1847.290268	1868.427904
**ENSG00000125691**	RPL23	7482.553736	7132.523487	7583.768206	8451.873726	8233.840467	8552.402865	4958.870467	4939.082467	5484.046673	5720.941851	5366.661047	6413.892619
**ENSG00000125703**	ATG4C	212.3408409	161.1473872	225.5426018	214.3318151	234.6251565	205.6249103	324.8034621	353.8595915	290.7567075	277.7893455	271.7820633	295.1930007
**ENSG00000125726**	CD70	16.87476881	38.53524477	26.05546302	9.209570182	38.14103529	16.69279715	17.36370709	33.22625272	21.57227185	12.65555105	20.69406573	2.53747565
**ENSG00000125730**	C3	243.277917	1345.230363	701.0548019	75.35102876	288.9472371	203.3486198	771.1528738	873.8504466	495.2243276	232.2293617	302.1333597	224.9895077
**ENSG00000125731**	SH2D3A	291.089762	178.6634076	254.0407645	366.70834	360.6061519	415.0436381	386.0871342	390.4084695	381.7354192	373.9715335	315.9294035	344.2508632
**ENSG00000125733**	TRIP10	1044.829435	1236.631037	1203.436698	1226.547301	1297.950989	1406.368159	1224.652047	1726.103829	1569.148296	1216.831233	1380.983987	1419.294714
**ENSG00000125734**	GPR108	222.184456	287.2627338	259.740397	241.123292	290.103026	296.6765311	277.8193135	320.6333388	287.005008	254.3765761	307.6517773	256.2850407
**ENSG00000125735**	TNFSF14	2.812461468	28.02563256	6.513865756	0.837233653	1.155788948	1.13814526	3.064183605	0	0.468962431	0.632777552	1.379604382	0.845825217
**ENSG00000125740**	FOSB	23.90592248	45.54165292	45.59706029	26.79147689	24.27156791	28.83301325	48.00554314	111.3079466	52.99275475	63.91053279	106.2295374	56.67028952
**ENSG00000125741**	OPA3	385.3072212	378.3460396	389.2034789	496.4795562	493.5218809	470.8127559	501.5047166	543.249232	485.3761166	544.8214726	549.0825442	548.9405657
**ENSG00000125743**	SNRPD2	922.4873616	879.3042217	740.1379965	970.3538037	991.6669176	938.2110759	690.4627056	800.7526906	834.2841656	839.695812	921.5757274	1040.365017
**ENSG00000125744**	RTN2	19.68723028	28.02563256	11.39926507	22.60530863	26.58314581	17.0721789	14.29952349	14.95181373	22.97915914	24.04554699	12.41643944	25.3747565
**ENSG00000125746**	EML2	465.462373	504.4613861	378.618447	447.9200043	396.4356093	396.4539322	518.8684237	420.3120969	632.1613576	520.7759256	423.5385454	515.107557
**ENSG00000125753**	VASP	1189.671201	1114.018894	1111.428345	1288.502592	1184.683672	1332.009336	904.9555579	907.0766993	1030.310462	905.5046775	880.1875959	1004.840358
**ENSG00000125755**	SYMPK	835.3010561	550.0030391	771.893092	726.7188107	703.8754695	727.2748211	712.9333853	603.0564869	759.719139	728.3269628	735.3291358	667.356096
**ENSG00000125772**	GPCPD1	314.9956845	329.3011826	392.4604118	454.6178735	359.4503629	388.4869154	818.1370224	372.1340305	767.2225379	840.9613671	524.2496653	858.512595
**ENSG00000125775**	SDCBP2	395.1508363	679.6215897	612.303381	215.1690488	332.8672171	446.1529419	22.47067977	44.85544118	28.60670832	18.98332657	6.898021912	16.91650434
**ENSG00000125779**	PANK2	1666.38342	1516.887362	1506.331456	1306.921732	1349.961492	1307.728904	1621.974521	1503.487936	1346.391141	1511.072795	1433.408953	1334.712192
**ENSG00000125787**	GNRH2	8.437384405	21.01922442	2.442699658	1.674467306	6.93473369	2.27629052	11.23533988	8.306563181	3.28273702	9.491663286	9.657230676	5.920776517
**ENSG00000125804**	FAM182A	8.437384405	0	0.814233219	4.186168264	16.18104528	4.931962793	7.149761744	4.983937908	10.78613592	1.898332657	9.657230676	4.229126084
**ENSG00000125810**	CD93	2.812461468	0	0	2.511700959	6.93473369	5.311344546	0	0	0.937924863	1.265555105	0	0.845825217
**ENSG00000125812**	GZF1	406.4006822	441.4037129	541.4650909	529.1316686	500.4566146	562.6231402	600.5799865	730.9775599	589.4857763	551.7820257	764.3008278	614.0691074
**ENSG00000125814**	NAPB	570.9296781	609.5575083	584.6194516	462.99021	396.4356093	396.833314	829.3723623	965.2226416	751.2778152	723.2647424	834.6606513	595.4609526
**ENSG00000125815**	CST8	0	0	0	0	2.311577897	0	14.29952349	74.75906863	4.689624315	22.14721433	80.01705417	2.53747565
**ENSG00000125817**	CENPB	1532.7915	1271.663078	1405.366537	1902.194859	1651.622407	1964.438719	1554.562482	1310.77567	2023.572892	1867.326557	1488.593129	1903.952563
**ENSG00000125818**	PSMF1	2169.814023	1793.640484	2367.790202	2120.712843	2194.843213	2174.61621	2183.741516	1998.559101	2366.853392	2124.234243	2110.794705	2238.053524
**ENSG00000125820**	NKX2-2	8.437384405	0	1.628466439	0	2.311577897	0.758763507	0	0	1.875849726	1.898332657	0	2.53747565
**ENSG00000125821**	DTD1	239.0592248	266.2435094	256.4834641	241.9605257	257.7409355	231.0434878	204.278907	177.7604521	265.9016986	249.9471332	175.2097566	212.3021294
**ENSG00000125823**	CSTL1	2.812461468	0	0	0	0	0	0	4.983937908	0	1.898332657	1.379604382	0.845825217
**ENSG00000125826**	RBCK1	1576.384653	1506.37775	1644.751103	690.7177636	656.4881226	694.6479903	1619.931732	1550.00469	1785.808939	1164.310696	1157.488077	1023.448512
**ENSG00000125827**	TMX4	369.8386831	388.8556518	372.1045813	523.271033	542.0650167	487.505553	1201.159973	782.4782516	914.9457038	1306.052868	1174.043329	1163.855498

**ENSG00000125834**	STK35	1582.009576	1646.505913	1681.391598	1689.537511	1843.483372	1844.933466	1522.899251	1604.828006	1737.505809	1580.678326	1655.525259	1619.75529
**ENSG00000125835**	SNRPB	1615.759114	1362.746383	1595.082877	1778.284279	1618.104528	1886.286077	1466.722552	1368.921612	1819.105272	1602.82554	1437.547766	1673.042279
**ENSG00000125841**	NRSN2	156.0916115	140.1281628	107.478785	130.6084498	183.7704428	132.7836137	128.6957114	132.9050109	105.0475846	132.2505084	151.7564821	117.5697051
**ENSG00000125843**	AP5S1	195.466072	213.6954483	165.2893435	240.2860584	220.7556891	224.2146162	243.0918993	239.2290196	263.087924	287.9137863	212.4590749	270.6640694
**ENSG00000125844**	RRBP1	6153.665693	5846.847594	6456.86943	5884.078112	5242.658669	6059.485364	5477.738891	8788.343845	5961.919391	5301.410334	9081.935649	6053.571076
**ENSG00000125845**	BMP2	52.03053716	66.56087734	108.2930182	49.39678552	48.54313583	72.08253313	0	0	5.158586746	2.53111021	8.277626294	1.691650434
**ENSG00000125846**	ZNF133	351.5576835	367.8364274	412.8162423	298.8924141	312.063016	321.7157268	698.6338619	573.1528595	537.8999089	476.4814969	507.6944127	533.7157118
**ENSG00000125848**	FLRT3	390.9321441	357.3268152	390.0177121	439.5476678	373.3198303	468.9158471	1.021394535	3.322625272	2.344812157	1.898332657	2.759208765	2.53747565
**ENSG00000125850**	OVOL2	50.62430643	21.01922442	43.15436063	49.39678552	49.69892478	58.04540826	126.6529223	79.74300653	158.9782643	174.0138269	133.8216251	198.7689259
**ENSG00000125863**	MKKS	347.3389913	245.2242849	429.9151399	384.2902467	351.3598403	439.3240703	412.6433921	333.9238399	397.2111794	387.2598621	355.9379306	430.5250353
**ENSG00000125864**	BFSP1	129.3732275	136.6249587	87.9371877	149.8648239	180.3030759	89.15471203	70.47622291	103.0013834	60.02719123	51.25498174	57.94338406	46.52038692
**ENSG00000125868**	DSTN	5357.739097	5062.129882	4613.445421	6214.785405	6288.647668	6037.860604	5641.162016	6425.957277	5851.71322	6261.966658	6761.441078	7148.068907
**ENSG00000125870**	SNRPB2	1413.261888	1320.707935	1501.446057	1325.340872	1146.542637	1265.996911	1338.026841	1327.388796	1323.411982	1291.498984	1212.672252	1329.637241
**ENSG00000125871**	MGME1	1316.231967	1187.58618	1353.255611	1509.532276	1346.494125	1369.188748	2205.190801	2585.002462	1853.339529	2081.838147	2117.692727	1919.177417
**ENSG00000125872**	LRRN4	1.406230734	7.006408141	23.61276336	3.348934611	2.311577897	11.3814526	11.23533988	8.306563181	5.158586746	5.062220419	0	7.612426951
**ENSG00000125875**	TBC1D20	708.74029	753.1888751	778.4069578	776.1155962	829.8564649	808.4625163	696.5910728	895.4475109	786.4499975	795.4013833	933.9921668	791.6924029
**ENSG00000125877**	ITPA	750.927212	535.9902228	601.7183492	663.0890531	585.9849968	762.1779424	544.4032871	385.4245316	580.1065277	632.7775524	449.7510286	521.0283335
**ENSG00000125885**	MCM8	485.1496033	697.13761	484.4687656	604.4826974	669.201801	589.1798629	788.5165809	735.9614978	760.1881014	799.1980487	739.4679489	867.8166724
**ENSG00000125895**	TMEM74B	0	24.52242849	0	0	2.311577897	1.13814526	0	0	1.875849726	2.53111021	2.759208765	0
**ENSG00000125898**	FAM110A	194.0598413	231.2114686	216.5860364	200.098843	224.223056	233.3197783	222.6640086	242.5516449	247.6121638	218.3082556	154.5156908	204.6897025
**ENSG00000125899**	C20orf187	5.624922937	0	3.256932878	0	3.467366845	0	0	0	2.344812157	0.632777552	0	0
**ENSG00000125900**	SIRPD	0	3.50320407	3.256932878	6.697869223	8.090522638	1.13814526	1.021394535	0	3.28273702	2.53111021	1.379604382	0
**ENSG00000125901**	MRPS26	241.8716863	234.7146727	284.9816268	308.9392179	223.067267	374.4497905	311.5253331	297.3749619	351.7218236	346.1293212	235.9123494	351.8632902
**ENSG00000125910**	S1PR4	0	0	2.442699658	3.348934611	2.311577897	4.931962793	5.106972674	0	0.468962431	1.265555105	9.657230676	0
**ENSG00000125912**	NCLN	817.0200565	641.0863449	761.3080602	816.3028115	902.6711686	1085.031814	472.9056696	433.602598	631.2234327	566.3359094	528.3884784	619.1440587
**ENSG00000125944**	HNRNPR	6132.572232	4718.815883	5496.074231	7068.763731	5960.403606	5886.487284	7791.197512	7130.353834	7252.504002	7595.228961	7179.461206	8336.453337
**ENSG00000125945**	ZNF436	539.9926019	525.4806106	425.8439738	496.4795562	413.7724435	388.8662971	545.4246816	618.0083006	506.9483884	578.3586829	535.2865003	555.7071674
**ENSG00000125952**	MAX	802.9577492	749.6856711	844.3598486	400.1976861	394.1240314	454.8787222	699.6552564	621.3309259	705.3194969	518.2448154	416.6405235	479.5828979
**ENSG00000125962**	ARMCX5	125.1545353	136.6249587	135.1627144	123.073347	108.6441611	130.5073231	166.4873092	99.67875817	151.4748654	153.1321677	180.7281741	137.0236851
**ENSG00000125965**	GDF5	1.406230734	0	3.256932878	3.348934611	3.467366845	0.758763507	1.021394535	1.661312636	1.875849726	6.960553076	2.759208765	0.845825217
**ENSG00000125966**	MMP24	0	0	0	1.674467306	3.467366845	1.13814526	5.106972674	0	0.468962431	2.53111021	0	0
**ENSG00000125967**	NECAB3	140.6230734	122.6121425	110.7357178	142.329721	160.6546638	142.6475392	161.3803365	162.8086383	177.2677991	177.1777147	215.2182836	168.3192181
**ENSG00000125968**	ID1	582.1795239	486.9453658	564.2636211	324.8466573	251.9619907	432.8745805	2370.656715	1898.880343	2972.752853	2305.841401	1811.420554	2557.775456
**ENSG00000125970**	RALY	1245.92043	1453.829689	1369.540275	1429.157845	1377.700426	1494.764108	1666.915881	1777.604521	1814.415647	1753.426598	1785.208071	1698.417035
**ENSG00000125971**	DYNLRB1	165.9352266	238.2178768	171.8032093	175.8190671	219.5999002	176.791897	189.9793835	191.0509532	242.4535771	189.2004882	252.467602	192.8481494
**ENSG00000125977**	EIF2S2	5838.670008	5387.92786	5515.615829	4619.018063	4048.728686	4746.065734	4755.612954	4482.221492	4699.472526	3804.891422	3317.948539	4079.415021
**ENSG00000125991**	ERGIC3	1445.605195	1163.063751	1583.683612	1445.902519	1453.982497	1699.630255	1820.125061	1952.042347	1897.89096	1874.919888	1978.352684	1789.766159
**ENSG00000125995**	ROMO1	240.4654555	248.727489	254.8549977	274.6126381	228.8462118	334.9940882	193.0435671	269.1326471	327.8047396	298.6710047	260.7452283	325.6427085
**ENSG00000126001**	CEP250	1047.641897	1366.249587	1135.041108	1156.219675	1635.441362	1166.978273	1435.059322	2388.967571	1359.522089	1688.25051	2654.358832	1450.590247
**ENSG00000126003**	PLAGL2	615.9290616	648.092753	793.877389	648.0188473	510.8587151	705.2706794	728.2543034	880.4956971	860.0770993	701.7503056	753.2639927	702.8807551
**ENSG00000126005**	MMP24OS	154.6853808	196.1794279	208.4437042	267.0775353	262.3640913	252.6682477	233.8993485	375.4566558	245.2673516	277.1565679	331.1050518	229.2186337
**ENSG00000126010**	GRPR	5.624922937	0	4.885399317	17.58190671	12.71367843	23.5216687	7.149761744	6.645250545	7.034436472	9.491663286	2.759208765	7.612426951
**ENSG00000126012**	KDM5C	1535.603962	1555.422607	1772.585719	1567.301398	1683.984498	1948.504685	1579.075951	1742.716955	1768.457329	1590.169989	1996.287541	1566.468301
**ENSG00000126016**	AMOT	298.1209156	231.2114686	127.8346155	456.2923408	334.023006	174.5156065	6732.011379	4065.232021	4555.501059	7370.59293	5039.694809	5455.572648
**ENSG00000126062**	TMEM115	275.6212239	213.6954483	309.4086234	265.403068	225.3788449	337.6497604	308.4611495	239.2290196	358.2872976	287.9137863	284.1985028	320.5677572
**ENSG00000126067**	PSMB2	5233.990793	5006.078617	5321.828322	4323.474583	4060.286575	4379.203578	3631.057571	3299.366895	3639.148468	3048.722247	2828.188984	3331.705529
**ENSG00000126070**	AGO3	1947.629567	2221.031381	1802.712348	1796.703419	1836.548639	1824.826233	1812.975299	1873.960654	1721.092123	1736.341604	1968.695454	1788.920333
**ENSG00000126088**	UROD	382.4947597	399.365264	442.9428714	485.5955187	500.4566146	457.5343945	341.1457746	373.7953431	454.8935585	441.045954	463.5470725	409.3794049
**ENSG00000126091**	ST3GAL3	313.5894537	168.1537954	294.7524254	303.915816	146.7851964	198.416657	208.3644851	184.4057026	164.6058134	213.2460352	230.3939318	175.9316451
**ENSG00000126106**	TMEM53	56.24922937	49.04485699	45.59706029	42.6989163	57.78944741	47.80210092	84.77574639	64.79119281	82.53738794	72.76941852	91.05388923	76.97009473
**ENSG00000126107**	HECTD3	576.554601	595.544692	549.6074231	462.1529764	515.4818709	449.5673777	368.7234271	475.1354139	434.728174	441.6787316	546.3233354	479.5828979
**ENSG00000126214**	KLC1	458.4312193	504.4613861	440.5001717	401.8721534	398.7471872	464.7426478	488.2265877	561.523671	449.7349718	441.6787316	481.4819294	384.8504736
**ENSG00000126215**	XRCC3	333.276684	245.2242849	241.8272662	221.0296844	245.027257	219.2826534	304.3755714	305.681525	355.0045606	345.4965436	275.9208765	306.1887285
**ENSG00000126216**	TUBGCP3	1518.729193	1324.211139	1731.059825	1827.681064	1585.742437	1886.665459	1382.9682	1342.34061	1399.852858	1368.697846	1363.04913	1407.453161
**ENSG00000126217**	MCF2L	223.5906867	161.1473872	221.4714357	216.0062824	269.2988249	261.394028	9.192550814	1.661312636	6.56547404	5.062220419	9.657230676	4.229126084
**ENSG00000126226**	PCID2	1434.355349	1373.255996	1626.837972	1365.528088	1029.807953	1351.737187	951.9397065	868.8665087	900.4078684	908.0357877	957.4454413	979.465601
**ENSG00000126243**	LRFN3	85.78007478	108.5993262	125.3919158	100.4680383	137.5388848	114.9526713	76.60459012	69.77513072	67.53059013	69.60553076	88.29468047	93.04077385
**ENSG00000126246**	IGFLR1	29.53084542	28.02563256	21.98429693	8.372336529	8.090522638	11.76083435	20.4278907	31.56494009	11.72406079	6.327775524	2.759208765	9.304077385
**ENSG00000126247**	CAPNS1	2324.499404	2588.867808	2324.635842	2376.069107	2486.102028	2408.694752	1894.686862	2013.510915	1920.401157	1940.095976	2101.137474	1774.541305
**ENSG00000126249**	PDCD2L	49.2180757	10.50961221	87.9371877	57.76912205	43.91998003	61.45984404	52.09112128	24.91968954	39.39284424	39.8649858	22.07367012	36.37048432
**ENSG00000126254**	RBM42	369.8386831	325.7979785	438.0574721	395.1742842	380.254564	504.9571136	317.6537003	289.0683987	369.0734336	322.7165517	418.0201278	347.6341641
**ENSG00000126259**	KIRREL2	0	0	3.256932878	0	4.623155793	0.379381753	0	0	0	0.632777552	0	0
**ENSG00000126261**	UBA2	2016.534873	1975.807096	1900.420334	2237.925554	2102.380097	2013.378965	1808.889721	2038.430605	1890.856524	1800.252137	2008.703981	2141.629449
**ENSG00000126262**	FFAR2	21.09346101	10.50961221	12.21349829	4.186168264	0	3.41443578	11.23533988	16.61312636	7.972361335	12.0227735	1.379604382	3.383300867
**ENSG00000126264**	HCST	2.812461468	0	0.814233219	0	0	2.655672273	0	0	0.468962431	0	1.379604382	0.845825217
**ENSG00000126266**	FFAR1	0	0	2.442699658	0	0	0.379381753	3.064183605	0	0.468962431	6.327775524	0	0
**ENSG00000126267**	COX6B1	739.6773662	907.3298542	782.4781239	768.5804933	766.2880727	880.1656677	708.8478072	777.4943137	833.8152031	808.6897119	917.4369142	890.6539533

**ENSG00000126351**	THRA	116.7171509	192.6762239	89.56565414	133.1201508	183.7704428	113.814526	46.9841486	61.46856754	39.86180667	29.74054496	45.52694462	38.06213475
**ENSG00000126368**	NR1D1	368.4324523	437.9005088	454.3421365	336.5679285	472.7176798	479.5385362	100.0966644	68.11381808	147.2542035	82.26108181	70.3598235	98.11572515
**ENSG00000126391**	FRMD8	590.6169083	816.2465484	648.1296427	550.8997436	576.7386852	677.5758114	521.9326073	538.2652941	710.0091212	557.4770236	587.7114669	527.7949353
**ENSG00000126432**	PRDX5	2009.503719	2217.528177	2329.521241	1673.630072	2094.289574	2189.412098	1869.151999	2020.156166	2108.924054	2017.927615	2124.590749	1977.539357
**ENSG00000126453**	BCL2L12	158.904073	178.6634076	157.1470114	148.1903566	144.4736185	177.1712788	91.92550814	124.5984477	157.1024145	130.9849533	111.747955	137.8695103
**ENSG00000126456**	IRF3	727.0212896	809.2401403	822.3755516	565.1327157	495.8334588	524.6849648	453.4991735	500.0551035	593.2374758	425.2265152	474.5839075	448.2873649
**ENSG00000126457**	PRMT1	809.9889029	560.5126513	729.5529646	807.0932414	575.5828962	871.4398873	452.477779	455.1996623	565.5686923	460.6620581	328.345843	535.4073622
**ENSG00000126458**	RRAS	179.997534	217.1986524	247.5268987	157.3999267	239.2483123	240.5280316	128.6957114	89.71088235	118.1785327	112.6344043	118.6459769	109.111453
**ENSG00000126460**	PRRG2	60.46792157	94.5865099	72.46675653	76.18826241	73.97049269	95.98358359	86.81853546	53.16200436	79.72361335	61.37942258	91.05388923	69.35766778
**ENSG00000126461**	SCAF1	665.1471373	592.0414879	732.8098975	701.6018011	644.9302331	823.2584047	561.7669942	478.4580392	584.3271896	496.0976011	593.2298844	529.4865857
**ENSG00000126464**	PRR12	472.4935267	469.4293454	502.3818964	510.7125282	587.1407857	578.177792	461.6703298	378.779281	463.8038447	404.9776335	429.0569629	379.7755223
**ENSG00000126522**	ASL	111.092228	105.0961221	115.6211172	104.6542066	132.9157291	130.8867049	79.66877372	111.3079466	84.41323766	78.4644165	102.0907243	81.19922081
**ENSG00000126524**	SBDS	1373.887427	1261.153465	1252.290692	1579.022669	1374.233059	1558.120861	996.881066	918.7058878	1053.289621	1041.551851	869.1507609	1280.579378
**ENSG00000126561**	STAT5A	66.09284451	133.1217547	82.23755516	30.1404115	43.91998003	28.83301325	24.51346884	18.274439	18.75849726	5.694997971	16.55525259	7.612426951
**ENSG00000126562**	WNK4	22.49969175	28.02563256	26.86969624	13.39573845	18.49262317	25.41857747	3.064183605	29.90362745	6.096511609	5.062220419	12.41643944	0.845825217
**ENSG00000126581**	BECN1	676.3969831	585.0350798	741.7664629	689.88053	670.35759	713.2376962	681.2701548	724.3323094	673.899014	742.8808465	645.6548509	612.3774569
**ENSG00000126602**	TRAP1	809.9889029	655.0991612	784.9208235	910.0729807	863.3743444	882.0625764	677.1845766	440.2478486	725.4848815	738.4514036	546.3233354	759.5510447
**ENSG00000126603**	GLIS2	120.9358431	80.57369362	144.1192798	128.9339825	144.4736185	166.1692079	74.56180105	121.2758224	99.42003547	76.56608384	120.0255813	59.20776517
**ENSG00000126653**	NSRP1	753.7396735	739.1760589	773.5215585	770.2549606	683.0712684	646.8458894	713.9547799	553.2171078	559.9411432	685.9308668	640.1364334	592.923477
**ENSG00000126698**	DNAJC8	1781.69434	1751.602035	1522.61612	1701.258783	1745.241312	1660.174552	1622.995916	1591.537505	1473.47996	1490.191136	1710.709434	1702.646161
**ENSG00000126705**	AHDC1	1217.795816	1674.531546	1254.733391	1386.458929	2161.325333	1693.180765	463.7131188	1417.099679	588.5478515	484.0748276	1418.233305	543.8656144
**ENSG00000126709**	IFI6	784.6767497	1036.948405	659.5289078	180.842469	347.8924734	271.6373354	870.2281437	606.3791122	785.9810351	372.7059784	318.6886123	322.2594076
**ENSG00000126746**	ZNF384	381.088529	329.3011826	481.2118327	346.6147323	373.3198303	376.3466993	588.3232521	475.1354139	573.5410537	591.0142339	553.2213573	526.9491101
**ENSG00000126749**	EMG1	556.8673707	350.320407	569.9632536	455.4551072	414.9282324	530.3756911	548.4888652	546.5718573	536.0240592	592.9125666	491.1391601	615.7607578
**ENSG00000126752**	SSX1	1011.079898	1254.147057	1147.254606	942.7250931	1159.256315	1046.334876	600.5799865	711.0418083	584.796152	527.7364787	578.0542362	590.3860013
**ENSG00000126756**	UXT	329.0579918	518.4742024	360.7053162	411.0817236	404.5261319	361.1714291	294.161626	357.1822168	351.7218236	395.4859702	339.382678	396.6920267
**ENSG00000126759**	CFP	0	3.50320407	0.814233219	7.535102876	0	6.449489806	5.106972674	1.661312636	1.406887294	3.796665314	1.379604382	0.845825217
**ENSG00000126767**	ELK1	393.7446056	399.365264	336.2783196	463.8274437	479.6524135	433.633344	298.2472042	270.7939597	324.990965	360.6832049	259.3656239	367.0881441
**ENSG00000126768**	TIMM17B	322.0268381	234.7146727	347.6775847	324.0094237	335.178795	423.7694184	320.717884	289.0683987	378.9216446	323.9821068	358.6971394	385.6962988
**ENSG00000126773**	PCNX4	1563.728576	1692.047566	1524.244587	1410.738705	1314.132034	1441.650663	1270.614801	1302.469107	1124.571911	1277.577878	1229.227505	1256.896272
**ENSG00000126775**	ATG14	338.9016069	392.3588559	423.4012741	350.8009006	401.058765	353.9631758	575.0451231	656.2184913	488.6588536	496.0976011	531.1476872	586.1568752
**ENSG00000126777**	KTN1	3758.854752	3811.486029	3693.361883	3630.245119	4045.261319	3302.138781	3849.636002	4199.798344	3720.747931	4002.318019	4420.252441	4394.062001
**ENSG00000126778**	SIX1	184.2162262	213.6954483	150.6331456	104.6542066	70.50312584	87.25780326	131.759895	146.195512	149.5990156	63.27775524	84.15586732	101.499026
**ENSG00000126785**	RHOJ	1.406230734	3.50320407	0	0.837233653	0	2.655672273	0	0	0	0	0	0
**ENSG00000126787**	DLGAP5	1614.352883	924.8458746	1272.646522	2097.2703	1969.464368	1887.803604	1788.461831	1473.584308	1950.883715	1880.614886	1754.856774	2352.239928
**ENSG00000126790**	L3HYPDH	344.5265299	238.2178768	341.9779522	175.8190671	165.2778196	176.791897	357.4880872	215.9706427	315.6117164	305.6315578	231.7735362	276.5848459
**ENSG00000126803**	HSPA2	0	3.50320407	8.956565414	2.511700959	0	2.27629052	0	1.661312636	3.751699452	2.53111021	2.759208765	0.845825217
**ENSG00000126804**	ZBTB1	566.7109859	966.8843234	551.2358896	505.6891263	654.1765447	443.4972696	626.1148499	1289.178606	741.4296041	683.3997566	1055.397352	759.5510447
**ENSG00000126814**	TRMT5	421.8692202	234.7146727	323.2505881	387.6391813	292.4146039	341.0641962	346.2527473	315.6494009	406.1214656	337.2704354	321.4478211	351.8632902
**ENSG00000126821**	SGPP1	71.71776744	28.02563256	61.06749146	61.95529031	88.99574902	94.84543833	249.2202665	181.0830773	217.1296058	273.3599026	169.691339	222.452032
**ENSG00000126822**	PLEKHG3	592.0231391	560.5126513	685.5843708	838.9081202	798.6501633	929.8646773	648.5855296	539.9266067	861.0150241	774.5197241	721.5330919	889.808128
**ENSG00000126838**	PZP	0	0	0	0	0	0	4.08557814	9.967875817	7.503398903	3.163887762	12.41643944	5.920776517
**ENSG00000126858**	RHOT1	735.458674	728.6664466	754.7941944	684.857128	757.0417611	747.7614358	669.0134203	656.2184913	673.899014	602.4042299	522.8700609	655.514543
**ENSG00000126870**	WDR60	379.6822982	924.8458746	361.5195494	457.1295745	767.4438616	300.8497304	901.8913743	1460.293807	627.9406957	828.9385936	1390.641217	676.6601734
**ENSG00000126878**	AIF1L	201.090995	262.7403053	121.3207497	215.1690488	216.1325333	171.1011707	42.89857046	34.88756536	25.79293373	43.02887356	64.84140597	47.36621214
**ENSG00000126882**	FAM78A	8.437384405	24.52242849	8.142332194	16.74467306	47.38734688	18.96908767	122.5673442	166.1312636	233.0743284	234.1276944	340.7622824	257.9766911
**ENSG00000126883**	NUP214	2674.650856	2627.403053	2991.492848	2725.19554	2594.746189	2868.505437	2586.170962	2776.053415	2829.250349	2991.772268	2931.659312	2704.949043
**ENSG00000126890**	CTAG2	260.1526858	203.1858361	175.8743754	237.7743574	228.8462118	276.5692982	170.5728873	142.8728867	197.4331836	199.324929	142.0992514	195.3856251
**ENSG00000126903**	SLC10A3	188.4349184	199.682632	218.2145028	226.8903199	182.6146538	241.6661769	203.2575124	247.5355828	252.3017881	218.3082556	274.5412721	251.2100894
**ENSG00000126934**	MAP2K2	960.4555914	1043.954813	838.660216	923.4687191	1113.024757	1056.578183	897.8057962	1038.320398	1002.641678	850.4530304	884.3264091	1039.519191
**ENSG00000126945**	HNRNPH2	1016.704821	1177.076568	1117.127977	923.4687191	1101.466868	991.3245214	926.4048431	1034.997772	1007.800265	963.0874347	1038.8421	1038.673366
**ENSG00000126947**	ARMCX1	73.12399818	122.6121425	83.05178838	21.76807497	43.91998003	51.9753002	45.96275407	38.21019063	31.42048291	44.92720622	49.66575776	41.44543562
**ENSG00000126953**	TIMM8A	185.6224569	94.5865099	147.3762127	209.3084132	157.187297	217.0063629	119.5031606	103.0013834	195.5573339	215.7771454	125.5439988	185.2357225
**ENSG00000126970**	ZC4H2	0	0	0	0	0	0	0	4.983937908	1.875849726	9.491663286	4.138813147	6.766601734
**ENSG00000127022**	CANX	33414.85471	33613.24306	33028.55631	25117.84682	25002.02653	26061.62954	18207.37898	20329.48273	19123.35003	17203.32332	17955.55104	19947.09609
**ENSG00000127054**	INTS11	473.8997574	465.9261414	460.8560022	467.1763783	421.8629661	495.8519516	405.4936303	388.7471569	475.0589431	443.5770642	409.7425015	429.6792101
**ENSG00000127080**	IPPK	662.3346758	490.4485699	597.6471831	612.8550339	444.9787451	581.9716096	420.8145484	415.328159	421.1282634	451.1703948	478.7227207	447.4415397
**ENSG00000127081**	ZNF484	359.9950679	437.9005088	336.2783196	413.5934245	316.6861718	300.4703486	541.3391035	526.6361057	453.0177088	432.8198458	521.4904565	554.015517
**ENSG00000127084**	FGD3	0	0	0.814233219	5.023401917	3.467366845	3.793817533	153.2091802	146.195512	111.6130587	293.6087843	135.2012295	126.8737825
**ENSG00000127124**	HIVEP3	64.68661377	91.08330583	74.09522297	67.81592588	83.21680428	119.884634	96.01108628	147.8568246	162.7299637	130.3521758	175.2097566	159.0151408
**ENSG00000127125**	PPCS	876.0817474	984.4003438	799.5770215	781.9762318	813.6754196	793.6666279	892.6988235	958.577391	909.787117	830.8369263	829.1422338	937.1743402
**ENSG00000127129**	EDN2	0	3.50320407	0	0	1.155788948	1.517527013	2.04278907	0	0	1.898332657	0	0
**ENSG00000127152**	BCL11B	182.8099954	136.6249587	180.7597747	225.2158526	216.1325333	201.451711	61.28367209	43.19412854	62.84096581	87.95607978	59.32298844	107.4198025
**ENSG00000127184**	COX7C	2796.99293	2469.75887	2415.015729	3298.700592	2776.205054	2903.029176	2201.105223	2480.339766	2447.045967	2524.782434	2483.287888	3088.953692
**ENSG00000127191**	TRAF2	867.644363	1054.464425	1185.523568	839.7453538	953.5258823	1080.099852	553.5958379	498.3937908	591.3616261	430.2887356	517.3516434	510.0326057
**ENSG00000127220**	ABHD8	88.59253625	108.5993262	65.13865756	105.4914403	124.8252064	134.6805224	112.3533988	127.921073	118.6474952	98.08052062	139.3400426	112.4947538
**ENSG00000127249**	ATP13A4	0	7.006408141	0.814233219	0	0	1.896908767	3.064183605	1.661312636	0.937924863	0.632777552	5.518417529	2.53747565

**ENSG00000127252**	HRASLS	0	0	0	0	6.93473369	0	6.128367209	11.62918845	6.096511609	27.20943475	34.49010956	1.691650434
**ENSG00000127311**	HELB	271.4025317	185.6698157	262.9973299	109.6776085	113.2673169	113.814526	247.1774774	255.842146	239.17084	229.065474	177.9689653	197.9231007
**ENSG00000127314**	RAP1B	1238.889277	1418.797649	1312.54395	1138.637768	1070.260566	1264.479384	1223.630653	1285.85598	1125.509835	1170.638472	1215.431461	1209.53006
**ENSG00000127324**	TSPAN8	1.406230734	0	4.071166097	1.674467306	3.467366845	1.517527013	6.128367209	6.645250545	3.28273702	7.593330629	1.379604382	2.53747565
**ENSG00000127325**	BEST3	30.93707615	63.05767327	43.15436063	10.04680383	12.71367843	14.79588838	3.064183605	1.661312636	3.28273702	1.898332657	5.518417529	1.691650434
**ENSG00000127328**	RAB3IP	923.8935923	1198.095792	1032.447722	812.1166433	828.7006759	821.3614959	577.0879122	473.4741013	498.0381022	487.2387153	491.1391601	589.5401761
**ENSG00000127329**	PTPRB	330.4642225	521.9774065	330.5786871	416.1051255	544.3765946	314.1280917	1.021394535	4.983937908	6.096511609	2.53111021	5.518417529	3.383300867
**ENSG00000127334**	DYRK2	3142.925691	2592.371012	2800.962275	2859.152925	2501.127284	2827.532207	898.8271907	795.7687527	1069.703306	947.2679959	765.6804322	977.7739506
**ENSG00000127337**	YEATS4	246.0903785	196.1794279	158.7754778	226.8903199	186.0820207	189.6908767	291.0974424	313.9880882	266.3706611	326.513217	271.7820633	313.8011554
**ENSG00000127362**	TAS2R3	50.62430643	35.0320407	44.78282707	56.09465474	41.60840214	34.9031213	78.64737919	86.38825708	77.37880119	75.30052873	52.42496653	36.37048432
**ENSG00000127364**	TAS2R4	125.1545353	273.2499175	109.9214846	107.1659076	166.4336086	103.1918369	187.9365944	224.2772059	154.7576024	131.6177309	241.4307669	151.4027138
**ENSG00000127366**	TAS2R5	18.28099954	56.05126513	32.56932878	15.07020575	25.42735686	18.96908767	49.02693767	49.83937908	36.57906965	20.24888168	44.14734023	32.14135824
**ENSG00000127377**	CRYGN	0	0	0.814233219	0	1.155788948	0	2.04278907	0	1.406887294	2.53111021	5.518417529	0
**ENSG00000127399**	LRRC61	15.46853808	35.0320407	31.75509556	11.72127114	12.71367843	11.76083435	18.38510163	8.306563181	16.4136851	20.88165923	27.59208765	11.84155303
**ENSG00000127412**	TRPV5	0	0	0	0	0	0	7.149761744	0	0	2.53111021	1.379604382	2.53747565
**ENSG00000127415**	IDUA	21.09346101	31.52883663	35.01202844	7.535102876	6.93473369	12.51959786	19.40649616	23.25837691	11.25509835	21.51443678	19.31446135	17.76232955
**ENSG00000127418**	FGFRL1	23.90592248	35.0320407	26.86969624	38.51274803	55.47786952	54.63097248	40.8557814	48.17806645	55.33756691	73.40219608	62.0821972	83.73669646
**ENSG00000127419**	TMEM175	44.99938349	31.52883663	52.11092604	72.00209415	68.19154795	56.907263	76.60459012	79.74300653	73.15813931	69.60553076	95.19270238	70.20349299
**ENSG00000127423**	AUNIP	393.7446056	231.2114686	342.7921854	521.5965657	410.3050766	455.6374857	436.1354664	363.8274673	440.3557231	454.3342826	358.6971394	473.6621214
**ENSG00000127445**	PIN1	345.9327606	409.8748762	355.0056837	390.9881159	425.330333	484.470499	327.8676457	289.0683987	352.190786	347.3948763	303.5129641	383.1588232
**ENSG00000127452**	FBXL12	170.1539188	101.592918	149.0046792	149.0275902	175.6799201	156.3052824	228.7923758	222.6158932	235.888103	239.8226924	191.7650091	201.3064016
**ENSG00000127463**	EMC1	1645.289959	1558.925811	1839.352843	1937.358673	1850.418106	2215.210058	1785.397647	1774.281895	2014.662606	2070.448151	2019.740816	2273.578183
**ENSG00000127481**	UBR4	8016.921415	9020.750481	9178.851083	7252.117901	7892.882728	8048.204515	8352.964506	8607.260768	8399.58611	7942.623837	8495.603786	7614.118601
**ENSG00000127483**	HP1BP3	4806.496649	4680.280638	4672.070213	5224.337994	5312.006006	5187.286713	6952.632599	7278.210659	6547.653468	7261.122414	7872.022605	7049.953182
**ENSG00000127507**	ADGRE2	44.99938349	77.07048955	28.49816268	34.32657977	93.61890481	33.38559429	4.08557814	21.59706427	4.689624315	4.429442867	12.41643944	8.458252168
**ENSG00000127511**	SIN3B	673.5845217	385.3524477	745.837629	684.0198944	641.4628663	730.3098751	729.2756979	515.0069172	712.3539334	842.2269222	608.4055326	668.2019213
**ENSG00000127526**	SLC35E1	808.5826721	935.3554868	840.2886825	994.6335796	1011.31533	1012.190518	642.4571624	1034.997772	819.2773677	762.4969506	993.3151553	856.8209446
**ENSG00000127527**	EPS15L1	452.8062964	514.9709984	577.2913526	540.8529398	566.3365846	617.2541126	465.7559079	485.1032897	483.0313044	485.9731602	568.3970055	523.5658092
**ENSG00000127528**	KLF2	5.624922937	14.01281628	5.699632536	0.837233653	4.623155793	6.070108053	3.064183605	18.274439	5.158586746	8.858885733	20.69406573	6.766601734
**ENSG00000127530**	OR7C1	9.843615139	0	12.21349829	13.39573845	20.80420107	12.89897961	13.27812895	11.62918845	4.220661883	8.226108181	13.79604382	6.766601734
**ENSG00000127540**	UQCR11	181.4037647	108.5993262	118.87805	174.1445998	158.3430859	148.3382655	200.1933288	127.921073	169.7644002	226.5343638	223.4959099	208.9188285
**ENSG00000127554**	GFER	219.3719945	136.6249587	217.4002696	236.9371238	221.9114781	307.2992202	171.5942819	254.1808333	186.1780853	228.4326964	201.4222398	208.0730033
**ENSG00000127561**	SYNGR3	25.31215321	52.54806106	26.05546302	23.44254228	61.25681426	37.17941182	17.36370709	43.19412854	15.00679781	11.38999594	8.277626294	13.53320347
**ENSG00000127564**	PKMYT1	503.4306028	413.3780803	487.7256984	608.6688656	673.8249568	633.9469098	391.1941069	405.3602832	481.6244171	456.8653928	527.008874	479.5828979
**ENSG00000127578**	WFIKKN1	2.812461468	14.01281628	11.39926507	11.72127114	15.02525633	13.65774312	14.29952349	13.29050109	17.8205724	10.75721839	35.86971394	17.76232955
**ENSG00000127580**	WDR24	199.6847643	147.134571	196.2302059	137.3063191	116.7346838	147.579502	180.7868327	162.8086383	184.3022356	158.8271656	139.3400426	169.1650434
**ENSG00000127585**	FBXL16	14.06230734	42.03844885	8.956565414	10.88403749	28.89472371	14.03712487	39.83438686	33.22625272	20.16538455	33.53721028	12.41643944	21.14563042
**ENSG00000127586**	CHTF18	306.5583	325.7979785	258.1119306	350.8009006	285.4798702	281.1218792	311.5253331	292.391024	241.9846146	280.9532333	314.5497992	277.4306711
**ENSG00000127589**	TUBBP1	18.28099954	10.50961221	13.84196473	19.25637402	32.36209055	17.45156065	14.29952349	13.29050109	13.13094808	7.593330629	5.518417529	15.2248539
**ENSG00000127603**	MACF1	33945.00369	36282.68456	34294.68897	32504.75934	31627.00878	32058.8963	14433.32617	19724.76493	15070.5767	12379.66003	17365.08036	14408.63257
**ENSG00000127616**	SMARCA4	2399.029632	1961.794279	2204.129325	2628.91367	2528.866219	2675.020743	2112.243898	2018.494853	2276.343642	2205.22977	2203.228199	2381.84381
**ENSG00000127663**	KDM4B	440.1502198	791.7241199	368.0334152	415.2678918	693.473369	466.6395566	628.1576389	1149.628344	694.533361	885.8885733	1146.451242	694.422503
**ENSG00000127666**	TICAM1	739.6773662	697.13761	875.3007109	503.1774254	444.9787451	733.7243109	157.2947584	144.5341993	215.253756	129.0866207	176.5893609	173.3941694
**ENSG00000127720**	METTL25	40.78069129	42.03844885	26.05546302	26.79147689	39.29682424	25.03919572	79.66877372	89.71088235	62.37200338	77.83163894	84.15586732	43.98291127
**ENSG00000127774**	EMC6	303.7458386	245.2242849	242.6414994	268.7520026	238.0925233	266.7053726	156.2733638	144.5341993	170.702325	160.0927208	148.9972733	191.156499
**ENSG00000127804**	METTL16	683.4281368	504.4613861	750.7230283	817.1400452	737.393349	826.6728405	603.6441701	622.9922385	705.3194969	748.5758445	652.5528728	822.1421107
**ENSG00000127824**	TUBA4A	68.90530597	255.7338971	150.6331456	82.88613163	295.8819708	253.0476295	33.70601965	69.77513072	26.26189616	51.8877593	78.63744979	44.82873649
**ENSG00000127831**	VIL1	8.437384405	10.50961221	2.442699658	0	4.623155793	4.931962793	4.08557814	0	0.937924863	1.265555105	0	0
**ENSG00000127837**	AAMP	777.645596	882.8074257	841.9171489	843.0942884	807.8964748	861.5759618	548.4888652	603.0564869	692.6575113	592.9125666	622.2015764	685.1184256
**ENSG00000127838**	PNKD	179.997534	301.2755501	162.8466439	325.683891	317.8419608	301.9878756	231.8565594	526.6361057	321.2392655	223.370476	509.0740171	363.7048432
**ENSG00000127863**	TNFRSF19	0	0	0	4.186168264	1.155788948	1.896908767	0	16.61312636	4.689624315	0	1.379604382	4.229126084
**ENSG00000127870**	RNF6	1663.570959	1534.403383	1826.325111	1662.746035	1562.626658	1723.531305	1240.99436	1202.790349	1180.37844	1152.9207	1146.451242	1360.086949
**ENSG00000127884**	ECHS1	777.645596	746.182467	674.1851057	751.8358203	780.1575401	741.6913277	768.0886902	654.5571786	692.6575113	789.7063854	775.3376629	825.5254116
**ENSG00000127903**	ZNF835	0	0	0	0.837233653	4.623155793	0	19.40649616	41.5328159	16.88264753	31.00610007	15.17564821	13.53320347
**ENSG00000127914**	AKAP9	2181.063869	2886.640154	2613.688634	2657.379614	3162.238562	2245.560598	3933.390354	3141.542195	2955.401243	3741.613667	3711.135788	3413.750575
**ENSG00000127920**	GNG11	23.90592248	31.52883663	29.3123959	12.55850479	12.71367843	29.97115851	1.021394535	0	0.937924863	1.265555105	0	3.383300867
**ENSG00000127922**	SEM1	1161.546586	1268.159873	1167.610437	1119.381394	1017.094274	1132.454534	986.6671207	1020.045959	972.6280828	1058.636845	1048.499331	1128.330839
**ENSG00000127928**	GNGT1	46.40561423	87.58010176	77.35215585	51.90848648	61.25681426	64.87427982	35.74880872	21.59706427	35.64114479	23.41276944	30.35129641	25.3747565
**ENSG00000127946**	HIP1	129.3732275	178.6634076	119.6922833	239.4488247	218.4441112	183.2413868	14.29952349	8.306563181	8.441323766	1.898332657	16.55525259	14.37902869
**ENSG00000127947**	PTPN12	3691.355677	3814.989233	3514.230575	2526.771164	2542.735686	2457.255616	2107.136925	2305.901939	2050.772713	2014.130949	2165.97888	2273.578183
**ENSG00000127948**	POR	403.5882207	406.3716722	380.2469135	501.5029581	466.9387351	530.7550729	310.5039386	340.5690904	366.7286214	351.8243191	395.9464577	372.1630954
**ENSG00000127952**	STYXL1	447.1813735	465.9261414	476.3264334	400.1976861	464.6271572	436.6683981	390.1727123	330.6012146	355.9424855	282.8515659	354.5583263	347.6341641
**ENSG00000127954**	STEAP4	2428.560478	3758.937968	2227.742088	1317.80577	1422.776195	1160.149402	735.4040651	814.0431917	895.2492816	993.4607572	1005.731595	1461.585975
**ENSG00000127955**	GNAI1	665.1471373	294.2691419	416.0731751	771.0921943	577.8944741	589.1798629	962.1536519	1239.339227	1061.730945	1095.337943	1185.080164	1225.600739
**ENSG00000127957**	PMS2P3	119.5296124	77.07048955	140.862347	135.6318518	151.4083522	109.261945	180.7868327	144.5341993	115.3647581	149.3355024	189.0058004	118.4155303
**ENSG00000127980**	PEX1	458.4312193	479.9389576	451.0852036	495.6423225	480.8082025	467.0189383	614.87951	408.6829085	571.6652039	643.5347708	462.1674681	669.8935717
**ENSG00000127989**	MTERF1	295.3084542	290.7659378	236.1276336	280.4732737	223.067267	272.0167171	411.6219976	388.7471569	358.7562601	415.1020744	379.3912051	410.2252301

ENSG00000127990	SGCE	790.3016726	704.1440182	542.2793242	1022.26229	904.9827465	668.8500311	2069.345328	2006.865664	1846.305093	1704.069949	1811.420554	1683.192181
ENSG00000127993	RBM48	284.0586083	371.3396315	276.0250614	275.4498718	266.987247	265.9466091	326.8462512	407.0215959	289.8187826	287.9137863	278.6800852	348.4799893
ENSG00000127995	CASD1	223.5906867	196.1794279	222.2856689	364.196639	242.7156791	256.0826835	483.119615	390.4084695	446.4522347	484.0748276	495.2779732	436.4458119
ENSG00000128000	ZNF780B	234.8405326	161.1473872	275.2108282	200.098843	160.6546638	226.4909067	240.0277157	224.2772059	189.4608223	249.3143556	195.9038223	207.2271781
ENSG00000128016	ZFP36	721.3963666	854.7817932	788.1777564	403.5466207	423.0187551	539.4808532	368.7234271	357.1822168	432.8523242	321.4509966	317.3090079	309.5720293
ENSG00000128039	SRD5A3	7.031153671	14.01281628	16.28466439	3.348934611	21.95999002	16.69279715	12.25673442	3.322625272	6.56547404	13.92110615	22.07367012	13.53320347
ENSG00000128045	RASL11B	0	0	0.814233219	0.837233653	0	0	10.21394535	6.645250545	7.503398903	9.491663286	2.759208765	14.37902869
ENSG00000128050	PAICS	3438.234145	2753.518399	2615.317101	3781.78441	3524.000503	3186.806728	2510.587767	3018.60506	2885.056878	2905.714521	2851.642258	3438.279506
ENSG00000128052	KDR	11.24984587	17.51602035	8.956565414	0	2.311577897	1.896908767	0	3.322625272	0	0.632777552	0	3.383300867
ENSG00000128059	PPAT	558.2736015	297.772346	394.0888782	426.989163	277.3893476	415.8024016	336.038802	310.665463	367.1975838	355.6209844	235.9123494	427.9875597
ENSG00000128159	TUBGCP6	226.4031482	220.7018564	281.7246939	245.3094603	246.183046	221.1795622	294.161626	255.842146	306.2324677	295.507117	248.3287888	279.1223215
ENSG00000128165	ADM2	64.68661377	52.54806106	116.4353504	39.34998168	39.29682424	103.5712187	54.13391035	11.62918845	42.67558126	23.41276944	23.4532745	27.06640694
ENSG00000128185	DGCR6L	143.4355349	224.2050605	175.0601422	114.7010104	189.5493875	187.0352044	201.2147234	197.6962037	249.956976	210.0821474	187.626196	285.0430981
ENSG00000128191	DGCR8	1409.043196	1446.823281	1428.9793	1612.512015	1239.005753	1312.660866	1545.369931	2026.801416	1410.638994	1555.367224	1823.836993	1366.85355
ENSG00000128203	ASPHD2	82.96761332	98.08971397	112.3641843	5.86063557	19.64841212	28.83301325	87.83993	44.85544118	57.68237907	35.43554293	38.6289227	15.2248539
ENSG00000128228	SDF2L1	465.462373	430.8941007	539.8366245	307.2647506	194.1725433	323.2332538	218.5784305	156.1633878	206.8124323	201.2232617	108.9887462	219.9145564
ENSG00000128245	YWHAH	1676.227035	1506.37775	1703.375895	1620.884352	1500.214055	1573.296131	993.8168824	1126.369967	1115.661624	914.3635632	935.3717712	1033.598415
ENSG00000128254	C22orf24	16.87476881	3.50320407	13.02773151	6.697869223	6.93473369	5.311344546	9.192550814	0	1.406887294	5.694997971	0	2.53747565
ENSG00000128262	POM121L9P	1.406230734	0	0	0	0	0	5.106972674	0	2.344812157	0	0	0.845825217
ENSG00000128266	GNAZ	78.74892111	63.05767327	56.18209214	112.1893095	137.5388848	78.53202293	20.4278907	31.56494009	26.73085859	34.80276538	35.86971394	11.84155303
ENSG00000128268	MGAT3	11.24984587	10.50961221	1.628466439	32.65211246	33.5178795	23.5216687	162.401731	513.3456046	172.1092123	146.8043922	521.4904565	209.7646538
ENSG00000128271	ADORA2A	2.812461468	31.52883663	10.58503185	12.55850479	2.311577897	3.035054026	3.064183605	6.645250545	3.751699452	1.898332657	6.898021912	8.458252168
ENSG00000128272	ATF4	6533.347991	6536.978795	7438.02046	5387.598556	5427.584901	6450.24857	4361.354664	4135.007151	4515.17029	3703.014237	3395.206385	3808.750951
ENSG00000128274	A4GALT	184.2162262	143.6313669	140.862347	129.7712162	112.111528	114.5732895	164.4445201	229.2611438	209.6262069	194.8954861	223.4959099	182.6982468
ENSG00000128283	CDC42EP1	608.8979079	665.6087734	805.276654	689.88053	806.7406859	999.2915382	38.81299233	184.4057026	51.58586746	34.80276538	147.6176689	27.06640694
ENSG00000128284	APOL3	3121.83223	2693.96393	2848.187802	246.9839276	358.294574	275.4311529	1434.037927	1392.179989	1407.825219	328.4115497	198.6630311	213.9937798
ENSG00000128294	TPST2	112.4984587	73.56728548	113.9926507	106.3286739	95.93048271	121.4021611	274.7551299	176.0991394	279.0326467	261.9699067	180.7281741	207.2271781
ENSG00000128298	BAIAP2L2	2.812461468	24.52242849	1.628466439	0	2.311577897	2.27629052	6.128367209	1.661312636	1.406887294	1.265555105	0	0
ENSG00000128309	MPST	392.3383748	301.2755501	359.891083	381.7785457	367.5408855	412.0085841	154.2305748	182.74439	203.5296953	182.2399351	168.3117346	197.9231007
ENSG00000128311	TST	126.5607661	175.1602035	157.9612446	169.1211979	124.8252064	169.204262	138.9096567	134.5663235	131.7784432	137.3127289	96.57230676	144.6361121
ENSG00000128335	APOL2	3981.039208	4315.947415	4149.332486	702.4390348	1072.572144	779.629503	2031.55373	2382.32232	2131.903213	813.7519324	797.411333	653.8228926
ENSG00000128340	RAC2	1565.134807	1713.06679	1843.424009	1219.849432	1410.062517	1549.015699	11.23533988	18.274439	29.54463318	8.858885733	5.518417529	27.06640694
ENSG00000128342	LIF	732.6462125	1394.27522	1331.271314	502.3401917	510.8587151	831.6048032	4.08557814	0	12.19302322	2.53111021	1.379604382	13.53320347
ENSG00000128346	C22orf23	4.218692202	3.50320407	2.442699658	18.41914036	9.246311586	3.793817533	14.29952349	6.645250545	6.56547404	8.858885733	23.4532745	2.53747565
ENSG00000128383	APOBEC3A	78.74892111	255.7338971	159.589711	3.348934611	12.71367843	15.93403364	5.106972674	6.645250545	17.35160996	0.632777552	5.518417529	5.074951301
ENSG00000128394	APOBEC3F	660.9284451	721.6600385	522.7377269	30.1404115	58.94523636	35.66188481	51.06972674	61.46856754	49.2410553	8.226108181	4.138813147	2.53747565
ENSG00000128408	RIBC2	123.7483046	206.6890402	106.6645517	115.5382441	161.8104528	90.67223904	207.3430906	152.8407625	174.9229869	196.1610412	129.6828119	167.4733929
ENSG00000128422	KRT17	652.4910606	700.6408141	1109.799878	664.7635204	285.4798702	818.7058236	818.1370224	1787.572396	1432.211266	711.2419689	1563.091765	1110.56851
ENSG00000128463	EMC4	767.8019808	739.1760589	658.7146745	698.2528665	703.8754695	831.2254215	500.4833221	485.1032897	528.5206602	538.4936971	491.1391601	489.7328005
ENSG00000128482	RNF112	4.218692202	0	6.513865756	4.186168264	4.623155793	0.379381753	4.08557814	3.322625272	2.813774589	0	4.138813147	0.845825217
ENSG00000128487	SPECC1	1245.92043	1685.041158	1412.694636	1280.130255	1536.043512	1533.081665	1660.787514	1955.364973	1423.300979	1583.209436	1612.757523	1481.039955
ENSG00000128510	CPA4	111.092228	77.07048955	89.56565414	65.30422492	27.73893476	66.01242507	214.4928523	94.69482026	265.9016986	310.0610007	148.9972733	409.3794049
ENSG00000128512	DOCK4	2798.399161	2956.704235	3440.135352	2780.452961	2523.087274	3091.961289	30.64183605	36.54887799	42.67558126	32.27165517	40.00852709	44.82873649
ENSG00000128513	POT1	756.552135	889.8138339	705.125968	745.1379511	691.1617911	644.1902171	896.7844016	868.8665087	845.0703015	856.7808059	787.7541023	893.1914289
ENSG00000128524	ATP6V1F	362.8075294	357.3268152	350.9345176	394.3370505	463.4713683	524.3055831	268.6267627	337.2464651	322.1771904	315.7559986	295.2353378	356.9382415
ENSG00000128534	LSM 8.00	929.5185153	879.3042217	861.4587462	840.5825875	809.0522638	738.2768919	1156.218613	1164.580158	1082.834254	1101.665719	1223.709087	1071.66055
ENSG00000128536	CDHR3	50.62430643	31.52883663	43.96859385	41.86168264	31.2063016	29.59177676	2.04278907	0	1.875849726	0	0	0
ENSG00000128563	PRKRIP1	303.7458386	283.7595297	329.7644539	337.4051621	233.4693675	288.3301325	375.8731888	440.2478486	328.273702	370.8076457	419.3997322	374.700571
ENSG00000128564	VGF	5.624922937	7.006408141	4.071166097	20.09360767	21.95999002	12.89897961	32.68462512	6.645250545	10.78613592	13.2883286	16.55525259	11.84155303
ENSG00000128567	PODXL	809.9889029	1478.352118	1016.163058	405.221088	531.6629162	575.142738	230.8351649	460.1836002	609.6511609	147.4371697	183.4873828	363.7048432
ENSG00000128573	FOXP2	0	7.006408141	1.628466439	0	0	0	1.021394535	0	2.813774589	0.632777552	4.138813147	0
ENSG00000128578	STRIP2	296.7146849	518.4742024	394.0888782	365.8711063	458.8482125	440.0828338	560.7455996	465.1675381	627.9406957	605.5681176	578.0542362	652.9770674
ENSG00000128581	IFT22	171.5601496	168.1537954	153.8900785	231.9137218	236.9367344	193.1053124	195.0863562	220.9545806	194.1504466	182.2399351	212.4590749	235.1394103
ENSG00000128585	MKLN1	2285.124943	2270.076238	2287.995347	2505.003089	2550.826209	2346.855526	2652.561607	2953.813867	2665.113498	2690.570153	2970.288235	2783.610788
ENSG00000128590	DNAJB9	455.6187579	700.6408141	539.8366245	231.9137218	340.9577397	325.8889261	348.2955364	320.6333388	335.7771009	321.4509966	281.439294	322.2594076
ENSG00000128591	FLNC	26.71838395	77.07048955	42.34012741	30.1404115	32.36209055	22.38352344	7.149761744	4.983937908	10.31717349	3.163887762	12.41643944	5.920776517
ENSG00000128595	CALU	5931.481237	6456.405102	6416.157769	5876.543009	6629.605407	6952.170629	4072.300011	4076.861209	4296.633797	4284.536807	4014.648753	5432.735367
ENSG00000128596	CCDC136	0	14.01281628	6.513865756	14.2329721	12.71367843	11.76083435	0	8.306563181	7.034436472	5.062220419	22.07367012	1.691650434
ENSG00000128604	IRF5	9.843615139	0	2.442699658	0	8.090522638	0	1.021394535	3.322625272	4.220661883	0	1.379604382	0.845825217
ENSG00000128606	LRRC17	0	0	0	0.837233653	3.467366845	0	6.128367209	1.661312636	2.813774589	5.694997971	4.138813147	2.53747565
ENSG00000128607	KLHDC10	1344.356582	1145.547731	1280.788854	1274.26962	1263.27732	1289.139198	1366.625888	1284.194668	1379.218511	1330.098415	1269.236032	1435.365393
ENSG00000128609	NDUFA5	836.7072868	823.2529565	818.3043855	1039.844197	1005.536385	816.8089149	1458.551396	1377.228175	1267.605452	1494.620579	1241.643944	1655.279949
ENSG00000128626	MRPS12	154.6853808	80.57369362	130.2773151	137.3063191	104.0210053	169.204262	101.118059	63.12988017	124.2750443	124.0244003	92.43349361	144.6361121
ENSG00000128641	MYO1B	1272.638814	1467.842506	1534.829619	664.7635204	760.509128	864.6110158	876.3565109	960.2387037	1256.350354	885.2557958	932.6125624	1272.966951
ENSG00000128654	MTX2	293.9022234	343.3139989	300.452058	308.9392179	321.3093276	286.8126055	280.8834971	295.7136492	323.5840777	331.5754374	288.3373159	329.0260093
ENSG00000128655	PDE11A	7.031153671	21.01922442	13.84196473	12.55850479	15.02525633	7.208253313	0	8.306563181	0	0	0	0
ENSG00000128656	CHN1	5.624922937	0	5.699632536	4.186168264	12.71367843	6.828871559	7.149761744	14.95181373	3.28273702	6.327775524	9.657230676	7.612426951

**ENSG00000128683**	GAD1	7.031153671	52.54806106	44.78282707	8.372336529	25.42735686	32.24744903	0	0	0	0	1.379604382	0
**ENSG00000128692**	EIF2S2P4	15.46853808	3.50320407	13.02773151	14.2329721	15.02525633	14.03712487	17.36370709	11.62918845	14.06887294	8.858885733	9.657230676	23.68310607
**ENSG00000128694**	OSGEPL1	168.7476881	122.6121425	135.1627144	115.5382441	159.4988749	142.6475392	158.3161529	152.8407625	177.2677991	178.4432698	151.7564821	181.8524216
**ENSG00000128699**	ORMDL1	520.3053716	402.8684681	469.8125676	509.8752946	432.2650667	498.1282421	484.1410095	456.8609749	486.3140414	487.2387153	420.7793366	491.4244509
**ENSG00000128708**	HAT1	1103.891126	1408.288036	1107.357178	1286.828124	1274.83521	1177.600962	1265.507829	1540.036814	1246.971105	1278.843433	1353.391899	1314.412387
**ENSG00000128731**	HERC2	1393.574658	1166.566955	1321.500515	1445.065285	1413.529884	1471.242439	1824.210639	1706.168077	1788.153751	1861.631559	1867.984334	1832.05742
**ENSG00000128739**	SNRPN	4.218692202	0	8.956565414	10.88403749	8.090522638	12.51959786	44.94135953	44.85544118	10.78613592	39.8649858	19.31446135	17.76232955
**ENSG00000128789**	PSMG2	514.6804487	564.0158553	484.4687656	561.7837811	439.1998003	571.3489205	547.4674707	541.5879194	513.0449	518.877593	438.7141936	602.2275543
**ENSG00000128791**	TWSG1	352.9639143	455.4165292	393.274645	452.1061725	524.7281825	624.4623659	437.1568609	531.6200436	532.2723597	476.4814969	558.7397748	650.4395917
**ENSG00000128805**	ARHGAP22	15.46853808	3.50320407	23.61276336	21.76807497	17.33683422	26.17734098	24.51346884	6.645250545	23.44812157	22.77999189	6.898021912	18.60815477
**ENSG00000128829**	EIF2AK4	1042.016974	686.6279978	960.7951989	1308.596199	1096.843712	1206.813357	3332.810367	2799.311792	3363.398558	2923.432292	2521.916811	3176.919514
**ENSG00000128833**	MYO5C	514.6804487	928.3490787	810.1620533	457.1295745	809.0522638	879.4069042	755.8319558	729.3162473	777.5397113	866.2724692	877.4283871	784.9258012
**ENSG00000128849**	CGNL1	164.5289959	94.5865099	169.3605096	231.0764882	186.0820207	210.9362548	101.118059	677.8155555	372.3561706	83.52663691	495.2779732	296.0388259
**ENSG00000128872**	TMOD2	144.8417656	157.6441832	96.07951989	77.02549606	100.5536385	125.1959786	113.3747934	154.5020752	71.75125201	68.97275321	117.2663725	66.82019213
**ENSG00000128881**	TTBK2	593.4293698	602.5511001	824.0040181	452.9434062	480.8082025	547.44787	292.118837	388.7471569	337.1839882	330.9426599	358.6971394	362.859018
**ENSG00000128891**	CCDC32	171.5601496	161.1473872	161.2181775	134.7946181	156.031508	162.3753904	260.4556064	229.2611438	240.5777273	268.2976822	291.0965247	233.4477598
**ENSG00000128908**	INO80	1226.2332	938.8586909	1104.100246	1145.335637	798.6501633	1040.264768	901.8913743	687.7834314	823.0290672	927.6518918	645.6548509	845.8252168
**ENSG00000128915**	ICE2	1247.326661	987.9035479	1159.468104	1229.896236	1108.401601	1253.097931	874.3137219	691.1060566	846.4771888	838.4302569	735.3291358	784.9258012
**ENSG00000128917**	DLL4	4.218692202	45.54165292	17.91313083	9.209570182	2.311577897	3.035054026	0	4.983937908	5.158586746	1.265555105	2.759208765	0.845825217
**ENSG00000128918**	ALDH1A2	1.406230734	10.50961221	4.885399317	5.023401917	3.467366845	5.6907263	0	8.306563181	0	5.062220419	0	0
**ENSG00000128923**	MINDY2	1222.014508	1289.179098	1041.404288	1367.202555	1271.367843	1393.46918	921.2978705	973.5292048	1047.662072	812.4863773	900.8816616	976.0823002
**ENSG00000128928**	IVD	215.1533023	157.6441832	250.7838316	273.7754045	236.9367344	287.9507508	218.5784305	274.116585	220.4123428	263.2354618	234.532745	236.8310607
**ENSG00000128944**	KNSTRN	911.2375157	662.1055693	854.9448804	1334.550443	1092.220556	1261.823711	745.6180105	594.7499237	776.6017865	687.1964219	678.7653561	992.1529793
**ENSG00000128951**	DUT	923.8935923	893.317038	638.358844	763.5570914	867.9975001	709.0644969	777.281241	774.1716884	792.0775467	725.7958526	798.7909374	938.0201654
**ENSG00000128965**	CHAC1	160.3103037	80.57369362	232.0564675	35.16381342	39.29682424	151.3733196	106.2250316	46.51675381	115.3647581	51.8877593	31.73090079	36.37048432
**ENSG00000128973**	CLN6	244.6841477	248.727489	294.7524254	298.8924141	242.7156791	277.3280617	179.7654381	174.4378268	189.9297847	186.669378	147.6176689	189.4648486
**ENSG00000128989**	ARPP19	4559.00004	4540.152475	4150.960953	4081.514058	4204.760194	4247.937492	2618.855587	2676.374657	2676.368596	2512.126883	2635.04437	2730.3238
**ENSG00000129003**	VPS13C	2490.43463	2799.060052	2630.787532	956.1208316	1280.614155	1096.792649	3334.853156	3462.175534	2821.74695	2027.419278	2614.350304	2049.4345
**ENSG00000129007**	CALML4	33.74953762	21.01922442	16.28466439	18.41914036	6.93473369	11.00207085	19.40649616	8.306563181	13.59991051	10.75721839	23.4532745	10.99572782
**ENSG00000129028**	THAP10	60.46792157	35.0320407	52.92515926	36.00104707	60.10102531	50.83715494	20.4278907	11.62918845	19.22745969	27.20943475	23.4532745	32.14135824
**ENSG00000129038**	LOXL1	18.28099954	35.0320407	5.699632536	5.86063557	25.42735686	18.58970591	3.064183605	0	0.468962431	0.632777552	0	1.691650434
**ENSG00000129048**	ACKR4	32.34330689	49.04485699	35.82626166	0	0	0	13.27812895	19.93575163	7.034436472	1.898332657	4.138813147	0.845825217
**ENSG00000129055**	ANAPC13	750.927212	816.2465484	736.8810636	782.8134654	710.8102032	787.9759016	625.0934553	523.3134804	621.3752217	631.5119973	477.3431163	701.1891047
**ENSG00000129071**	MBD4	697.4904441	928.3490787	687.2128372	652.2050156	663.4228563	652.9159974	745.6180105	842.2855065	714.6987455	735.9202934	778.0968716	763.7801707
**ENSG00000129083**	COPB1	2320.280711	2592.371012	2501.32445	2120.712843	2420.222058	2085.84088	2708.738306	2521.872582	2623.375842	2360.26027	2526.055624	2617.829046
**ENSG00000129084**	PSMA1	741.0835969	672.6151815	602.5325824	447.9200043	402.214554	434.7714893	486.1837986	450.2157244	558.0652934	408.7742988	438.7141936	537.9448379
**ENSG00000129103**	SUMF2	1172.796432	1198.095792	1030.819256	1280.130255	1362.67517	1396.124852	1488.171837	1360.615049	1576.182732	1530.688899	1513.426007	1601.992961
**ENSG00000129116**	PALLD	46.40561423	45.54165292	83.8660216	113.8637768	97.08627165	178.3094241	101.118059	131.2436983	76.44087633	137.3127289	161.4137127	100.6532008
**ENSG00000129128**	SPCS3	3096.520077	2890.143358	2892.970629	2471.513743	2514.996751	2804.010539	1724.113975	1473.584308	1594.472267	1735.076049	1536.879282	2062.121878
**ENSG00000129151**	BBOX1	0	0	0	0	0	1.13814526	0	0	0	5.062220419	0	0.845825217
**ENSG00000129158**	SERGEF	116.7171509	133.1217547	155.5185449	140.6552537	173.3683422	146.8207385	212.4500633	250.8582081	205.405545	186.0366004	306.2721729	193.6939746
**ENSG00000129167**	TPH1	11.24984587	0	4.885399317	7.535102876	5.778944741	6.828871559	15.32091802	14.95181373	8.910286198	15.81943881	5.518417529	5.920776517
**ENSG00000129173**	E2F8	556.8673707	521.9774065	421.7728077	797.0464375	540.9092278	594.4912074	567.8953614	508.3616667	650.4508924	641.6364381	684.2837736	762.9343455
**ENSG00000129187**	DCTD	532.9614482	472.9325495	575.6628861	507.3635936	535.130283	644.1902171	316.6323058	294.0523366	333.9012512	311.3265558	235.9123494	323.951058
**ENSG00000129194**	SOX15	549.8362171	360.8300193	438.8717053	516.5731638	499.3008257	556.5530321	311.5253331	352.1982789	351.7218236	286.0154537	380.7708095	274.8931955
**ENSG00000129195**	PIMREG	307.9645308	192.6762239	229.6137679	399.3604524	347.8924734	405.1797125	166.4873092	94.69482026	217.1296058	215.1443678	110.3683506	271.5098946
**ENSG00000129197**	RPAIN	412.0256051	406.3716722	440.5001717	484.758285	465.7829462	470.4333741	522.9540019	468.4901634	530.8654724	520.1431481	488.3799513	546.40309
**ENSG00000129214**	SHBG	1.406230734	0	0	1.674467306	2.311577897	1.896908767	1.021394535	0	0.468962431	1.898332657	0	0.845825217
**ENSG00000129219**	PLD2	406.4006822	322.2947745	331.3929203	320.660489	398.7471872	374.8291723	383.0229506	470.151476	435.1971364	520.7759256	684.2837736	419.5293075
**ENSG00000129235**	TXNDC17	406.4006822	465.9261414	350.1202844	497.3167898	433.4208556	471.1921376	303.3541769	362.1661547	393.9284424	336.6376579	328.345843	403.4586284
**ENSG00000129244**	ATP1B2	5.624922937	10.50961221	4.885399317	2.511700959	1.155788948	2.27629052	0	0	0.468962431	0	0	0
**ENSG00000129245**	FXR2	1012.486129	900.3234461	1044.661221	1088.403749	1015.938486	1140.42155	530.1037636	510.0229793	580.1065277	560.0081339	561.4989836	675.8143482
**ENSG00000129250**	KIF1C	2271.062636	1933.768647	2220.413989	2723.521073	2892.939738	2884.060089	1500.428572	1491.858747	1703.271551	1633.83164	1556.193743	1883.652758
**ENSG00000129255**	MPDU1	485.1496033	553.5062431	577.2913526	503.1774254	484.2755693	570.9695387	326.8462512	476.7967266	404.2456159	396.1187478	499.4167864	425.450084
**ENSG00000129292**	PHF20L1	1267.013891	1142.044527	1297.073519	1203.104759	1223.980496	1222.368009	1582.140135	1609.811944	1428.459566	1438.936154	1419.612909	1585.076456
**ENSG00000129295**	LRRC6	5.624922937	24.52242849	3.256932878	0.837233653	11.55788948	5.311344546	48.00554314	73.09775599	42.20661883	48.72387153	62.0821972	45.67456171
**ENSG00000129315**	CCNT1	1406.230734	1544.912995	1593.45441	1362.179153	1281.769944	1490.97029	1636.274045	1598.182756	1605.727365	1538.28223	1420.992514	1677.271405
**ENSG00000129317**	PUS7L	559.6798322	525.4806106	510.5242286	696.5783992	648.3976	592.2149169	963.1750464	925.3511383	842.7254893	941.5729979	945.0290019	1032.75259
**ENSG00000129347**	KRI1	434.5252969	339.8107948	386.7607792	488.9444533	369.8524634	449.5673777	452.477779	377.1179684	485.845079	506.8548195	484.2411382	561.6279439
**ENSG00000129351**	ILF3	5958.199621	4571.681312	5689.047504	6068.269516	5122.456619	5910.008953	5473.653312	4807.838769	5914.554185	5932.289554	5294.921619	5967.296904
**ENSG00000129353**	SLC44A2	6696.470756	4883.466474	7162.809631	7326.631696	6842.270574	8388.130566	4433.873676	3988.811639	5181.565905	4519.930057	4265.73675	4811.053833
**ENSG00000129354**	AP1M2	1193.889893	879.3042217	1134.226875	1251.664311	1328.001502	1401.056815	987.6885152	1088.159777	1284.957062	1058.636845	1091.267066	1297.495883
**ENSG00000129355**	CDKN2D	42.18692202	84.07689769	39.89742775	72.8393278	63.56839215	68.28871559	41.87717593	41.5328159	37.98595695	33.53721028	28.97169203	43.98291127
**ENSG00000129422**	MTUS1	125.1545353	210.1922442	113.1784175	176.6563008	343.2693176	137.7155764	51.06972674	132.9050109	50.6479426	69.60553076	142.0992514	51.59533822
**ENSG00000129437**	KLK14	1.406230734	0	2.442699658	10.88403749	4.623155793	7.208253313	0	3.322625272	3.28273702	0	0	6.766601734
**ENSG00000129450**	SIGLEC9	0	0	0	0.837233653	1.155788948	0.379381753	4.08557814	0	0.937924863	4.429442867	4.138813147	2.53747565
**ENSG00000129451**	KLK10	237.6529941	434.3973047	385.1323128	103.816973	205.7304328	166.5485897	4.08557814	6.645250545	14.53783538	0	2.759208765	5.074951301

**ENSG00000129455**	KLK8	142.0293041	196.1794279	139.2338805	144.0041883	153.7199301	141.509394	5.106972674	19.93575163	10.78613592	11.38999594	4.138813147	10.99572782
**ENSG00000129460**	NGDN	395.1508363	441.4037129	375.3615142	380.9413121	335.178795	341.0641962	418.7717593	461.8449128	407.5283529	367.0109804	415.2609191	467.7413449
**ENSG00000129465**	RIPK3	0	0	5.699632536	0.837233653	2.311577897	0.758763507	15.32091802	29.90362745	7.972361335	10.12444084	13.79604382	15.2248539
**ENSG00000129467**	ADCY4	0	0	0	1.674467306	1.155788948	0.379381753	4.08557814	0	2.344812157	6.960553076	0	1.691650434
**ENSG00000129472**	RAB2B	310.7769922	500.9581821	366.4049488	247.8211612	206.8862217	273.5342441	264.5411845	230.9224564	266.3706611	246.1504679	154.5156908	261.359992
**ENSG00000129473**	BCL2L2	511.8679872	381.8492437	571.5917201	478.0604158	429.9534888	547.8272518	594.4516193	639.6053649	619.4993719	590.3814564	529.7680828	650.4395917
**ENSG00000129474**	AJUBA	1752.163495	858.2849973	1717.21786	1321.154704	995.1342845	1350.978424	1953.927745	1905.525594	2602.741495	1635.729973	1507.90759	1980.076832
**ENSG00000129480**	DTD2	70.31153671	73.56728548	81.42332194	107.1659076	67.035759	92.56914781	210.4072742	81.40431917	217.5985682	166.4204963	85.5354717	192.0023242
**ENSG00000129484**	PARP2	507.649295	458.9197332	561.0066882	632.9486416	601.0102531	577.0396468	660.8422641	819.0271296	602.147762	563.1720216	724.2923007	676.6601734
**ENSG00000129493**	HEATR5A	258.7464551	318.7915704	258.1119306	300.5668814	279.7009255	288.7095143	492.3121658	332.2625272	549.1550072	522.0414807	307.6517773	606.4566804
**ENSG00000129515**	SNX6	1147.484279	1296.185506	1152.954239	867.3740644	818.2985754	827.431604	1171.539532	1292.501231	1158.337206	985.8674266	1169.904516	1152.013945
**ENSG00000129518**	EAPP	341.7140684	311.7851623	397.3458111	298.0551804	323.6209055	263.2909368	505.5902948	559.8623584	502.7277265	526.4709236	484.2411382	455.0539666
**ENSG00000129521**	EGLN3	895.7689777	3909.575743	742.5806961	1011.378253	3393.396352	908.6192992	646.5427406	5502.267451	919.1663656	779.5819445	5361.14263	1052.20657
**ENSG00000129534**	MIS18BP1	1430.136657	1166.566955	1143.997673	1450.92592	1257.498376	1221.609246	2030.532335	1526.746313	1813.00876	1952.118749	1793.485697	2084.959159
**ENSG00000129535**	NRL	4.218692202	0	11.39926507	12.55850479	9.246311586	10.24330734	19.40649616	21.59706427	26.73085859	28.47498986	17.93485697	8.458252168
**ENSG00000129538**	RNASE1	37.96822982	59.5544692	58.6247918	13.39573845	26.58314581	28.83301325	24.51346884	129.5823856	59.08926636	10.12444084	26.21248326	20.2998052
**ENSG00000129559**	NEDD8	364.2137601	329.3011826	322.4363549	284.659442	310.9072271	339.1672875	325.8248566	305.681525	370.0113584	320.1854415	278.6800852	356.9382415
**ENSG00000129562**	DAD1	727.0212896	742.6792629	802.8339544	704.9507357	716.5891479	832.3635668	570.959545	677.8155555	760.6570638	664.41643	735.3291358	732.4846377
**ENSG00000129566**	TEP1	936.5496689	1285.675894	1008.020726	614.5295012	785.9364848	701.0974801	787.4951864	1061.578774	755.0295146	604.3025625	925.7145405	526.9491101
**ENSG00000129595**	EPB41L4A	5.624922937	10.50961221	4.885399317	0.837233653	8.090522638	0.758763507	6.128367209	11.62918845	7.972361335	5.694997971	6.898021912	8.458252168
**ENSG00000129596**	CDO1	1.406230734	0	4.885399317	0.837233653	1.155788948	1.13814526	0	1.661312636	3.751699452	6.960553076	4.138813147	4.229126084
**ENSG00000129625**	REEP5	888.737824	781.2145077	785.7350568	1193.895189	1159.256315	1073.27098	1252.2297	1182.854597	1270.888189	1163.045141	1161.62689	1374.465977
**ENSG00000129636**	ITFG1	922.4873616	1145.547731	1026.74809	967.8421027	1094.532134	1048.611166	1096.97773	1141.321781	1082.834254	1028.8963	1045.740122	1152.013945
**ENSG00000129646**	QRICH2	70.31153671	70.06408141	68.39559043	52.74572013	99.39784955	72.08253313	52.09112128	63.12988017	59.55822879	59.48108992	52.42496653	56.67028952
**ENSG00000129657**	SEC14L1	811.3951336	732.1696507	792.2489225	707.4624367	714.27757	862.714107	487.2051931	810.7205664	582.4513399	437.2492887	638.756829	561.6279439
**ENSG00000129667**	RHBDF2	637.0225226	756.6920792	718.1536996	273.7754045	275.0777697	303.8847844	89.88271907	69.77513072	93.79248629	96.81496551	44.14734023	76.12426951
**ENSG00000129675**	ARHGEF6	1.406230734	17.51602035	14.65619795	15.9074394	6.93473369	8.725780326	15.32091802	6.645250545	13.13094808	12.65555105	26.21248326	20.2998052
**ENSG00000129680**	MAP7D3	586.3982161	367.8364274	549.6074231	788.674101	594.0755194	676.8170479	579.1307013	461.8449128	594.1754007	584.0536808	573.915423	714.7223082
**ENSG00000129682**	FGF13	1.406230734	0	0	0	3.467366845	0.758763507	3.064183605	1.661312636	6.096511609	3.163887762	0	4.229126084
**ENSG00000129691**	ASH2L	835.3010561	578.0286716	844.3598486	809.6049423	817.1427864	841.0893471	458.6061462	425.2960348	426.7558126	500.5270439	459.4082593	515.9533822
**ENSG00000129696**	TTI2	244.6841477	231.2114686	280.0962275	249.4956286	189.5493875	251.1507207	124.6101333	122.9371351	128.9646686	85.42496957	95.19270238	137.8695103
**ENSG00000129749**	CHRNA10	26.71838395	7.006408141	19.54159727	15.9074394	26.58314581	25.41857747	16.34231256	21.59706427	22.51019671	22.14721433	23.4532745	22.83728085
**ENSG00000129757**	CDKN1C	0	0	0.814233219	1.674467306	3.467366845	0	8.171156279	3.322625272	0.937924863	6.960553076	0	1.691650434
**ENSG00000129810**	SGO1	559.6798322	441.4037129	529.2515926	609.5060993	567.4923736	630.532474	796.6877372	917.0445751	832.4083158	861.8430263	936.7513756	1078.427151
**ENSG00000129910**	CDH15	12.65607661	17.51602035	5.699632536	0	0	1.13814526	0	0	0	0	0	0
**ENSG00000129911**	KLF16	420.4629895	420.3844885	392.4604118	388.4764149	375.6314082	503.4395866	247.1774774	227.5998311	278.0947219	213.8788127	238.6715581	251.2100894
**ENSG00000129925**	TMEM8A	206.7159179	234.7146727	283.3531604	231.0764882	324.7766945	369.138446	208.3644851	176.0991394	288.8808578	241.0882475	260.7452283	274.0473702
**ENSG00000129932**	DOHH	92.81122845	73.56728548	96.07951989	92.93293547	102.8652164	140.3712487	34.72741419	43.19412854	66.12370283	59.48108992	55.18417529	79.50757038
**ENSG00000129933**	MAU2	506.2430643	469.4293454	605.7895153	450.4317052	480.8082025	525.8231101	563.8097833	669.5089924	533.2102846	597.974787	594.6094888	497.3452275
**ENSG00000129968**	ABHD17A	313.5894537	189.1730198	167.7320432	271.2637035	238.0925233	256.841447	241.0491102	176.0991394	224.6330047	208.8165923	187.626196	237.6768859
**ENSG00000129990**	SYT5	271.4025317	189.1730198	187.2736405	272.1009372	299.3493376	236.3548323	28.59904698	26.58100218	25.3239713	30.37332251	13.79604382	20.2998052
**ENSG00000129991**	TNNI3	16.87476881	31.52883663	8.956565414	17.58190671	17.33683422	10.24330734	1.021394535	1.661312636	0.937924863	0.632777552	0	0
**ENSG00000130005**	GAMT	8.437384405	38.53524477	23.61276336	18.41914036	23.11577897	26.55672273	2.04278907	8.306563181	0.937924863	5.062220419	8.277626294	7.612426951
**ENSG00000130021**	PUDP	374.0573753	311.7851623	390.8319453	485.5955187	446.134534	491.2993705	574.0237286	591.4272985	529.4585851	623.9186666	511.8332258	581.0819239
**ENSG00000130023**	ERMARD	374.0573753	259.2371012	402.2312104	298.0551804	322.4651166	325.8889261	701.6980455	588.1046732	602.6167244	623.9186666	560.1193792	537.0990127
**ENSG00000130024**	PHF10	795.9265955	1135.038119	738.50953	989.6101777	1204.332084	951.4894373	1649.552174	2272.675686	1508.18318	1785.065475	2539.851668	1695.87956
**ENSG00000130045**	NXNL2	7.031153671	0	2.442699658	3.348934611	9.246311586	7.587635066	3.064183605	1.661312636	5.627549177	1.265555105	4.138813147	5.920776517
**ENSG00000130052**	STARD8	23.90592248	87.58010176	31.75509556	1.674467306	4.623155793	10.24330734	16.34231256	3.322625272	11.25509835	30.37332251	17.93485697	8.458252168
**ENSG00000130054**	FAM155B	5.624922937	0	8.142332194	5.86063557	4.623155793	8.725780326	0	1.661312636	2.344812157	0	0	6.766601734
**ENSG00000130055**	GDPD2	97.02992066	94.5865099	88.75142092	29.30317785	18.49262317	31.48868552	338.081591	327.2785893	461.4590325	306.8971129	249.7083932	380.6213475
**ENSG00000130066**	SAT1	1267.013891	1646.505913	1309.287017	869.8857653	694.6291579	836.9161478	1221.587864	1498.503998	1260.571016	1125.078488	1005.731595	1184.155303
**ENSG00000130119**	GNL3L	2491.840861	1471.34571	2350.691305	3392.470761	2612.083023	3087.78809	2851.733541	1717.797266	2968.532191	3020.247258	2164.599276	3065.270586
**ENSG00000130147**	SH3BP4	666.553368	448.410121	603.3468156	725.881577	621.8144542	679.0933384	58.21948849	152.8407625	118.6474952	56.31720216	129.6828119	100.6532008
**ENSG00000130150**	MOSPD2	825.4574409	851.2785891	790.6204561	539.1784724	555.9344841	643.8108354	538.2749199	546.5718573	526.1758481	430.2887356	503.5555995	448.2873649
**ENSG00000130158**	DOCK6	188.4349184	136.6249587	168.5462764	154.8882258	187.2378096	172.9980795	9.192550814	44.85544118	15.00679781	13.92110615	19.31446135	16.07067912
**ENSG00000130159**	ECSIT	212.3408409	217.1986524	251.5980648	261.2168997	334.023006	342.2023415	249.2202665	207.6640795	268.7154732	281.5860108	205.561053	234.293585
**ENSG00000130164**	LDLR	1808.412724	2080.903218	2085.251275	1485.2525	1391.569894	1765.263298	627.1362444	1226.048725	932.7662762	595.4436768	1174.043329	692.7308525
**ENSG00000130165**	ELOF1	277.0274546	262.7403053	284.1673936	294.7062458	269.2988249	343.7198685	225.7281922	232.5837691	286.0670832	250.5799107	252.467602	275.7390207
**ENSG00000130175**	PRKCSH	2328.718096	2427.720421	2530.636846	2091.409665	2234.140037	2434.492711	1916.136147	2197.916618	2352.315556	2088.165923	2212.885429	2305.719541
**ENSG00000130177**	CDC16	2022.159796	2070.393606	2261.12565	1948.24271	2108.159042	2046.764559	1581.11874	1578.247004	1465.976561	1504.112242	1425.131327	1547.014321
**ENSG00000130193**	THEM6	97.02992066	73.56728548	110.7357178	197.5871421	224.223056	207.9012008	177.7226491	189.3896405	260.2741495	258.8060189	220.7367012	250.3642642
**ENSG00000130202**	NECTIN2	330.4642225	332.8043867	468.9983344	242.7977593	191.8609654	304.6435479	265.5625791	204.3414542	322.1771904	232.2293617	216.597888	208.9188285
**ENSG00000130203**	APOE	1.406230734	0	1.628466439	3.348934611	4.623155793	3.793817533	0	0	0	0	1.379604382	0
**ENSG00000130204**	TOMM40	549.8362171	406.3716722	454.3421365	622.0646041	538.5976499	662.779923	393.2368959	289.0683987	489.5967784	477.1142745	353.1787219	485.5036744
**ENSG00000130208**	APOC1	0	0	8.142332194	3.348934611	8.090522638	1.13814526	3.064183605	0	2.813774589	0	0	0
**ENSG00000130222**	GADD45G	2.812461468	14.01281628	6.513865756	3.348934611	13.86946738	11.00207085	30.64183605	24.91968954	8.910286198	36.06832049	19.31446135	23.68310607
**ENSG00000130224**	LRCH2	16.87476881	77.07048955	55.36785892	0	0	1.517527013	9.192550814	6.645250545	2.813774589	1.265555105	2.759208765	0.845825217

**ENSG00000130226**	DPP6	178.5913032	367.8364274	387.5750125	262.891367	614.8797205	500.0251509	0	1.661312636	0.468962431	1.898332657	1.379604382	0
**ENSG00000130227**	XPO7	1936.379721	1828.672525	2051.05348	2111.503273	2165.948489	2205.346132	996.881066	838.9628812	981.0694066	949.1663286	938.13098	986.2322028
**ENSG00000130234**	ACE2	11.24984587	14.01281628	15.47043117	0.837233653	1.155788948	4.173199286	35.74880872	24.91968954	38.92388181	13.92110615	8.277626294	9.304077385
**ENSG00000130244**	FAM98C	26.71838395	24.52242849	35.82626166	65.30422492	67.035759	73.60006014	56.17669942	43.19412854	62.37200338	52.52053685	60.70259282	48.21203736
**ENSG00000130254**	SAFB2	718.5839052	805.7369362	859.0160465	713.3230722	783.6249069	724.9985306	806.9016826	943.6255773	764.8777257	713.1403015	961.5842545	861.8958959
**ENSG00000130255**	RPL36	2542.465167	2778.040828	2336.84934	2425.465892	2353.186299	2324.092621	1979.462609	2023.478791	2170.827095	1943.259863	1890.058004	2094.263237
**ENSG00000130270**	ATP8B3	0	7.006408141	3.256932878	5.86063557	3.467366845	6.449489806	1.021394535	1.661312636	0.468962431	1.265555105	0	2.53747565
**ENSG00000130287**	NCAN	1.406230734	0	7.328098975	0.837233653	8.090522638	3.41443578	0	0	1.406887294	3.163887762	0	0.845825217
**ENSG00000130294**	KIF1A	1.406230734	14.01281628	0.814233219	0	1.155788948	1.13814526	0	0	0	0.632777552	0	0
**ENSG00000130299**	GTPBP3	160.3103037	108.5993262	149.8189124	220.1924507	162.9662417	210.9362548	224.7067977	144.5341993	201.6538455	207.5510372	222.1163056	164.9359173
**ENSG00000130303**	BST2	1610.134191	1534.403383	1592.640177	229.4020209	322.4651166	287.1919873	1068.378683	1347.324548	1087.054916	307.5298905	372.4931832	249.5184389
**ENSG00000130304**	SLC27A1	122.3420739	70.06408141	62.6959579	108.8403749	107.4883722	110.0207085	288.0332588	398.7150327	417.376564	294.2415619	346.2807	380.6213475
**ENSG00000130305**	NSUN5	358.5888372	213.6954483	279.2819943	300.5668814	238.0925233	292.8827136	249.2202665	209.3253922	295.9152942	242.3538026	217.9774924	284.1972728
**ENSG00000130307**	USHBP1	0	0	0	0.837233653	0	2.27629052	0	0	1.406887294	0	0	0
**ENSG00000130309**	COLGALT1	1206.54597	1100.006078	1319.872049	1562.277996	1692.07502	1857.453064	1595.418263	1422.083617	1968.704287	1872.388777	1885.919191	2101.029838
**ENSG00000130311**	DDA1	652.4910606	651.5959571	757.2368941	640.4837444	692.31758	746.2439087	528.0609745	729.3162473	516.7965995	483.44205	671.8673342	537.9448379
**ENSG00000130312**	MRPL34	164.5289959	185.6698157	174.245909	231.0764882	269.2988249	256.841447	180.7868327	229.2611438	229.7915914	191.0988208	187.626196	221.6062068
**ENSG00000130313**	PGLS	323.4330689	332.8043867	265.4400295	344.940265	293.5703929	314.8868552	291.0974424	274.116585	318.8944534	282.8515659	274.5412721	275.7390207
**ENSG00000130332**	LSM 7.00	253.1215321	192.6762239	204.3725381	221.866918	246.183046	260.6352645	242.0705048	206.0027669	275.2809473	229.6982515	215.2182836	285.0430981
**ENSG00000130338**	TULP4	1918.098721	1457.332893	1838.53861	1969.173552	1946.348589	2240.628635	2239.918215	1722.781204	2198.495879	2080.572592	1865.225125	2156.008478
**ENSG00000130340**	SNX9	2563.558628	4428.049945	3043.603774	2031.128842	2582.03251	2370.377195	831.4151514	1186.177222	891.9665446	786.5424976	1074.711814	816.2213342
**ENSG00000130347**	RTN4IP1	181.4037647	150.637775	176.6886086	206.7967123	194.1725433	190.8290219	169.5514928	134.5663235	166.9506256	172.7482718	131.0624163	189.4648486
**ENSG00000130348**	QRSL1	750.927212	676.1183856	802.8339544	906.7240461	753.5743943	835.019239	744.5966159	824.0110675	693.5954361	715.0386342	623.5811808	841.5960907
**ENSG00000130349**	C6orf203	208.1221487	210.1922442	157.1470114	216.0062824	182.6146538	180.9650963	192.0221726	136.2276362	176.3298742	188.5677106	184.8669872	199.6147512
**ENSG00000130363**	RSPH3	597.648062	1012.425976	661.9716074	648.0188473	891.1132791	676.4376661	745.6180105	1001.77152	625.5958836	681.5014239	836.0402557	663.9727952
**ENSG00000130382**	MLLT1	756.552135	627.0735286	739.3237633	815.4655779	791.7154296	829.3285127	746.639405	556.5397331	814.118781	793.5030507	780.8560804	847.5168672
**ENSG00000130396**	AFDN	4902.120339	5920.414879	5125.598116	4365.336266	4989.54089	4791.591544	3845.550424	4462.285741	3560.831742	3765.026437	4307.124882	3586.298919
**ENSG00000130402**	ACTN4	6077.729233	5121.684351	6520.379621	6500.282081	5844.824711	7589.911357	4818.939416	4420.752925	5336.79247	4735.707202	4222.969014	4736.621214
**ENSG00000130413**	STK33	2.812461468	0	6.513865756	6.697869223	1.155788948	3.035054026	0	0	3.751699452	3.796665314	1.379604382	1.691650434
**ENSG00000130414**	NDUFA10	902.8001313	830.2593647	946.9532342	968.6793364	821.7659422	957.9389271	1106.170281	1043.304335	1086.116991	1238.978448	1175.422934	1120.718412
**ENSG00000130427**	EPO	1.406230734	0	3.256932878	0	0	0.379381753	2.04278907	0	0.937924863	0	0	0
**ENSG00000130429**	ARPC1B	30.93707615	45.54165292	39.89742775	34.32657977	33.5178795	31.10930377	24.51346884	19.93575163	25.3239713	15.18666126	38.6289227	20.2998052
**ENSG00000130449**	ZSWIM6	194.0598413	294.2691419	298.8235915	153.2137585	239.2483123	262.1527915	93.96829721	88.04956971	121.4612697	89.22163489	53.80457091	75.27844429
**ENSG00000130475**	FCHO1	254.5277629	150.637775	197.0444391	217.6807497	181.4588649	210.9362548	80.69016826	31.56494009	110.6751338	120.8605125	45.52694462	98.96155036
**ENSG00000130477**	UNC13A	21.09346101	122.6121425	32.56932878	38.51274803	40.45261319	56.907263	15.32091802	4.983937908	15.00679781	13.92110615	17.93485697	14.37902869
**ENSG00000130479**	MAP1S	241.8716863	318.7915704	261.3688634	241.9605257	224.223056	281.8806427	121.5459497	204.3414542	185.7091229	146.1716146	186.2465916	130.2570834
**ENSG00000130487**	KLHDC7B	869.0505937	1257.650261	1157.025405	15.9074394	27.73893476	19.34846942	108.2678207	126.2597603	174.4540245	5.694997971	2.759208765	10.1499026
**ENSG00000130508**	PXDN	2.812461468	21.01922442	10.58503185	6.697869223	9.246311586	13.27836137	4.08557814	9.967875817	6.56547404	8.858885733	9.657230676	5.074951301
**ENSG00000130511**	SSBP4	428.9003739	343.3139989	368.8476484	365.8711063	414.9282324	334.6147064	267.6053681	244.2129575	375.1699452	283.4843435	246.9491844	312.9553302
**ENSG00000130513**	GDF15	912.6437465	1107.012486	1104.914479	465.501911	648.3976	824.3965499	306.4183605	330.6012146	437.5419485	245.5176903	274.5412721	322.2594076
**ENSG00000130517**	PGPEP1	648.2723684	823.2529565	726.2960317	709.136904	954.6816713	798.9779725	935.5973939	958.577391	980.1314817	1061.800733	1094.026275	944.7867671
**ENSG00000130518**	IQCN	9.843615139	3.50320407	14.65619795	11.72127114	12.71367843	5.311344546	16.34231256	4.983937908	2.344812157	5.062220419	19.31446135	6.766601734
**ENSG00000130520**	LSM 4.00	1198.108586	1057.967629	1112.242578	1426.646144	1453.982497	1500.834216	1105.148887	1113.079466	1390.942572	1273.148435	1220.949878	1276.350252
**ENSG00000130522**	JUND	362.8075294	318.7915704	296.3808919	370.0572746	401.058765	338.4085239	674.120393	586.4433606	689.3747742	677.7047586	473.2043031	677.5059986
**ENSG00000130528**	HRC	1.406230734	3.50320407	6.513865756	5.86063557	0	0.758763507	2.04278907	1.661312636	6.56547404	4.429442867	4.138813147	1.691650434
**ENSG00000130529**	TRPM4	229.2156097	252.2306931	219.8429693	283.8222083	212.6651665	265.9466091	244.1132938	207.6640795	274.3430224	251.2126883	292.476129	262.2058172
**ENSG00000130544**	ZNF557	592.0231391	525.4806106	583.8052183	506.52636	396.4356093	443.8766514	597.5158029	591.4272985	568.3824669	565.7031318	524.2496653	658.8978439
**ENSG00000130545**	CRB3	253.1215321	245.2242849	255.6692309	180.0052354	172.2125533	261.394028	201.2147234	179.4217647	192.2745969	191.0988208	151.7564821	174.2399947
**ENSG00000130558**	OLFM1	0	3.50320407	0.814233219	0	0	0	0	1.661312636	2.813774589	0	0	4.229126084
**ENSG00000130559**	CAMSAP1	991.3926676	763.6984874	988.4791284	960.3069998	1058.702677	1014.84619	886.5704563	945.28689	869.9253103	1004.850753	911.9184967	1041.210842
**ENSG00000130560**	UBAC1	885.9253625	798.7305281	846.8025482	824.6751481	855.2838217	782.6645571	701.6980455	747.5906863	707.6643091	818.8141528	716.0146744	777.3133742
**ENSG00000130584**	ZBTB46	11.24984587	10.50961221	12.21349829	3.348934611	10.40210053	6.449489806	2.04278907	1.661312636	4.689624315	0	2.759208765	10.1499026
**ENSG00000130589**	HELZ2	2050.28441	2396.191584	1901.234567	632.9486416	714.27757	741.311946	435.1140719	476.7967266	514.9207497	97.44774307	136.5808338	71.04931821
**ENSG00000130590**	SAMD10	99.84238213	112.1025303	99.33645277	108.0031412	110.955739	144.1650663	54.13391035	64.79119281	52.05482989	72.76941852	45.52694462	74.43261908
**ENSG00000130592**	LSP1	19.68723028	38.53524477	14.65619795	30.1404115	39.29682424	15.93403364	3.064183605	0	8.910286198	3.163887762	0	5.074951301
**ENSG00000130595**	TNNT3	343.1202991	318.7915704	179.1313083	456.2923408	402.214554	224.2146162	1547.41272	2859.119047	1005.924415	1487.027248	2503.981954	897.420555
**ENSG00000130598**	TNNI2	188.4349184	157.6441832	78.16638907	226.8903199	205.7304328	118.367107	403.4508413	425.2960348	341.8736125	445.4753969	398.7056665	262.2058172
**ENSG00000130600**	H19	4966.806953	2214.024973	2571.348507	7610.453905	5220.698679	5223.707361	12527.40397	19311.09808	15310.68546	13503.47297	19637.28878	14465.30286
**ENSG00000130612**	CYP2G1P	0	0	3.256932878	3.348934611	1.155788948	1.896908767	0	3.322625272	0	1.265555105	0	0
**ENSG00000130635**	COL5A1	25.31215321	63.05767327	125.3919158	72.8393278	314.3745939	328.9239801	1482.04347	4241.33116	1764.70563	1738.239936	4872.762678	2165.312555
**ENSG00000130638**	ATXN10	753.7396735	728.6664466	737.6952968	840.5825875	698.0965248	795.1841549	1100.041914	991.8036438	1029.372537	1075.721839	1008.490803	1146.093169
**ENSG00000130640**	TUBGCP2	800.1452877	732.1696507	828.8894174	944.3995604	883.0227565	923.4151875	560.7455996	465.1675381	628.8786206	515.0809276	529.7680828	559.9362935
**ENSG00000130649**	CYP 20.00	9.843615139	0	5.699632536	2.511700959	2.311577897	2.27629052	2.04278907	0	1.406887294	3.163887762	4.138813147	1.691650434
**ENSG00000130653**	PNPLA7	7.031153671	7.006408141	15.47043117	12.55850479	10.40210053	17.83094241	8.171156279	14.95181373	10.31717349	8.858885733	12.41643944	2.53747565
**ENSG00000130669**	PAK4	314.9956845	287.2627338	289.0527929	405.221088	324.7766945	364.2064832	251.2630556	224.2772059	276.2188721	258.8060189	229.0143275	299.4221267
**ENSG00000130675**	MNX1	119.5296124	115.6057343	77.35215585	124.7478143	154.8757191	130.1279414	58.21948849	137.8889488	80.19257578	123.3916227	142.0992514	87.96582254
**ENSG00000130684**	ZNF337	517.4929102	581.5318757	588.6906177	425.3146957	686.5386353	569.8313935	630.200428	614.6856754	600.2719123	635.9414401	648.4140597	561.6279439

**ENSG00000130695**	CEP85	916.8624387	805.7369362	993.3645277	1077.519711	1022.873219	1174.94529	689.441311	659.5411165	818.8084053	729.5925179	671.8673342	911.7995837
**ENSG00000130699**	TAF4	427.4941432	339.8107948	422.5870409	409.4072563	414.9282324	472.3302829	496.3977439	423.6347222	562.2859553	551.1492481	491.1391601	547.2489153
**ENSG00000130701**	RBBP8NL	2.812461468	7.006408141	4.071166097	14.2329721	46.23155793	12.89897961	30.64183605	41.5328159	25.3239713	48.72387153	82.77626294	21.14563042
**ENSG00000130702**	LAMA5	3749.011137	2683.454318	2808.290374	3646.152558	3911.189801	3832.893854	4417.531363	3792.776748	5063.856335	4835.686055	4697.552922	4531.085686
**ENSG00000130703**	OSBPL2	1269.826353	1558.925811	1504.70299	1139.475002	1195.085773	1183.291689	1008.116406	1000.110207	1087.992841	1072.557951	1153.349264	1065.739773
**ENSG00000130706**	ADRM1	1317.638198	1551.919403	1562.513548	1192.220722	1162.723682	1562.29406	819.158417	785.8008769	1051.882734	877.6624652	757.4028059	937.1743402
**ENSG00000130707**	ASS1	177.1850725	185.6698157	200.301372	73.67656145	129.4483622	88.01656677	126.6529223	86.38825708	82.53738794	31.00610007	46.906549	35.5246591
**ENSG00000130713**	EXOSC2	1366.856274	956.3747112	1241.70566	1383.109995	1132.673169	1332.768099	944.7899448	711.0418083	899.0009811	1058.004068	829.1422338	1047.977444
**ENSG00000130714**	POMT1	322.0268381	378.3460396	381.8753799	477.2231821	465.7829462	520.5117655	432.0498883	544.9105446	390.6457054	515.0809276	710.4962569	444.904064
**ENSG00000130717**	UCK1	407.8069129	357.3268152	343.6064186	314.7998535	387.1892977	399.4889862	353.4025091	443.5704738	400.9628789	358.1520946	440.093798	398.3836771
**ENSG00000130724**	CHMP2A	556.8673707	707.6472222	510.5242286	496.4795562	560.5576399	572.8664475	512.7400565	618.0083006	592.2995509	589.1159013	551.8417529	566.7028952
**ENSG00000130725**	UBE2M	683.4281368	655.0991612	662.7858406	788.674101	755.8859722	867.6460698	425.921521	441.9091612	456.7694082	524.5725909	409.7425015	559.0904683
**ENSG00000130726**	TRIM28	2362.467633	1975.807096	2279.853014	2501.654155	2472.23256	2798.319812	2050.960226	1983.607288	2502.852497	2412.780807	2094.239452	2438.5141
**ENSG00000130731**	METTL26	181.4037647	161.1473872	162.0324107	175.8190671	199.951488	172.9980795	99.07526988	88.04956971	148.6610908	152.4993901	115.8867681	117.5697051
**ENSG00000130733**	YIPF2	253.1215321	224.2050605	248.3411319	224.378619	236.9367344	296.6765311	288.0332588	265.8100218	332.4943639	323.3493293	322.8274255	290.9638746
**ENSG00000130734**	ATG4D	106.8735358	129.6185506	120.5065165	119.7244124	113.2673169	149.097029	111.3320043	91.37219499	105.9855095	120.227735	73.11903226	131.9487338
**ENSG00000130741**	EIF2S3	7768.018575	7665.010506	8165.130725	7934.463328	7954.139542	8252.69128	5762.707966	5625.204586	6138.718228	5450.745836	5904.706756	6274.331458
**ENSG00000130748**	TMEM160	14.06230734	3.50320407	9.770798633	19.25637402	26.58314581	30.72992202	12.25673442	14.95181373	22.04123428	25.94387965	22.07367012	27.06640694
**ENSG00000130749**	ZC3H4	1077.172742	952.8715072	1106.542945	1046.542066	978.9532392	989.0482309	885.5490617	789.1235022	831.0014285	845.39081	807.0685637	849.2085176
**ENSG00000130758**	MAP3K10	97.02992066	87.58010176	92.0083538	70.32762684	60.10102531	81.18769521	49.02693767	76.42038126	43.14454369	63.27775524	63.46180159	39.75378519
**ENSG00000130762**	ARHGEF16	289.6835312	329.3011826	292.3097258	395.1742842	362.9177298	388.4869154	292.118837	295.7136492	306.2324677	313.857666	329.7254474	307.0345537
**ENSG00000130764**	LRRC47	673.5845217	606.0543042	749.9087951	734.2539136	686.5386353	817.5676784	673.0989985	692.7673693	796.2982086	752.3725098	744.9863664	762.9343455
**ENSG00000130766**	SESN2	354.370145	318.7915704	542.2793242	151.5392912	153.7199301	317.1631458	349.3169309	176.0991394	383.6112689	275.8910128	262.1248326	230.064459
**ENSG00000130768**	SMPDL3B	7.031153671	17.51602035	17.91313083	6.697869223	16.18104528	13.27836137	1.021394535	0	2.344812157	0	0	0
**ENSG00000130770**	ATP5IF1	787.4892111	833.7625688	692.0982365	955.2835979	937.344837	927.2090051	672.0776039	797.4300653	808.0222694	821.9780405	873.289574	887.2706524
**ENSG00000130772**	MED18	345.9327606	367.8364274	338.7210193	270.4264699	321.3093276	268.9816631	337.0601965	367.1500926	330.1495517	332.8409926	340.7622824	337.4842615
**ENSG00000130775**	THEMIS2	73.12399818	150.637775	87.9371877	29.30317785	26.58314581	24.65981396	5.106972674	11.62918845	4.689624315	0	4.138813147	0.845825217
**ENSG00000130779**	CLIP1	4213.06728	4140.787211	3966.130012	2982.226272	2649.068269	3015.326175	6620.679375	9235.236944	6096.511609	6000.629529	5751.57067	5733.849145
**ENSG00000130783**	CCDC62	11.24984587	14.01281628	10.58503185	8.372336529	9.246311586	9.105162079	11.23533988	24.91968954	7.503398903	10.12444084	4.138813147	10.99572782
**ENSG00000130787**	HIP1R	705.9278285	662.1055693	721.4106324	689.0432963	618.3470873	737.8975102	793.6235536	767.5264379	926.6697646	878.9280203	856.7343214	758.7052194
**ENSG00000130803**	ZNF317	568.1172166	518.4742024	658.7146745	587.7380243	525.8839715	663.1593048	626.1148499	671.170305	586.6720017	606.2008952	704.9778394	619.1440587
**ENSG00000130810**	PPAN	85.78007478	21.01922442	68.39559043	80.37443068	42.76419109	51.9753002	63.32646116	46.51675381	43.14454369	56.31720216	62.0821972	43.98291127
**ENSG00000130811**	EIF3G	1323.263121	1047.458017	1422.465434	1354.64405	1418.15304	1421.164048	1024.458718	913.7219499	1138.640784	961.8218796	1100.924297	1147.784819
**ENSG00000130812**	ANGPTL6	0	0	1.628466439	0	2.311577897	1.517527013	6.128367209	0	0.468962431	0.632777552	0	3.383300867
**ENSG00000130813**	C19orf66	458.4312193	511.4677943	434.8005392	166.6094969	247.3388349	213.2125454	469.841486	446.8930991	362.0389971	246.7832454	229.0143275	187.7731981
**ENSG00000130816**	DNMT1	2811.055238	3068.806766	2797.705342	3018.227319	2825.903979	2871.161109	3138.745406	3563.515604	3457.660007	3610.628714	3698.719349	3665.80649
**ENSG00000130818**	ZNF426	638.4287533	700.6408141	745.837629	615.3667349	610.2565647	609.6664776	834.479335	817.365817	824.4359545	775.7852792	717.3942788	735.0221134
**ENSG00000130821**	SLC6A8	9.843615139	136.6249587	35.82626166	41.86168264	105.1767943	40.5938476	92.94690267	593.0886111	62.84096581	61.37942258	434.5753804	21.14563042
**ENSG00000130822**	PNCK	0	0	0	0.837233653	0	0	0	6.645250545	1.406887294	1.265555105	5.518417529	0
**ENSG00000130826**	DKC1	1563.728576	830.2593647	1348.370211	1657.722633	1143.07527	1547.877553	1170.518137	744.268061	1331.853305	1328.200082	762.9212234	1424.369665
**ENSG00000130827**	PLXNA3	151.8729193	238.2178768	142.4908134	190.8892729	265.8314581	165.0310627	26.55625791	76.42038126	30.95152048	55.05164706	66.22101035	32.98718345
**ENSG00000130829**	DUSP9	2.812461468	0	6.513865756	1.674467306	23.11577897	6.449489806	84.77574639	124.5984477	89.57182441	113.8999594	131.0624163	93.04077385
**ENSG00000130830**	MPP1	53.4367679	59.5544692	66.76712399	53.58295378	67.035759	55.76911774	107.2464262	84.72694444	96.60626088	108.837739	96.57230676	87.11999733
**ENSG00000130844**	ZNF331	556.8673707	462.4229373	394.9031114	756.8592222	537.4418609	552.3798328	2270.560051	2026.801416	1809.257061	2525.415212	2310.83734	1966.543629
**ENSG00000130856**	ZNF236	683.4281368	634.0799367	664.4143071	517.4103975	581.361841	544.0334342	694.5482837	682.7994934	627.4717333	614.4270034	790.5133111	641.1355143
**ENSG00000130881**	LRP3	0	0	0.814233219	2.511700959	4.623155793	7.208253313	2.04278907	4.983937908	2.344812157	0	1.379604382	0
**ENSG00000130921**	C12orf65	261.5589166	189.1730198	279.2819943	236.0998901	227.6904228	268.6022813	321.7392785	358.8435294	357.3493728	359.4176498	357.317535	368.7797945
**ENSG00000130935**	NOL11	1313.419506	872.2978135	1257.176091	1398.1802	1030.963742	1277.757745	1033.651269	913.7219499	1026.0898	1003.585198	765.6804322	1115.643461
**ENSG00000130939**	UBE4B	1777.475648	1569.435424	1842.609776	2376.90634	2349.718932	2320.298803	2333.886512	2526.85652	2295.571102	2648.174057	2905.446829	2527.325748
**ENSG00000130940**	CASZ1	653.8972914	599.047896	634.2876779	419.4540601	354.8272071	408.9735301	105.2036371	117.9531972	107.8613592	32.90443272	40.00852709	78.66174516
**ENSG00000130956**	HABP4	223.5906867	150.637775	249.1553652	280.4732737	283.1682923	321.7157268	337.0601965	239.2290196	301.5428434	272.7271251	280.0596896	321.4135824
**ENSG00000130957**	FBP2	0	0	2.442699658	0	8.090522638	1.896908767	0	0	0	0	0	0
**ENSG00000130958**	SLC35D2	157.4978422	108.5993262	96.89375311	97.95633739	87.83996007	81.94645871	86.81853546	116.2918845	81.13050064	70.23830831	68.98021912	78.66174516
**ENSG00000130962**	PRRG1	898.5814391	1191.089384	1108.171412	790.3485683	829.8564649	896.8584648	523.9753964	591.4272985	557.596331	365.1126477	438.7141936	484.6578492
**ENSG00000130985**	UBA1	3955.727055	4088.23915	3894.477489	3858.809906	4097.271822	4436.490223	2754.701061	3080.073627	3202.544444	2897.488412	3333.124188	2995.067093
**ENSG00000130988**	RGN	0	3.50320407	1.628466439	0.837233653	5.778944741	4.173199286	3.064183605	1.661312636	3.28273702	1.898332657	1.379604382	5.920776517
**ENSG00000130997**	POLN	2.812461468	0	2.442699658	1.674467306	1.155788948	0	0	0	3.28273702	1.898332657	0	0
**ENSG00000131013**	PPIL4	800.1452877	809.2401403	802.0197212	746.8124184	752.4186053	708.6851152	932.5332103	1069.885338	880.2424838	882.7246856	1055.397352	913.4912341
**ENSG00000131015**	ULBP2	44.99938349	98.08971397	47.22552673	91.25846816	93.61890481	92.56914781	6.128367209	11.62918845	10.31717349	6.960553076	12.41643944	19.45397999
**ENSG00000131016**	AKAP12	21.09346101	14.01281628	42.34012741	18.41914036	8.090522638	14.41650663	37.79159779	36.54887799	141.1576919	28.47498986	26.21248326	186.0815477
**ENSG00000131018**	SYNE1	1444.198964	991.4067519	1765.25762	2469.839276	2480.323083	2937.173534	853.8858312	1169.564096	964.6557215	658.0886545	939.5105844	903.3413315
**ENSG00000131019**	ULBP3	167.3414574	115.6057343	119.6922833	379.2668447	258.8967244	302.3672574	26.55625791	33.22625272	38.45491938	32.27165517	44.14734023	43.13708606
**ENSG00000131023**	LATS1	1756.382187	1863.704565	1789.684616	1804.238522	1620.416105	1785.370531	1958.013323	1940.413159	1864.125665	1709.132169	1818.318576	1985.997609
**ENSG00000131037**	EPS8L1	299.5271464	402.8684681	318.3651888	344.940265	317.8419608	410.1116753	52.09112128	112.9692593	91.91663656	31.63887762	110.3683506	34.67883389
**ENSG00000131043**	AAR2	528.742756	416.8812844	602.5325824	546.7135753	439.1998003	558.4499409	640.4143734	586.4433606	635.4440946	655.5575443	573.915423	640.2896891
**ENSG00000131044**	TTLL9	1.406230734	0	0.814233219	0	6.93473369	0.758763507	5.106972674	9.967875817	0.937924863	1.898332657	1.379604382	0
**ENSG00000131051**	RBM39	5975.074389	5605.126513	5845.380282	5282.94435	4520.290577	5084.85364	8125.193525	7422.744858	7622.046398	7866.690531	7575.407663	7592.127146

**ENSG00000131061**	ZNF341	115.3109202	119.1089384	106.6645517	113.0265431	75.12628164	114.1939077	130.7385005	96.3561329	131.3094808	134.1488411	120.0255813	108.2656277
**ENSG00000131067**	GGT7	129.3732275	189.1730198	130.2773151	113.8637768	125.9809954	120.6433976	320.717884	327.2785893	303.4186932	287.2810088	379.3912051	221.6062068
**ENSG00000131069**	ACSS2	434.5252969	651.5959571	494.2395642	339.0796294	466.9387351	405.938476	627.1362444	925.3511383	678.1196759	605.5681176	776.7172672	650.4395917
**ENSG00000131089**	ARHGEF9	74.53022891	10.50961221	33.383562	108.0031412	39.29682424	55.38973598	94.98969174	38.21019063	46.89624315	74.03497363	52.42496653	54.97863909
**ENSG00000131094**	C1QL1	12.65607661	7.006408141	3.256932878	10.88403749	16.18104528	7.587635066	1.021394535	4.983937908	5.627549177	2.53111021	1.379604382	5.074951301
**ENSG00000131096**	PYY	2.812461468	0	0.814233219	1.674467306	2.311577897	0.758763507	2.04278907	0	0	2.53111021	1.379604382	5.920776517
**ENSG00000131100**	ATP6V1E1	2338.561711	2536.319747	2770.021413	2086.386263	2193.687424	2465.981396	1136.812117	1098.127652	1275.108851	1204.80846	1158.867681	1361.778599
**ENSG00000131115**	ZNF227	236.2467633	227.7082646	258.1119306	251.1700959	221.9114781	196.89913	293.1402315	372.1340305	308.5772799	317.0215537	347.6603043	330.7176598
**ENSG00000131116**	ZNF428	42.18692202	56.05126513	33.383562	35.16381342	60.10102531	46.2845739	20.4278907	41.5328159	32.35840777	35.43554293	34.49010956	20.2998052
**ENSG00000131127**	ZNF141	341.7140684	430.8941007	345.234885	276.2871054	457.6924235	326.6476896	841.6290967	1020.045959	689.8437367	763.7625057	997.4539684	858.512595
**ENSG00000131143**	COX4I1	2463.716246	2911.162583	2803.404975	2746.126381	2680.274571	2775.177525	2360.44277	2726.214036	2330.274322	2453.278571	2640.562788	2667.732734
**ENSG00000131148**	EMC8	382.4947597	245.2242849	378.618447	465.501911	351.3598403	445.0147966	301.3113878	307.3428377	353.5976733	305.6315578	302.1333597	388.2337745
**ENSG00000131149**	GSE1	974.5178988	966.8843234	1126.084542	1226.547301	1303.729934	1355.531005	2050.960226	1531.730251	2136.592838	1986.921514	1782.448862	2111.179741
**ENSG00000131153**	GINS2	489.3682955	451.9133251	426.658207	656.3911838	633.3723436	650.6397069	464.7345134	510.0229793	523.3620735	491.0353806	484.2411382	521.8741588
**ENSG00000131165**	CHMP1A	840.925979	998.4131601	909.4985061	892.491074	948.9027265	962.8708899	721.1045416	714.3644335	872.2701225	770.7230588	830.5218381	773.9300734
**ENSG00000131171**	SH3BGRL	0	0	0	5.023401917	0	0.379381753	167.5087037	161.1473257	126.6198565	149.9682799	176.5893609	99.80737558
**ENSG00000131174**	COX7B	895.7689777	865.2914054	824.0040181	884.1187374	909.6059023	775.8356855	792.6021591	898.7701361	852.104738	935.878	931.2329581	900.8038559
**ENSG00000131183**	SLC34A1	5.624922937	0	0.814233219	5.023401917	11.55788948	1.517527013	1.021394535	6.645250545	6.096511609	11.38999594	8.277626294	3.383300867
**ENSG00000131187**	F12	52.03053716	28.02563256	16.28466439	66.97869223	57.78944741	59.94231702	7.149761744	14.95181373	27.66878346	31.63887762	20.69406573	27.91223215
**ENSG00000131188**	PRR7	7.031153671	21.01922442	4.885399317	15.07020575	12.71367843	18.21032416	4.08557814	8.306563181	8.910286198	8.226108181	0	10.99572782
**ENSG00000131196**	NFATC1	1.406230734	7.006408141	2.442699658	5.023401917	3.467366845	4.931962793	10.21394535	11.62918845	14.53783538	2.53111021	6.898021912	6.766601734
**ENSG00000131203**	IDO1	1251.545353	1278.669486	1959.859359	0	4.623155793	2.655672273	191.000778	210.9867048	249.0190511	0.632777552	0	0
**ENSG00000131236**	CAP1	9650.961529	10436.04493	10695.76757	10169.03995	9640.435617	10851.07691	5285.716718	6296.374891	5797.78254	5241.929244	6012.315898	6261.64408
**ENSG00000131238**	PPT1	3488.858451	3734.415539	3899.362888	3710.61955	3760.937238	3807.095894	4335.819801	5078.632729	4442.481113	4323.136238	4823.096921	4457.498892
**ENSG00000131242**	RAB11FIP4	490.7745262	392.3588559	426.658207	528.294435	543.2208057	465.5014113	753.7891667	744.268061	842.2565269	726.4286301	615.3035545	684.2726004
**ENSG00000131263**	RLIM	2643.71378	2683.454318	2416.644195	2376.069107	2147.455866	2221.659547	2293.030731	1868.976716	2112.206791	2162.200896	1818.318576	2240.590999
**ENSG00000131269**	ABCB7	397.9632978	339.8107948	324.8790546	452.1061725	473.8734688	396.0745504	459.6275407	415.328159	372.3561706	446.740952	478.7227207	465.2038692
**ENSG00000131323**	TRAF3	738.2711354	861.7882013	1008.834959	752.6730539	687.6944242	931.7615861	458.6061462	576.4754847	595.5822879	404.344856	452.5102374	563.3195944
**ENSG00000131351**	HAUS8	153.27915	161.1473872	184.8309408	219.3552171	177.991498	212.8331636	236.9635321	209.3253922	260.2741495	272.0943475	224.8755143	260.5141668
**ENSG00000131368**	MRPS25	524.5240638	332.8043867	394.0888782	543.3646407	362.9177298	407.8353848	678.2059712	538.2652941	658.8922162	670.7442055	573.915423	636.9063882
**ENSG00000131370**	SH3BP5	91.40499772	262.7403053	145.7477463	133.9573845	261.2083023	164.2722992	37.79159779	395.3924074	24.38604644	32.90443272	293.8557334	29.60388259
**ENSG00000131373**	HACL1	234.8405326	203.1858361	201.1156052	195.9126748	186.0820207	255.7033017	310.5039386	320.6333388	346.5632368	353.7226518	286.9577115	300.267952
**ENSG00000131374**	TBC1D5	918.2686694	1089.496466	866.3441455	825.5123817	940.8122039	832.7429485	1726.156764	1857.347527	1568.210371	1638.893861	1844.531059	1663.738201
**ENSG00000131375**	CAPN7	748.1147506	739.1760589	742.5806961	829.69855	968.5511386	854.7470902	1211.373918	1292.501231	1192.102501	1170.005694	1191.978186	1261.125398
**ENSG00000131378**	RFTN1	36.56199909	80.57369362	39.89742775	28.4659442	43.91998003	31.10930377	9.192550814	16.61312636	4.220661883	6.327775524	13.79604382	3.383300867
**ENSG00000131379**	C3orf20	0	14.01281628	8.142332194	4.186168264	0	4.55258104	2.04278907	4.983937908	3.28273702	1.265555105	2.759208765	1.691650434
**ENSG00000131381**	RBSN	805.7702107	840.7689769	887.5142092	740.1145491	733.9259821	814.5326243	1092.892152	1098.127652	1069.703306	1074.456284	1103.683506	1014.144435
**ENSG00000131386**	GALNT15	0	3.50320407	5.699632536	0.837233653	0	0	2.04278907	0	2.813774589	0	2.759208765	1.691650434
**ENSG00000131389**	SLC6A6	2546.68386	2869.124134	2126.777169	2489.932884	2991.181798	1984.545952	1567.840611	4947.38903	1742.664395	1635.097195	3996.713896	1615.526164
**ENSG00000131398**	KCNC3	174.372611	182.1666117	158.7754778	242.7977593	209.1977996	215.4888359	274.7551299	257.5034586	304.8255804	242.9865801	269.0228546	236.8310607
**ENSG00000131400**	NAPSA	1.406230734	0	2.442699658	2.511700959	1.155788948	0.379381753	0	1.661312636	3.751699452	2.53111021	0	0
**ENSG00000131408**	NR1H2	348.7452221	493.9517739	425.0297406	330.7072929	371.0082524	457.1550127	255.3486337	333.9238399	324.5220026	246.7832454	238.6715581	248.6726137
**ENSG00000131437**	KIF3A	476.7122189	571.0222635	409.5593094	648.0188473	635.6839215	501.1632961	771.1528738	679.4768682	644.3543808	674.5408708	771.1988497	803.5339559
**ENSG00000131446**	MGAT1	1406.230734	1523.893771	1436.307399	1389.807864	1422.776195	1443.547571	1080.635418	1056.594837	1177.564665	1083.31517	979.5191114	1078.427151
**ENSG00000131459**	GFPT2	0	0	1.628466439	2.511700959	11.55788948	6.449489806	0	4.983937908	2.813774589	3.796665314	4.138813147	0
**ENSG00000131462**	TUBG1	970.2992066	987.9035479	925.7831705	1298.549396	1151.165792	1268.273201	583.2162794	521.6521677	784.1051854	676.4392035	547.7029398	852.5918185
**ENSG00000131467**	PSME3	2244.344252	1881.220586	2205.757791	2458.118005	2376.302078	2519.474224	1924.307304	1430.39018	1935.407955	1778.7377	1536.879282	1906.490039
**ENSG00000131469**	RPL27	4300.253585	3776.453988	4180.273349	4236.402284	3910.034012	4188.374556	3517.682778	3983.827701	4070.124943	3820.078084	3795.291656	3988.911722
**ENSG00000131470**	PSMC3IP	307.9645308	346.817203	288.2385597	264.5658343	294.7261818	319.818818	253.3058446	285.7457734	199.7779958	240.4554699	266.2636458	216.5312555
**ENSG00000131471**	AOC3	4.218692202	7.006408141	5.699632536	2.511700959	0	3.793817533	0	6.645250545	2.344812157	7.593330629	1.379604382	5.920776517
**ENSG00000131473**	ACLY	2706.994163	2108.92885	2366.161736	2948.736925	2695.299827	2981.181817	2011.125839	2000.220414	2368.260279	2358.361938	2182.534133	2579.766911
**ENSG00000131475**	VPS25	826.8636717	679.6215897	749.0945619	834.7219519	584.8292078	781.1470301	665.9492367	662.8637418	663.5818405	687.8291994	677.3857517	731.6388125
**ENSG00000131480**	AOC2	30.93707615	21.01922442	15.47043117	33.48934611	21.95999002	20.10723293	13.27812895	26.58100218	16.88264753	32.27165517	23.4532745	11.84155303
**ENSG00000131484**	AC091132.1	0	0	0	0	5.778944741	2.27629052	0	0	0	0	0	0
**ENSG00000131495**	NDUFA2	224.9969175	199.682632	211.7006371	272.1009372	294.7261818	302.7466391	223.6854031	235.9063943	263.5568865	296.1398945	251.0879976	323.951058
**ENSG00000131503**	ANKHD1	175.7788418	129.6185506	182.3882412	130.6084498	156.031508	132.7836137	255.3486337	176.0991394	193.6814842	199.324929	216.597888	131.9487338
**ENSG00000131504**	DIAPH1	6024.292465	6438.889081	6292.39432	6241.576882	6265.531889	6262.454602	6093.639795	6912.721879	6269.089784	5816.491261	6115.786227	6422.350871
**ENSG00000131507**	NDFIP1	1250.139123	1296.185506	1460.734396	1529.625884	1717.502377	1539.151773	1412.588642	1413.777053	1578.527544	1410.461164	1313.383372	1703.491987
**ENSG00000131508**	UBE2D2	1306.388352	1317.20473	1197.737066	1379.76106	1359.207803	1430.26921	1179.710688	1103.11159	1274.639889	1213.667345	1140.932824	1343.170444
**ENSG00000131558**	EXOC4	1224.826969	1198.095792	1244.148359	1175.476049	1332.624657	1133.213297	1526.98483	1525.085	1322.943019	1445.896707	1600.341083	1442.131995
**ENSG00000131584**	ACAP3	122.3420739	168.1537954	104.2218521	198.4243757	213.8209554	176.0331335	184.8724108	177.7604521	157.571377	215.1443678	273.1616677	163.2442668
**ENSG00000131591**	C1orf159	191.2473798	164.6505913	178.3170751	211.8201142	201.107277	218.9032717	182.8296217	187.7283279	179.1436488	217.0427005	235.9123494	162.3984416
**ENSG00000131620**	ANO1	178.5913032	276.7531216	521.9234937	469.6880793	796.3385854	1015.604954	142.9952349	127.921073	212.4399814	217.675478	143.4788558	268.9724189
**ENSG00000131626**	PPFIA1	1110.92228	1071.980446	1121.199143	1118.54416	1182.372094	1178.739108	1165.411164	1204.451661	1084.241142	1106.727939	1196.116999	1125.793364
**ENSG00000131650**	KREMEN2	4.218692202	0	8.142332194	5.86063557	9.246311586	6.070108053	4.08557814	1.661312636	6.096511609	0.632777552	4.138813147	4.229126084
**ENSG00000131652**	THOC6	250.3090707	269.7467134	177.5028418	264.5658343	246.183046	200.3135657	317.6537003	310.665463	343.7494623	310.6937782	366.9747657	330.7176598
**ENSG00000131653**	TRAF7	1143.265587	991.4067519	1100.843313	1232.407937	1281.769944	1260.685566	908.0197415	940.302952	1130.19946	1026.997968	979.5191114	965.9323976

**ENSG00000131669**	NINJ1	74.53022891	73.56728548	72.46675653	61.95529031	109.7999501	68.28871559	120.5245551	196.0348911	115.8337206	117.6966247	195.9038223	119.2613556
**ENSG00000131697**	NPHP4	233.4343019	245.2242849	225.5426018	278.7988064	240.4041012	215.4888359	370.7662162	343.8917157	394.8663673	442.3115091	422.158941	361.1673676
**ENSG00000131711**	MAP1B	56.24922937	59.5544692	74.09522297	27.62871054	39.29682424	77.77325943	16.34231256	6.645250545	7.503398903	3.163887762	1.379604382	4.229126084
**ENSG00000131721**	RHOXF2	0	14.01281628	2.442699658	0	2.311577897	1.13814526	1.021394535	0	0.468962431	1.265555105	1.379604382	0
**ENSG00000131724**	IL13RA1	583.5857547	816.2465484	752.3514948	721.6954088	966.2395607	905.9636269	997.9024606	1021.707271	918.2284408	1230.752339	1422.372118	1190.921905
**ENSG00000131725**	WDR44	444.368912	504.4613861	403.0454436	338.2423958	410.3050766	360.4126656	356.4666927	501.7164161	320.3013407	322.0837742	350.4195131	325.6427085
**ENSG00000131732**	ZCCHC9	576.554601	627.0735286	512.9669283	527.4572013	514.326082	496.9900968	870.2281437	971.8678921	778.0086738	744.1464016	823.6238162	839.9044403
**ENSG00000131737**	KRT34	257.3402244	182.1666117	232.0564675	346.6147323	161.8104528	289.8476595	22.47067977	49.83937908	83.94427523	51.8877593	55.18417529	96.42407471
**ENSG00000131746**	TNS4	3508.545682	1993.323116	3005.334813	3810.250354	2934.54814	4876.573057	2491.181271	1566.617816	2722.326915	3018.348925	1894.196817	2663.503608
**ENSG00000131747**	TOP2A	8213.793718	3587.280968	6685.668965	10456.21109	8706.558147	9382.110759	10978.96986	7583.892184	11618.54424	11448.21148	9312.329581	14128.66442
**ENSG00000131748**	STARD3	378.2760675	532.4870187	446.1998043	406.0583216	357.138785	418.8374556	251.2630556	307.3428377	333.9012512	286.0154537	313.1701948	297.7304763
**ENSG00000131759**	RARA	87.18630552	94.5865099	89.56565414	97.95633739	93.61890481	80.4289317	46.9841486	69.77513072	86.28908739	88.58885733	82.77626294	70.20349299
**ENSG00000131773**	KHDRBS3	53.4367679	21.01922442	22.79853014	32.65211246	38.14103529	26.17734098	75.58319558	147.8568246	134.5922178	106.3066288	110.3683506	136.1778599
**ENSG00000131778**	CHD1L	60.46792157	24.52242849	49.66822639	38.51274803	33.5178795	34.52373955	37.79159779	58.14594226	39.86180667	39.8649858	51.04536215	14.37902869
**ENSG00000131779**	PEX11B	84.37384405	126.1153465	87.12295448	125.5850479	146.7851964	98.25987411	208.3644851	269.1326471	225.1019671	203.7543719	180.7281741	213.1479546
**ENSG00000131781**	FMO5	1.406230734	0	0.814233219	2.511700959	4.623155793	6.828871559	68.43343384	61.46856754	27.66878346	39.23220825	33.11050518	26.22058172
**ENSG00000131788**	PIAS3	494.9932184	620.0671205	516.2238611	612.0178002	777.8459622	606.6314235	543.3818926	589.7659858	525.2379232	633.4103299	670.4877298	565.85707
**ENSG00000131791**	PRKAB2	480.9309111	413.3780803	561.8209214	381.7785457	439.1998003	422.6312732	967.2606245	1028.352522	854.4495501	959.2907694	1058.156561	883.8873515
**ENSG00000131797**	CLUHP3	396.557067	609.5575083	490.1683981	412.7561909	435.7324335	414.2848746	568.9167559	511.6842919	521.4862238	507.487597	605.6463238	451.6706658
**ENSG00000131828**	PDHA1	974.5178988	1043.954813	810.9762866	964.4931681	956.9932492	937.0729307	909.041136	855.5760076	940.7386375	990.929647	1022.286847	1107.185209
**ENSG00000131844**	MCCC2	1859.037031	1250.643853	1523.430354	1544.69609	1403.127783	1569.502313	1951.884956	1559.972565	1881.477275	1774.941034	1318.901789	1715.33354
**ENSG00000131845**	ZNF304	174.372611	185.6698157	190.5305734	192.5637402	157.187297	160.8578634	225.7281922	252.5195207	226.9778168	231.5965842	224.8755143	200.4605764
**ENSG00000131848**	ZSCAN5A	112.4984587	154.1409791	126.206149	123.073347	107.4883722	99.01863761	111.3320043	81.40431917	107.3923968	136.0471738	110.3683506	111.6489286
**ENSG00000131849**	ZNF132	21.09346101	7.006408141	9.770798633	13.39573845	8.090522638	6.070108053	19.40649616	24.91968954	30.48255804	29.74054496	24.83287888	22.83728085
**ENSG00000131871**	SELENOS	525.9302946	514.9709984	603.3468156	398.5232188	417.2398103	484.8498807	157.2947584	152.8407625	191.8056345	175.279382	155.8952952	181.0065964
**ENSG00000131873**	CHSY1	444.368912	616.5639164	514.5953947	514.8986965	628.7491879	623.3242207	225.7281922	290.7297113	235.4191406	263.8682393	314.5497992	267.2807685
**ENSG00000131876**	SNRPA1	870.4568244	679.6215897	796.3200886	783.6506991	760.509128	899.1347553	546.4460762	500.0551035	583.8582272	511.9170399	522.8700609	583.6193996
**ENSG00000131899**	LLGL1	323.4330689	231.2114686	304.5232241	340.7540967	365.2293077	321.336345	187.9365944	287.407086	251.3638633	222.7376984	252.467602	200.4605764
**ENSG00000131931**	THAP1	195.466072	196.1794279	201.1156052	159.9116277	195.3283323	196.1403665	123.5887387	156.1633878	121.9302322	114.532737	157.2748996	164.0900921
**ENSG00000131941**	RHPN2	250.3090707	210.1922442	232.0564675	292.1945449	217.2883223	317.9219093	323.7820676	441.9091612	402.8387286	261.3371291	379.3912051	373.8547458
**ENSG00000131943**	C19orf12	137.8106119	126.1153465	132.7200148	92.93293547	88.99574902	115.332053	108.2678207	86.38825708	115.8337206	84.15941447	81.39665856	76.12426951
**ENSG00000131944**	FAAP24	37.96822982	28.02563256	56.18209214	59.44358935	88.99574902	75.11758715	82.73295732	121.2758224	102.7027725	103.142741	122.78479	84.58252168
**ENSG00000131951**	LRRC9	0	0	0	0	9.246311586	0.379381753	1.021394535	0	0	0	0	0
**ENSG00000131966**	ACTR10	369.8386831	546.499835	389.2034789	424.477462	407.9934987	384.6930979	478.0126423	435.2639107	507.8863133	449.2720622	473.2043031	472.8162962
**ENSG00000131969**	ABHD12B	1.406230734	0	2.442699658	1.674467306	2.311577897	3.793817533	4.08557814	3.322625272	0.468962431	0	1.379604382	0.845825217
**ENSG00000131979**	GCH1	482.3371418	802.2337321	455.9706029	208.4711796	258.8967244	279.9837339	369.7448216	529.9587309	431.4454369	253.7437985	376.6319964	263.8974676
**ENSG00000131981**	LGALS3	233.4343019	287.2627338	298.8235915	412.7561909	398.7471872	408.5941483	984.6243316	767.5264379	648.5750427	969.4152102	813.9665856	824.6795864
**ENSG00000132000**	PODNL1	2.812461468	0	0	0.837233653	2.311577897	4.931962793	0	0	0.937924863	1.265555105	0	0
**ENSG00000132002**	DNAJB1	2122.002178	2613.390237	2820.503872	2607.982829	2666.405104	3159.491242	2041.767675	2159.706427	2568.9762	2027.419278	2316.355758	2571.308659
**ENSG00000132003**	ZSWIM4	402.18199	535.9902228	515.4096279	291.3573112	301.6609155	446.1529419	180.7868327	252.5195207	196.0262963	135.4143962	147.6176689	120.953006
**ENSG00000132004**	FBXW9	71.71776744	38.53524477	45.59706029	77.02549606	76.28207059	75.11758715	77.62598465	103.0013834	95.66833602	112.6344043	115.8867681	93.04077385
**ENSG00000132005**	RFX1	120.9358431	175.1602035	143.3050466	126.4222816	184.9262317	135.4392859	134.8240786	199.3575163	145.8473162	197.4265963	211.0794705	141.2528112
**ENSG00000132010**	ZNF20	16.87476881	3.50320407	5.699632536	10.88403749	5.778944741	6.828871559	16.34231256	11.62918845	22.04123428	19.61610412	6.898021912	29.60388259
**ENSG00000132016**	C19orf57	63.28038304	161.1473872	83.8660216	63.62975762	84.37259322	75.87635066	156.2733638	181.0830773	114.4268333	142.3749493	146.2380645	123.4904816
**ENSG00000132017**	DCAF15	241.8716863	129.6185506	276.8392946	239.4488247	251.9619907	305.0229297	167.5087037	204.3414542	242.9225395	211.98048	227.6347231	170.0108686
**ENSG00000132024**	CC2D1A	490.7745262	364.3332233	398.1600443	416.9423591	443.8229561	539.1014714	340.1243801	360.504842	389.7077805	406.2431886	394.5668533	408.5335797
**ENSG00000132026**	RTBDN	5.624922937	7.006408141	4.071166097	2.511700959	3.467366845	1.896908767	6.128367209	0	2.813774589	3.163887762	0	0
**ENSG00000132031**	MATN3	0	3.50320407	1.628466439	0	0	0	4.08557814	0	4.220661883	1.898332657	0	1.691650434
**ENSG00000132109**	TRIM21	1012.486129	1250.643853	1012.906125	244.4722266	285.4798702	231.4228695	962.1536519	1241.000539	1016.710551	385.3615294	444.2326111	432.2166858
**ENSG00000132122**	SPATA6	54.84299863	84.07689769	35.01202844	50.23401917	91.30732691	34.9031213	256.3700283	262.4873965	240.5777273	207.5510372	280.0596896	170.0108686
**ENSG00000132128**	LRRC41	705.9278285	507.9645902	642.4300101	589.4124916	545.5323836	632.050001	736.4254596	745.9293736	724.5469566	781.4802772	747.7455752	652.1312421
**ENSG00000132141**	CCT6B	30.93707615	28.02563256	18.72736405	23.44254228	23.11577897	13.27836137	53.11251581	28.24231481	37.51699452	61.37942258	37.24931832	45.67456171
**ENSG00000132153**	DHX30	696.0842134	567.5190594	678.2562718	766.0687924	641.4628663	753.4521621	717.0189635	588.1046732	818.8084053	723.8975199	742.2271577	782.3883255
**ENSG00000132155**	RAF1	1077.172742	928.3490787	1075.602083	1285.990891	1100.311079	1246.648441	1449.358845	1526.746313	1504.43148	1615.481091	1665.182489	1536.864419
**ENSG00000132164**	SLC6A11	7.031153671	0	7.328098975	7.535102876	4.623155793	5.311344546	0	3.322625272	1.406887294	0	0	2.53747565
**ENSG00000132170**	PPARG	199.6847643	112.1025303	145.7477463	331.5445265	264.6756692	270.1198084	637.3501898	882.1570098	774.2569743	656.1903218	924.3349361	855.1292942
**ENSG00000132182**	NUP210	1178.421355	900.3234461	1255.547624	886.6304384	1029.807953	1116.5205	1469.786736	1297.485169	1638.554735	1661.673853	1429.27014	1500.493935
**ENSG00000132185**	FCRLA	0	14.01281628	0	1.674467306	10.40210053	3.41443578	105.2036371	181.0830773	41.7376564	47.45831643	99.33151553	20.2998052
**ENSG00000132196**	HSD17B7	233.4343019	290.7659378	233.684934	221.866918	294.7261818	197.6578935	194.0649616	275.7778976	232.1364036	243.6193577	351.7991175	218.2229059
**ENSG00000132199**	ENOSF1	808.5826721	728.6664466	687.2128372	723.3698761	628.7491879	634.3262915	1071.442867	931.9963889	961.3729845	1159.248476	1018.148034	935.4826898
**ENSG00000132204**	LINC00470	794.5203648	644.589549	747.4660955	737.6028482	564.0250068	716.652132	817.1156279	724.3323094	806.6153821	910.5668979	772.5784541	778.1591994
**ENSG00000132205**	EMILIN2	0	0	0	0.837233653	13.86946738	4.55258104	15.32091802	61.46856754	22.97915914	15.18666126	51.04536215	27.91223215
**ENSG00000132254**	ARFIP2	669.3658295	623.5703245	665.2285403	653.8794829	618.3470873	629.3943287	751.7463777	860.5599455	815.9946307	727.6941852	722.9126963	720.6430847
**ENSG00000132256**	TRIM5	1168.57774	1744.595627	1032.447722	445.4083033	576.7386852	474.9859551	794.6449481	1026.691209	839.4427523	546.7198053	609.785137	581.9277491
**ENSG00000132274**	TRIM22	1985.597797	2921.672195	1548.671583	11.72127114	32.36209055	6.828871559	2141.86434	2408.903322	1541.01055	251.2126883	187.626196	102.3448512
**ENSG00000132275**	RRP8	303.7458386	213.6954483	343.6064186	382.6157794	302.8167044	372.9322635	487.2051931	372.1340305	493.8174403	428.390403	408.3628972	518.4908579
**ENSG00000132286**	TIMM10B	535.7739097	535.9902228	518.6665608	514.0614629	471.5618909	579.3159373	539.2963144	574.8141721	603.0856868	635.9414401	598.7483019	693.5766778

**ENSG00000132294**	EFR3A	1406.230734	1457.332893	1496.560657	1103.473954	1026.340586	1210.986557	1319.641739	1081.514526	1364.211713	1195.949574	1071.952605	1358.395298
**ENSG00000132297**	HHLA1	1.406230734	17.51602035	0.814233219	0.837233653	2.311577897	4.173199286	0	0	0	0	0	0
**ENSG00000132300**	PTCD3	1081.391435	1001.916364	1102.471779	1068.310141	983.576395	1119.934936	1444.251872	1224.387413	1344.515291	1440.834487	1291.309702	1302.570834
**ENSG00000132305**	IMMT	1454.042579	1502.874546	1429.793533	1475.205696	1546.445613	1404.471251	1230.780415	1445.341993	1256.819316	1368.697846	1422.372118	1352.474522
**ENSG00000132313**	MRPL35	518.8991409	441.4037129	577.2913526	699.9273338	669.201801	673.7819939	603.6441701	495.0711656	566.5066172	545.4542502	488.3799513	679.1976491
**ENSG00000132321**	IQCA1	0	0	0	0	0	0	4.08557814	11.62918845	13.59991051	8.858885733	6.898021912	8.458252168
**ENSG00000132323**	ILKAP	402.18199	280.2563256	430.7293731	512.3869956	369.8524634	393.7982599	349.3169309	395.3924074	380.7974943	396.1187478	429.0569629	491.4244509
**ENSG00000132326**	PER2	151.8729193	178.6634076	138.4196473	142.329721	177.991498	191.5877854	257.3914228	169.4538889	204.4676201	248.0488005	166.9321303	174.2399947
**ENSG00000132329**	RAMP1	53.4367679	28.02563256	27.68392946	64.46699127	45.07576898	41.73199286	9.192550814	13.29050109	30.95152048	29.74054496	20.69406573	42.29126084
**ENSG00000132330**	SCLY	101.2486129	42.03844885	82.23755516	99.63080469	68.19154795	62.21860754	211.4286687	136.2276362	203.9986577	227.1671413	129.6828119	195.3856251
**ENSG00000132334**	PTPRE	800.1452877	732.1696507	1075.602083	704.9507357	535.130283	734.8624562	160.358942	220.9545806	382.6733441	165.7877187	219.3570968	433.062511
**ENSG00000132341**	RAN	6163.509308	4890.472882	5943.088269	6591.540549	6185.782451	6781.828222	5939.40922	5445.782821	6607.211697	6469.517696	5717.08056	7354.45026
**ENSG00000132356**	PRKAA1	2922.147466	2837.595297	2629.973299	3406.703734	2979.623909	3200.464471	2422.747837	2327.499003	2207.406165	2279.897521	2215.644638	2467.272157
**ENSG00000132357**	CARD6	448.5876042	606.0543042	567.520554	241.123292	351.3598403	289.8476595	131.759895	124.5984477	112.5509835	127.8210656	117.2663725	136.1778599
**ENSG00000132359**	RAP1GAP2	1342.950351	1376.7592	1519.359187	1755.67897	1748.708679	2023.24289	39.83438686	63.12988017	75.50295146	34.16998783	75.87824103	89.65747298
**ENSG00000132361**	CLUH	759.3645964	658.6023652	814.2332194	874.9091672	796.3385854	1013.708045	720.0831471	412.0055338	706.7263842	789.0736078	496.6575776	698.6516291
**ENSG00000132376**	INPP5K	330.4642225	367.8364274	381.8753799	312.2881525	360.6061519	350.9281218	388.1299233	397.05372	399.0870292	344.863766	376.6319964	348.4799893
**ENSG00000132382**	MYBBP1A	918.2686694	427.3908966	904.6131068	1135.288833	804.429108	1160.528783	592.4088302	333.9238399	634.9751322	616.9581136	429.0569629	614.9149326
**ENSG00000132383**	RPA1	2199.344868	2532.816543	1897.163401	2755.335952	2586.655666	2436.38962	2435.004571	2501.93683	2549.279777	2500.104109	2652.979227	2695.644966
**ENSG00000132386**	SERPINF1	0	0	0	2.511700959	0	0.758763507	6.128367209	1.661312636	5.158586746	11.38999594	2.759208765	8.458252168
**ENSG00000132388**	UBE2G1	1798.569109	1562.429015	1688.719697	2120.712843	1856.197051	2105.568731	1688.365166	1619.77982	1680.292392	1764.816594	1552.05493	1793.14946
**ENSG00000132394**	EEFSEC	185.6224569	105.0961221	177.5028418	149.8648239	164.1220307	179.8269511	163.4231256	181.0830773	217.5985682	257.5404638	211.0794705	216.5312555
**ENSG00000132405**	TBC1D14	1042.016974	735.6728548	1001.50686	1198.081357	1034.431109	1177.980344	651.6497133	521.6521677	697.3471356	769.4575037	674.6265429	712.1848325
**ENSG00000132406**	TMEM128	164.5289959	164.6505913	198.6729055	137.3063191	113.2673169	157.4434276	162.401731	154.5020752	184.771198	170.2171616	114.5071637	168.3192181
**ENSG00000132423**	COQ3	174.372611	140.1281628	186.4594073	232.7509555	180.3030759	202.2104745	215.5142469	194.3735784	147.7231659	213.2460352	140.719647	225.8353329
**ENSG00000132424**	PNISR	2195.126176	2350.649931	2159.346498	2362.673368	2263.034761	1989.857296	3195.9435	3581.790043	2671.21001	3091.118343	3519.370779	2805.602244
**ENSG00000132429**	POPDC3	33.74953762	35.0320407	81.42332194	48.55955187	68.19154795	97.12172885	0	0	0	0	0	0
**ENSG00000132432**	SEC61G	741.0835969	1572.938628	916.0123719	836.3964192	935.0332591	794.8047732	471.8842751	1020.045959	529.9275475	475.8487194	916.0573099	594.6151274
**ENSG00000132434**	LANCL2	142.0293041	87.58010176	146.5619795	216.8435161	121.3578396	176.4125153	207.3430906	164.469951	158.5093018	182.8727126	151.7564821	151.4027138
**ENSG00000132436**	FIGNL1	1217.795816	1208.605404	1106.542945	1303.572798	1223.980496	1177.980344	1043.865215	1076.530588	1104.875489	1145.32737	1267.856427	1238.288117
**ENSG00000132463**	GRSF1	1919.504952	1460.836097	1876.807571	2103.130936	2141.676921	2049.799613	1542.305748	1653.006073	1712.6508	1557.898334	1634.831193	1753.395674
**ENSG00000132466**	ANKRD17	2877.148082	2550.332563	2991.492848	2984.737972	3017.764944	2777.453816	2499.352427	2432.161699	2403.432461	2398.859701	2148.044023	2567.079533
**ENSG00000132467**	UTP3	490.7745262	416.8812844	571.5917201	477.2231821	427.6419109	503.4395866	377.9159779	403.6989706	441.293648	447.3737295	362.8359525	455.8997918
**ENSG00000132470**	ITGB4	15604.94246	14450.71679	16516.72086	13438.43736	14883.09429	16958.74375	7838.181661	8469.371819	9962.168931	6961.818631	7856.846957	7326.538028
**ENSG00000132471**	WBP2	1008.267436	1240.134241	1016.163058	904.2123451	1126.894225	1095.275122	464.7345134	513.3456046	612.4649355	504.3237092	531.1476872	529.4865857
**ENSG00000132475**	H3F3B	7232.244666	6396.850633	7065.101645	7825.622953	6671.213809	7274.64512	4604.446563	3839.293502	5003.829144	4400.335099	3683.543701	4925.240237
**ENSG00000132478**	UNK	254.5277629	266.2435094	251.5980648	255.3562641	199.951488	234.0785418	288.0332588	338.9077778	321.2392655	373.9715335	353.1787219	281.6597972
**ENSG00000132481**	TRIM47	142.0293041	126.1153465	141.6765802	137.3063191	204.5746438	216.2475994	59.24088302	59.8072549	34.2342575	33.53721028	45.52694462	55.82446431
**ENSG00000132485**	ZRANB2	2542.465167	2529.313339	2385.703333	2446.396734	2215.647414	2141.230616	2839.476807	2317.531127	2514.107595	2801.939002	2379.817559	2710.023995
**ENSG00000132507**	EIF5A	7277.244049	5335.379799	6245.168793	6493.584212	5858.694179	6565.960004	4036.551202	3076.751002	4892.216085	4185.190731	3422.798473	4507.40258
**ENSG00000132510**	KDM6B	487.9620648	872.2978135	641.6157769	446.245537	626.43761	548.965397	611.8153264	863.8825708	697.3471356	638.4725504	863.6323433	643.67299
**ENSG00000132517**	SLC52A1	8.437384405	10.50961221	9.770798633	1.674467306	10.40210053	6.449489806	12.25673442	18.274439	17.8205724	13.2883286	19.31446135	15.2248539
**ENSG00000132518**	GUCY2D	36.56199909	31.52883663	27.68392946	43.53614995	58.94523636	39.45570234	3.064183605	1.661312636	4.689624315	8.858885733	1.379604382	9.304077385
**ENSG00000132522**	GPS2	12.65607661	17.51602035	8.956565414	13.39573845	18.49262317	10.24330734	7.149761744	18.274439	15.94472267	20.24888168	9.657230676	13.53320347
**ENSG00000132530**	XAF1	3834.791212	4168.812844	3693.361883	39.34998168	142.1620406	45.14642864	1362.54031	1520.101062	1128.32361	13.2883286	38.6289227	4.229126084
**ENSG00000132535**	DLG4	43.59315276	63.05767327	51.29669283	54.42018744	84.37259322	62.97737105	15.32091802	31.56494009	12.66198565	13.2883286	19.31446135	10.1499026
**ENSG00000132541**	RIDA	153.27915	136.6249587	190.5305734	178.3307681	217.2883223	143.4063027	106.2250316	131.2436983	83.00635037	107.5721839	85.5354717	142.9444616
**ENSG00000132549**	VPS13B	1558.103653	1439.816873	1421.651201	1626.744988	1525.641412	1573.675513	1672.022854	1440.358056	1604.320478	1555.367224	1547.916117	1535.172768
**ENSG00000132561**	MATN2	18.28099954	17.51602035	22.79853014	12.55850479	16.18104528	23.14228695	54.13391035	101.3400708	207.7503571	46.19276132	91.05388923	212.3021294
**ENSG00000132563**	REEP2	0	0	0	4.186168264	1.155788948	1.896908767	4.08557814	0	0	0	0	0
**ENSG00000132570**	PCBD2	227.8093789	154.1409791	206.0010045	267.0775353	202.2630659	242.0455586	324.8034621	244.2129575	315.1427539	460.0292806	259.3656239	285.0430981
**ENSG00000132581**	SDF2	351.5576835	458.9197332	397.3458111	371.7317419	398.7471872	411.2498206	372.8090052	478.4580392	385.9560811	378.4009763	427.6773585	374.700571
**ENSG00000132589**	FLOT2	659.5222143	805.7369362	737.6952968	585.2263234	740.8607158	710.5820239	518.8684237	553.2171078	513.9828249	487.8714929	607.0259282	523.5658092
**ENSG00000132591**	ERAL1	454.2125271	420.3844885	488.5399317	581.8773887	513.170293	651.7778522	449.4135953	407.0215959	523.8310359	476.4814969	460.7878637	543.0197892
**ENSG00000132600**	PRMT7	329.0579918	273.2499175	284.9816268	354.1498352	265.8314581	316.4043823	410.600603	305.681525	370.4803208	434.0854009	420.7793366	362.0131928
**ENSG00000132603**	NIP7	880.3004396	535.9902228	815.8616859	765.2315587	481.9639914	759.1428884	482.0982205	430.2799728	543.5274581	445.4753969	340.7622824	548.0947405
**ENSG00000132604**	TERF2	628.5851382	455.4165292	626.959579	565.1327157	537.4418609	552.0004511	422.8573374	539.9266067	562.7549177	467.6226112	535.2865003	451.6706658
**ENSG00000132612**	VPS4A	319.2143767	283.7595297	362.3337827	342.428564	318.9977497	404.0415673	303.3541769	275.7778976	294.9773694	281.5860108	209.6998661	292.655525
**ENSG00000132613**	MTSS1L	320.6206074	350.320407	288.2385597	682.3454271	616.0355094	707.1675882	759.9175339	679.4768682	1004.517528	1015.607972	667.728521	1049.669094
**ENSG00000132622**	HSPA12B	2.812461468	31.52883663	0	0	2.311577897	0	0	0	0	0.632777552	0	0
**ENSG00000132623**	ANKEF1	916.8624387	728.6664466	880.1861102	848.1176904	850.6606659	896.4790831	714.9761744	614.6856754	723.6090317	748.5758445	634.6180159	755.3219186
**ENSG00000132635**	PCED1A	334.6829147	203.1858361	303.7089909	185.0286373	247.3388349	196.1403665	488.2265877	463.5062255	453.9556336	477.747052	513.2128302	426.2959093
**ENSG00000132639**	SNAP25	2.812461468	0	0.814233219	0	0	0.379381753	0	8.306563181	0	0	1.379604382	0
**ENSG00000132640**	BTBD3	905.6125928	938.8586909	743.3949294	793.6975029	1014.782697	947.316238	610.7939319	843.9468192	615.7476725	647.3314361	749.1251796	791.6924029
**ENSG00000132646**	PCNA	3719.480292	4711.809475	3051.746106	3587.546203	3912.34559	3180.357238	4333.777011	5512.235327	4193.462062	3842.858076	4740.320658	4519.244133
**ENSG00000132661**	NXT1	343.1202991	325.7979785	362.3337827	367.5455736	249.6504128	423.7694184	398.3438686	340.5690904	374.7009827	359.4176498	286.9577115	377.2380467
**ENSG00000132664**	POLR3F	471.0872959	374.8428355	468.1841012	527.4572013	392.9682424	525.4437283	474.9484587	428.6186601	462.8659198	498.6287113	434.5753804	487.1953249

**ENSG00000132669**	RIN2	2085.440179	1142.044527	1820.625479	1866.193812	1490.967743	1968.611918	2819.048916	3580.128731	2559.127988	2433.662466	3192.404541	2172.079157
**ENSG00000132670**	PTPRA	1196.702355	1289.179098	1283.231554	1321.154704	1302.574145	1119.176172	1342.112419	1558.311253	1337.011892	1511.072795	1488.593129	1416.757238
**ENSG00000132676**	DAP3	1844.974723	1758.608443	2010.341819	1666.932203	1448.203552	1667.003424	1420.759798	1237.677914	1411.576919	1314.278976	1260.958405	1398.149083
**ENSG00000132677**	RHBG	4.218692202	0	6.513865756	0	3.467366845	1.896908767	1.021394535	0	3.751699452	2.53111021	0	1.691650434
**ENSG00000132680**	KHDC4	1348.575274	1614.977076	1457.477463	1249.989844	1167.346838	1204.157685	1386.032384	1181.193284	1302.777635	1244.040668	1225.088691	1274.658602
**ENSG00000132681**	ATP1A4	0	3.50320407	0.814233219	0	1.155788948	0	16.34231256	28.24231481	20.16538455	22.77999189	23.4532745	33.83300867
**ENSG00000132688**	NES	33.74953762	115.6057343	104.2218521	96.28187008	92.46311586	147.9588838	0	9.967875817	3.28273702	1.898332657	0	3.383300867
**ENSG00000132694**	ARHGEF11	1174.202663	1362.746383	1261.247257	1198.081357	1311.820456	1365.39493	1113.320043	1089.821089	1126.44776	1096.603498	1106.442715	1069.968899
**ENSG00000132698**	RAB25	772.0206731	735.6728548	763.7507598	862.3506625	834.4796206	836.536766	640.4143734	636.2827396	753.6226273	622.020334	549.0825442	717.2597838
**ENSG00000132703**	APCS	0	0	0	0	0	0	1.021394535	0	6.56547404	8.226108181	2.759208765	18.60815477
**ENSG00000132704**	FCRL2	5.624922937	3.50320407	2.442699658	0	2.311577897	0	0	0	0	0	0	0
**ENSG00000132716**	DCAF8	544.2112941	637.5831408	561.8209214	647.1816137	533.9744941	545.930343	927.4262377	762.5425	744.2433787	771.3558364	802.9297505	691.0392021
**ENSG00000132718**	SYT11	1.406230734	7.006408141	0.814233219	6.697869223	4.623155793	3.035054026	0	3.322625272	3.28273702	0.632777552	1.379604382	0.845825217
**ENSG00000132740**	IGHMBP2	262.9651473	343.3139989	381.0611467	375.0806765	303.9724934	321.7157268	235.9421376	171.1152015	278.5636843	230.3310291	173.8301522	265.5891181
**ENSG00000132744**	ACY3	26.71838395	38.53524477	37.45472809	0.837233653	5.778944741	3.41443578	3.064183605	0	2.813774589	0	0	3.383300867
**ENSG00000132746**	ALDH3B2	1.406230734	0	0	2.511700959	3.467366845	5.6907263	12.25673442	14.95181373	10.78613592	20.88165923	46.906549	6.766601734
**ENSG00000132749**	TESMIN	164.5289959	248.727489	102.5933857	93.77016912	135.2273069	122.5403063	15.32091802	16.61312636	17.8205724	22.14721433	22.07367012	21.14563042
**ENSG00000132763**	MMACHC	271.4025317	129.6185506	232.0564675	325.683891	286.6356592	266.7053726	180.7868327	109.646634	189.4608223	242.3538026	211.0794705	219.9145564
**ENSG00000132768**	DPH2	410.6193744	231.2114686	455.1563697	576.8539868	434.5766445	585.3860453	300.2899933	179.4217647	379.8595695	411.305409	255.2268107	427.9875597
**ENSG00000132773**	TOE1	358.5888372	329.3011826	306.9659237	376.7551438	287.7914481	364.2064832	431.0284937	297.3749619	453.0177088	455.5998377	320.0682167	407.6877545
**ENSG00000132780**	NASP	4189.161357	4585.694128	3494.688978	3646.152558	3550.583649	3035.81279	5017.089955	4701.51476	4587.390504	4972.366007	4796.884437	4876.182375
**ENSG00000132781**	MUTYH	98.43615139	84.07689769	87.12295448	75.35102876	57.78944741	49.69900968	141.9738403	111.3079466	88.16493711	118.3294023	60.70259282	92.19494863
**ENSG00000132792**	CTNNBL1	461.2436808	679.6215897	485.2829988	444.5710697	435.7324335	460.9488303	638.3715843	779.1556263	656.547404	570.7653522	666.3489167	601.3817291
**ENSG00000132793**	LPIN3	172.9663803	245.2242849	179.1313083	122.2361133	165.2778196	153.2702283	212.4500633	313.9880882	176.3298742	213.2460352	402.8444796	120.1071808
**ENSG00000132801**	ZSWIM3	61.8741523	31.52883663	68.39559043	52.74572013	47.38734688	55.38973598	56.17669942	54.82331699	48.30313044	50.62220419	41.38813147	55.82446431
**ENSG00000132819**	RBM38	236.2467633	262.7403053	243.4557326	468.8508456	350.2040513	428.3219995	172.6156764	129.5823856	223.6950798	189.2004882	128.3032076	225.8353329
**ENSG00000132821**	VSTM2L	4.218692202	0	3.256932878	0.837233653	0	0.379381753	0	0	0	0	1.379604382	0
**ENSG00000132823**	OSER1	860.6132093	823.2529565	851.6879475	936.0272239	729.3028264	883.9594852	792.6021591	817.365817	811.3050064	841.5941447	1005.731595	821.2962855
**ENSG00000132824**	SERINC3	2310.437096	2700.970338	2668.24226	2571.144548	2943.794451	2862.435329	2710.781096	3264.47933	2710.133891	2752.582353	3025.47241	2911.330396
**ENSG00000132825**	PPP1R3D	182.8099954	245.2242849	248.3411319	226.0530863	246.183046	297.4352946	325.8248566	304.0202124	287.9429329	270.8287924	229.0143275	270.6640694
**ENSG00000132837**	DMGDH	19.68723028	10.50961221	20.35583049	9.209570182	11.55788948	5.311344546	36.77020326	31.56494009	22.51019671	27.8422123	22.07367012	16.91650434
**ENSG00000132842**	AP3B1	1836.537339	1947.781463	1801.083881	1937.358673	2153.234811	1889.700513	1804.804143	1819.137337	1590.720567	1743.934934	1866.604729	1807.528488
**ENSG00000132846**	ZBED3	16.87476881	3.50320407	21.98429693	10.88403749	26.58314581	21.62475994	24.51346884	18.274439	20.16538455	27.8422123	5.518417529	9.304077385
**ENSG00000132849**	PATJ	2691.525625	3278.99901	2304.280011	2212.808545	2780.82821	2074.080045	421.8359429	719.3483714	406.1214656	375.2370886	507.6944127	441.5207632
**ENSG00000132854**	KANK4	1.406230734	0	2.442699658	3.348934611	6.93473369	7.967016819	505.5902948	717.6870588	418.7834513	634.675885	808.448168	444.904064
**ENSG00000132874**	SLC14A2	0	0	2.442699658	6.697869223	6.93473369	6.449489806	0	0	0	0	0	0
**ENSG00000132879**	FBXO44	75.93645964	108.5993262	99.33645277	130.6084498	161.8104528	141.509394	92.94690267	144.5341993	105.5165471	117.6966247	113.1275593	105.7281521
**ENSG00000132881**	RSG1	101.2486129	66.56087734	65.95289078	77.02549606	106.3325832	110.779472	60.26227756	54.82331699	77.37880119	68.97275321	55.18417529	57.51611474
**ENSG00000132906**	CASP9	39.37446056	87.58010176	54.5536257	64.46699127	119.0462617	72.84129663	118.481766	96.3561329	114.4268333	141.7421717	148.9972733	126.0279573
**ENSG00000132912**	DCTN4	1316.231967	1240.134241	1440.378565	1249.15261	1155.788948	1355.531005	1557.626666	1357.292424	1426.583716	1438.936154	1280.272867	1452.281897
**ENSG00000132915**	PDE6A	5.624922937	3.50320407	5.699632536	2.511700959	4.623155793	3.793817533	7.149761744	4.983937908	8.910286198	10.12444084	8.277626294	4.229126084
**ENSG00000132932**	ATP8A2	1058.891743	721.6600385	942.8820681	1100.962254	946.5911486	773.559395	2.04278907	3.322625272	0.937924863	1.265555105	1.379604382	0
**ENSG00000132938**	MTUS2	0	0	0	0	2.311577897	0	54.13391035	46.51675381	102.2338101	93.0183002	66.22101035	115.8780547
**ENSG00000132950**	ZMYM5	226.4031482	231.2114686	259.740397	228.5647872	206.8862217	212.4537819	226.7495867	252.5195207	234.9501782	238.5571372	212.4590749	216.5312555
**ENSG00000132952**	USPL1	589.2106776	514.9709984	611.4891478	615.3667349	663.4228563	637.7407273	417.7503648	420.3120969	450.2039342	462.5603908	422.158941	492.2702762
**ENSG00000132953**	XPO4	2057.315564	1650.009117	1950.902794	2393.651014	1863.131785	2288.051354	1532.091802	1207.774286	1588.844718	1664.204963	1314.762976	1693.342084
**ENSG00000132958**	TPTE2	19.68723028	17.51602035	12.21349829	7.535102876	15.02525633	14.41650663	5.106972674	4.983937908	7.972361335	8.858885733	6.898021912	0.845825217
**ENSG00000132963**	POMP	3200.581151	2981.226664	3182.837655	2155.039422	2149.767444	2189.79148	1537.198775	1764.31402	1460.349012	1233.28345	1484.454315	1262.817049
**ENSG00000132964**	CDK8	577.9608317	409.8748762	548.7931899	491.4561542	607.9449868	590.3180081	288.0332588	430.2799728	375.6389076	368.2765355	455.2694462	392.4629006
**ENSG00000132965**	ALOX5AP	2.812461468	0	6.513865756	0.837233653	4.623155793	4.55258104	0	0	0	0	0	0
**ENSG00000132967**	HMGB1P5	64.68661377	45.54165292	85.49448804	72.00209415	72.81470374	117.2289618	31.66323058	126.2597603	84.88220009	74.03497363	53.80457091	83.73669646
**ENSG00000132970**	WASF3	18.28099954	49.04485699	64.32442434	51.07125282	63.56839215	57.6660265	64.3478557	33.22625272	37.98595695	54.4188695	49.66575776	43.98291127
**ENSG00000132972**	RNF17	260.1526858	175.1602035	250.7838316	232.7509555	223.067267	208.2805826	239.0063212	229.2611438	251.3638633	270.8287924	204.1814486	267.2807685
**ENSG00000133019**	CHRM3	251.7153014	346.817203	271.9538953	352.4753679	456.5366346	292.5033318	268.6267627	217.6319553	232.605366	225.9015862	227.6347231	220.7603816
**ENSG00000133026**	MYH10	1916.692491	1478.352118	1316.615116	3084.368777	2995.804954	2437.527765	2062.195566	3242.882266	2758.437022	2586.161857	3453.149769	3731.780856
**ENSG00000133027**	PEMT	115.3109202	101.592918	109.9214846	95.44463643	122.5136285	122.1609246	87.83993	73.09775599	103.1717349	112.0016268	66.22101035	103.1906764
**ENSG00000133028**	SCO1	1227.639431	928.3490787	946.139001	766.906026	632.2165547	721.5840948	819.158417	867.2051961	896.6561689	748.5758445	754.6435971	821.2962855
**ENSG00000133030**	MPRIP	6366.006534	5601.623309	6096.164114	7889.252711	6713.978	7598.257755	6673.791891	4706.498698	6725.859192	7295.292401	5661.896385	6762.372608
**ENSG00000133056**	PIK3C2B	324.8392996	231.2114686	201.9298384	436.1987331	397.5913982	396.4539322	671.0562094	719.3483714	735.3330925	771.9886139	812.5869812	669.8935717
**ENSG00000133059**	DSTYK	482.3371418	542.9966309	519.480794	461.3157427	606.7891978	600.5613155	319.6964894	294.0523366	286.0670832	317.6543313	326.9662386	378.9296971
**ENSG00000133065**	SLC41A1	763.5832886	1057.967629	1047.918153	884.9559711	980.1090281	1060.751382	419.7931538	641.2666775	482.562342	396.1187478	618.0627633	444.0582388
**ENSG00000133069**	TMCC2	5.624922937	35.0320407	26.05546302	18.41914036	33.5178795	18.58970591	0	3.322625272	2.813774589	0	6.898021912	3.383300867
**ENSG00000133083**	DCLK1	0	3.50320407	2.442699658	0	6.93473369	1.13814526	12.25673442	3.322625272	43.14454369	15.18666126	24.83287888	61.74524082
**ENSG00000133103**	COG6	465.462373	469.4293454	509.7099954	493.1306215	498.1450367	537.2045627	513.761451	646.2506155	566.5066172	573.2964625	544.943731	669.0477465
**ENSG00000133104**	SPART	1328.888044	1282.17269	1283.231554	815.4655779	842.5701433	884.7182487	969.3034136	1151.289657	963.7177966	896.0130142	856.7343214	888.1164776
**ENSG00000133106**	EPSTI1	2768.868316	3135.367643	2484.225553	380.1040784	570.9597404	352.8250306	1036.715453	1506.810561	866.1736109	69.60553076	107.6091418	75.27844429
**ENSG00000133107**	TRPC4	64.68661377	91.08330583	105.0360853	160.7488614	181.4588649	181.3444781	3.064183605	3.322625272	9.848211061	1.898332657	4.138813147	21.99145564

**ENSG00000133110**	POSTN	0	0	0	2.511700959	0	2.655672273	0	0	0.937924863	1.265555105	0	0
**ENSG00000133111**	RFXAP	133.5919197	38.53524477	109.9214846	87.0722999	90.15153796	91.43100255	146.0594185	127.921073	145.8473162	127.188288	126.9236032	144.6361121
**ENSG00000133112**	TPT1	19415.82775	25394.72631	19601.03629	13339.64379	15315.35935	13501.43784	12196.47214	15883.81011	11799.56374	10623.06955	13034.5022	11841.55303
**ENSG00000133114**	GPALPP1	437.3377583	486.9453658	445.385571	376.7551438	469.250313	426.045709	530.1037636	450.2157244	492.410553	468.2553888	482.8615338	506.6493048
**ENSG00000133119**	RFC3	621.5539845	528.9838146	601.7183492	798.7209048	754.7301832	901.7904276	770.1314793	548.2331699	807.553307	829.5713712	618.0627633	924.4869619
**ENSG00000133121**	STARD13	777.645596	550.0030391	685.5843708	905.8868124	738.5491379	821.3614959	1042.84382	740.9454357	738.1468671	1061.800733	659.4508947	688.5017265
**ENSG00000133124**	IRS4	4.218692202	0	4.885399317	1.674467306	8.090522638	3.035054026	2.04278907	0	1.406887294	3.163887762	2.759208765	1.691650434
**ENSG00000133131**	MORC4	248.9028399	255.7338971	282.5389271	252.0073295	290.103026	287.1919873	1653.637752	1305.791732	1720.154199	1945.790974	1941.103366	2131.479546
**ENSG00000133134**	BEX2	39.37446056	63.05767327	63.51019112	61.11805666	50.85471372	69.80624261	197.1291452	244.2129575	196.0262963	238.5571372	205.561053	219.0687311
**ENSG00000133135**	RNF128	1.406230734	0	0.814233219	0	8.090522638	4.55258104	0	0	0	0	0	1.691650434
**ENSG00000133138**	TBC1D8B	134.9981505	374.8428355	187.2736405	140.6552537	273.9219807	212.8331636	170.5728873	295.7136492	152.4127902	203.1215943	205.561053	177.6232955
**ENSG00000133142**	TCEAL4	727.0212896	588.5382838	656.2719749	1006.354851	884.1785454	727.6542028	1526.98483	1523.423687	1250.253842	1869.857667	1867.984334	1634.134319
**ENSG00000133193**	FAM104A	537.1801404	427.3908966	515.4096279	472.1997802	619.5028763	550.482924	494.3549549	581.4594226	541.1826459	476.4814969	565.6377967	489.7328005
**ENSG00000133195**	SLC39A11	146.2479964	94.5865099	161.2181775	124.7478143	127.1367843	142.6475392	249.2202665	225.9385185	240.5777273	270.8287924	233.1531406	303.6512528
**ENSG00000133216**	EPHB2	40.78069129	10.50961221	40.71166097	61.11805666	67.035759	80.04954995	4.08557814	31.56494009	3.751699452	12.65555105	20.69406573	9.304077385
**ENSG00000133226**	SRRM1	3612.606756	3191.418908	3322.885769	3258.513377	2777.360843	2910.23743	3470.69863	3521.982789	3109.689883	3628.979263	3435.214912	3450.966884
**ENSG00000133243**	BTBD2	447.1813735	486.9453658	419.330108	370.8945082	456.5366346	456.3962492	538.2749199	463.5062255	531.8033973	418.2659621	495.2779732	448.2873649
**ENSG00000133247**	KMT5C	75.93645964	84.07689769	44.78282707	95.44463643	78.59364848	71.32376962	82.73295732	48.17806645	66.12370283	79.09719405	99.33151553	51.59533822
**ENSG00000133250**	ZNF414	47.81184496	45.54165292	32.56932878	43.53614995	38.14103529	34.9031213	57.19809395	44.85544118	52.05482989	50.62220419	51.04536215	41.44543562
**ENSG00000133265**	HSPBP1	192.6536106	185.6698157	230.4280011	277.9615728	290.103026	314.1280917	160.358942	93.03350762	182.8953483	157.5616105	143.4788558	192.0023242
**ENSG00000133275**	CSNK1G2	520.3053716	592.0414879	499.1249635	504.014659	521.2608157	466.6395566	471.8842751	574.8141721	445.9832723	487.2387153	434.5753804	455.0539666
**ENSG00000133302**	SLF1	580.7732932	777.7113036	606.6037485	609.5060993	622.9702431	667.3325041	810.9872607	863.8825708	705.3194969	675.8064259	840.1790688	814.5296838
**ENSG00000133313**	CNDP2	2877.148082	2764.028012	3093.272001	1506.183342	1408.906728	1485.279564	3330.767578	3337.577086	2936.642746	2370.384711	2509.500371	1991.918386
**ENSG00000133315**	MACROD1	203.9034565	140.1281628	106.6645517	184.1914036	247.3388349	199.5548022	126.6529223	149.5181373	153.8196775	168.3188289	139.3400426	176.7774703
**ENSG00000133316**	WDR74	473.8997574	325.7979785	459.2275358	487.269986	390.6566645	516.717948	394.2582905	255.842146	424.4110005	364.4798702	288.3373159	419.5293075
**ENSG00000133318**	RTN3	2788.555546	3110.845215	2678.013059	3267.722947	3493.949991	3084.373654	2742.444326	2937.200741	2762.188721	3099.344452	3166.192057	3158.311359
**ENSG00000133321**	RARRES3	1468.104886	1485.358526	1490.046792	44.3733836	105.1767943	160.0990999	1247.122727	1996.897789	1234.778082	205.019927	304.8925685	115.0322295
**ENSG00000133328**	HRASLS2	52.03053716	49.04485699	49.66822639	8.372336529	6.93473369	10.62268909	41.87717593	71.43644335	56.74445421	18.98332657	23.4532745	18.60815477
**ENSG00000133392**	MYH11	9.843615139	0	5.699632536	0	0	3.41443578	0	0	0.468962431	2.53111021	8.277626294	0.845825217
**ENSG00000133393**	FOPNL	645.459907	416.8812844	548.7931899	738.4400818	628.7491879	720.4459495	496.3977439	461.8449128	450.2039342	504.9564868	456.6490505	530.3324109
**ENSG00000133398**	MED10	594.8356005	693.6344059	653.015042	552.5742109	555.9344841	676.4376661	662.8850531	662.8637418	737.2089422	621.3875564	557.3601705	668.2019213
**ENSG00000133401**	PDZD2	639.834984	220.7018564	363.9622491	597.7848281	247.3388349	352.8250306	11328.28679	11517.88051	10597.14406	11270.40099	12477.14203	10135.52357
**ENSG00000133422**	MORC2	1715.601496	1390.772016	1696.862029	1699.584315	1546.445613	1651.448772	1125.576777	795.7687527	1217.895434	1223.791786	909.1592879	1277.196077
**ENSG00000133424**	LARGE1	9.843615139	21.01922442	13.84196473	21.76807497	18.49262317	25.41857747	96.01108628	137.8889488	77.37880119	139.8438391	103.4703287	85.42834689
**ENSG00000133433**	GSTT2B	28.12461468	49.04485699	13.84196473	14.2329721	8.090522638	11.00207085	5.106972674	1.661312636	13.13094808	7.593330629	6.898021912	10.1499026
**ENSG00000133454**	MYO18B	16.87476881	35.0320407	48.85399317	22.60530863	32.36209055	28.83301325	132.7812895	109.646634	124.2750443	110.1032941	110.3683506	84.58252168
**ENSG00000133460**	SLC2A11	140.6230734	77.07048955	84.68025482	123.073347	130.6041512	103.9506004	152.1877857	129.5823856	102.7027725	189.8332657	150.3768777	119.2613556
**ENSG00000133466**	C1QTNF6	1223.420739	1071.980446	1067.459751	1835.216167	1525.641412	1466.689858	1901.836624	1704.506765	2048.427901	2371.650266	1909.372465	2210.141291
**ENSG00000133477**	FAM83F	18.28099954	24.52242849	6.513865756	13.39573845	36.98524634	27.31548624	26.55625791	11.62918845	34.2342575	18.98332657	13.79604382	43.13708606
**ENSG00000133519**	ZDHHC8P1	1.406230734	0	10.58503185	25.95424324	18.49262317	13.65774312	177.7226491	277.4392102	158.9782643	189.2004882	400.0852709	157.3234903
**ENSG00000133561**	GIMAP6	0	0	0	0	3.467366845	0.379381753	6.128367209	3.322625272	0	0.632777552	0	0.845825217
**ENSG00000133597**	ADCK2	161.7165344	154.1409791	177.5028418	188.3775719	238.0925233	244.3218491	255.3486337	302.3588998	276.6878346	264.5010169	270.4024589	239.3685363
**ENSG00000133606**	MKRN1	2105.127409	2371.669156	2073.037777	1723.864091	2135.897976	1698.112728	1857.916659	2224.49762	1735.629959	1597.130542	1920.4093	1673.042279
**ENSG00000133612**	AGAP3	648.2723684	518.4742024	565.0778543	635.4603425	498.1450367	650.6397069	521.9326073	348.8756536	572.6031288	518.2448154	438.7141936	547.2489153
**ENSG00000133619**	KRBA1	118.1233817	126.1153465	109.1072514	108.8403749	138.6946738	133.1629954	130.7385005	144.5341993	130.3715559	149.3355024	157.2748996	104.0365017
**ENSG00000133624**	ZNF767P	286.8710698	252.2306931	290.6812593	263.7286007	227.6904228	231.4228695	371.7876107	440.2478486	344.6873871	361.94876	420.7793366	291.8096998
**ENSG00000133627**	ACTR3B	97.02992066	28.02563256	83.8660216	104.6542066	124.8252064	80.4289317	134.8240786	81.40431917	126.6198565	153.7649452	148.9972733	114.1864043
**ENSG00000133639**	BTG1	2489.028399	4116.264783	2939.381922	1286.828124	2603.9925	2123.779055	1959.034718	2938.862053	1819.105272	1776.20659	2472.251053	1793.14946
**ENSG00000133640**	LRRIQ1	12.65607661	49.04485699	1.628466439	15.07020575	23.11577897	10.62268909	13.27812895	4.983937908	6.096511609	7.593330629	1.379604382	6.766601734
**ENSG00000133641**	C12orf29	324.8392996	332.8043867	323.2505881	365.8711063	301.6609155	363.8271014	284.9690752	222.6158932	288.8808578	323.3493293	267.6432502	338.3300867
**ENSG00000133657**	ATP13A3	6091.79154	4736.331903	5452.919871	4283.287368	3804.857218	4432.696406	6655.406789	5523.864515	7022.243449	5149.543721	4060.175697	5699.170311
**ENSG00000133678**	TMEM254	269.996301	276.7531216	269.5111956	394.3370505	365.2293077	307.2992202	710.8905963	618.0083006	620.4372968	813.1191548	768.4396409	784.079976
**ENSG00000133687**	TMTC1	1.406230734	0	0.814233219	1.674467306	0	2.27629052	0	1.661312636	0.937924863	1.898332657	1.379604382	1.691650434
**ENSG00000133703**	KRAS	240.4654555	252.2306931	166.91781	280.4732737	263.5198802	287.1919873	628.1576389	498.3937908	515.8586746	582.1553482	451.130633	691.0392021
**ENSG00000133704**	IPO8	442.9626813	441.4037129	408.7450762	462.99021	466.9387351	511.0272217	700.6766509	672.8316176	745.1813036	779.5819445	698.0798174	836.5211394
**ENSG00000133706**	LARS	3919.165056	2932.181807	3782.927538	3832.855663	3640.735187	3837.446435	4177.503648	3601.725795	4183.144889	4042.183005	3356.577462	4098.869
**ENSG00000133710**	SPINK5	42.18692202	63.05767327	54.5536257	74.51379511	106.3325832	77.39387767	140.9524458	177.7604521	125.6819316	180.3416024	180.7281741	142.9444616
**ENSG00000133731**	IMPA1	579.3670625	402.8684681	716.5252331	493.9678552	446.134534	546.6891065	402.4294467	433.602598	464.2728071	357.5193171	311.7905904	373.0089206
**ENSG00000133739**	LRRCC1	826.8636717	854.7817932	759.6795937	801.2326058	899.2038017	636.9819638	1093.913547	912.0606372	932.2973137	985.2346491	865.0119477	980.3114262
**ENSG00000133740**	E2F5	257.3402244	189.1730198	170.9889761	121.3988797	80.90522638	94.84543833	70.47622291	33.22625272	68.93747742	66.441643	26.21248326	65.12854169
**ENSG00000133773**	CCDC59	393.7446056	371.3396315	331.3929203	329.0328256	283.1682923	312.231183	479.0340369	440.2478486	369.0734336	425.8592928	394.5668533	421.220958
**ENSG00000133789**	SWAP70	1217.795816	1516.887362	1217.278663	1285.153657	1623.883472	1398.780524	2229.70427	2737.843224	2236.012873	2363.424158	2699.885776	2486.726137
**ENSG00000133794**	ARNTL	731.2399818	767.2016914	661.1573742	797.8836712	613.7239315	654.4335245	598.5371974	848.9307571	587.1409642	551.7820257	682.9041692	562.4737692
**ENSG00000133805**	AMPD3	812.8013643	1485.358526	946.139001	749.3241193	1255.186798	806.5656075	204.278907	807.3979412	234.9501782	277.1565679	867.7711565	227.5269833
**ENSG00000133812**	SBF2	1051.860589	928.3490787	954.2813332	1069.147375	1029.807953	996.2564842	1525.963435	1267.581541	1375.466811	1562.327777	1535.499678	1501.33976
**ENSG00000133816**	MICAL2	3698.386831	3499.700866	3826.081898	3641.129156	2979.623909	3882.213482	2591.277935	2284.304875	3296.336931	2993.037823	2793.698874	3446.737758
**ENSG00000133818**	RRAS2	1593.259422	1359.243179	1349.998678	1321.991938	947.7469376	1108.932865	1207.28834	1405.47049	1312.625846	1188.356243	1267.856427	1327.94559

**ENSG00000133835**	HSD17B4	1245.92043	1380.262404	1273.460755	1414.08764	1341.870969	1320.627883	1554.562482	1752.684831	1569.617258	1588.271656	1800.383719	1856.586351
**ENSG00000133858**	ZFC3H1	1979.972874	1965.297484	1969.630158	1572.3248	1717.502377	1770.574643	2120.415054	2148.077239	2092.979332	1850.241563	1971.454662	2063.813529
**ENSG00000133863**	TEX15	580.7732932	896.820242	533.3227587	806.2560077	917.6964249	653.2953792	24.51346884	24.91968954	24.38604644	41.1305409	31.73090079	27.06640694
**ENSG00000133872**	SARAF	1749.351033	2301.605074	1965.558992	2280.62447	2594.746189	2278.946192	828.3509678	955.2547658	946.3661867	921.3241163	1027.805265	1099.572782
**ENSG00000133874**	RNF122	25.31215321	38.53524477	37.45472809	5.023401917	38.14103529	17.83094241	0	18.274439	1.875849726	2.53111021	5.518417529	2.53747565
**ENSG00000133884**	DPF2	804.3639799	672.6151815	881.8145767	836.3964192	857.5953996	975.3904877	792.6021591	805.7366285	878.3666341	828.3058161	880.1875959	799.3048299
**ENSG00000133895**	MEN1	334.6829147	311.7851623	280.9104607	366.70834	313.218805	342.5817232	430.0070992	340.5690904	414.5627894	404.9776335	384.9096227	444.0582388
**ENSG00000133935**	ERG28	262.9651473	322.2947745	232.0564675	289.6828439	316.6861718	294.0208588	423.878732	608.0404248	453.9556336	403.7120784	536.6661047	450.8248405
**ENSG00000133943**	DGLUCY	494.9932184	644.589549	554.4928224	208.4711796	264.6756692	188.9321131	434.0926773	440.2478486	390.6457054	194.2627086	172.4505478	149.7110634
**ENSG00000133958**	UNC79	5.624922937	0	0.814233219	0	11.55788948	2.655672273	8.171156279	4.983937908	14.06887294	10.12444084	15.17564821	10.99572782
**ENSG00000133961**	NUMB	527.3365253	830.2593647	681.5132047	657.2284175	754.7301832	677.5758114	782.3882137	927.012451	772.3811246	716.9369668	910.5388923	708.8015317
**ENSG00000133983**	COX16	8.437384405	14.01281628	10.58503185	11.72127114	17.33683422	12.14021611	25.53486337	8.306563181	22.97915914	22.14721433	20.69406573	16.91650434
**ENSG00000133985**	TTC9	179.997534	217.1986524	227.9853014	293.0317785	312.063016	397.9714592	290.0760479	232.5837691	252.7707506	256.9076863	280.0596896	259.6683416
**ENSG00000133997**	MED6	359.9950679	399.365264	382.6896131	333.2189938	350.2040513	347.513686	490.2693767	488.425915	436.1350613	449.9048397	492.5187645	491.4244509
**ENSG00000134001**	EIF2S1	2214.813406	1783.130872	2214.714357	2441.373332	2061.927484	2148.438869	2101.008558	2204.561868	2307.295163	2312.169176	2084.582222	2557.775456
**ENSG00000134007**	ADAM20	56.24922937	45.54165292	52.11092604	14.2329721	26.58314581	23.14228695	36.77020326	38.21019063	31.88944534	30.37332251	45.52694462	34.67883389
**ENSG00000134013**	LOXL2	16.87476881	35.0320407	27.68392946	36.83828073	169.9009754	42.49075637	8.171156279	166.1312636	27.19982102	44.92720622	165.5525259	15.2248539
**ENSG00000134014**	ELP3	757.9583657	679.6215897	826.4467177	688.2060627	708.4986253	712.099551	384.0443451	347.214341	386.8940059	369.5420906	409.7425015	373.8547458
**ENSG00000134020**	PEBP4	5.624922937	0	1.628466439	1.674467306	0	0	0	4.983937908	0.937924863	0	0	0
**ENSG00000134030**	CTIF	295.3084542	217.1986524	355.8199169	417.7795928	330.5556392	416.9405469	179.7654381	235.9063943	183.3643107	155.6632779	209.6998661	131.9487338
**ENSG00000134042**	MRO	0	0	0	0.837233653	8.090522638	1.13814526	0	0	0	0	0	0
**ENSG00000134046**	MBD2	1591.853191	1548.416199	1481.904459	1767.400241	1705.944488	1592.6446	2885.439561	2656.438905	2871.456968	3096.813341	2784.041643	3169.307087
**ENSG00000134049**	IER3IP1	400.7757592	444.9069169	388.3892457	406.8955553	360.6061519	430.9776718	318.6750949	269.1326471	361.1010722	394.2204151	322.8274255	414.4543562
**ENSG00000134056**	MRPS36	302.3396078	325.7979785	308.5943902	303.0785823	312.063016	290.606423	313.5681222	234.2450817	294.508407	283.4843435	350.4195131	350.1716397
**ENSG00000134057**	CCNB1	3206.206074	1233.127833	2713.83932	4014.535365	3377.215307	4054.073416	2323.672567	1528.407625	2770.161083	2543.132983	1852.808685	3222.594076
**ENSG00000134058**	CDK7	1001.236283	949.3683031	910.3127393	888.3049057	714.27757	980.3224505	793.6235536	662.8637418	760.1881014	790.9719405	622.2015764	859.3584202
**ENSG00000134061**	CD180	32.34330689	7.006408141	42.34012741	37.67551438	41.60840214	73.60006014	3.064183605	4.983937908	2.813774589	2.53111021	2.759208765	5.074951301
**ENSG00000134070**	IRAK2	255.9339936	602.5511001	460.8560022	137.3063191	269.2988249	301.6084939	38.81299233	112.9692593	58.1513415	60.11386748	68.98021912	62.59106604
**ENSG00000134072**	CAMK1	2.812461468	3.50320407	6.513865756	3.348934611	12.71367843	4.931962793	0	0	0.468962431	0	0	0.845825217
**ENSG00000134077**	THUMPD3	725.6150588	465.9261414	646.5011762	663.9262867	693.473369	615.7365856	722.1259362	848.9307571	742.8364914	701.117528	706.3574437	804.3797812
**ENSG00000134086**	VHL	600.4605235	683.1247937	714.0825335	545.039108	618.3470873	592.5942987	928.4476322	898.7701361	888.2148452	893.481904	887.0856178	985.3863775
**ENSG00000134107**	BHLHE40	555.46114	1621.983485	800.3912547	341.5913304	1371.921482	582.3509913	673.0989985	1986.929913	713.7608207	702.3830831	2269.449209	631.8314369
**ENSG00000134108**	ARL8B	667.9595987	718.1568344	647.3154095	537.5040051	648.3976	659.3654872	1212.395313	1069.885338	1079.551517	1127.609598	1147.830846	1058.973171
**ENSG00000134109**	EDEM1	2529.809091	2855.111317	2800.148042	1620.047118	2085.043263	2009.585147	2276.688418	2493.630267	2335.432909	1970.469298	2214.265034	2223.674495
**ENSG00000134115**	CNTN6	0	0	0	0	0	0	5.106972674	0	0	1.898332657	0	1.691650434
**ENSG00000134138**	MEIS2	399.3695285	381.8492437	324.0648213	452.1061725	486.5871472	412.0085841	265.5625791	304.0202124	206.8124323	267.0321271	321.4478211	262.2058172
**ENSG00000134146**	DPH6	213.7470716	129.6185506	209.2579374	248.6583949	180.3030759	175.27437	282.9262862	206.0027669	232.605366	288.5465639	209.6998661	231.7561094
**ENSG00000134152**	KATNBL1	645.459907	669.1119775	606.6037485	556.7603792	475.0292577	598.285025	576.0665177	523.3134804	536.961984	525.2053685	511.8332258	497.3452275
**ENSG00000134153**	EMC7	523.1178331	528.9838146	598.4614163	534.9923042	580.206052	653.6747609	375.8731888	455.1996623	461.927995	414.4692968	412.5017103	477.8912475
**ENSG00000134160**	TRPM1	5.624922937	0	0	1.674467306	0	0.379381753	0	4.983937908	0.937924863	0.632777552	0	5.074951301
**ENSG00000134183**	GNAT2	2.812461468	0	4.071166097	4.186168264	3.467366845	0	4.08557814	11.62918845	1.875849726	0.632777552	1.379604382	1.691650434
**ENSG00000134186**	PRPF38B	1622.790267	1191.089384	1411.880403	1613.349249	1280.614155	1489.452763	1586.225713	1536.714188	1637.616811	1667.368851	1498.250359	1684.883832
**ENSG00000134198**	TSPAN2	71.71776744	31.52883663	29.3123959	85.39783259	76.28207059	89.15471203	8.171156279	8.306563181	13.13094808	24.04554699	12.41643944	7.612426951
**ENSG00000134201**	GSTM5	0	0	0	0	0	0	31.66323058	46.51675381	21.10330942	44.92720622	53.80457091	32.98718345
**ENSG00000134202**	GSTM3	0	3.50320407	0.814233219	3.348934611	6.93473369	3.793817533	92.94690267	132.9050109	90.04078684	124.6571778	93.813098	97.26989993
**ENSG00000134207**	SYT6	64.68661377	122.6121425	105.8503185	119.7244124	216.1325333	140.7506305	1.021394535	3.322625272	6.56547404	1.265555105	1.379604382	6.766601734
**ENSG00000134215**	VAV3	247.4966092	122.6121425	113.9926507	264.5658343	210.3535886	122.1609246	2784.321502	2275.998311	2013.255718	2891.793414	2243.236726	2488.417788
**ENSG00000134222**	PSRC1	341.7140684	122.6121425	307.780157	382.6157794	308.5956492	340.3054327	327.8676457	202.6801416	341.4046501	332.208215	224.8755143	365.3964936
**ENSG00000134240**	HMGCS2	0	0	0	0	0	0	3.064183605	0	5.158586746	5.062220419	1.379604382	6.766601734
**ENSG00000134242**	PTPN22	1.406230734	7.006408141	5.699632536	0.837233653	5.778944741	3.41443578	2.04278907	4.983937908	1.406887294	0	1.379604382	0
**ENSG00000134243**	SORT1	2190.907484	2942.691419	3024.87641	2382.766976	2702.234561	2630.253696	3208.200234	3885.810256	3136.420742	3306.895489	3789.773238	3421.363002
**ENSG00000134245**	WNT2B	101.2486129	168.1537954	74.09522297	96.28187008	105.1767943	70.18562436	56.17669942	86.38825708	49.71001773	45.55998377	71.73942788	16.91650434
**ENSG00000134247**	PTGFRN	3241.361842	3187.915704	2888.085229	3500.473903	3163.394351	3261.544933	2385.977633	2410.564635	2667.45831	2244.461978	2212.885429	2614.445745
**ENSG00000134248**	LAMTOR5	487.9620648	655.0991612	593.576017	658.9028848	647.241811	697.3036626	596.4944084	521.6521677	553.8446315	592.279789	540.8049179	650.4395917
**ENSG00000134250**	NOTCH2	545.6175249	805.7369362	876.9291773	596.1103608	911.9174802	1052.025602	157.2947584	114.6305719	145.8473162	151.8666126	132.4420207	142.9444616
**ENSG00000134253**	TRIM45	63.28038304	77.07048955	44.78282707	87.90953355	99.39784955	50.45777319	48.00554314	126.2597603	63.30992825	77.83163894	108.9887462	65.12854169
**ENSG00000134255**	CEPT1	798.739057	826.7561606	810.9762866	916.7708499	798.6501633	893.8234108	929.4690267	990.1423311	976.8487447	1063.699066	916.0573099	1172.31375
**ENSG00000134256**	CD101	53.4367679	45.54165292	46.41129351	82.04889798	52.01050267	45.5258104	42.89857046	83.06563181	28.60670832	37.96665314	15.17564821	33.83300867
**ENSG00000134258**	VTCN1	0	0	2.442699658	1.674467306	3.467366845	3.035054026	23.4920743	18.274439	3.751699452	48.09109398	45.52694462	8.458252168
**ENSG00000134259**	NGF	74.53022891	31.52883663	26.05546302	77.02549606	33.5178795	72.08253313	37.79159779	63.12988017	58.1513415	24.67832454	41.38813147	38.06213475
**ENSG00000134262**	AP4B1	312.183223	227.7082646	327.3217542	277.9615728	225.3788449	303.5054026	274.7551299	169.4538889	200.7159207	239.8226924	176.5893609	228.3728085
**ENSG00000134265**	NAPG	839.5197483	865.2914054	890.7711421	799.5581385	646.0860221	897.6172283	715.9975689	593.0886111	603.5546493	642.9019932	630.4792027	728.2555116
**ENSG00000134278**	SPIRE1	1137.640664	1019.432384	1423.279668	1491.950369	1425.087773	1656.760117	705.7836236	769.1877505	944.0213745	734.0219608	744.9863664	1041.210842
**ENSG00000134283**	PPHLN1	561.0860629	511.4677943	509.7099954	599.4592955	477.3408356	538.7220897	741.5324323	662.8637418	696.8781731	811.2208222	722.9126963	862.7417211
**ENSG00000134285**	FKBP11	254.5277629	241.7210809	233.684934	175.8190671	203.4188549	190.0702584	235.9421376	284.0844608	190.3987472	206.2854821	275.9208765	204.6897025
**ENSG00000134287**	ARF3	2151.533023	1891.730198	2095.022074	1614.186483	1711.723432	1654.104444	1967.205874	1844.057026	2008.097131	1719.25661	1851.429081	1732.250044
**ENSG00000134291**	TMEM106C	1998.253873	2217.528177	2112.935204	2711.799802	2237.607404	2555.51549	2087.730429	2121.496236	2261.336844	2464.035789	2254.273561	2522.250796

**ENSG00000134294**	SLC38A2	16548.52328	13988.29385	15312.46992	14891.03775	16006.52114	15274.28877	16284.09307	18855.89842	16246.73448	14269.13381	15984.09637	15871.06437
**ENSG00000134297**	PLEKHA8P1	30.93707615	49.04485699	29.3123959	46.88508456	90.15153796	58.04540826	72.51901198	89.71088235	53.93067962	87.32330223	110.3683506	75.27844429
**ENSG00000134308**	YWHAQ	9705.804527	9556.740704	8442.784252	11511.12549	10658.68568	10409.09717	6691.155598	7455.971111	6235.793451	7031.424162	7888.577858	7573.518991
**ENSG00000134313**	KIDINS220	1774.663187	1762.111647	1555.185449	1743.957699	1955.5949	1944.710867	2039.724886	2355.741318	1903.987472	1943.892641	2058.369738	2149.241876
**ENSG00000134317**	GRHL1	701.7091363	1156.057343	731.9956643	909.235747	953.5258823	835.019239	1216.480891	1161.257533	1010.61404	1084.580725	1270.615636	969.3156984
**ENSG00000134318**	ROCK2	2226.063252	2714.983155	2560.763475	2228.715984	2610.927234	2508.092771	2167.399203	2653.11628	2119.71019	2042.605939	2392.233999	2221.137019
**ENSG00000134321**	RSAD2	899.9876699	2497.784502	1101.657546	38.51274803	90.15153796	55.38973598	115.4175824	249.1968954	148.1921283	17.71777147	35.86971394	12.68737825
**ENSG00000134323**	MYCN	1.406230734	14.01281628	18.72736405	26.79147689	9.246311586	17.0721789	80.69016826	172.7765142	121.9302322	74.66775118	180.7281741	85.42834689
**ENSG00000134324**	LPIN1	333.276684	627.0735286	376.9899806	284.659442	457.6924235	338.7879057	939.6829721	1558.311253	813.1808561	989.0313144	1730.023895	892.3456037
**ENSG00000134326**	CMPK2	532.9614482	1142.044527	533.3227587	8.372336529	39.29682424	17.83094241	85.79714093	91.37219499	86.28908739	32.27165517	22.07367012	16.07067912
**ENSG00000134330**	IAH1	234.8405326	262.7403053	280.0962275	289.6828439	303.9724934	246.5981396	379.958767	451.877037	372.825133	375.2370886	413.8813147	385.6962988
**ENSG00000134333**	LDHA	39460.24063	64847.81055	35241.6422	42832.03645	60432.73674	40479.27431	32597.80658	79178.16024	30577.75742	31346.53439	66327.23989	33501.44519
**ENSG00000134339**	SAA2	75.93645964	140.1281628	118.87805	4.186168264	9.246311586	16.69279715	17.36370709	23.25837691	22.51019671	1.898332657	2.759208765	9.304077385
**ENSG00000134343**	ANO3	97.02992066	70.06408141	91.19412058	21.76807497	35.8294574	19.34846942	200.1933288	149.5181373	167.8885505	103.7755186	99.33151553	77.81591994
**ENSG00000134352**	IL6ST	4019.007438	5895.892451	5080.001056	3347.260144	4290.288576	4552.58104	3119.33891	4581.90025	3189.413496	2736.762914	3846.337018	2980.688064
**ENSG00000134363**	FST	264.371378	161.1473872	222.2856689	210.1456469	114.4231059	163.5135357	2.04278907	0	2.344812157	0.632777552	0	0
**ENSG00000134365**	CFHR4	0	0	3.256932878	0.837233653	1.155788948	1.13814526	0	0	0	0	1.379604382	0
**ENSG00000134369**	NAV1	2460.903785	1208.605404	1977.77249	3275.25805	2231.828459	2739.515641	5280.609745	4453.979177	5522.970555	5107.780403	4945.881711	5078.334602
**ENSG00000134371**	CDC73	2095.283794	2070.393606	1994.057154	2203.598974	1830.769694	1997.065549	1465.701158	1835.750463	1555.548385	1552.203336	1640.349611	1708.566938
**ENSG00000134375**	TIMM17A	1077.172742	928.3490787	1074.78785	1285.153657	1013.626908	1209.089648	642.4571624	707.719183	697.3471356	756.8019526	553.2213573	797.6131794
**ENSG00000134419**	RPS15A	1733.882495	1583.44824	1491.675258	1635.954558	1666.647663	1566.846641	1428.930954	1653.006073	1496.459119	1452.224483	1352.012295	1574.926554
**ENSG00000134440**	NARS	4734.778882	3807.982825	5014.048165	4381.243705	4331.896978	4906.923597	3958.925217	3405.690904	4142.814119	3614.425379	3010.296762	4048.119487
**ENSG00000134444**	KIAA1468	1037.798282	998.4131601	1174.938536	910.9102143	1000.913229	1042.161676	1466.722552	1498.503998	1404.07352	1434.506711	1305.105746	1329.637241
**ENSG00000134452**	FBXO18	445.7751427	574.5254675	463.2987019	473.8742475	351.3598403	434.7714893	427.9643101	561.523671	440.3557231	384.7287518	496.6575776	397.5378519
**ENSG00000134453**	RBM17	1188.26497	1215.611812	1044.661221	1151.196273	1022.873219	958.6976906	1262.443645	1638.054259	1113.785775	1030.161855	1208.533439	1057.281521
**ENSG00000134461**	ANKRD16	26.71838395	0	38.26896131	35.16381342	30.05051265	37.93817533	38.81299233	63.12988017	49.71001773	27.8422123	42.76773585	53.28698866
**ENSG00000134470**	IL15RA	246.0903785	273.2499175	315.9224891	29.30317785	45.07576898	31.48868552	157.2947584	262.4873965	163.1989261	41.1305409	63.46180159	23.68310607
**ENSG00000134480**	CCNH	836.7072868	585.0350798	738.50953	884.9559711	745.4838716	894.5821743	855.9286202	611.3630501	723.6090317	796.6669384	771.1988497	895.7289046
**ENSG00000134489**	HRH4	0	3.50320407	0.814233219	0	1.155788948	1.517527013	0	1.661312636	0.468962431	3.163887762	0	0.845825217
**ENSG00000134490**	TMEM241	257.3402244	119.1089384	182.3882412	196.7499084	129.4483622	218.1445082	253.3058446	98.01744553	236.3570655	232.8621393	158.654504	225.8353329
**ENSG00000134504**	KCTD1	156.0916115	87.58010176	96.89375311	156.5626931	176.8357091	141.8887757	40.8557814	36.54887799	26.26189616	37.96665314	63.46180159	43.13708606
**ENSG00000134508**	CABLES1	1.406230734	10.50961221	1.628466439	10.88403749	5.778944741	4.55258104	85.79714093	156.1633878	273.4050975	125.9227329	129.6828119	255.4392155
**ENSG00000134516**	DOCK2	29.53084542	84.07689769	79.79485551	46.88508456	85.52838217	85.36089449	1.021394535	0	0.468962431	0.632777552	2.759208765	4.229126084
**ENSG00000134531**	EMP1	2528.40286	1898.736606	2388.146033	3893.973719	2426.001002	3489.553367	556.6600215	815.7045043	921.0422154	504.9564868	612.5443457	940.5576411
**ENSG00000134533**	RERG	59.06169083	38.53524477	53.73939248	33.48934611	33.5178795	38.31755708	108.2678207	96.3561329	106.4544719	95.54941041	51.04536215	72.74096864
**ENSG00000134539**	KLRD1	2.812461468	7.006408141	3.256932878	0.837233653	9.246311586	6.070108053	7.149761744	3.322625272	3.28273702	1.265555105	1.379604382	2.53747565
**ENSG00000134569**	LRP4	71.71776744	10.50961221	35.82626166	99.63080469	45.07576898	65.25366157	7.149761744	14.95181373	24.85500887	22.77999189	23.4532745	44.82873649
**ENSG00000134574**	DDB2	352.9639143	430.8941007	409.5593094	348.2891996	476.1850467	343.3404867	667.9920258	621.3309259	543.5274581	666.3147627	765.6804322	605.6108552
**ENSG00000134575**	ACP2	746.7085198	770.7048955	854.1306472	504.8518927	419.5513882	534.5488904	603.6441701	553.2171078	639.1957941	453.7015051	329.7254474	497.3452275
**ENSG00000134588**	USP26	2.812461468	0	4.885399317	1.674467306	1.155788948	1.13814526	5.106972674	0	0	1.898332657	0	3.383300867
**ENSG00000134590**	RTL8C	184.2162262	238.2178768	187.2736405	213.4945815	284.3240813	261.7734098	156.2733638	192.7122658	145.3783538	156.928833	270.4024589	194.5397999
**ENSG00000134594**	RAB33A	4.218692202	17.51602035	4.071166097	2.511700959	1.155788948	9.863925586	0	4.983937908	0	3.796665314	0	3.383300867
**ENSG00000134597**	RBMX2	243.277917	185.6698157	253.2265312	277.9615728	210.3535886	249.6331937	229.8137703	287.407086	200.7159207	229.6982515	229.0143275	243.5976624
**ENSG00000134602**	STK26	314.9956845	385.3524477	355.0056837	380.9413121	401.058765	396.4539322	968.2820191	770.8490632	911.1940043	946.6352184	829.1422338	1152.85977
**ENSG00000134627**	PIWIL4	2.812461468	3.50320407	8.142332194	5.86063557	10.40210053	4.173199286	3.064183605	1.661312636	7.034436472	5.062220419	2.759208765	3.383300867
**ENSG00000134644**	PUM1	2646.526242	2396.191584	2616.131334	2684.171091	2474.544138	2584.348504	2694.438783	2432.161699	2893.02924	2758.910128	2459.834614	2709.178169
**ENSG00000134668**	SPOCD1	2.812461468	3.50320407	4.885399317	1.674467306	6.93473369	1.517527013	0	0	0	0	0	0
**ENSG00000134684**	YARS	4217.285972	3412.120765	4633.801252	3891.462019	3722.796202	4818.90703	3827.165322	2855.796422	4074.345604	3314.488819	2348.086659	3433.204555
**ENSG00000134686**	PHC2	1378.106119	1117.522098	1537.272318	838.0708865	707.3428363	942.763657	1008.116406	882.1570098	1057.979245	751.7397322	756.0232015	749.4011421
**ENSG00000134690**	CDCA8	1106.703588	427.3908966	1051.98932	1481.066332	1269.056265	1549.015699	1158.261403	910.3993246	1364.211713	1393.37617	1122.997967	1510.643837
**ENSG00000134697**	GNL2	2633.870165	2466.255666	2542.850344	2347.603163	2122.028509	2283.119391	1707.771662	1789.233709	1782.526202	1659.77552	1568.610183	1779.616256
**ENSG00000134698**	AGO4	465.462373	648.092753	578.1055858	468.8508456	502.7681925	445.7735601	760.9389285	616.346988	629.8165454	759.9658404	773.9580585	620.8357091
**ENSG00000134709**	HOOK1	943.5808226	935.3554868	781.6638907	946.9112614	872.6206559	875.2337049	1317.59895	1284.194668	1094.558315	1269.984548	1296.828119	1315.258212
**ENSG00000134716**	CYP2J2	26.71838395	91.08330583	13.02773151	2.511700959	20.80420107	9.863925586	554.6172324	1586.553568	312.7979418	460.6620581	1360.289921	320.5677572
**ENSG00000134717**	BTF3L4	1402.012042	1131.534915	1295.445052	1326.178106	1131.51738	1247.786587	1461.615579	1124.708655	1291.522536	1199.113462	929.8533537	1135.097441
**ENSG00000134744**	ZCCHC11	1341.54412	1499.371342	1343.484812	1312.782368	1360.363592	1282.310326	2120.415054	1888.912467	1823.325933	2178.653113	1912.131674	1996.993337
**ENSG00000134748**	PRPF38A	1331.700505	1418.797649	1319.872049	1434.181247	1260.965743	1371.085656	1376.839833	1481.890871	1337.480855	1370.596178	1586.54504	1484.423255
**ENSG00000134755**	DSC2	2647.932472	3951.614191	2536.336479	3621.872782	3974.758193	3236.885119	3560.581349	4364.268295	3096.089972	3906.135831	4842.411382	3949.157937
**ENSG00000134757**	DSG3	234.8405326	178.6634076	184.8309408	298.0551804	138.6946738	252.6682477	5.106972674	16.61312636	61.90304095	27.8422123	26.21248326	57.51611474
**ENSG00000134758**	RNF138	530.1489868	441.4037129	379.4326803	555.9231455	360.6061519	475.3653369	583.2162794	408.6829085	528.9896227	625.1842218	429.0569629	676.6601734
**ENSG00000134759**	ELP2	939.3621304	935.3554868	1071.530917	1005.517617	958.1490381	988.6688491	1045.908004	812.3818791	956.2143977	967.5168776	877.4283871	985.3863775
**ENSG00000134760**	DSG1	2609.964243	1138.541323	1319.872049	8780.906551	5214.919735	5027.187613	2752.658271	1971.978099	3856.747036	2693.73404	1865.225125	4291.71715
**ENSG00000134762**	DSC3	6003.199004	4505.120435	4372.432388	7592.034764	5807.839465	5951.740946	13314.89916	10879.93645	14295.3818	14749.41197	12497.8361	16408.16338
**ENSG00000134765**	DSC1	18.28099954	7.006408141	4.071166097	39.34998168	38.14103529	18.58970591	168.5300983	147.8568246	136.93703	280.3204557	240.0511625	210.610479
**ENSG00000134775**	FHOD3	0	0	0	7.535102876	5.778944741	0.758763507	4.08557814	1.661312636	5.627549177	2.53111021	0	2.53747565
**ENSG00000134779**	TPGS2	835.3010561	942.3618949	721.4106324	992.9591123	1162.723682	940.8667482	848.7788585	1126.369967	925.2628773	972.579098	1095.40588	1000.611231
**ENSG00000134780**	DAGLA	16.87476881	17.51602035	4.071166097	5.023401917	10.40210053	8.725780326	0	0	2.813774589	4.429442867	1.379604382	0

**ENSG00000134802**	SLC43A3	142.0293041	147.134571	201.1156052	121.3988797	159.4988749	131.6454684	10.21394535	4.983937908	10.31717349	3.163887762	11.03683506	2.53747565
**ENSG00000134809**	TIMM10	285.464839	140.1281628	315.1082559	382.6157794	266.987247	372.1735	208.3644851	179.4217647	251.3638633	234.7604719	173.8301522	244.4434876
**ENSG00000134815**	DHX34	307.9645308	210.1922442	328.1359874	291.3573112	225.3788449	338.0291422	271.6909463	252.5195207	354.0666357	276.5237904	240.0511625	282.5056224
**ENSG00000134817**	APLNR	7.031153671	0	4.885399317	3.348934611	0	0.758763507	4.08557814	1.661312636	0	0	0	0.845825217
**ENSG00000134824**	FADS2	1904.036414	2396.191584	1880.878737	1696.235381	2367.055766	1788.026203	1052.036371	2294.27275	1518.031391	1307.318423	2418.446482	1576.618204
**ENSG00000134825**	TMEM258	310.7769922	283.7595297	292.3097258	313.1253862	305.1282823	292.12395	197.1291452	249.1968954	262.6189616	260.071574	306.2721729	330.7176598
**ENSG00000134827**	TCN1	4.218692202	0	0.814233219	5.86063557	0	3.41443578	11.23533988	4.983937908	15.47576024	18.98332657	8.277626294	22.83728085
**ENSG00000134830**	C5AR2	0	0	0	1.674467306	0	3.035054026	0	0	0.468962431	0	0	3.383300867
**ENSG00000134851**	TMEM165	952.018207	1029.941997	955.9097996	823.8379144	740.8607158	901.7904276	922.319265	986.8197059	894.3113568	842.8596998	836.0402557	918.5661854
**ENSG00000134852**	CLOCK	1625.602729	1422.300853	1606.482142	1693.72368	1818.056016	2027.41609	999.9452496	961.9000163	1163.964755	1139.632372	975.3802983	1362.624424
**ENSG00000134853**	PDGFRA	1.406230734	0	0	2.511700959	0	4.931962793	0	0	1.406887294	0	0	0.845825217
**ENSG00000134864**	GGACT	2.812461468	17.51602035	13.84196473	5.023401917	8.090522638	10.62268909	3.064183605	13.29050109	5.627549177	6.327775524	8.277626294	0.845825217
**ENSG00000134871**	COL4A2	0	14.01281628	0.814233219	1.674467306	0	0.758763507	1.021394535	0	0.468962431	0.632777552	0	0
**ENSG00000134874**	DZIP1	23.90592248	14.01281628	45.59706029	22.60530863	27.73893476	73.60006014	3.064183605	6.645250545	5.627549177	5.062220419	5.518417529	5.074951301
**ENSG00000134882**	UBAC2	1056.079281	840.7689769	990.1075948	1118.54416	1002.069018	1116.899882	1306.36361	1428.728867	1534.914038	1423.116715	1265.097219	1468.352576
**ENSG00000134884**	ARGLU1	3906.508979	4837.924821	3775.599439	3607.63981	3737.821459	3340.456338	4947.635127	4500.495931	4295.695872	5201.431481	4905.873183	4571.685297
**ENSG00000134897**	BIVM	361.4012987	304.7787541	390.8319453	420.2912937	448.4461119	402.1446585	604.6655646	420.3120969	588.078889	549.2509155	536.6661047	529.4865857
**ENSG00000134899**	ERCC5	87.18630552	91.08330583	98.52221955	88.7467672	93.61890481	93.70729307	112.3533988	204.3414542	120.9923073	140.4766166	136.5808338	129.4112582
**ENSG00000134900**	TPP2	3130.269614	3110.845215	3063.959605	2698.404063	2494.19255	2755.449674	3179.601187	2814.263606	3059.04194	3330.308258	2786.800852	3444.200283
**ENSG00000134901**	KDELC1	126.5607661	182.1666117	158.7754778	289.6828439	265.8314581	260.2558828	410.600603	370.4727179	445.9832723	456.8653928	413.8813147	469.4329953
**ENSG00000134905**	CARS2	1026.548436	931.8522827	1005.578026	906.7240461	1054.079521	995.8771024	1041.822426	1236.016601	935.5800507	932.0813347	1121.618363	844.9793916
**ENSG00000134909**	ARHGAP32	4026.038592	3667.854662	3893.663255	4695.206325	4270.640164	4473.669635	1734.32792	1363.937674	1863.656703	1927.440425	1873.502751	2003.759939
**ENSG00000134910**	STT3A	4564.624963	4347.476251	5066.973325	3912.39286	4313.404355	4526.024317	2540.208208	2716.24616	2911.318774	2675.383491	3208.959793	3127.015826
**ENSG00000134940**	ACRV1	2.812461468	17.51602035	2.442699658	0	1.155788948	0	1.021394535	0	2.344812157	1.898332657	1.379604382	3.383300867
**ENSG00000134954**	ETS1	4273.535201	6316.276939	5483.0465	2824.826345	3424.602654	3422.023415	556.6600215	1990.252538	644.8233432	432.8198458	1266.476823	596.3067778
**ENSG00000134955**	SLC37A2	1821.068801	2245.553809	1586.126311	1400.691901	1594.988749	1380.5702	195.0863562	194.3735784	196.4952588	176.5449371	158.654504	185.2357225
**ENSG00000134962**	KLB	4.218692202	0	2.442699658	0.837233653	1.155788948	3.793817533	4.08557814	0	1.406887294	1.898332657	0	0
**ENSG00000134970**	TMED7	2968.55308	2462.752462	2365.347502	1918.939532	1796.096026	2030.830525	1923.285909	1726.103829	2015.131568	1717.991055	1507.90759	2036.747122
**ENSG00000134982**	APC	1015.29859	1173.573364	1113.056811	1295.200461	1349.961492	1300.900032	1066.335894	1073.207963	979.6625193	1143.429037	1168.524912	1195.151031
**ENSG00000134986**	NREP	81.56138258	206.6890402	49.66822639	94.60740277	151.4083522	61.08046228	14.29952349	43.19412854	13.13094808	6.960553076	49.66575776	29.60388259
**ENSG00000134987**	WDR36	2241.53179	1390.772016	1775.842652	1832.704466	1579.963492	1905.255165	1407.481669	1111.418154	1473.010997	1443.365597	1122.997967	1600.30131
**ENSG00000134996**	OSTF1	956.2368992	1131.534915	1012.906125	1080.031412	1126.894225	1153.32053	1058.164738	1214.419537	1056.572358	1096.603498	1135.414407	1167.238799
**ENSG00000135002**	RFK	566.7109859	486.9453658	604.975282	810.442176	596.3870973	802.0130265	795.6663427	810.7205664	780.3534859	777.6836119	884.3264091	833.1378385
**ENSG00000135018**	UBQLN1	5409.769634	4718.815883	5410.579743	5035.960422	5266.930237	5561.357122	3768.945834	3643.258611	4055.587107	3743.512	3564.897724	4204.597153
**ENSG00000135040**	NAA35	1499.041963	1720.073199	1546.228884	1548.882258	1697.853965	1591.127073	1642.402412	1669.619199	1554.61046	1698.374951	1539.638491	1589.305582
**ENSG00000135045**	C9orf40	383.9009904	262.7403053	306.1516905	377.5923774	353.6714182	430.2189082	543.3818926	508.3616667	770.9742373	510.0187072	482.8615338	774.7758986
**ENSG00000135046**	ANXA1	28252.58168	25829.12361	22537.97551	23537.14968	18800.06303	17808.93826	19830.37489	17217.84416	18811.49001	16022.5604	12674.42546	17221.84724
**ENSG00000135047**	CTSL	5505.393324	5601.623309	6603.43141	3888.950318	3696.213057	4556.374857	1256.315278	1081.514526	1350.611803	1402.867834	1332.697833	1516.564614
**ENSG00000135048**	TMEM2	6650.065142	6393.347429	6139.318475	7258.81577	6399.603407	7336.104964	6949.568415	6367.811334	6495.598638	7116.216354	6071.638887	7280.017641
**ENSG00000135049**	AGTPBP1	608.8979079	763.6984874	681.5132047	742.6262501	746.6396606	738.2768919	710.8905963	666.1863671	567.4445421	756.1691751	692.5613999	747.7094916
**ENSG00000135052**	GOLM1	1621.384036	1706.060382	1780.728051	1520.416314	1477.098276	1836.587068	394.2582905	395.3924074	322.6461528	432.1870683	431.8161717	410.2252301
**ENSG00000135069**	PSAT1	4324.159508	2700.970338	4516.551668	3194.046386	3052.438612	4578.758381	5114.122436	3146.526133	5859.685581	3945.368039	2717.820633	3866.267066
**ENSG00000135070**	ISCA1	660.9284451	697.13761	576.4771194	719.1837078	766.2880727	693.8892268	1071.442867	1202.790349	1032.186312	1094.072388	1055.397352	1135.097441
**ENSG00000135074**	ADAM19	279.8399161	840.7689769	1257.990324	488.1072196	907.2943244	1638.549793	789.5379755	1189.499847	651.3888173	1036.489631	1637.590402	928.716088
**ENSG00000135077**	HAVCR2	1.406230734	3.50320407	4.071166097	3.348934611	6.93473369	11.00207085	4.08557814	0	0.937924863	0	0	0
**ENSG00000135083**	CCNJL	28.12461468	126.1153465	35.01202844	30.1404115	64.7241811	40.97322936	0	3.322625272	12.19302322	0	4.138813147	4.229126084
**ENSG00000135090**	TAOK3	1479.354732	1397.778424	1467.248261	1441.71635	1303.729934	1366.912457	2627.026744	2508.582081	2403.432461	2290.021962	2254.273561	2353.931578
**ENSG00000135093**	USP30	67.49907524	112.1025303	86.30872126	138.1435527	160.6546638	144.1650663	272.7123408	299.0362745	304.356618	326.513217	321.4478211	308.7262041
**ENSG00000135097**	MSI1	0	10.50961221	0	0.837233653	3.467366845	0	5.106972674	0	0.937924863	0	0	0
**ENSG00000135108**	FBXO21	554.0549093	721.6600385	669.2997064	738.4400818	614.8797205	684.404683	1249.165516	1259.274978	1274.639889	1311.747866	1310.624163	1296.650057
**ENSG00000135111**	TBX3	142.0293041	84.07689769	162.8466439	169.9584315	113.2673169	159.3403364	431.0284937	433.602598	520.0793365	384.0959743	493.8983689	436.4458119
**ENSG00000135114**	OASL	348.7452221	865.2914054	424.2155073	56.93188839	69.3473369	57.28664475	104.1822426	141.2115741	109.737209	27.20943475	26.21248326	17.76232955
**ENSG00000135116**	HRK	42.18692202	21.01922442	23.61276336	118.8871787	63.56839215	70.94438787	541.3391035	279.1005229	557.1273686	733.3891832	357.317535	605.6108552
**ENSG00000135119**	RNFT2	289.6835312	189.1730198	286.6100932	367.5455736	347.8924734	366.1033919	418.7717593	362.1661547	406.1214656	460.6620581	402.8444796	471.970471
**ENSG00000135124**	P2RX4	154.6853808	220.7018564	183.2024744	128.0967489	187.2378096	144.544448	348.2955364	451.877037	385.9560811	418.8987397	391.8076446	367.0881441
**ENSG00000135127**	BICDL1	603.272985	623.5703245	506.4530625	750.161353	759.353339	737.5181284	1689.386561	1202.790349	1567.741408	1587.638879	1067.813792	1368.545201
**ENSG00000135148**	TRAFD1	1047.641897	1047.458017	1187.152034	386.8019476	387.1892977	402.1446585	1507.578333	1611.473257	1447.218063	1037.755186	1107.822319	1014.99026
**ENSG00000135164**	DMTF1	1804.194032	2354.153135	1829.582044	1437.530182	1549.91298	1290.277343	1908.986386	2269.353061	1694.361265	1816.071575	2175.636111	1645.130047
**ENSG00000135185**	TMEM243	189.8411491	266.2435094	162.8466439	128.9339825	175.6799201	178.6888058	345.2313528	333.9238399	355.9424855	311.9593333	347.6603043	323.1052328
**ENSG00000135205**	CCDC146	60.46792157	52.54806106	55.36785892	43.53614995	92.46311586	44.38766514	26.55625791	44.85544118	28.13774589	29.10776741	38.6289227	21.99145564
**ENSG00000135211**	TMEM60	237.6529941	287.2627338	266.2542628	239.4488247	227.6904228	238.2517411	198.1505398	171.1152015	252.7707506	205.019927	206.9406573	223.2978572
**ENSG00000135218**	CD36	23.90592248	10.50961221	15.47043117	44.3733836	35.8294574	22.38352344	7.149761744	31.56494009	19.69642212	37.33387559	24.83287888	27.06640694
**ENSG00000135241**	PNPLA8	660.9284451	893.317038	618.8172468	495.6423225	716.5891479	548.965397	980.5387535	1156.273595	881.6493711	715.6714117	881.5672003	751.0927925
**ENSG00000135245**	HILPDA	109.6859973	241.7210809	70.02405687	79.53719702	288.9472371	102.0536916	130.7385005	524.974793	115.3647581	106.9394064	605.6463238	160.7067912
**ENSG00000135248**	FAM71F1	12.65607661	10.50961221	0	0	1.155788948	0.758763507	0	0	2.344812157	0	0	0
**ENSG00000135249**	RINT1	572.3359088	528.9838146	538.2081581	601.1337628	538.5976499	564.5200489	455.5419626	415.328159	451.1418591	452.43595	397.3260621	504.1118292
**ENSG00000135250**	SRPK2	665.1471373	592.0414879	639.9873105	918.4453172	631.0607658	784.9408476	718.040358	787.4621895	825.8428418	840.3285896	796.0317286	939.7118158

ENSG00000135253	KCP	8.437384405	3.50320407	2.442699658	5.023401917	9.246311586	6.449489806	4.08557814	4.983937908	0.937924863	1.898332657	9.657230676	0.845825217
ENSG00000135269	TES	2586.05832	2925.175399	2837.60277	3088.554945	3018.920733	3530.147214	4833.238939	5027.132037	4676.493366	4383.882883	4336.096574	4339.929187
ENSG00000135272	MDFIC	691.8655212	620.0671205	642.4300101	1007.192084	977.7974502	961.7327446	862.0569874	887.1409477	866.6425733	831.4697038	933.9921668	994.6904549
ENSG00000135297	MTO1	729.833751	585.0350798	733.6241307	790.3485683	743.1722937	823.2584047	886.5704563	875.5117592	828.1876539	828.9385936	758.7824103	799.3048299
ENSG00000135298	ADGRB3	0	0	0	0	0	0.379381753	1.021394535	0	0.468962431	1.265555105	5.518417529	5.074951301
ENSG00000135299	ANKRD6	1.406230734	7.006408141	10.58503185	4.186168264	5.778944741	4.931962793	218.5784305	167.7925762	202.5917704	267.6649047	233.1531406	257.9766911
ENSG00000135312	HTR1B	21.09346101	0	0.814233219	20.09360767	5.778944741	6.070108053	1.021394535	0	0	0	6.898021912	1.691650434
ENSG00000135314	KHDC1	23.90592248	21.01922442	25.2412298	18.41914036	15.02525633	17.45156065	13.27812895	11.62918845	10.78613592	17.71777147	17.93485697	12.68737825
ENSG00000135315	CEP162	725.6150588	830.2593647	650.5723423	631.2741743	725.8354595	498.1282421	1178.689293	1270.904167	936.5179756	977.0085409	1318.901789	960.011621
ENSG00000135316	SYNCRIP	7410.835969	5545.572043	6314.378617	8070.932414	6524.428613	7107.717148	7358.126229	6636.943981	7171.373502	7511.702324	6431.71563	8025.189657
ENSG00000135317	SNX14	1709.976573	1674.531546	1396.409971	1646.838595	1610.014005	1587.712638	1595.418263	1676.26445	1456.12835	1581.943881	1699.672599	1717.02519
ENSG00000135318	NT5E	7631.614194	7535.391955	6079.87945	5362.481547	5190.648167	5467.270447	1758.841389	1706.168077	1819.105272	1581.311103	1136.794011	1600.30131
ENSG00000135334	AKIRIN2	797.3328263	609.5575083	759.6795937	586.9007907	576.7386852	678.3345749	527.03958	425.2960348	563.2238802	587.2175686	378.0116008	609.8399813
ENSG00000135336	ORC3	1227.639431	1261.153465	1218.90713	1031.47186	1147.698426	1050.508075	1111.277254	1098.127652	1033.593199	970.6807654	961.5842545	1030.215114
ENSG00000135338	LCA5	9.843615139	17.51602035	13.02773151	24.27977593	39.29682424	14.79588838	97.03248081	174.4378268	58.62030393	98.08052062	97.95191114	62.59106604
ENSG00000135341	MAP3K7	1480.760963	1527.396975	1529.129986	1412.413172	1435.489874	1503.110507	1770.076729	1795.87896	1750.167794	1675.594959	1881.780377	1834.594895
ENSG00000135346	CGA	5242.428177	6365.321796	5532.714726	7008.482908	7259.510384	7566.76907	980.5387535	408.6829085	1140.047671	1571.81944	700.8390262	1829.519944
ENSG00000135362	PRR5L	88.59253625	133.1217547	54.5536257	85.39783259	67.035759	40.5938476	2.04278907	8.306563181	0.468962431	2.53111021	6.898021912	3.383300867
ENSG00000135363	LMO2	82.96761332	161.1473872	95.26528668	13.39573845	11.55788948	25.03919572	0	4.983937908	3.28273702	1.898332657	6.898021912	4.229126084
ENSG00000135365	PHF21A	192.6536106	332.8043867	267.8827292	253.6817968	299.3493376	293.2620953	359.5308763	342.230403	338.5908755	329.6771048	328.345843	297.7304763
ENSG00000135372	NAT10	1070.141589	952.8715072	1114.685277	1260.873881	1128.050014	1308.487667	1526.98483	1471.922996	1625.423787	1487.027248	1590.683853	1700.108686
ENSG00000135373	EHF	2919.335004	4435.056353	4845.501889	2047.873515	2481.478872	3281.652166	261.4770009	222.6158932	454.4245961	234.7604719	263.504437	479.5828979
ENSG00000135374	ELF5	21.09346101	0	13.02773151	46.04785091	43.91998003	38.31755708	4.08557814	0	14.06887294	10.12444084	1.379604382	38.06213475
ENSG00000135378	PRRG4	1181.233817	1660.518729	1109.799878	658.9028848	705.0312584	767.8686687	1491.236021	1692.877576	1765.174592	1058.636845	1077.471023	1416.757238
ENSG00000135387	CAPRIN1	8734.09909	7468.831078	8337.748167	7637.245381	7498.758696	7698.035156	10468.27259	9841.616056	10070.03029	9656.818227	9065.380396	9759.131351
ENSG00000135390	ATP5MC2	580.7732932	557.0094472	581.3625187	721.6954088	747.7954495	638.1201091	881.4634836	860.5599455	936.986938	978.9068735	1049.878935	1125.793364
ENSG00000135392	DNAJC14	354.370145	287.2627338	367.219182	384.2902467	366.3850966	369.8972095	455.5419626	483.4419771	470.8382812	446.1081744	482.8615338	540.4823135
ENSG00000135404	CD63	1521.541654	1912.749422	2054.310413	1563.952464	1760.266568	1903.737638	1150.090246	1352.308486	1238.529781	1236.447337	1587.924644	1307.645785
ENSG00000135406	PRPH	0	10.50961221	1.628466439	6.697869223	4.623155793	1.517527013	1.021394535	8.306563181	2.344812157	1.898332657	2.759208765	0.845825217
ENSG00000135407	AVIL	22.49969175	49.04485699	17.09889761	8.372336529	6.93473369	6.070108053	17.36370709	21.59706427	17.8205724	18.35054902	30.35129641	14.37902869
ENSG00000135414	GDF11	56.24922937	77.07048955	39.89742775	136.4690854	134.071518	126.7135056	109.2892152	116.2918845	125.6819316	165.1549412	154.5156908	188.6190233
ENSG00000135423	GLS2	12.65607661	0	9.770798633	22.60530863	23.11577897	19.72785117	34.72741419	23.25837691	18.75849726	27.8422123	15.17564821	19.45397999
ENSG00000135424	ITGA7	7.031153671	7.006408141	4.885399317	5.023401917	5.778944741	7.967016819	6.128367209	1.661312636	6.56547404	16.45221636	8.277626294	4.229126084
ENSG00000135426	TESPA1	15.46853808	7.006408141	4.071166097	0.837233653	4.623155793	2.655672273	0	0	0.468962431	0	0	0
ENSG00000135436	FAM186B	2.812461468	0	4.885399317	3.348934611	4.623155793	1.896908767	9.192550814	9.967875817	9.379248629	5.694997971	4.138813147	2.53747565
ENSG00000135437	RDH5	2.812461468	0	13.02773151	2.511700959	13.86946738	6.449489806	18.38510163	33.22625272	18.28953483	20.24888168	22.07367012	12.68737825
ENSG00000135439	AGAP2	0	14.01281628	10.58503185	5.86063557	10.40210053	7.967016819	3.064183605	4.983937908	3.28273702	0.632777552	5.518417529	0
ENSG00000135441	BLOC1S1	104.0610743	56.05126513	96.07951989	80.37443068	101.7094274	84.22274923	86.81853546	103.0013834	98.01314817	101.2444084	77.25784541	98.11572515
ENSG00000135446	CDK4	811.3951336	564.0158553	756.4226609	753.5102876	703.8754695	881.6831947	860.0141984	584.7820479	886.3389954	827.040261	841.5586732	850.0543429
ENSG00000135451	TROAP	404.9944514	150.637775	333.83562	529.1316686	365.2293077	523.9262013	550.5316543	353.8595915	597.9271001	560.6409114	499.4167864	554.8613422
ENSG00000135452	TSPAN31	203.9034565	273.2499175	261.3688634	180.0052354	233.4693675	179.0681876	211.4286687	249.1968954	190.3987472	213.8788127	205.561053	171.702519
ENSG00000135454	B4GALNT1	78.74892111	94.5865099	96.07951989	105.4914403	152.5641412	99.39801937	1.021394535	0	0.468962431	0	2.759208765	2.53747565
ENSG00000135457	TFCP2	933.7372075	746.182467	948.5817007	1040.681431	959.3048271	956.0420183	1027.522902	744.268061	1026.0898	1102.298496	851.2159039	1116.489286
ENSG00000135469	COQ10A	130.7794583	80.57369362	91.19412058	99.63080469	104.0210053	104.7093639	55.15530488	131.2436983	77.84776362	63.27775524	117.2663725	66.82019213
ENSG00000135472	FAIM2	0	7.006408141	0	0	0	0.379381753	12.25673442	134.5663235	7.503398903	16.45221636	161.4137127	7.612426951
ENSG00000135473	PAN2	196.8723028	227.7082646	204.3725381	202.610544	224.223056	156.3052824	452.477779	490.0872277	354.0666357	432.1870683	562.878588	352.7091154
ENSG00000135476	ESPL1	753.7396735	451.9133251	686.398604	797.0464375	735.0817711	766.7305234	966.23923	815.7045043	1108.627188	1099.134608	1042.980913	1115.643461
ENSG00000135480	KRT7	6017.261311	5640.158553	7499.087951	7889.252711	8735.452871	11233.8731	20643.40494	12738.94529	21084.08196	21879.54943	13812.59908	19361.78504
ENSG00000135482	ZC3H10	246.0903785	238.2178768	175.0601422	166.6094969	175.6799201	162.3753904	316.6323058	292.391024	243.3915019	305.6315578	286.9577115	260.5141668
ENSG00000135486	HNRNPA1	12034.52262	10064.70529	10866.75655	13341.31826	11465.42637	12044.99129	13745.92765	13179.19314	14227.85121	14638.6759	14494.12364	15657.07059
ENSG00000135503	ACVR1B	248.9028399	273.2499175	257.2976973	234.4254228	247.3388349	229.146579	185.8938053	167.7925762	170.2333626	181.6071575	172.4505478	148.8652382
ENSG00000135506	OS9	1662.164728	1954.787871	1752.229888	1416.599341	1561.470869	1484.900182	2302.223282	2518.594956	2340.591495	2233.071982	2837.846214	2216.907893
ENSG00000135519	KCNH3	4.218692202	14.01281628	3.256932878	20.09360767	5.778944741	11.00207085	9.192550814	0	6.096511609	1.898332657	2.759208765	2.53747565
ENSG00000135521	LTV1	547.0237556	346.817203	437.2432388	476.3859485	346.7366845	396.833314	362.5950599	322.2946514	317.4875661	353.7226518	227.6347231	426.2959093
ENSG00000135525	MAP7	908.4250543	1089.496466	990.1075948	1149.521805	1177.748938	1278.137127	1116.384227	1008.41677	1089.868691	1175.700692	1082.98944	1111.414335
ENSG00000135535	CD164	5138.367103	5475.507962	5096.285721	4472.502174	4628.934738	4684.60589	5775.986095	5568.719956	5900.485313	5695.630749	5543.250408	6039.192048
ENSG00000135537	AFG1L	161.7165344	136.6249587	183.2024744	147.3531229	190.7051765	167.3073532	192.0221726	122.9371351	166.0127007	172.1154942	118.6459769	159.860966
ENSG00000135540	NHSL1	824.0512102	602.5511001	718.9679328	838.0708865	663.4228563	814.5326243	854.9072257	833.9789433	1019.993288	963.7202123	853.9751126	943.0951167
ENSG00000135541	AHI1	1414.668119	1513.384158	1262.875723	1592.418408	1558.003502	1415.473322	2384.956239	1817.476024	1971.518062	2329.25417	2088.721035	2285.419736
ENSG00000135547	HEY2	0	0	0.814233219	0.837233653	0	0.758763507	4.08557814	0	9.848211061	1.898332657	11.03683506	2.53747565
ENSG00000135549	PKIB	310.7769922	385.3524477	256.4834641	312.2881525	307.4398602	292.8827136	353.4025091	390.4084695	425.3489253	336.6376579	368.3543701	400.9211528
ENSG00000135577	NMBR	1.406230734	0	0.814233219	0	0	1.13814526	2.04278907	0	0.937924863	0.632777552	0	0
ENSG00000135587	SMPD2	118.1233817	101.592918	83.05178838	146.5158893	159.4988749	144.9238298	179.7654381	144.5341993	142.0956167	161.9910534	102.0907243	125.1821321
ENSG00000135596	MICAL1	192.6536106	178.6634076	195.4159727	197.5871421	292.4146039	224.593998	122.5673442	147.8568246	106.9234344	130.9849533	177.9689653	84.58252168
ENSG00000135597	REPS1	1182.640047	956.3747112	1007.206492	1230.73347	1189.306828	1159.77002	1217.502286	1116.402091	1081.427367	1069.394064	1049.878935	1085.193753
ENSG00000135604	STX11	23.90592248	45.54165292	38.26896131	1.674467306	9.246311586	7.587635066	144.0166294	107.9853213	135.5301427	85.42496957	82.77626294	67.66601734
ENSG00000135605	TEC	198.2785335	364.3332233	236.9418669	102.1425056	161.8104528	155.1671371	41.87717593	21.59706427	37.04803208	25.3111021	52.42496653	22.83728085

**ENSG00000135617**	PRADC1	33.74953762	35.0320407	35.82626166	82.88613163	84.37259322	77.39387767	68.43343384	124.5984477	81.59946307	130.9849533	148.9972733	101.499026
**ENSG00000135622**	SEMA4F	146.2479964	178.6634076	190.5305734	181.6797027	273.9219807	227.2496702	188.957989	210.9867048	146.3162786	178.4432698	285.5781071	181.0065964
**ENSG00000135624**	CCT7	2619.807858	2753.518399	2811.547307	3034.134758	3223.495377	3351.079027	2165.356414	2020.156166	2362.63273	2198.269217	2135.627584	2508.717593
**ENSG00000135631**	RAB11FIP5	136.4043812	294.2691419	240.1987997	179.1680017	310.9072271	321.336345	85.79714093	192.7122658	98.01314817	105.0410737	160.0341083	104.0365017
**ENSG00000135632**	SMYD5	382.4947597	210.1922442	438.0574721	424.477462	441.5113782	497.7488603	389.1513178	325.6172767	443.6384602	367.0109804	360.0767438	429.6792101
**ENSG00000135636**	DYSF	80.15515185	73.56728548	104.2218521	92.93293547	82.06101533	147.9588838	260.4556064	136.2276362	327.3357772	210.7149249	201.4222398	343.405038
**ENSG00000135637**	CCDC142	293.9022234	252.2306931	280.9104607	229.4020209	201.107277	248.4950484	294.161626	260.8260839	238.2329152	256.9076863	274.5412721	215.6854303
**ENSG00000135643**	KCNMB4	29.53084542	56.05126513	27.68392946	118.8871787	125.9809954	105.0887457	56.17669942	51.50069172	73.62710174	118.3294023	96.57230676	136.1778599
**ENSG00000135655**	USP15	1255.764046	1422.300853	1502.26029	967.0048691	938.500626	960.5945994	2071.388117	1830.766525	1774.553841	1607.254983	1363.04913	1532.635293
**ENSG00000135677**	GNS	1238.889277	1383.765608	1485.161392	1352.132349	1613.481372	1502.351743	1822.16785	2066.672919	1993.559296	1950.220416	1996.287541	2215.216243
**ENSG00000135678**	CPM	82.96761332	217.1986524	157.9612446	40.18721534	106.3325832	97.12172885	16.34231256	13.29050109	11.72406079	21.51443678	73.11903226	19.45397999
**ENSG00000135679**	MDM2	2874.335621	2918.168991	2581.933539	3287.816555	2612.083023	2619.251625	3085.63289	2367.370506	2885.056878	3106.937782	2541.231272	3204.831746
**ENSG00000135686**	KLHL36	1009.673667	1240.134241	1198.551299	1296.037695	1575.340336	1519.04454	1040.801031	1113.079466	1118.475399	1030.794633	1259.578801	1164.701324
**ENSG00000135697**	BCO1	7.031153671	7.006408141	2.442699658	1.674467306	0	0	2.04278907	1.661312636	3.751699452	5.062220419	1.379604382	5.074951301
**ENSG00000135698**	MPHOSPH6	376.8698368	346.817203	387.5750125	366.70834	412.6166545	380.1405168	271.6909463	214.3093301	291.6946324	286.0154537	269.0228546	364.5506684
**ENSG00000135702**	CHST5	0	0	0	0.837233653	2.311577897	0.379381753	1.021394535	0	2.344812157	0.632777552	5.518417529	0.845825217
**ENSG00000135709**	KIAA0513	127.9669968	143.6313669	135.9769476	183.35417	167.5893975	242.8043221	173.6370709	448.5544118	317.0186037	208.8165923	402.8444796	319.7219319
**ENSG00000135720**	DYNC1LI2	2484.809707	2644.919073	2838.417003	2406.209518	2371.678922	2564.620652	2612.72722	2889.022674	2388.894626	2541.867428	2808.874522	2490.955263
**ENSG00000135722**	FBXL8	15.46853808	59.5544692	39.89742775	37.67551438	45.07576898	34.9031213	51.06972674	48.17806645	32.8273702	51.8877593	52.42496653	30.4497078
**ENSG00000135723**	FHOD1	264.371378	213.6954483	296.3808919	493.9678552	448.4461119	445.0147966	200.1933288	191.0509532	273.4050975	256.2749087	201.4222398	237.6768859
**ENSG00000135736**	CCDC102A	56.24922937	49.04485699	72.46675653	63.62975762	80.90522638	79.29078644	15.32091802	29.90362745	15.00679781	11.38999594	11.03683506	17.76232955
**ENSG00000135740**	SLC9A5	18.28099954	56.05126513	40.71166097	58.6063557	57.78944741	34.1443578	30.64183605	44.85544118	22.51019671	25.3111021	41.38813147	12.68737825
**ENSG00000135749**	PCNX2	701.7091363	434.3973047	678.2562718	697.4156328	654.1765447	685.5428282	1085.742391	940.302952	889.15277	937.7763326	924.3349361	742.6345403
**ENSG00000135750**	KCNK1	3601.35691	4158.303232	4071.98033	2860.827392	2639.821958	3253.577916	1682.236799	2525.195207	1321.067169	1311.747866	2022.500024	1230.67569
**ENSG00000135763**	URB2	724.2088281	259.2371012	612.303381	783.6506991	548.9997504	811.8769521	558.7028106	383.7632189	639.6647565	612.5286707	467.6858856	764.625996
**ENSG00000135766**	EGLN1	73.12399818	164.6505913	50.48245961	61.11805666	169.9009754	75.11758715	83.75435186	229.2611438	91.91663656	113.2671819	270.4024589	84.58252168
**ENSG00000135775**	COG2	608.8979079	381.8492437	534.136992	508.2008273	402.214554	520.1323838	660.8422641	480.1193518	590.4237012	626.4497769	488.3799513	581.9277491
**ENSG00000135776**	ABCB10	320.6206074	346.817203	317.5509556	417.7795928	439.1998003	427.9426177	586.280463	553.2171078	586.6720017	675.1736484	619.4423677	767.1634716
**ENSG00000135778**	NTPCR	416.2442973	378.3460396	398.1600443	586.9007907	476.1850467	454.1199587	612.8367209	608.0404248	656.0784416	764.3952833	680.1449605	632.6772621
**ENSG00000135801**	TAF5L	705.9278285	644.589549	816.6759191	832.210251	762.8207059	887.373921	712.9333853	724.3323094	846.9461512	840.3285896	773.9580585	765.4718212
**ENSG00000135821**	GLUL	59.06169083	94.5865099	75.72368941	41.86168264	50.85471372	47.80210092	3757.710494	1662.973949	2872.394893	3776.416433	1688.635764	2871.576611
**ENSG00000135823**	STX6	966.0805144	844.272181	942.0678349	848.954924	782.469118	853.9883267	659.8208695	611.3630501	753.6226273	729.5925179	678.7653561	809.4547325
**ENSG00000135828**	RNASEL	163.1227652	273.2499175	244.2699658	154.8882258	278.5451365	234.0785418	222.6640086	305.681525	206.3434698	213.8788127	213.8386793	183.544072
**ENSG00000135829**	DHX9	5956.79339	5233.786881	5610.066882	6306.881107	5447.233313	5947.188365	5946.558982	5271.344994	6152.318138	6020.878411	5692.247681	6510.316694
**ENSG00000135835**	KIAA1614	1.406230734	0	6.513865756	0.837233653	8.090522638	1.517527013	4.08557814	3.322625272	6.56547404	5.062220419	8.277626294	4.229126084
**ENSG00000135837**	CEP350	2574.808474	3664.351458	3079.430036	3003.994346	3500.884724	3080.579837	5314.315765	5585.333083	4957.401863	5850.028472	5881.253482	5826.889918
**ENSG00000135838**	NPL	174.372611	168.1537954	158.7754778	135.6318518	188.3935986	199.934184	66.39064477	114.6305719	94.73041115	65.80886545	70.3598235	80.35339559
**ENSG00000135842**	FAM129A	309.3707615	290.7659378	511.3384618	99.63080469	159.4988749	206.7630556	2676.053681	2166.351678	2981.663139	2677.281824	1925.927718	2830.131175
**ENSG00000135845**	PIGC	402.18199	371.3396315	431.5436063	441.2221351	389.5008756	469.2952288	464.7345134	578.1367974	457.7073331	442.9442867	533.906896	450.8248405
**ENSG00000135862**	LAMC1	5721.952857	5741.751471	6316.007083	8370.662061	8295.097282	8669.252445	9124.11738	9177.091002	10241.20158	10410.45629	10976.13247	12715.29048
**ENSG00000135870**	RC3H1	2079.815256	2108.92885	2316.493509	1947.405477	1986.801202	2327.886438	1949.842167	2154.722489	2009.035056	1914.152096	2146.664419	2033.363821
**ENSG00000135899**	SP110	2261.219021	2960.20744	2489.925185	344.940265	330.5556392	271.2579536	1865.066421	1576.585692	1563.520746	684.0325341	575.2950274	635.2147378
**ENSG00000135900**	MRPL44	383.9009904	364.3332233	322.4363549	257.8679651	268.143036	311.0930377	217.5570359	282.4231481	260.2741495	270.1960149	263.504437	282.5056224
**ENSG00000135903**	PAX3	156.0916115	87.58010176	135.1627144	188.3775719	106.3325832	104.7093639	1.021394535	0	0	0.632777552	0	0.845825217
**ENSG00000135905**	DOCK10	1442.792733	1481.855322	1788.870383	885.7932047	1113.024757	1129.040098	3.064183605	3.322625272	3.28273702	1.265555105	2.759208765	0
**ENSG00000135912**	TTLL4	575.1483703	437.9005088	599.2756495	646.34438	614.8797205	699.9593348	448.3922008	495.0711656	481.1554547	484.0748276	488.3799513	594.6151274
**ENSG00000135913**	USP37	952.018207	1404.784832	1017.791524	981.2378412	1217.045763	942.3842752	1005.052222	1545.020752	1029.372537	958.0252143	1367.187943	1006.532008
**ENSG00000135916**	ITM2C	132.185689	161.1473872	176.6886086	168.2839642	230.0020007	242.4249404	36.77020326	33.22625272	22.51019671	39.23220825	84.15586732	26.22058172
**ENSG00000135917**	SLC19A3	64.68661377	21.01922442	47.22552673	195.0754411	98.2420606	124.4372151	203.2575124	167.7925762	230.2605538	233.4949168	197.2834267	252.9017398
**ENSG00000135919**	SERPINE2	1157.327894	2413.707605	2149.575699	1662.746035	2593.5904	3646.238031	135.8454731	357.1822168	177.7367615	127.188288	336.6234693	186.0815477
**ENSG00000135924**	DNAJB2	184.2162262	462.4229373	390.8319453	330.7072929	596.3870973	571.3489205	349.3169309	606.3791122	356.4114479	325.8804395	640.1364334	338.3300867
**ENSG00000135925**	WNT10A	37.96822982	70.06408141	81.42332194	163.2605623	255.4293576	313.3693282	505.5902948	327.2785893	600.7408747	518.2448154	382.1504139	580.2360987
**ENSG00000135926**	TMBIM1	3391.828531	4207.348089	4067.094931	2959.620963	3512.442614	3555.18641	2142.885734	4995.567097	2704.975305	1788.229363	4429.909672	2232.132747
**ENSG00000135929**	CYP27A1	1.406230734	14.01281628	3.256932878	5.86063557	5.778944741	2.655672273	5.106972674	1.661312636	0	0	1.379604382	3.383300867
**ENSG00000135930**	EIF4E2	947.7995148	991.4067519	985.2221955	1023.099524	1166.191049	1095.275122	769.1100848	887.1409477	783.1672605	802.994714	796.0317286	888.9623028
**ENSG00000135931**	ARMC9	329.0579918	367.8364274	330.5786871	444.5710697	421.8629661	383.5549526	485.1624041	541.5879194	438.4798734	403.7120784	430.4365673	364.5506684
**ENSG00000135932**	CAB39	4494.313426	4280.915374	4377.317788	3819.459924	3134.499628	4040.795054	1387.053778	1528.407625	1574.775845	1208.605125	1389.261613	1622.292766
**ENSG00000135940**	COX5B	721.3963666	833.7625688	727.110265	862.3506625	865.6859222	890.0295933	771.1528738	865.5438834	977.7866696	958.6579919	924.3349361	1047.131618
**ENSG00000135945**	REV1	770.6144423	791.7241199	851.6879475	738.4400818	753.5743943	696.5448991	964.1964409	907.0766993	944.9592994	983.3363164	1012.629617	1033.598415
**ENSG00000135951**	TSGA10	92.81122845	105.0961221	75.72368941	69.49039319	83.21680428	54.25159072	71.49761744	89.71088235	68.46851499	73.40219608	102.0907243	51.59533822
**ENSG00000135953**	MFSD9	104.0610743	70.06408141	143.3050466	106.3286739	124.8252064	124.0578333	177.7226491	184.4057026	133.1853305	134.1488411	131.0624163	111.6489286
**ENSG00000135956**	TMEM127	845.1446712	1264.656669	1189.594734	932.6782893	1050.612154	1152.941148	925.3834486	955.2547658	971.2211955	825.1419283	902.261266	924.4869619
**ENSG00000135960**	EDAR	0	10.50961221	1.628466439	0	0	0.379381753	2.04278907	4.983937908	0	0	0	0.845825217
**ENSG00000135966**	TGFBRAP1	511.8679872	518.4742024	526.808893	547.550809	570.9597404	627.1180382	487.2051931	392.0697821	560.879068	547.3525828	460.7878637	546.40309
**ENSG00000135968**	GCC2	1354.200197	1961.794279	1448.520897	1154.545207	1323.378346	1094.136977	1643.423807	1428.728867	1285.426025	1392.110615	1255.439988	1324.562289
**ENSG00000135972**	MRPS9	452.8062964	406.3716722	491.7968645	522.4337994	483.1197804	534.5488904	501.5047166	506.700354	501.7898017	551.7820257	554.6009617	575.1611474
**ENSG00000135974**	C2orf49	520.3053716	630.5767327	547.9789567	587.7380243	616.0355094	523.9262013	550.5316543	549.8944826	625.1269211	539.7592522	687.0429824	575.1611474

ENSG00000135976	ANKRD36	606.0854464	977.3939356	534.136992	688.2060627	1006.692174	638.8788726	553.5958379	782.4782516	355.0045606	673.9080933	787.7541023	504.9576544
ENSG00000135999	EPC2	818.4262873	599.047896	648.1296427	806.2560077	622.9702431	737.1387467	741.5324323	719.3483714	663.5818405	713.7730791	624.9607852	733.3304629
ENSG00000136002	ARHGEF4	992.7988983	1005.419568	986.850662	1285.990891	1186.99525	1273.205164	42.89857046	33.22625272	159.4472267	81.62830426	22.07367012	118.4155303
ENSG00000136003	ISCU	502.0243721	528.9838146	552.864356	463.8274437	450.7576898	456.3962492	579.1307013	506.700354	599.8029498	622.020334	506.3148083	581.0819239
ENSG00000136010	ALDH1L2	64.68661377	63.05767327	65.13865756	16.74467306	55.47786952	61.45984404	109.2892152	61.46856754	38.45491938	61.37942258	78.63744979	41.44543562
ENSG00000136021	SCYL2	1175.608894	1292.682302	1345.927512	1457.62379	1416.997251	1532.322902	2535.101236	2641.487091	2426.41162	2345.706387	2501.222745	2759.081857
ENSG00000136026	CKAP4	265.7776088	297.772346	386.7607792	306.4275169	398.7471872	428.3219995	84.77574639	94.69482026	65.18577797	79.09719405	111.747955	109.111453
ENSG00000136040	PLXNC1	2.812461468	3.50320407	1.628466439	0	1.155788948	0	0	0	2.344812157	5.062220419	1.379604382	1.691650434
ENSG00000136044	APPL2	700.3029056	599.047896	676.6278054	814.6283442	759.353339	756.1078343	1237.930176	1355.631111	1201.481749	1206.074015	1321.660998	1256.896272
ENSG00000136045	PWP1	1295.138506	1005.419568	1133.412641	1167.940946	1080.662667	1102.862757	1342.112419	1300.807794	1292.460461	1335.160636	1292.689306	1517.410439
ENSG00000136048	DRAM1	509.0555258	507.9645902	565.0778543	169.1211979	221.9114781	204.486765	2676.053681	2412.225948	2743.430224	1326.934527	1117.47955	1670.504803
ENSG00000136051	WASHC4	2169.814023	2518.803727	2074.666243	1686.188577	1896.649664	1780.059187	3134.659828	3236.237015	2637.444714	2483.651893	2614.350304	2489.263613
ENSG00000136052	SLC41A2	59.06169083	168.1537954	113.1784175	73.67656145	168.7451864	147.2001203	887.5918508	1179.531972	568.3824669	580.2570155	860.8731346	409.3794049
ENSG00000136059	VILL	4.218692202	14.01281628	2.442699658	6.697869223	4.623155793	8.346398573	1.021394535	9.967875817	10.78613592	1.898332657	8.277626294	10.99572782
ENSG00000136068	FLNB	20574.56187	21425.59609	25798.16532	19439.72819	21019.17781	26449.73708	14073.7953	20362.70898	17854.33769	13926.16837	20212.58381	17283.59248
ENSG00000136098	NEK3	85.78007478	45.54165292	83.05178838	72.00209415	79.74943743	61.08046228	85.79714093	29.90362745	51.11690503	68.33997566	33.11050518	53.28698866
ENSG00000136100	VPS36	1096.859973	784.7177118	905.42734	983.7495421	790.5596406	888.1326845	953.9824956	752.5746242	846.9461512	794.7686058	669.1081254	783.2341507
ENSG00000136104	RNASEH2B	577.9608317	595.544692	495.8680306	473.8742475	480.8082025	485.6086442	941.7257612	792.4461274	776.1328241	843.4924773	698.0798174	788.309102
ENSG00000136108	CKAP2	3055.739385	2420.714013	3046.860707	3451.077117	3350.632161	3392.052256	2446.239911	2134.786737	2495.349098	2403.289144	2138.386793	2957.004958
ENSG00000136111	TBC1D4	50.62430643	35.0320407	33.383562	42.6989163	104.0210053	100.9155464	547.4674707	456.8609749	414.093827	574.5620176	504.9352039	501.5743535
ENSG00000136114	THSD1	12.65607661	21.01922442	20.35583049	5.023401917	3.467366845	6.449489806	10.21394535	3.322625272	11.72406079	6.960553076	0	4.229126084
ENSG00000136122	BORA	755.1459042	378.3460396	574.8486529	857.3272605	553.6229062	774.6975402	1035.694058	730.9775599	1085.179066	1101.032941	750.504784	1261.125398
ENSG00000136141	LRCH1	459.8374501	550.0030391	453.5279032	255.3562641	288.9472371	355.1013211	584.2376739	461.8449128	640.6026814	495.4648235	394.5668533	489.7328005
ENSG00000136143	SUCLA2	888.737824	1100.006078	696.9836358	782.8134654	796.3385854	788.7346651	754.8105613	1455.309869	721.2642196	576.4603502	866.3915521	668.2019213
ENSG00000136144	RCBTB1	350.1514528	234.7146727	318.3651888	305.5902833	309.7514381	324.3713991	452.477779	284.0844608	331.0874766	329.0443272	257.9860195	346.7883389
ENSG00000136146	MED4	518.8991409	521.9774065	508.0815289	560.9465474	505.0797704	553.5179781	615.9009045	697.7513072	528.5206602	499.8942664	608.4055326	576.8527978
ENSG00000136147	PHF11	627.1789074	693.6344059	614.7460807	298.8924141	262.3640913	297.0559128	491.2907713	589.7659858	484.4381917	332.8409926	288.3373159	271.5098946
ENSG00000136149	RPL13AP25	8.437384405	7.006408141	8.956565414	12.55850479	15.02525633	17.45156065	19.40649616	21.59706427	14.53783538	5.694997971	9.657230676	7.612426951
ENSG00000136152	COG3	707.3340593	795.227324	775.1500249	532.4806032	463.4713683	613.4602951	666.9706313	740.9454357	534.1482094	567.6014645	623.5811808	537.9448379
ENSG00000136153	LMO7	27043.22325	27591.23526	26949.4911	18797.56997	16107.07478	18582.87704	6993.48838	6658.541046	7417.578778	5346.33754	4905.873183	6728.539599
ENSG00000136155	SCEL	455.6187579	273.2499175	314.2940227	996.3080469	401.058765	655.5716697	93.96829721	104.6626961	126.1508941	71.50386342	180.7281741	159.0151408
ENSG00000136156	ITM2B	2908.085158	2609.887032	2488.296719	1732.236428	1985.645413	1929.914979	3832.272295	4028.683143	3833.298915	2764.605126	2908.206038	3243.739706
ENSG00000136158	SPRY2	937.9558997	1036.948405	1163.539271	755.1847549	598.6986752	858.161526	156.2733638	162.8086383	162.2610013	125.2899554	121.4051856	158.1693155
ENSG00000136159	NUDT15	479.5246803	364.3332233	330.5786871	329.0328256	359.4503629	340.3054327	285.9904698	940.302952	426.7558126	277.1565679	402.8444796	323.1052328
ENSG00000136160	EDNRB	35.15576835	63.05767327	55.36785892	46.04785091	47.38734688	66.01242507	1308.406399	1378.889488	1616.513501	1377.556732	1267.856427	1717.02519
ENSG00000136161	RCBTB2	33.74953762	21.01922442	26.86969624	20.09360767	40.45261319	35.28250306	18.38510163	39.87150327	7.503398903	20.24888168	56.56377967	9.304077385
ENSG00000136167	LCP1	1.406230734	0	30.12662912	21.76807497	9.246311586	24.28043221	185.8938053	157.8247004	178.6746864	203.7543719	177.9689653	219.0687311
ENSG00000136169	SETDB2	167.3414574	136.6249587	191.3448066	157.3999267	146.7851964	188.1733496	217.5570359	210.9867048	221.8192301	237.9243597	144.8584601	220.7603816
ENSG00000136193	SCRN1	1468.104886	1604.467464	1850.752108	1496.973771	1730.216056	1772.471551	372.8090052	412.0055338	460.9900701	235.3932495	361.4563482	460.1289179
ENSG00000136197	C7orf25	12.65607661	7.006408141	17.91313083	21.76807497	28.89472371	16.69279715	10.21394535	4.983937908	16.4136851	14.5538837	11.03683506	17.76232955
ENSG00000136205	TNS3	545.6175249	711.1504263	621.2599464	868.211298	954.6816713	1137.007115	2261.3675	1933.767908	2249.143821	2536.17243	1949.380992	2478.267885
ENSG00000136213	CHST12	78.74892111	66.56087734	78.16638907	103.816973	94.77469376	85.36089449	63.32646116	46.51675381	46.89624315	88.58885733	64.84140597	77.81591994
ENSG00000136231	IGF2BP3	3258.236611	2539.822951	3059.888439	3460.286687	3091.735437	3248.266572	4116.219976	3377.448589	4158.758842	3716.935343	3122.044717	3955.078714
ENSG00000136235	GPNMB	59.06169083	112.1025303	115.6211172	92.09570182	194.1725433	154.4083736	48.00554314	129.5823856	116.302683	37.33387559	91.05388923	109.111453
ENSG00000136237	RAPGEF5	0	3.50320407	6.513865756	0	1.155788948	2.655672273	0	4.983937908	0.468962431	3.163887762	0	5.074951301
ENSG00000136238	RAC1	4783.996958	4834.421617	4609.374255	4672.601017	4268.328586	4764.276058	4052.893514	5965.773676	4274.592563	3787.173651	5392.87353	4217.284531
ENSG00000136240	KDELR2	3031.833463	2574.854992	2798.519575	2946.225224	2582.03251	2866.98791	2294.052125	2342.450817	2642.603301	2356.463605	2461.214218	2720.173897
ENSG00000136243	NUPL2	502.0243721	458.9197332	435.6147724	465.501911	358.294574	437.8065433	561.7669942	294.0523366	487.7209287	482.8092725	378.0116008	475.3537718
ENSG00000136244	IL6	11.24984587	0	0	3.348934611	1.155788948	2.27629052	0	0	0.468962431	1.265555105	0	0
ENSG00000136247	ZDHHC4	375.463606	318.7915704	424.2155073	333.2189938	391.8124535	358.1363751	243.0918993	279.1005229	290.7567075	235.3932495	252.467602	296.0388259
ENSG00000136261	BZW2	1953.25449	1170.07016	1381.753773	2997.296477	2347.407354	2408.694752	6403.122339	8221.836236	5899.547388	7239.607977	8342.4677	6839.342703
ENSG00000136267	DGKB	2.812461468	0	10.58503185	8.372336529	4.623155793	5.311344546	0	0	2.813774589	1.265555105	4.138813147	10.1499026
ENSG00000136270	TBRG4	705.9278285	490.4485699	696.1694026	860.6761951	700.4081026	896.8584648	623.0506663	534.9426688	697.3471356	724.5302975	593.2298844	678.3518239
ENSG00000136271	DDX56	794.5203648	641.0863449	843.5456153	872.3974663	640.3070773	898.7553736	711.9119908	588.1046732	725.015919	675.1736484	669.1081254	707.9557064
ENSG00000136273	HUS1	493.5869877	571.0222635	569.1490204	524.1082667	510.8587151	551.2416876	405.4936303	400.3763453	418.3144889	440.4131765	434.5753804	455.0539666
ENSG00000136274	NACAD	2.812461468	0	0	1.674467306	0	0.379381753	0	1.661312636	3.751699452	0	0	0
ENSG00000136279	DBNL	940.7683612	1124.528507	951.8386335	1007.192084	1156.944737	1095.654504	982.5815426	1162.918845	995.1382795	966.2513225	1132.655198	921.1036611
ENSG00000136280	CCM2	372.6511446	395.86206	353.3772172	276.2871054	314.3745939	328.1652166	231.8565594	260.8260839	269.6533981	210.7149249	222.1163056	246.1351381
ENSG00000136286	MYO1G	12.65607661	0	9.770798633	4.186168264	5.778944741	6.828871559	0	0	7.034436472	0.632777552	0	2.53747565
ENSG00000136295	TTYH3	101.2486129	182.1666117	117.2495836	132.2829172	183.7704428	187.0352044	40.8557814	64.79119281	45.02039342	73.40219608	75.87824103	58.36193996
ENSG00000136305	CIDEB	2.812461468	0	0	2.511700959	2.311577897	0	1.021394535	0	0	2.53111021	0	2.53747565
ENSG00000136319	TTC5	126.5607661	112.1025303	182.3882412	165.7722633	171.0567643	157.8228094	290.0760479	182.74439	293.5704821	296.7726721	248.3287888	267.2807685
ENSG00000136367	ZFHX2	5.624922937	3.50320407	4.885399317	10.88403749	1.155788948	11.00207085	13.27812895	9.967875817	5.627549177	4.429442867	2.759208765	4.229126084
ENSG00000136371	MTHFS	15.46853808	14.01281628	5.699632536	10.04680383	11.55788948	10.62268909	8.171156279	6.645250545	17.8205724	21.51443678	22.07367012	16.07067912
ENSG00000136379	ABHD17C	825.4574409	854.7817932	732.8098975	809.6049423	889.9574902	675.2995209	221.6426141	227.5998311	225.1019671	236.026027	182.1077785	230.064459
ENSG00000136381	IREB2	973.111668	889.8138339	879.371877	1101.799487	1070.260566	1058.09571	1140.897695	1074.869276	1061.730945	1090.275723	974.0006939	1348.245396
ENSG00000136383	ALPK3	26.71838395	101.592918	52.92515926	51.90848648	80.90522638	53.11344546	25.53486337	19.93575163	11.25509835	12.0227735	33.11050518	8.458252168

**ENSG00000136404**	TM6SF1	0	0	0	2.511700959	8.090522638	3.035054026	0	0	0.937924863	2.53111021	2.759208765	0.845825217
**ENSG00000136425**	CIB2	11.24984587	17.51602035	8.142332194	10.88403749	10.40210053	20.48661468	0	0	0.937924863	0.632777552	2.759208765	0
**ENSG00000136436**	CALCOCO2	3189.331305	3352.566295	3145.382927	1287.665358	1541.822457	1484.900182	1940.649616	2068.334232	1903.049547	1215.565678	1219.570274	1206.146759
**ENSG00000136444**	RSAD1	343.1202991	147.134571	355.8199169	378.4296111	353.6714182	316.0250005	180.7868327	119.6145098	151.0059029	175.9121596	142.0992514	169.1650434
**ENSG00000136448**	NMT1	2510.12186	2329.630707	2437.000026	2386.953144	2424.845213	2472.051505	1958.013323	2021.817478	2041.393464	1900.863767	1934.205344	2024.059744
**ENSG00000136449**	MYCBPAP	1.406230734	0	2.442699658	4.186168264	4.623155793	2.27629052	8.171156279	4.983937908	1.875849726	1.265555105	0	5.074951301
**ENSG00000136450**	SRSF1	1770.444494	1177.076568	1450.149364	1797.540653	1435.489874	1602.887908	1461.615579	1118.063404	1618.858313	1492.722246	1203.015021	1844.744798
**ENSG00000136451**	VEZF1	1013.892359	1082.490058	1222.978296	1047.3793	1027.496375	1181.015398	1151.111641	1196.145098	1163.495792	1074.456284	1125.757176	1091.11453
**ENSG00000136463**	TACO1	224.9969175	217.1986524	282.5389271	267.0775353	240.4041012	295.5383858	180.7868327	167.7925762	240.1087649	199.324929	169.691339	226.6811581
**ENSG00000136478**	TEX2	2515.746783	2080.903218	2432.114626	3828.669495	3248.922734	3498.658529	1567.840611	1918.816095	1839.270656	1769.878814	1779.689653	2163.620905
**ENSG00000136485**	DCAF7	1250.139123	980.8971397	1492.489491	1244.966442	1198.553139	1442.788808	1202.181368	1068.224025	1280.7364	1229.486784	1256.819592	1459.048499
**ENSG00000136490**	LIMD2	5.624922937	0	0	1.674467306	3.467366845	1.13814526	3.064183605	0	1.875849726	3.163887762	0	0
**ENSG00000136492**	BRIP1	832.4885946	1170.07016	749.9087951	754.3475212	740.8607158	620.2891667	913.1267142	1061.578774	815.5256683	995.3590899	1096.785484	846.671042
**ENSG00000136504**	KAT7	1354.200197	1741.092423	1661.035768	1486.089734	1705.944488	1554.706425	1747.606049	2076.640795	1699.988814	1706.601059	1870.743542	1709.412763
**ENSG00000136514**	RTP4	348.7452221	574.5254675	363.9622491	8.372336529	40.45261319	20.10723293	86.81853546	116.2918845	70.81332715	7.593330629	4.138813147	5.920776517
**ENSG00000136518**	ACTL6A	1134.828202	1103.509282	1085.372882	1307.758966	1041.365842	1146.491658	929.4690267	953.5934531	1000.765829	1030.794633	909.1592879	1080.118802
**ENSG00000136521**	NDUFB5	396.557067	413.3780803	327.3217542	375.0806765	327.0882724	369.138446	400.3866577	423.6347222	309.5152048	299.3037823	248.3287888	395.0003762
**ENSG00000136522**	MRPL47	599.0542928	483.4421617	571.5917201	771.9294279	612.5681426	695.4067538	441.2424391	538.2652941	494.2864028	494.1992684	488.3799513	514.2617318
**ENSG00000136527**	TRA2B	3891.040441	2841.098501	3622.523593	3755.830167	3028.167044	3442.130648	3941.56151	3624.984172	4144.689969	4038.386339	3753.903524	4295.100451
**ENSG00000136531**	SCN2A	0	0	0	0	0	0	1.021394535	6.645250545	0.468962431	0	5.518417529	0
**ENSG00000136536**	7-Mar	1736.694957	1671.028342	1771.771486	1742.283232	1715.190799	1709.49418	1537.198775	1629.747696	1465.038636	1525.626679	1692.774577	1612.988688
**ENSG00000136542**	GALNT5	23.90592248	70.06408141	171.8032093	51.90848648	132.9157291	155.1671371	10.21394535	6.645250545	9.848211061	1.898332657	16.55525259	13.53320347
**ENSG00000136560**	TANK	494.9932184	476.4357536	419.330108	375.0806765	357.138785	382.0374256	397.3224741	549.8944826	445.5143099	423.3281825	445.6122155	432.2166858
**ENSG00000136573**	BLK	458.4312193	511.4677943	301.2662912	989.6101777	1036.742687	734.4830744	0	1.661312636	2.344812157	0	0	0
**ENSG00000136574**	GATA4	52.03053716	38.53524477	58.6247918	72.8393278	107.4883722	100.1567829	3.064183605	14.95181373	5.158586746	10.75721839	22.07367012	11.84155303
**ENSG00000136603**	SKIL	1271.232584	1797.143688	1855.637507	1569.813099	2052.681172	2180.686318	2236.854031	1656.328698	2247.736934	2283.061409	1706.570621	2372.539733
**ENSG00000136628**	EPRS	6571.316221	6694.622979	6425.114335	6137.759909	6171.912984	6435.832063	6726.904407	5439.137571	6472.619479	6084.788944	5425.984036	6372.447183
**ENSG00000136630**	HLX	2.812461468	10.50961221	0.814233219	0	0	1.896908767	0	0	0.937924863	0	0	0
**ENSG00000136631**	VPS45	749.5209813	630.5767327	801.2054879	824.6751481	775.5343843	756.4872161	918.2336869	858.8986329	951.9937358	956.1268816	936.7513756	898.2663802
**ENSG00000136636**	KCTD3	1213.577124	1208.605404	1032.447722	1493.624837	1623.883472	1287.242289	1719.007002	1573.263066	1359.991051	1595.864987	1502.389172	1523.331215
**ENSG00000136643**	RPS6KC1	1013.892359	959.8779153	1036.518888	876.5836346	811.3638417	817.9470601	778.3026356	759.2198747	706.7263842	684.6653117	684.2837736	760.3968699
**ENSG00000136682**	CBWD2	269.996301	427.3908966	309.4086234	335.7306948	318.9977497	302.7466391	367.7020326	375.4566558	395.8042921	389.7909723	362.8359525	338.3300867
**ENSG00000136688**	IL36G	7.031153671	7.006408141	15.47043117	14.2329721	5.778944741	10.62268909	3.064183605	16.61312636	4.220661883	10.12444084	0	9.304077385
**ENSG00000136689**	IL1RN	15.46853808	3.50320407	21.17006371	4.186168264	4.623155793	6.070108053	4.08557814	0	3.751699452	1.265555105	1.379604382	0.845825217
**ENSG00000136695**	IL36RN	30.93707615	24.52242849	80.60908873	41.86168264	36.98524634	42.49075637	98.05387535	114.6305719	93.32352386	111.3688492	179.3485697	122.6446564
**ENSG00000136696**	IL36B	4.218692202	10.50961221	15.47043117	8.372336529	6.93473369	10.24330734	1.021394535	0	0.468962431	0	0	4.229126084
**ENSG00000136699**	SMPD4	821.2387487	655.0991612	733.6241307	881.6070365	822.9217312	962.8708899	752.7677722	667.8476797	838.035865	810.5880446	871.9099696	811.9922081
**ENSG00000136709**	WDR33	1320.450659	1303.191914	1389.081872	1389.807864	1176.593149	1289.139198	1492.257415	1531.730251	1315.908583	1454.755593	1513.426007	1256.896272
**ENSG00000136710**	CCDC115	68.90530597	94.5865099	67.58135721	88.7467672	119.0462617	83.46398573	217.5570359	249.1968954	198.8400709	190.4660433	240.0511625	214.8396051
**ENSG00000136715**	SAP130	707.3340593	795.227324	646.5011762	575.1795195	649.5533889	633.9469098	416.7289702	551.5557952	532.7413221	554.9459134	544.943731	512.5700814
**ENSG00000136717**	BIN1	199.6847643	203.1858361	210.0721706	184.1914036	194.1725433	188.9321131	38.81299233	131.2436983	100.8269228	46.19276132	120.0255813	82.89087124
**ENSG00000136718**	IMP4	414.8380666	245.2242849	376.9899806	518.2476311	343.2693176	500.7839144	369.7448216	260.8260839	390.176743	434.0854009	280.0596896	414.4543562
**ENSG00000136720**	HS6ST1	324.8392996	259.2371012	389.2034789	293.0317785	286.6356592	306.5404567	315.6109113	338.9077778	362.0389971	468.2553888	492.5187645	447.4415397
**ENSG00000136731**	UGGT1	2837.773622	2893.646562	2891.342162	2459.792472	2514.996751	2584.727885	2928.338131	2545.130959	2797.360904	2669.055716	2617.109513	2894.413892
**ENSG00000136732**	GYPC	0	7.006408141	5.699632536	0	0	1.517527013	1.021394535	3.322625272	1.406887294	0.632777552	2.759208765	0.845825217
**ENSG00000136738**	STAM	1217.795816	1240.134241	1285.674254	1167.103712	1132.673169	1478.071311	634.2860062	559.8623584	660.2991035	673.9080933	571.1562143	721.4889099
**ENSG00000136754**	ABI1	1112.328511	1404.784832	1222.978296	1093.427151	1140.763692	1177.600962	853.8858312	983.4970806	833.8152031	795.4013833	863.6323433	833.1378385
**ENSG00000136758**	YME1L1	2124.814639	2084.406422	2328.707008	2085.549029	2221.426359	2313.849313	1979.462609	2139.770675	1958.856076	1973.633186	1939.723762	2002.914113
**ENSG00000136770**	DNAJC1	438.7439891	532.4870187	534.136992	266.2403016	389.5008756	345.6167773	363.6164544	353.8595915	348.908049	255.6421312	286.9577115	249.5184389
**ENSG00000136783**	NIPSNAP3A	355.7763757	455.4165292	338.7210193	308.9392179	406.8377098	418.4580739	546.4460762	496.7324782	549.1550072	553.6803583	444.2326111	545.5572648
**ENSG00000136802**	LRRC8A	2130.439562	2185.99934	2511.095249	2217.831946	1896.649664	2875.334308	945.8113393	1083.175839	1159.744093	1051.676292	1034.703287	1202.763458
**ENSG00000136807**	CDK9	646.8661377	690.1312019	696.1694026	570.1561176	613.7239315	608.1489505	650.6283187	666.1863671	578.6996404	584.6864584	655.3120816	567.5487205
**ENSG00000136810**	TXN	7183.02659	6400.353837	7370.439102	5314.759228	4980.294578	5429.711653	2197.019645	2128.141487	2694.189169	2140.053682	1972.834267	2605.141668
**ENSG00000136811**	ODF2	1275.451276	977.3939356	1203.436698	1487.764201	1330.313079	1333.147481	1486.129048	1365.598987	1709.368063	1705.335504	1609.998314	1718.71684
**ENSG00000136813**	ECPAS	6225.38346	6792.712693	7197.007427	5256.990106	5166.376599	5413.77762	4360.333269	3872.519755	4351.971364	3970.679141	3777.356799	3960.99949
**ENSG00000136816**	TOR1B	246.0903785	245.2242849	319.179422	341.5913304	343.2693176	351.3075036	283.9476807	337.2464651	307.639355	310.0610007	246.9491844	404.3044536
**ENSG00000136819**	C9orf78	712.9589822	686.6279978	855.7591136	946.9112614	931.5658923	1038.747241	804.8588935	824.0110675	848.3530385	878.9280203	903.6408704	912.6454089
**ENSG00000136824**	SMC2	3196.362459	3804.47962	3065.588071	4547.853202	4531.848466	3895.491843	4218.359429	4611.803878	4232.385944	4843.279386	5038.315204	5312.628187
**ENSG00000136826**	KLF4	3595.731987	3681.867478	3268.332143	3221.675096	3089.423859	3307.829507	1730.242342	2156.383802	1880.53935	1232.017894	1524.462842	1349.937046
**ENSG00000136827**	TOR1A	576.554601	553.5062431	605.7895153	606.9943983	522.4166046	633.9469098	627.1362444	559.8623584	621.8441841	541.6575848	587.7114669	569.2403709
**ENSG00000136828**	RALGPS1	286.8710698	392.3588559	346.0491183	310.6136852	338.6461618	301.9878756	454.520568	430.2799728	342.342575	513.8153725	418.0201278	344.2508632
**ENSG00000136830**	FAM129B	4270.72274	4431.553149	4450.598777	5259.501807	4819.639914	6278.009254	1436.080716	1181.193284	2006.690244	1564.858887	1223.709087	1929.327319
**ENSG00000136840**	ST6GALNAC	112.4984587	73.56728548	72.46675653	136.4690854	154.8757191	141.8887757	75.58319558	64.79119281	123.8060819	110.1032941	113.1275593	144.6361121
**ENSG00000136842**	TMOD1	16.87476881	31.52883663	21.17006371	4.186168264	30.05051265	11.00207085	55.15530488	96.3561329	34.2342575	42.39609601	75.87824103	57.51611474
**ENSG00000136848**	DAB2IP	15.46853808	31.52883663	25.2412298	15.9074394	10.40210053	40.5938476	30.64183605	68.11381808	27.19982102	20.24888168	35.86971394	46.52038692
**ENSG00000136854**	STXBP1	708.74029	788.2209158	701.8690352	860.6761951	861.0627665	879.0275224	248.198872	272.4552723	249.4880135	289.1793414	264.8840414	271.5098946
**ENSG00000136856**	SLC2A8	88.59253625	52.54806106	82.23755516	96.28187008	95.93048271	125.9547421	83.75435186	59.8072549	126.1508941	120.8605125	118.6459769	130.2570834

**ENSG00000136861**	CDK5RAP2	2676.057087	2189.502544	2682.084225	2784.639129	2614.394601	2700.059938	2954.894389	2928.894177	2753.278435	2810.16511	2715.061424	2817.443797
**ENSG00000136866**	ZFP37	73.12399818	101.592918	87.12295448	82.88613163	76.28207059	70.94438787	201.2147234	222.6158932	148.1921283	201.8560392	169.691339	192.0023242
**ENSG00000136867**	SLC31A2	175.7788418	231.2114686	227.1710682	163.2605623	160.6546638	201.451711	218.5784305	149.5181373	168.3575129	220.2065882	144.8584601	153.0943642
**ENSG00000136868**	SLC31A1	1115.140972	1057.967629	1161.910804	907.5612797	955.8374602	1148.009185	938.6615776	1159.59622	1127.854648	933.9796673	1129.895989	1091.960355
**ENSG00000136869**	TLR4	317.8081459	329.3011826	162.0324107	42.6989163	70.50312584	25.79795922	4.08557814	0	0.468962431	2.53111021	0	0
**ENSG00000136870**	ZNF189	435.9315276	535.9902228	447.0140375	426.1519293	392.9682424	441.2209791	500.4833221	601.3951743	509.2932006	507.487597	514.5924346	515.9533822
**ENSG00000136874**	STX17	2169.814023	2157.973707	1919.147698	1522.928015	1628.506628	1416.990849	2379.849266	2485.323704	2210.219939	1952.751527	1844.531059	1904.798388
**ENSG00000136875**	PRPF4	1382.324812	1313.701526	1558.442382	1460.972724	1334.936235	1555.465189	1117.405621	1059.917462	1197.73005	1087.111835	945.0290019	1270.429476
**ENSG00000136877**	FPGS	396.557067	238.2178768	339.5352525	444.5710697	361.7619408	402.5240403	199.1719343	141.2115741	285.5981208	227.1671413	206.9406573	257.1308659
**ENSG00000136878**	USP20	241.8716863	189.1730198	265.4400295	241.123292	292.4146039	298.1940581	339.1029856	269.1326471	387.3629684	332.8409926	354.5583263	331.563485
**ENSG00000136881**	BAAT	9.843615139	0	6.513865756	0	5.778944741	9.484543833	12.25673442	4.983937908	5.158586746	8.226108181	9.657230676	10.1499026
**ENSG00000136888**	ATP6V1G1	1411.855657	1415.294444	1520.987654	1280.967489	1403.127783	1382.467109	989.7313043	1030.013834	1082.365292	960.5563245	859.4935302	1029.369289
**ENSG00000136891**	TEX10	1669.195881	1418.797649	1606.482142	1947.405477	1634.285573	1748.191119	1685.300983	1433.712805	1534.445076	1712.296057	1485.83392	1729.712568
**ENSG00000136895**	GARNL3	126.5607661	87.58010176	105.8503185	67.81592588	79.74943743	52.35468196	27.57765244	24.91968954	24.85500887	34.80276538	20.69406573	22.83728085
**ENSG00000136897**	MRPL50	497.8056799	367.8364274	442.1286382	571.8305849	530.5071273	490.9199888	480.0554314	392.0697821	415.5007143	464.4587234	390.4280402	549.7863909
**ENSG00000136908**	DPM2	642.6474455	427.3908966	651.3865756	684.0198944	542.0650167	738.6562737	455.5419626	483.4419771	530.39651	546.7198053	448.3714243	534.561537
**ENSG00000136918**	WDR38	5.624922937	3.50320407	6.513865756	8.372336529	13.86946738	7.208253313	1.021394535	8.306563181	7.034436472	12.65555105	5.518417529	3.383300867
**ENSG00000136925**	TSTD2	473.8997574	472.9325495	455.1563697	460.4785091	510.8587151	492.4375158	634.2860062	725.993622	619.9683344	622.020334	572.5358187	559.0904683
**ENSG00000136928**	GABBR2	0	0	13.84196473	9.209570182	5.778944741	17.83094241	2.04278907	0	2.344812157	0	2.759208765	4.229126084
**ENSG00000136930**	PSMB7	4187.755126	4841.428025	4983.921536	3791.831214	3863.802454	4189.892083	2084.666246	2197.916618	2312.922712	2149.545345	2045.953299	2548.471378
**ENSG00000136932**	TRMO	277.0274546	206.6890402	249.1553652	236.0998901	206.8862217	228.0084337	248.198872	194.3735784	239.17084	250.5799107	198.6630311	228.3728085
**ENSG00000136933**	RABEPK	374.0573753	283.7595297	343.6064186	462.1529764	369.8524634	518.235475	310.5039386	159.4860131	353.1287109	311.9593333	205.561053	416.1460067
**ENSG00000136935**	GOLGA1	679.2094446	924.8458746	732.8098975	606.9943983	708.4986253	581.5922278	741.5324323	843.9468192	685.1541124	691.6258648	869.1507609	697.8058038
**ENSG00000136936**	XPA	362.8075294	353.8236111	278.4677611	344.940265	302.8167044	273.5342441	351.35972	383.7632189	308.1083175	296.1398945	328.345843	367.9339693
**ENSG00000136937**	NCBP1	2234.500637	1842.685341	2147.133	2469.002042	2355.497877	2567.655706	2590.25654	2309.224564	2506.604196	2619.699067	2454.316196	2973.075637
**ENSG00000136938**	ANP32B	4750.24742	4336.966639	4076.051497	5000.796609	4639.336838	4079.112612	4507.414082	3762.873121	4064.497393	4228.852383	3993.954687	4580.989374
**ENSG00000136940**	PDCL	510.4617565	437.9005088	474.6979669	437.0359668	380.254564	442.3591244	511.718662	468.4901634	439.4177983	480.2781623	481.4819294	547.2489153
**ENSG00000136942**	RPL35	5118.679872	5006.078617	4449.784544	4974.842365	5129.391352	4902.371016	3922.155014	3922.359134	4115.145336	4031.425786	3900.141589	4233.35521
**ENSG00000136943**	CTSV	300.9333771	353.8236111	302.0805244	462.99021	570.9597404	507.2334042	78.64737919	124.5984477	116.302683	109.4705166	151.7564821	197.9231007
**ENSG00000136950**	ARPC5L	1258.576507	1061.470833	1263.689957	1394.831266	1018.250063	1265.617529	773.1956629	702.7352451	845.0703015	803.6274915	751.8843884	949.8617184
**ENSG00000136960**	ENPP2	9.843615139	0	0.814233219	1.674467306	0	2.27629052	200.1933288	26.58100218	115.3647581	192.9971535	81.39665856	100.6532008
**ENSG00000136982**	DSCC1	365.6199909	374.8428355	345.234885	465.501911	423.0187551	400.6271315	312.5467277	299.0362745	359.2252225	330.9426599	297.9945466	514.2617318
**ENSG00000136986**	DERL1	1379.51235	1548.416199	1446.078198	1321.154704	1297.950989	1376.397001	1263.46504	1355.631111	1209.454111	1067.495731	1211.292648	1185.846954
**ENSG00000136997**	MYC	1469.511117	1152.554139	1518.544954	1268.408984	910.7616912	1294.071161	1140.897695	739.2841231	1086.585954	1132.671819	608.4055326	1047.977444
**ENSG00000136999**	NOV	5.624922937	52.54806106	40.71166097	3.348934611	2.311577897	11.00207085	1.021394535	1.661312636	6.096511609	1.265555105	0	0
**ENSG00000137038**	DMAC1	226.4031482	175.1602035	166.1035768	291.3573112	250.8062018	245.4599944	165.4659146	126.2597603	181.9574234	178.4432698	136.5808338	197.0772755
**ENSG00000137040**	RANBP6	614.5228308	721.6600385	654.6435084	714.9975395	648.3976	724.2397671	402.4294467	378.779281	361.1010722	336.6376579	418.0201278	472.8162962
**ENSG00000137054**	POLR1E	594.8356005	469.4293454	553.6785892	712.4858386	547.8439615	720.4459495	505.5902948	360.504842	562.7549177	460.6620581	416.6405235	562.4737692
**ENSG00000137055**	PLAA	1421.699272	1730.582811	1537.272318	1361.34192	1369.609904	1473.139348	1064.293105	960.2387037	969.8143082	940.9402204	841.5586732	985.3863775
**ENSG00000137070**	IL11RA	59.06169083	84.07689769	48.85399317	40.18721534	49.69892478	31.48868552	123.5887387	119.6145098	88.63389954	84.79219202	63.46180159	59.20776517
**ENSG00000137073**	UBAP2	1314.825736	1310.198322	1290.559653	1689.537511	1537.199301	1483.762037	1262.443645	1216.08085	1148.957957	1261.758439	1346.493877	1223.063263
**ENSG00000137074**	APTX	997.0175905	564.0158553	865.5299123	864.8623634	850.6606659	852.0914179	928.4476322	740.9454357	838.9737899	860.5774712	802.9297505	882.1957011
**ENSG00000137075**	RNF38	579.3670625	655.0991612	545.536257	619.5529031	625.281821	593.7324439	806.9016826	912.0606372	815.9946307	813.7519324	881.5672003	846.671042
**ENSG00000137076**	TLN1	3006.52131	3338.553479	3206.450418	3037.483693	3313.646915	3331.351176	3075.418945	3327.60921	3395.756966	3055.6828	3537.305636	3069.499712
**ENSG00000137090**	DMRT1	0	3.50320407	0	1.674467306	2.311577897	3.035054026	0	0	5.627549177	0.632777552	1.379604382	3.383300867
**ENSG00000137094**	DNAJB5	39.37446056	84.07689769	43.15436063	48.55955187	46.23155793	38.69693884	118.481766	117.9531972	143.502504	133.5160636	129.6828119	155.6318399
**ENSG00000137098**	SPAG8	7.031153671	3.50320407	1.628466439	0	4.623155793	0	3.064183605	0	3.28273702	2.53111021	0	0
**ENSG00000137100**	DCTN3	322.0268381	357.3268152	304.5232241	425.3146957	419.5513882	447.6704689	377.9159779	485.1032897	394.3974049	447.3737295	474.5839075	374.700571
**ENSG00000137101**	CD72	4.218692202	3.50320407	1.628466439	6.697869223	3.467366845	1.517527013	0	0	1.406887294	0	0	0
**ENSG00000137103**	TMEM8B	29.53084542	31.52883663	33.383562	27.62871054	25.42735686	34.52373955	14.29952349	21.59706427	22.97915914	24.04554699	33.11050518	23.68310607
**ENSG00000137106**	GRHPR	523.1178331	497.454978	607.4179817	622.0646041	592.9197305	644.1902171	575.0451231	505.0390414	576.8237907	632.7775524	539.4253135	562.4737692
**ENSG00000137124**	ALDH1B1	199.6847643	178.6634076	208.4437042	357.4987698	236.9367344	334.9940882	210.4072742	106.3240087	159.9161891	246.7832454	157.2748996	222.452032
**ENSG00000137133**	HINT2	18.28099954	10.50961221	13.84196473	14.2329721	25.42735686	12.89897961	9.192550814	11.62918845	10.31717349	17.08499391	16.55525259	8.458252168
**ENSG00000137135**	ARHGEF39	502.0243721	231.2114686	356.6341501	546.7135753	518.9492378	524.6849648	479.0340369	435.2639107	561.8169929	547.9853604	442.8530067	622.5273595
**ENSG00000137145**	DENND4C	1223.420739	1390.772016	1389.081872	1059.937805	1192.774195	1034.19466	1911.029175	1805.846835	1540.541587	1616.746646	1685.876555	1549.551797
**ENSG00000137154**	RPS6	7350.368047	7668.51371	7030.089617	8299.497201	7973.787954	7632.781495	5005.854615	5738.173845	4897.374672	5114.108178	5405.28997	5520.70119
**ENSG00000137161**	CNPY3	434.5252969	609.5575083	528.4373594	436.1987331	587.1407857	571.3489205	561.7669942	569.8302342	581.0444526	578.9914604	640.1364334	610.6858065
**ENSG00000137166**	FOXP4	323.4330689	381.8492437	359.891083	316.4743208	340.9577397	445.0147966	246.1560829	222.6158932	295.9152942	236.6588046	264.8840414	256.2850407
**ENSG00000137168**	PPIL1	693.2717519	444.9069169	723.0390989	735.9283809	531.6629162	690.8541728	639.3929788	365.4887799	687.9678869	536.5953644	437.3345892	625.0648352
**ENSG00000137171**	KLC4	106.8735358	192.6762239	122.9492161	112.1893095	132.9157291	132.0248501	192.0221726	264.1487091	214.7847936	218.3082556	336.6234693	179.314946
**ENSG00000137177**	KIF13A	1361.231351	980.8971397	1283.231554	1360.504686	1338.403602	1342.632025	2225.618691	2427.177761	2070.938097	1579.412771	1999.04675	1700.954511
**ENSG00000137185**	ZSCAN9	634.2100611	430.8941007	608.2322149	533.3178369	432.2650667	494.3344246	624.0720608	627.9761765	593.2374758	503.6909317	498.037182	479.5828979
**ENSG00000137193**	PIM1	1486.385886	1905.743014	2011.156052	1261.711115	1212.422607	1640.446701	1032.629875	787.4621895	1060.324058	850.4530304	671.8673342	851.7459933
**ENSG00000137198**	GMPR	0	0	0	0	1.155788948	2.27629052	1.021394535	6.645250545	0	0	5.518417529	0
**ENSG00000137200**	CMTR1	898.5814391	1071.980446	1060.131652	724.2071097	699.2523137	726.8954393	1537.198775	1157.934907	1522.721015	1227.588452	1128.516385	1238.288117
**ENSG00000137203**	TFAP2A	2393.40471	2112.432054	2356.390937	2345.928695	2143.988499	2182.203845	1825.232034	2189.610054	1852.401604	1704.069949	1585.165435	1579.15568
**ENSG00000137207**	YIPF3	590.6169083	578.0286716	600.904116	502.3401917	611.4123536	596.0087344	638.3715843	613.0243627	687.0299621	575.1947951	662.2101035	598.8442535

ENSG00000137210	TMEM14B	1300.763429	1397.778424	1086.187115	1347.946181	1303.729934	1285.724762	757.8747449	832.3176307	737.6779047	730.2252954	693.9410043	748.5553168
ENSG00000137216	TMEM63B	409.2131436	662.1055693	569.9632536	490.6189206	494.6776699	529.2375459	318.6750949	431.9412854	291.6946324	322.0837742	332.4846561	294.3471754
ENSG00000137218	FRS3	14.06230734	7.006408141	8.142332194	15.07020575	15.02525633	11.3814526	13.27812895	13.29050109	12.19302322	12.0227735	13.79604382	17.76232955
ENSG00000137221	TJAP1	448.5876042	430.8941007	576.4771194	449.5944716	533.9744941	534.5488904	536.2321308	667.8476797	527.5827354	542.2903624	700.8390262	466.8955197
ENSG00000137261	KIAA0319	23.90592248	98.08971397	37.45472809	37.67551438	70.50312584	31.10930377	20.4278907	23.25837691	11.72406079	43.66165111	19.31446135	31.29553302
ENSG00000137266	SLC22A23	516.0866794	381.8492437	341.9779522	752.6730539	606.7891978	758.0047431	860.0141984	475.1354139	919.1663656	1040.919074	729.8107182	957.4741454
ENSG00000137267	TUBB2A	226.4031482	189.1730198	169.3605096	469.6880793	278.5451365	322.4744903	23.4920743	28.24231481	18.75849726	15.81943881	16.55525259	26.22058172
ENSG00000137269	LRRC1	641.2412148	571.0222635	523.5519601	607.831632	681.9154795	662.0211595	1250.186911	1373.90555	1216.488547	1297.82676	1458.241832	1388.845006
ENSG00000137270	GCM1	1.406230734	0	0	0	0	0	4.08557814	1.661312636	0	8.858885733	0	0.845825217
ENSG00000137273	FOXF2	2.812461468	0	0	1.674467306	1.155788948	1.517527013	43.919965	84.72694444	20.63434698	36.06832049	107.6091418	22.83728085
ENSG00000137274	BPHL	133.5919197	161.1473872	153.8900785	122.2361133	167.5893975	102.0536916	154.2305748	112.9692593	130.8405184	150.6010575	150.3768777	138.7153356
ENSG00000137275	RIPK1	1286.701122	1464.339301	1303.587384	949.4229624	1117.647913	983.3575046	1182.774871	1345.663235	1124.102948	1054.207402	1208.533439	993.8446297
ENSG00000137285	TUBB2B	5.624922937	0	5.699632536	9.209570182	11.55788948	2.655672273	0	0	0	0	0	0.845825217
ENSG00000137288	UQCC2	483.7433725	332.8043867	483.6545324	560.9465474	462.3155793	550.482924	342.1671692	328.939902	380.7974943	367.6437579	444.2326111	384.0046484
ENSG00000137309	HMGA1	10550.9492	8915.654359	10390.43011	7890.927178	7236.394605	8524.707997	3391.029856	2993.68537	4321.957768	2976.585606	2869.577115	3606.598724
ENSG00000137310	TCF19	1852.005877	3187.915704	2100.721706	2385.278677	3224.651166	2530.855676	2769.000584	3460.514221	2709.664929	2585.529079	3715.274602	2706.640694
ENSG00000137312	FLOT1	3006.52131	3314.031051	3195.865386	2774.592326	3487.015257	3386.740912	2410.491102	2963.781743	2554.907327	2565.280197	3128.942739	2507.025943
ENSG00000137331	IER3	1698.726727	4666.267822	2991.492848	2096.433067	4477.526386	3097.272634	473.9270642	2653.11628	542.1205708	455.5998377	2687.469337	581.9277491
ENSG00000137337	MDC 1.00	4003.5389	3510.210479	4323.578395	5746.771793	5223.010257	5271.888844	5265.288827	5952.483175	4968.656961	5568.442461	6197.182885	5494.480608
ENSG00000137338	PGBD1	77.34269038	98.08971397	85.49448804	88.7467672	142.1620406	92.18976605	42.89857046	58.14594226	31.42048291	24.67832454	26.21248326	32.98718345
ENSG00000137343	ATAT1	288.2773005	231.2114686	288.2385597	272.9381708	257.7409355	257.9795922	244.1132938	235.9063943	233.0743284	240.4554699	234.532745	197.9231007
ENSG00000137364	TPMT	376.8698368	476.4357536	489.3541649	640.4837444	528.1955494	610.0458593	482.0982205	495.0711656	491.9415906	410.6726315	402.8444796	520.1825083
ENSG00000137393	RNF144B	2197.938637	3096.832398	1821.439712	1946.568243	2450.27257	1568.74355	5143.742878	8811.602222	4674.617517	4616.745022	8244.515789	4048.965313
ENSG00000137404	NRM	551.2424478	511.4677943	495.0537974	907.5612797	821.7659422	775.8356855	750.7249831	885.4796351	930.8904264	856.1480284	1051.258539	965.0865723
ENSG00000137409	MTCH1	1272.638814	1590.454648	1400.481137	1497.811005	1708.256066	1671.176623	1422.802587	1604.828006	1487.07987	1561.062222	1703.811412	1576.618204
ENSG00000137411	VARS2	43.59315276	63.05767327	52.92515926	59.44358935	23.11577897	39.07632059	38.81299233	71.43644335	33.76529506	36.06832049	60.70259282	24.52893129
ENSG00000137413	TAF8	738.2711354	553.5062431	656.2719749	664.7635204	657.6439116	564.1406672	891.6774289	833.9789433	871.3321976	665.6819851	623.5811808	634.3689126
ENSG00000137414	FAM8A1	1063.110435	1271.663078	1009.649192	1235.756872	1346.494125	1186.326743	1729.220948	1388.857364	1588.844718	1681.922734	1601.720688	1938.631397
ENSG00000137434	C6orf52	5.624922937	0	9.770798633	4.186168264	8.090522638	3.41443578	9.192550814	3.322625272	4.220661883	10.12444084	2.759208765	5.074951301
ENSG00000137440	FGFBP1	1908.255106	956.3747112	1214.02173	2024.430973	1299.106778	1351.737187	44.94135953	112.9692593	103.1717349	27.8422123	121.4051856	136.1778599
ENSG00000137449	CPEB2	21.09346101	31.52883663	52.11092604	32.65211246	19.64841212	54.63097248	157.2947584	299.0362745	206.3434698	157.5616105	277.3004808	233.4477598
ENSG00000137460	FHDC1	132.185689	189.1730198	134.3484812	138.9807864	97.08627165	106.9856544	296.2044151	280.7618355	352.190786	270.1960149	262.1248326	340.0217371
ENSG00000137462	TLR2	0	7.006408141	4.885399317	0.837233653	0	2.655672273	2.04278907	4.983937908	0.937924863	3.796665314	0	1.691650434
ENSG00000137463	MGARP	8.437384405	0	0.814233219	0.837233653	2.311577897	3.035054026	14.29952349	0	2.344812157	2.53111021	5.518417529	2.53747565
ENSG00000137474	MYO7A	2.812461468	10.50961221	9.770798633	0	5.778944741	2.655672273	0	3.322625272	4.220661883	0	5.518417529	4.229126084
ENSG00000137478	FCHSD2	673.5845217	749.6856711	783.2923571	726.7188107	743.1722937	797.4604454	627.1362444	824.0110675	724.0779942	679.6030913	935.3717712	636.9063882
ENSG00000137486	ARRB1	392.3383748	423.8876925	337.0925529	437.0359668	417.2398103	341.443578	540.3177089	323.955964	475.5279055	600.5058972	466.3062812	510.8784309
ENSG00000137492	THAP12	1153.109202	1215.611812	1063.388585	1435.855715	1301.418356	1350.978424	1127.619567	1134.67653	1004.048566	1207.33957	1194.737395	1187.538604
ENSG00000137494	ANKRD42	170.1539188	108.5993262	131.0915483	155.7254594	171.0567643	181.7238598	207.3430906	194.3735784	226.5088544	213.8788127	175.2097566	257.1308659
ENSG00000137496	IL18BP	364.2137601	311.7851623	433.986306	84.56059894	115.5788948	101.6743099	227.7709813	322.2946514	174.9229869	141.7421717	186.2465916	135.3320347
ENSG00000137497	NUMA1	3178.081459	2326.127503	2924.725724	3807.738653	3252.3901	3642.823595	3633.100361	3001.991933	4118.428073	4176.964623	3486.260274	4090.410748
ENSG00000137500	CCDC90B	992.7988983	1229.624629	1190.408967	812.9538769	916.540636	945.798711	818.1370224	795.7687527	806.6153821	734.6547383	718.7738832	849.2085176
ENSG00000137501	SYTL2	15.46853808	66.56087734	52.92515926	45.21061725	67.035759	70.18562436	7.149761744	46.51675381	30.01359561	20.24888168	41.38813147	29.60388259
ENSG00000137502	RAB30	85.78007478	108.5993262	107.478785	135.6318518	162.9662417	142.6475392	577.0879122	571.4915468	834.753128	568.8670196	493.8983689	928.716088
ENSG00000137504	CREBZF	1766.225802	1800.646892	1732.688291	1476.880164	1644.687673	1673.832296	1889.57989	1835.750463	1830.829332	1840.117122	1945.242179	1894.648486
ENSG00000137507	LRRC32	0	0	0.814233219	0	0	0	0	0	0.468962431	0.632777552	9.657230676	2.53747565
ENSG00000137509	PRCP	451.4000657	360.8300193	380.2469135	413.5934245	560.5576399	470.4333741	759.9175339	779.1556263	549.6239697	720.7336322	931.2329581	687.6559012
ENSG00000137513	NARS2	269.996301	182.1666117	267.068496	302.2413487	270.4546139	320.9569633	272.7123408	260.8260839	277.6257594	281.5860108	249.7083932	329.0260093
ENSG00000137522	RNF121	237.6529941	308.2819582	308.5943902	288.8456102	334.023006	333.8559429	346.2527473	380.4405937	278.5636843	318.2871088	318.6886123	307.0345537
ENSG00000137547	MRPL15	1229.045662	945.865099	1098.400613	1104.311188	940.8122039	1050.508075	715.9975689	451.877037	615.7476725	699.2191954	635.9976202	738.4054142
ENSG00000137558	PI15	4.218692202	0	8.956565414	0.837233653	2.311577897	3.41443578	0	1.661312636	2.813774589	1.898332657	0	0
ENSG00000137563	GGH	821.2387487	1026.438793	903.7988736	858.1644942	1097.999501	1050.508075	456.5633571	538.2652941	606.3684239	492.9337133	591.85028	654.6687178
ENSG00000137571	SLCO5A1	12.65607661	3.50320407	0.814233219	0	0	0	0	0	0.468962431	0	0	0
ENSG00000137573	SULF1	46.40561423	87.58010176	69.20982365	144.8414219	84.37259322	108.1237997	1.021394535	19.93575163	30.95152048	0.632777552	1.379604382	36.37048432
ENSG00000137574	TGS1	1479.354732	1152.554139	1231.934861	1207.290927	895.7364349	1093.378213	918.2336869	752.5746242	770.9742373	861.2102488	800.1705417	927.8702628
ENSG00000137575	SDCBP	2134.658254	2820.079277	2775.721045	2117.363908	2459.518882	3012.670503	2164.335019	2304.240626	1825.201783	2200.800327	2148.044023	1926.789844
ENSG00000137601	NEK1	485.1496033	525.4806106	541.4650909	481.4093504	398.7471872	408.5941483	397.3224741	466.8288508	362.5079595	367.0109804	445.6122155	384.0046484
ENSG00000137628	DDX60	2871.523159	4596.20374	2862.029766	535.8295378	820.6101533	586.9035724	1687.343772	2207.884493	1432.680228	380.299309	411.1221059	356.9382415
ENSG00000137642	SORL1	912.6437465	686.6279978	644.8727098	1880.426784	1843.483372	1413.197031	368.7234271	632.9601144	609.6511609	440.4131765	881.5672003	662.2811447
ENSG00000137648	TMPRSS4	136.4043812	287.2627338	157.9612446	178.3307681	239.2483123	151.3733196	19.40649616	23.25837691	40.79973154	16.45221636	38.6289227	36.37048432
ENSG00000137656	BUD13	403.5882207	525.4806106	449.4567371	362.5221717	338.6461618	434.3921075	360.5522708	425.2960348	432.8523242	371.4404232	349.0399087	373.8547458
ENSG00000137672	TRPC6	0	0	0	0	2.311577897	3.793817533	12.25673442	14.95181373	19.69642212	18.35054902	19.31446135	30.4497078
ENSG00000137691	C11orf70	29.53084542	63.05767327	16.28466439	37.67551438	70.50312584	24.65981396	211.4286687	111.3079466	128.0267438	231.5965842	146.2380645	147.1735877
ENSG00000137692	DCUN1D5	856.3945171	542.9966309	659.5289078	854.8155596	681.9154795	778.111976	534.1893417	397.05372	503.1966889	559.3753563	460.7878637	631.8314369
ENSG00000137693	YAP1	7453.022891	7132.523487	7086.271709	8245.914247	8569.019262	8339.190319	10176.15375	9050.831242	9788.652832	10305.41522	9602.046501	10758.05093
ENSG00000137699	TRIM29	1421.699272	1107.012486	1415.951569	2141.643684	2157.857966	2315.36684	6477.68414	8662.084085	6941.112948	6715.035386	8921.90154	6594.05339
ENSG00000137700	SLC37A4	410.6193744	325.7979785	403.0454436	521.5965657	465.7829462	486.7467895	373.8303998	373.7953431	339.0598379	396.1187478	435.9549848	405.9961041

ENSG00000137709	POU2F3	80.15515185	42.03844885	38.26896131	81.21166433	25.42735686	46.66395566	127.6743169	63.12988017	69.87540229	111.3688492	89.67428485	82.04504603
ENSG00000137710	RDX	3674.480908	4144.290415	3599.725063	4686.833989	4999.94299	4464.185091	4673.901392	4483.882805	4535.335675	4882.511594	4525.102374	5421.73964
ENSG00000137713	PPP2R1B	1494.82327	1709.563586	1494.117958	1903.869327	1685.140287	1805.098382	1028.544297	1036.659085	1145.67522	1142.79626	936.7513756	1358.395298
ENSG00000137714	FDX1	367.0262216	458.9197332	397.3458111	527.4572013	487.7429362	557.6911774	614.87951	470.151476	664.9887278	628.3481095	444.2326111	884.7331767
ENSG00000137720	C11orf1	125.1545353	210.1922442	161.2181775	122.2361133	146.7851964	132.4042319	125.6315278	234.2450817	137.4059924	118.9621798	165.5525259	119.2613556
ENSG00000137727	ARHGAP20	14.06230734	7.006408141	15.47043117	2.511700959	5.778944741	1.517527013	28.59904698	9.967875817	27.66878346	8.858885733	19.31446135	15.2248539
ENSG00000137745	MMP13	7.031153671	3.50320407	9.770798633	6.697869223	2.311577897	2.27629052	0	4.983937908	0	0	4.138813147	0
ENSG00000137747	TMPRSS13	1.406230734	0	5.699632536	4.186168264	0	3.41443578	28.59904698	24.91968954	87.22701225	62.01220013	62.0821972	113.340579
ENSG00000137752	CASP1	2323.093173	2697.467134	2222.042456	152.3765248	255.4293576	176.0331335	98.05387535	54.82331699	94.73041115	29.10776741	11.03683506	25.3747565
ENSG00000137760	ALKBH8	554.0549093	535.9902228	596.8329499	658.9028848	595.2313084	643.4314536	377.9159779	277.4392102	361.5700347	424.5937376	350.4195131	437.2916371
ENSG00000137764	MAP2K5	258.7464551	126.1153465	200.301372	237.7743574	208.0420107	219.2826534	178.7440436	176.0991394	193.6814842	220.8393658	220.7367012	170.0108686
ENSG00000137766	UNC13C	11.24984587	3.50320407	13.84196473	5.023401917	23.11577897	14.41650663	35.74880872	41.5328159	37.98595695	49.35664909	77.25784541	36.37048432
ENSG00000137767	SQOR	440.1502198	641.0863449	519.480794	231.9137218	316.6861718	240.1486498	25.53486337	26.58100218	25.3239713	17.08499391	27.59208765	7.612426951
ENSG00000137770	CTDSPL2	1276.857507	1639.499505	1379.311074	1265.897283	1323.378346	1305.073231	1117.405621	1559.972565	1198.667975	1163.045141	1252.680779	1259.433748
ENSG00000137776	SLTM	1777.475648	1863.704565	1891.463769	1963.312916	1733.683422	1848.727284	1240.99436	1269.242854	1163.495792	1073.190729	1082.98944	1124.101713
ENSG00000137801	THBS1	6827.250214	3846.518069	7801.168476	13288.57254	9408.122039	17036.13763	136.8668677	68.11381808	206.3434698	205.019927	77.25784541	357.7840667
ENSG00000137802	MAPKBP1	400.7757592	290.7659378	333.0213868	308.1019843	235.7809454	301.2291121	581.1734903	515.0069172	576.8237907	677.7047586	620.821972	531.1782361
ENSG00000137804	NUSAP1	9.843615139	7.006408141	9.770798633	20.09360767	10.40210053	7.208253313	23.4920743	23.25837691	23.917084	35.43554293	23.4532745	18.60815477
ENSG00000137806	NDUFAF1	144.8417656	52.54806106	172.6174425	132.2829172	100.5536385	158.5815729	110.3106098	136.2276362	126.1508941	107.5721839	110.3683506	133.6403843
ENSG00000137807	KIF23	1659.352266	620.0671205	1405.366537	2186.017068	1620.416105	1970.129445	1192.988817	1149.628344	1356.708314	1253.532331	1018.148034	1524.177041
ENSG00000137812	KNL1	2481.997246	1944.278259	2103.978639	3027.436889	2854.798702	2892.785869	3332.810367	2972.088306	3213.799543	3199.323305	3348.299836	3741.930759
ENSG00000137814	HAUS2	528.742756	571.0222635	524.3661933	576.0167532	517.7934488	562.2437584	360.5522708	390.4084695	437.0729861	395.4859702	376.6319964	417.8376571
ENSG00000137815	RTF1	801.5515185	711.1504263	825.6324845	837.2336529	732.7701932	797.0810637	911.0839251	872.189134	859.1391744	894.7474591	975.3802983	923.6411367
ENSG00000137817	PARP6	430.3066047	434.3973047	393.274645	398.5232188	340.9577397	304.2641662	291.0974424	274.116585	232.1364036	252.4782434	277.3004808	184.3898973
ENSG00000137818	RPLP1	3893.852903	3702.886702	3836.66693	4214.634209	3921.591901	4198.617864	2690.353205	3080.073627	2848.008846	2688.67182	2891.650785	2847.04768
ENSG00000137819	PAQR5	246.0903785	185.6698157	99.33645277	267.0775353	197.6399102	149.4764108	0	0	3.28273702	0	1.379604382	5.920776517
ENSG00000137821	LRRC49	106.8735358	143.6313669	121.3207497	124.7478143	120.2020506	99.01863761	82.73295732	74.75906863	64.24785311	65.80886545	78.63744979	58.36193996
ENSG00000137822	TUBGCP4	1032.173359	966.8843234	1035.704655	1353.806817	1192.774195	1231.093789	692.5054946	817.365817	771.4431997	762.4969506	758.7824103	795.0757038
ENSG00000137824	RMDN3	441.5564505	346.817203	482.0260659	338.2423958	343.2693176	320.1981998	329.9104348	144.5341993	314.2048291	217.675478	213.8386793	223.2978572
ENSG00000137825	ITPKA	0	0	3.256932878	0	3.467366845	0.379381753	0	0	0	0	5.518417529	1.691650434
ENSG00000137831	UACA	3124.644691	4501.61723	3002.07788	3113.671955	5099.34084	2918.204446	1371.73286	2747.8111	1022.338101	1261.125662	2982.704675	1029.369289
ENSG00000137834	SMAD6	0	3.50320407	4.885399317	4.186168264	17.33683422	6.828871559	117.4603715	132.9050109	137.8749548	145.538837	168.3117346	148.8652382
ENSG00000137841	PLCB2	7.031153671	0	13.02773151	13.39573845	13.86946738	9.484543833	1.021394535	4.983937908	10.31717349	7.593330629	9.657230676	5.920776517
ENSG00000137842	TMEM62	572.3359088	690.1312019	609.8606814	390.1508822	454.2250567	468.5364653	399.3652631	397.05372	401.4318413	360.0504273	440.093798	390.7712502
ENSG00000137843	PAK6	310.7769922	273.2499175	252.412298	360.8477044	395.2798203	360.7920474	595.4730138	345.5530283	522.4241486	594.1781217	527.008874	510.8784309
ENSG00000137845	ADAM10	2010.90995	2010.839136	2005.456419	2263.042564	2350.874721	2416.282387	1710.835846	1952.042347	1907.739171	1529.423344	1685.876555	2127.25042
ENSG00000137857	DUOX1	25.31215321	28.02563256	18.72736405	31.81487881	62.41260321	25.03919572	4.08557814	8.306563181	10.78613592	17.71777147	13.79604382	31.29553302
ENSG00000137868	STRA6	1.406230734	3.50320407	0	7.535102876	3.467366845	14.79588838	11.23533988	4.983937908	7.034436472	16.45221636	2.759208765	2.53747565
ENSG00000137871	ZNF280D	260.1526858	234.7146727	280.9104607	282.9849747	238.0925233	261.394028	222.6640086	134.5663235	232.605366	196.1610412	158.654504	210.610479
ENSG00000137872	SEMA6D	0	0	0	0	0	0.758763507	324.8034621	428.6186601	188.9918599	335.3721028	397.3260621	205.5355277
ENSG00000137876	RSL24D1	1864.661953	1401.281628	1745.716022	1630.093922	1445.891974	1730.739559	1155.197219	1249.307102	1258.695166	1133.937374	1019.527639	1162.163848
ENSG00000137877	SPTBN5	1.406230734	0	5.699632536	0.837233653	10.40210053	6.449489806	0	0	3.28273702	0	0	0
ENSG00000137878	GCOM1	28.12461468	87.58010176	37.45472809	51.07125282	34.67366845	67.15057033	5.106972674	13.29050109	17.8205724	0.632777552	22.07367012	18.60815477
ENSG00000137880	GCHFR	0	7.006408141	4.885399317	8.372336529	12.71367843	14.03712487	0	8.306563181	3.28273702	5.062220419	4.138813147	1.691650434
ENSG00000137936	BCAR3	5692.422012	5577.10088	5238.776534	4516.038324	4720.242065	4573.067654	3063.16221	4553.657936	3050.600617	2680.445712	4674.099647	2721.865548
ENSG00000137941	TTLL7	154.6853808	266.2435094	148.1904459	159.074394	276.2335586	228.3878155	510.6972674	604.7177995	305.7635053	487.8714929	663.5897079	352.7091154
ENSG00000137942	FNBP1L	1020.923513	1292.682302	935.5539691	1304.410031	1779.91498	1335.803153	2690.353205	4312.767603	2644.479151	2783.588453	4410.59521	3296.18087
ENSG00000137944	KYAT3	468.2748345	500.9581821	475.5122002	356.6615361	403.3703429	305.4023114	543.3818926	584.7820479	538.8378337	470.786499	522.8700609	459.2830927
ENSG00000137947	GTF2B	555.46114	658.6023652	780.8496574	513.2242292	490.0545141	562.6231402	467.798697	485.1032897	437.0729861	454.9670602	435.9549848	441.5207632
ENSG00000137948	BRDT	15.46853808	0	7.328098975	10.04680383	3.467366845	3.41443578	0	0	0.468962431	0.632777552	0	1.691650434
ENSG00000137955	RABGGTB	2818.086391	1793.640484	2254.611785	3294.514424	1964.841212	2587.383558	1914.093358	1324.066171	1862.718778	2005.272063	1331.318229	2150.087701
ENSG00000137959	IFI44L	471.0872959	1811.156504	429.9151399	4.186168264	19.64841212	9.484543833	228.7923758	320.6333388	81.13050064	3.163887762	12.41643944	0
ENSG00000137960	GIPC2	19.68723028	24.52242849	15.47043117	32.65211246	53.16629162	22.38352344	62.30506663	81.40431917	53.46171719	64.54331034	80.01705417	54.13281387
ENSG00000137962	ARHGAP29	2913.710081	1881.220586	1959.045126	2495.793519	1822.679171	1872.628334	3402.265196	3329.270523	3829.547215	3330.308258	3357.957067	4010.057353
ENSG00000137965	IFI44	1049.048128	2501.287706	1014.534591	164.097796	371.0082524	139.6124852	324.8034621	355.5209041	127.0888189	69.60553076	86.91507609	28.75805737
ENSG00000137968	SLC44A5	269.996301	311.7851623	222.2856689	319.8232554	316.6861718	291.7445683	1152.133035	604.7177995	898.5320187	1082.682392	716.0146744	870.3541481
ENSG00000137970	RPL7P9	40.78069129	17.51602035	35.82626166	66.97869223	79.74943743	47.42271916	24.51346884	31.56494009	33.29633263	21.51443678	44.14734023	17.76232955
ENSG00000137975	CLCA2	52.03053716	17.51602035	47.22552673	29.30317785	20.80420107	26.55672273	643.478557	654.5571786	373.7630579	832.7352589	851.2159039	412.7627058
ENSG00000137992	DBT	808.5826721	802.2337321	796.3200886	868.211298	987.0437618	819.0852054	1029.565691	1013.400708	951.5247734	899.1769019	1066.434188	1037.827541
ENSG00000137996	RTCA	1027.954667	893.317038	990.9218281	907.5612797	922.3195807	994.7389572	733.361276	784.1395643	782.6982981	784.644165	811.2073768	884.7331767
ENSG00000138002	IFT172	293.9022234	255.7338971	279.2819943	408.5700226	446.134534	363.8271014	836.5221241	624.6535512	645.7612681	794.1358282	720.1534876	664.8186204
ENSG00000138018	SELENOI	2981.209156	2045.871177	2639.744097	2690.86896	2428.31258	2783.903306	2028.489546	1470.261683	2069.53121	1759.754373	1498.250359	2008.83489
ENSG00000138028	CGREF1	39.37446056	31.52883663	13.84196473	18.41914036	32.36209055	12.14021611	0	6.645250545	0.468962431	2.53111021	0	0.845825217
ENSG00000138029	HADHB	1172.796432	1177.076568	1002.321093	1098.450553	1162.723682	1132.454534	1447.316056	1468.60037	1475.824772	1495.886134	1415.474096	1506.414711
ENSG00000138030	KHK	23.90592248	35.0320407	17.91313083	15.07020575	38.14103529	32.62683078	43.919965	43.19412854	31.42048291	36.70109804	42.76773585	21.14563042
ENSG00000138031	ADCY3	964.6742836	742.6792629	815.0474527	1046.542066	1054.079521	1031.538987	757.8747449	669.5089924	883.5252209	922.5896714	711.8758613	878.8124002
ENSG00000138032	PPM1B	241.8716863	206.6890402	203.5583049	265.403068	209.1977996	260.6352645	298.2472042	264.1487091	294.9773694	272.0943475	282.8188984	364.5506684

ENSG00000138035	PNPT1	1098.266203	991.4067519	1050.360853	1208.965395	783.6249069	1077.444179	875.3351164	671.170305	781.7603732	774.5197241	608.4055326	899.1122054
ENSG00000138036	DYNC2LI1	165.9352266	213.6954483	156.3327781	180.0052354	180.3030759	152.5114648	273.7337353	234.2450817	209.6262069	277.1565679	226.2551187	283.3514476
ENSG00000138050	THUMPD2	236.2467633	269.7467134	289.8670261	257.8679651	193.0167544	240.5280316	281.9048916	274.116585	217.5985682	275.8910128	246.9491844	241.0601868
ENSG00000138061	CYP1B1	475.3059881	245.2242849	408.7450762	330.7072929	251.9619907	268.9816631	371.7876107	363.8274673	374.7009827	287.9137863	162.7933171	251.2100894
ENSG00000138069	RAB1A	2084.033948	2150.967299	2047.796547	1908.055495	1972.931735	1998.962458	1411.567247	1785.911084	1728.595522	1600.29443	1607.239105	1788.074508
ENSG00000138071	ACTR2	6527.723068	6806.725509	6383.58844	6524.561857	6430.809708	6521.951721	6031.334728	6452.538279	6350.220284	5674.74909	6324.106489	6550.916304
ENSG00000138073	PREB	715.7714437	735.6728548	784.9208235	854.8155596	746.6396606	880.9244312	577.0879122	578.1367974	616.6855974	551.7820257	481.4819294	592.0776517
ENSG00000138074	SLC5A6	1719.820188	1100.006078	1618.69564	2165.92346	1756.799201	2384.41432	1678.151221	1098.127652	1932.125218	1888.840994	1191.978186	1830.365769
ENSG00000138078	PREPL	1182.640047	1348.733567	1150.511539	1512.043977	1537.199301	1395.74547	1516.770884	1456.971182	1599.630854	1638.261083	1501.009568	1845.590623
ENSG00000138080	EMILIN1	0	0	0.814233219	9.209570182	1.155788948	4.173199286	0	0	0.937924863	4.429442867	1.379604382	1.691650434
ENSG00000138081	FBXO11	1681.851958	2189.502544	1839.352843	1631.768389	1552.224558	1715.184907	1240.99436	1320.743546	1195.8542	1244.040668	1236.125527	1335.558017
ENSG00000138085	ATRAID	711.5527515	774.2080996	822.3755516	838.9081202	910.7616912	859.2996712	1068.378683	1144.644406	1092.213503	1016.873527	1025.046056	1015.836085
ENSG00000138092	CENPO	1061.704204	924.8458746	1156.211172	1273.432386	1108.401601	1476.933166	982.5815426	965.2226416	1185.537027	1057.37129	1060.91577	1261.971223
ENSG00000138095	LRPPRC	3425.578068	2602.880624	2726.052819	3504.660071	3028.167044	3354.114081	3703.576583	2766.085539	3665.410364	3720.732008	3203.441376	4148.772688
ENSG00000138101	DTNB	305.1520693	262.7403053	283.3531604	288.8456102	292.4146039	305.7816932	543.3818926	971.8678921	507.8863133	567.6014645	891.224431	493.9619266
ENSG00000138107	ACTR1A	1537.010192	1611.473872	1731.059825	1749.818334	1768.357091	1943.572722	1165.411164	1347.324548	1298.556973	1233.916227	1365.808338	1403.224035
ENSG00000138109	CYP2C9	1.406230734	0	8.142332194	0	0	0.758763507	0	0	0	0	0	0
ENSG00000138111	MFSD13A	137.8106119	94.5865099	114.8068839	191.7265065	131.7599401	179.8269511	87.83993	81.40431917	115.8337206	132.883286	132.4420207	126.8737825
ENSG00000138115	CYP2C8	0	3.50320407	1.628466439	5.023401917	1.155788948	1.517527013	3.064183605	0	0	0.632777552	5.518417529	0.845825217
ENSG00000138119	MYOF	21080.80494	16846.90837	19221.60361	20220.02995	20582.28959	19856.0822	18591.42332	23324.82941	18580.7605	17334.30827	21298.33245	17511.11946
ENSG00000138131	LOXL4	11.24984587	35.0320407	13.84196473	9.209570182	33.5178795	13.65774312	106.2250316	169.4538889	79.25465092	112.0016268	179.3485697	87.11999733
ENSG00000138134	STAMBPL1	999.830052	795.227324	957.5382661	740.1145491	557.0902731	796.3223002	368.7234271	328.939902	549.1550072	319.552664	288.3373159	421.220958
ENSG00000138135	CH25H	0	3.50320407	0	0	0	0	5.106972674	6.645250545	2.813774589	2.53111021	1.379604382	0
ENSG00000138138	ATAD1	1565.134807	1485.358526	1560.070848	1296.037695	1277.146788	1402.19496	1355.390548	1264.258916	1272.764039	1146.592925	1070.573001	1235.750642
ENSG00000138152	BTBD16	95.62368992	24.52242849	35.82626166	77.86272972	65.87997005	77.39387767	8.171156279	13.29050109	6.096511609	3.163887762	0	7.612426951
ENSG00000138160	KIF11	3130.269614	2052.877585	2622.6452	3760.016335	3450.030011	3179.219093	3191.857921	2920.587614	3257.413049	3406.874342	2814.39294	4138.622786
ENSG00000138161	CUZD1	4.218692202	7.006408141	7.328098975	9.209570182	5.778944741	4.173199286	11.23533988	13.29050109	5.627549177	3.796665314	9.657230676	10.1499026
ENSG00000138162	TACC2	724.2088281	493.9517739	783.2923571	745.1379511	694.6291579	736.3799832	247.1774774	186.0670152	392.0525927	168.9516065	168.3117346	266.4349433
ENSG00000138166	DUSP5	1542.635115	2392.68838	1672.435033	1069.147375	1019.405852	1252.718549	693.5268892	1493.52006	992.7934674	559.3753563	1316.142581	804.3797812
ENSG00000138172	CALHM2	192.6536106	175.1602035	174.245909	297.2179468	337.4903729	253.0476295	328.8890402	275.7778976	355.0045606	312.5921109	393.187249	331.563485
ENSG00000138175	ARL 3.00	120.9358431	248.727489	118.0638168	203.4477776	211.5093775	146.8207385	229.8137703	210.9867048	247.1432014	206.9182596	257.9860195	279.9681468
ENSG00000138180	CEP55	1387.949735	910.8330583	1459.105929	1824.33213	1754.487623	1723.531305	1197.074395	1147.967032	1328.570568	1299.092315	1280.272867	1682.346356
ENSG00000138182	KIF20B	2152.939254	1142.044527	2116.192137	3235.908068	2680.274571	2604.455736	2168.420598	1513.455812	1879.132463	2291.920295	1982.491497	2566.233708
ENSG00000138185	ENTPD1	9.843615139	17.51602035	13.02773151	6.697869223	6.93473369	3.793817533	18.38510163	53.16200436	25.79293373	19.61610412	34.49010956	15.2248539
ENSG00000138190	EXOC6	277.0274546	269.7467134	267.068496	181.6797027	172.2125533	207.9012008	219.599825	201.018829	231.6674411	231.5965842	177.9689653	222.452032
ENSG00000138193	PLCE1	0	0	1.628466439	1.674467306	9.246311586	2.655672273	12.25673442	16.61312636	32.35840777	18.35054902	12.41643944	54.13281387
ENSG00000138231	DBR1	385.3072212	290.7659378	345.234885	352.4753679	291.258815	338.7879057	367.7020326	300.6975871	367.1975838	389.7909723	288.3373159	362.859018
ENSG00000138246	DNAJC13	3149.956845	3615.306601	3234.134348	3122.881525	3081.333336	3133.313901	2772.064768	3108.315942	2640.727452	2674.750714	3098.591443	2953.621657
ENSG00000138271	GPR87	580.7732932	507.9645902	502.3818964	509.8752946	373.3198303	455.6374857	256.3700283	255.842146	238.2329152	310.0610007	219.3570968	277.4306711
ENSG00000138279	ANXA7	2564.964859	2035.361565	2580.305072	2294.857443	2312.733685	2401.86588	1989.676554	2028.462729	1907.739171	2009.068729	1819.69818	1952.1646
ENSG00000138286	FAM149B1	333.276684	371.3396315	359.0768498	261.2168997	332.8672171	259.4971193	453.4991735	470.151476	419.7213762	427.1248479	324.2070298	314.6469806
ENSG00000138303	ASCC1	828.2699024	1275.166282	754.7941944	781.9762318	1138.452114	775.8356855	1118.427016	1508.471874	974.0349701	940.3074428	1448.584601	836.5211394
ENSG00000138311	ZNF365	811.3951336	942.3618949	1045.475454	853.9783259	774.3785953	1044.058585	1.021394535	1.661312636	5.627549177	3.163887762	4.138813147	5.074951301
ENSG00000138316	ADAMTS14	7.031153671	7.006408141	8.142332194	10.04680383	21.95999002	10.24330734	0	0	2.344812157	0	0	0
ENSG00000138326	RPS24	11498.74871	10618.21154	10745.4358	12365.94105	12031.76295	11518.03003	9908.548383	11894.99847	10051.27179	10231.38024	10686.41555	10919.60355
ENSG00000138336	TET1	8.437384405	21.01922442	8.956565414	43.53614995	28.89472371	21.24537819	7.149761744	18.274439	18.28953483	21.51443678	40.00852709	25.3747565
ENSG00000138346	DNA2	1077.172742	907.3298542	927.411637	1119.381394	985.8879729	908.2399174	1025.480113	1104.772903	989.9796928	1144.061815	936.7513756	1053.052395
ENSG00000138347	MYPN	4595.562039	2483.771686	3339.170433	4099.933198	3022.3881	3395.08731	754.8105613	323.955964	443.1694977	658.0886545	296.6149422	422.0667832
ENSG00000138356	AOX1	66.09284451	98.08971397	43.96859385	92.09570182	144.4736185	92.56914781	8.171156279	19.93575163	8.910286198	9.491663286	11.03683506	10.1499026
ENSG00000138363	ATIC	1548.260038	1075.48365	1432.236233	1527.951416	1442.424607	1486.038328	1524.942041	1262.597603	1564.458671	1431.975601	1252.680779	1629.905193
ENSG00000138375	SMARCAL1	430.3066047	385.3524477	422.5870409	409.4072563	368.6966745	405.1797125	298.2472042	390.4084695	365.3217341	298.6710047	343.5214912	334.9467858
ENSG00000138376	BARD1	1051.860589	1191.089384	1057.688952	1064.961206	1019.405852	1089.205014	1067.357289	1179.531972	1185.068064	1024.466857	1076.091418	1118.180937
ENSG00000138378	STAT4	15.46853808	10.50961221	12.21349829	13.39573845	13.86946738	15.93403364	141.9738403	215.9706427	117.2406079	137.9455064	92.43349361	136.1778599
ENSG00000138380	CARF	50.62430643	21.01922442	43.96859385	31.81487881	32.36209055	25.79795922	99.07526988	54.82331699	58.62030393	91.7527451	74.49863664	62.59106604
ENSG00000138381	ASNSD1	134.9981505	213.6954483	170.1747429	154.0509921	159.4988749	177.9300423	139.9310513	152.8407625	133.1853305	163.8893861	113.1275593	159.0151408
ENSG00000138382	METTL5	451.4000657	283.7595297	372.9188145	388.4764149	329.3998503	423.0106549	335.0174074	338.9077778	374.7009827	342.3326558	284.1985028	383.1588232
ENSG00000138385	SSB	1439.980272	1436.313669	1473.762127	1733.073661	1610.014005	1500.075453	1288.999903	1272.565479	1377.811624	1406.664499	1263.717614	1585.922281
ENSG00000138386	NAB1	2294.968558	2427.720421	2261.12565	1832.704466	1917.453865	1864.661317	1208.309735	1353.969798	1294.336311	1092.174055	1281.652471	1432.827917
ENSG00000138395	CDK15	8.437384405	7.006408141	21.17006371	35.16381342	45.07576898	39.45570234	0	1.661312636	2.344812157	0.632777552	12.41643944	2.53747565
ENSG00000138398	PPIG	1435.76158	1264.656669	1296.259285	1429.995079	1183.527883	1160.908165	1579.075951	1621.441133	1368.901337	1564.226109	1600.341083	1666.275677
ENSG00000138399	FASTKD1	281.2461468	245.2242849	264.6257963	346.6147323	320.1535387	358.8951386	315.6109113	320.6333388	332.4943639	336.6376579	354.5583263	293.5013502
ENSG00000138400	MDH1B	9.843615139	14.01281628	10.58503185	4.186168264	16.18104528	2.655672273	19.40649616	4.983937908	4.689624315	20.24888168	4.138813147	5.920776517
ENSG00000138411	HECW2	552.6486785	234.7146727	512.9669283	921.7942518	769.7554395	806.9449893	360.5522708	408.6829085	355.473523	370.1748681	373.8727876	438.1374623
ENSG00000138413	IDH1	324.8392996	462.4229373	476.3264334	228.5647872	351.3598403	374.0704088	437.1568609	596.4112364	409.8731651	425.2265152	493.8983689	446.5957145
ENSG00000138430	OLA1	1191.077432	1285.675894	1289.74542	1388.133396	1420.464617	1478.830074	1372.754255	1231.032663	1174.281928	1247.837333	1142.312429	1313.566562
ENSG00000138433	CIR1	489.3682955	707.6472222	523.5519601	305.5902833	343.2693176	295.1590041	541.3391035	622.9922385	543.0584956	420.7970723	520.1108521	426.2959093
ENSG00000138434	SSFA2	9424.55838	8729.984543	10039.4956	9432.274333	8506.606659	10683.76955	2683.203443	2463.726639	2903.815376	2564.64742	2295.661692	2973.921462

**ENSG00000138439**	FAM117B	123.7483046	245.2242849	132.7200148	108.0031412	177.991498	133.1629954	228.7923758	305.681525	189.4608223	204.3871494	300.7537553	188.6190233
**ENSG00000138442**	WDR12	499.2119106	332.8043867	435.6147724	570.9933513	472.7176798	480.6766814	431.0284937	247.5355828	405.1835408	485.3403827	344.9010956	467.7413449
**ENSG00000138443**	ABI2	1047.641897	756.6920792	670.1139396	1083.380347	976.6416613	800.4954995	1214.438102	1156.273595	1194.916275	1096.603498	1256.819592	1325.408115
**ENSG00000138448**	ITGAV	1818.256339	2056.380789	2029.883416	2446.396734	2905.653416	2950.831277	1750.670233	1515.117124	1816.760459	1919.847094	1834.873828	2253.278377
**ENSG00000138449**	SLC40A1	0	0	0.814233219	0	0	0	14.29952349	9.967875817	12.19302322	31.63887762	1.379604382	25.3747565
**ENSG00000138459**	SLC35A5	393.7446056	413.3780803	499.1249635	424.477462	395.2798203	424.1488002	630.200428	629.6374891	567.4445421	603.0370074	452.5102374	564.1654196
**ENSG00000138463**	DIRC2	388.1196826	472.9325495	392.4604118	471.3625466	584.8292078	535.6870357	441.2424391	400.3763453	472.2451685	518.2448154	441.4734023	563.3195944
**ENSG00000138468**	SENP7	494.9932184	357.3268152	404.6739101	550.0625099	545.5323836	496.2313333	759.9175339	647.9119281	634.5061698	632.1447748	696.7002131	706.264056
**ENSG00000138495**	COX17	424.6816817	469.4293454	534.9512252	504.014659	490.0545141	520.1323838	397.3224741	435.2639107	440.3557231	391.0565274	420.7793366	463.5122188
**ENSG00000138496**	PARP9	6721.782909	7805.138669	6434.885133	1128.590964	1294.483622	1155.976202	4758.677138	5346.104063	4307.419933	960.5563245	980.8987158	782.3883255
**ENSG00000138587**	MNS1	291.089762	350.320407	211.7006371	413.5934245	340.9577397	218.1445082	236.9635321	194.3735784	192.2745969	246.1504679	238.6715581	257.9766911
**ENSG00000138592**	USP8	1529.979039	1527.396975	1540.529251	1544.69609	1553.380346	1531.564138	1371.73286	1324.066171	1344.046329	1251.633999	1392.020822	1374.465977
**ENSG00000138593**	SECISBP2L	693.2717519	816.2465484	926.5974037	761.8826241	864.5301333	902.9285729	868.1853546	875.5117592	839.4427523	756.1691751	780.8560804	870.3541481
**ENSG00000138594**	TMOD3	12514.0473	10712.79805	11613.40841	12991.35459	10996.17605	13062.11377	5706.531266	6507.361596	6172.483523	5258.38146	5775.023944	6091.633211
**ENSG00000138600**	SPPL2A	1902.630183	2052.877585	1976.958257	1804.238522	1779.91498	1961.403665	1908.986386	1819.137337	1729.533447	1688.25051	1619.655545	1887.036059
**ENSG00000138604**	GLCE	257.3402244	350.320407	253.2265312	330.7072929	371.0082524	325.8889261	59.24088302	86.38825708	62.84096581	69.60553076	73.11903226	94.73242428
**ENSG00000138606**	SHF	0	0	4.071166097	1.674467306	8.090522638	1.896908767	4.08557814	8.306563181	0	0	1.379604382	3.383300867
**ENSG00000138613**	APH1B	21.09346101	42.03844885	16.28466439	18.41914036	18.49262317	21.62475994	175.67986	227.5998311	127.5577814	192.3643759	166.9321303	164.9359173
**ENSG00000138614**	INTS14	392.3383748	290.7659378	376.1757474	329.8700592	360.6061519	396.4539322	256.3700283	220.9545806	277.6257594	313.2248884	288.3373159	296.8846511
**ENSG00000138617**	PARP16	89.99876699	98.08971397	68.39559043	102.1425056	132.9157291	105.0887457	67.4120393	122.9371351	90.50974927	96.81496551	143.4788558	112.4947538
**ENSG00000138621**	PPCDC	109.6859973	126.1153465	129.4630819	122.2361133	84.37259322	118.367107	80.69016826	64.79119281	80.19257578	103.7755186	85.5354717	72.74096864
**ENSG00000138623**	SEMA7A	258.7464551	521.9774065	488.5399317	328.1955919	227.6904228	454.1199587	42.89857046	159.4860131	57.68237907	33.53721028	111.747955	54.13281387
**ENSG00000138629**	UBL7	217.9657638	227.7082646	267.068496	252.0073295	278.5451365	287.1919873	146.0594185	164.469951	150.0679781	182.2399351	148.9972733	178.4691207
**ENSG00000138639**	ARHGAP24	2.812461468	21.01922442	1.628466439	2.511700959	8.090522638	4.931962793	9.192550814	9.967875817	18.28953483	20.24888168	41.38813147	18.60815477
**ENSG00000138640**	FAM13A	167.3414574	126.1153465	133.534248	342.428564	388.3450866	249.2538119	450.4349899	490.0872277	315.6117164	537.8609195	589.0910712	425.450084
**ENSG00000138641**	HERC3	89.99876699	108.5993262	74.90945619	85.39783259	101.7094274	81.18769521	66.39064477	117.9531972	51.58586746	52.52053685	137.9604382	60.89941561
**ENSG00000138642**	HERC6	876.0817474	1495.868138	904.6131068	246.1466939	396.4356093	262.5321733	332.9746184	408.6829085	311.3910545	57.58275727	82.77626294	33.83300867
**ENSG00000138646**	HERC5	627.1789074	1352.236771	639.9873105	303.915816	548.9997504	298.5734399	183.8510163	189.3896405	129.9025935	84.79219202	64.84140597	29.60388259
**ENSG00000138650**	PCDH10	2.812461468	0	1.628466439	2.511700959	0	2.27629052	28.59904698	8.306563181	18.28953483	11.38999594	15.17564821	17.76232955
**ENSG00000138653**	NDST4	0	0	1.628466439	1.674467306	0	1.896908767	1.021394535	0	0	0	0	0
**ENSG00000138658**	ZGRF1	587.8044469	907.3298542	587.0621512	625.4135387	580.206052	519.3736203	749.7035886	920.3672004	683.7472251	708.7108587	893.9836397	645.3646404
**ENSG00000138660**	AP1AR	980.1428217	770.7048955	831.3321171	801.2326058	915.384847	732.9655474	930.4904213	1026.691209	855.8564374	761.2313955	808.448168	928.716088
**ENSG00000138663**	COPS4	532.9614482	476.4357536	455.1563697	551.7369772	670.35759	526.2024918	374.8517943	387.0858442	423.4730756	391.0565274	431.8161717	468.5871701
**ENSG00000138668**	HNRNPD	6450.380378	5643.661757	5655.663942	6957.411655	6072.515134	5613.332422	5363.342703	4957.356906	4981.787909	5159.035385	5245.255862	5656.87905
**ENSG00000138669**	PRKG2	52.03053716	66.56087734	36.64049488	72.8393278	43.91998003	62.97737105	22.47067977	24.91968954	30.48255804	32.27165517	17.93485697	21.99145564
**ENSG00000138670**	RASGEF1B	15.46853808	7.006408141	13.02773151	1.674467306	1.155788948	3.41443578	30.64183605	23.25837691	18.28953483	20.88165923	26.21248326	18.60815477
**ENSG00000138674**	SEC31A	3761.667214	3993.65264	4002.770507	3187.348516	3263.94799	3392.431638	2328.77954	2581.679837	2265.088544	2166.630339	2381.197164	2285.419736
**ENSG00000138675**	FGF5	0	17.51602035	3.256932878	0	1.155788948	6.070108053	0	3.322625272	0	0	0	0
**ENSG00000138678**	GPAT3	1725.445111	1317.20473	1744.901789	2203.598974	1413.529884	2362.78956	45.96275407	58.14594226	66.12370283	72.76941852	68.98021912	85.42834689
**ENSG00000138686**	BBS7	298.1209156	273.2499175	247.5268987	372.5689755	321.3093276	305.4023114	242.0705048	184.4057026	185.2401604	240.4554699	215.2182836	301.1137772
**ENSG00000138688**	KIAA1109	2905.272697	2914.665787	3047.67494	2352.626565	2814.346089	2575.622723	2307.330254	2400.596759	2072.813947	2264.71086	2366.021516	2107.79644
**ENSG00000138696**	BMPR1B	4.218692202	0	0.814233219	7.535102876	1.155788948	3.793817533	56.17669942	139.5502614	52.99275475	68.33997566	153.1360864	56.67028952
**ENSG00000138698**	RAP1GDS1	500.6181414	374.8428355	517.0380943	680.6709598	598.6986752	711.7201692	303.3541769	299.0362745	256.52245	320.1854415	336.6234693	318.0302815
**ENSG00000138709**	LARP1B	409.2131436	381.8492437	502.3818964	487.269986	473.8734688	539.860235	273.7337353	295.7136492	258.3982997	277.1565679	280.0596896	323.951058
**ENSG00000138735**	PDE5A	30.93707615	87.58010176	89.56565414	77.02549606	136.3830959	148.3382655	110.3106098	187.7283279	110.2061714	126.5555105	160.0341083	154.7860147
**ENSG00000138738**	PRDM5	5.624922937	24.52242849	7.328098975	5.023401917	3.467366845	1.517527013	80.69016826	63.12988017	71.28228958	44.92720622	64.84140597	86.27417211
**ENSG00000138741**	TRPC3	0	0	0.814233219	0	0	0	19.40649616	24.91968954	22.97915914	21.51443678	33.11050518	38.90795997
**ENSG00000138744**	NAAA	28.12461468	38.53524477	37.45472809	59.44358935	53.16629162	71.70315137	66.39064477	61.46856754	91.44767413	94.2838553	88.29468047	87.11999733
**ENSG00000138750**	NUP54	911.2375157	1019.432384	894.028075	851.466625	739.7049269	794.4253914	729.2756979	647.9119281	673.899014	684.6653117	608.4055326	627.6023108
**ENSG00000138755**	CXCL9	77.34269038	315.2883663	254.8549977	0	0	0	18.38510163	78.0816939	23.917084	0.632777552	0	0
**ENSG00000138756**	BMP2K	534.367679	592.0414879	613.9318475	609.5060993	617.1912984	714.7552232	318.6750949	360.504842	360.6321098	307.5298905	467.6858856	389.0795997
**ENSG00000138757**	G3BP2	3095.113846	3503.20407	3046.046474	3350.609079	3511.286825	3190.600545	2224.597297	2510.243393	2225.6957	2188.144776	2483.287888	2543.396427
**ENSG00000138758**	11-Sep	2370.905018	2126.444871	1959.045126	2844.919952	2557.760943	2650.74031	2545.315181	2990.362745	2985.883801	2479.855228	2510.879976	3099.103594
**ENSG00000138759**	FRAS1	2.812461468	0	19.54159727	13.39573845	9.246311586	9.863925586	0	1.661312636	4.689624315	0.632777552	4.138813147	3.383300867
**ENSG00000138760**	SCARB2	2044.659487	2473.262074	2033.954582	1566.464165	2141.676921	1871.869571	2140.842945	2344.11213	1971.987024	1995.7804	2185.293342	2121.329644
**ENSG00000138764**	CCNG2	772.0206731	1040.451609	820.7470852	565.9699493	1193.929984	736.0006014	576.0665177	1157.934907	629.8165454	598.6075646	1303.726141	698.6516291
**ENSG00000138767**	CNOT6L	1129.20328	1275.166282	947.7674674	1152.033506	1438.957241	1169.254564	1227.716231	1252.629728	1381.563323	1251.001221	1267.856427	1428.598791
**ENSG00000138768**	USO1	3308.860917	3983.143028	3467.819282	2153.364955	2493.036761	2313.849313	1615.846154	1770.95927	1726.719673	1522.462791	1549.295721	1774.541305
**ENSG00000138769**	CDKL2	8.437384405	17.51602035	5.699632536	12.55850479	27.73893476	7.208253313	0	13.29050109	3.28273702	2.53111021	9.657230676	9.304077385
**ENSG00000138771**	SHROOM3	1943.410875	1986.316708	2357.20517	1977.545888	2064.239062	2509.98968	540.3177089	792.4461274	614.8097476	611.8958932	782.2356848	671.5852221
**ENSG00000138772**	ANXA3	2137.470716	2168.48332	2754.550981	2330.021256	2154.3906	3003.944723	674.120393	699.4126198	1065.013682	505.5892644	482.8615338	1028.523464
**ENSG00000138777**	PPA2	572.3359088	602.5511001	398.9742775	563.4582484	581.361841	541.7571437	542.360498	516.6682298	472.2451685	563.8047992	536.6661047	598.8442535
**ENSG00000138778**	CENPE	2497.465784	1324.211139	2502.952917	3781.78441	3001.583899	3033.915881	1979.462609	927.012451	1764.70563	2098.923141	1459.621436	2484.188662
**ENSG00000138780**	GSTCD	434.5252969	357.3268152	537.3939248	577.6912205	470.4061019	548.2066335	388.1299233	358.8435294	439.4177983	408.1415213	419.3997322	422.9126084
**ENSG00000138785**	INTS12	344.5265299	357.3268152	352.562984	390.9881159	316.6861718	294.4002406	193.0435671	137.8889488	202.5917704	191.7315984	166.9321303	256.2850407
**ENSG00000138792**	ENPEP	2.812461468	17.51602035	6.513865756	4.186168264	2.311577897	1.896908767	7.149761744	6.645250545	15.00679781	9.491663286	1.379604382	19.45397999
**ENSG00000138794**	CASP6	129.3732275	105.0961221	137.6054141	180.0052354	225.3788449	205.6249103	70.47622291	107.9853213	80.19257578	108.837739	103.4703287	86.27417211

ENSG00000138796	HADH	745.3022891	532.4870187	520.2950272	765.2315587	718.9007258	655.192288	885.5490617	706.0578704	775.6638616	851.7185855	866.3915521	956.6283202
ENSG00000138798	EGF	11.24984587	0	39.08319453	5.86063557	17.33683422	32.24744903	9.192550814	8.306563181	5.627549177	1.898332657	2.759208765	4.229126084
ENSG00000138801	PAPSS1	288.2773005	336.3075908	341.9779522	353.3126015	440.3555893	323.6126356	219.599825	237.567707	219.4744179	226.5343638	257.9860195	197.0772755
ENSG00000138802	SEC24B	952.018207	991.4067519	1183.895101	1136.126067	1087.5974	1306.590758	743.5752214	847.2694444	725.4848815	715.6714117	765.6804322	802.6881307
ENSG00000138814	PPP3CA	1493.41704	1050.961221	1292.188119	1896.334224	1878.157041	1968.9913	1484.086259	1442.019368	1655.437383	1719.25661	1604.479897	2009.680715
ENSG00000138821	SLC39A8	104.0610743	175.1602035	123.7634494	140.6552537	216.1325333	174.1362248	119.5031606	94.69482026	79.72361335	141.1093942	137.9604382	131.1029086
ENSG00000138823	MTTP	0	3.50320407	0	1.674467306	3.467366845	0	1.021394535	1.661312636	0.937924863	0.632777552	0	0
ENSG00000138829	FBN2	902.8001313	304.7787541	437.2432388	3763.36527	1481.721432	2663.63929	12469.18448	11160.69829	17098.37025	16928.69786	15738.52679	23895.4082
ENSG00000138834	MAPK8IP3	707.3340593	816.2465484	833.7748167	585.2263234	736.23756	698.8211896	889.6346399	862.2212581	826.7807667	782.1130547	1023.666452	617.4524082
ENSG00000138835	RGS3	150.4666886	101.592918	226.356835	160.7488614	176.8357091	297.4352946	182.8296217	79.74300653	148.1921283	155.6632779	96.57230676	153.9401895
ENSG00000138867	GUCD1	1154.515433	991.4067519	1297.887752	889.1421393	1003.224807	994.3595754	742.5538269	775.8330011	867.5804982	670.111428	691.1817955	720.6430847
ENSG00000138942	RNF185	618.741523	893.317038	761.3080602	684.857128	884.1785454	676.4376661	715.9975689	1398.82524	656.547404	651.1281014	1407.19647	583.6193996
ENSG00000138964	PARVG	7.031153671	17.51602035	8.956565414	0	0	0	0	4.983937908	0	0	0	0
ENSG00000139044	B4GALNT3	0	0	3.256932878	6.697869223	1.155788948	3.793817533	4.08557814	1.661312636	5.627549177	1.265555105	0	0
ENSG00000139055	ERP27	16.87476881	31.52883663	17.09889761	12.55850479	9.246311586	6.449489806	6.128367209	6.645250545	10.31717349	11.38999594	4.138813147	1.691650434
ENSG00000139083	ETV6	319.2143767	595.544692	417.7016416	136.4690854	172.2125533	182.1032416	395.279685	328.939902	330.1495517	187.9349331	252.467602	209.7646538
ENSG00000139112	GABARAPL1	535.7739097	795.227324	640.8015437	340.7540967	444.9787451	363.8271014	1266.529223	1212.758224	1265.729602	1013.709639	1016.76843	1031.906764
ENSG00000139116	KIF21A	33.74953762	14.01281628	20.35583049	52.74572013	25.42735686	28.4536315	279.8621026	222.6158932	265.4327362	299.3037823	233.1531406	333.2551354
ENSG00000139117	CPNE8	409.2131436	462.4229373	380.2469135	324.8466573	291.258815	317.1631458	584.2376739	799.091378	596.0512504	546.0870277	645.6548509	651.2854169
ENSG00000139131	YARS2	81.56138258	63.05767327	71.65252331	62.79252397	112.111528	94.08667482	130.7385005	154.5020752	149.5990156	154.3977228	168.3117346	148.8652382
ENSG00000139132	FGD4	57.6554601	133.1217547	61.06749146	113.0265431	143.3178296	107.3650362	82.73295732	139.5502614	72.22021444	147.4371697	206.9406573	112.4947538
ENSG00000139133	ALG10	99.84238213	80.57369362	92.82258702	90.42123451	112.111528	79.67016819	255.3486337	320.6333388	244.3294268	289.812119	222.1163056	257.9766911
ENSG00000139144	PIK3C2G	0	0	0.814233219	0	0	0	0	0	1.875849726	0.632777552	0	6.766601734
ENSG00000139146	SINHCAF	236.2467633	147.134571	166.1035768	185.0286373	186.0820207	179.0681876	451.4563844	382.1019063	390.176743	380.299309	322.8274255	447.4415397
ENSG00000139154	AEBP2	194.0598413	164.6505913	114.8068839	169.9584315	144.4736185	131.2660866	696.5910728	490.0872277	635.9130571	623.9186666	544.943731	659.7436691
ENSG00000139160	ETFBKMT	9.843615139	0	4.071166097	5.86063557	5.778944741	4.55258104	11.23533988	13.29050109	15.94472267	19.61610412	23.4532745	15.2248539
ENSG00000139163	ETNK1	338.9016069	308.2819582	309.4086234	243.634993	216.1325333	275.8105347	673.0989985	476.7967266	655.1405167	520.1431481	449.7510286	640.2896891
ENSG00000139168	ZCRB1	378.2760675	448.410121	393.274645	447.9200043	583.6734189	452.2230499	650.6283187	716.0257462	540.7136835	622.6531115	637.3772246	653.8228926
ENSG00000139173	TMEM117	29.53084542	42.03844885	76.53792263	59.44358935	98.2420606	72.08253313	99.07526988	84.72694444	90.50974927	117.6966247	84.15586732	100.6532008
ENSG00000139174	PRICKLE1	194.0598413	206.6890402	100.150686	71.16486049	24.27156791	48.18148267	1.021394535	1.661312636	7.503398903	3.163887762	1.379604382	9.304077385
ENSG00000139178	C1RL	296.7146849	357.3268152	328.1359874	102.9797393	135.2273069	112.296999	359.5308763	549.8944826	385.4871187	299.3037823	335.2438649	222.452032
ENSG00000139180	NDUFA9	172.9663803	199.682632	159.589711	123.073347	72.81470374	114.5732895	212.4500633	204.3414542	192.7435593	192.9971535	190.3854048	179.314946
ENSG00000139182	CLSTN3	257.3402244	311.7851623	289.0527929	96.28187008	106.3325832	98.63925586	241.0491102	274.116585	206.8124323	132.883286	216.597888	124.3363069
ENSG00000139187	KLRG1	0	0	0	0	1.155788948	3.035054026	0	0	2.813774589	0.632777552	0	0.845825217
ENSG00000139190	VAMP1	9.843615139	21.01922442	25.2412298	36.00104707	63.56839215	34.52373955	56.17669942	111.3079466	53.46171719	55.68442461	154.5156908	50.74951301
ENSG00000139192	TAPBPL	234.8405326	206.6890402	188.0878737	50.23401917	47.38734688	48.18148267	301.3113878	312.3267756	271.5292478	117.6966247	131.0624163	69.35766778
ENSG00000139197	PEX5	170.1539188	133.1217547	178.3170751	227.7275536	197.6399102	205.6249103	283.9476807	277.4392102	318.8944534	338.5359905	277.3004808	343.405038
ENSG00000139209	SLC38A4	116.7171509	122.6121425	79.79485551	45.21061725	26.58314581	26.17734098	90.9041136	179.4217647	88.63389954	62.01220013	75.87824103	43.13708606
ENSG00000139211	AMIGO2	167.3414574	206.6890402	149.0046792	142.329721	179.147287	127.0928874	390.1727123	651.2345534	477.4037552	361.94876	586.3318625	502.4201788
ENSG00000139218	SCAF11	2574.808474	2392.68838	2333.592407	2447.233967	2363.588399	2313.849313	4155.032968	4457.301803	3853.933262	3937.141931	4554.074066	4087.027447
ENSG00000139219	COL2A1	0	0	0	0.837233653	1.155788948	0	3.064183605	1.661312636	0.468962431	2.53111021	0	4.229126084
ENSG00000139233	LLPH	181.4037647	157.6441832	173.4316757	200.098843	218.4441112	198.416657	255.3486337	245.8742701	236.8260279	258.8060189	205.561053	236.8310607
ENSG00000139239	RPL14P1	29.53084542	45.54165292	31.75509556	30.1404115	36.98524634	25.79795922	22.47067977	26.58100218	28.60670832	34.80276538	28.97169203	30.4497078
ENSG00000139263	LRIG3	310.7769922	280.2563256	340.3494857	272.1009372	285.4798702	279.9837339	267.6053681	240.8903322	352.6597485	317.6543313	338.0030737	407.6877545
ENSG00000139266	9-Mar	64.68661377	129.6185506	65.95289078	40.18721534	80.90522638	65.63304332	55.15530488	126.2597603	53.46171719	89.22163489	122.78479	87.11999733
ENSG00000139269	INHBE	0	0	8.142332194	0	0	3.41443578	4.08557814	0	3.28273702	1.898332657	2.759208765	0.845825217
ENSG00000139278	GLIPR1	42.18692202	59.5544692	139.2338805	15.07020575	39.29682424	48.94024618	35.74880872	44.85544118	16.4136851	15.18666126	40.00852709	23.68310607
ENSG00000139287	TPH2	0	0	0.814233219	0	1.155788948	0.758763507	0	0	0.468962431	3.796665314	0	0
ENSG00000139289	PHLDA1	6035.542311	5079.645902	6234.583761	3457.774986	2471.076771	4288.151958	383.0229506	365.4887799	734.3951677	215.1443678	160.0341083	453.3623162
ENSG00000139291	TMEM19	251.7153014	238.2178768	245.0841991	346.6147323	394.1240314	346.3755408	311.5253331	279.1005229	307.639355	305.6315578	378.0116008	351.017465
ENSG00000139304	PTPRQ	2.812461468	3.50320407	3.256932878	0	0	4.173199286	1162.346981	1088.159777	846.9461512	1245.939001	1132.655198	1138.480742
ENSG00000139318	DUSP6	683.4281368	1064.974037	761.3080602	399.3604524	473.8734688	479.1591544	302.3327823	210.9867048	337.6529506	173.3810494	132.4420207	241.0601868
ENSG00000139323	POC1B	220.7782253	318.7915704	284.9816268	260.379666	250.8062018	273.5342441	407.5364194	294.0523366	360.6321098	350.558764	365.5951613	382.312998
ENSG00000139324	TMTC3	1004.048744	1159.560547	971.3802308	1405.715303	1390.414105	1341.49388	1338.026841	1470.261683	1401.728708	1509.174462	1451.34381	1712.796064
ENSG00000139329	LUM	8.437384405	14.01281628	4.071166097	2.511700959	3.467366845	2.655672273	701.6980455	598.072549	1046.724147	880.1935754	1081.609836	1595.226359
ENSG00000139343	SNRPF	808.5826721	714.6536304	624.5168793	782.8134654	709.6544142	632.8087645	689.441311	651.2345534	795.8292462	808.0569344	742.2271577	992.9988045
ENSG00000139344	AMDHD1	2.812461468	7.006408141	4.071166097	3.348934611	10.40210053	4.931962793	1.021394535	14.95181373	8.910286198	8.226108181	13.79604382	2.53747565
ENSG00000139350	NEDD1	1075.766512	1075.48365	1149.697306	1047.3793	997.4458624	1111.588537	1184.81766	833.9789433	1312.156883	1095.970721	888.4652222	1187.538604
ENSG00000139351	SYCP3	0	0	8.142332194	3.348934611	8.090522638	1.13814526	6.128367209	26.58100218	9.848211061	11.38999594	5.518417529	5.920776517
ENSG00000139352	ASCL1	1.406230734	0	0	0	1.155788948	0	0	0	2.813774589	0.632777552	0	1.691650434
ENSG00000139354	GAS2L3	877.4879781	514.9709984	677.4420386	1040.681431	1066.793199	1137.765878	893.720218	702.7352451	959.4971348	1113.055715	782.2356848	1276.350252
ENSG00000139364	TMEM132B	1.406230734	0	10.58503185	1.674467306	12.71367843	2.27629052	0	1.661312636	2.813774589	0	8.277626294	1.691650434
ENSG00000139370	SLC15A4	329.0579918	332.8043867	318.3651888	288.8456102	290.103026	306.9198384	385.0657396	440.2478486	424.4110005	465.7242786	513.2128302	480.4287231
ENSG00000139372	TDG	607.4916772	486.9453658	507.2672957	795.3719702	596.3870973	727.6542028	1136.812117	1025.029896	1113.316812	1323.137862	1058.156561	1301.725009
ENSG00000139405	RITA1	187.0286876	101.592918	175.0601422	202.610544	188.3935986	215.4888359	214.4928523	207.6640795	207.2813947	175.9121596	230.3939318	248.6726137
ENSG00000139410	SDSL	23.90592248	59.5544692	58.6247918	39.34998168	52.01050267	40.97322936	59.24088302	31.56494009	39.86180667	37.96665314	49.66575776	52.44116344
ENSG00000139428	MMAB	233.4343019	217.1986524	193.7875062	282.147741	293.5703929	259.4971193	465.7559079	632.9601144	456.3004458	554.3131359	680.1449605	538.7906631

**ENSG00000139433**	GLTP	1214.983354	900.3234461	1203.436698	1683.676876	1055.23531	1643.102374	1185.839055	1159.59622	1548.513949	1416.78894	1302.346537	1766.928878
**ENSG00000139436**	GIT2	1715.601496	1261.153465	1765.25762	1136.126067	906.1385354	1270.17011	1478.979287	1554.988627	1532.569226	1179.497358	1426.510931	1206.992584
**ENSG00000139437**	TCHP	464.0561423	476.4357536	529.2515926	585.2263234	516.6376599	573.625211	616.9222991	511.6842919	687.0299621	635.9414401	604.2667195	673.2768726
**ENSG00000139438**	FAM222A	9.843615139	3.50320407	11.39926507	15.07020575	16.18104528	18.96908767	1.021394535	4.983937908	10.31717349	18.35054902	17.93485697	27.06640694
**ENSG00000139496**	NUP58	3068.395462	3293.011826	2862.844	2793.011466	2757.712431	2877.989981	1783.354858	1722.781204	1739.381658	1621.176089	1735.542313	1938.631397
**ENSG00000139505**	MTMR6	1047.641897	872.2978135	1003.94956	1032.309094	1126.894225	1264.858766	471.8842751	408.6829085	471.776206	512.5498174	386.289227	622.5273595
**ENSG00000139508**	SLC46A3	1.406230734	0	10.58503185	0	0	1.13814526	1.021394535	0	5.627549177	1.265555105	1.379604382	4.229126084
**ENSG00000139514**	SLC7A1	5197.428793	3843.014865	5565.284055	4501.805351	3618.775197	5922.907933	2970.215307	2319.19244	3626.955445	2762.074016	2183.913737	2812.368846
**ENSG00000139515**	PDX1	0	0	0.814233219	0	1.155788948	3.035054026	0	0	0	0	2.759208765	1.691650434
**ENSG00000139517**	LNX2	610.3041386	637.5831408	687.2128372	576.8539868	701.5638916	699.5799531	474.9484587	431.9412854	569.3203918	401.1809682	482.8615338	514.2617318
**ENSG00000139531**	SUOX	98.43615139	77.07048955	93.63682024	122.2361133	106.3325832	130.5073231	265.5625791	166.1312636	241.9846146	232.2293617	238.6715581	213.9937798
**ENSG00000139540**	SLC39A5	0	0	2.442699658	0	0	1.517527013	0	0	3.751699452	3.163887762	1.379604382	2.53747565
**ENSG00000139546**	TARBP2	120.9358431	87.58010176	131.9057816	112.1893095	147.9409854	140.7506305	113.3747934	96.3561329	171.6402499	177.8104922	137.9604382	173.3941694
**ENSG00000139547**	RDH16	5.624922937	0	0.814233219	9.209570182	4.623155793	2.27629052	11.23533988	3.322625272	5.158586746	12.65555105	9.657230676	15.2248539
**ENSG00000139549**	DHH	0	0	1.628466439	1.674467306	8.090522638	4.55258104	2.04278907	0	1.406887294	0	1.379604382	2.53747565
**ENSG00000139567**	ACVRL1	91.40499772	63.05767327	118.87805	86.23506625	56.63365846	73.22067839	23.4920743	23.25837691	18.75849726	20.24888168	12.41643944	35.5246591
**ENSG00000139579**	NABP2	480.9309111	448.410121	473.8837337	531.6433696	498.1450367	523.1674378	494.3549549	435.2639107	558.0652934	482.1764949	466.3062812	582.7735744
**ENSG00000139597**	N4BP2L1	18.28099954	10.50961221	20.35583049	4.186168264	16.18104528	5.6907263	84.77574639	59.8072549	45.95831828	53.78609195	33.11050518	27.06640694
**ENSG00000139613**	SMARCC2	1777.475648	1979.3103	1906.119967	1968.336318	2085.043263	1862.764409	2335.929301	2172.996928	2224.757775	2220.416431	2310.83734	2217.753718
**ENSG00000139618**	BRCA2	2646.526242	3314.031051	3027.31911	2441.373332	2980.779698	2473.948413	2532.037052	2767.746852	2319.957148	2314.700287	2560.545734	2293.877988
**ENSG00000139620**	KANSL2	417.650528	371.3396315	468.1841012	490.6189206	420.7071772	478.4003909	580.1520958	553.2171078	599.3339874	553.0475808	652.5528728	652.9770674
**ENSG00000139624**	CERS5	600.4605235	542.9966309	604.975282	536.6667715	500.4566146	579.3159373	764.0031121	912.0606372	739.084792	754.2708424	702.2186306	752.7844429
**ENSG00000139625**	MAP3K12	37.96822982	56.05126513	29.3123959	48.55955187	58.94523636	46.66395566	72.51901198	64.79119281	50.6479426	51.8877593	67.60061473	54.13281387
**ENSG00000139626**	ITGB7	8.437384405	14.01281628	7.328098975	0.837233653	1.155788948	1.13814526	5.106972674	4.983937908	1.875849726	1.898332657	0	0
**ENSG00000139629**	GALNT6	1144.671818	966.8843234	1222.164062	1347.108947	1258.654165	1548.636317	2220.511719	1631.409009	2433.915019	2329.25417	1761.754796	2478.267885
**ENSG00000139631**	CSAD	112.4984587	119.1089384	114.8068839	135.6318518	100.5536385	103.1918369	257.3914228	214.3093301	206.8124323	229.6982515	155.8952952	159.860966
**ENSG00000139636**	LMBR1L	113.9046895	161.1473872	172.6174425	164.097796	166.4336086	176.4125153	133.8026841	142.8728867	162.2610013	158.8271656	173.8301522	124.3363069
**ENSG00000139641**	ESYT1	1032.173359	1135.038119	1003.135326	1331.201508	1297.950989	1332.768099	994.838277	1036.659085	1096.434165	1049.777959	1107.822319	1093.652005
**ENSG00000139644**	TMBIM6	7240.68205	7248.129222	7827.223939	6154.504582	6986.744192	7265.160576	7414.302929	8464.387881	8398.179222	7398.435142	8167.257943	7796.816848
**ENSG00000139645**	ANKRD52	1404.824503	1338.223955	1621.13834	1652.699231	1614.637161	2239.49049	1665.894486	1528.407625	1994.966183	1640.159416	1640.349611	1768.620528
**ENSG00000139648**	KRT71	0	0	0	0.837233653	0	0	0	0	1.875849726	7.593330629	0	0
**ENSG00000139651**	ZNF740	679.2094446	557.0094472	586.247918	537.5040051	557.0902731	564.8994307	839.5863077	860.5599455	827.7186915	743.513624	910.5388923	718.9514343
**ENSG00000139656**	SMIM2	1.406230734	0	0.814233219	0.837233653	0	3.035054026	6.128367209	13.29050109	3.751699452	4.429442867	0	5.920776517
**ENSG00000139668**	WDFY2	516.0866794	592.0414879	518.6665608	509.8752946	495.8334588	451.4642864	434.0926773	463.5062255	413.6248645	469.5209439	404.224084	400.0753275
**ENSG00000139675**	HNRNPA1L2	53.4367679	28.02563256	39.89742775	52.74572013	41.60840214	45.90519215	56.17669942	48.17806645	37.51699452	41.76331846	38.6289227	21.99145564
**ENSG00000139684**	ESD	400.7757592	315.2883663	363.9622491	442.0593687	457.6924235	437.4271616	422.8573374	538.2652941	409.8731651	448.0065071	448.3714243	493.9619266
**ENSG00000139687**	RB1	227.8093789	371.3396315	271.9538953	169.9584315	246.183046	209.0393461	273.7337353	307.3428377	265.4327362	277.7893455	309.0313816	224.9895077
**ENSG00000139697**	SBNO1	2158.564177	2301.605074	2195.17276	2778.778494	3009.674421	2926.930227	2560.636099	2624.873965	2531.928167	2884.832861	3111.007882	3448.429409
**ENSG00000139714**	MORN3	2.812461468	7.006408141	5.699632536	22.60530863	30.05051265	9.105162079	25.53486337	41.5328159	21.57227185	30.37332251	40.00852709	13.53320347
**ENSG00000139718**	SETD1B	1299.357198	851.2785891	1367.097575	941.0506258	736.23756	1164.322601	1525.963435	960.2387037	1792.374413	1376.923954	980.8987158	1517.410439
**ENSG00000139719**	VPS33A	296.7146849	301.2755501	328.1359874	329.0328256	375.6314082	372.9322635	541.3391035	586.4433606	535.0861343	473.9503867	517.3516434	489.7328005
**ENSG00000139722**	VPS37B	386.7134519	420.3844885	514.5953947	518.2476311	462.3155793	512.5447487	453.4991735	373.7953431	406.5904281	450.5376173	458.0286549	364.5506684
**ENSG00000139725**	RHOF	1666.38342	1537.906587	1803.526581	1656.048165	1315.287823	1921.189199	1457.530001	1297.485169	1738.443733	1409.195609	1080.230231	1571.543253
**ENSG00000139726**	DENR	2380.748633	2164.980116	2257.868718	2038.663945	1586.898226	1839.622122	2287.923758	2337.466879	2643.072264	2371.650266	2383.956373	2638.128851
**ENSG00000139734**	DIAPH3	2175.438946	2126.444871	2049.425013	2769.568924	2638.666169	2797.181667	1884.472917	2056.705044	1870.222177	1817.969908	1939.723762	2061.276053
**ENSG00000139737**	SLAIN1	5.624922937	7.006408141	10.58503185	10.04680383	8.090522638	7.587635066	13.27812895	9.967875817	19.22745969	16.45221636	8.277626294	27.06640694
**ENSG00000139746**	RBM26	4244.004356	3373.58552	4068.723398	4169.423591	3372.592151	3952.019724	3839.422057	3123.267756	3568.335141	3701.115904	3352.438649	3824.82163
**ENSG00000139793**	MBNL2	3048.708232	2935.685011	2750.479815	3167.254909	3867.269821	3204.63767	4970.105807	4656.659319	4756.21698	4839.482721	4620.295076	5114.705086
**ENSG00000139797**	RNF113B	12.65607661	17.51602035	15.47043117	7.535102876	12.71367843	16.31341539	15.32091802	3.322625272	15.00679781	6.960553076	8.277626294	10.1499026
**ENSG00000139800**	ZIC5	33.74953762	24.52242849	28.49816268	45.21061725	42.76419109	32.24744903	1.021394535	1.661312636	5.158586746	1.265555105	6.898021912	0
**ENSG00000139826**	ABHD13	584.9919854	585.0350798	553.6785892	629.599707	540.9092278	643.8108354	631.2218226	609.7017375	659.3611786	618.2236687	605.6463238	681.7351247
**ENSG00000139832**	RAB20	21.09346101	80.57369362	21.17006371	30.97764516	50.85471372	12.51959786	38.81299233	144.5341993	61.43407852	48.72387153	113.1275593	63.43689126
**ENSG00000139835**	GRTP1	275.6212239	304.7787541	244.2699658	397.6859851	367.5408855	258.358974	146.0594185	107.9853213	151.9438278	189.2004882	147.6176689	170.8566938
**ENSG00000139842**	CUL4A	4298.847354	3243.966969	4002.770507	3724.852522	3376.059518	3867.417593	2355.335797	2148.077239	2155.820297	2248.258644	2022.500024	2309.948667
**ENSG00000139874**	SSTR1	0	0	0	0	0	0	4.08557814	6.645250545	1.875849726	6.327775524	1.379604382	0
**ENSG00000139880**	CDH24	49.2180757	38.53524477	34.19779522	59.44358935	47.38734688	54.25159072	35.74880872	69.77513072	57.21341664	56.94997971	88.29468047	65.12854169
**ENSG00000139890**	REM2	8.437384405	0	0.814233219	2.511700959	9.246311586	1.896908767	2.04278907	3.322625272	7.503398903	3.796665314	5.518417529	0
**ENSG00000139899**	CBLN3	19.68723028	63.05767327	40.71166097	20.09360767	11.55788948	15.55465189	35.74880872	24.91968954	20.63434698	27.8422123	28.97169203	26.22058172
**ENSG00000139908**	TSSK4	0	10.50961221	1.628466439	2.511700959	1.155788948	2.27629052	12.25673442	23.25837691	13.59991051	4.429442867	23.4532745	1.691650434
**ENSG00000139914**	FITM1	0	7.006408141	2.442699658	3.348934611	1.155788948	6.449489806	5.106972674	1.661312636	6.096511609	1.898332657	1.379604382	0.845825217
**ENSG00000139915**	MDGA2	14.06230734	136.6249587	68.39559043	23.44254228	86.68417112	61.08046228	14.29952349	9.967875817	3.28273702	11.38999594	9.657230676	6.766601734
**ENSG00000139921**	TMX1	1184.046278	1247.140649	994.9929942	797.8836712	795.1827964	813.3944791	1065.3145	1267.581541	1077.675667	925.1207816	993.3151553	1073.3522
**ENSG00000139926**	FRMD6	1220.608277	1292.682302	1240.077193	1165.429245	1226.292074	1270.928874	610.7939319	1008.41677	886.8079579	684.0325341	974.0006939	904.1871567
**ENSG00000139971**	C14orf37	1.406230734	0	3.256932878	6.697869223	2.311577897	2.27629052	0	1.661312636	0.468962431	2.53111021	1.379604382	1.691650434
**ENSG00000139973**	SYT16	1676.227035	1117.522098	1287.30272	2322.486153	1823.83496	1810.030345	4216.31664	5130.13342	3587.562601	3696.686461	4973.473798	3638.740083
**ENSG00000139974**	SLC38A6	239.0592248	255.7338971	262.9973299	223.5413853	199.951488	218.1445082	332.9746184	274.116585	311.8600169	269.5632373	343.5214912	277.4306711
**ENSG00000139977**	NAA30	572.3359088	455.4165292	508.0815289	516.5731638	494.6776699	554.2767416	687.398522	843.9468192	765.3466881	738.4514036	771.1988497	756.1677438

**ENSG00000139985**	ADAM21	2.812461468	24.52242849	4.885399317	10.04680383	8.090522638	4.55258104	9.192550814	9.967875817	1.406887294	1.898332657	8.277626294	5.920776517
**ENSG00000139988**	RDH12	0	0	2.442699658	7.535102876	1.155788948	2.27629052	0	0	0.468962431	0	0	0
**ENSG00000139990**	DCAF5	444.368912	763.6984874	608.2322149	450.4317052	698.0965248	558.0705591	913.1267142	1179.531972	812.7118937	821.9780405	1063.674979	914.3370593
**ENSG00000139998**	RAB15	23.90592248	21.01922442	18.72736405	44.3733836	73.97049269	84.22274923	223.6854031	137.8889488	169.7644002	344.863766	284.1985028	285.0430981
**ENSG00000140006**	WDR89	202.4972257	220.7018564	185.645174	196.7499084	240.4041012	206.3836738	277.8193135	234.2450817	267.7775484	286.0154537	262.1248326	318.0302815
**ENSG00000140009**	ESR2	1.406230734	0	0	1.674467306	0	0.379381753	1.021394535	4.983937908	0.937924863	2.53111021	11.03683506	0.845825217
**ENSG00000140015**	KCNH5	134.9981505	45.54165292	146.5619795	117.2127114	137.5388848	149.097029	32.68462512	14.95181373	12.66198565	22.14721433	11.03683506	21.99145564
**ENSG00000140022**	STON2	469.6810652	269.7467134	294.7524254	645.5071464	547.8439615	546.6891065	503.5475057	1001.77152	627.4717333	483.44205	1165.765703	561.6279439
**ENSG00000140025**	EFCAB11	144.8417656	164.6505913	156.3327781	149.8648239	157.187297	127.4722691	318.6750949	237.567707	256.52245	268.9304598	318.6886123	323.1052328
**ENSG00000140030**	GPR65	1.406230734	0	10.58503185	0	0	0.379381753	0	1.661312636	1.875849726	0	0	0.845825217
**ENSG00000140043**	PTGR2	87.18630552	73.56728548	105.8503185	126.4222816	98.2420606	86.87842151	108.2678207	109.646634	125.2129692	139.2110615	150.3768777	132.794559
**ENSG00000140044**	JDP2	95.62368992	154.1409791	175.0601422	94.60740277	158.3430859	178.3094241	178.7440436	184.4057026	168.3575129	125.2899554	137.9604382	79.50757038
**ENSG00000140057**	AK7	7.031153671	10.50961221	4.885399317	13.39573845	18.49262317	3.035054026	44.94135953	58.14594226	30.01359561	53.78609195	60.70259282	44.82873649
**ENSG00000140090**	SLC24A4	0	0	3.256932878	2.511700959	0	0.758763507	2.04278907	11.62918845	0.937924863	3.163887762	0	2.53747565
**ENSG00000140092**	FBLN5	0	0	0.814233219	0	0	0	4.08557814	4.983937908	0.468962431	3.163887762	0	0
**ENSG00000140104**	CLBA1	98.43615139	31.52883663	67.58135721	95.44463643	80.90522638	72.08253313	134.8240786	121.2758224	101.2958852	130.9849533	104.8499331	114.1864043
**ENSG00000140105**	WARS	70310.13048	69591.14886	74270.28311	900.8634105	1033.27532	1308.867049	29848.21249	26984.70115	28260.61404	1194.684019	1252.680779	1148.630644
**ENSG00000140153**	WDR20	300.9333771	245.2242849	329.7644539	332.3817602	270.4546139	335.7528517	432.0498883	413.6668464	442.2315729	439.1476214	398.7056665	477.8912475
**ENSG00000140157**	NIPA2	1328.888044	1138.541323	1231.120628	1486.089734	1411.218306	1460.999132	994.838277	953.5934531	916.8215535	940.3074428	813.9665856	1091.960355
**ENSG00000140199**	SLC12A6	1472.323579	1373.255996	1485.161392	1357.155751	950.0585155	1545.601263	744.5966159	691.1060566	750.8088528	693.5241974	629.0995983	768.0092968
**ENSG00000140254**	DUOXA1	15.46853808	31.52883663	13.02773151	29.30317785	17.33683422	15.93403364	6.128367209	3.322625272	22.04123428	4.429442867	2.759208765	10.1499026
**ENSG00000140259**	MFAP1	901.3939006	1128.031711	1036.518888	856.4900269	918.8522139	857.7821442	640.4143734	697.7513072	595.1133255	594.8108992	765.6804322	686.810076
**ENSG00000140262**	TCF12	537.1801404	784.7177118	665.2285403	663.0890531	818.2985754	759.5222701	596.4944084	666.1863671	592.2995509	634.0431075	601.5075107	604.76503
**ENSG00000140263**	SORD	412.0256051	224.2050605	358.2626166	337.4051621	298.1935487	363.0683379	86.81853546	73.09775599	103.6406974	94.2838553	93.813098	96.42407471
**ENSG00000140264**	SERF2	1179.827586	1404.784832	1275.903455	1335.387676	1308.353089	1533.461047	838.5649131	996.7875817	1052.820659	1028.8963	943.6493975	1012.452784
**ENSG00000140265**	ZSCAN29	669.3658295	514.9709984	800.3912547	665.600754	590.6081526	720.8253313	535.2107363	490.0872277	553.3756691	487.8714929	473.2043031	530.3324109
**ENSG00000140279**	DUOX2	1.406230734	3.50320407	4.071166097	7.535102876	5.778944741	1.13814526	0	21.59706427	0	0	9.657230676	0.845825217
**ENSG00000140280**	LYSMD2	179.997534	70.06408141	100.150686	70.32762684	90.15153796	71.32376962	82.73295732	56.48462963	60.02719123	35.43554293	34.49010956	52.44116344
**ENSG00000140284**	SLC27A2	1.406230734	17.51602035	17.91313083	0	4.623155793	4.931962793	21.44928523	3.322625272	7.034436472	5.062220419	0	2.53747565
**ENSG00000140297**	GCNT3	149.0604578	175.1602035	118.0638168	195.9126748	196.4841212	132.4042319	31.66323058	78.0816939	39.86180667	36.70109804	48.28615338	36.37048432
**ENSG00000140299**	BNIP2	1261.388969	1439.816873	1294.630819	1367.202555	1537.199301	1360.083586	737.4468542	936.9803268	761.1260262	763.7625057	867.7711565	827.217062
**ENSG00000140307**	GTF2A2	531.5552175	507.9645902	574.8486529	759.3709232	617.1912984	730.6892569	273.7337353	284.0844608	296.8532191	277.7893455	282.8188984	304.497078
**ENSG00000140319**	SRP14	914.0499772	1054.464425	875.3007109	959.4697662	1116.492124	934.0378766	895.7630071	920.3672004	868.9873855	852.9841406	928.4737493	1015.836085
**ENSG00000140320**	BAHD1	295.3084542	402.8684681	293.123959	365.0338727	353.6714182	359.2745204	367.7020326	378.779281	415.5007143	292.9760068	398.7056665	447.4415397
**ENSG00000140323**	DISP2	181.4037647	199.682632	161.2181775	227.7275536	293.5703929	218.5238899	155.2519693	189.3896405	197.4331836	143.6405044	164.1729215	169.1650434
**ENSG00000140326**	CDAN1	201.090995	206.6890402	241.013033	284.659442	234.6251565	308.8167472	160.358942	202.6801416	209.1572444	195.5282637	249.7083932	180.1607712
**ENSG00000140332**	TLE3	209.5283794	280.2563256	256.4834641	338.2423958	303.9724934	358.1363751	615.9009045	418.6507843	671.0852394	739.0841812	571.1562143	755.3219186
**ENSG00000140350**	ANP32A	1673.414574	1530.900179	1363.840643	1929.82357	1865.443362	1509.939378	1101.063309	1131.353905	1001.234791	1097.869053	1313.383372	1213.759186
**ENSG00000140365**	COMMD4	410.6193744	465.9261414	419.330108	424.477462	386.0335087	446.9117054	219.599825	269.1326471	240.1087649	256.9076863	240.0511625	282.5056224
**ENSG00000140367**	UBE2Q2	333.276684	507.9645902	270.3254289	253.6817968	506.2355593	221.5589439	231.8565594	619.6696133	278.5636843	314.4904435	630.4792027	343.405038
**ENSG00000140368**	PSTPIP1	4.218692202	0	2.442699658	5.023401917	8.090522638	6.070108053	0	0	2.344812157	0	0	2.53747565
**ENSG00000140374**	ETFA	963.2680529	767.2016914	954.2813332	1085.892048	947.7469376	1191.258705	717.0189635	554.8784205	780.8224484	666.3147627	565.6377967	828.0628872
**ENSG00000140379**	BCL2A1	2.812461468	14.01281628	4.885399317	0	0	0.758763507	0	0	0	0	0	0
**ENSG00000140382**	HMG20A	427.4941432	371.3396315	432.3578395	437.0359668	453.0692677	372.9322635	352.3811145	328.939902	323.1151153	309.4282231	331.1050518	301.1137772
**ENSG00000140386**	SCAPER	277.0274546	322.2947745	290.6812593	286.3339093	315.5303829	309.1961289	363.6164544	368.8114052	291.2256699	321.4509966	362.8359525	328.1801841
**ENSG00000140391**	TSPAN3	521.7116024	486.9453658	478.769133	648.0188473	725.8354595	694.2686085	461.6703298	559.8623584	463.3348823	567.6014645	626.3403896	636.060563
**ENSG00000140395**	WDR61	282.6523776	315.2883663	382.6896131	334.0562275	322.4651166	344.0992502	230.8351649	245.8742701	208.688282	251.8454658	242.8103713	293.5013502
**ENSG00000140396**	NCOA2	1199.514816	1446.823281	1452.592063	1184.685619	1333.780446	1250.821641	1758.841389	1461.95512	1565.865559	1504.74502	1477.556293	1390.536656
**ENSG00000140398**	NEIL1	1.406230734	0	4.071166097	8.372336529	1.155788948	4.931962793	2.04278907	0	0.937924863	1.898332657	2.759208765	0.845825217
**ENSG00000140400**	MAN2C1	246.0903785	266.2435094	293.9381922	337.4051621	253.1177797	264.8084638	182.8296217	157.8247004	156.6334521	159.4599432	195.9038223	161.5526164
**ENSG00000140403**	DNAJA4	59.06169083	59.5544692	84.68025482	36.83828073	65.87997005	85.36089449	0	1.661312636	0.468962431	0.632777552	0	1.691650434
**ENSG00000140406**	TLNRD1	329.0579918	248.727489	324.8790546	282.9849747	302.8167044	325.1301626	153.2091802	157.8247004	166.0127007	151.233835	132.4420207	131.1029086
**ENSG00000140416**	TPM1	899.9876699	627.0735286	703.4975016	1556.417361	1039.054264	1175.324672	450.4349899	910.3993246	677.181751	603.669785	1121.618363	957.4741454
**ENSG00000140443**	IGF1R	1629.821421	2143.960891	1937.060829	2244.623423	3069.775447	2966.385929	2371.67811	2427.177761	2158.634072	2689.937375	2543.990481	2480.805361
**ENSG00000140450**	ARRDC4	105.4673051	122.6121425	160.4039442	171.6328988	223.067267	176.791897	245.1346884	194.3735784	158.9782643	230.3310291	212.4590749	206.3813529
**ENSG00000140451**	PIF1	140.6230734	3.50320407	83.8660216	169.1211979	83.21680428	116.0908165	55.15530488	28.24231481	59.55822879	80.9955267	48.28615338	72.74096864
**ENSG00000140455**	USP3	376.8698368	290.7659378	415.2589419	496.4795562	433.4208556	479.5385362	268.6267627	350.5369662	219.4744179	255.6421312	248.3287888	235.9852355
**ENSG00000140459**	CYP11A1	0	0	0.814233219	0	4.623155793	0	13.27812895	41.5328159	14.53783538	13.92110615	26.21248326	5.074951301
**ENSG00000140463**	BBS4	143.4355349	178.6634076	176.6886086	211.8201142	247.3388349	177.5506605	140.9524458	169.4538889	142.5645792	144.2732819	147.6176689	159.0151408
**ENSG00000140464**	PML	1375.293658	1951.284667	1941.131995	209.3084132	327.0882724	290.2270413	343.1885637	388.7471569	375.1699452	172.1154942	142.0992514	127.7196077
**ENSG00000140465**	CYP1A1	653.8972914	259.2371012	416.8874084	747.649652	580.206052	424.9075637	2557.571915	2540.147021	1657.782195	2551.991869	1954.89941	1449.744422
**ENSG00000140470**	ADAMTS17	19.68723028	0	10.58503185	3.348934611	1.155788948	14.41650663	1.021394535	0	0.468962431	0.632777552	0	1.691650434
**ENSG00000140471**	LINS1	260.1526858	371.3396315	270.3254289	162.4233287	156.031508	174.1362248	167.5087037	172.7765142	166.9506256	134.1488411	136.5808338	148.8652382
**ENSG00000140474**	ULK3	179.997534	182.1666117	153.8900785	170.7956652	226.5346339	196.89913	94.98969174	161.1473257	107.8613592	94.91663286	120.0255813	102.3448512
**ENSG00000140479**	PCSK6	472.4935267	651.5959571	902.9846404	476.3859485	618.3470873	759.9016519	321.7392785	438.5865359	251.3638633	284.7498986	366.9747657	220.7603816
**ENSG00000140488**	CELF6	0	3.50320407	4.071166097	0	0	0	0	1.661312636	1.875849726	0	4.138813147	0
**ENSG00000140497**	SCAMP2	632.8038304	627.0735286	588.6906177	568.4816503	655.3323337	726.8954393	561.7669942	546.5718573	573.5410537	530.9003664	485.6207426	597.152603

**ENSG00000140505**	CYP1A2	0	0	0	0	2.311577897	2.27629052	15.32091802	13.29050109	9.848211061	28.47498986	11.03683506	4.229126084
**ENSG00000140511**	HAPLN3	170.1539188	168.1537954	166.1035768	14.2329721	11.55788948	20.86599643	0	4.983937908	10.78613592	3.163887762	0	0.845825217
**ENSG00000140519**	RHCG	1.406230734	0	0	0	4.623155793	1.517527013	0	4.983937908	1.406887294	0.632777552	0	0
**ENSG00000140521**	POLG	445.7751427	497.454978	502.3818964	437.0359668	382.5661419	481.4354449	276.797919	318.9720261	279.5016091	252.4782434	310.410986	247.8267885
**ENSG00000140525**	FANCI	2931.991081	3552.248927	3033.832976	3434.332444	3199.223809	3079.441692	1785.397647	2237.788121	1778.774503	2017.294837	2192.191363	1983.460133
**ENSG00000140526**	ABHD2	1139.046895	1243.637445	1205.065165	1249.989844	1286.393099	1319.489738	627.1362444	556.5397331	619.4993719	572.6636849	561.4989836	626.7564856
**ENSG00000140527**	WDR93	0	0	1.628466439	4.186168264	5.778944741	2.27629052	3.064183605	4.983937908	0.468962431	3.163887762	5.518417529	5.074951301
**ENSG00000140534**	TICRR	766.3957501	469.4293454	703.4975016	818.8145125	740.8607158	828.1903675	580.1520958	546.5718573	568.3824669	547.9853604	575.2950274	617.4524082
**ENSG00000140543**	DET1	19.68723028	10.50961221	23.61276336	18.41914036	18.49262317	18.21032416	22.47067977	13.29050109	10.31717349	8.858885733	2.759208765	6.766601734
**ENSG00000140545**	MFGE8	535.7739097	476.4357536	436.4290056	593.5986599	501.6124035	542.1365255	24.51346884	46.51675381	56.27549177	31.63887762	33.11050518	57.51611474
**ENSG00000140548**	ZNF710	167.3414574	227.7082646	202.7440716	112.1893095	153.7199301	167.686735	70.47622291	83.06563181	95.66833602	55.68442461	37.24931832	68.51184256
**ENSG00000140553**	UNC45A	455.6187579	507.9645902	554.4928224	482.2465841	525.8839715	609.6664776	313.5681222	392.0697821	365.3217341	377.7681988	424.9181498	377.2380467
**ENSG00000140563**	MCTP2	1303.575891	781.2145077	973.8229305	1197.244124	869.1532891	931.3822044	1079.614023	734.3001852	1025.620838	765.6608384	579.4338406	852.5918185
**ENSG00000140564**	FURIN	1708.570342	1853.194953	2071.40931	1656.885399	1779.91498	2220.14202	359.5308763	413.6668464	586.2030393	409.4070764	331.1050518	556.5529926
**ENSG00000140575**	IQGAP1	5238.209485	6246.212858	5231.448435	4917.910477	5776.633163	4933.48032	4956.827678	5814.594226	5170.779769	5227.37536	5849.522581	6100.937289
**ENSG00000140577**	CRTC3	316.4019152	332.8043867	385.946546	318.1487881	291.258815	374.4497905	275.7765244	285.7457734	302.4807683	279.0549006	274.5412721	318.0302815
**ENSG00000140598**	EFL1	736.8649047	763.6984874	802.0197212	498.1540235	545.5323836	532.6519816	411.6219976	564.8462963	469.4313939	447.3737295	404.224084	419.5293075
**ENSG00000140600**	SH3GL3	104.0610743	178.6634076	55.36785892	219.3552171	173.3683422	133.1629954	1.021394535	1.661312636	1.875849726	0.632777552	4.138813147	0
**ENSG00000140612**	SEC11A	897.1752084	1036.948405	891.5853753	1061.612272	1108.401601	937.8316942	665.9492367	814.0431917	736.7399798	774.5197241	849.8362995	805.2256064
**ENSG00000140632**	GLYR1	864.8319015	753.1888751	923.3404709	966.1676354	858.7511886	943.5224205	877.3779055	877.1730719	819.7463302	825.1419283	896.7428485	883.8873515
**ENSG00000140650**	PMM2	1981.379104	1215.611812	1811.668913	1769.074709	1362.67517	1559.259006	1477.957892	1147.967032	1309.343109	1433.873934	1071.952605	1345.70792
**ENSG00000140675**	SLC5A2	5.624922937	7.006408141	5.699632536	0	9.246311586	0.379381753	0	0	0.468962431	3.796665314	0	0
**ENSG00000140682**	TGFB1I1	226.4031482	367.8364274	322.4363549	226.0530863	293.5703929	297.0559128	60.26227756	66.45250545	64.24785311	46.82553888	102.0907243	65.97436691
**ENSG00000140688**	C16orf58	1230.451892	1092.99967	1390.710339	1147.010104	1240.161541	1179.877253	1276.743169	1086.498464	1461.755899	1264.922327	1193.357791	1179.926177
**ENSG00000140691**	ARMC5	139.2168427	147.134571	129.4630819	123.9105806	159.4988749	179.8269511	138.9096567	139.5502614	150.5369405	177.8104922	147.6176689	143.7902869
**ENSG00000140694**	PARN	970.2992066	1124.528507	1047.10392	922.6314855	1100.311079	887.373921	1324.748712	1387.196051	1154.116544	1149.124035	1372.70636	1163.855498
**ENSG00000140718**	FTO	921.0811309	753.1888751	892.3996085	988.772944	1158.100526	987.9100856	1054.07916	1234.355289	978.7245944	1187.723466	1352.012295	1170.6221
**ENSG00000140740**	UQCRC2	1347.169043	1296.185506	1173.310069	1372.225957	1309.508878	1248.54535	1375.818438	1573.263066	1378.749548	1433.241156	1542.397699	1542.785195
**ENSG00000140743**	CDR2	570.9296781	329.3011826	468.9983344	594.4358935	424.174544	614.5984404	388.1299233	284.0844608	411.7490148	348.6604314	291.0965247	515.107557
**ENSG00000140750**	ARHGAP17	907.0188235	1110.51569	1078.044783	757.6964558	931.5658923	890.408975	715.9975689	737.6228104	590.8926636	558.7425788	812.5869812	562.4737692
**ENSG00000140795**	MYLK3	1.406230734	3.50320407	0.814233219	4.186168264	1.155788948	4.55258104	6.128367209	11.62918845	7.972361335	6.960553076	8.277626294	4.229126084
**ENSG00000140807**	NKD1	21.09346101	24.52242849	21.17006371	43.53614995	67.035759	49.69900968	1.021394535	3.322625272	2.344812157	5.694997971	5.518417529	5.074951301
**ENSG00000140829**	DHX38	1303.575891	1296.185506	1483.532926	1338.736611	1310.664667	1469.724912	1142.940485	1147.967032	1148.957957	1178.231803	1270.615636	1106.339384
**ENSG00000140830**	TXNL4B	260.1526858	315.2883663	312.6655563	183.35417	196.4841212	199.1754205	205.3003015	265.8100218	229.322629	219.5738107	227.6347231	230.064459
**ENSG00000140832**	MARVELD3	465.462373	542.9966309	496.6822639	493.9678552	621.8144542	568.3138665	289.0546534	338.9077778	286.0670832	277.7893455	386.289227	372.1630954
**ENSG00000140835**	CHST4	2.812461468	14.01281628	9.770798633	7.535102876	15.02525633	12.51959786	0	0	0	0	0	0
**ENSG00000140836**	ZFHX3	1733.882495	1681.537954	1797.826949	1687.863044	1806.498126	1981.131516	2881.353983	2541.808333	3042.159293	2955.07117	2622.627931	3037.358353
**ENSG00000140848**	CPNE2	226.4031482	325.7979785	220.6572025	233.5881891	246.183046	186.6558226	6.128367209	24.91968954	13.13094808	12.65555105	12.41643944	19.45397999
**ENSG00000140853**	NLRC5	2626.839011	2263.069829	2607.174769	82.04889798	206.8862217	99.39801937	1425.866771	1442.019368	1373.590962	22.77999189	16.55525259	18.60815477
**ENSG00000140854**	KATNB1	94.21745919	126.1153465	84.68025482	138.1435527	82.06101533	138.47434	116.438977	91.37219499	133.1853305	127.188288	84.15586732	120.953006
**ENSG00000140859**	KIFC3	426.0879124	493.9517739	489.3541649	606.1571647	569.8039515	646.4665076	216.5356414	189.3896405	226.039892	174.0138269	208.3202617	155.6318399
**ENSG00000140876**	NUDT7	40.78069129	45.54165292	43.96859385	39.34998168	46.23155793	29.212395	70.47622291	61.46856754	46.42728071	59.48108992	81.39665856	52.44116344
**ENSG00000140905**	GCSH	44.99938349	10.50961221	33.383562	38.51274803	27.73893476	39.07632059	23.4920743	29.90362745	48.30313044	55.68442461	52.42496653	65.12854169
**ENSG00000140931**	CMTM3	12.65607661	31.52883663	14.65619795	13.39573845	25.42735686	20.48661468	88.86132453	112.9692593	67.0616277	121.4932901	135.2012295	71.89514343
**ENSG00000140932**	CMTM2	4.218692202	3.50320407	8.142332194	3.348934611	2.311577897	1.896908767	2.04278907	6.645250545	1.875849726	6.960553076	2.759208765	0.845825217
**ENSG00000140939**	NOL3	174.372611	346.817203	219.8429693	234.4254228	401.058765	237.4929776	185.8938053	347.214341	190.3987472	202.4888168	292.476129	141.2528112
**ENSG00000140941**	MAP1LC3B	1770.444494	2343.643523	2294.509212	1692.049212	1970.620157	1957.609847	1258.358067	1388.857364	1331.853305	1275.679546	1336.836646	1400.686559
**ENSG00000140943**	MBTPS1	1863.255723	1898.736606	2081.994342	2187.691535	2249.165293	2381.758647	2193.955461	2050.059793	2243.516272	2295.71696	2372.919538	2474.884584
**ENSG00000140945**	CDH13	130.7794583	178.6634076	141.6765802	99.63080469	105.1767943	103.9506004	4.08557814	9.967875817	2.813774589	0.632777552	0	4.229126084
**ENSG00000140948**	ZCCHC14	916.8624387	931.8522827	1038.147355	1006.354851	1041.365842	1170.392709	759.9175339	734.3001852	754.0915898	781.4802772	784.9948935	854.2834689
**ENSG00000140950**	TLDC1	2944.647157	2291.095462	2278.224548	2960.458197	2754.245064	2468.637069	664.9278422	1064.9014	718.450445	620.7547789	1002.972386	657.2061934
**ENSG00000140961**	OSGIN1	167.3414574	77.07048955	148.1904459	133.1201508	112.111528	188.1733496	73.54040651	86.38825708	93.32352386	78.4644165	80.01705417	84.58252168
**ENSG00000140968**	IRF8	5.624922937	14.01281628	16.28466439	0	0	0	4.08557814	0	3.28273702	2.53111021	0	1.691650434
**ENSG00000140983**	RHOT2	424.6816817	364.3332233	445.385571	425.3146957	413.7724435	470.8127559	427.9643101	287.407086	407.0593905	423.3281825	353.1787219	369.6256197
**ENSG00000140986**	RPL3L	2.812461468	0	0.814233219	0.837233653	0	3.793817533	0	0	0.468962431	0.632777552	0	0
**ENSG00000140987**	ZSCAN32	224.9969175	203.1858361	206.8152377	205.9594786	194.1725433	202.5898563	235.9421376	201.018829	276.6878346	229.6982515	216.597888	222.452032
**ENSG00000140988**	RPS2	10676.10373	9395.593317	8697.63925	11603.2212	10580.09203	11008.89972	10857.42391	10589.20674	11782.21213	11381.76983	10845.07005	12602.79573
**ENSG00000140990**	NDUFB10	701.7091363	756.6920792	664.4143071	655.5539502	784.7806959	766.7305234	585.2590685	473.4741013	588.5478515	571.3981298	607.0259282	616.606583
**ENSG00000140992**	PDPK1	843.7384405	840.7689769	884.2572763	799.5581385	888.8017012	911.6543532	890.6560344	925.3511383	948.2420364	889.6852386	964.3434632	976.9281254
**ENSG00000140993**	TIGD7	89.99876699	87.58010176	121.3207497	94.60740277	95.93048271	91.05162079	148.1022076	146.195512	151.4748654	126.5555105	142.0992514	158.1693155
**ENSG00000140995**	DEF8	167.3414574	224.2050605	240.1987997	173.3073661	261.2083023	266.3259908	25.53486337	53.16200436	31.88944534	46.19276132	26.21248326	24.52893129
**ENSG00000141002**	TCF25	700.3029056	665.6087734	623.7026461	726.7188107	654.1765447	625.979893	520.9112128	475.1354139	502.2587641	589.7486788	544.943731	487.1953249
**ENSG00000141012**	GALNS	199.6847643	259.2371012	226.356835	236.9371238	312.063016	242.8043221	222.6640086	270.7939597	218.0675306	253.7437985	280.0596896	196.2314503
**ENSG00000141013**	GAS8	314.9956845	301.2755501	324.0648213	266.2403016	269.2988249	211.3156366	196.1077507	192.7122658	183.8332731	170.8499391	204.1814486	160.7067912
**ENSG00000141026**	MED9	165.9352266	213.6954483	202.7440716	193.4009738	166.4336086	201.8310928	262.4983955	186.0670152	206.3434698	277.1565679	233.1531406	257.1308659
**ENSG00000141027**	NCOR1	2670.432164	2669.441502	3193.422687	3015.715618	3237.364844	3189.4624	3054.991054	2990.362745	3018.242209	2931.6584	2941.316543	3110.099322
**ENSG00000141030**	COPS3	1082.797665	1131.534915	1122.82761	1250.827077	1364.986748	1288.759816	1144.983274	1209.435599	1087.523879	1121.9146	1027.805265	1219.679963

**ENSG00000141034**	GID4	344.5265299	360.8300193	393.274645	279.6360401	323.6209055	333.0971794	236.9635321	294.0523366	327.8047396	268.2976822	251.0879976	337.4842615
**ENSG00000141040**	ZNF287	29.53084542	38.53524477	24.42699658	29.30317785	17.33683422	34.9031213	56.17669942	44.85544118	34.2342575	45.55998377	45.52694462	38.90795997
**ENSG00000141052**	MYOCD	28.12461468	14.01281628	13.02773151	30.97764516	12.71367843	23.5216687	6.128367209	11.62918845	15.00679781	9.491663286	26.21248326	22.83728085
**ENSG00000141068**	KSR1	309.3707615	192.6762239	224.7283686	478.8976494	369.8524634	363.8271014	383.0229506	415.328159	370.4803208	385.994307	518.7312477	346.7883389
**ENSG00000141076**	UTP4	1310.607044	1019.432384	1170.86737	1280.130255	1036.742687	1331.629954	814.0514443	654.5571786	849.2909634	812.4863773	687.0429824	952.3991941
**ENSG00000141084**	RANBP10	299.5271464	311.7851623	430.7293731	422.8029947	354.8272071	384.6930979	450.4349899	692.7673693	477.4037552	482.8092725	602.8871151	459.2830927
**ENSG00000141086**	CTRL	1.406230734	10.50961221	0	0	0	1.517527013	0	6.645250545	0	0	0	0
**ENSG00000141098**	GFOD2	312.183223	157.6441832	281.7246939	344.940265	382.5661419	434.0127258	399.3652631	385.4245316	314.2048291	348.0276538	484.2411382	378.0838719
**ENSG00000141101**	NOB1	570.9296781	395.86206	572.4059533	458.8040418	402.214554	538.3427079	401.4080522	297.3749619	480.2175298	421.4298499	328.345843	377.2380467
**ENSG00000141127**	PRPSAP2	355.7763757	332.8043867	408.7450762	447.9200043	485.4313583	363.0683379	501.5047166	579.79811	416.9076016	438.5148438	527.008874	416.1460067
**ENSG00000141179**	PCTP	137.8106119	143.6313669	186.4594073	154.0509921	191.8609654	187.0352044	379.958767	350.5369662	298.2601064	297.4054496	338.0030737	307.8803789
**ENSG00000141198**	TOM1L1	1049.048128	914.3362624	904.6131068	910.9102143	895.7364349	941.2461299	807.9230771	624.6535512	666.3956151	728.3269628	633.2384115	719.7972595
**ENSG00000141219**	C17orf80	472.4935267	371.3396315	479.5833663	469.6880793	374.4756192	427.563236	403.4508413	416.9894717	358.7562601	364.4798702	373.8727876	408.5335797
**ENSG00000141232**	TOB1	1072.95405	2049.374381	1060.945885	983.7495421	1720.969744	1143.077223	692.5054946	1506.810561	898.0630562	666.3147627	1255.439988	1012.452784
**ENSG00000141252**	VPS53	1269.826353	1215.611812	1387.453406	1195.569656	1363.830959	1341.114498	1196.053	1242.661852	1046.255185	1155.451811	1396.159635	1144.401518
**ENSG00000141258**	SGSM2	143.4355349	203.1858361	132.7200148	180.0052354	245.027257	154.4083736	202.2361179	240.8903322	216.6606433	215.1443678	237.2919538	165.7817425
**ENSG00000141279**	NPEPPS	5301.489868	7013.414549	5702.889469	5515.695305	5916.483626	5619.40253	3247.013226	5479.009074	3045.910992	3211.346078	5632.924693	3425.592128
**ENSG00000141294**	LRRC46	23.90592248	10.50961221	9.770798633	38.51274803	28.89472371	19.72785117	33.70601965	39.87150327	19.22745969	28.47498986	33.11050518	15.2248539
**ENSG00000141295**	SCRN2	165.9352266	154.1409791	146.5619795	148.1903566	137.5388848	146.061975	165.4659146	139.5502614	165.5437383	151.233835	151.7564821	145.4819373
**ENSG00000141298**	SSH2	764.9895194	830.2593647	855.7591136	974.5399719	1103.778446	1082.755524	628.1576389	800.7526906	572.6031288	684.6653117	1040.221704	684.2726004
**ENSG00000141337**	ARSG	25.31215321	3.50320407	15.47043117	27.62871054	9.246311586	14.03712487	34.72741419	28.24231481	34.2342575	55.68442461	51.04536215	24.52893129
**ENSG00000141338**	ABCA8	0	0	0	0	0	0	1.021394535	0	0.468962431	3.796665314	6.898021912	5.074951301
**ENSG00000141349**	G6PC3	541.3988327	511.4677943	394.0888782	333.2189938	396.4356093	344.8580138	445.3280172	360.504842	398.6180667	336.6376579	240.0511625	350.1716397
**ENSG00000141367**	CLTC	12282.01923	12678.09553	12538.37735	11639.22224	12416.64067	12460.79369	8683.896336	8969.426922	9085.209185	8889.891833	9044.68633	10349.51735
**ENSG00000141371**	C17orf64	8.437384405	0	0	0.837233653	0	0	1.021394535	0	0	2.53111021	1.379604382	0
**ENSG00000141376**	BCAS3	326.2455303	360.8300193	318.3651888	256.1934978	287.7914481	292.12395	356.4666927	388.7471569	322.6461528	353.0898742	404.224084	326.4885337
**ENSG00000141378**	PTRH2	628.5851382	332.8043867	528.4373594	423.6402284	282.0125034	419.2168374	354.4239036	210.9867048	290.2877451	363.8470926	255.2268107	275.7390207
**ENSG00000141380**	SS18	2359.655172	2396.191584	2415.829962	2245.460657	2245.697926	2254.66576	2414.57668	2485.323704	2452.204554	2429.233024	2526.055624	2608.524969
**ENSG00000141384**	TAF4B	246.0903785	262.7403053	227.1710682	236.9371238	190.7051765	190.8290219	156.2733638	162.8086383	140.219767	177.8104922	124.1643944	119.2613556
**ENSG00000141385**	AFG3L2	1126.390818	840.7689769	1072.34515	1328.689807	1088.753189	1182.153543	1316.577555	1280.872042	1386.72191	1488.925581	1455.482623	1628.213542
**ENSG00000141391**	PRELID3A	8.437384405	17.51602035	13.02773151	5.023401917	8.090522638	7.587635066	7.149761744	1.661312636	3.28273702	1.265555105	11.03683506	3.383300867
**ENSG00000141401**	IMPA2	167.3414574	143.6313669	192.973273	267.0775353	223.067267	228.7671972	761.960323	358.8435294	686.5609996	832.7352589	469.06549	862.7417211
**ENSG00000141404**	GNAL	11.24984587	0	14.65619795	1.674467306	10.40210053	13.65774312	4.08557814	3.322625272	4.689624315	6.960553076	17.93485697	1.691650434
**ENSG00000141424**	SLC39A6	863.4256708	823.2529565	969.7517644	905.0495787	917.6964249	1028.883315	798.7305263	606.3791122	889.15277	788.4408303	702.2186306	1075.889676
**ENSG00000141425**	RPRD1A	876.0817474	882.8074257	788.9919896	838.9081202	999.7574402	807.324371	596.4944084	501.7164161	576.3548283	644.8003259	609.785137	599.6900787
**ENSG00000141428**	C18orf21	274.2149932	238.2178768	269.5111956	269.5892362	206.8862217	196.1403665	233.8993485	259.1647712	218.5364931	203.7543719	201.4222398	231.7561094
**ENSG00000141429**	GALNT1	1018.111052	900.3234461	941.2536017	1467.670593	1427.399351	1495.522872	2017.254206	1442.019368	2016.069493	2341.276944	1774.171236	2596.683416
**ENSG00000141434**	MEP1B	5.624922937	0	8.142332194	3.348934611	0	0	0	0	2.344812157	1.265555105	4.138813147	0
**ENSG00000141437**	SLC25A52	0	0	0.814233219	0	1.155788948	0	1.021394535	3.322625272	1.406887294	0.632777552	1.379604382	1.691650434
**ENSG00000141441**	GAREM1	539.9926019	833.7625688	653.8292752	734.2539136	709.6544142	697.6830443	715.9975689	647.9119281	672.9610891	762.4969506	633.2384115	707.9557064
**ENSG00000141446**	ESCO1	715.7714437	819.7497525	782.4781239	619.5529031	738.5491379	637.7407273	732.3398815	1001.77152	734.8641301	711.2419689	870.5303652	809.4547325
**ENSG00000141447**	OSBPL1A	634.2100611	528.9838146	464.9271683	560.1093138	604.4776199	547.8272518	1067.357289	1001.77152	1116.130587	1039.653519	856.7343214	1150.322295
**ENSG00000141448**	GATA6	120.9358431	185.6698157	166.1035768	128.9339825	147.9409854	229.146579	31.66323058	94.69482026	52.52379232	30.37332251	88.29468047	68.51184256
**ENSG00000141449**	GREB1L	19.68723028	28.02563256	37.45472809	24.27977593	46.23155793	76.63511417	340.1243801	299.0362745	415.9696767	379.0337539	338.0030737	537.0990127
**ENSG00000141452**	RMC1	449.9938349	521.9774065	498.3107303	437.0359668	365.2293077	484.0911172	391.1941069	400.3763453	435.1971364	425.2265152	389.0484358	418.6834823
**ENSG00000141456**	PELP1	1174.202663	746.182467	1115.499511	1168.778179	1070.260566	1257.27113	800.7733153	707.719183	944.0213745	890.3180162	783.6152892	811.9922081
**ENSG00000141458**	NPC1	1729.663803	1902.23981	1919.961931	1358.830219	1351.117281	1711.011707	854.9072257	990.1423311	1072.517081	886.5213509	1078.850627	1071.66055
**ENSG00000141469**	SLC14A1	5.624922937	14.01281628	29.3123959	11.72127114	18.49262317	15.55465189	0	0	0	0	0	0
**ENSG00000141480**	ARRB2	345.9327606	329.3011826	312.6655563	425.3146957	365.2293077	384.6930979	231.8565594	244.2129575	237.2949903	284.117121	257.9860195	246.1351381
**ENSG00000141497**	ZMYND15	47.81184496	122.6121425	64.32442434	24.27977593	27.73893476	29.212395	17.36370709	28.24231481	20.16538455	12.65555105	5.518417529	17.76232955
**ENSG00000141499**	WRAP53	154.6853808	136.6249587	144.9335131	181.6797027	156.031508	162.7547722	105.2036371	111.3079466	130.3715559	147.4371697	137.9604382	131.9487338
**ENSG00000141503**	MINK1	2199.344868	2024.851953	1846.680942	2115.689441	2356.653666	2079.012008	1619.931732	2086.608671	1735.160996	1729.381051	2120.451936	1574.926554
**ENSG00000141504**	SAT2	98.43615139	164.6505913	145.7477463	209.3084132	196.4841212	151.3733196	88.86132453	121.2758224	103.6406974	109.4705166	122.78479	109.9572782
**ENSG00000141505**	ASGR1	15.46853808	0	0.814233219	0	1.155788948	0.758763507	0	0	0.468962431	0	0	0
**ENSG00000141506**	PIK3R5	0	0	0	0	0	2.655672273	0	8.306563181	1.406887294	0.632777552	1.379604382	0
**ENSG00000141510**	TP53	2553.715013	2574.854992	2721.167419	2042.012879	2338.161042	2409.832897	2833.34844	2468.710577	3018.242209	2335.581946	2567.443755	2330.248472
**ENSG00000141519**	CCDC40	30.93707615	0	7.328098975	18.41914036	17.33683422	15.93403364	53.11251581	43.19412854	38.92388181	56.94997971	34.49010956	27.91223215
**ENSG00000141522**	ARHGDIA	3270.892688	2837.595297	3379.882094	3322.143135	3050.127034	3753.223685	1362.54031	1292.501231	1611.823877	1457.919481	1255.439988	1620.601115
**ENSG00000141524**	TMC6	136.4043812	119.1089384	163.6608771	129.7712162	122.5136285	173.756843	17.36370709	13.29050109	37.04803208	22.14721433	38.6289227	58.36193996
**ENSG00000141526**	SLC16A3	1372.481197	2904.156174	1308.472784	1572.3248	2610.927234	1725.807596	306.4183605	794.1074401	361.1010722	333.4737701	880.1875959	393.3087258
**ENSG00000141527**	CARD14	12.65607661	3.50320407	3.256932878	13.39573845	10.40210053	8.346398573	8.171156279	18.274439	11.25509835	17.71777147	6.898021912	9.304077385
**ENSG00000141540**	TTYH2	1.406230734	0	8.142332194	0.837233653	2.311577897	3.035054026	8.171156279	8.306563181	2.344812157	0.632777552	4.138813147	3.383300867
**ENSG00000141542**	RAB40B	2.812461468	3.50320407	8.142332194	15.07020575	26.58314581	15.55465189	41.87717593	28.24231481	16.88264753	35.43554293	45.52694462	41.44543562
**ENSG00000141543**	EIF4A3	1307.794583	1236.631037	1161.096571	1101.799487	1094.532134	1159.77002	1053.057765	1026.691209	1213.674773	1094.705166	1151.969659	1115.643461
**ENSG00000141551**	CSNK1D	1139.046895	788.2209158	1151.325772	1054.077169	862.2185554	1058.09571	1066.335894	1003.432832	1021.400176	1012.444084	985.037529	971.0073489
**ENSG00000141552**	ANAPC11	323.4330689	329.3011826	306.1516905	346.6147323	325.9324834	379.7611351	235.9421376	264.1487091	344.6873871	244.8849128	329.7254474	300.267952
**ENSG00000141556**	TBCD	541.3988327	567.5190594	530.8800591	644.6699127	666.8902231	676.0582844	724.1687252	518.3295425	706.2574218	772.6213915	671.8673342	691.0392021

**ENSG00000141560**	FN3KRP	341.7140684	399.365264	312.6655563	355.8243025	403.3703429	361.1714291	307.439755	343.8917157	323.1151153	353.7226518	409.7425015	359.4757171
**ENSG00000141562**	NARF	305.1520693	542.9966309	341.1637189	360.0104707	552.4671173	356.9982299	281.9048916	481.7806645	299.6669937	385.3615294	485.6207426	387.3879493
**ENSG00000141564**	RPTOR	705.9278285	521.9774065	759.6795937	756.0219885	603.321831	911.2749714	700.6766509	627.9761765	724.0779942	749.208622	620.821972	668.2019213
**ENSG00000141568**	FOXK2	1579.197114	1180.579772	1406.18077	1374.737658	1180.060516	1520.941449	1207.28834	976.85183	1297.150085	1166.841807	1154.728868	1272.966951
**ENSG00000141569**	TRIM65	279.8399161	304.7787541	300.452058	292.1945449	300.5051265	332.7177976	180.7868327	264.1487091	238.7018776	191.7315984	266.2636458	230.9102842
**ENSG00000141570**	CBX8	97.02992066	87.58010176	72.46675653	82.88613163	107.4883722	71.70315137	29.62044151	64.79119281	33.29633263	29.74054496	40.00852709	41.44543562
**ENSG00000141574**	SECTM1	1442.792733	1418.797649	1609.739075	92.93293547	129.4483622	101.6743099	201.2147234	327.2785893	310.4531296	15.81943881	44.14734023	12.68737825
**ENSG00000141576**	RNF157	125.1545353	189.1730198	112.3641843	198.4243757	188.3935986	171.8599342	194.0649616	166.1312636	177.7367615	265.766572	245.5695801	235.9852355
**ENSG00000141577**	CEP131	215.1533023	227.7082646	208.4437042	245.3094603	238.0925233	228.3878155	189.9793835	91.37219499	167.8885505	151.8666126	208.3202617	181.8524216
**ENSG00000141579**	ZNF750	130.7794583	87.58010176	88.75142092	92.93293547	53.16629162	49.31962793	60.26227756	18.274439	53.93067962	60.74664503	26.21248326	51.59533822
**ENSG00000141580**	WDR45B	883.1129011	907.3298542	860.644513	754.3475212	840.2585654	841.4687288	712.9333853	760.8811873	704.381572	641.0036606	822.2442119	643.67299
**ENSG00000141582**	CBX4	404.9944514	241.7210809	319.9936552	324.8466573	317.8419608	340.3054327	237.9849266	214.3093301	210.0951693	184.7710453	143.4788558	173.3941694
**ENSG00000141622**	RNF165	1.406230734	17.51602035	5.699632536	2.511700959	9.246311586	6.449489806	1.021394535	48.17806645	8.441323766	6.327775524	31.73090079	5.074951301
**ENSG00000141627**	DYM	731.2399818	781.2145077	679.070505	776.1155962	918.8522139	803.5305535	932.5332103	936.9803268	848.3530385	861.8430263	765.6804322	869.5083228
**ENSG00000141639**	MAPK4	299.5271464	329.3011826	193.7875062	621.2273704	718.9007258	472.3302829	1143.961879	679.4768682	1156.461356	1445.896707	758.7824103	1695.87956
**ENSG00000141642**	ELAC1	99.84238213	129.6185506	125.3919158	103.816973	94.77469376	75.11758715	127.6743169	101.3400708	129.4336311	133.5160636	64.84140597	133.6403843
**ENSG00000141644**	MBD1	970.2992066	994.909956	1279.160388	1064.123973	1092.220556	1179.877253	990.7526988	986.8197059	1089.399728	1017.506304	1158.867681	1006.532008
**ENSG00000141646**	SMAD4	1247.326661	1170.07016	1129.341475	1210.639862	1214.734185	1142.318459	1803.782749	2118.173611	1666.692481	1774.941034	2149.423628	1683.192181
**ENSG00000141655**	TNFRSF11A	14.06230734	42.03844885	21.98429693	20.93084132	33.5178795	20.48661468	6.128367209	9.967875817	8.910286198	4.429442867	5.518417529	3.383300867
**ENSG00000141664**	ZCCHC2	535.7739097	441.4037129	547.1647235	547.550809	536.286072	610.0458593	818.1370224	1096.46634	869.9253103	734.0219608	986.4171334	839.058615
**ENSG00000141665**	FBXO15	4.218692202	31.52883663	8.956565414	4.186168264	6.93473369	4.55258104	2.04278907	3.322625272	4.689624315	8.226108181	4.138813147	0
**ENSG00000141668**	CBLN2	0	0	1.628466439	0	1.155788948	0.379381753	180.7868327	119.6145098	185.2401604	158.8271656	132.4420207	242.7518372
**ENSG00000141682**	PMAIP1	3172.456536	3814.989233	3736.516244	1136.126067	1229.759441	1794.096311	1413.610036	908.738012	1576.182732	670.7442055	361.4563482	850.9001681
**ENSG00000141696**	P3H4	279.8399161	245.2242849	361.5195494	379.2668447	338.6461618	442.7385061	289.0546534	260.8260839	346.0942744	388.5254172	274.5412721	395.8462015
**ENSG00000141698**	NT5C3B	274.2149932	206.6890402	234.4991672	354.9870688	338.6461618	352.8250306	223.6854031	235.9063943	283.722271	299.3037823	251.0879976	307.0345537
**ENSG00000141699**	RETREG3	743.8960584	1050.961221	894.8423082	790.3485683	861.0627665	849.0563639	868.1853546	868.8665087	883.9941833	829.5713712	880.1875959	740.9428899
**ENSG00000141736**	ERBB2	507.649295	564.0158553	455.1563697	563.4582484	655.3323337	551.6210693	800.7733153	622.9922385	786.4499975	803.6274915	768.4396409	685.9642508
**ENSG00000141738**	GRB7	992.7988983	938.8586909	1004.763793	659.7401185	732.7701932	695.0273721	889.6346399	706.0578704	984.8211061	740.9825138	568.3970055	586.1568752
**ENSG00000141741**	MIEN1	483.7433725	455.4165292	508.8957622	475.5487148	439.1998003	552.3798328	319.6964894	312.3267756	383.1423065	239.8226924	182.1077785	319.7219319
**ENSG00000141744**	PNMT	1.406230734	0	0	1.674467306	0	0	0	1.661312636	0.937924863	1.265555105	1.379604382	0.845825217
**ENSG00000141753**	IGFBP4	113.9046895	84.07689769	135.1627144	87.0722999	128.2925733	147.9588838	5.106972674	24.91968954	26.73085859	14.5538837	16.55525259	21.14563042
**ENSG00000141756**	FKBP10	808.5826721	683.1247937	735.2525972	819.6517462	795.1827964	886.6151575	627.1362444	571.4915468	792.5465092	697.3208627	645.6548509	708.8015317
**ENSG00000141759**	TXNL4A	635.6162918	511.4677943	589.5048509	792.0230356	584.8292078	731.0686386	509.6758729	421.9734096	558.0652934	614.4270034	524.2496653	641.9813395
**ENSG00000141837**	CACNA1A	49.2180757	52.54806106	39.89742775	42.6989163	11.55788948	39.45570234	11.23533988	8.306563181	4.689624315	0	2.759208765	4.229126084
**ENSG00000141854**	MISP3	1.406230734	7.006408141	5.699632536	10.04680383	17.33683422	7.587635066	6.128367209	9.967875817	3.751699452	2.53111021	8.277626294	5.920776517
**ENSG00000141858**	SAMD1	478.1184496	581.5318757	384.3180796	628.7624733	752.4186053	644.9489806	597.5158029	624.6535512	711.4160085	700.4847505	811.2073768	698.6516291
**ENSG00000141867**	BRD4	2843.398544	2508.294114	2682.898458	2396.162715	2575.097777	2707.268192	2922.209764	3518.660163	3014.021547	2775.362345	3407.622824	2867.347485
**ENSG00000141873**	SLC39A3	115.3109202	105.0961221	72.46675653	122.2361133	94.77469376	133.9217589	69.45482837	64.79119281	88.63389954	110.7360717	38.6289227	66.82019213
**ENSG00000141905**	NFIC	1853.412108	1779.627668	1955.788193	2115.689441	2383.236811	2255.424523	1096.97773	1098.127652	1270.419227	1188.989021	1102.303901	1174.005401
**ENSG00000141933**	TPGS1	47.81184496	80.57369362	53.73939248	47.72231821	55.47786952	48.94024618	57.19809395	54.82331699	46.42728071	44.29442867	33.11050518	66.82019213
**ENSG00000141934**	PLPP2	75.93645964	77.07048955	83.8660216	95.44463643	104.0210053	82.32584047	1.021394535	1.661312636	5.627549177	1.898332657	1.379604382	0
**ENSG00000141956**	PRDM15	188.4349184	157.6441832	243.4557326	229.4020209	239.2483123	249.2538119	227.7709813	244.2129575	253.7086754	234.1276944	269.0228546	208.0730033
**ENSG00000141959**	PFKL	718.5839052	1198.095792	720.5963992	745.1379511	1102.622657	893.0646473	735.4040651	1295.823856	884.9321082	768.1919486	1214.051856	867.8166724
**ENSG00000141965**	FEM1A	461.2436808	381.8492437	640.8015437	720.8581751	584.8292078	784.1820841	551.5530488	426.9573475	583.8582272	539.1264746	463.5470725	619.9898839
**ENSG00000141968**	VAV1	33.74953762	3.50320407	16.28466439	37.67551438	20.80420107	33.00621254	1.021394535	0	3.28273702	3.163887762	1.379604382	3.383300867
**ENSG00000141971**	MVB12A	226.4031482	213.6954483	231.2422343	229.4020209	175.6799201	223.4558527	185.8938053	176.0991394	197.9021461	227.7999189	160.0341083	200.4605764
**ENSG00000141979**	AC008764.1	1.406230734	21.01922442	3.256932878	6.697869223	8.090522638	1.896908767	4.08557814	3.322625272	2.344812157	1.265555105	5.518417529	0
**ENSG00000141985**	SH3GL1	676.3969831	777.7113036	700.2405687	663.0890531	821.7659422	790.6315739	952.961101	913.7219499	1100.654827	968.1496551	1038.8421	962.5490967
**ENSG00000141994**	DUS3L	236.2467633	115.6057343	207.629471	232.7509555	160.6546638	248.1156667	185.8938053	117.9531972	178.6746864	173.3810494	166.9321303	160.7067912
**ENSG00000142002**	DPP9	1878.724261	1558.925811	1859.708673	1772.423643	1885.091775	2124.158437	952.961101	1081.514526	1159.275131	1086.479057	1240.26434	1113.95181
**ENSG00000142039**	CCDC97	310.7769922	311.7851623	355.8199169	288.8456102	284.3240813	324.7507808	300.2899933	219.293268	274.3430224	239.1899148	217.9774924	258.8225163
**ENSG00000142046**	TMEM91	0	14.01281628	0.814233219	1.674467306	13.86946738	5.6907263	20.4278907	18.274439	23.44812157	15.18666126	27.59208765	16.91650434
**ENSG00000142065**	ZFP14	108.2797665	147.134571	122.9492161	99.63080469	191.8609654	119.1258705	233.8993485	202.6801416	197.4331836	183.5054902	201.4222398	203.8438772
**ENSG00000142082**	SIRT3	161.7165344	164.6505913	145.7477463	149.8648239	162.9662417	128.2310326	301.3113878	309.0041503	264.0258489	248.0488005	344.9010956	264.7432929
**ENSG00000142089**	IFITM3	2712.619086	2676.44791	2372.675601	581.0401551	765.1322838	682.5077742	1886.515706	2124.818862	2458.770028	757.4347302	791.8929154	827.217062
**ENSG00000142102**	PGGHG	150.4666886	119.1089384	101.7791524	159.9116277	125.9809954	91.43100255	81.71156279	74.75906863	78.31672605	115.7982921	103.4703287	103.1906764
**ENSG00000142149**	HUNK	40.78069129	70.06408141	26.05546302	45.21061725	55.47786952	43.62890163	1105.148887	860.5599455	1378.749548	1212.40179	1014.009221	1558.855875
**ENSG00000142156**	COL6A1	205.3096872	423.8876925	362.3337827	270.4264699	446.134534	339.5466692	44.94135953	64.79119281	31.88944534	59.48108992	66.22101035	65.97436691
**ENSG00000142166**	IFNAR1	1094.047511	1303.191914	1266.132656	1100.12502	1354.584647	1244.372151	900.8699798	968.5452669	810.3670816	846.0235875	899.5020573	952.3991941
**ENSG00000142168**	SOD1	1784.506802	2024.851953	2012.784518	1777.447045	2083.887474	1766.022062	1223.630653	1329.050109	1201.950712	1181.39569	1241.643944	1392.228307
**ENSG00000142173**	COL6A2	1.406230734	7.006408141	5.699632536	2.511700959	4.623155793	3.41443578	0	0	0	0.632777552	0	1.691650434
**ENSG00000142178**	SIK1	108.2797665	129.6185506	93.63682024	108.8403749	121.3578396	100.1567829	36.77020326	48.17806645	49.71001773	30.37332251	34.49010956	32.98718345
**ENSG00000142185**	TRPM2	140.6230734	119.1089384	163.6608771	179.1680017	208.0420107	232.5610148	28.59904698	14.95181373	45.48935585	30.37332251	19.31446135	57.51611474
**ENSG00000142186**	SCYL1	618.741523	430.8941007	643.2442434	662.2518194	716.5891479	745.1057635	523.9753964	505.0390414	600.7408747	534.0642542	521.4904565	520.1825083
**ENSG00000142188**	TMEM50B	447.1813735	416.8812844	464.1129351	303.0785823	250.8062018	325.1301626	311.5253331	313.9880882	355.9424855	292.3432292	262.1248326	301.1137772
**ENSG00000142192**	APP	10452.51305	11627.13431	11828.36598	9213.75635	11962.41561	11091.60494	10892.15132	12599.39503	12050.45864	12170.21066	13735.34123	13660.07725
**ENSG00000142197**	DOPEY2	462.6499115	458.9197332	555.3070557	553.4114445	572.1155294	681.3696289	406.5150249	820.6884422	376.5768325	415.7348519	681.5245649	390.7712502

**ENSG00000142207**	URB1	1161.546586	700.6408141	1135.041108	1179.662217	865.6859222	1258.788657	840.6077022	372.1340305	858.670212	809.3224895	635.9976202	853.4376437
**ENSG00000142208**	AKT1	1046.235666	928.3490787	1028.376556	1081.70588	1006.692174	1129.798861	910.0625306	930.3350762	1114.7237	1102.931274	1005.731595	1013.29861
**ENSG00000142224**	IL19	0	3.50320407	4.071166097	4.186168264	12.71367843	10.24330734	0	0	0	0	0	0
**ENSG00000142227**	EMP3	50.62430643	52.54806106	83.05178838	31.81487881	71.65891479	62.5979893	9.192550814	31.56494009	14.06887294	8.858885733	22.07367012	14.37902869
**ENSG00000142230**	SAE1	1340.13789	1401.281628	1432.236233	1723.026858	1598.456115	1713.287998	1111.277254	1023.368584	1326.225756	1325.036195	1299.587328	1524.177041
**ENSG00000142233**	NTN5	1.406230734	10.50961221	0	0.837233653	0	2.27629052	1.021394535	0	0	1.265555105	0	1.691650434
**ENSG00000142235**	LMTK3	75.93645964	87.58010176	88.75142092	120.561646	121.3578396	104.7093639	21.44928523	29.90362745	21.57227185	31.00610007	34.49010956	19.45397999
**ENSG00000142252**	GEMIN7	118.1233817	45.54165292	105.0360853	100.4680383	84.37259322	95.98358359	84.77574639	136.2276362	98.01314817	101.2444084	111.747955	88.81164776
**ENSG00000142273**	CBLC	66.09284451	52.54806106	83.05178838	88.7467672	104.0210053	84.98151274	4.08557814	0	17.8205724	13.92110615	6.898021912	17.76232955
**ENSG00000142279**	WTIP	14.06230734	24.52242849	17.91313083	16.74467306	8.090522638	14.41650663	25.53486337	46.51675381	28.13774589	19.61610412	23.4532745	27.06640694
**ENSG00000142303**	ADAMTS10	0	0	0.814233219	0.837233653	3.467366845	3.793817533	0	0	0	0	0	0
**ENSG00000142327**	RNPEPL1	206.7159179	287.2627338	220.6572025	207.6339459	307.4398602	269.3610448	180.7868327	230.9224564	243.8604644	218.3082556	240.0511625	212.3021294
**ENSG00000142330**	CAPN10	89.99876699	84.07689769	89.56565414	95.44463643	75.12628164	132.7836137	85.79714093	98.01744553	75.9719139	96.81496551	78.63744979	96.42407471
**ENSG00000142347**	MYO1F	4.218692202	0	4.885399317	13.39573845	3.467366845	4.931962793	1.021394535	0	2.813774589	1.265555105	2.759208765	1.691650434
**ENSG00000142396**	ERVK3-1	189.8411491	171.6569995	146.5619795	148.1903566	116.7346838	117.2289618	209.3858796	184.4057026	215.253756	180.3416024	202.8018442	177.6232955
**ENSG00000142408**	CACNG8	2.812461468	7.006408141	13.84196473	5.023401917	5.778944741	2.27629052	2.04278907	0	0	0	0	0
**ENSG00000142409**	ZNF787	120.9358431	119.1089384	121.3207497	184.1914036	131.7599401	158.5815729	173.6370709	114.6305719	129.9025935	134.1488411	114.5071637	106.5739773
**ENSG00000142444**	TIMM29	88.59253625	98.08971397	81.42332194	107.1659076	100.5536385	102.8124551	140.9524458	131.2436983	133.654293	122.7588452	144.8584601	123.4904816
**ENSG00000142453**	CARM1	648.2723684	532.4870187	701.0548019	648.856081	559.401851	665.4355953	636.3287952	574.8141721	815.5256683	710.6091913	555.9805661	680.0434743
**ENSG00000142459**	EVI5L	120.9358431	178.6634076	144.1192798	188.3775719	196.4841212	185.5176774	182.8296217	151.1794499	235.888103	201.2232617	271.7820633	237.6768859
**ENSG00000142494**	SLC47A1	0	0	4.885399317	5.023401917	13.86946738	0.758763507	0	0	0	0	0	0
**ENSG00000142507**	PSMB6	1316.231967	1233.127833	1326.385914	1282.641956	1245.940486	1432.166119	818.1370224	897.1088235	997.9520541	1065.597398	990.5559465	1013.29861
**ENSG00000142528**	ZNF473	327.6517611	241.7210809	311.851323	385.1274803	295.8819708	335.3734699	294.161626	318.9720261	386.4250435	346.1293212	344.9010956	369.6256197
**ENSG00000142530**	FAM71E1	15.46853808	3.50320407	8.142332194	7.535102876	8.090522638	8.725780326	10.21394535	3.322625272	7.034436472	8.858885733	9.657230676	10.99572782
**ENSG00000142534**	RPS11	4885.24557	5041.110657	5023.004731	4433.989426	4409.334838	4418.659281	4126.433921	4791.225643	4645.072884	4210.501834	4545.79644	4447.34899
**ENSG00000142541**	RPL13A	5584.142245	4624.229373	4746.979669	5508.160202	5060.044016	5145.934102	5073.266655	5457.41201	5422.612595	5012.230992	5906.086361	5475.872453
**ENSG00000142544**	CTU1	18.28099954	70.06408141	22.79853014	36.83828073	48.54313583	32.24744903	26.55625791	36.54887799	34.70321993	39.23220825	34.49010956	41.44543562
**ENSG00000142546**	NOSIP	227.8093789	238.2178768	332.2071535	325.683891	288.9472371	365.7240102	181.8082272	214.3093301	237.7639527	183.5054902	176.5893609	229.2186337
**ENSG00000142552**	RCN3	25.31215321	17.51602035	22.79853014	16.74467306	11.55788948	12.89897961	1.021394535	0	2.813774589	2.53111021	5.518417529	0.845825217
**ENSG00000142556**	ZNF614	187.0286876	336.3075908	227.9853014	264.5658343	306.2840713	242.0455586	424.9001265	382.1019063	405.1835408	477.1142745	459.4082593	433.062511
**ENSG00000142583**	SLC2A5	29.53084542	42.03844885	10.58503185	52.74572013	35.8294574	13.65774312	489.2479822	1038.320398	304.356618	442.9442867	1047.119726	317.1844563
**ENSG00000142599**	RERE	752.3334428	826.7561606	810.9762866	764.3943251	938.500626	718.9284225	1304.320821	1262.597603	1141.923521	1273.781213	1260.958405	1179.080352
**ENSG00000142609**	CFAP74	5.624922937	0	0.814233219	4.186168264	8.090522638	1.517527013	0	1.661312636	0	0	1.379604382	0
**ENSG00000142611**	PRDM16	2.812461468	0	3.256932878	0	2.311577897	1.13814526	4.08557814	8.306563181	0	0	4.138813147	0
**ENSG00000142619**	PADI3	39.37446056	42.03844885	19.54159727	69.49039319	173.3683422	61.45984404	2.04278907	6.645250545	0.937924863	5.062220419	9.657230676	4.229126084
**ENSG00000142621**	FHAD1	697.4904441	574.5254675	738.50953	451.2689389	651.8649668	476.1241004	28.59904698	26.58100218	44.55143099	35.43554293	27.59208765	32.14135824
**ENSG00000142623**	PADI1	84.37384405	206.6890402	227.9853014	144.0041883	429.9534888	264.0497003	16.34231256	69.77513072	34.70321993	13.2883286	46.906549	27.06640694
**ENSG00000142627**	EPHA2	3003.708848	3047.787541	3642.065191	3280.281452	2531.177797	4314.70868	1280.828747	1134.67653	1693.892302	1182.661245	975.3802983	1454.819373
**ENSG00000142632**	ARHGEF19	75.93645964	42.03844885	74.90945619	65.30422492	108.6441611	73.60006014	31.66323058	48.17806645	36.11010722	41.76331846	27.59208765	45.67456171
**ENSG00000142634**	EFHD2	1169.983971	1457.332893	1432.236233	1028.960159	1085.285822	1459.481605	358.5094817	294.0523366	386.4250435	391.0565274	380.7708095	404.3044536
**ENSG00000142655**	PEX14	234.8405326	178.6634076	277.6535278	293.0317785	294.7261818	306.1610749	258.4128173	181.0830773	237.7639527	260.071574	257.9860195	235.1394103
**ENSG00000142657**	PGD	1231.858123	1278.669486	1312.54395	1141.149469	1389.258316	1357.048532	1463.658368	1822.459962	1835.987919	1702.171616	2113.553914	2211.832942
**ENSG00000142661**	MYOM3	5.624922937	35.0320407	8.956565414	4.186168264	2.311577897	0.379381753	1.021394535	0	0	0	0	0
**ENSG00000142669**	SH3BGRL3	1604.509268	1951.284667	1893.906468	1558.091828	1717.502377	1813.444781	356.4666927	405.3602832	436.1350613	364.4798702	474.5839075	526.9491101
**ENSG00000142675**	CNKSR1	314.9956845	213.6954483	358.2626166	308.9392179	243.8714681	325.8889261	330.9318293	259.1647712	324.0530401	325.2476619	288.3373159	243.5976624
**ENSG00000142676**	RPL11	8741.130244	8908.647951	7730.330185	8656.158737	7894.038517	7779.981615	7592.025578	8931.216732	7931.092641	7669.896712	8754.96941	8531.838962
**ENSG00000142677**	IL22RA1	233.4343019	462.4229373	439.6859385	126.4222816	187.2378096	162.7547722	26.55625791	39.87150327	34.70321993	3.163887762	12.41643944	21.14563042
**ENSG00000142684**	ZNF593	232.0280711	161.1473872	223.9141353	252.8445632	173.3683422	228.7671972	124.6101333	106.3240087	143.9714665	147.4371697	104.8499331	161.5526164
**ENSG00000142686**	C1orf216	216.5595331	234.7146727	162.0324107	238.6115911	221.9114781	206.7630556	96.01108628	134.5663235	136.4680676	115.1655145	155.8952952	110.8031034
**ENSG00000142687**	KIAA0319L	1245.92043	1369.752792	1380.125307	1228.221769	1289.860466	1240.957715	1828.296217	1795.87896	1668.568331	1676.227736	1938.344157	1475.965003
**ENSG00000142694**	EVA1B	18.28099954	14.01281628	27.68392946	13.39573845	38.14103529	33.76497604	11.23533988	6.645250545	8.441323766	12.0227735	28.97169203	11.84155303
**ENSG00000142731**	PLK4	652.4910606	507.9645902	626.959579	761.0453905	671.5133789	768.6274322	472.9056696	541.5879194	507.4173508	589.1159013	608.4055326	575.1611474
**ENSG00000142733**	MAP3K6	295.3084542	409.8748762	243.4557326	344.1030313	444.9787451	371.0353547	229.8137703	297.3749619	249.956976	258.1732414	346.2807	266.4349433
**ENSG00000142751**	GPN2	388.1196826	325.7979785	400.602744	453.7806399	409.1492877	495.8519516	484.1410095	529.9587309	497.5691398	503.0581541	558.7397748	506.6493048
**ENSG00000142765**	SYTL1	500.6181414	255.7338971	349.3060511	381.7785457	364.0735187	305.0229297	254.3272392	259.1647712	275.7499097	248.6815781	255.2268107	176.7774703
**ENSG00000142784**	WDTC1	840.925979	1135.038119	909.4985061	819.6517462	910.7616912	940.4873664	1219.545075	1181.193284	1200.074862	1132.039041	1205.77423	962.5490967
**ENSG00000142794**	NBPF3	291.089762	231.2114686	273.5823617	236.9371238	395.2798203	281.8806427	322.760673	372.1340305	357.3493728	360.0504273	379.3912051	332.4093102
**ENSG00000142798**	HSPG2	1276.857507	1212.108608	1122.82761	1848.611906	1940.569644	2176.8925	1415.652825	1817.476024	1958.387114	1678.758846	2152.182836	2014.755666
**ENSG00000142856**	ITGB3BP	583.5857547	451.9133251	518.6665608	513.2242292	481.9639914	546.3097248	788.5165809	649.5732407	677.181751	709.9764138	664.9693123	734.1762882
**ENSG00000142864**	SERBP1	9172.843079	6449.398694	8029.96801	11248.23413	9179.275827	9503.133539	11987.08626	11482.99294	11604.0064	10649.64621	10578.8064	11965.04352
**ENSG00000142867**	BCL10	1379.51235	1678.03475	1413.508869	900.0261768	1069.104777	1111.588537	592.4088302	682.7994934	646.2302305	526.4709236	732.569927	571.7778465
**ENSG00000142871**	CYR61	3466.35876	3030.271521	5781.055858	2941.201823	2221.426359	5450.957031	1005.052222	1445.341993	1267.13649	780.2147221	1033.323682	1140.172392
**ENSG00000142875**	PRKACB	357.1826065	402.8684681	404.6739101	246.9839276	346.7366845	365.3446284	777.281241	1094.805027	831.0014285	615.0597809	666.3489167	712.1848325
**ENSG00000142892**	PIGK	534.367679	532.4870187	550.4216563	714.1603059	613.7239315	754.2109256	761.960323	840.6241939	860.5460617	843.4924773	747.7455752	974.3906497
**ENSG00000142910**	TINAGL1	1857.6308	2235.044197	2476.897454	2016.89587	2346.251565	2948.554987	4215.295245	2560.082772	4015.256338	3922.588047	2595.035843	3397.679896
**ENSG00000142920**	AZIN2	12.65607661	21.01922442	17.09889761	10.88403749	6.93473369	10.24330734	8.171156279	14.95181373	3.28273702	4.429442867	8.277626294	5.920776517
**ENSG00000142937**	RPS8	10955.94365	10793.37174	10018.32553	10995.38956	10743.05827	10278.96922	9467.305944	10677.25631	9954.19657	9151.228963	9941.429179	10189.65639

ENSG00000142945	KIF2C	1632.633882	1022.935589	1532.386919	1994.290561	1636.597151	1833.552014	1550.476904	1490.197435	1783.464127	1654.713299	1521.703634	1891.265185
ENSG00000142949	PTPRF	5858.357238	5556.081656	6289.137387	7922.742057	8950.429615	9203.421953	6211.100167	7015.723262	7602.818939	7299.089067	8759.108223	7700.392774
ENSG00000142959	BEST4	0	0	0	4.186168264	1.155788948	2.27629052	1.021394535	3.322625272	0	1.265555105	0	1.691650434
ENSG00000142961	MOB3C	686.2405983	956.3747112	833.7748167	426.1519293	572.1155294	471.1921376	538.2749199	584.7820479	451.1418591	287.9137863	309.0313816	285.8889233
ENSG00000142973	CYP4B1	2.812461468	0	2.442699658	3.348934611	5.778944741	1.896908767	46.9841486	31.56494009	21.57227185	36.06832049	8.277626294	10.99572782
ENSG00000143013	LMO4	632.8038304	413.3780803	564.2636211	596.9475945	545.5323836	571.3489205	1011.18059	727.6549346	969.8143082	916.8946734	780.8560804	1042.056667
ENSG00000143033	MTF2	873.2692859	1005.419568	884.2572763	936.0272239	768.5996506	808.4625163	1077.571234	1094.805027	1014.365739	994.0935348	1051.258539	1117.335111
ENSG00000143036	SLC44A3	292.4959927	294.2691419	288.2385597	233.5881891	377.9429861	327.7858349	146.0594185	240.8903322	124.7440068	97.44774307	219.3570968	120.1071808
ENSG00000143061	IGSF3	904.2063621	1558.925811	1152.140006	1678.653474	2213.335836	2130.228545	1231.801809	1897.21903	1193.97835	1359.83896	1898.33563	1390.536656
ENSG00000143067	ZNF697	465.462373	416.8812844	547.9789567	377.5923774	476.1850467	416.5611651	86.81853546	83.06563181	99.8889979	101.8771859	71.73942788	132.794559
ENSG00000143079	CTTNBP2NL	1867.474415	1965.297484	1849.937875	1288.502592	1295.639411	1462.516659	1513.706701	1412.115741	1381.563323	1294.030095	1120.238758	1235.750642
ENSG00000143093	STRIP1	568.1172166	511.4677943	612.303381	606.9943983	540.9092278	519.3736203	653.6925023	548.2331699	581.513415	641.0036606	627.719994	506.6493048
ENSG00000143106	PSMA5	3692.761908	3016.258705	3405.937557	2015.221402	1830.769694	1965.956246	2328.77954	2222.836307	2609.775931	1537.016675	1369.947152	1750.012374
ENSG00000143107	FNDC7	0	0	8.142332194	0	1.155788948	3.41443578	5.106972674	0	1.406887294	0.632777552	2.759208765	4.229126084
ENSG00000143110	C1orf162	22.49969175	24.52242849	13.02773151	15.9074394	12.71367843	10.24330734	31.66323058	31.56494009	19.69642212	32.27165517	27.59208765	16.07067912
ENSG00000143119	CD53	2.812461468	14.01281628	0.814233219	0	0	0.758763507	3.064183605	3.322625272	1.875849726	8.858885733	0	4.229126084
ENSG00000143126	CELSR2	687.646829	935.3554868	561.8209214	806.2560077	899.2038017	644.1902171	909.041136	860.5599455	1151.302769	870.0691345	813.9665856	1007.377833
ENSG00000143127	ITGA10	1.406230734	0	0	4.186168264	4.623155793	1.517527013	1.021394535	1.661312636	1.406887294	0.632777552	2.759208765	3.383300867
ENSG00000143147	GPR161	7.031153671	21.01922442	16.28466439	19.25637402	20.80420107	38.31755708	1.021394535	8.306563181	2.813774589	3.163887762	6.898021912	4.229126084
ENSG00000143149	ALDH9A1	597.648062	472.9325495	516.2238611	585.2263234	542.0650167	471.9509011	373.8303998	397.05372	412.2179772	424.5937376	440.093798	432.2166858
ENSG00000143153	ATP1B1	1321.85689	2017.845545	1628.466439	1991.77886	2444.493626	2096.842951	629.1790335	918.7058878	812.7118937	777.6836119	863.6323433	1014.144435
ENSG00000143155	TIPRL	963.2680529	1138.541323	847.6167814	1002.168682	1059.858466	1077.064798	894.7416126	885.4796351	912.1319292	975.7429858	844.317882	1029.369289
ENSG00000143156	NME7	217.9657638	297.772346	306.9659237	278.7988064	255.4293576	258.7383558	188.957989	280.7618355	194.6194091	217.675478	275.9208765	195.3856251
ENSG00000143157	POGK	2039.034565	1706.060382	1975.32979	2173.458563	1971.775946	2266.805976	2042.78907	1913.832157	2245.392122	2024.25539	1936.964553	2190.687311
ENSG00000143158	MPC2	392.3383748	620.0671205	423.4012741	623.7390714	601.0102531	563.3819037	820.1798115	1038.320398	1018.586401	839.0630345	982.2783202	972.6989993
ENSG00000143162	CREG1	3591.513295	3447.152805	3182.837655	3277.769751	3312.491126	3071.095293	2810.87776	2702.955659	2471.900976	2686.773487	2659.877249	2463.888856
ENSG00000143164	DCAF6	1441.386503	1562.429015	1136.669574	1434.181247	1695.542387	1192.017469	1751.691627	2051.721106	1616.513501	1671.798293	1796.244906	1643.438396
ENSG00000143167	GPA33	0	0	0.814233219	1.674467306	2.311577897	3.035054026	10.21394535	4.983937908	5.627549177	12.0227735	16.55525259	1.691650434
ENSG00000143178	TBX19	43.59315276	59.5544692	78.16638907	46.88508456	55.47786952	55.01035423	24.51346884	29.90362745	24.38604644	24.04554699	45.52694462	26.22058172
ENSG00000143179	UCK2	1051.860589	662.1055693	968.9375311	1501.997173	1109.55739	1292.553634	847.7574639	554.8784205	971.690158	1021.30297	673.2469386	1017.527736
ENSG00000143183	TMCO1	1425.917964	1681.537954	1450.963597	1590.74394	1567.249814	1553.947662	1461.615579	1619.77982	1378.749548	1468.676699	1556.193743	1522.48539
ENSG00000143184	XCL1	8.437384405	21.01922442	15.47043117	8.372336529	34.67366845	16.69279715	11.23533988	26.58100218	7.503398903	21.51443678	8.277626294	9.304077385
ENSG00000143190	POU2F1	2174.032715	2129.948075	2069.780844	2255.507461	2320.824208	2162.855376	1763.948362	1938.751846	1968.235325	1859.733226	1975.593475	2073.117606
ENSG00000143194	MAEL	29.53084542	3.50320407	9.770798633	5.023401917	9.246311586	12.14021611	4.08557814	8.306563181	7.503398903	5.062220419	2.759208765	2.53747565
ENSG00000143195	ILDR2	12.65607661	14.01281628	8.956565414	39.34998168	28.89472371	29.97115851	4.08557814	6.645250545	5.627549177	6.960553076	8.277626294	6.766601734
ENSG00000143198	MGST3	524.5240638	732.1696507	517.0380943	737.6028482	768.5996506	683.2665377	454.520568	687.7834314	515.3897122	549.883693	676.0061473	625.0648352
ENSG00000143199	ADCY10	5.624922937	3.50320407	6.513865756	3.348934611	6.93473369	6.828871559	11.23533988	21.59706427	13.59991051	10.75721839	11.03683506	9.304077385
ENSG00000143207	COP 1	1016.704821	921.3426705	966.4948315	1082.543113	954.6816713	957.1801636	1201.159973	1089.821089	1013.427814	1079.518504	1065.054583	1223.063263
ENSG00000143217	NECTIN4	821.2387487	1338.223955	861.4587462	1215.663264	1455.138286	1053.543129	1148.047457	1840.734401	1018.117439	1192.152909	1890.058004	1074.198025
ENSG00000143222	UFC1	708.74029	781.2145077	832.1463503	942.7250931	982.420606	853.2295632	743.5752214	970.2065795	719.8573323	786.5424976	853.9751126	839.9044403
ENSG00000143224	PPOX	108.2797665	101.592918	74.90945619	66.97869223	91.30732691	95.98358359	117.4603715	134.5663235	134.1232554	116.4310696	122.78479	71.89514343
ENSG00000143226	FCGR2A	7.031153671	38.53524477	27.68392946	0	0	0	20.4278907	29.90362745	7.972361335	1.898332657	6.898021912	0.845825217
ENSG00000143228	NUF2	802.9577492	585.0350798	717.3394663	1080.868646	894.580646	920.7595153	764.0031121	699.4126198	725.4848815	784.0113874	729.8107182	935.4826898
ENSG00000143248	RGS5	611.7103694	332.8043867	333.83562	635.4603425	394.1240314	275.0517711	56.17669942	63.12988017	74.5650266	58.84831237	78.63744979	53.28698866
ENSG00000143252	SDHC	1001.236283	1001.916364	1058.503185	1139.475002	1050.612154	1174.186526	905.9769524	951.9321405	962.3109093	872.6002447	1182.320956	1076.735501
ENSG00000143256	PFDN2	577.9608317	444.9069169	596.0187166	653.8794829	501.6124035	745.864527	312.5467277	257.5034586	412.6869397	327.7787721	291.0965247	422.9126084
ENSG00000143258	USP21	289.6835312	143.6313669	255.6692309	270.4264699	279.7009255	282.6394062	339.1029856	370.4727179	380.7974943	417.6331846	400.0852709	345.9425137
ENSG00000143278	F13B	4.218692202	0	0.814233219	4.186168264	0	0	0	0	0.468962431	0	4.138813147	3.383300867
ENSG00000143294	PRCC	787.4892111	613.0607123	836.2175164	791.185802	700.4081026	898.3759918	776.2598465	725.993622	847.4151136	786.5424976	762.9212234	911.7995837
ENSG00000143297	FCRL5	0	3.50320407	0.814233219	0	11.55788948	2.27629052	1.021394535	0	1.875849726	0.632777552	2.759208765	1.691650434
ENSG00000143303	RRNAD1	284.0586083	294.2691419	248.3411319	257.8679651	272.7661918	257.9795922	316.6323058	254.1808333	301.073881	338.5359905	339.382678	308.7262041
ENSG00000143314	MRPL24	676.3969831	564.0158553	679.070505	828.8613163	814.8312085	841.0893471	652.6711078	448.5544118	640.6026814	594.1781217	515.972039	760.3968699
ENSG00000143315	PIGM	368.4324523	409.8748762	374.5472809	486.4327523	448.4461119	514.8210392	603.6441701	360.504842	540.244721	534.6970318	427.6773585	636.9063882
ENSG00000143318	CASQ1	0	0	0	0	0	0	20.4278907	29.90362745	11.72406079	17.71777147	31.73090079	18.60815477
ENSG00000143319	ISG20L2	777.645596	826.7561606	889.1426756	1028.960159	731.6144043	1083.134906	872.2709328	832.3176307	1090.337653	963.0874347	877.4283871	1097.035306
ENSG00000143320	CRABP2	4.218692202	10.50961221	8.956565414	20.93084132	15.02525633	19.72785117	5.106972674	0	5.158586746	6.327775524	1.379604382	8.458252168
ENSG00000143321	HDGF	6624.752989	5864.363614	6421.857402	7940.323964	7710.268074	7540.591729	7724.806867	7389.518605	7988.77502	7953.381056	8000.325813	9022.417587
ENSG00000143322	ABL2	4182.130203	3758.937968	4463.626509	3070.973039	2972.689175	3738.427797	1442.209083	1669.619199	1687.326828	1363.635625	1625.173962	1649.359173
ENSG00000143324	XPR1	1425.917964	893.317038	1196.922833	932.6782893	945.4353597	876.3718501	3282.762035	3139.880882	3201.137557	2447.583573	2388.095186	2322.636045
ENSG00000143333	RGS16	0	3.50320407	6.513865756	10.88403749	12.71367843	6.070108053	11.23533988	13.29050109	10.31717349	3.163887762	6.898021912	5.074951301
ENSG00000143337	TOR1AIP1	843.7384405	1145.547731	872.043778	885.7932047	895.7364349	940.1079847	1599.503842	1197.806411	1493.645344	1653.447744	1349.253086	1792.303634
ENSG00000143341	HMCN1	8.437384405	3.50320407	6.513865756	6.697869223	8.090522638	9.484543833	8.171156279	6.645250545	5.627549177	0	0	0
ENSG00000143344	RGL1	36.56199909	24.52242849	21.98429693	22.60530863	18.49262317	24.28043221	41.87717593	21.59706427	22.51019671	29.74054496	44.14734023	24.52893129
ENSG00000143353	LYPLAL1	600.4605235	557.0094472	579.7340522	469.6880793	503.9239814	463.2251208	501.5047166	446.8930991	497.5691398	473.9503867	430.4365673	439.8291127
ENSG00000143355	LHX9	1.406230734	3.50320407	7.328098975	4.186168264	4.623155793	3.035054026	3.064183605	0	0	0	0	0
ENSG00000143363	PRUNE1	347.3389913	255.7338971	278.4677611	304.7530496	290.103026	314.1280917	420.8145484	340.5690904	433.7902491	412.5709642	442.8530067	416.9918319
ENSG00000143365	RORC	0	0	3.256932878	0.837233653	3.467366845	0	1.021394535	0	1.875849726	0	0	0

**ENSG00000143367**	TUFT1	824.0512102	1121.025303	1060.131652	1506.183342	1193.929984	1745.914829	1466.722552	1297.485169	1484.266096	1407.297276	1398.918844	1394.765782
**ENSG00000143368**	SF3B4	1747.944803	1537.906587	1826.325111	1610.000314	1510.616155	1867.696372	1324.748712	1329.050109	1473.948922	1428.811713	1408.576074	1468.352576
**ENSG00000143369**	ECM1	4182.130203	4501.61723	4001.14204	2463.97864	2750.777697	2620.769152	244.1132938	192.7122658	359.6941849	187.9349331	197.2834267	243.5976624
**ENSG00000143373**	ZNF687	528.742756	690.1312019	642.4300101	507.3635936	540.9092278	510.6478399	547.4674707	802.4140032	631.6923952	584.6864584	640.1364334	546.40309
**ENSG00000143374**	TARS2	646.8661377	434.3973047	623.7026461	581.0401551	614.8797205	669.2294128	598.5371974	481.7806645	649.5129676	645.4331034	554.6009617	582.7735744
**ENSG00000143375**	CGN	247.4966092	266.2435094	307.780157	344.1030313	417.2398103	377.8642263	193.0435671	186.0670152	170.702325	296.7726721	317.3090079	283.3514476
**ENSG00000143376**	SNX27	1357.012658	1250.643853	1294.630819	1496.136538	1519.862467	1551.671371	1390.117962	1312.436983	1487.548833	1391.477838	1375.465569	1648.513347
**ENSG00000143379**	SETDB1	815.6138258	746.182467	789.8062229	728.393278	677.2923237	675.2995209	953.9824956	975.1905174	893.3734319	992.8279797	965.7230676	879.6582254
**ENSG00000143382**	ADAMTSL4	3626.669063	2424.217217	2949.152721	2628.076436	2141.676921	2280.084337	3972.203346	2378.999695	3958.511884	3933.978043	2397.752416	3069.499712
**ENSG00000143384**	MCL1	9791.584602	11007.06719	10411.60018	6836.850009	5667.989002	6917.267508	6806.57318	7316.420849	7434.930388	4874.285486	4945.881711	5878.485257
**ENSG00000143387**	CTSK	49.2180757	87.58010176	64.32442434	28.4659442	52.01050267	40.21446585	51.06972674	56.48462963	37.98595695	44.29442867	53.80457091	22.83728085
**ENSG00000143390**	RFX5	3137.300768	3373.58552	3262.63251	1012.215486	1057.546888	1164.322601	1083.699602	1149.628344	893.3734319	522.6742583	569.7766099	540.4823135
**ENSG00000143393**	PI4KB	994.205129	868.7946095	1065.017051	983.7495421	973.1742944	1011.052373	1108.21307	1146.305719	1061.261982	1105.462384	1082.98944	1178.234527
**ENSG00000143398**	PIP5K1A	3635.106448	2658.931889	3438.506886	3398.331397	2286.15054	3163.285059	3408.393563	2746.149788	3001.359561	3230.329405	2644.701601	2969.692336
**ENSG00000143401**	ANP32E	3110.582384	2550.332563	2687.783857	3585.871735	3038.569145	3085.5118	2508.544978	2164.690365	2646.823963	2808.899555	2476.389866	3261.502036
**ENSG00000143409**	MINDY1	44.99938349	56.05126513	54.5536257	49.39678552	77.43785953	75.87635066	78.64737919	96.3561329	70.81332715	71.50386342	56.56377967	61.74524082
**ENSG00000143412**	ANXA9	70.31153671	70.06408141	73.28098975	129.7712162	122.5136285	142.2681575	113.3747934	111.3079466	93.32352386	132.2505084	124.1643944	114.1864043
**ENSG00000143418**	CERS2	2199.344868	2294.598666	2506.209849	2365.185069	2635.198802	2641.255767	1182.774871	1103.11159	1352.01869	1294.662872	1171.284121	1487.806556
**ENSG00000143420**	ENSA	2321.686942	2252.560217	2312.422343	2802.221036	2673.339837	2540.34022	2638.262084	2810.94098	2627.127541	2751.316798	3087.554608	2796.298167
**ENSG00000143429**	AC116050.1	94.21745919	80.57369362	87.12295448	106.3286739	100.5536385	123.6784516	130.7385005	114.6305719	93.79248629	113.8999594	80.01705417	104.8823269
**ENSG00000143434**	SEMA6C	23.90592248	45.54165292	29.3123959	36.83828073	57.78944741	29.212395	25.53486337	11.62918845	34.2342575	48.09109398	30.35129641	36.37048432
**ENSG00000143436**	MRPL9	652.4910606	465.9261414	740.1379965	853.9783259	831.0122538	836.9161478	628.1576389	636.2827396	663.1128781	613.1614483	700.8390262	763.7801707
**ENSG00000143437**	ARNT	961.8618222	938.8586909	819.932852	833.8847183	869.1532891	798.219209	1344.155208	1619.77982	1374.059924	1147.225702	1343.734668	1261.125398
**ENSG00000143442**	POGZ	1549.666269	1488.86173	1611.367541	1446.739752	1458.605653	1481.865128	1283.89293	1495.181373	1417.204468	1484.496138	1458.241832	1371.082676
**ENSG00000143443**	C1orf56	25.31215321	24.52242849	48.85399317	38.51274803	32.36209055	44.38766514	39.83438686	38.21019063	44.55143099	48.72387153	59.32298844	39.75378519
**ENSG00000143450**	OAZ3	14.06230734	14.01281628	17.09889761	18.41914036	11.55788948	16.31341539	23.4920743	4.983937908	12.19302322	24.67832454	15.17564821	30.4497078
**ENSG00000143452**	HORMAD1	64.68661377	66.56087734	68.39559043	73.67656145	112.111528	59.94231702	260.4556064	206.0027669	166.0127007	260.7043516	224.8755143	181.0065964
**ENSG00000143457**	GOLPH3L	651.0848299	914.3362624	653.8292752	515.7359302	743.1722937	588.8004811	626.1148499	729.3162473	653.264667	556.8442461	733.9495314	702.0349299
**ENSG00000143458**	GABPB2	939.3621304	644.589549	798.7627883	967.0048691	824.0775201	884.7182487	785.4523973	515.0069172	759.2501765	828.9385936	710.4962569	773.9300734
**ENSG00000143469**	SYT14	2213.407176	2056.380789	1971.258624	1793.354484	1591.521382	1786.129295	29.62044151	34.88756536	56.27549177	20.88165923	19.31446135	28.75805737
**ENSG00000143473**	KCNH1	2.812461468	0	1.628466439	5.86063557	6.93473369	7.208253313	0	0	3.751699452	0	0	0.845825217
**ENSG00000143476**	DTL	1627.008959	2753.518399	1719.660559	1497.811005	1998.359092	1541.048682	1348.240786	2015.172228	1607.134253	1609.153316	1863.84552	1575.772379
**ENSG00000143479**	DYRK3	268.5900702	227.7082646	392.4604118	127.2595152	218.4441112	188.1733496	1.021394535	6.645250545	9.379248629	6.960553076	9.657230676	13.53320347
**ENSG00000143486**	EIF2D	814.2075951	861.7882013	866.3441455	879.9325692	766.2880727	839.1924383	817.1156279	880.4956971	821.1532175	882.7246856	863.6323433	783.2341507
**ENSG00000143493**	INTS7	894.3627469	1152.554139	1029.190789	920.9570182	1104.934235	1060.751382	982.5815426	1169.564096	940.2696751	1003.585198	1260.958405	844.1335663
**ENSG00000143494**	VASH2	203.9034565	196.1794279	187.2736405	47.72231821	23.11577897	19.34846942	86.81853546	144.5341993	69.40643986	118.9621798	107.6091418	104.0365017
**ENSG00000143498**	TAF1A	347.3389913	241.7210809	297.1951251	210.1456469	198.7956991	227.2496702	266.5839736	201.018829	250.4259384	251.8454658	223.4959099	296.0388259
**ENSG00000143499**	SMYD2	877.4879781	910.8330583	767.0076927	947.748495	948.9027265	946.5574745	1567.840611	2189.610054	1594.472267	1524.993901	2095.619057	1700.954511
**ENSG00000143502**	SUSD4	35.15576835	24.52242849	20.35583049	16.74467306	18.49262317	26.55672273	0	0	0.468962431	0	0	0
**ENSG00000143507**	DUSP10	1285.294891	2655.428685	2072.223543	721.6954088	1540.666668	1330.112427	1087.78518	1684.571013	761.5949887	842.2269222	1183.70056	597.152603
**ENSG00000143512**	HHIPL2	11.24984587	17.51602035	4.071166097	4.186168264	3.467366845	0.758763507	0	0	5.158586746	15.18666126	8.277626294	4.229126084
**ENSG00000143514**	TP53BP2	2403.248325	4007.665457	2881.571364	2141.643684	2420.222058	2587.004176	631.2218226	1061.578774	674.3679764	646.065881	859.4935302	680.0434743
**ENSG00000143515**	ATP8B2	53.4367679	63.05767327	44.78282707	34.32657977	38.14103529	28.83301325	0	1.661312636	1.875849726	2.53111021	4.138813147	0
**ENSG00000143537**	ADAM15	1167.171509	1029.941997	1062.574351	1097.613319	1196.241561	1250.821641	1041.822426	1021.707271	1304.184522	1233.916227	1071.952605	1344.016269
**ENSG00000143543**	JTB	201.090995	157.6441832	153.8900785	219.3552171	165.2778196	193.8640759	153.2091802	137.8889488	197.4331836	152.4993901	176.5893609	210.610479
**ENSG00000143545**	RAB13	177.1850725	143.6313669	118.87805	192.5637402	198.7956991	148.7176473	150.1449966	136.2276362	125.6819316	164.5221636	117.2663725	132.794559
**ENSG00000143546**	S100A8	33.74953762	14.01281628	56.99632536	33.48934611	5.778944741	49.31962793	102.1394535	78.0816939	222.7571549	144.2732819	95.19270238	282.5056224
**ENSG00000143549**	TPM3	7955.047263	8947.183196	8117.905198	7315.747659	6752.119036	7490.133955	5247.92512	5286.296808	5510.30857	5527.31192	5279.745971	5729.620018
**ENSG00000143552**	NUP210L	30.93707615	14.01281628	14.65619795	14.2329721	9.246311586	6.449489806	3.064183605	3.322625272	2.344812157	5.694997971	12.41643944	2.53747565
**ENSG00000143553**	SNAPIN	278.4336854	343.3139989	279.2819943	365.0338727	256.5851465	313.74871	265.5625791	375.4566558	306.2324677	278.422123	373.8727876	296.0388259
**ENSG00000143554**	SLC27A3	172.9663803	84.07689769	153.0758453	253.6817968	239.2483123	158.9609546	352.3811145	342.230403	364.8527717	367.0109804	378.0116008	314.6469806
**ENSG00000143556**	S100A7	36.56199909	0	70.02405687	72.8393278	17.33683422	103.5712187	289.0546534	111.3079466	331.0874766	541.0248073	233.1531406	500.7285283
**ENSG00000143569**	UBAP2L	3611.200525	3079.316378	3457.23425	3851.274803	3551.739438	3817.718584	3368.559176	3390.73909	3439.839435	3515.079303	3589.730603	3576.994842
**ENSG00000143570**	SLC39A1	25.31215321	17.51602035	22.79853014	26.79147689	41.60840214	32.24744903	27.57765244	26.58100218	36.57906965	30.37332251	28.97169203	35.5246591
**ENSG00000143575**	HAX1	1217.795816	809.2401403	1154.582705	1064.961206	1039.054264	1197.328813	729.2756979	765.8651253	877.8976717	761.8641731	633.2384115	836.5211394
**ENSG00000143578**	CREB3L4	174.372611	304.7787541	195.4159727	166.6094969	157.187297	121.0227793	235.9421376	284.0844608	219.0054555	237.9243597	229.0143275	176.7774703
**ENSG00000143590**	EFNA3	11.24984587	49.04485699	17.09889761	19.25637402	62.41260321	18.58970591	11.23533988	24.91968954	6.096511609	12.0227735	64.84140597	9.304077385
**ENSG00000143603**	KCNN3	0	0	3.256932878	9.209570182	1.155788948	6.828871559	0	3.322625272	2.344812157	2.53111021	2.759208765	1.691650434
**ENSG00000143612**	C1orf43	2453.872631	2557.338971	2656.842995	2436.34993	2556.605154	2921.2395	1921.24312	1829.105212	2258.054107	1809.7438	1746.579148	2139.091973
**ENSG00000143614**	GATAD2B	1639.665036	1772.62126	1712.33246	1957.45228	2045.746438	1902.599493	1858.938053	2078.302108	1882.4152	1962.24319	2069.406573	1746.629073
**ENSG00000143621**	ILF2	2837.773622	2242.050605	2920.654558	3171.441077	2644.445114	2878.369362	2688.310416	2634.841841	3101.248559	2763.972349	2644.701601	3214.981649
**ENSG00000143622**	RIT1	388.1196826	388.8556518	403.8596768	404.3838543	367.5408855	364.5858649	317.6537003	451.877037	337.6529506	289.1793414	373.8727876	341.7133876
**ENSG00000143624**	INTS3	1944.817105	1849.691749	1818.997012	1758.190671	1726.748689	1715.184907	1895.708257	1677.925762	1636.209923	1862.897114	1794.865301	1577.464029
**ENSG00000143630**	HCN3	37.96822982	91.08330583	35.82626166	44.3733836	58.94523636	51.9753002	52.09112128	89.71088235	68.93747742	84.79219202	102.0907243	65.97436691
**ENSG00000143631**	FLG	16.87476881	28.02563256	30.94086234	30.97764516	23.11577897	22.38352344	0	3.322625272	0.937924863	1.265555105	0	6.766601734
**ENSG00000143632**	ACTA1	0	7.006408141	0.814233219	0	3.467366845	2.655672273	0	0	0.937924863	5.062220419	1.379604382	0
**ENSG00000143633**	C1orf131	314.9956845	189.1730198	228.7995347	293.8690122	290.103026	254.5651565	402.4294467	380.4405937	427.2247751	440.4131765	333.8642605	427.1417345

**ENSG00000143641**	GALNT2	158.904073	283.7595297	395.7173447	228.5647872	502.7681925	513.3035122	417.7503648	744.268061	478.8106425	534.0642542	776.7172672	548.9405657
**ENSG00000143643**	TTC13	371.2449138	455.4165292	308.5943902	401.8721534	366.3850966	398.350841	419.7931538	398.7150327	437.0729861	433.4526234	375.252392	440.6749379
**ENSG00000143653**	SCCPDH	757.9583657	550.0030391	656.2719749	1304.410031	723.5238816	1026.607024	1143.961879	1008.41677	1391.411534	1224.424564	1051.258539	1574.080728
**ENSG00000143669**	LYST	2891.210389	3286.005418	3130.726729	2036.152244	1916.298076	2288.810118	583.2162794	893.7861982	669.2093897	578.9914604	852.5955083	685.1184256
**ENSG00000143674**	MAP3K21	224.9969175	378.3460396	260.5546302	221.866918	344.4251066	337.6497604	182.8296217	179.4217647	92.385599	188.5677106	194.5242179	120.1071808
**ENSG00000143702**	CEP170	440.1502198	423.8876925	511.3384618	500.6657244	495.8334588	509.8890764	399.3652631	470.151476	378.4526822	442.9442867	506.3148083	516.7992075
**ENSG00000143727**	ACP1	1416.074349	1548.416199	1516.916488	1458.461023	1356.896225	1577.848712	1543.327142	1503.487936	1649.340871	1611.684426	1364.428734	1753.395674
**ENSG00000143740**	SNAP47	344.5265299	343.3139989	326.507521	437.8732004	394.1240314	410.4910571	372.8090052	393.7310948	417.376564	406.8759662	435.9549848	405.9961041
**ENSG00000143742**	SRP9	3435.421684	3604.796988	2962.180452	3965.975814	3639.579398	3597.677167	3116.274726	3179.752386	3065.607414	3195.52664	3283.45843	3630.28183
**ENSG00000143748**	NVL	869.0505937	826.7561606	810.1620533	991.284645	892.2690681	870.3017421	1257.336672	1342.34061	1101.123789	1163.677919	1331.318229	1108.031034
**ENSG00000143751**	SDE2	833.8948254	693.6344059	900.5419407	841.4198211	622.9702431	932.8997314	530.1037636	548.2331699	649.0440051	568.234242	379.3912051	631.8314369
**ENSG00000143753**	DEGS1	1206.54597	1078.986854	1156.211172	1356.318518	1536.043512	1357.048532	1936.564038	3232.91439	2203.185503	1757.856041	2619.868722	2099.338188
**ENSG00000143756**	FBXO28	1738.101187	1909.246218	1600.782509	1954.103346	1894.338086	1810.030345	1380.925411	1892.235093	1429.397491	1357.940627	1752.097566	1650.204998
**ENSG00000143761**	ARF1	3871.353211	3594.287376	3900.177121	3456.937753	3730.886725	4010.823896	2175.570359	2448.774826	2816.588363	2396.328591	2490.18591	2835.206127
**ENSG00000143771**	CNIH4	703.1153671	833.7625688	645.686943	680.6709598	730.4586153	722.3428583	708.8478072	777.4943137	645.2923057	616.9581136	789.1337067	776.467549
**ENSG00000143772**	ITPKB	92.81122845	73.56728548	103.4076189	113.8637768	108.6441611	103.5712187	20.4278907	34.88756536	23.44812157	25.94387965	40.00852709	36.37048432
**ENSG00000143774**	GUK1	1664.977189	1523.893771	1550.30005	1025.611225	993.9784955	1182.153543	774.2170574	867.2051961	967.9384585	419.5315172	524.2496653	502.4201788
**ENSG00000143776**	CDC42BPA	3130.269614	2473.262074	3359.526263	3757.504634	3762.093027	4291.566393	2352.271614	2166.351678	2985.883801	2463.403011	2339.809032	3284.339317
**ENSG00000143786**	CNIH3	14.06230734	10.50961221	15.47043117	15.07020575	8.090522638	9.105162079	9.192550814	8.306563181	16.4136851	9.491663286	5.518417529	10.1499026
**ENSG00000143793**	C1orf35	203.9034565	185.6698157	179.9455415	190.0520392	175.6799201	181.7238598	187.9365944	220.9545806	197.4331836	210.0821474	189.0058004	191.156499
**ENSG00000143797**	MBOAT2	3531.045373	2904.156174	3235.762814	4670.926549	3999.029761	4725.958501	841.6290967	1142.983094	1310.281033	798.5652711	1254.060384	1519.102089
**ENSG00000143799**	PARP1	3812.29152	3075.813174	3721.045813	3933.323701	4000.18555	3956.572305	4260.236605	4115.0714	4536.273599	4240.875156	4381.623518	4860.111696
**ENSG00000143801**	PSEN2	94.21745919	31.52883663	88.75142092	66.97869223	58.94523636	67.90933384	37.79159779	36.54887799	29.07567075	28.47498986	23.4532745	47.36621214
**ENSG00000143811**	PYCR2	404.9944514	420.3844885	424.2155073	351.6381342	338.6461618	372.1735	385.0657396	300.6975871	418.7834513	418.2659621	313.1701948	383.1588232
**ENSG00000143815**	LBR	1355.606428	1082.490058	1166.796203	1493.624837	1291.016255	1495.902253	1324.748712	1167.902783	1435.02504	1484.496138	1233.366318	1654.434124
**ENSG00000143816**	WNT9A	208.1221487	252.2306931	276.0250614	322.3349564	338.6461618	439.7034521	106.2250316	126.2597603	179.1436488	162.623831	136.5808338	161.5526164
**ENSG00000143819**	EPHX1	28.12461468	70.06408141	36.64049488	51.90848648	80.90522638	68.66809735	42.89857046	41.5328159	55.80652934	66.441643	53.80457091	60.89941561
**ENSG00000143839**	REN	7.031153671	0	8.956565414	9.209570182	2.311577897	10.24330734	0	0	0	0	0	4.229126084
**ENSG00000143842**	SOX13	2137.470716	1727.079607	2187.844661	2044.52458	1604.23506	2140.471852	1966.18448	1400.486552	1811.13291	1670.532738	1231.986713	1475.965003
**ENSG00000143845**	ETNK2	87.18630552	112.1025303	103.4076189	113.8637768	128.2925733	175.6537518	5.106972674	6.645250545	6.56547404	0.632777552	12.41643944	16.07067912
**ENSG00000143847**	PPFIA4	26.71838395	444.9069169	36.64049488	23.44254228	338.6461618	29.59177676	42.89857046	1103.11159	26.26189616	39.23220825	831.9014425	27.91223215
**ENSG00000143850**	PLEKHA6	1288.107352	840.7689769	1209.136331	1978.383122	1036.742687	1579.745621	1320.663134	458.5222876	1115.192662	1334.527858	597.3686975	1048.823269
**ENSG00000143862**	ARL8A	133.5919197	231.2114686	171.8032093	149.0275902	176.8357091	182.1032416	30.64183605	38.21019063	30.01359561	56.31720216	67.60061473	43.98291127
**ENSG00000143867**	OSR1	49.2180757	42.03844885	33.383562	30.97764516	38.14103529	33.76497604	126.6529223	182.74439	120.5233449	133.5160636	217.9774924	104.0365017
**ENSG00000143870**	PDIA6	10698.60343	10835.41019	11202.22063	6406.511912	6749.807458	7025.770689	5390.920355	6555.539662	5749.948372	4882.511594	5500.482672	5706.782738
**ENSG00000143878**	RHOB	247.4966092	483.4421617	652.2008088	191.7265065	266.987247	374.8291723	49.02693767	78.0816939	46.42728071	73.40219608	95.19270238	46.52038692
**ENSG00000143882**	ATP6V1C2	16.87476881	35.0320407	17.91313083	18.41914036	10.40210053	6.449489806	18.38510163	4.983937908	3.751699452	3.796665314	2.759208765	1.691650434
**ENSG00000143889**	HNRNPLL	516.0866794	434.3973047	468.1841012	538.3412388	568.6481625	564.8994307	559.7242051	712.7031209	579.1686028	587.2175686	633.2384115	624.21901
**ENSG00000143891**	GALM	21.09346101	28.02563256	49.66822639	53.58295378	68.19154795	38.31755708	25.53486337	34.88756536	26.73085859	38.5994307	33.11050518	21.99145564
**ENSG00000143919**	CAMKMT	73.12399818	87.58010176	74.09522297	89.58400086	102.8652164	144.9238298	123.5887387	91.37219499	112.5509835	111.3688492	144.8584601	131.9487338
**ENSG00000143924**	EML4	1307.794583	1233.127833	1205.879398	1363.853621	1245.940486	1335.423772	1924.307304	1543.359439	2491.597398	2066.018709	1678.978533	2781.919138
**ENSG00000143933**	CALM2	8029.577492	6337.296163	7837.80897	9471.624315	8522.787705	10014.54014	4449.194594	3428.949281	4889.40231	4402.233432	3718.03381	5643.345846
**ENSG00000143942**	CHAC2	158.904073	66.56087734	83.05178838	159.9116277	110.955739	131.2660866	42.89857046	34.88756536	56.27549177	61.37942258	16.55525259	69.35766778
**ENSG00000143947**	RPS27A	9103.937773	9318.522827	7895.619529	9097.380872	8637.21081	7687.412467	7556.276769	8746.811029	7259.538439	7616.11062	8032.056714	8653.637793
**ENSG00000143951**	WDPCP	113.9046895	175.1602035	106.6645517	191.7265065	162.9662417	178.6888058	249.2202665	201.018829	214.7847936	206.9182596	246.9491844	263.0516424
**ENSG00000143952**	VPS54	923.8935923	959.8779153	850.8737143	722.5326424	753.5743943	780.7676483	786.4737919	676.1542429	788.3258473	740.3497363	635.9976202	688.5017265
**ENSG00000143970**	ASXL2	2607.151781	2609.887032	2821.318105	2573.656249	2857.11028	2688.299104	2393.127395	2684.68122	2415.625484	2264.078082	2600.554261	2530.709049
**ENSG00000143971**	ETAA1	517.4929102	588.5382838	500.75343	680.6709598	570.9597404	540.6189985	654.7138969	506.700354	530.8654724	627.715332	554.6009617	582.7735744
**ENSG00000143977**	SNRPG	1389.355965	1201.598996	1115.499511	1474.368463	1207.799451	1225.782445	846.7360694	958.577391	1040.627635	953.5957714	975.3802983	1074.198025
**ENSG00000143994**	ABHD1	1.406230734	0	5.699632536	3.348934611	0	3.41443578	0	0	0.937924863	0.632777552	0	0.845825217
**ENSG00000143995**	MEIS1	92.81122845	133.1217547	140.0481137	112.1893095	109.7999501	113.814526	158.3161529	139.5502614	167.8885505	166.4204963	158.654504	174.2399947
**ENSG00000144021**	CIAO1	1134.828202	924.8458746	1139.112274	1061.612272	914.2290581	1151.04424	936.6187885	824.0110675	1035.938011	959.923547	769.8192453	1008.223658
**ENSG00000144026**	ZNF514	500.6181414	448.410121	431.5436063	482.2465841	508.5471372	435.150871	545.4246816	613.0243627	546.3412326	629.6136646	662.2101035	566.7028952
**ENSG00000144028**	SNRNP200	4699.623114	4585.694128	5071.858724	5006.657244	5303.915484	5264.301209	5461.396578	5626.865899	5708.210716	5574.137459	6033.009964	5972.371856
**ENSG00000144029**	MRPS5	977.3303602	1029.941997	920.083538	1143.66117	1025.184797	981.081214	607.7297483	805.7366285	651.3888173	667.5803178	666.3489167	664.8186204
**ENSG00000144034**	TPRKB	369.8386831	378.3460396	466.5556347	507.3635936	468.094524	421.1137462	366.680638	473.4741013	401.4318413	392.95486	358.6971394	395.8462015
**ENSG00000144036**	EXOC6B	1897.00526	1741.092423	1884.13567	1995.965028	1953.283323	2008.826384	3602.458525	2943.845991	3428.584336	3413.202118	3193.784145	3516.095426
**ENSG00000144040**	SFXN5	147.6542271	297.772346	175.8743754	166.6094969	180.3030759	147.2001203	142.9952349	212.6480174	162.2610013	169.584384	238.6715581	181.0065964
**ENSG00000144043**	TEX261	362.8075294	423.8876925	438.0574721	610.3433329	560.5576399	669.9881763	591.3874357	568.1689216	677.181751	667.5803178	689.8021912	698.6516291
**ENSG00000144045**	DQX1	15.46853808	21.01922442	20.35583049	17.58190671	17.33683422	11.3814526	16.34231256	13.29050109	8.441323766	12.0227735	8.277626294	16.07067912
**ENSG00000144048**	DUSP11	568.1172166	595.544692	614.7460807	632.1114079	547.8439615	559.5880861	655.7352914	764.2038126	564.6307675	618.2236687	775.3376629	568.3945457
**ENSG00000144061**	NPHP1	101.2486129	126.1153465	80.60908873	87.90953355	143.3178296	74.7382054	87.83993	111.3079466	72.22021444	96.18218796	108.9887462	88.81164776
**ENSG00000144063**	MALL	327.6517611	297.772346	222.2856689	323.17219	291.258815	331.2002706	30.64183605	23.25837691	41.26869397	17.71777147	27.59208765	30.4497078
**ENSG00000144115**	THNSL2	223.5906867	329.3011826	293.9381922	170.7956652	256.5851465	199.5548022	283.9476807	313.9880882	230.2605538	242.3538026	336.6234693	190.3106738
**ENSG00000144118**	RALB	1326.075582	1513.384158	1443.635498	1265.060049	1485.188799	1317.592829	632.2432171	923.6898257	594.6443631	618.8564462	739.4679489	685.1184256
**ENSG00000144120**	TMEM177	101.2486129	84.07689769	88.75142092	102.9797393	75.12628164	94.46605657	67.4120393	58.14594226	93.79248629	98.08052062	81.39665856	71.04931821
**ENSG00000144130**	NT5DC4	137.8106119	206.6890402	129.4630819	108.8403749	119.0462617	131.2660866	14.29952349	14.95181373	14.06887294	4.429442867	9.657230676	20.2998052

**ENSG00000144134**	RABL2A	87.18630552	80.57369362	68.39559043	78.69996337	129.4483622	67.15057033	174.6584655	192.7122658	162.2610013	153.7649452	177.9689653	124.3363069
**ENSG00000144136**	SLC20A1	8244.730794	4883.466474	6511.423056	6636.751166	4915.570397	6371.716547	1862.002237	1352.308486	2245.861084	1938.83042	1509.287194	2101.029838
**ENSG00000144152**	FBLN7	85.78007478	66.56087734	85.49448804	129.7712162	115.5788948	89.53409378	99.07526988	131.2436983	58.1513415	82.26108181	106.2295374	61.74524082
**ENSG00000144158**	AL078621.1	1.406230734	0	5.699632536	1.674467306	5.778944741	1.896908767	4.08557814	0	0.468962431	0.632777552	2.759208765	2.53747565
**ENSG00000144161**	ZC3H8	178.5913032	140.1281628	185.645174	179.1680017	142.1620406	162.7547722	233.8993485	215.9706427	205.8745074	290.4448965	205.561053	241.906012
**ENSG00000144182**	LIPT1	149.0604578	94.5865099	139.2338805	89.58400086	68.19154795	74.35882365	86.81853546	64.79119281	108.3303217	76.56608384	62.0821972	60.05359039
**ENSG00000144199**	FAHD2B	59.06169083	66.56087734	58.6247918	59.44358935	64.7241811	62.97737105	93.96829721	61.46856754	69.40643986	85.42496957	85.5354717	71.89514343
**ENSG00000144214**	LYG1	21.09346101	14.01281628	17.91313083	15.07020575	20.80420107	13.27836137	16.34231256	43.19412854	20.16538455	24.04554699	20.69406573	9.304077385
**ENSG00000144218**	AFF3	0	3.50320407	7.328098975	6.697869223	9.246311586	4.931962793	234.920743	255.842146	226.5088544	255.6421312	284.1985028	258.8225163
**ENSG00000144224**	UBXN4	2718.244009	3121.354827	2559.135009	1678.653474	1844.639161	1802.063328	2358.399981	2447.113513	2086.88282	1984.390404	2010.083585	2164.46673
**ENSG00000144228**	SPOPL	420.4629895	497.454978	541.4650909	481.4093504	444.9787451	622.9448389	508.6544784	518.3295425	500.8518768	527.1037011	460.7878637	613.2232822
**ENSG00000144229**	THSD7B	606.0854464	294.2691419	381.0611467	216.8435161	109.7999501	112.6763807	0	0	0	0.632777552	0	0
**ENSG00000144230**	GPR17	0	0	0	4.186168264	1.155788948	3.793817533	0	0	0	0	0	0
**ENSG00000144231**	POLR2D	833.8948254	861.7882013	854.1306472	900.0261768	759.353339	982.9781228	694.5482837	554.8784205	731.1124306	758.7002853	539.4253135	822.1421107
**ENSG00000144233**	AMMECR1L	482.3371418	388.8556518	636.7303776	558.4348465	486.5871472	617.6334944	486.1837986	426.9573475	532.2723597	485.3403827	557.3601705	485.5036744
**ENSG00000144278**	GALNT13	9.843615139	38.53524477	13.84196473	10.04680383	16.18104528	6.449489806	5.106972674	3.322625272	5.158586746	5.062220419	0	1.691650434
**ENSG00000144283**	PKP4	1411.855657	1334.720751	1481.090226	1801.726821	1733.683422	1746.29421	1204.224157	1106.434216	1294.805273	1240.244003	1143.692033	1487.806556
**ENSG00000144285**	SCN1A	1.406230734	14.01281628	5.699632536	8.372336529	4.623155793	9.484543833	0	1.661312636	0.468962431	0	0	0
**ENSG00000144306**	SCRN3	244.6841477	259.2371012	214.1433367	257.8679651	282.0125034	237.8723593	349.3169309	345.5530283	351.2528612	353.7226518	401.4648753	366.2423189
**ENSG00000144320**	LNPK	412.0256051	735.6728548	473.8837337	678.1592588	965.0837718	719.3078043	795.6663427	991.8036438	690.3126991	835.2663691	1047.119726	819.6046351
**ENSG00000144331**	ZNF385B	29.53084542	14.01281628	41.52589419	51.07125282	35.8294574	25.41857747	23.4920743	1.661312636	15.47576024	22.77999189	4.138813147	16.91650434
**ENSG00000144354**	CDCA7	324.8392996	360.8300193	232.8707008	288.8456102	283.1682923	312.9899465	265.5625791	136.2276362	283.2533086	275.2582353	195.9038223	262.2058172
**ENSG00000144355**	DLX1	5.624922937	7.006408141	4.885399317	6.697869223	2.311577897	4.173199286	2.04278907	1.661312636	7.034436472	2.53111021	1.379604382	2.53747565
**ENSG00000144357**	UBR3	890.1440547	763.6984874	859.8302797	1033.146328	1044.833209	1014.087427	1111.277254	1154.612282	1074.861893	1144.061815	1029.184869	1170.6221
**ENSG00000144362**	PHOSPHO2	53.4367679	119.1089384	27.68392946	36.83828073	49.69892478	36.42064832	69.45482837	28.24231481	76.90983876	60.11386748	63.46180159	52.44116344
**ENSG00000144366**	GULP1	1342.950351	1345.230363	1363.026409	795.3719702	940.8122039	996.6358659	654.7138969	594.7499237	617.6235222	590.3814564	560.1193792	521.8741588
**ENSG00000144369**	FAM171B	14.06230734	24.52242849	26.86969624	26.79147689	76.28207059	50.45777319	179.7654381	54.82331699	83.94427523	244.2521352	103.4703287	160.7067912
**ENSG00000144381**	HSPD1	6621.940527	4774.867148	6241.097627	6930.620178	6047.087777	6704.813726	5033.432268	3644.919924	5794.499803	5068.548195	3924.974468	6731.077075
**ENSG00000144395**	CCDC150	289.6835312	154.1409791	292.3097258	437.0359668	349.0482624	388.1075336	283.9476807	373.7953431	290.7567075	356.253762	355.9379306	290.9638746
**ENSG00000144401**	METTL21A	149.0604578	136.6249587	171.8032093	161.586095	147.9409854	169.204262	125.6315278	79.74300653	129.4336311	105.0410737	126.9236032	123.4904816
**ENSG00000144407**	PTH2R	347.3389913	266.2435094	315.1082559	252.0073295	336.3345839	223.8352344	424.9001265	392.0697821	352.190786	389.7909723	295.2353378	317.1844563
**ENSG00000144410**	CPO	12.65607661	3.50320407	2.442699658	2.511700959	6.93473369	2.27629052	4.08557814	8.306563181	8.910286198	1.265555105	1.379604382	4.229126084
**ENSG00000144426**	NBEAL1	1181.233817	1415.294444	1161.096571	1131.939899	1271.367843	1022.813207	1270.614801	1432.051492	1095.49624	1235.181782	1376.845174	1272.121126
**ENSG00000144445**	KANSL1L	165.9352266	248.727489	197.0444391	131.4456835	216.1325333	170.3424072	203.2575124	257.5034586	190.3987472	137.9455064	219.3570968	193.6939746
**ENSG00000144451**	SPAG16	184.2162262	325.7979785	215.7718032	259.5424324	249.6504128	212.0744001	447.3708063	436.9252233	347.0321993	389.1581947	349.0399087	362.859018
**ENSG00000144452**	ABCA12	2245.750482	1751.602035	1837.724376	2566.121146	1885.091775	2187.135808	1969.248663	1430.39018	1564.458671	1886.942661	1259.578801	1477.656654
**ENSG00000144455**	SUMF1	163.1227652	178.6634076	219.028736	272.9381708	279.7009255	283.018788	431.0284937	443.5704738	416.4386391	502.4253766	544.943731	477.8912475
**ENSG00000144460**	NYAP2	1.406230734	7.006408141	0.814233219	1.674467306	5.778944741	4.931962793	0	0	0	0.632777552	0	0.845825217
**ENSG00000144468**	RHBDD1	717.1776744	479.9389576	634.2876779	521.5965657	469.250313	496.2313333	693.5268892	586.4433606	738.6158295	531.533144	478.7227207	511.7242561
**ENSG00000144476**	ACKR3	0	0	0	0	0	0	83.75435186	94.69482026	81.59946307	94.91663286	88.29468047	98.11572515
**ENSG00000144481**	TRPM8	4.218692202	24.52242849	9.770798633	0	5.778944741	6.070108053	0	1.661312636	0	0	1.379604382	0.845825217
**ENSG00000144485**	HES6	40.78069129	24.52242849	39.89742775	48.55955187	50.85471372	39.07632059	24.51346884	11.62918845	32.8273702	33.53721028	27.59208765	43.98291127
**ENSG00000144488**	ESPNL	0	0	0	0	0	1.517527013	3.064183605	0	0	0	5.518417529	0.845825217
**ENSG00000144504**	ANKMY1	188.4349184	206.6890402	182.3882412	190.0520392	164.1220307	170.3424072	327.8676457	255.842146	314.6737915	356.8865395	282.8188984	231.7561094
**ENSG00000144524**	COPS7B	357.1826065	371.3396315	290.6812593	363.3594053	295.8819708	313.74871	343.1885637	330.6012146	327.3357772	387.8926396	372.4931832	324.7968832
**ENSG00000144535**	DIS3L2	267.1838395	241.7210809	210.0721706	245.3094603	220.7556891	237.1135958	275.7765244	260.8260839	259.805187	315.7559986	311.7905904	268.1265937
**ENSG00000144554**	FANCD2	1074.360281	1180.579772	1065.017051	1249.15261	1267.900476	1138.904023	1713.90003	1943.735784	1737.505809	1829.359904	1901.094839	1997.839162
**ENSG00000144559**	TAMM41	203.9034565	122.6121425	202.7440716	218.5179834	171.0567643	192.7259307	324.8034621	202.6801416	282.3153837	299.9365598	275.9208765	252.9017398
**ENSG00000144560**	VGLL4	423.275451	539.4934268	503.1961296	440.3849014	513.170293	495.0931881	761.960323	905.4153867	815.5256683	816.9158201	1016.76843	737.559589
**ENSG00000144566**	RAB5A	914.0499772	980.8971397	937.1824356	751.8358203	904.9827465	884.7182487	1213.416707	1189.499847	1110.034075	1185.825133	1194.737395	1234.058991
**ENSG00000144567**	RETREG2	690.4592905	721.6600385	653.015042	710.8113713	862.2185554	751.934635	703.7408345	656.2184913	694.0643986	711.2419689	677.3857517	699.4974543
**ENSG00000144579**	CTDSP1	444.368912	819.7497525	500.75343	426.1519293	748.9512385	566.4169577	691.4841001	1129.692593	902.7526805	773.254169	1149.21045	708.8015317
**ENSG00000144580**	CNOT9	939.3621304	970.3875275	977.8940966	1049.053767	790.5596406	964.7677987	844.6932803	828.9950054	976.3797823	842.8596998	852.5955083	989.6155036
**ENSG00000144583**	4-Mar	1787.319263	889.8138339	1582.869379	2105.642637	1545.289824	2169.684247	149.1236021	84.72694444	258.8672622	117.0638472	91.05388923	286.7347485
**ENSG00000144589**	STK11IP	202.4972257	192.6762239	161.2181775	203.4477776	188.3935986	248.8744302	272.7123408	295.7136492	309.9841672	315.1232211	273.1616677	245.2893129
**ENSG00000144591**	GMPPA	400.7757592	395.86206	495.8680306	292.1945449	371.0082524	411.6292023	294.161626	323.955964	331.556439	353.7226518	230.3939318	354.4007658
**ENSG00000144596**	GRIP2	163.1227652	308.2819582	192.1590398	0	12.71367843	6.070108053	30.64183605	147.8568246	36.57906965	1.898332657	2.759208765	0
**ENSG00000144597**	EAF1	715.7714437	732.1696507	837.0317496	665.600754	628.7491879	789.1140469	589.3446466	563.1849836	662.1749532	589.7486788	535.2865003	603.0733796
**ENSG00000144635**	DYNC1LI1	468.2748345	444.9069169	555.3070557	526.6199677	520.1050267	567.9344847	498.440533	603.0564869	548.2170824	470.1537214	504.9352039	558.2446431
**ENSG00000144642**	RBMS3	0	0	0	0	0	0	5.106972674	3.322625272	2.813774589	2.53111021	1.379604382	2.53747565
**ENSG00000144645**	OSBPL10	1832.318647	1678.03475	2041.282681	1918.102299	1657.401352	2017.931546	1038.758242	1184.51591	1175.219853	866.9052468	1058.156561	982.0030767
**ENSG00000144647**	POMGNT2	122.3420739	115.6057343	165.2893435	200.9360767	165.2778196	209.4187278	76.60459012	81.40431917	77.37880119	84.15941447	103.4703287	110.8031034
**ENSG00000144648**	ACKR2	262.9651473	105.0961221	240.1987997	154.8882258	97.08627165	177.1712788	795.6663427	908.738012	652.7957046	644.1675483	855.354717	466.8955197
**ENSG00000144649**	FAM198A	77.34269038	105.0961221	94.45105346	56.09465474	98.2420606	87.25780326	66.39064477	89.71088235	44.08246856	44.29442867	107.6091418	27.91223215
**ENSG00000144655**	CSRNP1	167.3414574	388.8556518	156.3327781	131.4456835	211.5093775	119.5052523	226.7495867	425.2960348	255.5845251	177.8104922	485.6207426	213.1479546
**ENSG00000144659**	SLC25A38	209.5283794	136.6249587	186.4594073	195.0754411	187.2378096	212.0744001	300.2899933	292.391024	297.791144	327.1459946	353.1787219	296.8846511
**ENSG00000144668**	ITGA9	2.812461468	0	0	0	1.155788948	0	6.128367209	9.967875817	2.813774589	1.265555105	0	0

**ENSG00000144671**	SLC22A14	1.406230734	0	8.142332194	1.674467306	5.778944741	2.655672273	6.128367209	6.645250545	1.875849726	5.694997971	0	1.691650434
**ENSG00000144674**	GOLGA4	2581.839628	2736.002379	3044.418008	3041.669861	3021.232311	2750.517711	3547.30322	3168.123197	3225.992566	3349.924362	3471.084626	3806.213475
**ENSG00000144677**	CTDSPL	1534.197731	1243.637445	1288.116953	1560.603529	1630.818206	1666.624042	1923.285909	1486.874809	2457.832103	1828.727126	1335.457042	2264.274105
**ENSG00000144681**	STAC	97.02992066	234.7146727	161.2181775	166.6094969	226.5346339	289.4682778	0	0	0.468962431	0	0	0
**ENSG00000144711**	IQSEC1	573.7421395	630.5767327	641.6157769	672.2986233	862.2185554	765.9717599	255.3486337	154.5020752	276.2188721	260.071574	201.4222398	256.2850407
**ENSG00000144712**	CAND2	37.96822982	56.05126513	48.03975995	30.97764516	25.42735686	29.59177676	10.21394535	11.62918845	11.72406079	15.81943881	15.17564821	7.612426951
**ENSG00000144713**	RPL32	4620.874192	4123.271191	4291.009066	4823.303074	4430.139039	4872.020476	5119.229409	6040.532745	5983.0227	5500.102485	5586.018144	5874.256131
**ENSG00000144724**	PTPRG	143.4355349	87.58010176	195.4159727	134.7946181	109.7999501	208.2805826	142.9952349	285.7457734	460.0521453	160.0927208	339.382678	444.0582388
**ENSG00000144730**	IL17RD	52.03053716	80.57369362	83.8660216	51.90848648	83.21680428	127.0928874	11.23533988	8.306563181	12.66198565	6.327775524	8.277626294	3.383300867
**ENSG00000144736**	SHQ1	275.6212239	192.6762239	254.8549977	308.1019843	257.7409355	298.5734399	384.0443451	436.9252233	442.7005353	387.2598621	431.8161717	499.8827031
**ENSG00000144741**	SLC25A26	136.4043812	133.1217547	112.3641843	128.0967489	125.9809954	154.4083736	296.2044151	204.3414542	194.1504466	217.675478	224.8755143	223.2978572
**ENSG00000144744**	UBA3	850.7695942	721.6600385	766.1934595	915.9336162	1003.224807	819.8439689	949.8969174	950.2708279	890.5596573	971.9463205	929.8533537	1025.985988
**ENSG00000144746**	ARL6IP5	511.8679872	823.2529565	486.097232	656.3911838	847.1932991	630.9118557	1171.539532	1026.691209	1007.331303	1161.779586	1145.071637	1214.605011
**ENSG00000144747**	TMF1	1178.421355	1625.486689	1214.02173	1156.219675	1427.399351	1171.530854	1643.423807	1576.585692	1474.417884	1512.971128	1709.32983	1695.87956
**ENSG00000144749**	LRIG1	25.31215321	77.07048955	78.98062229	25.95424324	102.8652164	129.3691779	29.62044151	81.40431917	60.96511609	47.45831643	66.22101035	92.19494863
**ENSG00000144791**	LIMD1	961.8618222	1040.451609	1069.088217	1181.336684	1336.092024	1158.252493	1816.039483	4643.368818	2116.427453	1632.566085	4178.821674	1872.65703
**ENSG00000144802**	NFKBIZ	840.925979	1057.967629	1140.74074	499.8284908	372.1640413	522.0292925	367.7020326	451.877037	392.0525927	310.6937782	338.0030737	305.3429033
**ENSG00000144810**	COL8A1	1011.079898	679.6215897	386.7607792	639.6465108	359.4503629	220.8001804	2.04278907	11.62918845	32.35840777	3.796665314	23.4532745	23.68310607
**ENSG00000144815**	NXPE3	182.8099954	140.1281628	162.0324107	102.9797393	156.031508	99.01863761	171.5942819	182.74439	98.48211061	112.0016268	124.1643944	99.80737558
**ENSG00000144821**	MYH15	403.5882207	1131.534915	876.1149441	1027.285692	1989.11278	1506.524942	150.1449966	353.8595915	231.6674411	227.1671413	329.7254474	470.2788205
**ENSG00000144824**	PHLDB2	14741.51679	16815.37954	16030.62362	11089.15973	10293.45637	11561.27955	10299.74249	11411.5565	10361.25596	9497.358284	11005.10416	10245.48085
**ENSG00000144827**	ABHD10	390.9321441	367.8364274	395.7173447	450.4317052	501.6124035	515.9591845	592.4088302	714.3644335	559.9411432	574.5620176	740.8475533	794.2298786
**ENSG00000144837**	PLA1A	0	0	0.814233219	0	0	0.758763507	0	8.306563181	4.220661883	2.53111021	0	0
**ENSG00000144840**	RABL3	237.6529941	280.2563256	280.0962275	269.5892362	291.258815	261.0146463	279.8621026	269.1326471	303.4186932	254.3765761	235.9123494	285.0430981
**ENSG00000144843**	ADPRH	33.74953762	42.03844885	41.52589419	34.32657977	39.29682424	29.212395	27.57765244	99.67875817	15.00679781	17.08499391	31.73090079	10.1499026
**ENSG00000144847**	IGSF11	22.49969175	21.01922442	21.17006371	20.09360767	12.71367843	19.72785117	8.171156279	14.95181373	15.47576024	8.858885733	5.518417529	10.1499026
**ENSG00000144848**	ATG3	874.6755166	1050.961221	991.7360613	1043.193131	990.5111287	1010.293609	1179.710688	1066.562712	1331.384343	1269.984548	1273.374845	1545.322671
**ENSG00000144852**	NR1I2	0	7.006408141	4.071166097	0	4.623155793	1.517527013	0	0	0	0	0	0.845825217
**ENSG00000144857**	BOC	0	0	3.256932878	1.674467306	2.311577897	1.517527013	837.5435186	749.2519989	695.9402483	956.1268816	885.7060134	792.5382281
**ENSG00000144867**	SRPRB	763.5832886	486.9453658	894.8423082	1175.476049	880.7111786	1247.407205	821.201206	584.7820479	815.0567059	947.2679959	663.5897079	1105.493558
**ENSG00000144868**	TMEM108	8.437384405	10.50961221	1.628466439	5.86063557	11.55788948	5.311344546	0	0	0	0	0	0.845825217
**ENSG00000144891**	AGTR1	0	0	0	1.674467306	0	0	0	3.322625272	1.875849726	1.898332657	4.138813147	3.383300867
**ENSG00000144893**	MED12L	7.031153671	7.006408141	18.72736405	12.55850479	16.18104528	21.24537819	33.70601965	13.29050109	36.11010722	46.82553888	31.73090079	49.90368779
**ENSG00000144895**	EIF2A	1972.94172	1537.906587	1775.028418	1649.350296	1474.786698	1688.628184	1516.770884	1307.453045	1393.287384	1341.488411	1295.448515	1547.860147
**ENSG00000144908**	ALDH1L1	205.3096872	94.5865099	221.4714357	251.1700959	220.7556891	233.3197783	78.64737919	53.16200436	71.75125201	75.93330629	40.00852709	96.42407471
**ENSG00000144909**	OSBPL11	635.6162918	655.0991612	668.4854732	818.8145125	844.8817212	843.7450193	956.0252846	948.6095152	940.2696751	1032.060188	860.8731346	1092.80618
**ENSG00000144935**	TRPC1	49.2180757	112.1025303	24.42699658	91.25846816	99.39784955	74.7382054	199.1719343	284.0844608	165.5437383	239.1899148	271.7820633	208.0730033
**ENSG00000144959**	NCEH1	1473.729809	1415.294444	1597.525577	1802.564055	1254.031009	1968.232536	735.4040651	809.0592538	913.5388165	697.3208627	590.4706756	987.078028
**ENSG00000145002**	FAM86B2	11.24984587	14.01281628	3.256932878	7.535102876	10.40210053	11.76083435	1.021394535	0	4.689624315	6.327775524	8.277626294	4.229126084
**ENSG00000145012**	LPP	3469.171221	3478.681642	3361.15473	2965.481598	3099.825959	3175.045893	1996.826316	1634.731634	1793.312338	1712.296057	1338.216251	1573.234903
**ENSG00000145014**	TMEM44	108.2797665	234.7146727	153.8900785	159.9116277	196.4841212	153.6496101	67.4120393	74.75906863	45.02039342	64.54331034	82.77626294	43.98291127
**ENSG00000145016**	RUBCN	1380.918581	1191.089384	1419.208501	1100.962254	863.3743444	1207.572121	615.9009045	702.7352451	640.6026814	530.2675889	645.6548509	523.5658092
**ENSG00000145020**	AMT	1.406230734	0	2.442699658	2.511700959	1.155788948	0.379381753	0	6.645250545	2.344812157	2.53111021	0	0
**ENSG00000145022**	TCTA	118.1233817	108.5993262	167.7320432	141.4924873	146.7851964	165.0310627	261.4770009	252.5195207	232.605366	306.8971129	242.8103713	245.2893129
**ENSG00000145040**	UCN2	1.406230734	0	3.256932878	4.186168264	11.55788948	5.311344546	0	6.645250545	4.220661883	5.694997971	13.79604382	0
**ENSG00000145041**	DCAF1	1106.703588	896.820242	1139.926507	1127.75373	941.9679928	1095.654504	1392.160751	1285.85598	1335.605005	1318.075642	1216.811065	1366.85355
**ENSG00000145050**	MANF	1216.389585	1029.941997	1297.887752	513.2242292	462.3155793	624.4623659	524.9967909	702.7352451	575.8858658	416.3676295	446.9918199	459.2830927
**ENSG00000145063**	AC062028.1	5.624922937	7.006408141	8.142332194	10.04680383	6.93473369	6.828871559	2.04278907	0	0.468962431	0	0	0
**ENSG00000145087**	STXBP5L	0	0	1.628466439	0	0	0.758763507	6.128367209	6.645250545	0.937924863	0	0	0
**ENSG00000145088**	EAF2	237.6529941	294.2691419	251.5980648	144.0041883	211.5093775	183.6207686	231.8565594	230.9224564	231.1984787	339.1687681	253.8472063	276.5848459
**ENSG00000145103**	ILDR1	112.4984587	178.6634076	173.4316757	45.21061725	61.25681426	84.60213099	16.34231256	6.645250545	30.01359561	6.960553076	9.657230676	16.07067912
**ENSG00000145107**	TM4SF19	21.09346101	38.53524477	34.19779522	19.25637402	10.40210053	9.484543833	11.23533988	4.983973908	7.503398903	6.327775524	23.4532745	5.074951301
**ENSG00000145113**	MUC4	399.3695285	714.6536304	850.8737143	83.72336529	113.2673169	182.8620051	8.171156279	8.306563181	18.75849726	5.062220419	9.657230676	5.920776517
**ENSG00000145147**	SLIT2	1.406230734	0	6.513865756	4.186168264	5.778944741	6.828871559	40.8557814	94.69482026	83.94427523	68.97275321	74.49863664	98.11572515
**ENSG00000145191**	EIF2B5	589.2106776	595.544692	653.8292752	673.1358569	614.8797205	626.3592747	617.9436936	626.3148638	674.8369389	696.6880852	633.2384115	648.7479413
**ENSG00000145214**	DGKQ	73.12399818	59.5544692	77.35215585	50.23401917	92.46311586	74.35882365	92.94690267	54.82331699	85.82012496	51.8877593	62.0821972	70.20349299
**ENSG00000145216**	FIP1L1	362.8075294	374.8428355	301.2662912	391.8253495	373.3198303	342.961105	362.5950599	333.9238399	390.6457054	338.5359905	358.6971394	423.7584336
**ENSG00000145220**	LYAR	354.370145	157.6441832	350.9345176	400.1976861	264.6756692	403.2828038	234.920743	194.3735784	250.4259384	224.0032535	125.5439988	311.2636798
**ENSG00000145241**	CENPC	584.9919854	655.0991612	561.0066882	598.6220618	657.6439116	547.8272518	779.3240301	802.4140032	674.3679764	725.7958526	761.541619	729.9471621
**ENSG00000145244**	CORIN	14.06230734	7.006408141	7.328098975	5.86063557	13.86946738	3.41443578	8.171156279	0	3.751699452	3.163887762	1.379604382	1.691650434
**ENSG00000145246**	ATP10D	1043.423205	851.2785891	944.5105346	946.0740277	843.7259322	720.0665678	2035.639308	1714.47464	1994.497221	1552.203336	1204.394626	1536.018594
**ENSG00000145247**	OCIAD2	392.3383748	451.9133251	406.3023765	304.7530496	298.1935487	359.2745204	79.66877372	63.12988017	76.90983876	74.66775118	106.2295374	49.90368779
**ENSG00000145248**	SLC10A4	0	0	2.442699658	0	0	1.896908767	3.064183605	0	0.468962431	0.632777552	0	0
**ENSG00000145283**	SLC10A6	5.624922937	0	0.814233219	4.186168264	6.93473369	2.655672273	3.064183605	0	2.813774589	1.265555105	1.379604382	0.845825217
**ENSG00000145284**	SCD5	1967.316797	1741.092423	1671.6208	2456.443538	2066.550639	2330.921492	442.2638336	538.2652941	550.5618945	523.3070358	495.2779732	612.3774569
**ENSG00000145287**	PLAC8	1338.731659	823.2529565	429.1009066	639.6465108	895.7364349	287.571369	2520.801712	4811.161394	2000.593733	1821.133796	3769.079172	1270.429476
**ENSG00000145293**	ENOPH1	1137.640664	889.8138339	918.4550715	1225.710068	1014.782697	1185.188597	550.5316543	433.602598	702.5057223	596.7092319	418.0201278	684.2726004

**ENSG00000145331**	TRMT10A	119.5296124	140.1281628	74.90945619	114.7010104	92.46311586	116.0908165	156.2733638	106.3240087	146.785241	141.1093942	96.57230676	137.0236851
**ENSG00000145332**	KLHL8	251.7153014	329.3011826	293.9381922	278.7988064	280.8567144	338.0291422	165.4659146	89.71088235	148.1921283	139.8438391	133.8216251	213.9937798
**ENSG00000145348**	TBCK	390.9321441	469.4293454	475.5122002	370.0572746	402.214554	373.3116453	475.9698533	493.4098529	506.479426	461.2948357	475.9635119	465.2038692
**ENSG00000145349**	CAMK2D	2105.127409	2003.832728	2163.417664	1142.823936	1152.321581	1316.075302	822.2226006	772.5103758	833.3462407	563.1720216	547.7029398	614.0691074
**ENSG00000145354**	CISD2	565.3047551	770.7048955	604.975282	590.2497253	607.9449868	566.4169577	384.0443451	541.5879194	366.259659	402.4465233	481.4819294	422.9126084
**ENSG00000145358**	DDIT4L	7.031153671	10.50961221	4.885399317	15.9074394	13.86946738	12.89897961	2.04278907	18.274439	3.28273702	8.226108181	5.518417529	4.229126084
**ENSG00000145362**	ANK2	68.90530597	59.5544692	179.1313083	17.58190671	39.29682424	45.90519215	10.21394535	1.661312636	5.158586746	3.796665314	0	6.766601734
**ENSG00000145365**	TIFA	402.18199	651.5959571	370.4761148	346.6147323	581.361841	350.1693583	271.6909463	407.0215959	307.639355	267.0321271	382.1504139	285.8889233
**ENSG00000145375**	SPATA5	1317.638198	1194.592588	1470.505194	967.8421027	1057.546888	1161.287547	869.2067492	674.4929303	928.0766518	768.1919486	584.9522581	879.6582254
**ENSG00000145386**	CCNA2	1818.256339	900.3234461	1360.58371	2479.048846	2073.485373	2286.154445	1632.188467	1433.712805	1917.11842	1800.884914	1681.737742	2271.886532
**ENSG00000145388**	METTL14	575.1483703	697.13761	601.7183492	560.9465474	590.6081526	529.2375459	616.9222991	593.0886111	533.2102846	587.2175686	655.3120816	627.6023108
**ENSG00000145390**	USP53	3269.486457	5076.142698	3988.114309	3091.90388	3749.379348	3829.8588	1529.027619	1799.201585	1453.314575	1616.113869	2003.185563	1627.367717
**ENSG00000145391**	SETD7	2134.658254	2305.108278	2305.908477	2271.4149	2365.899977	2222.418311	1013.223379	802.4140032	1068.296419	1025.732412	796.0317286	1188.38443
**ENSG00000145414**	NAF1	314.9956845	259.2371012	328.1359874	362.5221717	313.218805	360.0332839	217.5570359	171.1152015	247.6121638	265.1337944	169.691339	235.9852355
**ENSG00000145416**	1-Mar	43.59315276	80.57369362	55.36785892	62.79252397	104.0210053	77.77325943	3.064183605	6.645250545	7.503398903	2.53111021	12.41643944	5.074951301
**ENSG00000145425**	RPS3A	8400.822406	7346.218936	7307.743145	8825.279935	8394.495131	8256.485097	5853.612079	6495.732407	6285.503469	6130.981705	6649.693123	6707.393969
**ENSG00000145431**	PDGFC	209.5283794	224.2050605	143.3050466	187.5403382	417.2398103	282.2600245	10.21394535	9.967875817	26.26189616	11.38999594	22.07367012	35.5246591
**ENSG00000145439**	CBR4	293.9022234	287.2627338	244.2699658	298.0551804	297.0377597	240.9074133	220.6212195	209.3253922	210.0951693	220.8393658	251.0879976	233.4477598
**ENSG00000145476**	CYP4V2	393.7446056	283.7595297	281.7246939	83.72336529	122.5136285	99.77740112	126.6529223	147.8568246	140.6887294	72.76941852	88.29468047	100.6532008
**ENSG00000145491**	ROPN1L	11.24984587	3.50320407	2.442699658	2.511700959	3.467366845	4.173199286	4.08557814	1.661312636	0.937924863	0.632777552	0	0.845825217
**ENSG00000145494**	NDUFS6	1026.548436	1029.941997	1105.728712	1360.504686	1182.372094	1317.592829	895.7630071	822.3497549	857.2633247	909.9341203	816.7257943	1225.600739
**ENSG00000145495**	6-Mar	5962.418313	6106.084695	5456.176804	6615.820325	6596.087528	6609.588906	5567.62161	5618.559335	5460.598552	5947.476215	5857.800207	5812.51089
**ENSG00000145506**	NKD2	1.406230734	0	0.814233219	0	4.623155793	1.896908767	0	0	0	0	0	2.53747565
**ENSG00000145526**	CDH18	944.9870534	949.3683031	713.2683002	838.0708865	828.7006759	530.3756911	412.6433921	515.0069172	294.508407	456.8653928	573.915423	373.0089206
**ENSG00000145536**	ADAMTS16	158.904073	469.4293454	486.097232	100.4680383	268.143036	262.5321733	45.96275407	227.5998311	111.1440963	47.45831643	166.9321303	101.499026
**ENSG00000145545**	SRD5A1	845.1446712	970.3875275	927.411637	977.8889065	1096.843712	991.7039031	878.3993	1249.307102	998.4210166	934.6124449	1189.218978	947.3242428
**ENSG00000145555**	MYO10	7936.766264	6372.328204	6826.531312	9112.451078	8000.3711	7761.391909	384.0443451	767.5264379	765.3466881	404.9776335	849.8362995	960.8574463
**ENSG00000145569**	FAM105A	12.65607661	14.01281628	21.98429693	8.372336529	26.58314581	17.0721789	1.021394535	9.967875817	2.344812157	2.53111021	6.898021912	16.07067912
**ENSG00000145592**	RPL37	11372.18795	9970.118784	10923.75287	10881.52579	9575.711436	10132.52787	8876.939903	9137.219499	9579.495587	8369.748685	8190.711218	9367.514276
**ENSG00000145604**	SKP2	2039.034565	1460.836097	1775.842652	2347.603163	1783.382347	2115.053275	7328.505788	5887.691982	7523.095325	6883.986992	5754.329879	7446.645208
**ENSG00000145623**	OSMR	2571.996013	4893.976086	3318.814602	1832.704466	3105.604904	2646.567111	1699.600506	3294.382957	2010.441944	1895.801547	3511.093153	2381.84381
**ENSG00000145632**	PLK2	6796.313138	4119.767987	5519.686995	5496.438931	4379.284325	5051.088663	3560.581349	4347.655169	3581.935051	2974.054496	3708.37658	3275.035239
**ENSG00000145675**	PIK3R1	513.274218	578.0286716	425.8439738	402.709387	492.366092	415.8024016	498.440533	721.0096841	330.1495517	391.0565274	771.1988497	404.3044536
**ENSG00000145681**	HAPLN1	0	0	0	0	0	0	15.32091802	8.306563181	33.29633263	23.41276944	17.93485697	44.82873649
**ENSG00000145685**	LHFPL2	137.8106119	199.682632	251.5980648	191.7265065	340.9577397	317.9219093	313.5681222	225.9385185	194.1504466	391.0565274	315.9294035	296.0388259
**ENSG00000145687**	SSBP2	106.8735358	182.1666117	85.49448804	210.9828805	217.2883223	133.9217589	731.318487	729.3162473	705.3194969	720.1008546	798.7909374	674.968523
**ENSG00000145700**	ANKRD31	39.37446056	10.50961221	24.42699658	39.34998168	20.80420107	24.28043221	50.04833221	31.56494009	40.79973154	47.45831643	55.18417529	49.90368779
**ENSG00000145703**	IQGAP2	0	0	0.814233219	0	0	0	22.47067977	23.25837691	41.26869397	31.00610007	30.35129641	35.5246591
**ENSG00000145715**	RASA1	1185.452509	1124.528507	1371.168742	1907.218261	1784.538136	1877.180915	1306.36361	1272.565479	1448.155988	1481.33225	1325.799811	1749.166548
**ENSG00000145723**	GIN1	331.8704533	196.1794279	272.7681285	258.7051987	253.1177797	232.181633	215.5142469	207.6640795	200.2469582	221.4721433	213.8386793	256.2850407
**ENSG00000145725**	PPIP5K2	773.4269038	907.3298542	911.1269726	930.1665883	976.6416613	967.0440892	1625.038705	1307.453045	1462.224861	1588.904434	1358.910317	1651.896648
**ENSG00000145730**	PAM	1224.826969	2280.58585	1219.721363	837.2336529	1318.75519	901.0316641	480.0554314	1581.56963	275.7499097	378.4009763	1219.570274	313.8011554
**ENSG00000145734**	BDP1	2701.36924	2651.925481	2705.696988	2774.592326	2831.682923	2563.861889	2972.258096	2599.954276	2592.893284	3010.122817	2734.375886	3058.503984
**ENSG00000145736**	GTF2H2	73.12399818	38.53524477	83.05178838	77.86272972	54.32208057	66.77118858	43.919965	53.16200436	66.59266527	56.94997971	40.00852709	65.97436691
**ENSG00000145740**	SLC30A5	1123.578357	1071.980446	1152.140006	1224.0356	1285.23731	1275.860836	1093.913547	1142.983094	1205.702411	1160.514031	1045.740122	1346.553745
**ENSG00000145741**	BTF3	5672.734782	5065.633086	5115.827318	5722.492017	5481.906982	5370.5281	4964.998834	5444.121509	4745.430844	4944.523794	5181.79406	5173.912851
**ENSG00000145743**	FBXL17	260.1526858	206.6890402	301.2662912	254.5190305	301.6609155	262.5321733	539.2963144	476.7967266	574.947941	566.9686869	481.4819294	576.0069726
**ENSG00000145757**	SPATA9	5.624922937	7.006408141	0	0	0	1.13814526	18.38510163	0	20.63434698	16.45221636	6.898021912	7.612426951
**ENSG00000145777**	TSLP	0	0	0	2.511700959	6.93473369	2.27629052	3.064183605	1.661312636	0.468962431	1.898332657	0	1.691650434
**ENSG00000145779**	TNFAIP8	764.9895194	1306.695118	823.1897849	693.2294646	955.8374602	710.5820239	1380.925411	2021.817478	1140.047671	1341.488411	2263.930791	1261.971223
**ENSG00000145780**	FEM1C	1707.164111	2080.903218	1697.676263	1414.924873	1591.521382	1533.081665	963.1750464	1174.548034	944.4903369	833.3680365	1169.904516	952.3991941
**ENSG00000145781**	COMMD10	333.276684	343.3139989	294.7524254	339.9168631	272.7661918	334.9940882	341.1457746	252.5195207	302.9497307	287.9137863	256.6064151	270.6640694
**ENSG00000145782**	ATG12	2421.529324	2322.624299	2476.08322	2099.782001	2052.681172	2293.74208	1116.384227	1377.228175	1157.399281	1102.931274	1172.663725	1215.450837
**ENSG00000145794**	MEGF10	4.218692202	14.01281628	6.513865756	3.348934611	3.467366845	6.070108053	1.021394535	0	0	2.53111021	0	1.691650434
**ENSG00000145817**	YIPF5	1500.448193	1397.778424	1459.105929	1144.498403	1423.931984	1313.41963	983.6029371	1166.241471	1161.619943	909.9341203	1008.490803	1203.609283
**ENSG00000145819**	ARHGAP26	862.01944	819.7497525	878.5576438	837.2336529	887.6459123	915.8275525	1902.858018	2360.725256	1961.200888	1688.25051	2083.202617	1782.999557
**ENSG00000145832**	SLC25A48	5.624922937	0	2.442699658	0.837233653	0	1.517527013	0	0	0.937924863	0	0	0
**ENSG00000145833**	DDX46	3148.550614	3121.354827	3078.615803	3794.342915	3871.892977	3387.120294	2981.450647	3237.898328	2763.595609	3273.991056	3213.098606	3527.936979
**ENSG00000145860**	RNF145	1576.384653	1607.970668	1258.804557	1816.797027	2012.228559	1634.755975	1426.888165	1905.525594	1526.472714	1437.037821	1741.06073	1696.725385
**ENSG00000145861**	C1QTNF2	11.24984587	28.02563256	22.79853014	10.88403749	24.27156791	25.79795922	0	0	0.468962431	0	5.518417529	2.53747565
**ENSG00000145868**	FBXO38	1023.735974	1092.99967	1057.688952	896.6772422	800.9617411	877.1306136	1074.507051	1192.822473	1088.461803	1087.111835	1204.394626	1018.373561
**ENSG00000145882**	PCYOX1L	91.40499772	52.54806106	46.41129351	131.4456835	156.031508	89.53409378	146.0594185	89.71088235	125.2129692	174.6466045	129.6828119	134.4862095
**ENSG00000145901**	TNIP1	4616.6555	6862.776774	5522.129694	3304.561228	4505.26532	3913.322785	1509.621123	2900.651863	1646.058134	1106.727939	2585.378612	1316.949863
**ENSG00000145907**	G3BP1	7125.37113	5626.145737	6254.125359	6800.848962	5980.052018	6399.790796	6554.28873	6733.300114	6786.824308	6734.65149	6375.151851	7600.585398
**ENSG00000145908**	ZNF300	618.741523	472.9325495	531.6942923	500.6657244	486.5871472	510.2684582	1099.02052	1103.11159	1041.096598	1056.738512	1087.128253	1021.756862
**ENSG00000145911**	N4BP3	172.9663803	231.2114686	178.3170751	138.9807864	235.7809454	193.8640759	35.74880872	59.8072549	64.71681554	36.06832049	64.84140597	44.82873649
**ENSG00000145912**	NHP2	798.739057	651.5959571	617.1887803	995.4708133	936.1890481	999.67092	578.1093067	363.8274673	631.2234327	594.1781217	513.2128302	597.9984283

**ENSG00000145916**	RMND5B	618.741523	623.5703245	745.837629	560.1093138	588.2965747	629.014947	601.601381	561.523671	552.9067067	575.8275727	521.4904565	603.9192048
**ENSG00000145919**	BOD1	721.3963666	641.0863449	805.276654	688.2060627	686.5386353	704.1325341	594.4516193	503.3777287	667.8025024	616.325336	587.7114669	663.12697
**ENSG00000145934**	TENM2	2.812461468	14.01281628	4.885399317	5.023401917	10.40210053	2.27629052	1.021394535	1.661312636	2.813774589	3.163887762	2.759208765	1.691650434
**ENSG00000145945**	FAM50B	0	0	3.256932878	0.837233653	0	0	5.106972674	0	0.468962431	0	0	0
**ENSG00000145949**	MYLK4	30.93707615	45.54165292	25.2412298	26.79147689	50.85471372	20.86599643	43.919965	78.0816939	46.89624315	48.72387153	75.87824103	36.37048432
**ENSG00000145979**	TBC1D7	234.8405326	143.6313669	219.8429693	235.2626565	234.6251565	231.0434878	193.0435671	187.7283279	201.1848831	210.7149249	183.4873828	259.6683416
**ENSG00000145982**	FARS2	185.6224569	196.1794279	197.8586723	161.586095	171.0567643	191.2084037	272.7123408	176.0991394	254.6466003	253.7437985	262.1248326	200.4605764
**ENSG00000145990**	GFOD1	420.4629895	458.9197332	643.2442434	413.5934245	411.4608656	635.8438185	708.8478072	1638.054259	656.0784416	702.3830831	1750.717961	624.21901
**ENSG00000145996**	CDKAL1	3453.702683	3422.630377	3595.653897	3513.869641	3351.78795	3569.602917	4242.872898	4448.99524	4260.52369	4113.05409	4155.3684	4080.260846
**ENSG00000146005**	PSD2	0	0	0.814233219	0	0	0	21.44928523	18.274439	6.096511609	24.67832454	11.03683506	9.304077385
**ENSG00000146006**	LRRTM2	1.406230734	0	1.628466439	0	0	1.13814526	9.192550814	3.322625272	2.344812157	6.327775524	9.657230676	2.53747565
**ENSG00000146007**	ZMAT2	1319.044429	1348.733567	1374.425674	1326.178106	1334.936235	1196.949432	1636.274045	1672.941825	1534.914038	1574.983328	1710.709434	1764.391402
**ENSG00000146013**	GFRA3	2.812461468	0	0	0	4.623155793	0	0	0	2.344812157	0	0	0
**ENSG00000146021**	KLHL3	2.812461468	3.50320407	8.956565414	1.674467306	8.090522638	3.035054026	8.171156279	6.645250545	7.034436472	8.858885733	5.518417529	1.691650434
**ENSG00000146038**	DCDC2	0	0	0.814233219	0	0	0.379381753	1.021394535	9.967875817	3.28273702	1.265555105	0	4.229126084
**ENSG00000146047**	HIST1H2BA	80.15515185	14.01281628	65.13865756	74.51379511	43.91998003	75.87635066	6.128367209	3.322625272	22.97915914	16.45221636	6.898021912	29.60388259
**ENSG00000146054**	TRIM7	196.8723028	210.1922442	201.9298384	151.5392912	305.1282823	202.5898563	18.38510163	21.59706427	23.44812157	16.45221636	15.17564821	10.99572782
**ENSG00000146063**	TRIM41	871.8630552	830.2593647	850.0594811	743.4634837	873.7764449	799.736736	887.5918508	933.6577015	944.4903369	858.046361	929.8533537	846.671042
**ENSG00000146066**	HIGD2A	241.8716863	192.6762239	281.7246939	291.3573112	288.9472371	325.5095443	218.5784305	287.407086	321.708228	308.162668	322.8274255	327.3343589
**ENSG00000146067**	FAM193B	434.5252969	557.0094472	475.5122002	343.2657977	314.3745939	357.3776116	425.921521	445.2317865	401.9008038	427.7576254	437.3345892	319.7219319
**ENSG00000146070**	PLA2G7	1.406230734	10.50961221	15.47043117	4.186168264	6.93473369	10.62268909	0	0	0.468962431	0	0	0
**ENSG00000146072**	TNFRSF21	5537.736631	3356.069499	4676.141379	4721.997802	2922.99025	4240.349857	1951.884956	940.302952	2105.641317	1871.756	1327.179416	2162.775079
**ENSG00000146083**	RNF44	790.3016726	781.2145077	801.2054879	746.8124184	766.2880727	839.9512018	634.2860062	446.8930991	689.3747742	645.4331034	471.8246987	553.1696918
**ENSG00000146085**	MUT	569.5234473	599.047896	512.152695	461.3157427	494.6776699	468.5364653	444.3066227	456.8609749	405.6525032	460.6620581	504.9352039	486.3494996
**ENSG00000146090**	RASGEF1C	4.218692202	3.50320407	0.814233219	3.348934611	4.623155793	14.03712487	1.021394535	0	0	0	0	0
**ENSG00000146094**	DOK3	33.74953762	38.53524477	24.42699658	20.93084132	42.76419109	20.10723293	8.171156279	8.306563181	7.034436472	10.12444084	5.518417529	6.766601734
**ENSG00000146109**	ABT1	385.3072212	364.3332233	432.3578395	368.3828073	359.4503629	446.1529419	435.1140719	426.9573475	429.1006248	408.1415213	402.8444796	433.9083362
**ENSG00000146112**	PPP1R18	7053.653363	7773.609832	9707.288442	5480.531492	5062.355593	6710.504452	2569.82865	3681.468802	3463.756519	2162.833674	2899.928412	2910.484571
**ENSG00000146143**	PRIM2	1060.297974	1198.095792	1058.503185	1057.426104	1014.782697	991.7039031	961.1322573	1028.352522	885.870033	874.4985774	1071.952605	992.9988045
**ENSG00000146147**	MLIP	1.406230734	0	1.628466439	0	0	2.27629052	2.04278907	0	1.875849726	0	0	0
**ENSG00000146166**	LGSN	160.3103037	381.8492437	146.5619795	117.2127114	253.1177797	86.87842151	230.8351649	162.8086383	152.8817527	177.8104922	126.9236032	150.5568886
**ENSG00000146192**	FGD2	23.90592248	24.52242849	11.39926507	0	3.467366845	1.13814526	3.064183605	0	0.937924863	0	0	0
**ENSG00000146205**	ANO7	12.65607661	7.006408141	12.21349829	3.348934611	5.778944741	6.828871559	9.192550814	8.306563181	10.31717349	1.265555105	0	5.074951301
**ENSG00000146215**	CRIP3	5.624922937	0	2.442699658	1.674467306	0	3.41443578	0	3.322625272	0.937924863	0	0	1.691650434
**ENSG00000146221**	TCTE1	0	0	0	1.674467306	2.311577897	0.379381753	2.04278907	3.322625272	0	0	0	1.691650434
**ENSG00000146223**	RPL7L1	2670.432164	1912.749422	2581.933539	3292.839957	2595.901978	3157.594333	2096.92298	1751.023518	2121.58604	2333.050836	1870.743542	2437.668275
**ENSG00000146232**	NFKBIE	383.9009904	385.3524477	560.192455	261.2168997	282.0125034	412.3879658	59.24088302	84.72694444	90.50974927	41.1305409	74.49863664	60.05359039
**ENSG00000146233**	CYP39A1	15.46853808	17.51602035	18.72736405	31.81487881	58.94523636	26.93610448	75.58319558	88.04956971	42.67558126	55.68442461	89.67428485	65.97436691
**ENSG00000146242**	TPBG	2566.37109	4126.774395	2761.064847	2123.224544	3898.476122	2565.000034	2037.682097	3575.144793	1921.339082	1785.698253	3566.277328	1769.466353
**ENSG00000146243**	IRAK1BP1	33.74953762	21.01922442	30.12662912	69.49039319	57.78944741	42.49075637	79.66877372	24.91968954	79.25465092	93.65107775	34.49010956	69.35766778
**ENSG00000146247**	PHIP	3608.388064	4655.75821	3888.777856	4346.917126	4261.393852	4033.207419	4826.089177	4498.834619	4435.915639	4791.391627	4726.524614	5124.009163
**ENSG00000146263**	MMS22L	2677.463318	2816.576073	2369.418669	3017.390085	2913.743939	2793.767231	2533.058446	2327.499003	2426.41162	2593.755187	2274.967626	2625.441473
**ENSG00000146267**	FAXC	178.5913032	220.7018564	169.3605096	263.7286007	295.8819708	248.8744302	204.278907	265.8100218	200.7159207	222.1049209	242.8103713	245.2893129
**ENSG00000146276**	GABRR1	1.406230734	0	0.814233219	0	0	0	4.08557814	8.306563181	0.937924863	5.694997971	0	5.074951301
**ENSG00000146278**	PNRC1	563.8985244	1257.650261	606.6037485	315.6370871	1003.224807	397.2126957	1184.81766	2948.829929	1134.420122	1113.055715	2450.177383	1059.818997
**ENSG00000146281**	PM20D2	94.21745919	157.6441832	101.7791524	133.9573845	150.2525633	98.63925586	132.7812895	260.8260839	145.8473162	151.8666126	340.7622824	158.1693155
**ENSG00000146282**	RARS2	700.3029056	686.6279978	802.0197212	802.0698394	783.6249069	792.1491009	849.800253	775.8330011	849.7599258	851.7185855	809.8277724	828.9087124
**ENSG00000146350**	TBC1D32	587.8044469	742.6792629	459.2275358	451.2689389	473.8734688	386.9693884	699.6552564	754.2359368	637.3199443	716.9369668	670.4877298	625.9106604
**ENSG00000146352**	CLVS2	0	0	0	0	0	0	67.4120393	26.58100218	24.38604644	53.78609195	16.55525259	22.83728085
**ENSG00000146373**	RNF217	4968.213184	4893.976086	3925.418351	7021.041413	6455.081276	6638.421919	7411.238745	6372.795272	6363.351232	7966.036607	6343.42095	7289.321718
**ENSG00000146376**	ARHGAP18	1441.386503	858.2849973	1026.74809	1239.105806	1166.191049	979.563687	1944.735194	1147.967032	1802.691587	1593.966654	893.9836397	1730.558394
**ENSG00000146386**	ABRACL	476.7122189	462.4229373	414.4447087	380.1040784	387.1892977	363.0683379	359.5308763	518.3295425	419.7213762	398.649858	409.7425015	429.6792101
**ENSG00000146409**	SLC18B1	60.46792157	52.54806106	92.0083538	56.09465474	75.12628164	59.56293527	149.1236021	79.74300653	101.7648476	135.4143962	78.63744979	109.9572782
**ENSG00000146410**	MTFR2	303.7458386	157.6441832	275.2108282	279.6360401	248.4946239	282.6394062	315.6109113	343.8917157	314.2048291	319.552664	311.7905904	340.8675624
**ENSG00000146411**	SLC2A12	140.6230734	290.7659378	150.6331456	48.55955187	93.61890481	56.907263	45.96275407	49.83937908	45.48935585	37.96665314	49.66575776	33.83300867
**ENSG00000146414**	SHPRH	1108.109819	896.820242	1104.914479	1190.546254	1100.311079	1061.130764	1926.350093	1704.506765	1855.215379	2155.240343	1887.298795	1957.239552
**ENSG00000146416**	AIG1	1105.297357	1012.425976	897.2850078	836.3964192	959.3048271	739.7944189	775.238452	825.6723802	842.7254893	690.3603096	921.5757274	727.4096864
**ENSG00000146425**	DYNLT1	1087.016358	998.4131601	1031.633489	1046.542066	978.9532392	921.5182788	932.5332103	1005.094145	837.5669026	787.8080527	807.0685637	779.0050247
**ENSG00000146426**	TIAM2	94.21745919	164.6505913	113.1784175	116.3754777	109.7999501	104.7093639	115.4175824	106.3240087	109.2682465	114.532737	161.4137127	94.73242428
**ENSG00000146433**	TMEM181	1642.477497	1296.185506	1520.987654	1629.256688	1410.062517	1803.580855	1119.44841	875.5117592	1167.716454	1288.967874	1020.907243	1300.879183
**ENSG00000146453**	PNLDC1	0	7.006408141	0	0	0	2.27629052	1.021394535	3.322625272	7.034436472	1.898332657	0	2.53747565
**ENSG00000146457**	WTAP	1853.412108	1853.194953	1709.889761	1781.633213	1423.931984	1586.574492	1739.434893	1883.928529	1835.987919	1895.801547	1844.531059	1867.582079
**ENSG00000146463**	ZMYM4	2739.33747	2459.249257	2545.293044	2690.86896	2791.23031	2579.795922	2691.374599	2578.357211	2412.342747	2502.002442	2710.922611	2446.126527
**ENSG00000146476**	ARMT1	811.3951336	742.6792629	731.9956643	871.5602326	876.0880228	814.9120061	842.6504913	1011.739395	864.2977612	804.8930466	986.4171334	954.0908445
**ENSG00000146521**	LINC01558	0	21.01922442	2.442699658	0	1.155788948	1.517527013	0	1.661312636	0	0	2.759208765	0
**ENSG00000146530**	VWDE	5.624922937	24.52242849	8.142332194	2.511700959	16.18104528	17.83094241	268.6267627	166.1312636	127.5577814	230.9638066	161.4137127	133.6403843
**ENSG00000146535**	GNA12	828.2699024	770.7048955	754.7941944	887.467672	1005.536385	947.316238	531.1251581	528.2974183	661.7059908	586.5847911	579.4338406	666.5102708

**ENSG00000146540**	C7orf50	340.3078377	301.2755501	338.7210193	416.9423591	479.6524135	506.4746407	331.9532238	272.4552723	430.5075121	405.6104111	340.7622824	416.9918319
**ENSG00000146555**	SDK1	25.31215321	24.52242849	26.05546302	25.11700959	36.98524634	39.8350841	1168.475348	1249.307102	1319.19132	1323.77064	1432.029349	1311.874911
**ENSG00000146556**	WASH2P	21.09346101	35.0320407	29.3123959	18.41914036	18.49262317	24.28043221	50.04833221	39.87150327	38.92388181	50.62220419	34.49010956	31.29553302
**ENSG00000146574**	CCZ1B	269.996301	322.2947745	315.1082559	297.2179468	314.3745939	269.3610448	262.4983955	242.5516449	205.8745074	233.4949168	216.597888	175.9316451
**ENSG00000146576**	C7orf26	125.1545353	84.07689769	121.3207497	135.6318518	141.0062517	151.3733196	113.3747934	167.7925762	173.0471372	120.8605125	117.2663725	140.406986
**ENSG00000146587**	RBAK	746.7085198	840.7689769	688.0270704	612.8550339	696.9407358	657.0891967	803.8374989	917.0445751	694.0643986	828.9385936	939.5105844	828.9087124
**ENSG00000146592**	CREB5	49.2180757	56.05126513	62.6959579	25.11700959	31.2063016	41.35261111	137.8882622	126.2597603	166.9506256	144.2732819	164.1729215	193.6939746
**ENSG00000146648**	EGFR	22018.76084	17505.51074	20147.38678	14291.57845	14335.25033	14054.19705	20734.30906	28368.57457	19630.76738	17479.21433	25551.65276	16253.37737
**ENSG00000146666**	LINC00525	0	0	1.628466439	0	0	0	9.192550814	9.967875817	23.44812157	6.327775524	0	1.691650434
**ENSG00000146670**	CDCA5	763.5832886	784.7177118	924.9689373	981.2378412	983.576395	1089.205014	822.2226006	913.7219499	1008.269228	891.5835713	982.2783202	1059.818997
**ENSG00000146674**	IGFBP3	2676.057087	4652.255006	4367.546989	2325.835088	3725.10778	3444.406938	3858.828553	20716.56857	3786.871634	3439.778775	22213.01016	3498.333097
**ENSG00000146676**	PURB	3033.239694	2711.479951	2841.673936	3069.298571	2696.455616	2841.948714	2621.919771	2428.839074	2791.733354	2648.174057	2632.285161	2962.079909
**ENSG00000146677**	AC004453.1	4.218692202	7.006408141	6.513865756	8.372336529	5.778944741	4.55258104	5.106972674	3.322625272	3.28273702	6.960553076	23.4532745	7.612426951
**ENSG00000146678**	IGFBP1	132.185689	59.5544692	103.4076189	94.60740277	62.41260321	111.1588537	2.04278907	6.645250545	6.096511609	6.327775524	9.657230676	3.383300867
**ENSG00000146700**	SSC4D	2.812461468	0	2.442699658	5.023401917	11.55788948	2.27629052	3.064183605	0	1.875849726	1.265555105	1.379604382	6.766601734
**ENSG00000146701**	MDH2	1555.291192	1425.804057	1709.075528	1764.051307	1551.068769	1876.422152	1576.011767	1430.39018	1928.373518	1876.185443	1747.958752	1944.552173
**ENSG00000146707**	POMZP3	95.62368992	136.6249587	79.79485551	87.0722999	94.77469376	86.49903975	78.64737919	88.04956971	76.44087633	63.27775524	99.33151553	77.81591994
**ENSG00000146729**	NIPSNAP2	599.0542928	662.1055693	511.3384618	802.9070731	862.2185554	633.567528	941.7257612	1111.418154	893.3734319	1052.30907	1063.674979	997.2279306
**ENSG00000146731**	CCT6A	5451.956556	4753.847924	5214.349537	6312.741743	5565.123786	6004.095628	3651.485462	3307.673459	4269.433976	3959.921923	3404.863616	5140.079842
**ENSG00000146733**	PSPH	358.5888372	245.2242849	362.3337827	304.7530496	372.1640413	379.3817533	283.9476807	255.842146	291.2256699	240.4554699	256.6064151	274.8931955
**ENSG00000146757**	ZNF92	126.5607661	161.1473872	118.87805	126.4222816	102.8652164	112.296999	248.198872	249.1968954	208.2193196	238.5571372	253.8472063	236.8310607
**ENSG00000146776**	ATXN7L1	216.5595331	185.6698157	253.2265312	188.3775719	171.0567643	182.1032416	104.1822426	101.3400708	108.3303217	104.4082961	100.7111199	109.9572782
**ENSG00000146802**	TMEM168	547.0237556	336.3075908	477.1406666	643.8326791	546.6881725	533.7901269	918.2336869	518.3295425	917.7594784	1025.099635	647.0344553	1103.801908
**ENSG00000146826**	C7orf43	129.3732275	84.07689769	108.2930182	101.305272	99.39784955	110.0207085	103.160848	94.69482026	123.8060819	108.837739	71.73942788	96.42407471
**ENSG00000146828**	SLC12A9	251.7153014	189.1730198	235.3134004	302.2413487	325.9324834	296.2971493	276.797919	177.7604521	292.1635948	282.8515659	235.9123494	279.9681468
**ENSG00000146830**	GIGYF1	637.0225226	609.5575083	630.2165119	597.7848281	613.7239315	515.9591845	746.639405	661.2024292	686.5609996	715.0386342	727.0515095	663.12697
**ENSG00000146833**	TRIM4	698.8966749	609.5575083	657.0862081	637.1348098	596.3870973	656.709815	735.4040651	779.1556263	713.2918582	692.2586423	747.7455752	664.8186204
**ENSG00000146834**	MEPCE	780.4580575	753.1888751	705.9402013	831.3730173	780.1575401	848.2976004	873.2923273	825.6723802	1004.986491	903.6063448	848.4566951	905.8788072
**ENSG00000146839**	ZAN	11.24984587	0	3.256932878	9.209570182	1.155788948	10.24330734	1.021394535	0	0	0	0	0.845825217
**ENSG00000146842**	TMEM209	1275.451276	1320.707935	1178.195469	1333.713209	1175.43736	1255.753603	1354.369153	1272.565479	1493.176382	1375.658399	1070.573001	1530.943642
**ENSG00000146856**	AGBL3	66.09284451	56.05126513	43.96859385	55.25742109	41.60840214	34.9031213	77.62598465	63.12988017	66.59266527	49.35664909	34.49010956	67.66601734
**ENSG00000146858**	ZC3HAV1L	314.9956845	511.4677943	307.780157	488.9444533	690.0060021	507.2334042	428.9857046	679.4768682	475.5279055	485.9731602	775.3376629	658.8978439
**ENSG00000146859**	TMEM140	281.2461468	423.8876925	368.8476484	10.04680383	25.42735686	12.89897961	484.1410095	372.1340305	435.1971364	68.33997566	44.14734023	38.90795997
**ENSG00000146872**	TLK2	873.2692859	830.2593647	893.2138417	670.6241559	663.4228563	749.6583445	935.5973939	780.816939	763.9398008	708.7108587	778.0968716	698.6516291
**ENSG00000146904**	EPHA1	244.6841477	66.56087734	219.028736	330.7072929	302.8167044	388.4869154	400.3866577	343.8917157	531.8033973	459.396503	286.9577115	464.358044
**ENSG00000146909**	NOM1	774.8331345	672.6151815	836.2175164	833.0474846	758.1975501	814.5326243	744.5966159	654.5571786	831.470391	838.4302569	805.6889593	881.3498759
**ENSG00000146918**	NCAPG2	2702.775471	3044.284337	2491.553652	3142.137899	3272.038513	3026.707628	3004.942722	3678.146176	3100.779597	3397.382679	3356.577462	3291.951744
**ENSG00000146938**	NLGN4X	73.12399818	24.52242849	60.25325824	44.3733836	38.14103529	47.04333741	100.0966644	151.1794499	124.2750443	99.34607572	140.719647	124.3363069
**ENSG00000146950**	SHROOM2	1.406230734	0	0.814233219	4.186168264	4.623155793	1.13814526	76.60459012	31.56494009	42.20661883	65.80886545	68.98021912	49.90368779
**ENSG00000146955**	RAB19	189.8411491	136.6249587	115.6211172	32.65211246	53.16629162	38.31755708	255.3486337	234.2450817	248.5500887	161.9910534	81.39665856	90.5032982
**ENSG00000146963**	LUC7L2	687.646829	514.9709984	485.2829988	617.8784358	562.8692178	512.165367	1088.806574	892.1248856	876.9597468	947.9007735	816.7257943	732.4846377
**ENSG00000146966**	DENND2A	2.812461468	24.52242849	7.328098975	11.72127114	34.67366845	3.035054026	0	1.661312636	0.937924863	1.898332657	4.138813147	0.845825217
**ENSG00000147003**	CLTRN	18.28099954	7.006408141	14.65619795	0	1.155788948	4.55258104	8.171156279	3.322625272	6.096511609	8.226108181	4.138813147	2.53747565
**ENSG00000147010**	SH3KBP1	216.5595331	658.6023652	718.9679328	175.8190671	520.1050267	547.8272518	2210.297773	2661.422843	2372.480941	2004.006508	2298.420901	2131.479546
**ENSG00000147027**	TMEM47	23.90592248	7.006408141	7.328098975	9.209570182	4.623155793	6.828871559	18.38510163	34.88756536	24.38604644	25.94387965	40.00852709	32.14135824
**ENSG00000147036**	LANCL3	30.93707615	84.07689769	79.79485551	68.65315954	72.81470374	135.0599042	1.021394535	8.306563181	2.344812157	5.694997971	1.379604382	1.691650434
**ENSG00000147044**	CASK	91.40499772	136.6249587	70.83829009	87.0722999	168.7451864	118.7464888	414.6861812	468.4901634	384.0802314	465.091501	475.9635119	481.2745483
**ENSG00000147050**	KDM6A	1338.731659	1625.486689	1307.65855	977.0516729	1014.782697	1107.415338	1762.926967	1440.358056	1431.742303	1390.212283	1529.98126	1406.607335
**ENSG00000147065**	MSN	4.218692202	14.01281628	14.65619795	1.674467306	4.623155793	10.62268909	4.08557814	6.645250545	3.28273702	2.53111021	16.55525259	7.612426951
**ENSG00000147082**	CCNB3	61.8741523	101.592918	57.81055858	56.09465474	72.81470374	94.84543833	3.064183605	0	0.937924863	0.632777552	11.03683506	4.229126084
**ENSG00000147099**	HDAC8	593.4293698	518.4742024	469.8125676	456.2923408	487.7429362	382.0374256	631.2218226	465.1675381	400.4939165	566.3359094	536.6661047	451.6706658
**ENSG00000147100**	SLC16A2	2496.059553	1940.775055	2172.374229	2862.501859	2610.927234	2715.993972	2104.072742	3872.519755	2409.06001	2201.433105	3951.186951	2282.88226
**ENSG00000147118**	ZNF182	178.5913032	199.682632	174.245909	148.1903566	145.6294075	133.9217589	259.4342119	214.3093301	196.9642212	225.2688086	190.3854048	176.7774703
**ENSG00000147119**	CHST7	36.56199909	59.5544692	39.89742775	46.04785091	52.01050267	38.31755708	18.38510163	29.90362745	19.22745969	51.8877593	28.97169203	34.67883389
**ENSG00000147121**	KRBOX4	272.8087624	336.3075908	319.9936552	314.7998535	273.9219807	367.620919	308.4611495	347.214341	320.7703031	337.2704354	346.2807	332.4093102
**ENSG00000147123**	NDUFB11	296.7146849	297.772346	290.6812593	315.6370871	339.8019508	310.3342742	258.4128173	307.3428377	316.0806788	355.6209844	376.6319964	360.3215423
**ENSG00000147124**	ZNF41	216.5595331	294.2691419	232.0564675	282.147741	249.6504128	226.8702885	290.0760479	277.4392102	266.8396235	267.0321271	286.9577115	240.2143616
**ENSG00000147130**	ZMYM3	381.088529	416.8812844	371.2903481	389.3136486	597.5428863	386.2106249	389.1513178	496.7324782	336.7150258	403.7120784	482.8615338	299.4221267
**ENSG00000147133**	TAF1	905.6125928	791.7241199	956.7240329	913.4219153	814.8312085	893.444029	1219.545075	1049.949586	1113.316812	1080.151282	1185.080164	1139.326567
**ENSG00000147140**	NONO	5025.868644	4284.418578	4707.082242	5030.099786	4990.696679	5235.088814	4700.457649	4970.647407	4875.8024	5069.180972	5532.213573	5242.424694
**ENSG00000147144**	CCDC120	382.4947597	346.817203	284.1673936	373.4062092	329.3998503	366.1033919	235.9421376	285.7457734	246.6742389	203.1215943	269.0228546	185.2357225
**ENSG00000147155**	EBP	257.3402244	185.6698157	207.629471	313.1253862	353.6714182	262.1527915	170.5728873	210.9867048	155.2265648	176.5449371	257.9860195	180.1607712
**ENSG00000147162**	OGT	5515.236939	5769.777104	5868.178813	4510.177688	4293.755943	4728.993555	7033.322767	6497.39372	6328.17905	6245.514442	5546.009617	5483.48488
**ENSG00000147164**	SNX12	395.1508363	353.8236111	422.5870409	550.0625099	505.0797704	561.8643766	791.5807645	606.3791122	846.0082263	900.442457	669.1081254	839.058615
**ENSG00000147166**	ITGB1BP2	16.87476881	14.01281628	13.02773151	6.697869223	15.02525633	6.449489806	10.21394535	4.983937908	7.972361335	9.491663286	5.518417529	6.766601734
**ENSG00000147168**	IL2RG	9.843615139	24.52242849	35.82626166	4.186168264	2.311577897	10.62268909	4.08557814	13.29050109	3.28273702	0	1.379604382	0.845825217
**ENSG00000147174**	GCNA	1.406230734	21.01922442	21.17006371	5.86063557	16.18104528	16.69279715	3.064183605	13.29050109	13.13094808	2.53111021	4.138813147	6.766601734

**ENSG00000147180**	ZNF711	219.3719945	210.1922442	134.3484812	188.3775719	181.4588649	148.3382655	41.87717593	21.59706427	26.26189616	29.74054496	66.22101035	38.06213475
**ENSG00000147202**	DIAPH2	5.624922937	3.50320407	15.47043117	8.372336529	13.86946738	13.65774312	10.21394535	9.967875817	8.910286198	11.38999594	5.518417529	9.304077385
**ENSG00000147224**	PRPS1	257.3402244	178.6634076	303.7089909	371.7317419	386.0335087	386.2106249	202.2361179	154.5020752	207.7503571	211.98048	197.2834267	244.4434876
**ENSG00000147231**	CXorf57	12.65607661	10.50961221	13.84196473	14.2329721	19.64841212	17.45156065	12.25673442	0	1.406887294	2.53111021	4.138813147	3.383300867
**ENSG00000147246**	HTR2C	7.031153671	10.50961221	1.628466439	0	1.155788948	0.379381753	2.04278907	4.983937908	3.28273702	2.53111021	4.138813147	1.691650434
**ENSG00000147251**	DOCK11	2.812461468	10.50961221	2.442699658	3.348934611	16.18104528	3.035054026	41.87717593	66.45250545	58.62030393	49.35664909	75.87824103	63.43689126
**ENSG00000147255**	IGSF1	44.99938349	35.0320407	43.96859385	18.41914036	30.05051265	36.42064832	14.29952349	8.306563181	9.848211061	8.858885733	8.277626294	7.612426951
**ENSG00000147274**	RBMX	4076.662898	2911.162583	3381.51056	4141.794881	3536.714182	3677.726717	3831.2509	3109.977255	3750.761527	3646.697034	3185.506519	4037.12376
**ENSG00000147316**	MCPH1	701.7091363	585.0350798	815.8616859	633.7858752	744.3280827	657.0891967	289.0546534	300.6975871	255.1155627	244.2521352	240.0511625	236.8310607
**ENSG00000147324**	MFHAS1	347.3389913	571.0222635	470.6268008	467.1763783	589.4523636	457.1550127	124.6101333	235.9063943	169.2954378	153.7649452	238.6715581	223.2978572
**ENSG00000147364**	FBXO25	215.1533023	133.1217547	198.6729055	226.8903199	236.9367344	253.0476295	289.0546534	328.939902	220.4123428	351.1915416	335.2438649	265.5891181
**ENSG00000147381**	MAGEA4	1267.013891	1292.682302	1296.259285	851.466625	803.273319	860.4378165	307.439755	413.6668464	422.5351507	291.7104516	259.3656239	330.7176598
**ENSG00000147383**	NSDHL	281.2461468	392.3588559	307.780157	277.9615728	334.023006	311.4724195	164.4445201	245.8742701	193.6814842	185.4038228	230.3939318	186.9273729
**ENSG00000147394**	ZNF185	1247.326661	1499.371342	1508.774156	1760.702372	2030.721182	2059.284157	358.5094817	343.8917157	365.3217341	405.6104111	467.6858856	411.0710554
**ENSG00000147400**	CETN2	224.9969175	164.6505913	179.9455415	311.4509189	359.4503629	261.7734098	250.241661	242.5516449	282.3153837	302.46767	278.6800852	330.7176598
**ENSG00000147403**	RPL10	8150.513335	7952.27324	7754.757182	7187.65091	6790.260071	7581.185576	5960.858506	6121.937064	6185.614471	5447.581948	5552.907639	5645.037497
**ENSG00000147408**	CSGALNACT	67.49907524	52.54806106	60.25325824	83.72336529	86.68417112	84.22274923	105.2036371	73.09775599	128.9646686	102.5099635	88.29468047	136.1778599
**ENSG00000147416**	ATP6V1B2	959.0493607	1092.99967	1023.491157	949.4229624	941.9679928	937.8316942	435.1140719	526.6361057	397.2111794	424.5937376	484.2411382	472.8162962
**ENSG00000147419**	CCDC25	582.1795239	514.9709984	553.6785892	658.0656512	598.6986752	486.7467895	436.1354664	413.6668464	390.176743	435.9837336	441.4734023	401.766978
**ENSG00000147421**	HMBOX1	521.7116024	697.13761	658.7146745	617.8784358	680.7596905	592.5942987	398.3438686	395.3924074	361.1010722	397.3843029	313.1701948	324.7968832
**ENSG00000147437**	GNRH1	57.6554601	73.56728548	35.82626166	49.39678552	48.54313583	17.0721789	12.25673442	29.90362745	22.04123428	24.67832454	41.38813147	23.68310607
**ENSG00000147439**	BIN3	399.3695285	490.4485699	399.7885107	370.0572746	403.3703429	384.3137161	150.1449966	169.4538889	188.5228974	146.8043922	194.5242179	174.2399947
**ENSG00000147454**	SLC25A37	1672.008343	1639.499505	1623.58104	1506.183342	1631.973995	1703.044691	462.6917243	609.7017375	533.679247	454.3342826	607.0259282	495.653577
**ENSG00000147457**	CHMP7	734.0524432	858.2849973	740.9522297	792.8602693	846.0375101	726.5160576	274.7551299	322.2946514	274.3430224	210.7149249	307.6517773	257.1308659
**ENSG00000147459**	DOCK5	6979.123134	5993.982164	6624.601473	8544.806661	8201.478377	8619.174053	2628.048138	4020.376579	2563.34865	2730.435139	4330.578156	2783.610788
**ENSG00000147465**	STAR	2.812461468	0	0.814233219	0	0	0	4.08557814	0	0.937924863	0.632777552	0	0
**ENSG00000147471**	PLPBP	365.6199909	325.7979785	388.3892457	324.0094237	303.9724934	325.8889261	271.6909463	172.7765142	230.7295163	162.623831	234.532745	192.8481494
**ENSG00000147475**	ERLIN2	1026.548436	599.047896	924.1547041	973.7027383	947.7469376	1013.708045	723.1473307	657.8798039	732.5193179	727.6941852	706.3574437	809.4547325
**ENSG00000147481**	SNTG1	0	0	0	0	0	0	9.192550814	14.95181373	11.72406079	10.75721839	17.93485697	12.68737825
**ENSG00000147485**	PXDNL	0	3.50320407	3.256932878	5.023401917	3.467366845	0.758763507	12.25673442	3.322625272	1.406887294	1.265555105	4.138813147	1.691650434
**ENSG00000147509**	RGS20	388.1196826	532.4870187	442.9428714	275.4498718	276.2335586	294.7796223	45.96275407	34.88756536	38.45491938	28.47498986	33.11050518	35.5246591
**ENSG00000147526**	TACC1	1667.789651	2210.521768	2018.484151	1445.902519	1794.940237	1657.898262	2389.041817	2069.995545	2460.645878	2209.026435	1816.938972	2265.965756
**ENSG00000147533**	GOLGA7	620.1477538	539.4934268	640.8015437	718.3464742	606.7891978	705.2706794	366.680638	421.9734096	355.0045606	377.1354212	402.8444796	415.3001814
**ENSG00000147535**	PLPP5	400.7757592	276.7531216	340.3494857	201.7733103	201.107277	286.4332237	141.9738403	139.5502614	128.0267438	106.9394064	126.9236032	125.1821321
**ENSG00000147536**	GINS4	357.1826065	521.9774065	333.0213868	532.4806032	580.206052	579.695319	229.8137703	322.2946514	288.8808578	297.4054496	366.9747657	356.9382415
**ENSG00000147548**	NSD3	2581.839628	2785.047236	2607.989002	2613.006231	2519.619907	2529.338149	1844.63853	1805.846835	1775.960728	1609.786093	1741.06073	1881.115282
**ENSG00000147571**	CRH	1.406230734	0	0	0	0	0	6.128367209	0	1.406887294	3.163887762	6.898021912	1.691650434
**ENSG00000147573**	TRIM55	0	7.006408141	0.814233219	0	1.155788948	1.896908767	0	4.983937908	0	0	0	0.845825217
**ENSG00000147576**	ADHFE1	29.53084542	7.006408141	1.628466439	4.186168264	2.311577897	4.55258104	5.106972674	9.967875817	7.972361335	8.226108181	13.79604382	5.074951301
**ENSG00000147586**	MRPS28	77.34269038	45.54165292	39.89742775	55.25742109	35.8294574	50.45777319	52.09112128	51.50069172	48.30313044	38.5994307	62.0821972	52.44116344
**ENSG00000147592**	LACTB2	428.9003739	483.4421617	376.1757474	312.2881525	401.058765	381.2786621	236.9635321	196.0348911	215.7227185	219.5738107	140.719647	208.9188285
**ENSG00000147601**	TERF1	1155.921663	1215.611812	1153.768472	1305.247265	1214.734185	1256.512367	867.1639601	912.0606372	753.6226273	786.5424976	749.1251796	784.9258012
**ENSG00000147604**	RPL7	13705.12474	12271.72386	12425.19893	16696.95074	14571.03127	14648.30888	8505.152292	9371.46458	8965.623765	8898.750719	9265.423032	9504.537961
**ENSG00000147606**	SLC26A7	0	0	2.442699658	0	4.623155793	7.208253313	1.021394535	3.322625272	0	0	0	1.691650434
**ENSG00000147614**	ATP6V0D2	0	0	1.628466439	0	2.311577897	1.896908767	1.021394535	1.661312636	4.689624315	1.898332657	0	1.691650434
**ENSG00000147642**	SYBU	0	0	1.628466439	1.674467306	0	0.379381753	1.021394535	0	1.406887294	3.163887762	0	1.691650434
**ENSG00000147649**	MTDH	2518.559245	2154.470503	2273.339149	2313.276583	2296.55264	2427.284458	1676.108432	1887.251155	1857.091229	1734.443271	1696.91339	2033.363821
**ENSG00000147650**	LRP12	134.9981505	227.7082646	170.9889761	164.9350296	261.2083023	258.7383558	76.60459012	71.43644335	70.81332715	101.2444084	103.4703287	95.5782495
**ENSG00000147654**	EBAG9	305.1520693	301.2755501	262.1830967	280.4732737	290.103026	281.1218792	240.0277157	245.8742701	242.9225395	242.3538026	235.9123494	271.5098946
**ENSG00000147669**	POLR2K	604.6792157	669.1119775	562.6351546	778.6272972	619.5028763	664.6768318	385.0657396	294.0523366	398.6180667	370.8076457	302.1333597	382.312998
**ENSG00000147676**	MAL2	10940.47511	10211.83987	10179.54371	11089.99697	9876.216563	10991.82754	3505.426044	2967.104368	3596.941849	3013.286704	2410.168856	3262.347861
**ENSG00000147677**	EIF3H	2470.7474	2441.733237	2360.462103	2386.953144	2540.424108	2469.395832	1650.573568	1837.411776	1676.540692	1654.080522	1739.681126	1777.07878
**ENSG00000147679**	UTP23	416.2442973	294.2691419	376.9899806	429.5008639	412.6166545	433.2539623	255.3486337	250.8582081	240.1087649	265.766572	215.2182836	254.5933902
**ENSG00000147684**	NDUFB9	997.0175905	858.2849973	1107.357178	1289.339825	1028.652164	1248.54535	1047.950793	1134.67653	1146.613145	1142.79626	1109.201923	1240.825593
**ENSG00000147687**	TATDN1	674.9907524	616.5639164	564.2636211	570.1561176	454.2250567	563.3819037	456.5633571	418.6507843	457.7073331	448.6392846	419.3997322	417.8376571
**ENSG00000147689**	FAM83A	2096.690025	5394.934268	2800.148042	2771.243391	4797.679924	3835.928908	554.6172324	1390.518676	503.1966889	485.3403827	1300.966933	530.3324109
**ENSG00000147697**	GSDMC	167.3414574	189.1730198	177.5028418	37.67551438	35.8294574	46.2845739	15.32091802	16.61312636	26.26189616	10.75721839	2.759208765	28.75805737
**ENSG00000147789**	ZNF7	414.8380666	490.4485699	442.1286382	400.1976861	346.7366845	360.4126656	501.5047166	500.0551035	455.8314834	477.747052	427.6773585	466.8955197
**ENSG00000147799**	ARHGAP39	73.12399818	115.6057343	59.43902502	134.7946181	82.06101533	92.94852956	142.9952349	86.38825708	115.8337206	118.3294023	137.9604382	117.5697051
**ENSG00000147804**	SLC39A4	168.7476881	147.134571	203.5583049	307.2647506	271.6104028	295.9177676	72.51901198	41.5328159	91.91663656	84.15941447	64.84140597	65.12854169
**ENSG00000147813**	NAPRT	480.9309111	644.589549	477.9548998	453.7806399	531.6629162	544.412816	86.81853546	89.71088235	164.6058134	99.97885328	104.8499331	120.1071808
**ENSG00000147852**	VLDLR	28.12461468	66.56087734	55.36785892	51.90848648	112.111528	97.5011106	24.51346884	24.91968954	6.56547404	8.226108181	22.07367012	6.766601734
**ENSG00000147853**	AK3	659.5222143	585.0350798	631.8449783	681.5081934	701.5638916	620.6685484	633.2646116	511.6842919	647.1681554	635.9414401	491.1391601	617.4524082
**ENSG00000147854**	UHRF2	1032.173359	980.8971397	1082.930182	968.6793364	968.5511386	921.8976605	661.8636586	556.5397331	550.0929321	563.1720216	568.3970055	580.2360987
**ENSG00000147862**	NFIB	457.0249886	476.4357536	319.9936552	552.5742109	564.0250068	429.080763	904.9555579	1036.659085	1079.551517	879.5607978	961.5842545	1122.410063
**ENSG00000147872**	PLIN2	592.0231391	816.2465484	530.0658259	370.0572746	773.2228064	417.6993104	1111.277254	2252.739935	1237.591857	1023.83408	2109.415101	1090.268704
**ENSG00000147874**	HAUS6	1058.891743	921.3426705	1060.945885	1105.985655	1059.858466	1154.079294	675.1417876	702.7352451	679.9955256	693.5241974	721.5330919	790.0007525

**ENSG00000147883**	CDKN2B	194.0598413	371.3396315	332.2071535	298.8924141	477.3408356	452.9818134	144.0166294	225.9385185	131.7784432	130.9849533	223.4959099	143.7902869
**ENSG00000147889**	CDKN2A	843.7384405	868.7946095	788.9919896	534.1550705	628.7491879	647.6046529	332.9746184	299.0362745	314.2048291	255.0093536	246.9491844	298.5763015
**ENSG00000147894**	C9orf72	265.7776088	199.682632	289.0527929	212.6573478	250.8062018	195.7609847	279.8621026	280.7618355	255.1155627	333.4737701	233.1531406	279.1223215
**ENSG00000147905**	ZCCHC7	660.9284451	599.047896	578.1055858	655.5539502	555.9344841	523.9262013	758.8961394	578.1367974	624.6579587	676.4392035	590.4706756	644.5188152
**ENSG00000147912**	FBXO10	22.49969175	28.02563256	39.89742775	36.83828073	42.76419109	34.52373955	12.25673442	9.967875817	8.910286198	12.65555105	6.898021912	15.2248539
**ENSG00000147955**	SIGMAR1	940.7683612	798.7305281	906.2415732	1273.432386	1101.466868	1285.34538	1020.37314	614.6856754	996.5451668	986.5002042	815.3461899	1154.551421
**ENSG00000147996**	CBWD5	416.2442973	416.8812844	301.2662912	370.0572746	300.5051265	462.845739	568.9167559	410.3442211	541.1826459	580.2570155	491.1391601	539.6364883
**ENSG00000148019**	CEP78	2074.190333	1958.291075	1880.064504	2838.222083	2725.35034	2498.987609	2590.25654	2709.60091	2388.425663	2650.072389	2648.840414	3052.583207
**ENSG00000148057**	IDNK	70.31153671	94.5865099	70.02405687	83.72336529	83.21680428	50.83715494	104.1822426	141.2115741	129.4336311	96.81496551	137.9604382	138.7153356
**ENSG00000148082**	SHC3	5.624922937	0	9.770798633	2.511700959	11.55788948	7.208253313	16.34231256	121.2758224	13.59991051	10.75721839	86.91507609	9.304077385
**ENSG00000148090**	AUH	279.8399161	343.3139989	300.452058	372.5689755	323.6209055	276.5692982	268.6267627	197.6962037	211.0330942	277.7893455	328.345843	241.0601868
**ENSG00000148110**	MFSD14B	3044.489539	2834.092093	2860.4013	2260.530863	2531.177797	2602.179446	2458.496645	1935.429221	2656.203212	2148.912568	1924.548113	2490.955263
**ENSG00000148120**	C9orf3	707.3340593	571.0222635	658.7146745	696.5783992	604.4776199	673.7819939	1078.592629	1088.159777	1000.296866	1129.507931	1074.711814	948.170068
**ENSG00000148143**	ZNF462	4618.061731	3089.82599	3597.282364	4312.590546	3483.54789	2967.144693	2213.361957	1383.873426	2502.852497	1990.71818	1386.502404	2445.280702
**ENSG00000148153**	INIP	867.644363	851.2785891	872.043778	999.6569815	885.3343344	992.8420484	752.7677722	820.6884422	810.3670816	745.4119567	862.2527389	872.8916237
**ENSG00000148154**	UGCG	4800.871726	4785.37676	3917.276019	3446.890949	3315.958493	3282.790311	2207.23359	4571.932375	2354.191406	2104.618139	3912.558028	2322.636045
**ENSG00000148158**	SNX30	936.5496689	812.7433443	867.9726119	1176.313282	1177.748938	1024.710116	399.3652631	574.8141721	449.2660093	425.8592928	525.6292697	465.2038692
**ENSG00000148175**	STOM	112.4984587	42.03844885	115.6211172	110.5148422	120.2020506	105.4681274	707.8264127	491.7485403	648.5750427	821.9780405	565.6377967	737.559589
**ENSG00000148180**	GSN	2614.182935	2837.595297	2547.735744	3453.588818	4731.799954	3760.811321	3597.351552	6332.923769	3733.409917	3896.644168	6651.072727	3892.487648
**ENSG00000148187**	MRRF	1444.198964	1292.682302	1278.346155	1522.090781	1267.900476	1260.685566	1213.416707	1232.693976	1195.8542	1390.212283	1063.674979	1247.592195
**ENSG00000148200**	NR6A1	161.7165344	175.1602035	147.3762127	118.0499451	125.9809954	124.8165968	168.5300983	210.9867048	161.3230764	172.7482718	173.8301522	132.794559
**ENSG00000148218**	ALAD	239.0592248	311.7851623	252.412298	353.3126015	355.9829961	302.3672574	493.3335603	558.2010457	461.927995	549.2509155	655.3120816	455.0539666
**ENSG00000148219**	ASTN2	95.62368992	108.5993262	121.3207497	148.1903566	169.9009754	184.0001504	216.5356414	367.1500926	269.6533981	311.3265558	372.4931832	383.1588232
**ENSG00000148225**	WDR31	109.6859973	266.2435094	113.1784175	127.2595152	198.7956991	106.9856544	98.05387535	107.9853213	71.28228958	103.142741	86.91507609	55.82446431
**ENSG00000148229**	POLE3	1416.074349	1008.922772	1275.089222	1760.702372	1512.927733	1647.275573	1405.43888	1262.597603	1476.762697	1532.587232	1461.001041	1683.192181
**ENSG00000148248**	SURF4	2813.867699	2623.899849	2988.235915	2755.335952	2692.988249	2997.115851	2065.25975	2257.723872	2330.274322	2286.858074	2182.534133	2617.829046
**ENSG00000148288**	GBGT1	4.218692202	17.51602035	2.442699658	0	3.467366845	4.173199286	0	3.322625272	0	0	0	1.691650434
**ENSG00000148290**	SURF1	300.9333771	231.2114686	320.8078885	314.7998535	315.5303829	328.5445984	312.5467277	365.4887799	357.8183352	274.6254577	379.3912051	293.5013502
**ENSG00000148291**	SURF2	164.5289959	147.134571	236.1276336	225.2158526	202.2630659	207.5218191	167.5087037	126.2597603	195.0883715	165.1549412	144.8584601	201.3064016
**ENSG00000148296**	SURF6	518.8991409	353.8236111	577.2913526	623.7390714	517.7934488	605.87266	452.477779	445.2317865	433.3212867	495.4648235	400.0852709	569.2403709
**ENSG00000148297**	MED22	593.4293698	493.9517739	716.5252331	709.136904	618.3470873	795.1841549	608.7511428	451.877037	615.7476725	620.1220013	502.1759952	648.7479413
**ENSG00000148300**	REXO4	395.1508363	374.8428355	497.4964971	466.3391446	488.8987251	584.2479001	476.9912478	460.1836002	480.2175298	464.4587234	434.5753804	498.1910527
**ENSG00000148303**	RPL7A	15436.19477	15459.63956	14600.83009	17020.96016	16044.66218	15733.34069	12234.26374	13709.15187	13213.01651	13135.19643	14059.54826	14285.14209
**ENSG00000148308**	GTF3C5	870.4568244	1082.490058	1132.598408	1043.193131	1025.184797	1165.460746	709.8692017	769.1877505	809.4291567	755.5363975	782.2356848	806.0714316
**ENSG00000148331**	ASB6	431.7128354	550.0030391	512.9669283	457.1295745	463.4713683	571.3489205	376.8945834	393.7310948	398.6180667	321.4509966	431.8161717	374.700571
**ENSG00000148334**	PTGES2	404.9944514	395.86206	381.0611467	573.5050522	494.6776699	520.1323838	420.8145484	309.0041503	414.5627894	444.2098418	357.317535	411.0710554
**ENSG00000148335**	NTMT1	423.275451	399.365264	486.097232	525.782734	394.1240314	514.4416575	259.4342119	282.4231481	287.4739705	346.7620987	326.9662386	324.7968832
**ENSG00000148337**	CIZ1	1098.266203	1170.07016	1389.896106	1249.989844	1462.07302	1619.580705	967.2606245	890.463573	1090.337653	930.183002	1020.907243	952.3991941
**ENSG00000148339**	SLC25A25	407.8069129	497.454978	582.1767519	443.733836	464.6271572	546.6891065	258.4128173	279.1005229	361.1010722	287.9137863	274.5412721	301.9596024
**ENSG00000148341**	SH3GLB2	1352.793966	1163.063751	1139.926507	1182.173918	1353.428858	1144.974131	1407.481669	1026.691209	1374.059924	1439.568932	1019.527639	1128.330839
**ENSG00000148343**	MIGA2	216.5595331	119.1089384	195.4159727	164.9350296	162.9662417	207.1424373	162.401731	132.9050109	212.9089439	197.4265963	184.8669872	208.9188285
**ENSG00000148344**	PTGES	673.5845217	479.9389576	725.4817985	1276.781321	1411.218306	1609.337398	1902.858018	1500.16531	2229.447399	2281.163076	1690.015368	2205.912165
**ENSG00000148346**	LCN2	4.218692202	10.50961221	7.328098975	0	0	1.896908767	0	13.29050109	1.406887294	1.898332657	4.138813147	0
**ENSG00000148356**	LRSAM1	412.0256051	381.8492437	495.0537974	428.6636303	448.4461119	403.2828038	394.2582905	441.9091612	405.1835408	486.6059378	481.4819294	400.9211528
**ENSG00000148357**	HMCN2	25.31215321	70.06408141	12.21349829	4.186168264	10.40210053	9.484543833	6.128367209	6.645250545	4.689624315	0.632777552	4.138813147	3.383300867
**ENSG00000148358**	GPR107	3023.396078	3373.58552	3502.017077	2865.01356	3570.232061	3403.433709	2527.951474	2668.068094	2631.817165	2398.859701	2657.11804	2762.465158
**ENSG00000148362**	PAXX	199.6847643	220.7018564	157.9612446	245.3094603	231.1577897	251.5301024	241.0491102	186.0670152	259.3362246	273.3599026	213.8386793	261.359992
**ENSG00000148384**	INPP5E	175.7788418	248.727489	181.5740079	138.9807864	147.9409854	147.9588838	174.6584655	124.5984477	140.6887294	165.7877187	129.6828119	122.6446564
**ENSG00000148396**	SEC16A	3737.761291	3201.92852	3992.999708	3274.420816	3612.996252	3813.545384	3138.745406	2942.184679	3549.107681	2977.851161	3080.656586	3065.270586
**ENSG00000148399**	DPH7	407.8069129	252.2306931	385.946546	404.3838543	265.8314581	366.8621554	465.7559079	264.1487091	405.6525032	429.6559581	364.2155569	377.2380467
**ENSG00000148400**	NOTCH1	1825.287493	2017.845545	1856.45174	1985.080991	1997.203303	2202.69046	737.4468542	659.5411165	831.9393534	813.7519324	633.2384115	830.6003629
**ENSG00000148408**	CACNA1B	0	0	4.071166097	1.674467306	2.311577897	2.655672273	0	1.661312636	0	0	1.379604382	0
**ENSG00000148411**	NACC2	279.8399161	343.3139989	313.4797895	419.4540601	495.8334588	456.3962492	302.3327823	302.3588998	331.0874766	353.7226518	343.5214912	301.9596024
**ENSG00000148426**	PROSER2	468.2748345	339.8107948	522.7377269	397.6859851	309.7514381	479.9179179	612.8367209	676.1542429	563.6928426	704.9141934	680.1449605	598.8442535
**ENSG00000148429**	USP6NL	1025.142205	1215.611812	1078.044783	1053.239935	848.349088	1095.654504	666.9706313	538.2652941	610.5890858	599.2403421	520.1108521	693.5766778
**ENSG00000148444**	COMMD3	88.59253625	42.03844885	67.58135721	69.49039319	73.97049269	71.70315137	74.56180105	89.71088235	89.10286198	86.69052468	81.39665856	70.20349299
**ENSG00000148450**	MSRB2	75.93645964	84.07689769	68.39559043	78.69996337	78.59364848	77.01449592	52.09112128	39.87150327	30.01359561	43.02887356	42.76773585	53.28698866
**ENSG00000148459**	PDSS1	161.7165344	136.6249587	148.1904459	236.9371238	98.2420606	160.8578634	142.9952349	83.06563181	145.8473162	155.6632779	117.2663725	162.3984416
**ENSG00000148481**	MINDY3	201.090995	231.2114686	227.9853014	228.5647872	218.4441112	219.2826534	271.6909463	307.3428377	266.8396235	265.1337944	220.7367012	285.8889233
**ENSG00000148483**	TMEM236	109.6859973	45.54165292	106.6645517	162.4233287	116.7346838	172.6186978	25.53486337	48.17806645	33.76529506	18.98332657	16.55525259	33.83300867
**ENSG00000148484**	RSU1	409.2131436	357.3268152	403.0454436	353.3126015	451.9134788	409.3529118	331.9532238	310.665463	360.1631474	360.6832049	369.7339745	375.5463962
**ENSG00000148488**	ST8SIA6	11.24984587	70.06408141	25.2412298	19.25637402	67.035759	28.4536315	1.021394535	3.322625272	0.468962431	0	5.518417529	1.691650434
**ENSG00000148498**	PARD3	324.8392996	315.2883663	255.6692309	282.147741	246.183046	264.0497003	879.4206945	797.4300653	876.021822	929.5502244	938.13098	901.6496811
**ENSG00000148513**	ANKRD30A	0	0	1.628466439	0	0	1.13814526	4.08557814	21.59706427	6.56547404	6.960553076	12.41643944	12.68737825
**ENSG00000148516**	ZEB1	28.12461468	45.54165292	91.19412058	13.39573845	11.55788948	9.484543833	6.128367209	4.983937908	1.875849726	1.898332657	0	2.53747565
**ENSG00000148572**	NRBF2	607.4916772	634.0799367	614.7460807	478.8976494	460.0040014	508.7509312	257.3914228	315.6494009	296.8532191	265.1337944	325.5866342	299.4221267
**ENSG00000148606**	POLR3A	1207.952201	1110.51569	1284.045787	1215.663264	1061.014255	1233.749462	999.9452496	892.1248856	1002.641678	955.4941041	1008.490803	1047.131618

**ENSG00000148634**	HERC4	5811.951624	4785.37676	5681.719405	4342.730957	3823.349841	4320.399407	4755.612954	4136.668464	3710.89972	4135.834082	3624.220712	3653.119111
**ENSG00000148655**	LRMDA	0	3.50320407	1.628466439	1.674467306	10.40210053	3.41443578	1.021394535	0	0.468962431	1.265555105	0	1.691650434
**ENSG00000148660**	CAMK2G	800.1452877	795.227324	856.5733469	997.1452806	879.5553896	816.0501514	2561.657493	1785.911084	2206.937202	2522.251324	1850.049477	2227.903621
**ENSG00000148671**	ADIRF	6543.191606	6792.712693	5306.357891	5792.819644	6673.525387	5408.086893	1485.107654	1734.410392	2046.083088	1330.731193	1513.426007	1509.798012
**ENSG00000148672**	GLUD1	3089.488923	2676.44791	2869.357865	3615.174913	3127.564894	3509.281218	3476.826997	3093.364128	3920.525927	3205.65108	2949.594169	3704.714449
**ENSG00000148677**	ANKRD1	94.21745919	35.0320407	166.91781	40.18721534	25.42735686	104.3299822	50.04833221	44.85544118	67.0616277	24.67832454	26.21248326	58.36193996
**ENSG00000148680**	HTR7	0	7.006408141	2.442699658	15.07020575	12.71367843	3.793817533	30.64183605	14.95181373	19.69642212	32.27165517	22.07367012	21.14563042
**ENSG00000148688**	RPP30	752.3334428	735.6728548	683.9559043	776.1155962	755.8859722	780.3882665	706.8050181	790.7848148	679.5265632	685.2980892	678.7653561	765.4718212
**ENSG00000148690**	FRA10AC1	701.7091363	788.2209158	653.015042	627.9252397	594.0755194	605.4932783	1069.400078	985.1583932	989.5107304	990.929647	1022.286847	1007.377833
**ENSG00000148700**	ADD3	216.5595331	231.2114686	167.7320432	242.7977593	277.3893476	223.4558527	30.64183605	44.85544118	47.83416801	34.80276538	57.94338406	59.20776517
**ENSG00000148719**	DNAJB12	756.552135	791.7241199	808.5335869	765.2315587	706.1870474	881.3038129	774.2170574	725.993622	745.1813036	748.5758445	670.4877298	740.0970647
**ENSG00000148730**	EIF4EBP2	2303.405943	2714.983155	2371.861368	2901.851841	3057.061768	2796.802285	3593.265974	3669.839613	3353.550347	3980.803582	4118.119081	3805.36765
**ENSG00000148734**	NPFFR1	0	17.51602035	4.885399317	0.837233653	0	0	0	0	0	0	2.759208765	0
**ENSG00000148735**	PLEKHS1	8.437384405	7.006408141	8.142332194	5.86063557	8.090522638	9.863925586	3.064183605	28.24231481	3.751699452	5.062220419	1.379604382	1.691650434
**ENSG00000148737**	TCF7L2	451.4000657	353.8236111	403.8596768	426.989163	435.7324335	394.9364052	449.4135953	430.2799728	400.024954	387.8926396	539.4253135	406.8419293
**ENSG00000148773**	MKI67	20410.03288	12853.25573	19090.51206	26044.66447	23075.32635	23813.41327	22242.90879	20573.69569	23815.78812	23659.55268	24016.15309	28682.77893
**ENSG00000148803**	FUOM	85.78007478	28.02563256	48.85399317	43.53614995	62.41260321	56.907263	59.24088302	46.51675381	64.24785311	73.40219608	55.18417529	70.20349299
**ENSG00000148814**	LRRC27	118.1233817	147.134571	100.150686	94.60740277	123.6694175	76.25573241	175.67986	191.0509532	163.6678886	160.0927208	217.9774924	129.4112582
**ENSG00000148824**	MTG1	139.2168427	80.57369362	95.26528668	87.0722999	65.87997005	97.5011106	131.759895	88.04956971	107.8613592	98.71329817	126.9236032	115.8780547
**ENSG00000148832**	PAOX	1.406230734	14.01281628	1.628466439	2.511700959	3.467366845	2.655672273	7.149761744	8.306563181	5.627549177	3.163887762	0	3.383300867
**ENSG00000148834**	GSTO1	1991.22272	2287.592258	1906.119967	1063.286739	1024.029008	963.2502716	786.4737919	963.561329	804.2705699	662.5180973	594.6094888	644.5188152
**ENSG00000148835**	TAF5	213.7470716	210.1922442	313.4797895	305.5902833	231.1577897	282.2600245	233.8993485	320.6333388	333.9012512	277.1565679	303.5129641	345.9425137
**ENSG00000148840**	PPRC1	1177.015124	504.4613861	1135.041108	1256.687713	761.6649169	1249.304114	883.5062727	554.8784205	922.4491027	963.7202123	664.9693123	903.3413315
**ENSG00000148841**	ITPRIP	319.2143767	357.3268152	542.2793242	106.3286739	213.8209554	199.1754205	375.8731888	250.8582081	300.6049186	144.2732819	110.3683506	169.1650434
**ENSG00000148842**	CNNM2	241.8716863	248.727489	288.2385597	301.404115	365.2293077	299.7115851	488.2265877	622.9922385	444.576385	554.9459134	623.5811808	478.7370727
**ENSG00000148843**	PDCD11	1565.134807	949.3683031	1475.390594	1743.957699	1359.207803	1683.696221	1534.134591	1159.59622	1730.940334	1657.877187	1280.272867	1840.515672
**ENSG00000148848**	ADAM12	0	7.006408141	8.142332194	0	10.40210053	12.89897961	53.11251581	124.5984477	47.83416801	57.58275727	107.6091418	56.67028952
**ENSG00000148908**	RGS10	473.8997574	462.4229373	452.71367	511.5497619	596.3870973	568.3138665	31.66323058	34.88756536	47.36520558	34.16998783	34.49010956	52.44116344
**ENSG00000148925**	BTBD10	812.8013643	1071.980446	914.3839054	762.7198578	708.4986253	952.6275825	1343.133813	1229.371351	1324.349906	1062.43351	961.5842545	1222.217438
**ENSG00000148926**	ADM	1112.328511	2578.358196	1263.689957	874.9091672	2059.615906	1175.324672	951.9397065	4070.215959	963.2488342	811.2208222	4667.201625	841.5960907
**ENSG00000148935**	GAS2	25.31215321	17.51602035	8.142332194	16.74467306	16.18104528	11.3814526	39.83438686	18.274439	23.917084	25.94387965	28.97169203	29.60388259
**ENSG00000148942**	SLC5A12	0	3.50320407	2.442699658	7.535102876	3.467366845	0.758763507	5.106972674	14.95181373	8.910286198	10.75721839	6.898021912	0.845825217
**ENSG00000148943**	LIN7C	1345.762813	1128.031711	1322.314748	1344.597247	1159.256315	1342.252643	1244.058543	1319.082233	1372.653037	1393.37617	1252.680779	1551.243448
**ENSG00000148950**	IMMP1L	189.8411491	126.1153465	187.2736405	245.3094603	205.7304328	270.1198084	307.439755	317.3107135	326.3978523	367.6437579	324.2070298	387.3879493
**ENSG00000148965**	SAA4	0	0	7.328098975	1.674467306	0	0	0	1.661312636	0	0	0	0
**ENSG00000148985**	PGAP2	246.0903785	192.6762239	246.7126655	294.7062458	275.0777697	277.3280617	276.797919	267.4713344	303.4186932	351.1915416	355.9379306	334.9467858
**ENSG00000149016**	TUT1	61.8741523	45.54165292	61.06749146	61.11805666	60.10102531	59.94231702	65.36925023	56.48462963	73.62710174	62.64497769	53.80457091	53.28698866
**ENSG00000149043**	SYT8	555.46114	388.8556518	258.9261638	537.5040051	509.7029262	343.7198685	1424.845376	1252.629728	1267.13649	1309.216756	1307.864954	888.9623028
**ENSG00000149050**	ZNF214	43.59315276	70.06408141	60.25325824	80.37443068	58.94523636	66.39180683	138.9096567	196.0348911	118.1785327	127.188288	161.4137127	164.0900921
**ENSG00000149054**	ZNF215	202.4972257	136.6249587	229.6137679	205.122245	321.3093276	244.7012309	109.2892152	174.4378268	112.0820211	113.2671819	131.0624163	128.5654329
**ENSG00000149084**	HSD17B12	679.2094446	679.6215897	622.0741797	699.0901001	709.6544142	727.2748211	921.2978705	744.268061	927.6076894	868.8035794	772.5784541	1007.377833
**ENSG00000149089**	APIP	382.4947597	168.1537954	340.3494857	387.6391813	331.7114282	341.8229597	452.477779	367.1500926	415.9696767	432.1870683	382.1504139	460.1289179
**ENSG00000149090**	PAMR1	0	10.50961221	0.814233219	2.511700959	4.623155793	5.6907263	9.192550814	1.661312636	5.158586746	8.858885733	8.277626294	8.458252168
**ENSG00000149091**	DGKZ	424.6816817	385.3524477	409.5593094	464.6646773	431.1092777	604.7345148	566.8739669	637.9440523	607.7753112	580.2570155	765.6804322	594.6151274
**ENSG00000149100**	EIF3M	2770.274546	2368.165952	2574.60544	2435.512696	2153.234811	2513.024734	2925.273948	2599.954276	2998.076824	2784.854008	2589.517426	3124.478351
**ENSG00000149115**	TNKS1BP1	2047.471949	2462.752462	2197.615459	2255.507461	2809.722933	2629.11555	3726.047263	3169.78451	3736.223691	3648.595367	3541.444449	3146.469806
**ENSG00000149124**	GLYAT	1.406230734	0	0	0	0	1.13814526	15.32091802	9.967875817	22.97915914	12.0227735	4.138813147	13.53320347
**ENSG00000149131**	SERPING1	1134.828202	1163.063751	1082.115949	5.023401917	5.778944741	8.725780326	1052.036371	1302.469107	1144.268333	266.3993496	441.4734023	402.6128032
**ENSG00000149136**	SSRP1	3629.481525	2858.614521	3230.063182	3936.672636	4010.58765	3672.03599	3901.727123	3724.66293	3868.940059	4065.595774	3886.345545	4225.742783
**ENSG00000149150**	SLC43A1	15.46853808	14.01281628	20.35583049	10.04680383	13.86946738	13.27836137	14.29952349	6.645250545	8.441323766	3.796665314	1.379604382	7.612426951
**ENSG00000149177**	PTPRJ	413.4318358	367.8364274	432.3578395	419.4540601	358.294574	515.5798027	674.120393	500.0551035	647.6371178	649.2297687	369.7339745	514.2617318
**ENSG00000149179**	C11orf49	272.8087624	175.1602035	243.4557326	295.5434795	337.4903729	254.9445382	460.6489352	468.4901634	419.7213762	467.6226112	510.4536215	386.5421241
**ENSG00000149182**	ARFGAP2	815.6138258	623.5703245	871.2295448	825.5123817	809.0522638	908.2399174	1125.576777	1049.949586	1133.951159	1181.39569	1212.672252	988.7696784
**ENSG00000149187**	CELF1	3169.644075	2354.153135	3242.27668	2873.385897	2671.028259	3219.054177	3001.878538	2855.796422	3178.158398	3023.411145	2749.551534	3035.666703
**ENSG00000149196**	HIKESHI	531.5552175	346.817203	559.3782218	540.0157061	573.2713183	572.107684	404.4722358	435.2639107	518.2034868	472.0520541	509.0740171	543.8656144
**ENSG00000149201**	CCDC81	23.90592248	42.03844885	24.42699658	10.88403749	15.02525633	14.79588838	27.57765244	16.61312636	28.13774589	6.960553076	24.83287888	21.99145564
**ENSG00000149212**	SESN3	284.0586083	280.2563256	232.8707008	458.8040418	412.6166545	405.1797125	153.2091802	220.9545806	466.1486569	199.324929	309.0313816	569.2403709
**ENSG00000149218**	ENDOD1	774.8331345	767.2016914	695.3551694	1484.415267	1555.691924	1439.753754	1121.491199	807.3979412	804.2705699	1221.893454	987.7967377	1085.193753
**ENSG00000149231**	CCDC82	902.8001313	1068.477241	1196.922833	757.6964558	792.8712185	795.5635367	971.3462027	1129.692593	835.6910528	818.8141528	1073.332209	807.763082
**ENSG00000149256**	TENM4	71.71776744	66.56087734	55.36785892	75.35102876	83.21680428	55.76911774	7.149761744	4.983937908	4.689624315	4.429442867	2.759208765	3.383300867
**ENSG00000149257**	SERPINH1	690.4592905	882.8074257	952.6528668	795.3719702	978.9532392	961.3533629	912.1053196	1171.225408	1232.902232	970.0479878	1273.374845	1351.628696
**ENSG00000149260**	CAPN5	5.624922937	17.51602035	18.72736405	1.674467306	16.18104528	12.14021611	1.021394535	8.306563181	0.468962431	1.265555105	8.277626294	0
**ENSG00000149262**	INTS4	644.0536762	676.1183856	618.0030136	570.9933513	518.9492378	608.1489505	505.5902948	461.8449128	464.2728071	480.2781623	480.102325	473.6621214
**ENSG00000149269**	PAK1	482.3371418	637.5831408	520.2950272	442.8966024	552.4671173	537.9633262	54.13391035	49.83937908	52.52379232	72.13664097	62.0821972	68.51184256
**ENSG00000149273**	RPS3	12757.32522	11143.69215	11542.57012	12846.51317	12059.50189	12169.04912	9606.215601	10408.12367	10137.09192	9718.830427	10262.877	10275.08473
**ENSG00000149289**	ZC3H12C	1582.009576	2207.018564	1734.316757	1275.944087	1420.464617	1382.846491	1018.330351	1106.434216	773.7880119	982.0707613	1027.805265	826.3712368
**ENSG00000149292**	TTC12	344.5265299	350.320407	277.6535278	353.3126015	373.3198303	248.1156667	535.2107363	511.6842919	474.5899806	551.7820257	520.1108521	489.7328005

**ENSG00000149294**	NCAM1	14.06230734	7.006408141	9.770798633	1.674467306	11.55788948	3.035054026	2.04278907	0	0	0	0	0
**ENSG00000149295**	DRD2	0	0	0.814233219	0	1.155788948	1.896908767	2.04278907	4.983937908	0.937924863	1.265555105	2.759208765	0.845825217
**ENSG00000149300**	C11orf52	5.624922937	0	4.885399317	2.511700959	3.467366845	4.931962793	5.106972674	1.661312636	17.8205724	5.694997971	8.277626294	11.84155303
**ENSG00000149308**	NPAT	1756.382187	2522.306931	1766.071853	1722.189624	1902.428609	1685.972512	1574.990373	1779.265833	1511.465917	1507.27613	1685.876555	1675.579754
**ENSG00000149311**	ATM	2815.27393	2221.031381	2686.969624	2535.980735	2645.600903	2394.657627	3006.985511	2692.987783	2518.328257	2750.051243	2753.690347	2649.124579
**ENSG00000149313**	AASDHPPT	1404.824503	1022.935589	1056.060486	1404.87807	1208.95524	1321.766029	1222.609258	898.7701361	1089.399728	1125.078488	979.5191114	1272.966951
**ENSG00000149328**	GLB1L2	212.3408409	80.57369362	127.8346155	252.0073295	266.987247	239.7692681	263.51979	214.3093301	266.3706611	351.1915416	339.382678	298.5763015
**ENSG00000149346**	SLX4IP	590.6169083	746.182467	735.2525972	722.5326424	758.1975501	610.4252411	1067.357289	1015.062021	1067.827456	1049.145182	1074.711814	1161.318023
**ENSG00000149357**	LAMTOR1	638.4287533	609.5575083	601.7183492	659.7401185	594.0755194	688.1985005	498.440533	416.9894717	645.2923057	592.9125666	487.000347	623.3731848
**ENSG00000149380**	P4HA3	2.812461468	7.006408141	2.442699658	3.348934611	5.778944741	1.13814526	4.08557814	0	0.937924863	2.53111021	1.379604382	1.691650434
**ENSG00000149403**	GRIK4	0	0	0	0	0	0	6.128367209	11.62918845	7.034436472	3.163887762	4.138813147	2.53747565
**ENSG00000149418**	ST14	2280.906251	2515.300523	2493.996351	2644.821109	2813.1903	3249.025335	1126.598172	878.8343845	1093.151428	1051.043515	1198.876208	1267.892
**ENSG00000149428**	HYOU1	4949.932184	4126.774395	6061.152086	2937.852888	2911.432361	3732.737071	2240.93961	1795.87896	2532.39713	2067.917041	1672.080511	2383.535461
**ENSG00000149474**	KAT14	268.5900702	150.637775	289.0527929	272.9381708	277.3893476	237.1135958	651.6497133	511.6842919	502.7277265	641.0036606	622.2015764	548.9405657
**ENSG00000149476**	TKFC	157.4978422	185.6698157	180.7597747	173.3073661	168.7451864	153.2702283	275.7765244	315.6494009	267.3085859	247.416023	280.0596896	268.9724189
**ENSG00000149480**	MTA2	1728.257572	1355.739975	1599.968276	1699.584315	1531.420356	1706.079745	1475.915103	1466.939058	1651.685684	1491.456691	1350.63269	1505.568886
**ENSG00000149483**	TMEM138	299.5271464	115.6057343	322.4363549	323.17219	169.9009754	333.0971794	368.7234271	201.018829	337.6529506	354.3554293	231.7735362	345.9425137
**ENSG00000149485**	FADS1	1542.635115	2235.044197	1547.85735	1566.464165	2636.354591	1794.475693	1303.299427	2129.8028	1374.997849	1445.896707	2178.39532	1474.273353
**ENSG00000149489**	ROM1	5.624922937	14.01281628	8.956565414	5.86063557	5.778944741	4.55258104	8.171156279	29.90362745	7.503398903	12.0227735	13.79604382	15.2248539
**ENSG00000149499**	EML3	203.9034565	245.2242849	234.4991672	245.3094603	248.4946239	206.7630556	224.7067977	184.4057026	241.0466898	232.2293617	238.6715581	276.5848459
**ENSG00000149503**	INCENP	878.8942088	455.4165292	938.810902	1136.963301	1004.380596	1231.852553	978.4959644	827.3336928	1154.585506	1094.705166	971.2414851	1327.94559
**ENSG00000149527**	PLCH2	0	3.50320407	2.442699658	1.674467306	1.155788948	1.13814526	0	0	1.406887294	0	0	0
**ENSG00000149531**	FRG1BP	239.0592248	196.1794279	138.4196473	157.3999267	167.5893975	118.7464888	428.9857046	392.0697821	421.1282634	373.9715335	368.3543701	395.8462015
**ENSG00000149532**	CPSF7	1452.636348	1061.470833	1567.398947	1490.275902	1277.146788	1570.261077	1689.386561	1814.153399	1739.381658	1792.658806	1832.11462	1730.558394
**ENSG00000149541**	B3GAT3	282.6523776	252.2306931	243.4557326	241.123292	294.7261818	292.12395	200.1933288	184.4057026	216.1916809	241.0882475	233.1531406	216.5312555
**ENSG00000149547**	EI24	1957.473182	1646.505913	2004.642186	2052.059683	2102.380097	2298.674043	1332.919868	1054.933524	1487.548833	1306.052868	1178.182142	1473.427528
**ENSG00000149548**	CCDC15	407.8069129	767.2016914	508.0815289	468.013612	509.7029262	481.4354449	373.8303998	405.3602832	271.0602854	337.2704354	412.5017103	376.3922215
**ENSG00000149554**	CHEK1	1559.509884	1586.951444	1529.129986	1476.04293	1554.536135	1604.784816	1089.827969	1128.03128	1110.972	1097.869053	1103.683506	1278.041903
**ENSG00000149557**	FEZ1	313.5894537	231.2114686	412.8162423	151.5392912	137.5388848	167.686735	60.26227756	46.51675381	158.9782643	44.92720622	35.86971394	141.2528112
**ENSG00000149564**	ESAM	8.437384405	10.50961221	13.84196473	10.04680383	13.86946738	14.79588838	6.128367209	6.645250545	5.627549177	5.694997971	5.518417529	5.920776517
**ENSG00000149571**	KIRREL3	9.843615139	0	12.21349829	27.62871054	24.27156791	28.4536315	113.3747934	61.46856754	133.654293	91.11996754	97.95191114	155.6318399
**ENSG00000149573**	MPZL2	10238.76598	11886.37141	9250.503606	6485.211875	7773.836466	8152.913879	2260.346106	2001.881727	2726.547576	1860.998782	1676.219325	2466.426332
**ENSG00000149575**	SCN2B	2.812461468	0	6.513865756	0.837233653	0	7.208253313	0	1.661312636	0	0	1.379604382	1.691650434
**ENSG00000149577**	SIDT2	147.6542271	108.5993262	179.1313083	255.3562641	250.8062018	259.1177375	227.7709813	206.0027669	265.4327362	249.9471332	227.6347231	260.5141668
**ENSG00000149582**	TMEM25	97.02992066	105.0961221	87.9371877	140.6552537	142.1620406	184.3795321	116.438977	131.2436983	129.9025935	174.0138269	162.7933171	145.4819373
**ENSG00000149591**	TAGLN	23.90592248	3.50320407	13.84196473	30.97764516	11.55788948	23.5216687	92.94690267	51.50069172	78.78568848	89.22163489	80.01705417	109.9572782
**ENSG00000149596**	JPH2	1718.413957	711.1504263	1600.782509	2033.640543	1381.167793	2006.170711	819.158417	799.091378	1082.834254	840.3285896	757.4028059	982.8489019
**ENSG00000149600**	COMMD7	32.34330689	56.05126513	57.81055858	46.88508456	61.25681426	58.42479001	116.438977	114.6305719	115.8337206	128.4538431	142.0992514	158.1693155
**ENSG00000149609**	C20orf144	1.406230734	0	3.256932878	0	3.467366845	1.517527013	8.171156279	4.983937908	5.158586746	3.163887762	15.17564821	10.99572782
**ENSG00000149633**	KIAA1755	25.31215321	24.52242849	10.58503185	10.88403749	18.49262317	15.17527013	9.192550814	29.90362745	9.379248629	3.163887762	34.49010956	5.074951301
**ENSG00000149634**	SPATA25	28.12461468	24.52242849	24.42699658	15.9074394	28.89472371	11.3814526	34.72741419	28.24231481	37.04803208	28.47498986	19.31446135	37.21630954
**ENSG00000149636**	DSN1	693.2717519	1036.948405	777.5927246	862.3506625	918.8522139	847.5388369	768.0886902	918.7058878	802.8636826	810.5880446	917.4369142	829.7545377
**ENSG00000149639**	SOGA1	2397.623402	1429.307261	2585.190472	3811.924822	3385.305829	4431.937642	4658.580474	3430.610594	5325.068409	4890.737702	3850.475831	5498.709734
**ENSG00000149646**	CNBD2	2.812461468	7.006408141	7.328098975	12.55850479	10.40210053	11.76083435	13.27812895	9.967875817	10.78613592	11.38999594	13.79604382	16.07067912
**ENSG00000149656**	LINC00266-1	0	0	1.628466439	0	0	0.758763507	8.171156279	0	1.406887294	1.898332657	8.277626294	0
**ENSG00000149657**	LSM14B	749.5209813	900.3234461	728.7387314	751.8358203	702.7196805	679.0933384	882.4848781	986.8197059	969.8143082	859.3119161	925.7145405	897.420555
**ENSG00000149658**	YTHDF1	1006.861206	875.8010176	1027.562323	908.3985134	922.3195807	1075.547271	952.961101	1041.643023	1151.302769	1073.190729	1193.357791	1199.380157
**ENSG00000149679**	CABLES2	392.3383748	557.0094472	359.891083	423.6402284	449.6019009	387.3487701	352.3811145	347.214341	326.3978523	316.3887762	306.2721729	313.8011554
**ENSG00000149716**	ORAOV1	330.4642225	255.7338971	351.7487508	367.5455736	262.3640913	316.0250005	404.4722358	460.1836002	347.5011617	346.7620987	322.8274255	347.6341641
**ENSG00000149743**	TRPT1	240.4654555	259.2371012	244.2699658	252.8445632	288.9472371	234.8373053	153.2091802	179.4217647	186.1780853	135.4143962	169.691339	140.406986
**ENSG00000149761**	NUDT22	257.3402244	231.2114686	246.7126655	245.3094603	285.4798702	192.7259307	205.3003015	212.6480174	231.1984787	248.0488005	246.9491844	204.6897025
**ENSG00000149781**	FERMT3	2.812461468	14.01281628	19.54159727	5.86063557	13.86946738	15.55465189	3.064183605	1.661312636	0.937924863	1.898332657	0	3.383300867
**ENSG00000149782**	PLCB3	1932.161029	1614.977076	1478.647527	1904.70656	1767.201302	1658.277644	1274.70038	1242.661852	1717.340424	1383.884507	1336.836646	1503.877235
**ENSG00000149792**	MRPL49	961.8618222	900.3234461	966.4948315	1089.240982	1047.144787	1103.242139	787.4951864	1005.094145	970.7522331	804.8930466	928.4737493	1024.294338
**ENSG00000149798**	CDC42EP2	0	0	0.814233219	0	0	1.896908767	0	1.661312636	0	0.632777552	1.379604382	2.53747565
**ENSG00000149806**	FAU	1468.104886	1348.733567	1390.710339	1327.852573	1471.319331	1315.69592	1174.603715	1314.098295	1522.721015	1281.374544	1345.114273	1422.678015
**ENSG00000149809**	TM7SF2	47.81184496	63.05767327	53.73939248	55.25742109	82.06101533	56.907263	28.59904698	69.77513072	29.54463318	37.33387559	28.97169203	37.21630954
**ENSG00000149823**	VPS51	292.4959927	325.7979785	319.179422	329.0328256	314.3745939	394.1776417	368.7234271	267.4713344	342.8115374	343.5982109	286.9577115	278.2764963
**ENSG00000149922**	TBX6	0	3.50320407	6.513865756	6.697869223	12.71367843	3.035054026	5.106972674	9.967875817	4.220661883	8.858885733	9.657230676	0.845825217
**ENSG00000149923**	PPP4C	1700.132958	1755.105239	1699.304729	1688.700278	1589.209804	1882.871642	1618.910338	1628.086383	1939.628616	1720.522165	1787.967279	1841.361497
**ENSG00000149925**	ALDOA	185.6224569	535.9902228	279.2819943	222.7041517	491.210303	183.2413868	209.3858796	322.2946514	258.3982997	201.2232617	387.6688314	230.064459
**ENSG00000149926**	FAM57B	23.90592248	17.51602035	26.05546302	32.65211246	57.78944741	26.55672273	14.29952349	16.61312636	15.47576024	24.04554699	9.657230676	16.07067912
**ENSG00000149927**	DOC2A	73.12399818	129.6185506	34.19779522	30.97764516	35.8294574	25.03919572	10.21394535	11.62918845	6.096511609	6.327775524	13.79604382	3.383300867
**ENSG00000149929**	HIRIP3	458.4312193	567.5190594	460.041769	604.4826974	706.1870474	587.2829541	554.6172324	825.6723802	591.3616261	640.370883	867.7711565	646.2104656
**ENSG00000149930**	TAOK2	1714.195265	1776.124464	1855.637507	1938.195906	1885.091775	2006.929475	1551.498298	1631.409009	1610.41699	1621.808867	1832.11462	1599.455485
**ENSG00000149932**	TMEM219	767.8019808	903.8266502	845.1740818	401.0349197	478.4966246	504.1983501	486.1837986	553.2171078	483.5002668	406.2431886	415.2609191	455.0539666
**ENSG00000149948**	HMGA2	2861.679544	2998.742684	3549.242604	3013.203917	3841.842464	3679.623625	1.021394535	18.274439	11.25509835	1.265555105	8.277626294	0.845825217

**ENSG00000149970**	CNKSR2	16.87476881	94.5865099	49.66822639	21.76807497	70.50312584	61.45984404	226.7495867	197.6962037	160.3851516	272.0943475	165.5525259	174.2399947
**ENSG00000149972**	CNTN5	14.06230734	3.50320407	1.628466439	10.88403749	5.778944741	2.655672273	0	0	0	1.265555105	0	2.53747565
**ENSG00000150048**	CLEC1A	0	0	0.814233219	0	0	0	113.3747934	64.79119281	125.2129692	106.9394064	63.46180159	142.0986364
**ENSG00000150054**	MPP7	89.99876699	59.5544692	52.11092604	85.39783259	117.8904727	69.42686085	201.2147234	151.1794499	208.2193196	210.7149249	198.6630311	287.5805737
**ENSG00000150093**	ITGB1	8995.658006	10369.48405	11332.49795	10497.23554	11544.02002	13576.93481	7202.87426	7284.855909	7311.124306	7336.422942	7354.670962	8621.496435
**ENSG00000150175**	FRMPD2B	11.24984587	3.50320407	8.142332194	15.9074394	12.71367843	3.793817533	8.171156279	13.29050109	7.503398903	11.38999594	2.759208765	6.766601734
**ENSG00000150276**	PPIAP26	14.06230734	42.03844885	56.18209214	7.535102876	28.89472371	39.07632059	0	1.661312636	1.406887294	0	0	0.845825217
**ENSG00000150281**	CTF1	12.65607661	52.54806106	17.09889761	13.39573845	20.80420107	18.58970591	31.66323058	58.14594226	43.61350613	26.5766572	55.18417529	25.3747565
**ENSG00000150316**	CWC15	833.8948254	868.7946095	1044.661221	1080.031412	1064.481621	1030.400842	861.0355929	877.1730719	780.8224484	785.9097201	841.5586732	914.3370593
**ENSG00000150347**	ARID5B	275.6212239	199.682632	216.5860364	207.6339459	172.2125533	196.89913	1573.968978	1103.11159	1547.576024	1410.461164	903.6408704	1342.324619
**ENSG00000150394**	CDH8	57.6554601	108.5993262	52.92515926	67.81592588	91.30732691	67.15057033	54.13391035	44.85544118	100.8269228	69.60553076	63.46180159	108.2656277
**ENSG00000150401**	DCUN1D2	361.4012987	413.3780803	458.4133025	420.2912937	398.7471872	441.6003608	410.600603	408.6829085	426.7558126	442.3115091	484.2411382	466.8955197
**ENSG00000150403**	TMCO3	1383.731042	1607.970668	1634.166071	1705.444951	1935.946488	1861.626263	1545.369931	1558.311253	1434.556078	1756.590485	1867.984334	1603.684611
**ENSG00000150433**	TMEM218	267.1838395	164.6505913	278.4677611	379.2668447	353.6714182	306.1610749	217.5570359	172.7765142	206.8124323	246.7832454	202.8018442	250.3642642
**ENSG00000150455**	TIRAP	392.3383748	252.2306931	381.8753799	339.0796294	396.4356093	324.3713991	207.3430906	217.6319553	209.6262069	205.6527045	233.1531406	226.6811581
**ENSG00000150456**	EEF1AKMT1	40.78069129	21.01922442	35.01202844	51.90848648	52.01050267	51.9753002	37.79159779	19.93575163	22.04123428	24.04554699	20.69406573	31.29553302
**ENSG00000150457**	LATS2	966.0805144	998.4131601	1108.171412	990.4474113	992.8227066	1138.14526	758.8961394	760.8811873	842.2565269	784.644165	600.1279063	775.6217238
**ENSG00000150459**	SAP18	1814.037647	1982.813504	1773.399952	1723.864091	1694.386598	1822.549943	1137.833512	1144.644406	1197.261087	1167.474584	1121.618363	1282.271029
**ENSG00000150471**	ADGRL3	94.21745919	73.56728548	16.28466439	96.28187008	71.65891479	30.72992202	55.15530488	56.48462963	28.13774589	63.27775524	42.76773585	50.74951301
**ENSG00000150477**	KIAA1328	206.7159179	182.1666117	184.8309408	214.3318151	257.7409355	197.2785117	227.7709813	277.4392102	250.4259384	248.0488005	310.410986	227.5269833
**ENSG00000150527**	CTAGE5	556.8673707	532.4870187	653.8292752	488.9444533	456.5366346	528.8581641	1143.961879	702.7352451	1057.04132	933.3468898	624.9607852	880.5040507
**ENSG00000150540**	HNMT	0	0	4.071166097	0.837233653	0	1.896908767	9.192550814	19.93575163	27.19982102	10.75721839	1.379604382	16.07067912
**ENSG00000150551**	LYPD1	0	0	11.39926507	5.023401917	6.93473369	3.035054026	4.08557814	3.322625272	2.344812157	0	1.379604382	4.229126084
**ENSG00000150556**	LYPD6B	120.9358431	108.5993262	94.45105346	78.69996337	95.93048271	60.32169878	40.8557814	73.09775599	60.96511609	17.08499391	37.24931832	62.59106604
**ENSG00000150593**	PDCD4	961.8618222	1100.006078	765.3792263	641.3209781	929.2543144	707.1675882	1715.942819	1726.103829	1435.02504	1695.84384	1960.417827	1595.226359
**ENSG00000150625**	GPM6A	22.49969175	14.01281628	9.770798633	0.837233653	0	0.379381753	28.59904698	1.661312636	10.78613592	3.163887762	1.379604382	2.53747565
**ENSG00000150627**	WDR17	40.78069129	14.01281628	26.05546302	15.07020575	30.05051265	22.38352344	22.47067977	9.967875817	16.88264753	16.45221636	19.31446135	21.99145564
**ENSG00000150630**	VEGFC	563.8985244	669.1119775	651.3865756	363.3594053	329.3998503	367.2415372	1.021394535	1.661312636	2.344812157	1.265555105	0	1.691650434
**ENSG00000150636**	CCDC102B	16.87476881	42.03844885	24.42699658	10.88403749	27.73893476	5.6907263	20.4278907	14.95181373	11.72406079	14.5538837	1.379604382	10.1499026
**ENSG00000150637**	CD226	25.31215321	73.56728548	40.71166097	29.30317785	26.58314581	25.79795922	6.128367209	9.967875817	13.59991051	5.062220419	13.79604382	9.304077385
**ENSG00000150656**	CNDP1	1.406230734	0	3.256932878	0	0	1.13814526	1.021394535	8.306563181	3.751699452	12.65555105	12.41643944	0.845825217
**ENSG00000150667**	FSIP1	11.24984587	3.50320407	3.256932878	17.58190671	16.18104528	8.346398573	11.23533988	8.306563181	8.910286198	20.88165923	23.4532745	10.99572782
**ENSG00000150672**	DLG2	32.34330689	7.006408141	37.45472809	30.1404115	43.91998003	23.90105046	155.2519693	116.2918845	51.58586746	224.0032535	132.4420207	85.42834689
**ENSG00000150681**	RGS18	0	0	1.628466439	3.348934611	0	2.27629052	3.064183605	0	1.875849726	0	0	0
**ENSG00000150687**	PRSS23	507.649295	420.3844885	430.7293731	668.112455	678.4481126	553.1385963	6.128367209	11.62918845	15.00679781	3.796665314	13.79604382	9.304077385
**ENSG00000150712**	MTMR12	1279.669968	1306.695118	1275.089222	1539.672688	1378.856215	1522.838358	1296.149665	1196.145098	1321.536132	1285.803986	1245.782757	1512.335488
**ENSG00000150722**	PPP1R1C	67.49907524	122.6121425	55.36785892	89.58400086	60.10102531	79.67016819	7.149761744	9.967875817	9.848211061	10.75721839	13.79604382	16.07067912
**ENSG00000150750**	C11orf53	0	0	0	0	0	0	2.04278907	0	0.937924863	5.062220419	6.898021912	0
**ENSG00000150753**	CCT5	13176.38198	10579.67629	12357.61757	14884.33988	13037.29934	13796.59684	9668.520667	8238.449363	10700.3158	10049.14031	8717.720092	12048.78021
**ENSG00000150756**	FAM173B	212.3408409	199.682632	260.5546302	348.2891996	338.6461618	347.513686	156.2733638	159.4860131	163.6678886	220.2065882	293.8557334	282.5056224
**ENSG00000150760**	DOCK1	1421.699272	1397.778424	1415.951569	1552.231192	1450.51513	1438.236227	1878.34455	2143.093301	1960.731926	2126.132576	2194.950572	2131.479546
**ENSG00000150764**	DIXDC1	189.8411491	161.1473872	61.88172468	239.4488247	242.7156791	140.3712487	185.8938053	244.2129575	193.2125218	229.6982515	186.2465916	295.1930007
**ENSG00000150768**	DLAT	1082.797665	952.8715072	1091.072514	1601.627978	1482.877221	1423.060957	1004.030828	888.8022603	990.9176177	1063.066288	880.1875959	1163.855498
**ENSG00000150773**	PIH1D2	101.2486129	52.54806106	66.76712399	67.81592588	98.2420606	73.60006014	107.2464262	91.37219499	75.03398903	99.34607572	80.01705417	68.51184256
**ENSG00000150776**	NKAPD1	866.2381322	637.5831408	734.4383639	802.9070731	835.6354096	842.6068741	809.9658662	757.5585621	792.0775467	709.9764138	842.9382776	913.4912341
**ENSG00000150779**	TIMM8B	357.1826065	360.8300193	326.507521	465.501911	413.7724435	401.0065132	241.0491102	252.5195207	271.9982102	284.117121	255.2268107	341.7133876
**ENSG00000150782**	IL18	177.1850725	122.6121425	124.5776826	149.0275902	165.2778196	177.9300423	389.1513178	279.1005229	363.4458844	360.0504273	284.1985028	326.4885337
**ENSG00000150783**	TEX12	8.437384405	0	10.58503185	3.348934611	0	3.793817533	4.08557814	4.983937908	5.158586746	3.796665314	8.277626294	4.229126084
**ENSG00000150787**	PTS	317.8081459	322.2947745	349.3060511	396.8487515	357.138785	382.4168073	342.1671692	428.6186601	368.6044711	423.3281825	362.8359525	444.0582388
**ENSG00000150867**	PIP4K2A	288.2773005	255.7338971	368.8476484	260.379666	284.3240813	322.0951086	169.5514928	192.7122658	203.0607328	172.7482718	158.654504	150.5568886
**ENSG00000150873**	C2orf50	1.406230734	14.01281628	13.84196473	1.674467306	8.090522638	4.931962793	0	1.661312636	0.937924863	5.062220419	1.379604382	1.691650434
**ENSG00000150893**	FREM2	56.24922937	17.51602035	48.03975995	363.3594053	411.4608656	283.7775515	2363.506954	1854.024902	2167.075396	3329.042703	2997.880323	3598.986297
**ENSG00000150907**	FOXO1	631.3975996	532.4870187	666.0427735	524.1082667	730.4586153	601.6994607	336.038802	627.9761765	400.4939165	303.7332251	627.719994	483.812024
**ENSG00000150938**	CRIM1	7765.206114	5895.892451	8039.738809	6424.931052	5776.633163	7898.728104	6859.685696	7630.408938	7438.213125	6289.176093	7274.653908	6784.364064
**ENSG00000150961**	SEC24D	1742.31988	1583.44824	1729.431358	1151.196273	1247.096275	1346.805224	952.961101	830.6563181	1053.758583	832.7352589	848.4566951	1009.915309
**ENSG00000150967**	ABCB9	165.9352266	175.1602035	155.5185449	187.5403382	234.6251565	202.5898563	208.3644851	210.9867048	210.0951693	290.4448965	274.5412721	285.8889233
**ENSG00000150977**	RILPL2	44.99938349	38.53524477	78.98062229	60.28082301	79.74943743	55.01035423	114.3961879	94.69482026	84.41323766	111.3688492	125.5439988	119.2613556
**ENSG00000150990**	DHX37	712.9589822	448.410121	685.5843708	614.5295012	494.6776699	706.0294429	699.6552564	519.9908551	809.4291567	754.2708424	609.785137	724.8722108
**ENSG00000150991**	UBC	16211.0279	20679.41363	16024.10976	11450.00744	12228.24707	10135.9423	12191.36517	15069.76692	11041.72045	10780.63116	11947.37395	10176.12318
**ENSG00000150995**	ITPR1	123.7483046	196.1794279	175.8743754	83.72336529	176.8357091	137.3361947	28.59904698	58.14594226	29.54463318	36.70109804	60.70259282	31.29553302
**ENSG00000151005**	TKTL2	0	0	4.885399317	0	0	0	0	0	1.875849726	0	0	0
**ENSG00000151006**	PRSS53	26.71838395	42.03844885	26.86969624	39.34998168	42.76419109	23.5216687	45.96275407	73.09775599	42.20661883	56.94997971	60.70259282	23.68310607
**ENSG00000151012**	SLC7A11	2685.900702	1797.143688	2870.986332	2525.933931	2347.407354	2890.509578	1426.888165	556.5397331	1418.142393	1048.512404	572.5358187	1144.401518
**ENSG00000151014**	NOCT	393.7446056	395.86206	551.2358896	315.6370871	453.0692677	349.031213	337.0601965	986.8197059	357.8183352	404.9776335	918.8165186	346.7883389
**ENSG00000151023**	ENKUR	73.12399818	339.8107948	94.45105346	18.41914036	53.16629162	35.66188481	32.68462512	99.67875817	24.85500887	24.04554699	56.56377967	27.91223215
**ENSG00000151025**	GPR158	684.8343675	599.047896	682.3274379	329.0328256	377.9429861	347.513686	605.6869592	649.5732407	671.0852394	420.1642948	412.5017103	416.9918319
**ENSG00000151067**	CACNA1C	1.406230734	10.50961221	10.58503185	24.27977593	1.155788948	10.62268909	129.7171059	157.8247004	129.9025935	152.4993901	114.5071637	112.4947538

**ENSG00000151079**	KCNA6	0	0	0.814233219	0	0	3.035054026	1.021394535	0	0	0	0	0
**ENSG00000151090**	THRB	1051.860589	669.1119775	1010.463425	1286.828124	929.2543144	1300.900032	1502.471361	1297.485169	1659.189082	1750.26271	1437.547766	1902.260913
**ENSG00000151092**	NGLY1	511.8679872	493.9517739	602.5325824	526.6199677	636.8397105	589.5592446	697.6124673	958.577391	663.5818405	653.0264341	1231.986713	742.6345403
**ENSG00000151093**	OXSM	84.37384405	63.05767327	89.56565414	87.90953355	100.5536385	95.22482008	91.92550814	189.3896405	113.9578708	91.7527451	146.2380645	117.5697051
**ENSG00000151116**	UEVLD	703.1153671	795.227324	722.2248656	490.6189206	588.2965747	555.4148868	719.0617526	951.9321405	815.5256683	711.8747464	780.8560804	775.6217238
**ENSG00000151117**	TMEM86A	60.46792157	140.1281628	94.45105346	36.00104707	60.10102531	50.45777319	21.44928523	26.58100218	25.3239713	29.10776741	53.80457091	15.2248539
**ENSG00000151131**	C12orf45	487.9620648	304.7787541	434.8005392	678.1592588	554.7786952	594.4912074	1090.849363	730.9775599	1080.02048	1173.80236	878.8079915	1225.600739
**ENSG00000151135**	TMEM263	807.1764414	956.3747112	905.42734	486.4327523	822.9217312	624.8417477	798.7305263	1129.692593	822.0911423	765.6608384	1009.870408	864.4333715
**ENSG00000151136**	BTBD11	0	7.006408141	8.142332194	7.535102876	0	6.449489806	0	13.29050109	1.406887294	0.632777552	5.518417529	4.229126084
**ENSG00000151148**	UBE3B	807.1764414	1005.419568	906.2415732	869.8857653	869.1532891	843.3656376	1305.342216	1277.549417	1261.977903	1381.353397	1368.567547	1260.279573
**ENSG00000151150**	ANK3	101.2486129	77.07048955	113.1784175	116.3754777	202.2630659	117.6083435	516.8256346	338.9077778	467.0865817	540.3920297	383.5300183	522.719984
**ENSG00000151151**	IPMK	248.9028399	567.5190594	331.3929203	245.3094603	369.8524634	324.7507808	871.2495382	1048.288273	824.4359545	746.6775118	965.7230676	918.5661854
**ENSG00000151164**	RAD9B	7.031153671	10.50961221	6.513865756	11.72127114	10.40210053	12.14021611	34.72741419	24.91968954	11.25509835	16.45221636	31.73090079	19.45397999
**ENSG00000151176**	PLBD2	525.9302946	465.9261414	625.3311125	551.7369772	694.6291579	638.8788726	761.960323	833.9789433	669.6783521	776.4180568	902.261266	718.9514343
**ENSG00000151208**	DLG5	2566.37109	2245.553809	2647.072196	3260.187844	3873.048766	3407.98629	2440.111544	2287.6275	2582.107148	2488.714114	2546.74969	2533.246524
**ENSG00000151224**	MAT1A	54.84299863	56.05126513	74.90945619	11.72127114	45.07576898	34.9031213	15.32091802	3.322625272	8.910286198	4.429442867	2.759208765	1.691650434
**ENSG00000151229**	SLC2A13	14.06230734	7.006408141	3.256932878	18.41914036	15.02525633	2.655672273	16.34231256	23.25837691	12.66198565	20.88165923	19.31446135	20.2998052
**ENSG00000151233**	GXYLT1	397.9632978	578.0286716	373.7330477	424.477462	462.3155793	459.4313033	651.6497133	614.6856754	612.4649355	697.3208627	689.8021912	722.3347351
**ENSG00000151239**	TWF1	901.3939006	942.3618949	885.0715095	882.4442701	995.1342845	984.1162681	843.6718858	1001.77152	884.4631457	819.4469303	718.7738832	979.465601
**ENSG00000151240**	DIP2C	39.37446056	10.50961221	35.01202844	30.1404115	48.54313583	39.45570234	1.021394535	9.967875817	10.78613592	15.81943881	8.277626294	15.2248539
**ENSG00000151247**	EIF4E	1126.390818	931.8522827	1017.791524	1341.248312	1077.1953	1220.4711	602.6227756	689.444744	660.2991035	632.1447748	578.0542362	702.0349299
**ENSG00000151276**	MAGI1	53.4367679	14.01281628	39.89742775	92.93293547	58.94523636	83.46398573	183.8510163	171.1152015	164.6058134	211.3477025	189.0058004	177.6232955
**ENSG00000151287**	TEX30	396.557067	546.499835	437.2432388	468.8508456	444.9787451	473.0890464	314.5895167	408.6829085	404.7145783	456.8653928	350.4195131	531.1782361
**ENSG00000151292**	CSNK1G3	791.7079033	749.6856711	726.2960317	769.417727	691.1617911	805.0480805	810.9872607	775.8330011	752.6847025	818.1813752	771.1988497	884.7331767
**ENSG00000151303**	AL136982.1	28.12461468	7.006408141	14.65619795	18.41914036	9.246311586	14.03712487	6.128367209	1.661312636	4.689624315	6.960553076	6.898021912	10.99572782
**ENSG00000151304**	SRFBP1	537.1801404	360.8300193	530.8800591	445.4083033	346.7366845	428.7013812	503.5475057	375.4566558	425.3489253	406.8759662	394.5668533	472.8162962
**ENSG00000151320**	AKAP6	40.78069129	24.52242849	52.92515926	85.39783259	61.25681426	60.70108053	130.7385005	48.17806645	88.16493711	117.0638472	89.67428485	127.7196077
**ENSG00000151322**	NPAS3	147.6542271	59.5544692	67.58135721	177.4935344	202.2630659	105.8475092	315.6109113	330.6012146	255.5845251	199.9577066	322.8274255	274.0473702
**ENSG00000151327**	FAM177A1	539.9926019	662.1055693	543.9077906	498.9912571	535.130283	505.3364954	569.9381505	534.9426688	490.5347033	547.3525828	493.8983689	510.0326057
**ENSG00000151332**	MBIP	219.3719945	234.7146727	217.4002696	219.3552171	224.223056	230.2847243	366.680638	355.5209041	345.625312	320.8182191	349.0399087	424.6042588
**ENSG00000151338**	MIPOL1	146.2479964	171.6569995	154.7043117	203.4477776	256.5851465	168.4454985	21.44928523	23.25837691	23.917084	36.06832049	20.69406573	25.3747565
**ENSG00000151348**	EXT2	826.8636717	900.3234461	1004.763793	1166.266478	1202.020506	1263.341239	1546.391326	1380.550801	1524.127902	1741.403824	1768.652818	1956.393726
**ENSG00000151353**	TMEM18	667.9595987	683.1247937	679.070505	601.9709964	587.1407857	664.6768318	675.1417876	770.8490632	633.0992825	685.9308668	722.9126963	695.2683282
**ENSG00000151364**	KCTD14	43.59315276	24.52242849	24.42699658	17.58190671	10.40210053	4.173199286	0	0	8.441323766	0	1.379604382	0
**ENSG00000151366**	NDUFC2	178.5913032	301.2755501	223.0999021	220.1924507	206.8862217	172.9980795	92.94690267	194.3735784	132.2474057	159.4599432	114.5071637	168.3192181
**ENSG00000151376**	ME3	170.1539188	136.6249587	210.8864038	102.9797393	117.8904727	107.7444179	0	8.306563181	0	3.796665314	0	0
**ENSG00000151388**	ADAMTS12	374.0573753	700.6408141	686.398604	851.466625	1297.950989	1272.825782	2.04278907	3.322625272	7.972361335	11.38999594	23.4532745	18.60815477
**ENSG00000151413**	NUBPL	253.1215321	91.08330583	177.5028418	267.9147689	273.9219807	284.9156967	516.8256346	420.3120969	511.6380127	565.7031318	511.8332258	523.5658092
**ENSG00000151414**	NEK7	3937.446056	3082.819582	3679.519919	3593.406838	2931.080773	3774.848445	2135.735972	1948.719722	2131.903213	1976.797074	1945.242179	2234.670223
**ENSG00000151422**	FER	1449.823887	1635.996301	1317.429349	1715.491755	1640.064518	1510.31876	1039.779637	1425.406242	898.5320187	1016.240749	1374.085965	1067.431424
**ENSG00000151445**	VIPAS39	310.7769922	437.9005088	305.3374573	293.0317785	373.3198303	298.1940581	515.8042401	495.0711656	467.0865817	454.9670602	576.6746318	429.6792101
**ENSG00000151458**	ANKRD50	2272.468866	1982.813504	1985.100589	3040.832627	2784.295576	2675.400124	452.477779	790.7848148	464.2728071	563.1720216	826.383025	621.6815343
**ENSG00000151461**	UPF2	1241.701738	1271.663078	1280.788854	1015.564421	1019.405852	966.6647074	1274.70038	1475.245621	1134.420122	1203.542905	1284.41168	1273.812776
**ENSG00000151465**	CDC123	911.2375157	746.182467	867.9726119	969.51657	797.4943743	897.2378466	654.7138969	608.0404248	638.7268316	723.2647424	605.6463238	734.1762882
**ENSG00000151466**	SCLT1	358.5888372	416.8812844	341.1637189	442.0593687	440.3555893	448.8086142	384.0443451	304.0202124	348.908049	310.6937782	344.9010956	387.3879493
**ENSG00000151468**	CCDC3	0	24.52242849	0	0.837233653	0	0.379381753	0	0	6.096511609	0.632777552	2.759208765	5.074951301
**ENSG00000151470**	C4orf33	130.7794583	192.6762239	108.2930182	102.1425056	117.8904727	79.29078644	122.5673442	187.7283279	125.6819316	112.0016268	125.5439988	96.42407471
**ENSG00000151474**	FRMD4A	198.2785335	84.07689769	186.4594073	490.6189206	243.8714681	336.5116152	710.8905963	737.6228104	845.0703015	716.9369668	656.691686	954.9366697
**ENSG00000151491**	EPS8	322.0268381	413.3780803	390.8319453	275.4498718	440.3555893	379.0023716	186.9151999	101.3400708	185.7091229	192.3643759	125.5439988	167.4733929
**ENSG00000151498**	ACAD8	274.2149932	269.7467134	233.684934	288.8456102	305.1282823	256.4620652	273.7337353	318.9720261	243.3915019	301.8348925	339.382678	307.8803789
**ENSG00000151500**	THYN1	154.6853808	238.2178768	164.4751103	204.2850113	249.6504128	189.3114949	204.278907	255.842146	211.0330942	253.111021	274.5412721	208.0730033
**ENSG00000151502**	VPS26B	457.0249886	385.3524477	528.4373594	663.9262867	654.1765447	723.8603853	617.9436936	556.5397331	643.8854184	673.9080933	678.7653561	585.31105
**ENSG00000151503**	NCAPD3	2069.971641	1860.201361	1788.05615	2629.750904	2112.782197	2267.944121	1857.916659	1760.991394	2048.427901	2129.296464	1950.760597	2342.93585
**ENSG00000151532**	VTI1A	582.1795239	672.6151815	710.8256006	598.6220618	603.321831	698.0624261	693.5268892	676.1542429	725.4848815	635.9414401	670.4877298	640.2896891
**ENSG00000151552**	QDPR	61.8741523	52.54806106	56.99632536	81.21166433	84.37259322	66.39180683	7.149761744	9.967875817	27.19982102	13.2883286	13.79604382	36.37048432
**ENSG00000151553**	FAM160B1	1695.914265	1219.115017	1692.790863	1579.859903	1042.521631	1581.263148	1220.566469	1129.692593	1180.847402	1154.186256	1016.76843	1199.380157
**ENSG00000151572**	ANO4	19.68723028	31.52883663	35.01202844	0	0	1.13814526	0	0	0.468962431	1.265555105	0	0
**ENSG00000151575**	TEX9	233.4343019	213.6954483	195.4159727	257.0307314	242.7156791	248.4950484	302.3327823	280.7618355	249.956976	281.5860108	264.8840414	307.8803789
**ENSG00000151576**	QTRT2	757.9583657	462.4229373	730.3671978	828.0240827	522.4166046	744.347	732.3398815	631.2988017	877.8976717	907.4030101	703.598235	1044.594143
**ENSG00000151611**	MMAA	113.9046895	136.6249587	170.1747429	87.90953355	85.52838217	87.63718501	57.19809395	51.50069172	72.22021444	67.07442055	57.94338406	55.82446431
**ENSG00000151612**	ZNF827	545.6175249	739.1760589	874.4864777	714.1603059	799.8059522	913.9306437	279.8621026	312.3267756	216.1916809	260.071574	271.7820633	252.0559146
**ENSG00000151623**	NR3C2	541.3988327	350.320407	419.330108	416.9423591	325.9324834	300.0909669	419.7931538	322.2946514	480.2175298	489.137048	351.7991175	513.4159066
**ENSG00000151640**	DPYSL4	2.812461468	14.01281628	8.142332194	2.511700959	5.778944741	6.449489806	1.021394535	0	0	1.265555105	11.03683506	0.845825217
**ENSG00000151651**	ADAM8	2182.470099	2280.58585	3023.247944	1610.000314	1744.085523	2787.697123	138.9096567	111.3079466	240.1087649	72.13664097	111.747955	188.6190233
**ENSG00000151657**	KIN	329.0579918	266.2435094	271.1396621	262.0541333	238.0925233	211.3156366	279.8621026	333.9238399	281.8464213	284.7498986	291.0965247	298.5763015
**ENSG00000151665**	PIGF	277.0274546	206.6890402	258.1119306	298.0551804	246.183046	261.394028	324.8034621	358.8435294	349.8459739	332.8409926	328.345843	396.6920267
**ENSG00000151687**	ANKAR	42.18692202	87.58010176	35.01202844	39.34998168	45.07576898	41.73199286	69.45482837	26.58100218	37.04803208	38.5994307	44.14734023	49.90368779

**ENSG00000151689**	INPP1	864.8319015	994.909956	872.043778	203.4477776	245.027257	227.2496702	705.7836236	1136.337843	682.3403378	396.7515253	540.8049179	378.0838719
**ENSG00000151690**	MFSD6	922.4873616	735.6728548	940.4393685	863.1878961	954.6816713	962.4915081	1036.715453	885.4796351	909.787117	1029.529078	844.317882	1098.726957
**ENSG00000151692**	RNF144A	234.8405326	336.3075908	157.9612446	167.4467306	169.9009754	115.332053	116.438977	121.2758224	83.00635037	98.71329817	129.6828119	59.20776517
**ENSG00000151693**	ASAP2	2355.43648	2459.249257	3025.690643	2258.019162	2474.544138	3048.71177	1699.600506	3043.524749	2096.262069	1576.881661	2590.89703	1850.665574
**ENSG00000151694**	ADAM17	1868.880646	2147.464095	1886.578369	1579.859903	1613.481372	1620.339468	2107.136925	2779.37604	2126.275664	2021.72428	2553.647712	2097.646538
**ENSG00000151702**	FLI1	35.15576835	77.07048955	29.3123959	14.2329721	21.95999002	18.96908767	0	0	0.937924863	2.53111021	0	0.845825217
**ENSG00000151715**	TMEM45B	66.09284451	45.54165292	53.73939248	89.58400086	56.63365846	83.46398573	0	0	10.31717349	0.632777552	0	20.2998052
**ENSG00000151718**	WWC2	871.8630552	1022.935589	1068.273984	731.7422126	826.389098	1080.858615	256.3700283	385.4245316	217.5985682	266.3993496	361.4563482	213.9937798
**ENSG00000151725**	CENPU	978.736591	1149.050935	889.9569089	933.5155229	863.3743444	804.6686988	617.9436936	785.8008769	615.2787101	741.6152914	651.1732684	674.968523
**ENSG00000151726**	ACSL1	807.1764414	795.227324	733.6241307	1052.402702	1097.999501	1094.89574	595.4730138	634.621427	647.1681554	700.4847505	721.5330919	724.8722108
**ENSG00000151729**	SLC25A4	268.5900702	234.7146727	269.5111956	314.7998535	359.4503629	273.1548624	185.8938053	603.0564869	160.3851516	184.1382677	419.3997322	173.3941694
**ENSG00000151743**	AMN1	32.34330689	21.01922442	16.28466439	20.09360767	8.090522638	18.21032416	18.38510163	36.54887799	23.44812157	20.24888168	22.07367012	19.45397999
**ENSG00000151746**	BICD1	177.1850725	189.1730198	171.8032093	206.7967123	159.4988749	150.6145561	246.1560829	210.9867048	246.2052765	286.0154537	344.9010956	301.9596024
**ENSG00000151748**	SAV1	614.5228308	830.2593647	610.6749146	552.5742109	722.3680927	589.5592446	492.3121658	875.5117592	524.7689608	554.3131359	838.7994644	578.5444483
**ENSG00000151773**	CCDC122	140.6230734	129.6185506	120.5065165	102.9797393	149.0967743	90.29285729	228.7923758	259.1647712	138.8128797	191.0988208	248.3287888	133.6403843
**ENSG00000151779**	NBAS	1732.476264	1758.608443	1762.000687	1483.578033	1596.144538	1531.94352	2283.83818	3548.563791	2256.64722	2075.510372	2818.531753	2102.721489
**ENSG00000151790**	TDO2	0	17.51602035	17.91313083	4.186168264	5.778944741	6.070108053	22.47067977	16.61312636	17.8205724	22.77999189	17.93485697	18.60815477
**ENSG00000151806**	GUF1	707.3340593	634.0799367	695.3551694	634.6231089	688.8502132	693.1304633	788.5165809	533.2813562	856.7943623	856.7808059	769.8192453	843.2877411
**ENSG00000151835**	SACS	119.5296124	164.6505913	267.068496	157.3999267	293.5703929	324.3713991	35.74880872	28.24231481	48.77209287	47.45831643	26.21248326	64.28271647
**ENSG00000151846**	PABPC3	2.812461468	7.006408141	8.142332194	5.023401917	10.40210053	3.793817533	3.064183605	6.645250545	3.751699452	3.796665314	8.277626294	3.383300867
**ENSG00000151849**	CENPJ	946.3932841	1152.554139	937.9966688	1013.889954	948.9027265	875.6130866	779.3240301	848.9307571	743.7744163	804.8930466	859.4935302	866.9708472
**ENSG00000151876**	FBXO4	168.7476881	133.1217547	120.5065165	108.8403749	109.7999501	67.52995209	229.8137703	184.4057026	195.0883715	230.3310291	194.5242179	209.7646538
**ENSG00000151881**	TMEM267	267.1838395	178.6634076	257.2976973	312.2881525	245.027257	306.1610749	299.2685987	154.5020752	256.9914124	218.3082556	177.9689653	262.2058172
**ENSG00000151882**	CCL28	11.24984587	3.50320407	21.17006371	0	9.246311586	6.449489806	43.919965	28.24231481	43.61350613	13.2883286	24.83287888	14.37902869
**ENSG00000151883**	PARP8	222.184456	280.2563256	284.9816268	22.60530863	57.78944741	14.03712487	90.9041136	73.09775599	38.92388181	12.65555105	13.79604382	7.612426951
**ENSG00000151892**	GFRA1	0	0	6.513865756	8.372336529	1.155788948	9.863925586	0	0	0.937924863	0	0	0
**ENSG00000151893**	CACUL1	1864.661953	1765.614851	1886.578369	1802.564055	1809.965493	1994.409877	1987.633765	1845.718339	2030.138366	1924.276537	1786.587675	2040.976248
**ENSG00000151914**	DST	35598.73104	32793.4933	33435.67292	47241.7461	43494.6497	41768.41351	32814.34222	32784.34356	28181.35939	31252.88331	35039.1921	31296.37885
**ENSG00000151917**	BEND6	4.218692202	21.01922442	16.28466439	5.023401917	6.93473369	12.14021611	4.08557814	1.661312636	3.751699452	8.226108181	0	11.84155303
**ENSG00000151923**	TIAL1	1975.754181	1646.505913	2064.895445	2027.779907	1791.47287	2143.506906	1714.921424	1687.893638	1743.133358	1797.721026	1757.615983	1958.931202
**ENSG00000151929**	BAG3	1677.633266	1460.836097	1573.09858	1626.744988	1521.018256	1556.982716	393.2368959	441.9091612	492.410553	438.5148438	487.000347	504.9576544
**ENSG00000151962**	RBM46	11.24984587	0	8.956565414	4.186168264	2.311577897	12.89897961	1.021394535	0	0	0	0	0
**ENSG00000151967**	SCHIP1	144.8417656	77.07048955	113.9926507	134.7946181	115.5788948	145.6825933	183.8510163	197.6962037	140.219767	154.3977228	191.7650091	122.6446564
**ENSG00000152034**	MCHR2	0	7.006408141	0	0	0	2.27629052	2.04278907	1.661312636	0	0	0	0.845825217
**ENSG00000152049**	KCNE4	0	0	0	0	0	0	16.34231256	23.25837691	9.379248629	11.38999594	27.59208765	10.1499026
**ENSG00000152056**	AP1S3	257.3402244	220.7018564	265.4400295	244.4722266	246.183046	276.5692982	141.9738403	109.646634	114.8957957	127.188288	85.5354717	149.7110634
**ENSG00000152061**	RABGAP1L	1324.669352	1362.746383	1217.278663	1086.729281	992.8227066	997.7740112	2111.222504	1550.00469	1908.208134	2203.964215	1572.748996	2202.528864
**ENSG00000152076**	CCDC74B	11.24984587	0	1.628466439	1.674467306	5.778944741	3.41443578	5.106972674	6.645250545	5.627549177	9.491663286	4.138813147	2.53747565
**ENSG00000152078**	TMEM56	434.5252969	648.092753	458.4133025	638.8092771	696.9407358	709.4438787	617.9436936	697.7513072	589.0168139	692.2586423	648.4140597	752.7844429
**ENSG00000152082**	MZT2B	170.1539188	112.1025303	92.0083538	159.9116277	193.0167544	213.5919271	155.2519693	134.5663235	197.4331836	180.97438	190.3854048	199.6147512
**ENSG00000152086**	TUBA3E	0	0	7.328098975	2.511700959	8.090522638	7.208253313	2.04278907	4.983937908	1.875849726	1.265555105	1.379604382	0.845825217
**ENSG00000152092**	ASTN1	8.437384405	7.006408141	10.58503185	0	18.49262317	9.484543833	1.021394535	0	4.689624315	4.429442867	11.03683506	0.845825217
**ENSG00000152102**	FAM168B	1605.915498	1618.480281	1633.351838	2373.557406	2249.165293	2466.360778	1449.358845	1486.874809	1446.749101	1642.057748	1834.873828	1815.140915
**ENSG00000152104**	PTPN14	5219.928485	3885.053314	4815.37526	6208.92477	5401.001755	6003.716246	6246.848975	5326.168311	6533.584595	6192.361128	5464.612958	6490.016888
**ENSG00000152117**	AC073869.1	151.8729193	192.6762239	223.9141353	174.1445998	166.4336086	169.5836437	214.4928523	282.4231481	207.7503571	246.1504679	229.0143275	156.4776651
**ENSG00000152127**	MGAT5	502.0243721	546.499835	501.5676632	615.3667349	606.7891978	612.3221498	559.7242051	584.7820479	633.0992825	691.6258648	685.663378	742.6345403
**ENSG00000152133**	GPATCH11	140.6230734	154.1409791	185.645174	267.9147689	217.2883223	277.3280617	304.3755714	367.1500926	329.2116269	341.6998783	368.3543701	410.2252301
**ENSG00000152137**	HSPB8	88.59253625	206.6890402	150.6331456	164.9350296	210.3535886	303.1260209	391.1941069	342.230403	510.2311254	327.7787721	369.7339745	521.0283335
**ENSG00000152147**	GEMIN6	319.2143767	192.6762239	225.5426018	383.453013	265.8314581	245.8393761	205.3003015	201.018829	196.9642212	203.7543719	146.2380645	231.7561094
**ENSG00000152154**	TMEM178A	0	0	1.628466439	0	0	0.379381753	0	0	3.28273702	0	0	0
**ENSG00000152192**	POU4F1	210.9346101	147.134571	190.5305734	231.9137218	189.5493875	174.5156065	100.0966644	98.01744553	133.654293	132.2505084	75.87824103	120.1071808
**ENSG00000152193**	RNF219	1254.357815	1348.733567	1175.752769	1090.91545	1007.847963	1138.14526	1063.271711	1119.724717	1170.530229	1039.653519	983.6579246	1281.425203
**ENSG00000152208**	GRID2	2.812461468	0	0.814233219	0	1.155788948	1.517527013	0	0	0.937924863	0	2.759208765	4.229126084
**ENSG00000152213**	ARL 11.00	60.46792157	59.5544692	52.92515926	56.09465474	83.21680428	71.32376962	36.77020326	48.17806645	45.02039342	53.1533144	89.67428485	31.29553302
**ENSG00000152217**	SETBP1	815.6138258	847.775385	795.5058554	837.2336529	1033.27532	853.6089449	1106.170281	1347.324548	943.5524121	1038.387963	1380.983987	859.3584202
**ENSG00000152219**	ARL14EP	382.4947597	273.2499175	387.5750125	347.4519659	307.4398602	334.2353247	384.0443451	400.3763453	441.7626104	436.6165111	397.3260621	411.0710554
**ENSG00000152223**	EPG5	1489.198347	1646.505913	1874.364871	1455.949322	1422.776195	1794.096311	1194.010211	1252.629728	1155.523431	1092.806833	1238.884735	1089.422879
**ENSG00000152229**	PSTPIP2	680.6156753	672.6151815	876.1149441	496.4795562	494.6776699	621.8066937	867.1639601	496.7324782	727.3607312	613.1614483	395.9464577	590.3860013
**ENSG00000152234**	ATP5F1A	4315.722123	4004.162252	4174.573716	4604.785091	4088.02551	4364.40769	4295.985414	4017.053954	4521.735764	4492.087844	4352.651826	4915.93616
**ENSG00000152240**	HAUS1	486.555834	560.5126513	397.3458111	478.8976494	403.3703429	416.9405469	447.3708063	470.151476	408.9352402	427.1248479	446.9918199	503.266004
**ENSG00000152242**	C18orf25	1373.887427	1306.695118	1617.881407	1391.482331	1139.607903	1529.667229	1011.18059	1194.483785	1086.116991	1039.653519	1111.961132	933.7910393
**ENSG00000152253**	SPC25	343.1202991	262.7403053	343.6064186	467.1763783	402.214554	446.5323236	374.8517943	355.5209041	387.3629684	406.8759662	336.6234693	485.5036744
**ENSG00000152256**	PDK1	476.7122189	2312.114686	420.9585745	394.3370505	1841.171795	357.7569934	826.3081787	3314.318709	651.3888173	779.5819445	3556.620098	822.9879359
**ENSG00000152270**	PDE3B	1.406230734	3.50320407	1.628466439	3.348934611	5.778944741	8.346398573	445.3280172	965.2226416	634.5061698	544.8214726	1189.218978	837.3669646
**ENSG00000152284**	TCF7L1	5.624922937	0	10.58503185	1.674467306	5.778944741	2.27629052	6.128367209	3.322625272	56.74445421	18.98332657	12.41643944	84.58252168
**ENSG00000152291**	TGOLN2	3411.515761	3247.470173	3904.248287	3374.051621	3526.312081	4026.378548	4337.86259	4146.63634	4699.472526	4242.140711	4250.561102	4464.265494
**ENSG00000152315**	KCNK13	0	3.50320407	0	3.348934611	0	1.13814526	0	0	0.937924863	3.163887762	0	0

**ENSG00000152332**	UHMK1	7250.525665	7542.398364	7025.204217	5261.176275	5249.593403	5540.491125	3299.104348	3194.704199	3536.445696	2938.618953	3124.803926	3741.084934
**ENSG00000152348**	ATG10	157.4978422	157.6441832	140.0481137	152.3765248	177.991498	138.47434	313.5681222	302.3588998	304.356618	344.2309885	340.7622824	308.7262041
**ENSG00000152359**	POC5	455.6187579	465.9261414	502.3818964	501.5029581	365.2293077	436.6683981	395.279685	395.3924074	460.9900701	454.9670602	430.4365673	501.5743535
**ENSG00000152377**	SPOCK1	1184.046278	1376.7592	1415.137335	908.3985134	1241.31733	1326.697991	76.60459012	147.8568246	255.1155627	65.1760879	122.78479	207.2271781
**ENSG00000152380**	FAM151B	43.59315276	59.5544692	30.94086234	52.74572013	36.98524634	35.28250306	66.39064477	56.48462963	88.16493711	104.4082961	74.49863664	60.89941561
**ENSG00000152382**	TADA1	279.8399161	308.2819582	254.8549977	363.3594053	287.7914481	320.1981998	345.2313528	299.0362745	316.0806788	346.7620987	306.2721729	333.2551354
**ENSG00000152402**	GUCY1A2	182.8099954	182.1666117	97.70798633	678.9964925	509.7029262	484.470499	104.1822426	164.469951	111.1440963	158.8271656	249.7083932	175.9316451
**ENSG00000152404**	CWF19L2	788.8954419	987.9035479	892.3996085	1011.378253	854.1280328	907.8605357	870.2281437	1104.772903	831.9393534	801.0963813	793.2725198	878.8124002
**ENSG00000152409**	JMY	521.7116024	599.047896	646.5011762	683.1826607	856.4396107	763.3160876	1291.042692	1023.368584	1140.985596	1256.063441	1027.805265	1196.842682
**ENSG00000152413**	HOMER1	29.53084542	91.08330583	32.56932878	34.32657977	54.32208057	39.07632059	697.6124673	627.9761765	654.2025919	700.4847505	584.9522581	742.6345403
**ENSG00000152422**	XRCC4	184.2162262	199.682632	168.5462764	201.7733103	220.7556891	176.4125153	342.1671692	392.0697821	392.0525927	368.2765355	384.9096227	460.1289179
**ENSG00000152433**	ZNF547	29.53084542	31.52883663	21.17006371	17.58190671	13.86946738	17.45156065	26.55625791	26.58100218	18.28953483	28.47498986	15.17564821	24.52893129
**ENSG00000152439**	ZNF773	4.218692202	7.006408141	0	0	0	1.517527013	5.106972674	1.661312636	2.344812157	1.898332657	0	0.845825217
**ENSG00000152443**	ZNF776	243.277917	266.2435094	219.8429693	220.1924507	193.0167544	221.5589439	394.2582905	412.0055338	346.5632368	350.558764	491.1391601	346.7883389
**ENSG00000152454**	ZNF256	88.59253625	98.08971397	91.19412058	105.4914403	100.5536385	89.91347553	187.9365944	265.8100218	163.6678886	182.8727126	206.9406573	180.1607712
**ENSG00000152455**	SUV39H2	479.5246803	371.3396315	398.9742775	540.0157061	421.8629661	512.5447487	517.8470292	478.4580392	514.4517873	541.0248073	498.037182	562.4737692
**ENSG00000152457**	DCLRE1C	822.6449795	672.6151815	728.7387314	504.8518927	424.174544	496.6107151	556.6600215	573.1528595	583.3892647	493.5664909	420.7793366	475.3537718
**ENSG00000152464**	RPP38	165.9352266	178.6634076	240.1987997	187.5403382	146.7851964	185.5176774	225.7281922	197.6962037	163.1989261	179.7088249	147.6176689	192.8481494
**ENSG00000152465**	NMT2	60.46792157	10.50961221	28.49816268	63.62975762	47.38734688	28.4536315	46.9841486	46.51675381	43.14454369	70.23830831	78.63744979	47.36621214
**ENSG00000152475**	ZNF837	0	0	0.814233219	0	0	0.379381753	1.021394535	0	2.813774589	1.265555105	0	0
**ENSG00000152484**	USP12	1155.921663	1779.627668	1280.788854	1400.691901	1388.102527	1525.114648	888.6132453	950.2708279	857.7322871	834.6335916	936.7513756	1040.365017
**ENSG00000152492**	CCDC50	3678.699601	3061.800358	3423.036455	2656.542381	2728.817707	2955.383858	1976.398425	2194.593992	1919.932194	1736.974381	1885.919191	1840.515672
**ENSG00000152495**	CAMK4	175.7788418	231.2114686	261.3688634	401.8721534	658.7997005	620.6685484	17.36370709	28.24231481	32.35840777	25.3111021	27.59208765	36.37048432
**ENSG00000152503**	TRIM36	14.06230734	59.5544692	25.2412298	10.04680383	15.02525633	13.65774312	45.96275407	79.74300653	14.06887294	17.71777147	57.94338406	32.14135824
**ENSG00000152518**	ZFP36L2	2095.283794	1261.153465	1446.892431	2115.689441	1618.104528	1813.444781	1565.797822	1350.647173	1815.822535	1276.945101	1230.607109	1678.11723
**ENSG00000152520**	PAN3	1222.014508	1064.974037	1187.152034	1152.033506	1113.024757	1141.180314	1269.593407	1307.453045	1207.578261	1313.646199	1158.867681	1176.542877
**ENSG00000152527**	PLEKHH2	1.406230734	14.01281628	8.142332194	12.55850479	15.02525633	5.6907263	1.021394535	4.983937908	1.875849726	3.163887762	1.379604382	3.383300867
**ENSG00000152556**	PFKM	407.8069129	315.2883663	338.7210193	558.4348465	524.7281825	429.8395265	314.5895167	272.4552723	283.722271	357.5193171	318.6886123	313.8011554
**ENSG00000152558**	TMEM123	4561.812502	3618.809805	3165.738757	3909.043925	4207.071772	3475.895624	4430.809492	4596.852064	4095.448914	4223.790162	4283.671607	4519.244133
**ENSG00000152580**	IGSF10	1.406230734	3.50320407	1.628466439	0	1.155788948	0.758763507	1.021394535	0	0.937924863	1.265555105	0	0.845825217
**ENSG00000152582**	SPEF2	11.24984587	14.01281628	6.513865756	11.72127114	1.155788948	4.55258104	39.83438686	29.90362745	41.7376564	31.63887762	42.76773585	33.83300867
**ENSG00000152583**	SPARCL1	33.74953762	52.54806106	15.47043117	10.04680383	10.40210053	10.24330734	15.32091802	9.967875817	12.66198565	6.327775524	20.69406573	13.53320347
**ENSG00000152601**	MBNL1	2843.398544	2508.294114	2564.020408	3047.530496	2681.43036	2924.274554	2991.664593	2699.633034	2870.988005	2508.330218	2561.925338	2691.41584
**ENSG00000152620**	NADK2	901.3939006	616.5639164	851.6879475	1084.21758	862.2185554	1026.227643	1396.246329	973.5292048	1375.466811	1453.490038	1111.961132	1552.935098
**ENSG00000152642**	GPD1L	80.15515185	28.02563256	92.0083538	113.0265431	75.12628164	135.0599042	98.05387535	39.87150327	102.2338101	111.3688492	80.01705417	133.6403843
**ENSG00000152661**	GJA1	753.7396735	371.3396315	234.4991672	910.0729807	464.6271572	392.2807329	1608.696392	2330.821628	2032.483178	938.4091102	1770.032423	1353.320347
**ENSG00000152669**	CCNO	44.99938349	17.51602035	54.5536257	58.6063557	47.38734688	53.11344546	67.4120393	53.16200436	53.46171719	71.50386342	46.906549	68.51184256
**ENSG00000152670**	DDX4	4.218692202	7.006408141	6.513865756	0	2.311577897	2.27629052	0	3.322625272	6.096511609	0	0	0.845825217
**ENSG00000152683**	SLC30A6	961.8618222	1124.528507	1000.692627	926.8176537	955.8374602	945.4193292	938.6615776	991.8036438	861.0150241	898.5441244	929.8533537	945.6325924
**ENSG00000152684**	PELO	790.3016726	945.865099	1010.463425	614.5295012	694.6291579	783.4233206	493.3335603	495.0711656	550.5618945	496.7303786	557.3601705	483.812024
**ENSG00000152689**	RASGRP3	14.06230734	56.05126513	39.89742775	0.837233653	2.311577897	3.41443578	22.47067977	3.322625272	15.47576024	2.53111021	2.759208765	2.53747565
**ENSG00000152700**	SAR1B	1510.291808	1198.095792	1389.081872	1682.002409	1497.902477	1604.026053	1344.155208	1206.112974	1411.576919	1354.77674	1285.791284	1550.397622
**ENSG00000152705**	CATSPER3	8.437384405	0	1.628466439	6.697869223	10.40210053	5.6907263	12.25673442	19.93575163	8.441323766	18.98332657	9.657230676	17.76232955
**ENSG00000152749**	GPR180	1947.629567	2129.948075	1709.075528	1797.540653	1855.041262	1800.925183	1423.823982	1501.826623	1510.527992	1552.203336	1430.649744	1605.376261
**ENSG00000152763**	WDR78	75.93645964	66.56087734	62.6959579	72.00209415	94.77469376	57.6660265	177.7226491	187.7283279	173.9850621	155.6632779	194.5242179	147.1735877
**ENSG00000152766**	ANKRD22	234.8405326	325.7979785	266.2542628	304.7530496	249.6504128	196.1403665	108.2678207	139.5502614	105.0475846	152.4993901	86.91507609	156.4776651
**ENSG00000152767**	FARP1	7.031153671	45.54165292	12.21349829	20.93084132	9.246311586	7.208253313	14.29952349	33.22625272	21.10330942	32.27165517	42.76773585	27.91223215
**ENSG00000152778**	IFIT5	2393.40471	2627.403053	2222.856689	756.0219885	821.7659422	809.9800433	1224.652047	1329.050109	1242.281481	443.5770642	474.5839075	521.0283335
**ENSG00000152779**	SLC16A12	0	0	1.628466439	12.55850479	2.311577897	4.931962793	28.59904698	4.983937908	27.66878346	33.53721028	22.07367012	19.45397999
**ENSG00000152782**	PANK1	187.0286876	189.1730198	119.6922833	249.4956286	241.5598902	234.8373053	354.4239036	395.3924074	364.3838092	345.4965436	430.4365673	433.062511
**ENSG00000152784**	PRDM8	1.406230734	14.01281628	1.628466439	3.348934611	5.778944741	10.24330734	0	0	2.813774589	0	0	0
**ENSG00000152795**	HNRNPDL	4111.818667	2658.931889	3365.225896	4567.109576	3461.5879	3486.138931	3594.287368	2877.393486	3497.052851	3723.895896	3046.166476	3825.667455
**ENSG00000152804**	HHEX	1.406230734	0	4.885399317	1.674467306	4.623155793	1.517527013	0	0	0.468962431	0	5.518417529	0
**ENSG00000152818**	UTRN	1330.294275	1345.230363	1049.54662	1696.235381	1683.984498	1185.188597	4332.755617	4726.43445	3691.67226	4326.300126	5115.57305	4093.794049
**ENSG00000152822**	GRM1	0	0	0	1.674467306	0	0	0	0	0.468962431	1.898332657	5.518417529	0
**ENSG00000152894**	PTPRK	4963.994492	4333.463435	5211.906838	3690.525942	3255.857467	3737.669034	4920.057474	4550.33531	4395.115908	3930.181378	3466.945813	3879.800269
**ENSG00000152904**	GGPS1	216.5595331	234.7146727	192.973273	163.2605623	165.2778196	160.4784816	175.67986	201.018829	169.2954378	163.8893861	220.7367012	161.5526164
**ENSG00000152910**	CNTNAP4	0	3.50320407	1.628466439	2.511700959	1.155788948	2.27629052	5.106972674	1.661312636	5.627549177	4.429442867	1.379604382	5.074951301
**ENSG00000152926**	ZNF117	37.96822982	63.05767327	27.68392946	30.1404115	26.58314581	29.212395	408.557814	259.1647712	305.7635053	365.7454253	355.9379306	352.7091154
**ENSG00000152931**	PART1	5.624922937	49.04485699	0.814233219	2.511700959	9.246311586	12.14021611	0	0	0	0	0	0
**ENSG00000152932**	RAB3C	0	0	0	0	1.155788948	2.655672273	0	0	0	0.632777552	0	0.845825217
**ENSG00000152936**	LMNTD1	0	0	0	2.511700959	0	0	0	0	0.468962431	2.53111021	0	3.383300867
**ENSG00000152939**	MARVELD2	959.0493607	896.820242	1084.558648	1344.597247	1304.885723	1473.51873	824.2653896	769.1877505	903.221643	835.2663691	722.9126963	1100.418607
**ENSG00000152942**	RAD17	672.1782909	595.544692	697.7978691	703.2762684	665.7344342	648.3634164	792.6021591	677.8155555	715.167708	710.6091913	698.0798174	749.4011421
**ENSG00000152944**	MED21	272.8087624	210.1922442	232.0564675	182.5169363	212.6651665	209.0393461	415.7075757	322.2946514	410.3421275	320.8182191	295.2353378	442.3665884
**ENSG00000152952**	PLOD2	1313.419506	3562.75854	1205.065165	1249.15261	2619.017757	1344.149552	1819.103667	6675.154172	1758.140156	1915.417651	6670.387188	1940.323047
**ENSG00000152953**	STK32B	137.8106119	59.5544692	118.0638168	149.8648239	122.5136285	159.7197181	4.08557814	3.322625272	8.910286198	8.226108181	4.138813147	7.612426951

**ENSG00000152977**	ZIC1	21.09346101	49.04485699	37.45472809	36.83828073	34.67366845	39.07632059	165.4659146	134.5663235	151.0059029	149.3355024	140.719647	153.9401895
**ENSG00000152990**	ADGRA3	412.0256051	616.5639164	537.3939248	647.1816137	601.0102531	613.0809133	827.3295732	667.8476797	714.6987455	1194.051241	896.7428485	1075.043851
**ENSG00000153006**	SREK1IP1	933.7372075	1100.006078	988.4791284	883.2815038	906.1385354	944.281184	770.1314793	662.8637418	807.553307	751.1069547	867.7711565	868.6624976
**ENSG00000153012**	LGI2	4.218692202	0	4.885399317	10.88403749	8.090522638	4.931962793	100.0966644	58.14594226	39.39284424	123.3916227	85.5354717	91.34912341
**ENSG00000153015**	CWC27	724.2088281	634.0799367	714.0825335	806.2560077	658.7997005	669.6087946	603.6441701	616.346988	586.6720017	613.1614483	647.0344553	717.2597838
**ENSG00000153029**	MR1	458.4312193	746.182467	536.5796916	187.5403382	272.7661918	179.0681876	662.8850531	554.8784205	491.4726282	408.7742988	326.9662386	323.951058
**ENSG00000153037**	SRP19	471.0872959	311.7851623	456.7848361	361.684938	283.1682923	354.3425576	356.4666927	357.1822168	353.1287109	292.3432292	289.7169203	323.951058
**ENSG00000153044**	CENPH	551.2424478	760.1952833	522.7377269	567.6444166	582.5176299	511.4066035	732.3398815	913.7219499	806.6153821	770.7230588	822.2442119	899.1122054
**ENSG00000153046**	CDYL	971.7054373	879.3042217	1179.823935	1037.332496	1086.441611	1280.413417	660.8422641	556.5397331	740.9606417	656.8230994	702.2186306	776.467549
**ENSG00000153048**	CARHSP1	2414.498171	3254.476581	2852.258968	1947.405477	2289.617907	2099.119241	2251.153555	3226.269139	2550.686665	2107.782027	2771.625204	2205.912165
**ENSG00000153060**	TEKT5	1.406230734	0	0	0	0	2.27629052	7.149761744	0	0.937924863	0	0	0.845825217
**ENSG00000153064**	BANK1	22.49969175	14.01281628	21.98429693	24.27977593	21.95999002	10.24330734	67.4120393	21.59706427	60.02719123	51.8877593	45.52694462	56.67028952
**ENSG00000153066**	TXNDC11	1579.197114	1881.220586	1928.104264	1311.945134	1485.188799	1583.160057	1353.347759	1458.632495	1525.065827	1415.523385	1270.615636	1476.810828
**ENSG00000153071**	DAB2	40.78069129	7.006408141	37.45472809	50.23401917	75.12628164	50.83715494	193.0435671	313.9880882	167.419588	196.1610412	240.0511625	200.4605764
**ENSG00000153093**	ACOXL	954.8306685	571.0222635	1054.432019	700.7645674	596.3870973	687.8191187	266.5839736	136.2276362	302.4807683	253.111021	128.3032076	235.1394103
**ENSG00000153094**	BCL2L11	607.4916772	921.3426705	785.7350568	475.5487148	498.1450367	545.1715795	449.4135953	392.0697821	580.5754901	489.7698255	444.2326111	504.9576544
**ENSG00000153107**	ANAPC1	1209.358431	774.2080996	1030.819256	1227.384535	1050.612154	1135.110206	962.1536519	902.0927614	1013.896777	1051.043515	990.5559465	1120.718412
**ENSG00000153113**	CAST	11119.06641	12187.64696	10648.54204	11850.20512	12615.43637	11155.34107	10352.85501	12177.42162	9315.938701	9167.681179	11265.84939	9280.394278
**ENSG00000153130**	SCOC	770.6144423	998.4131601	810.1620533	819.6517462	900.3595907	875.2337049	479.0340369	410.3442211	456.7694082	425.8592928	487.000347	543.8656144
**ENSG00000153132**	CLGN	2.812461468	0	10.58503185	4.186168264	0	0.758763507	2.04278907	0	2.344812157	1.898332657	0	8.458252168
**ENSG00000153140**	CETN3	350.1514528	448.410121	328.1359874	364.196639	383.7219308	370.655973	333.9960129	375.4566558	401.9008038	427.7576254	379.3912051	417.8376571
**ENSG00000153147**	SMARCA5	2193.719945	2669.441502	2175.631162	2045.361814	2276.904228	1985.304715	1607.674998	1765.975332	1530.224414	1518.666126	1676.219325	1776.232955
**ENSG00000153157**	SYCP2L	0	3.50320407	9.770798633	5.023401917	3.467366845	1.517527013	4.08557814	0	1.875849726	1.265555105	1.379604382	2.53747565
**ENSG00000153162**	BMP6	23.90592248	59.5544692	56.18209214	44.3733836	78.59364848	91.05162079	462.6917243	850.5920697	551.4998194	532.7986991	907.7796836	655.514543
**ENSG00000153165**	RGPD3	4.218692202	21.01922442	17.91313083	9.209570182	9.246311586	12.51959786	2.04278907	11.62918845	7.972361335	9.491663286	6.898021912	7.612426951
**ENSG00000153179**	RASSF3	589.2106776	732.1696507	605.7895153	513.2242292	542.0650167	599.802552	1209.331129	1206.112974	1186.943914	1158.615698	1081.609836	1210.375885
**ENSG00000153187**	HNRNPU	17676.32033	15606.77413	15305.14183	16817.51239	15567.32134	14694.97283	20302.25917	17515.21912	19094.27436	19022.55878	18052.12334	20622.06461
**ENSG00000153201**	RANBP2	4571.656117	4410.533925	4812.118327	5096.241245	5080.848217	5116.342325	5015.047166	4477.237554	4499.69453	5157.137052	4911.391601	5344.769545
**ENSG00000153207**	AHCTF1	4131.505897	3454.159213	3924.604118	4317.613948	3704.303579	4196.720955	3231.692308	2736.181912	3482.983978	3335.370479	3145.497992	3844.27561
**ENSG00000153208**	MERTK	154.6853808	241.7210809	218.2145028	182.5169363	180.3030759	222.3177074	7.149761744	31.56494009	10.78613592	16.45221636	13.79604382	14.37902869
**ENSG00000153214**	TMEM87B	939.3621304	1110.51569	889.9569089	649.6933146	805.5848969	702.6150071	830.3937569	782.4782516	723.1400693	699.2191954	841.5586732	839.058615
**ENSG00000153230**	OR14K1	0	0	0	2.511700959	1.155788948	1.13814526	0	1.661312636	0.468962431	0.632777552	0	4.229126084
**ENSG00000153233**	PTPRR	1071.547819	1243.637445	851.6879475	1155.382441	1292.172044	931.0028226	6.128367209	28.24231481	9.848211061	3.796665314	15.17564821	13.53320347
**ENSG00000153234**	NR4A2	111.092228	343.3139989	245.8984323	88.7467672	211.5093775	152.5114648	73.54040651	68.11381808	62.37200338	48.09109398	73.11903226	44.82873649
**ENSG00000153237**	CCDC148	7.031153671	17.51602035	11.39926507	10.88403749	25.42735686	21.62475994	10.21394535	6.645250545	9.848211061	4.429442867	6.898021912	10.1499026
**ENSG00000153246**	PLA2R1	40.78069129	10.50961221	31.75509556	21.76807497	13.86946738	18.96908767	23.4920743	21.59706427	30.01359561	12.0227735	17.93485697	27.06640694
**ENSG00000153250**	RBMS1	1593.259422	1275.166282	1646.37957	1571.487566	1358.052014	1908.669601	1775.183702	1854.024902	1847.71198	1873.021555	1858.327103	1985.997609
**ENSG00000153283**	CD96	7.031153671	38.53524477	4.071166097	13.39573845	30.05051265	17.0721789	22.47067977	11.62918845	10.31717349	17.71777147	13.79604382	14.37902869
**ENSG00000153291**	SLC25A27	30.93707615	80.57369362	31.75509556	52.74572013	79.74943743	58.04540826	284.9690752	224.2772059	190.3987472	314.4904435	229.0143275	193.6939746
**ENSG00000153292**	ADGRF1	5031.493567	4592.700536	4236.455441	3858.809906	2415.598902	2885.577616	148.1022076	182.74439	138.8128797	166.4204963	186.2465916	144.6361121
**ENSG00000153294**	ADGRF4	1233.264354	1187.58618	1083.744415	514.8986965	458.8482125	491.2993705	1071.442867	925.3511383	851.6357755	659.3542096	593.2298844	546.40309
**ENSG00000153303**	FRMD1	0	0	0	0	1.155788948	3.035054026	0	0	0	0.632777552	0	0
**ENSG00000153310**	FAM49B	2699.96301	2312.114686	2788.748777	2525.096697	2122.028509	2664.398053	1097.999125	993.4649564	1154.116544	969.4152102	837.4198601	1053.052395
**ENSG00000153317**	ASAP1	3068.395462	2553.835767	3351.383931	2948.736925	2980.779698	3172.769603	837.5435186	1177.870659	906.0354176	957.3924368	1042.980913	1010.761134
**ENSG00000153339**	TRAPPC8	1123.578357	1054.464425	1265.318423	1158.731376	1125.738436	1297.864978	1214.438102	1204.451661	1178.971553	1211.769013	1294.068911	1274.658602
**ENSG00000153363**	LINC00467	285.464839	462.4229373	267.8827292	182.5169363	208.0420107	182.1032416	251.2630556	249.1968954	271.0602854	263.2354618	242.8103713	257.9766911
**ENSG00000153391**	INO80C	134.9981505	108.5993262	105.8503185	238.6115911	128.2925733	174.1362248	178.7440436	142.8728867	166.4816632	199.9577066	124.1643944	158.1693155
**ENSG00000153395**	LPCAT1	1500.448193	1261.153465	1289.74542	1475.205696	1794.940237	1453.790879	1571.926189	3171.445822	1472.073072	1473.106142	2910.965247	1453.127722
**ENSG00000153404**	PLEKHG4B	0	0	6.513865756	2.511700959	0	2.27629052	7.149761744	18.274439	3.28273702	0.632777552	12.41643944	4.229126084
**ENSG00000153406**	NMRAL1	293.9022234	231.2114686	308.5943902	386.8019476	322.4651166	379.7611351	255.3486337	250.8582081	325.9288899	301.8348925	311.7905904	257.9766911
**ENSG00000153443**	UBALD1	81.56138258	63.05767327	95.26528668	61.95529031	60.10102531	113.0557625	81.71156279	89.71088235	93.79248629	101.8771859	68.98021912	79.50757038
**ENSG00000153485**	TMEM251	92.81122845	94.5865099	56.18209214	66.14145858	73.97049269	76.25573241	81.71156279	99.67875817	104.1096598	98.71329817	71.73942788	115.8780547
**ENSG00000153487**	ING1	531.5552175	550.0030391	540.6508577	521.5965657	585.9849968	569.07263	563.8097833	682.7994934	645.7612681	539.7592522	743.6067621	685.1184256
**ENSG00000153495**	TEX29	28.12461468	52.54806106	70.83829009	2.511700959	0	2.27629052	1.021394535	4.983937908	3.28273702	2.53111021	0	0.845825217
**ENSG00000153531**	ADPRHL1	92.81122845	182.1666117	96.89375311	125.5850479	202.2630659	150.6145561	29.62044151	36.54887799	35.64114479	28.47498986	41.38813147	27.91223215
**ENSG00000153551**	CMTM7	184.2162262	94.5865099	126.206149	161.586095	152.5641412	115.332053	3.064183605	21.59706427	18.28953483	12.65555105	11.03683506	10.1499026
**ENSG00000153558**	FBXL2	97.02992066	206.6890402	83.8660216	97.95633739	105.1767943	92.56914781	0	6.645250545	1.875849726	0.632777552	5.518417529	4.229126084
**ENSG00000153560**	UBP1	944.9870534	767.2016914	1158.653871	1141.149469	889.9574902	1068.339017	1427.90956	1312.436983	1452.845613	1326.934527	1357.530712	1532.635293
**ENSG00000153561**	RMND5A	898.5814391	1135.038119	1008.020726	1124.404796	1351.117281	1339.976353	1132.726539	995.126269	1120.820211	1414.890607	1364.428734	1627.367717
**ENSG00000153574**	RPIA	182.8099954	101.592918	186.4594073	166.6094969	125.9809954	148.3382655	211.4286687	194.3735784	197.4331836	186.669378	133.8216251	218.2229059
**ENSG00000153714**	LURAP1L	175.7788418	168.1537954	210.0721706	81.21166433	131.7599401	134.6805224	146.0594185	127.921073	92.385599	111.3688492	139.3400426	91.34912341
**ENSG00000153721**	CNKSR3	195.466072	119.1089384	121.3207497	232.7509555	172.2125533	122.9196881	16.34231256	4.983937908	13.59991051	10.12444084	5.518417529	12.68737825
**ENSG00000153767**	GTF2E1	379.6822982	252.2306931	364.7764823	311.4509189	382.5661419	297.8146763	469.841486	486.7646024	402.8387286	352.4570967	420.7793366	433.062511
**ENSG00000153774**	CFDP1	556.8673707	479.9389576	491.7968645	537.5040051	606.7891978	445.0147966	433.0712828	485.1032897	409.8731651	482.1764949	426.2977541	384.8504736
**ENSG00000153779**	TGIF2LX	0	0	0	0	0	0	16.34231256	0	4.689624315	3.163887762	4.138813147	7.612426951
**ENSG00000153786**	ZDHHC7	984.3615139	777.7113036	1069.90245	1249.15261	1113.024757	1378.673292	910.0625306	1569.940441	1215.550622	1061.800733	1604.479897	1271.275301
**ENSG00000153790**	C7orf31	5.624922937	17.51602035	3.256932878	6.697869223	9.246311586	5.6907263	46.9841486	23.25837691	27.19982102	27.20943475	23.4532745	16.91650434

ENSG00000153814	JAZF1	8.437384405	3.50320407	1.628466439	0.837233653	13.86946738	4.173199286	1.021394535	4.983937908	0.937924863	1.265555105	4.138813147	0
ENSG00000153815	CMIP	2195.126176	1975.807096	2241.584053	2638.960474	2523.087274	2838.534278	1983.548187	2254.401247	2274.936755	2206.495325	2593.656239	2293.032163
ENSG00000153822	KCNJ16	0	0	0.814233219	0	0	0.758763507	99.07526988	88.04956971	166.0127007	175.279382	84.15586732	179.314946
ENSG00000153823	PID1	43.59315276	31.52883663	42.34012741	44.3733836	45.07576898	49.69900968	0	0	0	0	0	0.845825217
ENSG00000153827	TRIP12	6309.757304	6978.382508	6679.155099	5857.286635	6066.736189	6161.159674	4888.394244	4691.546884	4755.279055	4514.235059	5053.490852	4853.345094
ENSG00000153832	FBXO36	4.218692202	14.01281628	6.513865756	12.55850479	25.42735686	14.79588838	22.47067977	26.58100218	22.51019671	32.27165517	22.07367012	30.4497078
ENSG00000153879	CEBPG	1102.484896	882.8074257	1051.98932	799.5581385	883.0227565	934.4172584	1021.394535	995.126269	1077.206705	859.3119161	776.7172672	880.5040507
ENSG00000153885	KCTD15	582.1795239	564.0158553	531.6942923	556.7603792	473.8734688	595.2499709	530.1037636	677.8155555	563.2238802	503.6909317	506.3148083	424.6042588
ENSG00000153896	ZNF599	56.24922937	70.06408141	69.20982365	45.21061725	85.52838217	74.35882365	93.96829721	94.69482026	83.4753128	91.7527451	85.5354717	101.499026
ENSG00000153898	MCOLN2	5.624922937	31.52883663	13.84196473	4.186168264	2.311577897	10.62268909	38.81299233	18.274439	10.78613592	41.1305409	2.759208765	4.229126084
ENSG00000153902	LGI4	19.68723028	3.50320407	20.35583049	15.07020575	16.18104528	12.89897961	0	0	6.096511609	1.898332657	0	1.691650434
ENSG00000153904	DDAH1	1634.040113	1411.79124	1767.700319	1297.712162	1196.241561	1774.747842	372.8090052	505.0390414	462.8659198	277.7893455	340.7622824	427.1417345
ENSG00000153914	SREK1	3021.989848	2907.659378	2742.337483	3055.902833	2827.059767	2705.750665	4464.515512	3970.5372	3567.866178	4173.167958	4290.569629	3939.008035
ENSG00000153922	CHD1	2656.369857	2448.739645	2547.735744	2707.613633	2300.020007	2494.435028	2811.899155	2545.130959	2543.183266	2582.365191	2186.672946	2687.186714
ENSG00000153930	ANKFN1	42.18692202	52.54806106	89.56565414	18.41914036	62.41260321	33.00621254	13.27812895	4.983937908	11.72406079	20.88165923	5.518417529	5.074951301
ENSG00000153933	DGKE	404.9944514	367.8364274	460.041769	374.2434428	344.4251066	415.4230199	319.6964894	397.05372	323.5840777	342.3326558	378.0116008	340.0217371
ENSG00000153936	HS2ST1	500.6181414	381.8492437	404.6739101	596.9475945	604.4776199	550.8623058	666.9706313	767.5264379	691.250624	767.559171	801.5501461	875.4290994
ENSG00000153944	MSI2	1147.484279	1029.941997	877.7434106	1601.627978	1383.479371	1493.625963	3092.782652	1727.765142	2869.112156	3461.925989	1934.205344	3099.949419
ENSG00000153956	CACNA2D1	1.406230734	0	4.885399317	0.837233653	1.155788948	10.24330734	1.021394535	0	3.28273702	0.632777552	1.379604382	0
ENSG00000153975	ZUFSP	392.3383748	507.9645902	390.0177121	328.1955919	275.0777697	351.3075036	366.680638	426.9573475	377.9837198	372.7059784	444.2326111	417.8376571
ENSG00000153982	GDPD1	28.12461468	21.01922442	6.513865756	13.39573845	24.27156791	16.31341539	9.192550814	4.983937908	23.917084	14.5538837	16.55525259	10.99572782
ENSG00000153989	NUS1	2141.689408	1415.294444	1998.128321	2070.478824	1494.43511	1995.168641	1283.89293	1018.384646	1469.72826	1290.866207	1099.544693	1588.459757
ENSG00000153993	SEMA3D	1742.31988	1961.794279	2594.96127	787.8368673	1102.622657	1250.821641	8.171156279	3.322625272	7.034436472	3.163887762	9.657230676	6.766601734
ENSG00000154001	PPP2R5E	1185.452509	1184.082976	1060.131652	1267.57175	1346.494125	1193.534996	1714.921424	1654.667386	1655.437383	1889.473771	1662.423281	1958.085377
ENSG00000154025	SLC5A10	0	0	0	0	3.467366845	0	0	8.306563181	4.220661883	0.632777552	0	5.074951301
ENSG00000154027	AK5	0	0	0	0	0	0	0	8.306563181	1.406887294	0	0	1.691650434
ENSG00000154040	CABYR	39.37446056	42.03844885	56.99632536	91.25846816	101.7094274	95.98358359	44.94135953	33.22625272	36.11010722	41.1305409	41.38813147	60.89941561
ENSG00000154059	IMPACT	691.8655212	581.5318757	651.3865756	524.9455003	514.326082	541.7571437	806.9016826	664.5250545	785.0431103	805.5258242	597.3686975	768.855122
ENSG00000154065	ANKRD29	1.406230734	7.006408141	9.770798633	6.697869223	8.090522638	2.27629052	1.021394535	0	1.875849726	0	2.759208765	0
ENSG00000154079	SDHAF4	18.28099954	38.53524477	28.49816268	35.16381342	27.73893476	19.72785117	54.13391035	44.85544118	40.79973154	39.8649858	26.21248326	37.21630954
ENSG00000154096	THY1	30.93707615	49.04485699	61.06749146	27.62871054	158.3430859	140.7506305	0	1.661312636	1.875849726	4.429442867	0	0
ENSG00000154102	C16orf74	127.9669968	140.1281628	148.1904459	196.7499084	85.52838217	138.0949582	35.74880872	33.22625272	61.90304095	27.20943475	41.38813147	77.81591994
ENSG00000154114	TBCEL	320.6206074	350.320407	347.6775847	382.6157794	428.7976998	337.2703787	338.081591	257.5034586	241.5156522	306.2643354	277.3004808	263.8974676
ENSG00000154122	ANKH	701.7091363	599.047896	582.1767519	1213.151563	1022.873219	1133.592679	491.2907713	338.9077778	497.5691398	600.5058972	500.7963908	688.5017265
ENSG00000154124	OTULIN	2114.971024	2263.069829	2219.599756	2391.139313	2152.079022	2261.874013	2317.5442	2477.01714	2437.197756	2369.751934	2530.194437	2671.96186
ENSG00000154127	UBASH3B	4334.003123	4872.956862	4238.083907	4826.652009	6640.007508	5513.175639	1246.101333	2884.038736	1022.338101	1809.111022	3471.084626	1585.076456
ENSG00000154133	ROBO4	97.02992066	143.6313669	162.0324107	72.8393278	83.21680428	110.779472	0	0	4.220661883	0	0	0
ENSG00000154134	ROBO3	142.0293041	185.6698157	156.3327781	114.7010104	106.3325832	95.22482008	1.021394535	8.306563181	3.751699452	1.898332657	0	0
ENSG00000154143	PANX3	7.031153671	7.006408141	1.628466439	6.697869223	10.40210053	5.6907263	3.064183605	11.62918845	1.875849726	3.796665314	6.898021912	9.304077385
ENSG00000154144	TBRG1	622.9602152	704.1440182	569.9632536	527.4572013	551.3113283	542.895289	588.3232521	496.7324782	542.1205708	506.2220419	536.6661047	466.8955197
ENSG00000154146	NRGN	14.06230734	10.50961221	7.328098975	15.9074394	21.95999002	13.27836137	5.106972674	18.274439	3.28273702	9.491663286	9.657230676	2.53747565
ENSG00000154153	RETREG1	403.5882207	364.3332233	317.5509556	674.8103242	807.8964748	715.8933685	1096.97773	1194.483785	1449.093913	1210.503458	1140.932824	1765.237227
ENSG00000154162	CDH12	84.37384405	59.5544692	40.71166097	68.65315954	75.12628164	65.25366157	1.021394535	0	0.468962431	0	0	0
ENSG00000154174	TOMM70	1653.727343	1261.153465	1480.275993	1806.750223	1771.824458	1694.698292	1895.708257	1397.163927	1742.195433	1880.614886	1485.83392	2275.269833
ENSG00000154175	ABI3BP	56.24922937	80.57369362	82.23755516	97.11910373	100.5536385	86.87842151	0	8.306563181	3.751699452	3.163887762	6.898021912	2.53747565
ENSG00000154188	ANGPT1	265.7776088	248.727489	218.2145028	272.1009372	246.183046	228.3878155	84.77574639	107.9853213	143.9714665	132.883286	131.0624163	158.1693155
ENSG00000154217	PITPNC1	940.7683612	588.5382838	546.3504902	612.0178002	388.3450866	401.385895	152.1877857	337.2464651	151.9438278	198.0593739	480.102325	232.6019346
ENSG00000154222	CC2D1B	662.3346758	924.8458746	626.959579	597.7848281	686.5386353	620.2891667	625.0934553	666.1863671	606.8373863	694.7897525	739.4679489	599.6900787
ENSG00000154227	CERS3	5.624922937	0	6.513865756	0	0	0.758763507	3.064183605	0	2.813774589	0	0	0
ENSG00000154229	PRKCA	4.218692202	7.006408141	13.84196473	9.209570182	28.89472371	15.55465189	15.32091802	11.62918845	4.689624315	17.08499391	20.69406573	17.76232955
ENSG00000154237	LRRK1	14.06230734	38.53524477	8.956565414	20.93084132	27.73893476	23.14228695	191.000778	161.1473257	171.6402499	205.6527045	197.2834267	171.702519
ENSG00000154258	ABCA9	7.031153671	0	1.628466439	7.535102876	2.311577897	1.13814526	3.064183605	0	4.220661883	6.960553076	4.138813147	7.612426951
ENSG00000154262	ABCA6	8.437384405	45.54165292	26.86969624	0.837233653	8.090522638	3.035054026	0	3.322625272	2.344812157	1.265555105	1.379604382	5.074951301
ENSG00000154263	ABCA10	63.28038304	63.05767327	74.09522297	10.04680383	33.5178795	14.79588838	28.59904698	26.58100218	22.04123428	15.81943881	13.79604382	15.2248539
ENSG00000154265	ABCA5	1406.230734	1818.162913	1496.560657	940.2133922	1130.361591	927.5883868	2426.833415	2777.714728	2179.268419	1938.197643	2423.9649	2047.74285
ENSG00000154274	C4orf19	468.2748345	311.7851623	487.7256984	491.4561542	621.8144542	388.4869154	801.7947099	819.0271296	815.9946307	681.5014239	709.1166525	757.8593942
ENSG00000154277	UCHL1	4.218692202	10.50961221	17.09889761	5.86063557	8.090522638	10.24330734	3.064183605	0	5.158586746	1.898332657	0	0
ENSG00000154305	MIA3	1607.321729	1576.441832	1618.69564	1542.184389	1615.79295	1474.277493	2021.339785	2154.722489	1791.436488	1788.229363	1945.242179	1903.952563
ENSG00000154309	DISP1	112.4984587	87.58010176	87.12295448	87.90953355	79.74943743	128.2310326	156.2733638	139.5502614	136.93703	132.883286	97.95191114	153.0943642
ENSG00000154310	TNIK	0	0	0.814233219	0	4.623155793	0.379381753	0	4.983937908	0.468962431	0.632777552	0	3.383300867
ENSG00000154319	FAM167A	2002.472565	1110.51569	1247.405292	2513.375426	2281.527384	1676.108586	3.064183605	8.306563181	4.689624315	13.2883286	9.657230676	0.845825217
ENSG00000154328	NEIL2	127.9669968	112.1025303	119.6922833	174.1445998	191.8609654	138.47434	36.77020326	34.88756536	41.7376564	32.27165517	46.906549	40.59961041
ENSG00000154358	OBSCN	14.06230734	105.0961221	26.86969624	17.58190671	76.28207059	25.41857747	4.08557814	58.14594226	4.689624315	3.796665314	31.73090079	1.691650434
ENSG00000154359	LONRF1	126.5607661	308.2819582	174.245909	62.79252397	92.46311586	115.332053	96.01108628	96.3561329	94.26144872	100.6116308	71.73942788	110.8031034
ENSG00000154370	TRIM11	686.2405983	795.227324	745.0233958	524.9455003	505.0797704	675.6789026	567.8953614	641.2666775	600.2719123	521.4087032	536.6661047	575.1611474
ENSG00000154380	ENAH	898.5814391	686.6279978	565.0778543	1413.250406	1212.422607	1126.384426	1701.643295	1249.307102	1367.025488	1642.690526	1520.324029	1766.928878
ENSG00000154415	PPP1R3A	0	0	4.885399317	0	0	0	0	3.322625272	0	3.163887762	0	0

**ENSG00000154429**	CCSAP	1517.322962	1369.752792	1266.946889	2396.162715	2406.35259	1982.649043	2760.829428	4743.047576	2816.119401	2424.803581	4503.028704	2962.079909
**ENSG00000154438**	ASZ1	0	0	7.328098975	0.837233653	3.467366845	0.758763507	1.021394535	3.322625272	3.28273702	0.632777552	1.379604382	0.845825217
**ENSG00000154447**	SH3RF1	637.0225226	479.9389576	756.4226609	695.7411655	557.0902731	843.3656376	422.8573374	365.4887799	545.8722702	437.2492887	382.1504139	598.8442535
**ENSG00000154451**	GBP5	9830.959062	12653.5731	12117.41877	0	5.778944741	7.587635066	5241.796753	4100.119586	3878.319308	15.81943881	16.55525259	20.2998052
**ENSG00000154473**	BUB3	2712.619086	2207.018564	2561.577708	2853.292289	2480.323083	2741.412549	2438.068755	2202.900556	2561.003838	2291.287517	2095.619057	2719.328072
**ENSG00000154478**	GPR26	0	17.51602035	1.628466439	0	3.467366845	1.517527013	0	0	0	0	0	0
**ENSG00000154479**	CCDC173	2.812461468	0	0	2.511700959	11.55788948	6.828871559	15.32091802	18.274439	15.47576024	18.35054902	5.518417529	12.68737825
**ENSG00000154511**	FAM69A	0	7.006408141	4.885399317	0	16.18104528	5.311344546	10.21394535	11.62918845	2.813774589	14.5538837	13.79604382	9.304077385
**ENSG00000154518**	ATP5MC3	732.6462125	665.6087734	678.2562718	986.2612431	871.464867	847.9182186	696.5910728	696.0899945	906.9733424	905.5046775	876.0487828	965.0865723
**ENSG00000154529**	CNTNAP3B	133.5919197	126.1153465	118.0638168	92.93293547	56.63365846	79.67016819	166.4873092	171.1152015	224.1640422	155.0305003	164.1729215	181.0065964
**ENSG00000154548**	SRSF12	0	0	0	0	0	0	1.021394535	3.322625272	3.751699452	6.327775524	11.03683506	6.766601734
**ENSG00000154553**	PDLIM3	4.218692202	0	1.628466439	0.837233653	18.49262317	0.379381753	7.149761744	24.91968954	8.441323766	11.38999594	26.21248326	10.1499026
**ENSG00000154556**	SORBS2	189.8411491	147.134571	222.2856689	209.3084132	164.1220307	211.3156366	166.4873092	64.79119281	119.58542	128.4538431	26.21248326	70.20349299
**ENSG00000154582**	ELOC	1177.015124	1254.147057	1207.507864	1249.989844	1121.11528	1423.81972	543.3818926	503.3777287	606.3684239	546.0870277	409.7425015	651.2854169
**ENSG00000154589**	LY96	0	3.50320407	10.58503185	2.511700959	1.155788948	1.13814526	1.021394535	0	1.406887294	0	2.759208765	0
**ENSG00000154608**	CEP170P1	4.218692202	3.50320407	2.442699658	3.348934611	1.155788948	2.27629052	8.171156279	3.322625272	1.406887294	4.429442867	9.657230676	0.845825217
**ENSG00000154611**	PSMA8	8.437384405	3.50320407	0.814233219	0	0	0	2.04278907	6.645250545	3.28273702	1.898332657	2.759208765	2.53747565
**ENSG00000154639**	CXADR	29.53084542	94.5865099	70.02405687	55.25742109	120.2020506	67.15057033	92.94690267	112.9692593	52.52379232	82.26108181	148.9972733	87.11999733
**ENSG00000154640**	BTG3	642.6474455	924.8458746	696.1694026	682.3454271	637.9954994	725.3779123	310.5039386	332.2625272	335.7771009	309.4282231	259.3656239	405.1502788
**ENSG00000154642**	C21orf91	248.9028399	189.1730198	173.4316757	249.4956286	153.7199301	185.8970591	434.0926773	362.1661547	505.5415011	358.7848722	242.8103713	389.9254249
**ENSG00000154646**	TMPRSS15	0	0	0	0	0	0	1.021394535	9.967875817	6.56547404	7.593330629	4.138813147	3.383300867
**ENSG00000154654**	NCAM2	224.9969175	343.3139989	232.0564675	276.2871054	230.0020007	222.6970892	131.759895	73.09775599	114.8957957	104.4082961	96.57230676	82.89087124
**ENSG00000154655**	L3MBTL4	57.6554601	42.03844885	47.22552673	3.348934611	0	2.27629052	14.29952349	19.93575163	11.72406079	1.898332657	12.41643944	3.383300867
**ENSG00000154678**	PDE1C	67.49907524	66.56087734	200.301372	67.81592588	158.3430859	274.2930076	0	1.661312636	0.468962431	0.632777552	0	0
**ENSG00000154710**	RABGEF1	120.9358431	45.54165292	108.2930182	111.3520758	68.19154795	100.9155464	71.49761744	78.0816939	85.82012496	96.18218796	95.19270238	65.12854169
**ENSG00000154719**	MRPL39	507.649295	595.544692	528.4373594	524.9455003	558.246062	492.8168975	417.7503648	420.3120969	411.2800524	406.8759662	474.5839075	477.0454223
**ENSG00000154721**	JAM2	1.406230734	7.006408141	11.39926507	0	3.467366845	5.6907263	1018.330351	1601.505381	769.56735	806.1586017	1356.151108	916.0287098
**ENSG00000154723**	ATP5PF	593.4293698	511.4677943	639.9873105	712.4858386	679.6039016	679.8521019	570.959545	571.4915468	551.9687818	592.9125666	567.0174011	754.4760934
**ENSG00000154727**	GABPA	898.5814391	718.1568344	753.9799612	889.1421393	870.309078	760.6604154	849.800253	958.577391	981.538369	899.1769019	971.2414851	1198.534332
**ENSG00000154734**	ADAMTS1	2127.627101	1650.009117	2570.534274	1286.828124	1012.471119	1802.44271	1457.530001	1498.503998	1594.941229	1147.225702	939.5105844	1199.380157
**ENSG00000154736**	ADAMTS5	1.406230734	0	0	0.837233653	2.311577897	3.41443578	7.149761744	29.90362745	11.25509835	6.327775524	1.379604382	25.3747565
**ENSG00000154743**	TSEN2	417.650528	227.7082646	347.6775847	543.3646407	357.138785	339.5466692	766.0459012	559.8623584	672.4921267	797.299716	604.2667195	652.1312421
**ENSG00000154760**	SLFN13	1330.294275	1895.233402	1292.188119	1170.452647	1328.001502	1180.256635	878.3993	749.2519989	857.2633247	846.6563651	736.7087402	876.2749246
**ENSG00000154764**	WNT7A	486.555834	374.8428355	521.1092604	667.2752213	633.3723436	791.3903374	262.4983955	239.2290196	353.5976733	317.6543313	280.0596896	407.6877545
**ENSG00000154767**	XPC	665.1471373	585.0350798	654.6435084	601.1337628	687.6944242	509.5096947	1639.338228	1633.070321	1446.280139	1543.977228	1534.120073	1212.913361
**ENSG00000154781**	CCDC174	271.4025317	315.2883663	317.5509556	198.4243757	205.7304328	251.1507207	393.2368959	428.6186601	356.4114479	372.0732008	464.9266768	347.6341641
**ENSG00000154783**	FGD5	0	0	1.628466439	0	1.155788948	1.517527013	783.4096082	1656.328698	409.4042027	659.3542096	1982.491497	381.4671728
**ENSG00000154803**	FLCN	274.2149932	388.8556518	378.618447	237.7743574	235.7809454	238.2517411	218.5784305	335.5851525	245.2673516	171.4827167	346.2807	199.6147512
**ENSG00000154813**	DPH3	427.4941432	437.9005088	485.2829988	477.2231821	507.3913483	552.0004511	488.2265877	505.0390414	597.9271001	516.3464827	503.5555995	571.7778465
**ENSG00000154814**	OXNAD1	181.4037647	203.1858361	191.3448066	252.8445632	202.2630659	220.8001804	341.1457746	333.9238399	362.5079595	342.9654334	335.2438649	325.6427085
**ENSG00000154822**	PLCL2	223.5906867	133.1217547	149.8189124	204.2850113	150.2525633	174.1362248	976.4531753	882.1570098	1094.558315	934.6124449	829.1422338	1069.123074
**ENSG00000154832**	CXXC1	334.6829147	290.7659378	381.0611467	301.404115	360.6061519	424.1488002	391.1941069	400.3763453	388.3008932	406.8759662	456.6490505	352.7091154
**ENSG00000154839**	SKA1	825.4574409	686.6279978	959.9809657	923.4687191	997.4458624	978.4255418	698.6338619	644.5893028	777.5397113	758.0675078	707.7370481	793.3840533
**ENSG00000154845**	PPP4R1	2796.99293	2490.778094	2765.950246	2777.94126	2506.906229	2875.334308	2161.270836	1859.00884	2180.675306	2045.137049	1983.871102	2013.064016
**ENSG00000154856**	APCDD1	7.031153671	3.50320407	4.885399317	18.41914036	9.246311586	21.24537819	28.59904698	26.58100218	11.25509835	36.06832049	33.11050518	21.99145564
**ENSG00000154864**	PIEZO2	5.624922937	3.50320407	21.17006371	12.55850479	17.33683422	14.79588838	5.106972674	16.61312636	27.66878346	6.327775524	17.93485697	30.4497078
**ENSG00000154874**	CCDC144B	137.8106119	129.6185506	123.7634494	69.49039319	99.39784955	104.3299822	135.8454731	94.69482026	66.12370283	106.3066288	80.01705417	43.98291127
**ENSG00000154889**	MPPE1	397.9632978	483.4421617	411.1877758	288.0083766	411.4608656	315.6456187	680.2487602	536.6039815	630.7544703	587.2175686	444.2326111	517.6450327
**ENSG00000154898**	CCDC144CF	84.37384405	98.08971397	87.9371877	44.3733836	123.6694175	65.63304332	133.8026841	147.8568246	86.28908739	62.01220013	96.57230676	94.73242428
**ENSG00000154914**	USP43	226.4031482	315.2883663	275.2108282	135.6318518	176.8357091	184.3795321	519.8898183	313.9880882	521.4862238	418.8987397	252.467602	441.5207632
**ENSG00000154917**	RAB6B	552.6486785	592.0414879	505.6388293	609.5060993	750.1070274	582.7303731	1.021394535	4.983937908	3.28273702	3.796665314	9.657230676	2.53747565
**ENSG00000154920**	EME1	295.3084542	276.7531216	268.6969624	294.7062458	248.4946239	257.6002105	352.3811145	330.6012146	291.2256699	317.0215537	357.317535	274.0473702
**ENSG00000154928**	EPHB1	32.34330689	10.50961221	56.99632536	33.48934611	32.36209055	61.45984404	34.72741419	33.22625272	46.42728071	32.90443272	30.35129641	31.29553302
**ENSG00000154930**	ACSS1	271.4025317	213.6954483	267.068496	62.79252397	83.21680428	63.73613456	35.74880872	46.51675381	35.17218236	19.61610412	24.83287888	3.383300867
**ENSG00000154945**	ANKRD40	1494.82327	1064.974037	1538.900785	1412.413172	1176.593149	1565.329114	1113.320043	837.3015686	1120.351249	970.0479878	882.9468047	1118.180937
**ENSG00000154957**	ZNF18	64.68661377	73.56728548	81.42332194	102.9797393	107.4883722	70.18562436	117.4603715	79.74300653	131.7784432	134.7816187	122.78479	98.11572515
**ENSG00000154975**	CA10	30.93707615	14.01281628	14.65619795	7.535102876	5.778944741	18.21032416	10.21394535	6.645250545	6.56547404	24.04554699	6.898021912	10.1499026
**ENSG00000154978**	VOPP1	171.5601496	276.7531216	267.8827292	204.2850113	312.063016	265.1878456	49.02693767	19.93575163	26.73085859	41.1305409	45.52694462	36.37048432
**ENSG00000154997**	14-Sep	1.406230734	0	0	0	3.467366845	2.27629052	0	0	1.406887294	0	0	0.845825217
**ENSG00000155008**	APOOL	632.8038304	550.0030391	584.6194516	708.2996703	676.1365347	677.9551932	1099.02052	917.0445751	975.9108199	1028.8963	985.037529	1100.418607
**ENSG00000155011**	DKK 2.00	7.031153671	0	1.628466439	0	0	1.517527013	4.08557814	14.95181373	5.627549177	1.265555105	4.138813147	0.845825217
**ENSG00000155016**	CYP2U1	54.84299863	38.53524477	36.64049488	67.81592588	90.15153796	50.83715494	204.278907	181.0830773	163.6678886	209.4493698	150.3768777	157.3234903
**ENSG00000155034**	FBXL18	693.2717519	486.9453658	815.0474527	864.0251298	641.4628663	933.6584949	624.0720608	513.3456046	786.4499975	630.2464422	635.9976202	691.0392021
**ENSG00000155066**	PROM2	369.8386831	269.7467134	284.9816268	859.0017278	662.2670674	682.1283924	488.2265877	383.7632189	446.9211972	618.2236687	601.5075107	473.6621214
**ENSG00000155085**	AK9	116.7171509	161.1473872	96.07951989	41.86168264	87.83996007	62.5979893	181.8082272	212.6480174	168.8264753	203.7543719	255.2268107	178.4691207
**ENSG00000155090**	KLF10	899.9876699	994.909956	1091.886747	771.9294279	674.9807458	925.3120963	1010.159195	973.5292048	1032.186312	887.1541284	871.9099696	910.1079333
**ENSG00000155093**	PTPRN2	14.06230734	49.04485699	12.21349829	2.511700959	17.33683422	11.00207085	4.08557814	3.322625272	3.751699452	6.327775524	9.657230676	0.845825217

**ENSG00000155096**	AZIN1	3200.581151	2410.2044	3069.659237	3874.717345	3009.674421	3982.370264	2643.369056	1702.845452	2965.718417	2475.425785	1830.735015	2836.897777
**ENSG00000155097**	ATP6V1C1	1679.039497	1811.156504	1799.455415	1887.961887	2048.058016	1888.94175	1016.287562	1119.724717	944.0213745	1047.879627	1214.051856	1187.538604
**ENSG00000155099**	PIP4P2	307.9645308	367.8364274	434.8005392	427.8263966	484.2755693	473.8478099	233.8993485	488.425915	378.9216446	230.9638066	378.0116008	332.4093102
**ENSG00000155100**	OTUD6B	554.0549093	462.4229373	585.4336848	729.2305116	631.0607658	761.7985606	524.9967909	310.665463	563.2238802	538.4936971	442.8530067	575.1611474
**ENSG00000155111**	CDK19	461.2436808	676.1183856	499.9391967	538.3412388	785.9364848	606.6314235	681.2701548	910.3993246	674.8369389	716.3041893	974.0006939	706.264056
**ENSG00000155115**	GTF3C6	296.7146849	304.7787541	289.0527929	329.0328256	367.5408855	352.0662671	210.4072742	154.5020752	204.9365825	267.6649047	155.8952952	219.9145564
**ENSG00000155158**	TTC39B	109.6859973	66.56087734	77.35215585	66.97869223	72.81470374	59.94231702	85.79714093	58.14594226	79.25465092	60.11386748	82.77626294	55.82446431
**ENSG00000155189**	AGPAT5	1254.357815	1075.48365	1108.171412	1337.062144	1204.332084	1336.182535	457.5847516	604.7177995	510.2311254	638.4725504	779.476476	651.2854169
**ENSG00000155229**	MMS19	673.5845217	774.2080996	698.6121023	877.4208682	891.1132791	817.5676784	740.5110378	604.7177995	697.816098	795.4013833	807.0685637	592.923477
**ENSG00000155252**	PI4K2A	610.3041386	760.1952833	741.7664629	612.8550339	790.5596406	711.7201692	522.9540019	802.4140032	637.3199443	505.5892644	833.2810469	663.12697
**ENSG00000155254**	MARVELD1	932.3309767	963.3811194	895.6565414	1062.449505	1428.55514	1255.753603	971.3462027	907.0766993	1141.454558	1064.964621	1174.043329	1108.876859
**ENSG00000155256**	ZFYVE27	352.9639143	430.8941007	429.1009066	389.3136486	394.1240314	410.8704388	316.6323058	307.3428377	377.9837198	329.6771048	354.5583263	323.1052328
**ENSG00000155265**	GOLGA7B	35.15576835	108.5993262	58.6247918	20.09360767	69.3473369	46.66395566	3.064183605	0	0.937924863	0	2.759208765	0.845825217
**ENSG00000155269**	GPR78	0	3.50320407	2.442699658	0	0	0	4.08557814	0	4.689624315	0	4.138813147	0.845825217
**ENSG00000155275**	TRMT44	191.2473798	115.6057343	168.5462764	146.5158893	92.46311586	183.6207686	185.8938053	179.4217647	174.4540245	167.0532738	176.5893609	157.3234903
**ENSG00000155287**	SLC25A28	459.8374501	472.9325495	413.6304755	213.4945815	230.0020007	248.1156667	300.2899933	199.3575163	292.1635948	208.1838147	172.4505478	261.359992
**ENSG00000155304**	HSPA13	1072.95405	1233.127833	1188.7805	949.4229624	945.4353597	1112.726682	775.238452	837.3015686	776.1328241	732.1236281	698.0798174	810.3005577
**ENSG00000155307**	SAMSN1	0	7.006408141	5.699632536	1.674467306	3.467366845	6.070108053	0	0	0	5.062220419	0	0
**ENSG00000155313**	USP25	1243.107969	1205.1022	1122.013376	939.3761585	947.7469376	889.2708297	1296.149665	1232.693976	1087.992841	1048.512404	983.6579246	991.3071541
**ENSG00000155324**	GRAMD2B	3777.135752	3492.694458	4488.053506	2754.498718	2132.43061	2747.103276	3643.314306	3433.933219	3747.009827	3280.318832	2910.965247	3677.648043
**ENSG00000155329**	ZCCHC10	336.0891455	294.2691419	408.7450762	389.3136486	294.7261818	360.0332839	431.0284937	476.7967266	448.7970469	408.7742988	390.4280402	493.9619266
**ENSG00000155330**	C16orf87	134.9981505	140.1281628	159.589711	226.0530863	172.2125533	224.9733797	165.4659146	154.5020752	153.8196775	167.0532738	114.5071637	161.5526164
**ENSG00000155363**	MOV10	3290.579918	3730.912335	3252.047478	1611.674782	1809.965493	1481.485747	2877.268405	2910.619738	2806.27119	1850.874341	2099.75787	1594.380534
**ENSG00000155366**	RHOC	1265.607661	1180.579772	1429.793533	1466.83336	1392.725683	1699.250873	736.4254596	692.7673693	919.6353281	792.8702731	826.383025	821.2962855
**ENSG00000155367**	PPM1J	14.06230734	0	6.513865756	31.81487881	12.71367843	11.00207085	10.21394535	0	5.627549177	6.960553076	6.898021912	8.458252168
**ENSG00000155368**	DBI	725.6150588	854.7817932	719.782166	855.6527932	811.3638417	810.3594251	631.2218226	780.816939	620.4372968	603.0370074	787.7541023	795.0757038
**ENSG00000155380**	SLC16A1	4346.659199	3009.252296	3719.417346	5914.218524	5004.566146	5801.885153	4711.692989	5553.768143	5585.342559	5324.190326	5561.185265	6059.491853
**ENSG00000155393**	HEATR3	435.9315276	318.7915704	348.4918179	465.501911	451.9134788	376.3466993	320.717884	382.1019063	327.3357772	379.0337539	376.6319964	387.3879493
**ENSG00000155428**	TRIM74	0	0	2.442699658	0.837233653	4.623155793	0.758763507	0	1.661312636	0	0	0	0.845825217
**ENSG00000155438**	NIFK	548.4299863	378.3460396	509.7099954	571.8305849	413.7724435	525.4437283	513.761451	365.4887799	532.2723597	444.8426193	390.4280402	478.7370727
**ENSG00000155463**	OXA1L	672.1782909	560.5126513	786.54929	705.7879694	687.6944242	699.9593348	1095.956336	946.9482026	989.0417679	1020.670192	1031.944078	1051.360744
**ENSG00000155465**	SLC7A7	1.406230734	14.01281628	0	2.511700959	1.155788948	4.173199286	15.32091802	28.24231481	48.30313044	31.00610007	15.17564821	40.59961041
**ENSG00000155495**	MAGEC1	165.9352266	262.7403053	286.6100932	138.1435527	184.9262317	286.4332237	241.0491102	171.1152015	211.5020566	189.2004882	132.4420207	182.6982468
**ENSG00000155506**	LARP1	9220.654924	6022.007797	9423.935282	8942.492646	7959.918487	9847.232789	5789.264224	3998.779515	6565.005078	5583.629122	4340.235387	6165.220005
**ENSG00000155508**	CNOT8	790.3016726	1180.579772	808.5335869	957.7952989	1076.039511	918.103843	877.3779055	1280.872042	946.3661867	928.2846693	1353.391899	1052.20657
**ENSG00000155511**	GRIA1	14.06230734	14.01281628	38.26896131	10.04680383	48.54313583	39.8350841	0	3.322625272	1.406887294	0	1.379604382	2.53747565
**ENSG00000155530**	LRGUK	5.624922937	0	0.814233219	9.209570182	8.090522638	4.173199286	8.171156279	4.983937908	1.875849726	6.960553076	17.93485697	5.920776517
**ENSG00000155542**	SETD9	74.53022891	45.54165292	66.76712399	123.073347	107.4883722	100.5361646	117.4603715	86.38825708	104.1096598	98.71329817	107.6091418	119.2613556
**ENSG00000155545**	MIER3	829.6761332	1128.031711	1049.54662	803.7443068	869.1532891	859.679053	840.6077022	998.4488943	942.6144872	811.8535997	778.0968716	954.0908445
**ENSG00000155561**	NUP205	3476.202375	3159.890072	3445.020751	3575.824931	3337.918483	3637.891632	3290.933191	3186.397636	3396.694891	3475.214318	3264.143969	3960.153665
**ENSG00000155592**	ZKSCAN2	762.1770579	907.3298542	670.1139396	828.8613163	760.509128	687.439737	802.8161044	840.6241939	660.7680659	858.6791386	751.8843884	738.4054142
**ENSG00000155621**	C9orf85	417.650528	388.8556518	364.7764823	270.4264699	258.8967244	325.5095443	409.5792085	388.7471569	359.2252225	356.253762	307.6517773	379.7755223
**ENSG00000155622**	XAGE2	39.37446056	21.01922442	30.12662912	61.95529031	63.56839215	75.87635066	427.9643101	546.5718573	438.9488358	576.4603502	584.9522581	674.1226978
**ENSG00000155629**	PIK3AP1	82.96761332	210.1922442	106.6645517	105.4914403	179.147287	114.5732895	3338.938735	5284.635496	2496.287023	2491.245224	4187.0993	1908.181689
**ENSG00000155636**	RBM45	144.8417656	175.1602035	166.1035768	123.9105806	151.4083522	127.4722691	214.4928523	146.195512	184.3022356	198.0593739	144.8584601	171.702519
**ENSG00000155657**	TTN	32.34330689	59.5544692	74.09522297	10.04680383	99.39784955	62.5979893	32.68462512	39.87150327	22.51019671	5.694997971	8.277626294	17.76232955
**ENSG00000155660**	PDIA4	7168.964283	5836.337981	7142.453801	3277.769751	2909.120783	3654.963811	2949.787417	3204.672075	3631.176107	2889.262304	2749.551534	3645.506684
**ENSG00000155666**	KDM8	61.8741523	63.05767327	106.6645517	96.28187008	83.21680428	102.4330734	129.7171059	134.5663235	108.7992841	132.883286	195.9038223	109.9572782
**ENSG00000155719**	OTOA	1.406230734	0	5.699632536	0	0	0.379381753	0	0	2.344812157	0	0	0
**ENSG00000155729**	KCTD18	237.6529941	262.7403053	206.8152377	226.0530863	209.1977996	190.8290219	278.840708	285.7457734	280.9084964	287.9137863	293.8557334	230.064459
**ENSG00000155744**	FAM126B	696.0842134	634.0799367	742.5806961	512.3869956	548.9997504	620.6685484	586.280463	676.1542429	592.2995509	603.669785	607.0259282	548.9405657
**ENSG00000155749**	ALS2CR12	7.031153671	3.50320407	8.956565414	4.186168264	5.778944741	5.311344546	20.4278907	3.322625272	9.848211061	15.81943881	15.17564821	1.691650434
**ENSG00000155754**	C2CD6	0	0	2.442699658	0	4.623155793	1.517527013	0	0	0	0	0	0
**ENSG00000155755**	TMEM237	395.1508363	343.3139989	355.8199169	401.8721534	412.6166545	373.691027	372.8090052	377.1179684	360.6321098	381.5648641	289.7169203	362.0131928
**ENSG00000155760**	FZD7	158.904073	231.2114686	216.5860364	193.4009738	273.9219807	318.301291	80.69016826	169.4538889	234.4812157	82.89385936	234.532745	225.8353329
**ENSG00000155761**	SPAG17	0	0	0	0.837233653	2.311577897	0	149.1236021	84.72694444	84.41323766	97.44774307	55.18417529	43.98291127
**ENSG00000155792**	DEPTOR	4.218692202	7.006408141	3.256932878	5.86063557	2.311577897	1.13814526	49.02693767	144.5341993	166.4816632	66.441643	205.561053	213.9937798
**ENSG00000155816**	FMN2	29.53084542	38.53524477	21.98429693	30.1404115	50.85471372	45.5258104	1.021394535	9.967875817	10.31717349	3.163887762	1.379604382	16.91650434
**ENSG00000155827**	RNF20	1569.353499	1856.698157	1624.395273	1732.236428	1836.548639	1679.523022	1788.461831	1981.945975	1850.056792	1910.355431	2062.508552	2047.74285
**ENSG00000155846**	PPARGC1B	247.4966092	115.6057343	148.1904459	425.3146957	156.031508	225.7321432	519.8898183	290.7297113	435.6660988	661.8853198	307.6517773	466.0496944
**ENSG00000155849**	ELMO1	64.68661377	59.5544692	124.5776826	12.55850479	26.58314581	34.1443578	2.04278907	0	3.28273702	2.53111021	1.379604382	9.304077385
**ENSG00000155850**	SLC26A2	8735.505321	6421.373061	9309.128398	9996.569815	9554.907235	10981.96361	5955.751533	4178.20128	6804.64488	7004.847505	5420.465618	8843.102641
**ENSG00000155858**	LSM 11.00	288.2773005	269.7467134	312.6655563	384.2902467	374.4756192	367.620919	632.2432171	727.6549346	624.6579587	603.0370074	782.2356848	706.264056
**ENSG00000155868**	MED7	306.5583	367.8364274	314.2940227	262.891367	346.7366845	283.018788	269.6481572	305.681525	292.6325572	243.6193577	262.1248326	315.4928059
**ENSG00000155875**	SAXO1	2.812461468	0	0	1.674467306	0	1.896908767	1.021394535	0	0.468962431	0	4.138813147	2.53747565
**ENSG00000155876**	RRAGA	376.8698368	385.3524477	345.234885	380.1040784	480.8082025	464.7426478	323.7820676	493.4098529	322.1771904	295.507117	409.7425015	296.0388259
**ENSG00000155886**	SLC24A2	15.46853808	28.02563256	3.256932878	3.348934611	5.778944741	4.931962793	4.08557814	0	3.751699452	1.265555105	1.379604382	3.383300867

**ENSG00000155893**	PXYLP1	265.7776088	378.3460396	226.356835	51.90848648	40.45261319	37.55879358	896.7844016	370.4727179	472.7141309	721.9991873	314.5497992	292.655525
**ENSG00000155903**	RASA2	815.6138258	938.8586909	845.988315	669.7869223	649.5533889	706.4088247	594.4516193	662.8637418	640.6026814	611.2631156	652.5528728	602.2275543
**ENSG00000155906**	RMND1	517.4929102	465.9261414	501.5676632	539.1784724	463.4713683	474.9859551	634.2860062	649.5732407	527.5827354	551.1492481	634.6180159	486.3494996
**ENSG00000155918**	RAET1L	37.96822982	7.006408141	28.49816268	52.74572013	31.2063016	50.07839144	0	6.645250545	4.689624315	2.53111021	0	5.074951301
**ENSG00000155957**	TMBIM4	113.9046895	164.6505913	97.70798633	109.6776085	84.37259322	97.12172885	180.7868327	149.5181373	159.4472267	185.4038228	144.8584601	193.6939746
**ENSG00000155959**	VBP1	679.2094446	672.6151815	625.3311125	836.3964192	693.473369	740.5531825	676.1631821	729.3162473	639.6647565	725.7958526	731.1903226	712.1848325
**ENSG00000155961**	RAB39B	5.624922937	0	0	0	0	2.27629052	64.3478557	53.16200436	41.26869397	57.58275727	40.00852709	29.60388259
**ENSG00000155962**	CLIC2	0	3.50320407	1.628466439	0	0	1.517527013	6.128367209	3.322625272	3.751699452	7.593330629	17.93485697	5.920776517
**ENSG00000155974**	GRIP1	0	3.50320407	1.628466439	2.511700959	0	1.517527013	3.064183605	1.661312636	4.220661883	0.632777552	13.79604382	3.383300867
**ENSG00000155975**	VPS37A	1292.326045	1352.236771	1243.334126	1148.684572	1025.184797	1229.576262	359.5308763	426.9573475	347.5011617	303.1004476	320.0682167	399.2295023
**ENSG00000155980**	KIF5A	16.87476881	66.56087734	18.72736405	22.60530863	48.54313583	32.62683078	4.08557814	9.967875817	5.158586746	6.960553076	11.03683506	5.920776517
**ENSG00000156009**	MAGEA8	241.8716863	297.772346	265.4400295	233.5881891	309.7514381	270.4991901	213.4714578	184.4057026	208.2193196	217.0427005	113.1275593	186.0815477
**ENSG00000156011**	PSD3	383.9009904	1040.451609	898.9134743	431.1753312	958.1490381	850.5738909	2.04278907	1.661312636	9.379248629	1.898332657	2.759208765	8.458252168
**ENSG00000156017**	CARNMT1	1065.922896	742.6792629	800.3912547	1183.848385	989.3553397	1162.805074	971.3462027	838.9628812	1125.978798	1175.700692	889.8448266	1470.890052
**ENSG00000156026**	MCU	760.7708272	588.5382838	582.9909851	798.7209048	760.509128	808.841898	585.2590685	624.6535512	537.4309464	583.4209033	622.2015764	629.2939613
**ENSG00000156030**	ELMSAN1	966.0805144	1390.772016	944.5105346	998.8197479	1583.430859	927.2090051	1306.36361	2521.872582	1328.570568	1158.615698	2425.344504	1137.634917
**ENSG00000156042**	CFAP70	30.93707615	7.006408141	17.91313083	10.04680383	30.05051265	11.76083435	28.59904698	49.83937908	20.16538455	25.3111021	49.66575776	26.22058172
**ENSG00000156050**	FAM161B	50.62430643	70.06408141	39.08319453	51.07125282	52.01050267	62.97737105	122.5673442	124.5984477	90.50974927	95.54941041	121.4051856	64.28271647
**ENSG00000156052**	GNAQ	5264.927869	3727.409131	3795.141036	6401.48851	5196.427111	5296.92804	7191.63892	5998.999929	6952.368046	7135.199681	6103.369787	7642.876659
**ENSG00000156097**	GPR61	0	0	5.699632536	0	4.623155793	0.379381753	1.021394535	0	0.468962431	0	0	0
**ENSG00000156103**	MMP16	28.12461468	84.07689769	54.5536257	101.305272	142.1620406	133.1629954	19.40649616	36.54887799	19.22745969	10.75721839	59.32298844	18.60815477
**ENSG00000156110**	ADK	2189.501253	2073.89681	2180.516562	1890.473588	1730.216056	2022.484127	1489.193232	1448.664619	1564.458671	1377.556732	1300.966933	1363.470249
**ENSG00000156113**	KCNMA1	829.6761332	592.0414879	586.247918	893.3283076	900.3595907	880.9244312	898.8271907	809.0592538	989.0417679	983.969094	946.4086063	1042.902492
**ENSG00000156136**	DCK	438.7439891	539.4934268	417.7016416	463.8274437	496.9892478	509.8890764	360.5522708	368.8114052	360.6321098	352.4570967	297.9945466	383.1588232
**ENSG00000156140**	ADAMTS3	0	0	4.071166097	0	1.155788948	3.793817533	12.25673442	3.322625272	37.98595695	22.77999189	30.35129641	48.21203736
**ENSG00000156162**	DPY19L4	631.3975996	651.5959571	657.9004413	760.2081568	721.2123037	755.7284526	594.4516193	604.7177995	593.7064382	616.325336	562.878588	666.5102708
**ENSG00000156170**	NDUFAF6	279.8399161	245.2242849	234.4991672	296.3807131	330.5556392	267.8435178	449.4135953	343.8917157	321.708228	437.2492887	402.8444796	317.1844563
**ENSG00000156171**	DRAM2	479.5246803	539.4934268	394.9031114	485.5955187	538.5976499	542.5159072	465.7559079	490.0872277	513.5138624	492.9337133	546.3233354	536.2531874
**ENSG00000156172**	C8orf37	64.68661377	42.03844885	52.11092604	78.69996337	85.52838217	62.97737105	94.98969174	114.6305719	93.79248629	112.0016268	124.1643944	99.80737558
**ENSG00000156206**	CFAP161	1.406230734	0	0	1.674467306	1.155788948	7.208253313	7.149761744	0	2.344812157	1.898332657	2.759208765	2.53747565
**ENSG00000156218**	ADAMTSL3	2.812461468	0	0	5.023401917	1.155788948	3.793817533	0	0	0	0	0	0
**ENSG00000156219**	ART3	1.406230734	0	11.39926507	0	0	0	4.08557814	0	2.813774589	0	5.518417529	3.383300867
**ENSG00000156222**	SLC28A1	7.031153671	0	0	2.511700959	1.155788948	0	1.021394535	1.661312636	0	0.632777552	2.759208765	1.691650434
**ENSG00000156232**	WHAMM	320.6206074	381.8492437	326.507521	219.3552171	270.4546139	251.1507207	165.4659146	172.7765142	212.4399814	137.9455064	172.4505478	186.9273729
**ENSG00000156239**	N6AMT1	241.8716863	199.682632	273.5823617	254.5190305	278.5451365	288.3301325	244.1132938	151.1794499	220.8813052	265.766572	171.0709434	264.7432929
**ENSG00000156253**	RWDD2B	354.370145	210.1922442	271.9538953	279.6360401	219.5999002	207.5218191	225.7281922	219.293268	221.8192301	178.4432698	160.0341083	186.0815477
**ENSG00000156256**	USP16	1400.605811	1268.159873	1345.113279	1494.46207	1279.458366	1284.965998	997.9024606	917.0445751	873.2080474	916.2618958	906.4000792	926.1786124
**ENSG00000156261**	CCT8	4011.976285	3657.34505	4036.968302	4079.002357	3800.234062	4021.446585	2119.39366	2285.966187	2492.535323	2366.588046	2294.282088	2819.135448
**ENSG00000156265**	MAP3K7CL	191.2473798	168.1537954	169.3605096	105.4914403	115.5788948	133.1629954	30.64183605	46.51675381	24.85500887	28.47498986	38.6289227	22.83728085
**ENSG00000156269**	NAA11	566.7109859	542.9966309	664.4143071	581.8773887	564.0250068	684.404683	592.4088302	538.2652941	563.6928426	603.0370074	458.0286549	563.3195944
**ENSG00000156273**	BACH1	5153.835641	6309.270531	5713.474501	2294.857443	2964.598652	2894.682778	3226.585336	5437.476258	3324.474677	2663.360718	4512.685935	2798.835642
**ENSG00000156284**	CLDN8	5.624922937	0	3.256932878	18.41914036	15.02525633	11.00207085	83.75435186	31.56494009	67.99955256	103.142741	42.76773585	160.7067912
**ENSG00000156299**	TIAM1	1004.048744	767.2016914	949.3959339	1584.046071	1425.087773	1550.153844	465.7559079	335.5851525	550.5618945	553.6803583	510.4536215	671.5852221
**ENSG00000156304**	SCAF4	949.2057456	1135.038119	1144.811907	1085.054814	1081.818456	1097.930794	1058.164738	995.126269	1078.14463	1100.400164	1175.422934	1179.926177
**ENSG00000156313**	RPGR	167.3414574	136.6249587	108.2930182	179.1680017	128.2925733	126.3341239	263.51979	204.3414542	228.3847041	226.5343638	166.9321303	197.9231007
**ENSG00000156345**	CDK20	123.7483046	63.05767327	89.56565414	81.21166433	86.68417112	77.77325943	116.438977	69.77513072	86.75804982	80.9955267	110.3683506	105.7281521
**ENSG00000156374**	PCGF6	329.0579918	248.727489	258.1119306	261.2168997	288.9472371	299.3322034	279.8621026	282.4231481	292.6325572	278.422123	304.8925685	311.2636798
**ENSG00000156381**	ANKRD9	127.9669968	136.6249587	96.07951989	124.7478143	164.1220307	137.3361947	265.5625791	285.7457734	260.7431119	241.721025	252.467602	253.747565
**ENSG00000156384**	SFR1	224.9969175	360.8300193	241.013033	240.2860584	358.294574	341.0641962	240.0277157	408.6829085	278.0947219	208.8165923	384.9096227	250.3642642
**ENSG00000156398**	SFXN2	261.5589166	119.1089384	199.4871388	227.7275536	143.3178296	184.3795321	297.2258096	167.7925762	298.7290688	392.95486	235.9123494	226.6811581
**ENSG00000156411**	ATP5MPL	665.1471373	725.1632426	767.0076927	786.1624	835.6354096	857.4027625	571.9809395	531.6200436	684.2161875	669.4786504	641.5160378	670.7393969
**ENSG00000156413**	FUT6	1.406230734	3.50320407	4.885399317	1.674467306	9.246311586	9.863925586	0	0	0	0	0	0
**ENSG00000156414**	TDRD9	30.93707615	66.56087734	58.6247918	60.28082301	99.39784955	78.53202293	223.6854031	206.0027669	136.4680676	242.3538026	295.2353378	159.860966
**ENSG00000156427**	FGF18	0	0	0.814233219	0	0	2.655672273	0	1.661312636	0	0	0	3.383300867
**ENSG00000156453**	PCDH1	7950.828571	7629.978465	9419.049883	7970.464375	8142.533141	9958.012261	4559.505204	3302.689521	5825.920286	4173.800735	3465.566208	4937.08179
**ENSG00000156463**	SH3RF2	1383.731042	865.2914054	1533.201152	1934.009738	1834.237061	2374.929776	458.6061462	594.7499237	501.7898017	512.5498174	482.8615338	488.8869753
**ENSG00000156467**	UQCRB	1497.635732	1516.887362	1415.951569	1888.799121	1792.628659	1533.840429	1072.464262	1275.888105	970.7522331	1120.649045	1289.930097	1374.465977
**ENSG00000156469**	MTERF3	371.2449138	227.7082646	302.0805244	255.3562641	255.4293576	294.7796223	219.599825	210.9867048	227.4467793	185.4038228	179.3485697	224.1436824
**ENSG00000156471**	PTDSS1	1053.26682	728.6664466	865.5299123	1334.550443	1230.91523	1277.378363	542.360498	775.8330011	712.8228958	687.8291994	844.317882	784.9258012
**ENSG00000156475**	PPP2R2B	492.180757	332.8043867	276.8392946	447.0827706	386.0335087	212.4537819	657.7780805	1033.33646	672.0231643	451.1703948	754.6435971	526.9491101
**ENSG00000156482**	RPL30	7886.141957	6540.481999	7446.977025	7520.03267	7236.394605	7787.948632	6849.471751	7972.639341	7807.755521	6803.624243	7067.713251	7298.625796
**ENSG00000156500**	FAM122C	134.9981505	108.5993262	91.19412058	55.25742109	67.035759	50.83715494	159.3375474	147.8568246	127.0888189	94.2838553	129.6828119	102.3448512
**ENSG00000156502**	SUPV3L1	559.6798322	423.8876925	624.5168793	565.1327157	480.8082025	613.8396768	545.4246816	436.9252233	511.1690503	550.5164706	437.3345892	565.0112448
**ENSG00000156504**	FAM122B	750.927212	704.1440182	692.9124697	937.7016912	911.9174802	901.7904276	841.6290967	790.7848148	945.4282618	1006.749086	855.354717	1057.281521
**ENSG00000156508**	EEF1A1	109411.7823	109709.8419	108327.216	89729.67951	91422.90581	93687.18583	96676.01412	115007.6899	104936.4405	86150.13265	97801.53427	92030.01271
**ENSG00000156509**	FBXO43	28.12461468	31.52883663	18.72736405	14.2329721	18.49262317	12.51959786	7.149761744	0	6.56547404	3.796665314	2.759208765	9.304077385
**ENSG00000156510**	HKDC1	18.28099954	31.52883663	28.49816268	15.9074394	32.36209055	13.65774312	1.021394535	3.322625272	10.31717349	3.796665314	5.518417529	3.383300867

**ENSG00000156515**	HK1	4439.470428	5331.876595	4015.798238	3784.296111	4342.299079	3227.779957	4281.68589	5709.93153	4421.377804	4422.482314	5612.230627	4871.107423
**ENSG00000156521**	TYSND1	670.7720602	374.8428355	512.9669283	699.9273338	535.130283	597.9056432	670.0348149	652.895866	760.6570638	746.0447343	696.7002131	771.3925977
**ENSG00000156531**	PHF6	1310.607044	1261.153465	1028.376556	1125.242029	1208.95524	1165.840128	970.3248081	1034.997772	990.9176177	990.929647	924.3349361	1171.467925
**ENSG00000156535**	CD109	4551.968886	4252.889741	3025.690643	5179.127377	5641.405856	3881.075336	3569.773899	3342.561024	2830.657236	3946.633594	3752.52392	3659.885713
**ENSG00000156587**	UBE2L6	3699.793062	3804.47962	3567.969968	409.4072563	587.1407857	498.5076238	3118.317515	3410.674842	3355.895159	606.2008952	572.5358187	557.3988179
**ENSG00000156599**	ZDHHC5	2671.838395	2676.44791	2827.831971	2605.471128	2550.826209	2940.208588	2164.335019	1905.525594	2120.179153	2047.66816	1830.735015	2083.267509
**ENSG00000156603**	MED19	215.1533023	192.6762239	219.8429693	205.9594786	236.9367344	212.0744001	235.9421376	196.0348911	177.2677991	178.4432698	168.3117346	210.610479
**ENSG00000156639**	ZFAND3	850.7695942	1047.458017	1088.629814	729.2305116	831.0122538	935.9347854	793.6235536	795.7687527	871.8011601	784.0113874	823.6238162	807.763082
**ENSG00000156642**	NPTN	739.6773662	630.5767327	728.7387314	765.2315587	804.429108	863.4728705	340.1243801	315.6494009	359.2252225	408.7742988	339.382678	455.0539666
**ENSG00000156650**	KAT6B	805.7702107	770.7048955	688.8413037	1055.751636	1281.769944	899.5141371	1077.571234	1368.921612	914.4767413	1240.87678	1717.607456	1125.793364
**ENSG00000156671**	SAMD8	703.1153671	931.8522827	774.3357917	646.34438	666.8902231	660.5036325	745.6180105	727.6549346	591.8305885	589.1159013	699.4594218	612.3774569
**ENSG00000156675**	RAB11FIP1	1275.451276	1124.528507	1301.144685	1515.392912	894.580646	1320.248501	582.1948849	352.1982789	568.3824669	491.6681582	315.9294035	561.6279439
**ENSG00000156689**	GLYATL2	26.71838395	45.54165292	36.64049488	34.32657977	48.54313583	17.83094241	55.15530488	76.42038126	57.21341664	45.55998377	62.0821972	62.59106604
**ENSG00000156697**	UTP14A	970.2992066	693.6344059	811.7905198	841.4198211	567.4923736	776.594449	635.3074007	576.4754847	596.9891752	613.1614483	510.4536215	699.4974543
**ENSG00000156709**	AIFM1	558.2736015	476.4357536	519.480794	659.7401185	603.321831	558.4499409	533.1679472	534.9426688	581.513415	551.1492481	547.7029398	636.9063882
**ENSG00000156711**	MAPK13	981.5490524	1103.509282	975.4513969	1022.26229	1074.883722	922.2770423	816.0942334	669.5089924	748.933003	883.3574631	771.1988497	814.5296838
**ENSG00000156735**	BAG4	822.6449795	805.7369362	655.4577417	775.2783626	835.6354096	751.934635	395.279685	392.0697821	485.3761166	366.3782028	433.195776	485.5036744
**ENSG00000156787**	TBC1D31	497.8056799	451.9133251	442.1286382	596.9475945	478.4966246	559.9674679	527.03958	450.2157244	499.9139519	556.8442461	484.2411382	601.3817291
**ENSG00000156795**	WDYHV1	329.0579918	238.2178768	317.5509556	309.7764516	352.5156292	239.7692681	228.7923758	260.8260839	272.4671727	271.46157	230.3939318	264.7432929
**ENSG00000156802**	ATAD2	4352.284122	5654.17137	4357.77619	5066.100833	5187.1808	4681.191454	4470.643879	4799.532206	4761.375567	4428.810089	4792.745624	5064.801398
**ENSG00000156804**	FBXO32	261.5589166	486.9453658	512.152695	231.0764882	566.3365846	383.9343343	558.7028106	503.3777287	359.6941849	479.6453847	484.2411382	329.8718345
**ENSG00000156831**	NSMCE2	622.9602152	560.5126513	565.8920875	584.3890897	515.4818709	550.8623058	509.6758729	614.6856754	488.6588536	458.1309479	555.9805661	555.7071674
**ENSG00000156853**	ZNF689	282.6523776	203.1858361	260.5546302	292.1945449	219.5999002	331.9590341	405.4936303	436.9252233	493.8174403	470.1537214	366.9747657	548.0947405
**ENSG00000156858**	PRR14	499.2119106	462.4229373	425.0297406	505.6891263	517.7934488	512.9241305	401.4080522	541.5879194	484.9071541	425.8592928	383.5300183	375.5463962
**ENSG00000156860**	FBRS	1759.194648	1975.807096	1822.253945	1935.684205	1918.609654	2065.733647	1576.011767	1433.712805	1573.368958	1456.021148	1528.601656	1523.331215
**ENSG00000156869**	FRRS1	494.9932184	676.1183856	413.6304755	627.088006	690.0060021	587.6623359	628.1576389	579.79811	607.3063487	637.2069952	684.2837736	669.0477465
**ENSG00000156873**	PHKG2	563.8985244	648.092753	602.5325824	570.9933513	492.366092	480.2972997	470.8628806	536.6039815	423.4730756	468.8881663	489.7595557	430.5250353
**ENSG00000156875**	MFSD14A	14.06230734	21.01922442	19.54159727	21.76807497	21.95999002	18.58970591	15.32091802	14.95181373	15.00679781	16.45221636	8.277626294	18.60815477
**ENSG00000156876**	SASS6	554.0549093	616.5639164	568.3347872	631.2741743	569.8039515	604.355133	685.3557329	599.7338616	629.8165454	734.0219608	698.0798174	751.0927925
**ENSG00000156920**	ADGRG4	0	0	4.071166097	11.72127114	1.155788948	2.27629052	4.08557814	0	0	1.898332657	0	3.383300867
**ENSG00000156928**	MALSU1	386.7134519	357.3268152	349.3060511	473.8742475	316.6861718	474.6065734	331.9532238	436.9252233	346.0942744	313.2248884	336.6234693	391.6170754
**ENSG00000156931**	VPS8	905.6125928	854.7817932	902.1704071	817.1400452	809.0522638	868.7842151	872.2709328	897.1088235	882.1183336	778.3163894	931.2329581	870.3541481
**ENSG00000156958**	GALK2	547.0237556	504.4613861	563.4493879	446.245537	448.4461119	420.7343644	382.001556	413.6668464	350.7838987	372.0732008	324.2070298	320.5677572
**ENSG00000156970**	BUB1B	1115.140972	567.5190594	934.7397359	1513.718444	1407.750939	1290.656725	947.8541284	724.3323094	1022.338101	1082.682392	987.7967377	1278.887728
**ENSG00000156973**	PDE6D	213.7470716	280.2563256	217.4002696	248.6583949	269.2988249	234.4579235	218.5784305	182.74439	222.7571549	167.0532738	168.3117346	201.3064016
**ENSG00000156976**	EIF4A2	3297.611072	3110.845215	3654.278689	3285.304854	3033.945989	3537.73485	3258.248566	2792.666541	3593.659112	3210.080523	2629.525953	3505.945524
**ENSG00000156983**	BRPF1	255.9339936	304.7787541	337.9067861	317.3115544	257.7409355	378.243608	413.6647866	353.8595915	423.942038	393.5876376	440.093798	379.7755223
**ENSG00000156990**	RPUSD3	343.1202991	283.7595297	256.4834641	382.6157794	365.2293077	363.4477197	456.5633571	403.6989706	451.6108215	551.7820257	491.1391601	512.5700814
**ENSG00000157014**	TATDN2	179.997534	182.1666117	201.1156052	248.6583949	201.107277	209.7981096	213.4714578	230.9224564	279.9705716	239.8226924	230.3939318	263.0516424
**ENSG00000157017**	GHRL	4.218692202	0	0.814233219	0.837233653	3.467366845	4.931962793	9.192550814	0	2.813774589	3.163887762	1.379604382	4.229126084
**ENSG00000157020**	SEC13	757.9583657	788.2209158	817.4901523	625.4135387	774.3785953	844.8831646	702.71944	774.1716884	863.3598363	878.9280203	804.3093549	929.5619132
**ENSG00000157021**	FAM92A1P1	0	0	0	0	0	1.896908767	6.128367209	0	0	1.265555105	2.759208765	0
**ENSG00000157036**	EXOG	89.99876699	77.07048955	127.0203822	97.95633739	114.4231059	123.6784516	187.9365944	234.2450817	174.4540245	211.98048	245.5695801	164.9359173
**ENSG00000157045**	NTAN1	181.4037647	231.2114686	150.6331456	158.2371604	175.6799201	168.8248802	73.54040651	96.3561329	115.3647581	112.6344043	157.2748996	110.8031034
**ENSG00000157064**	NMNAT2	2394.81094	1460.836097	1396.409971	1539.672688	1129.205802	1270.928874	634.2860062	614.6856754	653.7336294	480.2781623	469.06549	544.7114396
**ENSG00000157077**	ZFYVE9	383.9009904	455.4165292	423.4012741	617.0412022	581.361841	541.377762	695.5696783	747.5906863	511.1690503	651.1281014	696.7002131	657.2061934
**ENSG00000157103**	SLC6A1	0	0	2.442699658	0	4.623155793	1.13814526	0	0	3.751699452	0	0	0.845825217
**ENSG00000157106**	SMG1	10567.82397	10604.19872	10456.383	6501.956548	6455.081276	6319.741247	7681.908297	9233.575632	7344.889601	5768.400167	6809.727231	6127.15787
**ENSG00000157107**	FCHO2	1957.473182	1965.297484	1791.313083	1567.301398	1233.226808	1487.176473	883.5062727	908.738012	882.1183336	848.5546977	1087.128253	953.2450193
**ENSG00000157110**	RBPMS	0	0	5.699632536	0	0	0.379381753	4.08557814	6.645250545	7.503398903	3.163887762	6.898021912	9.304077385
**ENSG00000157111**	TMEM171	16.87476881	24.52242849	16.28466439	12.55850479	11.55788948	11.00207085	0	0	0.937924863	0	0	0
**ENSG00000157150**	TIMP4	5.624922937	0	4.885399317	6.697869223	18.49262317	12.14021611	2.04278907	3.322625272	2.813774589	8.858885733	9.657230676	4.229126084
**ENSG00000157152**	SYN2	0	0	0	0.837233653	1.155788948	2.27629052	0	0	3.28273702	1.898332657	0	0.845825217
**ENSG00000157168**	NRG1	5.624922937	0	18.72736405	4.186168264	11.55788948	6.828871559	0	0	0	0	0	0.845825217
**ENSG00000157181**	ODR4	974.5178988	994.909956	1002.321093	853.9783259	834.4796206	871.8192691	772.1742684	857.2373202	757.3743268	723.8975199	693.9410043	813.6838585
**ENSG00000157184**	CPT2	457.0249886	381.8492437	384.3180796	401.0349197	417.2398103	441.9797426	618.9650881	443.5704738	657.0163665	586.5847911	408.3628972	605.6108552
**ENSG00000157191**	NECAP2	1070.141589	1198.095792	1268.575356	727.5560443	768.5996506	863.0934888	711.9119908	837.3015686	761.1260262	546.7198053	710.4962569	720.6430847
**ENSG00000157193**	LRP8	652.4910606	623.5703245	543.0935574	631.2741743	503.9239814	435.9096345	180.7868327	116.2918845	176.7988367	218.3082556	189.0058004	180.1607712
**ENSG00000157212**	PAXIP1	714.365213	833.7625688	664.4143071	816.3028115	701.5638916	616.1159674	906.998347	1031.675147	856.7943623	902.3407897	987.7967377	894.8830793
**ENSG00000157214**	STEAP2	39.37446056	70.06408141	79.79485551	79.53719702	121.3578396	150.2351743	12.25673442	0	5.627549177	5.062220419	2.759208765	4.229126084
**ENSG00000157216**	SSBP3	149.0604578	248.727489	241.013033	229.4020209	265.8314581	257.2208287	708.8478072	797.4300653	798.1740583	744.7791792	798.7909374	866.125022
**ENSG00000157224**	CLDN12	732.6462125	802.2337321	717.3394663	629.599707	656.4881226	722.72224	557.681416	518.3295425	556.6584061	561.273689	553.2213573	581.9277491
**ENSG00000157227**	MMP14	606.0854464	725.1632426	750.7230283	732.5794463	782.469118	918.4832248	1619.931732	1172.886721	1917.11842	2043.238717	1643.108819	2221.137019
**ENSG00000157240**	FZD1	74.53022891	122.6121425	52.11092604	77.02549606	101.7094274	75.49696891	189.9793835	272.4552723	221.8192301	220.8393658	291.0965247	236.8310607
**ENSG00000157259**	GATAD1	500.6181414	574.5254675	555.3070557	413.5934245	387.1892977	482.952972	840.6077022	747.5906863	765.8156506	754.90362	676.0061473	738.4054142
**ENSG00000157303**	SUSD3	1.406230734	0	5.699632536	0	1.155788948	0.758763507	1.021394535	3.322625272	8.910286198	5.062220419	8.277626294	9.304077385
**ENSG00000157306**	ZFHX2-AS1	15.46853808	7.006408141	17.09889761	13.39573845	19.64841212	11.00207085	20.4278907	18.274439	39.86180667	34.16998783	24.83287888	23.68310607

**ENSG00000157315**	TMED6	2.812461468	7.006408141	4.071166097	0.837233653	4.623155793	0.758763507	0	0	0.937924863	0	1.379604382	1.691650434
**ENSG00000157326**	DHRS4	54.84299863	66.56087734	55.36785892	51.90848648	57.78944741	67.15057033	86.81853546	111.3079466	73.62710174	74.66775118	103.4703287	87.11999733
**ENSG00000157330**	C1orf158	316.4019152	133.1217547	125.3919158	180.0052354	102.8652164	74.35882365	16.34231256	34.88756536	30.01359561	12.0227735	17.93485697	15.2248539
**ENSG00000157349**	DDX19B	293.9022234	325.7979785	250.7838316	268.7520026	239.2483123	264.0497003	304.3755714	222.6158932	276.2188721	277.1565679	235.9123494	241.0601868
**ENSG00000157350**	ST3GAL2	157.4978422	108.5993262	169.3605096	222.7041517	186.0820207	193.8640759	69.45482837	34.88756536	48.30313044	70.23830831	51.04536215	56.67028952
**ENSG00000157353**	FUK	106.8735358	80.57369362	128.6488487	159.9116277	116.7346838	138.0949582	140.9524458	144.5341993	141.1576919	155.0305003	166.9321303	123.4904816
**ENSG00000157379**	DHRS1	164.5289959	133.1217547	142.4908134	110.5148422	116.7346838	125.1959786	114.3961879	167.7925762	153.8196775	139.8438391	169.691339	142.9444616
**ENSG00000157388**	CACNA1D	0	3.50320407	1.628466439	1.674467306	4.623155793	2.27629052	5.106972674	21.59706427	2.813774589	3.796665314	13.79604382	5.920776517
**ENSG00000157404**	KIT	8.437384405	0	1.628466439	8.372336529	28.89472371	4.173199286	0	0	0	0	0	1.691650434
**ENSG00000157423**	HYDIN	130.7794583	108.5993262	89.56565414	193.4009738	131.7599401	117.9877253	12.25673442	4.983937908	9.379248629	14.5538837	5.518417529	11.84155303
**ENSG00000157426**	AASDH	237.6529941	231.2114686	181.5740079	141.4924873	129.4483622	133.5423772	324.8034621	299.0362745	258.3982997	290.4448965	260.7452283	319.7219319
**ENSG00000157429**	ZNF19	82.96761332	52.54806106	56.18209214	103.816973	55.47786952	66.39180683	70.47622291	39.87150327	60.96511609	53.1533144	30.35129641	60.05359039
**ENSG00000157445**	CACNA2D3	535.7739097	199.682632	276.0250614	504.8518927	308.5956492	377.1054628	623.0506663	451.877037	634.9751322	608.0992278	627.719994	605.6108552
**ENSG00000157450**	RNF111	701.7091363	627.0735286	815.8616859	714.9975395	769.7554395	759.5222701	379.958767	465.1675381	415.9696767	364.4798702	437.3345892	433.9083362
**ENSG00000157456**	CCNB2	458.4312193	392.3588559	435.6147724	725.881577	695.7849469	676.0582844	537.2535253	498.3937908	582.4513399	577.7259053	499.4167864	699.4974543
**ENSG00000157470**	FAM81A	198.2785335	238.2178768	137.6054141	268.7520026	245.027257	211.6950183	19.40649616	14.95181373	51.11690503	46.82553888	45.52694462	63.43689126
**ENSG00000157483**	MYO1E	4300.253585	5797.802737	4571.919527	4094.072563	4394.309581	4629.974917	1532.091802	1842.395713	1450.969763	1465.512811	1535.499678	1480.194129
**ENSG00000157500**	APPL1	1013.892359	938.8586909	954.2813332	1291.851526	1354.584647	1179.497871	1672.022854	1870.638028	1604.78944	1893.270437	1942.48297	1907.335864
**ENSG00000157510**	AFAP1L1	715.7714437	557.0094472	608.2322149	1001.331449	902.6711686	833.8810938	323.7820676	586.4433606	314.2048291	373.9715335	609.785137	384.0046484
**ENSG00000157514**	TSC22D3	4.218692202	52.54806106	26.86969624	5.86063557	6.93473369	22.7629052	3.064183605	19.93575163	3.28273702	6.327775524	9.657230676	6.766601734
**ENSG00000157538**	VPS26C	957.64313	1022.935589	1106.542945	887.467672	884.1785454	896.8584648	736.4254596	885.4796351	792.5465092	756.1691751	793.2725198	761.2426951
**ENSG00000157540**	DYRK1A	2349.811557	2357.656339	2448.399291	2074.664992	2095.445363	2289.948263	2317.5442	2385.644945	2369.667166	2288.123629	2428.103713	2381.84381
**ENSG00000157542**	KCNJ6	7.031153671	7.006408141	4.885399317	2.511700959	5.778944741	1.896908767	4.08557814	3.322625272	0.937924863	2.53111021	1.379604382	0.845825217
**ENSG00000157551**	KCNJ15	14.06230734	10.50961221	35.01202844	22.60530863	42.76419109	21.62475994	145.038024	169.4538889	174.9229869	112.0016268	160.0341083	193.6939746
**ENSG00000157554**	ERG	5.624922937	14.01281628	7.328098975	0.837233653	0	1.13814526	0	1.661312636	0.937924863	1.898332657	4.138813147	7.612426951
**ENSG00000157557**	ETS2	1953.25449	1769.118056	1997.314087	1465.996126	1603.079271	1454.929024	1677.129826	2192.93268	1742.195433	2088.7987	2632.285161	1874.34868
**ENSG00000157570**	TSPAN18	0	0	4.071166097	1.674467306	3.467366845	8.725780326	2.04278907	6.645250545	1.406887294	0	17.93485697	1.691650434
**ENSG00000157578**	LCA5L	14.06230734	0	5.699632536	28.4659442	26.58314581	17.0721789	32.68462512	23.25837691	26.26189616	43.66165111	26.21248326	36.37048432
**ENSG00000157593**	SLC35B2	458.4312193	472.9325495	500.75343	498.1540235	491.210303	564.5200489	295.1830206	405.3602832	305.7635053	341.0671007	284.1985028	289.2722241
**ENSG00000157600**	TMEM164	241.8716863	143.6313669	222.2856689	216.8435161	217.2883223	204.8661468	254.3272392	262.4873965	272.9361351	239.1899148	237.2919538	245.2893129
**ENSG00000157601**	MX1	3505.73322	7640.488078	3488.175112	380.1040784	907.2943244	396.0745504	587.3018576	1018.384646	459.1142204	127.188288	139.3400426	49.05786257
**ENSG00000157617**	C2CD2	510.4617565	444.9069169	502.3818964	761.0453905	654.1765447	729.1717299	371.7876107	250.8582081	415.5007143	408.1415213	278.6800852	489.7328005
**ENSG00000157625**	TAB3	3445.265299	3643.332233	3935.18915	3340.562275	3732.042514	3686.452497	3763.838861	3367.480713	3033.717969	3564.435953	3297.254474	3236.127279
**ENSG00000157637**	SLC38A10	909.831285	1036.948405	1016.977291	835.5591856	1018.250063	996.6358659	840.6077022	799.091378	913.069854	909.3013428	971.2414851	866.9708472
**ENSG00000157653**	C9orf43	1.406230734	7.006408141	17.91313083	13.39573845	12.71367843	15.55465189	6.128367209	14.95181373	17.35160996	22.14721433	12.41643944	22.83728085
**ENSG00000157654**	PALM2-AKAP2	9939.238829	7990.808485	8989.134743	5869.007907	5062.355593	5650.132452	299.2685987	412.0055338	657.4853289	260.7043516	416.6405235	605.6108552
**ENSG00000157657**	ZNF618	28.12461468	31.52883663	52.92515926	20.93084132	52.01050267	66.01242507	20.4278907	9.967875817	30.95152048	8.858885733	17.93485697	10.99572782
**ENSG00000157680**	DGKI	0	10.50961221	21.98429693	3.348934611	9.246311586	11.3814526	0	0	1.406887294	3.163887762	1.379604382	0
**ENSG00000157693**	TMEM268	503.4306028	451.9133251	516.2238611	314.7998535	395.2798203	489.02308	255.3486337	181.0830773	238.7018776	192.9971535	150.3768777	183.544072
**ENSG00000157703**	SVOPL	15.46853808	17.51602035	30.94086234	24.27977593	11.55788948	12.14021611	29.62044151	38.21019063	25.3239713	32.90443272	33.11050518	24.52893129
**ENSG00000157734**	SNX22	4.218692202	28.02563256	8.142332194	11.72127114	2.311577897	3.793817533	5.106972674	0	5.627549177	7.593330629	6.898021912	3.383300867
**ENSG00000157741**	UBN2	1418.886811	1856.698157	1544.600417	1604.976913	1864.287574	1585.815729	2146.971312	2430.500387	2017.007418	2221.049209	2521.916811	2239.745174
**ENSG00000157764**	BRAF	1229.045662	1397.778424	1105.728712	1188.034553	1318.75519	1174.186526	1664.873092	1609.811944	1415.797581	1643.323304	1709.32983	1569.005777
**ENSG00000157778**	PSMG3	309.3707615	280.2563256	308.5943902	328.1955919	321.3093276	319.4394363	346.2527473	343.8917157	363.9148468	348.0276538	347.6603043	324.7968832
**ENSG00000157782**	CABP1	8.437384405	0	0	0	0	0	0	9.967875817	0	0	8.277626294	0
**ENSG00000157796**	WDR19	262.9651473	220.7018564	183.2024744	210.9828805	206.8862217	125.5753603	410.600603	526.6361057	380.7974943	411.9381866	438.7141936	422.9126084
**ENSG00000157800**	SLC37A3	680.6156753	882.8074257	758.8653605	572.6678186	533.9744941	618.7716396	565.8525723	657.8798039	522.8931111	444.2098418	477.3431163	484.6578492
**ENSG00000157823**	AP3S2	229.2156097	210.1922442	262.1830967	255.3562641	236.9367344	237.4929776	189.9793835	142.8728867	162.7299637	187.3021555	147.6176689	184.3898973
**ENSG00000157827**	FMNL2	2829.336237	2956.704235	3160.039125	2398.674415	2178.662167	2647.325875	2161.270836	2139.770675	2263.681657	1780.636032	1731.4035	2012.218191
**ENSG00000157833**	GAREM2	0	0	3.256932878	2.511700959	3.467366845	2.27629052	7.149761744	1.661312636	3.751699452	7.593330629	4.138813147	7.612426951
**ENSG00000157837**	SPPL3	593.4293698	595.544692	539.8366245	611.1805666	698.0965248	627.8768017	1004.030828	898.7701361	913.069854	1097.869053	1063.674979	1036.981716
**ENSG00000157856**	DRC1	0	0	1.628466439	0	0	0.758763507	0	0	0	3.163887762	0	0.845825217
**ENSG00000157869**	RAB28	192.6536106	133.1217547	153.0758453	235.2626565	135.2273069	178.3094241	256.3700283	309.0041503	294.9773694	293.6087843	264.8840414	321.4135824
**ENSG00000157870**	FAM213B	125.1545353	206.6890402	136.7911809	97.95633739	219.5999002	128.9897961	41.87717593	76.42038126	44.08246856	46.19276132	85.5354717	39.75378519
**ENSG00000157873**	TNFRSF14	331.8704533	462.4229373	372.9188145	36.00104707	113.2673169	52.35468196	146.0594185	134.5663235	112.0820211	18.98332657	38.6289227	8.458252168
**ENSG00000157881**	PANK4	330.4642225	287.2627338	307.780157	385.1274803	424.174544	365.3446284	313.5681222	333.9238399	266.3706611	334.7393252	311.7905904	301.1137772
**ENSG00000157895**	C12orf43	393.7446056	276.7531216	440.5001717	318.9860217	336.3345839	396.4539322	458.6061462	333.9238399	416.9076016	374.604311	361.4563482	330.7176598
**ENSG00000157911**	PEX10	367.0262216	329.3011826	341.1637189	341.5913304	360.6061519	364.2064832	273.7337353	282.4231481	339.0598379	321.4509966	293.8557334	281.6597972
**ENSG00000157916**	RER1	2085.440179	2340.140319	1917.519232	2044.52458	2108.159042	2059.663539	1743.520471	2018.494853	1701.395701	1705.335504	1796.244906	1803.299362
**ENSG00000157927**	RADIL	0	0	3.256932878	0	0	0	0	0	1.875849726	0	0	1.691650434
**ENSG00000157933**	SKI	674.9907524	693.6344059	666.8570067	818.8145125	846.0375101	841.4687288	609.7725373	646.2506155	658.4232538	630.8792197	707.7370481	691.8850273
**ENSG00000157954**	WIPI2	800.1452877	1061.470833	841.1029157	867.3740644	920.0080028	899.8935188	858.9928038	847.2694444	798.1740583	940.3074428	924.3349361	859.3584202
**ENSG00000157965**	SSX8	0	28.02563256	1.628466439	4.186168264	8.090522638	8.346398573	3.064183605	8.306563181	2.344812157	1.898332657	11.03683506	2.53747565
**ENSG00000157978**	LDLRAP1	506.2430643	490.4485699	510.5242286	609.5060993	657.6439116	636.2232003	570.959545	491.7485403	649.98193	612.5286707	524.2496653	630.1397865
**ENSG00000157985**	AGAP1	1158.734125	1152.554139	1249.033759	1814.285326	1940.569644	1922.327344	635.3074007	838.9628812	593.2374758	747.3102894	1027.805265	713.876483
**ENSG00000157992**	KRTCAP3	134.9981505	178.6634076	147.3762127	131.4456835	149.0967743	139.991867	70.47622291	48.17806645	44.55143099	68.97275321	68.98021912	81.19922081
**ENSG00000157999**	ANKRD61	0	0	4.885399317	7.535102876	0	3.035054026	0	8.306563181	2.344812157	0	2.759208765	0

**ENSG00000158006**	PAFAH2	350.1514528	227.7082646	416.8874084	488.9444533	409.1492877	471.5715194	264.5411845	215.9706427	304.356618	282.2187884	271.7820633	270.6640694
**ENSG00000158008**	EXTL1	0	3.50320407	3.256932878	1.674467306	3.467366845	3.035054026	2.04278907	1.661312636	0.937924863	0	0	0.845825217
**ENSG00000158014**	SLC30A2	0	0	4.071166097	5.023401917	0	10.24330734	4.08557814	1.661312636	0.468962431	15.81943881	8.277626294	3.383300867
**ENSG00000158019**	BABAM2	376.8698368	325.7979785	317.5509556	429.5008639	457.6924235	422.2518914	526.0181855	618.0083006	496.6312149	549.2509155	724.2923007	546.40309
**ENSG00000158023**	WDR66	298.1209156	434.3973047	251.5980648	204.2850113	331.7114282	204.1073833	124.6101333	282.4231481	84.41323766	64.54331034	195.9038223	82.04504603
**ENSG00000158042**	MRPL17	690.4592905	739.1760589	620.4457132	637.9720435	633.3723436	634.7056733	458.6061462	574.8141721	516.7965995	477.1142745	451.130633	537.9448379
**ENSG00000158050**	DUSP2	8.437384405	21.01922442	9.770798633	8.372336529	2.311577897	5.6907263	21.44928523	38.21019063	22.51019671	25.94387965	26.21248326	16.07067912
**ENSG00000158055**	GRHL3	867.644363	872.2978135	1125.270309	835.5591856	403.3703429	1090.343159	32.68462512	53.16200436	98.48211061	49.98942664	44.14734023	83.73669646
**ENSG00000158062**	UBXN11	247.4966092	217.1986524	232.0564675	289.6828439	232.3135786	270.4991901	292.118837	307.3428377	316.5496412	358.1520946	271.7820633	290.1180494
**ENSG00000158077**	NLRP14	4.218692202	3.50320407	2.442699658	3.348934611	1.155788948	2.655672273	11.23533988	1.661312636	0.937924863	0.632777552	5.518417529	0
**ENSG00000158079**	PTPDC1	365.6199909	525.4806106	420.9585745	458.8040418	461.1597904	411.2498206	379.958767	360.504842	289.3498202	352.4570967	361.4563482	315.4928059
**ENSG00000158089**	GALNT14	174.372611	329.3011826	466.5556347	172.4701325	382.5661419	438.9446886	3.064183605	0	4.220661883	4.429442867	8.277626294	1.691650434
**ENSG00000158092**	NCK1	414.8380666	434.3973047	423.4012741	518.2476311	406.8377098	458.6725397	606.7083537	510.0229793	629.8165454	540.3920297	532.5272916	658.8978439
**ENSG00000158104**	HPD	0	0	1.628466439	0.837233653	0	0	0	1.661312636	0.468962431	0.632777552	0	5.074951301
**ENSG00000158106**	RHPN1	74.53022891	168.1537954	78.98062229	82.88613163	69.3473369	74.7382054	65.36925023	88.04956971	61.43407852	110.1032941	106.2295374	46.52038692
**ENSG00000158109**	TPRG1L	504.8368336	497.454978	518.6665608	449.5944716	568.6481625	545.5509613	498.440533	491.7485403	540.7136835	541.6575848	573.915423	581.0819239
**ENSG00000158122**	AAED1	92.81122845	171.6569995	67.58135721	79.53719702	124.8252064	111.5382355	151.1663912	137.8889488	127.0888189	155.6632779	182.1077785	149.7110634
**ENSG00000158125**	XDH	5003.368952	3814.989233	4824.331825	2623.053034	2155.546389	2484.950484	1407.481669	980.1744553	1474.886847	628.3481095	474.5839075	721.4889099
**ENSG00000158156**	XKR8	77.34269038	73.56728548	121.3207497	140.6552537	177.991498	167.3073532	96.01108628	98.01744553	124.2750443	99.97885328	111.747955	126.0279573
**ENSG00000158158**	CNNM4	171.5601496	115.6057343	157.9612446	118.8871787	151.4083522	184.3795321	156.2733638	177.7604521	165.0747759	162.623831	217.9774924	155.6318399
**ENSG00000158161**	EYA3	960.4555914	1068.477241	1091.886747	1048.216533	1110.713179	1102.103993	955.0038901	1088.159777	1053.758583	1001.686865	1158.867681	1152.013945
**ENSG00000158163**	DZIP1L	0	10.50961221	1.628466439	1.674467306	2.311577897	4.55258104	0	0	0.937924863	1.898332657	0	0
**ENSG00000158164**	TMSB15A	1.406230734	0	0.814233219	0	0	0.758763507	9.192550814	8.306563181	6.096511609	5.062220419	5.518417529	4.229126084
**ENSG00000158169**	FANCC	712.9589822	665.6087734	650.5723423	783.6506991	777.8459622	785.3202293	830.3937569	815.7045043	787.8568848	942.2057755	983.6579246	828.9087124
**ENSG00000158186**	MRAS	22.49969175	17.51602035	37.45472809	8.372336529	12.71367843	9.484543833	206.321696	154.5020752	169.2954378	229.065474	93.813098	142.9444616
**ENSG00000158195**	WASF2	6735.845217	7812.145077	6876.199538	6172.086489	8140.221563	6821.283924	4529.884762	7208.435528	4605.211077	4340.854009	6855.254176	4297.637926
**ENSG00000158201**	ABHD3	284.0586083	613.0607123	519.480794	338.2423958	358.294574	432.8745805	247.1774774	287.407086	262.1499992	239.1899148	206.9406573	285.8889233
**ENSG00000158220**	ESYT3	7.031153671	38.53524477	27.68392946	10.04680383	20.80420107	15.55465189	85.79714093	18.274439	46.89624315	75.93330629	51.04536215	48.21203736
**ENSG00000158234**	FAIM	104.0610743	133.1217547	76.53792263	138.1435527	109.7999501	114.1939077	249.2202665	177.7604521	187.1160101	261.3371291	197.2834267	202.1522268
**ENSG00000158246**	FAM46B	88.59253625	91.08330583	70.83829009	244.4722266	132.9157291	155.5465189	160.358942	292.391024	130.3715559	220.8393658	256.6064151	188.6190233
**ENSG00000158286**	RNF207	133.5919197	227.7082646	192.1590398	125.5850479	146.7851964	150.6145561	284.9690752	141.2115741	144.9093913	251.2126883	175.2097566	109.111453
**ENSG00000158290**	CUL4B	1574.978422	1485.358526	1376.054141	1508.695042	1829.613905	1447.720771	1376.839833	1579.908317	1360.928976	1423.749493	1670.700907	1555.472574
**ENSG00000158292**	GPR153	50.62430643	45.54165292	62.6959579	44.3733836	50.85471372	80.04954995	8.171156279	4.983937908	31.42048291	29.10776741	4.138813147	24.52893129
**ENSG00000158296**	SLC13A3	4.218692202	0	5.699632536	5.86063557	5.778944741	5.6907263	21.44928523	11.62918845	18.28953483	15.81943881	9.657230676	8.458252168
**ENSG00000158301**	GPRASP2	22.49969175	73.56728548	52.92515926	46.88508456	77.43785953	56.52788124	80.69016826	79.74300653	92.385599	105.6738512	133.8216251	107.4198025
**ENSG00000158315**	RHBDL2	36.56199909	140.1281628	41.52589419	58.6063557	54.32208057	41.35261111	5.106972674	4.983937908	4.220661883	6.960553076	4.138813147	0
**ENSG00000158321**	AUTS2	0	7.006408141	0	0	1.155788948	0	39.83438686	61.46856754	30.48255804	52.52053685	57.94338406	17.76232955
**ENSG00000158352**	SHROOM4	8.437384405	17.51602035	8.142332194	2.511700959	10.40210053	5.6907263	13.27812895	8.306563181	4.689624315	20.88165923	6.898021912	10.99572782
**ENSG00000158373**	HIST1H2BD	1650.914882	1075.48365	1818.182779	2319.137218	1412.374095	2263.39154	2743.465721	2247.755997	3232.089078	3020.247258	2021.12042	3661.577363
**ENSG00000158402**	CDC25C	338.9016069	287.2627338	295.5666587	457.9668081	391.8124535	403.6621855	486.1837986	363.8274673	461.4590325	549.2509155	401.4648753	550.6322161
**ENSG00000158406**	HIST1H4H	866.2381322	346.817203	794.6916222	1370.55149	705.0312584	1130.937007	2152.078285	1491.858747	2683.871995	2358.361938	1583.785831	2848.73933
**ENSG00000158411**	MITD1	645.459907	767.2016914	656.2719749	366.70834	425.330333	364.2064832	479.0340369	564.8462963	443.1694977	390.4237498	557.3601705	376.3922215
**ENSG00000158417**	EIF5B	3037.458386	2792.053644	2872.614798	3470.333491	3073.242813	2842.707478	2774.107557	2432.161699	2390.770476	2899.386745	2486.047097	3009.446121
**ENSG00000158423**	RIBC1	5.624922937	17.51602035	13.02773151	10.88403749	21.95999002	10.24330734	39.83438686	44.85544118	22.51019671	24.04554699	44.14734023	24.52893129
**ENSG00000158427**	TMSB15B	1.406230734	3.50320407	6.513865756	2.511700959	5.778944741	0.758763507	5.106972674	1.661312636	0.468962431	0.632777552	0	0.845825217
**ENSG00000158428**	CATIP	0	0	0	0	0	0	1.021394535	0	2.344812157	1.265555105	0	3.383300867
**ENSG00000158435**	CNOT11	1094.047511	1177.076568	986.850662	1223.198367	1125.738436	1152.561767	1140.897695	1284.194668	1188.350801	1168.107362	1244.403153	1322.870639
**ENSG00000158445**	KCNB1	0	0	1.628466439	9.209570182	4.623155793	3.035054026	0	3.322625272	2.344812157	1.898332657	20.69406573	3.383300867
**ENSG00000158457**	TSPAN33	22.49969175	3.50320407	10.58503185	11.72127114	17.33683422	17.0721789	1.021394535	3.322625272	2.813774589	2.53111021	1.379604382	0
**ENSG00000158467**	AHCYL2	338.9016069	444.9069169	372.9188145	535.8295378	529.3513383	607.390187	454.520568	438.5865359	390.176743	502.4253766	426.2977541	432.2166858
**ENSG00000158470**	B4GALT5	2050.28441	2606.383828	2327.892774	1826.843831	1952.127534	2362.410178	3364.473598	4701.51476	3294.461081	3315.121597	4639.609538	3352.005334
**ENSG00000158473**	CD1D	0	0	0	0	0	0	5.106972674	3.322625272	0.468962431	0	1.379604382	0.845825217
**ENSG00000158480**	SPATA2	199.6847643	217.1986524	181.5740079	231.0764882	197.6399102	217.7651264	178.7440436	234.2450817	252.7707506	211.3477025	219.3570968	247.8267885
**ENSG00000158482**	SNX29P1	33.74953762	17.51602035	21.17006371	7.535102876	4.623155793	12.51959786	4.08557814	3.322625272	1.406887294	6.960553076	2.759208765	0
**ENSG00000158483**	FAM86C1	74.53022891	52.54806106	74.09522297	111.3520758	56.63365846	98.63925586	98.05387535	43.19412854	109.737209	160.0927208	52.42496653	122.6446564
**ENSG00000158486**	DNAH3	53.4367679	38.53524477	60.25325824	30.97764516	45.07576898	56.14849949	5.106972674	4.983937908	8.910286198	7.593330629	1.379604382	6.766601734
**ENSG00000158516**	CPA2	8.437384405	24.52242849	21.98429693	0	3.467366845	1.13814526	7.149761744	6.645250545	5.627549177	0	0	1.691650434
**ENSG00000158526**	TSR2	627.1789074	497.454978	645.686943	610.3433329	626.43761	777.3532125	743.5752214	820.6884422	800.5188705	718.8352995	786.3744979	787.4632768
**ENSG00000158528**	PPP1R9A	8.437384405	7.006408141	4.071166097	12.55850479	9.246311586	18.96908767	30.64183605	11.62918845	6.56547404	12.65555105	5.518417529	7.612426951
**ENSG00000158545**	ZC3H18	1099.672434	914.3362624	1117.127977	1087.566515	1064.481621	1135.489588	908.0197415	940.302952	1020.931213	975.1102082	1027.805265	1016.681911
**ENSG00000158552**	ZFAND2B	89.99876699	112.1025303	91.19412058	115.5382441	92.46311586	126.3341239	81.71156279	88.04956971	86.28908739	101.2444084	81.39665856	71.89514343
**ENSG00000158553**	POM121L2	11.24984587	35.0320407	21.98429693	11.72127114	10.40210053	14.79588838	0	0	1.875849726	0	0	0
**ENSG00000158555**	GDPD5	25.31215321	52.54806106	16.28466439	61.95529031	88.99574902	62.5979893	96.01108628	88.04956971	76.90983876	151.233835	142.0992514	69.35766778
**ENSG00000158560**	DYNC1I1	1.406230734	0	9.770798633	2.511700959	5.778944741	7.208253313	0	0	1.875849726	3.163887762	6.898021912	0.845825217
**ENSG00000158571**	PFKFB1	7.031153671	3.50320407	11.39926507	8.372336529	6.93473369	3.793817533	15.32091802	6.645250545	10.31717349	11.38999594	8.277626294	17.76232955
**ENSG00000158604**	TMED4	1146.078048	1198.095792	1081.301715	1218.174965	1233.226808	1470.104294	1016.287562	1036.659085	1126.44776	1085.84628	1125.757176	1303.416659
**ENSG00000158615**	PPP1R15B	2726.681394	2886.640154	2923.911491	2882.595467	2523.087274	3188.703637	3099.932413	2990.362745	2995.732012	2812.69622	2902.68762	2992.529617

ENSG00000158623	COPG2	594.8356005	525.4806106	471.4410341	568.4816503	461.1597904	467.0189383	946.8327338	898.7701361	897.5940938	852.9841406	831.9014425	852.5918185
ENSG00000158636	EMSY	776.2393653	987.9035479	918.4550715	879.9325692	918.8522139	894.2027925	1022.415929	1294.162544	1122.227098	1023.83408	1149.21045	1168.084624
ENSG00000158639	PAGE5	956.2368992	1296.185506	902.1704071	1127.75373	1175.43736	930.6234409	974.4103863	1307.453045	827.7186915	983.969094	976.7599027	1007.377833
ENSG00000158669	GPAT4	721.3963666	854.7817932	920.8977712	845.6059894	969.7069276	961.3533629	347.2741419	380.4405937	318.8944534	330.9426599	360.0767438	351.8632902
ENSG00000158683	PKD1L1	7.031153671	3.50320407	2.442699658	8.372336529	20.80420107	5.311344546	6.128367209	11.62918845	4.689624315	1.265555105	5.518417529	0.845825217
ENSG00000158691	ZSCAN12	130.7794583	119.1089384	165.2893435	229.4020209	172.2125533	222.3177074	201.2147234	121.2758224	188.9918599	212.6132576	126.9236032	215.6854303
ENSG00000158710	TAGLN2	1934.97349	2277.082646	2255.426018	2923.619916	3588.724684	3694.419514	4777.06224	5115.181607	4891.27816	5645.641322	5677.072033	5622.200216
ENSG00000158711	ELK4	4374.783814	3769.44758	4069.537631	3950.905608	3743.600403	3752.08554	4740.292036	4812.822707	5027.746228	4109.890203	4283.671607	4887.178103
ENSG00000158714	SLAMF8	1.406230734	0	5.699632536	0	0	0	2.04278907	3.322625272	2.813774589	1.898332657	4.138813147	0
ENSG00000158715	SLC45A3	53.4367679	35.0320407	55.36785892	66.97869223	60.10102531	90.29285729	10.21394535	9.967875817	9.379248629	23.41276944	5.518417529	29.60388259
ENSG00000158716	DUSP23	132.185689	175.1602035	164.4751103	198.4243757	216.1325333	181.7238598	199.1719343	199.3575163	239.6398025	222.1049209	255.2268107	220.7603816
ENSG00000158717	RNF166	66.09284451	35.0320407	55.36785892	70.32762684	46.23155793	66.39180683	78.64737919	98.01744553	96.13729845	80.36274915	78.63744979	104.8823269
ENSG00000158731	OR10J6P	0	3.50320407	0	3.348934611	0	0.758763507	0	0	0.937924863	0	5.518417529	0
ENSG00000158747	NBL1	219.3719945	311.7851623	336.2783196	384.2902467	411.4608656	416.5611651	17.36370709	14.95181373	27.66878346	13.92110615	15.17564821	20.2998052
ENSG00000158764	ITLN2	0	0	0	0	1.155788948	0.379381753	40.8557814	61.46856754	19.69642212	45.55998377	108.9887462	42.29126084
ENSG00000158769	F11R	1960.285643	2136.954483	2103.164406	2084.711796	1957.906478	2167.787338	3500.319071	3556.870354	3443.591134	3665.680361	4033.963214	3442.508632
ENSG00000158773	USF1	812.8013643	833.7625688	964.0521318	491.4561542	488.8987251	548.965397	731.318487	809.0592538	781.2914108	539.1264746	543.5641266	552.3238666
ENSG00000158786	PLA2G2F	14.06230734	17.51602035	15.47043117	21.76807497	16.18104528	12.51959786	2.04278907	0	3.28273702	5.062220419	0	3.383300867
ENSG00000158792	SPATA2L	5.624922937	24.52242849	36.64049488	31.81487881	42.76419109	46.66395566	37.79159779	34.88756536	60.96511609	34.16998783	26.21248326	37.21630954
ENSG00000158793	NIT1	338.9016069	385.3524477	407.1166097	349.9636669	331.7114282	374.8291723	364.637849	345.5530283	351.7218236	323.9821068	391.8076446	309.5720293
ENSG00000158796	DEDD	438.7439891	381.8492437	385.946546	428.6636303	448.4461119	435.9096345	325.8248566	387.0858442	326.3978523	325.8804395	372.4931832	333.2551354
ENSG00000158805	ZNF276	188.4349184	182.1666117	210.8864038	177.4935344	132.9157291	159.7197181	145.038024	161.1473257	166.0127007	171.4827167	190.3854048	139.5611608
ENSG00000158806	NPM2	12.65607661	0	4.885399317	1.674467306	5.778944741	6.449489806	0	0	5.158586746	0.632777552	0	0
ENSG00000158813	EDA	1.406230734	0	0	9.209570182	10.40210053	1.13814526	3.064183605	0	3.28273702	2.53111021	5.518417529	1.691650434
ENSG00000158816	VWA5B1	0	0	0	0	0	0	0	0	2.344812157	5.062220419	2.759208765	2.53747565
ENSG00000158825	CDA	2.812461468	14.01281628	21.98429693	12.55850479	50.85471372	30.72992202	35.74880872	81.40431917	43.61350613	48.72387153	75.87824103	58.36193996
ENSG00000158828	PINK1	1168.57774	1453.829689	1234.377561	1425.808911	2028.409604	1970.129445	1744.541866	2204.561868	1844.429243	1931.23709	2000.426354	2002.914113
ENSG00000158850	B4GALT3	314.9956845	231.2114686	390.8319453	368.3828073	331.7114282	431.7364353	252.2844501	272.4552723	327.8047396	328.4115497	249.7083932	321.4135824
ENSG00000158856	DMTN	25.31215321	66.56087734	33.383562	36.83828073	61.25681426	36.04126656	4.08557814	1.661312636	0	0	0	1.691650434
ENSG00000158859	ADAMTS4	5.624922937	0	0	0	2.311577897	3.793817533	1.021394535	3.322625272	0	3.163887762	4.138813147	5.920776517
ENSG00000158863	FAM160B2	220.7782253	217.1986524	289.0527929	263.7286007	277.3893476	286.4332237	118.481766	134.5663235	156.1644897	151.233835	160.0341083	130.2570834
ENSG00000158864	NDUFS2	1210.764662	1313.701526	1419.208501	1314.456835	1152.321581	1289.897961	1147.026063	1054.933524	1112.378887	1243.40789	1138.173615	1109.722684
ENSG00000158865	SLC5A11	1.406230734	0	8.956565414	1.674467306	2.311577897	1.517527013	3.064183605	0	0.937924863	0.632777552	8.277626294	3.383300867
ENSG00000158869	FCER1G	4.218692202	0	4.071166097	0.837233653	8.090522638	0.758763507	0	0	0.937924863	0.632777552	0	0
ENSG00000158882	TOMM40L	164.5289959	147.134571	166.1035768	201.7733103	166.4336086	183.2413868	155.2519693	141.2115741	153.8196775	180.3416024	194.5242179	158.1693155
ENSG00000158887	MPZ	9.843615139	0	5.699632536	0	11.55788948	2.655672273	0	1.661312636	0.937924863	0	0	0
ENSG00000158941	CCAR2	1375.293658	1233.127833	1417.580035	1588.232239	1511.771944	1589.988928	761.960323	744.268061	823.0290672	778.949167	917.4369142	886.4248272
ENSG00000158966	CACHD1	7.031153671	0	3.256932878	6.697869223	10.40210053	11.3814526	0	0	4.689624315	0.632777552	0	5.074951301
ENSG00000158985	CDC42SE2	1434.355349	1341.727159	1100.029079	1187.19732	1066.793199	1003.085356	2621.919771	2880.716111	2635.568865	2502.002442	2532.953646	2410.601868
ENSG00000158987	RAPGEF6	167.3414574	206.6890402	222.2856689	213.4945815	231.1577897	209.7981096	240.0277157	245.8742701	233.0743284	234.7604719	333.8642605	238.5227111
ENSG00000159023	EPB41	833.8948254	956.3747112	760.493827	860.6761951	953.5258823	742.829473	1282.871536	1133.015218	1183.661177	1417.421717	1339.595855	1271.275301
ENSG00000159055	MIS18A	243.277917	164.6505913	240.1987997	291.3573112	220.7556891	262.1527915	250.241661	289.0683987	294.508407	280.9532333	311.7905904	390.7712502
ENSG00000159063	ALG8	1342.950351	1142.044527	1369.540275	1522.090781	1227.447863	1441.650663	1091.870758	940.302952	1054.227546	1098.501831	971.2414851	1097.881131
ENSG00000159069	FBXW5	555.46114	553.5062431	542.2793242	514.8986965	544.3765946	629.7737105	409.5792085	377.1179684	442.7005353	372.7059784	389.0484358	405.1502788
ENSG00000159079	C21orf59	147.6542271	126.1153465	143.3050466	162.4233287	134.071518	130.5073231	137.8882622	137.8889488	134.1232554	114.532737	99.33151553	128.5654329
ENSG00000159082	SYNJ1	499.2119106	791.7241199	598.4614163	694.0666982	751.2628164	671.5057033	515.8042401	662.8637418	561.8169929	536.5953664	674.6265429	686.810076
ENSG00000159086	PAXBP1	1089.828819	1015.92918	889.9569089	1153.707974	983.576395	991.7039031	1022.415929	1034.997772	893.8423943	1087.744613	1008.490803	960.8574463
ENSG00000159110	IFNAR2	112.4984587	133.1217547	139.2338805	46.88508456	48.54313583	48.94024618	111.3320043	131.2436983	120.0543825	90.48718999	96.57230676	82.04504603
ENSG00000159111	MRPL10	378.2760675	378.3460396	388.3892457	399.3604524	351.3598403	420.3549827	342.1671692	307.3428377	372.3561706	386.6270845	379.3912051	358.6298919
ENSG00000159128	IFNGR2	646.8661377	718.1568344	587.0621512	586.063557	577.8944741	666.5737406	226.7495867	285.7457734	280.9084964	247.416023	329.7254474	238.5227111
ENSG00000159131	GART	2948.86585	2164.980116	2773.278345	2523.42223	2112.782197	2693.98983	1634.231256	1247.64579	1980.89731	1602.82554	1175.422934	1848.973924
ENSG00000159140	SON	6588.19099	5864.363614	6452.798264	7080.485002	6488.599156	6770.446769	6534.882234	6397.714962	6708.03862	6468.25214	6645.55431	7099.85687
ENSG00000159147	DONSON	615.9290616	893.317038	729.5529646	858.1644942	894.580646	769.0068139	559.7242051	644.5893028	548.2170824	570.1325747	629.0995983	604.76503
ENSG00000159164	SV2A	1.406230734	17.51602035	0	8.372336529	15.02525633	7.967016819	11.23533988	4.983937908	5.158586746	4.429442867	6.898021912	2.53747565
ENSG00000159166	LAD1	1296.544737	886.3106298	1108.171412	1501.997173	1148.854215	1272.446401	282.9262862	182.74439	498.9760271	316.3887762	231.7735362	493.1161014
ENSG00000159167	STC1	7395.367431	16223.33805	8774.177173	7634.73368	12766.84472	10223.20011	735.4040651	2051.721106	405.6525032	725.163075	2004.565167	503.266004
ENSG00000159176	CSRP1	2002.472565	1583.44824	2076.29471	2360.161667	1779.91498	2357.098833	1675.087037	1613.13457	1974.331836	1759.754373	1498.250359	1880.269457
ENSG00000159184	HOXB13	18.28099954	17.51602035	4.071166097	5.86063557	4.623155793	10.62268909	2.04278907	0	0.468962431	0	1.379604382	1.691650434
ENSG00000159199	ATP5MC1	606.0854464	353.8236111	532.5085255	629.599707	581.361841	642.2933083	383.0229506	239.2290196	411.7490148	451.1703948	408.3628972	426.2959093
ENSG00000159200	RCAN1	407.8069129	357.3268152	532.5085255	217.6807497	297.0377597	301.6084939	624.0720608	642.9279902	883.9941833	553.0475808	517.3516434	1070.814724
ENSG00000159202	UBE2Z	2553.715013	2672.944706	2794.448409	1484.415267	1818.056016	1709.873562	1612.781971	1810.830773	1690.609565	1178.231803	1409.955679	1259.433748
ENSG00000159208	CIART	127.9669968	122.6121425	135.9769476	103.816973	173.3683422	103.5712187	34.72741419	84.72694444	41.7376564	12.65555105	111.747955	14.37902869
ENSG00000159210	SNF8	622.9602152	599.047896	520.2950272	545.039108	548.9997504	555.7942686	583.2162794	549.8944826	472.7141309	508.1203746	513.2128302	602.2275543
ENSG00000159214	CCDC24	151.8729193	178.6634076	128.6488487	78.69996337	110.955739	111.9176172	33.70601965	63.12988017	43.14454369	48.72387153	92.43349361	49.90368779
ENSG00000159216	RUNX1	1565.134807	1257.650261	1680.577365	1363.853621	1539.510879	1561.155915	595.4730138	1614.795882	693.5954361	631.5119973	1538.258886	608.9941561
ENSG00000159217	IGF2BP1	2.812461468	3.50320407	0	2.511700959	0	0	0	9.967875817	7.503398903	3.796665314	12.41643944	16.91650434
ENSG00000159228	CBR1	583.5857547	507.9645902	582.9909851	597.7848281	570.9597404	500.7839144	316.6323058	318.9720261	375.1699452	321.4509966	314.5497992	399.2295023

**ENSG00000159231**	CBR3	244.6841477	241.7210809	281.7246939	76.18826241	85.52838217	80.04954995	91.92550814	71.43644335	82.53738794	32.27165517	44.14734023	35.5246591
**ENSG00000159247**	TUBBP5	18.28099954	42.03844885	8.142332194	7.535102876	19.64841212	8.346398573	6.128367209	1.661312636	2.813774589	3.163887762	2.759208765	0
**ENSG00000159251**	ACTC1	0	0	0	0	0	0	0	9.967875817	0.468962431	1.898332657	5.518417529	1.691650434
**ENSG00000159256**	MORC3	1236.076815	1695.55077	1226.235228	753.5102876	822.9217312	803.1511717	786.4737919	1372.244237	752.6847025	721.9991873	1380.983987	806.9172568
**ENSG00000159259**	CHAF1B	603.272985	718.1568344	639.1730773	576.8539868	684.2270574	632.4293828	456.5633571	551.5557952	500.3829144	528.3692562	533.906896	552.3238666
**ENSG00000159261**	CLDN14	14.06230734	66.56087734	109.1072514	11.72127114	38.14103529	78.53202293	0	1.661312636	0	0	0	0.845825217
**ENSG00000159263**	SIM2	0	0	3.256932878	2.511700959	1.155788948	3.035054026	0	0	0.468962431	0	4.138813147	0
**ENSG00000159267**	HLCS	604.6792157	683.1247937	602.5325824	684.857128	683.0712684	663.5386865	700.6766509	579.79811	667.8025024	597.974787	689.8021912	687.6559012
**ENSG00000159307**	SCUBE1	0	3.50320407	7.328098975	4.186168264	2.311577897	2.27629052	0	9.967875817	0.468962431	0	1.379604382	0
**ENSG00000159314**	ARHGAP27	300.9333771	336.3075908	349.3060511	335.7306948	355.9829961	345.2373955	242.0705048	365.4887799	246.6742389	251.2126883	335.2438649	279.1223215
**ENSG00000159322**	ADPGK	542.8050634	525.4806106	544.7220238	452.9434062	421.8629661	489.02308	357.4880872	299.0362745	402.8387286	353.7226518	325.5866342	354.4007658
**ENSG00000159335**	PTMS	731.2399818	893.317038	756.4226609	889.1421393	926.9427365	856.2646172	1005.052222	1254.29104	1207.578261	1212.40179	1320.281394	1374.465977
**ENSG00000159337**	PLA2G4D	39.37446056	52.54806106	24.42699658	20.09360767	25.42735686	16.31341539	0	0	0.468962431	0	0	0
**ENSG00000159339**	PADI4	1.406230734	7.006408141	2.442699658	6.697869223	5.778944741	6.449489806	0	0	0	0	0	0.845825217
**ENSG00000159346**	ADIPOR1	2885.585466	3124.858031	2882.385597	2650.681745	2555.449365	2936.035389	3006.985511	3148.187445	3508.776912	2938.618953	2990.982301	3602.369598
**ENSG00000159348**	CYB5R1	2120.595947	1961.794279	2251.354852	2200.25004	1638.908729	2364.307087	4432.852281	5156.714423	4492.191131	4719.254986	5125.23028	4939.619266
**ENSG00000159352**	PSMD4	2382.154864	2539.822951	2647.88643	2386.115911	2417.91048	2352.16687	1419.738403	1538.375501	1473.948922	1505.377797	1498.250359	1617.217814
**ENSG00000159363**	ATP13A2	320.6206074	385.3524477	359.891083	407.7327889	460.0040014	538.3427079	306.4183605	240.8903322	338.5908755	270.8287924	307.6517773	290.9638746
**ENSG00000159377**	PSMB4	3254.017919	2795.556848	3374.996695	2328.346789	2377.457867	2754.311529	1757.819995	1845.718339	2129.089439	1622.441644	1589.304248	1750.012374
**ENSG00000159388**	BTG2	1139.046895	1292.682302	1243.334126	1412.413172	1269.056265	1465.172331	3165.301664	2621.55134	3000.421636	3247.414399	2646.081205	3022.1335
**ENSG00000159398**	CES5A	0	0	0	0	3.467366845	2.655672273	0	0	0	0	0	0
**ENSG00000159399**	HK2	2913.710081	11648.15353	3151.082559	1971.685253	7209.811459	2018.690309	2407.426919	13981.60715	2308.233088	1930.604312	12492.31768	2209.295466
**ENSG00000159403**	C1R	440.1502198	592.0414879	489.3541649	11.72127114	15.02525633	11.00207085	476.9912478	641.2666775	506.0104635	29.10776741	22.07367012	18.60815477
**ENSG00000159423**	ALDH4A1	70.31153671	59.5544692	92.0083538	186.7031046	109.7999501	141.1300122	183.8510163	164.469951	250.8949008	212.6132576	177.9689653	231.7561094
**ENSG00000159433**	STARD9	274.2149932	294.2691419	218.2145028	267.9147689	332.8672171	191.9671672	43.919965	53.16200436	28.13774589	62.01220013	73.11903226	21.14563042
**ENSG00000159445**	THEM4	219.3719945	178.6634076	234.4991672	313.9626198	263.5198802	267.4641361	410.600603	309.0041503	375.6389076	417.000407	307.6517773	454.2081414
**ENSG00000159450**	TCHH	47.81184496	59.5544692	84.68025482	76.18826241	87.83996007	83.08460397	22.47067977	14.95181373	29.54463318	21.51443678	24.83287888	28.75805737
**ENSG00000159459**	UBR1	1754.975956	1765.614851	1452.592063	1193.895189	1039.054264	1058.475092	896.7844016	794.1074401	890.0906949	775.7852792	707.7370481	752.7844429
**ENSG00000159461**	AMFR	760.7708272	669.1119775	882.6288099	1075.845244	1110.713179	1312.281485	922.319265	918.7058878	1147.082107	1011.811306	924.3349361	1113.95181
**ENSG00000159479**	MED8	1027.954667	973.8907316	1088.629814	1151.196273	965.0837718	1221.988627	856.9500148	769.1877505	856.3253998	771.9886139	676.0061473	833.1378385
**ENSG00000159496**	RGL4	0	0	1.628466439	2.511700959	0	0	1.021394535	0	1.406887294	3.796665314	0	0.845825217
**ENSG00000159516**	SPRR2G	0	21.01922442	1.628466439	10.04680383	15.02525633	21.62475994	0	1.661312636	3.28273702	0	0	2.53747565
**ENSG00000159527**	PGLYRP3	1.406230734	0	13.84196473	4.186168264	2.311577897	10.24330734	2.04278907	3.322625272	13.13094808	1.898332657	2.759208765	16.07067912
**ENSG00000159556**	ISL2	37.96822982	3.50320407	35.82626166	35.16381342	6.93473369	48.94024618	6.128367209	13.29050109	6.56547404	6.327775524	22.07367012	9.304077385
**ENSG00000159579**	RSPRY1	683.4281368	641.0863449	810.1620533	674.8103242	592.9197305	658.6067237	676.1631821	709.3804956	692.6575113	575.1947951	651.1732684	605.6108552
**ENSG00000159588**	CCDC17	57.6554601	77.07048955	38.26896131	22.60530863	26.58314581	31.86806728	41.87717593	43.19412854	33.29633263	45.55998377	23.4532745	24.52893129
**ENSG00000159592**	GPBP1L1	2688.713164	2322.624299	2432.92886	2241.274489	2251.476871	2331.680256	2693.417388	2599.954276	2432.03917	2498.205777	2458.455009	2411.447693
**ENSG00000159593**	NAE1	1493.41704	1278.669486	1227.049462	1437.530182	1321.066768	1312.660866	1030.587086	966.8839542	993.2624298	1056.738512	996.074364	1072.506375
**ENSG00000159596**	TMEM69	409.2131436	325.7979785	434.8005392	459.6412754	359.4503629	451.4642864	410.600603	368.8114052	450.6728966	468.8881663	384.9096227	488.0411501
**ENSG00000159648**	TEPP	1.406230734	3.50320407	3.256932878	1.674467306	3.467366845	1.896908767	0	0	0	0	0	0
**ENSG00000159658**	EFCAB14	2462.310016	2501.287706	2387.331799	2000.151197	2089.666418	2180.306936	2871.140038	2864.102985	2789.857505	2530.477432	2826.809379	2764.156808
**ENSG00000159674**	SPON2	0	0	0.814233219	0.837233653	0	2.27629052	4.08557814	0	0.937924863	1.898332657	1.379604382	0.845825217
**ENSG00000159685**	CHCHD6	112.4984587	136.6249587	122.1349829	172.4701325	137.5388848	160.0990999	129.7171059	83.06563181	119.1164576	135.4143962	124.1643944	93.88659906
**ENSG00000159692**	CTBP1	656.7097529	725.1632426	682.3274379	706.625203	617.1912984	750.7964898	749.7035886	652.895866	846.4771888	811.8535997	783.6152892	718.9514343
**ENSG00000159712**	ANKRD18CF	0	0	0.814233219	0	0	0.758763507	3.064183605	11.62918845	2.344812157	1.265555105	6.898021912	0.845825217
**ENSG00000159713**	TPPP3	11.24984587	7.006408141	15.47043117	6.697869223	11.55788948	20.48661468	84.77574639	36.54887799	71.28228958	82.89385936	67.60061473	105.7281521
**ENSG00000159714**	ZDHHC1	4.218692202	7.006408141	11.39926507	9.209570182	18.49262317	14.79588838	20.4278907	16.61312636	15.00679781	24.04554699	9.657230676	7.612426951
**ENSG00000159720**	ATP6V0D1	607.4916772	504.4613861	696.9836358	636.2975762	690.0060021	809.9800433	581.1734903	659.5411165	680.9334505	650.4953238	684.2837736	712.1848325
**ENSG00000159733**	ZFYVE28	1.406230734	7.006408141	4.885399317	7.535102876	10.40210053	5.311344546	5.106972674	3.322625272	4.220661883	6.327775524	2.759208765	5.074951301
**ENSG00000159753**	CARMIL2	7.031153671	0	1.628466439	0.837233653	3.467366845	3.035054026	2.04278907	0	0.468962431	0.632777552	4.138813147	0
**ENSG00000159761**	C16orf86	2.812461468	7.006408141	1.628466439	5.023401917	8.090522638	7.208253313	0	3.322625272	2.813774589	2.53111021	2.759208765	9.304077385
**ENSG00000159784**	FAM131B	4.218692202	0	0	0	0	0	13.27812895	1.661312636	6.56547404	15.18666126	2.759208765	4.229126084
**ENSG00000159788**	RGS12	381.088529	357.3268152	433.986306	355.8243025	412.6166545	361.9301927	210.4072742	201.018829	223.2261174	237.2915821	184.8669872	282.5056224
**ENSG00000159792**	PSKH1	195.466072	238.2178768	257.2976973	288.0083766	238.0925233	258.7383558	223.6854031	169.4538889	233.0743284	282.8515659	144.8584601	184.3898973
**ENSG00000159840**	ZYX	1082.797665	847.775385	1223.792529	1156.219675	1048.300576	1639.687938	1148.047457	1008.41677	1506.30733	1276.312323	1058.156561	1373.620152
**ENSG00000159842**	ABR	396.557067	434.3973047	495.8680306	278.7988064	639.1512884	429.080763	15.32091802	21.59706427	18.75849726	5.694997971	19.31446135	14.37902869
**ENSG00000159860**	TCAF2P1	0	3.50320407	0	0	5.778944741	0.379381753	7.149761744	28.24231481	9.848211061	15.81943881	63.46180159	12.68737825
**ENSG00000159871**	LYPD5	1835.131108	1523.893771	1779.099584	613.6922676	453.0692677	553.5179781	615.9009045	795.7687527	711.8849709	251.2126883	176.5893609	255.4392155
**ENSG00000159873**	CCDC117	925.2998231	1131.534915	1222.164062	925.1431864	1155.788948	1193.155614	831.4151514	965.2226416	996.0762044	837.7974794	1020.907243	1211.22171
**ENSG00000159882**	ZNF230	71.71776744	101.592918	87.9371877	108.0031412	71.65891479	81.18769521	117.4603715	117.9531972	97.54418574	99.97885328	97.95191114	101.499026
**ENSG00000159884**	CCDC107	61.8741523	56.05126513	65.13865756	36.83828073	71.65891479	67.15057033	16.34231256	29.90362745	34.70321993	14.5538837	46.906549	43.13708606
**ENSG00000159885**	ZNF222	66.09284451	66.56087734	77.35215585	60.28082301	24.27156791	35.66188481	23.4920743	51.50069172	30.48255804	51.8877593	59.32298844	41.44543562
**ENSG00000159899**	NPR 2.00	28.12461468	42.03844885	48.03975995	30.1404115	52.01050267	53.11344546	7.149761744	4.983937908	12.19302322	3.796665314	4.138813147	12.68737825
**ENSG00000159905**	ZNF221	143.4355349	133.1217547	125.3919158	121.3988797	108.6441611	125.9547421	127.6743169	119.6145098	135.5301427	125.9227329	155.8952952	169.1650434
**ENSG00000159915**	ZNF233	0	3.50320407	0.814233219	3.348934611	2.311577897	3.035054026	13.27812895	9.967875817	2.813774589	10.12444084	16.55525259	4.229126084
**ENSG00000159917**	ZNF235	112.4984587	157.6441832	120.5065165	141.4924873	113.2673169	122.1609246	142.9952349	144.5341993	128.9646686	136.6799513	189.0058004	174.2399947
**ENSG00000159921**	GNE	164.5289959	112.1025303	164.4751103	189.2148055	199.951488	173.3774613	311.5253331	357.1822168	373.7630579	345.4965436	379.3912051	480.4287231

**ENSG00000159958**	TNFRSF13C	30.93707615	10.50961221	10.58503185	38.51274803	17.33683422	15.55465189	18.38510163	9.967875817	26.26189616	35.43554293	57.94338406	34.67883389
**ENSG00000160007**	ARHGAP35	1788.725494	1285.675894	1536.458085	2052.059683	2105.847464	1935.605705	1773.140913	1837.411776	1797.533	1784.432698	1867.984334	1967.389454
**ENSG00000160014**	CALM3	1522.947885	1408.288036	1454.22053	1405.715303	1538.35509	1418.508376	874.3137219	890.463573	1006.393378	985.2346491	1002.972386	1141.864043
**ENSG00000160049**	DFFA	1293.732275	938.8586909	1252.290692	1480.229098	1400.816205	1536.496101	1136.812117	814.0431917	1251.66073	1311.747866	1033.323682	1405.76151
**ENSG00000160050**	CCDC28B	36.56199909	24.52242849	17.09889761	43.53614995	46.23155793	35.28250306	30.64183605	28.24231481	30.01359561	48.72387153	27.59208765	30.4497078
**ENSG00000160051**	IQCC	172.9663803	143.6313669	162.0324107	239.4488247	228.8462118	161.9960087	191.000778	182.74439	172.1092123	205.019927	245.5695801	213.1479546
**ENSG00000160055**	TMEM234	267.1838395	353.8236111	327.3217542	275.4498718	314.3745939	257.9795922	282.9262862	317.3107135	271.9982102	271.46157	286.9577115	254.5933902
**ENSG00000160058**	BSDC1	1385.137273	1621.983485	1590.197478	1170.452647	1351.117281	1343.390788	1209.331129	1342.34061	1287.301874	1228.221229	1276.134054	1098.726957
**ENSG00000160062**	ZBTB8A	153.27915	185.6698157	146.5619795	171.6328988	187.2378096	152.1320831	133.8026841	124.5984477	138.3439173	140.4766166	161.4137127	181.0065964
**ENSG00000160072**	ATAD3B	554.0549093	437.9005088	546.3504902	552.5742109	456.5366346	588.0417176	339.1029856	358.8435294	390.6457054	434.0854009	274.5412721	386.5421241
**ENSG00000160075**	SSU72	1480.760963	1303.191914	1472.133661	1634.28009	1473.630909	1526.632175	1363.561704	1139.660468	1344.046329	1259.227329	1183.70056	1274.658602
**ENSG00000160087**	UBE2J2	608.8979079	528.9838146	604.975282	721.6954088	603.321831	739.4150372	488.2265877	571.4915468	513.9828249	541.6575848	591.85028	554.015517
**ENSG00000160094**	ZNF362	449.9938349	353.8236111	276.0250614	568.4816503	606.7891978	543.2746707	1011.18059	554.8784205	986.6969558	1076.354617	796.0317286	1086.039578
**ENSG00000160111**	CPAMD8	97.02992066	24.52242849	64.32442434	179.1680017	131.7599401	95.60420183	657.7780805	382.1019063	534.1482094	734.0219608	547.7029398	659.7436691
**ENSG00000160113**	NR2F6	340.3078377	304.7787541	297.1951251	361.684938	402.214554	364.2064832	479.0340369	466.8288508	519.6103741	479.0126072	422.158941	489.7328005
**ENSG00000160117**	ANKLE1	5.624922937	3.50320407	1.628466439	1.674467306	6.93473369	3.793817533	0	3.322625272	1.406887294	0.632777552	11.03683506	0.845825217
**ENSG00000160124**	CCDC58	359.9950679	413.3780803	381.8753799	434.5242658	372.1640413	366.4827737	262.4983955	345.5530283	277.6257594	295.507117	315.9294035	335.7926111
**ENSG00000160131**	VMA21	1082.797665	819.7497525	974.6371637	1169.615413	1089.908978	1214.400992	1057.143344	787.4621895	1187.412876	1112.422937	829.1422338	1165.547149
**ENSG00000160145**	KALRN	4.218692202	0	30.94086234	12.55850479	28.89472371	22.00414169	1.021394535	11.62918845	1.875849726	6.960553076	13.79604382	3.383300867
**ENSG00000160161**	CILP2	25.31215321	0	10.58503185	24.27977593	21.95999002	7.208253313	5.106972674	1.661312636	9.379248629	5.062220419	2.759208765	5.074951301
**ENSG00000160172**	FAM86C2P	47.81184496	35.0320407	43.96859385	46.88508456	49.69892478	43.24951988	38.81299233	31.56494009	52.52379232	60.74664503	40.00852709	46.52038692
**ENSG00000160179**	ABCG1	71.71776744	154.1409791	98.52221955	108.8403749	194.1725433	105.0887457	207.3430906	362.1661547	216.1916809	224.0032535	339.382678	181.8524216
**ENSG00000160181**	TFF2	0	24.52242849	11.39926507	5.023401917	18.49262317	12.14021611	1.021394535	0	0	5.062220419	9.657230676	0
**ENSG00000160183**	TMPRSS3	178.5913032	574.5254675	376.1757474	5.023401917	33.5178795	26.93610448	10.21394535	13.29050109	13.13094808	5.062220419	9.657230676	0.845825217
**ENSG00000160188**	RSPH1	8.437384405	0	3.256932878	13.39573845	5.778944741	0.758763507	1.021394535	4.983937908	10.78613592	4.429442867	6.898021912	2.53747565
**ENSG00000160190**	SLC37A1	149.0604578	217.1986524	201.1156052	116.3754777	176.8357091	151.3733196	81.71156279	89.71088235	116.7716454	48.09109398	60.70259282	71.89514343
**ENSG00000160191**	PDE9A	0	0	2.442699658	0	0	3.41443578	0	0	0	0	0	0
**ENSG00000160193**	WDR4	449.9938349	301.2755501	449.4567371	551.7369772	391.8124535	500.7839144	282.9262862	201.018829	355.0045606	329.0443272	209.6998661	340.0217371
**ENSG00000160194**	NDUFV3	330.4642225	280.2563256	349.3060511	375.0806765	343.2693176	364.5858649	351.35972	270.7939597	319.8323783	351.1915416	321.4478211	353.5549406
**ENSG00000160199**	PKNOX1	316.4019152	294.2691419	314.2940227	296.3807131	310.9072271	324.3713991	236.9635321	204.3414542	277.156797	253.111021	267.6432502	252.0559146
**ENSG00000160200**	CBS	129.3732275	73.56728548	103.4076189	72.8393278	71.65891479	65.25366157	21.44928523	9.967875817	15.00679781	15.18666126	11.03683506	16.07067912
**ENSG00000160201**	U2AF1	74.53022891	94.5865099	70.02405687	60.28082301	61.25681426	62.21860754	76.60459012	76.42038126	69.87540229	98.71329817	102.0907243	78.66174516
**ENSG00000160207**	HSF2BP	28.12461468	14.01281628	24.42699658	17.58190671	15.02525633	26.17734098	23.4920743	14.95181373	19.69642212	13.2883286	12.41643944	28.75805737
**ENSG00000160208**	RRP1B	1029.360897	861.7882013	1063.388585	1352.969583	1193.929984	1447.341389	732.3398815	528.2974183	885.4010706	946.0024408	695.3206087	903.3413315
**ENSG00000160209**	PDXK	1493.41704	1614.977076	1632.537605	1528.78865	1725.5929	1872.628334	1060.207527	1169.564096	1201.481749	1136.468484	1165.765703	1159.626372
**ENSG00000160211**	G6PD	610.3041386	511.4677943	581.3625187	507.3635936	577.8944741	592.9736804	467.798697	421.9734096	499.4449895	450.5376173	568.3970055	515.9533822
**ENSG00000160213**	CSTB	1011.079898	1593.957852	860.644513	985.4240094	1138.452114	935.1760219	279.8621026	280.7618355	369.542396	309.4282231	326.9662386	395.0003762
**ENSG00000160214**	RRP1	455.6187579	210.1922442	454.3421365	495.6423225	322.4651166	489.7818435	255.3486337	214.3093301	275.2809473	244.2521352	216.597888	268.1265937
**ENSG00000160216**	AGPAT3	1425.917964	1271.663078	1555.999682	947.748495	962.7721939	1105.518429	849.800253	775.8330011	840.3806772	715.0386342	503.5555995	727.4096864
**ENSG00000160218**	TRAPPC10	718.5839052	844.272181	753.9799612	707.4624367	609.1007757	729.9304934	583.2162794	619.6696133	578.6996404	577.7259053	616.6831589	508.3409553
**ENSG00000160219**	GAB3	2.812461468	14.01281628	13.02773151	6.697869223	9.246311586	7.587635066	6.128367209	3.322625272	1.406887294	3.796665314	0	3.383300867
**ENSG00000160221**	C21orf33	2.812461468	0	0.814233219	0	1.155788948	0	1.021394535	0	2.344812157	0.632777552	0	0
**ENSG00000160223**	ICOSLG	0	3.50320407	1.628466439	1.674467306	3.467366845	2.655672273	0	0	2.813774589	0	4.138813147	5.074951301
**ENSG00000160226**	C21orf2	14.06230734	17.51602035	17.09889761	12.55850479	17.33683422	7.208253313	33.70601965	9.967875817	21.57227185	25.3111021	13.79604382	15.2248539
**ENSG00000160229**	ZNF66	7.031153671	14.01281628	3.256932878	10.04680383	13.86946738	7.967016819	290.0760479	292.391024	245.2673516	315.1232211	295.2353378	298.5763015
**ENSG00000160233**	LRRC3	4.218692202	14.01281628	8.142332194	6.697869223	11.55788948	2.27629052	1.021394535	1.661312636	0.468962431	1.265555105	12.41643944	0
**ENSG00000160255**	ITGB2	4.218692202	14.01281628	1.628466439	0	1.155788948	2.655672273	0	1.661312636	0.468962431	1.898332657	0	2.53747565
**ENSG00000160256**	FAM207A	175.7788418	210.1922442	184.8309408	205.9594786	138.6946738	192.3465489	87.83993	111.3079466	94.26144872	103.142741	93.813098	134.4862095
**ENSG00000160271**	RALGDS	18.28099954	70.06408141	31.75509556	41.02444899	39.29682424	32.24744903	51.06972674	43.19412854	29.54463318	72.76941852	49.66575776	62.59106604
**ENSG00000160282**	FTCD	1.406230734	0	3.256932878	0	1.155788948	2.27629052	0	0	0.468962431	0	0	2.53747565
**ENSG00000160284**	SPATC1L	32.34330689	35.0320407	39.89742775	24.27977593	36.98524634	48.18148267	0	0	0	1.898332657	1.379604382	0.845825217
**ENSG00000160285**	LSS	390.9321441	420.3844885	356.6341501	509.8752946	566.3365846	513.682894	229.8137703	415.328159	264.0258489	273.3599026	543.5641266	286.7347485
**ENSG00000160293**	VAV2	490.7745262	560.5126513	739.3237633	776.9528299	1044.833209	1000.050302	1351.30497	1231.032663	1366.087563	1292.131762	1379.604382	1268.737825
**ENSG00000160294**	MCM3AP	1574.978422	1478.352118	1601.596743	1595.767342	1603.079271	1717.840579	1264.486434	1231.032663	1264.322715	1268.718993	1436.168162	1378.695103
**ENSG00000160298**	C21orf58	341.7140684	350.320407	334.6498532	426.1519293	509.7029262	376.3466993	418.7717593	478.4580392	428.6316623	496.7303786	478.7227207	438.1374623
**ENSG00000160299**	PCNT	1304.982121	1436.313669	1410.251936	1512.043977	1544.134035	1553.56828	1402.374696	1666.296574	1450.5008	1566.75722	1661.043676	1534.326943
**ENSG00000160305**	DIP2A	530.1489868	679.6215897	626.1453458	661.4145858	552.4671173	582.7303731	584.2376739	621.3309259	523.8310359	606.8336727	584.9522581	583.6193996
**ENSG00000160310**	PRMT2	476.7122189	742.6792629	591.9475505	473.8742475	646.0860221	586.1448089	452.477779	646.2506155	414.5627894	482.8092725	746.3659708	411.0710554
**ENSG00000160318**	CLDND2	1.406230734	0	0	4.186168264	4.623155793	1.896908767	1.021394535	0	4.689624315	1.898332657	2.759208765	2.53747565
**ENSG00000160321**	ZNF208	0	0	0	0	1.155788948	0.758763507	108.2678207	109.646634	127.5577814	115.7982921	121.4051856	132.794559
**ENSG00000160323**	ADAMTS13	15.46853808	7.006408141	13.84196473	4.186168264	8.090522638	9.105162079	1.021394535	3.322625272	5.627549177	1.265555105	11.03683506	1.691650434
**ENSG00000160325**	CACFD1	36.56199909	17.51602035	36.64049488	41.86168264	55.47786952	46.66395566	9.192550814	13.29050109	18.75849726	20.88165923	41.38813147	11.84155303
**ENSG00000160326**	SLC2A6	63.28038304	91.08330583	72.46675653	84.56059894	100.5536385	75.49696891	21.44928523	8.306563181	7.972361335	6.327775524	19.31446135	5.920776517
**ENSG00000160336**	ZNF761	419.0567588	507.9645902	430.7293731	394.3370505	507.3913483	417.6993104	1056.121949	1003.432832	952.9316607	1219.995121	1190.598582	1055.589871
**ENSG00000160345**	C9orf116	42.18692202	31.52883663	12.21349829	61.11805666	35.8294574	27.31548624	28.59904698	28.24231481	37.04803208	52.52053685	59.32298844	38.90795997
**ENSG00000160352**	ZNF714	219.3719945	231.2114686	196.2302059	354.9870688	271.6104028	268.9816631	602.6227756	501.7164161	508.3552757	618.8564462	543.5641266	627.6023108
**ENSG00000160360**	GPSM1	172.9663803	196.1794279	111.5499511	189.2148055	152.5641412	191.9671672	30.64183605	41.5328159	29.07567075	31.00610007	23.4532745	12.68737825

**ENSG00000160392**	C19orf47	165.9352266	203.1858361	140.0481137	177.4935344	198.7956991	195.7609847	179.7654381	224.2772059	200.7159207	268.9304598	269.0228546	211.4563042
**ENSG00000160396**	HIPK4	0	0	8.142332194	2.511700959	1.155788948	1.13814526	4.08557814	0	0.468962431	0	0	0
**ENSG00000160401**	CFAP157	0	10.50961221	0	0	0	1.517527013	2.04278907	4.983937908	0.937924863	0	0	0
**ENSG00000160404**	TOR2A	82.96761332	112.1025303	86.30872126	98.79357104	114.4231059	111.9176172	114.3961879	91.37219499	120.9923073	102.5099635	86.91507609	71.89514343
**ENSG00000160408**	ST6GALNAC	14.06230734	0	4.071166097	2.511700959	6.93473369	7.967016819	4.08557814	8.306563181	10.31717349	9.491663286	1.379604382	6.766601734
**ENSG00000160410**	SHKBP1	260.1526858	304.7787541	268.6969624	277.1243391	247.3388349	270.4991901	258.4128173	240.8903322	236.3570655	284.117121	246.9491844	243.5976624
**ENSG00000160439**	RDH13	451.4000657	795.227324	508.0815289	515.7359302	649.5533889	583.1097548	498.440533	1174.548034	559.4721807	505.5892644	1114.720341	518.4908579
**ENSG00000160445**	ZER1	459.8374501	599.047896	574.0344197	492.2933879	614.8797205	542.895289	660.8422641	749.2519989	694.533361	632.7775524	754.6435971	575.1611474
**ENSG00000160446**	ZDHHC12	449.9938349	444.9069169	429.9151399	545.039108	552.4671173	549.3447788	425.921521	431.9412854	469.4313939	417.000407	413.8813147	450.8248405
**ENSG00000160447**	PKN3	149.0604578	227.7082646	139.2338805	123.9105806	152.5641412	149.097029	112.3533988	132.9050109	119.1164576	122.7588452	143.4788558	123.4904816
**ENSG00000160460**	SPTBN4	0	28.02563256	7.328098975	6.697869223	11.55788948	3.793817533	5.106972674	1.661312636	4.220661883	3.796665314	6.898021912	0.845825217
**ENSG00000160469**	BRSK1	12.65607661	17.51602035	14.65619795	12.55850479	41.60840214	14.79588838	0	0	0.468962431	0	0	0
**ENSG00000160471**	COX6B2	1.406230734	0	1.628466439	5.86063557	4.623155793	2.655672273	0	0	0	0	0	0
**ENSG00000160505**	NLRP4	1.406230734	10.50961221	18.72736405	5.86063557	11.55788948	6.449489806	0	0	0.937924863	0.632777552	1.379604382	0.845825217
**ENSG00000160539**	PLPP7	7.031153671	0	4.885399317	9.209570182	2.311577897	6.070108053	4.08557814	6.645250545	2.813774589	1.265555105	13.79604382	6.766601734
**ENSG00000160551**	TAOK1	3450.890222	4144.290415	3423.036455	3650.338727	4029.080274	3746.774196	3134.659828	3382.432527	3146.268953	3016.450592	3322.087353	3467.037564
**ENSG00000160563**	MED27	351.5576835	308.2819582	394.0888782	377.5923774	413.7724435	445.7735601	352.3811145	338.9077778	324.990965	386.6270845	401.4648753	385.6962988
**ENSG00000160570**	DEDD2	178.5913032	164.6505913	206.8152377	130.6084498	190.7051765	200.3135657	122.5673442	89.71088235	153.3507151	86.69052468	131.0624163	159.860966
**ENSG00000160584**	SIK3	1940.598413	1247.140649	1798.641182	1220.686666	805.5848969	1382.467109	1165.411164	752.5746242	1121.289174	893.481904	637.3772246	930.4077385
**ENSG00000160588**	MPZL3	793.1141341	1047.458017	793.0631557	1913.078897	1594.988749	1740.603484	614.87951	724.3323094	692.1885488	848.5546977	877.4283871	995.5362801
**ENSG00000160593**	JAML	0	7.006408141	11.39926507	5.86063557	6.93473369	7.208253313	11.23533988	1.661312636	2.344812157	4.429442867	0	0
**ENSG00000160602**	NEK8	52.03053716	59.5544692	61.06749146	61.11805666	39.29682424	45.90519215	94.98969174	106.3240087	95.19937358	129.0866207	93.813098	77.81591994
**ENSG00000160606**	TLCD1	206.7159179	157.6441832	170.9889761	174.1445998	172.2125533	182.8620051	195.0863562	89.71088235	208.688282	185.4038228	113.1275593	198.7689259
**ENSG00000160613**	PCSK7	648.2723684	693.6344059	723.0390989	716.6720069	683.0712684	732.5861656	862.0569874	785.8008769	822.0911423	847.9219202	812.5869812	754.4760934
**ENSG00000160633**	SAFB	1686.07065	1485.358526	1613.810241	1675.304539	1560.31508	1614.648742	1808.889721	2033.446667	1834.112069	1811.642132	2052.851321	2101.029838
**ENSG00000160678**	S100A1	1.406230734	0	0	0	3.467366845	0	0	8.306563181	0.468962431	3.796665314	2.759208765	4.229126084
**ENSG00000160679**	CHTOP	1586.228268	1688.544362	1615.438707	1646.838595	1441.268818	1546.360026	1735.349315	1938.751846	1662.002857	1716.7255	1789.346884	1698.417035
**ENSG00000160683**	CXCR5	7.031153671	0	1.628466439	5.86063557	1.155788948	1.517527013	5.106972674	4.983937908	2.344812157	7.593330629	0	0
**ENSG00000160685**	ZBTB7B	487.9620648	550.0030391	591.1333173	608.6688656	552.4671173	664.29745	554.6172324	460.1836002	706.7263842	637.8397728	474.5839075	654.6687178
**ENSG00000160688**	FLAD1	473.8997574	332.8043867	534.136992	546.7135753	433.4208556	561.1056131	468.8200915	388.7471569	529.9275475	491.6681582	357.317535	496.4994022
**ENSG00000160691**	SHC1	3426.984299	2900.65297	3323.700002	3650.338727	3330.983749	3662.930828	2186.805699	1500.16531	2601.334607	2352.034162	1655.525259	2475.73041
**ENSG00000160695**	VPS11	577.9608317	693.6344059	751.5372615	625.4135387	591.7639415	643.4314536	588.3232521	639.6053649	472.2451685	563.8047992	521.4904565	556.5529926
**ENSG00000160703**	NLRX1	102.6548436	66.56087734	116.4353504	122.2361133	128.2925733	121.4021611	109.2892152	74.75906863	111.1440963	113.8999594	104.8499331	93.88659906
**ENSG00000160710**	ADAR	12393.11146	13553.89655	13078.21397	6228.181144	7147.398856	6376.269128	10955.47778	10491.1893	11231.65023	6481.540469	6172.350006	6687.939989
**ENSG00000160712**	IL6R	381.088529	441.4037129	358.2626166	400.1976861	425.330333	370.2765912	101.118059	260.8260839	105.0475846	98.08052062	280.0596896	151.4027138
**ENSG00000160714**	UBE2Q1	953.4244378	1036.948405	930.6685698	914.2591489	884.1785454	939.7286029	728.2543034	950.2708279	717.5125201	703.0158607	716.0146744	784.9258012
**ENSG00000160741**	CRTC2	419.0567588	451.9133251	545.536257	381.7785457	388.3450866	497.3694786	307.439755	378.779281	409.8731651	417.000407	322.8274255	382.312998
**ENSG00000160746**	ANO10	187.0286876	220.7018564	277.6535278	221.0296844	298.1935487	259.1177375	182.8296217	249.1968954	215.7227185	267.0321271	284.1985028	196.2314503
**ENSG00000160752**	FDPS	1809.818955	1898.736606	1797.012715	1602.465212	1547.601402	1607.440489	1128.640961	1290.839918	1238.060819	1251.001221	1458.241832	1216.296662
**ENSG00000160753**	RUSC1	278.4336854	416.8812844	311.851323	315.6370871	313.218805	315.6456187	220.6212195	277.4392102	287.4739705	272.0943475	295.2353378	253.747565
**ENSG00000160766**	GBAP1	142.0293041	161.1473872	151.4473788	101.305272	102.8652164	120.2640158	92.94690267	84.72694444	88.16493711	90.48718999	107.6091418	76.12426951
**ENSG00000160767**	FAM189B	351.5576835	245.2242849	285.79586	317.3115544	295.8819708	342.961105	263.51979	294.0523366	305.2945429	270.8287924	369.7339745	362.0131928
**ENSG00000160781**	PAQR6	16.87476881	38.53524477	25.2412298	15.07020575	24.27156791	17.0721789	8.171156279	28.24231481	20.63434698	25.94387965	49.66575776	14.37902869
**ENSG00000160783**	PMF1	171.5601496	112.1025303	166.1035768	149.8648239	151.4083522	213.9713089	200.1933288	206.0027669	189.4608223	211.98048	176.5893609	139.5611608
**ENSG00000160785**	SLC25A44	555.46114	504.4613861	673.3708725	741.7890164	598.6986752	725.3779123	512.7400565	662.8637418	627.4717333	640.370883	673.2469386	641.1355143
**ENSG00000160789**	LMNA	6387.099995	5237.290085	5609.252649	7328.306164	7290.716686	7438.538037	5840.33395	6528.95866	6665.832001	5838.638476	6157.174358	6029.042145
**ENSG00000160791**	CCR5	0	0	0	0	0	0	3.064183605	0	1.406887294	7.593330629	2.759208765	1.691650434
**ENSG00000160796**	NBEAL2	229.2156097	178.6634076	196.2302059	299.7296477	263.5198802	268.2228996	289.0546534	161.1473257	322.1771904	341.6998783	241.4307669	278.2764963
**ENSG00000160799**	CCDC12	184.2162262	203.1858361	235.3134004	221.866918	181.4588649	207.9012008	296.2044151	242.5516449	264.0258489	302.46767	215.2182836	253.747565
**ENSG00000160801**	PTH1R	1.406230734	0	1.628466439	0.837233653	3.467366845	2.27629052	0	9.967875817	0	0	2.759208765	0.845825217
**ENSG00000160803**	UBQLN4	807.1764414	714.6536304	784.1065903	1001.331449	907.2943244	1103.62152	538.2749199	583.1207353	695.9402483	675.1736484	571.1562143	719.7972595
**ENSG00000160813**	PPP1R35	158.904073	91.08330583	122.9492161	119.7244124	113.2673169	150.6145561	153.2091802	114.6305719	171.1712875	117.6966247	110.3683506	143.7902869
**ENSG00000160818**	GPATCH4	804.3639799	448.410121	796.3200886	1095.938852	768.5996506	941.2461299	615.9009045	330.6012146	554.313594	650.4953238	435.9549848	657.2061934
**ENSG00000160862**	AZGP1	4.218692202	7.006408141	8.142332194	7.535102876	36.98524634	21.24537819	0	0	1.406887294	1.265555105	0	0
**ENSG00000160867**	FGFR4	9.843615139	24.52242849	10.58503185	32.65211246	46.23155793	22.7629052	1.021394535	8.306563181	3.28273702	1.265555105	0	1.691650434
**ENSG00000160868**	CYP3A4	0	0	1.628466439	0.837233653	0	0	7.149761744	0	0.468962431	6.327775524	0	0.845825217
**ENSG00000160870**	CYP3A7	1.406230734	0	1.628466439	0	0	0	9.192550814	8.306563181	2.813774589	0.632777552	4.138813147	0
**ENSG00000160877**	NACC1	959.0493607	571.0222635	930.6685698	1064.123973	975.4858723	1176.842199	524.9967909	491.7485403	818.3394429	686.5636443	544.943731	724.8722108
**ENSG00000160886**	LY6K	614.5228308	886.3106298	639.9873105	536.6667715	639.1512884	625.2211294	889.6346399	1953.70366	921.0422154	771.3558364	1546.536513	790.0007525
**ENSG00000160888**	IER2	1359.82512	1481.855322	1559.256615	1379.76106	1124.582647	1559.259006	863.078382	800.7526906	1012.958852	769.4575037	692.5613999	886.4248272
**ENSG00000160908**	ZNF394	310.7769922	255.7338971	388.3892457	317.3115544	287.7914481	330.4415071	403.4508413	373.7953431	385.9560811	373.3387559	326.9662386	344.2508632
**ENSG00000160917**	CPSF4	220.7782253	227.7082646	233.684934	289.6828439	261.2083023	259.4971193	311.5253331	353.8595915	355.473523	298.6710047	311.7905904	315.4928059
**ENSG00000160932**	LY6E	3003.708848	2378.675564	2185.401961	1838.565102	1662.024508	1937.881996	92.94690267	99.67875817	155.6955272	91.7527451	95.19270238	104.8823269
**ENSG00000160948**	VPS28	568.1172166	542.9966309	546.3504902	557.5976128	529.3513383	585.7654271	419.7931538	300.6975871	418.3144889	372.0732008	357.317535	383.1588232
**ENSG00000160949**	TONSL	472.4935267	462.4229373	401.4169772	503.1774254	426.4861219	556.1736503	293.1402315	302.3588998	332.9633263	277.7893455	365.5951613	237.6768859
**ENSG00000160951**	PTGER1	2.812461468	7.006408141	1.628466439	4.186168264	2.311577897	1.896908767	3.064183605	13.29050109	3.751699452	1.898332657	0	5.920776517
**ENSG00000160953**	MUM1	524.5240638	567.5190594	486.9114652	504.8518927	486.5871472	555.7942686	436.1354664	403.6989706	476.4658304	365.7454253	451.130633	451.6706658

**ENSG00000160957**	RECQL4	234.8405326	336.3075908	302.8947576	309.7764516	321.3093276	375.208554	213.4714578	222.6158932	285.5981208	296.1398945	295.2353378	253.747565
**ENSG00000160959**	LRRC14	320.6206074	210.1922442	310.2228566	321.4977227	287.7914481	331.2002706	305.3969659	245.8742701	320.3013407	298.6710047	309.0313816	305.3429033
**ENSG00000160961**	ZNF333	250.3090707	238.2178768	209.2579374	154.8882258	158.3430859	172.6186978	342.1671692	260.8260839	309.5152048	292.9760068	227.6347231	262.2058172
**ENSG00000160963**	COL26A1	0	0	3.256932878	4.186168264	0	2.655672273	1.021394535	0	1.406887294	0	0	0.845825217
**ENSG00000160972**	PPP1R16A	184.2162262	259.2371012	203.5583049	277.9615728	271.6104028	246.5981396	223.6854031	224.2772059	195.0883715	258.1732414	266.2636458	232.6019346
**ENSG00000160973**	FOXH1	1.406230734	0	7.328098975	0	4.623155793	0	0	0	0	0.632777552	1.379604382	0.845825217
**ENSG00000160991**	ORAI2	157.4978422	84.07689769	96.07951989	158.2371604	151.4083522	121.7815428	15.32091802	54.82331699	41.7376564	23.41276944	26.21248326	45.67456171
**ENSG00000160993**	ALKBH4	92.81122845	56.05126513	65.95289078	95.44463643	55.47786952	82.32584047	80.69016826	84.72694444	64.24785311	91.11996754	78.63744979	73.58679386
**ENSG00000160999**	SH2B2	7.031153671	3.50320407	1.628466439	5.86063557	12.71367843	7.967016819	9.192550814	21.59706427	12.66198565	6.327775524	30.35129641	8.458252168
**ENSG00000161010**	MRNIP	132.185689	80.57369362	153.0758453	151.5392912	151.4083522	148.7176473	158.3161529	186.0670152	195.0883715	158.8271656	189.0058004	163.2442668
**ENSG00000161011**	SQSTM1	8350.198099	12488.92251	12689.01049	4670.089316	6737.093779	7732.938278	3892.534572	4341.009918	4859.857677	3152.497766	3066.860542	3399.371546
**ENSG00000161013**	MGAT4B	1404.824503	1867.20777	1569.027414	1317.80577	1561.470869	1514.871341	1024.458718	1207.774286	1200.543825	1121.281823	1233.366318	1130.02249
**ENSG00000161016**	RPL8	10144.54852	9283.490787	9758.585135	9937.96346	10059.98701	10516.84158	8652.233105	8788.343845	9912.927876	8830.410743	8772.904267	9782.814457
**ENSG00000161021**	MAML1	856.3945171	791.7241199	986.850662	918.4453172	921.1637918	952.6275825	914.1481087	983.4970806	993.2624298	984.6018715	987.7967377	991.3071541
**ENSG00000161036**	LRWD1	134.9981505	157.6441832	157.9612446	164.097796	171.0567643	207.9012008	108.2678207	101.3400708	136.93703	128.4538431	89.67428485	130.2570834
**ENSG00000161040**	FBXL13	32.34330689	3.50320407	4.885399317	18.41914036	17.33683422	17.0721789	21.44928523	19.93575163	15.00679781	20.88165923	15.17564821	15.2248539
**ENSG00000161048**	NAPEPLD	359.9950679	227.7082646	262.9973299	256.1934978	271.6104028	237.8723593	337.0601965	335.5851525	365.3217341	300.5693374	267.6432502	292.655525
**ENSG00000161057**	PSMC2	2535.434014	2585.364604	2616.945567	2278.112769	2265.346339	2277.808047	1584.182924	1780.927146	1635.271998	1666.103295	1659.664072	1916.639941
**ENSG00000161082**	CELF5	18.28099954	0	2.442699658	8.372336529	10.40210053	7.587635066	0	0	0	0	1.379604382	0.845825217
**ENSG00000161091**	MFSD12	1385.137273	1429.307261	1648.822269	936.0272239	998.6016513	1231.852553	440.2210445	357.1822168	537.4309464	437.8820662	383.5300183	483.812024
**ENSG00000161103**	AC008132.1	0	0	0	0	0	0.379381753	24.51346884	48.17806645	36.57906965	22.14721433	35.86971394	20.2998052
**ENSG00000161149**	TUBA3FP	14.06230734	7.006408141	10.58503185	9.209570182	9.246311586	1.896908767	18.38510163	38.21019063	49.2410553	36.06832049	30.35129641	44.82873649
**ENSG00000161179**	YDJC	254.5277629	175.1602035	195.4159727	240.2860584	165.2778196	261.0146463	306.4183605	212.6480174	346.0942744	330.3098823	262.1248326	347.6341641
**ENSG00000161180**	CCDC116	23.90592248	17.51602035	9.770798633	18.41914036	8.090522638	12.14021611	12.25673442	18.274439	7.034436472	13.2883286	13.79604382	11.84155303
**ENSG00000161202**	DVL3	907.0188235	879.3042217	961.6094322	1117.706927	1065.63741	1135.110206	1112.298648	1237.677914	1227.274683	1175.067915	1129.895989	1113.105985
**ENSG00000161203**	AP2M1	1340.13789	1222.618221	1264.50419	1602.465212	1708.256066	1769.057116	1499.407177	1614.795882	1783.933089	1568.022775	1706.570621	1633.288494
**ENSG00000161204**	ABCF3	445.7751427	511.4677943	522.7377269	545.8763417	499.3008257	561.1056131	424.9001265	505.0390414	463.3348823	494.1992684	565.6377967	494.8077518
**ENSG00000161217**	PCYT1A	1770.444494	1940.775055	2119.44907	956.9580652	1051.767943	1092.240068	1044.886609	1111.418154	1035.938011	875.1313549	816.7257943	927.8702628
**ENSG00000161243**	FBXO27	127.9669968	315.2883663	144.9335131	129.7712162	189.5493875	158.9609546	87.83993	111.3079466	93.79248629	72.76941852	71.73942788	96.42407471
**ENSG00000161249**	DMKN	562.4922937	564.0158553	578.919819	558.4348465	587.1407857	444.2560331	78.64737919	88.04956971	128.4957062	62.64497769	68.98021912	132.794559
**ENSG00000161265**	U2AF1L4	5.624922937	7.006408141	4.885399317	9.209570182	4.623155793	3.793817533	7.149761744	3.322625272	9.379248629	10.75721839	19.31446135	11.84155303
**ENSG00000161267**	BDH1	66.09284451	45.54165292	26.86969624	87.90953355	83.21680428	71.70315137	24.51346884	24.91968954	18.28953483	44.29442867	45.52694462	23.68310607
**ENSG00000161277**	THAP8	23.90592248	56.05126513	24.42699658	17.58190671	35.8294574	21.24537819	19.40649616	18.274439	18.75849726	15.81943881	41.38813147	19.45397999
**ENSG00000161298**	ZNF382	1.406230734	0	6.513865756	0.837233653	10.40210053	4.55258104	7.149761744	23.25837691	8.441323766	1.265555105	15.17564821	3.383300867
**ENSG00000161328**	LRRC56	49.2180757	91.08330583	39.89742775	63.62975762	53.16629162	33.38559429	60.26227756	48.17806645	45.48935585	47.45831643	63.46180159	46.52038692
**ENSG00000161381**	PLXDC1	0	0	0	0	1.155788948	0	2.04278907	6.645250545	3.751699452	0.632777552	0	0.845825217
**ENSG00000161395**	PGAP3	39.37446056	70.06408141	52.92515926	51.90848648	70.50312584	53.87220897	136.8668677	129.5823856	128.0267438	130.3521758	97.95191114	146.3277625
**ENSG00000161405**	IKZF3	14.06230734	3.50320407	25.2412298	25.11700959	26.58314581	22.38352344	18.38510163	6.645250545	22.04123428	32.90443272	6.898021912	26.22058172
**ENSG00000161509**	GRIN2C	0	0	4.885399317	0	1.155788948	1.896908767	0	0	0	1.265555105	11.03683506	2.53747565
**ENSG00000161513**	FDXR	94.21745919	101.592918	130.2773151	143.1669546	134.071518	119.1258705	94.98969174	124.5984477	135.9991051	127.188288	143.4788558	148.0194129
**ENSG00000161526**	SAP30BP	1120.765895	1114.018894	1187.152034	1019.750589	1036.742687	1067.580254	698.6338619	910.3993246	758.3122517	733.3891832	735.3291358	684.2726004
**ENSG00000161533**	ACOX1	776.2393653	816.2465484	758.0511273	1108.497356	1178.904727	1169.254564	2155.142469	1922.13872	2180.675306	2383.040262	2005.944772	2297.261289
**ENSG00000161542**	PRPSAP1	406.4006822	490.4485699	524.3661933	560.9465474	637.9954994	530.3756911	424.9001265	573.1528595	481.1554547	485.3403827	605.6463238	577.6986231
**ENSG00000161547**	SRSF2	3089.488923	1881.220586	2549.36421	2882.595467	2184.441112	2499.746373	2715.888068	2202.900556	2807.209115	2824.718994	2192.191363	2897.797193
**ENSG00000161551**	ZNF577	144.8417656	248.727489	101.7791524	178.3307681	188.3935986	136.5774312	297.2258096	385.4245316	282.7843462	273.3599026	435.9549848	271.5098946
**ENSG00000161558**	TMEM143	54.84299863	28.02563256	43.15436063	42.6989163	65.87997005	56.52788124	79.66877372	121.2758224	75.03398903	90.48718999	103.4703287	66.82019213
**ENSG00000161609**	CCDC155	1.406230734	17.51602035	16.28466439	4.186168264	9.246311586	1.517527013	2.04278907	11.62918845	2.344812157	1.265555105	4.138813147	2.53747565
**ENSG00000161618**	ALDH16A1	88.59253625	73.56728548	74.90945619	102.1425056	98.2420606	99.01863761	129.7171059	64.79119281	174.9229869	143.0077268	131.0624163	124.3363069
**ENSG00000161638**	ITGA5	250.3090707	1012.425976	433.1720727	358.3360034	985.8879729	505.7158772	43.919965	265.8100218	76.90983876	59.48108992	315.9294035	84.58252168
**ENSG00000161640**	SIGLEC11	2.812461468	0	0	7.535102876	0	2.655672273	0	0	0	0	0	0
**ENSG00000161642**	ZNF385A	42.18692202	38.53524477	35.01202844	94.60740277	76.28207059	62.97737105	229.8137703	224.2772059	324.0530401	246.1504679	326.9662386	396.6920267
**ENSG00000161647**	MPP3	109.6859973	143.6313669	93.63682024	171.6328988	149.0967743	125.9547421	100.0966644	119.6145098	107.3923968	133.5160636	111.747955	99.80737558
**ENSG00000161652**	IZUMO2	0	0	5.699632536	2.511700959	0	4.55258104	0	0	1.406887294	0	0	0
**ENSG00000161653**	NAGS	12.65607661	21.01922442	26.86969624	11.72127114	16.18104528	20.86599643	8.171156279	28.24231481	10.31717349	17.08499391	23.4532745	25.3747565
**ENSG00000161654**	LSM 12.00	783.2705189	749.6856711	710.0113674	591.0869589	527.0397604	568.3138665	521.9326073	541.5879194	556.1894437	415.1020744	394.5668533	491.4244509
**ENSG00000161664**	ASB16	0	24.52242849	1.628466439	0.837233653	5.778944741	0.758763507	1.021394535	0	0	0	2.759208765	0
**ENSG00000161671**	EMC10	547.0237556	574.5254675	723.0390989	679.8337261	819.4543643	885.856394	461.6703298	468.4901634	561.8169929	582.7881257	564.2581924	543.8656144
**ENSG00000161677**	JOSD2	39.37446056	38.53524477	35.01202844	69.49039319	52.01050267	55.01035423	41.87717593	24.91968954	36.11010722	26.5766572	13.79604382	16.91650434
**ENSG00000161681**	SHANK1	0	0	7.328098975	0	0	0.379381753	0	0	0	0	0	3.383300867
**ENSG00000161682**	FAM171A2	2.812461468	7.006408141	3.256932878	6.697869223	17.33683422	6.449489806	3.064183605	0	0	0	0	0
**ENSG00000161692**	DBF4B	288.2773005	224.2050605	322.4363549	408.5700226	329.3998503	381.6580438	322.760673	239.2290196	297.3221815	332.208215	267.6432502	327.3343589
**ENSG00000161714**	PLCD3	1070.141589	669.1119775	867.9726119	1229.059002	1383.479371	1561.155915	977.4745699	787.4621895	1239.936669	966.8841	865.0119477	1037.827541
**ENSG00000161791**	FMNL3	59.06169083	84.07689769	33.383562	33.48934611	45.07576898	46.66395566	16.34231256	13.29050109	18.75849726	31.63887762	15.17564821	15.2248539
**ENSG00000161800**	RACGAP1	4701.029344	3671.357866	4405.001717	5837.193028	5513.113283	5638.371618	4360.333269	4458.963115	4988.353383	5152.074831	4609.258241	6155.915928
**ENSG00000161813**	LARP4	5544.767785	4018.175069	5369.053849	5477.182557	4742.202055	5596.260243	4374.632793	3198.026825	4629.128161	4322.50346	3248.96832	5081.717902
**ENSG00000161835**	GRASP	0	0	0	4.186168264	0	0.379381753	1.021394535	3.322625272	2.344812157	0	0	0
**ENSG00000161847**	RAVER1	247.4966092	192.6762239	209.2579374	284.659442	211.5093775	258.358974	253.3058446	230.9224564	288.8808578	277.7893455	249.7083932	279.1223215

**ENSG00000161860**	SYCE2	11.24984587	7.006408141	8.956565414	7.535102876	16.18104528	8.346398573	6.128367209	24.91968954	24.38604644	11.38999594	46.906549	11.84155303
**ENSG00000161888**	SPC24	520.3053716	308.2819582	468.1841012	812.9538769	769.7554395	721.204713	689.441311	691.1060566	805.2084948	902.9735672	898.1224529	866.125022
**ENSG00000161896**	IP6K3	0	0	0	1.674467306	0	0	0	4.983937908	0.468962431	5.694997971	1.379604382	0
**ENSG00000161904**	LEMD2	714.365213	882.8074257	886.699976	731.7422126	784.7806959	931.7615861	744.5966159	745.9293736	793.484434	741.6152914	709.1166525	846.671042
**ENSG00000161912**	ADCY10P1	14.06230734	14.01281628	23.61276336	30.97764516	15.02525633	12.51959786	19.40649616	31.56494009	15.00679781	15.81943881	22.07367012	26.22058172
**ENSG00000161914**	ZNF653	39.37446056	94.5865099	52.92515926	42.6989163	69.3473369	45.14642864	32.68462512	48.17806645	37.51699452	44.29442867	48.28615338	34.67883389
**ENSG00000161920**	MED11	156.0916115	115.6057343	127.0203822	154.8882258	168.7451864	142.2681575	102.1394535	141.2115741	130.8405184	132.883286	144.8584601	134.4862095
**ENSG00000161921**	CXCL16	933.7372075	1327.714343	1602.410976	766.0687924	928.0985255	1202.260776	628.1576389	659.5411165	820.2152926	434.7181785	365.5951613	492.2702762
**ENSG00000161940**	BCL6B	5.624922937	7.006408141	0	6.697869223	6.93473369	1.13814526	0	1.661312636	0	0	0	0
**ENSG00000161956**	SENP3	146.2479964	66.56087734	131.0915483	164.097796	110.955739	162.7547722	130.7385005	89.71088235	136.4680676	125.9227329	82.77626294	110.8031034
**ENSG00000161958**	FGF11	2.812461468	0	5.699632536	6.697869223	16.18104528	9.863925586	19.40649616	14.95181373	12.19302322	13.2883286	23.4532745	14.37902869
**ENSG00000161960**	EIF4A1	147.6542271	98.08971397	97.70798633	138.1435527	56.63365846	96.74234709	109.2892152	84.72694444	98.48211061	111.3688492	80.01705417	107.4198025
**ENSG00000161970**	RPL26	863.4256708	599.047896	693.726703	823.0006808	750.1070274	632.4293828	621.0078772	588.1046732	665.4576902	638.4725504	642.8956422	706.264056
**ENSG00000161980**	POLR3K	237.6529941	210.1922442	258.1119306	276.2871054	251.9619907	369.5178277	186.9151999	89.71088235	205.8745074	204.3871494	125.5439988	224.9895077
**ENSG00000161981**	SNRNP25	372.6511446	350.320407	369.6618816	536.6667715	525.8839715	530.7550729	265.5625791	196.0348911	261.2120743	261.9699067	314.5497992	361.1673676
**ENSG00000161996**	WDR90	383.9009904	371.3396315	295.5666587	399.3604524	406.8377098	287.571369	453.4991735	450.2157244	456.7694082	562.5392441	482.8615338	362.859018
**ENSG00000161999**	JMJD8	333.276684	269.7467134	297.1951251	400.1976861	340.9577397	433.2539623	310.5039386	323.955964	373.2940954	366.3782028	353.1787219	325.6427085
**ENSG00000162004**	CCDC78	22.49969175	0	5.699632536	1.674467306	8.090522638	6.449489806	0	0	0	0.632777552	0	0.845825217
**ENSG00000162032**	SPSB3	157.4978422	224.2050605	187.2736405	171.6328988	228.8462118	219.2826534	205.3003015	204.3414542	195.5573339	169.584384	191.7650091	178.4691207
**ENSG00000162062**	TEDC2	130.7794583	108.5993262	117.2495836	156.5626931	137.5388848	154.4083736	119.5031606	114.6305719	128.4957062	119.5949574	111.747955	122.6446564
**ENSG00000162063**	CCNF	911.2375157	697.13761	951.8386335	1375.574892	1080.662667	1373.741329	895.7630071	626.3148638	1091.74454	999.1557552	842.9382776	1233.213166
**ENSG00000162065**	TBC1D24	226.4031482	301.2755501	311.0370898	290.5200775	287.7914481	323.9920173	154.2305748	136.2276362	153.3507151	146.1716146	135.2012295	171.702519
**ENSG00000162066**	AMDHD2	143.4355349	143.6313669	133.534248	133.1201508	166.4336086	165.4104444	87.83993	121.2758224	137.4059924	143.0077268	148.9972733	92.19494863
**ENSG00000162068**	NTN3	7.031153671	0	0.814233219	0.837233653	0	0.379381753	1.021394535	0	0	3.796665314	1.379604382	0
**ENSG00000162069**	BICDL2	146.2479964	157.6441832	153.8900785	137.3063191	108.6441611	144.1650663	99.07526988	81.40431917	103.1717349	115.1655145	95.19270238	89.65747298
**ENSG00000162073**	PAQR4	348.7452221	413.3780803	341.9779522	296.3807131	426.4861219	320.5775815	248.198872	257.5034586	242.4535771	209.4493698	208.3202617	177.6232955
**ENSG00000162076**	FLYWCH2	102.6548436	105.0961221	87.9371877	129.7712162	128.2925733	123.6784516	105.2036371	169.4538889	124.7440068	132.2505084	161.4137127	121.7988312
**ENSG00000162078**	ZG16B	71.71776744	161.1473872	71.65252331	31.81487881	40.45261319	45.5258104	0	0	0.468962431	3.163887762	0	1.691650434
**ENSG00000162086**	ZNF75A	448.5876042	399.365264	405.4881433	318.1487881	291.258815	300.0909669	491.2907713	519.9908551	453.9556336	425.2265152	446.9918199	474.5079466
**ENSG00000162104**	ADCY9	499.2119106	294.2691419	398.1600443	579.3656878	525.8839715	617.2541126	454.520568	400.3763453	568.8514294	424.5937376	491.1391601	473.6621214
**ENSG00000162105**	SHANK2	15.46853808	7.006408141	23.61276336	13.39573845	3.467366845	10.62268909	0	0	0.937924863	0	0	1.691650434
**ENSG00000162129**	CLPB	1044.829435	753.1888751	1077.230549	1247.478143	1026.340586	1224.264918	857.9714093	694.4286819	872.7390849	848.5546977	732.569927	925.3327872
**ENSG00000162139**	NEU3	212.3408409	87.58010176	303.7089909	334.8934611	268.143036	368.0003007	210.4072742	107.9853213	271.9982102	258.8060189	198.6630311	283.3514476
**ENSG00000162144**	CYB561A3	265.7776088	238.2178768	241.8272662	248.6583949	238.0925233	283.3981697	259.4342119	332.2625272	293.5704821	258.1732414	303.5129641	283.3514476
**ENSG00000162148**	PPP1R32	8.437384405	7.006408141	18.72736405	11.72127114	8.090522638	6.449489806	24.51346884	14.95181373	14.53783538	12.65555105	15.17564821	10.1499026
**ENSG00000162174**	ASRGL1	57.6554601	42.03844885	48.85399317	59.44358935	46.23155793	72.08253313	7.149761744	13.29050109	10.78613592	13.92110615	19.31446135	19.45397999
**ENSG00000162191**	UBXN1	568.1172166	437.9005088	575.6628861	547.550809	607.9449868	567.9344847	487.2051931	619.6696133	536.961984	463.1931683	622.2015764	498.1910527
**ENSG00000162194**	LBHD1	98.43615139	101.592918	97.70798633	65.30422492	98.2420606	62.97737105	89.88271907	96.3561329	64.24785311	70.87108587	45.52694462	49.05786257
**ENSG00000162222**	TTC9C	248.9028399	213.6954483	314.2940227	203.4477776	205.7304328	252.6682477	266.5839736	239.2290196	287.005008	259.4387965	238.6715581	324.7968832
**ENSG00000162227**	TAF6L	108.2797665	77.07048955	122.1349829	128.0967489	109.7999501	105.4681274	93.96829721	74.75906863	117.7095703	116.4310696	151.7564821	71.89514343
**ENSG00000162231**	NXF1	760.7708272	630.5767327	818.3043855	709.136904	720.0565148	772.4212497	761.960323	946.9482026	853.0426628	787.8080527	844.317882	794.2298786
**ENSG00000162236**	STX5	701.7091363	697.13761	876.1149441	694.9039319	717.7449369	732.5861656	598.5371974	664.5250545	664.9887278	572.0309074	645.6548509	636.9063882
**ENSG00000162241**	SLC25A45	61.8741523	164.6505913	100.9649192	69.49039319	72.81470374	66.77118858	117.4603715	101.3400708	89.10286198	101.2444084	117.2663725	65.12854169
**ENSG00000162244**	RPL29	4109.006205	3983.143028	3613.567028	3906.532224	3557.518383	3660.275156	3685.191482	4352.639107	4269.902938	3629.61204	4274.014376	4164.843367
**ENSG00000162298**	SYVN1	850.7695942	728.6664466	902.9846404	567.6444166	619.5028763	727.6542028	532.1465527	388.7471569	592.2995509	486.6059378	415.2609191	464.358044
**ENSG00000162300**	ZFPL1	317.8081459	329.3011826	355.0056837	298.0551804	247.3388349	356.6188481	332.9746184	397.05372	366.7286214	293.6087843	322.8274255	309.5720293
**ENSG00000162302**	RPS6KA4	989.9864368	844.272181	973.0086972	1151.196273	1035.586898	1371.085656	689.441311	558.2010457	876.4907844	715.0386342	640.1364334	721.4889099
**ENSG00000162337**	LRP5	494.9932184	329.3011826	455.1563697	458.8040418	537.4418609	554.2767416	667.9920258	323.955964	892.9044695	688.461977	502.1759952	828.9087124
**ENSG00000162341**	TPCN2	66.09284451	77.07048955	65.13865756	99.63080469	60.10102531	80.04954995	87.83993	89.71088235	86.28908739	62.01220013	93.813098	43.98291127
**ENSG00000162344**	FGF19	2.812461468	0	3.256932878	0	1.155788948	1.517527013	0	0	0	0	0	0
**ENSG00000162366**	PDZK1IP1	0	0	4.071166097	0	1.155788948	2.27629052	0	4.983937908	0.468962431	3.796665314	0	2.53747565
**ENSG00000162367**	TAL1	0	0	0	0	2.311577897	0.379381753	4.08557814	0	0.937924863	6.327775524	6.898021912	3.383300867
**ENSG00000162368**	CMPK1	2452.4664	2424.217217	2065.709678	2351.789331	2509.217807	2300.19157	2480.967325	2425.516449	2537.086754	2419.74136	2244.61633	2749.77778
**ENSG00000162377**	COA7	808.5826721	308.2819582	714.8967667	913.4219153	528.1955494	756.1078343	561.7669942	362.1661547	591.3616261	617.5908911	324.2070298	641.9813395
**ENSG00000162378**	ZYG11B	984.3615139	1075.48365	845.988315	1057.426104	1266.744687	997.3946294	1301.256637	1375.566863	1271.357152	1290.866207	1267.856427	1406.607335
**ENSG00000162384**	C1orf123	177.1850725	182.1666117	186.4594073	282.147741	272.7661918	272.3960989	316.6323058	357.1822168	289.8187826	329.0443272	344.9010956	340.8675624
**ENSG00000162385**	MAGOH	614.5228308	364.3332233	637.5446108	592.7614262	486.5871472	495.4725698	663.9064477	618.0083006	623.2510714	604.9353401	651.1732684	664.8186204
**ENSG00000162390**	ACOT11	136.4043812	189.1730198	141.6765802	224.378619	251.9619907	221.9383257	444.3066227	483.4419771	419.2524137	516.3464827	630.4792027	439.8291127
**ENSG00000162396**	PARS2	68.90530597	91.08330583	142.4908134	97.95633739	93.61890481	95.22482008	134.8240786	189.3896405	155.2265648	134.7816187	155.8952952	134.4862095
**ENSG00000162402**	USP24	3405.890838	3506.707274	3646.95059	3092.741114	3127.564894	3310.485179	2696.481572	2468.710577	2595.238096	2546.929648	2614.350304	2714.253121
**ENSG00000162407**	PLPP3	28.12461468	14.01281628	32.56932878	15.07020575	19.64841212	17.83094241	309.4825441	177.7604521	268.2465108	205.019927	143.4788558	175.9316451
**ENSG00000162408**	NOL9	1690.289342	973.8907316	1468.876728	1888.799121	1451.670919	1657.898262	1369.690071	1038.320398	1348.26699	1310.482311	1132.655198	1384.61588
**ENSG00000162409**	PRKAA2	0	14.01281628	4.885399317	5.86063557	24.27156791	2.655672273	106.2250316	275.7778976	122.3991946	152.4993901	244.1899757	129.4112582
**ENSG00000162413**	KLHL21	1238.889277	1152.554139	1448.520897	842.2570548	683.0712684	925.6914781	418.7717593	343.8917157	508.3552757	427.1248479	338.0030737	434.7541614
**ENSG00000162415**	ZSWIM5	4.218692202	7.006408141	4.071166097	3.348934611	1.155788948	4.55258104	9.192550814	1.661312636	5.158586746	12.65555105	9.657230676	7.612426951
**ENSG00000162419**	GMEB1	677.8032139	718.1568344	546.3504902	624.576305	605.6334089	596.7674979	532.1465527	519.9908551	534.1482094	568.8670196	601.5075107	529.4865857
**ENSG00000162426**	SLC45A1	1.406230734	0	0	3.348934611	2.311577897	0	2.04278907	0	0	1.265555105	0	0

**ENSG00000162430**	SELENON	904.2063621	994.909956	1051.175086	997.1452806	1169.658416	1209.089648	855.9286202	903.7540741	1151.771732	1101.665719	1056.776957	1050.514919
**ENSG00000162433**	AK4	282.6523776	711.1504263	168.5462764	94.60740277	327.0882724	76.63511417	42.89857046	254.1808333	64.24785311	15.18666126	129.6828119	27.91223215
**ENSG00000162434**	JAK1	2974.178003	3737.918743	3590.768498	3290.328256	3332.139538	4047.623926	5399.091511	4870.968649	4727.141309	5342.540875	5363.901838	5208.591685
**ENSG00000162437**	RAVER2	7.031153671	3.50320407	4.071166097	3.348934611	16.18104528	15.55465189	9.192550814	19.93575163	14.06887294	12.0227735	38.6289227	13.53320347
**ENSG00000162438**	CTRC	0	7.006408141	1.628466439	1.674467306	0	0.758763507	0	0	1.406887294	0.632777552	0	0
**ENSG00000162441**	LZIC	987.1739754	1082.490058	865.5299123	1151.196273	1251.719431	1090.722541	1069.400078	1219.403475	1052.351696	1062.43351	1306.48535	1086.885404
**ENSG00000162444**	RBP7	0	3.50320407	7.328098975	10.88403749	10.40210053	4.55258104	2.04278907	1.661312636	0.468962431	0.632777552	1.379604382	3.383300867
**ENSG00000162458**	FBLIM1	0	3.50320407	0.814233219	0.837233653	3.467366845	1.896908767	7.149761744	0	2.813774589	1.898332657	13.79604382	2.53747565
**ENSG00000162461**	SLC25A34	11.24984587	0	15.47043117	5.023401917	13.86946738	11.3814526	22.47067977	19.93575163	23.44812157	18.35054902	23.4532745	20.2998052
**ENSG00000162482**	AKR7A3	15.46853808	0	16.28466439	20.93084132	23.11577897	14.03712487	22.47067977	13.29050109	15.00679781	17.08499391	13.79604382	8.458252168
**ENSG00000162493**	PDPN	25.31215321	80.57369362	94.45105346	30.97764516	41.60840214	87.63718501	1.021394535	13.29050109	2.344812157	1.898332657	0	9.304077385
**ENSG00000162494**	LRRC38	99.84238213	329.3011826	194.6017394	84.56059894	191.8609654	132.4042319	1.021394535	0	0.937924863	0	0	0
**ENSG00000162496**	DHRS3	284.0586083	493.9517739	277.6535278	362.5221717	464.6271572	301.6084939	106.2250316	245.8742701	237.7639527	165.1549412	366.9747657	240.2143616
**ENSG00000162510**	MATN1	4.218692202	0	1.628466439	5.86063557	1.155788948	3.41443578	0	0	1.406887294	4.429442867	0	0.845825217
**ENSG00000162511**	LAPTM5	215.1533023	70.06408141	127.0203822	234.4254228	187.2378096	249.2538119	4.08557814	11.62918845	19.69642212	5.694997971	1.379604382	21.99145564
**ENSG00000162512**	SDC3	555.46114	571.0222635	615.5603139	797.8836712	774.3785953	822.4996412	216.5356414	197.6962037	255.5845251	260.7043516	284.1985028	299.4221267
**ENSG00000162517**	PEF1	527.3365253	497.454978	609.8606814	619.5529031	614.8797205	749.6583445	693.5268892	624.6535512	820.684255	728.3269628	670.4877298	798.4590046
**ENSG00000162520**	SYNC	1.406230734	35.0320407	8.956565414	9.209570182	21.95999002	6.449489806	1.021394535	23.25837691	7.503398903	5.062220419	13.79604382	10.1499026
**ENSG00000162521**	RBBP4	4783.996958	4641.745393	4263.325137	4701.066961	4742.202055	4155.747726	4719.864146	5306.23256	4679.307141	5083.102078	5282.50518	5215.358287
**ENSG00000162522**	KIAA1522	2611.370473	2487.27489	2183.773495	3523.916445	4161.996003	3493.726566	2872.161432	3206.333388	3365.274408	3051.886135	3287.597243	3055.120683
**ENSG00000162526**	TSSK3	39.37446056	38.53524477	39.08319453	44.3733836	21.95999002	26.17734098	62.30506663	36.54887799	54.39964205	55.68442461	63.46180159	54.97863909
**ENSG00000162542**	TMCO4	278.4336854	364.3332233	317.5509556	150.7020575	248.4946239	234.0785418	131.759895	122.9371351	112.5509835	106.3066288	93.813098	82.89087124
**ENSG00000162543**	UBXN10	142.0293041	143.6313669	65.13865756	205.9594786	221.9114781	99.77740112	801.7947099	549.8944826	477.8727177	923.8552265	605.6463238	614.9149326
**ENSG00000162545**	CAMK2N1	4369.158891	4557.668496	3398.609458	4978.1913	5372.107032	4246.419965	4229.594769	4246.315098	3966.953208	4357.939003	4596.841802	4389.832875
**ENSG00000162552**	WNT4	5.624922937	14.01281628	8.956565414	6.697869223	36.98524634	15.17527013	0	3.322625272	14.06887294	6.960553076	5.518417529	13.53320347
**ENSG00000162572**	SCNN1D	30.93707615	28.02563256	18.72736405	15.07020575	35.8294574	29.97115851	20.4278907	39.87150327	21.57227185	14.5538837	19.31446135	7.612426951
**ENSG00000162576**	MXRA8	5.624922937	3.50320407	4.071166097	2.511700959	5.778944741	7.967016819	0	0	0.468962431	1.265555105	0	0.845825217
**ENSG00000162585**	FAAP20	230.6218404	276.7531216	228.7995347	243.634993	277.3893476	302.7466391	206.321696	245.8742701	231.6674411	225.9015862	255.2268107	212.3021294
**ENSG00000162591**	MEGF6	1251.545353	1856.698157	867.1583787	1151.196273	1916.298076	803.9099353	138.9096567	172.7765142	86.75804982	173.3810494	201.4222398	100.6532008
**ENSG00000162594**	IL23R	9.843615139	24.52242849	39.08319453	10.88403749	16.18104528	16.31341539	22.47067977	24.91968954	8.441323766	3.163887762	16.55525259	6.766601734
**ENSG00000162595**	DIRAS3	28.12461468	7.006408141	13.02773151	10.04680383	10.40210053	5.6907263	18.38510163	3.322625272	9.379248629	6.327775524	2.759208765	3.383300867
**ENSG00000162599**	NFIA	2266.843943	1040.451609	964.0521318	2561.934978	2036.500127	1135.110206	5418.498008	6798.091307	4260.52369	5739.2924	8545.269544	4778.06665
**ENSG00000162600**	OMA1	682.0219061	721.6600385	616.3745471	457.9668081	494.6776699	492.8168975	584.2376739	608.0404248	541.1826459	573.92924	493.8983689	485.5036744
**ENSG00000162601**	MYSM1	1898.411491	2080.903218	1669.992333	1710.468353	1577.651914	1523.597121	1748.627444	1991.913851	1539.1347	1802.783247	2008.703981	1685.729657
**ENSG00000162604**	TM2D1	613.1166001	546.499835	543.9077906	546.7135753	440.3555893	458.293158	564.8311778	506.700354	459.1142204	506.2220419	543.5641266	499.0368779
**ENSG00000162607**	USP1	5163.679256	4505.120435	4267.396303	4999.122141	4537.627411	4505.537702	7291.735584	7640.376814	6916.726902	6863.738111	6853.874571	7436.495306
**ENSG00000162613**	FUBP1	4256.660432	3324.540663	4150.960953	4106.631067	3423.446865	3930.394964	4752.548771	4211.427533	4256.77199	4297.825136	3723.552228	4658.805294
**ENSG00000162614**	NEXN	372.6511446	304.7787541	155.5185449	0.837233653	12.71367843	3.41443578	81.7156279	51.50069172	37.98595695	3.163887762	0	0.845825217
**ENSG00000162616**	DNAJB4	419.0567588	585.0350798	392.4604118	427.8263966	899.2038017	523.5468196	648.5855296	493.4098529	664.0508029	573.2964625	475.9635119	707.1098812
**ENSG00000162618**	ADGRL4	0	0	3.256932878	0	0	0	2.04278907	0	4.220661883	0	2.759208765	5.074951301
**ENSG00000162620**	LRRIQ3	2.812461468	38.53524477	8.142332194	10.04680383	9.246311586	7.967016819	41.87717593	11.62918845	17.8205724	21.51443678	24.83287888	18.60815477
**ENSG00000162623**	TYW3	451.4000657	399.365264	448.6425039	547.550809	486.5871472	404.0415673	556.6600215	533.2813562	543.5274581	622.020334	480.102325	706.264056
**ENSG00000162627**	SNX7	410.6193744	700.6408141	381.0611467	303.915816	478.4966246	300.4703486	934.5759994	941.9642647	812.2429313	736.553071	789.1337067	719.7972595
**ENSG00000162630**	B3GALT2	0	10.50961221	0	0	2.311577897	0	1.021394535	0	0	2.53111021	0	0.845825217
**ENSG00000162631**	NTNG1	4.218692202	3.50320407	0	0	1.155788948	3.41443578	0	0	0	0	0	0
**ENSG00000162636**	FAM102B	94.21745919	101.592918	83.05178838	111.3520758	204.5746438	163.1341539	77.62598465	74.75906863	78.78568848	126.5555105	102.0907243	124.3363069
**ENSG00000162639**	HENMT1	305.1520693	185.6698157	273.5823617	376.7551438	346.7366845	297.8146763	262.4983955	305.681525	252.3017881	299.9365598	281.439294	296.0388259
**ENSG00000162641**	AKNAD1	26.71838395	21.01922442	22.79853014	28.4659442	30.05051265	18.96908767	12.25673442	19.93575163	4.220661883	8.858885733	13.79604382	17.76232955
**ENSG00000162642**	C1orf52	382.4947597	378.3460396	452.71367	401.8721534	345.5808955	536.0664174	377.9159779	357.1822168	402.8387286	387.8926396	314.5497992	407.6877545
**ENSG00000162643**	WDR63	0	0	0	0.837233653	0	0.379381753	5.106972674	0	0.468962431	0	4.138813147	0
**ENSG00000162645**	GBP2	2444.029016	3075.813174	2173.188463	10.88403749	17.33683422	15.93403364	7931.128563	6303.020141	5904.705974	1416.156162	1189.218978	1138.480742
**ENSG00000162650**	ATXN7L2	43.59315276	14.01281628	47.22552673	92.93293547	54.32208057	54.25159072	55.15530488	33.22625272	39.86180667	69.60553076	77.25784541	57.51611474
**ENSG00000162654**	GBP4	8568.163863	11049.10564	8604.816663	3.348934611	73.97049269	27.31548624	6127.345815	4877.6139	5413.233346	85.42496957	62.0821972	45.67456171
**ENSG00000162664**	ZNF326	1691.695573	1579.945036	1446.892431	1706.282185	1692.07502	1462.896041	1906.943597	2033.446667	1798.939887	1735.708826	1919.029696	1920.023242
**ENSG00000162676**	GFI1	0	0	5.699632536	7.535102876	9.246311586	7.587635066	0	11.62918845	5.158586746	6.327775524	5.518417529	5.074951301
**ENSG00000162685**	LSP1P3	4.218692202	0	0	0.837233653	4.623155793	1.517527013	12.25673442	1.661312636	12.66198565	13.2883286	11.03683506	8.458252168
**ENSG00000162687**	KCNT2	0	0	1.628466439	1.674467306	1.155788948	1.517527013	35.74880872	61.46856754	78.31672605	41.76331846	35.86971394	71.04931821
**ENSG00000162688**	AGL	1091.23505	791.7241199	931.482803	1147.847338	1302.574145	1037.609095	1595.418263	1727.765142	1510.059029	1529.423344	1767.273214	1685.729657
**ENSG00000162694**	EXTL2	316.4019152	378.3460396	314.2940227	410.2444899	375.6314082	328.9239801	331.9532238	265.8100218	290.7567075	301.2021149	318.6886123	326.4885337
**ENSG00000162695**	SLC30A7	2030.59718	1551.919403	1980.21519	1162.08031	1021.71743	1191.638087	1587.247107	1347.324548	1541.479512	1165.576251	1066.434188	1226.446564
**ENSG00000162702**	ZNF281	2157.157946	1923.259035	2100.721706	1749.818334	1256.342587	1700.7684	1288.999903	1015.062021	1351.080765	1037.122408	994.6947596	1117.335111
**ENSG00000162704**	ARPC5	3948.695902	3758.937968	3693.361883	3956.766243	3962.044515	3873.487701	4643.259556	4995.567097	5087.773419	4374.39122	4285.051211	5157.842172
**ENSG00000162706**	CADM3	1.406230734	10.50961221	8.142332194	0	0	0	0	0	0.468962431	0	0	1.691650434
**ENSG00000162711**	NLRP3	539.9926019	479.9389576	473.8837337	207.6339459	141.0062517	261.0146463	103.160848	209.3253922	60.49615366	31.00610007	60.70259282	11.84155303
**ENSG00000162714**	ZNF496	641.2412148	616.5639164	673.3708725	632.9486416	605.6334089	630.532474	785.4523973	867.2051961	746.1192284	716.9369668	689.8021912	623.3731848
**ENSG00000162722**	TRIM58	1233.264354	893.317038	1054.432019	1358.830219	1407.750939	1490.590909	10.21394535	18.274439	34.70321993	13.2883286	6.898021912	16.07067912
**ENSG00000162723**	SLAMF9	0	0	2.442699658	0	1.155788948	3.035054026	0	0	0.468962431	0	0	1.691650434

**ENSG00000162728**	KCNJ9	0	0	0.814233219	0	0	3.035054026	3.064183605	0	0	1.265555105	0	1.691650434
**ENSG00000162729**	IGSF8	236.2467633	311.7851623	258.9261638	189.2148055	203.4188549	255.32392	221.6426141	196.0348911	270.1223605	244.8849128	160.0341083	290.1180494
**ENSG00000162733**	DDR2	172.9663803	185.6698157	229.6137679	87.0722999	139.8504627	146.061975	437.1568609	614.6856754	563.2238802	297.4054496	394.5668533	313.8011554
**ENSG00000162734**	PEA15	2879.960544	2546.829359	3446.649218	4128.399142	3802.54564	5029.084522	2192.934066	2629.857903	2738.7406	1985.023182	2189.432155	2748.086129
**ENSG00000162735**	PEX19	402.18199	441.4037129	462.4844686	529.1316686	501.6124035	554.2767416	510.6972674	498.3937908	520.5482989	481.5437174	466.3062812	568.3945457
**ENSG00000162736**	NCSTN	1175.608894	1362.746383	1330.457081	1150.359039	1338.403602	1436.7187	1604.610814	1922.13872	1672.788993	1699.007728	1765.893609	1651.050823
**ENSG00000162738**	VANGL2	0	0	15.47043117	6.697869223	1.155788948	1.517527013	20.4278907	3.322625272	9.848211061	5.694997971	13.79604382	10.99572782
**ENSG00000162739**	SLAMF6	1.406230734	3.50320407	0	0	2.311577897	0.758763507	99.07526988	84.72694444	87.22701225	110.7360717	71.73942788	62.59106604
**ENSG00000162746**	FCRLB	1.406230734	3.50320407	6.513865756	12.55850479	10.40210053	12.51959786	39.83438686	109.646634	23.44812157	27.8422123	73.11903226	20.2998052
**ENSG00000162747**	FCGR3B	1.406230734	0	4.071166097	3.348934611	0	1.517527013	12.25673442	23.25837691	22.97915914	2.53111021	5.518417529	9.304077385
**ENSG00000162753**	SLC9C2	1.406230734	3.50320407	7.328098975	0.837233653	9.246311586	4.55258104	4.08557814	0	2.344812157	1.898332657	6.898021912	0
**ENSG00000162755**	KLHDC9	0	10.50961221	0	0.837233653	0	6.828871559	1.021394535	0	1.406887294	0	0	2.53747565
**ENSG00000162757**	C1orf74	1390.762196	3075.813174	1923.218864	1000.494215	1211.266818	1026.607024	458.6061462	315.6494009	409.8731651	354.3554293	378.0116008	415.3001814
**ENSG00000162769**	FLVCR1	184.2162262	234.7146727	181.5740079	174.9818334	131.7599401	180.5857146	313.5681222	274.116585	318.425491	322.0837742	293.8557334	315.4928059
**ENSG00000162771**	FAM71A	0	0	3.256932878	0.837233653	2.311577897	1.896908767	0	3.322625272	0.468962431	0.632777552	1.379604382	0
**ENSG00000162772**	ATF3	992.7988983	1502.874546	1310.10125	449.5944716	750.1070274	574.7633563	1373.775649	1872.299341	1336.54293	775.1525017	1211.292648	926.1786124
**ENSG00000162775**	RBM15	1157.327894	1103.509282	1266.946889	1243.291975	1137.296325	1225.782445	1501.449966	1581.56963	1590.720567	1574.35055	1666.562094	1648.513347
**ENSG00000162777**	DENND2D	403.5882207	332.8043867	394.0888782	334.8934611	469.250313	347.8930678	1238.951571	1056.594837	1007.331303	983.969094	855.354717	899.1122054
**ENSG00000162779**	AXDND1	2.812461468	14.01281628	9.770798633	2.511700959	4.623155793	2.27629052	10.21394535	14.95181373	6.56547404	6.327775524	2.759208765	8.458252168
**ENSG00000162783**	IER5	2881.366774	2995.23948	2871.800565	2386.115911	2241.074771	2560.068071	1258.358067	1212.758224	1259.633091	1117.485158	1058.156561	1262.817049
**ENSG00000162804**	SNED1	0	0	1.628466439	7.535102876	4.623155793	3.793817533	21.44928523	16.61312636	14.06887294	48.09109398	33.11050518	14.37902869
**ENSG00000162813**	BPNT1	490.7745262	395.86206	509.7099954	363.3594053	305.1282823	391.9013512	364.637849	363.8274673	386.8940059	345.4965436	335.2438649	315.4928059
**ENSG00000162814**	SPATA17	22.49969175	38.53524477	36.64049488	47.72231821	71.65891479	32.62683078	43.919965	48.17806645	30.95152048	29.10776741	41.38813147	31.29553302
**ENSG00000162817**	C1orf115	195.466072	234.7146727	143.3050466	221.866918	234.6251565	172.6186978	198.1505398	289.0683987	173.5160996	202.4888168	285.5781071	228.3728085
**ENSG00000162819**	BROX	3635.106448	3695.880294	3355.455097	2954.597561	3105.604904	3032.018972	2902.803268	2746.149788	2825.967612	2698.796261	2614.350304	2892.722241
**ENSG00000162825**	NBPF20	129.3732275	147.134571	184.8309408	171.6328988	175.6799201	196.1403665	147.080813	189.3896405	158.5093018	160.0927208	229.0143275	178.4691207
**ENSG00000162836**	ACP6	230.6218404	182.1666117	236.1276336	221.0296844	183.7704428	224.593998	517.8470292	325.6172767	433.7902491	525.2053685	513.2128302	432.2166858
**ENSG00000162840**	MT2P1	1.406230734	0	0.814233219	0	2.311577897	1.13814526	0	4.983937908	2.813774589	0	0	0
**ENSG00000162843**	WDR64	23.90592248	38.53524477	26.05546302	5.86063557	4.623155793	10.24330734	1.021394535	4.983937908	4.689624315	0	0	2.53747565
**ENSG00000162851**	TFB2M	423.275451	168.1537954	284.9816268	380.1040784	269.2988249	372.5528817	262.4983955	199.3575163	319.3634158	309.4282231	292.476129	285.8889233
**ENSG00000162852**	CNST	577.9608317	791.7241199	613.1176142	717.5092405	587.1407857	692.7510815	538.2749199	478.4580392	562.7549177	529.0020338	444.2326111	589.5401761
**ENSG00000162869**	PPP1R21	568.1172166	679.6215897	565.0778543	431.1753312	502.7681925	481.0560632	589.3446466	559.8623584	535.0861343	605.5681176	590.4706756	457.5914423
**ENSG00000162882**	HAAO	0	0	2.442699658	2.511700959	6.93473369	0	0	0	0	0	0	0
**ENSG00000162885**	B3GALNT2	1586.228268	1047.458017	1336.156713	1315.294069	998.6016513	1241.337097	950.918312	699.4126198	933.704201	966.2513225	711.8758613	1079.272977
**ENSG00000162889**	MAPKAPK2	950.6119763	959.8779153	1079.673249	883.2815038	910.7616912	1110.829774	673.0989985	677.8155555	740.9606417	718.202522	692.5613999	696.1141534
**ENSG00000162891**	IL20	14.06230734	7.006408141	32.56932878	12.55850479	19.64841212	60.70108053	5.106972674	1.661312636	5.158586746	0	0	7.612426951
**ENSG00000162892**	IL24	1324.669352	2238.547401	3408.380257	1016.401655	1486.344587	2995.218942	39.83438686	19.93575163	122.3991946	39.23220825	41.38813147	98.11572515
**ENSG00000162894**	FCMR	74.53022891	101.592918	95.26528668	62.79252397	98.2420606	88.01656677	4.08557814	8.306563181	6.096511609	6.960553076	12.41643944	6.766601734
**ENSG00000162896**	PIGR	168.7476881	262.7403053	236.9418669	21.76807497	31.2063016	37.17941182	1.021394535	14.95181373	2.813774589	2.53111021	0	0.845825217
**ENSG00000162897**	FCAMR	4.218692202	0	1.628466439	0	0	1.896908767	0	1.661312636	0	0	0	0
**ENSG00000162909**	CAPN2	8592.069786	7332.206119	8742.422077	9228.826556	8883.393856	9796.016252	3444.142372	4718.127887	3646.182905	3954.226925	4501.649099	4160.614241
**ENSG00000162910**	MRPL55	143.4355349	45.54165292	144.9335131	123.073347	99.39784955	156.6846641	102.1394535	96.3561329	98.48211061	130.3521758	104.8499331	111.6489286
**ENSG00000162923**	WDR26	2422.935555	2620.396645	2372.675601	2643.983876	2553.137787	2506.954626	2976.343675	3254.511454	3007.456073	3049.987802	3291.736056	3150.698932
**ENSG00000162924**	REL	444.368912	711.1504263	404.6739101	352.4753679	487.7429362	374.0704088	410.600603	408.6829085	419.7213762	386.6270845	411.1221059	548.0947405
**ENSG00000162927**	PUS10	149.0604578	178.6634076	106.6645517	121.3988797	147.9409854	96.36296534	146.0594185	78.0816939	127.5577814	145.538837	126.9236032	127.7196077
**ENSG00000162928**	PEX13	607.4916772	700.6408141	649.7581091	617.8784358	632.2165547	652.5366157	690.4627056	767.5264379	691.250624	734.6547383	641.5160378	740.9428899
**ENSG00000162929**	KIAA1841	386.7134519	571.0222635	333.0213868	393.4998168	545.5323836	403.6621855	363.6164544	583.1207353	393.9284424	405.6104111	571.1562143	469.4329953
**ENSG00000162931**	TRIM17	67.49907524	56.05126513	56.99632536	4.186168264	1.155788948	5.6907263	3.064183605	0	1.875849726	0	0	2.53747565
**ENSG00000162944**	RFTN2	0	7.006408141	3.256932878	1.674467306	0	1.13814526	3.064183605	0	2.344812157	3.796665314	0	1.691650434
**ENSG00000162946**	DISC1	0	0	2.442699658	3.348934611	1.155788948	1.896908767	0	3.322625272	0.468962431	0	1.379604382	0
**ENSG00000162959**	MEMO1	84.37384405	98.08971397	74.90945619	120.561646	75.12628164	118.367107	137.8882622	107.9853213	118.6474952	138.578284	115.8867681	109.9572782
**ENSG00000162961**	DPY30	559.6798322	441.4037129	552.864356	551.7369772	525.8839715	555.7942686	488.2265877	407.0215959	446.4522347	467.6226112	493.8983689	531.1782361
**ENSG00000162971**	TYW5	355.7763757	234.7146727	258.9261638	225.2158526	205.7304328	205.6249103	294.161626	227.5998311	310.4531296	249.3143556	291.0965247	288.4263989
**ENSG00000162972**	MAIP1	88.59253625	59.5544692	105.8503185	113.0265431	91.30732691	128.6104144	120.5245551	106.3240087	130.3715559	99.97885328	125.5439988	101.499026
**ENSG00000162975**	KCNF1	0	0	1.628466439	0	0	2.655672273	0	0	0	1.898332657	0	0
**ENSG00000162976**	PQLC3	132.185689	119.1089384	102.5933857	102.9797393	169.9009754	139.991867	144.0166294	98.01744553	80.19257578	114.532737	122.78479	128.5654329
**ENSG00000162980**	ARL5A	357.1826065	259.2371012	215.7718032	319.8232554	342.1135287	340.6848145	390.1727123	380.4405937	385.0181562	432.8198458	401.4648753	411.9168806
**ENSG00000162981**	FAM84A	271.4025317	371.3396315	287.4243265	353.3126015	442.6671672	340.6848145	846.7360694	1001.77152	606.8373863	775.1525017	1109.201923	577.6986231
**ENSG00000162994**	CLHC1	66.09284451	101.592918	88.75142092	86.23506625	64.7241811	81.56707696	105.2036371	119.6145098	77.84776362	113.2671819	106.2295374	104.0365017
**ENSG00000162997**	PRORSD1P	16.87476881	49.04485699	30.94086234	21.76807497	24.27156791	22.38352344	29.62044151	16.61312636	28.13774589	25.3111021	27.59208765	30.4497078
**ENSG00000162998**	FRZB	0	0	0	0.837233653	6.93473369	1.517527013	2.04278907	0	0.937924863	0	0	0.845825217
**ENSG00000162999**	DUSP19	57.6554601	56.05126513	38.26896131	49.39678552	31.2063016	51.9753002	83.75435186	64.79119281	45.48935585	75.93330629	60.70259282	81.19922081
**ENSG00000163001**	CFAP36	196.8723028	322.2947745	198.6729055	295.5434795	318.9977497	267.0847543	333.9960129	465.1675381	337.1839882	360.6832049	437.3345892	437.2916371
**ENSG00000163002**	NUP35	341.7140684	157.6441832	330.5786871	396.0115178	334.023006	351.6868853	340.1243801	224.2772059	364.8527717	419.5315172	281.439294	380.6213475
**ENSG00000163006**	CCDC138	233.4343019	154.1409791	195.4159727	303.0785823	251.9619907	256.841447	219.599825	299.0362745	208.2193196	236.6588046	303.5129641	241.906012
**ENSG00000163013**	FBXO41	203.9034565	259.2371012	217.4002696	256.1934978	320.1535387	295.9177676	267.6053681	277.4392102	277.6257594	312.5921109	231.7735362	271.5098946
**ENSG00000163016**	ALMS1P1	2.812461468	21.01922442	2.442699658	1.674467306	0	0	2.04278907	1.661312636	2.344812157	0	2.759208765	0

**ENSG00000163017**	ACTG2	0	0	0.814233219	0	0	2.27629052	32.68462512	21.59706427	20.63434698	28.47498986	24.83287888	21.14563042
**ENSG00000163026**	WDCP	320.6206074	353.8236111	327.3217542	364.196639	266.987247	330.8208889	233.8993485	249.1968954	265.9016986	285.3826761	245.5695801	287.5805737
**ENSG00000163029**	SMC6	2020.753565	2532.816543	2041.282681	1739.771531	1916.298076	1711.770471	1758.841389	2021.817478	1709.368063	1727.482718	1959.038223	1836.286546
**ENSG00000163032**	VSNL1	7.031153671	3.50320407	5.699632536	0.837233653	0	0.758763507	0	0	1.406887294	0	0	0.845825217
**ENSG00000163040**	CCDC74A	9.843615139	24.52242849	7.328098975	20.09360767	26.58314581	40.97322936	28.59904698	16.61312636	16.4136851	13.92110615	19.31446135	23.68310607
**ENSG00000163041**	H3F3A	1261.388969	1156.057343	1280.788854	1488.601435	1459.761442	1566.087878	1313.513372	1352.308486	1571.024145	1582.576659	1560.332556	1659.509075
**ENSG00000163050**	COQ8A	12.65607661	0	4.071166097	1.674467306	8.090522638	2.655672273	15.32091802	1.661312636	8.910286198	6.960553076	9.657230676	3.383300867
**ENSG00000163053**	SLC16A14	4.218692202	7.006408141	3.256932878	7.535102876	8.090522638	6.828871559	7.149761744	0	3.751699452	0	1.379604382	9.304077385
**ENSG00000163069**	SGCB	224.9969175	378.3460396	200.301372	217.6807497	262.3640913	215.1094541	253.3058446	285.7457734	203.5296953	234.1276944	286.9577115	227.5269833
**ENSG00000163071**	SPATA18	5.624922937	0	5.699632536	0.837233653	8.090522638	4.55258104	7.149761744	14.95181373	4.689624315	10.75721839	9.657230676	4.229126084
**ENSG00000163082**	SGPP2	9.843615139	17.51602035	20.35583049	6.697869223	9.246311586	8.725780326	2.04278907	19.93575163	7.034436472	5.694997971	2.759208765	4.229126084
**ENSG00000163083**	INHBB	88.59253625	84.07689769	155.5185449	153.2137585	166.4336086	241.2867951	38.81299233	146.195512	34.70321993	50.62220419	115.8867681	49.05786257
**ENSG00000163093**	BBS5	0	3.50320407	0	5.023401917	3.467366845	0.758763507	15.32091802	6.645250545	8.910286198	5.694997971	2.759208765	5.074951301
**ENSG00000163104**	SMARCAD1	1797.162878	1492.364934	1535.643852	1650.18753	1484.03301	1446.203244	1192.988817	868.8665087	1098.778977	1168.107362	975.3802983	1248.43802
**ENSG00000163110**	PDLIM5	9444.245611	7931.254015	9016.818672	8846.210776	8109.015261	9811.191522	3913.983858	4448.99524	4366.509199	3525.836522	4078.110554	4160.614241
**ENSG00000163116**	STPG2	36.56199909	10.50961221	17.91313083	20.09360767	39.29682424	22.38352344	2.04278907	3.322625272	6.096511609	0.632777552	6.898021912	4.229126084
**ENSG00000163121**	NEURL3	28.12461468	122.6121425	39.89742775	3.348934611	0	3.793817533	3.064183605	13.29050109	7.034436472	2.53111021	4.138813147	2.53747565
**ENSG00000163125**	RPRD2	1731.070034	1625.486689	1696.047796	1731.399194	1715.190799	1777.782896	1818.082272	1551.666002	1875.849726	1714.827167	1557.573348	1876.040331
**ENSG00000163126**	ANKRD23	19.68723028	35.0320407	19.54159727	18.41914036	28.89472371	17.83094241	21.44928523	16.61312636	12.19302322	18.98332657	15.17564821	12.68737825
**ENSG00000163131**	CTSS	2705.587933	4960.536964	4146.889787	25.11700959	49.69892478	79.67016819	345.2313528	458.5222876	342.342575	25.94387965	44.14734023	31.29553302
**ENSG00000163132**	MSX1	0	14.01281628	4.885399317	6.697869223	17.33683422	2.655672273	0	0	0	0	1.379604382	0
**ENSG00000163138**	PACRGL	275.6212239	325.7979785	258.9261638	391.8253495	354.8272071	322.8538721	445.3280172	438.5865359	394.3974049	465.7242786	427.6773585	456.7456171
**ENSG00000163141**	BNIPL	12.65607661	21.01922442	15.47043117	15.9074394	12.71367843	7.967016819	13.27812895	11.62918845	22.04123428	18.35054902	24.83287888	25.3747565
**ENSG00000163145**	C1QTNF7	0	0	0	0	0	0	4.08557814	3.322625272	0	0	15.17564821	2.53747565
**ENSG00000163155**	LYSMD1	151.8729193	161.1473872	153.0758453	135.6318518	136.3830959	150.9939378	146.0594185	179.4217647	133.1853305	129.7193982	114.5071637	140.406986
**ENSG00000163156**	SCNM1	459.8374501	448.410121	574.8486529	457.9668081	418.3955993	476.1241004	470.8628806	416.9894717	482.0933795	413.2037417	411.1221059	460.1289179
**ENSG00000163157**	TMOD4	26.71838395	38.53524477	29.3123959	30.1404115	24.27156791	21.62475994	34.72741419	19.93575163	24.85500887	34.80276538	22.07367012	26.22058172
**ENSG00000163159**	VPS72	815.6138258	658.6023652	925.7831705	932.6782893	831.0122538	900.6522823	706.8050181	529.9587309	640.1337189	570.7653522	626.3403896	661.4353195
**ENSG00000163161**	ERCC3	773.4269038	592.0414879	829.7036506	735.0911472	723.5238816	844.1244011	635.3074007	566.5076089	620.4372968	665.0492076	535.2865003	645.3646404
**ENSG00000163162**	RNF149	1342.950351	1450.326485	1496.560657	704.9507357	788.2480627	779.2501213	1246.101333	1245.984477	1202.419674	815.0174875	827.7626294	814.5296838
**ENSG00000163166**	IWS1	1468.104886	1156.057343	1467.248261	1224.0356	1162.723682	1241.337097	960.1108628	1104.772903	856.7943623	1014.975194	1125.757176	976.0823002
**ENSG00000163170**	BOLA3	147.6542271	199.682632	128.6488487	133.1201508	131.7599401	137.3361947	137.8882622	83.06563181	110.6751338	101.2444084	78.63744979	82.04504603
**ENSG00000163171**	CDC42EP3	1844.974723	1219.115017	1377.682607	2371.045705	1978.710679	2126.434727	3132.617038	3076.751002	2506.604196	3134.147217	2658.497645	2618.674871
**ENSG00000163191**	S100A11	12207.489	11798.79131	11309.69942	11458.37977	11581.00526	11316.9577	6398.015366	6676.815485	7142.297831	6379.030506	6161.313171	6870.638236
**ENSG00000163202**	LCE3D	7.031153671	14.01281628	10.58503185	8.372336529	11.55788948	3.41443578	0	0	0	0	0	0
**ENSG00000163207**	IVL	68.90530597	154.1409791	87.9371877	210.9828805	191.8609654	269.7404266	137.8882622	88.04956971	114.4268333	192.3643759	99.33151553	187.7731981
**ENSG00000163209**	SPRR3	4191.973819	5310.857371	4938.324476	7725.992149	9858.879729	9264.502416	104.1822426	112.9692593	256.0534876	110.1032941	157.2748996	438.1374623
**ENSG00000163214**	DHX57	655.3035221	504.4613861	752.3514948	741.7890164	700.4081026	720.0665678	835.5007295	855.5760076	769.56735	845.39081	773.9580585	833.9836637
**ENSG00000163216**	SPRR2D	1396.387119	2210.521768	2671.499193	1885.450186	2971.533386	3733.875216	14.29952349	53.16200436	76.90983876	22.14721433	31.73090079	115.0322295
**ENSG00000163218**	PGLYRP4	5.624922937	0	11.39926507	2.511700959	2.311577897	4.55258104	15.32091802	24.91968954	44.08246856	31.00610007	12.41643944	22.83728085
**ENSG00000163219**	ARHGAP25	12.65607661	17.51602035	51.29669283	0.837233653	15.02525633	8.725780326	11.23533988	24.91968954	12.66198565	5.062220419	8.277626294	8.458252168
**ENSG00000163220**	S100A9	161.7165344	91.08330583	245.8984323	319.8232554	168.7451864	340.3054327	377.9159779	277.4392102	485.845079	543.5559175	400.0852709	787.4632768
**ENSG00000163221**	S100A12	0	0	0	3.348934611	0	1.517527013	0	0	0.468962431	2.53111021	0	0
**ENSG00000163235**	TGFA	5205.866178	5440.475921	7643.207231	4649.995708	3744.756192	6495.394998	100.0966644	78.0816939	200.7159207	62.64497769	80.01705417	182.6982468
**ENSG00000163249**	CCNYL1	742.4898276	714.6536304	655.4577417	1029.797393	841.4143543	864.231634	877.3779055	1244.323164	953.8695856	823.8763732	1102.303901	943.0951167
**ENSG00000163251**	FZD5	386.7134519	332.8043867	396.5315779	246.9839276	293.5703929	256.841447	727.2329088	1196.145098	910.2560795	692.2586423	790.5133111	817.9129846
**ENSG00000163257**	DCAF16	901.3939006	900.3234461	929.8543366	1036.495262	1027.496375	922.2770423	1189.924633	941.9642647	1070.641231	1228.854007	931.2329581	1250.12967
**ENSG00000163263**	C1orf189	2.812461468	10.50961221	8.142332194	4.186168264	1.155788948	3.41443578	3.064183605	1.661312636	4.689624315	3.163887762	1.379604382	2.53747565
**ENSG00000163281**	GNPDA2	299.5271464	388.8556518	257.2976973	256.1934978	263.5198802	308.0579837	298.2472042	244.2129575	256.0534876	256.9076863	211.0794705	250.3642642
**ENSG00000163283**	ALPP	199.6847643	115.6057343	193.7875062	421.965761	367.5408855	405.1797125	2204.169406	3274.447206	2508.011083	3365.743801	4508.547121	3033.129227
**ENSG00000163285**	GABRG1	0	0	0	0	0	0.379381753	10.21394535	14.95181373	18.28953483	7.593330629	9.657230676	17.76232955
**ENSG00000163286**	ALPPL2	4.218692202	0	1.628466439	19.25637402	12.71367843	9.105162079	60.26227756	56.48462963	105.9855095	86.05774712	88.29468047	158.1693155
**ENSG00000163288**	GABRB1	0	0	0	0	0	0	1.021394535	0	4.689624315	3.163887762	0	2.53747565
**ENSG00000163291**	PAQR3	319.2143767	339.8107948	382.6896131	384.2902467	321.3093276	471.5715194	247.1774774	201.018829	280.9084964	284.7498986	197.2834267	303.6512528
**ENSG00000163293**	NIPAL1	464.0561423	472.9325495	508.0815289	315.6370871	286.6356592	404.420949	80.69016826	94.69482026	162.2610013	94.2838553	89.67428485	178.4691207
**ENSG00000163297**	ANTXR2	15.46853808	35.0320407	47.22552673	14.2329721	39.29682424	27.69486799	0	0	0	3.163887762	0	0
**ENSG00000163312**	HELQ	337.4953762	318.7915704	330.5786871	294.7062458	298.1935487	349.7899765	225.7281922	210.9867048	218.5364931	213.8788127	219.3570968	220.7603816
**ENSG00000163319**	MRPS18C	645.459907	581.5318757	637.5446108	804.5815404	606.7891978	712.8583145	394.2582905	428.6186601	442.7005353	376.5026437	393.187249	444.904064
**ENSG00000163320**	CGGBP1	1001.236283	1019.432384	1010.463425	772.7666616	902.6711686	894.5821743	1634.231256	1476.906934	1551.327723	1406.031721	1331.318229	1490.344032
**ENSG00000163322**	ABRAXAS1	216.5595331	136.6249587	115.6211172	168.2839642	180.3030759	149.8557926	168.5300983	172.7765142	119.58542	169.584384	121.4051856	156.4776651
**ENSG00000163328**	GPR155	1.406230734	7.006408141	1.628466439	5.86063557	13.86946738	9.105162079	103.160848	103.0013834	42.67558126	118.9621798	113.1275593	71.89514343
**ENSG00000163344**	PMVK	284.0586083	203.1858361	276.8392946	391.8253495	291.258815	347.1343043	271.6909463	202.6801416	321.2392655	348.0276538	353.1787219	348.4799893
**ENSG00000163346**	PBXIP1	212.3408409	413.3780803	284.9816268	280.4732737	405.6819208	311.8518012	328.8890402	323.955964	370.0113584	343.5982109	390.4280402	363.7048432
**ENSG00000163347**	CLDN1	4807.90288	5328.373391	5196.436407	3037.483693	3011.985999	4107.945625	3140.788195	1988.591225	3773.271723	2646.908502	1840.392246	3618.440277
**ENSG00000163348**	PYGO2	729.833751	697.13761	785.7350568	908.3985134	802.1175301	822.8790229	797.7091317	689.444744	967.0005337	817.5485977	840.1790688	954.0908445
**ENSG00000163349**	HIPK1	2154.345485	2108.92885	2202.500859	2664.914717	2690.676672	2746.723894	1973.334241	2234.465496	2052.1796	1943.259863	2218.403847	2408.064392
**ENSG00000163352**	LENEP	1.406230734	0	2.442699658	2.511700959	5.778944741	1.13814526	2.04278907	0	1.406887294	3.796665314	1.379604382	3.383300867

**ENSG00000163354**	DCST2	35.15576835	56.05126513	25.2412298	26.79147689	36.98524634	14.79588838	96.01108628	74.75906863	82.53738794	101.2444084	77.25784541	50.74951301
**ENSG00000163357**	DCST1	0	3.50320407	0	1.674467306	1.155788948	2.27629052	3.064183605	1.661312636	5.158586746	6.960553076	2.759208765	3.383300867
**ENSG00000163359**	COL6A3	206.7159179	178.6634076	164.4751103	185.8658709	230.0020007	198.0372752	11.23533988	23.25837691	18.75849726	11.38999594	37.24931832	23.68310607
**ENSG00000163362**	INAVA	288.2773005	322.2947745	344.4206518	208.4711796	173.3683422	231.0434878	26.55625791	43.19412854	79.72361335	52.52053685	48.28615338	89.65747298
**ENSG00000163364**	LINC01116	0	0	0.814233219	0	1.155788948	0.379381753	7.149761744	16.61312636	1.875849726	1.898332657	20.69406573	2.53747565
**ENSG00000163374**	YY1AP1	818.4262873	760.1952833	816.6759191	813.7911106	833.3238317	794.4253914	883.5062727	1059.917462	899.4699435	889.0524611	1096.785484	889.808128
**ENSG00000163376**	KBTBD8	16.87476881	10.50961221	21.17006371	48.55955187	17.33683422	23.5216687	103.160848	103.0013834	114.4268333	101.8771859	62.0821972	184.3898973
**ENSG00000163378**	EOGT	113.9046895	87.58010176	136.7911809	143.1669546	166.4336086	163.1341539	56.17669942	31.56494009	62.84096581	82.26108181	99.33151553	87.11999733
**ENSG00000163380**	LMOD3	1.406230734	0	3.256932878	3.348934611	0	0.758763507	0	0	0	0	0	0
**ENSG00000163382**	NAXE	441.5564505	423.8876925	573.2201865	639.6465108	658.7997005	677.5758114	509.6758729	571.4915468	512.1069751	544.188695	656.691686	576.0069726
**ENSG00000163389**	POGLUT1	295.3084542	231.2114686	284.9816268	380.9413121	312.063016	301.2291121	464.7345134	407.0215959	397.6801419	441.6787316	434.5753804	430.5250353
**ENSG00000163393**	SLC22A15	21.09346101	17.51602035	25.2412298	8.372336529	11.55788948	13.27836137	43.919965	48.17806645	40.79973154	40.49776335	37.24931832	44.82873649
**ENSG00000163395**	IGFN1	194.0598413	318.7915704	260.5546302	90.42123451	109.7999501	109.261945	1.021394535	0	1.875849726	1.898332657	16.55525259	2.53747565
**ENSG00000163399**	ATP1A1	6081.947925	6477.424326	5982.985696	6327.811948	7261.821962	6466.561985	6151.859284	5648.462963	6790.107045	6920.68809	6911.817955	7705.467725
**ENSG00000163406**	SLC15A2	116.7171509	161.1473872	156.3327781	108.8403749	139.8504627	105.4681274	307.439755	254.1808333	307.1703926	288.5465639	275.9208765	340.0217371
**ENSG00000163412**	EIF4E3	120.9358431	94.5865099	103.4076189	10.04680383	17.33683422	12.89897961	931.5118158	827.3336928	773.7880119	789.0736078	692.5613999	691.8850273
**ENSG00000163428**	LRRC58	3494.483374	3776.453988	3041.975308	3364.004817	3632.644664	3327.93674	2916.081397	4125.039276	2994.794087	2951.907282	3802.189678	3410.367274
**ENSG00000163430**	FSTL1	1456.855041	1310.198322	1573.912813	2148.341553	1778.759191	2137.81618	3212.285812	3556.870354	3385.439793	3150.599433	3306.911704	3848.504736
**ENSG00000163431**	LMOD1	0	0	1.628466439	1.674467306	5.778944741	0.379381753	1.021394535	4.983937908	0.468962431	0.632777552	0	0
**ENSG00000163435**	ELF3	485.1496033	889.8138339	542.2793242	439.5476678	737.393349	536.8251809	489.2479822	609.7017375	400.4939165	417.6331846	522.8700609	271.5098946
**ENSG00000163440**	PDCL2	0	0	2.442699658	0	1.155788948	4.173199286	0	0	0	0.632777552	0	2.53747565
**ENSG00000163444**	TMEM183A	472.4935267	634.0799367	501.5676632	740.9517828	654.1765447	699.9593348	649.6069242	782.4782516	719.3883698	747.3102894	848.4566951	861.0500707
**ENSG00000163449**	TMEM169	4.218692202	0	0	1.674467306	4.623155793	1.13814526	0	0	0	0	0	0
**ENSG00000163453**	IGFBP7	511.8679872	490.4485699	398.9742775	285.4966756	248.4946239	296.6765311	507.6330838	711.0418083	838.035865	574.5620176	638.756829	910.9537585
**ENSG00000163462**	TRIM46	28.12461468	42.03844885	29.3123959	30.97764516	57.78944741	30.35054026	5.106972674	1.661312636	4.689624315	5.062220419	2.759208765	0
**ENSG00000163463**	KRTCAP2	92.81122845	87.58010176	61.88172468	88.7467672	75.12628164	44.76704689	50.04833221	86.38825708	72.68917688	77.19886139	63.46180159	50.74951301
**ENSG00000163464**	CXCR1	0	0	0.814233219	7.535102876	1.155788948	5.311344546	0	0	0	0	0	0
**ENSG00000163466**	ARPC2	5027.274875	4449.069169	4387.90282	5018.378515	4558.431612	4326.090133	4494.135953	5214.860365	4549.404548	4573.716149	5201.108521	4825.432862
**ENSG00000163467**	TSACC	5.624922937	3.50320407	5.699632536	9.209570182	5.778944741	3.41443578	3.064183605	4.983937908	1.875849726	5.062220419	6.898021912	2.53747565
**ENSG00000163468**	CCT3	4045.725822	3639.829029	4385.46012	4751.30098	4411.646416	4907.302979	2894.632112	2678.035969	3106.407146	3122.757221	2724.718655	3472.95834
**ENSG00000163472**	TMEM79	140.6230734	147.134571	139.2338805	220.1924507	201.107277	185.8970591	157.2947584	201.018829	191.8056345	152.4993901	177.9689653	144.6361121
**ENSG00000163479**	SSR2	1357.012658	1299.68871	1499.81759	1460.972724	1338.403602	1628.685867	1433.016532	1491.858747	1571.96207	1532.587232	1720.366665	1722.100141
**ENSG00000163481**	RNF25	172.9663803	182.1666117	165.2893435	191.7265065	189.5493875	195.381603	109.2892152	136.2276362	129.4336311	130.3521758	157.2748996	96.42407471
**ENSG00000163482**	STK36	146.2479964	122.6121425	115.6211172	160.7488614	183.7704428	122.9196881	179.7654381	162.8086383	151.0059029	202.4888168	161.4137127	142.0986364
**ENSG00000163485**	ADORA1	9.843615139	10.50961221	8.142332194	31.81487881	68.19154795	48.18148267	10.21394535	3.322625272	8.441323766	4.429442867	0	15.2248539
**ENSG00000163491**	NEK10	19.68723028	10.50961221	30.12662912	23.44254228	42.76419109	39.07632059	61.28367209	31.56494009	53.46171719	69.60553076	64.84140597	61.74524082
**ENSG00000163492**	CCDC141	12.65607661	7.006408141	18.72736405	4.186168264	12.71367843	23.5216687	5.106972674	9.967875817	0.937924863	0.632777552	4.138813147	0
**ENSG00000163507**	CIP2A	1482.167194	1201.598996	1484.347159	1673.630072	1389.258316	1548.256935	1252.2297	956.9160784	1241.343556	1168.740139	1040.221704	1426.061315
**ENSG00000163510**	CWC22	665.1471373	774.2080996	715.7109999	651.3677819	631.0607658	611.9427681	584.2376739	732.6388725	592.7685134	589.1159013	615.3035545	689.3475517
**ENSG00000163512**	AZI2	397.9632978	409.8748762	393.274645	475.5487148	442.6671672	421.1137462	529.0823691	554.8784205	536.961984	585.319236	613.9239501	563.3195944
**ENSG00000163513**	TGFBR2	23.90592248	77.07048955	181.5740079	17.58190671	98.2420606	217.0063629	4.08557814	0	2.344812157	3.163887762	0	4.229126084
**ENSG00000163515**	RETNLB	2.812461468	17.51602035	3.256932878	0	0	1.13814526	1.021394535	0	0	0	0	0
**ENSG00000163516**	ANKZF1	268.5900702	938.8586909	329.7644539	243.634993	722.3680927	328.1652166	329.9104348	750.9133115	267.3085859	350.558764	798.7909374	296.0388259
**ENSG00000163517**	HDAC11	39.37446056	70.06408141	48.03975995	56.09465474	68.19154795	70.94438787	40.8557814	64.79119281	53.46171719	58.84831237	80.01705417	56.67028952
**ENSG00000163520**	FBLN2	1.406230734	0	0.814233219	0	2.311577897	2.27629052	1.021394535	0	0.468962431	0.632777552	4.138813147	0.845825217
**ENSG00000163521**	GLB1L	39.37446056	52.54806106	37.45472809	61.11805666	32.36209055	55.38973598	74.56180105	104.6626961	69.87540229	65.1760879	91.05388923	84.58252168
**ENSG00000163527**	STT3B	3303.235995	3089.82599	3306.601104	3286.979321	3393.396352	3459.202827	2606.598853	2515.227331	2812.836664	2881.668974	2840.605423	3338.472131
**ENSG00000163528**	CHCHD4	264.371378	140.1281628	231.2422343	193.4009738	213.8209554	232.181633	280.8834971	169.4538889	240.5777273	265.1337944	176.5893609	269.8182442
**ENSG00000163530**	DPPA2	77.34269038	45.54165292	80.60908873	29.30317785	19.64841212	24.65981396	883.5062727	956.9160784	1077.675667	415.7348519	536.6661047	641.1355143
**ENSG00000163531**	NFASC	49.2180757	17.51602035	36.64049488	3.348934611	17.33683422	16.31341539	3.064183605	11.62918845	8.910286198	0.632777552	6.898021912	6.766601734
**ENSG00000163534**	FCRL1	23.90592248	38.53524477	27.68392946	0	0	0	0	0	0	0	0	0
**ENSG00000163535**	SGO2	956.2368992	651.5959571	974.6371637	1296.874928	1182.372094	1218.953573	828.3509678	819.0271296	835.6910528	890.3180162	829.1422338	916.874535
**ENSG00000163536**	SERPINI1	7.031153671	7.006408141	13.02773151	5.023401917	38.14103529	13.27836137	15.32091802	19.93575163	8.910286198	7.593330629	9.657230676	2.53747565
**ENSG00000163539**	CLASP2	764.9895194	693.6344059	775.9642581	842.2570548	773.2228064	925.3120963	1015.266168	747.5906863	997.9520541	971.9463205	980.8987158	1064.893948
**ENSG00000163541**	SUCLG1	570.9296781	616.5639164	603.3468156	740.9517828	709.6544142	697.3036626	880.4420891	820.6884422	800.5188705	961.1891021	786.3744979	872.8916237
**ENSG00000163545**	NUAK2	1126.390818	1625.486689	1654.521902	1044.030365	913.0732691	1232.990698	792.6021591	556.5397331	893.8423943	695.4225301	427.6773585	783.2341507
**ENSG00000163554**	SPTA1	385.3072212	427.3908966	377.8042138	2.511700959	0	0	296.2044151	297.3749619	113.9578708	5.062220419	0	2.53747565
**ENSG00000163558**	PRKCI	1075.766512	952.8715072	924.9689373	1139.475002	1101.466868	1116.141118	1727.178158	1599.844069	1511.465917	1587.006101	1542.397699	1505.568886
**ENSG00000163564**	PYHIN1	23.90592248	91.08330583	33.383562	0	0	0	48.00554314	21.59706427	28.60670832	15.81943881	5.518417529	4.229126084
**ENSG00000163565**	IFI16	6163.509308	6403.857041	5339.741453	1257.524947	1574.184548	1004.602883	2539.186814	2804.29573	2529.583355	1186.457911	1356.151108	1129.176664
**ENSG00000163568**	AIM2	3875.571903	5268.818922	4208.771511	61.11805666	70.50312584	81.18769521	606.7083537	548.2331699	641.0716438	31.63887762	27.59208765	19.45397999
**ENSG00000163576**	EFHB	18.28099954	28.02563256	12.21349829	20.09360767	15.02525633	20.48661468	114.3961879	84.72694444	83.94427523	129.7193982	118.6459769	79.50757038
**ENSG00000163584**	RPL22L1	306.5583	245.2242849	293.9381922	365.8711063	249.6504128	297.8146763	233.8993485	219.293268	221.8192301	183.5054902	140.719647	255.4392155
**ENSG00000163590**	PPM1L	0	3.50320407	0.814233219	13.39573845	10.40210053	3.41443578	10.21394535	13.29050109	30.01359561	24.67832454	37.24931832	55.82446431
**ENSG00000163596**	ICA1L	12.65607661	31.52883663	12.21349829	21.76807497	15.02525633	12.51959786	38.81299233	21.59706427	25.79293373	19.61610412	35.86971394	32.98718345
**ENSG00000163597**	SNHG16	2242.938021	1198.095792	2003.827953	2500.816921	1714.03501	2510.369062	959.0894682	755.8972494	1063.137832	1138.999594	837.4198601	1183.309478
**ENSG00000163602**	RYBP	551.2424478	574.5254675	543.9077906	584.3890897	672.6691679	591.0767716	1040.801031	2375.67707	1184.130139	914.9963407	1921.788905	1098.726957

**ENSG00000163605**	PPP4R2	1094.047511	1107.012486	1130.155709	967.8421027	1096.843712	1070.615308	1254.272489	1324.066171	1338.418779	1164.943474	1281.652471	1474.273353
**ENSG00000163606**	CD200R1	7.031153671	7.006408141	0.814233219	5.023401917	0	3.41443578	1.021394535	0	4.689624315	1.898332657	1.379604382	2.53747565
**ENSG00000163607**	GTPBP8	247.4966092	178.6634076	245.8984323	300.5668814	284.3240813	292.5033318	320.717884	282.4231481	318.425491	293.6087843	310.410986	334.1009606
**ENSG00000163608**	NEPRO	617.3352923	476.4357536	541.4650909	586.9007907	547.8439615	564.8994307	874.3137219	918.7058878	893.3734319	855.5152508	892.6040354	872.0457985
**ENSG00000163611**	SPICE1	108.2797665	154.1409791	76.53792263	117.2127114	119.0462617	77.77325943	248.198872	302.3588998	211.0330942	199.9577066	340.7622824	230.064459
**ENSG00000163617**	CCDC191	134.9981505	105.0961221	103.4076189	79.53719702	104.0210053	59.56293527	243.0918993	267.4713344	168.8264753	161.9910534	189.0058004	133.6403843
**ENSG00000163623**	NKX6-1	7.031153671	0	19.54159727	2.511700959	12.71367843	17.45156065	3.064183605	1.661312636	6.56547404	3.796665314	2.759208765	7.612426951
**ENSG00000163624**	CDS1	923.8935923	1022.935589	939.6251352	1196.40689	1284.081522	1528.529084	931.5118158	711.0418083	867.5804982	942.2057755	782.2356848	1035.290065
**ENSG00000163625**	WDFY3	1837.94357	2017.845545	2161.789198	2169.272395	2315.045263	2415.903005	1545.369931	1447.003306	1580.872356	1686.352177	1676.219325	1586.768107
**ENSG00000163626**	COX18	157.4978422	150.637775	170.9889761	165.7722633	221.9114781	168.0661167	181.8082272	207.6640795	200.7159207	184.7710453	102.0907243	208.0730033
**ENSG00000163629**	PTPN13	4028.851053	2340.140319	2920.654558	4540.318099	2636.354591	3692.901987	1562.733638	1201.129036	1613.699727	1390.84506	1360.289921	1917.485766
**ENSG00000163633**	C4orf36	0	7.006408141	1.628466439	0	0	0	1.021394535	1.661312636	0.468962431	1.265555105	0	1.691650434
**ENSG00000163634**	THOC7	510.4617565	493.9517739	508.8957622	591.9241926	561.7134289	588.0417176	562.7883887	671.170305	646.699193	656.1903218	638.756829	653.8228926
**ENSG00000163635**	ATXN7	1535.603962	1653.512321	1615.438707	961.1442335	935.0332591	868.4048333	1621.974521	1942.074472	1575.71377	1238.34567	1396.159635	1236.596467
**ENSG00000163636**	PSMD6	808.5826721	861.7882013	852.5021808	942.7250931	774.3785953	884.7182487	1111.277254	948.6095152	1103.468601	1105.462384	1018.148034	1144.401518
**ENSG00000163637**	PRICKLE2	1.406230734	0	1.628466439	0	1.155788948	2.27629052	16.34231256	18.274439	21.57227185	18.35054902	6.898021912	10.99572782
**ENSG00000163638**	ADAMTS9	0	0	0.814233219	0	1.155788948	0	1.021394535	11.62918845	3.28273702	4.429442867	0	2.53747565
**ENSG00000163644**	PPM1K	144.8417656	301.2755501	189.7163401	148.1903566	255.4293576	218.1445082	116.438977	88.04956971	96.13729845	112.0016268	146.2380645	132.794559
**ENSG00000163645**	ERICH6	8.437384405	0	3.256932878	4.186168264	1.155788948	1.896908767	4.08557814	0	1.875849726	3.796665314	0	0
**ENSG00000163655**	GMPS	3879.790596	4144.290415	3493.060511	3368.190985	3634.956242	3249.025335	3457.420501	3399.045654	3341.826287	3303.731601	3102.730256	3590.528045
**ENSG00000163659**	TIPARP	2257.000328	2371.669156	2220.413989	1916.427831	1950.971745	1745.914829	1041.822426	1451.987244	983.4142188	1128.242376	1285.791284	1164.701324
**ENSG00000163660**	CCNL1	2775.899469	3236.960561	2874.243265	1457.62379	1272.523632	1517.527013	2054.02441	2053.382418	2005.283357	1647.752746	1495.49115	1734.78752
**ENSG00000163661**	PTX3	0	0	1.628466439	0.837233653	0	0.758763507	0	11.62918845	9.848211061	1.265555105	6.898021912	6.766601734
**ENSG00000163666**	HESX1	5.624922937	10.50961221	13.84196473	2.511700959	3.467366845	9.484543833	5.106972674	21.59706427	4.689624315	13.2883286	1.379604382	12.68737825
**ENSG00000163681**	SLMAP	1988.410258	1863.704565	2158.532265	1861.17041	1687.451864	1964.438719	1735.349315	1525.085	1618.858313	1564.858887	1619.655545	1645.975872
**ENSG00000163682**	RPL9	7167.558052	6561.501224	5746.858063	6994.249936	6490.910733	6223.757663	5288.780902	6175.099068	5575.025385	5491.876377	5595.675375	6342.843301
**ENSG00000163683**	SMIM14	592.0231391	812.7433443	403.0454436	293.0317785	288.9472371	245.0806126	638.3715843	641.2666775	546.3412326	537.8609195	564.2581924	571.7778465
**ENSG00000163684**	RPP14	52.03053716	63.05767327	88.75142092	104.6542066	105.1767943	91.05162079	126.6529223	93.03350762	105.5165471	106.9394064	108.9887462	82.04504603
**ENSG00000163686**	ABHD6	46.40561423	24.52242849	27.68392946	23.44254228	32.36209055	55.76911774	45.96275407	33.22625272	33.29633263	33.53721028	24.83287888	45.67456171
**ENSG00000163689**	C3orf67	112.4984587	245.2242849	146.5619795	73.67656145	135.2273069	114.1939077	31.66323058	24.91968954	37.98595695	19.61610412	66.22101035	30.4497078
**ENSG00000163694**	RBM47	289.6835312	364.3332233	464.1129351	216.0062824	268.143036	349.7899765	418.7717593	219.293268	387.8319308	413.2037417	299.374151	439.8291127
**ENSG00000163697**	APBB2	2228.875714	1306.695118	2023.36955	1665.257736	1101.466868	1763.366389	405.4936303	410.3442211	460.0521453	379.0337539	355.9379306	414.4543562
**ENSG00000163701**	IL17RE	52.03053716	66.56087734	47.22552673	59.44358935	27.73893476	52.73406371	85.79714093	73.09775599	57.21341664	94.2838553	115.8867681	51.59533822
**ENSG00000163702**	IL17RC	174.372611	147.134571	149.0046792	120.561646	115.5788948	122.1609246	182.8296217	171.1152015	182.4263858	146.1716146	133.8216251	132.794559
**ENSG00000163703**	CRELD1	18.28099954	17.51602035	44.78282707	18.41914036	4.623155793	18.58970591	42.89857046	28.24231481	24.38604644	41.1305409	19.31446135	24.52893129
**ENSG00000163704**	PRRT3	19.68723028	0	10.58503185	9.209570182	30.05051265	7.587635066	13.27812895	11.62918845	28.13774589	32.27165517	11.03683506	29.60388259
**ENSG00000163705**	FANCD2OS	0	0	0.814233219	0	0	0.758763507	0	4.983937908	0	0	1.379604382	2.53747565
**ENSG00000163710**	PCOLCE2	9.843615139	3.50320407	0.814233219	14.2329721	9.246311586	7.587635066	10.21394535	34.88756536	9.848211061	6.960553076	13.79604382	4.229126084
**ENSG00000163714**	U2SURP	4255.254202	3814.989233	3921.347185	4146.818283	3665.006755	3645.479268	3963.010795	3043.524749	3402.32244	4011.809682	3614.563482	3889.104347
**ENSG00000163719**	MTMR14	399.3695285	409.8748762	411.1877758	320.660489	345.5808955	334.6147064	584.2376739	466.8288508	480.2175298	454.9670602	529.7680828	404.3044536
**ENSG00000163728**	TTC14	836.7072868	774.2080996	837.8459828	706.625203	694.6291579	688.957264	1134.769328	1124.708655	1024.21395	1001.054088	974.0006939	994.6904549
**ENSG00000163734**	CXCL3	0	7.006408141	12.21349829	1.674467306	5.778944741	4.931962793	4.08557814	1.661312636	0.937924863	2.53111021	2.759208765	9.304077385
**ENSG00000163735**	CXCL5	4.218692202	3.50320407	4.071166097	5.86063557	3.467366845	2.27629052	0	0	0	0	1.379604382	1.691650434
**ENSG00000163738**	MTHFD2L	642.6474455	550.0030391	484.4687656	601.1337628	367.5408855	444.2560331	338.081591	249.1968954	304.8255804	308.162668	237.2919538	307.8803789
**ENSG00000163739**	CXCL1	4.218692202	35.0320407	27.68392946	2.511700959	12.71367843	7.587635066	1.021394535	3.322625272	2.344812157	0	6.898021912	5.074951301
**ENSG00000163743**	RCHY1	357.1826065	339.8107948	371.2903481	323.17219	277.3893476	380.8992803	295.1830206	299.0362745	307.639355	301.2021149	304.8925685	324.7968832
**ENSG00000163746**	PLSCR2	9.843615139	3.50320407	5.699632536	3.348934611	8.090522638	0.379381753	6.128367209	0	3.751699452	8.226108181	0	5.920776517
**ENSG00000163749**	CCDC158	0	0	0	0	0	0	6.128367209	4.983937908	3.28273702	5.062220419	2.759208765	1.691650434
**ENSG00000163754**	GYG1	579.3670625	620.0671205	655.4577417	632.9486416	733.9259821	688.1985005	485.1624041	438.5865359	511.1690503	518.877593	430.4365673	597.9984283
**ENSG00000163755**	HPS3	788.8954419	1180.579772	984.4079623	642.9954454	797.4943743	665.814977	1115.362832	1347.324548	1074.39293	940.9402204	1140.932824	1022.602687
**ENSG00000163781**	TOPBP1	2801.211622	3674.86107	2857.9586	3078.508142	3151.836462	3081.717982	3004.942722	2892.345299	2838.629598	2741.825134	2672.293689	3066.962236
**ENSG00000163785**	RYK	2029.190949	1856.698157	2034.768815	2019.407571	1846.950739	1992.89235	1363.561704	1252.629728	1420.487205	1297.82676	1358.910317	1653.588299
**ENSG00000163788**	SNRK	738.2711354	1166.566955	789.8062229	580.2029214	628.7491879	650.6397069	768.0886902	903.7540741	687.9678869	587.2175686	758.7824103	636.9063882
**ENSG00000163794**	UCN	5.624922937	3.50320407	4.885399317	2.511700959	6.93473369	1.13814526	9.192550814	8.306563181	4.689624315	8.226108181	6.898021912	6.766601734
**ENSG00000163795**	ZNF513	113.9046895	126.1153465	122.1349829	109.6776085	86.68417112	111.9176172	108.2678207	76.42038126	87.22701225	112.6344043	63.46180159	124.3363069
**ENSG00000163798**	SLC4A1AP	959.0493607	1047.458017	1045.475454	946.9112614	936.1890481	944.281184	852.8644366	976.85183	839.4427523	908.6685652	845.6974864	906.7246324
**ENSG00000163803**	PLB1	105.4673051	87.58010176	74.09522297	78.69996337	153.7199301	72.46191488	235.9421376	405.3602832	244.3294268	203.7543719	314.5497992	197.0772755
**ENSG00000163806**	SPDYA	144.8417656	182.1666117	114.8068839	113.8637768	120.2020506	89.53409378	138.9096567	166.1312636	92.85456143	124.0244003	153.1360864	85.42834689
**ENSG00000163807**	KIAA1143	638.4287533	616.5639164	638.358844	779.4645308	822.9217312	710.2026422	876.3565109	887.1409477	954.8075104	911.1996754	922.9553318	931.2535637
**ENSG00000163808**	KIF15	599.0542928	395.86206	504.0103628	800.3953721	599.8544641	625.2211294	1180.732082	1098.127652	1072.517081	1169.372917	1142.312429	1221.371613
**ENSG00000163810**	TGM4	1.406230734	3.50320407	2.442699658	21.76807497	6.93473369	15.55465189	0	0	0	0	2.759208765	0
**ENSG00000163811**	WDR43	2415.904401	1352.236771	2161.789198	2650.681745	1700.165543	2458.393761	1912.050569	1334.034047	1785.339977	1933.7682	1260.958405	1917.485766
**ENSG00000163812**	ZDHHC3	1116.547203	935.3554868	1082.115949	1424.134444	1338.403602	1529.667229	1823.189245	1782.588459	1830.829332	1860.998782	1894.196817	1845.590623
**ENSG00000163814**	CDCP1	4360.721507	4102.251966	5237.962301	2420.44249	2601.680923	3088.546854	2304.266071	1996.897789	2608.838006	1497.784466	1347.873482	1687.421307
**ENSG00000163815**	CLEC3B	0	0	0	1.674467306	0	1.517527013	0	0	0.937924863	0	2.759208765	0
**ENSG00000163818**	LZTFL1	206.7159179	283.7595297	201.1156052	266.2403016	325.9324834	186.6558226	522.9540019	606.3791122	449.7349718	509.3859297	666.3489167	461.8205684
**ENSG00000163820**	FYCO1	1044.829435	1096.502874	1166.796203	956.1208316	917.6964249	1057.336946	1203.202762	1282.533355	1183.192215	1188.989021	1347.873482	1079.272977

**ENSG00000163823**	CCR1	0	3.50320407	5.699632536	0.837233653	0	2.655672273	16.34231256	16.61312636	19.22745969	17.08499391	5.518417529	10.99572782
**ENSG00000163825**	RTP3	2.812461468	0	0.814233219	0.837233653	0	3.035054026	0	0	0	0	0	0
**ENSG00000163827**	LRRC2	19.68723028	31.52883663	30.12662912	9.209570182	10.40210053	24.65981396	0	8.306563181	4.689624315	8.858885733	6.898021912	1.691650434
**ENSG00000163832**	ELP6	201.090995	199.682632	229.6137679	196.7499084	183.7704428	203.3486198	283.9476807	259.1647712	247.6121638	271.46157	264.8840414	235.1394103
**ENSG00000163840**	DTX3L	11626.71571	13858.6753	12274.56578	3143.812367	3963.200304	3511.178127	7728.892445	9879.826247	7732.25257	2722.20903	2855.781071	2615.29157
**ENSG00000163848**	ZNF148	1541.228885	1930.265443	1594.268644	1742.283232	1747.55289	1727.704505	1582.140135	2025.140103	1659.189082	1654.080522	1768.652818	1844.744798
**ENSG00000163864**	NMNAT3	5.624922937	0	4.885399317	3.348934611	3.467366845	3.035054026	8.171156279	3.322625272	5.158586746	3.163887762	9.657230676	9.304077385
**ENSG00000163866**	SMIM12	673.5845217	592.0414879	705.125968	798.7209048	676.1365347	747.0026723	667.9920258	832.3176307	716.5745953	743.513624	798.7909374	707.9557064
**ENSG00000163867**	ZMYM6	272.8087624	318.7915704	257.2976973	299.7296477	314.3745939	269.3610448	330.9318293	184.4057026	317.0186037	335.3721028	289.7169203	297.7304763
**ENSG00000163870**	TPRA1	444.368912	255.7338971	493.425331	313.9626198	335.178795	523.1674378	431.0284937	214.3093301	562.7549177	387.8926396	274.5412721	471.1246457
**ENSG00000163872**	YEATS2	711.5527515	1254.147057	696.1694026	639.6465108	1170.814205	631.2912375	726.2115143	1613.13457	570.2583166	774.5197241	1609.998314	700.3432795
**ENSG00000163874**	ZC3H12A	288.2773005	399.365264	366.4049488	177.4935344	250.8062018	202.5898563	136.8668677	197.6962037	166.9506256	142.3749493	114.5071637	115.0322295
**ENSG00000163875**	MEAF6	899.9876699	1278.669486	913.5696722	859.0017278	1030.963742	851.7120362	1007.095011	1377.228175	1031.717349	973.8446531	1266.476823	1012.452784
**ENSG00000163877**	SNIP1	646.8661377	774.2080996	676.6278054	574.3422859	634.5281326	565.6581942	614.87951	684.4608061	543.0584956	489.7698255	656.691686	480.4287231
**ENSG00000163882**	POLR2H	472.4935267	546.499835	467.369868	565.1327157	576.7386852	502.3014414	297.2258096	446.8930991	389.2388181	435.9837336	492.5187645	389.9254249
**ENSG00000163884**	KLF15	14.06230734	0	3.256932878	20.09360767	0	7.587635066	5.106972674	0	2.344812157	6.327775524	2.759208765	5.920776517
**ENSG00000163888**	CAMK2N2	4.218692202	7.006408141	4.885399317	6.697869223	10.40210053	4.173199286	3.064183605	3.322625272	0.468962431	3.796665314	2.759208765	0.845825217
**ENSG00000163898**	LIPH	352.9639143	665.6087734	655.4577417	167.4467306	189.5493875	321.7157268	53.11251581	54.82331699	100.3579603	27.20943475	74.49863664	150.5568886
**ENSG00000163900**	TMEM41A	361.4012987	465.9261414	504.8245961	418.6168264	462.3155793	463.2251208	363.6164544	375.4566558	369.0734336	368.909313	387.6688314	408.5335797
**ENSG00000163902**	RPN1	4433.845505	4403.527517	4291.8233	3973.510917	3767.871971	4150.056999	3232.713703	3727.985555	3737.161616	3559.373732	3585.59179	3899.254249
**ENSG00000163904**	SENP2	1044.829435	1366.249587	1161.096571	1162.08031	1205.487873	1194.293759	1184.81766	1212.758224	1092.213503	1226.322897	1259.578801	1336.403843
**ENSG00000163909**	HEYL	0	0	1.628466439	0.837233653	0	1.13814526	21.44928523	4.983937908	16.4136851	34.80276538	11.03683506	28.75805737
**ENSG00000163913**	IFT122	241.8716863	227.7082646	233.684934	364.196639	336.3345839	331.2002706	375.8731888	305.681525	393.45948	430.9215132	420.7793366	393.3087258
**ENSG00000163918**	RFC4	862.01944	830.2593647	781.6638907	863.1878961	987.0437618	861.9553435	798.7305263	910.3993246	806.1464197	825.1419283	852.5955083	897.420555
**ENSG00000163930**	BAP1	514.6804487	711.1504263	606.6037485	598.6220618	686.5386353	728.7923481	703.7408345	671.170305	785.0431103	706.1797485	616.6831589	670.7393969
**ENSG00000163931**	TKT	2660.588549	2249.057013	2817.246939	2379.418041	2697.611405	2579.416541	4631.002821	5749.803034	4782.478876	4451.590081	5501.862277	4295.100451
**ENSG00000163932**	PRKCD	146.2479964	259.2371012	219.8429693	174.9818334	238.0925233	201.8310928	227.7709813	250.8582081	261.6810368	212.6132576	303.5129641	247.8267885
**ENSG00000163933**	RFT1	302.3396078	283.7595297	258.9261638	294.7062458	265.8314581	273.9136259	351.35972	338.9077778	371.4182457	382.8304192	371.1135788	345.9425137
**ENSG00000163935**	SFMBT1	538.5863712	662.1055693	564.2636211	567.6444166	525.8839715	559.5880861	761.960323	769.1877505	740.0227168	694.156975	711.8758613	733.3304629
**ENSG00000163938**	GNL3	1442.792733	910.8330583	1257.990324	1746.4694	1235.538386	1435.580555	1382.9682	873.8504466	1243.219406	1415.523385	893.9836397	1517.410439
**ENSG00000163939**	PBRM1	1226.2332	1366.249587	1249.033759	1357.155751	1287.548888	1363.877403	1691.42935	1970.316786	1612.761802	1863.529892	1979.732289	1862.507127
**ENSG00000163945**	UVSSA	426.0879124	315.2883663	434.8005392	354.1498352	387.1892977	401.0065132	494.3549549	463.5062255	426.2868502	510.0187072	462.1674681	400.9211528
**ENSG00000163946**	FAM208A	2708.400394	2525.810135	2428.04346	2638.12324	2865.200803	2544.892801	4543.162891	4311.106291	4080.911079	4336.424566	4468.538594	4528.548211
**ENSG00000163947**	ARHGEF3	81.56138258	154.1409791	128.6488487	67.81592588	98.2420606	115.332053	130.7385005	126.2597603	162.7299637	171.4827167	235.9123494	232.6019346
**ENSG00000163950**	SLBP	859.2069786	858.2849973	631.8449783	772.7666616	901.5153796	751.1758715	719.0617526	858.8986329	758.7812141	769.4575037	793.2725198	835.6753142
**ENSG00000163956**	LRPAP1	804.3639799	903.8266502	859.8302797	971.1910373	920.0080028	1014.087427	754.8105613	681.1381808	846.0082263	840.9613671	812.5869812	915.1828846
**ENSG00000163959**	SLC51A	2.812461468	0	0	0.837233653	0	3.035054026	0	0	0.937924863	0	0	0
**ENSG00000163960**	UBXN7	1484.979655	1618.480281	1318.243582	1457.62379	1717.502377	1403.333105	1970.270058	2025.140103	1751.574681	1840.117122	2368.780724	2183.92071
**ENSG00000163961**	RNF168	1520.135424	1842.685341	1430.607767	1569.813099	1488.656165	1308.487667	1281.850141	1657.990011	1369.839262	1218.729566	1656.904863	1468.352576
**ENSG00000163964**	PIGX	500.6181414	364.3332233	438.0574721	595.2731272	565.1807957	529.6169276	431.0284937	446.8930991	536.961984	468.2553888	449.7510286	605.6108552
**ENSG00000163975**	MELTF	2.812461468	35.0320407	3.256932878	4.186168264	5.778944741	6.449489806	10.21394535	9.967875817	8.910286198	3.163887762	11.03683506	4.229126084
**ENSG00000163993**	S100P	281.2461468	248.727489	265.4400295	128.9339825	109.7999501	193.8640759	814.0514443	581.4594226	932.7662762	647.9642136	408.3628972	574.3153222
**ENSG00000163995**	ABLIM2	16.87476881	21.01922442	26.05546302	17.58190671	25.42735686	14.79588838	3.064183605	4.983937908	3.751699452	8.226108181	2.759208765	5.074951301
**ENSG00000164002**	EXO5	378.2760675	346.817203	377.8042138	298.0551804	308.5956492	273.9136259	333.9960129	385.4245316	373.7630579	310.0610007	313.1701948	343.405038
**ENSG00000164008**	C1orf50	113.9046895	164.6505913	86.30872126	101.305272	80.90522638	69.42686085	136.8668677	134.5663235	119.58542	132.2505084	200.0426354	134.4862095
**ENSG00000164010**	ERMAP	68.90530597	84.07689769	111.5499511	72.8393278	86.68417112	92.56914781	231.8565594	202.6801416	162.7299637	246.7832454	202.8018442	169.1650434
**ENSG00000164011**	ZNF691	181.4037647	245.2242849	204.3725381	173.3073661	151.4083522	170.3424072	279.8621026	309.0041503	276.6878346	244.2521352	281.439294	182.6982468
**ENSG00000164022**	AIMP1	1642.477497	1530.900179	1291.373886	1476.880164	1385.790949	1440.133136	1061.228922	1154.612282	1090.806616	1137.101262	1140.932824	1098.726957
**ENSG00000164023**	SGMS2	2127.627101	1972.303892	1884.13567	2423.791425	2023.786448	2542.237129	1784.376252	1888.912467	1811.601873	1919.847094	1815.559367	1924.252368
**ENSG00000164024**	METAP1	898.5814391	938.8586909	917.6408383	1124.404796	940.8122039	1005.361646	641.4357679	589.7659858	754.5605522	653.0264341	709.1166525	731.6388125
**ENSG00000164031**	DNAJB14	1335.919197	1219.115017	1349.998678	1224.872834	1170.814205	1379.052673	1004.030828	848.9307571	1039.220748	904.8718999	997.4539684	1116.489286
**ENSG00000164032**	H2AFZ	6419.443301	5433.469513	5867.364579	7430.448669	6510.559146	7168.418229	3870.063893	3777.824935	4349.157589	3875.129731	3896.002776	4518.398308
**ENSG00000164037**	SLC9B1	2.812461468	10.50961221	17.09889761	8.372336529	4.623155793	4.55258104	2.04278907	1.661312636	3.28273702	1.898332657	1.379604382	18.60815477
**ENSG00000164038**	SLC9B2	120.9358431	112.1025303	118.87805	136.4690854	113.2673169	137.3361947	189.9793835	103.0013834	141.6266543	185.4038228	142.0992514	165.7817425
**ENSG00000164039**	BDH2	11.24984587	24.52242849	7.328098975	21.76807497	48.54313583	15.93403364	63.32646116	53.16200436	33.29633263	55.05164706	27.59208765	35.5246591
**ENSG00000164040**	PGRMC2	1385.137273	1404.784832	1175.752769	1167.103712	1303.729934	1250.821641	556.6600215	948.6095152	530.39651	565.0703543	847.0770907	578.5444483
**ENSG00000164045**	CDC25A	507.649295	511.4677943	489.3541649	632.9486416	468.094524	678.7139567	533.1679472	636.2827396	748.4640406	714.4058566	605.6463238	861.0500707
**ENSG00000164048**	ZNF589	105.4673051	168.1537954	106.6645517	118.8871787	153.7199301	121.7815428	302.3327823	186.0670152	265.4327362	260.071574	309.0313816	217.3770807
**ENSG00000164049**	FBXW12	0	3.50320407	0.814233219	0.837233653	1.155788948	0	6.128367209	0	2.813774589	0.632777552	1.379604382	0
**ENSG00000164050**	PLXNB1	511.8679872	528.9838146	489.3541649	750.161353	650.7091779	671.1263216	1462.636974	1136.337843	1307.936221	1751.528265	1343.734668	1124.947538
**ENSG00000164051**	CCDC51	139.2168427	115.6057343	191.3448066	173.3073661	195.3283323	190.0702584	311.5253331	353.8595915	249.956976	320.1854415	306.2721729	265.5891181
**ENSG00000164053**	ATRIP	146.2479964	164.6505913	137.6054141	198.4243757	182.6146538	151.7527013	282.9262862	206.0027669	235.4191406	230.3310291	168.3117346	268.9724189
**ENSG00000164054**	SHISA5	1414.668119	1492.364934	1399.666904	932.6782893	1036.742687	1103.62152	2158.206652	2169.674303	2423.128883	1570.553885	1412.714887	1569.851602
**ENSG00000164056**	SPRY1	78.74892111	143.6313669	62.6959579	154.8882258	173.3683422	91.05162079	9.192550814	29.90362745	18.28953483	10.75721839	24.83287888	31.29553302
**ENSG00000164061**	BSN	42.18692202	52.54806106	63.51019112	64.46699127	71.65891479	48.18148267	60.26227756	29.90362745	45.02039342	64.54331034	51.04536215	52.44116344
**ENSG00000164062**	APEH	817.0200565	637.5831408	745.0233958	727.5560443	797.4943743	868.4048333	836.5221241	629.6374891	932.2973137	906.7702326	709.1166525	860.2042455
**ENSG00000164066**	INTU	220.7782253	266.2435094	154.7043117	234.4254228	245.027257	197.6578935	279.8621026	255.842146	236.8260279	280.9532333	371.1135788	238.5227111

ENSG00000164068	RNF123	320.6206074	238.2178768	274.396595	257.0307314	297.0377597	319.4394363	318.6750949	307.3428377	345.625312	318.9198864	340.7622824	319.7219319
ENSG00000164070	HSPA4L	721.3963666	542.9966309	580.5482855	1028.960159	1029.807953	879.0275224	579.1307013	559.8623584	579.6375653	570.7653522	711.8758613	724.8722108
ENSG00000164073	MFSD8	375.463606	360.8300193	391.6461786	354.1498352	265.8314581	319.0600545	271.6909463	249.1968954	287.4739705	289.812119	244.1899757	285.8889233
ENSG00000164074	ABHD18	254.5277629	206.6890402	211.7006371	195.0754411	169.9009754	194.6228394	150.1449966	323.955964	210.0951693	157.5616105	211.0794705	208.0730033
ENSG00000164077	MON1A	46.40561423	35.0320407	49.66822639	107.1659076	56.63365846	98.25987411	40.8557814	39.87150327	70.81332715	77.83163894	44.14734023	80.35339559
ENSG00000164078	MST1R	1607.321729	1436.313669	1586.126311	1243.291975	1124.582647	1406.747541	1119.44841	995.126269	1319.19132	793.5030507	725.6719051	790.0007525
ENSG00000164080	RAD54L2	1316.231967	886.3106298	1442.007032	1259.199414	958.1490381	1493.625963	1737.392104	1531.730251	1729.533447	1392.110615	1386.502404	1599.455485
ENSG00000164081	TEX264	233.4343019	388.8556518	239.3845665	282.9849747	257.7409355	271.2579536	416.7289702	360.504842	361.5700347	361.94876	404.224084	357.7840667
ENSG00000164082	GRM2	2.812461468	3.50320407	4.071166097	0.837233653	5.778944741	1.896908767	5.106972674	0	0.468962431	0.632777552	8.277626294	0
ENSG00000164086	DUSP7	1895.59903	1492.364934	1833.65321	1532.974818	1260.965743	1706.079745	485.1624041	536.6039815	695.9402483	428.390403	444.2326111	633.5230874
ENSG00000164087	POC1A	265.7776088	234.7146727	302.0805244	319.8232554	337.4903729	353.2044123	344.2099583	307.3428377	407.9973154	385.994307	340.7622824	367.0881441
ENSG00000164088	PPM1M	28.12461468	45.54165292	52.11092604	42.6989163	52.01050267	46.66395566	31.66323058	39.87150327	45.95831828	43.66165111	60.70259282	29.60388259
ENSG00000164091	WDR82	1018.111052	872.2978135	1032.447722	1203.104759	959.3048271	1195.052523	1273.678985	1277.549417	1333.729155	1234.549005	1216.811065	1404.06986
ENSG00000164096	C4orf3	812.8013643	1954.787871	577.2913526	796.2092039	1396.19305	634.3262915	661.8636586	1643.038197	575.4169034	631.5119973	1512.046403	681.7351247
ENSG00000164104	HMGB2	1771.850725	1348.733567	1521.801887	1648.513062	1396.19305	1526.632175	1563.755033	1166.241471	1694.361265	1442.732819	1332.697833	1748.320723
ENSG00000164105	SAP30	164.5289959	143.6313669	78.98062229	135.6318518	228.8462118	103.9506004	71.49761744	147.8568246	100.3579603	99.34607572	176.5893609	115.8780547
ENSG00000164109	MAD2L1	1129.20328	658.6023652	944.5105346	1331.201508	1122.271069	1108.553483	739.4896433	598.072549	806.1464197	810.5880446	634.6180159	1028.523464
ENSG00000164111	ANXA5	3057.145616	3580.27456	3421.407988	3041.669861	3370.280573	3584.398805	2444.197122	2908.958426	2747.650886	2443.15413	2617.109513	2973.921462
ENSG00000164114	MAP9	143.4355349	238.2178768	154.7043117	170.7956652	210.3535886	155.5465189	2.04278907	0	0	0.632777552	2.759208765	0.845825217
ENSG00000164117	FBXO8	119.5296124	115.6057343	142.4908134	108.0031412	158.3430859	141.1300122	131.759895	144.5341993	126.6198565	117.0638472	144.8584601	121.7988312
ENSG00000164118	CEP44	261.5589166	227.7082646	236.1276336	277.9615728	254.2735686	232.9403965	272.7123408	269.1326471	232.605366	304.3660027	306.2721729	239.3685363
ENSG00000164120	HPGD	0	0	0	5.023401917	4.623155793	4.931962793	280.8834971	209.3253922	288.4118953	404.9776335	384.9096227	362.859018
ENSG00000164122	ASB5	0	0	0.814233219	0	1.155788948	4.173199286	0	9.967875817	0.937924863	0.632777552	2.759208765	0
ENSG00000164124	TMEM144	194.0598413	262.7403053	213.3291035	293.0317785	263.5198802	177.9300423	170.5728873	139.5502614	118.1785327	134.7816187	133.8216251	114.1864043
ENSG00000164125	FAM198B	767.8019808	462.4229373	444.5713378	1190.546254	1000.913229	1017.122481	138.9096567	88.04956971	136.4680676	172.1154942	115.8867681	153.0943642
ENSG00000164128	NPY1R	2.812461468	3.50320407	1.628466439	0.837233653	2.311577897	0	222.6640086	131.2436983	254.1776378	136.6799513	46.906549	169.1650434
ENSG00000164129	NPY5R	0	0	1.628466439	0	0	0	15.32091802	11.62918845	14.06887294	15.81943881	6.898021912	22.83728085
ENSG00000164134	NAA15	2684.494472	1793.640484	2344.991672	2950.411393	2406.35259	2843.086859	1592.35408	973.5292048	1721.092123	1658.509965	1055.397352	1880.269457
ENSG00000164136	IL15	255.9339936	353.8236111	289.0527929	50.23401917	90.15153796	50.45777319	140.9524458	132.9050109	92.85456143	30.37332251	30.35129641	7.612426951
ENSG00000164142	FAM160A1	534.367679	917.8394665	605.7895153	478.0604158	737.393349	548.5860153	373.8303998	539.9266067	350.3149363	422.695405	728.4311139	406.8419293
ENSG00000164144	ARFIP1	448.5876042	560.5126513	510.5242286	435.3614995	414.9282324	466.6395566	306.4183605	305.681525	274.8119848	251.2126883	241.4307669	279.1223215
ENSG00000164151	ICE1	4318.534585	4182.82566	4567.034128	3764.202503	3790.98775	3760.052557	4502.30711	4836.081084	4296.633797	3967.515253	4391.280749	4108.173078
ENSG00000164161	HHIP	0	0	0	0	0	0	3.064183605	0	2.344812157	0	0	4.229126084
ENSG00000164162	ANAPC10	89.99876699	59.5544692	52.92515926	69.49039319	68.19154795	88.77533027	64.3478557	33.22625272	42.20661883	67.07442055	71.73942788	77.81591994
ENSG00000164163	ABCE1	2512.934322	1509.880954	2120.263303	2885.944401	2087.354841	2814.633228	1438.123505	905.4153867	1456.12835	1574.35055	1092.646671	1769.466353
ENSG00000164164	OTUD4	3470.577452	2739.505583	3313.11497	1934.009738	1536.043512	2075.976954	1799.69717	1448.664619	1624.0169	1202.27735	1106.442715	1361.778599
ENSG00000164167	LSM 6.00	389.5259134	325.7979785	394.0888782	298.0551804	247.3388349	295.9177676	210.4072742	284.0844608	223.6950798	208.8165923	206.9406573	245.2893129
ENSG00000164168	TMEM184C	610.3041386	665.6087734	639.1730773	538.3412388	533.9744941	588.8004811	426.9429156	440.2478486	411.7490148	380.299309	452.5102374	438.9832875
ENSG00000164169	PRMT9	127.9669968	140.1281628	158.7754778	156.5626931	181.4588649	169.204262	99.07526988	101.3400708	91.91663656	125.2899554	110.3683506	131.9487338
ENSG00000164171	ITGA2	25222.15445	29794.75062	34870.35186	20696.4159	18246.44013	29716.59335	3599.394341	3056.81525	4436.853564	2989.873935	2851.642258	4141.160261
ENSG00000164172	MOCS2	696.0842134	683.1247937	495.8680306	831.3730173	729.3028264	734.1036926	369.7448216	448.5544118	405.1835408	394.2204151	427.6773585	525.2574596
ENSG00000164176	EDIL3	7.031153671	0	10.58503185	0.837233653	1.155788948	4.55258104	11.23533988	24.91968954	18.28953483	18.98332657	15.17564821	10.99572782
ENSG00000164180	TMEM161B	877.4879781	777.7113036	817.4901523	728.393278	693.473369	794.4253914	1027.522902	898.7701361	981.0694066	1113.688492	1163.006494	1058.127346
ENSG00000164181	ELOVL7	369.8386831	532.4870187	390.8319453	268.7520026	251.9619907	272.0167171	432.0498883	521.6521677	450.6728966	372.7059784	333.8642605	421.220958
ENSG00000164182	NDUFAF2	403.5882207	217.1986524	413.6304755	484.758285	435.7324335	445.7735601	374.8517943	259.1647712	355.0045606	432.1870683	253.8472063	414.4543562
ENSG00000164185	ZNF474	1.406230734	0	0.814233219	0.837233653	0	0.758763507	5.106972674	9.967875817	1.875849726	2.53111021	0	1.691650434
ENSG00000164187	LMBRD2	1060.297974	1604.467464	1230.306395	1045.704832	1398.504627	1203.01954	1625.038705	1581.56963	1621.672088	1477.535585	1643.108819	1808.374313
ENSG00000164188	RANBP3L	0	0	0	0.837233653	2.311577897	0	1.021394535	0	1.875849726	7.593330629	1.379604382	0.845825217
ENSG00000164190	NIPBL	6247.883152	7030.930569	6750.807622	6405.674678	6730.159046	6464.285695	8376.456581	7721.781133	7547.481372	7981.856046	8390.753853	8264.558193
ENSG00000164199	ADGRV1	18.28099954	28.02563256	21.98429693	20.93084132	79.74943743	25.79795922	267.6053681	282.4231481	156.1644897	263.2354618	313.1701948	220.7603816
ENSG00000164209	SLC25A46	749.5209813	718.1568344	717.3394663	782.8134654	891.1132791	784.9408476	1205.245551	1295.823856	1140.985596	1208.605125	1219.570274	1239.133943
ENSG00000164211	STARD4	1188.26497	1271.663078	1047.10392	663.0890531	851.8164549	739.0356554	570.959545	1051.610899	627.9406957	384.7287518	762.9212234	575.1611474
ENSG00000164219	PGGT1B	781.8642882	718.1568344	741.7664629	686.5315954	634.5281326	671.8850851	805.880288	684.4608061	673.4300516	804.2602691	674.6265429	716.4139586
ENSG00000164221	CCDC112	54.84299863	105.0961221	64.32442434	56.93188839	104.0210053	101.6743099	36.77020326	14.95181373	16.4136851	24.04554699	57.94338406	30.4497078
ENSG00000164236	ANKRD33B	336.0891455	444.9069169	225.5426018	269.5892362	275.0777697	248.1156667	63.32646116	76.42038126	101.7648476	67.07442055	74.49863664	155.6318399
ENSG00000164237	CMBL	229.2156097	339.8107948	225.5426018	284.659442	392.9682424	263.2909368	91.92550814	174.4378268	109.737209	134.1488411	246.9491844	178.4691207
ENSG00000164241	C5orf63	288.2773005	192.6762239	242.6414994	238.6115911	230.0020007	244.7012309	309.4825441	280.7618355	319.8323783	296.1398945	333.8642605	299.4221267
ENSG00000164244	PRRC1	1812.631416	1671.028342	1657.778835	1496.136538	1549.91298	1494.764108	1597.461053	1541.698126	1560.706972	1642.057748	1542.397699	1859.123826
ENSG00000164251	F2RL1	2591.683243	1800.646892	2314.865043	1644.326894	1159.256315	1649.172482	291.0974424	307.3428377	607.3063487	263.2354618	274.5412721	575.1611474
ENSG00000164252	AGGF1	316.4019152	374.8428355	337.9067861	344.940265	327.0882724	304.2641662	398.3438686	428.6186601	372.825133	351.1915416	393.187249	437.2916371
ENSG00000164253	WDR41	473.8997574	486.9453658	369.6618816	474.7114812	453.0692677	374.4497905	606.7083537	573.1528595	634.9751322	570.1325747	629.0995983	640.2896891
ENSG00000164256	PRDM9	216.5595331	266.2435094	227.1710682	72.00209415	112.111528	73.60006014	2.04278907	0	3.28273702	1.265555105	2.759208765	2.53747565
ENSG00000164258	NDUFS4	509.0555258	672.6151815	503.1961296	649.6933146	553.6229062	496.9900968	352.3811145	328.939902	361.5700347	380.9320865	378.0116008	402.6128032
ENSG00000164265	SCGB3A2	0	0	2.442699658	3.348934611	3.467366845	0.379381753	1.021394535	9.967875817	3.751699452	3.163887762	20.69406573	6.766601734
ENSG00000164266	SPINK1	16.87476881	17.51602035	38.26896131	12.55850479	16.18104528	32.24744903	15.32091802	4.983937908	11.72406079	11.38999594	8.277626294	3.383300867
ENSG00000164270	HTR4	4.218692202	14.01281628	4.885399317	0	1.155788948	1.896908767	0	0	0.468962431	0	6.898021912	0
ENSG00000164284	GRPEL2	1656.539805	1495.868138	1650.450736	1472.693995	1250.563642	1733.774613	1536.17738	1319.082233	1583.686131	1359.206183	940.8901887	1504.723061

**ENSG00000164287**	CDC20B	1.406230734	0	1.628466439	2.511700959	2.311577897	1.517527013	2.04278907	1.661312636	0.937924863	3.163887762	0	0
**ENSG00000164291**	ARSK	60.46792157	112.1025303	57.81055858	77.02549606	83.21680428	62.5979893	93.96829721	78.0816939	86.28908739	59.48108992	106.2295374	99.80737558
**ENSG00000164292**	RHOBTB3	1916.692491	592.0414879	927.411637	1158.731376	589.4523636	658.227342	31513.08558	20900.97428	30524.2957	26126.75236	17620.30717	27575.59372
**ENSG00000164294**	GPX8	707.3340593	497.454978	656.2719749	555.0859119	695.7849469	699.2005713	891.6774289	936.9803268	914.0077789	761.8641731	749.1251796	867.8166724
**ENSG00000164296**	TIGD6	127.9669968	143.6313669	131.0915483	119.7244124	184.9262317	134.3011407	223.6854031	207.6640795	169.2954378	171.4827167	194.5242179	168.3192181
**ENSG00000164300**	SERINC5	2154.345485	2378.675564	1981.843656	2434.675463	2555.449365	2596.868101	676.1631821	696.0899945	887.7458827	832.1024814	863.6323433	1012.452784
**ENSG00000164303**	ENPP6	0	0	2.442699658	0	0	0	6.128367209	4.983937908	1.406887294	0	6.898021912	5.920776517
**ENSG00000164304**	CAGE1	2.812461468	7.006408141	4.071166097	1.674467306	4.623155793	5.311344546	11.23533988	21.59706427	7.503398903	5.062220419	9.657230676	8.458252168
**ENSG00000164305**	CASP3	738.2711354	613.0607123	721.4106324	632.9486416	468.094524	648.7427982	314.5895167	397.05372	384.0802314	277.7893455	331.1050518	390.7712502
**ENSG00000164306**	PRIMPOL	309.3707615	248.727489	291.4954926	222.7041517	283.1682923	195.381603	268.6267627	197.6962037	202.5917704	196.7938188	194.5242179	201.3064016
**ENSG00000164307**	ERAP1	8758.005012	9234.44593	9197.578447	2329.184022	3215.404854	2892.406487	6742.225325	7643.699439	7047.098457	2723.474585	3013.055971	2913.867872
**ENSG00000164308**	ERAP2	1867.474415	2168.48332	1906.119967	357.4987698	462.3155793	392.2807329	1359.476126	1270.904167	1238.060819	456.2326153	354.5583263	484.6578492
**ENSG00000164309**	CMYA5	32.34330689	14.01281628	35.01202844	17.58190671	19.64841212	13.65774312	21.44928523	59.8072549	40.79973154	29.74054496	38.6289227	25.3747565
**ENSG00000164318**	EGFLAM	15.46853808	28.02563256	28.49816268	23.44254228	28.89472371	30.35054026	79.66877372	83.06563181	98.01314817	75.93330629	77.25784541	110.8031034
**ENSG00000164323**	CFAP97	936.5496689	980.8971397	885.8857428	987.9357104	983.576395	930.6234409	599.558592	639.6053649	573.0720912	621.3875564	540.8049179	593.7693022
**ENSG00000164327**	RICTOR	6641.627757	8379.664136	5665.434741	4102.444899	4876.273573	4080.250757	4108.048819	4221.395408	3523.78371	3409.405452	3632.498339	3338.472131
**ENSG00000164329**	PAPD4	915.4562079	917.8394665	862.2729794	860.6761951	832.1680428	770.9037227	1267.550618	1142.983094	1120.351249	1263.023995	1149.21045	1050.514919
**ENSG00000164331**	ANKRA2	196.8723028	203.1858361	223.9141353	225.2158526	204.5746438	208.6599643	237.9849266	259.1647712	266.8396235	254.3765761	215.2182836	290.1180494
**ENSG00000164332**	UBLCP1	871.8630552	767.2016914	764.5649931	849.7921577	713.1217811	770.9037227	723.1473307	627.9761765	690.3126991	700.4847505	568.3970055	766.3176464
**ENSG00000164338**	UTP15	998.4238213	641.0863449	990.1075948	1138.637768	813.6754196	1119.176172	687.398522	556.5397331	823.0290672	769.4575037	627.719994	920.2578359
**ENSG00000164342**	TLR3	1126.390818	1177.076568	769.4503924	157.3999267	239.2483123	116.0908165	953.9824956	1407.131803	763.9398008	423.9609601	633.2384115	328.1801841
**ENSG00000164346**	NSA2	1913.880029	1734.086015	1796.198482	2114.014973	1846.950739	1800.166419	1599.503842	1872.299341	1516.624503	1600.29443	1727.264687	1838.824021
**ENSG00000164347**	GFM2	715.7714437	641.0863449	788.9919896	936.8644576	711.9659921	817.1882966	1083.699602	930.3350762	1045.31726	1146.592925	1001.592782	1246.74637
**ENSG00000164362**	TERT	49.2180757	31.52883663	60.25325824	66.97869223	34.67366845	75.87635066	64.3478557	66.45250545	71.28228958	93.0183002	73.11903226	74.43261908
**ENSG00000164366**	CCDC127	352.9639143	388.8556518	414.4447087	333.2189938	432.2650667	391.1425877	360.5522708	513.3456046	358.2872976	308.7954456	524.2496653	351.017465
**ENSG00000164379**	FOXQ1	94.21745919	112.1025303	72.46675653	102.1425056	136.3830959	87.25780326	5.106972674	4.983937908	20.63434698	2.53111021	6.898021912	10.99572782
**ENSG00000164385**	LINC01600	7.031153671	14.01281628	21.98429693	7.535102876	15.02525633	13.27836137	0	4.983937908	3.28273702	3.163887762	2.759208765	1.691650434
**ENSG00000164393**	ADGRF2	4.218692202	10.50961221	9.770798633	8.372336529	17.33683422	7.208253313	1.021394535	6.645250545	1.875849726	1.898332657	2.759208765	3.383300867
**ENSG00000164400**	CSF2	4.218692202	7.006408141	13.02773151	6.697869223	3.467366845	16.69279715	0	0	0.468962431	0	0	0.845825217
**ENSG00000164402**	8-Sep	939.3621304	1324.211139	1038.961588	1011.378253	1148.854215	1041.782295	3023.327823	2948.829929	2928.201422	2576.670193	2664.016062	2587.379338
**ENSG00000164403**	SHROOM1	77.34269038	150.637775	144.1192798	86.23506625	116.7346838	106.9856544	118.481766	94.69482026	117.7095703	132.2505084	129.6828119	104.8823269
**ENSG00000164404**	GDF9	30.93707615	0	16.28466439	5.023401917	2.311577897	8.346398573	11.23533988	18.274439	12.66198565	16.45221636	11.03683506	16.07067912
**ENSG00000164405**	UQCRQ	922.4873616	977.3939356	861.4587462	1028.122926	1064.481621	1061.130764	712.9333853	757.5585621	845.0703015	796.0341609	716.0146744	986.2322028
**ENSG00000164406**	LEAP2	2.812461468	21.01922442	17.91313083	10.88403749	4.623155793	9.484543833	12.25673442	16.61312636	4.220661883	6.327775524	20.69406573	18.60815477
**ENSG00000164411**	GJB7	33.74953762	3.50320407	26.86969624	50.23401917	27.73893476	28.83301325	53.11251581	43.19412854	46.42728071	55.68442461	44.14734023	38.90795997
**ENSG00000164414**	SLC35A1	61.8741523	91.08330583	61.88172468	73.67656145	68.19154795	63.73613456	102.1394535	78.0816939	110.2061714	100.6116308	140.719647	112.4947538
**ENSG00000164418**	GRIK2	54.84299863	80.57369362	54.5536257	128.9339825	141.0062517	103.9506004	2.04278907	4.983937908	0	0.632777552	0	0
**ENSG00000164430**	CGAS	490.7745262	770.7048955	582.1767519	251.1700959	429.9534888	448.8086142	252.2844501	249.1968954	247.6121638	199.9577066	246.9491844	201.3064016
**ENSG00000164434**	FABP7	0	0	0	0	0	0	0	0	1.875849726	5.062220419	1.379604382	0
**ENSG00000164442**	CITED2	2719.65024	3040.781133	2675.570359	2931.155019	3265.103779	3575.293643	2593.320724	3312.657396	2310.5779	2457.708013	3604.906251	2151.779351
**ENSG00000164463**	CREBRF	779.0518267	1285.675894	901.3561739	310.6136852	685.3828463	414.2848746	926.4048431	1083.175839	810.3670816	697.9536403	845.6974864	604.76503
**ENSG00000164465**	DCBLD1	329.0579918	364.3332233	348.4918179	271.2637035	332.8672171	307.6786019	304.3755714	451.877037	332.0254015	267.0321271	406.9832928	306.1887285
**ENSG00000164466**	SFXN1	1905.442645	2094.916034	1850.752108	2049.547982	2108.159042	2092.669751	1349.262181	1294.162544	1456.597312	1421.218383	1380.983987	1597.763834
**ENSG00000164484**	TMEM200A	8.437384405	0	9.770798633	4.186168264	9.246311586	13.65774312	84.77574639	63.12988017	33.29633263	55.05164706	23.4532745	22.83728085
**ENSG00000164485**	IL22RA2	2.812461468	7.006408141	1.628466439	7.535102876	0	3.41443578	0	0	1.406887294	1.265555105	0	0
**ENSG00000164494**	PDSS2	367.0262216	325.7979785	379.4326803	334.8934611	389.5008756	364.2064832	332.9746184	312.3267756	310.4531296	308.7954456	350.4195131	330.7176598
**ENSG00000164506**	STXBP5	888.737824	1089.496466	1154.582705	1289.339825	1137.296325	1268.273201	1371.73286	1212.758224	1355.770389	1294.662872	1336.836646	1458.202674
**ENSG00000164508**	HIST1H2AA	11.24984587	0	10.58503185	5.86063557	9.246311586	9.484543833	2.04278907	1.661312636	1.875849726	0.632777552	2.759208765	4.229126084
**ENSG00000164512**	ANKRD55	0	0	0	0	0	0	4.08557814	4.983937908	3.28273702	1.898332657	11.03683506	5.920776517
**ENSG00000164520**	RAET1E	39.37446056	38.53524477	18.72736405	51.90848648	24.27156791	21.62475994	20.4278907	31.56494009	31.42048291	18.98332657	9.657230676	31.29553302
**ENSG00000164532**	TBX20	0	0	10.58503185	12.55850479	6.93473369	15.17527013	17.36370709	1.661312636	11.72406079	21.51443678	16.55525259	11.84155303
**ENSG00000164535**	DAGLB	341.7140684	196.1794279	319.9936552	402.709387	351.3598403	423.3900367	337.0601965	455.1996623	337.1839882	359.4176498	551.8417529	367.9339693
**ENSG00000164542**	KIAA0895	271.4025317	518.4742024	231.2422343	550.0625099	815.9869975	396.4539322	433.0712828	814.0431917	360.6321098	446.740952	909.1592879	470.2788205
**ENSG00000164543**	STK17A	1227.639431	1057.967629	1189.594734	1028.122926	857.5953996	902.5491911	868.1853546	819.0271296	760.1881014	722.6319648	782.2356848	908.4162828
**ENSG00000164548**	TRA2A	1551.0725	1678.03475	1656.964602	1535.486519	1478.254065	1458.34346	1502.471361	1342.34061	1417.67343	1554.101669	1398.918844	1497.956459
**ENSG00000164574**	GALNT10	629.9913689	581.5318757	631.8449783	844.7687557	1146.542637	965.9059439	264.5411845	224.2772059	300.6049186	365.1126477	350.4195131	308.7262041
**ENSG00000164576**	SAP30L	1101.078665	1233.127833	1060.131652	887.467672	972.0185055	955.6626366	996.881066	1134.67653	1032.655274	902.3407897	956.0658369	926.1786124
**ENSG00000164587**	RPS14	7782.080883	6557.99802	7414.407696	7589.523063	6717.445367	7838.406405	6626.807742	7397.825169	7868.720637	6885.252547	7350.532149	7433.112005
**ENSG00000164597**	COG5	843.7384405	753.1888751	837.0317496	852.3038586	924.6311586	864.6110158	1057.143344	1053.272211	1025.151875	1083.31517	913.2981011	1007.377833
**ENSG00000164603**	BMT2	84.37384405	136.6249587	101.7791524	119.7244124	143.3178296	94.84543833	321.7392785	435.2639107	297.791144	334.1065477	600.1279063	309.5720293
**ENSG00000164604**	GPR85	0	7.006408141	2.442699658	2.511700959	2.311577897	2.655672273	15.32091802	14.95181373	13.13094808	30.37332251	20.69406573	15.2248539
**ENSG00000164609**	SLU7	1312.013275	1664.021933	1362.212176	1210.639862	1395.037261	1214.780374	1312.491977	1465.277745	1164.433717	1249.102888	1385.1228	1239.979768
**ENSG00000164610**	RP9	142.0293041	175.1602035	118.0638168	139.81802	161.8104528	114.9526713	105.2036371	74.75906863	113.019946	70.87108587	126.9236032	117.5697051
**ENSG00000164611**	PTTG1	1314.825736	879.3042217	1123.641843	1626.744988	1605.390849	1517.527013	972.3675972	815.7045043	1145.206258	1018.771859	922.9553318	1203.609283
**ENSG00000164615**	CAMLG	326.2455303	364.3332233	315.1082559	352.4753679	318.9977497	382.7961891	471.8842751	415.328159	484.9071541	446.1081744	372.4931832	456.7456171
**ENSG00000164619**	BMPER	92.81122845	378.3460396	320.8078885	176.6563008	343.2693176	459.4313033	48.00554314	166.1312636	70.81332715	68.97275321	150.3768777	102.3448512
**ENSG00000164620**	RELL2	115.3109202	80.57369362	128.6488487	125.5850479	137.5388848	139.2331035	58.21948849	74.75906863	64.71681554	62.01220013	74.49863664	70.20349299

**ENSG00000164621**	SMAD5-AS1	4.218692202	17.51602035	4.071166097	1.674467306	5.778944741	0	2.04278907	1.661312636	0	4.429442867	5.518417529	2.53747565
**ENSG00000164626**	KCNK5	0	0	0	0	0	1.896908767	2.04278907	0	2.813774589	2.53111021	2.759208765	0
**ENSG00000164631**	ZNF12	891.5502855	1138.541323	931.482803	920.1197845	948.9027265	813.0150973	1171.539532	1249.307102	1001.703754	1023.201302	1179.561747	1081.810452
**ENSG00000164638**	SLC29A4	0	0	3.256932878	11.72127114	13.86946738	5.311344546	0	0	0.468962431	3.796665314	1.379604382	4.229126084
**ENSG00000164647**	STEAP1	89.99876699	94.5865099	146.5619795	147.3531229	135.2273069	173.756843	0	6.645250545	5.158586746	3.163887762	0	3.383300867
**ENSG00000164649**	CDCA7L	254.5277629	245.2242849	258.9261638	253.6817968	375.6314082	266.7053726	440.2210445	593.0886111	357.8183352	445.4753969	664.9693123	381.4671728
**ENSG00000164651**	SP8	7.031153671	0	3.256932878	0.837233653	2.311577897	1.13814526	0	0	0	0	1.379604382	5.074951301
**ENSG00000164654**	MIOS	819.832518	697.13761	818.3043855	1098.450553	974.3300834	962.1121264	1105.148887	1023.368584	1165.840605	1066.862953	976.7599027	1055.589871
**ENSG00000164659**	KIAA1324L	97.02992066	112.1025303	95.26528668	156.5626931	143.3178296	115.7114348	160.358942	176.0991394	98.95107304	173.3810494	161.4137127	95.5782495
**ENSG00000164663**	USP49	137.8106119	227.7082646	160.4039442	185.0286373	202.2630659	153.2702283	321.7392785	265.8100218	290.7567075	337.903213	400.0852709	288.4263989
**ENSG00000164669**	INTS4P1	9.843615139	10.50961221	12.21349829	6.697869223	9.246311586	9.484543833	29.62044151	49.83937908	28.13774589	16.45221636	44.14734023	24.52893129
**ENSG00000164674**	SYTL3	327.6517611	427.3908966	309.4086234	219.3552171	306.2840713	246.5981396	69.45482837	112.9692593	51.58586746	54.4188695	115.8867681	55.82446431
**ENSG00000164675**	IQUB	23.90592248	10.50961221	17.09889761	30.1404115	27.73893476	13.27836137	27.57765244	51.50069172	23.917084	24.67832454	28.97169203	17.76232955
**ENSG00000164683**	HEY1	338.9016069	395.86206	113.1784175	610.3433329	1051.767943	227.2496702	327.8676457	1010.078083	121.4612697	299.3037823	1583.785831	179.314946
**ENSG00000164684**	ZNF704	1458.261271	1397.778424	876.9291773	2930.317785	2556.605154	2039.935688	5787.221435	5827.884728	5967.54694	6304.362754	6106.128996	6533.9998
**ENSG00000164687**	FABP5	464.0561423	444.9069169	336.2783196	543.3646407	481.9639914	468.9158471	1300.235243	3392.400403	1427.521641	1454.755593	2952.353378	1750.858199
**ENSG00000164691**	TAGAP	0	0	4.885399317	0.837233653	0	2.27629052	2.04278907	0	0	0	0	2.53747565
**ENSG00000164692**	COL1A2	208.1221487	56.05126513	170.1747429	418.6168264	217.2883223	439.3240703	4431.830887	4693.208197	6013.505259	4618.643355	4963.816568	6602.511642
**ENSG00000164695**	CHMP4C	1116.547203	1289.179098	1285.674254	950.260196	1019.405852	1000.050302	840.6077022	757.5585621	833.3462407	789.7063854	820.8646075	731.6388125
**ENSG00000164707**	SLC13A4	29.53084542	7.006408141	28.49816268	41.86168264	35.8294574	45.5258104	20.4278907	18.274439	18.28953483	25.3111021	22.07367012	40.59961041
**ENSG00000164713**	BRI3	988.5802061	679.6215897	722.2248656	648.0188473	517.7934488	586.1448089	841.6290967	837.3015686	832.8772783	720.7336322	696.7002131	859.3584202
**ENSG00000164715**	LMTK2	1300.763429	1131.534915	1235.191794	1152.87074	974.3300834	1228.817499	847.7574639	784.1395643	863.3598363	866.9052468	783.6152892	897.420555
**ENSG00000164733**	CTSB	1354.200197	1611.473872	1607.296375	1129.428198	1336.092024	1347.943369	369.7448216	425.2960348	338.5908755	381.5648641	344.9010956	345.0966884
**ENSG00000164741**	DLC1	2303.405943	1947.781463	2742.337483	1949.079944	2119.716931	2643.532057	303.3541769	441.9091612	370.9492833	265.766572	415.2609191	349.3258145
**ENSG00000164742**	ADCY1	18.28099954	24.52242849	47.22552673	20.09360767	13.86946738	25.79795922	27.57765244	18.274439	20.16538455	27.8422123	22.07367012	37.21630954
**ENSG00000164743**	C8orf48	67.49907524	87.58010176	62.6959579	69.49039319	75.12628164	73.22067839	0	0	0.468962431	3.163887762	2.759208765	1.691650434
**ENSG00000164744**	SUN3	136.4043812	199.682632	184.0167076	88.7467672	152.5641412	196.5197482	10.21394535	18.274439	8.441323766	16.45221636	9.657230676	19.45397999
**ENSG00000164746**	C7orf57	15.46853808	49.04485699	32.56932878	17.58190671	38.14103529	32.62683078	7.149761744	11.62918845	4.220661883	8.226108181	17.93485697	4.229126084
**ENSG00000164749**	HNF4G	82.96761332	87.58010176	65.95289078	10.88403749	3.467366845	22.00414169	3.064183605	0	0	1.898332657	2.759208765	3.383300867
**ENSG00000164751**	PEX2	440.1502198	507.9645902	429.9151399	559.2720801	550.1555394	495.0931881	346.2527473	383.7632189	285.1291583	309.4282231	406.9832928	313.8011554
**ENSG00000164754**	RAD21	4963.994492	4319.450619	4370.803922	5658.025026	5450.70068	4998.733982	4275.557523	4581.90025	4180.800076	4344.017897	4512.685935	4928.623538
**ENSG00000164756**	SLC30A8	179.997534	213.6954483	169.3605096	290.5200775	271.6104028	236.7342141	239.0063212	245.8742701	191.336672	301.8348925	311.7905904	198.7689259
**ENSG00000164758**	MED30	118.1233817	150.637775	79.79485551	101.305272	71.65891479	88.39594852	107.2464262	59.8072549	74.5650266	64.54331034	56.56377967	96.42407471
**ENSG00000164761**	TNFRSF11B	39.37446056	339.8107948	344.4206518	23.44254228	158.3430859	321.7157268	12.25673442	19.93575163	73.62710174	14.5538837	9.657230676	57.51611474
**ENSG00000164776**	PHKG1	9.843615139	14.01281628	6.513865756	8.372336529	5.778944741	9.105162079	5.106972674	18.274439	13.59991051	8.858885733	8.277626294	10.99572782
**ENSG00000164778**	EN2	9.843615139	3.50320407	4.885399317	5.86063557	9.246311586	10.24330734	20.4278907	19.93575163	40.79973154	28.47498986	22.07367012	31.29553302
**ENSG00000164808**	SPIDR	968.8929758	1152.554139	917.6408383	938.5389249	998.6016513	918.4832248	796.6877372	744.268061	839.4427523	864.3741366	881.5672003	799.3048299
**ENSG00000164815**	ORC5	338.9016069	304.7787541	351.7487508	375.0806765	325.9324834	363.0683379	401.4080522	348.8756536	355.0045606	369.5420906	285.5781071	422.0667832
**ENSG00000164818**	DNAAF5	531.5552175	448.410121	495.8680306	530.8061359	609.1007757	573.2458292	501.5047166	387.0858442	528.0516978	513.8153725	455.2694462	510.0326057
**ENSG00000164823**	OSGIN2	1001.236283	1268.159873	1258.804557	819.6517462	739.7049269	953.0069643	916.1908978	915.3832625	908.3802297	767.559171	728.4311139	932.0993889
**ENSG00000164825**	DEFB1	0	7.006408141	1.628466439	0	0	0	1.021394535	1.661312636	3.28273702	1.898332657	2.759208765	9.304077385
**ENSG00000164828**	SUN1	5201.647486	5380.921452	5039.289395	5439.507043	4788.433613	5440.713724	2938.552077	3875.84238	2968.532191	3186.667754	3745.625898	3137.165729
**ENSG00000164830**	OXR1	915.4562079	1327.714343	996.6214606	1090.078216	1145.386848	1000.050302	1024.458718	953.5934531	988.5728055	1053.574625	958.8250457	1126.639189
**ENSG00000164845**	FAM86FP	8.437384405	10.50961221	7.328098975	7.535102876	2.311577897	5.311344546	24.51346884	13.29050109	25.3239713	28.47498986	28.97169203	19.45397999
**ENSG00000164849**	GPR146	1.406230734	73.56728548	10.58503185	11.72127114	35.8294574	14.79588838	7.149761744	38.21019063	11.25509835	6.960553076	24.83287888	7.612426951
**ENSG00000164855**	TMEM184A	195.466072	171.6569995	194.6017394	251.1700959	343.2693176	271.6373354	324.8034621	523.3134804	331.0874766	349.2932089	446.9918199	300.267952
**ENSG00000164867**	NOS3	77.34269038	115.6057343	109.9214846	25.95424324	41.60840214	40.97322936	0	6.645250545	3.28273702	0	1.379604382	5.074951301
**ENSG00000164877**	MICALL2	99.84238213	133.1217547	127.0203822	130.6084498	147.9409854	103.1918369	37.79159779	83.06563181	49.2410553	41.1305409	56.56377967	29.60388259
**ENSG00000164879**	CA3	0	10.50961221	3.256932878	4.186168264	9.246311586	10.24330734	8.171156279	18.274439	5.158586746	6.327775524	9.657230676	7.612426951
**ENSG00000164880**	INTS1	1226.2332	1040.451609	1132.598408	1169.615413	1343.026758	1396.504234	1135.790723	1051.610899	1264.791678	1232.650672	1288.550493	1099.572782
**ENSG00000164885**	CDK5	150.4666886	143.6313669	158.7754778	186.7031046	263.5198802	203.3486198	154.2305748	134.5663235	170.702325	161.3582759	150.3768777	230.9102842
**ENSG00000164889**	SLC4A2	1347.169043	1100.006078	1240.077193	1557.254594	1360.363592	1607.819871	799.7519208	711.0418083	969.3453458	951.0646612	848.4566951	1028.523464
**ENSG00000164896**	FASTK	426.0879124	280.2563256	435.6147724	399.3604524	381.4103529	451.8436682	353.4025091	347.214341	416.4386391	417.6331846	362.8359525	370.4714449
**ENSG00000164897**	TMUB1	194.0598413	252.2306931	190.5305734	214.3318151	199.951488	228.7671972	153.2091802	139.5502614	201.6538455	170.8499391	150.3768777	205.5355277
**ENSG00000164898**	FMC1	5.624922937	3.50320407	0.814233219	3.348934611	5.778944741	4.55258104	1.021394535	1.661312636	2.813774589	5.694997971	8.277626294	11.84155303
**ENSG00000164902**	PHAX	1122.172126	1040.451609	999.8783935	1059.937805	1013.626908	1053.163747	746.639405	800.7526906	748.933003	775.1525017	782.2356848	888.9623028
**ENSG00000164904**	ALDH7A1	1012.486129	907.3298542	849.2452479	731.7422126	837.9469875	695.7861356	1162.346981	1392.179989	1041.56556	1268.086215	1367.187943	1261.125398
**ENSG00000164916**	FOXK1	3884.009288	2448.739645	3250.419012	2166.760694	2182.129534	2423.111258	2840.498201	2003.543039	3155.648201	2565.912975	2160.460463	2576.38361
**ENSG00000164919**	COX6C	1022.329744	854.7817932	884.2572763	997.9825142	962.7721939	859.679053	717.0189635	795.7687527	717.9814826	855.5152508	787.7541023	908.4162828
**ENSG00000164920**	OSR2	286.8710698	224.2050605	276.0250614	524.1082667	342.1135287	486.3674077	325.8248566	257.5034586	302.4807683	255.0093536	168.3117346	241.0601868
**ENSG00000164924**	YWHAZ	19166.92491	17897.8696	17950.58556	22030.12911	19851.83098	20770.01285	8379.520764	7846.37958	9267.63557	8569.706392	8138.286251	10024.72047
**ENSG00000164930**	FZD6	691.8655212	707.6472222	681.5132047	802.0698394	758.1975501	706.0294429	680.2487602	717.6870588	750.8088528	770.7230588	667.728521	884.7331767
**ENSG00000164932**	CTHRC1	5.624922937	0	0	1.674467306	1.155788948	0	2.04278907	0	4.689624315	3.796665314	2.759208765	0.845825217
**ENSG00000164933**	SLC25A32	28.12461468	38.53524477	11.39926507	5.86063557	13.86946738	23.90105046	21.44928523	6.645250545	21.10330942	24.04554699	6.898021912	18.60815477
**ENSG00000164934**	DCAF13	1051.860589	686.6279978	1004.763793	1013.889954	874.9322338	1125.625662	634.2860062	440.2478486	693.1264737	680.8686464	547.7029398	857.6667698
**ENSG00000164938**	TP53INP1	171.5601496	224.2050605	227.9853014	145.6786556	173.3683422	150.6145561	425.921521	651.2345534	408.9352402	434.0854009	584.9522581	445.7498892
**ENSG00000164941**	INTS8	824.0512102	910.8330583	689.6555369	854.8155596	717.7449369	754.2109256	726.2115143	809.0592538	599.8029498	627.715332	659.4508947	678.3518239

**ENSG00000164944**	VIRMA	1659.352266	1744.595627	1733.502524	1724.701325	1782.226558	1682.558076	1242.015754	1383.873426	1204.295524	1304.787313	1361.669525	1323.716464
**ENSG00000164946**	FREM1	52.03053716	122.6121425	79.79485551	48.55955187	67.035759	49.69900968	10.21394535	3.322625272	10.31717349	2.53111021	1.379604382	9.304077385
**ENSG00000164949**	GEM	23.90592248	108.5993262	61.88172468	22.60530863	88.99574902	58.80417176	0	1.661312636	1.875849726	0	2.759208765	0
**ENSG00000164951**	PDP1	2706.994163	2960.20744	2967.880085	1098.450553	1178.904727	1214.780374	463.7131188	554.8784205	483.5002668	283.4843435	338.0030737	373.8547458
**ENSG00000164953**	TMEM67	163.1227652	154.1409791	142.4908134	195.0754411	248.4946239	183.2413868	306.4183605	315.6494009	276.2188721	341.6998783	364.2155569	302.8054276
**ENSG00000164961**	WASHC5	1258.576507	998.4131601	1186.337801	1099.287786	1134.984747	1166.598891	1291.042692	1285.85598	1184.599102	1215.565678	1266.476823	1166.392974
**ENSG00000164967**	RPP25L	95.62368992	66.56087734	89.56565414	72.00209415	94.77469376	84.60213099	84.77574639	84.72694444	105.0475846	90.48718999	108.9887462	91.34912341
**ENSG00000164970**	FAM219A	476.7122189	651.5959571	490.1683981	284.659442	403.3703429	438.1859251	250.241661	410.3442211	263.087924	293.6087843	333.8642605	264.7432929
**ENSG00000164972**	C9orf24	1.406230734	0	2.442699658	0.837233653	0	1.517527013	0	4.983937908	0.937924863	6.327775524	2.759208765	1.691650434
**ENSG00000164975**	SNAPC3	213.7470716	133.1217547	221.4714357	236.9371238	205.7304328	208.2805826	329.9104348	410.3442211	333.9012512	307.5298905	416.6405235	375.5463962
**ENSG00000164976**	MYORG	52.03053716	175.1602035	52.92515926	118.0499451	208.0420107	151.3733196	250.241661	249.1968954	301.5428434	489.137048	437.3345892	455.0539666
**ENSG00000164978**	NUDT2	210.9346101	217.1986524	258.9261638	270.4264699	206.8862217	243.1837039	247.1774774	229.2611438	225.1019671	243.6193577	216.597888	235.1394103
**ENSG00000164983**	TMEM65	88.59253625	245.2242849	140.862347	126.4222816	201.107277	161.9960087	329.9104348	1036.659085	309.0462423	330.9426599	975.3802983	357.7840667
**ENSG00000164985**	PSIP1	433.1190661	535.9902228	407.1166097	502.3401917	601.0102531	401.7652767	1118.427016	1126.369967	1029.372537	1166.841807	1106.442715	1183.309478
**ENSG00000164989**	CCDC171	15.46853808	17.51602035	29.3123959	19.25637402	20.80420107	16.69279715	84.77574639	98.01744553	63.30992825	86.69052468	77.25784541	59.20776517
**ENSG00000165006**	UBAP1	577.9608317	816.2465484	798.7627883	655.5539502	738.5491379	759.5222701	515.8042401	626.3148638	574.0100161	529.6348113	623.5811808	587.0027004
**ENSG00000165025**	SYK	4.218692202	31.52883663	24.42699658	1.674467306	13.86946738	11.3814526	3.064183605	1.661312636	4.689624315	3.163887762	13.79604382	5.074951301
**ENSG00000165028**	NIPSNAP3B	5.624922937	17.51602035	8.956565414	7.535102876	25.42735686	14.79588838	31.66323058	31.56494009	24.85500887	34.80276538	30.35129641	19.45397999
**ENSG00000165029**	ABCA1	856.3945171	1275.166282	1131.784175	1997.639496	2342.784198	2000.100603	1819.103667	2098.237859	1494.114307	1903.394878	2599.174656	1522.48539
**ENSG00000165030**	NFIL3	397.9632978	627.0735286	539.8366245	479.7348831	736.23756	571.7283022	369.7448216	529.9587309	288.8808578	283.4843435	547.7029398	263.0516424
**ENSG00000165046**	LETM2	132.185689	59.5544692	144.9335131	128.9339825	48.54313583	139.2331035	67.4120393	38.21019063	64.24785311	46.82553888	31.73090079	56.67028952
**ENSG00000165055**	METTL2B	660.9284451	553.5062431	648.1296427	727.5560443	632.2165547	814.1532426	600.5799865	468.4901634	580.1065277	604.9353401	522.8700609	601.3817291
**ENSG00000165060**	FXN	119.5296124	94.5865099	100.150686	151.5392912	90.15153796	154.0289918	108.2678207	96.3561329	128.9646686	130.3521758	91.05388923	146.3277625
**ENSG00000165061**	ZMAT4	0	0	0	0	0	0	1.021394535	8.306563181	0.468962431	1.265555105	2.759208765	0
**ENSG00000165071**	TMEM71	0	0	0	0	0	3.41443578	3.064183605	0	2.344812157	8.858885733	6.898021912	3.383300867
**ENSG00000165072**	MAMDC2	7.031153671	3.50320407	25.2412298	2.511700959	17.33683422	40.21446585	8.171156279	16.61312636	16.4136851	16.45221636	22.07367012	20.2998052
**ENSG00000165084**	C8orf34	8.437384405	21.01922442	6.513865756	8.372336529	10.40210053	6.070108053	0	0	0	0	0	0
**ENSG00000165091**	TMC1	15.46853808	3.50320407	30.12662912	49.39678552	36.98524634	36.04126656	7.149761744	6.645250545	9.379248629	10.75721839	17.93485697	8.458252168
**ENSG00000165092**	ALDH1A1	4.218692202	0	0.814233219	1.674467306	3.467366845	0.379381753	823.2439951	797.4300653	478.3416801	1131.406264	1038.8421	759.5510447
**ENSG00000165097**	KDM1B	710.1465207	735.6728548	730.3671978	771.0921943	931.5658923	852.0914179	689.441311	539.9266067	745.650266	691.6258648	688.4225868	767.1634716
**ENSG00000165102**	HGSNAT	390.9321441	381.8492437	381.0611467	399.3604524	510.8587151	429.4601447	509.6758729	431.9412854	448.7970469	550.5164706	507.6944127	456.7456171
**ENSG00000165105**	RASEF	974.5178988	1380.262404	1064.202818	881.6070365	1052.923732	999.67092	1246.101333	1272.565479	1174.750891	1389.579505	1283.032076	1281.425203
**ENSG00000165113**	GKAP1	50.62430643	80.57369362	60.25325824	56.09465474	152.5641412	67.90933384	184.8724108	187.7283279	153.3507151	201.8560392	158.654504	178.4691207
**ENSG00000165115**	KIF27	247.4966092	336.3075908	207.629471	190.0520392	211.5093775	161.9960087	361.5736653	431.9412854	329.6805893	257.5404638	306.2721729	282.5056224
**ENSG00000165118**	C9orf64	285.464839	213.6954483	333.83562	288.0083766	260.0525134	287.9507508	252.2844501	352.1982789	302.9497307	256.2749087	259.3656239	314.6469806
**ENSG00000165119**	HNRNPK	12717.95076	12331.27833	12496.03722	12880.83975	12576.13955	12356.08432	11664.32559	12760.54236	12309.3259	11494.40424	12020.49298	12430.24739
**ENSG00000165121**	AL353743.1	71.71776744	70.06408141	104.2218521	36.83828073	87.83996007	62.97737105	137.8882622	192.7122658	129.9025935	152.4993901	122.78479	148.0194129
**ENSG00000165124**	SVEP1	2323.093173	2312.114686	1999.756787	2026.10544	1960.218056	1674.970441	63.32646116	58.14594226	66.12370283	39.23220825	63.46180159	57.51611474
**ENSG00000165125**	TRPV6	18.28099954	17.51602035	17.91313083	41.86168264	52.01050267	47.42271916	59.24088302	38.21019063	69.87540229	63.91053279	57.94338406	95.5782495
**ENSG00000165138**	ANKS6	693.2717519	641.0863449	645.686943	911.747448	888.8017012	932.1409679	273.7337353	262.4873965	261.6810368	294.8743394	278.6800852	279.1223215
**ENSG00000165140**	FBP1	0	0	0.814233219	3.348934611	2.311577897	3.793817533	2.04278907	1.661312636	3.28273702	1.898332657	4.138813147	12.68737825
**ENSG00000165152**	TMEM246	0	0	0.814233219	3.348934611	0	0.379381753	0	1.661312636	0.937924863	3.163887762	0	0.845825217
**ENSG00000165156**	ZHX1	233.4343019	259.2371012	250.7838316	380.9413121	407.9934987	339.1672875	891.6774289	1033.33646	887.2769203	950.4318837	1033.323682	1004.840358
**ENSG00000165164**	CFAP47	0	0	0	5.023401917	0	0	3.064183605	3.322625272	4.220661883	1.265555105	5.518417529	4.229126084
**ENSG00000165168**	CYBB	11.24984587	3.50320407	44.78282707	15.07020575	50.85471372	39.45570234	12.25673442	4.983937908	3.751699452	17.08499391	11.03683506	5.920776517
**ENSG00000165169**	DYNLT3	607.4916772	669.1119775	628.5880454	641.3209781	647.241811	701.0974801	391.1941069	378.779281	397.2111794	393.5876376	317.3090079	429.6792101
**ENSG00000165171**	METTL27	70.31153671	73.56728548	79.79485551	56.09465474	64.7241811	53.11344546	41.87717593	26.58100218	52.05482989	44.29442867	46.906549	25.3747565
**ENSG00000165175**	MID1IP1	1030.767128	1029.941997	901.3561739	822.1634471	930.4101034	905.9636269	966.23923	717.6870588	997.4830917	887.1541284	637.3772246	758.7052194
**ENSG00000165181**	C9orf84	14.06230734	10.50961221	2.442699658	0	0	1.896908767	3.064183605	1.661312636	1.875849726	6.960553076	1.379604382	3.383300867
**ENSG00000165182**	CXorf58	12.65607661	10.50961221	8.956565414	5.86063557	11.55788948	11.3814526	9.192550814	16.61312636	5.158586746	11.38999594	9.657230676	15.2248539
**ENSG00000165185**	KIAA1958	594.8356005	528.9838146	525.1804265	781.1389981	713.1217811	730.3098751	912.1053196	1076.530588	889.6217325	908.0357877	1129.895989	1008.223658
**ENSG00000165186**	PTCHD1	0	0	0	10.88403749	0	0.379381753	1.021394535	0	0	0.632777552	1.379604382	0
**ENSG00000165188**	RNF183	1.406230734	7.006408141	0	4.186168264	0	0	1.021394535	0	0	1.898332657	0	3.383300867
**ENSG00000165195**	PIGA	302.3396078	444.9069169	389.2034789	295.5434795	323.6209055	278.0868252	353.4025091	423.6347222	360.6321098	335.3721028	391.8076446	318.0302815
**ENSG00000165197**	VEGFD	8.437384405	0	5.699632536	4.186168264	3.467366845	3.793817533	1.021394535	0	4.220661883	0	4.138813147	0.845825217
**ENSG00000165202**	OR1Q1	0	0	9.770798633	0	0	0.379381753	0	0	0	0	0	0
**ENSG00000165209**	STRBP	1126.390818	907.3298542	1202.622465	1337.062144	1243.628908	1382.846491	1109.234465	981.835768	1086.585954	1183.294023	1074.711814	1360.932774
**ENSG00000165215**	CLDN3	4.218692202	38.53524477	4.885399317	11.72127114	12.71367843	17.83094241	1.021394535	0	0	0	0	2.53747565
**ENSG00000165219**	GAPVD1	3116.207307	2988.233072	3098.1574	3410.889902	3349.476372	3527.870924	2983.493436	2900.651863	2889.746503	3025.309478	2697.126567	3255.581259
**ENSG00000165233**	CARD19	125.1545353	140.1281628	128.6488487	105.4914403	105.1767943	115.7114348	67.4120393	73.09775599	116.302683	111.3688492	99.33151553	87.96582254
**ENSG00000165238**	WNK2	1.406230734	7.006408141	8.956565414	5.023401917	17.33683422	9.105162079	648.5855296	1116.402091	152.8817527	568.234242	1116.099945	115.0322295
**ENSG00000165240**	ATP7A	157.4978422	182.1666117	151.4473788	165.7722633	218.4441112	151.3733196	157.2947584	181.0830773	133.1853305	134.7816187	148.9972733	134.4862095
**ENSG00000165244**	ZNF367	839.5197483	1390.772016	841.1029157	895.0027749	1096.843712	968.1822344	1114.341438	1339.017985	1194.447313	1047.246849	1145.071637	1192.613556
**ENSG00000165259**	HDX	30.93707615	31.52883663	25.2412298	12.55850479	10.40210053	6.828871559	45.96275407	23.25837691	30.48255804	24.67832454	2.759208765	12.68737825
**ENSG00000165264**	NDUFB6	409.2131436	441.4037129	302.8947576	456.2923408	398.7471872	446.5323236	329.9104348	300.6975871	363.9148468	332.208215	373.8727876	483.812024
**ENSG00000165269**	AQP7	8.437384405	0	1.628466439	10.88403749	5.778944741	3.035054026	1.021394535	3.322625272	0.937924863	4.429442867	0	0.845825217
**ENSG00000165271**	NOL6	1612.946652	973.8907316	1650.450736	1513.718444	1021.71743	1764.125153	872.2709328	443.5704738	1018.117439	822.6108181	471.8246987	943.0951167

ENSG00000165272	AQP3	6135.384693	3906.072539	4859.343854	5534.951679	4030.236063	5386.462133	2328.77954	800.7526906	3132.669042	2222.947542	914.6777055	2545.088077
ENSG00000165275	TRMT10B	170.1539188	143.6313669	131.9057816	139.81802	135.2273069	152.1320831	180.7868327	161.1473257	155.6955272	186.0366004	176.5893609	131.1029086
ENSG00000165280	VCP	5584.142245	5128.690759	6386.03114	5301.36349	5058.888227	5677.447938	4687.179521	3865.874504	5003.360181	4622.44002	4536.139209	5299.094983
ENSG00000165282	PIGO	402.18199	294.2691419	365.5907155	483.9210514	417.2398103	465.1220296	474.9484587	353.8595915	474.1210182	528.3692562	531.1476872	521.0283335
ENSG00000165283	STOML2	728.4275203	781.2145077	799.5770215	840.5825875	825.2333091	874.4749414	658.799475	566.5076089	729.7055433	654.9247667	579.4338406	743.4803655
ENSG00000165288	BRWD3	412.0256051	329.3011826	216.5860364	378.4296111	343.2693176	243.5630856	904.9555579	1199.467723	830.5324661	869.436357	1168.524912	929.5619132
ENSG00000165300	SLITRK5	0	0	1.628466439	0	0	0	0	0	0.468962431	3.163887762	9.657230676	0
ENSG00000165304	MELK	1527.166577	1593.957852	1643.122637	1621.721586	1599.611904	1510.698142	1435.059322	1478.568246	1365.6186	1273.148435	1346.493877	1507.260536
ENSG00000165312	OTUD1	683.4281368	662.1055693	644.8727098	491.4561542	444.9787451	498.1282421	337.0601965	392.0697821	476.4658304	377.7681988	369.7339745	444.0582388
ENSG00000165322	ARHGAP12	1657.946036	1103.509282	1349.184445	1225.710068	1304.885723	1119.176172	875.3351164	1189.499847	809.8981191	749.208622	863.6323433	776.467549
ENSG00000165323	FAT3	26.71838395	7.006408141	22.79853014	54.42018744	26.58314581	47.04333741	3.064183605	1.661312636	4.220661883	2.53111021	2.759208765	2.53747565
ENSG00000165338	HECTD2	46.40561423	112.1025303	71.65252331	77.86272972	146.7851964	81.56707696	32.68462512	36.54887799	20.16538455	23.41276944	13.79604382	21.14563042
ENSG00000165355	FBXO33	156.0916115	213.6954483	190.5305734	185.0286373	212.6651665	224.593998	386.0871342	355.5209041	353.1287109	357.5193171	404.224084	357.7840667
ENSG00000165359	INTS6L	331.8704533	364.3332233	346.0491183	320.660489	377.9429861	324.3713991	337.0601965	360.504842	314.6737915	308.162668	270.4024589	280.813972
ENSG00000165379	LRFN5	7.031153671	0	0	0	1.155788948	0.379381753	4.08557814	6.645250545	5.627549177	5.694997971	0	10.99572782
ENSG00000165389	SPTSSA	344.5265299	557.0094472	345.234885	365.0338727	353.6714182	310.3342742	485.1624041	599.7338616	443.6384602	580.2570155	517.3516434	578.5444483
ENSG00000165392	WRN	902.8001313	683.1247937	856.5733469	1031.47186	761.6649169	852.4707997	584.2376739	368.8114052	531.3344348	605.5681176	413.8813147	535.4073622
ENSG00000165406	8-Mar	260.1526858	231.2114686	249.9695984	144.0041883	199.951488	178.6888058	304.3755714	230.9224564	257.4603749	261.9699067	242.8103713	230.064459
ENSG00000165410	CFL2	451.4000657	378.3460396	452.71367	475.5487148	545.5323836	520.5117655	550.5316543	614.6856754	540.7136835	483.44205	561.4989836	597.152603
ENSG00000165416	SUGT1	2245.750482	2308.611482	2382.4464	2559.423277	2644.445114	2686.402195	1978.441214	1770.95927	1753.919494	1845.179343	1681.737742	2051.971976
ENSG00000165417	GTF2A1	1169.983971	1156.057343	1155.396938	1457.62379	1353.428858	1430.26921	1546.391326	1385.534739	1662.940782	1571.186663	1480.315502	1880.269457
ENSG00000165424	ZCCHC24	21.09346101	24.52242849	37.45472809	16.74467306	55.47786952	35.66188481	3.064183605	0	3.28273702	5.694997971	5.518417529	2.53747565
ENSG00000165434	PGM2L1	33.74953762	56.05126513	52.92515926	36.00104707	112.111528	71.32376962	17.36370709	19.93575163	14.53783538	30.37332251	51.04536215	22.83728085
ENSG00000165443	PHYHIPL	14.06230734	14.01281628	13.02773151	8.372336529	8.090522638	5.311344546	4.08557814	3.322625272	3.28273702	1.265555105	0	0.845825217
ENSG00000165458	INPPL1	1535.603962	984.4003438	1440.378565	1858.658709	1411.218306	1890.459277	2086.709035	1207.774286	2062.965736	2304.575846	1419.612909	1914.102466
ENSG00000165474	GJB2	1603.103037	1607.970668	1477.833293	2170.109628	2132.43061	2206.863659	1507.578333	1136.337843	2163.323696	1724.951608	1340.97546	2596.683416
ENSG00000165475	CRYL1	99.84238213	108.5993262	103.4076189	110.5148422	122.5136285	91.43100255	189.9793835	152.8407625	155.6955272	190.4660433	150.3768777	141.2528112
ENSG00000165476	REEP3	819.832518	500.9581821	607.4179817	591.0869589	570.9597404	641.1551631	298.2472042	312.3267756	383.1423065	318.9198864	306.2721729	345.9425137
ENSG00000165478	HEPACAM	0	3.50320407	3.256932878	5.86063557	4.623155793	5.6907263	1.021394535	0	0	0.632777552	0	0
ENSG00000165480	SKA3	1234.670585	865.2914054	1157.839638	1383.109995	1180.060516	1337.32068	764.0031121	750.9133115	831.470391	783.3786098	768.4396409	1043.748318
ENSG00000165487	MICU2	790.3016726	721.6600385	764.5649931	708.2996703	661.1112784	658.227342	475.9698533	510.0229793	482.0933795	504.3237092	488.3799513	521.8741588
ENSG00000165490	DDIAS	458.4312193	416.8812844	543.9077906	641.3209781	544.3765946	625.6005112	405.4936303	431.9412854	455.3625209	427.7576254	441.4734023	577.6986231
ENSG00000165494	PCF11	1742.31988	1373.255996	1693.605096	1762.376839	1709.411854	1599.473472	1901.836624	2079.96342	1932.125218	1987.554292	1776.930444	2029.98052
ENSG00000165495	PKNOX2	1.406230734	14.01281628	8.142332194	7.535102876	1.155788948	2.655672273	0	0	0	0	0	0
ENSG00000165496	RPL10L	1.406230734	3.50320407	2.442699658	3.348934611	1.155788948	1.896908767	2.04278907	0	3.28273702	0	0	1.691650434
ENSG00000165501	LRR1	343.1202991	245.2242849	291.4954926	392.6625832	288.9472371	383.1755708	428.9857046	382.1019063	382.6733441	429.6559581	386.289227	378.9296971
ENSG00000165502	RPL36AL	842.3322098	907.3298542	1044.661221	842.2570548	776.6901732	807.7037528	835.5007295	1156.273595	859.6081369	842.8596998	976.7599027	890.6539533
ENSG00000165506	DNAAF2	165.9352266	119.1089384	125.3919158	143.1669546	91.30732691	143.7856845	119.5031606	94.69482026	161.7920389	127.188288	85.5354717	153.0943642
ENSG00000165507	DEPP1	8.437384405	126.1153465	43.96859385	24.27977593	67.035759	38.69693884	278.840708	119.6145098	238.2329152	223.370476	99.33151553	236.8310607
ENSG00000165509	MAGEC3	0	0	2.442699658	0.837233653	1.155788948	3.793817533	1.021394535	3.322625272	2.813774589	1.265555105	4.138813147	0
ENSG00000165511	C10orf25	0	0	0	0	0	1.13814526	4.08557814	11.62918845	5.627549177	3.796665314	1.379604382	5.920776517
ENSG00000165512	ZNF22	7.031153671	28.02563256	4.071166097	11.72127114	9.246311586	14.41650663	5.106972674	14.95181373	12.66198565	16.45221636	4.138813147	16.91650434
ENSG00000165516	KLHDC2	417.650528	486.9453658	414.4447087	396.0115178	468.094524	410.8704388	683.3129438	599.7338616	582.9203023	748.5758445	710.4962569	739.2512395
ENSG00000165521	EML5	35.15576835	77.07048955	42.34012741	40.18721534	43.91998003	45.90519215	168.5300983	112.9692593	92.385599	241.721025	147.6176689	154.7860147
ENSG00000165525	NEMF	1191.077432	1029.941997	1143.997673	1209.802628	1189.306828	1115.761736	1629.124283	1483.552184	1443.935326	1626.871087	1461.001041	1584.230631
ENSG00000165526	RPUSD4	320.6206074	290.7659378	415.2589419	385.964714	315.5303829	472.7096646	292.118837	254.1808333	273.87406	282.8515659	260.7452283	263.0516424
ENSG00000165527	ARF6	2687.306933	2525.810135	2377.561001	2614.680698	2428.31258	2311.952405	2640.304873	2545.130959	2869.112156	2560.850754	2625.38714	2714.253121
ENSG00000165533	TTC8	177.1850725	182.1666117	202.7440716	152.3765248	175.6799201	171.8599342	494.3549549	358.8435294	364.3838092	462.5603908	393.187249	334.1009606
ENSG00000165555	NOXRED1	1.406230734	31.52883663	19.54159727	15.07020575	27.73893476	10.24330734	27.57765244	33.22625272	20.16538455	17.71777147	49.66575776	11.84155303
ENSG00000165572	KBTBD6	170.1539188	122.6121425	187.2736405	204.2850113	161.8104528	201.8310928	303.3541769	159.4860131	317.0186037	242.9865801	164.1729215	355.246591
ENSG00000165583	SSX5	52.03053716	73.56728548	79.79485551	42.6989163	69.3473369	62.21860754	27.57765244	29.90362745	31.88944534	29.74054496	23.4532745	27.06640694
ENSG00000165584	SSX3	870.4568244	770.7048955	749.9087951	716.6720069	650.7091779	595.2499709	694.5482837	701.0739324	641.0716438	669.4786504	553.2213573	529.4865857
ENSG00000165588	OTX2	0	0	0	0	0	0	0	0	5.158586746	1.265555105	0	0.845825217
ENSG00000165591	FAAH2	0	3.50320407	0	0	0	0.379381753	78.64737919	93.03350762	53.93067962	72.13664097	44.14734023	46.52038692
ENSG00000165609	NUDT5	1143.265587	1054.464425	1035.704655	1024.773991	955.8374602	1023.192589	968.2820191	1025.029896	890.5596573	951.0646612	865.0119477	971.0073489
ENSG00000165617	DACT1	0	0	2.442699658	1.674467306	0	3.793817533	5.106972674	6.645250545	2.813774589	8.858885733	9.657230676	4.229126084
ENSG00000165621	OXGR1	0	0	0	0	0	0	4.08557814	0	1.406887294	0	1.379604382	3.383300867
ENSG00000165626	BEND7	40.78069129	24.52242849	44.78282707	3.348934611	10.40210053	2.655672273	7.149761744	3.322625272	5.627549177	0	13.79604382	4.229126084
ENSG00000165629	ATP5F1C	1247.326661	1352.236771	976.2656301	1270.083451	1343.026758	1225.782445	1159.282797	956.9160784	1042.034523	1137.734039	1088.507858	1156.243071
ENSG00000165630	PRPF18	97.02992066	126.1153465	97.70798633	110.5148422	80.90522638	89.15471203	96.01108628	74.75906863	99.42003547	109.4705166	93.813098	118.4155303
ENSG00000165632	TAF3	333.276684	245.2242849	321.6221217	384.2902467	396.4356093	360.0332839	242.0705048	287.407086	285.5981208	260.7043516	328.345843	304.497078
ENSG00000165637	VDAC2	2979.802926	3128.361235	2993.121315	2997.296477	2882.537637	3205.017052	1937.585433	1880.605904	1936.814842	1817.969908	1901.094839	2179.691584
ENSG00000165643	SOHLH1	0	0	1.628466439	0	6.93473369	1.517527013	0	0	0	0	0	0
ENSG00000165644	COMTD1	43.59315276	31.52883663	47.22552673	71.16486049	55.47786952	47.04333741	82.73295732	21.59706427	68.93747742	96.81496551	57.94338406	59.20776517
ENSG00000165650	PDZD8	967.4867451	728.6664466	1015.348825	1275.944087	1170.814205	1349.460897	1045.908004	985.1583932	1114.254737	1163.045141	1071.952605	1397.303258
ENSG00000165655	ZNF503	37.96822982	35.0320407	51.29669283	29.30317785	57.78944741	59.56293527	3.064183605	9.967875817	8.910286198	6.327775524	9.657230676	5.074951301
ENSG00000165660	ABRAXAS2	641.2412148	542.9966309	586.247918	534.9923042	591.7639415	585.7654271	324.8034621	448.5544118	443.6384602	420.1642948	527.008874	506.6493048

ENSG00000165661	QSOX2	584.9919854	476.4357536	705.125968	762.7198578	624.1260321	781.1470301	549.5102598	423.6347222	566.0376548	601.7714523	487.000347	600.5359039
ENSG00000165669	FAM204A	691.8655212	700.6408141	678.2562718	742.6262501	710.8102032	666.9531223	797.7091317	1056.594837	837.5669026	917.527451	964.3434632	829.7545377
ENSG00000165671	NSD1	4065.413052	3275.495806	4065.466465	4870.188159	4242.901229	4794.626598	3708.683556	3226.269139	3759.671813	3645.431479	3348.299836	3966.074441
ENSG00000165672	PRDX3	1213.577124	896.820242	994.9929942	1409.901471	1100.311079	1228.058735	855.9286202	824.0110675	946.3661867	988.3985368	825.0034206	1036.981716
ENSG00000165675	ENOX2	272.8087624	231.2114686	230.4280011	221.866918	285.4798702	262.1527915	258.4128173	265.8100218	238.7018776	213.2460352	212.4590749	270.6640694
ENSG00000165678	GHITM	3587.294603	3205.431724	3233.320114	2896.828439	3021.232311	3131.03761	2958.979968	2870.748235	2885.525841	2742.457912	2721.959446	2879.189038
ENSG00000165684	SNAPC4	316.4019152	283.7595297	398.1600443	328.1955919	305.1282823	328.1652166	278.840708	247.5355828	271.5292478	315.1232211	306.2721729	252.9017398
ENSG00000165685	TMEM52B	32.34330689	0	7.328098975	18.41914036	12.71367843	13.65774312	172.6156764	127.921073	191.8056345	180.97438	102.0907243	186.9273729
ENSG00000165688	PMPCA	876.0817474	700.6408141	793.0631557	1026.448458	775.5343843	1076.306034	659.8208695	534.9426688	702.9746847	758.0675078	707.7370481	745.172016
ENSG00000165689	SDCCAG3	926.7060538	535.9902228	860.644513	916.7708499	680.7596905	941.2461299	712.9333853	558.2010457	746.1192284	664.41643	573.915423	673.2768726
ENSG00000165695	AK8	0	3.50320407	4.885399317	0.837233653	2.311577897	3.035054026	1.021394535	3.322625272	9.379248629	5.694997971	16.55525259	3.383300867
ENSG00000165698	SPACA9	60.46792157	35.0320407	53.73939248	35.16381342	68.19154795	57.28664475	53.11251581	64.79119281	77.84776362	91.7527451	133.8216251	85.42834689
ENSG00000165699	TSC1	1664.977189	1727.079607	1862.151373	1512.881211	1534.887723	1605.922962	1333.941263	1388.857364	1265.729602	1358.573405	1564.47137	1428.598791
ENSG00000165702	GFI1B	1.406230734	0	0.814233219	5.023401917	0	1.896908767	1.021394535	0	0	1.898332657	2.759208765	0
ENSG00000165704	HPRT1	462.6499115	476.4357536	330.5786871	544.2018744	579.0502631	445.3941784	552.5744434	626.3148638	519.1414116	673.2753157	619.4423677	687.6559012
ENSG00000165714	BORCS5	81.56138258	73.56728548	73.28098975	88.7467672	83.21680428	74.7382054	172.6156764	144.5341993	141.1576919	180.3416024	165.5525259	158.1693155
ENSG00000165716	FAM69B	14.06230734	0	4.071166097	7.535102876	12.71367843	7.208253313	0	0	0	0	0	0.845825217
ENSG00000165724	ZMYND19	388.1196826	420.3844885	333.83562	452.9434062	399.9029761	420.7343644	236.9635321	267.4713344	294.508407	316.3887762	277.3004808	361.1673676
ENSG00000165730	STOX1	132.185689	154.1409791	99.33645277	128.9339825	166.4336086	130.8867049	69.45482837	141.2115741	84.88220009	107.5721839	147.6176689	113.340579
ENSG00000165731	RET	2.812461468	10.50961221	5.699632536	4.186168264	1.155788948	8.346398573	4.08557814	3.322625272	0	1.898332657	0	0
ENSG00000165732	DDX21	7143.65213	4407.030721	6977.978691	8146.283442	5495.776449	7959.049803	4467.579696	2884.038736	5079.332095	4640.157792	2624.007535	5557.917499
ENSG00000165733	BMS1	1510.291808	1320.707935	1472.133661	1628.419455	1499.058266	1511.836287	1198.095789	1133.015218	1176.626741	1099.134608	976.7599027	1148.630644
ENSG00000165752	STK32C	163.1227652	196.1794279	183.2024744	205.122245	249.6504128	213.9713089	120.5245551	122.9371351	151.4748654	127.188288	137.9604382	124.3363069
ENSG00000165757	JCAD	105.4673051	479.9389576	335.4640864	102.1425056	292.4146039	316.783764	9.192550814	38.21019063	64.71681554	33.53721028	45.52694462	59.20776517
ENSG00000165775	FUNDC2	293.9022234	339.8107948	284.9816268	397.6859851	324.7766945	393.0394964	382.001556	501.7164161	424.8799629	458.1309479	492.5187645	497.3452275
ENSG00000165782	PIP4P1	282.6523776	350.320407	302.8947576	277.1243391	253.1177797	268.9816631	347.2741419	357.1822168	360.1631474	325.8804395	311.7905904	344.2508632
ENSG00000165792	METTL17	381.088529	269.7467134	443.7571046	336.5679285	302.8167044	365.7240102	433.0712828	490.0872277	438.010911	505.5892644	469.06549	388.2337745
ENSG00000165794	SLC39A2	4.218692202	7.006408141	8.956565414	0	4.623155793	4.931962793	20.4278907	36.54887799	34.70321993	22.77999189	4.138813147	16.91650434
ENSG00000165795	NDRG2	222.184456	304.7787541	297.1951251	246.1466939	266.987247	247.7362849	175.67986	152.8407625	215.7227185	225.9015862	176.5893609	292.655525
ENSG00000165799	RNASE7	1621.384036	2693.96393	2430.48616	970.3538037	1415.841462	1562.29406	519.8898183	573.1528595	582.4513399	538.4936971	496.6575776	549.7863909
ENSG00000165801	ARHGEF40	213.7470716	290.7659378	297.1951251	241.123292	269.2988249	260.6352645	251.2630556	277.4392102	266.8396235	280.9532333	278.6800852	279.1223215
ENSG00000165802	NSMF	575.1483703	567.5190594	487.7256984	864.0251298	844.8817212	832.7429485	273.7337353	382.1019063	435.6660988	396.7515253	513.2128302	443.2124136
ENSG00000165804	ZNF219	205.3096872	175.1602035	216.5860364	169.1211979	198.7956991	212.0744001	206.321696	179.4217647	236.8260279	228.4326964	209.6998661	237.6768859
ENSG00000165805	C12orf50	0	0	0	0.837233653	0	3.035054026	0	0	0.937924863	0	0	0
ENSG00000165806	CASP7	822.6449795	970.3875275	850.8737143	379.2668447	444.9787451	421.8725097	694.5482837	669.5089924	741.8985666	439.1476214	500.7963908	531.1782361
ENSG00000165807	PPP1R36	7.031153671	0	8.142332194	3.348934611	1.155788948	2.655672273	4.08557814	3.322625272	3.28273702	1.265555105	5.518417529	0.845825217
ENSG00000165810	BTNL9	7.031153671	35.0320407	1.628466439	0	11.55788948	1.13814526	0	0	0	1.265555105	2.759208765	1.691650434
ENSG00000165813	CCDC186	1376.699889	1723.576403	1648.008036	1342.922779	1317.599401	1225.403063	972.3675972	927.012451	918.6974032	1035.224076	997.4539684	1027.677638
ENSG00000165816	VWA2	11.24984587	14.01281628	23.61276336	12.55850479	10.40210053	20.48661468	0	1.661312636	0	0	0	0
ENSG00000165819	METTL3	563.8985244	378.3460396	618.8172468	519.9220984	442.6671672	455.6374857	675.1417876	589.7659858	662.1749532	542.2903624	499.4167864	614.0691074
ENSG00000165832	TRUB1	524.5240638	311.7851623	469.8125676	441.2221351	350.2040513	501.9220596	359.5308763	309.0041503	397.2111794	362.5815375	252.467602	360.3215423
ENSG00000165861	ZFYVE1	188.4349184	287.2627338	280.0962275	243.634993	334.023006	267.8435178	307.439755	453.5383497	351.2528612	291.7104516	509.0740171	306.1887285
ENSG00000165868	HSPA12A	94.21745919	136.6249587	113.1784175	146.5158893	254.2735686	187.4145861	164.4445201	139.5502614	137.8749548	215.1443678	204.1814486	160.7067912
ENSG00000165874	FAM35BP	5.624922937	0	0	4.186168264	2.311577897	1.517527013	1.021394535	3.322625272	0	3.163887762	0	4.229126084
ENSG00000165879	FRAT1	26.71838395	91.08330583	62.6959579	23.44254228	56.63365846	51.9753002	61.28367209	66.45250545	61.43407852	80.36274915	51.04536215	50.74951301
ENSG00000165886	UBTD1	44.99938349	38.53524477	46.41129351	57.76912205	95.93048271	92.94852956	20.4278907	74.75906863	43.61350613	47.45831643	57.94338406	39.75378519
ENSG00000165887	ANKRD2	8.437384405	35.0320407	12.21349829	19.25637402	42.76419109	12.51959786	10.21394535	43.19412854	10.31717349	15.18666126	15.17564821	10.1499026
ENSG00000165891	E2F7	1897.00526	2354.153135	2389.774499	1400.691901	1615.79295	1544.842499	1515.74949	1930.445283	1678.416542	1396.540058	1757.615983	1699.262861
ENSG00000165895	ARHGAP42	1019.517282	991.4067519	924.1547041	885.7932047	941.9679928	784.5614658	222.6640086	164.469951	175.3919494	163.8893861	171.0709434	157.3234903
ENSG00000165898	ISCA2	203.9034565	210.1922442	160.4039442	168.2839642	208.0420107	168.4454985	176.7012545	210.9867048	229.322629	203.1215943	230.3939318	247.8267885
ENSG00000165899	OTOGL	21.09346101	28.02563256	17.09889761	7.535102876	6.93473369	3.793817533	500.4833221	431.9412854	870.3942728	475.8487194	605.6463238	1001.457057
ENSG00000165905	LARGE2	7.031153671	17.51602035	12.21349829	20.09360767	26.58314581	18.96908767	33.70601965	6.645250545	45.48935585	79.7299716	35.86971394	72.74096864
ENSG00000165912	PACSIN3	658.1159836	672.6151815	666.8570067	746.8124184	785.9364848	854.7470902	683.3129438	503.3777287	838.035865	802.994714	562.878588	868.6624976
ENSG00000165914	TTC7B	99.84238213	84.07689769	78.98062229	56.93188839	90.15153796	80.80831345	28.59904698	14.95181373	42.20661883	28.47498986	30.35129641	49.90368779
ENSG00000165915	SLC39A13	177.1850725	171.6569995	179.9455415	123.073347	175.6799201	181.3444781	140.9524458	151.1794499	149.1300532	158.8271656	137.9604382	118.4155303
ENSG00000165916	PSMC3	2653.557395	3103.838806	2884.828297	2882.595467	2742.687174	2851.81264	1969.248663	1930.445283	1970.580137	1939.463198	1891.437608	2235.516048
ENSG00000165917	RAPSN	0	0	0	1.674467306	0	2.655672273	1.021394535	0	0.937924863	2.53111021	0	0
ENSG00000165923	AGBL2	67.49907524	70.06408141	78.16638907	47.72231821	46.23155793	28.07424974	125.6315278	111.3079466	98.48211061	77.83163894	84.15586732	69.35766778
ENSG00000165929	TC2N	1.406230734	0	4.885399317	0	4.623155793	3.793817533	4.08557814	18.274439	12.66198565	27.20943475	28.97169203	25.3747565
ENSG00000165934	CPSF2	1857.6308	1793.640484	1716.403627	2043.687347	1959.062267	1887.044841	2025.425363	1918.816095	1980.428348	1999.577066	1964.55664	2267.657406
ENSG00000165935	SMCO2	11.24984587	10.50961221	4.071166097	0.837233653	0	1.13814526	3.064183605	6.645250545	1.875849726	0	0	0
ENSG00000165943	MOAP1	195.466072	224.2050605	253.2265312	178.3307681	251.9619907	219.6620352	199.1719343	179.4217647	225.1019671	205.6527045	260.7452283	272.3557198
ENSG00000165948	IFI27L1	171.5601496	206.6890402	144.1192798	105.4914403	124.8252064	127.0928874	119.5031606	169.4538889	111.6130587	113.2671819	144.8584601	109.9572782
ENSG00000165949	IFI27	907.0188235	2070.393606	897.2850078	22.60530863	204.5746438	36.42064832	364.637849	551.5557952	449.7349718	12.0227735	23.4532745	18.60815477
ENSG00000165959	CLMN	37.96822982	28.02563256	40.71166097	98.79357104	61.25681426	51.2165367	902.9127688	1770.95927	910.7250419	889.0524611	1658.284468	868.6624976
ENSG00000165972	CCDC38	0	0	6.513865756	0.837233653	8.090522638	3.035054026	0	1.661312636	0.937924863	0.632777552	9.657230676	0.845825217
ENSG00000165983	PTER	772.0206731	889.8138339	912.755439	545.8763417	577.8944741	601.6994607	556.6600215	476.7967266	562.2859553	489.7698255	448.3714243	540.4823135

**ENSG00000165996**	HACD1	133.5919197	150.637775	142.4908134	212.6573478	199.951488	183.6207686	56.17669942	58.14594226	58.1513415	63.27775524	70.3598235	58.36193996
**ENSG00000165997**	ARL5B	1438.574041	1776.124464	1337.78518	1218.174965	1197.39735	1196.949432	1059.186133	933.6577015	1026.0898	851.7185855	907.7796836	1043.748318
**ENSG00000166002**	SMCO4	119.5296124	91.08330583	115.6211172	100.4680383	139.8504627	143.4063027	76.60459012	84.72694444	76.44087633	101.8771859	99.33151553	80.35339559
**ENSG00000166004**	CEP295	3173.862767	3426.133581	3109.556665	3151.347469	3051.282823	2723.960989	2284.859575	2266.030436	2214.909564	2262.812527	2085.961826	2546.779728
**ENSG00000166012**	TAF1D	4644.780115	2988.233072	3943.331482	4245.611854	2807.411355	3573.776116	3288.890402	2626.535278	3103.593371	3236.65718	2629.525953	3577.840667
**ENSG00000166016**	ABTB2	260.1526858	486.9453658	318.3651888	319.8232554	389.5008756	402.903422	254.3272392	304.0202124	330.1495517	283.4843435	255.2268107	258.8225163
**ENSG00000166024**	R3HCC1L	762.1770579	1029.941997	940.4393685	788.674101	755.8859722	866.8873063	516.8256346	712.7031209	520.5482989	501.792599	780.8560804	544.7114396
**ENSG00000166025**	AMOTL1	11726.55809	11494.01256	11319.47022	11514.47443	12307.99651	12212.29864	8444.890014	9506.030904	9180.877521	8503.264749	9426.836744	8881.164776
**ENSG00000166033**	HTRA1	584.9919854	469.4293454	432.3578395	570.9933513	572.1155294	488.2643165	2345.121852	3274.447206	3033.249007	2529.844654	3487.639878	3488.183194
**ENSG00000166037**	CEP57	1566.541038	1730.582811	1586.940545	1368.039789	1689.763442	1515.250723	1601.546631	1676.26445	1632.927186	1521.830013	1830.735015	1719.562666
**ENSG00000166046**	TCP11L2	194.0598413	493.9517739	175.8743754	121.3988797	288.9472371	143.026921	555.638627	810.7205664	414.5627894	512.5498174	727.0515095	409.3794049
**ENSG00000166049**	PASD1	22.49969175	49.04485699	68.39559043	15.07020575	54.32208057	53.49282722	15.32091802	14.95181373	24.38604644	15.18666126	17.93485697	11.84155303
**ENSG00000166068**	SPRED1	390.9321441	430.8941007	360.7053162	417.7795928	428.7976998	463.9838843	19.40649616	33.22625272	45.02039342	32.27165517	44.14734023	45.67456171
**ENSG00000166073**	GPR176	92.81122845	49.04485699	103.4076189	77.86272972	113.2673169	120.2640158	166.4873092	166.1312636	195.5573339	171.4827167	184.8669872	201.3064016
**ENSG00000166086**	JAM3	8.437384405	0	0	3.348934611	0	1.896908767	0	0	2.344812157	8.226108181	4.138813147	1.691650434
**ENSG00000166104**	AC126323.1	0	0	7.328098975	0	3.467366845	2.27629052	0	8.306563181	0	0.632777552	0	0
**ENSG00000166105**	GLB1L3	151.8729193	164.6505913	173.4316757	121.3988797	99.39784955	100.5361646	136.8668677	112.9692593	148.1921283	170.8499391	129.6828119	107.4198025
**ENSG00000166106**	ADAMTS15	0	0	6.513865756	5.023401917	8.090522638	3.41443578	2209.276379	3166.461884	2870.988005	2626.026842	4006.371126	3184.531941
**ENSG00000166123**	GPT2	734.0524432	521.9774065	758.8653605	493.9678552	484.2755693	668.8500311	417.7503648	428.6186601	410.81109	295.507117	401.4648753	283.3514476
**ENSG00000166126**	AMN	33.74953762	17.51602035	26.86969624	26.79147689	35.8294574	18.96908767	32.68462512	39.87150327	28.13774589	27.20943475	38.6289227	16.91650434
**ENSG00000166128**	RAB8B	205.3096872	479.9389576	268.6969624	177.4935344	220.7556891	179.0681876	575.0451231	910.3993246	549.6239697	516.9792603	784.9948935	545.5572648
**ENSG00000166130**	IKBIP	136.4043812	203.1858361	151.4473788	170.7956652	228.8462118	157.4434276	224.7067977	387.0858442	268.2465108	240.4554699	418.0201278	328.1801841
**ENSG00000166133**	RPUSD2	106.8735358	105.0961221	105.0360853	162.4233287	116.7346838	117.2289618	104.1822426	151.1794499	127.0888189	124.0244003	81.39665856	149.7110634
**ENSG00000166135**	HIF1AN	1636.852575	1902.23981	1974.515557	1809.261924	2021.474871	2091.152224	2180.677332	2594.970338	2216.316451	2004.639286	2463.973427	2359.00653
**ENSG00000166136**	NDUFB8	108.2797665	108.5993262	65.13865756	94.60740277	101.7094274	85.74027625	86.81853546	74.75906863	75.9719139	84.79219202	81.39665856	128.5654329
**ENSG00000166140**	ZFYVE19	260.1526858	339.8107948	275.2108282	195.9126748	247.3388349	215.4888359	281.9048916	440.2478486	285.1291583	267.6649047	378.0116008	230.064459
**ENSG00000166145**	SPINT1	895.7689777	889.8138339	1173.310069	1124.404796	1271.367843	1562.673442	914.1481087	838.9628812	1171.468154	1083.947947	905.0204748	1059.818997
**ENSG00000166147**	FBN1	2375.12371	1583.44824	1932.17543	4926.282813	4981.450367	5163.385662	11638.79072	9918.036438	12601.4895	14126.75886	12937.9299	16119.73698
**ENSG00000166148**	AVPR1A	2.812461468	3.50320407	13.02773151	0	17.33683422	25.79795922	3.064183605	0	2.344812157	0.632777552	0	1.691650434
**ENSG00000166153**	DEPDC4	22.49969175	14.01281628	16.28466439	33.48934611	39.29682424	15.17527013	86.81853546	69.77513072	69.87540229	73.40219608	70.3598235	53.28698866
**ENSG00000166164**	BRD7	1756.382187	1674.531546	1775.842652	1823.494896	1711.723432	1701.527164	1545.369931	1531.730251	1541.948475	1464.880034	1411.335283	1659.509075
**ENSG00000166165**	CKB	143.4355349	210.1922442	107.478785	325.683891	368.6966745	291.7445683	13.27812895	16.61312636	10.78613592	8.858885733	8.277626294	6.766601734
**ENSG00000166166**	TRMT61A	134.9981505	73.56728548	127.0203822	166.6094969	91.30732691	197.6578935	142.9952349	131.2436983	218.0675306	151.8666126	114.5071637	200.4605764
**ENSG00000166167**	BTRC	551.2424478	557.0094472	535.7654584	524.1082667	580.206052	575.5221198	683.3129438	749.2519989	740.0227168	780.2147221	706.3574437	710.4931821
**ENSG00000166169**	POLL	253.1215321	248.727489	293.123959	259.5424324	251.9619907	259.4971193	276.797919	252.5195207	334.8391761	358.7848722	355.9379306	285.8889233
**ENSG00000166170**	BAG5	1054.673051	1075.48365	1067.459751	1152.033506	1104.934235	1067.200872	1307.385005	1295.823856	1274.639889	1243.40789	1369.947152	1415.065588
**ENSG00000166171**	DPCD	115.3109202	63.05767327	79.79485551	159.9116277	161.8104528	107.3650362	88.86132453	191.0509532	111.6130587	158.1943881	160.0341083	126.0279573
**ENSG00000166173**	LARP6	18.28099954	52.54806106	43.96859385	5.023401917	24.27156791	34.1443578	0	0	0	0	0	0
**ENSG00000166181**	API5	2257.000328	2249.057013	2191.915827	1877.07785	2123.184298	1943.952104	2457.475251	2251.078622	2457.832103	2185.613666	2145.284814	2577.229436
**ENSG00000166188**	ZNF319	57.6554601	45.54165292	66.76712399	66.14145858	87.83996007	77.77325943	65.36925023	51.50069172	94.26144872	77.19886139	67.60061473	64.28271647
**ENSG00000166189**	HPS6	220.7782253	101.592918	237.7561001	188.3775719	187.2378096	228.0084337	139.9310513	122.9371351	188.5228974	202.4888168	151.7564821	238.5227111
**ENSG00000166192**	SENP8	66.09284451	42.03844885	32.56932878	53.58295378	45.07576898	34.9031213	26.55625791	23.25837691	25.79293373	29.74054496	30.35129641	22.83728085
**ENSG00000166197**	NOLC1	2735.118778	1807.6533	2312.422343	2854.129523	1969.464368	2613.181517	2003.976077	1373.90555	2094.855181	2013.498172	1477.556293	2319.252744
**ENSG00000166199**	ALKBH3	220.7782253	227.7082646	250.7838316	267.0775353	251.9619907	232.181633	396.3010795	333.9238399	341.4046501	384.7287518	426.2977541	382.312998
**ENSG00000166200**	COPS2	1307.794583	1432.810465	1313.358183	1436.692948	1412.374095	1462.516659	1115.362832	1031.675147	1033.593199	1003.585198	985.037529	1201.071808
**ENSG00000166206**	GABRB3	4.218692202	0	0	0	2.311577897	0.379381753	56.17669942	36.54887799	30.48255804	29.10776741	37.24931832	28.75805737
**ENSG00000166224**	SGPL1	1667.789651	1800.646892	2112.120971	1723.026858	2157.857966	2233.420382	1291.042692	1016.723333	1246.502143	1095.337943	1041.601309	1177.388702
**ENSG00000166225**	FRS2	752.3334428	823.2529565	631.0307451	708.2996703	641.4628663	688.957264	1080.635418	961.9000163	1077.675667	818.1813752	844.317882	938.0201654
**ENSG00000166226**	CCT2	2264.031482	1996.82632	2153.646865	2702.590231	2331.226309	2725.099134	2011.125839	1751.023518	2129.558401	1941.994308	1720.366665	2236.361873
**ENSG00000166228**	PCBD1	262.9651473	339.8107948	328.1359874	409.4072563	434.5766445	474.2271916	162.401731	201.018829	184.3022356	210.7149249	193.1446135	224.1436824
**ENSG00000166233**	ARIH1	999.830052	802.2337321	1069.088217	996.3080469	1063.325832	986.3925586	837.5435186	832.3176307	899.938906	815.0174875	902.261266	946.4784176
**ENSG00000166246**	C16orf71	0	0	4.885399317	5.023401917	5.778944741	3.41443578	3.064183605	0	1.406887294	1.898332657	1.379604382	5.074951301
**ENSG00000166250**	CLMP	337.4953762	178.6634076	103.4076189	78.69996337	38.14103529	47.80210092	12.25673442	33.22625272	52.05482989	10.75721839	26.21248326	55.82446431
**ENSG00000166257**	SCN3B	4.218692202	0	7.328098975	2.511700959	15.02525633	4.931962793	1.021394535	8.306563181	7.034436472	3.163887762	9.657230676	5.920776517
**ENSG00000166260**	COX11	478.1184496	262.7403053	379.4326803	441.2221351	453.0692677	492.8168975	544.4032871	528.2974183	490.5347033	508.7531521	539.4253135	442.3665884
**ENSG00000166261**	ZNF202	406.4006822	234.7146727	425.0297406	424.477462	275.0777697	397.5920775	237.9849266	255.842146	288.8808578	277.1565679	248.3287888	302.8054276
**ENSG00000166262**	FAM227B	37.96822982	66.56087734	39.89742775	37.67551438	40.45261319	42.87013812	42.89857046	28.24231481	26.73085859	28.47498986	20.69406573	19.45397999
**ENSG00000166263**	STXBP4	296.7146849	283.7595297	236.9418669	301.404115	425.330333	310.3342742	367.7020326	355.5209041	376.10787	367.0109804	382.1504139	374.700571
**ENSG00000166265**	CYYR1	1.406230734	3.50320407	4.885399317	0.837233653	5.778944741	12.89897961	0	0	4.220661883	0.632777552	1.379604382	1.691650434
**ENSG00000166266**	CUL5	1569.353499	1310.198322	1526.687286	1501.15994	1508.304577	1704.182836	1416.67422	1289.178606	1257.288279	1357.30785	1245.782757	1523.331215
**ENSG00000166268**	MYRFL	4.218692202	3.50320407	7.328098975	0.837233653	16.18104528	2.27629052	2.04278907	3.322625272	4.689624315	5.062220419	2.759208765	8.458252168
**ENSG00000166272**	WBP1L	615.9290616	868.7946095	704.3117348	840.5825875	900.3595907	891.1677385	1067.357289	1425.406242	1144.737295	1135.835707	1365.808338	1155.397246
**ENSG00000166275**	BORCS7	80.15515185	94.5865099	87.9371877	113.0265431	130.6041512	143.7856845	183.8510163	294.0523366	194.1504466	219.5738107	205.561053	226.6811581
**ENSG00000166278**	C2	23.90592248	0	24.42699658	23.44254228	26.58314581	16.31341539	37.79159779	28.24231481	25.79293373	31.00610007	13.79604382	20.2998052
**ENSG00000166289**	PLEKHF1	184.2162262	150.637775	211.7006371	97.11910373	142.1620406	131.2660866	111.3320043	182.74439	146.3162786	87.32330223	75.87824103	130.2570834
**ENSG00000166295**	ANAPC16	1331.700505	1303.191914	1220.535596	1327.852573	1734.839211	1450.376443	1679.172615	2114.850986	1534.914038	1644.588859	2203.228199	1535.172768
**ENSG00000166311**	SMPD1	132.185689	213.6954483	179.9455415	188.3775719	203.4188549	290.9858048	258.4128173	313.9880882	335.3081385	350.558764	317.3090079	360.3215423

**ENSG00000166313**	APBB1	157.4978422	259.2371012	131.9057816	185.8658709	236.9367344	210.1774913	50.04833221	53.16200436	30.48255804	44.29442867	57.94338406	62.59106604
**ENSG00000166317**	SYNPO2L	0	0	1.628466439	0	2.311577897	0.379381753	0	0	0.937924863	0.632777552	0	1.691650434
**ENSG00000166321**	NUDT13	43.59315276	63.05767327	48.03975995	70.32762684	88.99574902	55.01035423	60.26227756	71.43644335	54.86860448	102.5099635	86.91507609	49.90368779
**ENSG00000166323**	C11orf65	19.68723028	38.53524477	39.89742775	25.95424324	30.05051265	12.51959786	34.72741419	33.22625272	21.10330942	22.14721433	27.59208765	16.07067912
**ENSG00000166326**	TRIM44	5127.117257	3720.402723	4396.859385	3840.390766	3718.173047	3589.71015	5965.965478	5914.272985	5686.169481	4786.329406	4603.739824	4928.623538
**ENSG00000166333**	ILK	1500.448193	1495.868138	1634.166071	1049.891001	1240.161541	1228.058735	1013.223379	1214.419537	1149.895882	800.4636038	1071.952605	926.1786124
**ENSG00000166337**	TAF10	85.78007478	80.57369362	88.75142092	88.7467672	69.3473369	67.15057033	129.7171059	132.9050109	99.42003547	122.7588452	86.91507609	102.3448512
**ENSG00000166340**	TPP1	1314.825736	1317.20473	1414.323102	868.211298	1115.336335	959.0770724	1419.738403	1530.068938	1408.294182	1214.300123	1327.179416	1195.996857
**ENSG00000166341**	DCHS1	4.218692202	10.50961221	8.956565414	0.837233653	9.246311586	12.14021611	3.064183605	0	0	4.429442867	2.759208765	2.53747565
**ENSG00000166343**	MSS51	57.6554601	28.02563256	40.71166097	33.48934611	43.91998003	28.07424974	61.28367209	68.11381808	53.93067962	47.45831643	44.14734023	49.05786257
**ENSG00000166347**	CYB5A	0	0	0.814233219	0	0	0	4.08557814	33.22625272	7.034436472	10.12444084	44.14734023	6.766601734
**ENSG00000166348**	USP54	350.1514528	343.3139989	323.2505881	472.1997802	505.0797704	403.6621855	577.0879122	534.9426688	493.3484779	687.8291994	510.4536215	537.9448379
**ENSG00000166349**	RAG1	260.1526858	259.2371012	243.4557326	149.8648239	100.5536385	88.77533027	121.5459497	38.21019063	96.60626088	34.80276538	30.35129641	38.06213475
**ENSG00000166352**	C11orf74	171.5601496	154.1409791	152.261612	155.7254594	208.0420107	159.7197181	125.6315278	111.3079466	84.88220009	126.5555105	106.2295374	120.953006
**ENSG00000166359**	WDR88	0	0	0	0	0	3.41443578	0	0	0.937924863	0	1.379604382	0
**ENSG00000166368**	OR2D2	9.843615139	3.50320407	2.442699658	3.348934611	2.311577897	5.311344546	1.021394535	4.983937908	1.406887294	2.53111021	1.379604382	3.383300867
**ENSG00000166377**	ATP9B	122.3420739	171.6569995	125.3919158	108.0031412	109.7999501	146.061975	418.7717593	322.2946514	355.0045606	404.344856	445.6122155	350.1716397
**ENSG00000166387**	PPFIBP2	12.65607661	17.51602035	17.91313083	20.93084132	32.36209055	18.21032416	1366.625888	1083.175839	1243.219406	1425.015048	1340.97546	1233.213166
**ENSG00000166394**	CYB5R2	26.71838395	14.01281628	23.61276336	7.535102876	9.246311586	15.93403364	177.7226491	144.5341993	139.2818421	170.8499391	121.4051856	163.2442668
**ENSG00000166396**	SERPINB7	1195.296124	917.8394665	955.0955664	1865.356579	1619.260317	1866.558226	81.71156279	111.3079466	158.0403394	102.5099635	135.2012295	213.9937798
**ENSG00000166398**	KIAA0355	445.7751427	511.4677943	561.8209214	447.0827706	597.5428863	561.4849949	516.8256346	745.9293736	612.9338979	546.7198053	680.1449605	543.0197892
**ENSG00000166401**	SERPINB8	631.3975996	777.7113036	667.6712399	450.4317052	266.987247	471.9509011	212.4500633	358.8435294	304.356618	205.6527045	284.1985028	203.8438772
**ENSG00000166402**	TUB	0	7.006408141	4.071166097	2.511700959	4.623155793	4.55258104	18.38510163	1.661312636	13.13094808	17.71777147	19.31446135	11.84155303
**ENSG00000166405**	RIC3	2.812461468	0	8.142332194	1.674467306	3.467366845	1.13814526	11.23533988	0	12.66198565	2.53111021	0	0.845825217
**ENSG00000166408**	OR5P1P	0	7.006408141	3.256932878	2.511700959	8.090522638	7.967016819	84.77574639	34.88756536	59.08926636	82.26108181	74.49863664	53.28698866
**ENSG00000166411**	IDH3A	677.8032139	402.8684681	696.9836358	955.2835979	866.8417112	907.8605357	365.6592435	230.9224564	433.7902491	377.1354212	328.345843	491.4244509
**ENSG00000166415**	WDR72	4.218692202	14.01281628	8.956565414	7.535102876	11.55788948	10.24330734	0	1.661312636	0.937924863	0	1.379604382	0
**ENSG00000166432**	ZMAT1	0	0	5.699632536	4.186168264	3.467366845	5.311344546	10.21394535	0	4.220661883	8.226108181	1.379604382	5.920776517
**ENSG00000166435**	XRRA1	240.4654555	304.7787541	255.6692309	236.0998901	300.5051265	245.4599944	272.7123408	330.6012146	260.7431119	259.4387965	360.0767438	257.9766911
**ENSG00000166436**	TRIM66	305.1520693	420.3844885	373.7330477	329.8700592	297.0377597	281.501261	908.0197415	749.2519989	791.1396219	964.3529898	866.3915521	614.0691074
**ENSG00000166439**	RNF169	1773.256956	2129.948075	1973.701324	1332.038742	1459.761442	1589.230165	1346.197997	1631.409009	1246.971105	1219.362343	1444.445788	1294.958407
**ENSG00000166441**	RPL27A	8977.377007	7847.177118	7876.892165	8416.709912	8152.935241	8232.963428	10197.60304	11880.04666	10569.94424	10032.68809	10963.71603	11228.32975
**ENSG00000166444**	ST5	1837.94357	1744.595627	2051.867713	1252.501545	1002.069018	1286.862907	1025.480113	1058.256149	1155.523431	777.0508343	753.2639927	850.0543429
**ENSG00000166446**	CDYL2	105.4673051	143.6313669	188.0878737	85.39783259	130.6041512	179.8269511	963.1750464	651.2345534	1058.91717	1154.186256	707.7370481	1259.433748
**ENSG00000166448**	TMEM130	4.218692202	0	11.39926507	1.674467306	2.311577897	1.896908767	0	0	0	0	0	0
**ENSG00000166450**	PRTG	324.8392996	220.7018564	211.7006371	646.34438	417.2398103	407.456003	390.1727123	244.2129575	336.2460634	491.0353806	343.5214912	459.2830927
**ENSG00000166451**	CENPN	1465.292425	1289.179098	1413.508869	1583.208838	1466.696175	1611.234306	1305.342216	1483.552184	1504.43148	1144.061815	1288.550493	1426.061315
**ENSG00000166452**	AKIP1	582.1795239	378.3460396	490.1683981	384.2902467	387.1892977	356.6188481	341.1457746	423.6347222	420.659301	319.552664	406.9832928	398.3836771
**ENSG00000166454**	ATMIN	1918.098721	1607.970668	1881.69297	1890.473588	1883.935986	2116.19142	1521.877857	1657.990011	1541.948475	1495.253356	1735.542313	1797.378586
**ENSG00000166455**	C16orf46	1.406230734	14.01281628	1.628466439	5.023401917	0	3.41443578	1.021394535	11.62918845	2.344812157	1.265555105	1.379604382	0
**ENSG00000166471**	TMEM41B	963.2680529	893.317038	911.1269726	830.5357836	832.1680428	937.0729307	1017.308957	1064.9014	998.889979	1038.387963	1129.895989	1064.893948
**ENSG00000166473**	PKD1L2	471.0872959	1001.916364	706.7544345	493.1306215	1110.713179	720.0665678	433.0712828	1496.842685	459.1142204	557.4770236	1455.482623	479.5828979
**ENSG00000166477**	LEO1	576.554601	798.7305281	557.7497553	574.3422859	641.4628663	477.6416274	450.4349899	541.5879194	431.4454369	423.3281825	459.4082593	422.9126084
**ENSG00000166478**	ZNF143	376.8698368	441.4037129	421.7728077	405.221088	461.1597904	417.6993104	377.9159779	415.328159	425.3489253	347.3948763	412.5017103	480.4287231
**ENSG00000166479**	TMX3	1704.35165	1541.409791	1381.753773	1473.531229	1405.439361	1511.836287	1248.144122	1006.755457	1308.405184	1175.700692	998.8335728	1278.041903
**ENSG00000166483**	WEE1	1781.69434	1432.810465	1620.324107	1871.217214	1591.521382	1707.21789	3539.132063	3264.47933	3355.426197	3401.812122	3602.147042	3444.200283
**ENSG00000166484**	MAPK7	206.7159179	416.8812844	226.356835	154.0509921	338.6461618	251.9094842	159.3375474	277.4392102	207.7503571	194.8954861	266.2636458	179.314946
**ENSG00000166492**	FAM86GP	0	0	0	8.372336529	0	0.379381753	0	0	1.406887294	1.265555105	0	0.845825217
**ENSG00000166503**	HDGFL3	1008.267436	1285.675894	975.4513969	1232.407937	1308.353089	1148.388567	395.279685	342.230403	407.9973154	437.2492887	491.1391601	486.3494996
**ENSG00000166507**	NDST2	9.843615139	24.52242849	11.39926507	12.55850479	4.623155793	14.41650663	14.29952349	6.645250545	13.59991051	8.858885733	4.138813147	3.383300867
**ENSG00000166508**	MCM7	2192.313715	2641.415869	2315.679276	2132.434114	2367.055766	2089.255315	2546.336575	2805.957042	2635.099902	2475.425785	2748.17193	2711.715645
**ENSG00000166510**	CCDC68	241.8716863	259.2371012	290.6812593	104.6542066	53.16629162	94.84543833	61.28367209	58.14594226	81.59946307	22.77999189	23.4532745	35.5246591
**ENSG00000166526**	ZNF3	343.1202991	325.7979785	355.8199169	363.3594053	266.987247	313.3693282	405.4936303	385.4245316	366.7286214	406.2431886	398.7056665	384.0046484
**ENSG00000166529**	ZSCAN21	122.3420739	84.07689769	127.8346155	108.0031412	116.7346838	89.53409378	116.438977	79.74300653	102.7027725	75.93330629	78.63744979	80.35339559
**ENSG00000166532**	RIMKLB	109.6859973	84.07689769	107.478785	123.9105806	49.69892478	120.2640158	1314.534766	1141.321781	1604.78944	1491.456691	1183.70056	1578.309855
**ENSG00000166535**	A2ML1	0	0	3.256932878	2.511700959	1.155788948	0	8.171156279	11.62918845	9.379248629	24.67832454	8.277626294	29.60388259
**ENSG00000166548**	TK2	123.7483046	87.58010176	100.150686	138.9807864	147.9409854	132.0248501	159.3375474	127.921073	178.205724	175.279382	182.1077785	167.4733929
**ENSG00000166557**	TMED3	804.3639799	606.0543042	916.0123719	901.7006441	1004.380596	1088.44625	494.3549549	523.3134804	585.2651145	533.4314767	433.195776	605.6108552
**ENSG00000166562**	SEC11C	556.8673707	662.1055693	622.8884129	504.014659	442.6671672	533.4107451	314.5895167	317.3107135	340.9356877	384.7287518	300.7537553	416.1460067
**ENSG00000166575**	TMEM135	496.3994492	493.9517739	496.6822639	509.0380609	439.1998003	464.363266	457.5847516	456.8609749	469.9003563	386.6270845	445.6122155	411.9168806
**ENSG00000166578**	IQCD	33.74953762	70.06408141	25.2412298	59.44358935	84.37259322	45.14642864	125.6315278	114.6305719	114.8957957	113.2671819	125.5439988	131.1029086
**ENSG00000166579**	NDEL1	1162.952817	1085.993262	1199.365532	979.5633739	981.2648171	1056.957565	943.7685502	958.577391	829.5945412	861.2102488	852.5955083	802.6881307
**ENSG00000166589**	CDH16	4.218692202	0	1.628466439	0	2.311577897	0	7.149761744	6.645250545	3.751699452	14.5538837	2.759208765	0.845825217
**ENSG00000166592**	RRAD	14.06230734	73.56728548	32.56932878	24.27977593	45.07576898	29.212395	62.30506663	58.14594226	43.14454369	53.1533144	38.6289227	25.3747565
**ENSG00000166595**	FAM96B	314.9956845	283.7595297	335.4640864	361.684938	358.294574	366.8621554	221.6426141	202.6801416	235.4191406	227.7999189	190.3854048	230.9102842
**ENSG00000166596**	CFAP52	0	0	0	0	0	0	1.021394535	4.983937908	1.875849726	1.265555105	1.379604382	1.691650434
**ENSG00000166598**	HSP90B1	34510.30845	34229.80697	33640.04546	14531.02728	13217.60241	14201.77655	15603.84431	15564.83809	15179.37598	12873.22653	13227.64682	15719.66165

**ENSG00000166619**	BLCAP	3566.201142	3317.534255	3130.726729	3482.054762	3169.173296	3071.854057	4563.590782	7027.352451	4393.240058	4337.057344	6219.256555	4065.035992
**ENSG00000166634**	SERPINB12	0	0	0	0	9.246311586	4.55258104	1.021394535	0	0.468962431	0	0	2.53747565
**ENSG00000166669**	ATF7IP2	43.59315276	73.56728548	70.02405687	36.00104707	43.91998003	40.97322936	72.51901198	79.74300653	88.16493711	77.83163894	66.22101035	103.1906764
**ENSG00000166676**	TVP23A	30.93707615	161.1473872	56.99632536	6.697869223	11.55788948	5.311344546	38.81299233	191.0509532	15.94472267	13.92110615	48.28615338	5.074951301
**ENSG00000166681**	BEX3	444.368912	374.8428355	319.9936552	503.1774254	477.3408356	428.3219995	1601.546631	1375.566863	1744.071283	1726.84994	1554.814139	1612.988688
**ENSG00000166682**	TMPRSS5	30.93707615	45.54165292	14.65619795	20.09360767	16.18104528	18.58970591	16.34231256	11.62918845	17.8205724	17.08499391	22.07367012	14.37902869
**ENSG00000166685**	COG1	371.2449138	325.7979785	449.4567371	351.6381342	324.7766945	398.7302227	367.7020326	367.1500926	347.5011617	350.558764	325.5866342	309.5720293
**ENSG00000166689**	PLEKHA7	1236.076815	1184.082976	1160.282338	1123.567562	906.1385354	1117.279263	1904.900808	1717.797266	1938.690692	1492.089469	1302.346537	1504.723061
**ENSG00000166704**	ZNF606	137.8106119	206.6890402	151.4473788	129.7712162	152.5641412	124.8165968	328.8890402	370.4727179	277.6257594	343.5982109	311.7905904	282.5056224
**ENSG00000166710**	B2M	57559.83641	71398.80216	59230.58132	6768.19685	10436.7742	8301.631526	24476.69863	30958.56097	24963.33919	3885.886949	5242.496653	4270.57152
**ENSG00000166716**	ZNF592	694.6779827	942.3618949	798.7627883	740.9517828	960.460616	821.7408777	498.440533	534.9426688	523.8310359	543.5559175	598.7483019	482.9661988
**ENSG00000166734**	CASC4	1233.264354	1366.249587	1307.65855	1153.707974	1361.519381	1355.151623	1135.790723	794.1074401	926.2008021	939.0418877	907.7796836	1083.502103
**ENSG00000166741**	NNMT	4.218692202	3.50320407	13.02773151	2.511700959	0	0.758763507	73.54040651	106.3240087	160.3851516	60.11386748	55.18417529	159.860966
**ENSG00000166743**	ACSM1	4.218692202	3.50320407	4.071166097	0	0	0	1.021394535	3.322625272	4.689624315	0	4.138813147	2.53747565
**ENSG00000166747**	AP1G1	4083.694052	3594.287376	4090.707695	4139.28318	4108.829711	4187.615793	3277.655062	3390.73909	3131.262155	3229.06385	3298.634078	3417.979701
**ENSG00000166750**	SLFN5	1262.795199	2171.986524	1774.214185	111.3520758	142.1620406	150.9939378	718.040358	654.5571786	474.1210182	326.513217	383.5300183	202.1522268
**ENSG00000166762**	CATSPER2	144.8417656	119.1089384	114.8068839	168.2839642	139.8504627	113.814526	130.7385005	146.195512	106.9234344	143.0077268	139.3400426	131.9487338
**ENSG00000166763**	STRCP1	9.843615139	0	0.814233219	0	0	0.379381753	0	1.661312636	1.406887294	3.163887762	0	0.845825217
**ENSG00000166770**	ZNF667-AS1	42.18692202	31.52883663	46.41129351	42.6989163	49.69892478	57.28664475	37.79159779	34.88756536	53.46171719	46.19276132	35.86971394	53.28698866
**ENSG00000166780**	C16orf45	4.218692202	7.006408141	10.58503185	3.348934611	9.246311586	8.346398573	8.171156279	3.322625272	6.56547404	2.53111021	4.138813147	5.920776517
**ENSG00000166783**	MARF1	1487.792117	1870.710974	1547.043117	1155.382441	1455.138286	1224.264918	1491.236021	1817.476024	1412.045881	1331.36397	1800.383719	1316.104037
**ENSG00000166788**	SAAL1	381.088529	420.3844885	380.2469135	403.5466207	354.8272071	346.7549225	359.5308763	328.939902	339.0598379	350.558764	361.4563482	440.6749379
**ENSG00000166793**	YPEL4	29.53084542	84.07689769	30.12662912	17.58190671	26.58314581	28.07424974	4.08557814	29.90362745	12.19302322	12.65555105	33.11050518	3.383300867
**ENSG00000166794**	PPIB	3518.389297	2995.23948	3355.455097	2107.317104	2014.540137	2289.948263	1275.721774	1164.580158	1314.501695	1177.599025	1238.884735	1316.949863
**ENSG00000166797**	FAM96A	361.4012987	301.2755501	340.3494857	409.4072563	307.4398602	355.1013211	254.3272392	267.4713344	291.6946324	254.3765761	208.3202617	265.5891181
**ENSG00000166800**	LDHAL6A	0	0	0.814233219	0	0	3.035054026	0	3.322625272	0.468962431	0	0	0
**ENSG00000166801**	FAM111A	5190.39764	4575.184516	4707.896475	3302.049527	3514.754192	3258.130497	5529.830012	5065.342228	5199.85544	4176.964623	4009.130335	4556.460443
**ENSG00000166803**	PCLAF	81.56138258	94.5865099	67.58135721	111.3520758	73.97049269	97.88049235	70.47622291	66.45250545	85.35116252	62.01220013	113.1275593	79.50757038
**ENSG00000166813**	KIF7	30.93707615	14.01281628	26.05546302	39.34998168	34.67366845	50.07839144	100.0966644	96.3561329	92.85456143	103.7755186	111.747955	79.50757038
**ENSG00000166816**	LDHD	12.65607661	21.01922442	0.814233219	3.348934611	3.467366845	1.896908767	2.04278907	0	1.875849726	3.796665314	6.898021912	1.691650434
**ENSG00000166819**	PLIN1	0	0	0	0.837233653	0	1.517527013	1.021394535	0	1.406887294	1.265555105	4.138813147	0
**ENSG00000166821**	PEX11A	60.46792157	136.6249587	60.25325824	48.55955187	46.23155793	50.83715494	53.11251581	64.79119281	52.99275475	43.02887356	63.46180159	39.75378519
**ENSG00000166822**	TMEM170A	603.272985	728.6664466	623.7026461	435.3614995	433.4208556	436.6683981	591.3874357	566.5076089	514.4517873	489.137048	594.6094888	526.1032848
**ENSG00000166823**	MESP1	15.46853808	7.006408141	5.699632536	2.511700959	1.155788948	4.931962793	3.064183605	0	0	0.632777552	1.379604382	4.229126084
**ENSG00000166825**	ANPEP	1.406230734	0	2.442699658	0.837233653	0	1.517527013	1.021394535	0	0.937924863	0.632777552	0	2.53747565
**ENSG00000166828**	SCNN1G	40.78069129	259.2371012	139.2338805	19.25637402	28.89472371	57.28664475	1.021394535	3.322625272	4.689624315	0	6.898021912	4.229126084
**ENSG00000166831**	RBPMS2	1.406230734	0	1.628466439	1.674467306	6.93473369	2.655672273	0	0	0	1.265555105	0	0
**ENSG00000166833**	NAV2	1243.107969	1078.986854	1113.871044	1484.415267	1631.973995	1337.700062	4576.868911	8035.769221	4026.042474	4502.212285	7652.665509	3866.267066
**ENSG00000166839**	ANKDD1A	28.12461468	7.006408141	8.956565414	13.39573845	16.18104528	11.3814526	2.04278907	11.62918845	1.406887294	8.226108181	5.518417529	2.53747565
**ENSG00000166840**	GLYATL1	15.46853808	21.01922442	8.142332194	7.535102876	17.33683422	10.62268909	28.59904698	19.93575163	26.26189616	31.63887762	22.07367012	20.2998052
**ENSG00000166845**	C18orf54	303.7458386	367.8364274	271.9538953	447.0827706	446.134534	407.456003	650.6283187	591.4272985	766.7535754	869.436357	847.0770907	1059.818997
**ENSG00000166847**	DCTN5	1452.636348	1464.339301	1726.174425	1843.588504	1672.426608	1954.954175	1627.081494	1536.714188	1662.940782	1727.482718	1677.598929	1847.282273
**ENSG00000166848**	TERF2IP	517.4929102	364.3332233	683.9559043	615.3667349	662.2670674	756.4872161	548.4888652	573.1528595	686.0920372	732.7564057	607.0259282	879.6582254
**ENSG00000166851**	PLK1	907.0188235	276.7531216	933.1112695	1544.69609	1050.612154	1580.883766	715.9975689	448.5544118	886.3389954	777.6836119	517.3516434	910.1079333
**ENSG00000166855**	CLPX	656.7097529	613.0607123	608.2322149	750.9985866	728.1470374	684.7840647	555.638627	515.0069172	519.6103741	540.3920297	535.2865003	543.8656144
**ENSG00000166860**	ZBTB39	174.372611	94.5865099	140.862347	145.6786556	156.031508	133.5423772	127.6743169	172.7765142	181.9574234	127.8210656	195.9038223	200.4605764
**ENSG00000166863**	TAC3	0	0	1.628466439	0	0	0	0	11.62918845	12.19302322	0.632777552	12.41643944	10.99572782
**ENSG00000166866**	MYO1A	0	3.50320407	0	0.837233653	6.93473369	1.517527013	1.021394535	4.983937908	0.937924863	0.632777552	11.03683506	0
**ENSG00000166869**	CHP2	184.2162262	175.1602035	210.8864038	423.6402284	372.1640413	417.3199286	195.0863562	93.03350762	238.2329152	249.9471332	168.3117346	390.7712502
**ENSG00000166881**	NEMP1	1673.414574	1825.169321	1678.134665	1632.605623	1824.990749	1564.190969	1663.851697	1885.589842	1738.443733	1628.136642	1963.177036	2019.830618
**ENSG00000166886**	NAB2	229.2156097	423.8876925	214.1433367	226.0530863	278.5451365	190.8290219	227.7709813	377.1179684	322.6461528	205.6527045	391.8076446	278.2764963
**ENSG00000166887**	VPS39	752.3334428	914.3362624	759.6795937	694.9039319	870.309078	825.5346952	1123.533988	1231.032663	974.972895	920.0585612	1120.238758	962.5490967
**ENSG00000166888**	STAT6	393.7446056	462.4229373	395.7173447	292.1945449	383.7219308	299.3322034	150.1449966	151.1794499	160.854114	115.1655145	169.691339	106.5739773
**ENSG00000166889**	PATL1	2396.217171	1930.265443	2174.816929	1525.439716	1694.386598	1655.621971	1973.334241	2302.579314	1975.738724	1383.884507	1696.91339	1460.740149
**ENSG00000166896**	ATP23	60.46792157	80.57369362	56.99632536	67.81592588	83.21680428	72.46191488	112.3533988	107.9853213	95.19937358	98.71329817	103.4703287	110.8031034
**ENSG00000166897**	ELFN2	32.34330689	63.05767327	74.90945619	49.39678552	125.9809954	112.296999	0	0	1.875849726	2.53111021	0	1.691650434
**ENSG00000166900**	STX3	2016.534873	1713.06679	1967.187458	1711.305586	1615.79295	1723.531305	1581.11874	1993.575163	1600.099816	1395.274503	1705.191017	1672.196454
**ENSG00000166902**	MRPL16	521.7116024	427.3908966	507.2672957	510.7125282	425.330333	465.1220296	412.6433921	318.9720261	330.6185142	350.558764	339.382678	408.5335797
**ENSG00000166908**	PIP4K2C	937.9558997	1107.012486	1209.950564	1023.936757	997.4458624	1111.209155	910.0625306	1033.33646	984.8211061	907.4030101	881.5672003	996.3821054
**ENSG00000166912**	MTMR10	209.5283794	140.1281628	199.4871388	197.5871421	204.5746438	176.0331335	743.5752214	539.9266067	768.1604627	533.4314767	444.2326111	633.5230874
**ENSG00000166913**	YWHAB	6510.848299	6305.767327	6919.353899	6288.461967	6713.978	6984.038697	6897.477294	7369.582854	7403.509905	6773.250921	7313.282831	7895.778399
**ENSG00000166920**	C15orf48	95.62368992	648.092753	353.3772172	64.46699127	202.2630659	265.9466091	23.4920743	44.85544118	18.75849726	13.92110615	42.76773585	20.2998052
**ENSG00000166922**	SCG5	8.437384405	0	8.956565414	6.697869223	0	4.55258104	4.08557814	8.306563181	4.689624315	4.429442867	1.379604382	4.229126084
**ENSG00000166924**	NYAP1	4.218692202	7.006408141	4.885399317	0	1.155788948	1.517527013	0	3.322625272	4.689624315	6.327775524	2.759208765	1.691650434
**ENSG00000166925**	TSC22D4	149.0604578	206.6890402	210.8864038	181.6797027	231.1577897	222.3177074	219.599825	177.7604521	272.4671727	252.4782434	190.3854048	208.0730033
**ENSG00000166927**	MS4A7	0	0	0	0	0	0	8.171156279	9.967875817	18.75849726	6.960553076	6.898021912	22.83728085
**ENSG00000166928**	MS4A14	0	0	0	0	0	0	4.08557814	3.322625272	3.28273702	0	0	1.691650434

ENSG00000166938	DIS3L	336.0891455	276.7531216	280.0962275	396.0115178	375.6314082	346.3755408	360.5522708	252.5195207	306.7014302	324.6148844	277.3004808	356.9382415
ENSG00000166946	CCNDBP1	293.9022234	339.8107948	276.8392946	232.7509555	305.1282823	239.7692681	324.8034621	398.7150327	368.6044711	325.2476619	360.0767438	316.3386311
ENSG00000166948	TGM6	0	0	0	0	0	3.41443578	16.34231256	8.306563181	2.344812157	2.53111021	5.518417529	9.304077385
ENSG00000166949	SMAD3	611.7103694	784.7177118	802.8339544	562.6210147	603.321831	807.7037528	506.6116893	868.8665087	509.762163	515.7137052	866.3915521	543.8656144
ENSG00000166961	MS4A15	0	3.50320407	0	1.674467306	0	1.13814526	166.4873092	338.9077778	166.9506256	215.1443678	343.5214912	174.2399947
ENSG00000166963	MAP1A	73.12399818	73.56728548	30.12662912	101.305272	116.7346838	59.94231702	59.24088302	103.0013834	86.28908739	132.883286	99.33151553	96.42407471
ENSG00000166965	RCCD1	371.2449138	245.2242849	286.6100932	313.1253862	337.4903729	298.9528216	193.0435671	157.8247004	165.5437383	195.5282637	201.4222398	242.7518372
ENSG00000166971	AKTIP	365.6199909	378.3460396	369.6618816	440.3849014	423.0187551	480.2972997	535.2107363	553.2171078	453.9556336	538.4936971	535.2865003	447.4415397
ENSG00000166974	MAPRE2	215.1533023	329.3011826	345.234885	343.2657977	465.7829462	497.7488603	304.3755714	531.6200436	242.9225395	289.1793414	571.1562143	171.702519
ENSG00000166979	EVA1C	1.406230734	0	2.442699658	3.348934611	0	1.517527013	1.021394535	0	0.468962431	0.632777552	0	0
ENSG00000166986	MARS	2507.309399	2091.41283	3033.832976	2218.66918	2271.125283	3011.532358	3096.86823	2357.402631	3338.074587	2377.978042	1894.196817	2368.310607
ENSG00000166987	MBD6	350.1514528	262.7403053	391.6461786	339.0796294	366.3850966	370.2765912	410.600603	407.0215959	440.3557231	388.5254172	402.8444796	332.4093102
ENSG00000166997	CNPY4	56.24922937	94.5865099	56.99632536	62.79252397	105.1767943	69.0474791	76.60459012	144.5341993	94.26144872	107.5721839	103.4703287	111.6489286
ENSG00000167004	PDIA3	10974.22465	10271.39433	10195.01414	6333.672584	6460.860221	6264.351511	5413.391035	6585.44329	5470.915725	4475.00285	5482.547815	5394.673233
ENSG00000167005	NUDT21	2954.490772	2725.492767	2554.249609	2772.080625	2540.424108	2623.424824	2826.198678	2855.796422	2851.760546	2889.895082	2709.543007	3077.112139
ENSG00000167034	NKX3-1	191.2473798	140.1281628	149.0046792	74.51379511	86.68417112	64.11551631	154.2305748	224.2772059	167.8885505	118.9621798	169.691339	113.340579
ENSG00000167037	SGSM1	40.78069129	35.0320407	44.78282707	36.83828073	73.97049269	39.8350841	0	0	0	1.265555105	0	0
ENSG00000167046	AL357033.1	7.031153671	49.04485699	7.328098975	5.86063557	17.33683422	11.00207085	11.23533988	21.59706427	7.034436472	6.960553076	9.657230676	3.383300867
ENSG00000167065	DUSP18	240.4654555	217.1986524	207.629471	267.0775353	286.6356592	287.571369	145.038024	156.1633878	220.8813052	196.1610412	171.0709434	208.0730033
ENSG00000167074	TEF	136.4043812	269.7467134	249.1553652	175.8190671	285.4798702	206.3836738	73.54040651	109.646634	126.1508941	106.9394064	92.43349361	88.81164776
ENSG00000167077	MEI1	7.031153671	0	7.328098975	0	0	4.931962793	2.04278907	0	1.406887294	0	0	0
ENSG00000167081	PBX3	343.1202991	507.9645902	438.0574721	685.6943617	770.9112285	661.6417778	527.03958	566.5076089	626.064846	673.9080933	691.1817955	724.8722108
ENSG00000167083	GNGT2	29.53084542	14.01281628	12.21349829	5.023401917	12.71367843	16.31341539	0	0	0.937924863	0.632777552	0	0.845825217
ENSG00000167085	PHB	1181.233817	956.3747112	1305.215851	1378.923826	1155.788948	1536.496101	750.7249831	596.4112364	944.9592994	885.8885733	880.1875959	955.782495
ENSG00000167088	SNRPD1	1366.856274	970.3875275	1279.974621	1511.206743	1220.513129	1369.188748	1106.170281	892.1248856	1145.206258	1251.633999	949.167815	1373.620152
ENSG00000167100	SAMD14	2.812461468	3.50320407	2.442699658	1.674467306	4.623155793	2.655672273	1.021394535	1.661312636	0	0	0	0
ENSG00000167103	PIP5KL1	154.6853808	245.2242849	97.70798633	153.2137585	179.147287	119.884634	91.92550814	103.0013834	85.82012496	87.95607978	108.9887462	82.89087124
ENSG00000167105	TMEM92	5.624922937	52.54806106	20.35583049	8.372336529	30.05051265	20.86599643	0	1.661312636	0	0	0	0
ENSG00000167106	FAM102A	1071.547819	1320.707935	1291.373886	940.2133922	1117.647913	1177.980344	800.7733153	915.3832625	1072.986043	879.5607978	922.9553318	911.7995837
ENSG00000167107	ACSF2	260.1526858	329.3011826	266.2542628	300.5668814	307.4398309	261.0146463	900.8699798	1051.610899	718.450445	738.4514036	862.2527389	608.9941561
ENSG00000167110	GOLGA2	1909.661337	2094.916034	2005.456419	1888.799121	2214.491625	1990.995441	1481.022076	1667.957887	1630.113412	1606.622205	1721.746269	1642.592571
ENSG00000167112	TRUB2	776.2393653	557.0094472	771.0788588	1060.775038	884.1785454	999.67092	669.0134203	450.2157244	786.4499975	804.2602691	582.1930493	734.1762882
ENSG00000167113	COQ4	478.1184496	434.3973047	545.536257	610.3433329	494.6776699	519.753002	662.8850531	453.5383497	692.1885488	703.6486382	558.7397748	675.8143482
ENSG00000167114	SLC27A4	729.833751	437.9005088	590.3190841	971.1910373	886.4901233	1080.858615	468.8200915	220.9545806	644.8233432	587.2175686	306.2721729	602.2275543
ENSG00000167118	URM1	535.7739097	613.0607123	635.9161444	729.2305116	646.0860221	781.5264118	458.6061462	450.2157244	604.0236117	535.9625869	520.1108521	569.2403709
ENSG00000167123	CERCAM	97.02992066	150.637775	135.1627144	104.6542066	156.031508	159.7197181	58.21948849	64.79119281	41.26869397	41.76331846	60.70259282	41.44543562
ENSG00000167130	DOLPP1	254.5277629	150.637775	201.1156052	239.4488247	297.0377597	289.4682778	273.7337353	300.6975871	312.3289793	335.3721028	271.7820633	379.7755223
ENSG00000167136	ENDOG	88.59253625	84.07689769	65.95289078	100.4680383	101.7094274	122.9196881	84.77574639	152.8407625	114.4268333	130.3521758	189.0058004	153.0943642
ENSG00000167157	PRRX2	5.624922937	10.50961221	0.814233219	0.837233653	8.090522638	5.6907263	0	0	0	0	0	0.845825217
ENSG00000167165	UGT1A6	267.1838395	248.727489	276.8392946	221.0296844	349.0482624	255.7033017	388.1299233	352.1982789	288.4118953	328.4115497	409.7425015	235.9852355
ENSG00000167173	C15orf39	438.7439891	574.5254675	658.7146745	423.6402284	410.3050766	667.7118858	141.9738403	191.0509532	218.0675306	141.7421717	238.6715581	142.0986364
ENSG00000167178	ISLR2	1.406230734	0	0.814233219	2.511700959	0	0	1.021394535	0	0.468962431	1.898332657	1.379604382	0
ENSG00000167182	SP2	444.368912	381.8492437	428.2866734	452.9434062	386.0335087	417.3199286	329.9104348	360.504842	371.4182457	370.8076457	358.6971394	367.9339693
ENSG00000167183	PRR15L	0	3.50320407	11.39926507	26.79147689	30.05051265	36.04126656	14.29952349	13.29050109	15.47576024	15.81943881	24.83287888	10.99572782
ENSG00000167186	COQ7	202.4972257	147.134571	240.1987997	242.7977593	236.9367344	211.6950183	187.9365944	149.5181373	211.5020566	236.026027	186.2465916	226.6811581
ENSG00000167191	GPRC5B	16.87476881	66.56087734	99.33645277	30.97764516	54.32208057	108.5031814	23.4920743	14.95181373	54.39964205	36.06832049	35.86971394	84.58252168
ENSG00000167193	CRK	1245.92043	1555.422607	1477.01906	1401.529135	1375.388848	1516.76825	1239.972965	1319.082233	1353.89454	1190.254576	1277.513658	1312.720736
ENSG00000167196	FBXO22	1049.048128	956.3747112	848.4310147	871.5602326	873.7764449	724.2397671	705.7836236	747.5906863	649.98193	611.8958932	725.6719051	608.9941561
ENSG00000167202	TBC1D2B	327.6517611	448.410121	379.4326803	294.7062458	411.4608656	394.9364052	413.6647866	323.955964	369.542396	326.513217	344.9010956	304.497078
ENSG00000167207	NOD2	70.31153671	360.8300193	112.3641843	35.16381342	91.30732691	36.80003007	8.171156279	51.50069172	14.53783538	3.163887762	13.79604382	5.074951301
ENSG00000167208	SNX20	0	0	0	0	1.155788948	0	3.064183605	14.95181373	0	0	2.759208765	0
ENSG00000167216	KATNAL2	0	0	4.071166097	5.86063557	1.155788948	3.41443578	1.021394535	6.645250545	0	1.898332657	2.759208765	0
ENSG00000167220	HDHD2	122.3420739	143.6313669	115.6211172	90.42123451	150.2525633	105.8475092	145.038024	111.3079466	118.1785327	156.928833	161.4137127	145.4819373
ENSG00000167232	ZNF91	84.37384405	91.08330583	74.90945619	96.28187008	62.41260321	97.12172885	1027.522902	1039.98171	838.9737899	1064.964621	1038.8421	844.9793916
ENSG00000167244	IGF2	538.5863712	395.86206	472.2552673	830.5357836	647.241811	625.2211294	3355.281047	4651.675381	3458.597932	3296.13827	4842.411382	3569.382415
ENSG00000167257	RNF214	637.0225226	658.6023652	703.4975016	592.7614262	604.4776199	649.1221799	475.9698533	533.2813562	424.8799629	447.3737295	503.5555995	480.4287231
ENSG00000167258	CDK12	2927.772389	2501.287706	2953.223887	2947.899692	2422.533636	2751.276475	2689.33181	2453.758764	2533.804017	2618.433512	2499.843141	2438.5141
ENSG00000167261	DPEP2	2.812461468	0	7.328098975	3.348934611	0	11.76083435	19.40649616	9.967875817	9.379248629	25.94387965	1.379604382	11.84155303
ENSG00000167264	DUS2	319.2143767	346.817203	319.179422	417.7795928	283.1682923	339.5466692	242.0705048	146.195512	230.2605538	242.3538026	266.2636458	263.0516424
ENSG00000167272	POP5	262.9651473	164.6505913	224.7283686	332.3817602	235.7809454	268.2228996	324.8034621	297.3749619	398.1491043	365.7454253	338.0030737	405.9961041
ENSG00000167280	ENGASE	115.3109202	168.1537954	163.6608771	124.7478143	123.6694175	171.8599342	144.0166294	191.0509532	171.1712875	209.4493698	219.3570968	159.860966
ENSG00000167283	ATP5MG	438.7439891	346.817203	382.6896131	658.0656512	479.6524135	464.7426478	315.6109113	400.3763453	368.6044711	383.4631967	373.8727876	447.4415397
ENSG00000167291	TBC1D16	206.7159179	171.6569995	143.3050466	196.7499084	180.3030759	167.686735	64.3478557	76.42038126	42.20661883	57.58275727	78.63744979	54.97863909
ENSG00000167302	TEPSIN	91.40499772	112.1025303	100.9649192	91.25846816	108.6441611	112.296999	105.2036371	147.8568246	123.8060819	146.1716146	117.2663725	101.499026
ENSG00000167306	MYO5B	1528.572808	1692.047566	1972.072858	1668.60667	1813.43286	1943.952104	1797.654381	1943.735784	1823.325933	1820.501018	1840.392246	1903.952563
ENSG00000167311	ART5	0	0	4.071166097	1.674467306	3.467366845	6.070108053	3.064183605	0	0	0.632777552	0	0.845825217
ENSG00000167315	ACAA2	118.1233817	108.5993262	143.3050466	168.2839642	130.6041512	164.2722992	45.96275407	33.22625272	70.34436472	87.32330223	66.22101035	91.34912341

ENSG00000167323	STIM1	392.3383748	301.2755501	408.7450762	559.2720801	491.210303	552.3798328	914.1481087	779.1556263	837.0979401	796.6669384	800.1705417	907.5704576
ENSG00000167325	RRM1	2590.277012	2553.835767	2410.13033	3277.769751	3318.27007	3184.909819	4260.236605	4545.351372	4093.104102	4406.662875	4628.572703	4801.749756
ENSG00000167333	TRIM68	185.6224569	178.6634076	266.2542628	182.5169363	217.2883223	231.0434878	335.0174074	378.779281	400.4939165	356.8865395	405.6036884	347.6341641
ENSG00000167355	OR51B5	21.09346101	45.54165292	52.92515926	25.11700959	49.69892478	43.62890163	19.40649616	9.967875817	10.78613592	20.24888168	4.138813147	10.99572782
ENSG00000167359	OR51I1	9.843615139	10.50961221	13.84196473	1.674467306	15.02525633	9.863925586	0	0	1.406887294	2.53111021	0	3.383300867
ENSG00000167363	FN3K	11.24984587	7.006408141	4.071166097	7.535102876	12.71367843	7.587635066	8.171156279	14.95181373	3.751699452	1.898332657	13.79604382	2.53747565
ENSG00000167371	PRRT2	40.78069129	35.0320407	30.12662912	8.372336529	47.38734688	14.41650663	46.9841486	64.79119281	34.70321993	39.8649858	70.3598235	30.4497078
ENSG00000167377	ZNF23	53.4367679	28.02563256	56.18209214	51.07125282	47.38734688	56.907263	66.39064477	48.17806645	35.64114479	41.76331846	33.11050518	34.67883389
ENSG00000167378	IRGQ	846.550902	560.5126513	947.7674674	836.3964192	804.429108	1075.926652	505.5902948	402.0376579	622.3131465	529.6348113	489.7595557	650.4395917
ENSG00000167380	ZNF226	234.8405326	332.8043867	339.5352525	290.5200775	233.4693675	239.0105046	428.9857046	519.9908551	395.3353297	333.4737701	437.3345892	417.8376571
ENSG00000167384	ZNF180	184.2162262	164.6505913	193.7875062	239.4488247	197.6399102	168.0661167	304.3755714	264.1487091	188.053935	219.5738107	215.2182836	247.8267885
ENSG00000167393	PPP2R3B	78.74892111	108.5993262	111.5499511	72.8393278	92.46311586	91.43100255	116.438977	109.646634	102.7027725	100.6116308	108.9887462	80.35339559
ENSG00000167394	ZNF668	192.6536106	171.6569995	147.3762127	152.3765248	135.2273069	184.3795321	148.1022076	156.1633878	187.1160101	158.1943881	177.9689653	137.8695103
ENSG00000167395	ZNF646	1255.764046	1667.525138	1406.18077	1095.101618	1251.719431	1249.304114	1319.641739	1629.747696	1535.851963	1377.556732	1531.360864	1349.091221
ENSG00000167397	VKORC1	119.5296124	122.6121425	91.19412058	154.8882258	164.1220307	106.6062727	134.8240786	107.9853213	122.3991946	108.837739	172.4505478	140.406986
ENSG00000167419	LPO	0	3.50320407	0.814233219	0	3.467366845	0.379381753	0	1.661312636	0.468962431	0	1.379604382	0.845825217
ENSG00000167447	SMG8	336.0891455	252.2306931	351.7487508	319.8232554	295.8819708	352.0662671	222.6640086	265.8100218	270.5913229	249.3143556	318.6886123	323.1052328
ENSG00000167460	TPM4	7066.309439	6610.546081	7711.602821	6252.46092	5123.612408	6488.566127	5267.331616	5761.432222	5426.364294	5462.76861	6044.046799	5990.134185
ENSG00000167461	RAB8A	989.9864368	1110.51569	1073.973616	1193.895189	1230.91523	1197.708195	1031.60848	1048.288273	1086.116991	983.3363164	1078.850627	1164.701324
ENSG00000167468	GPX4	1223.420739	1285.675894	1214.835963	1418.273808	1692.07502	1523.21774	1327.812895	1827.4439	1517.093466	1442.100042	1939.723762	1550.397622
ENSG00000167470	MIDN	1392.168427	1320.707935	1402.109604	1234.082404	1171.969994	1425.337247	1105.148887	882.1570098	1210.392036	969.4152102	870.5303652	964.2407471
ENSG00000167476	JSRP1	1.406230734	0	3.256932878	0	2.311577897	4.931962793	6.128367209	6.645250545	2.344812157	6.327775524	0	0.845825217
ENSG00000167483	FAM129C	0	0	0	0	2.311577897	4.931962793	0	0	1.875849726	0.632777552	1.379604382	0
ENSG00000167487	KLHL26	80.15515185	105.0961221	59.43902502	70.32762684	75.12628164	95.60420183	94.98969174	88.04956971	104.5786222	94.2838553	99.33151553	104.8823269
ENSG00000167491	GATAD2A	1454.042579	1317.20473	1448.520897	1711.305586	1724.437111	1869.972662	1280.828747	1103.11159	1478.638546	1268.086215	1237.505131	1295.804232
ENSG00000167508	MVD	248.9028399	283.7595297	197.8586723	211.8201142	285.4798702	235.216687	150.1449966	215.9706427	164.6058134	133.5160636	190.3854048	172.5483442
ENSG00000167513	CDT1	326.2455303	441.4037129	405.4881433	353.3126015	397.5913982	460.1900668	353.4025091	473.4741013	369.542396	362.5815375	442.8530067	345.9425137
ENSG00000167515	TRAPPC2L	268.5900702	350.320407	240.1987997	268.7520026	275.0777697	310.713656	211.4286687	247.5355828	212.4399814	246.7832454	256.6064151	254.5933902
ENSG00000167522	ANKRD11	3123.238461	3002.245888	3894.477489	2849.943354	2843.240813	3349.5615	2696.481572	2116.512298	2490.659473	2228.642539	2146.664419	2117.946343
ENSG00000167523	SPATA33	75.93645964	94.5865099	96.07951989	110.5148422	83.21680428	75.87635066	79.66877372	88.04956971	95.66833602	112.6344043	117.2663725	86.27417211
ENSG00000167524	SGK494	116.7171509	119.1089384	117.2495836	153.2137585	123.6694175	116.4701983	101.118059	89.71088235	97.07522331	89.22163489	115.8867681	84.58252168
ENSG00000167525	PROCA1	1.406230734	7.006408141	1.628466439	0.837233653	1.155788948	1.896908767	2.04278907	4.983937908	0	2.53111021	0	3.383300867
ENSG00000167526	RPL13	6084.760387	5815.318757	5692.304437	5592.720801	5376.730187	5718.041786	5141.700089	5678.36659	5763.548283	5184.979264	5849.522581	5720.315941
ENSG00000167528	ZNF641	144.8417656	287.2627338	251.5980648	130.6084498	193.0167544	178.3094241	163.4231256	142.8728867	127.0888189	126.5555105	102.0907243	132.794559
ENSG00000167531	LALBA	1.406230734	0	0	3.348934611	0	0	7.149761744	0	0	0	0	0
ENSG00000167535	CACNB3	142.0293041	266.2435094	156.3327781	182.5169363	278.5451365	183.2413868	122.5673442	214.3093301	129.4336311	157.5616105	155.8952952	125.1821321
ENSG00000167536	DHRS13	106.8735358	105.0961221	107.478785	200.098843	179.147287	154.7877553	145.038024	172.7765142	158.5093018	161.3582759	211.0794705	163.2442668
ENSG00000167543	TP53I13	92.81122845	77.07048955	126.206149	141.4924873	177.991498	182.8620051	96.01108628	104.6626961	146.785241	158.8271656	133.8216251	148.0194129
ENSG00000167548	KMT2D	3268.080226	2837.595297	3721.045813	3289.491022	3430.381598	3841.619634	3779.159779	3440.578469	4232.854906	4014.340792	3769.079172	4005.828227
ENSG00000167549	CORO6	1.406230734	0	0	1.674467306	0	2.655672273	0	0	1.875849726	1.265555105	1.379604382	0
ENSG00000167550	RHEBL1	95.62368992	182.1666117	165.2893435	41.86168264	108.6441611	58.04540826	54.13391035	58.14594226	57.68237907	39.8649858	38.6289227	29.60388259
ENSG00000167552	TUBA1A	18.28099954	24.52242849	30.12662912	30.97764516	31.2063016	18.58970591	193.0435671	225.9385185	146.3162786	231.5965842	271.7820633	213.1479546
ENSG00000167553	TUBA1C	7805.986805	5794.299532	7806.053875	7690.828335	7205.188303	9133.995093	5345.978996	4761.322015	6680.838798	5562.114685	4907.252788	6566.986983
ENSG00000167554	ZNF610	60.46792157	66.56087734	68.39559043	31.81487881	60.10102531	48.18148267	145.038024	131.2436983	152.4127902	116.4310696	128.3032076	131.9487338
ENSG00000167555	ZNF528	608.8979079	676.1183856	567.520554	472.1997802	506.2355593	433.2539623	917.2122923	948.6095152	923.3870275	875.7641325	1018.148034	870.3541481
ENSG00000167562	ZNF701	433.1190661	472.9325495	428.2866734	331.5445265	350.2040513	314.1280917	610.7939319	606.3791122	584.796152	551.7820257	615.3035545	560.7821187
ENSG00000167565	SERTAD3	179.997534	224.2050605	209.2579374	187.5403382	195.3283323	166.9279715	205.3003015	229.2611438	245.7363141	205.6527045	193.1446135	228.3728085
ENSG00000167566	NCKAP5L	36.56199909	24.52242849	44.78282707	48.55955187	61.25681426	57.6660265	123.5887387	131.2436983	118.1785327	128.4538431	139.3400426	117.5697051
ENSG00000167578	RAB4B	39.37446056	10.50961221	28.49816268	12.55850479	9.246311586	17.83094241	30.64183605	48.17806645	29.54463318	39.8649858	27.59208765	28.75805737
ENSG00000167588	GPD1	0	0	0	0.837233653	0	1.517527013	1.021394535	0	0.937924863	0.632777552	2.759208765	0
ENSG00000167595	PROSER3	379.6822982	311.7851623	343.6064186	458.8040418	449.6019009	445.7735601	465.7559079	403.6989706	450.2039342	442.9442867	423.5385454	395.0003762
ENSG00000167600	CYP2S1	12.65607661	80.57369362	97.70798633	30.97764516	50.85471372	103.5712187	4.08557814	1.661312636	2.344812157	0	2.759208765	6.766601734
ENSG00000167601	AXL	1708.570342	1408.288036	2230.999021	1909.729962	2235.295826	2602.179446	3.064183605	11.62918845	22.97915914	0	6.898021912	18.60815477
ENSG00000167604	NFKBID	21.09346101	70.06408141	50.48245961	39.34998168	54.32208057	48.18148267	32.68462512	69.77513072	52.99275475	39.8649858	44.14734023	36.37048432
ENSG00000167608	TMC4	5.624922937	35.0320407	15.47043117	13.39573845	31.2063016	8.346398573	14.29952349	9.967875817	7.503398903	19.61610412	17.93485697	4.229126084
ENSG00000167612	ANKRD33	4.218692202	0	4.885399317	0	2.311577897	1.517527013	0	3.322625272	1.406887294	0.632777552	0	0.845825217
ENSG00000167613	LAIR1	0	0	4.885399317	0	0	0	5.106972674	0	0.468962431	0	0	0
ENSG00000167614	TTYH1	11.24984587	3.50320407	4.071166097	2.511700959	5.778944741	4.931962793	0	0	2.344812157	0	0	1.691650434
ENSG00000167615	LENG8	693.2717519	613.0607123	781.6638907	713.3230722	643.7744442	649.8809434	626.1148499	671.170305	570.2583166	655.5575443	707.7370481	547.2489153
ENSG00000167617	CDC42EP5	23.90592248	49.04485699	25.2412298	41.02444899	28.89472371	36.04126656	1.021394535	0	0.468962431	0.632777552	0	0.845825217
ENSG00000167618	LAIR2	0	0	1.628466439	0	0	0	1.021394535	9.967875817	5.627549177	2.53111021	9.657230676	5.074951301
ENSG00000167619	TMEM145	8.437384405	3.50320407	5.699632536	10.88403749	5.778944741	4.931962793	0	0	0.468962431	0.632777552	6.898021912	0.845825217
ENSG00000167625	ZNF526	191.2473798	224.2050605	217.4002696	205.9594786	171.0567643	199.934184	152.1877857	121.2758224	194.1504466	173.3810494	168.3117346	181.8524216
ENSG00000167632	TRAPPC9	711.5527515	777.7113036	696.1694026	616.2039685	589.4523636	622.9448389	824.2653896	637.9440523	759.719139	877.6624652	842.9382776	719.7972595
ENSG00000167634	NLRP7	15.46853808	17.51602035	13.02773151	17.58190671	19.64841212	37.55879358	2.04278907	8.306563181	4.220661883	1.898332657	6.898021912	0.845825217
ENSG00000167635	ZNF146	1223.420739	851.2785891	1208.322098	1306.921732	1035.586898	1259.168039	1444.251872	1292.501231	1357.177277	1425.647826	1132.655198	1493.727333
ENSG00000167637	ZNF283	555.46114	514.9709984	528.4373594	562.6210147	449.6019009	459.4313033	474.9484587	466.8288508	471.3072436	484.7076051	405.6036884	473.6621214

**ENSG00000167642**	SPINT2	1622.790267	1306.695118	1443.635498	1407.38977	1362.67517	1548.256935	1462.636974	1370.582925	1621.203126	1402.235056	1382.363591	1540.24772
**ENSG00000167644**	C19orf33	12.65607661	0	0	0	3.467366845	2.27629052	2.04278907	0	4.220661883	1.265555105	1.379604382	0
**ENSG00000167645**	YIF1B	119.5296124	73.56728548	127.0203822	138.9807864	132.9157291	154.4083736	105.2036371	63.12988017	98.48211061	106.3066288	135.2012295	100.6532008
**ENSG00000167646**	DNAAF3	74.53022891	14.01281628	28.49816268	37.67551438	57.78944741	32.24744903	11.23533988	8.306563181	5.627549177	3.163887762	4.138813147	4.229126084
**ENSG00000167653**	PSCA	44.99938349	133.1217547	108.2930182	69.49039319	129.4483622	129.7485596	199.1719343	330.6012146	198.8400709	200.5904841	252.467602	212.3021294
**ENSG00000167656**	LY6D	2.812461468	0	0	0.837233653	0	0.379381753	0	1.661312636	7.503398903	0	2.759208765	5.920776517
**ENSG00000167657**	DAPK3	164.5289959	245.2242849	264.6257963	210.9828805	213.8209554	256.841447	195.0863562	287.407086	239.17084	215.1443678	256.6064151	235.9852355
**ENSG00000167658**	EEF2	19131.76914	16969.52052	17455.53176	18303.60212	18486.84423	19465.319	14707.05991	15466.82064	17268.60361	15356.87842	16068.25224	16875.90473
**ENSG00000167670**	CHAF1A	967.4867451	1355.739975	1020.234224	969.51657	1043.67742	1019.398771	892.6988235	980.1744553	961.3729845	942.2057755	1048.499331	970.1615236
**ENSG00000167671**	UBXN6	378.2760675	346.817203	355.8199169	462.99021	419.5513882	473.4684281	391.1941069	440.2478486	444.576385	417.6331846	474.5839075	400.0753275
**ENSG00000167674**	HDGFL2	427.4941432	500.9581821	433.1720727	472.1997802	527.0397604	438.1859251	463.7131188	505.0390414	401.4318413	484.0748276	452.5102374	438.9832875
**ENSG00000167676**	PLIN4	4.218692202	0	1.628466439	0	0	0.758763507	0	0	3.28273702	1.898332657	0	0
**ENSG00000167680**	SEMA6B	102.6548436	150.637775	107.478785	245.3094603	264.6756692	159.3403364	6.128367209	0	0.937924863	0	4.138813147	2.53747565
**ENSG00000167685**	ZNF444	81.56138258	101.592918	94.45105346	105.4914403	124.8252064	94.46605657	106.2250316	142.8728867	110.6751338	115.1655145	80.01705417	99.80737558
**ENSG00000167693**	NXN	2206.376022	1678.03475	1816.554313	2058.757552	1915.142287	2091.152224	511.718662	372.1340305	536.961984	463.8259459	463.5470725	551.4780413
**ENSG00000167695**	FAM57A	258.7464551	308.2819582	201.1156052	272.9381708	317.8419608	248.1156667	186.9151999	408.6829085	226.9778168	218.3082556	440.093798	229.2186337
**ENSG00000167699**	GLOD4	897.1752084	1047.458017	909.4985061	920.1197845	894.580646	851.7120362	649.6069242	677.8155555	618.0924847	699.2191954	682.9041692	616.606583
**ENSG00000167700**	MFSD3	42.18692202	21.01922442	56.99632536	78.69996337	98.2420606	67.52995209	33.70601965	23.25837691	49.2410553	41.1305409	66.22101035	60.05359039
**ENSG00000167701**	GPT	5.624922937	0	2.442699658	2.511700959	0	0.379381753	3.064183605	3.322625272	0.937924863	1.265555105	1.379604382	2.53747565
**ENSG00000167702**	KIFC2	257.3402244	360.8300193	197.0444391	368.3828073	416.0840214	260.2558828	235.9421376	232.5837691	207.7503571	282.2187884	310.410986	240.2143616
**ENSG00000167703**	SLC43A2	97.02992066	66.56087734	28.49816268	110.5148422	83.21680428	81.18769521	217.5570359	79.74300653	227.9157417	233.4949168	131.0624163	213.1479546
**ENSG00000167705**	RILP	43.59315276	38.53524477	44.78282707	4.186168264	9.246311586	13.65774312	21.44928523	6.645250545	24.85500887	13.92110615	12.41643944	23.68310607
**ENSG00000167711**	SERPINF2	5.624922937	0	3.256932878	0	11.55788948	1.896908767	0	6.645250545	1.406887294	3.796665314	0	0
**ENSG00000167716**	WDR81	412.0256051	248.727489	525.9946598	341.5913304	351.3598403	414.2848746	403.4508413	212.6480174	407.9973154	354.9882069	260.7452283	283.3514476
**ENSG00000167720**	SRR	292.4959927	399.365264	257.2976973	257.0307314	330.5556392	220.0414169	288.0332588	275.7778976	253.7086754	251.2126883	257.9860195	208.9188285
**ENSG00000167721**	TSR1	1985.597797	1762.111647	1944.388928	2124.061777	1663.180297	1961.403665	1480.000681	1287.517293	1365.6186	1375.025621	1120.238758	1543.631021
**ENSG00000167723**	TRPV3	89.99876699	94.5865099	132.7200148	102.9797393	98.2420606	155.9259006	2.04278907	0	4.220661883	5.694997971	2.759208765	6.766601734
**ENSG00000167733**	HSD11B1L	15.46853808	21.01922442	11.39926507	5.86063557	20.80420107	7.967016819	7.149761744	33.22625272	12.66198565	32.27165517	17.93485697	10.1499026
**ENSG00000167740**	CYB5D2	126.5607661	98.08971397	101.7791524	123.073347	106.3325832	94.46605657	176.7012545	284.0844608	190.3987472	213.2460352	242.8103713	162.3984416
**ENSG00000167741**	GGT6	8.437384405	0	2.442699658	1.674467306	0	0	0	0	0	0	0	0
**ENSG00000167747**	C19orf48	381.088529	381.8492437	433.1720727	456.2923408	345.5808955	463.9838843	367.7020326	400.3763453	483.9692293	448.6392846	404.224084	357.7840667
**ENSG00000167748**	KLK1	16.87476881	31.52883663	21.98429693	17.58190671	46.23155793	21.62475994	2.04278907	1.661312636	10.31717349	2.53111021	1.379604382	6.766601734
**ENSG00000167749**	KLK4	0	0	2.442699658	2.511700959	1.155788948	2.27629052	0	0	0	0	0	0
**ENSG00000167751**	KLK2	16.87476881	35.0320407	18.72736405	13.39573845	27.73893476	16.69279715	2.04278907	21.59706427	7.034436472	8.226108181	13.79604382	15.2248539
**ENSG00000167754**	KLK5	2536.840244	2914.665787	2842.488169	4108.305535	4786.122035	4553.719185	40.8557814	11.62918845	67.53059013	30.37332251	33.11050518	88.81164776
**ENSG00000167755**	KLK6	327.6517611	518.4742024	582.9909851	598.6220618	1041.365842	1098.689558	13.27812895	21.59706427	30.48255804	29.74054496	23.4532745	42.29126084
**ENSG00000167757**	KLK11	11.24984587	17.51602035	5.699632536	12.55850479	26.58314581	8.725780326	1.021394535	0	0.937924863	0	0	2.53747565
**ENSG00000167759**	KLK13	0	0	0	0	0	3.793817533	0	0	6.096511609	3.796665314	0	0.845825217
**ENSG00000167766**	ZNF83	1324.669352	1709.563586	1356.512544	1355.481284	1167.346838	949.9719103	1120.469805	1044.965648	829.1255788	992.8279797	1067.813792	928.716088
**ENSG00000167767**	KRT80	81.56138258	122.6121425	122.1349829	102.9797393	219.5999002	241.2867951	62.30506663	26.58100218	28.60670832	72.76941852	27.59208765	43.98291127
**ENSG00000167768**	KRT1	14.06230734	7.006408141	26.86969624	39.34998168	2.311577897	33.00621254	1038.758242	898.7701361	1717.809386	1110.524604	702.2186306	1668.813153
**ENSG00000167770**	OTUB1	604.6792157	546.499835	673.3708725	658.9028848	555.9344841	654.4335245	397.3224741	470.151476	569.7893542	500.5270439	492.5187645	552.3238666
**ENSG00000167771**	RCOR2	5.624922937	0	0	3.348934611	11.55788948	3.41443578	0	0	0	0	5.518417529	0
**ENSG00000167772**	ANGPTL4	167.3414574	1828.672525	192.973273	139.81802	754.7301832	168.0661167	61.28367209	488.425915	75.03398903	42.39609601	433.195776	66.82019213
**ENSG00000167775**	CD320	12.65607661	21.01922442	1.628466439	7.535102876	6.93473369	5.6907263	12.25673442	4.983937908	8.910286198	8.226108181	2.759208765	5.074951301
**ENSG00000167778**	SPRYD3	541.3988327	402.8684681	672.5566393	465.501911	455.3808456	545.930343	328.8890402	375.4566558	427.2247751	354.3554293	295.2353378	349.3258145
**ENSG00000167779**	IGFBP6	187.0286876	269.7467134	180.7597747	98.79357104	94.77469376	105.8475092	13.27812895	14.95181373	10.78613592	3.796665314	8.277626294	0
**ENSG00000167785**	ZNF558	364.2137601	451.9133251	341.1637189	332.3817602	249.6504128	249.6331937	466.7773024	481.7806645	444.1074226	431.5542907	441.4734023	512.5700814
**ENSG00000167792**	NDUFV1	697.4904441	560.5126513	703.4975016	843.0942884	926.9427365	843.3656376	835.5007295	833.9789433	832.8772783	841.5941447	909.1592879	877.966575
**ENSG00000167797**	CDK2AP2	458.4312193	360.8300193	346.0491183	352.4753679	342.1135287	310.713656	343.1885637	348.8756536	378.9216446	327.1459946	314.5497992	315.4928059
**ENSG00000167799**	NUDT8	15.46853808	17.51602035	27.68392946	24.27977593	36.98524634	39.07632059	21.44928523	43.19412854	28.13774589	36.70109804	20.69406573	30.4497078
**ENSG00000167815**	PRDX2	1039.204513	1432.810465	1241.70566	1247.478143	1469.007753	1353.254714	805.880288	1181.193284	882.587296	926.3863367	1289.930097	1175.697051
**ENSG00000167840**	ZNF232	251.7153014	287.2627338	194.6017394	203.4477776	168.7451864	189.6908767	177.7226491	187.7283279	168.3575129	141.7421717	155.8952952	199.6147512
**ENSG00000167842**	MIS12	667.9595987	837.2657728	696.9836358	539.1784724	491.210303	478.0210092	511.718662	701.0739324	539.7757586	546.7198053	521.4904565	504.1118292
**ENSG00000167850**	CD300C	2.812461468	14.01281628	26.05546302	3.348934611	12.71367843	11.00207085	0	0	0	0	1.379604382	0
**ENSG00000167851**	CD300A	0	3.50320407	1.628466439	0	16.18104528	0	0	19.93575163	0.937924863	0	6.898021912	0
**ENSG00000167861**	HID1	28.12461468	7.006408141	23.61276336	34.32657977	18.49262317	26.93610448	31.66323058	28.24231481	22.97915914	33.53721028	31.73090079	21.99145564
**ENSG00000167862**	MRPL58	288.2773005	259.2371012	330.5786871	346.6147323	279.7009255	323.2332538	244.1132938	239.2290196	206.8124323	220.8393658	195.9038223	254.5933902
**ENSG00000167863**	ATP5PD	1560.916115	1527.396975	1359.769476	1525.439716	1571.87297	1573.675513	1401.353302	1427.067554	1519.90724	1445.26393	1520.324029	1659.509075
**ENSG00000167874**	TMEM88	1.406230734	24.52242849	14.65619795	5.023401917	9.246311586	4.931962793	2.04278907	1.661312636	4.220661883	4.429442867	5.518417529	2.53747565
**ENSG00000167880**	EVPL	563.8985244	490.4485699	545.536257	923.4687191	889.9574902	874.0955596	426.9429156	378.779281	466.1486569	463.8259459	438.7141936	410.2252301
**ENSG00000167881**	SRP68	1709.976573	1692.047566	1976.958257	1990.941627	2012.228559	2029.312998	1493.27881	1787.572396	1608.54114	1521.197236	1626.553567	1656.9716
**ENSG00000167889**	MGAT5B	7.031153671	10.50961221	19.54159727	7.535102876	15.02525633	17.0721789	0	0	1.406887294	1.265555105	4.138813147	0
**ENSG00000167895**	TMC8	1.406230734	0	4.885399317	0	1.155788948	1.517527013	1.021394535	0	0	0	0	1.691650434
**ENSG00000167900**	TK1	613.1166001	770.7048955	539.0223913	606.1571647	735.0817711	712.8583145	453.4991735	764.2038126	630.7544703	510.0187072	637.3772246	527.7949353
**ENSG00000167904**	TMEM68	399.3695285	364.3332233	325.6932878	385.964714	383.7219308	336.5116152	319.6964894	284.0844608	289.8187826	330.3098823	257.9860195	279.1223215
**ENSG00000167912**	AC090152.1	4.218692202	0	4.885399317	7.535102876	5.778944741	11.3814526	2.04278907	0	2.813774589	0.632777552	0	2.53747565

**ENSG00000167914**	GSDMA	22.49969175	73.56728548	23.61276336	11.72127114	18.49262317	15.93403364	0	8.306563181	6.096511609	0.632777552	4.138813147	11.84155303
**ENSG00000167916**	KRT24	674.9907524	276.7531216	381.8753799	477.2231821	113.2673169	258.358974	149.1236021	196.0348911	120.9923073	143.6405044	177.9689653	98.96155036
**ENSG00000167920**	TMEM99	213.7470716	161.1473872	217.4002696	293.0317785	284.3240813	253.0476295	166.4873092	227.5998311	162.2610013	182.8727126	212.4590749	160.7067912
**ENSG00000167925**	GHDC	81.56138258	94.5865099	95.26528668	98.79357104	114.4231059	100.5361646	102.1394535	69.77513072	90.50974927	103.7755186	89.67428485	97.26989993
**ENSG00000167930**	FAM234A	149.0604578	161.1473872	189.7163401	179.1680017	206.8862217	206.3836738	446.3494117	290.7297113	450.6728966	439.1476214	452.5102374	432.2166858
**ENSG00000167962**	ZNF598	406.4006822	350.320407	464.1129351	463.8274437	426.4861219	561.4849949	304.3755714	305.681525	325.4599274	321.4509966	309.0313816	347.6341641
**ENSG00000167964**	RAB26	21.09346101	59.5544692	8.142332194	48.55955187	47.38734688	24.65981396	5.106972674	4.983937908	6.096511609	11.38999594	22.07367012	3.383300867
**ENSG00000167965**	MLST8	284.0586083	178.6634076	252.412298	305.5902833	316.6861718	347.1343043	196.1077507	192.7122658	251.8328257	227.7999189	240.0511625	202.998052
**ENSG00000167967**	E4F1	202.4972257	136.6249587	219.028736	203.4477776	195.3283323	219.6620352	182.8296217	171.1152015	242.9225395	218.9410331	235.9123494	205.5355277
**ENSG00000167968**	DNASE1L2	12.65607661	28.02563256	3.256932878	2.511700959	2.311577897	2.655672273	9.192550814	0	4.689624315	1.265555105	2.759208765	0
**ENSG00000167969**	ECI1	539.9926019	364.3332233	436.4290056	490.6189206	461.1597904	474.6065734	670.0348149	515.0069172	682.3403378	638.4725504	500.7963908	624.21901
**ENSG00000167971**	CASKIN1	5.624922937	0	0.814233219	9.209570182	6.93473369	4.931962793	1.021394535	6.645250545	3.28273702	3.796665314	2.759208765	0.845825217
**ENSG00000167972**	ABCA3	19.68723028	24.52242849	22.79853014	16.74467306	26.58314581	9.863925586	1.021394535	0	1.875849726	8.226108181	4.138813147	5.074951301
**ENSG00000167977**	KCTD5	624.366446	546.499835	680.6989715	569.3188839	522.4166046	682.1283924	413.6647866	481.7806645	498.0381022	523.9398134	499.4167864	565.0112448
**ENSG00000167978**	SRRM2	12516.85976	12422.36163	12777.76191	13518.81179	12978.3541	12706.63306	12720.44754	12712.36429	11328.72546	13683.81457	14113.35283	12053.00934
**ENSG00000167981**	ZNF597	137.8106119	112.1025303	146.5619795	163.2605623	156.031508	133.5423772	187.9365944	106.3240087	154.7576024	120.8605125	142.0992514	133.6403843
**ENSG00000167985**	SDHAF2	68.90530597	52.54806106	54.5536257	51.90848648	32.36209055	75.11758715	54.13391035	74.75906863	67.99955256	74.03497363	52.42496653	42.29126084
**ENSG00000167986**	DDB1	3103.55123	2785.047236	2933.68229	3334.701639	3225.806955	3391.293493	6621.70077	5686.673153	6357.254721	7175.064666	6379.290664	6456.18388
**ENSG00000167987**	VPS37C	368.4324523	416.8812844	387.5750125	352.4753679	321.3093276	331.9590341	296.2044151	355.5209041	373.7630579	293.6087843	288.3373159	322.2594076
**ENSG00000167992**	VWCE	0	0	0.814233219	0	0	0	3.064183605	6.645250545	0.937924863	0	4.138813147	0.845825217
**ENSG00000167994**	RAB3IL1	82.96761332	84.07689769	101.7791524	74.51379511	102.8652164	89.53409378	2.04278907	9.967875817	1.406887294	0.632777552	1.379604382	3.383300867
**ENSG00000167995**	BEST1	29.53084542	52.54806106	52.92515926	44.3733836	63.56839215	33.38559429	29.62044151	24.91968954	35.17218236	42.39609601	68.98021912	27.91223215
**ENSG00000167996**	FTH1	20113.31819	33248.90983	22604.74264	20457.80431	31748.36662	21888.80964	13894.02986	20309.54698	13409.98073	13312.37415	18792.9709	13516.28696
**ENSG00000168000**	BSCL2	77.34269038	63.05767327	79.79485551	76.18826241	72.81470374	68.28871559	101.118059	114.6305719	81.13050064	113.8999594	102.0907243	87.96582254
**ENSG00000168002**	POLR2G	538.5863712	504.4613861	524.3661933	566.807183	601.0102531	694.6479903	540.3177089	500.0551035	592.2995509	628.9808871	550.4621485	713.0306577
**ENSG00000168003**	SLC3A2	4190.567588	3156.386867	4205.514578	4958.097692	5139.793453	5651.649979	3332.810367	3061.799188	3699.644622	3517.610414	3167.571662	3262.347861
**ENSG00000168005**	SPINDOC	343.1202991	343.3139989	359.891083	349.9636669	406.8377098	416.5611651	290.0760479	305.681525	329.6805893	304.3660027	372.4931832	360.3215423
**ENSG00000168010**	ATG16L2	122.3420739	262.7403053	152.261612	182.5169363	161.8104528	106.9856544	120.5245551	222.6158932	120.0543825	199.324929	244.1899757	101.499026
**ENSG00000168014**	C2CD3	1608.72796	1429.307261	1521.801887	1754.841736	1655.089774	1698.112728	2421.726442	2093.253922	2407.653123	2107.149249	2044.573695	2006.297414
**ENSG00000168016**	TRANK1	1043.423205	1713.06679	1125.270309	87.90953355	234.6251565	140.7506305	828.3509678	805.7366285	584.3271896	212.6132576	120.0255813	81.19922081
**ENSG00000168026**	TTC21A	35.15576835	56.05126513	26.86969624	23.44254228	36.98524634	17.83094241	73.54040651	154.5020752	86.28908739	108.837739	129.6828119	77.81591994
**ENSG00000168028**	RPSA	6440.536762	5647.164962	5592.967984	6380.557669	5919.950993	6046.965766	7522.570749	7583.892184	8186.208203	7663.568937	7641.628674	7871.249467
**ENSG00000168032**	ENTPD3	532.9614482	406.3716722	578.1055858	102.9797393	151.4083522	129.3691779	3.064183605	23.25837691	22.97915914	7.593330629	6.898021912	18.60815477
**ENSG00000168036**	CTNNB1	5001.962721	4512.126843	4812.93256	5555.045287	5333.965996	6033.687405	4840.388701	5442.460196	5755.106959	5248.257019	5345.966981	5942.767973
**ENSG00000168038**	ULK4	310.7769922	304.7787541	220.6572025	339.0796294	331.7114282	256.0826835	615.9009045	549.8944826	625.1269211	759.9658404	707.7370481	625.9106604
**ENSG00000168040**	FADD	307.9645308	171.6569995	378.618447	348.2891996	293.5703929	389.6250606	207.3430906	206.0027669	263.5568865	211.3477025	233.1531406	261.359992
**ENSG00000168056**	LTBP3	403.5882207	266.2435094	282.5389271	511.5497619	544.3765946	457.1550127	538.2749199	453.5383497	485.3761166	568.234242	575.2950274	466.0496944
**ENSG00000168061**	SAC3D1	168.7476881	98.08971397	143.3050466	198.4243757	205.7304328	199.5548022	170.5728873	122.9371351	158.9782643	121.4932901	126.9236032	181.0065964
**ENSG00000168062**	BATF2	1586.228268	1604.467464	1631.723372	36.83828073	13.86946738	15.93403364	162.401731	215.9706427	174.9229869	2.53111021	0	2.53747565
**ENSG00000168065**	SLC22A11	0	3.50320407	1.628466439	0	0	0.379381753	0	8.306563181	0.937924863	9.491663286	0	4.229126084
**ENSG00000168066**	SF1	3251.205457	2606.383828	3018.362544	3017.390085	2675.651415	3052.505587	3134.659828	2779.37604	3474.542655	3208.814968	2746.792325	3084.724566
**ENSG00000168067**	MAP4K2	227.8093789	311.7851623	234.4991672	243.634993	247.3388349	262.5321733	220.6212195	191.0509532	227.4467793	213.2460352	234.532745	207.2271781
**ENSG00000168071**	CCDC88B	29.53084542	28.02563256	22.79853014	56.09465474	54.32208057	39.45570234	77.62598465	88.04956971	42.67558126	69.60553076	70.3598235	44.82873649
**ENSG00000168077**	SCARA3	355.7763757	325.7979785	253.2265312	725.881577	562.8692178	563.7612854	862.0569874	646.2506155	962.3109093	984.6018715	731.1903226	992.9988045
**ENSG00000168078**	PBK	908.4250543	760.1952833	914.3839054	1170.452647	1128.050014	1146.112277	403.4508413	348.8756536	412.6869397	396.7515253	302.1333597	480.4287231
**ENSG00000168079**	SCARA5	0	0	1.628466439	1.674467306	28.89472371	5.6907263	59.24088302	21.59706427	17.8205724	47.45831643	41.38813147	21.99145564
**ENSG00000168090**	COPS6	795.9265955	826.7561606	807.7193537	1004.680383	1007.847963	1014.84619	760.9389285	878.8343845	801.4567954	790.3391629	860.8731346	887.2706524
**ENSG00000168092**	PAFAH1B2	3367.922608	2858.614521	2914.140692	3362.33035	3181.886975	3259.648024	1997.84771	1857.347527	2209.750977	2105.250917	2153.562441	2381.84381
**ENSG00000168096**	ANKS3	70.31153671	77.07048955	88.75142092	96.28187008	73.97049269	109.6413267	117.4603715	89.71088235	103.6406974	109.4705166	95.19270238	87.96582254
**ENSG00000168101**	NUDT16L1	46.40561423	63.05767327	72.46675653	87.0722999	86.68417112	67.90933384	33.70601965	43.19412854	69.40643986	88.58885733	59.32298844	69.35766778
[truncated: 3,158,501 more chars]
